# Supplementary material for: Time-series transcriptome analysis identified differentially expressed genes in broiler chicken infected with mixed Eimeria species
Source: Front Genet. 2022 Aug 8;13:886781. doi: 10.3389/fgene.2022.886781 (PMC9393255; doi:10.3389/fgene.2022.886781)
Supplement: Supplementary file 2 [file DataSheet1.ZIP › 7dpi_GO.Gsea.1625106495923/gsea_report_for_0_1625106495923.html]

Report for 0 1625106495923 [GSEA]

| GS  follow link to MSigDB | GS DETAILS | SIZE | ES | NES | NOM p-val | FDR q-val | FWER p-val | RANK AT MAX | LEADING EDGE || 1 | GOMF\_PHOSPHOTYROSINE\_RESIDUE\_BINDING | Details ... | 34 | -0.63 | -1.94 | 0.000 | 0.037 | 0.224 | 2599 | tags=65%, list=22%, signal=82% |
| 2 | GOBP\_MICROTUBULE\_DEPOLYMERIZATION | Details ... | 29 | -0.63 | -1.90 | 0.000 | 0.066 | 0.404 | 3551 | tags=79%, list=30%, signal=112% |
| 3 | GOBP\_LEUKOCYTE\_HOMEOSTASIS | Details ... | 56 | -0.56 | -1.89 | 0.000 | 0.077 | 0.492 | 1951 | tags=45%, list=16%, signal=53% |
| 4 | GOBP\_REGULATION\_OF\_TRANSLATION\_NCRNA\_MEDIATED | Details ... | 13 | -0.75 | -1.88 | 0.000 | 0.077 | 0.527 | 2080 | tags=69%, list=17%, signal=84% |
| 5 | GOMF\_PROTEIN\_TYROSINE\_KINASE\_BINDING | Details ... | 68 | -0.54 | -1.87 | 0.000 | 0.092 | 0.627 | 1016 | tags=28%, list=8%, signal=30% |
| 6 | GOBP\_PHOSPHATIDYLINOSITOL\_3\_PHOSPHATE\_BIOSYNTHETIC\_PROCESS | Details ... | 14 | -0.74 | -1.87 | 0.001 | 0.085 | 0.629 | 1884 | tags=79%, list=16%, signal=93% |
| 7 | GOBP\_POSITIVE\_REGULATION\_OF\_GLYCOLYTIC\_PROCESS | Details ... | 17 | -0.70 | -1.86 | 0.001 | 0.096 | 0.703 | 685 | tags=35%, list=6%, signal=37% |
| 8 | GOMF\_CYCLASE\_ACTIVITY | Details ... | 12 | -0.76 | -1.85 | 0.001 | 0.099 | 0.736 | 1687 | tags=58%, list=14%, signal=68% |
| 9 | GOMF\_PHOSPHORUS\_OXYGEN\_LYASE\_ACTIVITY | Details ... | 13 | -0.74 | -1.85 | 0.000 | 0.099 | 0.763 | 1687 | tags=54%, list=14%, signal=63% |
| 10 | GOBP\_POSITIVE\_REGULATION\_OF\_SMOOTH\_MUSCLE\_CELL\_MIGRATION | Details ... | 32 | -0.60 | -1.85 | 0.000 | 0.097 | 0.779 | 3286 | tags=59%, list=27%, signal=82% |
| 11 | GOBP\_CYCLIC\_NUCLEOTIDE\_METABOLIC\_PROCESS | Details ... | 24 | -0.64 | -1.84 | 0.000 | 0.099 | 0.805 | 1687 | tags=50%, list=14%, signal=58% |
| 12 | GOBP\_REGULATION\_OF\_RAS\_PROTEIN\_SIGNAL\_TRANSDUCTION | Details ... | 146 | -0.50 | -1.84 | 0.000 | 0.094 | 0.806 | 3534 | tags=52%, list=29%, signal=73% |
| 13 | GOMF\_PROTEIN\_PHOSPHORYLATED\_AMINO\_ACID\_BINDING | Details ... | 45 | -0.56 | -1.83 | 0.000 | 0.110 | 0.869 | 2599 | tags=53%, list=22%, signal=68% |
| 14 | GOBP\_COLLATERAL\_SPROUTING | Details ... | 15 | -0.70 | -1.82 | 0.001 | 0.113 | 0.892 | 1402 | tags=47%, list=12%, signal=53% |
| 15 | GOBP\_PROTEIN\_O\_LINKED\_MANNOSYLATION | Details ... | 13 | -0.73 | -1.82 | 0.001 | 0.108 | 0.892 | 1173 | tags=46%, list=10%, signal=51% |
| 16 | GOBP\_LIPID\_PHOSPHORYLATION | Details ... | 57 | -0.54 | -1.82 | 0.000 | 0.106 | 0.899 | 2930 | tags=53%, list=24%, signal=69% |
| 17 | GOBP\_EMBRYONIC\_AXIS\_SPECIFICATION | Details ... | 15 | -0.70 | -1.82 | 0.002 | 0.106 | 0.909 | 1764 | tags=53%, list=15%, signal=62% |
| 18 | GOBP\_EMBRYONIC\_CAMERA\_TYPE\_EYE\_FORMATION | Details ... | 5 | -0.94 | -1.82 | 0.000 | 0.110 | 0.922 | 295 | tags=60%, list=2%, signal=61% |
| 19 | GOBP\_RETINAL\_METABOLIC\_PROCESS | Details ... | 11 | -0.75 | -1.81 | 0.001 | 0.123 | 0.950 | 51 | tags=27%, list=0%, signal=27% |
| 20 | GOBP\_BLASTODERM\_SEGMENTATION | Details ... | 11 | -0.75 | -1.81 | 0.001 | 0.121 | 0.953 | 295 | tags=36%, list=2%, signal=37% |
| 21 | GOMF\_RECEPTOR\_TYROSINE\_KINASE\_BINDING |  | 50 | -0.54 | -1.80 | 0.000 | 0.120 | 0.958 | 2640 | tags=40%, list=22%, signal=51% |
| 22 | GOCC\_SITE\_OF\_POLARIZED\_GROWTH |  | 137 | -0.49 | -1.80 | 0.000 | 0.122 | 0.963 | 3079 | tags=42%, list=26%, signal=55% |
| 23 | GOBP\_AXIS\_SPECIFICATION |  | 46 | -0.55 | -1.80 | 0.000 | 0.125 | 0.969 | 1991 | tags=39%, list=17%, signal=47% |
| 24 | GOBP\_DNA\_DEPENDENT\_DNA\_REPLICATION\_MAINTENANCE\_OF\_FIDELITY |  | 39 | -0.57 | -1.79 | 0.000 | 0.125 | 0.972 | 3310 | tags=54%, list=28%, signal=74% |
| 25 | GOBP\_POSITIVE\_REGULATION\_OF\_BEHAVIOR |  | 13 | -0.71 | -1.79 | 0.002 | 0.122 | 0.973 | 2901 | tags=77%, list=24%, signal=101% |
| 26 | GOMF\_PHOSPHATIDYLINOSITOL\_3\_KINASE\_ACTIVITY |  | 12 | -0.73 | -1.79 | 0.001 | 0.120 | 0.975 | 1884 | tags=83%, list=16%, signal=99% |
| 27 | GOBP\_REGULATION\_OF\_DENDRITE\_EXTENSION |  | 18 | -0.66 | -1.79 | 0.001 | 0.119 | 0.976 | 1360 | tags=44%, list=11%, signal=50% |
| 28 | GOBP\_REPLICATION\_FORK\_PROCESSING |  | 32 | -0.58 | -1.79 | 0.001 | 0.126 | 0.984 | 3214 | tags=56%, list=27%, signal=77% |
| 29 | GOMF\_MAP\_KINASE\_SCAFFOLD\_ACTIVITY |  | 9 | -0.78 | -1.79 | 0.002 | 0.123 | 0.984 | 1522 | tags=56%, list=13%, signal=64% |
| 30 | GOBP\_TRIPARTITE\_REGIONAL\_SUBDIVISION |  | 8 | -0.81 | -1.79 | 0.001 | 0.122 | 0.986 | 295 | tags=50%, list=2%, signal=51% |
| 31 | GOBP\_POSITIVE\_REGULATION\_OF\_PHOSPHATIDYLINOSITOL\_3\_KINASE\_SIGNALING |  | 64 | -0.52 | -1.78 | 0.000 | 0.130 | 0.990 | 3534 | tags=53%, list=29%, signal=75% |
| 32 | GOBP\_POSITIVE\_REGULATION\_OF\_TYROSINE\_PHOSPHORYLATION\_OF\_STAT\_PROTEIN |  | 30 | -0.59 | -1.78 | 0.001 | 0.134 | 0.992 | 1823 | tags=43%, list=15%, signal=51% |
| 33 | GOBP\_LYMPHOCYTE\_HOMEOSTASIS |  | 39 | -0.56 | -1.77 | 0.000 | 0.133 | 0.993 | 2474 | tags=49%, list=21%, signal=61% |
| 34 | GOBP\_POSITIVE\_REGULATION\_OF\_LYMPHOCYTE\_DIFFERENTIATION |  | 67 | -0.52 | -1.77 | 0.000 | 0.134 | 0.994 | 2408 | tags=46%, list=20%, signal=58% |
| 35 | GOBP\_REGULATION\_OF\_COLLATERAL\_SPROUTING |  | 10 | -0.75 | -1.77 | 0.001 | 0.134 | 0.996 | 488 | tags=40%, list=4%, signal=42% |
| 36 | GOBP\_HETEROPHILIC\_CELL\_CELL\_ADHESION\_VIA\_PLASMA\_MEMBRANE\_CELL\_ADHESION\_MOLECULES |  | 26 | -0.60 | -1.77 | 0.001 | 0.133 | 0.996 | 3538 | tags=65%, list=29%, signal=92% |
| 37 | GOBP\_AMINO\_ACID\_BETAINE\_METABOLIC\_PROCESS |  | 12 | -0.72 | -1.77 | 0.002 | 0.139 | 0.997 | 2534 | tags=58%, list=21%, signal=74% |
| 38 | GOMF\_PHOSPHATIDYLSERINE\_BINDING |  | 43 | -0.54 | -1.76 | 0.001 | 0.141 | 0.998 | 2050 | tags=35%, list=17%, signal=42% |
| 39 | GOMF\_EPHRIN\_RECEPTOR\_BINDING |  | 22 | -0.62 | -1.76 | 0.003 | 0.145 | 0.998 | 1670 | tags=41%, list=14%, signal=47% |
| 40 | GOMF\_ORGANIC\_ACID\_SODIUM\_SYMPORTER\_ACTIVITY |  | 17 | -0.65 | -1.76 | 0.002 | 0.153 | 0.999 | 1222 | tags=47%, list=10%, signal=52% |
| 41 | GOBP\_DEVELOPMENTAL\_CELL\_GROWTH |  | 151 | -0.47 | -1.75 | 0.000 | 0.156 | 0.999 | 3235 | tags=42%, list=27%, signal=56% |
| 42 | GOBP\_POSITIVE\_REGULATION\_OF\_LONG\_TERM\_SYNAPTIC\_POTENTIATION |  | 16 | -0.66 | -1.75 | 0.002 | 0.161 | 0.999 | 2466 | tags=63%, list=21%, signal=79% |
| 43 | GOBP\_HEMATOPOIETIC\_STEM\_CELL\_MIGRATION |  | 7 | -0.82 | -1.75 | 0.000 | 0.159 | 0.999 | 1608 | tags=57%, list=13%, signal=66% |
| 44 | GOCC\_PHOSPHATIDYLINOSITOL\_3\_KINASE\_COMPLEX |  | 26 | -0.60 | -1.75 | 0.001 | 0.158 | 0.999 | 2670 | tags=62%, list=22%, signal=79% |
| 45 | GOBP\_REGULATION\_OF\_LIPID\_KINASE\_ACTIVITY |  | 54 | -0.53 | -1.75 | 0.001 | 0.156 | 0.999 | 2879 | tags=44%, list=24%, signal=58% |
| 46 | GOBP\_REGULATION\_OF\_MODIFICATION\_OF\_SYNAPTIC\_STRUCTURE |  | 9 | -0.77 | -1.75 | 0.002 | 0.154 | 0.999 | 198 | tags=33%, list=2%, signal=34% |
| 47 | GOBP\_PROTEIN\_KINASE\_C\_ACTIVATING\_G\_PROTEIN\_COUPLED\_RECEPTOR\_SIGNALING\_PATHWAY |  | 23 | -0.61 | -1.75 | 0.001 | 0.152 | 0.999 | 948 | tags=39%, list=8%, signal=42% |
| 48 | GOBP\_MYELOID\_PROGENITOR\_CELL\_DIFFERENTIATION |  | 4 | -0.96 | -1.75 | 0.000 | 0.151 | 0.999 | 199 | tags=50%, list=2%, signal=51% |
| 49 | GOBP\_REGULATION\_OF\_LYMPHOCYTE\_DIFFERENTIATION |  | 120 | -0.47 | -1.74 | 0.000 | 0.152 | 0.999 | 2408 | tags=39%, list=20%, signal=48% |
| 50 | GOMF\_IMMUNE\_RECEPTOR\_ACTIVITY |  | 77 | -0.50 | -1.74 | 0.000 | 0.149 | 0.999 | 3130 | tags=48%, list=26%, signal=65% |
| 51 | GOCC\_T\_CELL\_RECEPTOR\_COMPLEX |  | 14 | -0.68 | -1.74 | 0.002 | 0.148 | 0.999 | 2640 | tags=71%, list=22%, signal=91% |
| 52 | GOBP\_CD8\_POSITIVE\_ALPHA\_BETA\_T\_CELL\_ACTIVATION |  | 16 | -0.67 | -1.74 | 0.002 | 0.148 | 1.000 | 778 | tags=44%, list=6%, signal=47% |
| 53 | GOBP\_TYROSINE\_PHOSPHORYLATION\_OF\_STAT\_PROTEIN |  | 42 | -0.54 | -1.74 | 0.001 | 0.150 | 1.000 | 1823 | tags=38%, list=15%, signal=45% |
| 54 | GOBP\_INNERVATION |  | 16 | -0.66 | -1.74 | 0.002 | 0.149 | 1.000 | 2903 | tags=50%, list=24%, signal=66% |
| 55 | GOBP\_POSITIVE\_REGULATION\_OF\_LEUKOCYTE\_CELL\_CELL\_ADHESION |  | 150 | -0.47 | -1.74 | 0.000 | 0.150 | 1.000 | 2408 | tags=40%, list=20%, signal=49% |
| 56 | GOBP\_REGULATION\_OF\_MODIFICATION\_OF\_POSTSYNAPTIC\_STRUCTURE |  | 6 | -0.85 | -1.74 | 0.001 | 0.150 | 1.000 | 198 | tags=50%, list=2%, signal=51% |
| 57 | GOBP\_NEUTROPHIL\_DIFFERENTIATION |  | 5 | -0.90 | -1.74 | 0.001 | 0.150 | 1.000 | 902 | tags=80%, list=8%, signal=86% |
| 58 | GOBP\_DENSE\_CORE\_GRANULE\_TRANSPORT |  | 7 | -0.82 | -1.73 | 0.001 | 0.151 | 1.000 | 1426 | tags=86%, list=12%, signal=97% |
| 59 | GOBP\_NOSE\_DEVELOPMENT |  | 10 | -0.74 | -1.73 | 0.004 | 0.152 | 1.000 | 1871 | tags=60%, list=16%, signal=71% |
| 60 | GOMF\_FIBROBLAST\_GROWTH\_FACTOR\_RECEPTOR\_BINDING |  | 11 | -0.72 | -1.73 | 0.003 | 0.156 | 1.000 | 1146 | tags=45%, list=10%, signal=50% |
| 61 | GOBP\_LUNG\_SECRETORY\_CELL\_DIFFERENTIATION |  | 7 | -0.81 | -1.73 | 0.002 | 0.156 | 1.000 | 1871 | tags=71%, list=16%, signal=85% |
| 62 | GOBP\_REGULATION\_OF\_JUN\_KINASE\_ACTIVITY |  | 56 | -0.51 | -1.73 | 0.000 | 0.159 | 1.000 | 3142 | tags=46%, list=26%, signal=63% |
| 63 | GOBP\_REGULATION\_OF\_AXONOGENESIS |  | 112 | -0.48 | -1.73 | 0.000 | 0.158 | 1.000 | 3196 | tags=42%, list=27%, signal=57% |
| 64 | GOBP\_REGULATION\_OF\_PHOSPHATIDYLINOSITOL\_3\_KINASE\_SIGNALING |  | 88 | -0.49 | -1.73 | 0.000 | 0.159 | 1.000 | 3534 | tags=49%, list=29%, signal=69% |
| 65 | GOMF\_PHOSPHATIDYLINOSITOL\_PHOSPHATE\_4\_PHOSPHATASE\_ACTIVITY |  | 5 | -0.89 | -1.73 | 0.000 | 0.158 | 1.000 | 1322 | tags=100%, list=11%, signal=112% |
| 66 | GOBP\_PHOSPHATIDYLINOSITOL\_PHOSPHORYLATION |  | 45 | -0.53 | -1.72 | 0.000 | 0.156 | 1.000 | 2930 | tags=51%, list=24%, signal=67% |
| 67 | GOBP\_POSITIVE\_REGULATION\_OF\_CELL\_CELL\_ADHESION |  | 180 | -0.46 | -1.72 | 0.000 | 0.159 | 1.000 | 2408 | tags=39%, list=20%, signal=49% |
| 68 | GOMF\_TRANSMEMBRANE\_RECEPTOR\_PROTEIN\_TYROSINE\_KINASE\_ADAPTOR\_ACTIVITY |  | 9 | -0.75 | -1.72 | 0.004 | 0.159 | 1.000 | 747 | tags=56%, list=6%, signal=59% |
| 69 | GOBP\_NEGATIVE\_REGULATION\_OF\_BEHAVIOR |  | 7 | -0.81 | -1.72 | 0.002 | 0.158 | 1.000 | 801 | tags=57%, list=7%, signal=61% |
| 70 | GOMF\_INSULIN\_RECEPTOR\_BINDING |  | 18 | -0.64 | -1.72 | 0.004 | 0.157 | 1.000 | 2158 | tags=44%, list=18%, signal=54% |
| 71 | GOMF\_MAP\_KINASE\_KINASE\_ACTIVITY |  | 15 | -0.66 | -1.72 | 0.002 | 0.156 | 1.000 | 3510 | tags=53%, list=29%, signal=75% |
| 72 | GOMF\_MICROTUBULE\_MINUS\_END\_BINDING |  | 10 | -0.73 | -1.72 | 0.002 | 0.154 | 1.000 | 2927 | tags=80%, list=24%, signal=106% |
| 73 | GOBP\_REGULATION\_OF\_PHOSPHATIDYLINOSITOL\_3\_KINASE\_ACTIVITY |  | 43 | -0.53 | -1.72 | 0.001 | 0.155 | 1.000 | 2879 | tags=47%, list=24%, signal=61% |
| 74 | GOBP\_REGULATION\_OF\_NUCLEOTIDE\_BINDING\_OLIGOMERIZATION\_DOMAIN\_CONTAINING\_SIGNALING\_PATHWAY |  | 7 | -0.81 | -1.72 | 0.002 | 0.154 | 1.000 | 2256 | tags=71%, list=19%, signal=88% |
| 75 | GOBP\_REGULATION\_OF\_T\_CELL\_ACTIVATION |  | 200 | -0.45 | -1.72 | 0.000 | 0.153 | 1.000 | 2408 | tags=37%, list=20%, signal=46% |
| 76 | GOMF\_TAU\_PROTEIN\_BINDING |  | 34 | -0.56 | -1.72 | 0.002 | 0.152 | 1.000 | 2634 | tags=41%, list=22%, signal=53% |
| 77 | GOBP\_NEGATIVE\_REGULATION\_OF\_AXONOGENESIS |  | 49 | -0.52 | -1.72 | 0.002 | 0.152 | 1.000 | 2631 | tags=39%, list=22%, signal=49% |
| 78 | GOBP\_NEGATIVE\_REGULATION\_OF\_LEUKOCYTE\_PROLIFERATION |  | 45 | -0.53 | -1.72 | 0.002 | 0.150 | 1.000 | 3344 | tags=53%, list=28%, signal=74% |
| 79 | GOBP\_REGULATION\_OF\_T\_CELL\_DIFFERENTIATION |  | 98 | -0.48 | -1.72 | 0.000 | 0.149 | 1.000 | 2408 | tags=41%, list=20%, signal=51% |
| 80 | GOBP\_RESPONSE\_TO\_GLUCAGON |  | 27 | -0.58 | -1.71 | 0.002 | 0.155 | 1.000 | 2997 | tags=59%, list=25%, signal=79% |
| 81 | GOMF\_PHOSPHATIDYLINOSITOL\_KINASE\_ACTIVITY |  | 15 | -0.66 | -1.71 | 0.003 | 0.156 | 1.000 | 2227 | tags=67%, list=19%, signal=82% |
| 82 | GOBP\_ACTIVATION\_OF\_MAPK\_ACTIVITY |  | 107 | -0.47 | -1.71 | 0.000 | 0.156 | 1.000 | 2964 | tags=41%, list=25%, signal=54% |
| 83 | GOBP\_POSITIVE\_REGULATION\_OF\_RECEPTOR\_SIGNALING\_PATHWAY\_VIA\_STAT |  | 24 | -0.59 | -1.71 | 0.004 | 0.156 | 1.000 | 1336 | tags=38%, list=11%, signal=42% |
| 84 | GOBP\_REGULATION\_OF\_SMALL\_GTPASE\_MEDIATED\_SIGNAL\_TRANSDUCTION |  | 251 | -0.45 | -1.71 | 0.000 | 0.161 | 1.000 | 3286 | tags=45%, list=27%, signal=61% |
| 85 | GOMF\_GUANYL\_NUCLEOTIDE\_EXCHANGE\_FACTOR\_ACTIVITY |  | 153 | -0.46 | -1.71 | 0.000 | 0.160 | 1.000 | 3219 | tags=48%, list=27%, signal=65% |
| 86 | GOCC\_KINESIN\_COMPLEX |  | 38 | -0.54 | -1.71 | 0.002 | 0.159 | 1.000 | 2160 | tags=45%, list=18%, signal=54% |
| 87 | GOBP\_PEPTIDYL\_SERINE\_AUTOPHOSPHORYLATION |  | 8 | -0.78 | -1.71 | 0.003 | 0.160 | 1.000 | 2127 | tags=63%, list=18%, signal=76% |
| 88 | GOMF\_SIGNALING\_ADAPTOR\_ACTIVITY |  | 54 | -0.51 | -1.70 | 0.001 | 0.160 | 1.000 | 3825 | tags=57%, list=32%, signal=84% |
| 89 | GOBP\_REGULATION\_OF\_MODIFICATION\_OF\_POSTSYNAPTIC\_ACTIN\_CYTOSKELETON |  | 5 | -0.88 | -1.70 | 0.001 | 0.159 | 1.000 | 198 | tags=60%, list=2%, signal=61% |
| 90 | GOBP\_CELLULAR\_RESPONSE\_TO\_GLUCAGON\_STIMULUS |  | 19 | -0.62 | -1.70 | 0.003 | 0.159 | 1.000 | 1709 | tags=47%, list=14%, signal=55% |
| 91 | GOBP\_HISTONE\_H4\_K16\_ACETYLATION |  | 16 | -0.65 | -1.70 | 0.004 | 0.158 | 1.000 | 2656 | tags=50%, list=22%, signal=64% |
| 92 | GOCC\_ATG1\_ULK1\_KINASE\_COMPLEX |  | 7 | -0.79 | -1.70 | 0.002 | 0.157 | 1.000 | 2252 | tags=86%, list=19%, signal=105% |
| 93 | GOBP\_POSITIVE\_REGULATION\_OF\_CELL\_ACTIVATION |  | 214 | -0.45 | -1.70 | 0.000 | 0.156 | 1.000 | 2639 | tags=39%, list=22%, signal=49% |
| 94 | GOBP\_NEGATIVE\_REGULATION\_OF\_TUMOR\_NECROSIS\_FACTOR\_SUPERFAMILY\_CYTOKINE\_PRODUCTION |  | 27 | -0.57 | -1.70 | 0.005 | 0.157 | 1.000 | 2249 | tags=41%, list=19%, signal=50% |
| 95 | GOBP\_NEGATIVE\_REGULATION\_OF\_MICROTUBULE\_POLYMERIZATION\_OR\_DEPOLYMERIZATION |  | 25 | -0.58 | -1.70 | 0.002 | 0.156 | 1.000 | 3148 | tags=60%, list=26%, signal=81% |
| 96 | GOBP\_POSITIVE\_REGULATION\_OF\_MAST\_CELL\_ACTIVATION |  | 12 | -0.69 | -1.70 | 0.005 | 0.155 | 1.000 | 2599 | tags=50%, list=22%, signal=64% |
| 97 | GOCC\_PLASMA\_MEMBRANE\_SIGNALING\_RECEPTOR\_COMPLEX |  | 104 | -0.47 | -1.70 | 0.000 | 0.153 | 1.000 | 3856 | tags=53%, list=32%, signal=77% |
| 98 | GOBP\_REGULATION\_OF\_HOMOTYPIC\_CELL\_CELL\_ADHESION |  | 20 | -0.61 | -1.70 | 0.003 | 0.152 | 1.000 | 2769 | tags=55%, list=23%, signal=71% |
| 99 | GOBP\_HOMOTYPIC\_CELL\_CELL\_ADHESION |  | 58 | -0.50 | -1.70 | 0.000 | 0.153 | 1.000 | 4172 | tags=53%, list=35%, signal=81% |
| 100 | GOBP\_REGULATION\_OF\_ALPHA\_BETA\_T\_CELL\_ACTIVATION |  | 62 | -0.50 | -1.70 | 0.001 | 0.153 | 1.000 | 2408 | tags=45%, list=20%, signal=56% |
| 101 | GOMF\_TAU\_PROTEIN\_KINASE\_ACTIVITY |  | 16 | -0.64 | -1.70 | 0.005 | 0.152 | 1.000 | 2681 | tags=56%, list=22%, signal=72% |
| 102 | GOBP\_PROTEIN\_DEPOLYMERIZATION |  | 81 | -0.48 | -1.70 | 0.000 | 0.151 | 1.000 | 2927 | tags=43%, list=24%, signal=57% |
| 103 | GOBP\_NEURON\_PROJECTION\_GUIDANCE |  | 190 | -0.45 | -1.70 | 0.000 | 0.150 | 1.000 | 4152 | tags=48%, list=35%, signal=73% |
| 104 | GOBP\_CENTRIOLE\_CENTRIOLE\_COHESION |  | 13 | -0.68 | -1.70 | 0.002 | 0.152 | 1.000 | 2811 | tags=77%, list=23%, signal=100% |
| 105 | GOBP\_POSITIVE\_REGULATION\_OF\_DEVELOPMENTAL\_GROWTH |  | 109 | -0.47 | -1.70 | 0.000 | 0.152 | 1.000 | 3331 | tags=42%, list=28%, signal=58% |
| 106 | GOMF\_PHOSPHATIDYLINOSITOL\_3\_KINASE\_REGULATORY\_SUBUNIT\_BINDING |  | 8 | -0.77 | -1.69 | 0.002 | 0.154 | 1.000 | 2339 | tags=75%, list=19%, signal=93% |
| 107 | GOBP\_NEURON\_REMODELING |  | 9 | -0.74 | -1.69 | 0.005 | 0.155 | 1.000 | 1007 | tags=33%, list=8%, signal=36% |
| 108 | GOBP\_PLATELET\_ACTIVATION |  | 111 | -0.46 | -1.69 | 0.000 | 0.153 | 1.000 | 3588 | tags=43%, list=30%, signal=61% |
| 109 | GOMF\_PHOSPHATIDYLINOSITOL\_3\_KINASE\_BINDING |  | 25 | -0.59 | -1.69 | 0.003 | 0.153 | 1.000 | 2339 | tags=52%, list=19%, signal=64% |
| 110 | GOBP\_AXON\_DEVELOPMENT |  | 365 | -0.43 | -1.69 | 0.000 | 0.152 | 1.000 | 3565 | tags=42%, list=30%, signal=58% |
| 111 | GOCC\_RNAI\_EFFECTOR\_COMPLEX |  | 10 | -0.72 | -1.69 | 0.006 | 0.151 | 1.000 | 2080 | tags=60%, list=17%, signal=73% |
| 112 | GOBP\_NEGATIVE\_REGULATION\_OF\_COLLATERAL\_SPROUTING |  | 5 | -0.87 | -1.69 | 0.002 | 0.152 | 1.000 | 23 | tags=40%, list=0%, signal=40% |
| 113 | GOCC\_DENSE\_CORE\_GRANULE |  | 20 | -0.61 | -1.69 | 0.005 | 0.151 | 1.000 | 1298 | tags=45%, list=11%, signal=50% |
| 114 | GOCC\_AXONAL\_GROWTH\_CONE |  | 23 | -0.59 | -1.69 | 0.003 | 0.151 | 1.000 | 4025 | tags=65%, list=34%, signal=98% |
| 115 | GOBP\_POSITIVE\_REGULATION\_OF\_ORGAN\_GROWTH |  | 30 | -0.56 | -1.69 | 0.002 | 0.152 | 1.000 | 3613 | tags=50%, list=30%, signal=71% |
| 116 | GOBP\_DEVELOPMENTAL\_GROWTH\_INVOLVED\_IN\_MORPHOGENESIS |  | 162 | -0.45 | -1.69 | 0.000 | 0.153 | 1.000 | 3814 | tags=44%, list=32%, signal=64% |
| 117 | GOMF\_CYTOKINE\_RECEPTOR\_ACTIVITY |  | 71 | -0.49 | -1.69 | 0.000 | 0.151 | 1.000 | 3130 | tags=48%, list=26%, signal=64% |
| 118 | GOBP\_ALPHA\_BETA\_T\_CELL\_PROLIFERATION |  | 24 | -0.58 | -1.69 | 0.003 | 0.151 | 1.000 | 2408 | tags=50%, list=20%, signal=62% |
| 119 | GOBP\_NEURON\_PROJECTION\_EXTENSION |  | 118 | -0.46 | -1.69 | 0.000 | 0.151 | 1.000 | 3235 | tags=42%, list=27%, signal=57% |
| 120 | GOMF\_MANNOSIDASE\_ACTIVITY |  | 13 | -0.68 | -1.69 | 0.006 | 0.151 | 1.000 | 2715 | tags=62%, list=23%, signal=79% |
| 121 | GOMF\_MICROTUBULE\_BINDING |  | 194 | -0.44 | -1.69 | 0.000 | 0.151 | 1.000 | 3966 | tags=51%, list=33%, signal=74% |
| 122 | GOBP\_SMOOTH\_MUSCLE\_CELL\_MIGRATION |  | 57 | -0.50 | -1.69 | 0.002 | 0.152 | 1.000 | 3400 | tags=46%, list=28%, signal=63% |
| 123 | GOBP\_CGMP\_METABOLIC\_PROCESS |  | 8 | -0.76 | -1.69 | 0.002 | 0.152 | 1.000 | 1687 | tags=63%, list=14%, signal=73% |
| 124 | GOMF\_PHOSPHATIDYLINOSITOL\_PHOSPHATE\_PHOSPHATASE\_ACTIVITY |  | 30 | -0.56 | -1.68 | 0.002 | 0.156 | 1.000 | 3934 | tags=67%, list=33%, signal=99% |
| 125 | GOBP\_REGULATION\_OF\_VASCULAR\_ENDOTHELIAL\_GROWTH\_FACTOR\_RECEPTOR\_SIGNALING\_PATHWAY |  | 21 | -0.60 | -1.68 | 0.005 | 0.156 | 1.000 | 2337 | tags=43%, list=19%, signal=53% |
| 126 | GOMF\_EXCITATORY\_EXTRACELLULAR\_LIGAND\_GATED\_ION\_CHANNEL\_ACTIVITY |  | 11 | -0.70 | -1.68 | 0.006 | 0.156 | 1.000 | 374 | tags=36%, list=3%, signal=37% |
| 127 | GOBP\_NEURON\_PROJECTION\_ORGANIZATION |  | 67 | -0.49 | -1.68 | 0.001 | 0.154 | 1.000 | 2158 | tags=36%, list=18%, signal=43% |
| 128 | GOBP\_ANTERIOR\_POSTERIOR\_AXIS\_SPECIFICATION |  | 26 | -0.57 | -1.68 | 0.003 | 0.158 | 1.000 | 1991 | tags=42%, list=17%, signal=51% |
| 129 | GOBP\_EMBRYONIC\_CAMERA\_TYPE\_EYE\_DEVELOPMENT |  | 19 | -0.61 | -1.68 | 0.006 | 0.157 | 1.000 | 1499 | tags=37%, list=12%, signal=42% |
| 130 | GOMF\_PEPTIDE\_LYSINE\_N\_ACETYLTRANSFERASE\_ACTIVITY |  | 33 | -0.55 | -1.68 | 0.002 | 0.160 | 1.000 | 4184 | tags=55%, list=35%, signal=83% |
| 131 | GOMF\_PROTEIN\_SERINE\_THREONINE\_TYROSINE\_KINASE\_ACTIVITY |  | 35 | -0.54 | -1.68 | 0.002 | 0.159 | 1.000 | 4528 | tags=60%, list=38%, signal=96% |
| 132 | GOBP\_NEURAL\_PRECURSOR\_CELL\_PROLIFERATION |  | 95 | -0.47 | -1.68 | 0.000 | 0.158 | 1.000 | 3328 | tags=42%, list=28%, signal=58% |
| 133 | GOMF\_GLYCOSPHINGOLIPID\_BINDING |  | 4 | -0.92 | -1.68 | 0.001 | 0.158 | 1.000 | 921 | tags=100%, list=8%, signal=108% |
| 134 | GOMF\_PEPTIDE\_HORMONE\_RECEPTOR\_BINDING |  | 12 | -0.68 | -1.68 | 0.007 | 0.157 | 1.000 | 2351 | tags=67%, list=20%, signal=83% |
| 135 | GOBP\_POSITIVE\_REGULATION\_OF\_LEUKOCYTE\_DEGRANULATION |  | 13 | -0.67 | -1.67 | 0.004 | 0.162 | 1.000 | 2599 | tags=46%, list=22%, signal=59% |
| 136 | GOBP\_POSITIVE\_REGULATION\_OF\_SMALL\_GTPASE\_MEDIATED\_SIGNAL\_TRANSDUCTION |  | 59 | -0.50 | -1.67 | 0.001 | 0.162 | 1.000 | 3286 | tags=46%, list=27%, signal=63% |
| 137 | GOMF\_PHOSPHATIDYLINOSITOL\_MONOPHOSPHATE\_PHOSPHATASE\_ACTIVITY |  | 17 | -0.63 | -1.67 | 0.005 | 0.161 | 1.000 | 3533 | tags=76%, list=29%, signal=108% |
| 138 | GOBP\_POSITIVE\_REGULATION\_OF\_MAP\_KINASE\_ACTIVITY |  | 166 | -0.45 | -1.67 | 0.000 | 0.160 | 1.000 | 3557 | tags=42%, list=30%, signal=59% |
| 139 | GOMF\_CYCLIC\_NUCLEOTIDE\_PHOSPHODIESTERASE\_ACTIVITY |  | 17 | -0.62 | -1.67 | 0.006 | 0.159 | 1.000 | 2524 | tags=53%, list=21%, signal=67% |
| 140 | GOMF\_ATP\_DEPENDENT\_MICROTUBULE\_MOTOR\_ACTIVITY\_PLUS\_END\_DIRECTED |  | 12 | -0.68 | -1.67 | 0.006 | 0.161 | 1.000 | 2160 | tags=67%, list=18%, signal=81% |
| 141 | GOBP\_POSITIVE\_REGULATION\_OF\_PEPTIDYL\_TYROSINE\_PHOSPHORYLATION |  | 120 | -0.45 | -1.67 | 0.000 | 0.165 | 1.000 | 1823 | tags=33%, list=15%, signal=38% |
| 142 | GOBP\_T\_CELL\_DIFFERENTIATION |  | 170 | -0.45 | -1.67 | 0.000 | 0.165 | 1.000 | 2408 | tags=39%, list=20%, signal=48% |
| 143 | GOMF\_ATPASE\_COUPLED\_INTRAMEMBRANE\_LIPID\_TRANSPORTER\_ACTIVITY |  | 21 | -0.60 | -1.67 | 0.004 | 0.165 | 1.000 | 1410 | tags=43%, list=12%, signal=48% |
| 144 | GOBP\_CLATHRIN\_DEPENDENT\_ENDOCYTOSIS |  | 36 | -0.53 | -1.67 | 0.002 | 0.165 | 1.000 | 1677 | tags=33%, list=14%, signal=39% |
| 145 | GOCC\_NEURONAL\_DENSE\_CORE\_VESICLE |  | 11 | -0.69 | -1.67 | 0.008 | 0.164 | 1.000 | 1298 | tags=64%, list=11%, signal=71% |
| 146 | GOMF\_ACETYLCHOLINE\_GATED\_CATION\_SELECTIVE\_CHANNEL\_ACTIVITY |  | 5 | -0.86 | -1.67 | 0.002 | 0.165 | 1.000 | 205 | tags=60%, list=2%, signal=61% |
| 147 | GOCC\_RECEPTOR\_COMPLEX |  | 251 | -0.43 | -1.67 | 0.000 | 0.166 | 1.000 | 3898 | tags=47%, list=32%, signal=68% |
| 148 | GOBP\_NEUROMUSCULAR\_SYNAPTIC\_TRANSMISSION |  | 14 | -0.65 | -1.67 | 0.007 | 0.165 | 1.000 | 1131 | tags=50%, list=9%, signal=55% |
| 149 | GOBP\_CELL\_PART\_MORPHOGENESIS |  | 484 | -0.42 | -1.67 | 0.000 | 0.165 | 1.000 | 3574 | tags=42%, list=30%, signal=58% |
| 150 | GOBP\_REGULATION\_OF\_CD8\_POSITIVE\_ALPHA\_BETA\_T\_CELL\_ACTIVATION |  | 12 | -0.68 | -1.67 | 0.007 | 0.164 | 1.000 | 1601 | tags=50%, list=13%, signal=58% |
| 151 | GOMF\_SH2\_DOMAIN\_BINDING |  | 28 | -0.56 | -1.67 | 0.004 | 0.164 | 1.000 | 2879 | tags=54%, list=24%, signal=70% |
| 152 | GOBP\_LYMPHANGIOGENESIS |  | 13 | -0.67 | -1.67 | 0.007 | 0.164 | 1.000 | 540 | tags=38%, list=4%, signal=40% |
| 153 | GOBP\_SECRETORY\_GRANULE\_LOCALIZATION |  | 9 | -0.73 | -1.66 | 0.008 | 0.167 | 1.000 | 1426 | tags=67%, list=12%, signal=76% |
| 154 | GOBP\_POSITIVE\_REGULATION\_OF\_JUN\_KINASE\_ACTIVITY |  | 45 | -0.51 | -1.66 | 0.002 | 0.166 | 1.000 | 3377 | tags=49%, list=28%, signal=68% |
| 155 | GOBP\_PROTEIN\_MANNOSYLATION |  | 17 | -0.62 | -1.66 | 0.006 | 0.167 | 1.000 | 1359 | tags=41%, list=11%, signal=46% |
| 156 | GOCC\_DISTAL\_AXON |  | 196 | -0.44 | -1.66 | 0.000 | 0.168 | 1.000 | 2399 | tags=35%, list=20%, signal=43% |
| 157 | GOBP\_BONE\_MORPHOGENESIS |  | 58 | -0.49 | -1.66 | 0.001 | 0.168 | 1.000 | 3269 | tags=47%, list=27%, signal=64% |
| 158 | GOBP\_EPHRIN\_RECEPTOR\_SIGNALING\_PATHWAY |  | 69 | -0.48 | -1.66 | 0.001 | 0.167 | 1.000 | 1670 | tags=29%, list=14%, signal=33% |
| 159 | GOMF\_PROTEIN\_DEMETHYLASE\_ACTIVITY |  | 17 | -0.62 | -1.66 | 0.006 | 0.172 | 1.000 | 4327 | tags=82%, list=36%, signal=129% |
| 160 | GOBP\_LYMPHOCYTE\_COSTIMULATION |  | 44 | -0.51 | -1.66 | 0.003 | 0.171 | 1.000 | 3344 | tags=50%, list=28%, signal=69% |
| 161 | GOMF\_UDP\_XYLOSYLTRANSFERASE\_ACTIVITY |  | 8 | -0.76 | -1.66 | 0.005 | 0.170 | 1.000 | 182 | tags=38%, list=2%, signal=38% |
| 162 | GOBP\_REGULATION\_OF\_GRANULOCYTE\_CHEMOTAXIS |  | 34 | -0.54 | -1.66 | 0.003 | 0.169 | 1.000 | 3093 | tags=47%, list=26%, signal=63% |
| 163 | GOCC\_POSTSYNAPTIC\_CYTOSKELETON |  | 6 | -0.81 | -1.66 | 0.005 | 0.170 | 1.000 | 2087 | tags=67%, list=17%, signal=81% |
| 164 | GOBP\_MODIFICATION\_OF\_POSTSYNAPTIC\_ACTIN\_CYTOSKELETON |  | 8 | -0.75 | -1.66 | 0.007 | 0.171 | 1.000 | 198 | tags=38%, list=2%, signal=38% |
| 165 | GOBP\_CEREBELLAR\_GRANULAR\_LAYER\_MORPHOGENESIS |  | 7 | -0.78 | -1.66 | 0.007 | 0.172 | 1.000 | 172 | tags=29%, list=1%, signal=29% |
| 166 | GOBP\_ACTIVATION\_OF\_PROTEIN\_KINASE\_ACTIVITY |  | 240 | -0.43 | -1.66 | 0.000 | 0.171 | 1.000 | 3196 | tags=40%, list=27%, signal=53% |
| 167 | GOCC\_VOLTAGE\_GATED\_CALCIUM\_CHANNEL\_COMPLEX |  | 19 | -0.60 | -1.65 | 0.008 | 0.172 | 1.000 | 2201 | tags=37%, list=18%, signal=45% |
| 168 | GOBP\_POSITIVE\_REGULATION\_OF\_HEART\_GROWTH |  | 22 | -0.59 | -1.65 | 0.006 | 0.172 | 1.000 | 3510 | tags=59%, list=29%, signal=83% |
| 169 | GOBP\_REGULATION\_OF\_LEUKOCYTE\_DEGRANULATION |  | 25 | -0.57 | -1.65 | 0.004 | 0.172 | 1.000 | 3050 | tags=48%, list=25%, signal=64% |
| 170 | GOCC\_MAIN\_AXON |  | 40 | -0.52 | -1.65 | 0.004 | 0.177 | 1.000 | 2970 | tags=45%, list=25%, signal=60% |
| 171 | GOBP\_CELL\_PROLIFERATION\_IN\_FOREBRAIN |  | 15 | -0.63 | -1.65 | 0.009 | 0.177 | 1.000 | 2299 | tags=60%, list=19%, signal=74% |
| 172 | GOBP\_CD8\_POSITIVE\_ALPHA\_BETA\_T\_CELL\_PROLIFERATION |  | 5 | -0.85 | -1.65 | 0.004 | 0.176 | 1.000 | 770 | tags=60%, list=6%, signal=64% |
| 173 | GOBP\_POSITIVE\_REGULATION\_OF\_EXTRINSIC\_APOPTOTIC\_SIGNALING\_PATHWAY |  | 33 | -0.54 | -1.65 | 0.005 | 0.175 | 1.000 | 3392 | tags=52%, list=28%, signal=72% |
| 174 | GOMF\_MICROTUBULE\_MOTOR\_ACTIVITY |  | 45 | -0.51 | -1.65 | 0.005 | 0.175 | 1.000 | 3555 | tags=56%, list=30%, signal=79% |
| 175 | GOBP\_REGULATION\_OF\_MICROTUBULE\_DEPOLYMERIZATION |  | 19 | -0.61 | -1.65 | 0.010 | 0.175 | 1.000 | 3551 | tags=74%, list=30%, signal=104% |
| 176 | GOBP\_EMBRYONIC\_CAMERA\_TYPE\_EYE\_MORPHOGENESIS |  | 14 | -0.65 | -1.65 | 0.009 | 0.174 | 1.000 | 1499 | tags=43%, list=12%, signal=49% |
| 177 | GOMF\_1\_PHOSPHATIDYLINOSITOL\_3\_KINASE\_ACTIVITY |  | 10 | -0.71 | -1.65 | 0.011 | 0.174 | 1.000 | 1884 | tags=80%, list=16%, signal=95% |
| 178 | GOBP\_CELL\_MORPHOGENESIS\_INVOLVED\_IN\_NEURON\_DIFFERENTIATION |  | 411 | -0.42 | -1.65 | 0.000 | 0.173 | 1.000 | 3836 | tags=45%, list=32%, signal=63% |
| 179 | GOBP\_REGULATION\_OF\_CHONDROCYTE\_DIFFERENTIATION |  | 33 | -0.54 | -1.65 | 0.004 | 0.173 | 1.000 | 2002 | tags=45%, list=17%, signal=54% |
| 180 | GOBP\_N\_GLYCAN\_PROCESSING |  | 15 | -0.64 | -1.65 | 0.008 | 0.172 | 1.000 | 2949 | tags=60%, list=25%, signal=79% |
| 181 | GOMF\_FRUCTOSE\_2\_6\_BISPHOSPHATE\_2\_PHOSPHATASE\_ACTIVITY |  | 4 | -0.92 | -1.65 | 0.001 | 0.172 | 1.000 | 320 | tags=50%, list=3%, signal=51% |
| 182 | GOBP\_FRUCTOSE\_2\_6\_BISPHOSPHATE\_METABOLIC\_PROCESS |  | 4 | -0.92 | -1.65 | 0.001 | 0.172 | 1.000 | 320 | tags=50%, list=3%, signal=51% |
| 183 | GOBP\_POSITIVE\_REGULATION\_OF\_ALPHA\_BETA\_T\_CELL\_PROLIFERATION |  | 14 | -0.65 | -1.65 | 0.007 | 0.173 | 1.000 | 2408 | tags=57%, list=20%, signal=71% |
| 184 | GOBP\_CARNITINE\_METABOLIC\_PROCESS |  | 10 | -0.70 | -1.65 | 0.008 | 0.173 | 1.000 | 2431 | tags=50%, list=20%, signal=63% |
| 185 | GOBP\_TORC1\_SIGNALING |  | 36 | -0.53 | -1.65 | 0.002 | 0.172 | 1.000 | 3425 | tags=47%, list=29%, signal=66% |
| 186 | GOBP\_MICROTUBULE\_POLYMERIZATION\_OR\_DEPOLYMERIZATION |  | 78 | -0.47 | -1.65 | 0.001 | 0.172 | 1.000 | 3551 | tags=46%, list=30%, signal=65% |
| 187 | GOBP\_PHOSPHATIDYLINOSITOL\_3\_KINASE\_SIGNALING |  | 108 | -0.45 | -1.65 | 0.000 | 0.171 | 1.000 | 3613 | tags=46%, list=30%, signal=66% |
| 188 | GOBP\_PROTEIN\_LIPID\_COMPLEX\_ASSEMBLY |  | 17 | -0.62 | -1.65 | 0.007 | 0.171 | 1.000 | 3217 | tags=59%, list=27%, signal=80% |
| 189 | GOBP\_REGULATION\_OF\_CELL\_CELL\_ADHESION |  | 275 | -0.42 | -1.65 | 0.000 | 0.171 | 1.000 | 2408 | tags=35%, list=20%, signal=43% |
| 190 | GOMF\_SH3\_DOMAIN\_BINDING |  | 100 | -0.46 | -1.64 | 0.000 | 0.172 | 1.000 | 2611 | tags=40%, list=22%, signal=51% |
| 191 | GOBP\_REGULATION\_OF\_CELL\_ACTIVATION |  | 349 | -0.42 | -1.64 | 0.000 | 0.173 | 1.000 | 3344 | tags=41%, list=28%, signal=56% |
| 192 | GOBP\_MAST\_CELL\_ACTIVATION |  | 40 | -0.52 | -1.64 | 0.004 | 0.174 | 1.000 | 3062 | tags=50%, list=25%, signal=67% |
| 193 | GOBP\_STRESS\_RESPONSE\_TO\_METAL\_ION |  | 5 | -0.85 | -1.64 | 0.004 | 0.176 | 1.000 | 1583 | tags=60%, list=13%, signal=69% |
| 194 | GOMF\_ACTIVATING\_TRANSCRIPTION\_FACTOR\_BINDING |  | 62 | -0.48 | -1.64 | 0.002 | 0.176 | 1.000 | 1247 | tags=27%, list=10%, signal=30% |
| 195 | GOBP\_FC\_EPSILON\_RECEPTOR\_SIGNALING\_PATHWAY |  | 89 | -0.46 | -1.64 | 0.000 | 0.176 | 1.000 | 3143 | tags=37%, list=26%, signal=50% |
| 196 | GOBP\_INHIBITORY\_SYNAPSE\_ASSEMBLY |  | 7 | -0.78 | -1.64 | 0.007 | 0.176 | 1.000 | 488 | tags=43%, list=4%, signal=45% |
| 197 | GOMF\_DIACYLGLYCEROL\_KINASE\_ACTIVITY |  | 10 | -0.70 | -1.64 | 0.007 | 0.176 | 1.000 | 648 | tags=40%, list=5%, signal=42% |
| 198 | GOBP\_ALPHA\_BETA\_T\_CELL\_ACTIVATION |  | 91 | -0.46 | -1.64 | 0.000 | 0.177 | 1.000 | 2408 | tags=42%, list=20%, signal=52% |
| 199 | GOBP\_THYMOCYTE\_MIGRATION |  | 5 | -0.85 | -1.64 | 0.005 | 0.178 | 1.000 | 1845 | tags=100%, list=15%, signal=118% |
| 200 | GOBP\_ACTIVATION\_OF\_PROTEIN\_KINASE\_B\_ACTIVITY |  | 29 | -0.54 | -1.64 | 0.005 | 0.181 | 1.000 | 1743 | tags=31%, list=15%, signal=36% |
| 201 | GOMF\_NON\_MEMBRANE\_SPANNING\_PROTEIN\_TYROSINE\_KINASE\_ACTIVITY |  | 39 | -0.52 | -1.64 | 0.004 | 0.181 | 1.000 | 2879 | tags=49%, list=24%, signal=64% |
| 202 | GOBP\_REGULATION\_OF\_ERYTHROCYTE\_DIFFERENTIATION |  | 37 | -0.52 | -1.64 | 0.005 | 0.180 | 1.000 | 3657 | tags=54%, list=30%, signal=77% |
| 203 | GOBP\_REGULATION\_OF\_MAP\_KINASE\_ACTIVITY |  | 220 | -0.43 | -1.64 | 0.000 | 0.182 | 1.000 | 3557 | tags=42%, list=30%, signal=58% |
| 204 | GOCC\_NUCLEOTIDE\_ACTIVATED\_PROTEIN\_KINASE\_COMPLEX |  | 8 | -0.74 | -1.63 | 0.012 | 0.184 | 1.000 | 685 | tags=25%, list=6%, signal=26% |
| 205 | GOBP\_REGULATION\_OF\_PLATELET\_ACTIVATION |  | 23 | -0.57 | -1.63 | 0.006 | 0.184 | 1.000 | 3308 | tags=48%, list=28%, signal=66% |
| 206 | GOBP\_POSITIVE\_REGULATION\_OF\_ALPHA\_BETA\_T\_CELL\_ACTIVATION |  | 41 | -0.51 | -1.63 | 0.005 | 0.184 | 1.000 | 2408 | tags=46%, list=20%, signal=58% |
| 207 | GOBP\_T\_CELL\_ACTIVATION |  | 294 | -0.42 | -1.63 | 0.000 | 0.184 | 1.000 | 2608 | tags=37%, list=22%, signal=46% |
| 208 | GOBP\_POSITIVE\_REGULATION\_OF\_CD8\_POSITIVE\_ALPHA\_BETA\_T\_CELL\_ACTIVATION |  | 6 | -0.80 | -1.63 | 0.006 | 0.183 | 1.000 | 1601 | tags=67%, list=13%, signal=77% |
| 209 | GOMF\_RETINAL\_DEHYDROGENASE\_ACTIVITY |  | 6 | -0.81 | -1.63 | 0.008 | 0.185 | 1.000 | 51 | tags=33%, list=0%, signal=33% |
| 210 | GOBP\_CELL\_ADHESION\_MEDIATED\_BY\_INTEGRIN |  | 56 | -0.48 | -1.63 | 0.003 | 0.185 | 1.000 | 2955 | tags=46%, list=25%, signal=61% |
| 211 | GOBP\_LIPID\_DIGESTION |  | 9 | -0.71 | -1.63 | 0.013 | 0.184 | 1.000 | 1236 | tags=44%, list=10%, signal=50% |
| 212 | GOMF\_TUBULIN\_BINDING |  | 266 | -0.42 | -1.63 | 0.000 | 0.185 | 1.000 | 3834 | tags=46%, list=32%, signal=66% |
| 213 | GOBP\_CELLULAR\_COMPONENT\_MORPHOGENESIS |  | 535 | -0.41 | -1.63 | 0.000 | 0.189 | 1.000 | 3574 | tags=41%, list=30%, signal=56% |
| 214 | GOBP\_FC\_RECEPTOR\_MEDIATED\_STIMULATORY\_SIGNALING\_PATHWAY |  | 70 | -0.47 | -1.63 | 0.002 | 0.189 | 1.000 | 3069 | tags=43%, list=26%, signal=57% |
| 215 | GOBP\_REGULATION\_OF\_PLATELET\_AGGREGATION |  | 14 | -0.64 | -1.63 | 0.012 | 0.188 | 1.000 | 2357 | tags=50%, list=20%, signal=62% |
| 216 | GOBP\_INOSITOL\_LIPID\_MEDIATED\_SIGNALING |  | 139 | -0.44 | -1.63 | 0.000 | 0.187 | 1.000 | 3613 | tags=47%, list=30%, signal=66% |
| 217 | GOMF\_PHOSPHOPROTEIN\_BINDING |  | 69 | -0.47 | -1.63 | 0.001 | 0.187 | 1.000 | 3974 | tags=54%, list=33%, signal=80% |
| 218 | GOBP\_REGULATION\_OF\_EXTRINSIC\_APOPTOTIC\_SIGNALING\_PATHWAY\_VIA\_DEATH\_DOMAIN\_RECEPTORS |  | 42 | -0.51 | -1.63 | 0.006 | 0.188 | 1.000 | 2820 | tags=50%, list=23%, signal=65% |
| 219 | GOBP\_PRIMITIVE\_STREAK\_FORMATION |  | 5 | -0.84 | -1.63 | 0.005 | 0.188 | 1.000 | 405 | tags=40%, list=3%, signal=41% |
| 220 | GOBP\_POSITIVE\_REGULATION\_OF\_INTERFERON\_GAMMA\_PRODUCTION |  | 34 | -0.53 | -1.63 | 0.005 | 0.187 | 1.000 | 3487 | tags=56%, list=29%, signal=79% |
| 221 | GOMF\_PHOSPHATIDYLINOSITOL\_BISPHOSPHATE\_KINASE\_ACTIVITY |  | 7 | -0.76 | -1.63 | 0.010 | 0.188 | 1.000 | 1869 | tags=86%, list=16%, signal=101% |
| 222 | GOBP\_SKELETAL\_SYSTEM\_MORPHOGENESIS |  | 132 | -0.44 | -1.63 | 0.000 | 0.188 | 1.000 | 4111 | tags=50%, list=34%, signal=75% |
| 223 | GOBP\_PROTEIN\_DEMETHYLATION |  | 20 | -0.59 | -1.63 | 0.007 | 0.187 | 1.000 | 4327 | tags=75%, list=36%, signal=117% |
| 224 | GOBP\_REGULATION\_OF\_B\_CELL\_DIFFERENTIATION |  | 20 | -0.59 | -1.63 | 0.010 | 0.187 | 1.000 | 3326 | tags=55%, list=28%, signal=76% |
| 225 | GOMF\_DIOXYGENASE\_ACTIVITY |  | 58 | -0.48 | -1.63 | 0.002 | 0.187 | 1.000 | 2716 | tags=41%, list=23%, signal=53% |
| 226 | GOBP\_GROWTH\_HORMONE\_RECEPTOR\_SIGNALING\_PATHWAY\_VIA\_JAK\_STAT |  | 9 | -0.71 | -1.63 | 0.011 | 0.186 | 1.000 | 960 | tags=44%, list=8%, signal=48% |
| 227 | GOBP\_CRANIOFACIAL\_SUTURE\_MORPHOGENESIS |  | 10 | -0.69 | -1.63 | 0.011 | 0.185 | 1.000 | 3100 | tags=70%, list=26%, signal=94% |
| 228 | GOBP\_REGULATION\_OF\_FEEDING\_BEHAVIOR |  | 12 | -0.66 | -1.62 | 0.011 | 0.188 | 1.000 | 2340 | tags=58%, list=19%, signal=72% |
| 229 | GOBP\_CAMERA\_TYPE\_EYE\_MORPHOGENESIS |  | 74 | -0.46 | -1.62 | 0.002 | 0.189 | 1.000 | 1520 | tags=30%, list=13%, signal=34% |
| 230 | GOMF\_VOLTAGE\_GATED\_CALCIUM\_CHANNEL\_ACTIVITY\_INVOLVED\_IN\_CARDIAC\_MUSCLE\_CELL\_ACTION\_POTENTIAL |  | 5 | -0.84 | -1.62 | 0.006 | 0.190 | 1.000 | 69 | tags=20%, list=1%, signal=20% |
| 231 | GOMF\_PROTEIN\_TYROSINE\_KINASE\_ACTIVITY |  | 111 | -0.44 | -1.62 | 0.000 | 0.192 | 1.000 | 3751 | tags=44%, list=31%, signal=64% |
| 232 | GOBP\_NEGATIVE\_REGULATION\_OF\_CELL\_ADHESION |  | 186 | -0.43 | -1.62 | 0.000 | 0.192 | 1.000 | 3724 | tags=45%, list=31%, signal=64% |
| 233 | GOBP\_PHOSPHATIDYLINOSITOL\_DEPHOSPHORYLATION |  | 27 | -0.55 | -1.62 | 0.007 | 0.192 | 1.000 | 3934 | tags=67%, list=33%, signal=99% |
| 234 | GOBP\_LUNG\_ALVEOLUS\_DEVELOPMENT |  | 28 | -0.54 | -1.62 | 0.009 | 0.192 | 1.000 | 2515 | tags=50%, list=21%, signal=63% |
| 235 | GOBP\_AXON\_EXTENSION |  | 84 | -0.46 | -1.62 | 0.001 | 0.191 | 1.000 | 3235 | tags=43%, list=27%, signal=58% |
| 236 | GOBP\_TUMOR\_NECROSIS\_FACTOR\_SUPERFAMILY\_CYTOKINE\_PRODUCTION |  | 79 | -0.46 | -1.62 | 0.000 | 0.193 | 1.000 | 2357 | tags=35%, list=20%, signal=44% |
| 237 | GOBP\_NATURAL\_KILLER\_CELL\_ACTIVATION |  | 35 | -0.52 | -1.62 | 0.007 | 0.193 | 1.000 | 3009 | tags=49%, list=25%, signal=65% |
| 238 | GOBP\_REGULATION\_OF\_DENDRITIC\_SPINE\_MAINTENANCE |  | 6 | -0.80 | -1.62 | 0.007 | 0.193 | 1.000 | 1495 | tags=67%, list=12%, signal=76% |
| 239 | GOBP\_CYCLIC\_NUCLEOTIDE\_BIOSYNTHETIC\_PROCESS |  | 10 | -0.69 | -1.62 | 0.012 | 0.193 | 1.000 | 1687 | tags=50%, list=14%, signal=58% |
| 240 | GOBP\_BONE\_DEVELOPMENT |  | 134 | -0.44 | -1.62 | 0.000 | 0.193 | 1.000 | 3100 | tags=43%, list=26%, signal=58% |
| 241 | GOBP\_ECTODERMAL\_PLACODE\_FORMATION |  | 7 | -0.76 | -1.62 | 0.009 | 0.194 | 1.000 | 295 | tags=43%, list=2%, signal=44% |
| 242 | GOBP\_REGULATION\_OF\_LYMPHOCYTE\_ACTIVATION |  | 275 | -0.42 | -1.62 | 0.000 | 0.196 | 1.000 | 3205 | tags=41%, list=27%, signal=54% |
| 243 | GOCC\_IMMUNOLOGICAL\_SYNAPSE |  | 28 | -0.54 | -1.62 | 0.009 | 0.196 | 1.000 | 2604 | tags=46%, list=22%, signal=59% |
| 244 | GOBP\_REGULATION\_OF\_NEUTROPHIL\_CHEMOTAXIS |  | 24 | -0.56 | -1.62 | 0.012 | 0.195 | 1.000 | 1988 | tags=38%, list=17%, signal=45% |
| 245 | GOMF\_SUMO\_TRANSFERASE\_ACTIVITY |  | 12 | -0.66 | -1.61 | 0.014 | 0.198 | 1.000 | 1520 | tags=42%, list=13%, signal=48% |
| 246 | GOBP\_FC\_RECEPTOR\_SIGNALING\_PATHWAY |  | 142 | -0.43 | -1.61 | 0.000 | 0.198 | 1.000 | 3143 | tags=35%, list=26%, signal=47% |
| 247 | GOBP\_NUCLEOTIDE\_BINDING\_DOMAIN\_LEUCINE\_RICH\_REPEAT\_CONTAINING\_RECEPTOR\_SIGNALING\_PATHWAY |  | 28 | -0.54 | -1.61 | 0.011 | 0.200 | 1.000 | 2894 | tags=43%, list=24%, signal=56% |
| 248 | GOBP\_REGULATION\_OF\_EXTRINSIC\_APOPTOTIC\_SIGNALING\_PATHWAY |  | 107 | -0.44 | -1.61 | 0.000 | 0.200 | 1.000 | 2820 | tags=39%, list=23%, signal=51% |
| 249 | GOBP\_B\_CELL\_HOMEOSTASIS |  | 22 | -0.57 | -1.61 | 0.011 | 0.199 | 1.000 | 2474 | tags=50%, list=21%, signal=63% |
| 250 | GOBP\_DENDRITE\_EXTENSION |  | 25 | -0.56 | -1.61 | 0.008 | 0.198 | 1.000 | 1360 | tags=36%, list=11%, signal=41% |
| 251 | GOBP\_MULTICELLULAR\_ORGANISMAL\_RESPONSE\_TO\_STRESS |  | 44 | -0.50 | -1.61 | 0.004 | 0.198 | 1.000 | 4572 | tags=61%, list=38%, signal=99% |
| 252 | GOBP\_REGULATION\_OF\_INTESTINAL\_CHOLESTEROL\_ABSORPTION |  | 6 | -0.80 | -1.61 | 0.005 | 0.200 | 1.000 | 1109 | tags=50%, list=9%, signal=55% |
| 253 | GOBP\_POSITIVE\_REGULATION\_OF\_SYNAPTIC\_TRANSMISSION |  | 83 | -0.45 | -1.61 | 0.002 | 0.201 | 1.000 | 3699 | tags=49%, list=31%, signal=71% |
| 254 | GOBP\_POSITIVE\_REGULATION\_OF\_NATURAL\_KILLER\_CELL\_ACTIVATION |  | 9 | -0.71 | -1.61 | 0.013 | 0.201 | 1.000 | 1903 | tags=44%, list=16%, signal=53% |
| 255 | GOMF\_PROTEIN\_SERINE\_KINASE\_ACTIVITY |  | 178 | -0.43 | -1.61 | 0.000 | 0.201 | 1.000 | 3954 | tags=53%, list=33%, signal=78% |
| 256 | GOMF\_GTPASE\_ACTIVATOR\_ACTIVITY |  | 206 | -0.42 | -1.61 | 0.000 | 0.200 | 1.000 | 4324 | tags=55%, list=36%, signal=84% |
| 257 | GOBP\_EATING\_BEHAVIOR |  | 17 | -0.60 | -1.61 | 0.015 | 0.200 | 1.000 | 1743 | tags=59%, list=15%, signal=69% |
| 258 | GOBP\_NEUROBLAST\_PROLIFERATION |  | 40 | -0.51 | -1.61 | 0.007 | 0.200 | 1.000 | 4457 | tags=63%, list=37%, signal=99% |
| 259 | GOBP\_LYMPH\_NODE\_DEVELOPMENT |  | 14 | -0.63 | -1.61 | 0.013 | 0.200 | 1.000 | 1951 | tags=57%, list=16%, signal=68% |
| 260 | GOBP\_EXTRINSIC\_APOPTOTIC\_SIGNALING\_PATHWAY\_VIA\_DEATH\_DOMAIN\_RECEPTORS |  | 56 | -0.48 | -1.61 | 0.004 | 0.199 | 1.000 | 2820 | tags=45%, list=23%, signal=58% |
| 261 | GOBP\_AMINO\_ACID\_BETAINE\_BIOSYNTHETIC\_PROCESS |  | 6 | -0.79 | -1.61 | 0.007 | 0.199 | 1.000 | 2534 | tags=83%, list=21%, signal=106% |
| 262 | GOBP\_POSITIVE\_REGULATION\_OF\_CHONDROCYTE\_DIFFERENTIATION |  | 11 | -0.67 | -1.61 | 0.014 | 0.199 | 1.000 | 2002 | tags=64%, list=17%, signal=76% |
| 263 | GOMF\_NETRIN\_RECEPTOR\_ACTIVITY |  | 4 | -0.89 | -1.61 | 0.004 | 0.199 | 1.000 | 223 | tags=50%, list=2%, signal=51% |
| 264 | GOBP\_REGULATION\_OF\_INTESTINAL\_ABSORPTION |  | 7 | -0.75 | -1.61 | 0.009 | 0.201 | 1.000 | 1109 | tags=43%, list=9%, signal=47% |
| 265 | GOBP\_EMBRYONIC\_PATTERN\_SPECIFICATION |  | 34 | -0.52 | -1.61 | 0.006 | 0.200 | 1.000 | 2002 | tags=35%, list=17%, signal=42% |
| 266 | GOBP\_REGULATION\_OF\_LEUKOCYTE\_PROLIFERATION |  | 142 | -0.43 | -1.61 | 0.000 | 0.201 | 1.000 | 3163 | tags=44%, list=26%, signal=59% |
| 267 | GOBP\_REGULATION\_OF\_MAINTENANCE\_OF\_SISTER\_CHROMATID\_COHESION |  | 6 | -0.78 | -1.61 | 0.011 | 0.202 | 1.000 | 2520 | tags=83%, list=21%, signal=105% |
| 268 | GOCC\_PERINUCLEAR\_ENDOPLASMIC\_RETICULUM |  | 18 | -0.59 | -1.60 | 0.014 | 0.203 | 1.000 | 747 | tags=28%, list=6%, signal=30% |
| 269 | GOBP\_SMALL\_GTPASE\_MEDIATED\_SIGNAL\_TRANSDUCTION |  | 383 | -0.41 | -1.60 | 0.000 | 0.204 | 1.000 | 3286 | tags=40%, list=27%, signal=54% |
| 270 | GOBP\_REGULATION\_OF\_MAST\_CELL\_ACTIVATION |  | 26 | -0.55 | -1.60 | 0.011 | 0.204 | 1.000 | 3062 | tags=46%, list=25%, signal=62% |
| 271 | GOBP\_GAMMA\_DELTA\_T\_CELL\_ACTIVATION |  | 11 | -0.67 | -1.60 | 0.012 | 0.203 | 1.000 | 2357 | tags=55%, list=20%, signal=68% |
| 272 | GOBP\_SECONDARY\_PALATE\_DEVELOPMENT |  | 14 | -0.63 | -1.60 | 0.015 | 0.205 | 1.000 | 2604 | tags=50%, list=22%, signal=64% |
| 273 | GOMF\_MANNOSYL\_OLIGOSACCHARIDE\_MANNOSIDASE\_ACTIVITY |  | 9 | -0.71 | -1.60 | 0.011 | 0.205 | 1.000 | 2715 | tags=67%, list=23%, signal=86% |
| 274 | GOBP\_POSITIVE\_REGULATION\_OF\_POSTTRANSCRIPTIONAL\_GENE\_SILENCING |  | 23 | -0.56 | -1.60 | 0.009 | 0.210 | 1.000 | 2384 | tags=39%, list=20%, signal=49% |
| 275 | GOBP\_REGULATION\_OF\_LYMPHOCYTE\_APOPTOTIC\_PROCESS |  | 35 | -0.51 | -1.60 | 0.007 | 0.210 | 1.000 | 1852 | tags=40%, list=15%, signal=47% |
| 276 | GOBP\_NEGATIVE\_REGULATION\_OF\_LYMPHOCYTE\_ACTIVATION |  | 87 | -0.45 | -1.60 | 0.001 | 0.210 | 1.000 | 2604 | tags=39%, list=22%, signal=50% |
| 277 | GOCC\_ACETYLCHOLINE\_GATED\_CHANNEL\_COMPLEX |  | 6 | -0.79 | -1.60 | 0.012 | 0.210 | 1.000 | 1298 | tags=67%, list=11%, signal=75% |
| 278 | GOMF\_TRANSMITTER\_GATED\_CHANNEL\_ACTIVITY |  | 14 | -0.62 | -1.60 | 0.011 | 0.210 | 1.000 | 2005 | tags=50%, list=17%, signal=60% |
| 279 | GOBP\_RAC\_PROTEIN\_SIGNAL\_TRANSDUCTION |  | 32 | -0.53 | -1.60 | 0.009 | 0.210 | 1.000 | 3797 | tags=66%, list=32%, signal=96% |
| 280 | GOBP\_POSITIVE\_REGULATION\_OF\_EXTRINSIC\_APOPTOTIC\_SIGNALING\_PATHWAY\_VIA\_DEATH\_DOMAIN\_RECEPTORS |  | 10 | -0.68 | -1.60 | 0.018 | 0.209 | 1.000 | 3841 | tags=90%, list=32%, signal=132% |
| 281 | GOBP\_REGULATION\_OF\_DEVELOPMENTAL\_GROWTH |  | 208 | -0.42 | -1.60 | 0.000 | 0.209 | 1.000 | 3407 | tags=40%, list=28%, signal=55% |
| 282 | GOBP\_NATURAL\_KILLER\_CELL\_MEDIATED\_IMMUNITY |  | 21 | -0.57 | -1.60 | 0.014 | 0.209 | 1.000 | 2547 | tags=43%, list=21%, signal=54% |
| 283 | GOBP\_AMMONIUM\_ION\_METABOLIC\_PROCESS |  | 16 | -0.60 | -1.60 | 0.014 | 0.210 | 1.000 | 2768 | tags=56%, list=23%, signal=73% |
| 284 | GOBP\_REGULATION\_OF\_PEPTIDYL\_TYROSINE\_PHOSPHORYLATION |  | 166 | -0.42 | -1.60 | 0.000 | 0.210 | 1.000 | 1605 | tags=27%, list=13%, signal=31% |
| 285 | GOMF\_PHOSPHATIDYLETHANOLAMINE\_FLIPPASE\_ACTIVITY |  | 4 | -0.88 | -1.60 | 0.005 | 0.211 | 1.000 | 1410 | tags=100%, list=12%, signal=113% |
| 286 | GOBP\_MITRAL\_VALVE\_DEVELOPMENT |  | 10 | -0.68 | -1.60 | 0.014 | 0.211 | 1.000 | 2933 | tags=50%, list=24%, signal=66% |
| 287 | GOBP\_REGULATION\_OF\_MITOTIC\_CENTROSOME\_SEPARATION |  | 9 | -0.69 | -1.60 | 0.013 | 0.211 | 1.000 | 1488 | tags=33%, list=12%, signal=38% |
| 288 | GOBP\_POSITIVE\_REGULATION\_OF\_CELL\_ADHESION |  | 289 | -0.41 | -1.60 | 0.000 | 0.210 | 1.000 | 2488 | tags=34%, list=21%, signal=42% |
| 289 | GOBP\_HISTONE\_H4\_ACETYLATION |  | 53 | -0.48 | -1.60 | 0.004 | 0.211 | 1.000 | 3395 | tags=45%, list=28%, signal=63% |
| 290 | GOMF\_QUATERNARY\_AMMONIUM\_GROUP\_TRANSMEMBRANE\_TRANSPORTER\_ACTIVITY |  | 6 | -0.79 | -1.59 | 0.010 | 0.212 | 1.000 | 2557 | tags=100%, list=21%, signal=127% |
| 291 | GOBP\_KINETOCHORE\_ORGANIZATION |  | 21 | -0.57 | -1.59 | 0.012 | 0.212 | 1.000 | 4279 | tags=81%, list=36%, signal=126% |
| 292 | GOBP\_PHOSPHATIDYLINOSITOL\_BIOSYNTHETIC\_PROCESS |  | 100 | -0.44 | -1.59 | 0.001 | 0.213 | 1.000 | 4600 | tags=54%, list=38%, signal=87% |
| 293 | GOBP\_POSITIVE\_REGULATION\_OF\_GASTRO\_INTESTINAL\_SYSTEM\_SMOOTH\_MUSCLE\_CONTRACTION |  | 3 | -0.96 | -1.59 | 0.000 | 0.212 | 1.000 | 199 | tags=67%, list=2%, signal=68% |
| 294 | GOBP\_ESTABLISHMENT\_OF\_LYMPHOCYTE\_POLARITY |  | 9 | -0.70 | -1.59 | 0.012 | 0.213 | 1.000 | 1843 | tags=44%, list=15%, signal=52% |
| 295 | GOMF\_1\_PHOSPHATIDYLINOSITOL\_3\_KINASE\_REGULATOR\_ACTIVITY |  | 13 | -0.63 | -1.59 | 0.017 | 0.215 | 1.000 | 2670 | tags=69%, list=22%, signal=89% |
| 296 | GOBP\_COMMISSURAL\_NEURON\_AXON\_GUIDANCE |  | 10 | -0.68 | -1.59 | 0.015 | 0.215 | 1.000 | 3196 | tags=70%, list=27%, signal=95% |
| 297 | GOBP\_SPLEEN\_DEVELOPMENT |  | 27 | -0.54 | -1.59 | 0.012 | 0.214 | 1.000 | 1187 | tags=30%, list=10%, signal=33% |
| 298 | GOBP\_PIRNA\_BIOSYNTHETIC\_PROCESS |  | 3 | -0.95 | -1.59 | 0.002 | 0.214 | 1.000 | 529 | tags=67%, list=4%, signal=70% |
| 299 | GOCC\_AXON\_INITIAL\_SEGMENT |  | 10 | -0.68 | -1.59 | 0.017 | 0.216 | 1.000 | 1638 | tags=60%, list=14%, signal=69% |
| 300 | GOBP\_LEUKOCYTE\_CELL\_CELL\_ADHESION |  | 218 | -0.42 | -1.59 | 0.000 | 0.215 | 1.000 | 2408 | tags=35%, list=20%, signal=43% |
| 301 | GOMF\_FLIPPASE\_ACTIVITY |  | 13 | -0.63 | -1.59 | 0.016 | 0.215 | 1.000 | 1410 | tags=46%, list=12%, signal=52% |
| 302 | GOBP\_POSITIVE\_REGULATION\_OF\_INTERLEUKIN\_8\_PRODUCTION |  | 33 | -0.51 | -1.59 | 0.007 | 0.217 | 1.000 | 2480 | tags=42%, list=21%, signal=53% |
| 303 | GOMF\_T\_CELL\_RECEPTOR\_BINDING |  | 4 | -0.88 | -1.59 | 0.006 | 0.217 | 1.000 | 1386 | tags=100%, list=12%, signal=113% |
| 304 | GOCC\_COLLAGEN\_TRIMER |  | 51 | -0.48 | -1.59 | 0.004 | 0.217 | 1.000 | 2486 | tags=35%, list=21%, signal=44% |
| 305 | GOMF\_ANION\_CHANNEL\_ACTIVITY |  | 37 | -0.50 | -1.59 | 0.009 | 0.219 | 1.000 | 3220 | tags=51%, list=27%, signal=70% |
| 306 | GOMF\_SOLUTE\_SODIUM\_SYMPORTER\_ACTIVITY |  | 42 | -0.50 | -1.59 | 0.007 | 0.219 | 1.000 | 1668 | tags=40%, list=14%, signal=47% |
| 307 | GOBP\_NEGATIVE\_REGULATION\_OF\_LYMPHOCYTE\_APOPTOTIC\_PROCESS |  | 22 | -0.56 | -1.59 | 0.012 | 0.219 | 1.000 | 2362 | tags=50%, list=20%, signal=62% |
| 308 | GOBP\_STARTLE\_RESPONSE |  | 10 | -0.68 | -1.59 | 0.019 | 0.219 | 1.000 | 2563 | tags=80%, list=21%, signal=102% |
| 309 | GOBP\_MICROTUBULE\_POLYMERIZATION |  | 56 | -0.47 | -1.59 | 0.005 | 0.219 | 1.000 | 3148 | tags=41%, list=26%, signal=55% |
| 310 | GOMF\_PROTEIN\_KINASE\_ACTIVITY |  | 416 | -0.40 | -1.59 | 0.000 | 0.220 | 1.000 | 3781 | tags=45%, list=31%, signal=63% |
| 311 | GOBP\_NEGATIVE\_REGULATION\_OF\_CELL\_ACTIVATION |  | 119 | -0.43 | -1.59 | 0.001 | 0.220 | 1.000 | 2345 | tags=34%, list=20%, signal=41% |
| 312 | GOBP\_POSITIVE\_REGULATION\_OF\_PLASMA\_MEMBRANE\_BOUNDED\_CELL\_PROJECTION\_ASSEMBLY |  | 75 | -0.46 | -1.58 | 0.003 | 0.221 | 1.000 | 2757 | tags=36%, list=23%, signal=46% |
| 313 | GOBP\_MANNOSYLATION |  | 27 | -0.54 | -1.58 | 0.010 | 0.220 | 1.000 | 1497 | tags=33%, list=12%, signal=38% |
| 314 | GOMF\_2\_OXOGLUTARATE\_DEPENDENT\_DIOXYGENASE\_ACTIVITY |  | 37 | -0.50 | -1.58 | 0.010 | 0.220 | 1.000 | 2716 | tags=43%, list=23%, signal=56% |
| 315 | GOBP\_HINDBRAIN\_MORPHOGENESIS |  | 29 | -0.53 | -1.58 | 0.011 | 0.221 | 1.000 | 1452 | tags=28%, list=12%, signal=31% |
| 316 | GOBP\_DETECTION\_OF\_BIOTIC\_STIMULUS |  | 19 | -0.57 | -1.58 | 0.012 | 0.221 | 1.000 | 3195 | tags=53%, list=27%, signal=72% |
| 317 | GOBP\_INTERFERON\_GAMMA\_PRODUCTION |  | 57 | -0.47 | -1.58 | 0.005 | 0.223 | 1.000 | 3487 | tags=49%, list=29%, signal=69% |
| 318 | GOBP\_POSITIVE\_REGULATION\_OF\_AXONOGENESIS |  | 58 | -0.47 | -1.58 | 0.005 | 0.222 | 1.000 | 3196 | tags=43%, list=27%, signal=58% |
| 319 | GOMF\_MODIFIED\_AMINO\_ACID\_BINDING |  | 63 | -0.46 | -1.58 | 0.005 | 0.224 | 1.000 | 2050 | tags=30%, list=17%, signal=36% |
| 320 | GOBP\_ANATOMICAL\_STRUCTURE\_ARRANGEMENT |  | 9 | -0.69 | -1.58 | 0.018 | 0.225 | 1.000 | 1143 | tags=44%, list=10%, signal=49% |
| 321 | GOBP\_VENOUS\_BLOOD\_VESSEL\_DEVELOPMENT |  | 11 | -0.66 | -1.58 | 0.016 | 0.225 | 1.000 | 3256 | tags=55%, list=27%, signal=75% |
| 322 | GOBP\_CELL\_MORPHOGENESIS\_INVOLVED\_IN\_DIFFERENTIATION |  | 520 | -0.40 | -1.58 | 0.000 | 0.225 | 1.000 | 4112 | tags=45%, list=34%, signal=66% |
| 323 | GOMF\_ALDEHYDE\_DEHYDROGENASE\_NAD\_P\_PLUS\_ACTIVITY |  | 10 | -0.68 | -1.58 | 0.015 | 0.225 | 1.000 | 2534 | tags=50%, list=21%, signal=63% |
| 324 | GOBP\_LEUKOCYTE\_PROLIFERATION |  | 180 | -0.42 | -1.58 | 0.000 | 0.225 | 1.000 | 3165 | tags=43%, list=26%, signal=57% |
| 325 | GOCC\_PERICENTRIOLAR\_MATERIAL |  | 15 | -0.60 | -1.58 | 0.018 | 0.225 | 1.000 | 2299 | tags=53%, list=19%, signal=66% |
| 326 | GOBP\_POSITIVE\_REGULATION\_OF\_MIRNA\_MEDIATED\_INHIBITION\_OF\_TRANSLATION |  | 6 | -0.78 | -1.58 | 0.012 | 0.225 | 1.000 | 2080 | tags=67%, list=17%, signal=81% |
| 327 | GOCC\_DENSE\_CORE\_GRANULE\_MEMBRANE |  | 6 | -0.78 | -1.58 | 0.012 | 0.226 | 1.000 | 1298 | tags=67%, list=11%, signal=75% |
| 328 | GOCC\_CILIARY\_TRANSITION\_FIBER |  | 8 | -0.72 | -1.58 | 0.016 | 0.228 | 1.000 | 2521 | tags=75%, list=21%, signal=95% |
| 329 | GOBP\_HOMEOSTASIS\_OF\_NUMBER\_OF\_CELLS |  | 187 | -0.42 | -1.58 | 0.000 | 0.228 | 1.000 | 4104 | tags=50%, list=34%, signal=75% |
| 330 | GOBP\_REGULATION\_OF\_NATURAL\_KILLER\_CELL\_MEDIATED\_IMMUNITY |  | 13 | -0.63 | -1.58 | 0.015 | 0.227 | 1.000 | 3285 | tags=62%, list=27%, signal=85% |
| 331 | GOMF\_H4\_HISTONE\_ACETYLTRANSFERASE\_ACTIVITY |  | 7 | -0.74 | -1.58 | 0.013 | 0.227 | 1.000 | 1247 | tags=29%, list=10%, signal=32% |
| 332 | GOMF\_CALCIUM\_CATION\_ANTIPORTER\_ACTIVITY |  | 5 | -0.82 | -1.58 | 0.008 | 0.226 | 1.000 | 933 | tags=40%, list=8%, signal=43% |
| 333 | GOMF\_INSULIN\_BINDING |  | 5 | -0.82 | -1.58 | 0.013 | 0.226 | 1.000 | 2218 | tags=100%, list=18%, signal=123% |
| 334 | GOMF\_PEPTIDE\_N\_ACETYLTRANSFERASE\_ACTIVITY |  | 38 | -0.50 | -1.58 | 0.010 | 0.225 | 1.000 | 4184 | tags=50%, list=35%, signal=76% |
| 335 | GOBP\_PEPTIDYL\_TYROSINE\_MODIFICATION |  | 256 | -0.41 | -1.58 | 0.000 | 0.225 | 1.000 | 2654 | tags=32%, list=22%, signal=41% |
| 336 | GOCC\_AXON |  | 417 | -0.40 | -1.58 | 0.000 | 0.226 | 1.000 | 2638 | tags=34%, list=22%, signal=42% |
| 337 | GOBP\_REGULATION\_OF\_ACTIN\_FILAMENT\_BASED\_MOVEMENT |  | 23 | -0.55 | -1.58 | 0.017 | 0.226 | 1.000 | 3918 | tags=57%, list=33%, signal=84% |
| 338 | GOBP\_CELLULAR\_RESPONSE\_TO\_GAMMA\_RADIATION |  | 20 | -0.56 | -1.58 | 0.016 | 0.225 | 1.000 | 3927 | tags=75%, list=33%, signal=111% |
| 339 | GOBP\_REGULATION\_OF\_GTPASE\_ACTIVITY |  | 358 | -0.41 | -1.58 | 0.000 | 0.226 | 1.000 | 3530 | tags=43%, list=29%, signal=59% |
| 340 | GOBP\_CAMP\_MEDIATED\_SIGNALING |  | 43 | -0.49 | -1.58 | 0.007 | 0.226 | 1.000 | 1939 | tags=37%, list=16%, signal=44% |
| 341 | GOBP\_POSITIVE\_REGULATION\_OF\_B\_CELL\_DIFFERENTIATION |  | 9 | -0.69 | -1.58 | 0.014 | 0.226 | 1.000 | 3326 | tags=67%, list=28%, signal=92% |
| 342 | GOBP\_TONGUE\_DEVELOPMENT |  | 10 | -0.67 | -1.57 | 0.018 | 0.226 | 1.000 | 624 | tags=40%, list=5%, signal=42% |
| 343 | GOBP\_MHC\_CLASS\_II\_BIOSYNTHETIC\_PROCESS |  | 10 | -0.67 | -1.57 | 0.017 | 0.226 | 1.000 | 3395 | tags=80%, list=28%, signal=111% |
| 344 | GOBP\_REGULATION\_OF\_RHO\_PROTEIN\_SIGNAL\_TRANSDUCTION |  | 62 | -0.47 | -1.57 | 0.004 | 0.226 | 1.000 | 3493 | tags=45%, list=29%, signal=63% |
| 345 | GOBP\_TAXIS |  | 405 | -0.40 | -1.57 | 0.000 | 0.225 | 1.000 | 3588 | tags=41%, list=30%, signal=56% |
| 346 | GOMF\_NEUROTROPHIN\_TRK\_RECEPTOR\_BINDING |  | 6 | -0.77 | -1.57 | 0.013 | 0.226 | 1.000 | 747 | tags=50%, list=6%, signal=53% |
| 347 | GOBP\_MICTURITION |  | 4 | -0.87 | -1.57 | 0.007 | 0.225 | 1.000 | 205 | tags=50%, list=2%, signal=51% |
| 348 | GOCC\_CHLORIDE\_CHANNEL\_COMPLEX |  | 16 | -0.59 | -1.57 | 0.019 | 0.227 | 1.000 | 2102 | tags=50%, list=18%, signal=61% |
| 349 | GOMF\_INTRAMEMBRANE\_LIPID\_TRANSPORTER\_ACTIVITY |  | 23 | -0.55 | -1.57 | 0.017 | 0.226 | 1.000 | 1410 | tags=39%, list=12%, signal=44% |
| 350 | GOBP\_REGULATION\_OF\_LAMELLIPODIUM\_MORPHOGENESIS |  | 9 | -0.69 | -1.57 | 0.016 | 0.226 | 1.000 | 591 | tags=33%, list=5%, signal=35% |
| 351 | GOBP\_HISTONE\_MRNA\_CATABOLIC\_PROCESS |  | 8 | -0.72 | -1.57 | 0.015 | 0.225 | 1.000 | 1756 | tags=50%, list=15%, signal=59% |
| 352 | GOBP\_SYNAPSE\_MATURATION |  | 16 | -0.60 | -1.57 | 0.015 | 0.228 | 1.000 | 3431 | tags=44%, list=29%, signal=61% |
| 353 | GOMF\_EXTRACELLULAR\_MATRIX\_STRUCTURAL\_CONSTITUENT |  | 107 | -0.43 | -1.57 | 0.002 | 0.228 | 1.000 | 3186 | tags=38%, list=27%, signal=52% |
| 354 | GOBP\_REGULATION\_OF\_LEUKOCYTE\_MEDIATED\_CYTOTOXICITY |  | 27 | -0.53 | -1.57 | 0.013 | 0.230 | 1.000 | 3285 | tags=56%, list=27%, signal=76% |
| 355 | GOMF\_GAMMA\_TUBULIN\_BINDING |  | 23 | -0.55 | -1.57 | 0.010 | 0.229 | 1.000 | 2731 | tags=52%, list=23%, signal=67% |
| 356 | GOBP\_NEGATIVE\_REGULATION\_OF\_CHROMOSOME\_ORGANIZATION |  | 71 | -0.45 | -1.57 | 0.005 | 0.229 | 1.000 | 3812 | tags=46%, list=32%, signal=68% |
| 357 | GOBP\_POSITIVE\_THYMIC\_T\_CELL\_SELECTION |  | 11 | -0.65 | -1.57 | 0.019 | 0.228 | 1.000 | 3692 | tags=82%, list=31%, signal=118% |
| 358 | GOBP\_INTERKINETIC\_NUCLEAR\_MIGRATION |  | 5 | -0.81 | -1.57 | 0.013 | 0.229 | 1.000 | 2299 | tags=100%, list=19%, signal=124% |
| 359 | GOMF\_DNA\_HELICASE\_ACTIVITY |  | 59 | -0.46 | -1.57 | 0.005 | 0.229 | 1.000 | 5195 | tags=75%, list=43%, signal=131% |
| 360 | GOBP\_MYOTUBE\_CELL\_DEVELOPMENT |  | 18 | -0.58 | -1.57 | 0.016 | 0.230 | 1.000 | 1495 | tags=39%, list=12%, signal=44% |
| 361 | GOBP\_THROMBOPOIETIN\_MEDIATED\_SIGNALING\_PATHWAY |  | 3 | -0.93 | -1.57 | 0.004 | 0.231 | 1.000 | 811 | tags=100%, list=7%, signal=107% |
| 362 | GOBP\_INTERMEDIATE\_FILAMENT\_ORGANIZATION |  | 11 | -0.65 | -1.57 | 0.023 | 0.231 | 1.000 | 3765 | tags=73%, list=31%, signal=106% |
| 363 | GOBP\_PROTEIN\_FOLDING\_IN\_ENDOPLASMIC\_RETICULUM |  | 10 | -0.66 | -1.57 | 0.020 | 0.231 | 1.000 | 2564 | tags=50%, list=21%, signal=64% |
| 364 | GOBP\_REGULATION\_OF\_CELL\_ADHESION\_MEDIATED\_BY\_INTEGRIN |  | 36 | -0.50 | -1.57 | 0.012 | 0.231 | 1.000 | 2955 | tags=53%, list=25%, signal=70% |
| 365 | GOBP\_REGULATION\_OF\_SMOOTH\_MUSCLE\_CONTRACTION |  | 43 | -0.49 | -1.57 | 0.009 | 0.232 | 1.000 | 1261 | tags=28%, list=10%, signal=31% |
| 366 | GOBP\_DOPAMINE\_SECRETION |  | 21 | -0.56 | -1.57 | 0.018 | 0.234 | 1.000 | 954 | tags=24%, list=8%, signal=26% |
| 367 | GOMF\_EXTRACELLULAR\_MATRIX\_STRUCTURAL\_CONSTITUENT\_CONFERRING\_TENSILE\_STRENGTH |  | 27 | -0.53 | -1.56 | 0.015 | 0.236 | 1.000 | 3056 | tags=52%, list=25%, signal=69% |
| 368 | GOBP\_PHOSPHATIDYLINOSITOL\_METABOLIC\_PROCESS |  | 143 | -0.42 | -1.56 | 0.001 | 0.237 | 1.000 | 4057 | tags=50%, list=34%, signal=74% |
| 369 | GOBP\_POSITIVE\_REGULATION\_OF\_NATURAL\_KILLER\_CELL\_MEDIATED\_IMMUNITY |  | 10 | -0.67 | -1.56 | 0.017 | 0.237 | 1.000 | 3285 | tags=70%, list=27%, signal=96% |
| 370 | GOBP\_INTERLEUKIN\_8\_PRODUCTION |  | 45 | -0.48 | -1.56 | 0.011 | 0.237 | 1.000 | 2480 | tags=40%, list=21%, signal=50% |
| 371 | GOBP\_VASCULAR\_ENDOTHELIAL\_GROWTH\_FACTOR\_RECEPTOR\_SIGNALING\_PATHWAY |  | 75 | -0.45 | -1.56 | 0.003 | 0.237 | 1.000 | 3797 | tags=51%, list=32%, signal=74% |
| 372 | GOBP\_POSITIVE\_REGULATION\_OF\_GTPASE\_ACTIVITY |  | 300 | -0.40 | -1.56 | 0.000 | 0.237 | 1.000 | 3274 | tags=41%, list=27%, signal=55% |
| 373 | GOBP\_ATRIAL\_SEPTUM\_DEVELOPMENT |  | 13 | -0.62 | -1.56 | 0.025 | 0.236 | 1.000 | 3314 | tags=54%, list=28%, signal=74% |
| 374 | GOBP\_REGULATION\_OF\_EXTENT\_OF\_CELL\_GROWTH |  | 73 | -0.45 | -1.56 | 0.005 | 0.237 | 1.000 | 3235 | tags=40%, list=27%, signal=54% |
| 375 | GOMF\_METALLOENDOPEPTIDASE\_ACTIVITY |  | 69 | -0.46 | -1.56 | 0.003 | 0.237 | 1.000 | 2979 | tags=33%, list=25%, signal=44% |
| 376 | GOMF\_CYCLIC\_NUCLEOTIDE\_BINDING |  | 22 | -0.55 | -1.56 | 0.020 | 0.236 | 1.000 | 1323 | tags=32%, list=11%, signal=36% |
| 377 | GOBP\_POSITIVE\_T\_CELL\_SELECTION |  | 23 | -0.55 | -1.56 | 0.015 | 0.236 | 1.000 | 2608 | tags=52%, list=22%, signal=67% |
| 378 | GOCC\_CENTRIOLE |  | 98 | -0.43 | -1.56 | 0.002 | 0.236 | 1.000 | 3317 | tags=43%, list=28%, signal=59% |
| 379 | GOBP\_PROGRAMMED\_CELL\_DEATH\_INVOLVED\_IN\_CELL\_DEVELOPMENT |  | 8 | -0.71 | -1.56 | 0.018 | 0.237 | 1.000 | 1013 | tags=50%, list=8%, signal=55% |
| 380 | GOBP\_LYMPH\_VESSEL\_MORPHOGENESIS |  | 16 | -0.59 | -1.56 | 0.020 | 0.239 | 1.000 | 540 | tags=31%, list=4%, signal=33% |
| 381 | GOBP\_HIPPO\_SIGNALING |  | 40 | -0.49 | -1.56 | 0.012 | 0.239 | 1.000 | 3899 | tags=52%, list=32%, signal=77% |
| 382 | GOCC\_PODOSOME |  | 26 | -0.53 | -1.56 | 0.016 | 0.238 | 1.000 | 3052 | tags=50%, list=25%, signal=67% |
| 383 | GOBP\_ASYMMETRIC\_NEUROBLAST\_DIVISION |  | 5 | -0.80 | -1.56 | 0.014 | 0.238 | 1.000 | 1803 | tags=60%, list=15%, signal=71% |
| 384 | GOBP\_OXIDATIVE\_DEMETHYLATION |  | 13 | -0.62 | -1.56 | 0.022 | 0.239 | 1.000 | 2526 | tags=62%, list=21%, signal=78% |
| 385 | GOCC\_FILOPODIUM |  | 76 | -0.45 | -1.56 | 0.004 | 0.240 | 1.000 | 3619 | tags=46%, list=30%, signal=65% |
| 386 | GOBP\_SINGLE\_STRAND\_BREAK\_REPAIR |  | 9 | -0.68 | -1.56 | 0.021 | 0.241 | 1.000 | 3871 | tags=100%, list=32%, signal=147% |
| 387 | GOBP\_REGULATION\_OF\_NUCLEOTIDE\_BINDING\_OLIGOMERIZATION\_DOMAIN\_CONTAINING\_2\_SIGNALING\_PATHWAY |  | 5 | -0.81 | -1.56 | 0.012 | 0.240 | 1.000 | 2256 | tags=80%, list=19%, signal=98% |
| 388 | GOBP\_HISTONE\_H3\_K36\_DEMETHYLATION |  | 6 | -0.76 | -1.56 | 0.015 | 0.240 | 1.000 | 2716 | tags=83%, list=23%, signal=108% |
| 389 | GOBP\_POSITIVE\_REGULATION\_OF\_LAMELLIPODIUM\_ORGANIZATION |  | 28 | -0.52 | -1.56 | 0.016 | 0.240 | 1.000 | 1743 | tags=36%, list=15%, signal=42% |
| 390 | GOBP\_POSITIVE\_REGULATION\_OF\_CELL\_PROJECTION\_ORGANIZATION |  | 249 | -0.40 | -1.56 | 0.000 | 0.240 | 1.000 | 4701 | tags=51%, list=39%, signal=81% |
| 391 | GOBP\_POSITIVE\_REGULATION\_OF\_CYTOSOLIC\_CALCIUM\_ION\_CONCENTRATION\_INVOLVED\_IN\_PHOSPHOLIPASE\_C\_ACTIVATING\_G\_PROTEIN\_COUPLED\_SIGNALING\_PATHWAY |  | 25 | -0.53 | -1.56 | 0.019 | 0.241 | 1.000 | 2652 | tags=40%, list=22%, signal=51% |
| 392 | GOBP\_NEGATIVE\_REGULATION\_OF\_NEURON\_PROJECTION\_DEVELOPMENT |  | 93 | -0.44 | -1.56 | 0.002 | 0.240 | 1.000 | 1464 | tags=25%, list=12%, signal=28% |
| 393 | GOBP\_REGULATION\_OF\_NATURAL\_KILLER\_CELL\_ACTIVATION |  | 16 | -0.59 | -1.56 | 0.022 | 0.240 | 1.000 | 3009 | tags=50%, list=25%, signal=67% |
| 394 | GOBP\_CENTRAL\_NERVOUS\_SYSTEM\_NEURON\_AXONOGENESIS |  | 28 | -0.52 | -1.56 | 0.017 | 0.241 | 1.000 | 3425 | tags=43%, list=29%, signal=60% |
| 395 | GOCC\_POSTSYNAPTIC\_ACTIN\_CYTOSKELETON |  | 5 | -0.81 | -1.56 | 0.010 | 0.240 | 1.000 | 2087 | tags=60%, list=17%, signal=73% |
| 396 | GOBP\_NEGATIVE\_REGULATION\_OF\_FEEDING\_BEHAVIOR |  | 5 | -0.81 | -1.56 | 0.015 | 0.241 | 1.000 | 801 | tags=60%, list=7%, signal=64% |
| 397 | GOBP\_CELL\_MORPHOGENESIS |  | 712 | -0.39 | -1.56 | 0.000 | 0.241 | 1.000 | 4152 | tags=46%, list=35%, signal=66% |
| 398 | GOBP\_COLLAGEN\_ACTIVATED\_SIGNALING\_PATHWAY |  | 10 | -0.67 | -1.56 | 0.017 | 0.241 | 1.000 | 2357 | tags=50%, list=20%, signal=62% |
| 399 | GOBP\_NUCLEOTIDE\_BINDING\_OLIGOMERIZATION\_DOMAIN\_CONTAINING\_2\_SIGNALING\_PATHWAY |  | 9 | -0.68 | -1.56 | 0.025 | 0.242 | 1.000 | 2660 | tags=56%, list=22%, signal=71% |
| 400 | GOCC\_AXONEMAL\_MICROTUBULE |  | 6 | -0.77 | -1.55 | 0.018 | 0.242 | 1.000 | 1160 | tags=33%, list=10%, signal=37% |
| 401 | GOMF\_METALLOPEPTIDASE\_ACTIVITY |  | 119 | -0.42 | -1.55 | 0.002 | 0.242 | 1.000 | 2979 | tags=33%, list=25%, signal=43% |
| 402 | GOBP\_DENDRITIC\_SPINE\_MORPHOGENESIS |  | 45 | -0.48 | -1.55 | 0.010 | 0.244 | 1.000 | 3760 | tags=51%, list=31%, signal=74% |
| 403 | GOMF\_DNA\_DIRECTED\_DNA\_POLYMERASE\_ACTIVITY |  | 17 | -0.58 | -1.55 | 0.019 | 0.244 | 1.000 | 2359 | tags=41%, list=20%, signal=51% |
| 404 | GOBP\_DITERPENOID\_BIOSYNTHETIC\_PROCESS |  | 6 | -0.76 | -1.55 | 0.019 | 0.243 | 1.000 | 51 | tags=33%, list=0%, signal=33% |
| 405 | GOMF\_PHOSPHATIDYLINOSITOL\_3\_KINASE\_REGULATOR\_ACTIVITY |  | 16 | -0.59 | -1.55 | 0.022 | 0.244 | 1.000 | 2670 | tags=63%, list=22%, signal=80% |
| 406 | GOMF\_NEUROTROPHIN\_RECEPTOR\_BINDING |  | 7 | -0.73 | -1.55 | 0.020 | 0.244 | 1.000 | 747 | tags=43%, list=6%, signal=46% |
| 407 | GOBP\_NEGATIVE\_REGULATION\_OF\_HIPPO\_SIGNALING |  | 12 | -0.64 | -1.55 | 0.021 | 0.244 | 1.000 | 3699 | tags=67%, list=31%, signal=96% |
| 408 | GOBP\_HETEROTYPIC\_CELL\_CELL\_ADHESION |  | 37 | -0.49 | -1.55 | 0.011 | 0.244 | 1.000 | 3158 | tags=43%, list=26%, signal=58% |
| 409 | GOCC\_BRUSH\_BORDER\_MEMBRANE |  | 41 | -0.49 | -1.55 | 0.009 | 0.244 | 1.000 | 2897 | tags=56%, list=24%, signal=74% |
| 410 | GOBP\_SPERM\_CAPACITATION |  | 8 | -0.70 | -1.55 | 0.019 | 0.244 | 1.000 | 2067 | tags=63%, list=17%, signal=75% |
| 411 | GOMF\_REGULATORY\_RNA\_BINDING |  | 25 | -0.53 | -1.55 | 0.018 | 0.246 | 1.000 | 2088 | tags=44%, list=17%, signal=53% |
| 412 | GOMF\_3\_5\_CYCLIC\_AMP\_PHOSPHODIESTERASE\_ACTIVITY |  | 8 | -0.70 | -1.55 | 0.020 | 0.245 | 1.000 | 2481 | tags=75%, list=21%, signal=94% |
| 413 | GOMF\_CYTOKINE\_BINDING |  | 91 | -0.43 | -1.55 | 0.004 | 0.246 | 1.000 | 3130 | tags=41%, list=26%, signal=55% |
| 414 | GOBP\_REGULATION\_OF\_EATING\_BEHAVIOR |  | 4 | -0.86 | -1.55 | 0.015 | 0.246 | 1.000 | 1743 | tags=100%, list=15%, signal=117% |
| 415 | GOMF\_HISTONE\_DEMETHYLASE\_ACTIVITY\_H3\_K36\_SPECIFIC |  | 6 | -0.76 | -1.55 | 0.020 | 0.246 | 1.000 | 2716 | tags=83%, list=23%, signal=108% |
| 416 | GOMF\_PHOSPHOFRUCTOKINASE\_ACTIVITY |  | 5 | -0.81 | -1.55 | 0.014 | 0.246 | 1.000 | 320 | tags=40%, list=3%, signal=41% |
| 417 | GOBP\_POSITIVE\_REGULATION\_OF\_LEUKOCYTE\_PROLIFERATION |  | 91 | -0.43 | -1.55 | 0.004 | 0.247 | 1.000 | 2474 | tags=40%, list=21%, signal=49% |
| 418 | GOBP\_NEGATIVE\_REGULATION\_OF\_AUTOPHAGY |  | 66 | -0.45 | -1.55 | 0.005 | 0.246 | 1.000 | 2503 | tags=32%, list=21%, signal=40% |
| 419 | GOMF\_MICROFILAMENT\_MOTOR\_ACTIVITY |  | 17 | -0.58 | -1.55 | 0.023 | 0.246 | 1.000 | 4122 | tags=71%, list=34%, signal=107% |
| 420 | GOBP\_AMINE\_CATABOLIC\_PROCESS |  | 18 | -0.57 | -1.55 | 0.024 | 0.246 | 1.000 | 2996 | tags=50%, list=25%, signal=67% |
| 421 | GOMF\_CHEMOREPELLENT\_ACTIVITY |  | 22 | -0.54 | -1.55 | 0.021 | 0.245 | 1.000 | 488 | tags=23%, list=4%, signal=24% |
| 422 | GOBP\_CYTOPLASMIC\_MICROTUBULE\_ORGANIZATION |  | 47 | -0.48 | -1.55 | 0.012 | 0.245 | 1.000 | 3451 | tags=49%, list=29%, signal=68% |
| 423 | GOMF\_NEUROTRANSMITTER\_RECEPTOR\_ACTIVITY\_INVOLVED\_IN\_REGULATION\_OF\_POSTSYNAPTIC\_MEMBRANE\_POTENTIAL |  | 11 | -0.64 | -1.55 | 0.023 | 0.245 | 1.000 | 462 | tags=36%, list=4%, signal=38% |
| 424 | GOBP\_PEPTIDYL\_SERINE\_MODIFICATION |  | 231 | -0.40 | -1.55 | 0.000 | 0.244 | 1.000 | 4111 | tags=48%, list=34%, signal=71% |
| 425 | GOMF\_PHOSPHORIC\_DIESTER\_HYDROLASE\_ACTIVITY |  | 66 | -0.45 | -1.55 | 0.007 | 0.249 | 1.000 | 3033 | tags=44%, list=25%, signal=58% |
| 426 | GOBP\_INTERSTRAND\_CROSS\_LINK\_REPAIR |  | 47 | -0.47 | -1.55 | 0.009 | 0.249 | 1.000 | 4752 | tags=68%, list=40%, signal=112% |
| 427 | GOCC\_GROWTH\_CONE\_MEMBRANE |  | 8 | -0.70 | -1.55 | 0.023 | 0.250 | 1.000 | 2399 | tags=50%, list=20%, signal=62% |
| 428 | GOBP\_NEGATIVE\_REGULATION\_OF\_EXTRINSIC\_APOPTOTIC\_SIGNALING\_PATHWAY |  | 74 | -0.44 | -1.55 | 0.004 | 0.250 | 1.000 | 2820 | tags=41%, list=23%, signal=53% |
| 429 | GOMF\_PHOSPHATIDYLINOSITOL\_BISPHOSPHATE\_PHOSPHATASE\_ACTIVITY |  | 21 | -0.56 | -1.55 | 0.019 | 0.250 | 1.000 | 3934 | tags=67%, list=33%, signal=99% |
| 430 | GOMF\_PROTEIN\_SERINE\_THREONINE\_KINASE\_ACTIVITY |  | 315 | -0.40 | -1.55 | 0.000 | 0.250 | 1.000 | 4559 | tags=54%, list=38%, signal=85% |
| 431 | GOBP\_CHONDROCYTE\_DIFFERENTIATION |  | 73 | -0.44 | -1.54 | 0.006 | 0.250 | 1.000 | 2886 | tags=41%, list=24%, signal=54% |
| 432 | GOBP\_POSITIVE\_REGULATION\_OF\_BMP\_SIGNALING\_PATHWAY |  | 22 | -0.55 | -1.54 | 0.020 | 0.251 | 1.000 | 3017 | tags=50%, list=25%, signal=67% |
| 433 | GOBP\_T\_CELL\_TOLERANCE\_INDUCTION |  | 6 | -0.76 | -1.54 | 0.018 | 0.253 | 1.000 | 1238 | tags=50%, list=10%, signal=56% |
| 434 | GOBP\_MAINTENANCE\_OF\_SISTER\_CHROMATID\_COHESION |  | 9 | -0.67 | -1.54 | 0.026 | 0.253 | 1.000 | 3836 | tags=89%, list=32%, signal=131% |
| 435 | GOBP\_LYMPHOCYTE\_ACTIVATION |  | 434 | -0.39 | -1.54 | 0.000 | 0.254 | 1.000 | 3243 | tags=41%, list=27%, signal=54% |
| 436 | GOMF\_EXTRACELLULAR\_LIGAND\_GATED\_ION\_CHANNEL\_ACTIVITY |  | 20 | -0.56 | -1.54 | 0.020 | 0.254 | 1.000 | 1285 | tags=35%, list=11%, signal=39% |
| 437 | GOMF\_DEMETHYLASE\_ACTIVITY |  | 25 | -0.53 | -1.54 | 0.019 | 0.254 | 1.000 | 4508 | tags=72%, list=38%, signal=115% |
| 438 | GOBP\_POSITIVE\_REGULATION\_OF\_TUMOR\_NECROSIS\_FACTOR\_SUPERFAMILY\_CYTOKINE\_PRODUCTION |  | 50 | -0.47 | -1.54 | 0.011 | 0.253 | 1.000 | 2547 | tags=40%, list=21%, signal=51% |
| 439 | GOBP\_MICROTUBULE\_NUCLEATION |  | 22 | -0.54 | -1.54 | 0.022 | 0.254 | 1.000 | 3551 | tags=55%, list=30%, signal=77% |
| 440 | GOMF\_MOTOR\_ACTIVITY |  | 82 | -0.44 | -1.54 | 0.003 | 0.253 | 1.000 | 4165 | tags=54%, list=35%, signal=82% |
| 441 | GOBP\_COVALENT\_CHROMATIN\_MODIFICATION |  | 332 | -0.40 | -1.54 | 0.000 | 0.254 | 1.000 | 4528 | tags=50%, list=38%, signal=78% |
| 442 | GOBP\_NCRNA\_EXPORT\_FROM\_NUCLEUS |  | 33 | -0.50 | -1.54 | 0.017 | 0.254 | 1.000 | 4080 | tags=58%, list=34%, signal=87% |
| 443 | GOMF\_SMALL\_MOLECULE\_SENSOR\_ACTIVITY |  | 7 | -0.73 | -1.54 | 0.022 | 0.253 | 1.000 | 207 | tags=29%, list=2%, signal=29% |
| 444 | GOBP\_NEGATIVE\_REGULATION\_OF\_CELL\_ADHESION\_MEDIATED\_BY\_INTEGRIN |  | 8 | -0.70 | -1.54 | 0.026 | 0.253 | 1.000 | 1914 | tags=63%, list=16%, signal=74% |
| 445 | GOMF\_ACETYLCHOLINE\_RECEPTOR\_ACTIVITY |  | 8 | -0.70 | -1.54 | 0.023 | 0.254 | 1.000 | 929 | tags=50%, list=8%, signal=54% |
| 446 | GOBP\_DEVELOPMENTAL\_GROWTH |  | 415 | -0.39 | -1.54 | 0.000 | 0.253 | 1.000 | 3408 | tags=38%, list=28%, signal=51% |
| 447 | GOBP\_POSTSYNAPSE\_ORGANIZATION |  | 111 | -0.42 | -1.54 | 0.001 | 0.253 | 1.000 | 3488 | tags=41%, list=29%, signal=58% |
| 448 | GOBP\_CARDIAC\_PACEMAKER\_CELL\_DIFFERENTIATION |  | 4 | -0.85 | -1.54 | 0.013 | 0.253 | 1.000 | 848 | tags=50%, list=7%, signal=54% |
| 449 | GOBP\_NEGATIVE\_REGULATION\_OF\_DNA\_METABOLIC\_PROCESS |  | 76 | -0.44 | -1.54 | 0.005 | 0.252 | 1.000 | 2518 | tags=42%, list=21%, signal=53% |
| 450 | GOBP\_NEURON\_DEVELOPMENT |  | 766 | -0.39 | -1.54 | 0.000 | 0.252 | 1.000 | 3836 | tags=42%, list=32%, signal=58% |
| 451 | GOBP\_MEGAKARYOCYTE\_DIFFERENTIATION |  | 56 | -0.46 | -1.54 | 0.008 | 0.252 | 1.000 | 2820 | tags=45%, list=23%, signal=58% |
| 452 | GOCC\_PINOSOME |  | 5 | -0.80 | -1.54 | 0.012 | 0.252 | 1.000 | 1061 | tags=60%, list=9%, signal=66% |
| 453 | GOBP\_FOLIC\_ACID\_CONTAINING\_COMPOUND\_METABOLIC\_PROCESS |  | 21 | -0.54 | -1.54 | 0.023 | 0.251 | 1.000 | 4242 | tags=62%, list=35%, signal=96% |
| 454 | GOCC\_CONDENSED\_NUCLEAR\_CHROMOSOME\_KINETOCHORE |  | 13 | -0.61 | -1.54 | 0.027 | 0.252 | 1.000 | 3759 | tags=77%, list=31%, signal=112% |
| 455 | GOBP\_VESICLE\_UNCOATING |  | 5 | -0.79 | -1.54 | 0.022 | 0.253 | 1.000 | 1793 | tags=60%, list=15%, signal=70% |
| 456 | GOCC\_PHOSPHATIDYLINOSITOL\_3\_KINASE\_COMPLEX\_CLASS\_I |  | 5 | -0.80 | -1.54 | 0.015 | 0.253 | 1.000 | 1092 | tags=80%, list=9%, signal=88% |
| 457 | GOBP\_NEUROTRANSMITTER\_SECRETION |  | 104 | -0.43 | -1.54 | 0.003 | 0.253 | 1.000 | 3946 | tags=54%, list=33%, signal=79% |
| 458 | GOMF\_ORGANIC\_CATION\_TRANSMEMBRANE\_TRANSPORTER\_ACTIVITY |  | 12 | -0.63 | -1.54 | 0.023 | 0.254 | 1.000 | 3277 | tags=67%, list=27%, signal=92% |
| 459 | GOBP\_CAMP\_METABOLIC\_PROCESS |  | 15 | -0.60 | -1.54 | 0.021 | 0.254 | 1.000 | 2481 | tags=53%, list=21%, signal=67% |
| 460 | GOBP\_MOTOR\_NEURON\_APOPTOTIC\_PROCESS |  | 8 | -0.69 | -1.54 | 0.027 | 0.254 | 1.000 | 2903 | tags=50%, list=24%, signal=66% |
| 461 | GOBP\_MACROPHAGE\_CYTOKINE\_PRODUCTION |  | 12 | -0.63 | -1.54 | 0.025 | 0.254 | 1.000 | 2889 | tags=67%, list=24%, signal=88% |
| 462 | GOBP\_NEGATIVE\_REGULATION\_OF\_TELOMERE\_MAINTENANCE |  | 26 | -0.52 | -1.54 | 0.020 | 0.255 | 1.000 | 3722 | tags=58%, list=31%, signal=83% |
| 463 | GOCC\_DNA\_REPAIR\_COMPLEX |  | 30 | -0.51 | -1.54 | 0.015 | 0.254 | 1.000 | 4029 | tags=67%, list=34%, signal=100% |
| 464 | GOBP\_NEGATIVE\_REGULATION\_OF\_INTERLEUKIN\_6\_PRODUCTION |  | 22 | -0.54 | -1.54 | 0.022 | 0.256 | 1.000 | 1601 | tags=36%, list=13%, signal=42% |
| 465 | GOBP\_RAS\_PROTEIN\_SIGNAL\_TRANSDUCTION |  | 256 | -0.40 | -1.54 | 0.000 | 0.255 | 1.000 | 3534 | tags=42%, list=29%, signal=58% |
| 466 | GOBP\_REGULATION\_OF\_CELL\_ADHESION |  | 486 | -0.39 | -1.54 | 0.000 | 0.256 | 1.000 | 3205 | tags=37%, list=27%, signal=49% |
| 467 | GOCC\_BRUSH\_BORDER |  | 72 | -0.44 | -1.54 | 0.005 | 0.257 | 1.000 | 2897 | tags=43%, list=24%, signal=56% |
| 468 | GOCC\_CELL\_BODY\_MEMBRANE |  | 16 | -0.58 | -1.53 | 0.032 | 0.256 | 1.000 | 1668 | tags=38%, list=14%, signal=43% |
| 469 | GOBP\_POSITIVE\_REGULATION\_OF\_CILIUM\_ASSEMBLY |  | 16 | -0.58 | -1.53 | 0.025 | 0.256 | 1.000 | 4187 | tags=63%, list=35%, signal=96% |
| 470 | GOMF\_ENDODEOXYRIBONUCLEASE\_ACTIVITY\_PRODUCING\_5\_PHOSPHOMONOESTERS |  | 10 | -0.66 | -1.53 | 0.027 | 0.255 | 1.000 | 2733 | tags=60%, list=23%, signal=78% |
| 471 | GOBP\_INFLAMMATORY\_RESPONSE\_TO\_WOUNDING |  | 9 | -0.67 | -1.53 | 0.022 | 0.256 | 1.000 | 1734 | tags=56%, list=14%, signal=65% |
| 472 | GOBP\_CLATHRIN\_COAT\_DISASSEMBLY |  | 4 | -0.85 | -1.53 | 0.011 | 0.256 | 1.000 | 1793 | tags=75%, list=15%, signal=88% |
| 473 | GOBP\_PEPTIDYL\_TYROSINE\_AUTOPHOSPHORYLATION |  | 16 | -0.58 | -1.53 | 0.026 | 0.256 | 1.000 | 3400 | tags=63%, list=28%, signal=87% |
| 474 | GOBP\_MITOTIC\_SISTER\_CHROMATID\_COHESION |  | 19 | -0.56 | -1.53 | 0.024 | 0.257 | 1.000 | 4279 | tags=68%, list=36%, signal=106% |
| 475 | GOMF\_ALKALI\_METAL\_ION\_BINDING |  | 12 | -0.62 | -1.53 | 0.029 | 0.257 | 1.000 | 2070 | tags=42%, list=17%, signal=50% |
| 476 | GOBP\_POSITIVE\_REGULATION\_OF\_NEUTROPHIL\_MIGRATION |  | 23 | -0.54 | -1.53 | 0.021 | 0.257 | 1.000 | 2011 | tags=39%, list=17%, signal=47% |
| 477 | GOMF\_NUCLEOSIDE\_TRIPHOSPHATASE\_REGULATOR\_ACTIVITY |  | 382 | -0.39 | -1.53 | 0.000 | 0.257 | 1.000 | 3723 | tags=45%, list=31%, signal=62% |
| 478 | GOMF\_LYSINE\_ACETYLATED\_HISTONE\_BINDING |  | 14 | -0.60 | -1.53 | 0.030 | 0.257 | 1.000 | 2500 | tags=50%, list=21%, signal=63% |
| 479 | GOBP\_REGULATION\_OF\_MYELOID\_LEUKOCYTE\_MEDIATED\_IMMUNITY |  | 31 | -0.50 | -1.53 | 0.017 | 0.258 | 1.000 | 4108 | tags=52%, list=34%, signal=78% |
| 480 | GOMF\_COMPLEMENT\_BINDING |  | 12 | -0.63 | -1.53 | 0.024 | 0.258 | 1.000 | 1808 | tags=33%, list=15%, signal=39% |
| 481 | GOBP\_REGULATION\_OF\_NEURON\_PROJECTION\_DEVELOPMENT |  | 311 | -0.39 | -1.53 | 0.000 | 0.257 | 1.000 | 2631 | tags=32%, list=22%, signal=39% |
| 482 | GOBP\_EMBRYONIC\_EYE\_MORPHOGENESIS |  | 19 | -0.55 | -1.53 | 0.023 | 0.260 | 1.000 | 1499 | tags=32%, list=12%, signal=36% |
| 483 | GOBP\_NUCLEOTIDE\_BINDING\_OLIGOMERIZATION\_DOMAIN\_CONTAINING\_1\_SIGNALING\_PATHWAY |  | 6 | -0.76 | -1.53 | 0.018 | 0.260 | 1.000 | 1852 | tags=50%, list=15%, signal=59% |
| 484 | GOBP\_REGULATION\_OF\_LEUKOCYTE\_ADHESION\_TO\_ARTERIAL\_ENDOTHELIAL\_CELL |  | 3 | -0.92 | -1.53 | 0.006 | 0.261 | 1.000 | 231 | tags=67%, list=2%, signal=68% |
| 485 | GOBP\_POSITIVE\_REGULATION\_OF\_HEMOPOIESIS |  | 101 | -0.42 | -1.53 | 0.004 | 0.261 | 1.000 | 2408 | tags=40%, list=20%, signal=49% |
| 486 | GOBP\_THYROID\_GLAND\_DEVELOPMENT |  | 12 | -0.62 | -1.53 | 0.027 | 0.260 | 1.000 | 3565 | tags=67%, list=30%, signal=95% |
| 487 | GOBP\_POSITIVE\_REGULATION\_OF\_PROTEIN\_SERINE\_THREONINE\_KINASE\_ACTIVITY |  | 228 | -0.40 | -1.53 | 0.000 | 0.260 | 1.000 | 3655 | tags=38%, list=30%, signal=54% |
| 488 | GOMF\_SODIUM\_ION\_BINDING |  | 6 | -0.76 | -1.53 | 0.019 | 0.260 | 1.000 | 2070 | tags=67%, list=17%, signal=81% |
| 489 | GOBP\_ARF\_PROTEIN\_SIGNAL\_TRANSDUCTION |  | 14 | -0.60 | -1.53 | 0.031 | 0.260 | 1.000 | 3212 | tags=64%, list=27%, signal=88% |
| 490 | GOBP\_NEUROBLAST\_DIVISION |  | 10 | -0.66 | -1.53 | 0.027 | 0.260 | 1.000 | 1803 | tags=40%, list=15%, signal=47% |
| 491 | GOBP\_WOUND\_HEALING\_INVOLVED\_IN\_INFLAMMATORY\_RESPONSE |  | 4 | -0.84 | -1.53 | 0.020 | 0.261 | 1.000 | 726 | tags=50%, list=6%, signal=53% |
| 492 | GOBP\_POSITIVE\_REGULATION\_OF\_PROTEIN\_MATURATION |  | 14 | -0.60 | -1.53 | 0.029 | 0.262 | 1.000 | 1843 | tags=50%, list=15%, signal=59% |
| 493 | GOBP\_PLATELET\_AGGREGATION |  | 42 | -0.48 | -1.53 | 0.013 | 0.262 | 1.000 | 3588 | tags=43%, list=30%, signal=61% |
| 494 | GOMF\_IRON\_ION\_BINDING |  | 87 | -0.43 | -1.53 | 0.003 | 0.262 | 1.000 | 2803 | tags=37%, list=23%, signal=48% |
| 495 | GOBP\_MESODERM\_MORPHOGENESIS |  | 43 | -0.48 | -1.53 | 0.013 | 0.263 | 1.000 | 3005 | tags=40%, list=25%, signal=53% |
| 496 | GOBP\_MONONUCLEAR\_CELL\_DIFFERENTIATION |  | 270 | -0.40 | -1.53 | 0.000 | 0.262 | 1.000 | 3326 | tags=43%, list=28%, signal=58% |
| 497 | GOBP\_ACTIVATION\_OF\_MAPKK\_ACTIVITY |  | 35 | -0.49 | -1.53 | 0.018 | 0.262 | 1.000 | 2168 | tags=43%, list=18%, signal=52% |
| 498 | GOBP\_DEMETHYLATION |  | 39 | -0.48 | -1.53 | 0.019 | 0.266 | 1.000 | 2716 | tags=46%, list=23%, signal=59% |
| 499 | GOBP\_MATURE\_B\_CELL\_DIFFERENTIATION\_INVOLVED\_IN\_IMMUNE\_RESPONSE |  | 18 | -0.56 | -1.53 | 0.028 | 0.266 | 1.000 | 1432 | tags=39%, list=12%, signal=44% |
| 500 | GOBP\_REGULATION\_OF\_RECEPTOR\_SIGNALING\_PATHWAY\_VIA\_STAT |  | 53 | -0.46 | -1.52 | 0.013 | 0.266 | 1.000 | 1541 | tags=30%, list=13%, signal=34% |
| 501 | GOBP\_POSITIVE\_REGULATION\_OF\_PROTEIN\_KINASE\_ACTIVITY |  | 383 | -0.39 | -1.52 | 0.000 | 0.266 | 1.000 | 3613 | tags=39%, list=30%, signal=54% |
| 502 | GOBP\_NEGATIVE\_REGULATION\_OF\_HOMOTYPIC\_CELL\_CELL\_ADHESION |  | 10 | -0.64 | -1.52 | 0.029 | 0.266 | 1.000 | 2303 | tags=40%, list=19%, signal=49% |
| 503 | GOBP\_POSITIVE\_REGULATION\_OF\_CALCIUM\_ION\_DEPENDENT\_EXOCYTOSIS |  | 12 | -0.62 | -1.52 | 0.031 | 0.266 | 1.000 | 278 | tags=25%, list=2%, signal=26% |
| 504 | GOMF\_VOLTAGE\_GATED\_ANION\_CHANNEL\_ACTIVITY |  | 12 | -0.62 | -1.52 | 0.028 | 0.266 | 1.000 | 2059 | tags=50%, list=17%, signal=60% |
| 505 | GOBP\_POSITIVE\_REGULATION\_OF\_REGULATED\_SECRETORY\_PATHWAY |  | 32 | -0.50 | -1.52 | 0.013 | 0.266 | 1.000 | 2599 | tags=34%, list=22%, signal=44% |
| 506 | GOBP\_POSITIVE\_REGULATION\_OF\_MAPK\_CASCADE |  | 332 | -0.39 | -1.52 | 0.000 | 0.266 | 1.000 | 3408 | tags=39%, list=28%, signal=53% |
| 507 | GOBP\_PARAXIAL\_MESODERM\_DEVELOPMENT |  | 10 | -0.65 | -1.52 | 0.029 | 0.266 | 1.000 | 3822 | tags=80%, list=32%, signal=117% |
| 508 | GOBP\_REGULATION\_OF\_MICROTUBULE\_POLYMERIZATION\_OR\_DEPOLYMERIZATION |  | 52 | -0.46 | -1.52 | 0.012 | 0.267 | 1.000 | 3148 | tags=42%, list=26%, signal=57% |
| 509 | GOBP\_REGULATION\_OF\_PROTEIN\_KINASE\_ACTIVITY |  | 563 | -0.39 | -1.52 | 0.000 | 0.267 | 1.000 | 3934 | tags=42%, list=33%, signal=60% |
| 510 | GOMF\_GLUCOCORTICOID\_RECEPTOR\_BINDING |  | 11 | -0.63 | -1.52 | 0.029 | 0.268 | 1.000 | 2405 | tags=64%, list=20%, signal=79% |
| 511 | GOBP\_JNK\_CASCADE |  | 130 | -0.41 | -1.52 | 0.002 | 0.268 | 1.000 | 3377 | tags=43%, list=28%, signal=59% |
| 512 | GOBP\_T\_CELL\_SELECTION |  | 33 | -0.50 | -1.52 | 0.017 | 0.268 | 1.000 | 2608 | tags=45%, list=22%, signal=58% |
| 513 | GOBP\_ADENYLATE\_CYCLASE\_ACTIVATING\_G\_PROTEIN\_COUPLED\_RECEPTOR\_SIGNALING\_PATHWAY |  | 80 | -0.43 | -1.52 | 0.007 | 0.267 | 1.000 | 3308 | tags=40%, list=28%, signal=55% |
| 514 | GOBP\_REGULATION\_OF\_GAMMA\_DELTA\_T\_CELL\_ACTIVATION |  | 8 | -0.69 | -1.52 | 0.028 | 0.267 | 1.000 | 2357 | tags=63%, list=20%, signal=78% |
| 515 | GOBP\_NEGATIVE\_T\_CELL\_SELECTION |  | 11 | -0.64 | -1.52 | 0.026 | 0.267 | 1.000 | 2608 | tags=64%, list=22%, signal=81% |
| 516 | GOBP\_POSITIVE\_REGULATION\_OF\_RESPIRATORY\_BURST |  | 5 | -0.79 | -1.52 | 0.018 | 0.267 | 1.000 | 675 | tags=60%, list=6%, signal=64% |
| 517 | GOBP\_RESPONSE\_TO\_NICOTINE |  | 19 | -0.55 | -1.52 | 0.027 | 0.267 | 1.000 | 1748 | tags=37%, list=15%, signal=43% |
| 518 | GOMF\_ATP\_DEPENDENT\_MICROTUBULE\_MOTOR\_ACTIVITY |  | 19 | -0.56 | -1.52 | 0.031 | 0.267 | 1.000 | 3769 | tags=68%, list=31%, signal=100% |
| 519 | GOBP\_MUSCLE\_CELL\_MIGRATION |  | 67 | -0.44 | -1.52 | 0.007 | 0.267 | 1.000 | 3400 | tags=39%, list=28%, signal=54% |
| 520 | GOBP\_POSITIVE\_REGULATION\_OF\_MACROPHAGE\_CHEMOTAXIS |  | 12 | -0.62 | -1.52 | 0.029 | 0.267 | 1.000 | 3093 | tags=67%, list=26%, signal=90% |
| 521 | GOBP\_CARDIAC\_SEPTUM\_MORPHOGENESIS |  | 49 | -0.46 | -1.52 | 0.014 | 0.268 | 1.000 | 2560 | tags=31%, list=21%, signal=39% |
| 522 | GOBP\_ACTIVATION\_OF\_JUN\_KINASE\_ACTIVITY |  | 27 | -0.51 | -1.52 | 0.021 | 0.268 | 1.000 | 3142 | tags=52%, list=26%, signal=70% |
| 523 | GOBP\_RECOMBINATIONAL\_REPAIR |  | 101 | -0.42 | -1.52 | 0.004 | 0.268 | 1.000 | 3434 | tags=47%, list=29%, signal=65% |
| 524 | GOBP\_POSITIVE\_REGULATION\_OF\_SYNAPSE\_ASSEMBLY |  | 31 | -0.50 | -1.52 | 0.022 | 0.268 | 1.000 | 3629 | tags=52%, list=30%, signal=74% |
| 525 | GOCC\_DENDRITIC\_SHAFT |  | 24 | -0.53 | -1.52 | 0.025 | 0.268 | 1.000 | 1498 | tags=29%, list=12%, signal=33% |
| 526 | GOBP\_ORGAN\_GROWTH |  | 105 | -0.42 | -1.52 | 0.004 | 0.269 | 1.000 | 3613 | tags=42%, list=30%, signal=59% |
| 527 | GOBP\_SKELETAL\_SYSTEM\_DEVELOPMENT |  | 315 | -0.39 | -1.52 | 0.000 | 0.270 | 1.000 | 3100 | tags=38%, list=26%, signal=50% |
| 528 | GOBP\_CELL\_CELL\_ADHESION |  | 527 | -0.39 | -1.52 | 0.000 | 0.271 | 1.000 | 3222 | tags=36%, list=27%, signal=47% |
| 529 | GOBP\_PINOCYTOSIS |  | 15 | -0.59 | -1.52 | 0.027 | 0.271 | 1.000 | 1903 | tags=40%, list=16%, signal=47% |
| 530 | GOMF\_RNA\_POLYMERASE\_II\_ACTIVATING\_TRANSCRIPTION\_FACTOR\_BINDING |  | 35 | -0.49 | -1.52 | 0.019 | 0.271 | 1.000 | 3314 | tags=46%, list=28%, signal=63% |
| 531 | GOCC\_PLASMA\_MEMBRANE\_RAFT |  | 79 | -0.43 | -1.52 | 0.006 | 0.271 | 1.000 | 1777 | tags=27%, list=15%, signal=31% |
| 532 | GOBP\_TRACHEA\_CARTILAGE\_DEVELOPMENT |  | 4 | -0.83 | -1.52 | 0.019 | 0.271 | 1.000 | 405 | tags=50%, list=3%, signal=52% |
| 533 | GOMF\_PHOSPHATIDYLINOSITOL\_PHOSPHATE\_KINASE\_ACTIVITY |  | 13 | -0.61 | -1.52 | 0.028 | 0.271 | 1.000 | 2289 | tags=54%, list=19%, signal=66% |
| 534 | GOBP\_NEUROMUSCULAR\_PROCESS |  | 48 | -0.46 | -1.52 | 0.013 | 0.270 | 1.000 | 3431 | tags=46%, list=29%, signal=64% |
| 535 | GOBP\_QUATERNARY\_AMMONIUM\_GROUP\_TRANSPORT |  | 8 | -0.69 | -1.52 | 0.029 | 0.271 | 1.000 | 2557 | tags=75%, list=21%, signal=95% |
| 536 | GOBP\_RESPONSE\_TO\_AMYLOID\_BETA |  | 30 | -0.50 | -1.52 | 0.019 | 0.271 | 1.000 | 2313 | tags=40%, list=19%, signal=49% |
| 537 | GOBP\_REGULATION\_OF\_VASOCONSTRICTION |  | 45 | -0.47 | -1.52 | 0.014 | 0.271 | 1.000 | 1269 | tags=29%, list=11%, signal=32% |
| 538 | GOBP\_REGULATION\_OF\_CLATHRIN\_DEPENDENT\_ENDOCYTOSIS |  | 16 | -0.57 | -1.52 | 0.027 | 0.271 | 1.000 | 1677 | tags=38%, list=14%, signal=44% |
| 539 | GOBP\_RESPIRATORY\_SYSTEM\_DEVELOPMENT |  | 131 | -0.41 | -1.52 | 0.003 | 0.271 | 1.000 | 2515 | tags=33%, list=21%, signal=41% |
| 540 | GOBP\_REGULATION\_OF\_BILE\_ACID\_METABOLIC\_PROCESS |  | 11 | -0.62 | -1.52 | 0.030 | 0.272 | 1.000 | 643 | tags=36%, list=5%, signal=38% |
| 541 | GOBP\_T\_CELL\_HOMEOSTASIS |  | 23 | -0.53 | -1.52 | 0.026 | 0.272 | 1.000 | 2362 | tags=43%, list=20%, signal=54% |
| 542 | GOMF\_SOLUTE\_CATION\_ANTIPORTER\_ACTIVITY |  | 20 | -0.55 | -1.51 | 0.029 | 0.274 | 1.000 | 1128 | tags=40%, list=9%, signal=44% |
| 543 | GOBP\_COAGULATION |  | 224 | -0.40 | -1.51 | 0.000 | 0.274 | 1.000 | 3394 | tags=39%, list=28%, signal=53% |
| 544 | GOBP\_MESODERM\_DEVELOPMENT |  | 71 | -0.44 | -1.51 | 0.008 | 0.273 | 1.000 | 3306 | tags=38%, list=28%, signal=52% |
| 545 | GOBP\_NEGATIVE\_REGULATION\_OF\_T\_CELL\_PROLIFERATION |  | 34 | -0.49 | -1.51 | 0.022 | 0.274 | 1.000 | 3565 | tags=53%, list=30%, signal=75% |
| 546 | GOBP\_REGULATION\_OF\_GAMMA\_DELTA\_T\_CELL\_DIFFERENTIATION |  | 8 | -0.69 | -1.51 | 0.036 | 0.275 | 1.000 | 2357 | tags=63%, list=20%, signal=78% |
| 547 | GOBP\_REGULATION\_OF\_T\_CELL\_EXTRAVASATION |  | 4 | -0.83 | -1.51 | 0.016 | 0.275 | 1.000 | 2011 | tags=100%, list=17%, signal=120% |
| 548 | GOBP\_CARDIAC\_ATRIUM\_DEVELOPMENT |  | 20 | -0.54 | -1.51 | 0.029 | 0.275 | 1.000 | 3314 | tags=55%, list=28%, signal=76% |
| 549 | GOBP\_VESICLE\_CARGO\_LOADING |  | 19 | -0.55 | -1.51 | 0.027 | 0.275 | 1.000 | 4369 | tags=68%, list=36%, signal=107% |
| 550 | GOBP\_EYE\_MORPHOGENESIS |  | 90 | -0.42 | -1.51 | 0.006 | 0.275 | 1.000 | 1520 | tags=26%, list=13%, signal=29% |
| 551 | GOCC\_NEURON\_PROJECTION\_TERMINUS |  | 81 | -0.43 | -1.51 | 0.007 | 0.275 | 1.000 | 2150 | tags=33%, list=18%, signal=40% |
| 552 | GOBP\_POSITIVE\_REGULATION\_OF\_GAMMA\_DELTA\_T\_CELL\_DIFFERENTIATION |  | 6 | -0.75 | -1.51 | 0.022 | 0.275 | 1.000 | 2357 | tags=67%, list=20%, signal=83% |
| 553 | GOBP\_POSITIVE\_REGULATION\_OF\_CELL\_MORPHOGENESIS\_INVOLVED\_IN\_DIFFERENTIATION |  | 62 | -0.44 | -1.51 | 0.009 | 0.275 | 1.000 | 1786 | tags=26%, list=15%, signal=30% |
| 554 | GOBP\_EXPLORATION\_BEHAVIOR |  | 11 | -0.63 | -1.51 | 0.031 | 0.275 | 1.000 | 3483 | tags=73%, list=29%, signal=102% |
| 555 | GOBP\_PROTEIN\_LOCALIZATION\_TO\_CELL\_SURFACE |  | 46 | -0.46 | -1.51 | 0.015 | 0.275 | 1.000 | 4057 | tags=50%, list=34%, signal=75% |
| 556 | GOBP\_SYNAPSE\_ASSEMBLY |  | 94 | -0.42 | -1.51 | 0.005 | 0.275 | 1.000 | 3699 | tags=41%, list=31%, signal=59% |
| 557 | GOBP\_NEURAL\_PLATE\_DEVELOPMENT |  | 4 | -0.83 | -1.51 | 0.021 | 0.277 | 1.000 | 1764 | tags=75%, list=15%, signal=88% |
| 558 | GOBP\_CYCLIC\_NUCLEOTIDE\_MEDIATED\_SIGNALING |  | 56 | -0.45 | -1.51 | 0.010 | 0.277 | 1.000 | 2941 | tags=41%, list=24%, signal=54% |
| 559 | GOBP\_POSITIVE\_REGULATION\_OF\_SENSORY\_PERCEPTION\_OF\_PAIN |  | 4 | -0.83 | -1.51 | 0.016 | 0.276 | 1.000 | 1743 | tags=75%, list=15%, signal=88% |
| 560 | GOBP\_POSITIVE\_REGULATION\_OF\_NATURAL\_KILLER\_CELL\_MEDIATED\_CYTOTOXICITY |  | 9 | -0.66 | -1.51 | 0.031 | 0.277 | 1.000 | 3285 | tags=67%, list=27%, signal=92% |
| 561 | GOBP\_NEGATIVE\_REGULATION\_OF\_B\_CELL\_ACTIVATION |  | 23 | -0.53 | -1.51 | 0.028 | 0.276 | 1.000 | 2771 | tags=48%, list=23%, signal=62% |
| 562 | GOMF\_HISTONE\_DEMETHYLASE\_ACTIVITY\_H3\_K9\_SPECIFIC |  | 9 | -0.66 | -1.51 | 0.032 | 0.276 | 1.000 | 4114 | tags=100%, list=34%, signal=152% |
| 563 | GOBP\_DENDRITE\_DEVELOPMENT |  | 174 | -0.40 | -1.51 | 0.001 | 0.276 | 1.000 | 3833 | tags=44%, list=32%, signal=63% |
| 564 | GOMF\_KINASE\_BINDING |  | 551 | -0.38 | -1.51 | 0.000 | 0.276 | 1.000 | 3715 | tags=41%, list=31%, signal=56% |
| 565 | GOBP\_ESTABLISHMENT\_OF\_CELL\_POLARITY |  | 114 | -0.41 | -1.51 | 0.003 | 0.276 | 1.000 | 4111 | tags=44%, list=34%, signal=66% |
| 566 | GOBP\_NEGATIVE\_REGULATION\_OF\_LEUKOCYTE\_APOPTOTIC\_PROCESS |  | 32 | -0.50 | -1.51 | 0.021 | 0.276 | 1.000 | 1903 | tags=41%, list=16%, signal=48% |
| 567 | GOBP\_TYPE\_II\_PNEUMOCYTE\_DIFFERENTIATION |  | 4 | -0.83 | -1.51 | 0.018 | 0.276 | 1.000 | 1838 | tags=75%, list=15%, signal=89% |
| 568 | GOBP\_REGULATION\_OF\_LEUKOCYTE\_DIFFERENTIATION |  | 181 | -0.40 | -1.51 | 0.000 | 0.276 | 1.000 | 2408 | tags=35%, list=20%, signal=43% |
| 569 | GOMF\_HORMONE\_ACTIVITY |  | 39 | -0.48 | -1.51 | 0.020 | 0.276 | 1.000 | 1539 | tags=33%, list=13%, signal=38% |
| 570 | GOBP\_POSITIVE\_REGULATION\_OF\_T\_CELL\_PROLIFERATION |  | 57 | -0.45 | -1.51 | 0.009 | 0.276 | 1.000 | 2408 | tags=39%, list=20%, signal=48% |
| 571 | GOBP\_ANTEROGRADE\_AXONAL\_TRANSPORT |  | 37 | -0.48 | -1.51 | 0.020 | 0.276 | 1.000 | 3668 | tags=49%, list=31%, signal=70% |
| 572 | GOMF\_MAGNESIUM\_DEPENDENT\_PROTEIN\_SERINE\_THREONINE\_PHOSPHATASE\_ACTIVITY |  | 10 | -0.64 | -1.51 | 0.030 | 0.277 | 1.000 | 951 | tags=40%, list=8%, signal=43% |
| 573 | GOBP\_SOMITOGENESIS |  | 42 | -0.47 | -1.51 | 0.019 | 0.278 | 1.000 | 3560 | tags=43%, list=30%, signal=61% |
| 574 | GOCC\_EXTERNAL\_SIDE\_OF\_PLASMA\_MEMBRANE |  | 183 | -0.40 | -1.51 | 0.000 | 0.278 | 1.000 | 3163 | tags=42%, list=26%, signal=56% |
| 575 | GOBP\_REGULATION\_OF\_HEMOPOIESIS |  | 265 | -0.39 | -1.51 | 0.000 | 0.278 | 1.000 | 2408 | tags=34%, list=20%, signal=41% |
| 576 | GOBP\_RESPONSE\_TO\_GAMMA\_RADIATION |  | 40 | -0.47 | -1.51 | 0.022 | 0.278 | 1.000 | 3927 | tags=60%, list=33%, signal=89% |
| 577 | GOBP\_ZINC\_ION\_HOMEOSTASIS |  | 17 | -0.56 | -1.51 | 0.032 | 0.278 | 1.000 | 3112 | tags=59%, list=26%, signal=79% |
| 578 | GOBP\_REGULATION\_OF\_LEUKOCYTE\_APOPTOTIC\_PROCESS |  | 54 | -0.45 | -1.51 | 0.015 | 0.278 | 1.000 | 1903 | tags=37%, list=16%, signal=44% |
| 579 | GOBP\_PRODUCTION\_OF\_SMALL\_RNA\_INVOLVED\_IN\_GENE\_SILENCING\_BY\_RNA |  | 38 | -0.48 | -1.51 | 0.018 | 0.278 | 1.000 | 2384 | tags=37%, list=20%, signal=46% |
| 580 | GOBP\_V\_D\_J\_RECOMBINATION |  | 14 | -0.59 | -1.51 | 0.031 | 0.279 | 1.000 | 4652 | tags=79%, list=39%, signal=128% |
| 581 | GOBP\_REGULATION\_OF\_NEUROTRANSMITTER\_TRANSPORT |  | 73 | -0.43 | -1.51 | 0.007 | 0.279 | 1.000 | 2876 | tags=38%, list=24%, signal=50% |
| 582 | GOCC\_DNA\_PACKAGING\_COMPLEX |  | 18 | -0.56 | -1.51 | 0.032 | 0.279 | 1.000 | 4592 | tags=83%, list=38%, signal=135% |
| 583 | GOCC\_CHROMOSOME\_CENTROMERIC\_REGION |  | 163 | -0.40 | -1.51 | 0.000 | 0.279 | 1.000 | 5914 | tags=70%, list=49%, signal=136% |
| 584 | GOBP\_NEUROTRANSMITTER\_METABOLIC\_PROCESS |  | 16 | -0.57 | -1.50 | 0.029 | 0.281 | 1.000 | 2768 | tags=63%, list=23%, signal=81% |
| 585 | GOBP\_REGULATION\_OF\_ORGANELLE\_TRANSPORT\_ALONG\_MICROTUBULE |  | 8 | -0.69 | -1.50 | 0.029 | 0.281 | 1.000 | 1749 | tags=38%, list=15%, signal=44% |
| 586 | GOBP\_LEUKOCYTE\_MIGRATION |  | 270 | -0.39 | -1.50 | 0.000 | 0.282 | 1.000 | 3217 | tags=40%, list=27%, signal=53% |
| 587 | GOBP\_TOLERANCE\_INDUCTION |  | 14 | -0.59 | -1.50 | 0.040 | 0.282 | 1.000 | 1983 | tags=50%, list=17%, signal=60% |
| 588 | GOMF\_CAMP\_BINDING |  | 14 | -0.59 | -1.50 | 0.031 | 0.281 | 1.000 | 1323 | tags=36%, list=11%, signal=40% |
| 589 | GOBP\_CYCLIC\_NUCLEOTIDE\_CATABOLIC\_PROCESS |  | 11 | -0.63 | -1.50 | 0.034 | 0.281 | 1.000 | 2481 | tags=55%, list=21%, signal=69% |
| 590 | GOBP\_NEGATIVE\_REGULATION\_OF\_EXTRINSIC\_APOPTOTIC\_SIGNALING\_PATHWAY\_VIA\_DEATH\_DOMAIN\_RECEPTORS |  | 29 | -0.50 | -1.50 | 0.026 | 0.281 | 1.000 | 2731 | tags=48%, list=23%, signal=62% |
| 591 | GOBP\_POSITIVE\_REGULATION\_OF\_NOTCH\_SIGNALING\_PATHWAY |  | 42 | -0.47 | -1.50 | 0.019 | 0.282 | 1.000 | 4415 | tags=62%, list=37%, signal=98% |
| 592 | GOBP\_ACTIVATION\_OF\_TRANSMEMBRANE\_RECEPTOR\_PROTEIN\_TYROSINE\_KINASE\_ACTIVITY |  | 10 | -0.63 | -1.50 | 0.035 | 0.282 | 1.000 | 648 | tags=30%, list=5%, signal=32% |
| 593 | GOMF\_NADPLUS\_KINASE\_ACTIVITY |  | 14 | -0.59 | -1.50 | 0.036 | 0.284 | 1.000 | 648 | tags=36%, list=5%, signal=38% |
| 594 | GOBP\_ACTIVATED\_T\_CELL\_PROLIFERATION |  | 24 | -0.52 | -1.50 | 0.026 | 0.284 | 1.000 | 3163 | tags=54%, list=26%, signal=73% |
| 595 | GOBP\_SEBACEOUS\_GLAND\_DEVELOPMENT |  | 5 | -0.77 | -1.50 | 0.024 | 0.284 | 1.000 | 2212 | tags=80%, list=18%, signal=98% |
| 596 | GOBP\_REGULATION\_OF\_SYNAPSE\_STRUCTURE\_OR\_ACTIVITY |  | 139 | -0.40 | -1.50 | 0.002 | 0.283 | 1.000 | 3510 | tags=40%, list=29%, signal=55% |
| 597 | GOBP\_ADHERENS\_JUNCTION\_ORGANIZATION |  | 50 | -0.45 | -1.50 | 0.017 | 0.285 | 1.000 | 3269 | tags=38%, list=27%, signal=52% |
| 598 | GOMF\_BHLH\_TRANSCRIPTION\_FACTOR\_BINDING |  | 22 | -0.53 | -1.50 | 0.027 | 0.284 | 1.000 | 2656 | tags=36%, list=22%, signal=47% |
| 599 | GOBP\_INDOLE\_CONTAINING\_COMPOUND\_METABOLIC\_PROCESS |  | 11 | -0.62 | -1.50 | 0.033 | 0.284 | 1.000 | 2516 | tags=45%, list=21%, signal=57% |
| 600 | GOBP\_REGULATION\_OF\_CHROMATIN\_ASSEMBLY\_OR\_DISASSEMBLY |  | 15 | -0.58 | -1.50 | 0.039 | 0.285 | 1.000 | 1784 | tags=40%, list=15%, signal=47% |
| 601 | GOMF\_ADENYLATE\_CYCLASE\_ACTIVITY |  | 6 | -0.73 | -1.50 | 0.030 | 0.285 | 1.000 | 1392 | tags=50%, list=12%, signal=57% |
| 602 | GOBP\_POSITIVE\_REGULATION\_OF\_GAMMA\_DELTA\_T\_CELL\_ACTIVATION |  | 6 | -0.75 | -1.50 | 0.030 | 0.284 | 1.000 | 2357 | tags=67%, list=20%, signal=83% |
| 603 | GOBP\_PROTEIN\_AUTOPHOSPHORYLATION |  | 172 | -0.40 | -1.50 | 0.001 | 0.288 | 1.000 | 3980 | tags=45%, list=33%, signal=67% |
| 604 | GOBP\_NEGATIVE\_REGULATION\_OF\_CELLULAR\_AMINE\_METABOLIC\_PROCESS |  | 3 | -0.90 | -1.50 | 0.016 | 0.289 | 1.000 | 319 | tags=67%, list=3%, signal=68% |
| 605 | GOBP\_POSITIVE\_REGULATION\_OF\_RHO\_PROTEIN\_SIGNAL\_TRANSDUCTION |  | 26 | -0.51 | -1.50 | 0.030 | 0.289 | 1.000 | 3286 | tags=46%, list=27%, signal=63% |
| 606 | GOMF\_INSULIN\_RECEPTOR\_SUBSTRATE\_BINDING |  | 10 | -0.64 | -1.50 | 0.033 | 0.290 | 1.000 | 2158 | tags=60%, list=18%, signal=73% |
| 607 | GOBP\_T\_CELL\_ACTIVATION\_INVOLVED\_IN\_IMMUNE\_RESPONSE |  | 57 | -0.44 | -1.50 | 0.012 | 0.289 | 1.000 | 2912 | tags=44%, list=24%, signal=58% |
| 608 | GOBP\_MICROTUBULE\_NUCLEATION\_BY\_MICROTUBULE\_ORGANIZING\_CENTER |  | 5 | -0.77 | -1.50 | 0.026 | 0.291 | 1.000 | 1249 | tags=60%, list=10%, signal=67% |
| 609 | GOBP\_REGULATION\_OF\_GERMINAL\_CENTER\_FORMATION |  | 6 | -0.74 | -1.50 | 0.028 | 0.291 | 1.000 | 2474 | tags=83%, list=21%, signal=105% |
| 610 | GOBP\_POSITIVE\_REGULATION\_OF\_PROTEIN\_IMPORT |  | 33 | -0.49 | -1.50 | 0.027 | 0.291 | 1.000 | 3565 | tags=42%, list=30%, signal=60% |
| 611 | GOMF\_DNA\_REPLICATION\_ORIGIN\_BINDING |  | 19 | -0.54 | -1.50 | 0.036 | 0.291 | 1.000 | 4465 | tags=74%, list=37%, signal=117% |
| 612 | GOBP\_RETINOL\_METABOLIC\_PROCESS |  | 28 | -0.50 | -1.50 | 0.028 | 0.290 | 1.000 | 2278 | tags=32%, list=19%, signal=40% |
| 613 | GOBP\_MYELIN\_ASSEMBLY |  | 16 | -0.57 | -1.50 | 0.034 | 0.293 | 1.000 | 1048 | tags=31%, list=9%, signal=34% |
| 614 | GOBP\_NEURON\_DIFFERENTIATION |  | 913 | -0.37 | -1.50 | 0.000 | 0.293 | 1.000 | 3797 | tags=40%, list=32%, signal=54% |
| 615 | GOBP\_REGULATION\_OF\_ORGAN\_GROWTH |  | 62 | -0.44 | -1.49 | 0.011 | 0.293 | 1.000 | 3613 | tags=47%, list=30%, signal=67% |
| 616 | GOBP\_LYMPHATIC\_ENDOTHELIAL\_CELL\_DIFFERENTIATION |  | 7 | -0.70 | -1.49 | 0.030 | 0.293 | 1.000 | 540 | tags=43%, list=4%, signal=45% |
| 617 | GOBP\_CELL\_MATRIX\_ADHESION |  | 165 | -0.40 | -1.49 | 0.001 | 0.293 | 1.000 | 3575 | tags=42%, list=30%, signal=60% |
| 618 | GOBP\_MAMMARY\_GLAND\_ALVEOLUS\_DEVELOPMENT |  | 14 | -0.59 | -1.49 | 0.035 | 0.293 | 1.000 | 3196 | tags=43%, list=27%, signal=58% |
| 619 | GOBP\_POSITIVE\_REGULATION\_OF\_MACROPHAGE\_MIGRATION |  | 16 | -0.57 | -1.49 | 0.034 | 0.295 | 1.000 | 3093 | tags=56%, list=26%, signal=76% |
| 620 | GOBP\_REGULATION\_OF\_PROTEIN\_SERINE\_THREONINE\_KINASE\_ACTIVITY |  | 348 | -0.38 | -1.49 | 0.000 | 0.295 | 1.000 | 3723 | tags=39%, list=31%, signal=55% |
| 621 | GOBP\_HISTONE\_H3\_K4\_METHYLATION |  | 38 | -0.47 | -1.49 | 0.025 | 0.296 | 1.000 | 4698 | tags=68%, list=39%, signal=112% |
| 622 | GOBP\_EXTRINSIC\_APOPTOTIC\_SIGNALING\_PATHWAY |  | 149 | -0.40 | -1.49 | 0.002 | 0.296 | 1.000 | 2820 | tags=36%, list=23%, signal=46% |
| 623 | GOBP\_REGULATION\_OF\_SPROUTING\_ANGIOGENESIS |  | 23 | -0.52 | -1.49 | 0.031 | 0.295 | 1.000 | 3905 | tags=65%, list=33%, signal=96% |
| 624 | GOBP\_REGULATION\_OF\_SISTER\_CHROMATID\_COHESION |  | 18 | -0.55 | -1.49 | 0.037 | 0.295 | 1.000 | 3836 | tags=61%, list=32%, signal=90% |
| 625 | GOBP\_REGULATION\_OF\_B\_CELL\_APOPTOTIC\_PROCESS |  | 15 | -0.57 | -1.49 | 0.035 | 0.296 | 1.000 | 1560 | tags=40%, list=13%, signal=46% |
| 626 | GOCC\_ANCHORED\_COMPONENT\_OF\_EXTERNAL\_SIDE\_OF\_PLASMA\_MEMBRANE |  | 8 | -0.67 | -1.49 | 0.035 | 0.297 | 1.000 | 533 | tags=25%, list=4%, signal=26% |
| 627 | GOBP\_POSITIVE\_REGULATION\_OF\_HISTONE\_METHYLATION |  | 27 | -0.50 | -1.49 | 0.032 | 0.296 | 1.000 | 4698 | tags=70%, list=39%, signal=115% |
| 628 | GOCC\_ADHERENS\_JUNCTION |  | 128 | -0.40 | -1.49 | 0.003 | 0.296 | 1.000 | 3196 | tags=36%, list=27%, signal=48% |
| 629 | GOMF\_RNA\_POLYMERASE\_II\_TRANSCRIPTION\_FACTOR\_BINDING |  | 33 | -0.49 | -1.49 | 0.027 | 0.296 | 1.000 | 3561 | tags=55%, list=30%, signal=77% |
| 630 | GOBP\_POSITIVE\_REGULATION\_OF\_NEURAL\_PRECURSOR\_CELL\_PROLIFERATION |  | 36 | -0.48 | -1.49 | 0.022 | 0.295 | 1.000 | 3196 | tags=44%, list=27%, signal=60% |
| 631 | GOBP\_RADIAL\_GLIAL\_CELL\_DIFFERENTIATION |  | 8 | -0.68 | -1.49 | 0.034 | 0.295 | 1.000 | 1146 | tags=50%, list=10%, signal=55% |
| 632 | GOBP\_KINETOCHORE\_ASSEMBLY |  | 16 | -0.56 | -1.49 | 0.035 | 0.296 | 1.000 | 4279 | tags=81%, list=36%, signal=126% |
| 633 | GOMF\_ACETYLCHOLINE\_BINDING |  | 6 | -0.74 | -1.49 | 0.027 | 0.295 | 1.000 | 205 | tags=50%, list=2%, signal=51% |
| 634 | GOBP\_SMOOTH\_MUSCLE\_CONTRACTION |  | 78 | -0.43 | -1.49 | 0.010 | 0.295 | 1.000 | 1411 | tags=24%, list=12%, signal=27% |
| 635 | GOBP\_REACTIVE\_OXYGEN\_SPECIES\_BIOSYNTHETIC\_PROCESS |  | 73 | -0.43 | -1.49 | 0.012 | 0.295 | 1.000 | 2518 | tags=36%, list=21%, signal=45% |
| 636 | GOBP\_NEGATIVE\_REGULATION\_OF\_TELOMERE\_MAINTENANCE\_VIA\_TELOMERE\_LENGTHENING |  | 20 | -0.54 | -1.49 | 0.037 | 0.295 | 1.000 | 3722 | tags=60%, list=31%, signal=87% |
| 637 | GOBP\_REGULATION\_OF\_LAMELLIPODIUM\_ORGANIZATION |  | 39 | -0.47 | -1.49 | 0.025 | 0.295 | 1.000 | 1743 | tags=31%, list=15%, signal=36% |
| 638 | GOMF\_LYSOPHOSPHOLIPID\_ACYLTRANSFERASE\_ACTIVITY |  | 16 | -0.56 | -1.49 | 0.038 | 0.294 | 1.000 | 639 | tags=19%, list=5%, signal=20% |
| 639 | GOCC\_CHROMOSOMAL\_REGION |  | 262 | -0.39 | -1.49 | 0.000 | 0.295 | 1.000 | 5914 | tags=67%, list=49%, signal=129% |
| 640 | GOMF\_TPR\_DOMAIN\_BINDING |  | 6 | -0.74 | -1.49 | 0.029 | 0.295 | 1.000 | 2307 | tags=50%, list=19%, signal=62% |
| 641 | GOBP\_HISTONE\_H3\_K9\_DEMETHYLATION |  | 9 | -0.66 | -1.49 | 0.045 | 0.295 | 1.000 | 4114 | tags=100%, list=34%, signal=152% |
| 642 | GOCC\_GROWTH\_FACTOR\_COMPLEX |  | 3 | -0.89 | -1.49 | 0.017 | 0.295 | 1.000 | 153 | tags=33%, list=1%, signal=34% |
| 643 | GOBP\_DETECTION\_OF\_LIGHT\_STIMULUS\_INVOLVED\_IN\_SENSORY\_PERCEPTION |  | 8 | -0.68 | -1.49 | 0.037 | 0.296 | 1.000 | 3525 | tags=88%, list=29%, signal=124% |
| 644 | GOMF\_KINASE\_ACTIVITY |  | 542 | -0.38 | -1.49 | 0.000 | 0.295 | 1.000 | 3657 | tags=42%, list=30%, signal=57% |
| 645 | GOBP\_T\_CELL\_PROLIFERATION |  | 113 | -0.41 | -1.49 | 0.003 | 0.295 | 1.000 | 3163 | tags=41%, list=26%, signal=55% |
| 646 | GOBP\_PLATELET\_MORPHOGENESIS |  | 17 | -0.55 | -1.49 | 0.036 | 0.295 | 1.000 | 2474 | tags=41%, list=21%, signal=52% |
| 647 | GOBP\_B\_CELL\_PROLIFERATION |  | 53 | -0.45 | -1.49 | 0.017 | 0.294 | 1.000 | 2547 | tags=38%, list=21%, signal=48% |
| 648 | GOMF\_NEUROPEPTIDE\_HORMONE\_ACTIVITY |  | 10 | -0.64 | -1.49 | 0.038 | 0.295 | 1.000 | 2351 | tags=70%, list=20%, signal=87% |
| 649 | GOBP\_SEGMENTATION |  | 59 | -0.44 | -1.49 | 0.018 | 0.295 | 1.000 | 3596 | tags=41%, list=30%, signal=58% |
| 650 | GOBP\_REGULATION\_OF\_DOUBLE\_STRAND\_BREAK\_REPAIR\_VIA\_HOMOLOGOUS\_RECOMBINATION |  | 34 | -0.48 | -1.49 | 0.029 | 0.295 | 1.000 | 3434 | tags=50%, list=29%, signal=70% |
| 651 | GOBP\_POSITIVE\_REGULATION\_OF\_ERK1\_AND\_ERK2\_CASCADE |  | 116 | -0.41 | -1.49 | 0.005 | 0.295 | 1.000 | 2791 | tags=37%, list=23%, signal=48% |
| 652 | GOCC\_KINETOCHORE |  | 116 | -0.41 | -1.49 | 0.004 | 0.295 | 1.000 | 5914 | tags=67%, list=49%, signal=131% |
| 653 | GOBP\_REGULATION\_OF\_CELL\_PROJECTION\_ORGANIZATION |  | 449 | -0.38 | -1.49 | 0.000 | 0.295 | 1.000 | 2688 | tags=31%, list=22%, signal=38% |
| 654 | GOBP\_NEGATIVE\_REGULATION\_OF\_DOUBLE\_STRAND\_BREAK\_REPAIR\_VIA\_HOMOLOGOUS\_RECOMBINATION |  | 12 | -0.61 | -1.49 | 0.037 | 0.295 | 1.000 | 2379 | tags=58%, list=20%, signal=73% |
| 655 | GOBP\_POSITIVE\_REGULATION\_OF\_CHROMOSOME\_ORGANIZATION |  | 121 | -0.40 | -1.49 | 0.005 | 0.296 | 1.000 | 3998 | tags=47%, list=33%, signal=70% |
| 656 | GOBP\_CAMP\_BIOSYNTHETIC\_PROCESS |  | 6 | -0.73 | -1.49 | 0.034 | 0.298 | 1.000 | 1392 | tags=50%, list=12%, signal=57% |
| 657 | GOCC\_EQUATORIAL\_MICROTUBULE\_ORGANIZING\_CENTER |  | 5 | -0.77 | -1.49 | 0.030 | 0.297 | 1.000 | 2265 | tags=80%, list=19%, signal=99% |
| 658 | GOBP\_MARGINAL\_ZONE\_B\_CELL\_DIFFERENTIATION |  | 8 | -0.68 | -1.49 | 0.033 | 0.297 | 1.000 | 1432 | tags=50%, list=12%, signal=57% |
| 659 | GOBP\_POSITIVE\_REGULATION\_OF\_STEM\_CELL\_PROLIFERATION |  | 15 | -0.57 | -1.49 | 0.045 | 0.298 | 1.000 | 2798 | tags=47%, list=23%, signal=61% |
| 660 | GOBP\_CELL\_MIGRATION |  | 975 | -0.37 | -1.49 | 0.000 | 0.298 | 1.000 | 3231 | tags=36%, list=27%, signal=45% |
| 661 | GOBP\_REGULATION\_OF\_TRANS\_SYNAPTIC\_SIGNALING |  | 259 | -0.39 | -1.49 | 0.000 | 0.297 | 1.000 | 3833 | tags=43%, list=32%, signal=62% |
| 662 | GOBP\_ACTIVATION\_OF\_PROTEIN\_KINASE\_A\_ACTIVITY |  | 12 | -0.61 | -1.49 | 0.041 | 0.297 | 1.000 | 2997 | tags=58%, list=25%, signal=78% |
| 663 | GOBP\_HAIR\_CYCLE\_PHASE |  | 6 | -0.73 | -1.49 | 0.029 | 0.297 | 1.000 | 87 | tags=17%, list=1%, signal=17% |
| 664 | GOBP\_REGULATION\_OF\_CARTILAGE\_DEVELOPMENT |  | 42 | -0.46 | -1.49 | 0.025 | 0.298 | 1.000 | 2002 | tags=38%, list=17%, signal=46% |
| 665 | GOMF\_SIGNALING\_RECEPTOR\_COMPLEX\_ADAPTOR\_ACTIVITY |  | 33 | -0.48 | -1.48 | 0.025 | 0.298 | 1.000 | 1603 | tags=33%, list=13%, signal=38% |
| 666 | GOBP\_SYNAPSE\_ORGANIZATION |  | 264 | -0.39 | -1.48 | 0.000 | 0.298 | 1.000 | 3961 | tags=41%, list=33%, signal=60% |
| 667 | GOBP\_ADHERENS\_JUNCTION\_ASSEMBLY |  | 12 | -0.60 | -1.48 | 0.044 | 0.298 | 1.000 | 2811 | tags=42%, list=23%, signal=54% |
| 668 | GOBP\_G\_PROTEIN\_COUPLED\_RECEPTOR\_SIGNALING\_PATHWAY |  | 483 | -0.38 | -1.48 | 0.000 | 0.299 | 1.000 | 3321 | tags=39%, list=28%, signal=52% |
| 669 | GOBP\_DENDRITIC\_SPINE\_DEVELOPMENT |  | 77 | -0.42 | -1.48 | 0.014 | 0.299 | 1.000 | 3483 | tags=42%, list=29%, signal=58% |
| 670 | GOBP\_POSITIVE\_REGULATION\_OF\_CHEMOTAXIS |  | 95 | -0.41 | -1.48 | 0.007 | 0.299 | 1.000 | 3286 | tags=40%, list=27%, signal=55% |
| 671 | GOMF\_WATER\_TRANSMEMBRANE\_TRANSPORTER\_ACTIVITY |  | 7 | -0.70 | -1.48 | 0.035 | 0.299 | 1.000 | 1035 | tags=43%, list=9%, signal=47% |
| 672 | GOBP\_BIOLOGICAL\_PHASE |  | 6 | -0.73 | -1.48 | 0.032 | 0.299 | 1.000 | 87 | tags=17%, list=1%, signal=17% |
| 673 | GOBP\_POSITIVE\_REGULATION\_OF\_SUBSTRATE\_ADHESION\_DEPENDENT\_CELL\_SPREADING |  | 33 | -0.48 | -1.48 | 0.027 | 0.299 | 1.000 | 1777 | tags=30%, list=15%, signal=35% |
| 674 | GOBP\_GAMMA\_DELTA\_T\_CELL\_DIFFERENTIATION |  | 9 | -0.65 | -1.48 | 0.039 | 0.299 | 1.000 | 2357 | tags=56%, list=20%, signal=69% |
| 675 | GOBP\_REGULATION\_OF\_NEUTROPHIL\_MIGRATION |  | 32 | -0.48 | -1.48 | 0.026 | 0.299 | 1.000 | 2679 | tags=44%, list=22%, signal=56% |
| 676 | GOBP\_REGULATION\_OF\_PROTEIN\_CONTAINING\_COMPLEX\_DISASSEMBLY |  | 86 | -0.42 | -1.48 | 0.010 | 0.301 | 1.000 | 3148 | tags=37%, list=26%, signal=50% |
| 677 | GOCC\_PLATELET\_DENSE\_TUBULAR\_NETWORK |  | 7 | -0.70 | -1.48 | 0.035 | 0.301 | 1.000 | 351 | tags=29%, list=3%, signal=29% |
| 678 | GOBP\_DOSAGE\_COMPENSATION |  | 14 | -0.58 | -1.48 | 0.040 | 0.301 | 1.000 | 2424 | tags=43%, list=20%, signal=54% |
| 679 | GOBP\_PROTEIN\_LOCALIZATION\_TO\_CHROMOSOME |  | 74 | -0.43 | -1.48 | 0.012 | 0.301 | 1.000 | 5796 | tags=70%, list=48%, signal=135% |
| 680 | GOBP\_FEEDING\_BEHAVIOR |  | 43 | -0.46 | -1.48 | 0.020 | 0.301 | 1.000 | 2007 | tags=37%, list=17%, signal=45% |
| 681 | GOBP\_CHROMATIN\_ORGANIZATION |  | 513 | -0.38 | -1.48 | 0.000 | 0.302 | 1.000 | 4528 | tags=50%, list=38%, signal=76% |
| 682 | GOBP\_LYMPHOCYTE\_APOPTOTIC\_PROCESS |  | 50 | -0.45 | -1.48 | 0.020 | 0.301 | 1.000 | 1852 | tags=34%, list=15%, signal=40% |
| 683 | GOBP\_SISTER\_CHROMATID\_COHESION |  | 44 | -0.46 | -1.48 | 0.025 | 0.301 | 1.000 | 5914 | tags=77%, list=49%, signal=152% |
| 684 | GOMF\_CGMP\_BINDING |  | 7 | -0.70 | -1.48 | 0.035 | 0.301 | 1.000 | 2524 | tags=57%, list=21%, signal=72% |
| 685 | GOBP\_RECEPTOR\_SIGNALING\_PATHWAY\_VIA\_STAT |  | 93 | -0.41 | -1.48 | 0.009 | 0.301 | 1.000 | 1608 | tags=28%, list=13%, signal=32% |
| 686 | GOBP\_POSITIVE\_REGULATION\_OF\_GLUCOSE\_TRANSMEMBRANE\_TRANSPORT |  | 31 | -0.49 | -1.48 | 0.028 | 0.304 | 1.000 | 1991 | tags=35%, list=17%, signal=42% |
| 687 | GOBP\_CELL\_CYCLE\_CHECKPOINT |  | 166 | -0.39 | -1.48 | 0.001 | 0.304 | 1.000 | 3988 | tags=42%, list=33%, signal=62% |
| 688 | GOBP\_SEMICIRCULAR\_CANAL\_DEVELOPMENT |  | 4 | -0.82 | -1.48 | 0.028 | 0.304 | 1.000 | 1146 | tags=50%, list=10%, signal=55% |
| 689 | GOBP\_MEIOTIC\_CHROMOSOME\_SEGREGATION |  | 52 | -0.45 | -1.48 | 0.019 | 0.306 | 1.000 | 4146 | tags=52%, list=35%, signal=79% |
| 690 | GOMF\_PLATELET\_DERIVED\_GROWTH\_FACTOR\_RECEPTOR\_BINDING |  | 14 | -0.58 | -1.48 | 0.045 | 0.306 | 1.000 | 3487 | tags=57%, list=29%, signal=80% |
| 691 | GOBP\_CARDIAC\_SEPTUM\_DEVELOPMENT |  | 77 | -0.42 | -1.48 | 0.011 | 0.306 | 1.000 | 2308 | tags=30%, list=19%, signal=37% |
| 692 | GOBP\_ADAPTIVE\_IMMUNE\_RESPONSE |  | 217 | -0.39 | -1.48 | 0.001 | 0.306 | 1.000 | 2656 | tags=37%, list=22%, signal=47% |
| 693 | GOBP\_NEGATIVE\_REGULATION\_OF\_T\_CELL\_APOPTOTIC\_PROCESS |  | 13 | -0.59 | -1.48 | 0.043 | 0.306 | 1.000 | 2362 | tags=62%, list=20%, signal=77% |
| 694 | GOBP\_REGULATION\_OF\_REGULATED\_SECRETORY\_PATHWAY |  | 89 | -0.42 | -1.48 | 0.010 | 0.306 | 1.000 | 3909 | tags=43%, list=33%, signal=63% |
| 695 | GOBP\_NEGATIVE\_REGULATION\_OF\_MUSCLE\_ORGAN\_DEVELOPMENT |  | 8 | -0.67 | -1.48 | 0.038 | 0.306 | 1.000 | 668 | tags=25%, list=6%, signal=26% |
| 696 | GOBP\_POSITIVE\_REGULATION\_OF\_REACTIVE\_OXYGEN\_SPECIES\_BIOSYNTHETIC\_PROCESS |  | 33 | -0.48 | -1.48 | 0.028 | 0.305 | 1.000 | 2010 | tags=30%, list=17%, signal=36% |
| 697 | GOBP\_POSITIVE\_REGULATION\_OF\_LIPID\_KINASE\_ACTIVITY |  | 28 | -0.50 | -1.48 | 0.029 | 0.305 | 1.000 | 3286 | tags=39%, list=27%, signal=54% |
| 698 | GOBP\_MEIOSIS\_I\_CELL\_CYCLE\_PROCESS |  | 70 | -0.43 | -1.48 | 0.012 | 0.305 | 1.000 | 5395 | tags=63%, list=45%, signal=113% |
| 699 | GOMF\_OPSONIN\_BINDING |  | 12 | -0.60 | -1.48 | 0.041 | 0.305 | 1.000 | 2448 | tags=42%, list=20%, signal=52% |
| 700 | GOBP\_NEGATIVE\_REGULATION\_OF\_NF\_KAPPAB\_TRANSCRIPTION\_FACTOR\_ACTIVITY |  | 50 | -0.45 | -1.48 | 0.022 | 0.305 | 1.000 | 3132 | tags=40%, list=26%, signal=54% |
| 701 | GOMF\_GLYCEROPHOSPHOLIPID\_FLIPPASE\_ACTIVITY |  | 11 | -0.62 | -1.48 | 0.040 | 0.306 | 1.000 | 1410 | tags=45%, list=12%, signal=51% |
| 702 | GOBP\_POSITIVE\_REGULATION\_OF\_CHROMATIN\_ORGANIZATION |  | 68 | -0.43 | -1.48 | 0.013 | 0.306 | 1.000 | 3640 | tags=49%, list=30%, signal=69% |
| 703 | GOBP\_PROTEIN\_LOCALIZATION\_TO\_PRESYNAPSE |  | 7 | -0.70 | -1.48 | 0.041 | 0.306 | 1.000 | 3668 | tags=100%, list=31%, signal=144% |
| 704 | GOBP\_KERATINOCYTE\_DEVELOPMENT |  | 5 | -0.77 | -1.48 | 0.033 | 0.306 | 1.000 | 2730 | tags=80%, list=23%, signal=103% |
| 705 | GOBP\_HINDLIMB\_MORPHOGENESIS |  | 20 | -0.53 | -1.48 | 0.036 | 0.306 | 1.000 | 3890 | tags=55%, list=32%, signal=81% |
| 706 | GOBP\_TUBE\_FORMATION |  | 100 | -0.41 | -1.48 | 0.008 | 0.307 | 1.000 | 4271 | tags=46%, list=36%, signal=71% |
| 707 | GOBP\_BLASTOCYST\_FORMATION |  | 27 | -0.50 | -1.48 | 0.031 | 0.307 | 1.000 | 3279 | tags=48%, list=27%, signal=66% |
| 708 | GOBP\_REFLEX |  | 7 | -0.69 | -1.47 | 0.037 | 0.309 | 1.000 | 1871 | tags=57%, list=16%, signal=68% |
| 709 | GOMF\_ATPASE\_ACTIVITY |  | 336 | -0.38 | -1.47 | 0.000 | 0.309 | 1.000 | 4143 | tags=48%, list=34%, signal=71% |
| 710 | GOBP\_REGULATION\_OF\_T\_CELL\_APOPTOTIC\_PROCESS |  | 21 | -0.53 | -1.47 | 0.041 | 0.309 | 1.000 | 2362 | tags=48%, list=20%, signal=59% |
| 711 | GOBP\_REGULATION\_OF\_LONG\_TERM\_SYNAPTIC\_POTENTIATION |  | 28 | -0.49 | -1.47 | 0.034 | 0.309 | 1.000 | 2871 | tags=46%, list=24%, signal=61% |
| 712 | GOMF\_HISTONE\_BINDING |  | 161 | -0.39 | -1.47 | 0.002 | 0.312 | 1.000 | 4512 | tags=52%, list=38%, signal=81% |
| 713 | GOMF\_MANNOSYL\_OLIGOSACCHARIDE\_1\_2\_ALPHA\_MANNOSIDASE\_ACTIVITY |  | 7 | -0.70 | -1.47 | 0.043 | 0.311 | 1.000 | 2715 | tags=71%, list=23%, signal=92% |
| 714 | GOBP\_REGULATION\_OF\_NEUROTRANSMITTER\_LEVELS |  | 139 | -0.40 | -1.47 | 0.003 | 0.311 | 1.000 | 3946 | tags=50%, list=33%, signal=73% |
| 715 | GOBP\_DOPAMINE\_METABOLIC\_PROCESS |  | 22 | -0.52 | -1.47 | 0.041 | 0.311 | 1.000 | 2569 | tags=45%, list=21%, signal=58% |
| 716 | GOCC\_FACIT\_COLLAGEN\_TRIMER |  | 5 | -0.77 | -1.47 | 0.031 | 0.311 | 1.000 | 1493 | tags=60%, list=12%, signal=68% |
| 717 | GOBP\_NEGATIVE\_REGULATION\_OF\_PLATELET\_AGGREGATION |  | 8 | -0.67 | -1.47 | 0.043 | 0.312 | 1.000 | 1993 | tags=38%, list=17%, signal=45% |
| 718 | GOBP\_INTESTINAL\_LIPID\_ABSORPTION |  | 11 | -0.61 | -1.47 | 0.048 | 0.312 | 1.000 | 2915 | tags=55%, list=24%, signal=72% |
| 719 | GOBP\_POSITIVE\_REGULATION\_OF\_STRIATED\_MUSCLE\_CELL\_DIFFERENTIATION |  | 22 | -0.52 | -1.47 | 0.044 | 0.312 | 1.000 | 3565 | tags=50%, list=30%, signal=71% |
| 720 | GOBP\_BUNDLE\_OF\_HIS\_CELL\_TO\_PURKINJE\_MYOCYTE\_COMMUNICATION |  | 8 | -0.67 | -1.47 | 0.039 | 0.312 | 1.000 | 3918 | tags=75%, list=33%, signal=111% |
| 721 | GOBP\_REGULATION\_OF\_CELL\_MATRIX\_ADHESION |  | 93 | -0.41 | -1.47 | 0.010 | 0.312 | 1.000 | 3575 | tags=43%, list=30%, signal=61% |
| 722 | GOBP\_RENAL\_WATER\_HOMEOSTASIS |  | 24 | -0.51 | -1.47 | 0.037 | 0.312 | 1.000 | 4035 | tags=63%, list=34%, signal=94% |
| 723 | GOBP\_FEAR\_RESPONSE |  | 20 | -0.53 | -1.47 | 0.041 | 0.312 | 1.000 | 4572 | tags=70%, list=38%, signal=113% |
| 724 | GOBP\_LIPOPOLYSACCHARIDE\_MEDIATED\_SIGNALING\_PATHWAY |  | 40 | -0.46 | -1.47 | 0.026 | 0.313 | 1.000 | 3613 | tags=45%, list=30%, signal=64% |
| 725 | GOBP\_REGULATION\_OF\_CELLULAR\_COMPONENT\_MOVEMENT |  | 701 | -0.37 | -1.47 | 0.000 | 0.312 | 1.000 | 3728 | tags=40%, list=31%, signal=55% |
| 726 | GOMF\_HYDROLASE\_ACTIVITY\_ACTING\_ON\_GLYCOSYL\_BONDS |  | 85 | -0.42 | -1.47 | 0.012 | 0.312 | 1.000 | 3487 | tags=46%, list=29%, signal=64% |
| 727 | GOBP\_REGULATION\_OF\_CHEMOTAXIS |  | 147 | -0.39 | -1.47 | 0.003 | 0.312 | 1.000 | 3196 | tags=37%, list=27%, signal=50% |
| 728 | GOBP\_ENDOSOME\_TO\_LYSOSOME\_TRANSPORT |  | 46 | -0.45 | -1.47 | 0.024 | 0.312 | 1.000 | 2774 | tags=35%, list=23%, signal=45% |
| 729 | GOBP\_IMMUNE\_RESPONSE\_REGULATING\_SIGNALING\_PATHWAY |  | 252 | -0.38 | -1.47 | 0.000 | 0.311 | 1.000 | 3143 | tags=35%, list=26%, signal=46% |
| 730 | GOBP\_PEPTIDYL\_LYSINE\_ACETYLATION |  | 131 | -0.40 | -1.47 | 0.004 | 0.311 | 1.000 | 3626 | tags=40%, list=30%, signal=56% |
| 731 | GOBP\_REGULATION\_OF\_CELL\_MORPHOGENESIS\_INVOLVED\_IN\_DIFFERENTIATION |  | 75 | -0.42 | -1.47 | 0.014 | 0.311 | 1.000 | 1786 | tags=25%, list=15%, signal=30% |
| 732 | GOBP\_POSITIVE\_REGULATION\_OF\_GRANULOCYTE\_CHEMOTAXIS |  | 19 | -0.54 | -1.47 | 0.041 | 0.311 | 1.000 | 2679 | tags=42%, list=22%, signal=54% |
| 733 | GOBP\_POSITIVE\_REGULATION\_OF\_ANTIGEN\_RECEPTOR\_MEDIATED\_SIGNALING\_PATHWAY |  | 17 | -0.55 | -1.47 | 0.040 | 0.310 | 1.000 | 2640 | tags=47%, list=22%, signal=60% |
| 734 | GOBP\_MICROTUBULE\_ANCHORING |  | 18 | -0.54 | -1.47 | 0.044 | 0.310 | 1.000 | 2548 | tags=50%, list=21%, signal=63% |
| 735 | GOBP\_DIACYLGLYCEROL\_METABOLIC\_PROCESS |  | 20 | -0.53 | -1.47 | 0.046 | 0.311 | 1.000 | 2857 | tags=45%, list=24%, signal=59% |
| 736 | GOBP\_REGULATION\_OF\_BEHAVIOR |  | 33 | -0.48 | -1.47 | 0.030 | 0.311 | 1.000 | 2474 | tags=42%, list=21%, signal=53% |
| 737 | GOBP\_REGULATION\_OF\_PROTEIN\_DEPOLYMERIZATION |  | 61 | -0.43 | -1.47 | 0.020 | 0.312 | 1.000 | 2634 | tags=34%, list=22%, signal=44% |
| 738 | GOCC\_SCHAFFER\_COLLATERAL\_CA1\_SYNAPSE |  | 40 | -0.46 | -1.47 | 0.031 | 0.312 | 1.000 | 4084 | tags=48%, list=34%, signal=72% |
| 739 | GOBP\_REGULATION\_OF\_CARDIAC\_MUSCLE\_CELL\_ACTION\_POTENTIAL |  | 18 | -0.55 | -1.47 | 0.040 | 0.312 | 1.000 | 3918 | tags=61%, list=33%, signal=91% |
| 740 | GOBP\_MAST\_CELL\_ACTIVATION\_INVOLVED\_IN\_IMMUNE\_RESPONSE |  | 31 | -0.48 | -1.47 | 0.032 | 0.313 | 1.000 | 2943 | tags=45%, list=25%, signal=60% |
| 741 | GOBP\_VESICLE\_FUSION\_TO\_PLASMA\_MEMBRANE |  | 13 | -0.59 | -1.47 | 0.049 | 0.314 | 1.000 | 1979 | tags=46%, list=16%, signal=55% |
| 742 | GOBP\_VENOUS\_BLOOD\_VESSEL\_MORPHOGENESIS |  | 7 | -0.69 | -1.47 | 0.045 | 0.314 | 1.000 | 3256 | tags=71%, list=27%, signal=98% |
| 743 | GOCC\_CLATHRIN\_COAT |  | 34 | -0.47 | -1.47 | 0.033 | 0.314 | 1.000 | 4775 | tags=65%, list=40%, signal=107% |
| 744 | GOBP\_MYELOID\_LEUKOCYTE\_CYTOKINE\_PRODUCTION |  | 22 | -0.52 | -1.47 | 0.039 | 0.313 | 1.000 | 2889 | tags=55%, list=24%, signal=72% |
| 745 | GOMF\_HIGH\_VOLTAGE\_GATED\_CALCIUM\_CHANNEL\_ACTIVITY |  | 6 | -0.72 | -1.47 | 0.036 | 0.313 | 1.000 | 1063 | tags=33%, list=9%, signal=37% |
| 746 | GOBP\_INORGANIC\_ANION\_TRANSPORT |  | 99 | -0.41 | -1.47 | 0.008 | 0.313 | 1.000 | 2884 | tags=43%, list=24%, signal=57% |
| 747 | GOCC\_CONDENSED\_CHROMOSOME\_CENTROMERIC\_REGION |  | 97 | -0.41 | -1.47 | 0.009 | 0.313 | 1.000 | 5914 | tags=66%, list=49%, signal=129% |
| 748 | GOBP\_NEGATIVE\_CHEMOTAXIS |  | 35 | -0.47 | -1.47 | 0.032 | 0.314 | 1.000 | 488 | tags=17%, list=4%, signal=18% |
| 749 | GOBP\_MESENCHYMAL\_EPITHELIAL\_CELL\_SIGNALING |  | 7 | -0.69 | -1.47 | 0.042 | 0.314 | 1.000 | 1146 | tags=43%, list=10%, signal=47% |
| 750 | GOBP\_NEGATIVE\_REGULATION\_OF\_PLATELET\_ACTIVATION |  | 12 | -0.60 | -1.47 | 0.054 | 0.314 | 1.000 | 1993 | tags=25%, list=17%, signal=30% |
| 751 | GOCC\_PLASMA\_MEMBRANE\_PROTEIN\_COMPLEX |  | 327 | -0.38 | -1.47 | 0.000 | 0.315 | 1.000 | 3318 | tags=39%, list=28%, signal=52% |
| 752 | GOBP\_MAST\_CELL\_PROLIFERATION |  | 5 | -0.76 | -1.47 | 0.039 | 0.315 | 1.000 | 1470 | tags=80%, list=12%, signal=91% |
| 753 | GOBP\_POSITIVE\_REGULATION\_OF\_PROTEIN\_PHOSPHORYLATION |  | 554 | -0.37 | -1.47 | 0.000 | 0.315 | 1.000 | 3408 | tags=36%, list=28%, signal=48% |
| 754 | GOBP\_DNA\_INTEGRITY\_CHECKPOINT |  | 122 | -0.40 | -1.47 | 0.007 | 0.316 | 1.000 | 3958 | tags=43%, list=33%, signal=64% |
| 755 | GOBP\_REGULATION\_OF\_MAST\_CELL\_ACTIVATION\_INVOLVED\_IN\_IMMUNE\_RESPONSE |  | 19 | -0.53 | -1.47 | 0.041 | 0.315 | 1.000 | 2920 | tags=42%, list=24%, signal=56% |
| 756 | GOBP\_URINARY\_TRACT\_SMOOTH\_MUSCLE\_CONTRACTION |  | 6 | -0.72 | -1.47 | 0.041 | 0.315 | 1.000 | 374 | tags=33%, list=3%, signal=34% |
| 757 | GOMF\_NAADP\_SENSITIVE\_CALCIUM\_RELEASE\_CHANNEL\_ACTIVITY |  | 4 | -0.81 | -1.46 | 0.027 | 0.316 | 1.000 | 1855 | tags=75%, list=15%, signal=89% |
| 758 | GOBP\_REGULATION\_OF\_MUSCLE\_ORGAN\_DEVELOPMENT |  | 26 | -0.50 | -1.46 | 0.037 | 0.315 | 1.000 | 2503 | tags=35%, list=21%, signal=44% |
| 759 | GOBP\_LYMPHOCYTE\_ACTIVATION\_INVOLVED\_IN\_IMMUNE\_RESPONSE |  | 116 | -0.40 | -1.46 | 0.008 | 0.316 | 1.000 | 3243 | tags=41%, list=27%, signal=56% |
| 760 | GOBP\_LAMELLIPODIUM\_ASSEMBLY |  | 54 | -0.44 | -1.46 | 0.021 | 0.316 | 1.000 | 2784 | tags=37%, list=23%, signal=48% |
| 761 | GOMF\_ALPHA\_GLUCOSIDASE\_ACTIVITY |  | 5 | -0.76 | -1.46 | 0.035 | 0.317 | 1.000 | 2814 | tags=80%, list=23%, signal=104% |
| 762 | GOBP\_REGULATION\_OF\_MEMBRANE\_LIPID\_DISTRIBUTION |  | 40 | -0.46 | -1.46 | 0.027 | 0.316 | 1.000 | 1410 | tags=33%, list=12%, signal=37% |
| 763 | GOBP\_REGULATION\_OF\_LYMPHANGIOGENESIS |  | 4 | -0.81 | -1.46 | 0.031 | 0.317 | 1.000 | 257 | tags=50%, list=2%, signal=51% |
| 764 | GOMF\_ACTIN\_DEPENDENT\_ATPASE\_ACTIVITY |  | 17 | -0.55 | -1.46 | 0.048 | 0.317 | 1.000 | 4122 | tags=65%, list=34%, signal=98% |
| 765 | GOBP\_DENDRITIC\_CELL\_DIFFERENTIATION |  | 28 | -0.49 | -1.46 | 0.038 | 0.316 | 1.000 | 3446 | tags=46%, list=29%, signal=65% |
| 766 | GOMF\_MAP\_KINASE\_KINASE\_KINASE\_ACTIVITY |  | 17 | -0.55 | -1.46 | 0.043 | 0.317 | 1.000 | 1431 | tags=41%, list=12%, signal=47% |
| 767 | GOBP\_LEUKOCYTE\_DIFFERENTIATION |  | 339 | -0.38 | -1.46 | 0.000 | 0.317 | 1.000 | 3395 | tags=42%, list=28%, signal=56% |
| 768 | GOBP\_T\_CELL\_DIFFERENTIATION\_IN\_THYMUS |  | 57 | -0.43 | -1.46 | 0.020 | 0.317 | 1.000 | 2031 | tags=33%, list=17%, signal=40% |
| 769 | GOBP\_EMBRYONIC\_HINDLIMB\_MORPHOGENESIS |  | 15 | -0.56 | -1.46 | 0.049 | 0.319 | 1.000 | 3890 | tags=60%, list=32%, signal=89% |
| 770 | GOBP\_REGULATION\_OF\_ALPHA\_BETA\_T\_CELL\_DIFFERENTIATION |  | 44 | -0.45 | -1.46 | 0.029 | 0.319 | 1.000 | 2408 | tags=43%, list=20%, signal=54% |
| 771 | GOBP\_CELLULAR\_RESPONSE\_TO\_CALCIUM\_ION |  | 53 | -0.44 | -1.46 | 0.023 | 0.322 | 1.000 | 2779 | tags=36%, list=23%, signal=46% |
| 772 | GOBP\_REGULATION\_OF\_TORC1\_SIGNALING |  | 28 | -0.49 | -1.46 | 0.040 | 0.323 | 1.000 | 3425 | tags=43%, list=29%, signal=60% |
| 773 | GOBP\_VESICLE\_CYTOSKELETAL\_TRAFFICKING |  | 59 | -0.43 | -1.46 | 0.022 | 0.323 | 1.000 | 3569 | tags=47%, list=30%, signal=67% |
| 774 | GOBP\_REGULATION\_OF\_SARCOMERE\_ORGANIZATION |  | 4 | -0.81 | -1.46 | 0.029 | 0.323 | 1.000 | 1840 | tags=75%, list=15%, signal=89% |
| 775 | GOBP\_POSITIVE\_REGULATION\_OF\_MACROAUTOPHAGY |  | 48 | -0.45 | -1.46 | 0.025 | 0.323 | 1.000 | 4948 | tags=58%, list=41%, signal=99% |
| 776 | GOBP\_THYMIC\_T\_CELL\_SELECTION |  | 18 | -0.54 | -1.46 | 0.049 | 0.323 | 1.000 | 2408 | tags=50%, list=20%, signal=62% |
| 777 | GOMF\_HISTONE\_KINASE\_ACTIVITY |  | 12 | -0.59 | -1.46 | 0.050 | 0.323 | 1.000 | 4528 | tags=67%, list=38%, signal=107% |
| 778 | GOMF\_FLAP\_ENDONUCLEASE\_ACTIVITY |  | 6 | -0.71 | -1.46 | 0.045 | 0.323 | 1.000 | 2733 | tags=67%, list=23%, signal=86% |
| 779 | GOBP\_AXIS\_ELONGATION |  | 21 | -0.52 | -1.46 | 0.046 | 0.323 | 1.000 | 3615 | tags=52%, list=30%, signal=75% |
| 780 | GOBP\_CELLULAR\_CARBOHYDRATE\_CATABOLIC\_PROCESS |  | 36 | -0.47 | -1.46 | 0.032 | 0.323 | 1.000 | 2814 | tags=39%, list=23%, signal=51% |
| 781 | GOBP\_LEUKOCYTE\_APOPTOTIC\_PROCESS |  | 71 | -0.42 | -1.46 | 0.015 | 0.323 | 1.000 | 1903 | tags=34%, list=16%, signal=40% |
| 782 | GOBP\_POSITIVE\_REGULATION\_OF\_FEAR\_RESPONSE |  | 5 | -0.76 | -1.46 | 0.035 | 0.323 | 1.000 | 2901 | tags=100%, list=24%, signal=132% |
| 783 | GOBP\_DENDRITE\_MORPHOGENESIS |  | 102 | -0.40 | -1.46 | 0.007 | 0.323 | 1.000 | 3833 | tags=45%, list=32%, signal=66% |
| 784 | GOCC\_HISTONE\_METHYLTRANSFERASE\_COMPLEX |  | 44 | -0.45 | -1.46 | 0.027 | 0.322 | 1.000 | 4512 | tags=52%, list=38%, signal=83% |
| 785 | GOBP\_NEURON\_PROJECTION\_EXTENSION\_INVOLVED\_IN\_NEURON\_PROJECTION\_GUIDANCE |  | 27 | -0.49 | -1.46 | 0.040 | 0.324 | 1.000 | 558 | tags=22%, list=5%, signal=23% |
| 786 | GOBP\_ANTERIOR\_COMMISSURE\_MORPHOGENESIS |  | 4 | -0.80 | -1.46 | 0.037 | 0.324 | 1.000 | 1464 | tags=50%, list=12%, signal=57% |
| 787 | GOCC\_ALPHA\_BETA\_T\_CELL\_RECEPTOR\_COMPLEX |  | 4 | -0.81 | -1.46 | 0.036 | 0.324 | 1.000 | 2249 | tags=100%, list=19%, signal=123% |
| 788 | GOBP\_REGULATION\_OF\_FEAR\_RESPONSE |  | 5 | -0.76 | -1.46 | 0.039 | 0.324 | 1.000 | 2901 | tags=100%, list=24%, signal=132% |
| 789 | GOMF\_PHOSPHORIC\_ESTER\_HYDROLASE\_ACTIVITY |  | 278 | -0.38 | -1.46 | 0.000 | 0.324 | 1.000 | 3692 | tags=41%, list=31%, signal=58% |
| 790 | GOBP\_FACE\_DEVELOPMENT |  | 31 | -0.48 | -1.46 | 0.036 | 0.324 | 1.000 | 4319 | tags=52%, list=36%, signal=80% |
| 791 | GOBP\_POSITIVE\_REGULATION\_OF\_ORGANELLE\_ASSEMBLY |  | 52 | -0.44 | -1.46 | 0.028 | 0.324 | 1.000 | 4427 | tags=50%, list=37%, signal=79% |
| 792 | GOCC\_CLUSTER\_OF\_ACTIN\_BASED\_CELL\_PROJECTIONS |  | 98 | -0.41 | -1.46 | 0.008 | 0.324 | 1.000 | 2753 | tags=38%, list=23%, signal=49% |
| 793 | GOMF\_CATALYTIC\_ACTIVITY\_ACTING\_ON\_DNA |  | 146 | -0.39 | -1.46 | 0.005 | 0.324 | 1.000 | 5296 | tags=60%, list=44%, signal=107% |
| 794 | GOMF\_LIPID\_TRANSPORTER\_ACTIVITY |  | 99 | -0.41 | -1.46 | 0.007 | 0.324 | 1.000 | 1745 | tags=27%, list=15%, signal=32% |
| 795 | GOBP\_POSITIVE\_REGULATION\_OF\_NUCLEOCYTOPLASMIC\_TRANSPORT |  | 48 | -0.44 | -1.46 | 0.026 | 0.324 | 1.000 | 3583 | tags=42%, list=30%, signal=59% |
| 796 | GOCC\_BASAL\_DENDRITE |  | 5 | -0.75 | -1.46 | 0.038 | 0.325 | 1.000 | 1308 | tags=60%, list=11%, signal=67% |
| 797 | GOMF\_NEUROTRANSMITTER\_BINDING |  | 9 | -0.64 | -1.46 | 0.046 | 0.326 | 1.000 | 2218 | tags=67%, list=18%, signal=82% |
| 798 | GOBP\_REGULATION\_OF\_CHLORIDE\_TRANSPORT |  | 7 | -0.69 | -1.46 | 0.046 | 0.326 | 1.000 | 2038 | tags=57%, list=17%, signal=69% |
| 799 | GOCC\_BASEMENT\_MEMBRANE |  | 70 | -0.42 | -1.46 | 0.020 | 0.327 | 1.000 | 3495 | tags=40%, list=29%, signal=56% |
| 800 | GOBP\_CARDIAC\_CELL\_DEVELOPMENT |  | 49 | -0.44 | -1.45 | 0.024 | 0.330 | 1.000 | 3530 | tags=43%, list=29%, signal=60% |
| 801 | GOBP\_CELL\_MIGRATION\_INVOLVED\_IN\_HEART\_DEVELOPMENT |  | 14 | -0.57 | -1.45 | 0.048 | 0.331 | 1.000 | 628 | tags=21%, list=5%, signal=23% |
| 802 | GOBP\_STRESS\_ACTIVATED\_PROTEIN\_KINASE\_SIGNALING\_CASCADE |  | 195 | -0.38 | -1.45 | 0.002 | 0.333 | 1.000 | 3655 | tags=44%, list=30%, signal=62% |
| 803 | GOBP\_SECOND\_MESSENGER\_MEDIATED\_SIGNALING |  | 196 | -0.38 | -1.45 | 0.002 | 0.333 | 1.000 | 2941 | tags=38%, list=24%, signal=50% |
| 804 | GOBP\_LIPID\_TRANSLOCATION |  | 34 | -0.47 | -1.45 | 0.037 | 0.333 | 1.000 | 1410 | tags=35%, list=12%, signal=40% |
| 805 | GOBP\_REGULATION\_OF\_VASCULAR\_ASSOCIATED\_SMOOTH\_MUSCLE\_CONTRACTION |  | 8 | -0.66 | -1.45 | 0.051 | 0.333 | 1.000 | 925 | tags=50%, list=8%, signal=54% |
| 806 | GOBP\_EMBRYONIC\_RETINA\_MORPHOGENESIS\_IN\_CAMERA\_TYPE\_EYE |  | 5 | -0.76 | -1.45 | 0.039 | 0.334 | 1.000 | 1205 | tags=60%, list=10%, signal=67% |
| 807 | GOBP\_CELLULAR\_RESPONSE\_TO\_STEROL |  | 15 | -0.56 | -1.45 | 0.058 | 0.335 | 1.000 | 3600 | tags=53%, list=30%, signal=76% |
| 808 | GOBP\_CORTICAL\_ACTIN\_CYTOSKELETON\_ORGANIZATION |  | 33 | -0.47 | -1.45 | 0.040 | 0.335 | 1.000 | 3674 | tags=48%, list=31%, signal=70% |
| 809 | GOBP\_POSITIVE\_REGULATION\_OF\_LOCOMOTION |  | 395 | -0.37 | -1.45 | 0.000 | 0.335 | 1.000 | 3724 | tags=40%, list=31%, signal=56% |
| 810 | GOBP\_REGULATION\_OF\_B\_CELL\_PROLIFERATION |  | 42 | -0.45 | -1.45 | 0.030 | 0.335 | 1.000 | 2474 | tags=40%, list=21%, signal=51% |
| 811 | GOBP\_VENTRICULAR\_SEPTUM\_DEVELOPMENT |  | 50 | -0.44 | -1.45 | 0.030 | 0.334 | 1.000 | 2308 | tags=30%, list=19%, signal=37% |
| 812 | GOBP\_REGULATION\_OF\_CELL\_KILLING |  | 37 | -0.46 | -1.45 | 0.036 | 0.334 | 1.000 | 2547 | tags=43%, list=21%, signal=55% |
| 813 | GOBP\_NATURAL\_KILLER\_CELL\_DIFFERENTIATION |  | 14 | -0.57 | -1.45 | 0.051 | 0.335 | 1.000 | 3009 | tags=57%, list=25%, signal=76% |
| 814 | GOBP\_REGULATION\_OF\_APPETITE |  | 12 | -0.58 | -1.45 | 0.051 | 0.334 | 1.000 | 3135 | tags=58%, list=26%, signal=79% |
| 815 | GOCC\_CELL\_CELL\_JUNCTION |  | 338 | -0.37 | -1.45 | 0.000 | 0.334 | 1.000 | 3089 | tags=33%, list=26%, signal=43% |
| 816 | GOCC\_MICROTUBULE\_END |  | 26 | -0.49 | -1.45 | 0.046 | 0.334 | 1.000 | 3837 | tags=50%, list=32%, signal=73% |
| 817 | GOBP\_CARNITINE\_BIOSYNTHETIC\_PROCESS |  | 4 | -0.80 | -1.45 | 0.034 | 0.334 | 1.000 | 2431 | tags=75%, list=20%, signal=94% |
| 818 | GOBP\_CHLORIDE\_TRANSPORT |  | 57 | -0.43 | -1.45 | 0.024 | 0.335 | 1.000 | 2159 | tags=40%, list=18%, signal=49% |
| 819 | GOBP\_DNA\_RECOMBINATION |  | 198 | -0.38 | -1.45 | 0.001 | 0.335 | 1.000 | 3701 | tags=44%, list=31%, signal=62% |
| 820 | GOBP\_RESPONSE\_TO\_FIBROBLAST\_GROWTH\_FACTOR |  | 96 | -0.40 | -1.45 | 0.009 | 0.335 | 1.000 | 2896 | tags=34%, list=24%, signal=45% |
| 821 | GOBP\_REGULATION\_OF\_KERATINOCYTE\_PROLIFERATION |  | 23 | -0.51 | -1.45 | 0.043 | 0.335 | 1.000 | 1777 | tags=39%, list=15%, signal=46% |
| 822 | GOCC\_NEURON\_TO\_NEURON\_SYNAPSE |  | 223 | -0.38 | -1.45 | 0.001 | 0.335 | 1.000 | 2604 | tags=30%, list=22%, signal=38% |
| 823 | GOCC\_H4\_HISTONE\_ACETYLTRANSFERASE\_COMPLEX |  | 39 | -0.46 | -1.45 | 0.038 | 0.337 | 1.000 | 3811 | tags=41%, list=32%, signal=60% |
| 824 | GOBP\_CELL\_JUNCTION\_ORGANIZATION |  | 475 | -0.37 | -1.45 | 0.000 | 0.336 | 1.000 | 3977 | tags=43%, list=33%, signal=61% |
| 825 | GOBP\_PERICARDIUM\_MORPHOGENESIS |  | 8 | -0.66 | -1.45 | 0.050 | 0.336 | 1.000 | 1239 | tags=38%, list=10%, signal=42% |
| 826 | GOCC\_H3\_HISTONE\_ACETYLTRANSFERASE\_COMPLEX |  | 10 | -0.62 | -1.45 | 0.051 | 0.337 | 1.000 | 3411 | tags=60%, list=28%, signal=84% |
| 827 | GOBP\_POSITIVE\_REGULATION\_OF\_FILOPODIUM\_ASSEMBLY |  | 24 | -0.50 | -1.45 | 0.043 | 0.337 | 1.000 | 3340 | tags=42%, list=28%, signal=58% |
| 828 | GOBP\_NEGATIVE\_REGULATION\_OF\_B\_CELL\_APOPTOTIC\_PROCESS |  | 10 | -0.62 | -1.45 | 0.056 | 0.338 | 1.000 | 1560 | tags=40%, list=13%, signal=46% |
| 829 | GOCC\_PROTEIN\_ACETYLTRANSFERASE\_COMPLEX |  | 80 | -0.41 | -1.45 | 0.013 | 0.338 | 1.000 | 3626 | tags=39%, list=30%, signal=55% |
| 830 | GOBP\_REGULATION\_OF\_HIPPO\_SIGNALING |  | 16 | -0.55 | -1.45 | 0.050 | 0.337 | 1.000 | 3699 | tags=56%, list=31%, signal=81% |
| 831 | GOBP\_MULTICELLULAR\_ORGANISM\_GROWTH |  | 94 | -0.40 | -1.45 | 0.015 | 0.337 | 1.000 | 2903 | tags=34%, list=24%, signal=45% |
| 832 | GOBP\_RESPONSE\_TO\_PARATHYROID\_HORMONE |  | 4 | -0.79 | -1.45 | 0.041 | 0.337 | 1.000 | 2474 | tags=100%, list=21%, signal=126% |
| 833 | GOBP\_BILE\_ACID\_METABOLIC\_PROCESS |  | 38 | -0.46 | -1.45 | 0.034 | 0.337 | 1.000 | 2993 | tags=37%, list=25%, signal=49% |
| 834 | GOBP\_AMINOPHOSPHOLIPID\_TRANSPORT |  | 8 | -0.66 | -1.45 | 0.051 | 0.337 | 1.000 | 990 | tags=50%, list=8%, signal=54% |
| 835 | GOBP\_RENAL\_SYSTEM\_PROCESS\_INVOLVED\_IN\_REGULATION\_OF\_SYSTEMIC\_ARTERIAL\_BLOOD\_PRESSURE |  | 15 | -0.56 | -1.45 | 0.053 | 0.337 | 1.000 | 2480 | tags=40%, list=21%, signal=50% |
| 836 | GOBP\_BIOLOGICAL\_ADHESION |  | 902 | -0.36 | -1.45 | 0.000 | 0.337 | 1.000 | 3205 | tags=34%, list=27%, signal=43% |
| 837 | GOBP\_POSITIVE\_REGULATION\_OF\_NITRIC\_OXIDE\_SYNTHASE\_BIOSYNTHETIC\_PROCESS |  | 8 | -0.65 | -1.45 | 0.054 | 0.338 | 1.000 | 3609 | tags=75%, list=30%, signal=107% |
| 838 | GOBP\_NEURON\_PROJECTION\_MAINTENANCE |  | 7 | -0.68 | -1.45 | 0.050 | 0.338 | 1.000 | 551 | tags=29%, list=5%, signal=30% |
| 839 | GOBP\_ESTABLISHMENT\_OF\_MITOTIC\_SPINDLE\_LOCALIZATION |  | 30 | -0.48 | -1.45 | 0.047 | 0.338 | 1.000 | 3834 | tags=50%, list=32%, signal=73% |
| 840 | GOBP\_CELLULAR\_RESPONSE\_TO\_LEUCINE\_STARVATION |  | 8 | -0.66 | -1.45 | 0.056 | 0.339 | 1.000 | 3574 | tags=63%, list=30%, signal=89% |
| 841 | GOBP\_PROTEIN\_K29\_LINKED\_DEUBIQUITINATION |  | 5 | -0.74 | -1.45 | 0.047 | 0.338 | 1.000 | 1740 | tags=60%, list=14%, signal=70% |
| 842 | GOBP\_RESPONSE\_TO\_UV\_A |  | 8 | -0.65 | -1.45 | 0.052 | 0.338 | 1.000 | 1580 | tags=50%, list=13%, signal=58% |
| 843 | GOBP\_NEUROTRANSMITTER\_TRANSPORT |  | 135 | -0.39 | -1.45 | 0.004 | 0.338 | 1.000 | 3603 | tags=45%, list=30%, signal=64% |
| 844 | GOCC\_TOR\_COMPLEX |  | 14 | -0.56 | -1.45 | 0.056 | 0.338 | 1.000 | 1743 | tags=43%, list=15%, signal=50% |
| 845 | GOBP\_ANTIGEN\_PROCESSING\_AND\_PRESENTATION\_OF\_PEPTIDE\_OR\_POLYSACCHARIDE\_ANTIGEN\_VIA\_MHC\_CLASS\_II |  | 68 | -0.42 | -1.44 | 0.023 | 0.338 | 1.000 | 4343 | tags=54%, list=36%, signal=85% |
| 846 | GOBP\_NEGATIVE\_REGULATION\_OF\_CELL\_CELL\_ADHESION |  | 111 | -0.40 | -1.44 | 0.009 | 0.339 | 1.000 | 3692 | tags=43%, list=31%, signal=62% |
| 847 | GOBP\_CHOLINE\_METABOLIC\_PROCESS |  | 6 | -0.71 | -1.44 | 0.043 | 0.339 | 1.000 | 2534 | tags=67%, list=21%, signal=84% |
| 848 | GOMF\_UBIQUITIN\_CONJUGATING\_ENZYME\_BINDING |  | 22 | -0.51 | -1.44 | 0.048 | 0.339 | 1.000 | 1748 | tags=32%, list=15%, signal=37% |
| 849 | GOBP\_SPROUTING\_ANGIOGENESIS |  | 90 | -0.41 | -1.44 | 0.015 | 0.339 | 1.000 | 2837 | tags=37%, list=24%, signal=48% |
| 850 | GOCC\_MICROTUBULE |  | 309 | -0.37 | -1.44 | 0.000 | 0.338 | 1.000 | 4443 | tags=45%, list=37%, signal=69% |
| 851 | GOBP\_RESPONSE\_TO\_VITAMIN\_D |  | 23 | -0.51 | -1.44 | 0.046 | 0.338 | 1.000 | 1579 | tags=35%, list=13%, signal=40% |
| 852 | GOMF\_NEUROTRANSMITTER\_TRANSMEMBRANE\_TRANSPORTER\_ACTIVITY |  | 7 | -0.68 | -1.44 | 0.051 | 0.338 | 1.000 | 2392 | tags=57%, list=20%, signal=71% |
| 853 | GOBP\_PRESYNAPSE\_ORGANIZATION |  | 28 | -0.48 | -1.44 | 0.047 | 0.337 | 1.000 | 5068 | tags=57%, list=42%, signal=99% |
| 854 | GOBP\_MODIFICATION\_OF\_POSTSYNAPTIC\_STRUCTURE |  | 12 | -0.59 | -1.44 | 0.059 | 0.337 | 1.000 | 198 | tags=25%, list=2%, signal=25% |
| 855 | GOMF\_CHROMATIN\_BINDING |  | 360 | -0.37 | -1.44 | 0.000 | 0.337 | 1.000 | 4660 | tags=51%, list=39%, signal=81% |
| 856 | GOBP\_POSITIVE\_REGULATION\_OF\_T\_CELL\_RECEPTOR\_SIGNALING\_PATHWAY |  | 10 | -0.61 | -1.44 | 0.055 | 0.337 | 1.000 | 2640 | tags=50%, list=22%, signal=64% |
| 857 | GOBP\_B\_CELL\_DIFFERENTIATION |  | 83 | -0.41 | -1.44 | 0.017 | 0.337 | 1.000 | 2357 | tags=35%, list=20%, signal=43% |
| 858 | GOBP\_BLOOD\_VESSEL\_REMODELING |  | 26 | -0.49 | -1.44 | 0.053 | 0.336 | 1.000 | 2474 | tags=42%, list=21%, signal=53% |
| 859 | GOBP\_CEREBELLAR\_GRANULAR\_LAYER\_DEVELOPMENT |  | 8 | -0.66 | -1.44 | 0.049 | 0.337 | 1.000 | 172 | tags=25%, list=1%, signal=25% |
| 860 | GOBP\_POSITIVE\_REGULATION\_OF\_MUSCLE\_CELL\_DIFFERENTIATION |  | 47 | -0.44 | -1.44 | 0.030 | 0.339 | 1.000 | 3658 | tags=45%, list=30%, signal=64% |
| 861 | GOBP\_CENTRAL\_NERVOUS\_SYSTEM\_PROJECTION\_NEURON\_AXONOGENESIS |  | 20 | -0.52 | -1.44 | 0.051 | 0.338 | 1.000 | 3425 | tags=40%, list=29%, signal=56% |
| 862 | GOBP\_GRANULOCYTE\_CHEMOTAXIS |  | 70 | -0.42 | -1.44 | 0.021 | 0.338 | 1.000 | 3212 | tags=44%, list=27%, signal=60% |
| 863 | GOBP\_GRANULOCYTE\_MIGRATION |  | 83 | -0.41 | -1.44 | 0.015 | 0.340 | 1.000 | 3212 | tags=46%, list=27%, signal=62% |
| 864 | GOBP\_POSITIVE\_REGULATION\_OF\_CELLULAR\_EXTRAVASATION |  | 12 | -0.59 | -1.44 | 0.059 | 0.340 | 1.000 | 2011 | tags=42%, list=17%, signal=50% |
| 865 | GOBP\_POSITIVE\_REGULATION\_OF\_PROTEIN\_KINASE\_B\_SIGNALING |  | 118 | -0.39 | -1.44 | 0.008 | 0.339 | 1.000 | 2879 | tags=37%, list=24%, signal=49% |
| 866 | GOBP\_SIGNAL\_RELEASE |  | 312 | -0.37 | -1.44 | 0.000 | 0.339 | 1.000 | 2367 | tags=31%, list=20%, signal=37% |
| 867 | GOBP\_LAMELLIPODIUM\_ORGANIZATION |  | 68 | -0.42 | -1.44 | 0.024 | 0.340 | 1.000 | 2784 | tags=32%, list=23%, signal=42% |
| 868 | GOBP\_NEGATIVE\_REGULATION\_OF\_DNA\_RECOMBINATION |  | 24 | -0.50 | -1.44 | 0.049 | 0.340 | 1.000 | 2379 | tags=46%, list=20%, signal=57% |
| 869 | GOBP\_NEGATIVE\_REGULATION\_OF\_CELL\_PROJECTION\_ORGANIZATION |  | 124 | -0.39 | -1.44 | 0.010 | 0.341 | 1.000 | 1464 | tags=22%, list=12%, signal=25% |
| 870 | GOBP\_REGULATION\_OF\_FILOPODIUM\_ASSEMBLY |  | 36 | -0.46 | -1.44 | 0.044 | 0.341 | 1.000 | 3473 | tags=42%, list=29%, signal=58% |
| 871 | GOBP\_MITOCHONDRION\_DISTRIBUTION |  | 16 | -0.54 | -1.44 | 0.058 | 0.342 | 1.000 | 4052 | tags=63%, list=34%, signal=94% |
| 872 | GOBP\_REGULATION\_OF\_MAPK\_CASCADE |  | 453 | -0.37 | -1.44 | 0.000 | 0.341 | 1.000 | 3728 | tags=40%, list=31%, signal=56% |
| 873 | GOBP\_CRANIAL\_NERVE\_MORPHOGENESIS |  | 12 | -0.59 | -1.44 | 0.063 | 0.341 | 1.000 | 1309 | tags=42%, list=11%, signal=47% |
| 874 | GOMF\_NEUTRAL\_AMINO\_ACID\_TRANSMEMBRANE\_TRANSPORTER\_ACTIVITY |  | 23 | -0.50 | -1.44 | 0.050 | 0.342 | 1.000 | 3155 | tags=52%, list=26%, signal=71% |
| 875 | GOBP\_POSITIVE\_REGULATION\_OF\_VASOCONSTRICTION |  | 20 | -0.52 | -1.44 | 0.054 | 0.342 | 1.000 | 1269 | tags=35%, list=11%, signal=39% |
| 876 | GOBP\_RESPONSE\_TO\_PLATELET\_DERIVED\_GROWTH\_FACTOR |  | 14 | -0.56 | -1.44 | 0.062 | 0.342 | 1.000 | 3286 | tags=64%, list=27%, signal=88% |
| 877 | GOBP\_OUTFLOW\_TRACT\_MORPHOGENESIS |  | 53 | -0.43 | -1.44 | 0.027 | 0.342 | 1.000 | 3314 | tags=36%, list=28%, signal=49% |
| 878 | GOBP\_REGULATION\_OF\_CELL\_SUBSTRATE\_ADHESION |  | 159 | -0.38 | -1.44 | 0.004 | 0.343 | 1.000 | 3196 | tags=36%, list=27%, signal=48% |
| 879 | GOMF\_UBIQUITIN\_LIKE\_PROTEIN\_LIGASE\_ACTIVITY |  | 185 | -0.38 | -1.44 | 0.002 | 0.343 | 1.000 | 4641 | tags=49%, list=39%, signal=79% |
| 880 | GOMF\_O\_PALMITOYLTRANSFERASE\_ACTIVITY |  | 3 | -0.86 | -1.44 | 0.030 | 0.343 | 1.000 | 1369 | tags=67%, list=11%, signal=75% |
| 881 | GOBP\_REGULATION\_OF\_SUBSTRATE\_ADHESION\_DEPENDENT\_CELL\_SPREADING |  | 46 | -0.44 | -1.44 | 0.034 | 0.344 | 1.000 | 1777 | tags=28%, list=15%, signal=33% |
| 882 | GOBP\_LOCOMOTORY\_EXPLORATION\_BEHAVIOR |  | 6 | -0.71 | -1.44 | 0.055 | 0.344 | 1.000 | 3483 | tags=100%, list=29%, signal=141% |
| 883 | GOBP\_REGULATION\_OF\_CELL\_CYCLE\_CHECKPOINT |  | 27 | -0.49 | -1.44 | 0.044 | 0.344 | 1.000 | 3958 | tags=52%, list=33%, signal=77% |
| 884 | GOBP\_REGULATION\_OF\_SYNAPTIC\_TRANSMISSION\_GLUTAMATERGIC |  | 36 | -0.46 | -1.44 | 0.039 | 0.344 | 1.000 | 3699 | tags=50%, list=31%, signal=72% |
| 885 | GOBP\_ACYLGLYCEROL\_HOMEOSTASIS |  | 19 | -0.52 | -1.44 | 0.057 | 0.344 | 1.000 | 3205 | tags=47%, list=27%, signal=65% |
| 886 | GOBP\_ARTERY\_MORPHOGENESIS |  | 45 | -0.44 | -1.44 | 0.031 | 0.345 | 1.000 | 3348 | tags=44%, list=28%, signal=61% |
| 887 | GOBP\_REGULATION\_OF\_CALCIUM\_ION\_IMPORT |  | 17 | -0.54 | -1.44 | 0.065 | 0.344 | 1.000 | 3286 | tags=53%, list=27%, signal=73% |
| 888 | GOMF\_PHOSPHATIDYLINOSITOL\_3\_5\_BISPHOSPHATE\_PHOSPHATASE\_ACTIVITY |  | 9 | -0.63 | -1.44 | 0.057 | 0.344 | 1.000 | 3244 | tags=67%, list=27%, signal=91% |
| 889 | GOBP\_RESPONSE\_TO\_TUMOR\_CELL |  | 10 | -0.61 | -1.44 | 0.059 | 0.344 | 1.000 | 3730 | tags=70%, list=31%, signal=101% |
| 890 | GOMF\_DNA\_DEPENDENT\_ATPASE\_ACTIVITY |  | 43 | -0.45 | -1.44 | 0.033 | 0.344 | 1.000 | 5049 | tags=65%, list=42%, signal=112% |
| 891 | GOCC\_ASTROCYTE\_PROJECTION |  | 10 | -0.62 | -1.44 | 0.059 | 0.344 | 1.000 | 3004 | tags=50%, list=25%, signal=67% |
| 892 | GOMF\_PATTERN\_RECOGNITION\_RECEPTOR\_ACTIVITY |  | 12 | -0.58 | -1.44 | 0.055 | 0.344 | 1.000 | 1236 | tags=33%, list=10%, signal=37% |
| 893 | GOBP\_POSITIVE\_REGULATION\_OF\_CELL\_SUBSTRATE\_ADHESION |  | 92 | -0.40 | -1.44 | 0.018 | 0.344 | 1.000 | 3196 | tags=35%, list=27%, signal=47% |
| 894 | GOBP\_LIVER\_REGENERATION |  | 22 | -0.51 | -1.44 | 0.058 | 0.344 | 1.000 | 1580 | tags=32%, list=13%, signal=37% |
| 895 | GOBP\_REGULATION\_OF\_PROTEIN\_PHOSPHORYLATION |  | 805 | -0.36 | -1.44 | 0.000 | 0.344 | 1.000 | 4148 | tags=42%, list=35%, signal=60% |
| 896 | GOMF\_5\_3\_DNA\_HELICASE\_ACTIVITY |  | 5 | -0.74 | -1.44 | 0.048 | 0.344 | 1.000 | 2616 | tags=60%, list=22%, signal=77% |
| 897 | GOBP\_ADENYLATE\_CYCLASE\_MODULATING\_G\_PROTEIN\_COUPLED\_RECEPTOR\_SIGNALING\_PATHWAY |  | 122 | -0.39 | -1.44 | 0.008 | 0.344 | 1.000 | 3788 | tags=43%, list=32%, signal=63% |
| 898 | GOBP\_DESMOSOME\_ORGANIZATION |  | 8 | -0.65 | -1.44 | 0.055 | 0.344 | 1.000 | 3918 | tags=75%, list=33%, signal=111% |
| 899 | GOBP\_REGULATION\_OF\_PROTEIN\_MATURATION |  | 37 | -0.46 | -1.44 | 0.043 | 0.345 | 1.000 | 3964 | tags=54%, list=33%, signal=80% |
| 900 | GOBP\_NERVE\_DEVELOPMENT |  | 41 | -0.45 | -1.44 | 0.038 | 0.346 | 1.000 | 2903 | tags=34%, list=24%, signal=45% |
| 901 | GOBP\_REGULATION\_OF\_CHROMOSOME\_ORGANIZATION |  | 209 | -0.38 | -1.43 | 0.002 | 0.347 | 1.000 | 4465 | tags=48%, list=37%, signal=75% |
| 902 | GOMF\_DOLICHYL\_PHOSPHATE\_MANNOSE\_PROTEIN\_MANNOSYLTRANSFERASE\_ACTIVITY |  | 8 | -0.65 | -1.43 | 0.057 | 0.346 | 1.000 | 912 | tags=38%, list=8%, signal=41% |
| 903 | GOBP\_CONVERGENT\_EXTENSION |  | 11 | -0.60 | -1.43 | 0.059 | 0.346 | 1.000 | 1579 | tags=27%, list=13%, signal=31% |
| 904 | GOMF\_CARNITINE\_O\_ACYLTRANSFERASE\_ACTIVITY |  | 4 | -0.79 | -1.43 | 0.042 | 0.346 | 1.000 | 598 | tags=25%, list=5%, signal=26% |
| 905 | GOBP\_POSITIVE\_REGULATION\_OF\_MHC\_CLASS\_I\_BIOSYNTHETIC\_PROCESS |  | 3 | -0.85 | -1.43 | 0.031 | 0.346 | 1.000 | 1763 | tags=100%, list=15%, signal=117% |
| 906 | GOCC\_HIPPOCAMPAL\_MOSSY\_FIBER\_TO\_CA3\_SYNAPSE |  | 21 | -0.51 | -1.43 | 0.054 | 0.346 | 1.000 | 2399 | tags=38%, list=20%, signal=48% |
| 907 | GOBP\_CHOLESTEROL\_EFFLUX |  | 33 | -0.47 | -1.43 | 0.044 | 0.347 | 1.000 | 3565 | tags=45%, list=30%, signal=64% |
| 908 | GOBP\_COLLAGEN\_ACTIVATED\_TYROSINE\_KINASE\_RECEPTOR\_SIGNALING\_PATHWAY |  | 8 | -0.65 | -1.43 | 0.056 | 0.347 | 1.000 | 2357 | tags=50%, list=20%, signal=62% |
| 909 | GOBP\_CHROMOSOME\_ORGANIZATION\_INVOLVED\_IN\_MEIOTIC\_CELL\_CYCLE |  | 37 | -0.46 | -1.43 | 0.039 | 0.348 | 1.000 | 5365 | tags=70%, list=45%, signal=127% |
| 910 | GOCC\_RISC\_LOADING\_COMPLEX |  | 4 | -0.79 | -1.43 | 0.043 | 0.348 | 1.000 | 1503 | tags=75%, list=13%, signal=86% |
| 911 | GOMF\_PEPTIDE\_HORMONE\_BINDING |  | 31 | -0.47 | -1.43 | 0.046 | 0.348 | 1.000 | 1264 | tags=29%, list=11%, signal=32% |
| 912 | GOCC\_NEUROMUSCULAR\_JUNCTION |  | 49 | -0.43 | -1.43 | 0.031 | 0.348 | 1.000 | 2876 | tags=35%, list=24%, signal=45% |
| 913 | GOMF\_HYDROLASE\_ACTIVITY\_HYDROLYZING\_O\_GLYCOSYL\_COMPOUNDS |  | 59 | -0.42 | -1.43 | 0.033 | 0.348 | 1.000 | 3409 | tags=49%, list=28%, signal=68% |
| 914 | GOBP\_GLOMERULAR\_BASEMENT\_MEMBRANE\_DEVELOPMENT |  | 8 | -0.65 | -1.43 | 0.059 | 0.347 | 1.000 | 1615 | tags=38%, list=13%, signal=43% |
| 915 | GOMF\_KINASE\_REGULATOR\_ACTIVITY |  | 161 | -0.38 | -1.43 | 0.004 | 0.347 | 1.000 | 3408 | tags=37%, list=28%, signal=51% |
| 916 | GOBP\_RENAL\_SYSTEM\_PROCESS\_INVOLVED\_IN\_REGULATION\_OF\_BLOOD\_VOLUME |  | 9 | -0.63 | -1.43 | 0.058 | 0.347 | 1.000 | 2480 | tags=44%, list=21%, signal=56% |
| 917 | GOBP\_REGULATION\_OF\_MULTICELLULAR\_ORGANISMAL\_DEVELOPMENT |  | 859 | -0.36 | -1.43 | 0.000 | 0.348 | 1.000 | 3657 | tags=39%, list=30%, signal=52% |
| 918 | GOBP\_REGULATION\_OF\_GLOMERULAR\_FILTRATION |  | 9 | -0.63 | -1.43 | 0.063 | 0.347 | 1.000 | 2480 | tags=44%, list=21%, signal=56% |
| 919 | GOBP\_POSITIVE\_REGULATION\_OF\_TRANSLATIONAL\_INITIATION |  | 21 | -0.51 | -1.43 | 0.049 | 0.348 | 1.000 | 4407 | tags=67%, list=37%, signal=105% |
| 920 | GOBP\_TRACHEA\_DEVELOPMENT |  | 13 | -0.57 | -1.43 | 0.069 | 0.349 | 1.000 | 1692 | tags=31%, list=14%, signal=36% |
| 921 | GOBP\_MICROTUBULE\_BASED\_PROCESS |  | 577 | -0.36 | -1.43 | 0.000 | 0.349 | 1.000 | 3668 | tags=38%, list=31%, signal=52% |
| 922 | GOCC\_NEURONAL\_DENSE\_CORE\_VESICLE\_MEMBRANE |  | 4 | -0.79 | -1.43 | 0.041 | 0.349 | 1.000 | 1298 | tags=75%, list=11%, signal=84% |
| 923 | GOBP\_T\_CELL\_RECEPTOR\_SIGNALING\_PATHWAY |  | 135 | -0.39 | -1.43 | 0.006 | 0.349 | 1.000 | 3069 | tags=36%, list=26%, signal=47% |
| 924 | GOMF\_SINGLE\_STRANDED\_DNA\_HELICASE\_ACTIVITY |  | 17 | -0.53 | -1.43 | 0.061 | 0.351 | 1.000 | 3292 | tags=53%, list=27%, signal=73% |
| 925 | GOBP\_REGULATION\_OF\_BILE\_ACID\_BIOSYNTHETIC\_PROCESS |  | 8 | -0.65 | -1.43 | 0.055 | 0.350 | 1.000 | 2656 | tags=63%, list=22%, signal=80% |
| 926 | GOMF\_LYSOPHOSPHATIDIC\_ACID\_RECEPTOR\_ACTIVITY |  | 5 | -0.74 | -1.43 | 0.047 | 0.351 | 1.000 | 92 | tags=20%, list=1%, signal=20% |
| 927 | GOBP\_CELL\_FATE\_COMMITMENT |  | 132 | -0.39 | -1.43 | 0.007 | 0.351 | 1.000 | 2560 | tags=32%, list=21%, signal=40% |
| 928 | GOBP\_MYELOID\_LEUKOCYTE\_MIGRATION |  | 130 | -0.39 | -1.43 | 0.007 | 0.351 | 1.000 | 3212 | tags=42%, list=27%, signal=56% |
| 929 | GOBP\_POSITIVE\_REGULATION\_OF\_SYNAPTIC\_TRANSMISSION\_GLUTAMATERGIC |  | 16 | -0.54 | -1.43 | 0.055 | 0.351 | 1.000 | 3699 | tags=69%, list=31%, signal=99% |
| 930 | GOMF\_3\_5\_DNA\_HELICASE\_ACTIVITY |  | 13 | -0.57 | -1.43 | 0.065 | 0.351 | 1.000 | 5159 | tags=100%, list=43%, signal=175% |
| 931 | GOCC\_LAMININ\_COMPLEX |  | 11 | -0.60 | -1.43 | 0.057 | 0.352 | 1.000 | 908 | tags=36%, list=8%, signal=39% |
| 932 | GOBP\_POSITIVE\_REGULATION\_OF\_NMDA\_GLUTAMATE\_RECEPTOR\_ACTIVITY |  | 3 | -0.86 | -1.43 | 0.029 | 0.352 | 1.000 | 1659 | tags=100%, list=14%, signal=116% |
| 933 | GOBP\_REGULATION\_OF\_ACTOMYOSIN\_STRUCTURE\_ORGANIZATION |  | 75 | -0.41 | -1.43 | 0.022 | 0.352 | 1.000 | 2588 | tags=37%, list=22%, signal=47% |
| 934 | GOBP\_GLYCEROLIPID\_BIOSYNTHETIC\_PROCESS |  | 203 | -0.38 | -1.43 | 0.002 | 0.352 | 1.000 | 4057 | tags=43%, list=34%, signal=64% |
| 935 | GOBP\_POSITIVE\_REGULATION\_OF\_ATP\_METABOLIC\_PROCESS |  | 26 | -0.48 | -1.43 | 0.049 | 0.352 | 1.000 | 685 | tags=23%, list=6%, signal=24% |
| 936 | GOBP\_CEREBELLAR\_PURKINJE\_CELL\_LAYER\_MORPHOGENESIS |  | 8 | -0.65 | -1.43 | 0.052 | 0.352 | 1.000 | 2528 | tags=50%, list=21%, signal=63% |
| 937 | GOMF\_OXIDOREDUCTASE\_ACTIVITY\_ACTING\_ON\_SINGLE\_DONORS\_WITH\_INCORPORATION\_OF\_MOLECULAR\_OXYGEN |  | 12 | -0.58 | -1.43 | 0.066 | 0.352 | 1.000 | 267 | tags=25%, list=2%, signal=26% |
| 938 | GOBP\_HISTONE\_DEPHOSPHORYLATION |  | 3 | -0.86 | -1.43 | 0.035 | 0.352 | 1.000 | 951 | tags=67%, list=8%, signal=72% |
| 939 | GOBP\_REGULATION\_OF\_WOUND\_HEALING\_SPREADING\_OF\_EPIDERMAL\_CELLS |  | 8 | -0.65 | -1.43 | 0.066 | 0.353 | 1.000 | 3551 | tags=75%, list=30%, signal=106% |
| 940 | GOBP\_NEGATIVE\_REGULATION\_OF\_B\_CELL\_PROLIFERATION |  | 11 | -0.59 | -1.43 | 0.067 | 0.353 | 1.000 | 1951 | tags=45%, list=16%, signal=54% |
| 941 | GOBP\_RESPONSE\_TO\_EPIDERMAL\_GROWTH\_FACTOR |  | 34 | -0.46 | -1.43 | 0.052 | 0.353 | 1.000 | 2494 | tags=38%, list=21%, signal=48% |
| 942 | GOBP\_DOPAMINE\_BIOSYNTHETIC\_PROCESS |  | 8 | -0.65 | -1.43 | 0.059 | 0.353 | 1.000 | 2516 | tags=50%, list=21%, signal=63% |
| 943 | GOMF\_SNRNP\_BINDING |  | 3 | -0.85 | -1.43 | 0.035 | 0.353 | 1.000 | 1068 | tags=33%, list=9%, signal=37% |
| 944 | GOBP\_FORMATION\_OF\_PRIMARY\_GERM\_LAYER |  | 76 | -0.41 | -1.43 | 0.023 | 0.352 | 1.000 | 3306 | tags=37%, list=28%, signal=51% |
| 945 | GOCC\_MICROTUBULE\_ASSOCIATED\_COMPLEX |  | 100 | -0.40 | -1.43 | 0.014 | 0.353 | 1.000 | 3555 | tags=41%, list=30%, signal=58% |
| 946 | GOBP\_REGULATION\_OF\_NMDA\_RECEPTOR\_ACTIVITY |  | 17 | -0.53 | -1.43 | 0.063 | 0.354 | 1.000 | 2604 | tags=47%, list=22%, signal=60% |
| 947 | GOCC\_PROXIMAL\_DENDRITE |  | 5 | -0.74 | -1.43 | 0.053 | 0.354 | 1.000 | 1222 | tags=60%, list=10%, signal=67% |
| 948 | GOBP\_ALPHA\_BETA\_T\_CELL\_DIFFERENTIATION |  | 70 | -0.41 | -1.43 | 0.026 | 0.354 | 1.000 | 2408 | tags=40%, list=20%, signal=50% |
| 949 | GOBP\_CELL\_SUBSTRATE\_JUNCTION\_ORGANIZATION |  | 88 | -0.40 | -1.43 | 0.017 | 0.354 | 1.000 | 3905 | tags=50%, list=33%, signal=74% |
| 950 | GOBP\_AMINO\_ACID\_BETAINE\_TRANSPORT |  | 5 | -0.74 | -1.43 | 0.049 | 0.355 | 1.000 | 2557 | tags=80%, list=21%, signal=102% |
| 951 | GOBP\_GLUTAMATE\_SECRETION |  | 29 | -0.48 | -1.43 | 0.050 | 0.354 | 1.000 | 2313 | tags=48%, list=19%, signal=60% |
| 952 | GOBP\_CELLULAR\_RESPONSE\_TO\_ESTRADIOL\_STIMULUS |  | 23 | -0.50 | -1.43 | 0.055 | 0.355 | 1.000 | 2829 | tags=48%, list=24%, signal=62% |
| 953 | GOBP\_POSITIVE\_REGULATION\_OF\_ERYTHROCYTE\_DIFFERENTIATION |  | 23 | -0.50 | -1.43 | 0.055 | 0.355 | 1.000 | 3657 | tags=57%, list=30%, signal=81% |
| 954 | GOBP\_DEADENYLATION\_DEPENDENT\_DECAPPING\_OF\_NUCLEAR\_TRANSCRIBED\_MRNA |  | 10 | -0.61 | -1.43 | 0.061 | 0.355 | 1.000 | 1691 | tags=30%, list=14%, signal=35% |
| 955 | GOBP\_REGULATION\_OF\_STORE\_OPERATED\_CALCIUM\_CHANNEL\_ACTIVITY |  | 4 | -0.79 | -1.42 | 0.049 | 0.356 | 1.000 | 1373 | tags=50%, list=11%, signal=56% |
| 956 | GOCC\_ENDOCYTIC\_VESICLE\_LUMEN |  | 10 | -0.61 | -1.42 | 0.061 | 0.356 | 1.000 | 3255 | tags=60%, list=27%, signal=82% |
| 957 | GOBP\_NEURON\_MATURATION |  | 32 | -0.47 | -1.42 | 0.051 | 0.356 | 1.000 | 3836 | tags=47%, list=32%, signal=69% |
| 958 | GOBP\_REGULATION\_OF\_CELL\_SIZE |  | 122 | -0.39 | -1.42 | 0.012 | 0.356 | 1.000 | 3235 | tags=36%, list=27%, signal=49% |
| 959 | GOBP\_B\_CELL\_RECEPTOR\_SIGNALING\_PATHWAY |  | 40 | -0.45 | -1.42 | 0.041 | 0.356 | 1.000 | 2474 | tags=43%, list=21%, signal=53% |
| 960 | GOBP\_REGULATION\_OF\_CELLULAR\_RESPONSE\_TO\_INSULIN\_STIMULUS |  | 48 | -0.43 | -1.42 | 0.037 | 0.357 | 1.000 | 3692 | tags=46%, list=31%, signal=66% |
| 961 | GOBP\_POSITIVE\_REGULATION\_OF\_VASCULAR\_ENDOTHELIAL\_GROWTH\_FACTOR\_RECEPTOR\_SIGNALING\_PATHWAY |  | 9 | -0.63 | -1.42 | 0.064 | 0.357 | 1.000 | 2145 | tags=44%, list=18%, signal=54% |
| 962 | GOBP\_REGULATION\_OF\_POSITIVE\_CHEMOTAXIS |  | 21 | -0.51 | -1.42 | 0.065 | 0.357 | 1.000 | 3196 | tags=52%, list=27%, signal=71% |
| 963 | GOBP\_POSITIVE\_REGULATION\_OF\_SISTER\_CHROMATID\_COHESION |  | 8 | -0.65 | -1.42 | 0.064 | 0.357 | 1.000 | 2855 | tags=63%, list=24%, signal=82% |
| 964 | GOMF\_NUCLEAR\_EXPORT\_SIGNAL\_RECEPTOR\_ACTIVITY |  | 10 | -0.60 | -1.42 | 0.060 | 0.357 | 1.000 | 4706 | tags=90%, list=39%, signal=148% |
| 965 | GOBP\_ERK1\_AND\_ERK2\_CASCADE |  | 190 | -0.38 | -1.42 | 0.002 | 0.357 | 1.000 | 3724 | tags=42%, list=31%, signal=59% |
| 966 | GOBP\_POSITIVE\_REGULATION\_OF\_TRANSFERASE\_ACTIVITY |  | 500 | -0.36 | -1.42 | 0.000 | 0.357 | 1.000 | 2965 | tags=31%, list=25%, signal=39% |
| 967 | GOBP\_REGULATION\_OF\_POSTSYNAPTIC\_MEMBRANE\_POTENTIAL |  | 58 | -0.42 | -1.42 | 0.029 | 0.357 | 1.000 | 3708 | tags=43%, list=31%, signal=62% |
| 968 | GOBP\_SINOATRIAL\_NODE\_DEVELOPMENT |  | 3 | -0.85 | -1.42 | 0.041 | 0.358 | 1.000 | 226 | tags=33%, list=2%, signal=34% |
| 969 | GOBP\_NEGATIVE\_REGULATION\_OF\_ADAPTIVE\_IMMUNE\_RESPONSE |  | 23 | -0.50 | -1.42 | 0.058 | 0.359 | 1.000 | 3968 | tags=65%, list=33%, signal=97% |
| 970 | GOBP\_CARTILAGE\_DEVELOPMENT |  | 118 | -0.39 | -1.42 | 0.014 | 0.359 | 1.000 | 2002 | tags=28%, list=17%, signal=33% |
| 971 | GOBP\_NEUTROPHIL\_CHEMOTAXIS |  | 58 | -0.42 | -1.42 | 0.034 | 0.359 | 1.000 | 2968 | tags=41%, list=25%, signal=55% |
| 972 | GOCC\_CILIARY\_ROOTLET |  | 8 | -0.64 | -1.42 | 0.066 | 0.358 | 1.000 | 3555 | tags=63%, list=30%, signal=89% |
| 973 | GOMF\_VERY\_LONG\_CHAIN\_FATTY\_ACID\_COA\_LIGASE\_ACTIVITY |  | 7 | -0.67 | -1.42 | 0.063 | 0.358 | 1.000 | 1412 | tags=43%, list=12%, signal=49% |
| 974 | GOBP\_REGULATION\_OF\_T\_CELL\_MEDIATED\_CYTOTOXICITY |  | 11 | -0.59 | -1.42 | 0.071 | 0.358 | 1.000 | 1519 | tags=45%, list=13%, signal=52% |
| 975 | GOBP\_REGULATION\_OF\_CHROMATIN\_ORGANIZATION |  | 128 | -0.39 | -1.42 | 0.011 | 0.358 | 1.000 | 3644 | tags=45%, list=30%, signal=63% |
| 976 | GOBP\_REGULATION\_OF\_NUCLEAR\_TRANSCRIBED\_MRNA\_POLY\_A\_TAIL\_SHORTENING |  | 9 | -0.62 | -1.42 | 0.063 | 0.358 | 1.000 | 2441 | tags=67%, list=20%, signal=84% |
| 977 | GOBP\_POSITIVE\_REGULATION\_OF\_MACROPHAGE\_CYTOKINE\_PRODUCTION |  | 7 | -0.67 | -1.42 | 0.060 | 0.358 | 1.000 | 2654 | tags=71%, list=22%, signal=92% |
| 978 | GOBP\_NATURAL\_KILLER\_CELL\_CHEMOTAXIS |  | 6 | -0.70 | -1.42 | 0.058 | 0.358 | 1.000 | 1305 | tags=67%, list=11%, signal=75% |
| 979 | GOBP\_POSITIVE\_REGULATION\_OF\_INTRACELLULAR\_SIGNAL\_TRANSDUCTION |  | 654 | -0.36 | -1.42 | 0.000 | 0.358 | 1.000 | 3286 | tags=37%, list=27%, signal=48% |
| 980 | GOBP\_PROTEIN\_DEGLYCOSYLATION |  | 22 | -0.50 | -1.42 | 0.061 | 0.358 | 1.000 | 2715 | tags=36%, list=23%, signal=47% |
| 981 | GOBP\_NEURAL\_CREST\_CELL\_DIFFERENTIATION |  | 68 | -0.41 | -1.42 | 0.025 | 0.358 | 1.000 | 2560 | tags=29%, list=21%, signal=37% |
| 982 | GOBP\_PROTEIN\_LOCALIZATION\_TO\_CELL\_JUNCTION |  | 75 | -0.41 | -1.42 | 0.024 | 0.358 | 1.000 | 4655 | tags=57%, list=39%, signal=93% |
| 983 | GOBP\_SINOATRIAL\_NODE\_CELL\_DEVELOPMENT |  | 3 | -0.85 | -1.42 | 0.042 | 0.359 | 1.000 | 226 | tags=33%, list=2%, signal=34% |
| 984 | GOBP\_TOLL\_SIGNALING\_PATHWAY |  | 4 | -0.78 | -1.42 | 0.051 | 0.358 | 1.000 | 2606 | tags=100%, list=22%, signal=128% |
| 985 | GOBP\_MICROTUBULE\_CYTOSKELETON\_ORGANIZATION |  | 418 | -0.36 | -1.42 | 0.000 | 0.358 | 1.000 | 3892 | tags=40%, list=32%, signal=58% |
| 986 | GOBP\_REGULATION\_OF\_T\_CELL\_MIGRATION |  | 29 | -0.47 | -1.42 | 0.051 | 0.358 | 1.000 | 2011 | tags=38%, list=17%, signal=45% |
| 987 | GOBP\_POSITIVE\_REGULATION\_OF\_NERVOUS\_SYSTEM\_PROCESS |  | 23 | -0.50 | -1.42 | 0.059 | 0.358 | 1.000 | 3289 | tags=48%, list=27%, signal=66% |
| 988 | GOBP\_REGULATION\_OF\_MICROTUBULE\_CYTOSKELETON\_ORGANIZATION |  | 102 | -0.39 | -1.42 | 0.015 | 0.359 | 1.000 | 3575 | tags=42%, list=30%, signal=60% |
| 989 | GOBP\_POSITIVE\_REGULATION\_OF\_NATURAL\_KILLER\_CELL\_CHEMOTAXIS |  | 4 | -0.78 | -1.42 | 0.046 | 0.359 | 1.000 | 1038 | tags=75%, list=9%, signal=82% |
| 990 | GOBP\_CELL\_CELL\_JUNCTION\_MAINTENANCE |  | 6 | -0.70 | -1.42 | 0.057 | 0.359 | 1.000 | 3093 | tags=83%, list=26%, signal=112% |
| 991 | GOMF\_NEUROTRANSMITTER\_RECEPTOR\_ACTIVITY |  | 30 | -0.47 | -1.42 | 0.056 | 0.359 | 1.000 | 1285 | tags=30%, list=11%, signal=34% |
| 992 | GOBP\_HEART\_GROWTH |  | 55 | -0.42 | -1.42 | 0.032 | 0.359 | 1.000 | 3510 | tags=44%, list=29%, signal=61% |
| 993 | GOBP\_LIVER\_MORPHOGENESIS |  | 17 | -0.53 | -1.42 | 0.069 | 0.359 | 1.000 | 3937 | tags=53%, list=33%, signal=79% |
| 994 | GOCC\_CENTRIOLAR\_SUBDISTAL\_APPENDAGE |  | 8 | -0.64 | -1.42 | 0.069 | 0.359 | 1.000 | 3281 | tags=75%, list=27%, signal=103% |
| 995 | GOBP\_MODIFICATION\_OF\_SYNAPTIC\_STRUCTURE |  | 15 | -0.55 | -1.42 | 0.064 | 0.359 | 1.000 | 198 | tags=20%, list=2%, signal=20% |
| 996 | GOBP\_REGULATION\_OF\_NATURAL\_KILLER\_CELL\_CHEMOTAXIS |  | 4 | -0.78 | -1.42 | 0.047 | 0.359 | 1.000 | 1038 | tags=75%, list=9%, signal=82% |
| 997 | GOMF\_HISTONE\_ACETYLTRANSFERASE\_BINDING |  | 23 | -0.50 | -1.42 | 0.056 | 0.359 | 1.000 | 2986 | tags=43%, list=25%, signal=58% |
| 998 | GOBP\_REGULATION\_OF\_NIK\_NF\_KAPPAB\_SIGNALING |  | 67 | -0.41 | -1.42 | 0.026 | 0.359 | 1.000 | 2261 | tags=31%, list=19%, signal=38% |
| 999 | GOMF\_G\_PROTEIN\_COUPLED\_RECEPTOR\_BINDING |  | 139 | -0.38 | -1.42 | 0.009 | 0.360 | 1.000 | 4277 | tags=48%, list=36%, signal=74% |
| 1000 | GOBP\_CHROMOSOME\_ORGANIZATION |  | 847 | -0.36 | -1.42 | 0.000 | 0.359 | 1.000 | 4698 | tags=49%, list=39%, signal=74% |
| 1001 | GOBP\_LONG\_TERM\_SYNAPTIC\_POTENTIATION |  | 48 | -0.43 | -1.42 | 0.042 | 0.359 | 1.000 | 3699 | tags=48%, list=31%, signal=69% |
| 1002 | GOBP\_ACTIVATION\_INDUCED\_CELL\_DEATH\_OF\_T\_CELLS |  | 8 | -0.64 | -1.42 | 0.064 | 0.360 | 1.000 | 2362 | tags=63%, list=20%, signal=78% |
| 1003 | GOBP\_NEGATIVE\_REGULATION\_OF\_NERVOUS\_SYSTEM\_DEVELOPMENT |  | 90 | -0.40 | -1.42 | 0.020 | 0.360 | 1.000 | 2631 | tags=30%, list=22%, signal=38% |
| 1004 | GOBP\_AORTA\_MORPHOGENESIS |  | 23 | -0.50 | -1.42 | 0.059 | 0.359 | 1.000 | 3314 | tags=43%, list=28%, signal=60% |
| 1005 | GOBP\_REGULATION\_OF\_SYNAPSE\_ASSEMBLY |  | 56 | -0.42 | -1.42 | 0.032 | 0.359 | 1.000 | 3125 | tags=38%, list=26%, signal=50% |
| 1006 | GOBP\_NEGATIVE\_REGULATION\_OF\_CHONDROCYTE\_PROLIFERATION |  | 3 | -0.85 | -1.42 | 0.039 | 0.359 | 1.000 | 1771 | tags=100%, list=15%, signal=117% |
| 1007 | GOBP\_ATP\_DEPENDENT\_CHROMATIN\_REMODELING |  | 63 | -0.41 | -1.42 | 0.034 | 0.360 | 1.000 | 3900 | tags=48%, list=32%, signal=70% |
| 1008 | GOBP\_CHROMOSOME\_SEPARATION |  | 80 | -0.40 | -1.42 | 0.024 | 0.361 | 1.000 | 4419 | tags=46%, list=37%, signal=73% |
| 1009 | GOBP\_MHC\_CLASS\_I\_BIOSYNTHETIC\_PROCESS |  | 4 | -0.79 | -1.42 | 0.052 | 0.361 | 1.000 | 1763 | tags=75%, list=15%, signal=88% |
| 1010 | GOBP\_PTERIDINE\_CONTAINING\_COMPOUND\_METABOLIC\_PROCESS |  | 27 | -0.48 | -1.42 | 0.054 | 0.362 | 1.000 | 4242 | tags=56%, list=35%, signal=86% |
| 1011 | GOBP\_ANTEROGRADE\_AXONAL\_PROTEIN\_TRANSPORT |  | 6 | -0.70 | -1.42 | 0.059 | 0.362 | 1.000 | 3668 | tags=100%, list=31%, signal=144% |
| 1012 | GOCC\_L\_TYPE\_VOLTAGE\_GATED\_CALCIUM\_CHANNEL\_COMPLEX |  | 6 | -0.70 | -1.42 | 0.063 | 0.362 | 1.000 | 69 | tags=17%, list=1%, signal=17% |
| 1013 | GOBP\_PROTEIN\_SUMOYLATION |  | 63 | -0.41 | -1.42 | 0.033 | 0.362 | 1.000 | 4080 | tags=51%, list=34%, signal=77% |
| 1014 | GOBP\_REGULATION\_OF\_CALCIUM\_ION\_DEPENDENT\_EXOCYTOSIS |  | 26 | -0.48 | -1.42 | 0.053 | 0.361 | 1.000 | 1699 | tags=19%, list=14%, signal=22% |
| 1015 | GOBP\_POSITIVE\_REGULATION\_OF\_MHC\_CLASS\_II\_BIOSYNTHETIC\_PROCESS |  | 5 | -0.73 | -1.42 | 0.055 | 0.361 | 1.000 | 3205 | tags=100%, list=27%, signal=136% |
| 1016 | GOBP\_CELL\_CHEMOTAXIS |  | 181 | -0.37 | -1.42 | 0.004 | 0.361 | 1.000 | 3313 | tags=40%, list=28%, signal=55% |
| 1017 | GOCC\_X\_CHROMOSOME |  | 5 | -0.74 | -1.42 | 0.056 | 0.361 | 1.000 | 2016 | tags=60%, list=17%, signal=72% |
| 1018 | GOCC\_CELL\_BODY |  | 357 | -0.36 | -1.42 | 0.000 | 0.361 | 1.000 | 2970 | tags=33%, list=25%, signal=42% |
| 1019 | GOBP\_POSITIVE\_REGULATION\_OF\_LAMELLIPODIUM\_ASSEMBLY |  | 21 | -0.50 | -1.42 | 0.073 | 0.361 | 1.000 | 2757 | tags=43%, list=23%, signal=56% |
| 1020 | GOMF\_PHOSPHATASE\_BINDING |  | 142 | -0.38 | -1.41 | 0.007 | 0.362 | 1.000 | 3510 | tags=43%, list=29%, signal=60% |
| 1021 | GOBP\_NEGATIVE\_REGULATION\_OF\_CELL\_MATRIX\_ADHESION |  | 28 | -0.48 | -1.41 | 0.057 | 0.362 | 1.000 | 3575 | tags=50%, list=30%, signal=71% |
| 1022 | GOBP\_BEHAVIORAL\_RESPONSE\_TO\_PAIN |  | 10 | -0.60 | -1.41 | 0.072 | 0.362 | 1.000 | 1255 | tags=30%, list=10%, signal=33% |
| 1023 | GOBP\_MACROPINOCYTOSIS |  | 7 | -0.66 | -1.41 | 0.066 | 0.363 | 1.000 | 1386 | tags=43%, list=12%, signal=48% |
| 1024 | GOBP\_ECTOPIC\_GERM\_CELL\_PROGRAMMED\_CELL\_DEATH |  | 4 | -0.78 | -1.41 | 0.049 | 0.363 | 1.000 | 1013 | tags=75%, list=8%, signal=82% |
| 1025 | GOBP\_REGULATION\_OF\_DENDRITIC\_SPINE\_MORPHOGENESIS |  | 35 | -0.45 | -1.41 | 0.046 | 0.365 | 1.000 | 3760 | tags=49%, list=31%, signal=70% |
| 1026 | GOBP\_DORSAL\_SPINAL\_CORD\_DEVELOPMENT |  | 8 | -0.64 | -1.41 | 0.068 | 0.364 | 1.000 | 1871 | tags=50%, list=16%, signal=59% |
| 1027 | GOBP\_NEURAL\_CREST\_FORMATION |  | 9 | -0.62 | -1.41 | 0.073 | 0.364 | 1.000 | 1708 | tags=44%, list=14%, signal=52% |
| 1028 | GOBP\_MAMMARY\_GLAND\_EPITHELIAL\_CELL\_DIFFERENTIATION |  | 11 | -0.59 | -1.41 | 0.073 | 0.364 | 1.000 | 1861 | tags=27%, list=15%, signal=32% |
| 1029 | GOBP\_REGULATION\_OF\_REACTIVE\_OXYGEN\_SPECIES\_BIOSYNTHETIC\_PROCESS |  | 59 | -0.42 | -1.41 | 0.037 | 0.364 | 1.000 | 2518 | tags=32%, list=21%, signal=41% |
| 1030 | GOBP\_REGULATION\_OF\_TRANSFERASE\_ACTIVITY |  | 732 | -0.36 | -1.41 | 0.000 | 0.364 | 1.000 | 2997 | tags=31%, list=25%, signal=39% |
| 1031 | GOCC\_POSTSYNAPSE |  | 397 | -0.36 | -1.41 | 0.000 | 0.364 | 1.000 | 4052 | tags=40%, list=34%, signal=58% |
| 1032 | GOBP\_MRNA\_CIS\_SPLICING\_VIA\_SPLICEOSOME |  | 17 | -0.53 | -1.41 | 0.069 | 0.364 | 1.000 | 3485 | tags=35%, list=29%, signal=50% |
| 1033 | GOBP\_NEUTROPHIL\_MIGRATION |  | 70 | -0.41 | -1.41 | 0.027 | 0.364 | 1.000 | 2357 | tags=37%, list=20%, signal=46% |
| 1034 | GOBP\_HOMOLOGOUS\_RECOMBINATION |  | 39 | -0.44 | -1.41 | 0.051 | 0.364 | 1.000 | 5365 | tags=67%, list=45%, signal=120% |
| 1035 | GOMF\_HYDROXYMETHYL\_FORMYL\_AND\_RELATED\_TRANSFERASE\_ACTIVITY |  | 6 | -0.69 | -1.41 | 0.061 | 0.363 | 1.000 | 2929 | tags=50%, list=24%, signal=66% |
| 1036 | GOCC\_EXTERNAL\_SIDE\_OF\_APICAL\_PLASMA\_MEMBRANE |  | 5 | -0.73 | -1.41 | 0.062 | 0.364 | 1.000 | 3155 | tags=80%, list=26%, signal=108% |
| 1037 | GOBP\_NEGATIVE\_REGULATION\_OF\_TORC1\_SIGNALING |  | 12 | -0.57 | -1.41 | 0.071 | 0.364 | 1.000 | 3425 | tags=50%, list=29%, signal=70% |
| 1038 | GOMF\_THYROID\_HORMONE\_BINDING |  | 4 | -0.78 | -1.41 | 0.047 | 0.364 | 1.000 | 51 | tags=25%, list=0%, signal=25% |
| 1039 | GOBP\_FORMATE\_METABOLIC\_PROCESS |  | 5 | -0.73 | -1.41 | 0.057 | 0.364 | 1.000 | 1970 | tags=60%, list=16%, signal=72% |
| 1040 | GOBP\_PROTEIN\_DE\_ADP\_RIBOSYLATION |  | 3 | -0.85 | -1.41 | 0.043 | 0.364 | 1.000 | 259 | tags=33%, list=2%, signal=34% |
| 1041 | GOMF\_VOLTAGE\_GATED\_CHLORIDE\_CHANNEL\_ACTIVITY |  | 8 | -0.64 | -1.41 | 0.063 | 0.363 | 1.000 | 2059 | tags=50%, list=17%, signal=60% |
| 1042 | GOBP\_HEART\_VALVE\_FORMATION |  | 9 | -0.62 | -1.41 | 0.069 | 0.363 | 1.000 | 3314 | tags=56%, list=28%, signal=77% |
| 1043 | GOBP\_REGULATION\_OF\_TELOMERE\_CAPPING |  | 21 | -0.50 | -1.41 | 0.066 | 0.364 | 1.000 | 1851 | tags=43%, list=15%, signal=51% |
| 1044 | GOBP\_NEGATIVE\_REGULATION\_OF\_DEVELOPMENTAL\_GROWTH |  | 72 | -0.41 | -1.41 | 0.026 | 0.364 | 1.000 | 2708 | tags=36%, list=23%, signal=46% |
| 1045 | GOMF\_HORMONE\_BINDING |  | 53 | -0.42 | -1.41 | 0.042 | 0.365 | 1.000 | 2218 | tags=26%, list=18%, signal=32% |
| 1046 | GOCC\_GUANYL\_NUCLEOTIDE\_EXCHANGE\_FACTOR\_COMPLEX |  | 12 | -0.57 | -1.41 | 0.071 | 0.365 | 1.000 | 4837 | tags=92%, list=40%, signal=153% |
| 1047 | GOBP\_REGULATION\_OF\_PHOSPHATASE\_ACTIVITY |  | 117 | -0.39 | -1.41 | 0.015 | 0.364 | 1.000 | 3547 | tags=41%, list=30%, signal=58% |
| 1048 | GOMF\_RNA\_POLYMERASE\_I\_CORE\_BINDING |  | 3 | -0.84 | -1.41 | 0.044 | 0.365 | 1.000 | 1784 | tags=67%, list=15%, signal=78% |
| 1049 | GOBP\_POSITIVE\_REGULATION\_OF\_NUCLEOTIDE\_BINDING\_OLIGOMERIZATION\_DOMAIN\_CONTAINING\_SIGNALING\_PATHWAY |  | 3 | -0.84 | -1.41 | 0.039 | 0.365 | 1.000 | 1852 | tags=67%, list=15%, signal=79% |
| 1050 | GOBP\_VIRION\_ATTACHMENT\_TO\_HOST\_CELL |  | 5 | -0.73 | -1.41 | 0.059 | 0.366 | 1.000 | 2399 | tags=40%, list=20%, signal=50% |
| 1051 | GOBP\_GOLGI\_TO\_LYSOSOME\_TRANSPORT |  | 8 | -0.64 | -1.41 | 0.063 | 0.367 | 1.000 | 3227 | tags=63%, list=27%, signal=85% |
| 1052 | GOBP\_NEGATIVE\_REGULATION\_OF\_CALCIUM\_ION\_DEPENDENT\_EXOCYTOSIS |  | 8 | -0.64 | -1.41 | 0.068 | 0.367 | 1.000 | 12 | tags=13%, list=0%, signal=13% |
| 1053 | GOBP\_POSITIVE\_REGULATION\_OF\_NEURON\_PROJECTION\_DEVELOPMENT |  | 115 | -0.39 | -1.41 | 0.016 | 0.367 | 1.000 | 4907 | tags=54%, list=41%, signal=90% |
| 1054 | GOBP\_PHOSPHOLIPID\_DEPHOSPHORYLATION |  | 36 | -0.45 | -1.41 | 0.055 | 0.367 | 1.000 | 3934 | tags=56%, list=33%, signal=82% |
| 1055 | GOBP\_HEMIDESMOSOME\_ASSEMBLY |  | 8 | -0.64 | -1.41 | 0.068 | 0.367 | 1.000 | 3856 | tags=88%, list=32%, signal=129% |
| 1056 | GOBP\_POSITIVE\_REGULATION\_OF\_MULTICELLULAR\_ORGANISMAL\_PROCESS |  | 864 | -0.35 | -1.41 | 0.000 | 0.367 | 1.000 | 3448 | tags=38%, list=29%, signal=49% |
| 1057 | GOBP\_REGULATION\_OF\_DENDRITIC\_SPINE\_DEVELOPMENT |  | 48 | -0.43 | -1.41 | 0.039 | 0.368 | 1.000 | 4673 | tags=56%, list=39%, signal=92% |
| 1058 | GOBP\_HARD\_PALATE\_DEVELOPMENT |  | 4 | -0.78 | -1.41 | 0.054 | 0.368 | 1.000 | 2604 | tags=75%, list=22%, signal=96% |
| 1059 | GOBP\_NEUROPEPTIDE\_SIGNALING\_PATHWAY |  | 33 | -0.46 | -1.41 | 0.055 | 0.369 | 1.000 | 3788 | tags=55%, list=32%, signal=79% |
| 1060 | GOBP\_PROTEIN\_LOCALIZATION\_TO\_CHROMOSOME\_CENTROMERIC\_REGION |  | 22 | -0.49 | -1.41 | 0.069 | 0.368 | 1.000 | 5641 | tags=82%, list=47%, signal=154% |
| 1061 | GOBP\_LUNG\_CELL\_DIFFERENTIATION |  | 16 | -0.53 | -1.41 | 0.071 | 0.369 | 1.000 | 2212 | tags=50%, list=18%, signal=61% |
| 1062 | GOBP\_REGULATION\_OF\_NEURAL\_PRECURSOR\_CELL\_PROLIFERATION |  | 57 | -0.42 | -1.41 | 0.039 | 0.368 | 1.000 | 3565 | tags=40%, list=30%, signal=57% |
| 1063 | GOBP\_CENTROMERE\_COMPLEX\_ASSEMBLY |  | 39 | -0.44 | -1.41 | 0.049 | 0.368 | 1.000 | 4349 | tags=59%, list=36%, signal=92% |
| 1064 | GOBP\_ACTIN\_CROSSLINK\_FORMATION |  | 11 | -0.58 | -1.41 | 0.074 | 0.368 | 1.000 | 2598 | tags=45%, list=22%, signal=58% |
| 1065 | GOBP\_NEGATIVE\_REGULATION\_OF\_CELLULAR\_RESPONSE\_TO\_VASCULAR\_ENDOTHELIAL\_GROWTH\_FACTOR\_STIMULUS |  | 9 | -0.62 | -1.41 | 0.074 | 0.368 | 1.000 | 3724 | tags=67%, list=31%, signal=97% |
| 1066 | GOBP\_POSITIVE\_REGULATION\_OF\_SMOOTH\_MUSCLE\_CONTRACTION |  | 18 | -0.52 | -1.41 | 0.074 | 0.368 | 1.000 | 786 | tags=28%, list=7%, signal=30% |
| 1067 | GOBP\_NEGATIVE\_REGULATION\_OF\_VASCULAR\_ENDOTHELIAL\_GROWTH\_FACTOR\_RECEPTOR\_SIGNALING\_PATHWAY |  | 9 | -0.62 | -1.41 | 0.070 | 0.368 | 1.000 | 2337 | tags=56%, list=19%, signal=69% |
| 1068 | GOBP\_VESICLE\_TETHERING |  | 7 | -0.67 | -1.41 | 0.071 | 0.368 | 1.000 | 2797 | tags=57%, list=23%, signal=74% |
| 1069 | GOBP\_HISTONE\_H3\_K4\_TRIMETHYLATION |  | 13 | -0.56 | -1.41 | 0.077 | 0.368 | 1.000 | 4677 | tags=77%, list=39%, signal=126% |
| 1070 | GOBP\_CAMERA\_TYPE\_EYE\_DEVELOPMENT |  | 186 | -0.37 | -1.41 | 0.004 | 0.368 | 1.000 | 3638 | tags=40%, list=30%, signal=57% |
| 1071 | GOMF\_PROTEIN\_TYROSINE\_KINASE\_ACTIVATOR\_ACTIVITY |  | 13 | -0.56 | -1.41 | 0.075 | 0.368 | 1.000 | 1490 | tags=38%, list=12%, signal=44% |
| 1072 | GOMF\_GLUCURONOSYLTRANSFERASE\_ACTIVITY |  | 13 | -0.56 | -1.41 | 0.071 | 0.368 | 1.000 | 1000 | tags=31%, list=8%, signal=34% |
| 1073 | GOBP\_T\_CELL\_APOPTOTIC\_PROCESS |  | 34 | -0.46 | -1.41 | 0.050 | 0.368 | 1.000 | 1852 | tags=35%, list=15%, signal=42% |
| 1074 | GOBP\_PROXIMAL\_DISTAL\_PATTERN\_FORMATION |  | 18 | -0.52 | -1.41 | 0.065 | 0.367 | 1.000 | 1146 | tags=28%, list=10%, signal=31% |
| 1075 | GOBP\_CHEMOKINE\_C\_X\_C\_MOTIF\_LIGAND\_2\_PRODUCTION |  | 14 | -0.55 | -1.41 | 0.068 | 0.367 | 1.000 | 3007 | tags=57%, list=25%, signal=76% |
| 1076 | GOBP\_MYELIN\_MAINTENANCE |  | 10 | -0.59 | -1.41 | 0.076 | 0.367 | 1.000 | 4312 | tags=80%, list=36%, signal=125% |
| 1077 | GOCC\_GAP\_JUNCTION |  | 17 | -0.53 | -1.41 | 0.067 | 0.367 | 1.000 | 2313 | tags=59%, list=19%, signal=73% |
| 1078 | GOBP\_ANTERIOR\_POSTERIOR\_PATTERN\_SPECIFICATION |  | 121 | -0.38 | -1.41 | 0.017 | 0.367 | 1.000 | 3126 | tags=36%, list=26%, signal=49% |
| 1079 | GOCC\_PARANODE\_REGION\_OF\_AXON |  | 7 | -0.67 | -1.41 | 0.066 | 0.368 | 1.000 | 1847 | tags=43%, list=15%, signal=51% |
| 1080 | GOBP\_BILE\_ACID\_SECRETION |  | 7 | -0.67 | -1.41 | 0.069 | 0.368 | 1.000 | 1269 | tags=43%, list=11%, signal=48% |
| 1081 | GOMF\_MOLECULAR\_TRANSDUCER\_ACTIVITY |  | 551 | -0.36 | -1.41 | 0.000 | 0.367 | 1.000 | 3557 | tags=38%, list=30%, signal=52% |
| 1082 | GOMF\_PHOSPHATIDYLSERINE\_FLIPPASE\_ACTIVITY |  | 5 | -0.73 | -1.41 | 0.061 | 0.369 | 1.000 | 952 | tags=60%, list=8%, signal=65% |
| 1083 | GOBP\_NEGATIVE\_REGULATION\_OF\_CELL\_DEVELOPMENT |  | 111 | -0.39 | -1.41 | 0.015 | 0.368 | 1.000 | 1707 | tags=23%, list=14%, signal=26% |
| 1084 | GOBP\_REGULATION\_OF\_PSEUDOPODIUM\_ASSEMBLY |  | 9 | -0.62 | -1.41 | 0.073 | 0.368 | 1.000 | 2480 | tags=44%, list=21%, signal=56% |
| 1085 | GOBP\_REGULATION\_OF\_MEGAKARYOCYTE\_DIFFERENTIATION |  | 42 | -0.44 | -1.41 | 0.049 | 0.368 | 1.000 | 3235 | tags=45%, list=27%, signal=62% |
| 1086 | GOMF\_INTRACELLULAR\_LIGAND\_GATED\_ION\_CHANNEL\_ACTIVITY |  | 18 | -0.52 | -1.41 | 0.073 | 0.368 | 1.000 | 2038 | tags=39%, list=17%, signal=47% |
| 1087 | GOBP\_REGULATION\_OF\_T\_CELL\_RECEPTOR\_SIGNALING\_PATHWAY |  | 30 | -0.47 | -1.40 | 0.060 | 0.369 | 1.000 | 2857 | tags=43%, list=24%, signal=57% |
| 1088 | GOBP\_CELL\_MIGRATION\_INVOLVED\_IN\_SPROUTING\_ANGIOGENESIS |  | 41 | -0.44 | -1.40 | 0.054 | 0.369 | 1.000 | 2837 | tags=39%, list=24%, signal=51% |
| 1089 | GOBP\_CALCIUM\_MEDIATED\_SIGNALING |  | 128 | -0.38 | -1.40 | 0.011 | 0.369 | 1.000 | 4627 | tags=59%, list=39%, signal=94% |
| 1090 | GOBP\_NEGATIVE\_REGULATION\_OF\_PROTEIN\_EXIT\_FROM\_ENDOPLASMIC\_RETICULUM |  | 9 | -0.62 | -1.40 | 0.074 | 0.369 | 1.000 | 2442 | tags=33%, list=20%, signal=42% |
| 1091 | GOBP\_PYRIMIDINE\_DIMER\_REPAIR |  | 7 | -0.66 | -1.40 | 0.065 | 0.369 | 1.000 | 2656 | tags=57%, list=22%, signal=73% |
| 1092 | GOBP\_CARDIOCYTE\_DIFFERENTIATION |  | 88 | -0.40 | -1.40 | 0.021 | 0.369 | 1.000 | 3331 | tags=36%, list=28%, signal=50% |
| 1093 | GOBP\_POSITIVE\_REGULATION\_OF\_HYDROLASE\_ACTIVITY |  | 543 | -0.36 | -1.40 | 0.000 | 0.370 | 1.000 | 3349 | tags=37%, list=28%, signal=49% |
| 1094 | GOBP\_THROMBIN\_ACTIVATED\_RECEPTOR\_SIGNALING\_PATHWAY |  | 11 | -0.58 | -1.40 | 0.082 | 0.369 | 1.000 | 2652 | tags=55%, list=22%, signal=70% |
| 1095 | GOBP\_POSITIVE\_REGULATION\_OF\_CYTOPLASMIC\_MRNA\_PROCESSING\_BODY\_ASSEMBLY |  | 5 | -0.72 | -1.40 | 0.063 | 0.369 | 1.000 | 2441 | tags=60%, list=20%, signal=75% |
| 1096 | GOBP\_PLATELET\_DERIVED\_GROWTH\_FACTOR\_RECEPTOR\_ALPHA\_SIGNALING\_PATHWAY |  | 5 | -0.72 | -1.40 | 0.067 | 0.369 | 1.000 | 1016 | tags=40%, list=8%, signal=44% |
| 1097 | GOMF\_KINETOCHORE\_BINDING |  | 6 | -0.69 | -1.40 | 0.071 | 0.369 | 1.000 | 3026 | tags=83%, list=25%, signal=111% |
| 1098 | GOMF\_CARBOHYDRATE\_CATION\_SYMPORTER\_ACTIVITY |  | 11 | -0.59 | -1.40 | 0.077 | 0.370 | 1.000 | 894 | tags=45%, list=7%, signal=49% |
| 1099 | GOCC\_GAMMA\_TUBULIN\_COMPLEX |  | 15 | -0.54 | -1.40 | 0.077 | 0.370 | 1.000 | 2265 | tags=47%, list=19%, signal=57% |
| 1100 | GOBP\_NEGATIVE\_REGULATION\_OF\_T\_CELL\_MIGRATION |  | 4 | -0.77 | -1.40 | 0.052 | 0.370 | 1.000 | 249 | tags=25%, list=2%, signal=26% |
| 1101 | GOBP\_FOREBRAIN\_GENERATION\_OF\_NEURONS |  | 33 | -0.46 | -1.40 | 0.054 | 0.370 | 1.000 | 2466 | tags=33%, list=21%, signal=42% |
| 1102 | GOCC\_CD40\_RECEPTOR\_COMPLEX |  | 9 | -0.61 | -1.40 | 0.080 | 0.370 | 1.000 | 2458 | tags=56%, list=20%, signal=70% |
| 1103 | GOBP\_NEURON\_MIGRATION |  | 97 | -0.39 | -1.40 | 0.019 | 0.370 | 1.000 | 3222 | tags=36%, list=27%, signal=49% |
| 1104 | GOBP\_REGULATION\_OF\_CYTOPLASMIC\_MRNA\_PROCESSING\_BODY\_ASSEMBLY |  | 7 | -0.66 | -1.40 | 0.069 | 0.370 | 1.000 | 2441 | tags=57%, list=20%, signal=72% |
| 1105 | GOBP\_NEGATIVE\_REGULATION\_OF\_ACTIVATED\_T\_CELL\_PROLIFERATION |  | 8 | -0.64 | -1.40 | 0.075 | 0.370 | 1.000 | 3163 | tags=63%, list=26%, signal=85% |
| 1106 | GOBP\_POSITIVE\_REGULATION\_OF\_ANION\_TRANSMEMBRANE\_TRANSPORT |  | 44 | -0.43 | -1.40 | 0.045 | 0.370 | 1.000 | 2038 | tags=34%, list=17%, signal=41% |
| 1107 | GOBP\_REGULATION\_OF\_INSULIN\_RECEPTOR\_SIGNALING\_PATHWAY |  | 49 | -0.43 | -1.40 | 0.041 | 0.370 | 1.000 | 3692 | tags=49%, list=31%, signal=70% |
| 1108 | GOBP\_CGMP\_CATABOLIC\_PROCESS |  | 4 | -0.78 | -1.40 | 0.054 | 0.370 | 1.000 | 548 | tags=50%, list=5%, signal=52% |
| 1109 | GOBP\_SPINDLE\_LOCALIZATION |  | 42 | -0.44 | -1.40 | 0.053 | 0.370 | 1.000 | 3834 | tags=43%, list=32%, signal=63% |
| 1110 | GOBP\_MAMMARY\_GLAND\_FORMATION |  | 6 | -0.69 | -1.40 | 0.064 | 0.371 | 1.000 | 1146 | tags=50%, list=10%, signal=55% |
| 1111 | GOBP\_LEUKOCYTE\_MIGRATION\_INVOLVED\_IN\_INFLAMMATORY\_RESPONSE |  | 13 | -0.56 | -1.40 | 0.075 | 0.372 | 1.000 | 1734 | tags=54%, list=14%, signal=63% |
| 1112 | GOMF\_VOLTAGE\_GATED\_ION\_CHANNEL\_ACTIVITY |  | 102 | -0.39 | -1.40 | 0.018 | 0.373 | 1.000 | 2216 | tags=28%, list=18%, signal=35% |
| 1113 | GOBP\_POSITIVE\_REGULATION\_OF\_LEUKOCYTE\_APOPTOTIC\_PROCESS |  | 17 | -0.52 | -1.40 | 0.072 | 0.372 | 1.000 | 3219 | tags=65%, list=27%, signal=88% |
| 1114 | GOBP\_SERTOLI\_CELL\_DEVELOPMENT |  | 7 | -0.66 | -1.40 | 0.072 | 0.372 | 1.000 | 2625 | tags=57%, list=22%, signal=73% |
| 1115 | GOBP\_REGULATION\_OF\_TELOMERE\_MAINTENANCE |  | 65 | -0.41 | -1.40 | 0.028 | 0.372 | 1.000 | 4935 | tags=55%, list=41%, signal=94% |
| 1116 | GOBP\_NEGATIVE\_REGULATION\_OF\_CELLULAR\_RESPONSE\_TO\_INSULIN\_STIMULUS |  | 29 | -0.46 | -1.40 | 0.059 | 0.372 | 1.000 | 2456 | tags=38%, list=20%, signal=48% |
| 1117 | GOBP\_CENTROSOME\_SEPARATION |  | 13 | -0.56 | -1.40 | 0.084 | 0.372 | 1.000 | 1488 | tags=23%, list=12%, signal=26% |
| 1118 | GOBP\_HISTONE\_H3\_K4\_MONOMETHYLATION |  | 5 | -0.73 | -1.40 | 0.065 | 0.371 | 1.000 | 2123 | tags=80%, list=18%, signal=97% |
| 1119 | GOBP\_CELL\_JUNCTION\_ASSEMBLY |  | 279 | -0.36 | -1.40 | 0.002 | 0.371 | 1.000 | 4429 | tags=48%, list=37%, signal=74% |
| 1120 | GOBP\_REGULATION\_OF\_OSSIFICATION |  | 71 | -0.40 | -1.40 | 0.034 | 0.371 | 1.000 | 4484 | tags=55%, list=37%, signal=87% |
| 1121 | GOBP\_POSITIVE\_REGULATION\_OF\_PHOSPHORUS\_METABOLIC\_PROCESS |  | 662 | -0.35 | -1.40 | 0.000 | 0.371 | 1.000 | 3344 | tags=34%, list=28%, signal=44% |
| 1122 | GOMF\_HEPARAN\_SULFATE\_SULFOTRANSFERASE\_ACTIVITY |  | 8 | -0.63 | -1.40 | 0.075 | 0.371 | 1.000 | 3719 | tags=88%, list=31%, signal=127% |
| 1123 | GOMF\_TRANSLATION\_ACTIVATOR\_ACTIVITY |  | 8 | -0.64 | -1.40 | 0.070 | 0.371 | 1.000 | 765 | tags=38%, list=6%, signal=40% |
| 1124 | GOMF\_TRANSFERASE\_ACTIVITY\_TRANSFERRING\_PHOSPHORUS\_CONTAINING\_GROUPS |  | 643 | -0.35 | -1.40 | 0.000 | 0.372 | 1.000 | 4426 | tags=47%, list=37%, signal=70% |
| 1125 | GOBP\_LYMPH\_VESSEL\_DEVELOPMENT |  | 20 | -0.51 | -1.40 | 0.075 | 0.372 | 1.000 | 3196 | tags=50%, list=27%, signal=68% |
| 1126 | GOBP\_REGULATION\_OF\_CELL\_DEVELOPMENT |  | 337 | -0.36 | -1.40 | 0.001 | 0.372 | 1.000 | 3196 | tags=33%, list=27%, signal=43% |
| 1127 | GOBP\_VERY\_LOW\_DENSITY\_LIPOPROTEIN\_PARTICLE\_ASSEMBLY |  | 8 | -0.64 | -1.40 | 0.076 | 0.372 | 1.000 | 3217 | tags=63%, list=27%, signal=85% |
| 1128 | GOBP\_PLACENTA\_DEVELOPMENT |  | 91 | -0.39 | -1.40 | 0.025 | 0.372 | 1.000 | 2879 | tags=36%, list=24%, signal=47% |
| 1129 | GOBP\_POSITIVE\_REGULATION\_OF\_TRANSCRIPTION\_FROM\_RNA\_POLYMERASE\_II\_PROMOTER\_INVOLVED\_IN\_HEART\_DEVELOPMENT |  | 5 | -0.72 | -1.40 | 0.071 | 0.372 | 1.000 | 1459 | tags=40%, list=12%, signal=46% |
| 1130 | GOBP\_REGULATION\_OF\_RESPONSE\_TO\_FOOD |  | 10 | -0.59 | -1.40 | 0.083 | 0.372 | 1.000 | 1748 | tags=40%, list=15%, signal=47% |
| 1131 | GOBP\_INTERLEUKIN\_18\_MEDIATED\_SIGNALING\_PATHWAY |  | 6 | -0.68 | -1.40 | 0.075 | 0.372 | 1.000 | 3613 | tags=67%, list=30%, signal=95% |
| 1132 | GOBP\_CEREBELLAR\_CORTEX\_MORPHOGENESIS |  | 21 | -0.50 | -1.40 | 0.068 | 0.373 | 1.000 | 1452 | tags=24%, list=12%, signal=27% |
| 1133 | GOMF\_DNA\_POLYMERASE\_ACTIVITY |  | 22 | -0.50 | -1.40 | 0.068 | 0.373 | 1.000 | 3722 | tags=45%, list=31%, signal=66% |
| 1134 | GOCC\_INTRINSIC\_COMPONENT\_OF\_EXTERNAL\_SIDE\_OF\_PLASMA\_MEMBRANE |  | 11 | -0.58 | -1.40 | 0.076 | 0.373 | 1.000 | 1913 | tags=36%, list=16%, signal=43% |
| 1135 | GOBP\_POSITIVE\_REGULATION\_OF\_LONG\_TERM\_SYNAPTIC\_DEPRESSION |  | 4 | -0.78 | -1.40 | 0.054 | 0.372 | 1.000 | 2087 | tags=50%, list=17%, signal=60% |
| 1136 | GOBP\_POSITIVE\_REGULATION\_OF\_DEVELOPMENTAL\_PROCESS |  | 820 | -0.35 | -1.40 | 0.000 | 0.372 | 1.000 | 3658 | tags=38%, list=30%, signal=51% |
| 1137 | GOBP\_CELLULAR\_RESPONSE\_TO\_ETHANOL |  | 13 | -0.56 | -1.40 | 0.075 | 0.372 | 1.000 | 685 | tags=31%, list=6%, signal=33% |
| 1138 | GOBP\_POSITIVE\_REGULATION\_OF\_CATALYTIC\_ACTIVITY |  | 996 | -0.35 | -1.40 | 0.000 | 0.372 | 1.000 | 3672 | tags=37%, list=31%, signal=49% |
| 1139 | GOCC\_CHROMOSOME\_TELOMERIC\_REGION |  | 110 | -0.38 | -1.40 | 0.020 | 0.372 | 1.000 | 5009 | tags=53%, list=42%, signal=90% |
| 1140 | GOBP\_ANTIGEN\_RECEPTOR\_MEDIATED\_SIGNALING\_PATHWAY |  | 164 | -0.37 | -1.40 | 0.009 | 0.372 | 1.000 | 3069 | tags=35%, list=26%, signal=47% |
| 1141 | GOMF\_CLATHRIN\_BINDING |  | 51 | -0.42 | -1.40 | 0.046 | 0.372 | 1.000 | 4775 | tags=53%, list=40%, signal=88% |
| 1142 | GOBP\_REGULATION\_OF\_TOLL\_LIKE\_RECEPTOR\_4\_SIGNALING\_PATHWAY |  | 17 | -0.52 | -1.40 | 0.075 | 0.375 | 1.000 | 2480 | tags=47%, list=21%, signal=59% |
| 1143 | GOBP\_EPITHELIAL\_TUBE\_FORMATION |  | 87 | -0.39 | -1.40 | 0.025 | 0.375 | 1.000 | 4271 | tags=45%, list=36%, signal=69% |
| 1144 | GOBP\_COPII\_COATED\_VESICLE\_CARGO\_LOADING |  | 14 | -0.55 | -1.40 | 0.080 | 0.376 | 1.000 | 4239 | tags=71%, list=35%, signal=110% |
| 1145 | GOBP\_POSITIVE\_REGULATION\_OF\_PROTEIN\_MODIFICATION\_PROCESS |  | 727 | -0.35 | -1.40 | 0.000 | 0.376 | 1.000 | 3657 | tags=36%, list=30%, signal=49% |
| 1146 | GOBP\_BRONCHIOLE\_DEVELOPMENT |  | 4 | -0.77 | -1.40 | 0.059 | 0.375 | 1.000 | 2515 | tags=75%, list=21%, signal=95% |
| 1147 | GOBP\_CARDIAC\_MUSCLE\_CELL\_DIFFERENTIATION |  | 66 | -0.41 | -1.40 | 0.030 | 0.376 | 1.000 | 3331 | tags=39%, list=28%, signal=54% |
| 1148 | GOBP\_MAPK\_CASCADE |  | 592 | -0.35 | -1.40 | 0.000 | 0.375 | 1.000 | 3728 | tags=39%, list=31%, signal=54% |
| 1149 | GOBP\_GLYCEROLIPID\_METABOLIC\_PROCESS |  | 316 | -0.36 | -1.40 | 0.001 | 0.375 | 1.000 | 4057 | tags=44%, list=34%, signal=65% |
| 1150 | GOCC\_PROTEIN\_COMPLEX\_INVOLVED\_IN\_CELL\_ADHESION |  | 27 | -0.48 | -1.40 | 0.066 | 0.375 | 1.000 | 2951 | tags=41%, list=25%, signal=54% |
| 1151 | GOBP\_REGULATION\_OF\_ALKALINE\_PHOSPHATASE\_ACTIVITY |  | 5 | -0.72 | -1.40 | 0.070 | 0.375 | 1.000 | 2474 | tags=80%, list=21%, signal=101% |
| 1152 | GOBP\_SCHWANN\_CELL\_DEVELOPMENT |  | 22 | -0.49 | -1.40 | 0.069 | 0.375 | 1.000 | 4548 | tags=59%, list=38%, signal=95% |
| 1153 | GOBP\_NEGATIVE\_REGULATION\_OF\_ACTIN\_FILAMENT\_BUNDLE\_ASSEMBLY |  | 22 | -0.49 | -1.40 | 0.072 | 0.375 | 1.000 | 3934 | tags=59%, list=33%, signal=88% |
| 1154 | GOBP\_NUCLEOSOME\_ASSEMBLY |  | 53 | -0.42 | -1.40 | 0.045 | 0.374 | 1.000 | 5406 | tags=62%, list=45%, signal=113% |
| 1155 | GOMF\_TUMOR\_NECROSIS\_FACTOR\_RECEPTOR\_SUPERFAMILY\_BINDING |  | 32 | -0.46 | -1.40 | 0.059 | 0.374 | 1.000 | 2769 | tags=44%, list=23%, signal=57% |
| 1156 | GOCC\_H4\_H2A\_HISTONE\_ACETYLTRANSFERASE\_COMPLEX |  | 16 | -0.53 | -1.40 | 0.077 | 0.374 | 1.000 | 3279 | tags=44%, list=27%, signal=60% |
| 1157 | GOBP\_POSITIVE\_REGULATION\_OF\_INTERLEUKIN\_2\_PRODUCTION |  | 24 | -0.48 | -1.40 | 0.071 | 0.375 | 1.000 | 2345 | tags=42%, list=20%, signal=52% |
| 1158 | GOBP\_IRIS\_MORPHOGENESIS |  | 6 | -0.69 | -1.39 | 0.072 | 0.375 | 1.000 | 3126 | tags=67%, list=26%, signal=90% |
| 1159 | GOMF\_THIOESTERASE\_BINDING |  | 10 | -0.60 | -1.39 | 0.073 | 0.375 | 1.000 | 2995 | tags=50%, list=25%, signal=67% |
| 1160 | GOBP\_INOSITOL\_METABOLIC\_PROCESS |  | 9 | -0.61 | -1.39 | 0.076 | 0.375 | 1.000 | 3772 | tags=78%, list=31%, signal=113% |
| 1161 | GOBP\_POSITIVE\_REGULATION\_OF\_RESPONSE\_TO\_TUMOR\_CELL |  | 5 | -0.72 | -1.39 | 0.068 | 0.375 | 1.000 | 3240 | tags=80%, list=27%, signal=110% |
| 1162 | GOBP\_POSITIVE\_REGULATION\_BY\_HOST\_OF\_VIRAL\_GENOME\_REPLICATION |  | 7 | -0.66 | -1.39 | 0.074 | 0.375 | 1.000 | 2916 | tags=71%, list=24%, signal=94% |
| 1163 | GOBP\_B\_CELL\_ACTIVATION |  | 168 | -0.37 | -1.39 | 0.008 | 0.375 | 1.000 | 2771 | tags=35%, list=23%, signal=45% |
| 1164 | GOBP\_FIBROBLAST\_GROWTH\_FACTOR\_RECEPTOR\_SIGNALING\_PATHWAY |  | 74 | -0.40 | -1.39 | 0.035 | 0.375 | 1.000 | 2896 | tags=34%, list=24%, signal=44% |
| 1165 | GOBP\_RESPONSE\_TO\_CALCIUM\_ION |  | 98 | -0.39 | -1.39 | 0.024 | 0.375 | 1.000 | 2820 | tags=32%, list=23%, signal=41% |
| 1166 | GOMF\_PROTEIN\_PHOSPHATASE\_2A\_BINDING |  | 27 | -0.47 | -1.39 | 0.068 | 0.375 | 1.000 | 4177 | tags=59%, list=35%, signal=91% |
| 1167 | GOBP\_REGULATION\_OF\_MULTICELLULAR\_ORGANISM\_GROWTH |  | 40 | -0.44 | -1.39 | 0.051 | 0.376 | 1.000 | 2670 | tags=33%, list=22%, signal=42% |
| 1168 | GOBP\_WATER\_TRANSPORT |  | 13 | -0.55 | -1.39 | 0.077 | 0.376 | 1.000 | 2421 | tags=46%, list=20%, signal=58% |
| 1169 | GOBP\_MEIOTIC\_CHROMOSOME\_SEPARATION |  | 19 | -0.51 | -1.39 | 0.074 | 0.376 | 1.000 | 3976 | tags=63%, list=33%, signal=94% |
| 1170 | GOCC\_NEURON\_PROJECTION |  | 830 | -0.35 | -1.39 | 0.000 | 0.376 | 1.000 | 4052 | tags=41%, list=34%, signal=58% |
| 1171 | GOMF\_PHOSPHATIDYLINOSITOL\_4\_5\_BISPHOSPHATE\_PHOSPHATASE\_ACTIVITY |  | 11 | -0.58 | -1.39 | 0.085 | 0.377 | 1.000 | 796 | tags=36%, list=7%, signal=39% |
| 1172 | GOBP\_ORGANIC\_HYDROXY\_COMPOUND\_TRANSPORT |  | 168 | -0.37 | -1.39 | 0.009 | 0.377 | 1.000 | 2752 | tags=30%, list=23%, signal=39% |
| 1173 | GOBP\_TRANSFORMING\_GROWTH\_FACTOR\_BETA2\_PRODUCTION |  | 6 | -0.69 | -1.39 | 0.071 | 0.377 | 1.000 | 2002 | tags=50%, list=17%, signal=60% |
| 1174 | GOBP\_DENDRITIC\_SPINE\_MAINTENANCE |  | 14 | -0.54 | -1.39 | 0.090 | 0.379 | 1.000 | 2158 | tags=43%, list=18%, signal=52% |
| 1175 | GOBP\_POSITIVE\_REGULATION\_OF\_MAINTENANCE\_OF\_SISTER\_CHROMATID\_COHESION |  | 4 | -0.77 | -1.39 | 0.061 | 0.380 | 1.000 | 2520 | tags=75%, list=21%, signal=95% |
| 1176 | GOBP\_SENSORY\_PERCEPTION\_OF\_BITTER\_TASTE |  | 5 | -0.72 | -1.39 | 0.064 | 0.380 | 1.000 | 1240 | tags=40%, list=10%, signal=45% |
| 1177 | GOBP\_POSITIVE\_REGULATION\_BY\_HOST\_OF\_VIRAL\_PROCESS |  | 11 | -0.58 | -1.39 | 0.087 | 0.381 | 1.000 | 3093 | tags=64%, list=26%, signal=86% |
| 1178 | GOBP\_POSITIVE\_REGULATION\_OF\_MAST\_CELL\_ACTIVATION\_INVOLVED\_IN\_IMMUNE\_RESPONSE |  | 8 | -0.63 | -1.39 | 0.081 | 0.381 | 1.000 | 2599 | tags=38%, list=22%, signal=48% |
| 1179 | GOCC\_TRANSCRIPTION\_REPRESSOR\_COMPLEX |  | 56 | -0.41 | -1.39 | 0.046 | 0.381 | 1.000 | 3465 | tags=41%, list=29%, signal=57% |
| 1180 | GOBP\_CLUSTERING\_OF\_VOLTAGE\_GATED\_SODIUM\_CHANNELS |  | 4 | -0.77 | -1.39 | 0.060 | 0.380 | 1.000 | 1638 | tags=50%, list=14%, signal=58% |
| 1181 | GOBP\_NEGATIVE\_REGULATION\_OF\_SMALL\_GTPASE\_MEDIATED\_SIGNAL\_TRANSDUCTION |  | 48 | -0.42 | -1.39 | 0.053 | 0.380 | 1.000 | 3530 | tags=46%, list=29%, signal=65% |
| 1182 | GOBP\_REGULATION\_OF\_MICROTUBULE\_POLYMERIZATION |  | 35 | -0.45 | -1.39 | 0.062 | 0.380 | 1.000 | 3069 | tags=40%, list=26%, signal=54% |
| 1183 | GOBP\_PRO\_B\_CELL\_DIFFERENTIATION |  | 11 | -0.58 | -1.39 | 0.080 | 0.380 | 1.000 | 3219 | tags=55%, list=27%, signal=74% |
| 1184 | GOBP\_PROTEIN\_KINASE\_B\_SIGNALING |  | 180 | -0.37 | -1.39 | 0.009 | 0.380 | 1.000 | 2879 | tags=34%, list=24%, signal=44% |
| 1185 | GOBP\_CEREBELLAR\_CORTEX\_FORMATION |  | 15 | -0.53 | -1.39 | 0.083 | 0.380 | 1.000 | 2528 | tags=33%, list=21%, signal=42% |
| 1186 | GOBP\_NUCLEUS\_LOCALIZATION |  | 22 | -0.49 | -1.39 | 0.076 | 0.380 | 1.000 | 2299 | tags=45%, list=19%, signal=56% |
| 1187 | GOMF\_HISTONE\_METHYLTRANSFERASE\_BINDING |  | 7 | -0.66 | -1.39 | 0.082 | 0.380 | 1.000 | 2154 | tags=43%, list=18%, signal=52% |
| 1188 | GOBP\_POSITIVE\_REGULATION\_OF\_ADENYLATE\_CYCLASE\_ACTIVITY |  | 7 | -0.65 | -1.39 | 0.080 | 0.380 | 1.000 | 501 | tags=29%, list=4%, signal=30% |
| 1189 | GOMF\_STRUCTURAL\_CONSTITUENT\_OF\_NUCLEAR\_PORE |  | 20 | -0.50 | -1.39 | 0.076 | 0.381 | 1.000 | 4080 | tags=65%, list=34%, signal=98% |
| 1190 | GOBP\_NOTCH\_SIGNALING\_INVOLVED\_IN\_HEART\_DEVELOPMENT |  | 11 | -0.57 | -1.39 | 0.081 | 0.381 | 1.000 | 2212 | tags=45%, list=18%, signal=56% |
| 1191 | GOBP\_PREPULSE\_INHIBITION |  | 7 | -0.65 | -1.39 | 0.077 | 0.381 | 1.000 | 3392 | tags=86%, list=28%, signal=119% |
| 1192 | GOMF\_VOLTAGE\_GATED\_CALCIUM\_CHANNEL\_ACTIVITY |  | 22 | -0.49 | -1.39 | 0.076 | 0.381 | 1.000 | 2448 | tags=32%, list=20%, signal=40% |
| 1193 | GOMF\_GTPASE\_BINDING |  | 172 | -0.37 | -1.39 | 0.007 | 0.382 | 1.000 | 3797 | tags=44%, list=32%, signal=63% |
| 1194 | GOBP\_POSITIVE\_REGULATION\_OF\_NERVOUS\_SYSTEM\_DEVELOPMENT |  | 183 | -0.37 | -1.39 | 0.008 | 0.382 | 1.000 | 3196 | tags=34%, list=27%, signal=46% |
| 1195 | GOBP\_CHEMICAL\_SYNAPTIC\_TRANSMISSION\_POSTSYNAPTIC |  | 51 | -0.42 | -1.39 | 0.051 | 0.382 | 1.000 | 3708 | tags=43%, list=31%, signal=62% |
| 1196 | GOBP\_POSITIVE\_REGULATION\_OF\_PHOSPHATASE\_ACTIVITY |  | 24 | -0.48 | -1.39 | 0.076 | 0.382 | 1.000 | 2518 | tags=42%, list=21%, signal=53% |
| 1197 | GOMF\_SIRNA\_BINDING |  | 4 | -0.77 | -1.39 | 0.062 | 0.383 | 1.000 | 1680 | tags=75%, list=14%, signal=87% |
| 1198 | GOBP\_PROTEIN\_DNA\_COMPLEX\_SUBUNIT\_ORGANIZATION |  | 157 | -0.37 | -1.39 | 0.010 | 0.383 | 1.000 | 5406 | tags=59%, list=45%, signal=106% |
| 1199 | GOMF\_INTRONIC\_TRANSCRIPTION\_REGULATORY\_REGION\_SEQUENCE\_SPECIFIC\_DNA\_BINDING |  | 4 | -0.77 | -1.39 | 0.067 | 0.383 | 1.000 | 1560 | tags=50%, list=13%, signal=57% |
| 1200 | GOBP\_VASCULAR\_ASSOCIATED\_SMOOTH\_MUSCLE\_CELL\_MIGRATION |  | 18 | -0.51 | -1.39 | 0.084 | 0.384 | 1.000 | 3069 | tags=39%, list=26%, signal=52% |
| 1201 | GOMF\_TRANSMEMBRANE\_RECEPTOR\_PROTEIN\_TYROSINE\_KINASE\_ACTIVITY |  | 50 | -0.42 | -1.39 | 0.051 | 0.384 | 1.000 | 3557 | tags=38%, list=30%, signal=54% |
| 1202 | GOBP\_NEGATIVE\_REGULATION\_OF\_LEUKOCYTE\_CELL\_CELL\_ADHESION |  | 73 | -0.40 | -1.39 | 0.038 | 0.384 | 1.000 | 3692 | tags=45%, list=31%, signal=65% |
| 1203 | GOBP\_NETRIN\_ACTIVATED\_SIGNALING\_PATHWAY |  | 9 | -0.60 | -1.39 | 0.084 | 0.383 | 1.000 | 223 | tags=22%, list=2%, signal=23% |
| 1204 | GOMF\_DYNEIN\_COMPLEX\_BINDING |  | 17 | -0.52 | -1.39 | 0.079 | 0.384 | 1.000 | 5307 | tags=76%, list=44%, signal=137% |
| 1205 | GOBP\_INTERLEUKIN\_1\_ALPHA\_PRODUCTION |  | 6 | -0.68 | -1.39 | 0.074 | 0.385 | 1.000 | 1075 | tags=33%, list=9%, signal=37% |
| 1206 | GOBP\_ANTEROGRADE\_AXONAL\_TRANSPORT\_OF\_MITOCHONDRION |  | 5 | -0.72 | -1.39 | 0.068 | 0.385 | 1.000 | 1707 | tags=40%, list=14%, signal=47% |
| 1207 | GOBP\_HISTONE\_EXCHANGE |  | 38 | -0.44 | -1.39 | 0.062 | 0.385 | 1.000 | 4349 | tags=55%, list=36%, signal=86% |
| 1208 | GOBP\_EMBRYONIC\_SKELETAL\_SYSTEM\_MORPHOGENESIS |  | 50 | -0.42 | -1.39 | 0.048 | 0.385 | 1.000 | 2921 | tags=36%, list=24%, signal=47% |
| 1209 | GOBP\_SOMITE\_DEVELOPMENT |  | 54 | -0.41 | -1.39 | 0.050 | 0.386 | 1.000 | 4271 | tags=46%, list=36%, signal=72% |
| 1210 | GOBP\_REGULATION\_OF\_LUNG\_BLOOD\_PRESSURE |  | 3 | -0.83 | -1.39 | 0.049 | 0.386 | 1.000 | 2002 | tags=100%, list=17%, signal=120% |
| 1211 | GOBP\_HISTONE\_METHYLATION |  | 92 | -0.39 | -1.39 | 0.026 | 0.386 | 1.000 | 3647 | tags=47%, list=30%, signal=67% |
| 1212 | GOBP\_MICROTUBULE\_ORGANIZING\_CENTER\_ORGANIZATION |  | 112 | -0.38 | -1.39 | 0.021 | 0.387 | 1.000 | 4716 | tags=51%, list=39%, signal=83% |
| 1213 | GOMF\_GUANYLATE\_CYCLASE\_ACTIVITY |  | 3 | -0.83 | -1.38 | 0.049 | 0.387 | 1.000 | 1687 | tags=67%, list=14%, signal=78% |
| 1214 | GOBP\_REGULATION\_OF\_LEUKOCYTE\_CHEMOTAXIS |  | 78 | -0.40 | -1.38 | 0.034 | 0.387 | 1.000 | 3196 | tags=41%, list=27%, signal=56% |
| 1215 | GOBP\_TOLL\_LIKE\_RECEPTOR\_4\_SIGNALING\_PATHWAY |  | 30 | -0.46 | -1.38 | 0.069 | 0.387 | 1.000 | 2480 | tags=40%, list=21%, signal=50% |
| 1216 | GOBP\_POSITIVE\_REGULATION\_OF\_PROTEIN\_POLYMERIZATION |  | 88 | -0.39 | -1.38 | 0.029 | 0.387 | 1.000 | 3187 | tags=35%, list=27%, signal=48% |
| 1217 | GOMF\_TRANSMEMBRANE\_RECEPTOR\_PROTEIN\_KINASE\_ACTIVITY |  | 63 | -0.41 | -1.38 | 0.038 | 0.387 | 1.000 | 3657 | tags=38%, list=30%, signal=54% |
| 1218 | GOBP\_SLEEP |  | 17 | -0.52 | -1.38 | 0.080 | 0.388 | 1.000 | 2351 | tags=41%, list=20%, signal=51% |
| 1219 | GOMF\_CYTOSKELETAL\_PROTEIN\_BINDING |  | 693 | -0.35 | -1.38 | 0.000 | 0.388 | 1.000 | 3032 | tags=32%, list=25%, signal=40% |
| 1220 | GOMF\_SOLUTE\_CATION\_SYMPORTER\_ACTIVITY |  | 61 | -0.41 | -1.38 | 0.043 | 0.388 | 1.000 | 2395 | tags=38%, list=20%, signal=47% |
| 1221 | GOBP\_INTERLEUKIN\_35\_MEDIATED\_SIGNALING\_PATHWAY |  | 8 | -0.63 | -1.38 | 0.088 | 0.387 | 1.000 | 960 | tags=63%, list=8%, signal=68% |
| 1222 | GOBP\_RESPONSE\_TO\_FOOD |  | 18 | -0.51 | -1.38 | 0.087 | 0.388 | 1.000 | 3640 | tags=50%, list=30%, signal=72% |
| 1223 | GOBP\_REGULATION\_OF\_NEURON\_DIFFERENTIATION |  | 122 | -0.38 | -1.38 | 0.018 | 0.388 | 1.000 | 3654 | tags=41%, list=30%, signal=58% |
| 1224 | GOBP\_RESPONSE\_TO\_TRANSFORMING\_GROWTH\_FACTOR\_BETA |  | 183 | -0.37 | -1.38 | 0.007 | 0.387 | 1.000 | 3570 | tags=40%, list=30%, signal=56% |
| 1225 | GOMF\_BETA\_GALACTOSIDASE\_ACTIVITY |  | 4 | -0.77 | -1.38 | 0.064 | 0.387 | 1.000 | 2517 | tags=75%, list=21%, signal=95% |
| 1226 | GOBP\_HISTAMINE\_TRANSPORT |  | 9 | -0.61 | -1.38 | 0.090 | 0.387 | 1.000 | 2943 | tags=44%, list=25%, signal=59% |
| 1227 | GOMF\_PROTEIN\_KINASE\_B\_BINDING |  | 7 | -0.65 | -1.38 | 0.077 | 0.390 | 1.000 | 1914 | tags=57%, list=16%, signal=68% |
| 1228 | GOBP\_NEGATIVE\_REGULATION\_OF\_ALPHA\_BETA\_T\_CELL\_PROLIFERATION |  | 6 | -0.68 | -1.38 | 0.084 | 0.391 | 1.000 | 3344 | tags=83%, list=28%, signal=115% |
| 1229 | GOBP\_REGULATION\_OF\_BODY\_FLUID\_LEVELS |  | 318 | -0.36 | -1.38 | 0.001 | 0.391 | 1.000 | 3396 | tags=37%, list=28%, signal=51% |
| 1230 | GOBP\_PARAXIAL\_MESODERM\_MORPHOGENESIS |  | 5 | -0.72 | -1.38 | 0.072 | 0.392 | 1.000 | 2886 | tags=80%, list=24%, signal=105% |
| 1231 | GOBP\_CARTILAGE\_MORPHOGENESIS |  | 5 | -0.72 | -1.38 | 0.080 | 0.392 | 1.000 | 1783 | tags=60%, list=15%, signal=70% |
| 1232 | GOBP\_PRESYNAPTIC\_ENDOCYTOSIS |  | 40 | -0.43 | -1.38 | 0.061 | 0.391 | 1.000 | 3031 | tags=43%, list=25%, signal=57% |
| 1233 | GOBP\_AUTONOMIC\_NERVOUS\_SYSTEM\_DEVELOPMENT |  | 26 | -0.47 | -1.38 | 0.076 | 0.392 | 1.000 | 3186 | tags=46%, list=27%, signal=63% |
| 1234 | GOBP\_MEMBRANE\_DEPOLARIZATION\_DURING\_ACTION\_POTENTIAL |  | 20 | -0.50 | -1.38 | 0.089 | 0.391 | 1.000 | 1433 | tags=25%, list=12%, signal=28% |
| 1235 | GOBP\_VOCALIZATION\_BEHAVIOR |  | 11 | -0.57 | -1.38 | 0.097 | 0.391 | 1.000 | 4102 | tags=73%, list=34%, signal=110% |
| 1236 | GOBP\_EMBRYONIC\_NEUROCRANIUM\_MORPHOGENESIS |  | 7 | -0.65 | -1.38 | 0.075 | 0.391 | 1.000 | 4242 | tags=100%, list=35%, signal=155% |
| 1237 | GOBP\_MUSCLE\_CELL\_FATE\_COMMITMENT |  | 6 | -0.68 | -1.38 | 0.072 | 0.391 | 1.000 | 2474 | tags=67%, list=21%, signal=84% |
| 1238 | GOBP\_PROTEIN\_LOCALIZATION\_TO\_SYNAPSE |  | 49 | -0.42 | -1.38 | 0.061 | 0.391 | 1.000 | 4655 | tags=57%, list=39%, signal=93% |
| 1239 | GOBP\_DETECTION\_OF\_MECHANICAL\_STIMULUS\_INVOLVED\_IN\_SENSORY\_PERCEPTION\_OF\_SOUND |  | 4 | -0.77 | -1.38 | 0.060 | 0.391 | 1.000 | 199 | tags=25%, list=2%, signal=25% |
| 1240 | GOBP\_RETINAL\_BLOOD\_VESSEL\_MORPHOGENESIS |  | 5 | -0.72 | -1.38 | 0.071 | 0.391 | 1.000 | 2333 | tags=60%, list=19%, signal=74% |
| 1241 | GOBP\_LEARNING |  | 82 | -0.39 | -1.38 | 0.032 | 0.391 | 1.000 | 2631 | tags=33%, list=22%, signal=42% |
| 1242 | GOBP\_SYNAPTIC\_SIGNALING |  | 398 | -0.35 | -1.38 | 0.000 | 0.390 | 1.000 | 3946 | tags=41%, list=33%, signal=60% |
| 1243 | GOBP\_PRIMARY\_ALCOHOL\_METABOLIC\_PROCESS |  | 54 | -0.42 | -1.38 | 0.054 | 0.390 | 1.000 | 2462 | tags=30%, list=20%, signal=37% |
| 1244 | GOBP\_POSITIVE\_REGULATION\_OF\_TELOMERE\_MAINTENANCE |  | 42 | -0.43 | -1.38 | 0.059 | 0.390 | 1.000 | 1851 | tags=29%, list=15%, signal=34% |
| 1245 | GOBP\_VASCULAR\_ENDOTHELIAL\_GROWTH\_FACTOR\_SIGNALING\_PATHWAY |  | 33 | -0.45 | -1.38 | 0.069 | 0.390 | 1.000 | 3724 | tags=52%, list=31%, signal=74% |
| 1246 | GOBP\_NEGATIVE\_REGULATION\_OF\_RESPONSE\_TO\_FOOD |  | 7 | -0.65 | -1.38 | 0.083 | 0.390 | 1.000 | 1748 | tags=43%, list=15%, signal=50% |
| 1247 | GOBP\_TRNA\_TRANSPORT |  | 32 | -0.45 | -1.38 | 0.064 | 0.389 | 1.000 | 4080 | tags=56%, list=34%, signal=85% |
| 1248 | GOBP\_REGULATION\_OF\_MYOTUBE\_DIFFERENTIATION |  | 19 | -0.50 | -1.38 | 0.089 | 0.390 | 1.000 | 3993 | tags=53%, list=33%, signal=79% |
| 1249 | GOBP\_STEROL\_TRANSPORT |  | 73 | -0.40 | -1.38 | 0.040 | 0.391 | 1.000 | 3789 | tags=45%, list=32%, signal=66% |
| 1250 | GOBP\_POSITIVE\_REGULATION\_OF\_CELL\_DIFFERENTIATION |  | 551 | -0.35 | -1.38 | 0.000 | 0.390 | 1.000 | 3658 | tags=38%, list=30%, signal=53% |
| 1251 | GOBP\_POSITIVE\_REGULATION\_OF\_AXON\_EXTENSION |  | 32 | -0.45 | -1.38 | 0.065 | 0.390 | 1.000 | 4617 | tags=56%, list=38%, signal=91% |
| 1252 | GOBP\_CIRCADIAN\_SLEEP\_WAKE\_CYCLE\_SLEEP |  | 10 | -0.59 | -1.38 | 0.089 | 0.390 | 1.000 | 2351 | tags=50%, list=20%, signal=62% |
| 1253 | GOBP\_BILE\_ACID\_SIGNALING\_PATHWAY |  | 9 | -0.61 | -1.38 | 0.082 | 0.390 | 1.000 | 1109 | tags=44%, list=9%, signal=49% |
| 1254 | GOBP\_SEQUESTERING\_OF\_CALCIUM\_ION |  | 80 | -0.39 | -1.38 | 0.034 | 0.390 | 1.000 | 3313 | tags=36%, list=28%, signal=50% |
| 1255 | GOBP\_CALCIUM\_ION\_TRANSMEMBRANE\_TRANSPORT\_VIA\_HIGH\_VOLTAGE\_GATED\_CALCIUM\_CHANNEL |  | 10 | -0.59 | -1.38 | 0.085 | 0.390 | 1.000 | 1597 | tags=30%, list=13%, signal=35% |
| 1256 | GOMF\_ACETYLGLUCOSAMINYLTRANSFERASE\_ACTIVITY |  | 39 | -0.44 | -1.38 | 0.066 | 0.390 | 1.000 | 2047 | tags=31%, list=17%, signal=37% |
| 1257 | GOBP\_DOUBLE\_STRAND\_BREAK\_REPAIR |  | 184 | -0.36 | -1.38 | 0.009 | 0.390 | 1.000 | 4773 | tags=54%, list=40%, signal=89% |
| 1258 | GOMF\_CYTOKINE\_RECEPTOR\_BINDING |  | 124 | -0.37 | -1.38 | 0.017 | 0.390 | 1.000 | 2894 | tags=37%, list=24%, signal=48% |
| 1259 | GOBP\_POSITIVE\_CHEMOTAXIS |  | 42 | -0.43 | -1.38 | 0.061 | 0.389 | 1.000 | 3439 | tags=45%, list=29%, signal=63% |
| 1260 | GOBP\_ORGANIC\_CATION\_TRANSPORT |  | 22 | -0.49 | -1.38 | 0.078 | 0.390 | 1.000 | 2557 | tags=45%, list=21%, signal=58% |
| 1261 | GOBP\_PHOSPHOLIPID\_TRANSPORT |  | 61 | -0.41 | -1.38 | 0.043 | 0.390 | 1.000 | 1410 | tags=26%, list=12%, signal=30% |
| 1262 | GOBP\_POSITIVE\_REGULATION\_OF\_DENDRITIC\_CELL\_ANTIGEN\_PROCESSING\_AND\_PRESENTATION |  | 5 | -0.72 | -1.38 | 0.080 | 0.390 | 1.000 | 2847 | tags=40%, list=24%, signal=52% |
| 1263 | GOBP\_NEGATIVE\_REGULATION\_OF\_LEUKOCYTE\_MEDIATED\_IMMUNITY |  | 21 | -0.49 | -1.38 | 0.083 | 0.389 | 1.000 | 2249 | tags=43%, list=19%, signal=53% |
| 1264 | GOBP\_LYMPHOCYTE\_ANERGY |  | 3 | -0.82 | -1.38 | 0.061 | 0.390 | 1.000 | 1016 | tags=67%, list=8%, signal=73% |
| 1265 | GOBP\_POSITIVE\_REGULATION\_OF\_MULTICELLULAR\_ORGANISM\_GROWTH |  | 17 | -0.52 | -1.38 | 0.086 | 0.390 | 1.000 | 2362 | tags=29%, list=20%, signal=37% |
| 1266 | GOBP\_POSITIVE\_REGULATION\_OF\_CELL\_MATRIX\_ADHESION |  | 43 | -0.43 | -1.38 | 0.059 | 0.390 | 1.000 | 3196 | tags=37%, list=27%, signal=51% |
| 1267 | GOBP\_REGULATION\_OF\_CARDIAC\_MUSCLE\_CELL\_ACTION\_POTENTIAL\_INVOLVED\_IN\_REGULATION\_OF\_CONTRACTION |  | 5 | -0.72 | -1.38 | 0.078 | 0.390 | 1.000 | 1304 | tags=40%, list=11%, signal=45% |
| 1268 | GOBP\_T\_CELL\_DIFFERENTIATION\_INVOLVED\_IN\_IMMUNE\_RESPONSE |  | 44 | -0.43 | -1.38 | 0.061 | 0.389 | 1.000 | 2912 | tags=43%, list=24%, signal=57% |
| 1269 | GOCC\_SPANNING\_COMPONENT\_OF\_PLASMA\_MEMBRANE |  | 6 | -0.68 | -1.38 | 0.081 | 0.390 | 1.000 | 933 | tags=50%, list=8%, signal=54% |
| 1270 | GOBP\_REGULATION\_OF\_TELOMERE\_MAINTENANCE\_VIA\_TELOMERE\_LENGTHENING |  | 50 | -0.42 | -1.38 | 0.057 | 0.390 | 1.000 | 4922 | tags=58%, list=41%, signal=98% |
| 1271 | GOBP\_REGULATION\_OF\_RESPONSE\_TO\_EXTRACELLULAR\_STIMULUS |  | 15 | -0.53 | -1.38 | 0.091 | 0.390 | 1.000 | 4358 | tags=60%, list=36%, signal=94% |
| 1272 | GOBP\_CD8\_POSITIVE\_ALPHA\_BETA\_T\_CELL\_DIFFERENTIATION |  | 10 | -0.59 | -1.38 | 0.086 | 0.390 | 1.000 | 1601 | tags=40%, list=13%, signal=46% |
| 1273 | GOBP\_INTERLEUKIN\_23\_PRODUCTION |  | 4 | -0.76 | -1.38 | 0.064 | 0.390 | 1.000 | 2322 | tags=75%, list=19%, signal=93% |
| 1274 | GOBP\_ESTABLISHMENT\_OF\_MITOTIC\_SPINDLE\_ORIENTATION |  | 25 | -0.48 | -1.38 | 0.077 | 0.390 | 1.000 | 3834 | tags=48%, list=32%, signal=70% |
| 1275 | GOBP\_MYELOID\_CELL\_DIFFERENTIATION |  | 277 | -0.36 | -1.38 | 0.002 | 0.391 | 1.000 | 3692 | tags=42%, list=31%, signal=60% |
| 1276 | GOBP\_LEUKOCYTE\_CHEMOTAXIS |  | 132 | -0.37 | -1.38 | 0.018 | 0.391 | 1.000 | 3212 | tags=40%, list=27%, signal=54% |
| 1277 | GOBP\_GLYCEROPHOSPHOLIPID\_METABOLIC\_PROCESS |  | 260 | -0.36 | -1.38 | 0.002 | 0.390 | 1.000 | 4057 | tags=43%, list=34%, signal=64% |
| 1278 | GOMF\_MOLECULAR\_ADAPTOR\_ACTIVITY |  | 247 | -0.36 | -1.38 | 0.004 | 0.391 | 1.000 | 3825 | tags=40%, list=32%, signal=57% |
| 1279 | GOMF\_METALLOAMINOPEPTIDASE\_ACTIVITY |  | 21 | -0.49 | -1.38 | 0.083 | 0.391 | 1.000 | 2881 | tags=33%, list=24%, signal=44% |
| 1280 | GOBP\_CGMP\_BIOSYNTHETIC\_PROCESS |  | 3 | -0.83 | -1.38 | 0.057 | 0.391 | 1.000 | 1687 | tags=67%, list=14%, signal=78% |
| 1281 | GOCC\_CORTICAL\_ENDOPLASMIC\_RETICULUM |  | 4 | -0.76 | -1.38 | 0.074 | 0.391 | 1.000 | 846 | tags=25%, list=7%, signal=27% |
| 1282 | GOBP\_POSITIVE\_REGULATION\_OF\_TRANSCRIPTION\_BY\_RNA\_POLYMERASE\_II |  | 743 | -0.35 | -1.38 | 0.000 | 0.391 | 1.000 | 4001 | tags=42%, list=33%, signal=59% |
| 1283 | GOBP\_REGULATION\_OF\_FC\_RECEPTOR\_MEDIATED\_STIMULATORY\_SIGNALING\_PATHWAY |  | 11 | -0.57 | -1.38 | 0.093 | 0.391 | 1.000 | 3062 | tags=55%, list=25%, signal=73% |
| 1284 | GOCC\_ASTER |  | 10 | -0.58 | -1.38 | 0.089 | 0.390 | 1.000 | 1540 | tags=30%, list=13%, signal=34% |
| 1285 | GOMF\_SEMAPHORIN\_RECEPTOR\_BINDING |  | 17 | -0.51 | -1.38 | 0.093 | 0.391 | 1.000 | 1180 | tags=29%, list=10%, signal=33% |
| 1286 | GOMF\_TYPE\_5\_METABOTROPIC\_GLUTAMATE\_RECEPTOR\_BINDING |  | 4 | -0.76 | -1.38 | 0.071 | 0.391 | 1.000 | 623 | tags=50%, list=5%, signal=53% |
| 1287 | GOBP\_LEUKOCYTE\_ADHESION\_TO\_ARTERIAL\_ENDOTHELIAL\_CELL |  | 4 | -0.76 | -1.38 | 0.066 | 0.391 | 1.000 | 2258 | tags=75%, list=19%, signal=92% |
| 1288 | GOBP\_MITOTIC\_DNA\_REPLICATION |  | 14 | -0.54 | -1.38 | 0.087 | 0.391 | 1.000 | 4233 | tags=71%, list=35%, signal=110% |
| 1289 | GOMF\_GAP\_JUNCTION\_CHANNEL\_ACTIVITY\_INVOLVED\_IN\_CELL\_COMMUNICATION\_BY\_ELECTRICAL\_COUPLING |  | 5 | -0.71 | -1.38 | 0.075 | 0.390 | 1.000 | 2313 | tags=80%, list=19%, signal=99% |
| 1290 | GOCC\_NUCLEAR\_INNER\_MEMBRANE |  | 43 | -0.43 | -1.38 | 0.062 | 0.391 | 1.000 | 4672 | tags=58%, list=39%, signal=95% |
| 1291 | GOBP\_POSITIVE\_REGULATION\_OF\_ACTIN\_FILAMENT\_DEPOLYMERIZATION |  | 9 | -0.60 | -1.38 | 0.087 | 0.391 | 1.000 | 2480 | tags=33%, list=21%, signal=42% |
| 1292 | GOBP\_POSITIVE\_REGULATION\_OF\_LONG\_TERM\_NEURONAL\_SYNAPTIC\_PLASTICITY |  | 5 | -0.71 | -1.38 | 0.083 | 0.391 | 1.000 | 2466 | tags=60%, list=21%, signal=75% |
| 1293 | GOBP\_CARDIAC\_NEURAL\_CREST\_CELL\_DEVELOPMENT\_INVOLVED\_IN\_OUTFLOW\_TRACT\_MORPHOGENESIS |  | 8 | -0.62 | -1.38 | 0.091 | 0.391 | 1.000 | 480 | tags=25%, list=4%, signal=26% |
| 1294 | GOBP\_POSITIVE\_REGULATION\_OF\_NITRIC\_OXIDE\_METABOLIC\_PROCESS |  | 25 | -0.47 | -1.38 | 0.083 | 0.390 | 1.000 | 2010 | tags=32%, list=17%, signal=38% |
| 1295 | GOBP\_PROSTHETIC\_GROUP\_METABOLIC\_PROCESS |  | 6 | -0.68 | -1.38 | 0.083 | 0.390 | 1.000 | 282 | tags=33%, list=2%, signal=34% |
| 1296 | GOBP\_REGULATION\_OF\_RAC\_PROTEIN\_SIGNAL\_TRANSDUCTION |  | 16 | -0.52 | -1.38 | 0.082 | 0.390 | 1.000 | 3534 | tags=63%, list=29%, signal=88% |
| 1297 | GOBP\_ENDOCARDIUM\_DEVELOPMENT |  | 9 | -0.60 | -1.37 | 0.089 | 0.391 | 1.000 | 2212 | tags=44%, list=18%, signal=54% |
| 1298 | GOCC\_SOMATODENDRITIC\_COMPARTMENT |  | 540 | -0.35 | -1.37 | 0.000 | 0.391 | 1.000 | 3502 | tags=37%, list=29%, signal=49% |
| 1299 | GOBP\_POSITIVE\_REGULATION\_OF\_CELLULAR\_COMPONENT\_BIOGENESIS |  | 369 | -0.35 | -1.37 | 0.001 | 0.390 | 1.000 | 3096 | tags=33%, list=26%, signal=42% |
| 1300 | GOBP\_LEUKOCYTE\_MEDIATED\_CYTOTOXICITY |  | 42 | -0.43 | -1.37 | 0.062 | 0.391 | 1.000 | 2547 | tags=36%, list=21%, signal=45% |
| 1301 | GOBP\_ACTIN\_FILAMENT\_NETWORK\_FORMATION |  | 11 | -0.57 | -1.37 | 0.087 | 0.391 | 1.000 | 2046 | tags=36%, list=17%, signal=44% |
| 1302 | GOBP\_SOMATIC\_DIVERSIFICATION\_OF\_T\_CELL\_RECEPTOR\_GENES |  | 5 | -0.71 | -1.37 | 0.079 | 0.392 | 1.000 | 922 | tags=60%, list=8%, signal=65% |
| 1303 | GOBP\_ANATOMICAL\_STRUCTURE\_FORMATION\_INVOLVED\_IN\_MORPHOGENESIS |  | 706 | -0.35 | -1.37 | 0.000 | 0.392 | 1.000 | 3619 | tags=37%, list=30%, signal=50% |
| 1304 | GOBP\_REGULATION\_OF\_SYNAPSE\_MATURATION |  | 10 | -0.59 | -1.37 | 0.089 | 0.392 | 1.000 | 3431 | tags=40%, list=29%, signal=56% |
| 1305 | GOBP\_NEGATIVE\_REGULATION\_OF\_DNA\_REPAIR |  | 23 | -0.47 | -1.37 | 0.081 | 0.392 | 1.000 | 2379 | tags=43%, list=20%, signal=54% |
| 1306 | GOBP\_POSITIVE\_REGULATION\_OF\_LEUKOCYTE\_MIGRATION |  | 90 | -0.38 | -1.37 | 0.035 | 0.392 | 1.000 | 3196 | tags=38%, list=27%, signal=51% |
| 1307 | GOBP\_ACTIVATION\_OF\_IMMUNE\_RESPONSE |  | 276 | -0.36 | -1.37 | 0.002 | 0.392 | 1.000 | 3165 | tags=35%, list=26%, signal=46% |
| 1308 | GOMF\_N\_ACETYLTRANSFERASE\_ACTIVITY |  | 49 | -0.42 | -1.37 | 0.061 | 0.392 | 1.000 | 3083 | tags=39%, list=26%, signal=52% |
| 1309 | GOBP\_UBIQUITIN\_DEPENDENT\_GLYCOPROTEIN\_ERAD\_PATHWAY |  | 5 | -0.72 | -1.37 | 0.074 | 0.393 | 1.000 | 2715 | tags=80%, list=23%, signal=103% |
| 1310 | GOBP\_POSITIVE\_REGULATION\_OF\_NEUROBLAST\_PROLIFERATION |  | 16 | -0.52 | -1.37 | 0.083 | 0.393 | 1.000 | 3565 | tags=50%, list=30%, signal=71% |
| 1311 | GOBP\_MEGAKARYOCYTE\_DEVELOPMENT |  | 13 | -0.55 | -1.37 | 0.094 | 0.393 | 1.000 | 2546 | tags=54%, list=21%, signal=68% |
| 1312 | GOMF\_ENZYME\_ACTIVATOR\_ACTIVITY |  | 383 | -0.35 | -1.37 | 0.000 | 0.393 | 1.000 | 4324 | tags=45%, list=36%, signal=68% |
| 1313 | GOCC\_SUPER\_ELONGATION\_COMPLEX |  | 5 | -0.71 | -1.37 | 0.073 | 0.393 | 1.000 | 1118 | tags=60%, list=9%, signal=66% |
| 1314 | GOCC\_ORGANELLE\_MEMBRANE\_CONTACT\_SITE |  | 31 | -0.45 | -1.37 | 0.076 | 0.393 | 1.000 | 3600 | tags=58%, list=30%, signal=83% |
| 1315 | GOMF\_ENZYME\_REGULATOR\_ACTIVITY |  | 822 | -0.34 | -1.37 | 0.000 | 0.393 | 1.000 | 3590 | tags=36%, list=30%, signal=48% |
| 1316 | GOCC\_MICROTUBULE\_MINUS\_END |  | 5 | -0.71 | -1.37 | 0.073 | 0.393 | 1.000 | 2927 | tags=60%, list=24%, signal=79% |
| 1317 | GOBP\_POSITIVE\_REGULATION\_OF\_FIBROBLAST\_MIGRATION |  | 14 | -0.54 | -1.37 | 0.094 | 0.393 | 1.000 | 3613 | tags=57%, list=30%, signal=82% |
| 1318 | GOBP\_REGULATION\_OF\_NERVOUS\_SYSTEM\_DEVELOPMENT |  | 290 | -0.35 | -1.37 | 0.001 | 0.392 | 1.000 | 3439 | tags=34%, list=29%, signal=47% |
| 1319 | GOBP\_SPONTANEOUS\_NEUROTRANSMITTER\_SECRETION |  | 3 | -0.83 | -1.37 | 0.060 | 0.392 | 1.000 | 2087 | tags=100%, list=17%, signal=121% |
| 1320 | GOBP\_REGULATION\_OF\_MONONUCLEAR\_CELL\_MIGRATION |  | 70 | -0.39 | -1.37 | 0.042 | 0.392 | 1.000 | 3093 | tags=39%, list=26%, signal=52% |
| 1321 | GOMF\_FERROUS\_IRON\_BINDING |  | 20 | -0.50 | -1.37 | 0.084 | 0.392 | 1.000 | 2803 | tags=50%, list=23%, signal=65% |
| 1322 | GOBP\_ACTIVATION\_OF\_GTPASE\_ACTIVITY |  | 85 | -0.39 | -1.37 | 0.041 | 0.391 | 1.000 | 3576 | tags=44%, list=30%, signal=62% |
| 1323 | GOBP\_REGULATION\_OF\_CELL\_MORPHOGENESIS |  | 225 | -0.36 | -1.37 | 0.005 | 0.392 | 1.000 | 4152 | tags=44%, list=35%, signal=65% |
| 1324 | GOBP\_BILE\_ACID\_BIOSYNTHETIC\_PROCESS |  | 30 | -0.46 | -1.37 | 0.077 | 0.392 | 1.000 | 4053 | tags=47%, list=34%, signal=70% |
| 1325 | GOBP\_CELL\_SUBSTRATE\_ADHESION |  | 259 | -0.36 | -1.37 | 0.004 | 0.392 | 1.000 | 3575 | tags=37%, list=30%, signal=52% |
| 1326 | GOMF\_PROTEIN\_MACROMOLECULE\_ADAPTOR\_ACTIVITY |  | 199 | -0.36 | -1.37 | 0.007 | 0.392 | 1.000 | 3825 | tags=41%, list=32%, signal=59% |
| 1327 | GOBP\_T\_CELL\_MIGRATION |  | 39 | -0.43 | -1.37 | 0.069 | 0.392 | 1.000 | 2955 | tags=44%, list=25%, signal=58% |
| 1328 | GOBP\_REGULATION\_OF\_HISTONE\_MODIFICATION |  | 102 | -0.38 | -1.37 | 0.029 | 0.392 | 1.000 | 3644 | tags=45%, list=30%, signal=64% |
| 1329 | GOBP\_REGULATION\_OF\_PHOSPHOLIPASE\_C\_ACTIVITY |  | 33 | -0.45 | -1.37 | 0.071 | 0.392 | 1.000 | 1411 | tags=24%, list=12%, signal=27% |
| 1330 | GOBP\_LIPID\_EXPORT\_FROM\_CELL |  | 57 | -0.41 | -1.37 | 0.050 | 0.392 | 1.000 | 2367 | tags=35%, list=20%, signal=43% |
| 1331 | GOBP\_RESPONSE\_TO\_INTERLEUKIN\_15 |  | 13 | -0.54 | -1.37 | 0.090 | 0.392 | 1.000 | 960 | tags=31%, list=8%, signal=33% |
| 1332 | GOBP\_REGULATION\_OF\_CELL\_PROJECTION\_ASSEMBLY |  | 134 | -0.37 | -1.37 | 0.017 | 0.393 | 1.000 | 4601 | tags=46%, list=38%, signal=74% |
| 1333 | GOBP\_PEPTIDYL\_THREONINE\_MODIFICATION |  | 90 | -0.39 | -1.37 | 0.034 | 0.392 | 1.000 | 4702 | tags=54%, list=39%, signal=89% |
| 1334 | GOBP\_RETINA\_VASCULATURE\_DEVELOPMENT\_IN\_CAMERA\_TYPE\_EYE |  | 14 | -0.54 | -1.37 | 0.090 | 0.392 | 1.000 | 5077 | tags=79%, list=42%, signal=136% |
| 1335 | GOBP\_MORPHOGENESIS\_OF\_AN\_EPITHELIAL\_SHEET |  | 44 | -0.43 | -1.37 | 0.061 | 0.392 | 1.000 | 1743 | tags=27%, list=15%, signal=32% |
| 1336 | GOCC\_ATPASE\_COMPLEX |  | 76 | -0.39 | -1.37 | 0.039 | 0.392 | 1.000 | 4349 | tags=49%, list=36%, signal=76% |
| 1337 | GOMF\_DNA\_BINDING\_TRANSCRIPTION\_FACTOR\_ACTIVITY |  | 541 | -0.35 | -1.37 | 0.000 | 0.392 | 1.000 | 3907 | tags=43%, list=33%, signal=61% |
| 1338 | GOBP\_P\_BODY\_ASSEMBLY |  | 19 | -0.50 | -1.37 | 0.094 | 0.392 | 1.000 | 3620 | tags=47%, list=30%, signal=68% |
| 1339 | GOCC\_APICAL\_JUNCTION\_COMPLEX |  | 97 | -0.38 | -1.37 | 0.034 | 0.392 | 1.000 | 2970 | tags=35%, list=25%, signal=46% |
| 1340 | GOCC\_MITOTIC\_SPINDLE\_ASTRAL\_MICROTUBULE |  | 7 | -0.65 | -1.37 | 0.086 | 0.392 | 1.000 | 1540 | tags=29%, list=13%, signal=33% |
| 1341 | GOBP\_REGULATION\_OF\_HYDROLASE\_ACTIVITY |  | 847 | -0.34 | -1.37 | 0.000 | 0.392 | 1.000 | 3547 | tags=37%, list=30%, signal=48% |
| 1342 | GOBP\_POSITIVE\_REGULATION\_OF\_HISTONE\_H4\_ACETYLATION |  | 4 | -0.76 | -1.37 | 0.070 | 0.392 | 1.000 | 2798 | tags=75%, list=23%, signal=98% |
| 1343 | GOBP\_POSITIVE\_REGULATION\_OF\_ALPHA\_BETA\_T\_CELL\_DIFFERENTIATION |  | 31 | -0.45 | -1.37 | 0.074 | 0.392 | 1.000 | 2408 | tags=42%, list=20%, signal=52% |
| 1344 | GOMF\_VOLTAGE\_GATED\_SODIUM\_CHANNEL\_ACTIVITY |  | 10 | -0.58 | -1.37 | 0.096 | 0.392 | 1.000 | 748 | tags=30%, list=6%, signal=32% |
| 1345 | GOBP\_POSITIVE\_REGULATION\_OF\_KERATINOCYTE\_PROLIFERATION |  | 9 | -0.60 | -1.37 | 0.103 | 0.392 | 1.000 | 1777 | tags=56%, list=15%, signal=65% |
| 1346 | GOBP\_CELLULAR\_RESPONSE\_TO\_VASCULAR\_ENDOTHELIAL\_GROWTH\_FACTOR\_STIMULUS |  | 51 | -0.41 | -1.37 | 0.060 | 0.392 | 1.000 | 3724 | tags=49%, list=31%, signal=71% |
| 1347 | GOBP\_POSITIVE\_REGULATION\_OF\_PROTEIN\_LOCALIZATION\_TO\_CELL\_SURFACE |  | 8 | -0.62 | -1.37 | 0.095 | 0.392 | 1.000 | 3613 | tags=63%, list=30%, signal=89% |
| 1348 | GOBP\_RESPONSE\_TO\_GROWTH\_FACTOR |  | 501 | -0.35 | -1.37 | 0.000 | 0.393 | 1.000 | 3809 | tags=40%, list=32%, signal=55% |
| 1349 | GOBP\_POSITIVE\_REGULATION\_OF\_LAMELLIPODIUM\_MORPHOGENESIS |  | 6 | -0.67 | -1.37 | 0.084 | 0.393 | 1.000 | 591 | tags=33%, list=5%, signal=35% |
| 1350 | GOBP\_HISTONE\_H2A\_ACETYLATION |  | 13 | -0.54 | -1.37 | 0.099 | 0.393 | 1.000 | 3279 | tags=46%, list=27%, signal=63% |
| 1351 | GOMF\_HYDROLASE\_ACTIVITY\_HYDROLYZING\_N\_GLYCOSYL\_COMPOUNDS |  | 25 | -0.47 | -1.37 | 0.082 | 0.392 | 1.000 | 3487 | tags=40%, list=29%, signal=56% |
| 1352 | GOBP\_POSITIVE\_REGULATION\_OF\_MYELOID\_LEUKOCYTE\_CYTOKINE\_PRODUCTION\_INVOLVED\_IN\_IMMUNE\_RESPONSE |  | 13 | -0.55 | -1.37 | 0.091 | 0.393 | 1.000 | 4686 | tags=77%, list=39%, signal=126% |
| 1353 | GOBP\_SENSORY\_ORGAN\_MORPHOGENESIS |  | 139 | -0.37 | -1.37 | 0.015 | 0.393 | 1.000 | 1692 | tags=24%, list=14%, signal=27% |
| 1354 | GOMF\_INTEGRIN\_BINDING |  | 102 | -0.38 | -1.37 | 0.028 | 0.393 | 1.000 | 2879 | tags=31%, list=24%, signal=41% |
| 1355 | GOBP\_POSITIVE\_REGULATION\_OF\_NF\_KAPPAB\_TRANSCRIPTION\_FACTOR\_ACTIVITY |  | 104 | -0.38 | -1.37 | 0.030 | 0.393 | 1.000 | 3557 | tags=40%, list=30%, signal=57% |
| 1356 | GOBP\_NEURAL\_PLATE\_MORPHOGENESIS |  | 3 | -0.82 | -1.37 | 0.064 | 0.393 | 1.000 | 1764 | tags=67%, list=15%, signal=78% |
| 1357 | GOBP\_EOSINOPHIL\_MIGRATION |  | 11 | -0.57 | -1.37 | 0.099 | 0.393 | 1.000 | 1038 | tags=36%, list=9%, signal=40% |
| 1358 | GOBP\_PROTEIN\_DEMANNOSYLATION |  | 16 | -0.52 | -1.37 | 0.092 | 0.393 | 1.000 | 2715 | tags=44%, list=23%, signal=56% |
| 1359 | GOBP\_PHAGOCYTOSIS |  | 192 | -0.36 | -1.37 | 0.008 | 0.393 | 1.000 | 3130 | tags=33%, list=26%, signal=44% |
| 1360 | GOBP\_POSITIVE\_REGULATION\_OF\_DNA\_BINDING\_TRANSCRIPTION\_FACTOR\_ACTIVITY |  | 171 | -0.36 | -1.37 | 0.009 | 0.393 | 1.000 | 3613 | tags=39%, list=30%, signal=55% |
| 1361 | GOBP\_POSITIVE\_REGULATION\_OF\_CELL\_KILLING |  | 23 | -0.47 | -1.37 | 0.084 | 0.393 | 1.000 | 2547 | tags=43%, list=21%, signal=55% |
| 1362 | GOBP\_PROTEIN\_LOCALIZATION\_TO\_CHROMOSOME\_TELOMERIC\_REGION |  | 25 | -0.47 | -1.37 | 0.087 | 0.394 | 1.000 | 1704 | tags=24%, list=14%, signal=28% |
| 1363 | GOBP\_EMBRYONIC\_SKELETAL\_SYSTEM\_DEVELOPMENT |  | 69 | -0.40 | -1.37 | 0.045 | 0.393 | 1.000 | 2921 | tags=36%, list=24%, signal=48% |
| 1364 | GOBP\_RESPONSE\_TO\_INACTIVITY |  | 7 | -0.64 | -1.37 | 0.090 | 0.394 | 1.000 | 3295 | tags=71%, list=27%, signal=98% |
| 1365 | GOCC\_SPINDLE\_POLE |  | 132 | -0.37 | -1.37 | 0.021 | 0.394 | 1.000 | 4983 | tags=52%, list=41%, signal=88% |
| 1366 | GOBP\_INSULIN\_METABOLIC\_PROCESS |  | 3 | -0.82 | -1.37 | 0.061 | 0.394 | 1.000 | 2218 | tags=100%, list=18%, signal=123% |
| 1367 | GOMF\_PHOSPHORYLATION\_DEPENDENT\_PROTEIN\_BINDING |  | 4 | -0.76 | -1.37 | 0.078 | 0.395 | 1.000 | 2908 | tags=100%, list=24%, signal=132% |
| 1368 | GOBP\_REGULATION\_OF\_DNA\_TEMPLATED\_TRANSCRIPTION\_INITIATION |  | 36 | -0.44 | -1.37 | 0.069 | 0.395 | 1.000 | 4990 | tags=61%, list=42%, signal=104% |
| 1369 | GOBP\_GASTRO\_INTESTINAL\_SYSTEM\_SMOOTH\_MUSCLE\_CONTRACTION |  | 9 | -0.60 | -1.37 | 0.095 | 0.395 | 1.000 | 866 | tags=33%, list=7%, signal=36% |
| 1370 | GOBP\_ALDITOL\_CATABOLIC\_PROCESS |  | 6 | -0.67 | -1.37 | 0.089 | 0.395 | 1.000 | 1531 | tags=33%, list=13%, signal=38% |
| 1371 | GOBP\_NEGATIVE\_REGULATION\_OF\_CELL\_DIVISION |  | 11 | -0.56 | -1.37 | 0.096 | 0.395 | 1.000 | 3214 | tags=55%, list=27%, signal=74% |
| 1372 | GOBP\_REGULATION\_OF\_CD4\_POSITIVE\_ALPHA\_BETA\_T\_CELL\_ACTIVATION |  | 41 | -0.43 | -1.37 | 0.069 | 0.395 | 1.000 | 1951 | tags=34%, list=16%, signal=41% |
| 1373 | GOMF\_GPI\_LINKED\_EPHRIN\_RECEPTOR\_ACTIVITY |  | 4 | -0.76 | -1.37 | 0.074 | 0.396 | 1.000 | 1250 | tags=25%, list=10%, signal=28% |
| 1374 | GOCC\_POLYMERIC\_CYTOSKELETAL\_FIBER |  | 438 | -0.35 | -1.37 | 0.001 | 0.395 | 1.000 | 3837 | tags=37%, list=32%, signal=52% |
| 1375 | GOBP\_MYOBLAST\_DIFFERENTIATION |  | 63 | -0.40 | -1.37 | 0.050 | 0.395 | 1.000 | 2474 | tags=32%, list=21%, signal=40% |
| 1376 | GOBP\_ACTIN\_POLYMERIZATION\_OR\_DEPOLYMERIZATION |  | 150 | -0.37 | -1.37 | 0.016 | 0.396 | 1.000 | 3032 | tags=33%, list=25%, signal=43% |
| 1377 | GOBP\_NEGATIVE\_REGULATION\_OF\_TRANSFORMING\_GROWTH\_FACTOR\_BETA\_RECEPTOR\_SIGNALING\_PATHWAY |  | 57 | -0.40 | -1.37 | 0.059 | 0.396 | 1.000 | 4105 | tags=49%, list=34%, signal=74% |
| 1378 | GOBP\_POSITIVE\_REGULATION\_OF\_HISTONE\_H3\_K4\_METHYLATION |  | 15 | -0.53 | -1.37 | 0.094 | 0.396 | 1.000 | 4698 | tags=80%, list=39%, signal=131% |
| 1379 | GOBP\_MORPHOGENESIS\_OF\_EMBRYONIC\_EPITHELIUM |  | 99 | -0.38 | -1.37 | 0.030 | 0.396 | 1.000 | 3565 | tags=35%, list=30%, signal=50% |
| 1380 | GOMF\_INTRACELLULAR\_CALCIUM\_ACTIVATED\_CHLORIDE\_CHANNEL\_ACTIVITY |  | 9 | -0.59 | -1.37 | 0.103 | 0.396 | 1.000 | 3141 | tags=56%, list=26%, signal=75% |
| 1381 | GOBP\_PERIPHERAL\_NERVOUS\_SYSTEM\_AXON\_REGENERATION |  | 6 | -0.67 | -1.36 | 0.091 | 0.396 | 1.000 | 1308 | tags=33%, list=11%, signal=37% |
| 1382 | GOBP\_PRIMARY\_AMINO\_COMPOUND\_METABOLIC\_PROCESS |  | 9 | -0.59 | -1.36 | 0.104 | 0.396 | 1.000 | 3653 | tags=56%, list=30%, signal=80% |
| 1383 | GOBP\_ERYTHROCYTE\_HOMEOSTASIS |  | 95 | -0.38 | -1.36 | 0.037 | 0.397 | 1.000 | 4368 | tags=53%, list=36%, signal=82% |
| 1384 | GOBP\_POSITIVE\_REGULATION\_OF\_CARTILAGE\_DEVELOPMENT |  | 19 | -0.50 | -1.36 | 0.093 | 0.397 | 1.000 | 2002 | tags=47%, list=17%, signal=57% |
| 1385 | GOCC\_APICAL\_PLASMA\_MEMBRANE |  | 228 | -0.36 | -1.36 | 0.005 | 0.397 | 1.000 | 3033 | tags=39%, list=25%, signal=52% |
| 1386 | GOCC\_VCP\_NPL4\_UFD1\_AAA\_ATPASE\_COMPLEX |  | 5 | -0.71 | -1.36 | 0.078 | 0.397 | 1.000 | 2774 | tags=60%, list=23%, signal=78% |
| 1387 | GOBP\_TELOMERE\_MAINTENANCE\_VIA\_TELOMERE\_LENGTHENING |  | 66 | -0.40 | -1.36 | 0.054 | 0.397 | 1.000 | 4935 | tags=56%, list=41%, signal=95% |
| 1388 | GOBP\_CELLULAR\_RESPONSE\_TO\_CHOLESTEROL |  | 11 | -0.57 | -1.36 | 0.093 | 0.397 | 1.000 | 3600 | tags=55%, list=30%, signal=78% |
| 1389 | GOBP\_POSITIVE\_REGULATION\_OF\_CELL\_DEVELOPMENT |  | 208 | -0.36 | -1.36 | 0.008 | 0.397 | 1.000 | 3196 | tags=34%, list=27%, signal=45% |
| 1390 | GOBP\_REGULATION\_OF\_RAB\_PROTEIN\_SIGNAL\_TRANSDUCTION |  | 6 | -0.67 | -1.36 | 0.088 | 0.397 | 1.000 | 1916 | tags=67%, list=16%, signal=79% |
| 1391 | GOBP\_REGULATION\_OF\_SUPEROXIDE\_METABOLIC\_PROCESS |  | 20 | -0.49 | -1.36 | 0.094 | 0.397 | 1.000 | 4249 | tags=60%, list=35%, signal=93% |
| 1392 | GOBP\_REGULATION\_OF\_DENDRITE\_DEVELOPMENT |  | 73 | -0.39 | -1.36 | 0.042 | 0.397 | 1.000 | 5227 | tags=60%, list=44%, signal=106% |
| 1393 | GOBP\_REGULATION\_OF\_LEUKOCYTE\_ADHESION\_TO\_VASCULAR\_ENDOTHELIAL\_CELL |  | 18 | -0.50 | -1.36 | 0.089 | 0.397 | 1.000 | 1734 | tags=39%, list=14%, signal=45% |
| 1394 | GOBP\_CEREBRAL\_CORTEX\_CELL\_MIGRATION |  | 28 | -0.46 | -1.36 | 0.086 | 0.397 | 1.000 | 633 | tags=18%, list=5%, signal=19% |
| 1395 | GOBP\_CELLULAR\_RESPONSE\_TO\_IONIZING\_RADIATION |  | 49 | -0.41 | -1.36 | 0.062 | 0.397 | 1.000 | 3927 | tags=55%, list=33%, signal=82% |
| 1396 | GOBP\_HAIR\_CELL\_DIFFERENTIATION |  | 28 | -0.46 | -1.36 | 0.081 | 0.396 | 1.000 | 4949 | tags=68%, list=41%, signal=115% |
| 1397 | GOBP\_REGULATION\_OF\_PROTEIN\_LOCALIZATION\_TO\_CENTROSOME |  | 6 | -0.67 | -1.36 | 0.093 | 0.396 | 1.000 | 2479 | tags=50%, list=21%, signal=63% |
| 1398 | GOBP\_NUCLEAR\_CHROMOSOME\_SEGREGATION |  | 200 | -0.36 | -1.36 | 0.009 | 0.396 | 1.000 | 4465 | tags=45%, list=37%, signal=70% |
| 1399 | GOBP\_REGULATION\_OF\_SYNAPTIC\_VESICLE\_FUSION\_TO\_PRESYNAPTIC\_ACTIVE\_ZONE\_MEMBRANE |  | 6 | -0.67 | -1.36 | 0.084 | 0.396 | 1.000 | 207 | tags=33%, list=2%, signal=34% |
| 1400 | GOBP\_POSITIVE\_REGULATION\_OF\_GLYCOPROTEIN\_METABOLIC\_PROCESS |  | 21 | -0.49 | -1.36 | 0.085 | 0.397 | 1.000 | 909 | tags=19%, list=8%, signal=21% |
| 1401 | GOBP\_T\_HELPER\_17\_TYPE\_IMMUNE\_RESPONSE |  | 16 | -0.51 | -1.36 | 0.101 | 0.397 | 1.000 | 1951 | tags=38%, list=16%, signal=45% |
| 1402 | GOBP\_HEART\_VALVE\_DEVELOPMENT |  | 47 | -0.42 | -1.36 | 0.066 | 0.396 | 1.000 | 2933 | tags=40%, list=24%, signal=53% |
| 1403 | GOBP\_NEURAL\_TUBE\_FORMATION |  | 73 | -0.39 | -1.36 | 0.044 | 0.397 | 1.000 | 4242 | tags=42%, list=35%, signal=65% |
| 1404 | GOBP\_REGULATION\_OF\_EXTRACELLULAR\_MATRIX\_DISASSEMBLY |  | 14 | -0.53 | -1.36 | 0.096 | 0.398 | 1.000 | 3551 | tags=57%, list=30%, signal=81% |
| 1405 | GOBP\_CELLULAR\_RESPONSE\_TO\_HEPARIN |  | 5 | -0.70 | -1.36 | 0.087 | 0.398 | 1.000 | 2374 | tags=60%, list=20%, signal=75% |
| 1406 | GOBP\_ACETATE\_ESTER\_TRANSPORT |  | 3 | -0.82 | -1.36 | 0.067 | 0.397 | 1.000 | 322 | tags=67%, list=3%, signal=68% |
| 1407 | GOCC\_NUCLEAR\_PORE |  | 59 | -0.40 | -1.36 | 0.057 | 0.398 | 1.000 | 5623 | tags=63%, list=47%, signal=117% |
| 1408 | GOBP\_MEIOTIC\_CELL\_CYCLE\_PROCESS |  | 108 | -0.38 | -1.36 | 0.029 | 0.398 | 1.000 | 3976 | tags=43%, list=33%, signal=63% |
| 1409 | GOMF\_DYNEIN\_LIGHT\_CHAIN\_BINDING |  | 3 | -0.82 | -1.36 | 0.066 | 0.399 | 1.000 | 2161 | tags=67%, list=18%, signal=81% |
| 1410 | GOBP\_ENERGY\_HOMEOSTASIS |  | 27 | -0.46 | -1.36 | 0.089 | 0.400 | 1.000 | 4528 | tags=63%, list=38%, signal=101% |
| 1411 | GOBP\_POSITIVE\_REGULATION\_OF\_T\_CELL\_TOLERANCE\_INDUCTION |  | 3 | -0.82 | -1.36 | 0.071 | 0.400 | 1.000 | 1016 | tags=67%, list=8%, signal=73% |
| 1412 | GOBP\_NEGATIVE\_REGULATION\_OF\_JUN\_KINASE\_ACTIVITY |  | 10 | -0.58 | -1.36 | 0.102 | 0.400 | 1.000 | 1959 | tags=40%, list=16%, signal=48% |
| 1413 | GOMF\_CYTOSKELETAL\_ANCHOR\_ACTIVITY |  | 18 | -0.50 | -1.36 | 0.098 | 0.400 | 1.000 | 4110 | tags=67%, list=34%, signal=101% |
| 1414 | GOMF\_PHOSPHATASE\_ACTIVITY |  | 211 | -0.36 | -1.36 | 0.006 | 0.400 | 1.000 | 3533 | tags=37%, list=29%, signal=52% |
| 1415 | GOCC\_DENDRITIC\_TREE |  | 403 | -0.35 | -1.36 | 0.000 | 0.399 | 1.000 | 2160 | tags=27%, list=18%, signal=32% |
| 1416 | GOMF\_CHROMO\_SHADOW\_DOMAIN\_BINDING |  | 3 | -0.81 | -1.36 | 0.067 | 0.399 | 1.000 | 902 | tags=67%, list=8%, signal=72% |
| 1417 | GOMF\_RIBOSOMAL\_PROTEIN\_S6\_KINASE\_ACTIVITY |  | 6 | -0.67 | -1.36 | 0.097 | 0.400 | 1.000 | 2456 | tags=67%, list=20%, signal=84% |
| 1418 | GOBP\_ANTEROGRADE\_NEURONAL\_DENSE\_CORE\_VESICLE\_TRANSPORT |  | 4 | -0.75 | -1.36 | 0.085 | 0.400 | 1.000 | 1192 | tags=75%, list=10%, signal=83% |
| 1419 | GOCC\_CELL\_CELL\_CONTACT\_ZONE |  | 47 | -0.41 | -1.36 | 0.062 | 0.399 | 1.000 | 4110 | tags=49%, list=34%, signal=74% |
| 1420 | GOMF\_UDP\_GLYCOSYLTRANSFERASE\_ACTIVITY |  | 92 | -0.38 | -1.36 | 0.041 | 0.400 | 1.000 | 3539 | tags=39%, list=29%, signal=55% |
| 1421 | GOBP\_POSITIVE\_REGULATION\_OF\_TRANSLATION\_IN\_RESPONSE\_TO\_STRESS |  | 6 | -0.67 | -1.36 | 0.091 | 0.399 | 1.000 | 3691 | tags=83%, list=31%, signal=120% |
| 1422 | GOBP\_REGULATION\_OF\_STRIATED\_MUSCLE\_CELL\_DIFFERENTIATION |  | 47 | -0.42 | -1.36 | 0.062 | 0.400 | 1.000 | 4139 | tags=51%, list=34%, signal=78% |
| 1423 | GOBP\_CHROMOSOME\_SEGREGATION |  | 248 | -0.35 | -1.36 | 0.006 | 0.400 | 1.000 | 4589 | tags=46%, list=38%, signal=73% |
| 1424 | GOBP\_POSITIVE\_REGULATION\_OF\_ENDOCYTOSIS |  | 71 | -0.39 | -1.36 | 0.051 | 0.400 | 1.000 | 3814 | tags=45%, list=32%, signal=66% |
| 1425 | GOMF\_CCR\_CHEMOKINE\_RECEPTOR\_BINDING |  | 13 | -0.54 | -1.36 | 0.101 | 0.400 | 1.000 | 1845 | tags=54%, list=15%, signal=64% |
| 1426 | GOBP\_CYTOSKELETON\_ORGANIZATION |  | 957 | -0.34 | -1.36 | 0.000 | 0.400 | 1.000 | 4220 | tags=41%, list=35%, signal=58% |
| 1427 | GOBP\_GLYCOSPHINGOLIPID\_BIOSYNTHETIC\_PROCESS |  | 21 | -0.48 | -1.36 | 0.098 | 0.401 | 1.000 | 2915 | tags=48%, list=24%, signal=63% |
| 1428 | GOCC\_RNA\_POLYMERASE\_III\_TRANSCRIPTION\_REGULATOR\_COMPLEX |  | 7 | -0.63 | -1.36 | 0.110 | 0.403 | 1.000 | 1760 | tags=43%, list=15%, signal=50% |
| 1429 | GOMF\_5\_3\_EXONUCLEASE\_ACTIVITY |  | 15 | -0.52 | -1.36 | 0.098 | 0.403 | 1.000 | 1095 | tags=27%, list=9%, signal=29% |
| 1430 | GOBP\_NATURAL\_KILLER\_CELL\_PROLIFERATION |  | 3 | -0.81 | -1.36 | 0.074 | 0.403 | 1.000 | 1566 | tags=67%, list=13%, signal=77% |
| 1431 | GOBP\_PERISTALSIS |  | 5 | -0.71 | -1.36 | 0.092 | 0.403 | 1.000 | 2604 | tags=80%, list=22%, signal=102% |
| 1432 | GOBP\_PULMONARY\_VALVE\_DEVELOPMENT |  | 18 | -0.50 | -1.36 | 0.100 | 0.403 | 1.000 | 3561 | tags=44%, list=30%, signal=63% |
| 1433 | GOBP\_INTERLEUKIN\_6\_MEDIATED\_SIGNALING\_PATHWAY |  | 19 | -0.49 | -1.36 | 0.102 | 0.403 | 1.000 | 3692 | tags=63%, list=31%, signal=91% |
| 1434 | GOBP\_SMOOTH\_MUSCLE\_CELL\_MATRIX\_ADHESION |  | 4 | -0.75 | -1.36 | 0.080 | 0.403 | 1.000 | 378 | tags=25%, list=3%, signal=26% |
| 1435 | GOCC\_SPECIFIC\_GRANULE |  | 95 | -0.38 | -1.36 | 0.038 | 0.402 | 1.000 | 4398 | tags=54%, list=37%, signal=84% |
| 1436 | GOBP\_BRAIN\_MORPHOGENESIS |  | 27 | -0.46 | -1.36 | 0.090 | 0.403 | 1.000 | 3864 | tags=48%, list=32%, signal=71% |
| 1437 | GOBP\_POSTSYNAPTIC\_CYTOSKELETON\_ORGANIZATION |  | 7 | -0.64 | -1.36 | 0.101 | 0.403 | 1.000 | 2546 | tags=43%, list=21%, signal=54% |
| 1438 | GOMF\_1\_PHOSPHATIDYLINOSITOL\_4\_PHOSPHATE\_3\_KINASE\_ACTIVITY |  | 7 | -0.64 | -1.36 | 0.094 | 0.403 | 1.000 | 1884 | tags=71%, list=16%, signal=85% |
| 1439 | GOBP\_POSTSYNAPSE\_TO\_NUCLEUS\_SIGNALING\_PATHWAY |  | 6 | -0.67 | -1.36 | 0.094 | 0.403 | 1.000 | 1675 | tags=50%, list=14%, signal=58% |
| 1440 | GOBP\_EMBRYONIC\_MORPHOGENESIS |  | 366 | -0.35 | -1.36 | 0.001 | 0.403 | 1.000 | 3565 | tags=34%, list=30%, signal=47% |
| 1441 | GOBP\_RESPONSE\_TO\_L\_GLUTAMATE |  | 7 | -0.64 | -1.36 | 0.096 | 0.404 | 1.000 | 711 | tags=43%, list=6%, signal=46% |
| 1442 | GOBP\_POSITIVE\_REGULATION\_OF\_MITOCHONDRIAL\_DEPOLARIZATION |  | 8 | -0.62 | -1.36 | 0.100 | 0.405 | 1.000 | 3376 | tags=50%, list=28%, signal=70% |
| 1443 | GOBP\_NEURON\_PROJECTION\_REGENERATION |  | 46 | -0.42 | -1.36 | 0.069 | 0.405 | 1.000 | 1462 | tags=24%, list=12%, signal=27% |
| 1444 | GOBP\_REGULATION\_OF\_NON\_CANONICAL\_WNT\_SIGNALING\_PATHWAY |  | 19 | -0.49 | -1.36 | 0.103 | 0.405 | 1.000 | 1991 | tags=32%, list=17%, signal=38% |
| 1445 | GOBP\_REGULATION\_OF\_VASCULAR\_ASSOCIATED\_SMOOTH\_MUSCLE\_CELL\_DIFFERENTIATION |  | 13 | -0.54 | -1.36 | 0.100 | 0.405 | 1.000 | 481 | tags=23%, list=4%, signal=24% |
| 1446 | GOBP\_NEGATIVE\_REGULATION\_OF\_NEURON\_APOPTOTIC\_PROCESS |  | 79 | -0.38 | -1.36 | 0.050 | 0.405 | 1.000 | 3149 | tags=35%, list=26%, signal=48% |
| 1447 | GOBP\_AMINOPHOSPHOLIPID\_TRANSLOCATION |  | 6 | -0.67 | -1.36 | 0.089 | 0.405 | 1.000 | 952 | tags=50%, list=8%, signal=54% |
| 1448 | GOBP\_NUCLEOSOME\_ORGANIZATION |  | 79 | -0.39 | -1.36 | 0.047 | 0.406 | 1.000 | 3900 | tags=42%, list=32%, signal=61% |
| 1449 | GOBP\_REGULATION\_OF\_POSTSYNAPSE\_ORGANIZATION |  | 60 | -0.40 | -1.35 | 0.055 | 0.406 | 1.000 | 1793 | tags=27%, list=15%, signal=31% |
| 1450 | GOBP\_REGULATION\_OF\_LEUKOCYTE\_MIGRATION |  | 133 | -0.37 | -1.35 | 0.025 | 0.406 | 1.000 | 2955 | tags=35%, list=25%, signal=46% |
| 1451 | GOBP\_VENTRICULAR\_SEPTUM\_MORPHOGENESIS |  | 29 | -0.45 | -1.35 | 0.083 | 0.406 | 1.000 | 2308 | tags=28%, list=19%, signal=34% |
| 1452 | GOCC\_PHOSPHATIDYLINOSITOL\_3\_KINASE\_COMPLEX\_CLASS\_III |  | 6 | -0.67 | -1.35 | 0.094 | 0.406 | 1.000 | 905 | tags=33%, list=8%, signal=36% |
| 1453 | GOMF\_CATION\_CATION\_ANTIPORTER\_ACTIVITY |  | 16 | -0.51 | -1.35 | 0.099 | 0.406 | 1.000 | 1021 | tags=38%, list=9%, signal=41% |
| 1454 | GOCC\_CHROMATIN |  | 666 | -0.34 | -1.35 | 0.000 | 0.406 | 1.000 | 3907 | tags=42%, list=33%, signal=58% |
| 1455 | GOMF\_GAMMA\_CATENIN\_BINDING |  | 10 | -0.57 | -1.35 | 0.101 | 0.406 | 1.000 | 2079 | tags=50%, list=17%, signal=60% |
| 1456 | GOCC\_ENDOPLASMIC\_RETICULUM\_EXIT\_SITE |  | 24 | -0.47 | -1.35 | 0.097 | 0.407 | 1.000 | 4427 | tags=58%, list=37%, signal=92% |
| 1457 | GOBP\_ENSHEATHMENT\_OF\_NEURONS |  | 92 | -0.38 | -1.35 | 0.043 | 0.406 | 1.000 | 4598 | tags=51%, list=38%, signal=82% |
| 1458 | GOBP\_POSITIVE\_REGULATION\_OF\_MACROPHAGE\_ACTIVATION |  | 14 | -0.53 | -1.35 | 0.098 | 0.406 | 1.000 | 5369 | tags=86%, list=45%, signal=155% |
| 1459 | GOBP\_HISTONE\_H3\_DEACETYLATION |  | 16 | -0.51 | -1.35 | 0.107 | 0.406 | 1.000 | 2855 | tags=38%, list=24%, signal=49% |
| 1460 | GOBP\_PERICARDIUM\_DEVELOPMENT |  | 15 | -0.52 | -1.35 | 0.106 | 0.406 | 1.000 | 3256 | tags=60%, list=27%, signal=82% |
| 1461 | GOBP\_REGULATION\_OF\_MUSCLE\_CELL\_DIFFERENTIATION |  | 93 | -0.38 | -1.35 | 0.043 | 0.406 | 1.000 | 3658 | tags=42%, list=30%, signal=60% |
| 1462 | GOCC\_TORC2\_COMPLEX |  | 12 | -0.55 | -1.35 | 0.110 | 0.406 | 1.000 | 2850 | tags=50%, list=24%, signal=65% |
| 1463 | GOBP\_SARCOMERE\_ORGANIZATION |  | 17 | -0.51 | -1.35 | 0.101 | 0.407 | 1.000 | 2070 | tags=29%, list=17%, signal=35% |
| 1464 | GOBP\_POLYSACCHARIDE\_BIOSYNTHETIC\_PROCESS |  | 52 | -0.41 | -1.35 | 0.067 | 0.408 | 1.000 | 4203 | tags=52%, list=35%, signal=80% |
| 1465 | GOBP\_T\_CELL\_EXTRAVASATION |  | 7 | -0.63 | -1.35 | 0.100 | 0.407 | 1.000 | 2011 | tags=57%, list=17%, signal=69% |
| 1466 | GOBP\_CELL\_ACTIVATION |  | 894 | -0.34 | -1.35 | 0.000 | 0.407 | 1.000 | 3483 | tags=36%, list=29%, signal=47% |
| 1467 | GOBP\_CARDIAC\_VENTRICLE\_DEVELOPMENT |  | 80 | -0.38 | -1.35 | 0.045 | 0.407 | 1.000 | 2474 | tags=25%, list=21%, signal=31% |
| 1468 | GOBP\_BEHAVIOR |  | 325 | -0.35 | -1.35 | 0.002 | 0.407 | 1.000 | 3135 | tags=34%, list=26%, signal=44% |
| 1469 | GOBP\_POSITIVE\_REGULATION\_OF\_VASCULAR\_ASSOCIATED\_SMOOTH\_MUSCLE\_CELL\_MIGRATION |  | 12 | -0.55 | -1.35 | 0.109 | 0.408 | 1.000 | 3069 | tags=50%, list=26%, signal=67% |
| 1470 | GOMF\_SOLUTE\_BICARBONATE\_SYMPORTER\_ACTIVITY |  | 7 | -0.64 | -1.35 | 0.100 | 0.408 | 1.000 | 2644 | tags=86%, list=22%, signal=110% |
| 1471 | GOBP\_POSITIVE\_REGULATION\_OF\_CELL\_ADHESION\_MEDIATED\_BY\_INTEGRIN |  | 16 | -0.51 | -1.35 | 0.100 | 0.408 | 1.000 | 2730 | tags=56%, list=23%, signal=73% |
| 1472 | GOBP\_DOPAMINE\_TRANSPORT |  | 31 | -0.44 | -1.35 | 0.092 | 0.408 | 1.000 | 954 | tags=19%, list=8%, signal=21% |
| 1473 | GOBP\_POSITIVE\_REGULATION\_OF\_APOPTOTIC\_SIGNALING\_PATHWAY |  | 80 | -0.38 | -1.35 | 0.049 | 0.408 | 1.000 | 4673 | tags=50%, list=39%, signal=81% |
| 1474 | GOMF\_G\_PROTEIN\_GAMMA\_SUBUNIT\_BINDING |  | 4 | -0.75 | -1.35 | 0.080 | 0.409 | 1.000 | 1753 | tags=50%, list=15%, signal=59% |
| 1475 | GOCC\_LATERAL\_ELEMENT |  | 7 | -0.64 | -1.35 | 0.099 | 0.409 | 1.000 | 3214 | tags=71%, list=27%, signal=97% |
| 1476 | GOBP\_POSITIVE\_REGULATION\_OF\_CHEMOKINE\_C\_X\_C\_MOTIF\_LIGAND\_2\_PRODUCTION |  | 9 | -0.60 | -1.35 | 0.110 | 0.409 | 1.000 | 3007 | tags=67%, list=25%, signal=89% |
| 1477 | GOBP\_REGULATION\_OF\_TRANSLATION\_IN\_RESPONSE\_TO\_ENDOPLASMIC\_RETICULUM\_STRESS |  | 9 | -0.60 | -1.35 | 0.100 | 0.409 | 1.000 | 3872 | tags=56%, list=32%, signal=82% |
| 1478 | GOBP\_REGULATION\_OF\_MYELOID\_CELL\_DIFFERENTIATION |  | 158 | -0.36 | -1.35 | 0.019 | 0.410 | 1.000 | 3692 | tags=43%, list=31%, signal=61% |
| 1479 | GOBP\_ALKALOID\_METABOLIC\_PROCESS |  | 5 | -0.70 | -1.35 | 0.096 | 0.410 | 1.000 | 2516 | tags=80%, list=21%, signal=101% |
| 1480 | GOBP\_REGULATION\_OF\_PROTEIN\_IMPORT |  | 51 | -0.41 | -1.35 | 0.066 | 0.410 | 1.000 | 4032 | tags=41%, list=34%, signal=62% |
| 1481 | GOCC\_CUL3\_RING\_UBIQUITIN\_LIGASE\_COMPLEX |  | 32 | -0.44 | -1.35 | 0.080 | 0.410 | 1.000 | 3102 | tags=47%, list=26%, signal=63% |
| 1482 | GOBP\_SUBSTRATE\_ADHESION\_DEPENDENT\_CELL\_SPREADING |  | 87 | -0.38 | -1.35 | 0.045 | 0.410 | 1.000 | 3186 | tags=33%, list=27%, signal=45% |
| 1483 | GOBP\_NEGATIVE\_REGULATION\_OF\_MACROAUTOPHAGY |  | 29 | -0.45 | -1.35 | 0.088 | 0.410 | 1.000 | 2503 | tags=34%, list=21%, signal=43% |
| 1484 | GOBP\_SMALL\_RNA\_LOADING\_ONTO\_RISC |  | 6 | -0.66 | -1.35 | 0.096 | 0.410 | 1.000 | 1503 | tags=50%, list=13%, signal=57% |
| 1485 | GOBP\_CELLULAR\_DEFENSE\_RESPONSE |  | 23 | -0.47 | -1.35 | 0.099 | 0.411 | 1.000 | 3003 | tags=48%, list=25%, signal=64% |
| 1486 | GOBP\_COMPLEMENT\_ACTIVATION\_LECTIN\_PATHWAY |  | 5 | -0.70 | -1.35 | 0.090 | 0.411 | 1.000 | 1460 | tags=60%, list=12%, signal=68% |
| 1487 | GOBP\_GROWTH\_HORMONE\_SECRETION |  | 6 | -0.66 | -1.35 | 0.103 | 0.411 | 1.000 | 2351 | tags=50%, list=20%, signal=62% |
| 1488 | GOMF\_PHOSPHOPROTEIN\_PHOSPHATASE\_ACTIVITY |  | 136 | -0.36 | -1.35 | 0.025 | 0.411 | 1.000 | 3483 | tags=39%, list=29%, signal=54% |
| 1489 | GOMF\_ESTROGEN\_16\_ALPHA\_HYDROXYLASE\_ACTIVITY |  | 3 | -0.81 | -1.35 | 0.080 | 0.411 | 1.000 | 2278 | tags=100%, list=19%, signal=123% |
| 1490 | GOBP\_REGULATION\_OF\_CELL\_MATURATION |  | 12 | -0.55 | -1.35 | 0.107 | 0.411 | 1.000 | 3483 | tags=58%, list=29%, signal=82% |
| 1491 | GOBP\_CELL\_AGING |  | 81 | -0.38 | -1.35 | 0.050 | 0.411 | 1.000 | 3668 | tags=40%, list=31%, signal=56% |
| 1492 | GOBP\_REGULATION\_OF\_IMMUNE\_SYSTEM\_PROCESS |  | 860 | -0.34 | -1.35 | 0.000 | 0.411 | 1.000 | 3565 | tags=37%, list=30%, signal=49% |
| 1493 | GOMF\_ALPHA\_TUBULIN\_BINDING |  | 27 | -0.46 | -1.35 | 0.099 | 0.411 | 1.000 | 3499 | tags=44%, list=29%, signal=63% |
| 1494 | GOBP\_REGULATION\_OF\_VOLTAGE\_GATED\_SODIUM\_CHANNEL\_ACTIVITY |  | 3 | -0.81 | -1.35 | 0.066 | 0.411 | 1.000 | 7 | tags=33%, list=0%, signal=33% |
| 1495 | GOMF\_HISTONE\_METHYLTRANSFERASE\_ACTIVITY\_H3\_K4\_SPECIFIC |  | 10 | -0.57 | -1.35 | 0.100 | 0.411 | 1.000 | 2041 | tags=40%, list=17%, signal=48% |
| 1496 | GOBP\_POSITIVE\_REGULATION\_OF\_CLATHRIN\_DEPENDENT\_ENDOCYTOSIS |  | 4 | -0.75 | -1.35 | 0.085 | 0.411 | 1.000 | 1663 | tags=50%, list=14%, signal=58% |
| 1497 | GOBP\_APPENDAGE\_DEVELOPMENT |  | 106 | -0.37 | -1.35 | 0.038 | 0.411 | 1.000 | 4102 | tags=40%, list=34%, signal=60% |
| 1498 | GOBP\_NEGATIVE\_REGULATION\_OF\_MICROTUBULE\_POLYMERIZATION |  | 10 | -0.58 | -1.35 | 0.107 | 0.410 | 1.000 | 2311 | tags=40%, list=19%, signal=49% |
| 1499 | GOBP\_FAS\_SIGNALING\_PATHWAY |  | 4 | -0.75 | -1.35 | 0.090 | 0.411 | 1.000 | 2162 | tags=75%, list=18%, signal=91% |
| 1500 | GOBP\_POSITIVE\_REGULATION\_OF\_PROTEIN\_CONTAINING\_COMPLEX\_DISASSEMBLY |  | 24 | -0.47 | -1.35 | 0.101 | 0.412 | 1.000 | 3967 | tags=46%, list=33%, signal=68% |
| 1501 | GOBP\_CENTROSOME\_DUPLICATION |  | 59 | -0.40 | -1.35 | 0.065 | 0.412 | 1.000 | 5075 | tags=61%, list=42%, signal=105% |
| 1502 | GOBP\_RESPONSE\_TO\_AXON\_INJURY |  | 53 | -0.40 | -1.35 | 0.071 | 0.412 | 1.000 | 1462 | tags=23%, list=12%, signal=26% |
| 1503 | GOBP\_MATURE\_B\_CELL\_DIFFERENTIATION |  | 21 | -0.48 | -1.35 | 0.100 | 0.412 | 1.000 | 1432 | tags=33%, list=12%, signal=38% |
| 1504 | GOBP\_POSITIVE\_REGULATION\_OF\_GLUCOSE\_IMPORT |  | 27 | -0.45 | -1.35 | 0.091 | 0.412 | 1.000 | 1991 | tags=33%, list=17%, signal=40% |
| 1505 | GOBP\_HISTONE\_MONOUBIQUITINATION |  | 25 | -0.46 | -1.35 | 0.099 | 0.411 | 1.000 | 5009 | tags=64%, list=42%, signal=110% |
| 1506 | GOBP\_REGULATION\_OF\_HISTONE\_METHYLATION |  | 45 | -0.42 | -1.35 | 0.071 | 0.411 | 1.000 | 4698 | tags=67%, list=39%, signal=109% |
| 1507 | GOBP\_REGULATION\_OF\_PROTEIN\_LOCALIZATION\_TO\_CHROMATIN |  | 6 | -0.66 | -1.35 | 0.095 | 0.412 | 1.000 | 3836 | tags=83%, list=32%, signal=122% |
| 1508 | GOCC\_MSL\_COMPLEX |  | 3 | -0.81 | -1.35 | 0.074 | 0.412 | 1.000 | 1056 | tags=33%, list=9%, signal=37% |
| 1509 | GOBP\_POLYOL\_TRANSPORT |  | 8 | -0.61 | -1.35 | 0.099 | 0.411 | 1.000 | 2215 | tags=50%, list=18%, signal=61% |
| 1510 | GOCC\_PCG\_PROTEIN\_COMPLEX |  | 37 | -0.43 | -1.35 | 0.082 | 0.411 | 1.000 | 4500 | tags=54%, list=37%, signal=86% |
| 1511 | GOBP\_BASIC\_AMINO\_ACID\_TRANSMEMBRANE\_TRANSPORT |  | 9 | -0.59 | -1.35 | 0.109 | 0.411 | 1.000 | 2026 | tags=33%, list=17%, signal=40% |
| 1512 | GOBP\_EMBRYONIC\_ORGAN\_DEVELOPMENT |  | 255 | -0.35 | -1.35 | 0.006 | 0.411 | 1.000 | 3615 | tags=36%, list=30%, signal=50% |
| 1513 | GOMF\_AMINOPHOSPHOLIPID\_FLIPPASE\_ACTIVITY |  | 6 | -0.67 | -1.35 | 0.096 | 0.412 | 1.000 | 952 | tags=50%, list=8%, signal=54% |
| 1514 | GOBP\_DETECTION\_OF\_CELL\_DENSITY |  | 9 | -0.59 | -1.35 | 0.108 | 0.411 | 1.000 | 3195 | tags=56%, list=27%, signal=76% |
| 1515 | GOBP\_BRANCH\_ELONGATION\_OF\_AN\_EPITHELIUM |  | 14 | -0.53 | -1.35 | 0.117 | 0.411 | 1.000 | 3615 | tags=57%, list=30%, signal=82% |
| 1516 | GOBP\_RESPONSE\_TO\_ANTIBIOTIC |  | 37 | -0.43 | -1.35 | 0.076 | 0.411 | 1.000 | 2913 | tags=43%, list=24%, signal=57% |
| 1517 | GOMF\_ANTIGEN\_BINDING |  | 20 | -0.48 | -1.35 | 0.098 | 0.411 | 1.000 | 1646 | tags=35%, list=14%, signal=40% |
| 1518 | GOBP\_AMYLOID\_BETA\_CLEARANCE |  | 26 | -0.46 | -1.35 | 0.089 | 0.411 | 1.000 | 3007 | tags=38%, list=25%, signal=51% |
| 1519 | GOBP\_RENIN\_SECRETION\_INTO\_BLOOD\_STREAM |  | 4 | -0.75 | -1.35 | 0.091 | 0.411 | 1.000 | 2480 | tags=75%, list=21%, signal=94% |
| 1520 | GOBP\_NEUROFILAMENT\_CYTOSKELETON\_ORGANIZATION |  | 6 | -0.66 | -1.35 | 0.099 | 0.411 | 1.000 | 1749 | tags=33%, list=15%, signal=39% |
| 1521 | GOBP\_SODIUM\_ION\_TRANSPORT |  | 139 | -0.36 | -1.35 | 0.022 | 0.411 | 1.000 | 3089 | tags=39%, list=26%, signal=52% |
| 1522 | GOBP\_CELLULAR\_RESPONSE\_TO\_MURAMYL\_DIPEPTIDE |  | 6 | -0.66 | -1.35 | 0.105 | 0.411 | 1.000 | 250 | tags=17%, list=2%, signal=17% |
| 1523 | GOBP\_POSITIVE\_REGULATION\_OF\_TAU\_PROTEIN\_KINASE\_ACTIVITY |  | 4 | -0.75 | -1.35 | 0.084 | 0.411 | 1.000 | 2902 | tags=75%, list=24%, signal=99% |
| 1524 | GOCC\_SPINDLE |  | 290 | -0.35 | -1.35 | 0.006 | 0.411 | 1.000 | 5063 | tags=50%, list=42%, signal=85% |
| 1525 | GOBP\_HISTAMINE\_SECRETION |  | 7 | -0.63 | -1.35 | 0.102 | 0.412 | 1.000 | 2943 | tags=43%, list=25%, signal=57% |
| 1526 | GOBP\_NEGATIVE\_REGULATION\_OF\_OSSIFICATION |  | 21 | -0.48 | -1.35 | 0.106 | 0.412 | 1.000 | 2474 | tags=38%, list=21%, signal=48% |
| 1527 | GOBP\_CALCIUM\_ION\_EXPORT |  | 6 | -0.66 | -1.35 | 0.103 | 0.413 | 1.000 | 1659 | tags=50%, list=14%, signal=58% |
| 1528 | GOBP\_SOMATIC\_STEM\_CELL\_DIVISION |  | 6 | -0.66 | -1.34 | 0.101 | 0.414 | 1.000 | 1187 | tags=33%, list=10%, signal=37% |
| 1529 | GOMF\_SIGNALING\_RECEPTOR\_BINDING |  | 873 | -0.34 | -1.34 | 0.000 | 0.414 | 1.000 | 3789 | tags=38%, list=32%, signal=51% |
| 1530 | GOBP\_ESTABLISHMENT\_OR\_MAINTENANCE\_OF\_CELL\_POLARITY |  | 173 | -0.36 | -1.34 | 0.017 | 0.414 | 1.000 | 4152 | tags=41%, list=35%, signal=62% |
| 1531 | GOMF\_COMPLEMENT\_COMPONENT\_C3B\_BINDING |  | 4 | -0.74 | -1.34 | 0.087 | 0.415 | 1.000 | 1808 | tags=50%, list=15%, signal=59% |
| 1532 | GOBP\_NEGATIVE\_REGULATION\_OF\_ERYTHROCYTE\_DIFFERENTIATION |  | 8 | -0.61 | -1.34 | 0.106 | 0.415 | 1.000 | 3546 | tags=50%, list=30%, signal=71% |
| 1533 | GOBP\_SENSORY\_ORGAN\_DEVELOPMENT |  | 312 | -0.35 | -1.34 | 0.004 | 0.415 | 1.000 | 3638 | tags=38%, list=30%, signal=53% |
| 1534 | GOCC\_INTRINSIC\_COMPONENT\_OF\_POSTSYNAPTIC\_MEMBRANE |  | 47 | -0.41 | -1.34 | 0.076 | 0.415 | 1.000 | 2623 | tags=32%, list=22%, signal=41% |
| 1535 | GOCC\_MITOTIC\_SPINDLE |  | 119 | -0.37 | -1.34 | 0.033 | 0.415 | 1.000 | 4982 | tags=48%, list=41%, signal=81% |
| 1536 | GOBP\_REGULATION\_OF\_TRANSCRIPTION\_FROM\_RNA\_POLYMERASE\_II\_PROMOTER\_INVOLVED\_IN\_HEART\_DEVELOPMENT |  | 8 | -0.61 | -1.34 | 0.114 | 0.416 | 1.000 | 2212 | tags=38%, list=18%, signal=46% |
| 1537 | GOCC\_TIGHT\_JUNCTION |  | 87 | -0.38 | -1.34 | 0.048 | 0.416 | 1.000 | 2702 | tags=33%, list=22%, signal=43% |
| 1538 | GOBP\_POSITIVE\_REGULATION\_OF\_HEMATOPOIETIC\_STEM\_CELL\_PROLIFERATION |  | 4 | -0.74 | -1.34 | 0.093 | 0.416 | 1.000 | 2798 | tags=75%, list=23%, signal=98% |
| 1539 | GOBP\_REGULATION\_OF\_PROTEIN\_DEACETYLATION |  | 24 | -0.47 | -1.34 | 0.098 | 0.417 | 1.000 | 4184 | tags=63%, list=35%, signal=96% |
| 1540 | GOCC\_CONDENSED\_CHROMOSOME\_OUTER\_KINETOCHORE |  | 11 | -0.56 | -1.34 | 0.109 | 0.417 | 1.000 | 3822 | tags=73%, list=32%, signal=107% |
| 1541 | GOBP\_TRYPTOPHAN\_CATABOLIC\_PROCESS\_TO\_KYNURENINE |  | 3 | -0.80 | -1.34 | 0.081 | 0.417 | 1.000 | 2220 | tags=67%, list=18%, signal=82% |
| 1542 | GOCC\_METHYLTRANSFERASE\_COMPLEX |  | 67 | -0.39 | -1.34 | 0.063 | 0.417 | 1.000 | 4512 | tags=46%, list=38%, signal=74% |
| 1543 | GOCC\_NUCLEAR\_PERIPHERY |  | 101 | -0.37 | -1.34 | 0.043 | 0.417 | 1.000 | 4781 | tags=50%, list=40%, signal=83% |
| 1544 | GOBP\_LEFT\_RIGHT\_AXIS\_SPECIFICATION |  | 10 | -0.57 | -1.34 | 0.112 | 0.417 | 1.000 | 1764 | tags=30%, list=15%, signal=35% |
| 1545 | GOBP\_TELOMERE\_ORGANIZATION |  | 120 | -0.37 | -1.34 | 0.035 | 0.417 | 1.000 | 4024 | tags=40%, list=34%, signal=60% |
| 1546 | GOBP\_EXTERNAL\_ENCAPSULATING\_STRUCTURE\_ORGANIZATION |  | 271 | -0.35 | -1.34 | 0.005 | 0.417 | 1.000 | 3186 | tags=30%, list=27%, signal=40% |
| 1547 | GOCC\_PERICENTRIC\_HETEROCHROMATIN |  | 17 | -0.50 | -1.34 | 0.110 | 0.417 | 1.000 | 4790 | tags=71%, list=40%, signal=117% |
| 1548 | GOBP\_REGULATION\_OF\_NEUROGENESIS |  | 245 | -0.35 | -1.34 | 0.009 | 0.417 | 1.000 | 4698 | tags=47%, list=39%, signal=75% |
| 1549 | GOBP\_DNA\_GEOMETRIC\_CHANGE |  | 92 | -0.38 | -1.34 | 0.049 | 0.417 | 1.000 | 5223 | tags=65%, list=43%, signal=115% |
| 1550 | GOCC\_COLLAGEN\_TYPE\_IV\_TRIMER |  | 3 | -0.81 | -1.34 | 0.082 | 0.417 | 1.000 | 2333 | tags=100%, list=19%, signal=124% |
| 1551 | GOBP\_OLFACTORY\_LOBE\_DEVELOPMENT |  | 16 | -0.51 | -1.34 | 0.108 | 0.417 | 1.000 | 4696 | tags=56%, list=39%, signal=92% |
| 1552 | GOBP\_REGULATION\_OF\_LEUKOCYTE\_MEDIATED\_IMMUNITY |  | 106 | -0.37 | -1.34 | 0.036 | 0.417 | 1.000 | 2639 | tags=34%, list=22%, signal=43% |
| 1553 | GOBP\_DNA\_DAMAGE\_RESPONSE\_SIGNAL\_TRANSDUCTION\_BY\_P53\_CLASS\_MEDIATOR |  | 84 | -0.38 | -1.34 | 0.049 | 0.417 | 1.000 | 2695 | tags=33%, list=22%, signal=43% |
| 1554 | GOBP\_LAMELLIPODIUM\_MORPHOGENESIS |  | 14 | -0.52 | -1.34 | 0.110 | 0.417 | 1.000 | 591 | tags=21%, list=5%, signal=23% |
| 1555 | GOBP\_METAPHASE\_ANAPHASE\_TRANSITION\_OF\_CELL\_CYCLE |  | 56 | -0.40 | -1.34 | 0.068 | 0.417 | 1.000 | 4419 | tags=45%, list=37%, signal=70% |
| 1556 | GOMF\_NUCLEAR\_RECEPTOR\_BINDING |  | 77 | -0.38 | -1.34 | 0.058 | 0.417 | 1.000 | 2811 | tags=34%, list=23%, signal=44% |
| 1557 | GOBP\_REGULATION\_OF\_VESICLE\_TRANSPORT\_ALONG\_MICROTUBULE |  | 3 | -0.80 | -1.34 | 0.076 | 0.417 | 1.000 | 1620 | tags=67%, list=13%, signal=77% |
| 1558 | GOBP\_REGULATION\_OF\_DNA\_METABOLIC\_PROCESS |  | 247 | -0.35 | -1.34 | 0.008 | 0.417 | 1.000 | 3452 | tags=37%, list=29%, signal=51% |
| 1559 | GOBP\_ENDODERM\_DEVELOPMENT |  | 49 | -0.41 | -1.34 | 0.071 | 0.417 | 1.000 | 4336 | tags=51%, list=36%, signal=80% |
| 1560 | GOCC\_UBIQUITIN\_LIGASE\_COMPLEX |  | 226 | -0.35 | -1.34 | 0.008 | 0.417 | 1.000 | 4019 | tags=41%, list=33%, signal=60% |
| 1561 | GOMF\_TRANSITION\_METAL\_ION\_BINDING |  | 654 | -0.34 | -1.34 | 0.000 | 0.418 | 1.000 | 3718 | tags=38%, list=31%, signal=52% |
| 1562 | GOBP\_REGULATION\_OF\_JNK\_CASCADE |  | 87 | -0.38 | -1.34 | 0.046 | 0.418 | 1.000 | 2894 | tags=37%, list=24%, signal=48% |
| 1563 | GOMF\_GTP\_DEPENDENT\_PROTEIN\_BINDING |  | 18 | -0.50 | -1.34 | 0.112 | 0.418 | 1.000 | 3112 | tags=44%, list=26%, signal=60% |
| 1564 | GOBP\_INNER\_EAR\_AUDITORY\_RECEPTOR\_CELL\_DIFFERENTIATION |  | 23 | -0.47 | -1.34 | 0.106 | 0.418 | 1.000 | 4204 | tags=61%, list=35%, signal=93% |
| 1565 | GOBP\_POSITIVE\_REGULATION\_OF\_AMYLOID\_PRECURSOR\_PROTEIN\_BIOSYNTHETIC\_PROCESS |  | 4 | -0.74 | -1.34 | 0.090 | 0.418 | 1.000 | 68 | tags=25%, list=1%, signal=25% |
| 1566 | GOBP\_CAMP\_CATABOLIC\_PROCESS |  | 7 | -0.63 | -1.34 | 0.104 | 0.418 | 1.000 | 2481 | tags=57%, list=21%, signal=72% |
| 1567 | GOBP\_CYTOSKELETON\_DEPENDENT\_INTRACELLULAR\_TRANSPORT |  | 155 | -0.36 | -1.34 | 0.022 | 0.419 | 1.000 | 3668 | tags=37%, list=31%, signal=53% |
| 1568 | GOBP\_NEGATIVE\_REGULATION\_OF\_AXON\_EXTENSION\_INVOLVED\_IN\_AXON\_GUIDANCE |  | 19 | -0.49 | -1.34 | 0.103 | 0.418 | 1.000 | 488 | tags=21%, list=4%, signal=22% |
| 1569 | GOBP\_REGULATION\_OF\_MACROPHAGE\_MIGRATION |  | 27 | -0.45 | -1.34 | 0.096 | 0.418 | 1.000 | 3093 | tags=44%, list=26%, signal=60% |
| 1570 | GOBP\_NEGATIVE\_REGULATION\_OF\_PRI\_MIRNA\_TRANSCRIPTION\_BY\_RNA\_POLYMERASE\_II |  | 10 | -0.57 | -1.34 | 0.105 | 0.418 | 1.000 | 408 | tags=30%, list=3%, signal=31% |
| 1571 | GOBP\_POSITIVE\_REGULATION\_OF\_CYTOKINE\_PRODUCTION\_INVOLVED\_IN\_INFLAMMATORY\_RESPONSE |  | 8 | -0.60 | -1.34 | 0.121 | 0.419 | 1.000 | 2322 | tags=50%, list=19%, signal=62% |
| 1572 | GOBP\_ENDODERMAL\_CELL\_DIFFERENTIATION |  | 31 | -0.44 | -1.34 | 0.094 | 0.419 | 1.000 | 2630 | tags=39%, list=22%, signal=49% |
| 1573 | GOBP\_CANONICAL\_WNT\_SIGNALING\_PATHWAY\_INVOLVED\_IN\_REGULATION\_OF\_CELL\_PROLIFERATION |  | 4 | -0.75 | -1.34 | 0.098 | 0.419 | 1.000 | 2811 | tags=75%, list=23%, signal=98% |
| 1574 | GOCC\_EUCHROMATIN |  | 19 | -0.49 | -1.34 | 0.109 | 0.419 | 1.000 | 4508 | tags=53%, list=38%, signal=84% |
| 1575 | GOBP\_POSITIVE\_REGULATION\_OF\_WOUND\_HEALING\_SPREADING\_OF\_EPIDERMAL\_CELLS |  | 4 | -0.74 | -1.34 | 0.096 | 0.420 | 1.000 | 1743 | tags=50%, list=15%, signal=58% |
| 1576 | GOBP\_IMMUNE\_SYSTEM\_DEVELOPMENT |  | 660 | -0.34 | -1.34 | 0.000 | 0.420 | 1.000 | 3468 | tags=37%, list=29%, signal=49% |
| 1577 | GOMF\_BETA\_CATENIN\_BINDING |  | 66 | -0.39 | -1.34 | 0.067 | 0.420 | 1.000 | 3560 | tags=42%, list=30%, signal=60% |
| 1578 | GOMF\_GROWTH\_HORMONE\_RECEPTOR\_BINDING |  | 4 | -0.74 | -1.34 | 0.100 | 0.420 | 1.000 | 2670 | tags=75%, list=22%, signal=96% |
| 1579 | GOBP\_CELL\_DIFFERENTIATION\_IN\_HINDBRAIN |  | 12 | -0.55 | -1.34 | 0.113 | 0.421 | 1.000 | 2528 | tags=33%, list=21%, signal=42% |
| 1580 | GOCC\_SIDE\_OF\_MEMBRANE |  | 329 | -0.34 | -1.34 | 0.003 | 0.421 | 1.000 | 3163 | tags=37%, list=26%, signal=49% |
| 1581 | GOBP\_CALCIUM\_ION\_TRANSPORT\_INTO\_CYTOSOL |  | 95 | -0.37 | -1.34 | 0.045 | 0.421 | 1.000 | 3313 | tags=34%, list=28%, signal=46% |
| 1582 | GOBP\_NEGATIVE\_REGULATION\_OF\_KINASE\_ACTIVITY |  | 188 | -0.35 | -1.34 | 0.017 | 0.421 | 1.000 | 3934 | tags=41%, list=33%, signal=60% |
| 1583 | GOBP\_MEIOTIC\_CELL\_CYCLE |  | 144 | -0.36 | -1.34 | 0.024 | 0.421 | 1.000 | 4695 | tags=47%, list=39%, signal=77% |
| 1584 | GOBP\_REGULATION\_OF\_TRANSFORMING\_GROWTH\_FACTOR\_BETA\_ACTIVATION |  | 9 | -0.59 | -1.34 | 0.121 | 0.421 | 1.000 | 3064 | tags=67%, list=26%, signal=89% |
| 1585 | GOBP\_NEGATIVE\_REGULATION\_OF\_GLIAL\_CELL\_DIFFERENTIATION |  | 11 | -0.56 | -1.34 | 0.123 | 0.421 | 1.000 | 507 | tags=27%, list=4%, signal=28% |
| 1586 | GOBP\_POSITIVE\_REGULATION\_OF\_CELL\_POPULATION\_PROLIFERATION |  | 577 | -0.34 | -1.34 | 0.000 | 0.421 | 1.000 | 3331 | tags=35%, list=28%, signal=46% |
| 1587 | GOBP\_HISTONE\_H2A\_MONOUBIQUITINATION |  | 15 | -0.52 | -1.34 | 0.112 | 0.421 | 1.000 | 5009 | tags=73%, list=42%, signal=126% |
| 1588 | GOBP\_EMBRYO\_DEVELOPMENT |  | 662 | -0.34 | -1.34 | 0.000 | 0.421 | 1.000 | 4002 | tags=37%, list=33%, signal=53% |
| 1589 | GOBP\_REGULATION\_OF\_AXON\_GUIDANCE |  | 10 | -0.56 | -1.34 | 0.123 | 0.421 | 1.000 | 4316 | tags=70%, list=36%, signal=109% |
| 1590 | GOBP\_DNA\_DEPENDENT\_DNA\_REPLICATION |  | 135 | -0.36 | -1.34 | 0.030 | 0.421 | 1.000 | 4146 | tags=44%, list=35%, signal=66% |
| 1591 | GOMF\_ONCOSTATIN\_M\_RECEPTOR\_ACTIVITY |  | 6 | -0.66 | -1.34 | 0.103 | 0.421 | 1.000 | 1472 | tags=33%, list=12%, signal=38% |
| 1592 | GOBP\_REGULATION\_OF\_NEURONAL\_SYNAPTIC\_PLASTICITY |  | 25 | -0.46 | -1.34 | 0.101 | 0.422 | 1.000 | 3833 | tags=48%, list=32%, signal=70% |
| 1593 | GOBP\_MULTICELLULAR\_ORGANISMAL\_MOVEMENT |  | 24 | -0.46 | -1.34 | 0.110 | 0.422 | 1.000 | 2458 | tags=33%, list=20%, signal=42% |
| 1594 | GOMF\_OXYSTEROL\_BINDING |  | 7 | -0.63 | -1.34 | 0.108 | 0.422 | 1.000 | 1393 | tags=43%, list=12%, signal=48% |
| 1595 | GOBP\_REGULATION\_OF\_CD4\_POSITIVE\_ALPHA\_BETA\_T\_CELL\_DIFFERENTIATION |  | 34 | -0.43 | -1.34 | 0.090 | 0.422 | 1.000 | 2345 | tags=38%, list=20%, signal=47% |
| 1596 | GOBP\_RETINA\_VASCULATURE\_MORPHOGENESIS\_IN\_CAMERA\_TYPE\_EYE |  | 9 | -0.59 | -1.34 | 0.117 | 0.422 | 1.000 | 4887 | tags=89%, list=41%, signal=150% |
| 1597 | GOBP\_REGULATION\_OF\_NEUROBLAST\_PROLIFERATION |  | 20 | -0.48 | -1.34 | 0.117 | 0.422 | 1.000 | 3565 | tags=50%, list=30%, signal=71% |
| 1598 | GOBP\_POSITIVE\_REGULATION\_OF\_CELL\_JUNCTION\_ASSEMBLY |  | 62 | -0.39 | -1.34 | 0.063 | 0.422 | 1.000 | 3196 | tags=39%, list=27%, signal=52% |
| 1599 | GOBP\_CELLULAR\_RESPONSE\_TO\_LEUCINE |  | 9 | -0.59 | -1.34 | 0.120 | 0.422 | 1.000 | 3375 | tags=44%, list=28%, signal=62% |
| 1600 | GOBP\_NEGATIVE\_REGULATION\_OF\_DNA\_BINDING\_TRANSCRIPTION\_FACTOR\_ACTIVITY |  | 109 | -0.37 | -1.34 | 0.043 | 0.422 | 1.000 | 2667 | tags=31%, list=22%, signal=40% |
| 1601 | GOBP\_REGULATION\_OF\_GROWTH\_HORMONE\_SECRETION |  | 6 | -0.66 | -1.34 | 0.108 | 0.421 | 1.000 | 2351 | tags=50%, list=20%, signal=62% |
| 1602 | GOBP\_DNA\_PACKAGING |  | 117 | -0.37 | -1.34 | 0.036 | 0.421 | 1.000 | 3624 | tags=42%, list=30%, signal=59% |
| 1603 | GOBP\_DNA\_CONFORMATION\_CHANGE |  | 208 | -0.35 | -1.34 | 0.015 | 0.421 | 1.000 | 5406 | tags=63%, list=45%, signal=112% |
| 1604 | GOCC\_POSTSYNAPTIC\_MEMBRANE |  | 132 | -0.36 | -1.34 | 0.031 | 0.421 | 1.000 | 3708 | tags=39%, list=31%, signal=55% |
| 1605 | GOBP\_HISTONE\_PHOSPHORYLATION |  | 29 | -0.44 | -1.34 | 0.092 | 0.421 | 1.000 | 3744 | tags=45%, list=31%, signal=65% |
| 1606 | GOMF\_FERRIC\_IRON\_BINDING |  | 6 | -0.66 | -1.34 | 0.108 | 0.421 | 1.000 | 1316 | tags=67%, list=11%, signal=75% |
| 1607 | GOMF\_SEQUENCE\_SPECIFIC\_DNA\_BINDING |  | 728 | -0.34 | -1.34 | 0.000 | 0.422 | 1.000 | 3907 | tags=41%, list=33%, signal=57% |
| 1608 | GOBP\_HOMOPHILIC\_CELL\_ADHESION\_VIA\_PLASMA\_MEMBRANE\_ADHESION\_MOLECULES |  | 76 | -0.38 | -1.34 | 0.061 | 0.422 | 1.000 | 4217 | tags=45%, list=35%, signal=69% |
| 1609 | GOBP\_PEPTIDYL\_AMINO\_ACID\_MODIFICATION |  | 900 | -0.34 | -1.33 | 0.000 | 0.422 | 1.000 | 4032 | tags=39%, list=34%, signal=55% |
| 1610 | GOBP\_SEMAPHORIN\_PLEXIN\_SIGNALING\_PATHWAY |  | 32 | -0.43 | -1.33 | 0.102 | 0.422 | 1.000 | 1816 | tags=28%, list=15%, signal=33% |
| 1611 | GOMF\_CORECEPTOR\_ACTIVITY |  | 37 | -0.42 | -1.33 | 0.088 | 0.422 | 1.000 | 4887 | tags=57%, list=41%, signal=95% |
| 1612 | GOCC\_SYNAPTIC\_MEMBRANE |  | 205 | -0.35 | -1.33 | 0.017 | 0.422 | 1.000 | 3796 | tags=40%, list=32%, signal=57% |
| 1613 | GOBP\_INOSITOL\_PHOSPHATE\_METABOLIC\_PROCESS |  | 55 | -0.40 | -1.33 | 0.071 | 0.422 | 1.000 | 3934 | tags=47%, list=33%, signal=70% |
| 1614 | GOBP\_POSITIVE\_REGULATION\_OF\_ALKALINE\_PHOSPHATASE\_ACTIVITY |  | 3 | -0.79 | -1.33 | 0.091 | 0.422 | 1.000 | 2474 | tags=100%, list=21%, signal=126% |
| 1615 | GOBP\_BLEB\_ASSEMBLY |  | 9 | -0.59 | -1.33 | 0.116 | 0.421 | 1.000 | 1048 | tags=33%, list=9%, signal=36% |
| 1616 | GOBP\_LENS\_FIBER\_CELL\_DEVELOPMENT |  | 9 | -0.59 | -1.33 | 0.118 | 0.421 | 1.000 | 1325 | tags=44%, list=11%, signal=50% |
| 1617 | GOBP\_PROTEIN\_POLYMERIZATION |  | 200 | -0.35 | -1.33 | 0.014 | 0.421 | 1.000 | 3187 | tags=34%, list=27%, signal=45% |
| 1618 | GOMF\_PROTEIN\_KINASE\_A\_BINDING |  | 38 | -0.43 | -1.33 | 0.091 | 0.421 | 1.000 | 4008 | tags=53%, list=33%, signal=79% |
| 1619 | GOCC\_TRANSCRIPTIONALLY\_ACTIVE\_CHROMATIN |  | 17 | -0.50 | -1.33 | 0.114 | 0.421 | 1.000 | 3128 | tags=47%, list=26%, signal=64% |
| 1620 | GOBP\_SISTER\_CHROMATID\_SEGREGATION |  | 159 | -0.36 | -1.33 | 0.025 | 0.421 | 1.000 | 4465 | tags=45%, list=37%, signal=70% |
| 1621 | GOBP\_PRODUCTION\_OF\_MOLECULAR\_MEDIATOR\_OF\_IMMUNE\_RESPONSE |  | 113 | -0.36 | -1.33 | 0.037 | 0.421 | 1.000 | 2660 | tags=35%, list=22%, signal=45% |
| 1622 | GOMF\_GATED\_CHANNEL\_ACTIVITY |  | 163 | -0.36 | -1.33 | 0.019 | 0.421 | 1.000 | 2216 | tags=27%, list=18%, signal=33% |
| 1623 | GOMF\_CYSTEINE\_TYPE\_ENDOPEPTIDASE\_INHIBITOR\_ACTIVITY\_INVOLVED\_IN\_APOPTOTIC\_PROCESS |  | 12 | -0.55 | -1.33 | 0.120 | 0.421 | 1.000 | 1559 | tags=17%, list=13%, signal=19% |
| 1624 | GOBP\_REGULATION\_OF\_PROTEIN\_POLYMERIZATION |  | 155 | -0.36 | -1.33 | 0.026 | 0.420 | 1.000 | 3069 | tags=32%, list=26%, signal=43% |
| 1625 | GOBP\_PHOSPHOLIPASE\_C\_ACTIVATING\_G\_PROTEIN\_COUPLED\_RECEPTOR\_SIGNALING\_PATHWAY |  | 58 | -0.40 | -1.33 | 0.069 | 0.420 | 1.000 | 2871 | tags=34%, list=24%, signal=45% |
| 1626 | GOBP\_TETRAHYDROFOLATE\_METABOLIC\_PROCESS |  | 15 | -0.52 | -1.33 | 0.114 | 0.420 | 1.000 | 4242 | tags=60%, list=35%, signal=93% |
| 1627 | GOMF\_PHOSPHATE\_ION\_TRANSMEMBRANE\_TRANSPORTER\_ACTIVITY |  | 6 | -0.66 | -1.33 | 0.112 | 0.421 | 1.000 | 1679 | tags=50%, list=14%, signal=58% |
| 1628 | GOBP\_CARDIAC\_ATRIUM\_MORPHOGENESIS |  | 17 | -0.49 | -1.33 | 0.116 | 0.420 | 1.000 | 3314 | tags=47%, list=28%, signal=65% |
| 1629 | GOMF\_PHOSPHOLIPID\_TRANSPORTER\_ACTIVITY |  | 35 | -0.43 | -1.33 | 0.088 | 0.421 | 1.000 | 3366 | tags=43%, list=28%, signal=59% |
| 1630 | GOMF\_SYNTAXIN\_BINDING |  | 59 | -0.40 | -1.33 | 0.066 | 0.422 | 1.000 | 3698 | tags=46%, list=31%, signal=66% |
| 1631 | GOMF\_POTASSIUM\_ION\_BINDING |  | 7 | -0.63 | -1.33 | 0.123 | 0.422 | 1.000 | 582 | tags=29%, list=5%, signal=30% |
| 1632 | GOMF\_PHOSPHATIDYLINOSITOL\_3\_5\_BISPHOSPHATE\_3\_PHOSPHATASE\_ACTIVITY |  | 7 | -0.63 | -1.33 | 0.113 | 0.422 | 1.000 | 3244 | tags=71%, list=27%, signal=98% |
| 1633 | GOBP\_CYTOSOLIC\_TRANSPORT |  | 126 | -0.36 | -1.33 | 0.036 | 0.422 | 1.000 | 4134 | tags=41%, list=34%, signal=62% |
| 1634 | GOMF\_CIS\_REGULATORY\_REGION\_SEQUENCE\_SPECIFIC\_DNA\_BINDING |  | 507 | -0.34 | -1.33 | 0.001 | 0.422 | 1.000 | 3907 | tags=42%, list=33%, signal=60% |
| 1635 | GOBP\_REGULATION\_OF\_DNA\_BINDING\_TRANSCRIPTION\_FACTOR\_ACTIVITY |  | 285 | -0.34 | -1.33 | 0.007 | 0.423 | 1.000 | 3285 | tags=34%, list=27%, signal=46% |
| 1636 | GOBP\_POSITIVE\_REGULATION\_OF\_NECROTIC\_CELL\_DEATH |  | 7 | -0.62 | -1.33 | 0.120 | 0.423 | 1.000 | 611 | tags=29%, list=5%, signal=30% |
| 1637 | GOBP\_CYTOKINE\_PRODUCTION |  | 444 | -0.34 | -1.33 | 0.001 | 0.423 | 1.000 | 3534 | tags=39%, list=29%, signal=54% |
| 1638 | GOBP\_FILOPODIUM\_ASSEMBLY |  | 49 | -0.41 | -1.33 | 0.083 | 0.423 | 1.000 | 3473 | tags=41%, list=29%, signal=57% |
| 1639 | GOCC\_CONDENSIN\_COMPLEX |  | 8 | -0.61 | -1.33 | 0.118 | 0.423 | 1.000 | 3228 | tags=75%, list=27%, signal=102% |
| 1640 | GOBP\_TOR\_SIGNALING |  | 93 | -0.37 | -1.33 | 0.051 | 0.423 | 1.000 | 3613 | tags=40%, list=30%, signal=56% |
| 1641 | GOBP\_BLOOD\_VESSEL\_ENDOTHELIAL\_CELL\_DIFFERENTIATION |  | 8 | -0.61 | -1.33 | 0.116 | 0.423 | 1.000 | 3314 | tags=50%, list=28%, signal=69% |
| 1642 | GOBP\_HISTONE\_MRNA\_METABOLIC\_PROCESS |  | 18 | -0.49 | -1.33 | 0.121 | 0.422 | 1.000 | 2545 | tags=33%, list=21%, signal=42% |
| 1643 | GOBP\_REGULATION\_OF\_TUMOR\_NECROSIS\_FACTOR\_MEDIATED\_SIGNALING\_PATHWAY |  | 35 | -0.43 | -1.33 | 0.095 | 0.422 | 1.000 | 2606 | tags=34%, list=22%, signal=44% |
| 1644 | GOCC\_CULLIN\_RING\_UBIQUITIN\_LIGASE\_COMPLEX |  | 125 | -0.36 | -1.33 | 0.038 | 0.422 | 1.000 | 4019 | tags=42%, list=33%, signal=62% |
| 1645 | GOBP\_MYELOID\_CELL\_HOMEOSTASIS |  | 112 | -0.36 | -1.33 | 0.043 | 0.422 | 1.000 | 4368 | tags=50%, list=36%, signal=78% |
| 1646 | GOBP\_REGULATION\_OF\_PROTEIN\_EXIT\_FROM\_ENDOPLASMIC\_RETICULUM |  | 22 | -0.47 | -1.33 | 0.112 | 0.422 | 1.000 | 2924 | tags=36%, list=24%, signal=48% |
| 1647 | GOBP\_REGULATION\_OF\_PHOSPHOPROTEIN\_PHOSPHATASE\_ACTIVITY |  | 78 | -0.38 | -1.33 | 0.052 | 0.422 | 1.000 | 4627 | tags=49%, list=39%, signal=79% |
| 1648 | GOBP\_REGULATION\_OF\_IMMUNE\_EFFECTOR\_PROCESS |  | 222 | -0.35 | -1.33 | 0.015 | 0.422 | 1.000 | 3487 | tags=41%, list=29%, signal=57% |
| 1649 | GOBP\_LYSOSOME\_LOCALIZATION |  | 55 | -0.40 | -1.33 | 0.067 | 0.422 | 1.000 | 1935 | tags=27%, list=16%, signal=32% |
| 1650 | GOBP\_REGULATION\_OF\_CHROMATIN\_ASSEMBLY |  | 11 | -0.55 | -1.33 | 0.126 | 0.422 | 1.000 | 1784 | tags=36%, list=15%, signal=43% |
| 1651 | GOBP\_REGULATION\_OF\_DEPHOSPHORYLATION |  | 145 | -0.36 | -1.33 | 0.031 | 0.422 | 1.000 | 4122 | tags=43%, list=34%, signal=65% |
| 1652 | GOBP\_MITOTIC\_CELL\_CYCLE\_CHECKPOINT |  | 127 | -0.36 | -1.33 | 0.035 | 0.422 | 1.000 | 4076 | tags=40%, list=34%, signal=60% |
| 1653 | GOBP\_WOUND\_HEALING |  | 354 | -0.34 | -1.33 | 0.003 | 0.422 | 1.000 | 3394 | tags=36%, list=28%, signal=48% |
| 1654 | GOBP\_POSITIVE\_REGULATION\_OF\_LEUKOCYTE\_CHEMOTAXIS |  | 60 | -0.39 | -1.33 | 0.071 | 0.422 | 1.000 | 3196 | tags=42%, list=27%, signal=56% |
| 1655 | GOCC\_MICROTUBULE\_CYTOSKELETON |  | 906 | -0.33 | -1.33 | 0.000 | 0.422 | 1.000 | 3852 | tags=37%, list=32%, signal=50% |
| 1656 | GOBP\_REGULATION\_OF\_MICROTUBULE\_BASED\_PROCESS |  | 158 | -0.35 | -1.33 | 0.025 | 0.422 | 1.000 | 3638 | tags=39%, list=30%, signal=56% |
| 1657 | GOMF\_CALCIUM\_ION\_BINDING |  | 390 | -0.34 | -1.33 | 0.002 | 0.423 | 1.000 | 3340 | tags=35%, list=28%, signal=46% |
| 1658 | GOBP\_POSITIVE\_REGULATION\_OF\_CARDIAC\_MUSCLE\_CELL\_PROLIFERATION |  | 15 | -0.51 | -1.33 | 0.125 | 0.422 | 1.000 | 3510 | tags=53%, list=29%, signal=75% |
| 1659 | GOBP\_CALCIUM\_ION\_IMPORT\_INTO\_CYTOSOL |  | 9 | -0.58 | -1.33 | 0.124 | 0.422 | 1.000 | 3313 | tags=44%, list=28%, signal=61% |
| 1660 | GOBP\_SUPRAMOLECULAR\_FIBER\_ORGANIZATION |  | 495 | -0.34 | -1.33 | 0.001 | 0.422 | 1.000 | 3578 | tags=36%, list=30%, signal=49% |
| 1661 | GOBP\_LATERAL\_MESODERM\_DEVELOPMENT |  | 6 | -0.65 | -1.33 | 0.111 | 0.422 | 1.000 | 3565 | tags=50%, list=30%, signal=71% |
| 1662 | GOBP\_SEROTONIN\_METABOLIC\_PROCESS |  | 5 | -0.68 | -1.33 | 0.111 | 0.423 | 1.000 | 2516 | tags=60%, list=21%, signal=76% |
| 1663 | GOBP\_NEGATIVE\_REGULATION\_OF\_SPROUTING\_ANGIOGENESIS |  | 9 | -0.58 | -1.33 | 0.124 | 0.423 | 1.000 | 3724 | tags=78%, list=31%, signal=113% |
| 1664 | GOBP\_CELLULAR\_RESPONSE\_TO\_PARATHYROID\_HORMONE\_STIMULUS |  | 3 | -0.79 | -1.33 | 0.094 | 0.423 | 1.000 | 2474 | tags=100%, list=21%, signal=126% |
| 1665 | GOMF\_PHOSPHATIDYLINOSITOL\_3\_4\_5\_TRISPHOSPHATE\_BINDING |  | 29 | -0.44 | -1.33 | 0.101 | 0.423 | 1.000 | 3769 | tags=55%, list=31%, signal=80% |
| 1666 | GOMF\_RNA\_POLYMERASE\_II\_INTRONIC\_TRANSCRIPTION\_REGULATORY\_REGION\_SEQUENCE\_SPECIFIC\_DNA\_BINDING |  | 3 | -0.79 | -1.33 | 0.091 | 0.423 | 1.000 | 522 | tags=33%, list=4%, signal=35% |
| 1667 | GOMF\_PROTEIN\_PHOSPHATASE\_BINDING |  | 109 | -0.37 | -1.33 | 0.046 | 0.423 | 1.000 | 3613 | tags=43%, list=30%, signal=61% |
| 1668 | GOBP\_POSITIVE\_REGULATION\_OF\_SYNAPTIC\_PLASTICITY |  | 8 | -0.60 | -1.33 | 0.129 | 0.424 | 1.000 | 1392 | tags=38%, list=12%, signal=42% |
| 1669 | GOBP\_NEGATIVE\_REGULATION\_OF\_DEPHOSPHORYLATION |  | 71 | -0.39 | -1.33 | 0.058 | 0.424 | 1.000 | 3547 | tags=41%, list=30%, signal=58% |
| 1670 | GOBP\_POSITIVE\_REGULATION\_OF\_NUCLEOTIDE\_METABOLIC\_PROCESS |  | 28 | -0.45 | -1.33 | 0.105 | 0.424 | 1.000 | 1748 | tags=29%, list=15%, signal=33% |
| 1671 | GOBP\_REGULATION\_OF\_NATURAL\_KILLER\_CELL\_DIFFERENTIATION |  | 7 | -0.62 | -1.33 | 0.110 | 0.425 | 1.000 | 3009 | tags=57%, list=25%, signal=76% |
| 1672 | GOBP\_TRYPTOPHAN\_CATABOLIC\_PROCESS |  | 5 | -0.69 | -1.33 | 0.109 | 0.425 | 1.000 | 2220 | tags=40%, list=18%, signal=49% |
| 1673 | GOBP\_REGULATION\_OF\_PROTEIN\_DEUBIQUITINATION |  | 9 | -0.58 | -1.33 | 0.119 | 0.425 | 1.000 | 4909 | tags=78%, list=41%, signal=131% |
| 1674 | GOBP\_CATECHOLAMINE\_SECRETION |  | 34 | -0.43 | -1.33 | 0.097 | 0.425 | 1.000 | 1699 | tags=24%, list=14%, signal=27% |
| 1675 | GOMF\_PHOSPHATIDYLCHOLINE\_TRANSPORTER\_ACTIVITY |  | 13 | -0.53 | -1.33 | 0.122 | 0.425 | 1.000 | 3366 | tags=54%, list=28%, signal=75% |
| 1676 | GOBP\_REGULATION\_OF\_MUSCLE\_CONTRACTION |  | 97 | -0.37 | -1.33 | 0.051 | 0.425 | 1.000 | 1890 | tags=24%, list=16%, signal=28% |
| 1677 | GOBP\_NEGATIVE\_REGULATION\_OF\_MYELOID\_CELL\_DIFFERENTIATION |  | 53 | -0.40 | -1.33 | 0.077 | 0.425 | 1.000 | 4846 | tags=53%, list=40%, signal=88% |
| 1678 | GOCC\_SYNAPSE |  | 827 | -0.33 | -1.33 | 0.000 | 0.425 | 1.000 | 4052 | tags=39%, list=34%, signal=55% |
| 1679 | GOMF\_CYCLIC\_NUCLEOTIDE\_GATED\_ION\_CHANNEL\_ACTIVITY |  | 3 | -0.80 | -1.33 | 0.096 | 0.425 | 1.000 | 748 | tags=67%, list=6%, signal=71% |
| 1680 | GOMF\_STRUCTURAL\_CONSTITUENT\_OF\_SYNAPSE |  | 8 | -0.60 | -1.33 | 0.121 | 0.425 | 1.000 | 4455 | tags=75%, list=37%, signal=119% |
| 1681 | GOBP\_MECHANOSENSORY\_BEHAVIOR |  | 5 | -0.69 | -1.33 | 0.105 | 0.425 | 1.000 | 3431 | tags=80%, list=29%, signal=112% |
| 1682 | GOBP\_POSITIVE\_REGULATION\_OF\_NEUROGENESIS |  | 157 | -0.35 | -1.33 | 0.027 | 0.425 | 1.000 | 4698 | tags=48%, list=39%, signal=78% |
| 1683 | GOBP\_ATRIOVENTRICULAR\_VALVE\_FORMATION |  | 6 | -0.66 | -1.33 | 0.113 | 0.424 | 1.000 | 3314 | tags=67%, list=28%, signal=92% |
| 1684 | GOCC\_FLEMMING\_BODY |  | 28 | -0.44 | -1.33 | 0.110 | 0.424 | 1.000 | 3125 | tags=43%, list=26%, signal=58% |
| 1685 | GOBP\_MONOAMINE\_TRANSPORT |  | 54 | -0.40 | -1.33 | 0.080 | 0.424 | 1.000 | 2633 | tags=30%, list=22%, signal=38% |
| 1686 | GOCC\_DEATH\_INDUCING\_SIGNALING\_COMPLEX |  | 8 | -0.61 | -1.33 | 0.118 | 0.424 | 1.000 | 2494 | tags=63%, list=21%, signal=79% |
| 1687 | GOCC\_CATENIN\_COMPLEX |  | 22 | -0.46 | -1.33 | 0.110 | 0.424 | 1.000 | 4033 | tags=50%, list=34%, signal=75% |
| 1688 | GOBP\_OLIGOSACCHARIDE\_CATABOLIC\_PROCESS |  | 7 | -0.63 | -1.33 | 0.113 | 0.425 | 1.000 | 4074 | tags=71%, list=34%, signal=108% |
| 1689 | GOBP\_CD4\_POSITIVE\_ALPHA\_BETA\_T\_CELL\_ACTIVATION |  | 61 | -0.39 | -1.33 | 0.075 | 0.425 | 1.000 | 2345 | tags=36%, list=20%, signal=45% |
| 1690 | GOMF\_CAMP\_RESPONSE\_ELEMENT\_BINDING |  | 14 | -0.52 | -1.33 | 0.122 | 0.425 | 1.000 | 5643 | tags=86%, list=47%, signal=161% |
| 1691 | GOBP\_ATRIAL\_SEPTUM\_MORPHOGENESIS |  | 9 | -0.58 | -1.33 | 0.131 | 0.425 | 1.000 | 3314 | tags=44%, list=28%, signal=61% |
| 1692 | GOBP\_EAR\_DEVELOPMENT |  | 123 | -0.36 | -1.33 | 0.044 | 0.425 | 1.000 | 3565 | tags=41%, list=30%, signal=57% |
| 1693 | GOMF\_MIRNA\_BINDING |  | 22 | -0.47 | -1.33 | 0.116 | 0.425 | 1.000 | 4270 | tags=59%, list=36%, signal=92% |
| 1694 | GOBP\_REGIONALIZATION |  | 190 | -0.35 | -1.33 | 0.019 | 0.424 | 1.000 | 3126 | tags=33%, list=26%, signal=44% |
| 1695 | GOCC\_CONTRACTILE\_RING |  | 9 | -0.58 | -1.33 | 0.123 | 0.424 | 1.000 | 1843 | tags=33%, list=15%, signal=39% |
| 1696 | GOBP\_NEGATIVE\_REGULATION\_OF\_RESPONSE\_TO\_WOUNDING |  | 54 | -0.40 | -1.33 | 0.076 | 0.424 | 1.000 | 2820 | tags=35%, list=23%, signal=46% |
| 1697 | GOBP\_RIGHTING\_REFLEX |  | 4 | -0.73 | -1.33 | 0.098 | 0.424 | 1.000 | 1222 | tags=50%, list=10%, signal=56% |
| 1698 | GOBP\_LYSOSOMAL\_TRANSPORT |  | 88 | -0.37 | -1.33 | 0.052 | 0.424 | 1.000 | 3227 | tags=36%, list=27%, signal=49% |
| 1699 | GOBP\_NEGATIVE\_REGULATION\_OF\_PROTEIN\_CONTAINING\_COMPLEX\_DISASSEMBLY |  | 57 | -0.39 | -1.33 | 0.078 | 0.424 | 1.000 | 3148 | tags=37%, list=26%, signal=50% |
| 1700 | GOBP\_PROTEIN\_O\_LINKED\_GLYCOSYLATION |  | 63 | -0.39 | -1.33 | 0.070 | 0.424 | 1.000 | 2617 | tags=32%, list=22%, signal=40% |
| 1701 | GOBP\_NEUTROPHIL\_HOMEOSTASIS |  | 10 | -0.57 | -1.33 | 0.128 | 0.424 | 1.000 | 1903 | tags=50%, list=16%, signal=59% |
| 1702 | GOBP\_POSITIVE\_REGULATION\_OF\_PHOSPHOPROTEIN\_PHOSPHATASE\_ACTIVITY |  | 16 | -0.50 | -1.33 | 0.126 | 0.424 | 1.000 | 4627 | tags=56%, list=39%, signal=91% |
| 1703 | GOMF\_OXIDOREDUCTASE\_ACTIVITY\_ACTING\_ON\_PAIRED\_DONORS\_WITH\_INCORPORATION\_OR\_REDUCTION\_OF\_MOLECULAR\_OXYGEN |  | 94 | -0.37 | -1.33 | 0.048 | 0.423 | 1.000 | 2716 | tags=33%, list=23%, signal=42% |
| 1704 | GOBP\_TRANSFORMING\_GROWTH\_FACTOR\_BETA\_ACTIVATION |  | 10 | -0.57 | -1.33 | 0.124 | 0.424 | 1.000 | 3064 | tags=60%, list=26%, signal=80% |
| 1705 | GOBP\_G0\_TO\_G1\_TRANSITION |  | 35 | -0.43 | -1.33 | 0.095 | 0.424 | 1.000 | 5049 | tags=63%, list=42%, signal=108% |
| 1706 | GOBP\_PURINE\_NUCLEOBASE\_METABOLIC\_PROCESS |  | 13 | -0.53 | -1.33 | 0.126 | 0.424 | 1.000 | 2929 | tags=54%, list=24%, signal=71% |
| 1707 | GOBP\_ESTABLISHMENT\_OF\_SPINDLE\_ORIENTATION |  | 30 | -0.44 | -1.33 | 0.103 | 0.424 | 1.000 | 3834 | tags=43%, list=32%, signal=63% |
| 1708 | GOBP\_REGULATION\_OF\_DNA\_RECOMBINATION |  | 66 | -0.39 | -1.33 | 0.071 | 0.424 | 1.000 | 3434 | tags=44%, list=29%, signal=61% |
| 1709 | GOBP\_VASOCONSTRICTION |  | 62 | -0.39 | -1.33 | 0.072 | 0.424 | 1.000 | 1799 | tags=27%, list=15%, signal=32% |
| 1710 | GOMF\_NOTCH\_BINDING |  | 18 | -0.49 | -1.33 | 0.126 | 0.425 | 1.000 | 2255 | tags=33%, list=19%, signal=41% |
| 1711 | GOBP\_IMMUNE\_RESPONSE\_TO\_TUMOR\_CELL |  | 7 | -0.62 | -1.33 | 0.119 | 0.425 | 1.000 | 3240 | tags=57%, list=27%, signal=78% |
| 1712 | GOBP\_CYTOLYSIS |  | 12 | -0.54 | -1.32 | 0.131 | 0.425 | 1.000 | 4135 | tags=42%, list=34%, signal=63% |
| 1713 | GOBP\_REGULATION\_OF\_PROTEIN\_LOCALIZATION\_TO\_CELL\_SURFACE |  | 25 | -0.46 | -1.32 | 0.104 | 0.425 | 1.000 | 3814 | tags=48%, list=32%, signal=70% |
| 1714 | GOBP\_OSTEOCLAST\_DIFFERENTIATION |  | 54 | -0.40 | -1.32 | 0.082 | 0.426 | 1.000 | 1999 | tags=35%, list=17%, signal=42% |
| 1715 | GOBP\_MIRNA\_METABOLIC\_PROCESS |  | 17 | -0.50 | -1.32 | 0.120 | 0.426 | 1.000 | 2666 | tags=47%, list=22%, signal=60% |
| 1716 | GOBP\_DNA\_REPLICATION\_INDEPENDENT\_NUCLEOSOME\_ORGANIZATION |  | 36 | -0.42 | -1.32 | 0.096 | 0.426 | 1.000 | 4440 | tags=53%, list=37%, signal=83% |
| 1717 | GOBP\_ACTIN\_FILAMENT\_BASED\_TRANSPORT |  | 19 | -0.48 | -1.32 | 0.116 | 0.426 | 1.000 | 4456 | tags=53%, list=37%, signal=84% |
| 1718 | GOBP\_NEGATIVE\_REGULATION\_OF\_GENE\_EXPRESSION\_EPIGENETIC |  | 44 | -0.41 | -1.32 | 0.092 | 0.426 | 1.000 | 4649 | tags=52%, list=39%, signal=85% |
| 1719 | GOBP\_SENSORY\_SYSTEM\_DEVELOPMENT |  | 222 | -0.35 | -1.32 | 0.017 | 0.425 | 1.000 | 3638 | tags=37%, list=30%, signal=52% |
| 1720 | GOBP\_NEGATIVE\_REGULATION\_OF\_T\_CELL\_MEDIATED\_IMMUNITY |  | 6 | -0.65 | -1.32 | 0.118 | 0.425 | 1.000 | 1519 | tags=67%, list=13%, signal=76% |
| 1721 | GOCC\_EARLY\_ENDOSOME\_MEMBRANE |  | 108 | -0.36 | -1.32 | 0.042 | 0.426 | 1.000 | 3401 | tags=33%, list=28%, signal=46% |
| 1722 | GOBP\_POSITIVE\_REGULATION\_OF\_SARCOMERE\_ORGANIZATION |  | 3 | -0.79 | -1.32 | 0.091 | 0.426 | 1.000 | 1840 | tags=67%, list=15%, signal=79% |
| 1723 | GOBP\_CELL\_CYCLE\_G2\_M\_PHASE\_TRANSITION |  | 213 | -0.35 | -1.32 | 0.015 | 0.426 | 1.000 | 5379 | tags=51%, list=45%, signal=90% |
| 1724 | GOMF\_G\_PROTEIN\_BETA\_GAMMA\_SUBUNIT\_COMPLEX\_BINDING |  | 12 | -0.54 | -1.32 | 0.122 | 0.426 | 1.000 | 4450 | tags=67%, list=37%, signal=106% |
| 1725 | GOBP\_CHOLINE\_CATABOLIC\_PROCESS |  | 3 | -0.79 | -1.32 | 0.087 | 0.426 | 1.000 | 2534 | tags=100%, list=21%, signal=127% |
| 1726 | GOMF\_FOUR\_WAY\_JUNCTION\_HELICASE\_ACTIVITY |  | 5 | -0.69 | -1.32 | 0.110 | 0.426 | 1.000 | 3778 | tags=100%, list=31%, signal=146% |
| 1727 | GOBP\_NEGATIVE\_REGULATION\_OF\_OSTEOCLAST\_DIFFERENTIATION |  | 14 | -0.52 | -1.32 | 0.125 | 0.425 | 1.000 | 1579 | tags=43%, list=13%, signal=49% |
| 1728 | GOBP\_EMBRYO\_DEVELOPMENT\_ENDING\_IN\_BIRTH\_OR\_EGG\_HATCHING |  | 412 | -0.34 | -1.32 | 0.003 | 0.426 | 1.000 | 4247 | tags=41%, list=35%, signal=61% |
| 1729 | GOCC\_GAMMA\_TUBULIN\_LARGE\_COMPLEX |  | 6 | -0.65 | -1.32 | 0.119 | 0.426 | 1.000 | 2001 | tags=67%, list=17%, signal=80% |
| 1730 | GOBP\_MITOTIC\_RECOMBINATION |  | 18 | -0.48 | -1.32 | 0.115 | 0.425 | 1.000 | 5365 | tags=72%, list=45%, signal=130% |
| 1731 | GOCC\_NUCLEOTIDE\_EXCISION\_REPAIR\_COMPLEX |  | 9 | -0.58 | -1.32 | 0.122 | 0.425 | 1.000 | 3667 | tags=78%, list=31%, signal=112% |
| 1732 | GOBP\_REGULATION\_OF\_B\_CELL\_ACTIVATION |  | 81 | -0.37 | -1.32 | 0.064 | 0.425 | 1.000 | 3326 | tags=41%, list=28%, signal=56% |
| 1733 | GOBP\_POSITIVE\_REGULATION\_OF\_PROTEIN\_DEPOLYMERIZATION |  | 13 | -0.53 | -1.32 | 0.122 | 0.426 | 1.000 | 2480 | tags=31%, list=21%, signal=39% |
| 1734 | GOBP\_POSITIVE\_REGULATION\_OF\_CELL\_CYCLE\_CHECKPOINT |  | 9 | -0.58 | -1.32 | 0.129 | 0.426 | 1.000 | 3655 | tags=56%, list=30%, signal=80% |
| 1735 | GOCC\_CELL\_SURFACE |  | 478 | -0.34 | -1.32 | 0.001 | 0.427 | 1.000 | 3201 | tags=34%, list=27%, signal=45% |
| 1736 | GOBP\_INTERLEUKIN\_6\_PRODUCTION |  | 75 | -0.38 | -1.32 | 0.069 | 0.427 | 1.000 | 2480 | tags=31%, list=21%, signal=38% |
| 1737 | GOBP\_MITOTIC\_NUCLEAR\_ENVELOPE\_DISASSEMBLY |  | 10 | -0.57 | -1.32 | 0.135 | 0.427 | 1.000 | 4378 | tags=70%, list=36%, signal=110% |
| 1738 | GOBP\_SCHWANN\_CELL\_DIFFERENTIATION |  | 28 | -0.44 | -1.32 | 0.106 | 0.427 | 1.000 | 4548 | tags=50%, list=38%, signal=80% |
| 1739 | GOCC\_NUCLEAR\_INCLUSION\_BODY |  | 10 | -0.56 | -1.32 | 0.122 | 0.427 | 1.000 | 3019 | tags=50%, list=25%, signal=67% |
| 1740 | GOBP\_PURINE\_DEOXYRIBONUCLEOTIDE\_CATABOLIC\_PROCESS |  | 5 | -0.68 | -1.32 | 0.122 | 0.426 | 1.000 | 3094 | tags=80%, list=26%, signal=108% |
| 1741 | GOMF\_NEUROPEPTIDE\_RECEPTOR\_BINDING |  | 16 | -0.50 | -1.32 | 0.121 | 0.426 | 1.000 | 4164 | tags=69%, list=35%, signal=105% |
| 1742 | GOBP\_AXO\_DENDRITIC\_TRANSPORT |  | 56 | -0.40 | -1.32 | 0.080 | 0.426 | 1.000 | 3834 | tags=45%, list=32%, signal=65% |
| 1743 | GOMF\_UBIQUITIN\_LIKE\_PROTEIN\_TRANSFERASE\_ACTIVITY |  | 288 | -0.34 | -1.32 | 0.008 | 0.426 | 1.000 | 4645 | tags=47%, list=39%, signal=75% |
| 1744 | GOCC\_AP\_1\_ADAPTOR\_COMPLEX |  | 3 | -0.79 | -1.32 | 0.093 | 0.427 | 1.000 | 1825 | tags=67%, list=15%, signal=79% |
| 1745 | GOBP\_CELL\_CELL\_JUNCTION\_ORGANIZATION |  | 142 | -0.35 | -1.32 | 0.033 | 0.427 | 1.000 | 4548 | tags=50%, list=38%, signal=80% |
| 1746 | GOCC\_ACTIN\_BASED\_CELL\_PROJECTION |  | 144 | -0.36 | -1.32 | 0.037 | 0.427 | 1.000 | 3522 | tags=41%, list=29%, signal=57% |
| 1747 | GOBP\_REGULATION\_OF\_VESICLE\_MEDIATED\_TRANSPORT |  | 356 | -0.34 | -1.32 | 0.004 | 0.427 | 1.000 | 3062 | tags=31%, list=25%, signal=41% |
| 1748 | GOBP\_EOSINOPHIL\_ACTIVATION |  | 4 | -0.74 | -1.32 | 0.105 | 0.427 | 1.000 | 2480 | tags=75%, list=21%, signal=94% |
| 1749 | GOMF\_TRANSFERASE\_ACTIVITY\_TRANSFERRING\_HEXOSYL\_GROUPS |  | 132 | -0.36 | -1.32 | 0.036 | 0.427 | 1.000 | 4291 | tags=46%, list=36%, signal=71% |
| 1750 | GOBP\_POSITIVE\_REGULATION\_OF\_STEROL\_TRANSPORT |  | 20 | -0.47 | -1.32 | 0.125 | 0.428 | 1.000 | 3789 | tags=45%, list=32%, signal=66% |
| 1751 | GOBP\_POSITIVE\_REGULATION\_OF\_CD8\_POSITIVE\_ALPHA\_BETA\_T\_CELL\_DIFFERENTIATION |  | 4 | -0.73 | -1.32 | 0.108 | 0.428 | 1.000 | 1601 | tags=50%, list=13%, signal=58% |
| 1752 | GOMF\_GLUCOSIDASE\_ACTIVITY |  | 9 | -0.58 | -1.32 | 0.129 | 0.428 | 1.000 | 3388 | tags=67%, list=28%, signal=93% |
| 1753 | GOMF\_PHOSPHATIDYLINOSITOL\_TRANSFER\_ACTIVITY |  | 7 | -0.62 | -1.32 | 0.128 | 0.428 | 1.000 | 846 | tags=29%, list=7%, signal=31% |
| 1754 | GOBP\_MITOTIC\_SISTER\_CHROMATID\_SEGREGATION |  | 133 | -0.36 | -1.32 | 0.036 | 0.428 | 1.000 | 4443 | tags=44%, list=37%, signal=68% |
| 1755 | GOBP\_PROTEIN\_EXIT\_FROM\_ENDOPLASMIC\_RETICULUM |  | 39 | -0.42 | -1.32 | 0.097 | 0.429 | 1.000 | 3039 | tags=36%, list=25%, signal=48% |
| 1756 | GOMF\_SODIUM\_ION\_TRANSMEMBRANE\_TRANSPORTER\_ACTIVITY |  | 81 | -0.38 | -1.32 | 0.063 | 0.429 | 1.000 | 1302 | tags=27%, list=11%, signal=30% |
| 1757 | GOBP\_NEGATIVE\_REGULATION\_OF\_CYSTEINE\_TYPE\_ENDOPEPTIDASE\_ACTIVITY\_INVOLVED\_IN\_APOPTOTIC\_SIGNALING\_PATHWAY |  | 4 | -0.73 | -1.32 | 0.097 | 0.429 | 1.000 | 3061 | tags=75%, list=25%, signal=101% |
| 1758 | GOBP\_RESPONSE\_TO\_AUDITORY\_STIMULUS |  | 11 | -0.55 | -1.32 | 0.128 | 0.429 | 1.000 | 3431 | tags=82%, list=29%, signal=114% |
| 1759 | GOBP\_ONCOSTATIN\_M\_MEDIATED\_SIGNALING\_PATHWAY |  | 6 | -0.66 | -1.32 | 0.110 | 0.429 | 1.000 | 1472 | tags=33%, list=12%, signal=38% |
| 1760 | GOMF\_POSTSYNAPTIC\_NEUROTRANSMITTER\_RECEPTOR\_ACTIVITY |  | 17 | -0.50 | -1.32 | 0.127 | 0.429 | 1.000 | 929 | tags=29%, list=8%, signal=32% |
| 1761 | GOBP\_PHOSPHOLIPID\_BIOSYNTHETIC\_PROCESS |  | 213 | -0.35 | -1.32 | 0.016 | 0.429 | 1.000 | 4540 | tags=46%, list=38%, signal=73% |
| 1762 | GOMF\_MITOGEN\_ACTIVATED\_PROTEIN\_KINASE\_KINASE\_KINASE\_BINDING |  | 15 | -0.51 | -1.32 | 0.131 | 0.429 | 1.000 | 1781 | tags=33%, list=15%, signal=39% |
| 1763 | GOBP\_CARNITINE\_TRANSPORT |  | 3 | -0.79 | -1.32 | 0.092 | 0.429 | 1.000 | 2557 | tags=100%, list=21%, signal=127% |
| 1764 | GOBP\_ALANINE\_CATABOLIC\_PROCESS |  | 3 | -0.79 | -1.32 | 0.103 | 0.429 | 1.000 | 2302 | tags=67%, list=19%, signal=82% |
| 1765 | GOBP\_DNA\_METHYLATION\_OR\_DEMETHYLATION |  | 57 | -0.39 | -1.32 | 0.079 | 0.430 | 1.000 | 4130 | tags=46%, list=34%, signal=69% |
| 1766 | GOBP\_FOLIC\_ACID\_METABOLIC\_PROCESS |  | 14 | -0.51 | -1.32 | 0.133 | 0.430 | 1.000 | 4242 | tags=57%, list=35%, signal=88% |
| 1767 | GOBP\_ENDOCYTOSIS |  | 393 | -0.34 | -1.32 | 0.002 | 0.430 | 1.000 | 3059 | tags=33%, list=25%, signal=42% |
| 1768 | GOBP\_CELLULAR\_RESPONSE\_TO\_VITAMIN\_D |  | 16 | -0.50 | -1.32 | 0.127 | 0.431 | 1.000 | 1579 | tags=38%, list=13%, signal=43% |
| 1769 | GOBP\_PARAXIAL\_MESODERM\_FORMATION |  | 3 | -0.78 | -1.32 | 0.098 | 0.431 | 1.000 | 1778 | tags=67%, list=15%, signal=78% |
| 1770 | GOBP\_MRNA\_3\_END\_PROCESSING\_BY\_STEM\_LOOP\_BINDING\_AND\_CLEAVAGE |  | 4 | -0.72 | -1.32 | 0.111 | 0.430 | 1.000 | 427 | tags=25%, list=4%, signal=26% |
| 1771 | GOBP\_ASTROCYTE\_ACTIVATION |  | 16 | -0.50 | -1.32 | 0.132 | 0.430 | 1.000 | 597 | tags=25%, list=5%, signal=26% |
| 1772 | GOCC\_SPINDLE\_MIDZONE |  | 30 | -0.43 | -1.32 | 0.110 | 0.430 | 1.000 | 1540 | tags=20%, list=13%, signal=23% |
| 1773 | GOBP\_PEPTIDYL\_LYSINE\_MODIFICATION |  | 294 | -0.34 | -1.32 | 0.008 | 0.430 | 1.000 | 4698 | tags=48%, list=39%, signal=77% |
| 1774 | GOBP\_REGULATION\_OF\_RESPONSE\_TO\_EXTERNAL\_STIMULUS |  | 612 | -0.33 | -1.32 | 0.001 | 0.431 | 1.000 | 3551 | tags=37%, list=30%, signal=49% |
| 1775 | GOCC\_BLEB |  | 9 | -0.57 | -1.32 | 0.122 | 0.431 | 1.000 | 1519 | tags=33%, list=13%, signal=38% |
| 1776 | GOCC\_INTRINSIC\_COMPONENT\_OF\_PLASMA\_MEMBRANE |  | 918 | -0.33 | -1.32 | 0.000 | 0.432 | 1.000 | 3754 | tags=38%, list=31%, signal=52% |
| 1777 | GOBP\_REGULATION\_OF\_NUCLEOCYTOPLASMIC\_TRANSPORT |  | 85 | -0.37 | -1.32 | 0.059 | 0.432 | 1.000 | 4032 | tags=40%, list=34%, signal=60% |
| 1778 | GOMF\_KINASE\_INHIBITOR\_ACTIVITY |  | 53 | -0.40 | -1.32 | 0.082 | 0.432 | 1.000 | 3715 | tags=42%, list=31%, signal=60% |
| 1779 | GOBP\_PHOSPHATIDYLETHANOLAMINE\_METABOLIC\_PROCESS |  | 19 | -0.48 | -1.32 | 0.121 | 0.433 | 1.000 | 4166 | tags=58%, list=35%, signal=88% |
| 1780 | GOBP\_PRIMARY\_NEURAL\_TUBE\_FORMATION |  | 67 | -0.38 | -1.32 | 0.076 | 0.433 | 1.000 | 4242 | tags=42%, list=35%, signal=64% |
| 1781 | GOCC\_EXTRINSIC\_COMPONENT\_OF\_SYNAPTIC\_MEMBRANE |  | 9 | -0.58 | -1.32 | 0.130 | 0.433 | 1.000 | 1298 | tags=33%, list=11%, signal=37% |
| 1782 | GOCC\_PHAGOPHORE\_ASSEMBLY\_SITE\_MEMBRANE |  | 12 | -0.53 | -1.32 | 0.128 | 0.433 | 1.000 | 3566 | tags=58%, list=30%, signal=83% |
| 1783 | GOCC\_DENDRITIC\_GROWTH\_CONE |  | 8 | -0.60 | -1.32 | 0.124 | 0.433 | 1.000 | 2010 | tags=50%, list=17%, signal=60% |
| 1784 | GOBP\_POSITIVE\_REGULATION\_OF\_IMMUNE\_SYSTEM\_PROCESS |  | 568 | -0.33 | -1.32 | 0.000 | 0.433 | 1.000 | 3377 | tags=36%, list=28%, signal=48% |
| 1785 | GOBP\_LIMBIC\_SYSTEM\_DEVELOPMENT |  | 58 | -0.39 | -1.32 | 0.077 | 0.433 | 1.000 | 647 | tags=16%, list=5%, signal=16% |
| 1786 | GOBP\_CENTRAL\_NERVOUS\_SYSTEM\_NEURON\_DIFFERENTIATION |  | 116 | -0.36 | -1.32 | 0.048 | 0.433 | 1.000 | 3425 | tags=34%, list=29%, signal=48% |
| 1787 | GOBP\_HEART\_MORPHOGENESIS |  | 157 | -0.35 | -1.32 | 0.029 | 0.433 | 1.000 | 3348 | tags=31%, list=28%, signal=43% |
| 1788 | GOMF\_SCAFFOLD\_PROTEIN\_BINDING |  | 42 | -0.41 | -1.32 | 0.090 | 0.433 | 1.000 | 2466 | tags=31%, list=21%, signal=39% |
| 1789 | GOBP\_NEURONAL\_ACTION\_POTENTIAL |  | 16 | -0.50 | -1.32 | 0.125 | 0.433 | 1.000 | 4110 | tags=44%, list=34%, signal=66% |
| 1790 | GOBP\_REGENERATION |  | 135 | -0.36 | -1.32 | 0.039 | 0.433 | 1.000 | 2518 | tags=29%, list=21%, signal=36% |
| 1791 | GOCC\_SYNAPTONEMAL\_STRUCTURE |  | 19 | -0.48 | -1.32 | 0.130 | 0.433 | 1.000 | 3241 | tags=47%, list=27%, signal=65% |
| 1792 | GOCC\_ANCHORING\_JUNCTION |  | 617 | -0.33 | -1.32 | 0.000 | 0.433 | 1.000 | 3089 | tags=29%, list=26%, signal=37% |
| 1793 | GOMF\_CALMODULIN\_DEPENDENT\_PROTEIN\_PHOSPHATASE\_ACTIVITY |  | 4 | -0.73 | -1.32 | 0.112 | 0.433 | 1.000 | 3072 | tags=75%, list=26%, signal=101% |
| 1794 | GOBP\_STRESS\_GRANULE\_ASSEMBLY |  | 18 | -0.49 | -1.32 | 0.128 | 0.433 | 1.000 | 5195 | tags=78%, list=43%, signal=137% |
| 1795 | GOBP\_SMOOTH\_MUSCLE\_CELL\_CHEMOTAXIS |  | 6 | -0.65 | -1.32 | 0.121 | 0.433 | 1.000 | 3286 | tags=50%, list=27%, signal=69% |
| 1796 | GOMF\_I\_SMAD\_BINDING |  | 12 | -0.54 | -1.32 | 0.138 | 0.433 | 1.000 | 4519 | tags=67%, list=38%, signal=107% |
| 1797 | GOBP\_GLUCOCORTICOID\_SECRETION |  | 5 | -0.68 | -1.32 | 0.122 | 0.433 | 1.000 | 2367 | tags=60%, list=20%, signal=75% |
| 1798 | GOBP\_REGULATION\_OF\_HEART\_GROWTH |  | 41 | -0.41 | -1.31 | 0.101 | 0.434 | 1.000 | 3510 | tags=46%, list=29%, signal=65% |
| 1799 | GOBP\_HINDBRAIN\_RADIAL\_GLIA\_GUIDED\_CELL\_MIGRATION |  | 7 | -0.62 | -1.31 | 0.131 | 0.436 | 1.000 | 3889 | tags=71%, list=32%, signal=106% |
| 1800 | GOBP\_PIGMENT\_CELL\_DIFFERENTIATION |  | 26 | -0.45 | -1.31 | 0.116 | 0.436 | 1.000 | 2911 | tags=46%, list=24%, signal=61% |
| 1801 | GOBP\_NATURAL\_KILLER\_CELL\_ACTIVATION\_INVOLVED\_IN\_IMMUNE\_RESPONSE |  | 7 | -0.62 | -1.31 | 0.128 | 0.436 | 1.000 | 3009 | tags=43%, list=25%, signal=57% |
| 1802 | GOBP\_GRANZYME\_MEDIATED\_PROGRAMMED\_CELL\_DEATH\_SIGNALING\_PATHWAY |  | 5 | -0.68 | -1.31 | 0.120 | 0.436 | 1.000 | 3656 | tags=80%, list=30%, signal=115% |
| 1803 | GOMF\_SNARE\_BINDING |  | 89 | -0.37 | -1.31 | 0.059 | 0.436 | 1.000 | 3815 | tags=43%, list=32%, signal=62% |
| 1804 | GOBP\_NEGATIVE\_REGULATION\_OF\_VASCULAR\_PERMEABILITY |  | 13 | -0.52 | -1.31 | 0.134 | 0.436 | 1.000 | 719 | tags=23%, list=6%, signal=25% |
| 1805 | GOBP\_CELL\_CELL\_ADHESION\_MEDIATED\_BY\_INTEGRIN |  | 15 | -0.51 | -1.31 | 0.126 | 0.437 | 1.000 | 1944 | tags=47%, list=16%, signal=56% |
| 1806 | GOBP\_POSITIVE\_REGULATION\_OF\_IMMUNE\_RESPONSE |  | 376 | -0.34 | -1.31 | 0.006 | 0.437 | 1.000 | 3240 | tags=35%, list=27%, signal=46% |
| 1807 | GOBP\_CELLULAR\_RESPONSE\_TO\_OXIDISED\_LOW\_DENSITY\_LIPOPROTEIN\_PARTICLE\_STIMULUS |  | 7 | -0.62 | -1.31 | 0.131 | 0.437 | 1.000 | 4433 | tags=86%, list=37%, signal=136% |
| 1808 | GOBP\_REPLACEMENT\_OSSIFICATION |  | 21 | -0.47 | -1.31 | 0.125 | 0.437 | 1.000 | 4433 | tags=52%, list=37%, signal=83% |
| 1809 | GOBP\_POSITIVE\_REGULATION\_OF\_FEEDING\_BEHAVIOR |  | 3 | -0.79 | -1.31 | 0.101 | 0.438 | 1.000 | 1935 | tags=67%, list=16%, signal=79% |
| 1810 | GOBP\_NEGATIVE\_REGULATION\_OF\_CELL\_SUBSTRATE\_ADHESION |  | 47 | -0.40 | -1.31 | 0.093 | 0.438 | 1.000 | 3575 | tags=40%, list=30%, signal=57% |
| 1811 | GOBP\_REGULATION\_OF\_ACTIN\_FILAMENT\_BUNDLE\_ASSEMBLY |  | 80 | -0.37 | -1.31 | 0.074 | 0.438 | 1.000 | 2560 | tags=34%, list=21%, signal=43% |
| 1812 | GOCC\_APICAL\_PART\_OF\_CELL |  | 277 | -0.34 | -1.31 | 0.011 | 0.438 | 1.000 | 3033 | tags=38%, list=25%, signal=50% |
| 1813 | GOBP\_REGULATION\_OF\_WNT\_SIGNALING\_PATHWAY\_PLANAR\_CELL\_POLARITY\_PATHWAY |  | 11 | -0.54 | -1.31 | 0.141 | 0.438 | 1.000 | 1991 | tags=36%, list=17%, signal=44% |
| 1814 | GOBP\_ETHANOLAMINE\_CONTAINING\_COMPOUND\_METABOLIC\_PROCESS |  | 6 | -0.65 | -1.31 | 0.131 | 0.438 | 1.000 | 3669 | tags=67%, list=31%, signal=96% |
| 1815 | GOBP\_POSITIVE\_REGULATION\_OF\_PROTEIN\_TARGETING\_TO\_MEMBRANE |  | 20 | -0.47 | -1.31 | 0.122 | 0.438 | 1.000 | 795 | tags=20%, list=7%, signal=21% |
| 1816 | GOCC\_PCAF\_COMPLEX |  | 5 | -0.68 | -1.31 | 0.116 | 0.438 | 1.000 | 2292 | tags=40%, list=19%, signal=49% |
| 1817 | GOBP\_ASSOCIATIVE\_LEARNING |  | 44 | -0.41 | -1.31 | 0.098 | 0.438 | 1.000 | 2631 | tags=34%, list=22%, signal=43% |
| 1818 | GOBP\_CRANIAL\_SUTURE\_MORPHOGENESIS |  | 5 | -0.68 | -1.31 | 0.124 | 0.438 | 1.000 | 2457 | tags=60%, list=20%, signal=75% |
| 1819 | GOBP\_MALE\_MEIOSIS\_I |  | 13 | -0.53 | -1.31 | 0.126 | 0.439 | 1.000 | 3903 | tags=54%, list=32%, signal=80% |
| 1820 | GOBP\_REGULATION\_OF\_MITOPHAGY |  | 7 | -0.62 | -1.31 | 0.131 | 0.440 | 1.000 | 2263 | tags=43%, list=19%, signal=53% |
| 1821 | GOBP\_RESPONSE\_TO\_WOUNDING |  | 431 | -0.33 | -1.31 | 0.002 | 0.439 | 1.000 | 3394 | tags=35%, list=28%, signal=47% |
| 1822 | GOCC\_SYMMETRIC\_SYNAPSE |  | 8 | -0.60 | -1.31 | 0.128 | 0.439 | 1.000 | 2294 | tags=63%, list=19%, signal=77% |
| 1823 | GOBP\_POSITIVE\_REGULATION\_OF\_TRANSCRIPTION\_BY\_RNA\_POLYMERASE\_III |  | 9 | -0.58 | -1.31 | 0.133 | 0.439 | 1.000 | 1743 | tags=44%, list=15%, signal=52% |
| 1824 | GOBP\_REGULATION\_OF\_MUSCLE\_SYSTEM\_PROCESS |  | 148 | -0.35 | -1.31 | 0.035 | 0.440 | 1.000 | 3968 | tags=40%, list=33%, signal=59% |
| 1825 | GOBP\_POSITIVE\_REGULATION\_OF\_MYELOID\_CELL\_DIFFERENTIATION |  | 67 | -0.38 | -1.31 | 0.082 | 0.440 | 1.000 | 1605 | tags=30%, list=13%, signal=34% |
| 1826 | GOBP\_CARDIAC\_NEURAL\_CREST\_CELL\_DIFFERENTIATION\_INVOLVED\_IN\_HEART\_DEVELOPMENT |  | 9 | -0.58 | -1.31 | 0.137 | 0.440 | 1.000 | 1692 | tags=33%, list=14%, signal=39% |
| 1827 | GOBP\_ESTABLISHMENT\_OR\_MAINTENANCE\_OF\_MONOPOLAR\_CELL\_POLARITY |  | 17 | -0.49 | -1.31 | 0.129 | 0.440 | 1.000 | 3212 | tags=41%, list=27%, signal=56% |
| 1828 | GOBP\_SENSORY\_PERCEPTION\_OF\_PAIN |  | 53 | -0.39 | -1.31 | 0.086 | 0.440 | 1.000 | 1771 | tags=26%, list=15%, signal=31% |
| 1829 | GOBP\_REGULATION\_OF\_ORGANELLE\_ASSEMBLY |  | 140 | -0.35 | -1.31 | 0.035 | 0.440 | 1.000 | 5206 | tags=51%, list=43%, signal=90% |
| 1830 | GOBP\_DIVALENT\_INORGANIC\_CATION\_HOMEOSTASIS |  | 298 | -0.34 | -1.31 | 0.008 | 0.440 | 1.000 | 3178 | tags=33%, list=26%, signal=44% |
| 1831 | GOBP\_POSTSYNAPTIC\_SIGNAL\_TRANSDUCTION |  | 24 | -0.46 | -1.31 | 0.126 | 0.440 | 1.000 | 1675 | tags=25%, list=14%, signal=29% |
| 1832 | GOBP\_REGULATION\_OF\_ANTIGEN\_RECEPTOR\_MEDIATED\_SIGNALING\_PATHWAY |  | 46 | -0.40 | -1.31 | 0.098 | 0.440 | 1.000 | 2857 | tags=39%, list=24%, signal=51% |
| 1833 | GOBP\_CHEMOKINE\_PRODUCTION |  | 54 | -0.39 | -1.31 | 0.090 | 0.440 | 1.000 | 3093 | tags=43%, list=26%, signal=57% |
| 1834 | GOBP\_RESPONSE\_TO\_GLYCOPROTEIN |  | 6 | -0.64 | -1.31 | 0.129 | 0.440 | 1.000 | 2715 | tags=67%, list=23%, signal=86% |
| 1835 | GOCC\_RETROMER\_COMPLEX |  | 17 | -0.49 | -1.31 | 0.127 | 0.440 | 1.000 | 2252 | tags=29%, list=19%, signal=36% |
| 1836 | GOBP\_ACTIVATION\_OF\_ADENYLATE\_CYCLASE\_ACTIVITY |  | 23 | -0.46 | -1.31 | 0.127 | 0.440 | 1.000 | 3909 | tags=48%, list=33%, signal=71% |
| 1837 | GOBP\_CYTOSOLIC\_CALCIUM\_ION\_TRANSPORT |  | 112 | -0.36 | -1.31 | 0.050 | 0.441 | 1.000 | 3909 | tags=38%, list=33%, signal=56% |
| 1838 | GOBP\_CEREBELLAR\_GRANULAR\_LAYER\_FORMATION |  | 5 | -0.68 | -1.31 | 0.120 | 0.441 | 1.000 | 149 | tags=20%, list=1%, signal=20% |
| 1839 | GOBP\_REGULATION\_OF\_CELLULAR\_COMPONENT\_SIZE |  | 260 | -0.34 | -1.31 | 0.011 | 0.441 | 1.000 | 3235 | tags=33%, list=27%, signal=44% |
| 1840 | GOBP\_NK\_T\_CELL\_DIFFERENTIATION |  | 5 | -0.67 | -1.31 | 0.124 | 0.442 | 1.000 | 3112 | tags=80%, list=26%, signal=108% |
| 1841 | GOMF\_DISORDERED\_DOMAIN\_SPECIFIC\_BINDING |  | 22 | -0.46 | -1.31 | 0.128 | 0.441 | 1.000 | 5082 | tags=68%, list=42%, signal=118% |
| 1842 | GOBP\_MEIOTIC\_CHROMOSOME\_CONDENSATION |  | 6 | -0.65 | -1.31 | 0.129 | 0.441 | 1.000 | 3228 | tags=83%, list=27%, signal=114% |
| 1843 | GOBP\_IMPORT\_INTO\_NUCLEUS |  | 120 | -0.36 | -1.31 | 0.048 | 0.441 | 1.000 | 4032 | tags=40%, list=34%, signal=60% |
| 1844 | GOMF\_MITOGEN\_ACTIVATED\_PROTEIN\_KINASE\_BINDING |  | 27 | -0.44 | -1.31 | 0.120 | 0.442 | 1.000 | 3647 | tags=44%, list=30%, signal=64% |
| 1845 | GOBP\_CELLULAR\_RESPONSE\_TO\_ALCOHOL |  | 63 | -0.38 | -1.31 | 0.084 | 0.442 | 1.000 | 3613 | tags=49%, list=30%, signal=70% |
| 1846 | GOBP\_RESPONSE\_TO\_INSECTICIDE |  | 4 | -0.72 | -1.31 | 0.116 | 0.442 | 1.000 | 3331 | tags=100%, list=28%, signal=138% |
| 1847 | GOBP\_REGULATION\_OF\_ENDOCYTOSIS |  | 146 | -0.35 | -1.31 | 0.043 | 0.442 | 1.000 | 3059 | tags=32%, list=25%, signal=42% |
| 1848 | GOMF\_MAGNESIUM\_ION\_BINDING |  | 167 | -0.35 | -1.31 | 0.034 | 0.442 | 1.000 | 4286 | tags=46%, list=36%, signal=71% |
| 1849 | GOBP\_CELLULAR\_RESPONSE\_TO\_CADMIUM\_ION |  | 17 | -0.49 | -1.31 | 0.138 | 0.443 | 1.000 | 2458 | tags=35%, list=20%, signal=44% |
| 1850 | GOBP\_PROTEIN\_ACETYLATION |  | 152 | -0.35 | -1.31 | 0.035 | 0.442 | 1.000 | 3626 | tags=38%, list=30%, signal=53% |
| 1851 | GOBP\_FORMATION\_OF\_EXTRACHROMOSOMAL\_CIRCULAR\_DNA |  | 12 | -0.53 | -1.31 | 0.142 | 0.442 | 1.000 | 3927 | tags=67%, list=33%, signal=99% |
| 1852 | GOBP\_RHO\_PROTEIN\_SIGNAL\_TRANSDUCTION |  | 98 | -0.36 | -1.31 | 0.060 | 0.443 | 1.000 | 3286 | tags=36%, list=27%, signal=49% |
| 1853 | GOBP\_VENTRICULAR\_CARDIAC\_MUSCLE\_CELL\_DIFFERENTIATION |  | 9 | -0.57 | -1.31 | 0.136 | 0.443 | 1.000 | 3331 | tags=56%, list=28%, signal=77% |
| 1854 | GOBP\_CEREBELLAR\_PURKINJE\_CELL\_LAYER\_FORMATION |  | 6 | -0.64 | -1.31 | 0.132 | 0.443 | 1.000 | 4002 | tags=67%, list=33%, signal=100% |
| 1855 | GOBP\_CD4\_POSITIVE\_ALPHA\_BETA\_T\_CELL\_DIFFERENTIATION |  | 52 | -0.40 | -1.31 | 0.091 | 0.443 | 1.000 | 2345 | tags=38%, list=20%, signal=48% |
| 1856 | GOBP\_INTERMEDIATE\_FILAMENT\_BASED\_PROCESS |  | 29 | -0.44 | -1.31 | 0.118 | 0.443 | 1.000 | 3765 | tags=38%, list=31%, signal=55% |
| 1857 | GOBP\_NEGATIVE\_REGULATION\_OF\_PEPTIDYL\_TYROSINE\_PHOSPHORYLATION |  | 38 | -0.41 | -1.31 | 0.104 | 0.443 | 1.000 | 2267 | tags=29%, list=19%, signal=36% |
| 1858 | GOBP\_VACUOLAR\_PROTON\_TRANSPORTING\_V\_TYPE\_ATPASE\_COMPLEX\_ASSEMBLY |  | 4 | -0.72 | -1.31 | 0.120 | 0.444 | 1.000 | 2924 | tags=75%, list=24%, signal=99% |
| 1859 | GOBP\_REGULATION\_OF\_PINOCYTOSIS |  | 8 | -0.59 | -1.31 | 0.139 | 0.444 | 1.000 | 1903 | tags=38%, list=16%, signal=45% |
| 1860 | GOBP\_POSITIVE\_REGULATION\_OF\_TELOMERE\_CAPPING |  | 14 | -0.51 | -1.31 | 0.142 | 0.444 | 1.000 | 1704 | tags=43%, list=14%, signal=50% |
| 1861 | GOBP\_CEREBELLAR\_PURKINJE\_CELL\_DIFFERENTIATION |  | 6 | -0.64 | -1.31 | 0.137 | 0.445 | 1.000 | 4002 | tags=67%, list=33%, signal=100% |
| 1862 | GOMF\_R\_SMAD\_BINDING |  | 16 | -0.49 | -1.31 | 0.142 | 0.444 | 1.000 | 3376 | tags=50%, list=28%, signal=69% |
| 1863 | GOBP\_REGULATION\_OF\_FC\_GAMMA\_RECEPTOR\_SIGNALING\_PATHWAY\_INVOLVED\_IN\_PHAGOCYTOSIS |  | 7 | -0.61 | -1.31 | 0.125 | 0.444 | 1.000 | 1519 | tags=43%, list=13%, signal=49% |
| 1864 | GOMF\_CILIARY\_NEUROTROPHIC\_FACTOR\_RECEPTOR\_BINDING |  | 8 | -0.59 | -1.31 | 0.143 | 0.445 | 1.000 | 1472 | tags=25%, list=12%, signal=28% |
| 1865 | GOCC\_CLATHRIN\_VESICLE\_COAT |  | 19 | -0.48 | -1.31 | 0.135 | 0.445 | 1.000 | 4775 | tags=68%, list=40%, signal=113% |
| 1866 | GOBP\_MUSCLE\_CELL\_CELLULAR\_HOMEOSTASIS |  | 14 | -0.51 | -1.31 | 0.143 | 0.446 | 1.000 | 2503 | tags=29%, list=21%, signal=36% |
| 1867 | GOBP\_ANTERIOR\_POSTERIOR\_AXON\_GUIDANCE |  | 3 | -0.78 | -1.31 | 0.101 | 0.446 | 1.000 | 223 | tags=33%, list=2%, signal=34% |
| 1868 | GOMF\_ACTIN\_MONOMER\_BINDING |  | 14 | -0.51 | -1.30 | 0.132 | 0.447 | 1.000 | 1402 | tags=29%, list=12%, signal=32% |
| 1869 | GOBP\_POSITIVE\_REGULATION\_OF\_DNA\_TEMPLATED\_TRANSCRIPTION\_INITIATION |  | 29 | -0.44 | -1.30 | 0.119 | 0.447 | 1.000 | 4990 | tags=59%, list=42%, signal=100% |
| 1870 | GOBP\_DEDIFFERENTIATION |  | 5 | -0.68 | -1.30 | 0.127 | 0.448 | 1.000 | 125 | tags=20%, list=1%, signal=20% |
| 1871 | GOCC\_SWR1\_COMPLEX |  | 9 | -0.57 | -1.30 | 0.145 | 0.448 | 1.000 | 3857 | tags=56%, list=32%, signal=82% |
| 1872 | GOBP\_POSITIVE\_REGULATION\_OF\_CHEMOKINE\_PRODUCTION |  | 37 | -0.42 | -1.30 | 0.110 | 0.448 | 1.000 | 3326 | tags=49%, list=28%, signal=67% |
| 1873 | GOBP\_DNA\_CATABOLIC\_PROCESS |  | 24 | -0.45 | -1.30 | 0.134 | 0.448 | 1.000 | 5564 | tags=71%, list=46%, signal=132% |
| 1874 | GOBP\_NEGATIVE\_REGULATION\_OF\_NEUROTRANSMITTER\_TRANSPORT |  | 11 | -0.54 | -1.30 | 0.147 | 0.448 | 1.000 | 2087 | tags=27%, list=17%, signal=33% |
| 1875 | GOCC\_TRANSFERASE\_COMPLEX |  | 600 | -0.33 | -1.30 | 0.001 | 0.448 | 1.000 | 4525 | tags=44%, list=38%, signal=66% |
| 1876 | GOBP\_PROTEIN\_KINASE\_C\_SIGNALING |  | 16 | -0.49 | -1.30 | 0.140 | 0.448 | 1.000 | 3609 | tags=44%, list=30%, signal=62% |
| 1877 | GOBP\_REGULATION\_OF\_RESPONSE\_TO\_OSMOTIC\_STRESS |  | 6 | -0.64 | -1.30 | 0.132 | 0.448 | 1.000 | 1410 | tags=33%, list=12%, signal=38% |
| 1878 | GOBP\_CELL\_CELL\_ADHESION\_VIA\_PLASMA\_MEMBRANE\_ADHESION\_MOLECULES |  | 135 | -0.35 | -1.30 | 0.042 | 0.449 | 1.000 | 3589 | tags=37%, list=30%, signal=52% |
| 1879 | GOBP\_REGULATION\_OF\_NEUTROPHIL\_DEGRANULATION |  | 4 | -0.72 | -1.30 | 0.130 | 0.449 | 1.000 | 3050 | tags=75%, list=25%, signal=100% |
| 1880 | GOBP\_PHYSIOLOGICAL\_CARDIAC\_MUSCLE\_HYPERTROPHY |  | 17 | -0.49 | -1.30 | 0.136 | 0.449 | 1.000 | 3177 | tags=47%, list=26%, signal=64% |
| 1881 | GOBP\_REGULATION\_OF\_STEROL\_TRANSPORT |  | 35 | -0.42 | -1.30 | 0.117 | 0.450 | 1.000 | 3789 | tags=46%, list=32%, signal=67% |
| 1882 | GOBP\_REGULATION\_OF\_SYNAPTIC\_PLASTICITY |  | 114 | -0.36 | -1.30 | 0.055 | 0.450 | 1.000 | 3833 | tags=42%, list=32%, signal=61% |
| 1883 | GOBP\_POSITIVE\_REGULATION\_OF\_HOMOTYPIC\_CELL\_CELL\_ADHESION |  | 6 | -0.63 | -1.30 | 0.136 | 0.450 | 1.000 | 1038 | tags=50%, list=9%, signal=55% |
| 1884 | GOCC\_WNT\_SIGNALOSOME |  | 9 | -0.57 | -1.30 | 0.142 | 0.450 | 1.000 | 3483 | tags=56%, list=29%, signal=78% |
| 1885 | GOMF\_METHYLATED\_HISTONE\_BINDING |  | 46 | -0.40 | -1.30 | 0.108 | 0.450 | 1.000 | 3624 | tags=41%, list=30%, signal=59% |
| 1886 | GOMF\_LONG\_CHAIN\_FATTY\_ACID\_COA\_LIGASE\_ACTIVITY |  | 11 | -0.54 | -1.30 | 0.138 | 0.450 | 1.000 | 4053 | tags=64%, list=34%, signal=96% |
| 1887 | GOBP\_REGULATION\_OF\_CYTOSKELETON\_ORGANIZATION |  | 370 | -0.33 | -1.30 | 0.005 | 0.450 | 1.000 | 3093 | tags=33%, list=26%, signal=43% |
| 1888 | GOBP\_MEMBRANE\_DEPOLARIZATION\_DURING\_SA\_NODE\_CELL\_ACTION\_POTENTIAL |  | 4 | -0.72 | -1.30 | 0.116 | 0.451 | 1.000 | 3295 | tags=75%, list=27%, signal=103% |
| 1889 | GOBP\_REGULATION\_OF\_VENTRICULAR\_CARDIAC\_MUSCLE\_CELL\_ACTION\_POTENTIAL |  | 10 | -0.55 | -1.30 | 0.148 | 0.451 | 1.000 | 3918 | tags=60%, list=33%, signal=89% |
| 1890 | GOBP\_INTERLEUKIN\_2\_PRODUCTION |  | 44 | -0.40 | -1.30 | 0.103 | 0.451 | 1.000 | 2345 | tags=34%, list=20%, signal=42% |
| 1891 | GOBP\_NEUTRAL\_LIPID\_METABOLIC\_PROCESS |  | 91 | -0.36 | -1.30 | 0.069 | 0.451 | 1.000 | 2684 | tags=34%, list=22%, signal=44% |
| 1892 | GOBP\_PROTEIN\_DEPHOSPHORYLATION |  | 237 | -0.34 | -1.30 | 0.018 | 0.451 | 1.000 | 4631 | tags=48%, list=39%, signal=77% |
| 1893 | GOCC\_GATOR2\_COMPLEX |  | 9 | -0.57 | -1.30 | 0.144 | 0.451 | 1.000 | 3512 | tags=44%, list=29%, signal=63% |
| 1894 | GOBP\_NEGATIVE\_REGULATION\_OF\_IMMUNE\_SYSTEM\_PROCESS |  | 232 | -0.34 | -1.30 | 0.018 | 0.450 | 1.000 | 3180 | tags=35%, list=26%, signal=47% |
| 1895 | GOBP\_POSITIVE\_REGULATION\_OF\_PINOCYTOSIS |  | 6 | -0.64 | -1.30 | 0.130 | 0.450 | 1.000 | 1903 | tags=50%, list=16%, signal=59% |
| 1896 | GOCC\_SEMAPHORIN\_RECEPTOR\_COMPLEX |  | 8 | -0.59 | -1.30 | 0.144 | 0.451 | 1.000 | 3895 | tags=75%, list=32%, signal=111% |
| 1897 | GOBP\_ZYMOGEN\_ACTIVATION |  | 27 | -0.44 | -1.30 | 0.129 | 0.450 | 1.000 | 2205 | tags=37%, list=18%, signal=45% |
| 1898 | GOBP\_MICROTUBULE\_CYTOSKELETON\_ORGANIZATION\_INVOLVED\_IN\_MITOSIS |  | 121 | -0.35 | -1.30 | 0.053 | 0.450 | 1.000 | 5928 | tags=64%, list=49%, signal=124% |
| 1899 | GOCC\_INCLUSION\_BODY |  | 57 | -0.39 | -1.30 | 0.093 | 0.450 | 1.000 | 3766 | tags=46%, list=31%, signal=66% |
| 1900 | GOMF\_PROTEIN\_HORMONE\_RECEPTOR\_ACTIVITY |  | 10 | -0.56 | -1.30 | 0.139 | 0.450 | 1.000 | 2158 | tags=40%, list=18%, signal=49% |
| 1901 | GOCC\_TRANSCRIPTION\_ELONGATION\_FACTOR\_COMPLEX |  | 44 | -0.40 | -1.30 | 0.104 | 0.450 | 1.000 | 4704 | tags=50%, list=39%, signal=82% |
| 1902 | GOBP\_POSITIVE\_REGULATION\_OF\_GLOMERULAR\_MESANGIAL\_CELL\_PROLIFERATION |  | 5 | -0.68 | -1.30 | 0.127 | 0.450 | 1.000 | 2494 | tags=40%, list=21%, signal=50% |
| 1903 | GOCC\_MITOCHONDRIAL\_CRISTA |  | 4 | -0.72 | -1.30 | 0.118 | 0.450 | 1.000 | 698 | tags=50%, list=6%, signal=53% |
| 1904 | GOCC\_LIPOPOLYSACCHARIDE\_RECEPTOR\_COMPLEX |  | 5 | -0.68 | -1.30 | 0.122 | 0.450 | 1.000 | 2470 | tags=40%, list=21%, signal=50% |
| 1905 | GOBP\_CONNECTIVE\_TISSUE\_DEVELOPMENT |  | 163 | -0.35 | -1.30 | 0.040 | 0.450 | 1.000 | 3510 | tags=36%, list=29%, signal=50% |
| 1906 | GOMF\_ACTIN\_FILAMENT\_BINDING |  | 151 | -0.35 | -1.30 | 0.043 | 0.450 | 1.000 | 2948 | tags=31%, list=25%, signal=41% |
| 1907 | GOBP\_TRACHEA\_MORPHOGENESIS |  | 7 | -0.61 | -1.30 | 0.133 | 0.450 | 1.000 | 1692 | tags=43%, list=14%, signal=50% |
| 1908 | GOBP\_MUSCLE\_CELL\_DEVELOPMENT |  | 98 | -0.36 | -1.30 | 0.064 | 0.450 | 1.000 | 3331 | tags=34%, list=28%, signal=46% |
| 1909 | GOBP\_RESPONSE\_TO\_BMP |  | 112 | -0.36 | -1.30 | 0.059 | 0.451 | 1.000 | 3377 | tags=42%, list=28%, signal=58% |
| 1910 | GOMF\_PRIMARY\_AMINE\_OXIDASE\_ACTIVITY |  | 5 | -0.67 | -1.30 | 0.127 | 0.450 | 1.000 | 1782 | tags=60%, list=15%, signal=70% |
| 1911 | GOBP\_HEART\_DEVELOPMENT |  | 380 | -0.33 | -1.30 | 0.006 | 0.451 | 1.000 | 3348 | tags=32%, list=28%, signal=43% |
| 1912 | GOBP\_POSITIVE\_REGULATION\_OF\_TELOMERE\_MAINTENANCE\_VIA\_TELOMERE\_LENGTHENING |  | 32 | -0.42 | -1.30 | 0.119 | 0.451 | 1.000 | 1704 | tags=28%, list=14%, signal=33% |
| 1913 | GOMF\_MANNOSYLTRANSFERASE\_ACTIVITY |  | 21 | -0.46 | -1.30 | 0.134 | 0.452 | 1.000 | 1497 | tags=29%, list=12%, signal=33% |
| 1914 | GOBP\_CELL\_DIFFERENTIATION\_INVOLVED\_IN\_EMBRYONIC\_PLACENTA\_DEVELOPMENT |  | 14 | -0.51 | -1.30 | 0.141 | 0.452 | 1.000 | 1783 | tags=43%, list=15%, signal=50% |
| 1915 | GOMF\_CYCLIC\_NUCLEOTIDE\_DEPENDENT\_PROTEIN\_KINASE\_ACTIVITY |  | 7 | -0.61 | -1.30 | 0.143 | 0.452 | 1.000 | 3920 | tags=71%, list=33%, signal=106% |
| 1916 | GOMF\_ACTIN\_BINDING |  | 297 | -0.34 | -1.30 | 0.011 | 0.452 | 1.000 | 3139 | tags=30%, list=26%, signal=40% |
| 1917 | GOBP\_MICROTUBULE\_ANCHORING\_AT\_MICROTUBULE\_ORGANIZING\_CENTER |  | 10 | -0.56 | -1.30 | 0.143 | 0.452 | 1.000 | 2479 | tags=50%, list=21%, signal=63% |
| 1918 | GOBP\_TUBE\_DEVELOPMENT |  | 700 | -0.33 | -1.30 | 0.001 | 0.452 | 1.000 | 3968 | tags=40%, list=33%, signal=56% |
| 1919 | GOBP\_MEMBRANE\_INVAGINATION |  | 54 | -0.39 | -1.30 | 0.096 | 0.452 | 1.000 | 1843 | tags=24%, list=15%, signal=28% |
| 1920 | GOBP\_REGULATION\_OF\_LONG\_TERM\_NEURONAL\_SYNAPTIC\_PLASTICITY |  | 15 | -0.50 | -1.30 | 0.144 | 0.453 | 1.000 | 3476 | tags=47%, list=29%, signal=66% |
| 1921 | GOBP\_DEPHOSPHORYLATION |  | 359 | -0.33 | -1.30 | 0.006 | 0.452 | 1.000 | 3988 | tags=41%, list=33%, signal=59% |
| 1922 | GOCC\_NUCLEAR\_MEMBRANE |  | 225 | -0.34 | -1.30 | 0.021 | 0.452 | 1.000 | 4120 | tags=44%, list=34%, signal=66% |
| 1923 | GOBP\_POSITIVE\_REGULATION\_OF\_CELLULAR\_COMPONENT\_ORGANIZATION |  | 797 | -0.33 | -1.30 | 0.000 | 0.453 | 1.000 | 3723 | tags=36%, list=31%, signal=49% |
| 1924 | GOBP\_POSITIVE\_REGULATION\_OF\_CELL\_GROWTH\_INVOLVED\_IN\_CARDIAC\_MUSCLE\_CELL\_DEVELOPMENT |  | 5 | -0.67 | -1.30 | 0.127 | 0.453 | 1.000 | 2866 | tags=60%, list=24%, signal=79% |
| 1925 | GOCC\_NUCLEAR\_CHROMOSOME |  | 160 | -0.35 | -1.30 | 0.040 | 0.453 | 1.000 | 3857 | tags=42%, list=32%, signal=61% |
| 1926 | GOBP\_REGULATION\_OF\_ACTIN\_FILAMENT\_BASED\_PROCESS |  | 281 | -0.33 | -1.30 | 0.015 | 0.453 | 1.000 | 3313 | tags=33%, list=28%, signal=45% |
| 1927 | GOMF\_CD4\_RECEPTOR\_BINDING |  | 6 | -0.64 | -1.30 | 0.144 | 0.453 | 1.000 | 3062 | tags=67%, list=25%, signal=89% |
| 1928 | GOMF\_PEPTIDE\_RECEPTOR\_ACTIVITY |  | 73 | -0.37 | -1.30 | 0.085 | 0.453 | 1.000 | 4541 | tags=56%, list=38%, signal=90% |
| 1929 | GOBP\_NEGATIVE\_REGULATION\_OF\_METAPHASE\_ANAPHASE\_TRANSITION\_OF\_CELL\_CYCLE |  | 36 | -0.42 | -1.30 | 0.120 | 0.453 | 1.000 | 3976 | tags=42%, list=33%, signal=62% |
| 1930 | GOBP\_BRANCHING\_INVOLVED\_IN\_BLOOD\_VESSEL\_MORPHOGENESIS |  | 23 | -0.45 | -1.30 | 0.135 | 0.453 | 1.000 | 3895 | tags=52%, list=32%, signal=77% |
| 1931 | GOBP\_ENDODERM\_FORMATION |  | 35 | -0.42 | -1.30 | 0.117 | 0.453 | 1.000 | 2630 | tags=34%, list=22%, signal=44% |
| 1932 | GOBP\_REGULATION\_OF\_CHROMOSOME\_SEPARATION |  | 61 | -0.38 | -1.30 | 0.091 | 0.453 | 1.000 | 4419 | tags=43%, list=37%, signal=67% |
| 1933 | GOBP\_REGULATION\_OF\_METANEPHROS\_DEVELOPMENT |  | 3 | -0.77 | -1.30 | 0.106 | 0.453 | 1.000 | 2356 | tags=67%, list=20%, signal=83% |
| 1934 | GOCC\_NEURON\_SPINE |  | 120 | -0.35 | -1.30 | 0.053 | 0.453 | 1.000 | 4052 | tags=43%, list=34%, signal=63% |
| 1935 | GOBP\_POSITIVE\_REGULATION\_OF\_PROTEIN\_LOCALIZATION\_TO\_CENTROSOME |  | 4 | -0.72 | -1.30 | 0.126 | 0.453 | 1.000 | 2479 | tags=75%, list=21%, signal=94% |
| 1936 | GOCC\_ACTIN\_FILAMENT |  | 87 | -0.37 | -1.30 | 0.080 | 0.453 | 1.000 | 2757 | tags=29%, list=23%, signal=37% |
| 1937 | GOCC\_RECYCLING\_ENDOSOME |  | 134 | -0.35 | -1.30 | 0.050 | 0.453 | 1.000 | 3728 | tags=38%, list=31%, signal=55% |
| 1938 | GOBP\_CARDIAC\_MUSCLE\_CELL\_CARDIAC\_MUSCLE\_CELL\_ADHESION |  | 5 | -0.67 | -1.30 | 0.139 | 0.453 | 1.000 | 3918 | tags=80%, list=33%, signal=119% |
| 1939 | GOMF\_FOUR\_WAY\_JUNCTION\_DNA\_BINDING |  | 15 | -0.50 | -1.30 | 0.146 | 0.454 | 1.000 | 5752 | tags=80%, list=48%, signal=153% |
| 1940 | GOBP\_TELOMERE\_CAPPING |  | 33 | -0.42 | -1.30 | 0.119 | 0.454 | 1.000 | 2918 | tags=42%, list=24%, signal=56% |
| 1941 | GOBP\_FOLIC\_ACID\_CONTAINING\_COMPOUND\_BIOSYNTHETIC\_PROCESS |  | 8 | -0.58 | -1.30 | 0.150 | 0.454 | 1.000 | 4242 | tags=63%, list=35%, signal=97% |
| 1942 | GOBP\_REGULATION\_OF\_TRANSCRIPTION\_BY\_RNA\_POLYMERASE\_III |  | 17 | -0.49 | -1.30 | 0.135 | 0.454 | 1.000 | 4704 | tags=65%, list=39%, signal=106% |
| 1943 | GOBP\_G2\_DNA\_DAMAGE\_CHECKPOINT |  | 29 | -0.43 | -1.30 | 0.126 | 0.454 | 1.000 | 3852 | tags=45%, list=32%, signal=66% |
| 1944 | GOBP\_ENDOCYTOSIS\_INVOLVED\_IN\_VIRAL\_ENTRY\_INTO\_HOST\_CELL |  | 5 | -0.67 | -1.30 | 0.139 | 0.454 | 1.000 | 1200 | tags=40%, list=10%, signal=44% |
| 1945 | GOBP\_REGULATION\_OF\_DENDRITE\_MORPHOGENESIS |  | 47 | -0.40 | -1.30 | 0.106 | 0.454 | 1.000 | 5358 | tags=60%, list=45%, signal=107% |
| 1946 | GOMF\_ZINC\_ION\_BINDING |  | 496 | -0.33 | -1.30 | 0.002 | 0.454 | 1.000 | 3718 | tags=38%, list=31%, signal=53% |
| 1947 | GOBP\_GASTRULATION |  | 122 | -0.35 | -1.30 | 0.057 | 0.454 | 1.000 | 3306 | tags=33%, list=28%, signal=45% |
| 1948 | GOBP\_DETECTION\_OF\_STIMULUS\_INVOLVED\_IN\_SENSORY\_PERCEPTION |  | 36 | -0.41 | -1.30 | 0.117 | 0.454 | 1.000 | 2209 | tags=31%, list=18%, signal=37% |
| 1949 | GOBP\_SYNAPTIC\_TRANSMISSION\_CHOLINERGIC |  | 11 | -0.54 | -1.30 | 0.145 | 0.454 | 1.000 | 322 | tags=36%, list=3%, signal=37% |
| 1950 | GOCC\_CLATHRIN\_COATED\_PIT |  | 55 | -0.39 | -1.30 | 0.091 | 0.454 | 1.000 | 4869 | tags=60%, list=41%, signal=100% |
| 1951 | GOBP\_CIRCULATORY\_SYSTEM\_DEVELOPMENT |  | 734 | -0.33 | -1.30 | 0.000 | 0.454 | 1.000 | 3408 | tags=34%, list=28%, signal=45% |
| 1952 | GOBP\_PYRAMIDAL\_NEURON\_DIFFERENTIATION |  | 6 | -0.64 | -1.30 | 0.139 | 0.454 | 1.000 | 2369 | tags=67%, list=20%, signal=83% |
| 1953 | GOBP\_PERIPHERAL\_NERVOUS\_SYSTEM\_DEVELOPMENT |  | 55 | -0.39 | -1.30 | 0.105 | 0.454 | 1.000 | 3613 | tags=36%, list=30%, signal=52% |
| 1954 | GOBP\_PROTEIN\_O\_LINKED\_GLYCOSYLATION\_VIA\_THREONINE |  | 7 | -0.61 | -1.30 | 0.139 | 0.454 | 1.000 | 4702 | tags=100%, list=39%, signal=164% |
| 1955 | GOBP\_SODIUM\_ION\_TRANSMEMBRANE\_TRANSPORT |  | 100 | -0.36 | -1.30 | 0.066 | 0.454 | 1.000 | 3089 | tags=40%, list=26%, signal=53% |
| 1956 | GOBP\_POSITIVE\_REGULATION\_OF\_RECEPTOR\_MEDIATED\_ENDOCYTOSIS |  | 34 | -0.42 | -1.30 | 0.124 | 0.454 | 1.000 | 2629 | tags=35%, list=22%, signal=45% |
| 1957 | GOBP\_POSITIVE\_REGULATION\_OF\_GROWTH\_HORMONE\_SECRETION |  | 3 | -0.78 | -1.30 | 0.119 | 0.454 | 1.000 | 2351 | tags=67%, list=20%, signal=83% |
| 1958 | GOBP\_INTERLEUKIN\_27\_MEDIATED\_SIGNALING\_PATHWAY |  | 8 | -0.59 | -1.30 | 0.143 | 0.454 | 1.000 | 960 | tags=50%, list=8%, signal=54% |
| 1959 | GOMF\_MHC\_PROTEIN\_COMPLEX\_BINDING |  | 11 | -0.54 | -1.30 | 0.150 | 0.454 | 1.000 | 2239 | tags=36%, list=19%, signal=45% |
| 1960 | GOBP\_DNA\_DOUBLE\_STRAND\_BREAK\_PROCESSING |  | 21 | -0.46 | -1.30 | 0.142 | 0.454 | 1.000 | 5251 | tags=71%, list=44%, signal=127% |
| 1961 | GOBP\_TISSUE\_REMODELING |  | 109 | -0.36 | -1.29 | 0.067 | 0.455 | 1.000 | 2474 | tags=31%, list=21%, signal=39% |
| 1962 | GOMF\_VASCULAR\_ENDOTHELIAL\_GROWTH\_FACTOR\_ACTIVATED\_RECEPTOR\_ACTIVITY |  | 7 | -0.60 | -1.29 | 0.146 | 0.455 | 1.000 | 2791 | tags=57%, list=23%, signal=74% |
| 1963 | GOMF\_NUCLEOCYTOPLASMIC\_CARRIER\_ACTIVITY |  | 27 | -0.44 | -1.29 | 0.123 | 0.455 | 1.000 | 4866 | tags=59%, list=41%, signal=99% |
| 1964 | GOBP\_POSITIVE\_REGULATION\_OF\_PROTEIN\_CONTAINING\_COMPLEX\_ASSEMBLY |  | 171 | -0.34 | -1.29 | 0.035 | 0.455 | 1.000 | 4060 | tags=41%, list=34%, signal=61% |
| 1965 | GOBP\_NEGATIVE\_REGULATION\_OF\_PLASMINOGEN\_ACTIVATION |  | 4 | -0.71 | -1.29 | 0.124 | 0.455 | 1.000 | 2820 | tags=75%, list=23%, signal=98% |
| 1966 | GOBP\_PROTEIN\_LOCALIZATION\_TO\_ADHERENS\_JUNCTION |  | 5 | -0.67 | -1.29 | 0.141 | 0.455 | 1.000 | 3825 | tags=80%, list=32%, signal=117% |
| 1967 | GOCC\_TORC1\_COMPLEX |  | 7 | -0.61 | -1.29 | 0.138 | 0.455 | 1.000 | 2850 | tags=57%, list=24%, signal=75% |
| 1968 | GOBP\_SENSORY\_PERCEPTION\_OF\_SMELL |  | 10 | -0.55 | -1.29 | 0.142 | 0.455 | 1.000 | 933 | tags=30%, list=8%, signal=32% |
| 1969 | GOBP\_T\_FOLLICULAR\_HELPER\_CELL\_DIFFERENTIATION |  | 4 | -0.71 | -1.29 | 0.128 | 0.455 | 1.000 | 1951 | tags=75%, list=16%, signal=90% |
| 1970 | GOBP\_PLATELET\_DERIVED\_GROWTH\_FACTOR\_RECEPTOR\_SIGNALING\_PATHWAY |  | 52 | -0.39 | -1.29 | 0.107 | 0.454 | 1.000 | 4433 | tags=48%, list=37%, signal=76% |
| 1971 | GOBP\_VASCULATURE\_DEVELOPMENT |  | 503 | -0.33 | -1.29 | 0.002 | 0.454 | 1.000 | 3615 | tags=37%, list=30%, signal=51% |
| 1972 | GOBP\_PROTEIN\_LOCALIZATION\_TO\_CHROMATIN |  | 21 | -0.46 | -1.29 | 0.140 | 0.454 | 1.000 | 4660 | tags=67%, list=39%, signal=109% |
| 1973 | GOBP\_PIGMENT\_ACCUMULATION |  | 10 | -0.55 | -1.29 | 0.148 | 0.454 | 1.000 | 3569 | tags=70%, list=30%, signal=100% |
| 1974 | GOBP\_POSITIVE\_REGULATION\_OF\_EXTRACELLULAR\_MATRIX\_DISASSEMBLY |  | 6 | -0.64 | -1.29 | 0.141 | 0.454 | 1.000 | 3551 | tags=83%, list=30%, signal=118% |
| 1975 | GOBP\_NEGATIVE\_REGULATION\_OF\_LYMPHOCYTE\_MEDIATED\_IMMUNITY |  | 16 | -0.49 | -1.29 | 0.140 | 0.454 | 1.000 | 2249 | tags=44%, list=19%, signal=54% |
| 1976 | GOBP\_REGULATION\_OF\_ANATOMICAL\_STRUCTURE\_MORPHOGENESIS |  | 665 | -0.33 | -1.29 | 0.001 | 0.454 | 1.000 | 3948 | tags=38%, list=33%, signal=53% |
| 1977 | GOCC\_EXTRINSIC\_COMPONENT\_OF\_MEMBRANE |  | 219 | -0.34 | -1.29 | 0.028 | 0.454 | 1.000 | 2466 | tags=30%, list=21%, signal=37% |
| 1978 | GOBP\_REGULATION\_OF\_RESPONSE\_TO\_TUMOR\_CELL |  | 6 | -0.64 | -1.29 | 0.139 | 0.454 | 1.000 | 3240 | tags=67%, list=27%, signal=91% |
| 1979 | GOBP\_CARDIAC\_CHAMBER\_DEVELOPMENT |  | 107 | -0.36 | -1.29 | 0.063 | 0.454 | 1.000 | 2560 | tags=26%, list=21%, signal=33% |
| 1980 | GOMF\_HYDROLASE\_ACTIVITY\_ACTING\_ON\_CARBON\_NITROGEN\_BUT\_NOT\_PEPTIDE\_BONDS\_IN\_CYCLIC\_AMIDINES |  | 20 | -0.47 | -1.29 | 0.145 | 0.454 | 1.000 | 2903 | tags=40%, list=24%, signal=53% |
| 1981 | GOBP\_NEGATIVE\_REGULATION\_OF\_AXON\_EXTENSION |  | 29 | -0.43 | -1.29 | 0.129 | 0.454 | 1.000 | 2631 | tags=34%, list=22%, signal=44% |
| 1982 | GOBP\_MUSCLE\_HYPERTROPHY |  | 62 | -0.38 | -1.29 | 0.095 | 0.455 | 1.000 | 3376 | tags=37%, list=28%, signal=51% |
| 1983 | GOBP\_MITOTIC\_DNA\_INTEGRITY\_CHECKPOINT |  | 81 | -0.37 | -1.29 | 0.083 | 0.455 | 1.000 | 3888 | tags=41%, list=32%, signal=60% |
| 1984 | GOMF\_SOLUTE\_PROTON\_ANTIPORTER\_ACTIVITY |  | 15 | -0.50 | -1.29 | 0.141 | 0.455 | 1.000 | 1128 | tags=40%, list=9%, signal=44% |
| 1985 | GOBP\_REGULATION\_OF\_CYTOKINESIS |  | 67 | -0.38 | -1.29 | 0.092 | 0.455 | 1.000 | 3027 | tags=34%, list=25%, signal=46% |
| 1986 | GOBP\_POSITIVE\_REGULATION\_OF\_AUTOPHAGY |  | 89 | -0.36 | -1.29 | 0.072 | 0.455 | 1.000 | 4324 | tags=44%, list=36%, signal=68% |
| 1987 | GOBP\_POSITIVE\_REGULATION\_OF\_VIRAL\_GENOME\_REPLICATION |  | 28 | -0.43 | -1.29 | 0.129 | 0.455 | 1.000 | 3520 | tags=43%, list=29%, signal=60% |
| 1988 | GOBP\_CARDIAC\_CHAMBER\_MORPHOGENESIS |  | 77 | -0.37 | -1.29 | 0.082 | 0.455 | 1.000 | 2560 | tags=26%, list=21%, signal=33% |
| 1989 | GOBP\_NEGATIVE\_REGULATION\_OF\_PHOSPHORUS\_METABOLIC\_PROCESS |  | 352 | -0.33 | -1.29 | 0.010 | 0.455 | 1.000 | 4008 | tags=39%, list=33%, signal=58% |
| 1990 | GOMF\_PROTEIN\_DOMAIN\_SPECIFIC\_BINDING |  | 483 | -0.33 | -1.29 | 0.002 | 0.455 | 1.000 | 3970 | tags=39%, list=33%, signal=56% |
| 1991 | GOCC\_HAUS\_COMPLEX |  | 4 | -0.72 | -1.29 | 0.119 | 0.455 | 1.000 | 1216 | tags=50%, list=10%, signal=56% |
| 1992 | GOBP\_MEMBRANE\_HYPERPOLARIZATION |  | 10 | -0.55 | -1.29 | 0.147 | 0.455 | 1.000 | 2038 | tags=30%, list=17%, signal=36% |
| 1993 | GOBP\_POSITIVE\_REGULATION\_OF\_SKELETAL\_MUSCLE\_TISSUE\_GROWTH |  | 4 | -0.72 | -1.29 | 0.126 | 0.455 | 1.000 | 2503 | tags=50%, list=21%, signal=63% |
| 1994 | GOMF\_EXOGENOUS\_PROTEIN\_BINDING |  | 47 | -0.40 | -1.29 | 0.108 | 0.455 | 1.000 | 2515 | tags=34%, list=21%, signal=43% |
| 1995 | GOBP\_CHROMOSOME\_CONDENSATION |  | 23 | -0.45 | -1.29 | 0.140 | 0.455 | 1.000 | 3331 | tags=52%, list=28%, signal=72% |
| 1996 | GOCC\_CONDENSED\_CHROMOSOME |  | 160 | -0.35 | -1.29 | 0.035 | 0.455 | 1.000 | 5518 | tags=56%, list=46%, signal=102% |
| 1997 | GOMF\_STEROID\_HORMONE\_RECEPTOR\_BINDING |  | 62 | -0.38 | -1.29 | 0.092 | 0.455 | 1.000 | 4358 | tags=50%, list=36%, signal=78% |
| 1998 | GOBP\_VESICLE\_TRANSPORT\_ALONG\_MICROTUBULE |  | 37 | -0.41 | -1.29 | 0.128 | 0.455 | 1.000 | 3967 | tags=49%, list=33%, signal=72% |
| 1999 | GOBP\_ACTOMYOSIN\_STRUCTURE\_ORGANIZATION |  | 137 | -0.35 | -1.29 | 0.046 | 0.455 | 1.000 | 3578 | tags=37%, list=30%, signal=52% |
| 2000 | GOMF\_DEATH\_RECEPTOR\_BINDING |  | 15 | -0.50 | -1.29 | 0.147 | 0.455 | 1.000 | 2494 | tags=40%, list=21%, signal=50% |
| 2001 | GOBP\_EMBRYONIC\_ORGAN\_MORPHOGENESIS |  | 162 | -0.35 | -1.29 | 0.040 | 0.455 | 1.000 | 3565 | tags=35%, list=30%, signal=48% |
| 2002 | GOCC\_SMOOTH\_ENDOPLASMIC\_RETICULUM |  | 21 | -0.46 | -1.29 | 0.141 | 0.455 | 1.000 | 3261 | tags=38%, list=27%, signal=52% |
| 2003 | GOBP\_TELOMERIC\_LOOP\_DISASSEMBLY |  | 8 | -0.58 | -1.29 | 0.153 | 0.455 | 1.000 | 3722 | tags=75%, list=31%, signal=109% |
| 2004 | GOBP\_NEURAL\_NUCLEUS\_DEVELOPMENT |  | 39 | -0.41 | -1.29 | 0.112 | 0.455 | 1.000 | 1871 | tags=28%, list=16%, signal=33% |
| 2005 | GOBP\_CELLULAR\_COMPONENT\_MAINTENANCE |  | 42 | -0.40 | -1.29 | 0.117 | 0.456 | 1.000 | 3093 | tags=45%, list=26%, signal=61% |
| 2006 | GOBP\_REGULATION\_OF\_TRANSLATION\_INITIATION\_IN\_RESPONSE\_TO\_ENDOPLASMIC\_RETICULUM\_STRESS |  | 6 | -0.64 | -1.29 | 0.150 | 0.456 | 1.000 | 3872 | tags=67%, list=32%, signal=98% |
| 2007 | GOCC\_CAMP\_DEPENDENT\_PROTEIN\_KINASE\_COMPLEX |  | 6 | -0.63 | -1.29 | 0.137 | 0.456 | 1.000 | 4008 | tags=83%, list=33%, signal=125% |
| 2008 | GOBP\_HISTONE\_H2A\_K119\_MONOUBIQUITINATION |  | 8 | -0.59 | -1.29 | 0.147 | 0.456 | 1.000 | 4500 | tags=75%, list=37%, signal=120% |
| 2009 | GOBP\_RECEPTOR\_GUANYLYL\_CYCLASE\_SIGNALING\_PATHWAY |  | 3 | -0.77 | -1.29 | 0.124 | 0.456 | 1.000 | 2421 | tags=67%, list=20%, signal=83% |
| 2010 | GOBP\_REGULATION\_OF\_DNA\_DAMAGE\_CHECKPOINT |  | 12 | -0.52 | -1.29 | 0.160 | 0.456 | 1.000 | 3958 | tags=67%, list=33%, signal=99% |
| 2011 | GOBP\_TELOMERE\_MAINTENANCE\_VIA\_RECOMBINATION |  | 12 | -0.53 | -1.29 | 0.154 | 0.457 | 1.000 | 3668 | tags=50%, list=31%, signal=72% |
| 2012 | GOBP\_NEGATIVE\_REGULATION\_OF\_CYTOKINE\_PRODUCTION\_INVOLVED\_IN\_IMMUNE\_RESPONSE |  | 12 | -0.53 | -1.29 | 0.153 | 0.457 | 1.000 | 2889 | tags=50%, list=24%, signal=66% |
| 2013 | GOBP\_MECHANORECEPTOR\_DIFFERENTIATION |  | 38 | -0.41 | -1.29 | 0.120 | 0.456 | 1.000 | 3868 | tags=45%, list=32%, signal=66% |
| 2014 | GOBP\_GANGLIOSIDE\_BIOSYNTHETIC\_PROCESS |  | 11 | -0.54 | -1.29 | 0.152 | 0.457 | 1.000 | 2541 | tags=45%, list=21%, signal=58% |
| 2015 | GOMF\_MODIFICATION\_DEPENDENT\_PROTEIN\_BINDING |  | 105 | -0.36 | -1.29 | 0.068 | 0.457 | 1.000 | 3667 | tags=41%, list=31%, signal=58% |
| 2016 | GOBP\_REGULATION\_OF\_IMMUNE\_RESPONSE |  | 502 | -0.33 | -1.29 | 0.002 | 0.457 | 1.000 | 3240 | tags=34%, list=27%, signal=44% |
| 2017 | GOBP\_EPITHELIAL\_TUBE\_MORPHOGENESIS |  | 215 | -0.34 | -1.29 | 0.029 | 0.457 | 1.000 | 4341 | tags=43%, list=36%, signal=66% |
| 2018 | GOBP\_CELLULAR\_RESPONSE\_TO\_IRON\_ION |  | 5 | -0.67 | -1.29 | 0.140 | 0.457 | 1.000 | 3331 | tags=80%, list=28%, signal=111% |
| 2019 | GOBP\_CELL\_COMMUNICATION\_BY\_ELECTRICAL\_COUPLING |  | 20 | -0.46 | -1.29 | 0.141 | 0.458 | 1.000 | 4627 | tags=50%, list=39%, signal=81% |
| 2020 | GOMF\_PDZ\_DOMAIN\_BINDING |  | 57 | -0.38 | -1.29 | 0.105 | 0.458 | 1.000 | 4319 | tags=47%, list=36%, signal=74% |
| 2021 | GOBP\_INTRINSIC\_APOPTOTIC\_SIGNALING\_PATHWAY\_IN\_RESPONSE\_TO\_HYDROGEN\_PEROXIDE |  | 4 | -0.71 | -1.29 | 0.137 | 0.458 | 1.000 | 1151 | tags=25%, list=10%, signal=28% |
| 2022 | GOBP\_CENTRAL\_NERVOUS\_SYSTEM\_NEURON\_DEVELOPMENT |  | 61 | -0.38 | -1.29 | 0.104 | 0.458 | 1.000 | 3425 | tags=38%, list=29%, signal=52% |
| 2023 | GOBP\_GANGLIOSIDE\_METABOLIC\_PROCESS |  | 18 | -0.48 | -1.29 | 0.148 | 0.458 | 1.000 | 2541 | tags=44%, list=21%, signal=56% |
| 2024 | GOCC\_SPANNING\_COMPONENT\_OF\_MEMBRANE |  | 10 | -0.55 | -1.29 | 0.150 | 0.458 | 1.000 | 2897 | tags=50%, list=24%, signal=66% |
| 2025 | GOBP\_NEUROMUSCULAR\_PROCESS\_CONTROLLING\_POSTURE |  | 10 | -0.55 | -1.29 | 0.157 | 0.458 | 1.000 | 4548 | tags=70%, list=38%, signal=113% |
| 2026 | GOBP\_POSITIVE\_REGULATION\_OF\_MEMBRANE\_DEPOLARIZATION |  | 9 | -0.57 | -1.29 | 0.155 | 0.458 | 1.000 | 4110 | tags=56%, list=34%, signal=84% |
| 2027 | GOBP\_REGULATION\_OF\_STRESS\_ACTIVATED\_PROTEIN\_KINASE\_SIGNALING\_CASCADE |  | 125 | -0.35 | -1.29 | 0.058 | 0.457 | 1.000 | 2634 | tags=33%, list=22%, signal=42% |
| 2028 | GOBP\_CYTOKINE\_PRODUCTION\_INVOLVED\_IN\_INFLAMMATORY\_RESPONSE |  | 27 | -0.44 | -1.29 | 0.137 | 0.458 | 1.000 | 1748 | tags=30%, list=15%, signal=35% |
| 2029 | GOMF\_LEUCINE\_BINDING |  | 6 | -0.64 | -1.29 | 0.147 | 0.457 | 1.000 | 3375 | tags=50%, list=28%, signal=70% |
| 2030 | GOMF\_VOLTAGE\_GATED\_CATION\_CHANNEL\_ACTIVITY |  | 66 | -0.38 | -1.29 | 0.095 | 0.457 | 1.000 | 2192 | tags=27%, list=18%, signal=33% |
| 2031 | GOMF\_PROTEIN\_PHOSPHATASE\_1\_BINDING |  | 16 | -0.49 | -1.29 | 0.155 | 0.458 | 1.000 | 3473 | tags=50%, list=29%, signal=70% |
| 2032 | GOBP\_ESTABLISHMENT\_OR\_MAINTENANCE\_OF\_BIPOLAR\_CELL\_POLARITY |  | 33 | -0.42 | -1.29 | 0.133 | 0.457 | 1.000 | 3005 | tags=36%, list=25%, signal=48% |
| 2033 | GOBP\_NEGATIVE\_REGULATION\_OF\_CELL\_POPULATION\_PROLIFERATION |  | 437 | -0.33 | -1.29 | 0.006 | 0.457 | 1.000 | 3408 | tags=33%, list=28%, signal=45% |
| 2034 | GOBP\_MIRNA\_LOADING\_ONTO\_RISC\_INVOLVED\_IN\_GENE\_SILENCING\_BY\_MIRNA |  | 5 | -0.67 | -1.29 | 0.136 | 0.457 | 1.000 | 1503 | tags=60%, list=13%, signal=69% |
| 2035 | GOMF\_PHOSPHATIDYLINOSITOL\_PHOSPHATE\_5\_PHOSPHATASE\_ACTIVITY |  | 12 | -0.52 | -1.29 | 0.154 | 0.457 | 1.000 | 1202 | tags=33%, list=10%, signal=37% |
| 2036 | GOMF\_3\_5\_CYCLIC\_GMP\_PHOSPHODIESTERASE\_ACTIVITY |  | 8 | -0.58 | -1.29 | 0.147 | 0.457 | 1.000 | 2524 | tags=50%, list=21%, signal=63% |
| 2037 | GOBP\_REGULATION\_OF\_NOTCH\_SIGNALING\_PATHWAY |  | 75 | -0.37 | -1.29 | 0.087 | 0.457 | 1.000 | 4768 | tags=51%, list=40%, signal=83% |
| 2038 | GOBP\_PROTEIN\_ACYLATION |  | 182 | -0.34 | -1.29 | 0.036 | 0.457 | 1.000 | 3626 | tags=36%, list=30%, signal=50% |
| 2039 | GOBP\_AMYLOID\_BETA\_CLEARANCE\_BY\_CELLULAR\_CATABOLIC\_PROCESS |  | 6 | -0.64 | -1.29 | 0.148 | 0.458 | 1.000 | 3007 | tags=67%, list=25%, signal=89% |
| 2040 | GOBP\_DOUBLE\_STRAND\_BREAK\_REPAIR\_VIA\_CLASSICAL\_NONHOMOLOGOUS\_END\_JOINING |  | 6 | -0.63 | -1.29 | 0.148 | 0.458 | 1.000 | 3815 | tags=83%, list=32%, signal=122% |
| 2041 | GOMF\_DAMAGED\_DNA\_BINDING |  | 52 | -0.39 | -1.29 | 0.109 | 0.457 | 1.000 | 4660 | tags=52%, list=39%, signal=84% |
| 2042 | GOBP\_ACTIN\_FILAMENT\_BASED\_PROCESS |  | 552 | -0.33 | -1.29 | 0.003 | 0.457 | 1.000 | 3336 | tags=32%, list=28%, signal=42% |
| 2043 | GOBP\_NUCLEAR\_TRANSCRIBED\_MRNA\_POLY\_A\_TAIL\_SHORTENING |  | 24 | -0.45 | -1.29 | 0.141 | 0.457 | 1.000 | 2441 | tags=38%, list=20%, signal=47% |
| 2044 | GOMF\_RNA\_STEM\_LOOP\_BINDING |  | 12 | -0.52 | -1.29 | 0.163 | 0.457 | 1.000 | 5307 | tags=83%, list=44%, signal=149% |
| 2045 | GOCC\_CAVEOLA |  | 57 | -0.38 | -1.29 | 0.106 | 0.458 | 1.000 | 3538 | tags=37%, list=29%, signal=52% |
| 2046 | GOBP\_ENDOPLASMIC\_RETICULUM\_MEMBRANE\_ORGANIZATION |  | 4 | -0.71 | -1.29 | 0.130 | 0.458 | 1.000 | 191 | tags=25%, list=2%, signal=25% |
| 2047 | GOBP\_RESPONSE\_TO\_HEAT |  | 122 | -0.35 | -1.29 | 0.065 | 0.458 | 1.000 | 4407 | tags=46%, list=37%, signal=72% |
| 2048 | GOBP\_HEPARAN\_SULFATE\_PROTEOGLYCAN\_BIOSYNTHETIC\_PROCESS\_POLYSACCHARIDE\_CHAIN\_BIOSYNTHETIC\_PROCESS |  | 7 | -0.60 | -1.29 | 0.157 | 0.458 | 1.000 | 1117 | tags=43%, list=9%, signal=47% |
| 2049 | GOBP\_INTERLEUKIN\_1\_BETA\_PRODUCTION |  | 42 | -0.40 | -1.29 | 0.126 | 0.459 | 1.000 | 2480 | tags=36%, list=21%, signal=45% |
| 2050 | GOBP\_CHONDROCYTE\_DEVELOPMENT |  | 17 | -0.48 | -1.29 | 0.152 | 0.459 | 1.000 | 1441 | tags=29%, list=12%, signal=33% |
| 2051 | GOBP\_REGULATION\_OF\_HISTONE\_H3\_K4\_METHYLATION |  | 21 | -0.46 | -1.29 | 0.147 | 0.458 | 1.000 | 4698 | tags=76%, list=39%, signal=125% |
| 2052 | GOBP\_REGULATION\_OF\_DOUBLE\_STRAND\_BREAK\_REPAIR |  | 60 | -0.38 | -1.29 | 0.103 | 0.458 | 1.000 | 3434 | tags=42%, list=29%, signal=58% |
| 2053 | GOBP\_REGULATION\_OF\_PROTEIN\_TYROSINE\_PHOSPHATASE\_ACTIVITY |  | 5 | -0.67 | -1.29 | 0.137 | 0.459 | 1.000 | 1519 | tags=40%, list=13%, signal=46% |
| 2054 | GOBP\_POSITIVE\_REGULATION\_OF\_CYSTEINE\_TYPE\_ENDOPEPTIDASE\_ACTIVITY |  | 100 | -0.36 | -1.29 | 0.069 | 0.458 | 1.000 | 2982 | tags=34%, list=25%, signal=45% |
| 2055 | GOBP\_NEGATIVE\_REGULATION\_OF\_PEPTIDYL\_THREONINE\_PHOSPHORYLATION |  | 14 | -0.50 | -1.29 | 0.153 | 0.459 | 1.000 | 3934 | tags=64%, list=33%, signal=95% |
| 2056 | GOBP\_ENZYME\_LINKED\_RECEPTOR\_PROTEIN\_SIGNALING\_PATHWAY |  | 761 | -0.32 | -1.29 | 0.001 | 0.459 | 1.000 | 3151 | tags=33%, list=26%, signal=42% |
| 2057 | GOBP\_NEGATIVE\_REGULATION\_OF\_CELL\_CYCLE\_G1\_S\_PHASE\_TRANSITION |  | 78 | -0.37 | -1.29 | 0.086 | 0.459 | 1.000 | 4393 | tags=47%, list=37%, signal=74% |
| 2058 | GOBP\_NEGATIVE\_REGULATION\_OF\_SIGNAL\_TRANSDUCTION\_BY\_P53\_CLASS\_MEDIATOR |  | 21 | -0.46 | -1.29 | 0.152 | 0.459 | 1.000 | 1725 | tags=29%, list=14%, signal=33% |
| 2059 | GOMF\_VITAMIN\_BINDING |  | 95 | -0.36 | -1.29 | 0.079 | 0.460 | 1.000 | 4068 | tags=45%, list=34%, signal=68% |
| 2060 | GOBP\_VENTRICULAR\_CARDIAC\_MUSCLE\_TISSUE\_DEVELOPMENT |  | 31 | -0.42 | -1.29 | 0.143 | 0.460 | 1.000 | 3918 | tags=42%, list=33%, signal=62% |
| 2061 | GOMF\_CARBOHYDRATE\_PHOSPHATASE\_ACTIVITY |  | 7 | -0.60 | -1.29 | 0.152 | 0.460 | 1.000 | 320 | tags=29%, list=3%, signal=29% |
| 2062 | GOBP\_EXTRACELLULAR\_MATRIX\_DISASSEMBLY |  | 48 | -0.39 | -1.28 | 0.115 | 0.460 | 1.000 | 3156 | tags=33%, list=26%, signal=45% |
| 2063 | GOBP\_NEGATIVE\_REGULATION\_OF\_OLIGODENDROCYTE\_DIFFERENTIATION |  | 6 | -0.63 | -1.28 | 0.160 | 0.460 | 1.000 | 501 | tags=33%, list=4%, signal=35% |
| 2064 | GOBP\_NEGATIVE\_REGULATION\_OF\_MITOPHAGY |  | 4 | -0.71 | -1.28 | 0.130 | 0.460 | 1.000 | 2263 | tags=50%, list=19%, signal=62% |
| 2065 | GOBP\_BLOOD\_VESSEL\_MORPHOGENESIS |  | 419 | -0.33 | -1.28 | 0.005 | 0.460 | 1.000 | 3408 | tags=37%, list=28%, signal=49% |
| 2066 | GOCC\_KINETOCHORE\_MICROTUBULE |  | 6 | -0.63 | -1.28 | 0.141 | 0.460 | 1.000 | 3812 | tags=83%, list=32%, signal=122% |
| 2067 | GOCC\_TRANSPORTER\_COMPLEX |  | 165 | -0.34 | -1.28 | 0.047 | 0.461 | 1.000 | 2201 | tags=27%, list=18%, signal=32% |
| 2068 | GOBP\_CELLULAR\_RESPONSE\_TO\_LIPOPROTEIN\_PARTICLE\_STIMULUS |  | 24 | -0.45 | -1.28 | 0.137 | 0.461 | 1.000 | 3613 | tags=54%, list=30%, signal=77% |
| 2069 | GOMF\_N\_ACYLTRANSFERASE\_ACTIVITY |  | 61 | -0.38 | -1.28 | 0.107 | 0.461 | 1.000 | 3458 | tags=39%, list=29%, signal=55% |
| 2070 | GOBP\_REGULATION\_OF\_CYSTEINE\_TYPE\_ENDOPEPTIDASE\_ACTIVITY\_INVOLVED\_IN\_APOPTOTIC\_SIGNALING\_PATHWAY |  | 13 | -0.51 | -1.28 | 0.158 | 0.460 | 1.000 | 3061 | tags=54%, list=25%, signal=72% |
| 2071 | GOBP\_TUBE\_MORPHOGENESIS |  | 576 | -0.32 | -1.28 | 0.001 | 0.460 | 1.000 | 3619 | tags=37%, list=30%, signal=50% |
| 2072 | GOBP\_RESPONSE\_TO\_CHEMOKINE |  | 45 | -0.39 | -1.28 | 0.115 | 0.460 | 1.000 | 2955 | tags=42%, list=25%, signal=56% |
| 2073 | GOBP\_MULTINUCLEAR\_OSTEOCLAST\_DIFFERENTIATION |  | 6 | -0.63 | -1.28 | 0.150 | 0.460 | 1.000 | 2423 | tags=50%, list=20%, signal=63% |
| 2074 | GOMF\_BETA\_TUBULIN\_BINDING |  | 31 | -0.42 | -1.28 | 0.137 | 0.460 | 1.000 | 3215 | tags=35%, list=27%, signal=48% |
| 2075 | GOBP\_REGULATION\_OF\_LYMPHOCYTE\_MIGRATION |  | 38 | -0.41 | -1.28 | 0.128 | 0.460 | 1.000 | 2955 | tags=39%, list=25%, signal=52% |
| 2076 | GOBP\_POSITIVE\_REGULATION\_OF\_CYTOKINE\_PRODUCTION |  | 257 | -0.33 | -1.28 | 0.021 | 0.460 | 1.000 | 3205 | tags=37%, list=27%, signal=50% |
| 2077 | GOMF\_PROTEIN\_CONTAINING\_COMPLEX\_BINDING |  | 854 | -0.32 | -1.28 | 0.001 | 0.460 | 1.000 | 4122 | tags=38%, list=34%, signal=53% |
| 2078 | GOCC\_FASCIA\_ADHERENS |  | 8 | -0.58 | -1.28 | 0.148 | 0.460 | 1.000 | 3851 | tags=63%, list=32%, signal=92% |
| 2079 | GOCC\_GLIAL\_CELL\_PROJECTION |  | 20 | -0.46 | -1.28 | 0.147 | 0.460 | 1.000 | 2215 | tags=35%, list=18%, signal=43% |
| 2080 | GOCC\_TRANSCRIPTION\_REGULATOR\_COMPLEX |  | 288 | -0.33 | -1.28 | 0.014 | 0.460 | 1.000 | 4743 | tags=47%, list=39%, signal=76% |
| 2081 | GOBP\_REGULATION\_OF\_T\_CELL\_DIFFERENTIATION\_IN\_THYMUS |  | 18 | -0.47 | -1.28 | 0.147 | 0.461 | 1.000 | 1079 | tags=33%, list=9%, signal=37% |
| 2082 | GOMF\_ION\_GATED\_CHANNEL\_ACTIVITY |  | 23 | -0.45 | -1.28 | 0.148 | 0.460 | 1.000 | 3515 | tags=43%, list=29%, signal=61% |
| 2083 | GOBP\_MAST\_CELL\_CYTOKINE\_PRODUCTION |  | 6 | -0.63 | -1.28 | 0.158 | 0.460 | 1.000 | 1935 | tags=67%, list=16%, signal=79% |
| 2084 | GOBP\_NEGATIVE\_REGULATION\_OF\_RNA\_CATABOLIC\_PROCESS |  | 44 | -0.40 | -1.28 | 0.124 | 0.460 | 1.000 | 5518 | tags=64%, list=46%, signal=117% |
| 2085 | GOBP\_DEATH\_INDUCING\_SIGNALING\_COMPLEX\_ASSEMBLY |  | 8 | -0.58 | -1.28 | 0.156 | 0.460 | 1.000 | 1781 | tags=38%, list=15%, signal=44% |
| 2086 | GOCC\_PML\_BODY |  | 81 | -0.36 | -1.28 | 0.087 | 0.460 | 1.000 | 3979 | tags=46%, list=33%, signal=68% |
| 2087 | GOCC\_VOLTAGE\_GATED\_SODIUM\_CHANNEL\_COMPLEX |  | 8 | -0.58 | -1.28 | 0.155 | 0.460 | 1.000 | 372 | tags=25%, list=3%, signal=26% |
| 2088 | GOBP\_NEGATIVE\_REGULATION\_OF\_VASOCONSTRICTION |  | 6 | -0.63 | -1.28 | 0.150 | 0.460 | 1.000 | 925 | tags=50%, list=8%, signal=54% |
| 2089 | GOMF\_ENDODEOXYRIBONUCLEASE\_ACTIVITY |  | 25 | -0.44 | -1.28 | 0.140 | 0.461 | 1.000 | 2944 | tags=40%, list=25%, signal=53% |
| 2090 | GOBP\_VESICLE\_MEDIATED\_TRANSPORT\_IN\_SYNAPSE |  | 133 | -0.35 | -1.28 | 0.057 | 0.461 | 1.000 | 4008 | tags=47%, list=33%, signal=69% |
| 2091 | GOBP\_EAR\_MORPHOGENESIS |  | 57 | -0.38 | -1.28 | 0.104 | 0.461 | 1.000 | 1692 | tags=25%, list=14%, signal=28% |
| 2092 | GOBP\_REGULATION\_OF\_CELLULAR\_RESPONSE\_TO\_GROWTH\_FACTOR\_STIMULUS |  | 193 | -0.34 | -1.28 | 0.036 | 0.461 | 1.000 | 3126 | tags=34%, list=26%, signal=45% |
| 2093 | GOCC\_PLASMA\_MEMBRANE\_REGION |  | 747 | -0.32 | -1.28 | 0.000 | 0.461 | 1.000 | 3069 | tags=33%, list=26%, signal=41% |
| 2094 | GOBP\_INORGANIC\_ANION\_TRANSMEMBRANE\_TRANSPORT |  | 65 | -0.37 | -1.28 | 0.101 | 0.461 | 1.000 | 2159 | tags=34%, list=18%, signal=41% |
| 2095 | GOBP\_CELLULAR\_RESPONSE\_TO\_CAMP |  | 36 | -0.41 | -1.28 | 0.136 | 0.462 | 1.000 | 2038 | tags=28%, list=17%, signal=33% |
| 2096 | GOBP\_CENTRIOLE\_ASSEMBLY |  | 34 | -0.41 | -1.28 | 0.133 | 0.462 | 1.000 | 3718 | tags=44%, list=31%, signal=64% |
| 2097 | GOBP\_REGULATION\_OF\_CELL\_CELL\_ADHESION\_MEDIATED\_BY\_INTEGRIN |  | 10 | -0.54 | -1.28 | 0.168 | 0.464 | 1.000 | 1944 | tags=60%, list=16%, signal=72% |
| 2098 | GOMF\_INORGANIC\_ANION\_TRANSMEMBRANE\_TRANSPORTER\_ACTIVITY |  | 81 | -0.36 | -1.28 | 0.091 | 0.464 | 1.000 | 2884 | tags=41%, list=24%, signal=53% |
| 2099 | GOMF\_COMPLEMENT\_RECEPTOR\_ACTIVITY |  | 3 | -0.77 | -1.28 | 0.128 | 0.464 | 1.000 | 1988 | tags=67%, list=17%, signal=80% |
| 2100 | GOBP\_REGULATION\_OF\_DNA\_METHYLATION |  | 14 | -0.50 | -1.28 | 0.159 | 0.464 | 1.000 | 3972 | tags=57%, list=33%, signal=85% |
| 2101 | GOMF\_SEMAPHORIN\_RECEPTOR\_ACTIVITY |  | 9 | -0.56 | -1.28 | 0.162 | 0.465 | 1.000 | 3895 | tags=67%, list=32%, signal=99% |
| 2102 | GOBP\_STRESS\_GRANULE\_DISASSEMBLY |  | 5 | -0.66 | -1.28 | 0.146 | 0.464 | 1.000 | 4104 | tags=80%, list=34%, signal=121% |
| 2103 | GOBP\_RESPONSE\_TO\_ATP |  | 22 | -0.45 | -1.28 | 0.148 | 0.464 | 1.000 | 4715 | tags=59%, list=39%, signal=97% |
| 2104 | GOBP\_MYOTUBE\_DIFFERENTIATION |  | 58 | -0.38 | -1.28 | 0.112 | 0.464 | 1.000 | 2747 | tags=34%, list=23%, signal=44% |
| 2105 | GOCC\_GOLGI\_ASSOCIATED\_VESICLE\_MEMBRANE |  | 37 | -0.41 | -1.28 | 0.130 | 0.465 | 1.000 | 5524 | tags=70%, list=46%, signal=130% |
| 2106 | GOCC\_SITE\_OF\_DOUBLE\_STRAND\_BREAK |  | 56 | -0.38 | -1.28 | 0.110 | 0.465 | 1.000 | 3632 | tags=39%, list=30%, signal=56% |
| 2107 | GOBP\_NEGATIVE\_REGULATION\_OF\_DIGESTIVE\_SYSTEM\_PROCESS |  | 8 | -0.58 | -1.28 | 0.162 | 0.465 | 1.000 | 2895 | tags=63%, list=24%, signal=82% |
| 2108 | GOBP\_LIPID\_LOCALIZATION |  | 309 | -0.33 | -1.28 | 0.015 | 0.465 | 1.000 | 3410 | tags=37%, list=28%, signal=51% |
| 2109 | GOBP\_CARDIAC\_MUSCLE\_TISSUE\_DEVELOPMENT |  | 126 | -0.35 | -1.28 | 0.062 | 0.465 | 1.000 | 3392 | tags=34%, list=28%, signal=47% |
| 2110 | GOBP\_EOSINOPHIL\_CHEMOTAXIS |  | 9 | -0.56 | -1.28 | 0.163 | 0.465 | 1.000 | 1038 | tags=33%, list=9%, signal=36% |
| 2111 | GOBP\_NEGATIVE\_REGULATION\_OF\_DNA\_DAMAGE\_RESPONSE\_SIGNAL\_TRANSDUCTION\_BY\_P53\_CLASS\_MEDIATOR |  | 13 | -0.51 | -1.28 | 0.167 | 0.465 | 1.000 | 2656 | tags=46%, list=22%, signal=59% |
| 2112 | GOCC\_SITE\_OF\_DNA\_DAMAGE |  | 72 | -0.37 | -1.28 | 0.096 | 0.465 | 1.000 | 3632 | tags=39%, list=30%, signal=55% |
| 2113 | GOCC\_EXOCYTIC\_VESICLE |  | 143 | -0.34 | -1.28 | 0.050 | 0.465 | 1.000 | 2055 | tags=25%, list=17%, signal=30% |
| 2114 | GOBP\_NEURON\_APOPTOTIC\_PROCESS |  | 144 | -0.35 | -1.28 | 0.060 | 0.465 | 1.000 | 3444 | tags=36%, list=29%, signal=50% |
| 2115 | GOBP\_MALE\_MEIOTIC\_NUCLEAR\_DIVISION |  | 25 | -0.44 | -1.28 | 0.151 | 0.466 | 1.000 | 2642 | tags=36%, list=22%, signal=46% |
| 2116 | GOBP\_REGULATION\_OF\_DNA\_REPAIR |  | 94 | -0.36 | -1.28 | 0.084 | 0.466 | 1.000 | 3452 | tags=38%, list=29%, signal=53% |
| 2117 | GOBP\_PROTEIN\_LOCALIZATION\_TO\_CELL\_PERIPHERY |  | 242 | -0.33 | -1.28 | 0.024 | 0.466 | 1.000 | 4398 | tags=45%, list=37%, signal=70% |
| 2118 | GOBP\_NEGATIVE\_REGULATION\_OF\_MAP\_KINASE\_ACTIVITY |  | 53 | -0.38 | -1.28 | 0.125 | 0.466 | 1.000 | 2382 | tags=32%, list=20%, signal=40% |
| 2119 | GOBP\_ARTERY\_DEVELOPMENT |  | 66 | -0.37 | -1.28 | 0.104 | 0.466 | 1.000 | 5330 | tags=59%, list=44%, signal=106% |
| 2120 | GOBP\_NIK\_NF\_KAPPAB\_SIGNALING |  | 123 | -0.35 | -1.28 | 0.066 | 0.467 | 1.000 | 2731 | tags=27%, list=23%, signal=34% |
| 2121 | GOBP\_REGULATION\_OF\_MITOTIC\_SISTER\_CHROMATID\_SEGREGATION |  | 39 | -0.40 | -1.28 | 0.134 | 0.467 | 1.000 | 6407 | tags=69%, list=53%, signal=148% |
| 2122 | GOBP\_REGULATION\_OF\_PEPTIDE\_HORMONE\_SECRETION |  | 136 | -0.34 | -1.28 | 0.057 | 0.466 | 1.000 | 2351 | tags=29%, list=20%, signal=36% |
| 2123 | GOBP\_CELLULAR\_RESPONSE\_TO\_ATP |  | 10 | -0.54 | -1.28 | 0.163 | 0.466 | 1.000 | 5191 | tags=70%, list=43%, signal=123% |
| 2124 | GOBP\_CELL\_DIVISION |  | 461 | -0.33 | -1.28 | 0.005 | 0.467 | 1.000 | 4457 | tags=42%, list=37%, signal=65% |
| 2125 | GOBP\_INFLAMMATORY\_RESPONSE |  | 417 | -0.33 | -1.28 | 0.008 | 0.467 | 1.000 | 3794 | tags=40%, list=32%, signal=57% |
| 2126 | GOMF\_DNA\_BINDING\_TRANSCRIPTION\_FACTOR\_BINDING |  | 294 | -0.33 | -1.28 | 0.020 | 0.467 | 1.000 | 4649 | tags=44%, list=39%, signal=70% |
| 2127 | GOBP\_POSITIVE\_REGULATION\_OF\_CATABOLIC\_PROCESS |  | 335 | -0.33 | -1.28 | 0.013 | 0.467 | 1.000 | 3988 | tags=38%, list=33%, signal=56% |
| 2128 | GOCC\_POSTSYNAPTIC\_SPECIALIZATION\_MEMBRANE |  | 46 | -0.39 | -1.28 | 0.125 | 0.467 | 1.000 | 3629 | tags=41%, list=30%, signal=59% |
| 2129 | GOBP\_IMMUNOGLOBULIN\_V\_D\_J\_RECOMBINATION |  | 5 | -0.66 | -1.28 | 0.145 | 0.468 | 1.000 | 3632 | tags=80%, list=30%, signal=115% |
| 2130 | GOBP\_NEUROTRANSMITTER\_BIOSYNTHETIC\_PROCESS |  | 8 | -0.58 | -1.28 | 0.154 | 0.467 | 1.000 | 2543 | tags=63%, list=21%, signal=79% |
| 2131 | GOMF\_SYNTAXIN\_1\_BINDING |  | 22 | -0.45 | -1.28 | 0.151 | 0.467 | 1.000 | 2055 | tags=45%, list=17%, signal=55% |
| 2132 | GOBP\_SENSORY\_PERCEPTION\_OF\_CHEMICAL\_STIMULUS |  | 30 | -0.42 | -1.28 | 0.138 | 0.467 | 1.000 | 1240 | tags=33%, list=10%, signal=37% |
| 2133 | GOBP\_GLYCOPROTEIN\_CATABOLIC\_PROCESS |  | 17 | -0.48 | -1.28 | 0.158 | 0.468 | 1.000 | 2802 | tags=53%, list=23%, signal=69% |
| 2134 | GOMF\_O\_ACYLTRANSFERASE\_ACTIVITY |  | 37 | -0.41 | -1.28 | 0.135 | 0.468 | 1.000 | 1685 | tags=22%, list=14%, signal=25% |
| 2135 | GOBP\_REGULATION\_OF\_METAL\_ION\_TRANSPORT |  | 149 | -0.34 | -1.28 | 0.057 | 0.468 | 1.000 | 2908 | tags=29%, list=24%, signal=38% |
| 2136 | GOBP\_REGULATION\_OF\_CHROMOSOME\_SEGREGATION |  | 74 | -0.37 | -1.28 | 0.099 | 0.470 | 1.000 | 4419 | tags=43%, list=37%, signal=68% |
| 2137 | GOMF\_STRUCTURAL\_CONSTITUENT\_OF\_POSTSYNAPSE |  | 4 | -0.71 | -1.28 | 0.145 | 0.470 | 1.000 | 2604 | tags=50%, list=22%, signal=64% |
| 2138 | GOBP\_GROWTH |  | 615 | -0.32 | -1.28 | 0.001 | 0.471 | 1.000 | 3408 | tags=33%, list=28%, signal=44% |
| 2139 | GOBP\_NEGATIVE\_REGULATION\_OF\_TRANSFERASE\_ACTIVITY |  | 214 | -0.33 | -1.28 | 0.032 | 0.470 | 1.000 | 2292 | tags=26%, list=19%, signal=32% |
| 2140 | GOBP\_REGULATION\_OF\_CYSTEINE\_TYPE\_ENDOPEPTIDASE\_ACTIVITY |  | 151 | -0.34 | -1.28 | 0.055 | 0.470 | 1.000 | 3345 | tags=34%, list=28%, signal=47% |
| 2141 | GOMF\_FATTY\_ACID\_LIGASE\_ACTIVITY |  | 13 | -0.51 | -1.28 | 0.167 | 0.471 | 1.000 | 4053 | tags=54%, list=34%, signal=81% |
| 2142 | GOBP\_DETECTION\_OF\_CALCIUM\_ION |  | 10 | -0.54 | -1.28 | 0.163 | 0.471 | 1.000 | 1269 | tags=30%, list=11%, signal=34% |
| 2143 | GOBP\_SEQUESTERING\_OF\_ZINC\_ION |  | 3 | -0.76 | -1.28 | 0.140 | 0.471 | 1.000 | 2899 | tags=100%, list=24%, signal=132% |
| 2144 | GOBP\_POSITIVE\_REGULATION\_OF\_CHOLESTEROL\_EFFLUX |  | 15 | -0.49 | -1.27 | 0.162 | 0.471 | 1.000 | 3007 | tags=40%, list=25%, signal=53% |
| 2145 | GOBP\_RESPONSE\_TO\_PAIN |  | 20 | -0.46 | -1.27 | 0.151 | 0.471 | 1.000 | 4402 | tags=50%, list=37%, signal=79% |
| 2146 | GOMF\_STEROL\_TRANSPORTER\_ACTIVITY |  | 29 | -0.43 | -1.27 | 0.150 | 0.471 | 1.000 | 3600 | tags=45%, list=30%, signal=64% |
| 2147 | GOBP\_POSITIVE\_REGULATION\_OF\_PROTEIN\_SUMOYLATION |  | 11 | -0.53 | -1.27 | 0.176 | 0.471 | 1.000 | 435 | tags=18%, list=4%, signal=19% |
| 2148 | GOBP\_POSITIVE\_REGULATION\_OF\_COLD\_INDUCED\_THERMOGENESIS |  | 65 | -0.37 | -1.27 | 0.108 | 0.471 | 1.000 | 2411 | tags=32%, list=20%, signal=40% |
| 2149 | GOBP\_MYELOID\_DENDRITIC\_CELL\_ACTIVATION |  | 16 | -0.49 | -1.27 | 0.161 | 0.472 | 1.000 | 6186 | tags=100%, list=52%, signal=206% |
| 2150 | GOMF\_HISTONE\_DEACETYLASE\_BINDING |  | 84 | -0.36 | -1.27 | 0.100 | 0.472 | 1.000 | 3595 | tags=35%, list=30%, signal=49% |
| 2151 | GOBP\_PEPTIDE\_HORMONE\_SECRETION |  | 165 | -0.34 | -1.27 | 0.051 | 0.472 | 1.000 | 2351 | tags=28%, list=20%, signal=35% |
| 2152 | GOMF\_LIGAND\_GATED\_CALCIUM\_CHANNEL\_ACTIVITY |  | 18 | -0.47 | -1.27 | 0.157 | 0.472 | 1.000 | 3398 | tags=44%, list=28%, signal=62% |
| 2153 | GOBP\_REGULATION\_OF\_CELL\_JUNCTION\_ASSEMBLY |  | 137 | -0.34 | -1.27 | 0.064 | 0.472 | 1.000 | 3790 | tags=41%, list=32%, signal=59% |
| 2154 | GOBP\_POSITIVE\_REGULATION\_OF\_DENDRITIC\_SPINE\_MORPHOGENESIS |  | 15 | -0.49 | -1.27 | 0.167 | 0.472 | 1.000 | 4673 | tags=60%, list=39%, signal=98% |
| 2155 | GOBP\_PIGMENT\_GRANULE\_MATURATION |  | 8 | -0.58 | -1.27 | 0.166 | 0.472 | 1.000 | 3569 | tags=75%, list=30%, signal=107% |
| 2156 | GOMF\_CALCIUM\_DEPENDENT\_PHOSPHOLIPID\_BINDING |  | 35 | -0.41 | -1.27 | 0.143 | 0.472 | 1.000 | 1072 | tags=20%, list=9%, signal=22% |
| 2157 | GOBP\_SYNAPTIC\_VESICLE\_RECYCLING |  | 48 | -0.39 | -1.27 | 0.124 | 0.472 | 1.000 | 3909 | tags=50%, list=33%, signal=74% |
| 2158 | GOBP\_SOMATIC\_DIVERSIFICATION\_OF\_IMMUNE\_RECEPTORS |  | 56 | -0.38 | -1.27 | 0.118 | 0.472 | 1.000 | 2639 | tags=34%, list=22%, signal=43% |
| 2159 | GOBP\_RECEPTOR\_TRANSACTIVATION |  | 5 | -0.66 | -1.27 | 0.155 | 0.472 | 1.000 | 125 | tags=20%, list=1%, signal=20% |
| 2160 | GOBP\_REGULATION\_OF\_HISTONE\_H3\_K9\_TRIMETHYLATION |  | 3 | -0.76 | -1.27 | 0.136 | 0.472 | 1.000 | 2656 | tags=67%, list=22%, signal=86% |
| 2161 | GOBP\_DENDRITIC\_CELL\_ANTIGEN\_PROCESSING\_AND\_PRESENTATION |  | 7 | -0.60 | -1.27 | 0.161 | 0.472 | 1.000 | 2847 | tags=43%, list=24%, signal=56% |
| 2162 | GOBP\_CALCIUM\_ION\_IMPORT |  | 42 | -0.40 | -1.27 | 0.131 | 0.472 | 1.000 | 3682 | tags=38%, list=31%, signal=55% |
| 2163 | GOBP\_PATTERN\_RECOGNITION\_RECEPTOR\_SIGNALING\_PATHWAY |  | 135 | -0.34 | -1.27 | 0.062 | 0.472 | 1.000 | 3343 | tags=39%, list=28%, signal=54% |
| 2164 | GOBP\_MEMORY |  | 72 | -0.37 | -1.27 | 0.096 | 0.473 | 1.000 | 2466 | tags=29%, list=21%, signal=36% |
| 2165 | GOBP\_POSITIVE\_REGULATION\_OF\_ACTIN\_NUCLEATION |  | 12 | -0.52 | -1.27 | 0.168 | 0.472 | 1.000 | 3797 | tags=58%, list=32%, signal=85% |
| 2166 | GOBP\_INTERLEUKIN\_7\_MEDIATED\_SIGNALING\_PATHWAY |  | 13 | -0.51 | -1.27 | 0.168 | 0.473 | 1.000 | 3326 | tags=54%, list=28%, signal=74% |
| 2167 | GOCC\_APICAL\_DENDRITE |  | 15 | -0.49 | -1.27 | 0.172 | 0.472 | 1.000 | 1308 | tags=33%, list=11%, signal=37% |
| 2168 | GOBP\_TROPHOBLAST\_CELL\_MIGRATION |  | 10 | -0.54 | -1.27 | 0.171 | 0.472 | 1.000 | 3856 | tags=60%, list=32%, signal=88% |
| 2169 | GOBP\_RETINA\_DEVELOPMENT\_IN\_CAMERA\_TYPE\_EYE |  | 91 | -0.36 | -1.27 | 0.091 | 0.473 | 1.000 | 3638 | tags=37%, list=30%, signal=53% |
| 2170 | GOMF\_METALLOEXOPEPTIDASE\_ACTIVITY |  | 42 | -0.40 | -1.27 | 0.125 | 0.473 | 1.000 | 3630 | tags=38%, list=30%, signal=54% |
| 2171 | GOBP\_REGULATION\_OF\_PROTEIN\_DEPHOSPHORYLATION |  | 96 | -0.35 | -1.27 | 0.084 | 0.473 | 1.000 | 4627 | tags=47%, list=39%, signal=76% |
| 2172 | GOBP\_REGULATION\_OF\_AEROBIC\_RESPIRATION |  | 5 | -0.66 | -1.27 | 0.151 | 0.473 | 1.000 | 3080 | tags=60%, list=26%, signal=81% |
| 2173 | GOBP\_REGULATION\_OF\_CYTOSOLIC\_CALCIUM\_ION\_CONCENTRATION |  | 209 | -0.33 | -1.27 | 0.040 | 0.474 | 1.000 | 3027 | tags=32%, list=25%, signal=42% |
| 2174 | GOMF\_OXIDOREDUCTASE\_ACTIVITY\_ACTING\_ON\_THE\_CH\_NH2\_GROUP\_OF\_DONORS\_OXYGEN\_AS\_ACCEPTOR |  | 15 | -0.49 | -1.27 | 0.167 | 0.474 | 1.000 | 2105 | tags=33%, list=18%, signal=40% |
| 2175 | GOBP\_SKELETAL\_MUSCLE\_SATELLITE\_CELL\_ACTIVATION |  | 5 | -0.66 | -1.27 | 0.155 | 0.474 | 1.000 | 2070 | tags=40%, list=17%, signal=48% |
| 2176 | GOBP\_CELLULAR\_RESPONSE\_TO\_INORGANIC\_SUBSTANCE |  | 133 | -0.35 | -1.27 | 0.065 | 0.474 | 1.000 | 3712 | tags=39%, list=31%, signal=56% |
| 2177 | GOBP\_RESPONSE\_TO\_CGMP |  | 5 | -0.66 | -1.27 | 0.158 | 0.474 | 1.000 | 1650 | tags=60%, list=14%, signal=70% |
| 2178 | GOBP\_REGULATION\_OF\_ANTIGEN\_PROCESSING\_AND\_PRESENTATION |  | 10 | -0.54 | -1.27 | 0.172 | 0.474 | 1.000 | 4776 | tags=60%, list=40%, signal=100% |
| 2179 | GOBP\_POSITIVE\_REGULATION\_OF\_FC\_RECEPTOR\_MEDIATED\_STIMULATORY\_SIGNALING\_PATHWAY |  | 7 | -0.60 | -1.27 | 0.167 | 0.474 | 1.000 | 2364 | tags=57%, list=20%, signal=71% |
| 2180 | GOBP\_REGULATION\_OF\_GLUTAMINE\_FAMILY\_AMINO\_ACID\_METABOLIC\_PROCESS |  | 5 | -0.66 | -1.27 | 0.155 | 0.474 | 1.000 | 273 | tags=20%, list=2%, signal=20% |
| 2181 | GOBP\_HORMONE\_METABOLIC\_PROCESS |  | 126 | -0.35 | -1.27 | 0.075 | 0.474 | 1.000 | 2666 | tags=30%, list=22%, signal=38% |
| 2182 | GOBP\_COMPLEMENT\_RECEPTOR\_MEDIATED\_SIGNALING\_PATHWAY |  | 3 | -0.77 | -1.27 | 0.133 | 0.474 | 1.000 | 1988 | tags=67%, list=17%, signal=80% |
| 2183 | GOBP\_MAINTENANCE\_OF\_LOCATION |  | 208 | -0.33 | -1.27 | 0.041 | 0.474 | 1.000 | 4187 | tags=43%, list=35%, signal=65% |
| 2184 | GOBP\_HEPARAN\_SULFATE\_PROTEOGLYCAN\_BIOSYNTHETIC\_PROCESS\_ENZYMATIC\_MODIFICATION |  | 4 | -0.70 | -1.27 | 0.145 | 0.474 | 1.000 | 3572 | tags=100%, list=30%, signal=142% |
| 2185 | GOBP\_TOLL\_LIKE\_RECEPTOR\_SIGNALING\_PATHWAY |  | 104 | -0.35 | -1.27 | 0.082 | 0.474 | 1.000 | 3217 | tags=38%, list=27%, signal=52% |
| 2186 | GOCC\_ASTROCYTE\_END\_FOOT |  | 4 | -0.70 | -1.27 | 0.137 | 0.474 | 1.000 | 3004 | tags=75%, list=25%, signal=100% |
| 2187 | GOBP\_REGULATION\_OF\_TOR\_SIGNALING |  | 74 | -0.36 | -1.27 | 0.096 | 0.474 | 1.000 | 3512 | tags=36%, list=29%, signal=51% |
| 2188 | GOBP\_POSITIVE\_REGULATION\_OF\_CELL\_SUBSTRATE\_JUNCTION\_ORGANIZATION |  | 27 | -0.43 | -1.27 | 0.157 | 0.474 | 1.000 | 3196 | tags=44%, list=27%, signal=60% |
| 2189 | GOBP\_INDOLALKYLAMINE\_METABOLIC\_PROCESS |  | 8 | -0.58 | -1.27 | 0.166 | 0.474 | 1.000 | 2516 | tags=38%, list=21%, signal=47% |
| 2190 | GOBP\_CELL\_CYCLE\_PHASE\_TRANSITION |  | 478 | -0.32 | -1.27 | 0.005 | 0.474 | 1.000 | 4443 | tags=41%, list=37%, signal=62% |
| 2191 | GOMF\_CALMODULIN\_BINDING |  | 133 | -0.34 | -1.27 | 0.068 | 0.474 | 1.000 | 3781 | tags=41%, list=31%, signal=60% |
| 2192 | GOMF\_TUMOR\_NECROSIS\_FACTOR\_ACTIVATED\_RECEPTOR\_ACTIVITY |  | 10 | -0.54 | -1.27 | 0.165 | 0.474 | 1.000 | 4300 | tags=70%, list=36%, signal=109% |
| 2193 | GOBP\_AMEBOIDAL\_TYPE\_CELL\_MIGRATION |  | 316 | -0.33 | -1.27 | 0.020 | 0.474 | 1.000 | 3196 | tags=32%, list=27%, signal=43% |
| 2194 | GOBP\_CEREBRAL\_CORTEX\_DEVELOPMENT |  | 77 | -0.36 | -1.27 | 0.099 | 0.474 | 1.000 | 633 | tags=14%, list=5%, signal=15% |
| 2195 | GOCC\_INTRINSIC\_COMPONENT\_OF\_POSTSYNAPTIC\_SPECIALIZATION\_MEMBRANE |  | 25 | -0.44 | -1.27 | 0.159 | 0.474 | 1.000 | 1346 | tags=28%, list=11%, signal=31% |
| 2196 | GOBP\_POSITIVE\_REGULATION\_OF\_MONONUCLEAR\_CELL\_MIGRATION |  | 39 | -0.40 | -1.27 | 0.139 | 0.474 | 1.000 | 2955 | tags=38%, list=25%, signal=51% |
| 2197 | GOBP\_RESOLUTION\_OF\_MEIOTIC\_RECOMBINATION\_INTERMEDIATES |  | 13 | -0.51 | -1.27 | 0.171 | 0.476 | 1.000 | 3969 | tags=62%, list=33%, signal=92% |
| 2198 | GOBP\_POSITIVE\_REGULATION\_OF\_DNA\_DEMETHYLATION |  | 3 | -0.76 | -1.27 | 0.133 | 0.476 | 1.000 | 727 | tags=33%, list=6%, signal=35% |
| 2199 | GOMF\_BETA\_1\_3\_GALACTOSYLTRANSFERASE\_ACTIVITY |  | 5 | -0.66 | -1.27 | 0.161 | 0.476 | 1.000 | 402 | tags=40%, list=3%, signal=41% |
| 2200 | GOBP\_MYOBLAST\_FUSION\_INVOLVED\_IN\_SKELETAL\_MUSCLE\_REGENERATION |  | 3 | -0.76 | -1.27 | 0.142 | 0.476 | 1.000 | 280 | tags=33%, list=2%, signal=34% |
| 2201 | GOBP\_FLOOR\_PLATE\_DEVELOPMENT |  | 4 | -0.70 | -1.27 | 0.138 | 0.476 | 1.000 | 1402 | tags=50%, list=12%, signal=57% |
| 2202 | GOBP\_GLIAL\_CELL\_MIGRATION |  | 35 | -0.41 | -1.27 | 0.148 | 0.476 | 1.000 | 4456 | tags=49%, list=37%, signal=77% |
| 2203 | GOBP\_REGULATION\_OF\_CELL\_COMMUNICATION\_BY\_ELECTRICAL\_COUPLING |  | 10 | -0.54 | -1.27 | 0.168 | 0.476 | 1.000 | 4627 | tags=70%, list=39%, signal=114% |
| 2204 | GOBP\_POSITIVE\_REGULATION\_OF\_EXCITATORY\_POSTSYNAPTIC\_POTENTIAL |  | 16 | -0.48 | -1.27 | 0.169 | 0.476 | 1.000 | 6011 | tags=94%, list=50%, signal=187% |
| 2205 | GOBP\_EXOCYTIC\_PROCESS |  | 59 | -0.37 | -1.27 | 0.121 | 0.476 | 1.000 | 3476 | tags=41%, list=29%, signal=57% |
| 2206 | GOMF\_VITAMIN\_D\_BINDING |  | 4 | -0.70 | -1.27 | 0.147 | 0.476 | 1.000 | 3114 | tags=75%, list=26%, signal=101% |
| 2207 | GOBP\_TRICUSPID\_VALVE\_DEVELOPMENT |  | 5 | -0.66 | -1.27 | 0.152 | 0.476 | 1.000 | 3314 | tags=80%, list=28%, signal=110% |
| 2208 | GOBP\_MESONEPHROS\_DEVELOPMENT |  | 66 | -0.37 | -1.27 | 0.111 | 0.477 | 1.000 | 2356 | tags=32%, list=20%, signal=39% |
| 2209 | GOBP\_NEGATIVE\_REGULATION\_OF\_DNA\_REPLICATION |  | 25 | -0.43 | -1.27 | 0.164 | 0.478 | 1.000 | 3888 | tags=52%, list=32%, signal=77% |
| 2210 | GOCC\_ENDOPLASMIC\_RETICULUM\_LUMEN |  | 214 | -0.33 | -1.27 | 0.035 | 0.477 | 1.000 | 5092 | tags=53%, list=42%, signal=90% |
| 2211 | GOBP\_REGULATION\_OF\_SUPRAMOLECULAR\_FIBER\_ORGANIZATION |  | 260 | -0.33 | -1.27 | 0.028 | 0.477 | 1.000 | 3069 | tags=32%, list=26%, signal=41% |
| 2212 | GOBP\_PHOSPHATIDYLCHOLINE\_BIOSYNTHETIC\_PROCESS |  | 29 | -0.42 | -1.27 | 0.151 | 0.477 | 1.000 | 1864 | tags=28%, list=16%, signal=33% |
| 2213 | GOBP\_POSITIVE\_REGULATION\_OF\_JNK\_CASCADE |  | 58 | -0.37 | -1.27 | 0.124 | 0.477 | 1.000 | 2769 | tags=36%, list=23%, signal=47% |
| 2214 | GOBP\_NEGATIVE\_REGULATION\_OF\_COAGULATION |  | 31 | -0.42 | -1.27 | 0.144 | 0.477 | 1.000 | 2205 | tags=29%, list=18%, signal=35% |
| 2215 | GOMF\_HORMONE\_RECEPTOR\_BINDING |  | 129 | -0.34 | -1.27 | 0.072 | 0.477 | 1.000 | 4455 | tags=49%, list=37%, signal=77% |
| 2216 | GOBP\_AMYLOID\_PRECURSOR\_PROTEIN\_CATABOLIC\_PROCESS |  | 42 | -0.40 | -1.27 | 0.134 | 0.478 | 1.000 | 2460 | tags=29%, list=20%, signal=36% |
| 2217 | GOBP\_TRIGLYCERIDE\_METABOLIC\_PROCESS |  | 70 | -0.37 | -1.27 | 0.107 | 0.478 | 1.000 | 2684 | tags=36%, list=22%, signal=46% |
| 2218 | GOCC\_CENTROSOME |  | 461 | -0.32 | -1.27 | 0.007 | 0.478 | 1.000 | 3914 | tags=36%, list=33%, signal=52% |
| 2219 | GOBP\_ANDROGEN\_RECEPTOR\_SIGNALING\_PATHWAY |  | 28 | -0.43 | -1.27 | 0.155 | 0.478 | 1.000 | 4467 | tags=54%, list=37%, signal=85% |
| 2220 | GOBP\_HEPATICOBILIARY\_SYSTEM\_DEVELOPMENT |  | 106 | -0.35 | -1.27 | 0.090 | 0.478 | 1.000 | 2187 | tags=25%, list=18%, signal=31% |
| 2221 | GOBP\_POSITIVE\_REGULATION\_OF\_SPINDLE\_ASSEMBLY |  | 5 | -0.66 | -1.27 | 0.156 | 0.478 | 1.000 | 3448 | tags=60%, list=29%, signal=84% |
| 2222 | GOBP\_CARDIAC\_MUSCLE\_CELL\_FATE\_COMMITMENT |  | 3 | -0.76 | -1.27 | 0.146 | 0.478 | 1.000 | 2212 | tags=67%, list=18%, signal=82% |
| 2223 | GOCC\_AXON\_HILLOCK |  | 6 | -0.62 | -1.27 | 0.168 | 0.478 | 1.000 | 3148 | tags=50%, list=26%, signal=68% |
| 2224 | GOBP\_SUBSTRATE\_DEPENDENT\_CELL\_MIGRATION |  | 20 | -0.46 | -1.27 | 0.164 | 0.478 | 1.000 | 3691 | tags=45%, list=31%, signal=65% |
| 2225 | GOBP\_AUTOPHAGOSOME\_ORGANIZATION |  | 77 | -0.36 | -1.27 | 0.107 | 0.478 | 1.000 | 4585 | tags=42%, list=38%, signal=67% |
| 2226 | GOBP\_LUTEINIZING\_HORMONE\_SECRETION |  | 4 | -0.70 | -1.27 | 0.156 | 0.478 | 1.000 | 1100 | tags=50%, list=9%, signal=55% |
| 2227 | GOBP\_COGNITION |  | 167 | -0.34 | -1.27 | 0.056 | 0.479 | 1.000 | 2631 | tags=27%, list=22%, signal=34% |
| 2228 | GOCC\_PHOSPHOLIPID\_TRANSLOCATING\_ATPASE\_COMPLEX |  | 7 | -0.60 | -1.27 | 0.162 | 0.478 | 1.000 | 1201 | tags=43%, list=10%, signal=48% |
| 2229 | GOBP\_DEFINITIVE\_HEMOPOIESIS |  | 17 | -0.47 | -1.27 | 0.173 | 0.479 | 1.000 | 2921 | tags=35%, list=24%, signal=47% |
| 2230 | GOBP\_NEGATIVE\_REGULATION\_OF\_NIK\_NF\_KAPPAB\_SIGNALING |  | 20 | -0.46 | -1.27 | 0.160 | 0.479 | 1.000 | 3447 | tags=45%, list=29%, signal=63% |
| 2231 | GOBP\_TETRAHYDROFOLATE\_BIOSYNTHETIC\_PROCESS |  | 6 | -0.63 | -1.27 | 0.167 | 0.479 | 1.000 | 4242 | tags=83%, list=35%, signal=129% |
| 2232 | GOBP\_MEMBRANE\_DISASSEMBLY |  | 13 | -0.50 | -1.27 | 0.168 | 0.479 | 1.000 | 4378 | tags=54%, list=36%, signal=85% |
| 2233 | GOBP\_NEGATIVE\_REGULATION\_OF\_STEROID\_METABOLIC\_PROCESS |  | 20 | -0.45 | -1.27 | 0.168 | 0.479 | 1.000 | 2117 | tags=40%, list=18%, signal=48% |
| 2234 | GOBP\_REGULATION\_OF\_CELLULAR\_COMPONENT\_BIOGENESIS |  | 682 | -0.32 | -1.27 | 0.002 | 0.479 | 1.000 | 3723 | tags=36%, list=31%, signal=49% |
| 2235 | GOBP\_HISTONE\_H3\_ACETYLATION |  | 48 | -0.39 | -1.27 | 0.134 | 0.480 | 1.000 | 3523 | tags=40%, list=29%, signal=56% |
| 2236 | GOBP\_MITOTIC\_CHROMOSOME\_CONDENSATION |  | 11 | -0.53 | -1.27 | 0.177 | 0.480 | 1.000 | 5193 | tags=82%, list=43%, signal=144% |
| 2237 | GOBP\_MOTOR\_NEURON\_AXON\_GUIDANCE |  | 17 | -0.47 | -1.27 | 0.164 | 0.480 | 1.000 | 4827 | tags=53%, list=40%, signal=88% |
| 2238 | GOBP\_STEROL\_HOMEOSTASIS |  | 56 | -0.38 | -1.26 | 0.133 | 0.480 | 1.000 | 3403 | tags=41%, list=28%, signal=57% |
| 2239 | GOBP\_NEGATIVE\_REGULATION\_OF\_CYTOSKELETON\_ORGANIZATION |  | 105 | -0.35 | -1.26 | 0.083 | 0.480 | 1.000 | 2634 | tags=30%, list=22%, signal=39% |
| 2240 | GOBP\_REGULATION\_OF\_DENDRITIC\_CELL\_ANTIGEN\_PROCESSING\_AND\_PRESENTATION |  | 7 | -0.60 | -1.26 | 0.167 | 0.480 | 1.000 | 2847 | tags=43%, list=24%, signal=56% |
| 2241 | GOBP\_POSITIVE\_REGULATION\_OF\_CYTOSKELETON\_ORGANIZATION |  | 162 | -0.34 | -1.26 | 0.055 | 0.480 | 1.000 | 3069 | tags=31%, list=26%, signal=42% |
| 2242 | GOBP\_TISSUE\_MORPHOGENESIS |  | 422 | -0.32 | -1.26 | 0.010 | 0.480 | 1.000 | 3619 | tags=33%, list=30%, signal=46% |
| 2243 | GOBP\_PATTERN\_SPECIFICATION\_PROCESS |  | 252 | -0.33 | -1.26 | 0.032 | 0.480 | 1.000 | 3596 | tags=35%, list=30%, signal=49% |
| 2244 | GOCC\_SPECIFIC\_GRANULE\_MEMBRANE |  | 59 | -0.37 | -1.26 | 0.121 | 0.480 | 1.000 | 4763 | tags=59%, list=40%, signal=98% |
| 2245 | GOBP\_REGULATION\_OF\_HORMONE\_LEVELS |  | 318 | -0.33 | -1.26 | 0.016 | 0.480 | 1.000 | 2446 | tags=27%, list=20%, signal=33% |
| 2246 | GOBP\_AMYLOID\_PRECURSOR\_PROTEIN\_BIOSYNTHETIC\_PROCESS |  | 12 | -0.51 | -1.26 | 0.180 | 0.480 | 1.000 | 190 | tags=17%, list=2%, signal=17% |
| 2247 | GOMF\_SELENIUM\_BINDING |  | 5 | -0.65 | -1.26 | 0.161 | 0.480 | 1.000 | 1253 | tags=40%, list=10%, signal=45% |
| 2248 | GOBP\_CONVERGENT\_EXTENSION\_INVOLVED\_IN\_ORGANOGENESIS |  | 4 | -0.70 | -1.26 | 0.153 | 0.480 | 1.000 | 1318 | tags=25%, list=11%, signal=28% |
| 2249 | GOBP\_STEM\_CELL\_PROLIFERATION |  | 43 | -0.39 | -1.26 | 0.135 | 0.480 | 1.000 | 3400 | tags=35%, list=28%, signal=48% |
| 2250 | GOBP\_RESPONSE\_TO\_MUSCLE\_INACTIVITY |  | 6 | -0.62 | -1.26 | 0.169 | 0.480 | 1.000 | 3295 | tags=67%, list=27%, signal=92% |
| 2251 | GOBP\_LYSOSOMAL\_MICROAUTOPHAGY |  | 4 | -0.69 | -1.26 | 0.164 | 0.480 | 1.000 | 3675 | tags=100%, list=31%, signal=144% |
| 2252 | GOBP\_SPERMATID\_DIFFERENTIATION |  | 76 | -0.36 | -1.26 | 0.102 | 0.480 | 1.000 | 2761 | tags=32%, list=23%, signal=41% |
| 2253 | GOMF\_CILIARY\_NEUROTROPHIC\_FACTOR\_RECEPTOR\_ACTIVITY |  | 8 | -0.57 | -1.26 | 0.177 | 0.480 | 1.000 | 4205 | tags=50%, list=35%, signal=77% |
| 2254 | GOBP\_POSITIVE\_REGULATION\_OF\_DENDRITIC\_SPINE\_DEVELOPMENT |  | 33 | -0.41 | -1.26 | 0.146 | 0.480 | 1.000 | 4673 | tags=52%, list=39%, signal=84% |
| 2255 | GOMF\_DNA\_BINDING\_TRANSCRIPTION\_ACTIVATOR\_ACTIVITY |  | 233 | -0.33 | -1.26 | 0.036 | 0.480 | 1.000 | 3862 | tags=42%, list=32%, signal=61% |
| 2256 | GOBP\_RESPONSE\_TO\_VITAMIN |  | 60 | -0.37 | -1.26 | 0.116 | 0.480 | 1.000 | 2808 | tags=35%, list=23%, signal=45% |
| 2257 | GOBP\_NEGATIVE\_REGULATION\_OF\_GLIOGENESIS |  | 20 | -0.45 | -1.26 | 0.158 | 0.480 | 1.000 | 1297 | tags=25%, list=11%, signal=28% |
| 2258 | GOCC\_FIBRINOGEN\_COMPLEX |  | 6 | -0.62 | -1.26 | 0.163 | 0.480 | 1.000 | 3186 | tags=67%, list=27%, signal=91% |
| 2259 | GOBP\_MODULATION\_OF\_EXCITATORY\_POSTSYNAPTIC\_POTENTIAL |  | 26 | -0.43 | -1.26 | 0.161 | 0.480 | 1.000 | 6011 | tags=73%, list=50%, signal=146% |
| 2260 | GOBP\_RESPONSE\_TO\_CADMIUM\_ION |  | 33 | -0.41 | -1.26 | 0.151 | 0.480 | 1.000 | 1692 | tags=27%, list=14%, signal=32% |
| 2261 | GOMF\_MANGANESE\_ION\_BINDING |  | 51 | -0.38 | -1.26 | 0.132 | 0.480 | 1.000 | 2617 | tags=29%, list=22%, signal=37% |
| 2262 | GOBP\_REGULATION\_OF\_HISTONE\_H4\_ACETYLATION |  | 11 | -0.53 | -1.26 | 0.178 | 0.480 | 1.000 | 3395 | tags=55%, list=28%, signal=76% |
| 2263 | GOBP\_ACTIVATION\_OF\_PHOSPHOLIPASE\_C\_ACTIVITY |  | 20 | -0.45 | -1.26 | 0.171 | 0.480 | 1.000 | 1411 | tags=25%, list=12%, signal=28% |
| 2264 | GOCC\_MEIOTIC\_SPINDLE |  | 6 | -0.62 | -1.26 | 0.166 | 0.480 | 1.000 | 2962 | tags=67%, list=25%, signal=88% |
| 2265 | GOBP\_POSITIVE\_REGULATION\_OF\_NEURON\_MIGRATION |  | 11 | -0.53 | -1.26 | 0.173 | 0.480 | 1.000 | 3833 | tags=55%, list=32%, signal=80% |
| 2266 | GOBP\_TRICUSPID\_VALVE\_MORPHOGENESIS |  | 5 | -0.66 | -1.26 | 0.163 | 0.480 | 1.000 | 3314 | tags=80%, list=28%, signal=110% |
| 2267 | GOBP\_REGULATION\_OF\_PROTEIN\_CONTAINING\_COMPLEX\_ASSEMBLY |  | 309 | -0.32 | -1.26 | 0.022 | 0.480 | 1.000 | 3072 | tags=32%, list=26%, signal=42% |
| 2268 | GOBP\_NEGATIVE\_REGULATION\_OF\_CELLULAR\_RESPONSE\_TO\_GROWTH\_FACTOR\_STIMULUS |  | 70 | -0.37 | -1.26 | 0.119 | 0.479 | 1.000 | 3126 | tags=41%, list=26%, signal=56% |
| 2269 | GOMF\_TRANSCRIPTION\_REGULATOR\_ACTIVITY |  | 890 | -0.32 | -1.26 | 0.001 | 0.480 | 1.000 | 3907 | tags=40%, list=33%, signal=54% |
| 2270 | GOBP\_FOREBRAIN\_DEVELOPMENT |  | 240 | -0.33 | -1.26 | 0.033 | 0.480 | 1.000 | 4822 | tags=48%, list=40%, signal=79% |
| 2271 | GOBP\_LENS\_DEVELOPMENT\_IN\_CAMERA\_TYPE\_EYE |  | 44 | -0.39 | -1.26 | 0.141 | 0.480 | 1.000 | 3615 | tags=48%, list=30%, signal=68% |
| 2272 | GOCC\_MICROTUBULE\_ORGANIZING\_CENTER |  | 580 | -0.32 | -1.26 | 0.003 | 0.480 | 1.000 | 3914 | tags=36%, list=33%, signal=51% |
| 2273 | GOBP\_DNA\_REPAIR |  | 416 | -0.32 | -1.26 | 0.014 | 0.480 | 1.000 | 3969 | tags=40%, list=33%, signal=58% |
| 2274 | GOBP\_DNA\_METHYLATION |  | 43 | -0.39 | -1.26 | 0.139 | 0.480 | 1.000 | 4130 | tags=47%, list=34%, signal=71% |
| 2275 | GOBP\_MEMBRANE\_PROTEIN\_PROTEOLYSIS |  | 48 | -0.39 | -1.26 | 0.128 | 0.480 | 1.000 | 3168 | tags=33%, list=26%, signal=45% |
| 2276 | GOMF\_MUTLALPHA\_COMPLEX\_BINDING |  | 5 | -0.66 | -1.26 | 0.160 | 0.480 | 1.000 | 2104 | tags=60%, list=18%, signal=73% |
| 2277 | GOBP\_ESTROGEN\_BIOSYNTHETIC\_PROCESS |  | 5 | -0.65 | -1.26 | 0.168 | 0.480 | 1.000 | 101 | tags=20%, list=1%, signal=20% |
| 2278 | GOCC\_POSTSYNAPTIC\_DENSITY\_MEMBRANE |  | 39 | -0.40 | -1.26 | 0.135 | 0.480 | 1.000 | 2604 | tags=33%, list=22%, signal=42% |
| 2279 | GOBP\_MICROTUBULE\_BASED\_MOVEMENT |  | 228 | -0.33 | -1.26 | 0.041 | 0.480 | 1.000 | 3612 | tags=36%, list=30%, signal=50% |
| 2280 | GOMF\_HELICASE\_ACTIVITY |  | 118 | -0.35 | -1.26 | 0.082 | 0.480 | 1.000 | 4717 | tags=55%, list=39%, signal=90% |
| 2281 | GOBP\_FOREBRAIN\_RADIAL\_GLIAL\_CELL\_DIFFERENTIATION |  | 5 | -0.65 | -1.26 | 0.176 | 0.480 | 1.000 | 624 | tags=40%, list=5%, signal=42% |
| 2282 | GOBP\_SNRNA\_TRANSCRIPTION\_BY\_RNA\_POLYMERASE\_III |  | 8 | -0.57 | -1.26 | 0.179 | 0.480 | 1.000 | 1548 | tags=38%, list=13%, signal=43% |
| 2283 | GOMF\_LIPID\_TRANSFER\_ACTIVITY |  | 34 | -0.41 | -1.26 | 0.148 | 0.480 | 1.000 | 3217 | tags=41%, list=27%, signal=56% |
| 2284 | GOMF\_GABA\_RECEPTOR\_BINDING |  | 9 | -0.55 | -1.26 | 0.171 | 0.480 | 1.000 | 1707 | tags=44%, list=14%, signal=52% |
| 2285 | GOBP\_WNT\_SIGNALING\_PATHWAY\_CALCIUM\_MODULATING\_PATHWAY |  | 29 | -0.42 | -1.26 | 0.165 | 0.480 | 1.000 | 4145 | tags=52%, list=35%, signal=79% |
| 2286 | GOBP\_NEGATIVE\_REGULATION\_OF\_NUCLEOBASE\_CONTAINING\_COMPOUND\_METABOLIC\_PROCESS |  | 901 | -0.32 | -1.26 | 0.001 | 0.480 | 1.000 | 4362 | tags=43%, list=36%, signal=62% |
| 2287 | GOMF\_TRANSMEMBRANE\_RECEPTOR\_PROTEIN\_TYROSINE\_KINASE\_ACTIVATOR\_ACTIVITY |  | 5 | -0.66 | -1.26 | 0.166 | 0.481 | 1.000 | 648 | tags=40%, list=5%, signal=42% |
| 2288 | GOMF\_PALMITOYLTRANSFERASE\_ACTIVITY |  | 28 | -0.42 | -1.26 | 0.155 | 0.481 | 1.000 | 2162 | tags=29%, list=18%, signal=35% |
| 2289 | GOBP\_REGULATION\_OF\_ANION\_CHANNEL\_ACTIVITY |  | 5 | -0.66 | -1.26 | 0.161 | 0.480 | 1.000 | 2891 | tags=60%, list=24%, signal=79% |
| 2290 | GOBP\_GLAND\_DEVELOPMENT |  | 293 | -0.33 | -1.26 | 0.024 | 0.480 | 1.000 | 3225 | tags=31%, list=27%, signal=42% |
| 2291 | GOBP\_POSITIVE\_REGULATION\_OF\_CELL\_CYCLE\_G2\_M\_PHASE\_TRANSITION |  | 23 | -0.44 | -1.26 | 0.163 | 0.481 | 1.000 | 5627 | tags=74%, list=47%, signal=139% |
| 2292 | GOBP\_REGULATION\_OF\_PLASMINOGEN\_ACTIVATION |  | 8 | -0.57 | -1.26 | 0.178 | 0.481 | 1.000 | 2820 | tags=63%, list=23%, signal=82% |
| 2293 | GOBP\_NEGATIVE\_REGULATION\_OF\_MULTICELLULAR\_ORGANISMAL\_PROCESS |  | 616 | -0.32 | -1.26 | 0.002 | 0.481 | 1.000 | 3186 | tags=32%, list=27%, signal=42% |
| 2294 | GOMF\_TOXIC\_SUBSTANCE\_BINDING |  | 8 | -0.57 | -1.26 | 0.174 | 0.481 | 1.000 | 1628 | tags=38%, list=14%, signal=43% |
| 2295 | GOBP\_TELOMERASE\_HOLOENZYME\_COMPLEX\_ASSEMBLY |  | 6 | -0.62 | -1.26 | 0.181 | 0.481 | 1.000 | 2010 | tags=50%, list=17%, signal=60% |
| 2296 | GOBP\_SYNAPTIC\_VESICLE\_PRIMING |  | 15 | -0.48 | -1.26 | 0.181 | 0.481 | 1.000 | 2312 | tags=60%, list=19%, signal=74% |
| 2297 | GOMF\_KINASE\_ACTIVATOR\_ACTIVITY |  | 66 | -0.37 | -1.26 | 0.121 | 0.481 | 1.000 | 3408 | tags=35%, list=28%, signal=48% |
| 2298 | GOCC\_INTRINSIC\_COMPONENT\_OF\_GOLGI\_MEMBRANE |  | 51 | -0.38 | -1.26 | 0.136 | 0.481 | 1.000 | 2916 | tags=31%, list=24%, signal=41% |
| 2299 | GOCC\_TERMINAL\_BOUTON |  | 35 | -0.41 | -1.26 | 0.144 | 0.481 | 1.000 | 2876 | tags=43%, list=24%, signal=56% |
| 2300 | GOBP\_POSITIVE\_REGULATION\_OF\_NON\_MOTILE\_CILIUM\_ASSEMBLY |  | 5 | -0.66 | -1.26 | 0.168 | 0.481 | 1.000 | 2688 | tags=40%, list=22%, signal=52% |
| 2301 | GOMF\_OXIDOREDUCTASE\_ACTIVITY\_ACTING\_ON\_THE\_ALDEHYDE\_OR\_OXO\_GROUP\_OF\_DONORS\_NAD\_OR\_NADP\_AS\_ACCEPTOR |  | 25 | -0.43 | -1.26 | 0.162 | 0.481 | 1.000 | 2534 | tags=28%, list=21%, signal=35% |
| 2302 | GOBP\_IMMUNOLOGICAL\_SYNAPSE\_FORMATION |  | 8 | -0.57 | -1.26 | 0.184 | 0.481 | 1.000 | 2604 | tags=38%, list=22%, signal=48% |
| 2303 | GOBP\_REGULATION\_OF\_GLIAL\_CELL\_MIGRATION |  | 13 | -0.50 | -1.26 | 0.181 | 0.481 | 1.000 | 4868 | tags=62%, list=41%, signal=103% |
| 2304 | GOBP\_GOLGI\_TO\_VACUOLE\_TRANSPORT |  | 14 | -0.49 | -1.26 | 0.180 | 0.481 | 1.000 | 3227 | tags=43%, list=27%, signal=59% |
| 2305 | GOCC\_AXON\_CYTOPLASM |  | 47 | -0.38 | -1.26 | 0.135 | 0.481 | 1.000 | 4052 | tags=45%, list=34%, signal=67% |
| 2306 | GOBP\_PROTEIN\_TRANSPORT\_TO\_VACUOLE\_INVOLVED\_IN\_UBIQUITIN\_DEPENDENT\_PROTEIN\_CATABOLIC\_PROCESS\_VIA\_THE\_MULTIVESICULAR\_BODY\_SORTING\_PATHWAY |  | 4 | -0.69 | -1.26 | 0.160 | 0.483 | 1.000 | 1824 | tags=50%, list=15%, signal=59% |
| 2307 | GOBP\_REGULATION\_OF\_DOPAMINERGIC\_NEURON\_DIFFERENTIATION |  | 6 | -0.62 | -1.26 | 0.175 | 0.483 | 1.000 | 1579 | tags=33%, list=13%, signal=38% |
| 2308 | GOCC\_PROTEIN\_KINASE\_COMPLEX |  | 80 | -0.36 | -1.26 | 0.107 | 0.483 | 1.000 | 2458 | tags=30%, list=20%, signal=37% |
| 2309 | GOBP\_NEURON\_CELLULAR\_HOMEOSTASIS |  | 24 | -0.44 | -1.26 | 0.170 | 0.483 | 1.000 | 2835 | tags=38%, list=24%, signal=49% |
| 2310 | GOCC\_SORTING\_ENDOSOME |  | 5 | -0.66 | -1.26 | 0.165 | 0.483 | 1.000 | 2791 | tags=80%, list=23%, signal=104% |
| 2311 | GOCC\_CUL4\_RING\_E3\_UBIQUITIN\_LIGASE\_COMPLEX |  | 26 | -0.43 | -1.26 | 0.165 | 0.485 | 1.000 | 3958 | tags=50%, list=33%, signal=74% |
| 2312 | GOBP\_SYNAPTIC\_MEMBRANE\_ADHESION |  | 15 | -0.48 | -1.26 | 0.178 | 0.484 | 1.000 | 5068 | tags=73%, list=42%, signal=127% |
| 2313 | GOCC\_TRANSCRIPTION\_FACTOR\_TFIIIC\_COMPLEX |  | 5 | -0.65 | -1.26 | 0.163 | 0.484 | 1.000 | 3625 | tags=60%, list=30%, signal=86% |
| 2314 | GOBP\_TYPE\_2\_IMMUNE\_RESPONSE |  | 21 | -0.45 | -1.26 | 0.175 | 0.484 | 1.000 | 1788 | tags=43%, list=15%, signal=50% |
| 2315 | GOMF\_VOLUME\_SENSITIVE\_ANION\_CHANNEL\_ACTIVITY |  | 6 | -0.62 | -1.26 | 0.175 | 0.484 | 1.000 | 4189 | tags=83%, list=35%, signal=128% |
| 2316 | GOBP\_REGULATION\_OF\_DNA\_METHYLATION\_DEPENDENT\_HETEROCHROMATIN\_ASSEMBLY |  | 7 | -0.59 | -1.26 | 0.178 | 0.484 | 1.000 | 875 | tags=29%, list=7%, signal=31% |
| 2317 | GOBP\_RESPONSE\_TO\_IONIZING\_RADIATION |  | 110 | -0.35 | -1.26 | 0.087 | 0.484 | 1.000 | 3927 | tags=45%, list=33%, signal=67% |
| 2318 | GOBP\_RECEPTOR\_MEDIATED\_VIRION\_ATTACHMENT\_TO\_HOST\_CELL |  | 3 | -0.75 | -1.26 | 0.144 | 0.484 | 1.000 | 1799 | tags=33%, list=15%, signal=39% |
| 2319 | GOBP\_NEGATIVE\_REGULATION\_OF\_MRNA\_CATABOLIC\_PROCESS |  | 34 | -0.41 | -1.26 | 0.153 | 0.484 | 1.000 | 5048 | tags=59%, list=42%, signal=101% |
| 2320 | GOBP\_FOREBRAIN\_CELL\_MIGRATION |  | 36 | -0.40 | -1.26 | 0.156 | 0.484 | 1.000 | 4112 | tags=44%, list=34%, signal=67% |
| 2321 | GOMF\_ARYLSULFATASE\_ACTIVITY |  | 11 | -0.53 | -1.26 | 0.188 | 0.484 | 1.000 | 3456 | tags=55%, list=29%, signal=77% |
| 2322 | GOBP\_REGULATION\_OF\_ANATOMICAL\_STRUCTURE\_SIZE |  | 348 | -0.32 | -1.26 | 0.019 | 0.484 | 1.000 | 3069 | tags=31%, list=26%, signal=40% |
| 2323 | GOBP\_CHROMATIN\_SILENCING |  | 31 | -0.41 | -1.26 | 0.154 | 0.484 | 1.000 | 4649 | tags=52%, list=39%, signal=84% |
| 2324 | GOBP\_POSITIVE\_REGULATION\_OF\_I\_KAPPAB\_KINASE\_NF\_KAPPAB\_SIGNALING |  | 127 | -0.34 | -1.26 | 0.078 | 0.484 | 1.000 | 3166 | tags=35%, list=26%, signal=47% |
| 2325 | GOBP\_N\_ACYLETHANOLAMINE\_METABOLIC\_PROCESS |  | 4 | -0.69 | -1.26 | 0.150 | 0.484 | 1.000 | 3669 | tags=100%, list=31%, signal=144% |
| 2326 | GOCC\_GLUTAMATERGIC\_SYNAPSE |  | 218 | -0.33 | -1.26 | 0.045 | 0.484 | 1.000 | 3739 | tags=35%, list=31%, signal=50% |
| 2327 | GOMF\_L\_ASCORBIC\_ACID\_BINDING |  | 17 | -0.47 | -1.26 | 0.178 | 0.484 | 1.000 | 4639 | tags=53%, list=39%, signal=86% |
| 2328 | GOBP\_MEMBRANE\_DEPOLARIZATION |  | 59 | -0.37 | -1.26 | 0.129 | 0.484 | 1.000 | 1641 | tags=20%, list=14%, signal=23% |
| 2329 | GOBP\_DNA\_ENDOREDUPLICATION |  | 5 | -0.65 | -1.26 | 0.171 | 0.485 | 1.000 | 167 | tags=20%, list=1%, signal=20% |
| 2330 | GOMF\_NADPLUS\_NUCLEOSIDASE\_ACTIVITY |  | 10 | -0.54 | -1.26 | 0.181 | 0.485 | 1.000 | 3487 | tags=60%, list=29%, signal=84% |
| 2331 | GOMF\_MYOSIN\_LIGHT\_CHAIN\_BINDING |  | 5 | -0.65 | -1.26 | 0.170 | 0.485 | 1.000 | 4112 | tags=80%, list=34%, signal=122% |
| 2332 | GOMF\_SPHINGOLIPID\_TRANSPORTER\_ACTIVITY |  | 13 | -0.50 | -1.26 | 0.186 | 0.486 | 1.000 | 3366 | tags=54%, list=28%, signal=75% |
| 2333 | GOMF\_PASSIVE\_TRANSMEMBRANE\_TRANSPORTER\_ACTIVITY |  | 231 | -0.33 | -1.26 | 0.047 | 0.486 | 1.000 | 2216 | tags=26%, list=18%, signal=31% |
| 2334 | GOBP\_TOLERANCE\_INDUCTION\_TO\_SELF\_ANTIGEN |  | 4 | -0.69 | -1.26 | 0.164 | 0.486 | 1.000 | 1470 | tags=50%, list=12%, signal=57% |
| 2335 | GOMF\_DNA\_BINDING\_TRANSCRIPTION\_REPRESSOR\_ACTIVITY |  | 154 | -0.33 | -1.26 | 0.073 | 0.486 | 1.000 | 3979 | tags=46%, list=33%, signal=68% |
| 2336 | GOBP\_ESTABLISHMENT\_OF\_EPITHELIAL\_CELL\_POLARITY |  | 23 | -0.44 | -1.26 | 0.171 | 0.486 | 1.000 | 2995 | tags=39%, list=25%, signal=52% |
| 2337 | GOBP\_MITOTIC\_CELL\_CYCLE |  | 783 | -0.32 | -1.26 | 0.001 | 0.486 | 1.000 | 4598 | tags=43%, list=38%, signal=66% |
| 2338 | GOMF\_UBIQUITIN\_LIKE\_PROTEIN\_CONJUGATING\_ENZYME\_BINDING |  | 27 | -0.42 | -1.26 | 0.166 | 0.486 | 1.000 | 1748 | tags=26%, list=15%, signal=30% |
| 2339 | GOBP\_REGULATION\_OF\_EXOCYTOSIS |  | 135 | -0.34 | -1.26 | 0.078 | 0.486 | 1.000 | 3603 | tags=37%, list=30%, signal=52% |
| 2340 | GOBP\_ROOF\_OF\_MOUTH\_DEVELOPMENT |  | 62 | -0.37 | -1.26 | 0.130 | 0.485 | 1.000 | 5410 | tags=53%, list=45%, signal=96% |
| 2341 | GOBP\_POSITIVE\_REGULATION\_OF\_GROWTH |  | 162 | -0.33 | -1.26 | 0.067 | 0.485 | 1.000 | 3341 | tags=33%, list=28%, signal=46% |
| 2342 | GOMF\_TELOMERIC\_DNA\_BINDING |  | 32 | -0.41 | -1.26 | 0.157 | 0.485 | 1.000 | 3927 | tags=50%, list=33%, signal=74% |
| 2343 | GOBP\_REGULATION\_OF\_RNA\_EXPORT\_FROM\_NUCLEUS |  | 12 | -0.51 | -1.26 | 0.188 | 0.485 | 1.000 | 4278 | tags=58%, list=36%, signal=91% |
| 2344 | GOMF\_OXIDOREDUCTASE\_ACTIVITY\_ACTING\_ON\_THE\_CH\_NH2\_GROUP\_OF\_DONORS |  | 17 | -0.47 | -1.26 | 0.177 | 0.486 | 1.000 | 2105 | tags=29%, list=18%, signal=36% |
| 2345 | GOBP\_REGULATION\_OF\_CELL\_SHAPE |  | 109 | -0.34 | -1.26 | 0.096 | 0.486 | 1.000 | 4152 | tags=47%, list=35%, signal=71% |
| 2346 | GOBP\_NEGATIVE\_REGULATION\_OF\_PROTEIN\_MODIFICATION\_PROCESS |  | 378 | -0.32 | -1.26 | 0.015 | 0.486 | 1.000 | 4008 | tags=39%, list=33%, signal=57% |
| 2347 | GOBP\_NEGATIVE\_REGULATION\_OF\_HISTONE\_ACETYLATION |  | 13 | -0.50 | -1.26 | 0.184 | 0.486 | 1.000 | 3395 | tags=46%, list=28%, signal=64% |
| 2348 | GOBP\_INTERMEMBRANE\_LIPID\_TRANSFER |  | 34 | -0.41 | -1.25 | 0.158 | 0.486 | 1.000 | 3217 | tags=41%, list=27%, signal=56% |
| 2349 | GOBP\_HORMONE\_TRANSPORT |  | 203 | -0.33 | -1.25 | 0.049 | 0.487 | 1.000 | 2367 | tags=27%, list=20%, signal=33% |
| 2350 | GOBP\_DNA\_REPLICATION |  | 232 | -0.33 | -1.25 | 0.043 | 0.487 | 1.000 | 5292 | tags=53%, list=44%, signal=94% |
| 2351 | GOBP\_NEGATIVE\_REGULATION\_OF\_LIPID\_BIOSYNTHETIC\_PROCESS |  | 37 | -0.40 | -1.25 | 0.155 | 0.487 | 1.000 | 2667 | tags=41%, list=22%, signal=52% |
| 2352 | GOBP\_STRAND\_INVASION |  | 4 | -0.69 | -1.25 | 0.162 | 0.486 | 1.000 | 2724 | tags=50%, list=23%, signal=65% |
| 2353 | GOBP\_POSITIVE\_REGULATION\_OF\_STRESS\_FIBER\_ASSEMBLY |  | 44 | -0.39 | -1.25 | 0.146 | 0.486 | 1.000 | 2560 | tags=32%, list=21%, signal=40% |
| 2354 | GOCC\_EXTRINSIC\_COMPONENT\_OF\_PRESYNAPTIC\_MEMBRANE |  | 5 | -0.65 | -1.25 | 0.173 | 0.486 | 1.000 | 1298 | tags=40%, list=11%, signal=45% |
| 2355 | GOBP\_PLASMINOGEN\_ACTIVATION |  | 13 | -0.50 | -1.25 | 0.176 | 0.486 | 1.000 | 2820 | tags=54%, list=23%, signal=70% |
| 2356 | GOBP\_MESODERMAL\_CELL\_FATE\_SPECIFICATION |  | 4 | -0.70 | -1.25 | 0.162 | 0.486 | 1.000 | 14 | tags=25%, list=0%, signal=25% |
| 2357 | GOBP\_PROTEIN\_LOCALIZATION\_TO\_MICROTUBULE\_ORGANIZING\_CENTER |  | 29 | -0.42 | -1.25 | 0.166 | 0.487 | 1.000 | 2479 | tags=34%, list=21%, signal=43% |
| 2358 | GOCC\_SPECIFIC\_GRANULE\_LUMEN |  | 33 | -0.41 | -1.25 | 0.158 | 0.486 | 1.000 | 4357 | tags=55%, list=36%, signal=85% |
| 2359 | GOBP\_TYPE\_I\_PNEUMOCYTE\_DIFFERENTIATION |  | 4 | -0.69 | -1.25 | 0.160 | 0.487 | 1.000 | 711 | tags=50%, list=6%, signal=53% |
| 2360 | GOBP\_NEGATIVE\_REGULATION\_OF\_SMOOTHENED\_SIGNALING\_PATHWAY |  | 26 | -0.42 | -1.25 | 0.171 | 0.487 | 1.000 | 4097 | tags=46%, list=34%, signal=70% |
| 2361 | GOBP\_ACTIN\_FILAMENT\_ORGANIZATION |  | 309 | -0.32 | -1.25 | 0.029 | 0.486 | 1.000 | 3069 | tags=31%, list=26%, signal=41% |
| 2362 | GOBP\_SPINDLE\_ORGANIZATION |  | 144 | -0.34 | -1.25 | 0.077 | 0.486 | 1.000 | 5629 | tags=58%, list=47%, signal=107% |
| 2363 | GOBP\_POSITIVE\_REGULATION\_OF\_CREB\_TRANSCRIPTION\_FACTOR\_ACTIVITY |  | 12 | -0.51 | -1.25 | 0.189 | 0.487 | 1.000 | 3196 | tags=50%, list=27%, signal=68% |
| 2364 | GOBP\_NEGATIVE\_REGULATION\_OF\_RHO\_PROTEIN\_SIGNAL\_TRANSDUCTION |  | 17 | -0.47 | -1.25 | 0.188 | 0.487 | 1.000 | 3107 | tags=41%, list=26%, signal=55% |
| 2365 | GOBP\_REGULATION\_OF\_CELLULAR\_RESPONSE\_TO\_TRANSFORMING\_GROWTH\_FACTOR\_BETA\_STIMULUS |  | 86 | -0.35 | -1.25 | 0.115 | 0.487 | 1.000 | 4358 | tags=47%, list=36%, signal=72% |
| 2366 | GOBP\_GLUCOSYLCERAMIDE\_METABOLIC\_PROCESS |  | 5 | -0.65 | -1.25 | 0.173 | 0.486 | 1.000 | 2915 | tags=60%, list=24%, signal=79% |
| 2367 | GOCC\_PSEUDOPODIUM |  | 11 | -0.53 | -1.25 | 0.180 | 0.487 | 1.000 | 2480 | tags=27%, list=21%, signal=34% |
| 2368 | GOBP\_DETECTION\_OF\_MECHANICAL\_STIMULUS\_INVOLVED\_IN\_SENSORY\_PERCEPTION |  | 16 | -0.48 | -1.25 | 0.170 | 0.487 | 1.000 | 1741 | tags=25%, list=14%, signal=29% |
| 2369 | GOBP\_LEUKEMIA\_INHIBITORY\_FACTOR\_SIGNALING\_PATHWAY |  | 7 | -0.59 | -1.25 | 0.179 | 0.487 | 1.000 | 4205 | tags=57%, list=35%, signal=88% |
| 2370 | GOMF\_LIGAND\_GATED\_ION\_CHANNEL\_ACTIVITY |  | 59 | -0.37 | -1.25 | 0.137 | 0.487 | 1.000 | 2038 | tags=29%, list=17%, signal=35% |
| 2371 | GOBP\_NEGATIVE\_REGULATION\_OF\_P38MAPK\_CASCADE |  | 6 | -0.62 | -1.25 | 0.171 | 0.487 | 1.000 | 2604 | tags=50%, list=22%, signal=64% |
| 2372 | GOBP\_CELL\_MIGRATION\_IN\_HINDBRAIN |  | 8 | -0.57 | -1.25 | 0.189 | 0.487 | 1.000 | 3889 | tags=63%, list=32%, signal=92% |
| 2373 | GOMF\_NUCLEOSOME\_BINDING |  | 42 | -0.39 | -1.25 | 0.150 | 0.487 | 1.000 | 5049 | tags=50%, list=42%, signal=86% |
| 2374 | GOBP\_NEGATIVE\_REGULATION\_OF\_PHOSPHORYLATION |  | 279 | -0.32 | -1.25 | 0.030 | 0.487 | 1.000 | 4008 | tags=39%, list=33%, signal=58% |
| 2375 | GOBP\_GLYCOLIPID\_CATABOLIC\_PROCESS |  | 10 | -0.53 | -1.25 | 0.175 | 0.487 | 1.000 | 4037 | tags=70%, list=34%, signal=105% |
| 2376 | GOBP\_ORGANELLE\_FISSION |  | 344 | -0.32 | -1.25 | 0.021 | 0.488 | 1.000 | 4529 | tags=43%, list=38%, signal=67% |
| 2377 | GOBP\_IONOTROPIC\_GLUTAMATE\_RECEPTOR\_SIGNALING\_PATHWAY |  | 13 | -0.50 | -1.25 | 0.186 | 0.488 | 1.000 | 4846 | tags=62%, list=40%, signal=103% |
| 2378 | GOBP\_POSITIVE\_REGULATION\_OF\_DEVELOPMENTAL\_PIGMENTATION |  | 7 | -0.60 | -1.25 | 0.180 | 0.488 | 1.000 | 4116 | tags=86%, list=34%, signal=130% |
| 2379 | GOCC\_ATP\_BINDING\_CASSETTE\_ABC\_TRANSPORTER\_COMPLEX |  | 6 | -0.62 | -1.25 | 0.177 | 0.488 | 1.000 | 1109 | tags=50%, list=9%, signal=55% |
| 2380 | GOBP\_POLYSACCHARIDE\_METABOLIC\_PROCESS |  | 78 | -0.36 | -1.25 | 0.114 | 0.488 | 1.000 | 4205 | tags=47%, list=35%, signal=73% |
| 2381 | GOBP\_NEGATIVE\_REGULATION\_OF\_CHEMOKINE\_MEDIATED\_SIGNALING\_PATHWAY |  | 5 | -0.65 | -1.25 | 0.175 | 0.488 | 1.000 | 1038 | tags=40%, list=9%, signal=44% |
| 2382 | GOMF\_PROTEIN\_GLUTAMINE\_GAMMA\_GLUTAMYLTRANSFERASE\_ACTIVITY |  | 3 | -0.75 | -1.25 | 0.154 | 0.488 | 1.000 | 81 | tags=33%, list=1%, signal=34% |
| 2383 | GOBP\_LYMPHOCYTE\_CHEMOTAXIS |  | 27 | -0.42 | -1.25 | 0.167 | 0.488 | 1.000 | 1845 | tags=37%, list=15%, signal=44% |
| 2384 | GOMF\_ACID\_THIOL\_LIGASE\_ACTIVITY |  | 20 | -0.45 | -1.25 | 0.171 | 0.488 | 1.000 | 4053 | tags=45%, list=34%, signal=68% |
| 2385 | GOBP\_DNA\_CATABOLIC\_PROCESS\_ENDONUCLEOLYTIC |  | 18 | -0.46 | -1.25 | 0.177 | 0.488 | 1.000 | 5564 | tags=72%, list=46%, signal=134% |
| 2386 | GOBP\_POSITIVE\_REGULATION\_OF\_MYELOID\_LEUKOCYTE\_MEDIATED\_IMMUNITY |  | 10 | -0.53 | -1.25 | 0.191 | 0.488 | 1.000 | 4686 | tags=60%, list=39%, signal=98% |
| 2387 | GOBP\_REGULATION\_OF\_TOLERANCE\_INDUCTION |  | 7 | -0.58 | -1.25 | 0.178 | 0.487 | 1.000 | 1238 | tags=43%, list=10%, signal=48% |
| 2388 | GOBP\_INTRACELLULAR\_STEROID\_HORMONE\_RECEPTOR\_SIGNALING\_PATHWAY |  | 80 | -0.35 | -1.25 | 0.116 | 0.487 | 1.000 | 4512 | tags=52%, list=38%, signal=84% |
| 2389 | GOBP\_RESPONSE\_TO\_NUTRIENT |  | 121 | -0.34 | -1.25 | 0.091 | 0.487 | 1.000 | 3392 | tags=37%, list=28%, signal=51% |
| 2390 | GOMF\_PROTEIN\_TYROSINE\_PHOSPHATASE\_ACTIVITY |  | 79 | -0.36 | -1.25 | 0.119 | 0.487 | 1.000 | 3392 | tags=38%, list=28%, signal=53% |
| 2391 | GOBP\_AXON\_CHOICE\_POINT\_RECOGNITION |  | 4 | -0.69 | -1.25 | 0.164 | 0.487 | 1.000 | 2300 | tags=50%, list=19%, signal=62% |
| 2392 | GOBP\_LENS\_FIBER\_CELL\_DIFFERENTIATION |  | 20 | -0.45 | -1.25 | 0.189 | 0.487 | 1.000 | 2002 | tags=40%, list=17%, signal=48% |
| 2393 | GOBP\_POSITIVE\_REGULATION\_OF\_B\_CELL\_PROLIFERATION |  | 29 | -0.42 | -1.25 | 0.171 | 0.487 | 1.000 | 2474 | tags=38%, list=21%, signal=48% |
| 2394 | GOBP\_NEGATIVE\_REGULATION\_OF\_REGULATED\_SECRETORY\_PATHWAY |  | 14 | -0.48 | -1.25 | 0.183 | 0.488 | 1.000 | 1608 | tags=21%, list=13%, signal=25% |
| 2395 | GOBP\_LIPID\_MODIFICATION |  | 201 | -0.33 | -1.25 | 0.051 | 0.488 | 1.000 | 3934 | tags=41%, list=33%, signal=60% |
| 2396 | GOBP\_REGULATION\_OF\_MACROPHAGE\_CHEMOTAXIS |  | 20 | -0.45 | -1.25 | 0.183 | 0.488 | 1.000 | 3093 | tags=50%, list=26%, signal=67% |
| 2397 | GOBP\_REGULATION\_OF\_DEACETYLASE\_ACTIVITY |  | 7 | -0.59 | -1.25 | 0.183 | 0.488 | 1.000 | 3483 | tags=71%, list=29%, signal=101% |
| 2398 | GOBP\_REGULATION\_OF\_INFLAMMATORY\_RESPONSE |  | 204 | -0.33 | -1.25 | 0.052 | 0.488 | 1.000 | 2920 | tags=33%, list=24%, signal=43% |
| 2399 | GOBP\_FIBROBLAST\_MIGRATION |  | 36 | -0.40 | -1.25 | 0.152 | 0.488 | 1.000 | 3613 | tags=39%, list=30%, signal=55% |
| 2400 | GOBP\_POST\_GOLGI\_VESICLE\_MEDIATED\_TRANSPORT |  | 86 | -0.35 | -1.25 | 0.112 | 0.488 | 1.000 | 3426 | tags=40%, list=29%, signal=55% |
| 2401 | GOBP\_POSITIVE\_REGULATION\_OF\_HORMONE\_SECRETION |  | 76 | -0.36 | -1.25 | 0.123 | 0.488 | 1.000 | 3269 | tags=37%, list=27%, signal=50% |
| 2402 | GOBP\_NEGATIVE\_REGULATION\_OF\_RESPONSE\_TO\_DNA\_DAMAGE\_STIMULUS |  | 53 | -0.38 | -1.25 | 0.132 | 0.488 | 1.000 | 2379 | tags=32%, list=20%, signal=40% |
| 2403 | GOBP\_MICROTUBULE\_ANCHORING\_AT\_CENTROSOME |  | 8 | -0.57 | -1.25 | 0.181 | 0.488 | 1.000 | 2299 | tags=50%, list=19%, signal=62% |
| 2404 | GOBP\_MAINTENANCE\_OF\_CELL\_NUMBER |  | 108 | -0.34 | -1.25 | 0.102 | 0.488 | 1.000 | 4337 | tags=43%, list=36%, signal=66% |
| 2405 | GOCC\_PRESYNAPTIC\_MEMBRANE |  | 85 | -0.35 | -1.25 | 0.109 | 0.489 | 1.000 | 2876 | tags=32%, list=24%, signal=41% |
| 2406 | GOBP\_ESTABLISHMENT\_OF\_ORGANELLE\_LOCALIZATION |  | 338 | -0.32 | -1.25 | 0.024 | 0.488 | 1.000 | 3577 | tags=34%, list=30%, signal=47% |
| 2407 | GOBP\_NEURON\_DEATH |  | 215 | -0.33 | -1.25 | 0.048 | 0.488 | 1.000 | 3165 | tags=31%, list=26%, signal=42% |
| 2408 | GOBP\_NEGATIVE\_REGULATION\_OF\_CELLULAR\_SENESCENCE |  | 16 | -0.48 | -1.25 | 0.187 | 0.488 | 1.000 | 5835 | tags=81%, list=49%, signal=158% |
| 2409 | GOBP\_SYNAPTIC\_TRANSMISSION\_GLUTAMATERGIC |  | 46 | -0.38 | -1.25 | 0.147 | 0.488 | 1.000 | 3708 | tags=48%, list=31%, signal=69% |
| 2410 | GOBP\_REGULATION\_OF\_PROTEIN\_TARGETING\_TO\_MEMBRANE |  | 24 | -0.43 | -1.25 | 0.172 | 0.488 | 1.000 | 1222 | tags=21%, list=10%, signal=23% |
| 2411 | GOBP\_5S\_CLASS\_RRNA\_TRANSCRIPTION\_BY\_RNA\_POLYMERASE\_III |  | 5 | -0.65 | -1.25 | 0.175 | 0.488 | 1.000 | 3625 | tags=60%, list=30%, signal=86% |
| 2412 | GOBP\_NEGATIVE\_REGULATION\_OF\_T\_CELL\_MEDIATED\_CYTOTOXICITY |  | 3 | -0.75 | -1.25 | 0.161 | 0.488 | 1.000 | 3031 | tags=100%, list=25%, signal=134% |
| 2413 | GOBP\_REPLICATIVE\_SENESCENCE |  | 12 | -0.51 | -1.25 | 0.197 | 0.488 | 1.000 | 2888 | tags=50%, list=24%, signal=66% |
| 2414 | GOBP\_PROTEIN\_LOCALIZATION\_TO\_CELL\_CELL\_JUNCTION |  | 17 | -0.47 | -1.25 | 0.185 | 0.488 | 1.000 | 5178 | tags=76%, list=43%, signal=134% |
| 2415 | GOMF\_PROTEIN\_C\_TERMINUS\_BINDING |  | 145 | -0.33 | -1.25 | 0.081 | 0.488 | 1.000 | 3668 | tags=37%, list=31%, signal=53% |
| 2416 | GOBP\_REGULATION\_OF\_DEFENSE\_RESPONSE |  | 389 | -0.32 | -1.25 | 0.017 | 0.488 | 1.000 | 3165 | tags=34%, list=26%, signal=45% |
| 2417 | GOCC\_CYTOPLASMIC\_MICROTUBULE |  | 55 | -0.38 | -1.25 | 0.137 | 0.489 | 1.000 | 3551 | tags=36%, list=30%, signal=51% |
| 2418 | GOMF\_LIPOPEPTIDE\_BINDING |  | 4 | -0.69 | -1.25 | 0.165 | 0.489 | 1.000 | 222 | tags=25%, list=2%, signal=25% |
| 2419 | GOBP\_NEGATIVE\_REGULATION\_OF\_CELL\_KILLING |  | 6 | -0.61 | -1.25 | 0.179 | 0.489 | 1.000 | 3031 | tags=50%, list=25%, signal=67% |
| 2420 | GOBP\_DENDRITIC\_CELL\_APOPTOTIC\_PROCESS |  | 8 | -0.57 | -1.25 | 0.178 | 0.490 | 1.000 | 1903 | tags=38%, list=16%, signal=45% |
| 2421 | GOBP\_RESPONSE\_TO\_INTERLEUKIN\_3 |  | 3 | -0.75 | -1.25 | 0.161 | 0.490 | 1.000 | 2225 | tags=67%, list=19%, signal=82% |
| 2422 | GOBP\_VISUAL\_BEHAVIOR |  | 30 | -0.41 | -1.25 | 0.163 | 0.490 | 1.000 | 1743 | tags=27%, list=15%, signal=31% |
| 2423 | GOBP\_CYTOKINE\_PRODUCTION\_INVOLVED\_IN\_IMMUNE\_RESPONSE |  | 56 | -0.37 | -1.25 | 0.139 | 0.490 | 1.000 | 2660 | tags=41%, list=22%, signal=53% |
| 2424 | GOBP\_PROTEIN\_LOCALIZATION\_TO\_KINETOCHORE |  | 16 | -0.48 | -1.25 | 0.184 | 0.490 | 1.000 | 5627 | tags=75%, list=47%, signal=141% |
| 2425 | GOBP\_NEGATIVE\_REGULATION\_OF\_NEURON\_DEATH |  | 117 | -0.34 | -1.25 | 0.097 | 0.490 | 1.000 | 3733 | tags=36%, list=31%, signal=52% |
| 2426 | GOCC\_INTRACELLULAR\_PROTEIN\_CONTAINING\_COMPLEX |  | 576 | -0.32 | -1.25 | 0.004 | 0.490 | 1.000 | 4525 | tags=41%, list=38%, signal=63% |
| 2427 | GOMF\_H3\_HISTONE\_ACETYLTRANSFERASE\_ACTIVITY |  | 7 | -0.59 | -1.25 | 0.190 | 0.490 | 1.000 | 881 | tags=29%, list=7%, signal=31% |
| 2428 | GOBP\_NEGATIVE\_REGULATION\_OF\_KERATINOCYTE\_PROLIFERATION |  | 9 | -0.55 | -1.25 | 0.191 | 0.490 | 1.000 | 4431 | tags=67%, list=37%, signal=106% |
| 2429 | GOBP\_HISTONE\_H3\_K9\_METHYLATION |  | 24 | -0.43 | -1.25 | 0.178 | 0.490 | 1.000 | 3644 | tags=54%, list=30%, signal=78% |
| 2430 | GOCC\_CLATHRIN\_ADAPTOR\_COMPLEX |  | 15 | -0.48 | -1.25 | 0.194 | 0.490 | 1.000 | 4775 | tags=67%, list=40%, signal=111% |
| 2431 | GOBP\_GLIAL\_CELL\_DEVELOPMENT |  | 76 | -0.36 | -1.25 | 0.132 | 0.490 | 1.000 | 4548 | tags=45%, list=38%, signal=72% |
| 2432 | GOBP\_RESPONSE\_TO\_LIPOTEICHOIC\_ACID |  | 9 | -0.55 | -1.25 | 0.194 | 0.490 | 1.000 | 1236 | tags=33%, list=10%, signal=37% |
| 2433 | GOBP\_RESPONSE\_TO\_MURAMYL\_DIPEPTIDE |  | 15 | -0.48 | -1.25 | 0.193 | 0.490 | 1.000 | 2256 | tags=27%, list=19%, signal=33% |
| 2434 | GOMF\_ACID\_PHOSPHATASE\_ACTIVITY |  | 5 | -0.65 | -1.25 | 0.173 | 0.490 | 1.000 | 773 | tags=40%, list=6%, signal=43% |
| 2435 | GOBP\_NEGATIVE\_REGULATION\_OF\_EPITHELIAL\_CELL\_APOPTOTIC\_PROCESS |  | 32 | -0.41 | -1.25 | 0.173 | 0.490 | 1.000 | 4211 | tags=50%, list=35%, signal=77% |
| 2436 | GOMF\_SPHINGOLIPID\_BINDING |  | 17 | -0.47 | -1.25 | 0.189 | 0.490 | 1.000 | 1952 | tags=35%, list=16%, signal=42% |
| 2437 | GOMF\_G\_PROTEIN\_COUPLED\_ADENOSINE\_RECEPTOR\_ACTIVITY |  | 3 | -0.74 | -1.25 | 0.159 | 0.491 | 1.000 | 1796 | tags=67%, list=15%, signal=78% |
| 2438 | GOCC\_FILOPODIUM\_MEMBRANE |  | 14 | -0.49 | -1.25 | 0.191 | 0.491 | 1.000 | 3141 | tags=43%, list=26%, signal=58% |
| 2439 | GOBP\_REGULATION\_OF\_INORGANIC\_ANION\_TRANSMEMBRANE\_TRANSPORT |  | 4 | -0.69 | -1.25 | 0.170 | 0.491 | 1.000 | 2038 | tags=50%, list=17%, signal=60% |
| 2440 | GOCC\_NBAF\_COMPLEX |  | 11 | -0.52 | -1.25 | 0.195 | 0.491 | 1.000 | 3521 | tags=45%, list=29%, signal=64% |
| 2441 | GOBP\_POSITIVE\_REGULATION\_OF\_CELL\_DEATH |  | 427 | -0.32 | -1.25 | 0.017 | 0.492 | 1.000 | 2880 | tags=29%, list=24%, signal=37% |
| 2442 | GOBP\_MAINTENANCE\_OF\_LOCATION\_IN\_CELL |  | 139 | -0.34 | -1.25 | 0.085 | 0.492 | 1.000 | 4240 | tags=42%, list=35%, signal=64% |
| 2443 | GOBP\_NEGATIVE\_REGULATION\_OF\_CATION\_CHANNEL\_ACTIVITY |  | 27 | -0.42 | -1.25 | 0.171 | 0.492 | 1.000 | 1455 | tags=19%, list=12%, signal=21% |
| 2444 | GOMF\_CAMP\_DEPENDENT\_PROTEIN\_KINASE\_ACTIVITY |  | 5 | -0.65 | -1.25 | 0.179 | 0.492 | 1.000 | 3920 | tags=80%, list=33%, signal=119% |
| 2445 | GOMF\_DNA\_POLYMERASE\_BINDING |  | 17 | -0.46 | -1.25 | 0.194 | 0.492 | 1.000 | 4793 | tags=53%, list=40%, signal=88% |
| 2446 | GOBP\_REGULATION\_OF\_VITAMIN\_METABOLIC\_PROCESS |  | 6 | -0.61 | -1.25 | 0.184 | 0.492 | 1.000 | 2666 | tags=50%, list=22%, signal=64% |
| 2447 | GOBP\_TRANSMEMBRANE\_RECEPTOR\_PROTEIN\_TYROSINE\_KINASE\_SIGNALING\_PATHWAY |  | 546 | -0.32 | -1.25 | 0.008 | 0.492 | 1.000 | 3151 | tags=32%, list=26%, signal=41% |
| 2448 | GOBP\_NEGATIVE\_REGULATION\_OF\_MYOTUBE\_DIFFERENTIATION |  | 8 | -0.56 | -1.25 | 0.202 | 0.493 | 1.000 | 4940 | tags=63%, list=41%, signal=106% |
| 2449 | GOBP\_REPLICATION\_BORN\_DOUBLE\_STRAND\_BREAK\_REPAIR\_VIA\_SISTER\_CHROMATID\_EXCHANGE |  | 4 | -0.69 | -1.25 | 0.171 | 0.493 | 1.000 | 3778 | tags=100%, list=31%, signal=146% |
| 2450 | GOBP\_REGULATION\_OF\_CELL\_SUBSTRATE\_JUNCTION\_ORGANIZATION |  | 61 | -0.37 | -1.24 | 0.130 | 0.495 | 1.000 | 3575 | tags=44%, list=30%, signal=63% |
| 2451 | GOMF\_STRUCTURAL\_CONSTITUENT\_OF\_EYE\_LENS |  | 3 | -0.75 | -1.24 | 0.169 | 0.495 | 1.000 | 1325 | tags=33%, list=11%, signal=37% |
| 2452 | GOMF\_G\_PROTEIN\_COUPLED\_RECEPTOR\_ACTIVITY |  | 196 | -0.33 | -1.24 | 0.062 | 0.495 | 1.000 | 3553 | tags=39%, list=30%, signal=54% |
| 2453 | GOMF\_ORGANIC\_HYDROXY\_COMPOUND\_TRANSMEMBRANE\_TRANSPORTER\_ACTIVITY |  | 26 | -0.42 | -1.24 | 0.170 | 0.495 | 1.000 | 2392 | tags=31%, list=20%, signal=38% |
| 2454 | GOBP\_CIRCADIAN\_RHYTHM |  | 146 | -0.33 | -1.24 | 0.086 | 0.495 | 1.000 | 2907 | tags=33%, list=24%, signal=43% |
| 2455 | GOBP\_CHORION\_DEVELOPMENT |  | 4 | -0.69 | -1.24 | 0.170 | 0.495 | 1.000 | 2639 | tags=75%, list=22%, signal=96% |
| 2456 | GOMF\_IMMUNOGLOBULIN\_RECEPTOR\_ACTIVITY |  | 3 | -0.75 | -1.24 | 0.161 | 0.495 | 1.000 | 2836 | tags=67%, list=24%, signal=87% |
| 2457 | GOCC\_PHOTORECEPTOR\_OUTER\_SEGMENT\_MEMBRANE |  | 6 | -0.61 | -1.24 | 0.184 | 0.495 | 1.000 | 1193 | tags=33%, list=10%, signal=37% |
| 2458 | GOBP\_CELLULAR\_RESPONSE\_TO\_UV\_A |  | 5 | -0.65 | -1.24 | 0.178 | 0.495 | 1.000 | 1455 | tags=40%, list=12%, signal=45% |
| 2459 | GOBP\_HISTONE\_UBIQUITINATION |  | 40 | -0.39 | -1.24 | 0.158 | 0.495 | 1.000 | 4687 | tags=55%, list=39%, signal=90% |
| 2460 | GOCC\_LAMELLIPODIUM |  | 160 | -0.33 | -1.24 | 0.078 | 0.495 | 1.000 | 2883 | tags=31%, list=24%, signal=40% |
| 2461 | GOBP\_TELOMERIC\_D\_LOOP\_DISASSEMBLY |  | 6 | -0.62 | -1.24 | 0.185 | 0.495 | 1.000 | 3722 | tags=83%, list=31%, signal=121% |
| 2462 | GOBP\_NEGATIVE\_REGULATION\_OF\_PROTEIN\_SERINE\_THREONINE\_KINASE\_ACTIVITY |  | 97 | -0.35 | -1.24 | 0.108 | 0.495 | 1.000 | 3687 | tags=40%, list=31%, signal=58% |
| 2463 | GOCC\_SCF\_UBIQUITIN\_LIGASE\_COMPLEX |  | 47 | -0.38 | -1.24 | 0.150 | 0.495 | 1.000 | 4754 | tags=49%, list=40%, signal=81% |
| 2464 | GOBP\_REGULATION\_OF\_CHOLESTEROL\_EFFLUX |  | 20 | -0.45 | -1.24 | 0.192 | 0.495 | 1.000 | 3565 | tags=45%, list=30%, signal=64% |
| 2465 | GOBP\_HISTONE\_SERINE\_PHOSPHORYLATION |  | 8 | -0.56 | -1.24 | 0.193 | 0.495 | 1.000 | 4528 | tags=75%, list=38%, signal=120% |
| 2466 | GOBP\_POSITIVE\_REGULATION\_OF\_COLLATERAL\_SPROUTING |  | 5 | -0.64 | -1.24 | 0.183 | 0.495 | 1.000 | 488 | tags=40%, list=4%, signal=42% |
| 2467 | GOBP\_POSTSYNAPTIC\_MEMBRANE\_ASSEMBLY |  | 6 | -0.61 | -1.24 | 0.181 | 0.495 | 1.000 | 3431 | tags=67%, list=29%, signal=93% |
| 2468 | GOBP\_CHOLINE\_TRANSPORT |  | 3 | -0.74 | -1.24 | 0.172 | 0.495 | 1.000 | 2119 | tags=67%, list=18%, signal=81% |
| 2469 | GOBP\_POSITIVE\_REGULATION\_OF\_SPROUTING\_ANGIOGENESIS |  | 11 | -0.51 | -1.24 | 0.201 | 0.496 | 1.000 | 3905 | tags=64%, list=33%, signal=94% |
| 2470 | GOCC\_NUCLEAR\_REPLICATION\_FORK |  | 27 | -0.42 | -1.24 | 0.173 | 0.496 | 1.000 | 3744 | tags=44%, list=31%, signal=64% |
| 2471 | GOMF\_TRANSFERASE\_ACTIVITY\_TRANSFERRING\_PENTOSYL\_GROUPS |  | 35 | -0.40 | -1.24 | 0.170 | 0.496 | 1.000 | 1704 | tags=23%, list=14%, signal=27% |
| 2472 | GOBP\_MITOTIC\_NUCLEAR\_DIVISION |  | 234 | -0.32 | -1.24 | 0.046 | 0.497 | 1.000 | 4529 | tags=42%, list=38%, signal=66% |
| 2473 | GOBP\_NEGATIVE\_REGULATION\_OF\_SMOOTH\_MUSCLE\_CELL\_APOPTOTIC\_PROCESS |  | 5 | -0.64 | -1.24 | 0.184 | 0.497 | 1.000 | 405 | tags=40%, list=3%, signal=41% |
| 2474 | GOBP\_RESPONSE\_TO\_EXTRACELLULAR\_STIMULUS |  | 329 | -0.32 | -1.24 | 0.027 | 0.497 | 1.000 | 3701 | tags=36%, list=31%, signal=51% |
| 2475 | GOBP\_CELLULAR\_RESPONSE\_TO\_GROWTH\_HORMONE\_STIMULUS |  | 15 | -0.48 | -1.24 | 0.194 | 0.497 | 1.000 | 960 | tags=33%, list=8%, signal=36% |
| 2476 | GOBP\_TOXIN\_TRANSPORT |  | 33 | -0.41 | -1.24 | 0.167 | 0.497 | 1.000 | 6086 | tags=64%, list=51%, signal=129% |
| 2477 | GOBP\_POSITIVE\_REGULATION\_OF\_DNA\_DEPENDENT\_DNA\_REPLICATION |  | 9 | -0.55 | -1.24 | 0.201 | 0.497 | 1.000 | 434 | tags=22%, list=4%, signal=23% |
| 2478 | GOBP\_CELL\_CYCLE\_DNA\_REPLICATION |  | 57 | -0.37 | -1.24 | 0.142 | 0.497 | 1.000 | 5533 | tags=61%, list=46%, signal=113% |
| 2479 | GOBP\_RESPONSE\_TO\_CAMPTOTHECIN |  | 4 | -0.69 | -1.24 | 0.182 | 0.497 | 1.000 | 3778 | tags=100%, list=31%, signal=146% |
| 2480 | GOBP\_REGULATION\_OF\_HORMONE\_SECRETION |  | 166 | -0.33 | -1.24 | 0.076 | 0.497 | 1.000 | 2367 | tags=27%, list=20%, signal=33% |
| 2481 | GOBP\_POSITIVE\_REGULATION\_OF\_IMMUNE\_EFFECTOR\_PROCESS |  | 123 | -0.34 | -1.24 | 0.098 | 0.497 | 1.000 | 3487 | tags=40%, list=29%, signal=56% |
| 2482 | GOBP\_RESPONSE\_TO\_RADIATION |  | 295 | -0.32 | -1.24 | 0.032 | 0.497 | 1.000 | 3961 | tags=39%, list=33%, signal=57% |
| 2483 | GOBP\_REGULATION\_OF\_CELLULAR\_RESPONSE\_TO\_HEAT |  | 65 | -0.36 | -1.24 | 0.144 | 0.497 | 1.000 | 4282 | tags=46%, list=36%, signal=71% |
| 2484 | GOBP\_REGULATION\_OF\_TRANSCRIPTION\_INITIATION\_FROM\_RNA\_POLYMERASE\_II\_PROMOTER |  | 29 | -0.41 | -1.24 | 0.171 | 0.497 | 1.000 | 4896 | tags=59%, list=41%, signal=99% |
| 2485 | GOBP\_REGULATION\_OF\_NEURON\_MATURATION |  | 9 | -0.55 | -1.24 | 0.195 | 0.497 | 1.000 | 3483 | tags=67%, list=29%, signal=94% |
| 2486 | GOCC\_CELL\_PROJECTION\_MEMBRANE |  | 212 | -0.33 | -1.24 | 0.060 | 0.497 | 1.000 | 2757 | tags=31%, list=23%, signal=40% |
| 2487 | GOBP\_REGULATION\_OF\_HISTONE\_DEACETYLATION |  | 15 | -0.47 | -1.24 | 0.186 | 0.497 | 1.000 | 3640 | tags=53%, list=30%, signal=76% |
| 2488 | GOBP\_REGULATION\_OF\_SECRETION |  | 388 | -0.32 | -1.24 | 0.021 | 0.497 | 1.000 | 3050 | tags=31%, list=25%, signal=41% |
| 2489 | GOBP\_AMINO\_ACID\_TRANSPORT |  | 98 | -0.34 | -1.24 | 0.111 | 0.497 | 1.000 | 2313 | tags=31%, list=19%, signal=38% |
| 2490 | GOBP\_INSULIN\_SECRETION |  | 140 | -0.33 | -1.24 | 0.089 | 0.497 | 1.000 | 1771 | tags=24%, list=15%, signal=28% |
| 2491 | GOBP\_CORTICOSPINAL\_TRACT\_MORPHOGENESIS |  | 5 | -0.64 | -1.24 | 0.187 | 0.497 | 1.000 | 1081 | tags=20%, list=9%, signal=22% |
| 2492 | GOBP\_AUTOPHAGOSOME\_MATURATION |  | 33 | -0.41 | -1.24 | 0.174 | 0.497 | 1.000 | 4268 | tags=36%, list=36%, signal=56% |
| 2493 | GOBP\_NEGATIVE\_REGULATION\_OF\_TOLL\_LIKE\_RECEPTOR\_SIGNALING\_PATHWAY |  | 21 | -0.44 | -1.24 | 0.186 | 0.497 | 1.000 | 2480 | tags=43%, list=21%, signal=54% |
| 2494 | GOBP\_POSITIVE\_REGULATION\_OF\_CARBOHYDRATE\_METABOLIC\_PROCESS |  | 52 | -0.37 | -1.24 | 0.145 | 0.497 | 1.000 | 4801 | tags=52%, list=40%, signal=86% |
| 2495 | GOBP\_REGULATION\_OF\_PROTEIN\_LOCALIZATION\_TO\_CILIUM |  | 6 | -0.61 | -1.24 | 0.185 | 0.497 | 1.000 | 1695 | tags=50%, list=14%, signal=58% |
| 2496 | GOBP\_ACTIN\_FILAMENT\_BUNDLE\_ORGANIZATION |  | 121 | -0.34 | -1.24 | 0.091 | 0.497 | 1.000 | 2598 | tags=30%, list=22%, signal=38% |
| 2497 | GOMF\_SELENOCYSTEINE\_INSERTION\_SEQUENCE\_BINDING |  | 4 | -0.69 | -1.24 | 0.181 | 0.497 | 1.000 | 483 | tags=25%, list=4%, signal=26% |
| 2498 | GOBP\_CELLULAR\_RESPONSE\_TO\_LITHIUM\_ION |  | 9 | -0.55 | -1.24 | 0.200 | 0.497 | 1.000 | 3632 | tags=56%, list=30%, signal=80% |
| 2499 | GOBP\_TRANSMEMBRANE\_RECEPTOR\_PROTEIN\_SERINE\_THREONINE\_KINASE\_SIGNALING\_PATHWAY |  | 240 | -0.32 | -1.24 | 0.047 | 0.498 | 1.000 | 3570 | tags=39%, list=30%, signal=54% |
| 2500 | GOBP\_REGULATION\_OF\_LAMELLIPODIUM\_ASSEMBLY |  | 29 | -0.41 | -1.24 | 0.179 | 0.498 | 1.000 | 4108 | tags=48%, list=34%, signal=73% |
| 2501 | GOBP\_MORPHOGENESIS\_OF\_A\_BRANCHING\_STRUCTURE |  | 132 | -0.33 | -1.24 | 0.093 | 0.497 | 1.000 | 4341 | tags=45%, list=36%, signal=69% |
| 2502 | GOCC\_INTERCALATED\_DISC |  | 33 | -0.40 | -1.24 | 0.172 | 0.497 | 1.000 | 4110 | tags=52%, list=34%, signal=78% |
| 2503 | GOBP\_CARNITINE\_SHUTTLE |  | 9 | -0.55 | -1.24 | 0.202 | 0.498 | 1.000 | 2469 | tags=44%, list=21%, signal=56% |
| 2504 | GOBP\_FRUCTOSE\_METABOLIC\_PROCESS |  | 12 | -0.50 | -1.24 | 0.198 | 0.498 | 1.000 | 3238 | tags=50%, list=27%, signal=68% |
| 2505 | GOBP\_MYOBLAST\_FATE\_COMMITMENT |  | 4 | -0.68 | -1.24 | 0.178 | 0.498 | 1.000 | 2411 | tags=75%, list=20%, signal=94% |
| 2506 | GOBP\_DE\_NOVO\_NAD\_BIOSYNTHETIC\_PROCESS |  | 4 | -0.68 | -1.24 | 0.182 | 0.498 | 1.000 | 2220 | tags=50%, list=18%, signal=61% |
| 2507 | GOBP\_POSITIVE\_REGULATION\_OF\_CHOLESTEROL\_BIOSYNTHETIC\_PROCESS |  | 5 | -0.64 | -1.24 | 0.187 | 0.498 | 1.000 | 685 | tags=40%, list=6%, signal=42% |
| 2508 | GOBP\_BONE\_MATURATION |  | 14 | -0.48 | -1.24 | 0.199 | 0.498 | 1.000 | 2933 | tags=57%, list=24%, signal=76% |
| 2509 | GOBP\_LOCOMOTORY\_BEHAVIOR |  | 97 | -0.35 | -1.24 | 0.117 | 0.498 | 1.000 | 3557 | tags=40%, list=30%, signal=57% |
| 2510 | GOBP\_GANGLIOSIDE\_CATABOLIC\_PROCESS |  | 4 | -0.68 | -1.24 | 0.190 | 0.497 | 1.000 | 3178 | tags=75%, list=26%, signal=102% |
| 2511 | GOBP\_POSITIVE\_REGULATION\_OF\_HEPATOCYTE\_PROLIFERATION |  | 7 | -0.58 | -1.24 | 0.195 | 0.497 | 1.000 | 3615 | tags=86%, list=30%, signal=123% |
| 2512 | GOMF\_STEROL\_BINDING |  | 47 | -0.38 | -1.24 | 0.154 | 0.498 | 1.000 | 3157 | tags=34%, list=26%, signal=46% |
| 2513 | GOCC\_PHAGOPHORE\_ASSEMBLY\_SITE |  | 25 | -0.43 | -1.24 | 0.175 | 0.498 | 1.000 | 3675 | tags=56%, list=31%, signal=81% |
| 2514 | GOBP\_ORGANELLE\_LOCALIZATION |  | 516 | -0.32 | -1.24 | 0.009 | 0.498 | 1.000 | 3591 | tags=34%, list=30%, signal=46% |
| 2515 | GOBP\_NEGATIVE\_REGULATION\_OF\_FAT\_CELL\_DIFFERENTIATION |  | 35 | -0.40 | -1.24 | 0.173 | 0.498 | 1.000 | 3962 | tags=49%, list=33%, signal=72% |
| 2516 | GOBP\_HISTONE\_H3\_K9\_MODIFICATION |  | 31 | -0.41 | -1.24 | 0.174 | 0.498 | 1.000 | 3644 | tags=52%, list=30%, signal=74% |
| 2517 | GOBP\_SKELETAL\_MUSCLE\_CONTRACTION |  | 15 | -0.48 | -1.24 | 0.197 | 0.498 | 1.000 | 2458 | tags=33%, list=20%, signal=42% |
| 2518 | GOBP\_PHOSPHOLIPID\_METABOLIC\_PROCESS |  | 312 | -0.32 | -1.24 | 0.029 | 0.498 | 1.000 | 4540 | tags=46%, list=38%, signal=72% |
| 2519 | GOBP\_CELLULAR\_RESPONSE\_TO\_DNA\_DAMAGE\_STIMULUS |  | 640 | -0.31 | -1.24 | 0.005 | 0.498 | 1.000 | 3969 | tags=39%, list=33%, signal=55% |
| 2520 | GOBP\_EMBRYONIC\_CLEAVAGE |  | 5 | -0.64 | -1.24 | 0.191 | 0.498 | 1.000 | 4249 | tags=80%, list=35%, signal=124% |
| 2521 | GOBP\_CARDIAC\_CONDUCTION\_SYSTEM\_DEVELOPMENT |  | 8 | -0.56 | -1.24 | 0.205 | 0.499 | 1.000 | 3918 | tags=50%, list=33%, signal=74% |
| 2522 | GOBP\_NEUTRAL\_AMINO\_ACID\_TRANSPORT |  | 30 | -0.41 | -1.24 | 0.175 | 0.499 | 1.000 | 3155 | tags=47%, list=26%, signal=63% |
| 2523 | GOBP\_MORPHOGENESIS\_OF\_A\_POLARIZED\_EPITHELIUM |  | 104 | -0.34 | -1.24 | 0.114 | 0.499 | 1.000 | 2174 | tags=20%, list=18%, signal=24% |
| 2524 | GOBP\_POLYSACCHARIDE\_CATABOLIC\_PROCESS |  | 21 | -0.44 | -1.24 | 0.190 | 0.499 | 1.000 | 4815 | tags=67%, list=40%, signal=111% |
| 2525 | GOBP\_POSITIVE\_REGULATION\_OF\_REACTIVE\_OXYGEN\_SPECIES\_METABOLIC\_PROCESS |  | 69 | -0.36 | -1.24 | 0.139 | 0.499 | 1.000 | 2480 | tags=26%, list=21%, signal=33% |
| 2526 | GOBP\_CELLULAR\_SENESCENCE |  | 54 | -0.37 | -1.24 | 0.148 | 0.499 | 1.000 | 3668 | tags=39%, list=31%, signal=56% |
| 2527 | GOBP\_RESPONSE\_TO\_PHENYLPROPANOID |  | 3 | -0.74 | -1.24 | 0.166 | 0.499 | 1.000 | 1535 | tags=67%, list=13%, signal=76% |
| 2528 | GOBP\_RESPONSE\_TO\_UV\_C |  | 9 | -0.55 | -1.24 | 0.195 | 0.499 | 1.000 | 2282 | tags=56%, list=19%, signal=69% |
| 2529 | GOBP\_REGULATION\_OF\_HISTONE\_DEACETYLASE\_ACTIVITY |  | 5 | -0.64 | -1.24 | 0.180 | 0.499 | 1.000 | 3483 | tags=80%, list=29%, signal=113% |
| 2530 | GOCC\_FANCONI\_ANAEMIA\_NUCLEAR\_COMPLEX |  | 12 | -0.50 | -1.24 | 0.192 | 0.500 | 1.000 | 4576 | tags=67%, list=38%, signal=108% |
| 2531 | GOBP\_REGULATION\_OF\_GENE\_EXPRESSION\_BY\_GENETIC\_IMPRINTING |  | 11 | -0.52 | -1.24 | 0.203 | 0.500 | 1.000 | 2877 | tags=45%, list=24%, signal=60% |
| 2532 | GOCC\_ACTIN\_CYTOSKELETON |  | 352 | -0.32 | -1.24 | 0.027 | 0.500 | 1.000 | 2948 | tags=28%, list=25%, signal=36% |
| 2533 | GOBP\_POSITIVE\_REGULATION\_OF\_INSULIN\_LIKE\_GROWTH\_FACTOR\_RECEPTOR\_SIGNALING\_PATHWAY |  | 9 | -0.54 | -1.24 | 0.206 | 0.500 | 1.000 | 2351 | tags=33%, list=20%, signal=41% |
| 2534 | GOBP\_CELLULAR\_RESPONSE\_TO\_AMYLOID\_BETA |  | 24 | -0.43 | -1.24 | 0.182 | 0.500 | 1.000 | 3376 | tags=42%, list=28%, signal=58% |
| 2535 | GOBP\_PHOSPHATIDIC\_ACID\_METABOLIC\_PROCESS |  | 35 | -0.40 | -1.24 | 0.171 | 0.500 | 1.000 | 3033 | tags=31%, list=25%, signal=42% |
| 2536 | GOBP\_VESICLE\_TETHERING\_TO\_GOLGI |  | 4 | -0.68 | -1.24 | 0.175 | 0.500 | 1.000 | 2797 | tags=75%, list=23%, signal=98% |
| 2537 | GOMF\_PROLINE\_RICH\_REGION\_BINDING |  | 15 | -0.48 | -1.24 | 0.191 | 0.500 | 1.000 | 2337 | tags=40%, list=19%, signal=50% |
| 2538 | GOBP\_REGULATION\_OF\_CELL\_DIVISION |  | 117 | -0.34 | -1.24 | 0.106 | 0.500 | 1.000 | 3214 | tags=33%, list=27%, signal=45% |
| 2539 | GOBP\_LYMPHOCYTE\_MIGRATION |  | 62 | -0.36 | -1.24 | 0.140 | 0.500 | 1.000 | 2955 | tags=37%, list=25%, signal=49% |
| 2540 | GOCC\_MEMBRANE\_MICRODOMAIN |  | 230 | -0.32 | -1.24 | 0.055 | 0.500 | 1.000 | 3323 | tags=34%, list=28%, signal=47% |
| 2541 | GOBP\_RESPONSE\_TO\_LIGHT\_STIMULUS |  | 196 | -0.32 | -1.24 | 0.069 | 0.500 | 1.000 | 2849 | tags=29%, list=24%, signal=38% |
| 2542 | GOBP\_POSITIVE\_REGULATION\_OF\_GENE\_EXPRESSION |  | 651 | -0.31 | -1.24 | 0.003 | 0.500 | 1.000 | 3408 | tags=34%, list=28%, signal=45% |
| 2543 | GOBP\_APPENDAGE\_MORPHOGENESIS |  | 83 | -0.35 | -1.24 | 0.129 | 0.500 | 1.000 | 4102 | tags=41%, list=34%, signal=62% |
| 2544 | GOBP\_EMBRYONIC\_HEMOPOIESIS |  | 19 | -0.45 | -1.24 | 0.191 | 0.500 | 1.000 | 3907 | tags=58%, list=33%, signal=86% |
| 2545 | GOBP\_RESPONSE\_TO\_INTERLEUKIN\_6 |  | 30 | -0.41 | -1.24 | 0.187 | 0.501 | 1.000 | 3930 | tags=53%, list=33%, signal=79% |
| 2546 | GOMF\_CARBOHYDRATE\_BINDING |  | 120 | -0.34 | -1.24 | 0.100 | 0.501 | 1.000 | 4342 | tags=46%, list=36%, signal=71% |
| 2547 | GOBP\_POSITIVE\_REGULATION\_OF\_NATURAL\_KILLER\_CELL\_DIFFERENTIATION |  | 5 | -0.64 | -1.24 | 0.190 | 0.501 | 1.000 | 1903 | tags=40%, list=16%, signal=48% |
| 2548 | GOCC\_INTRINSIC\_COMPONENT\_OF\_NUCLEAR\_INNER\_MEMBRANE |  | 13 | -0.49 | -1.24 | 0.204 | 0.501 | 1.000 | 902 | tags=23%, list=8%, signal=25% |
| 2549 | GOBP\_POSTSYNAPTIC\_MEMBRANE\_ORGANIZATION |  | 21 | -0.44 | -1.24 | 0.192 | 0.501 | 1.000 | 3460 | tags=43%, list=29%, signal=60% |
| 2550 | GOBP\_TRYPTOPHAN\_TRANSPORT |  | 3 | -0.74 | -1.24 | 0.180 | 0.502 | 1.000 | 3155 | tags=100%, list=26%, signal=136% |
| 2551 | GOBP\_REGULATION\_OF\_PIGMENT\_CELL\_DIFFERENTIATION |  | 7 | -0.58 | -1.24 | 0.197 | 0.501 | 1.000 | 4277 | tags=86%, list=36%, signal=133% |
| 2552 | GOMF\_PROTEIN\_KINASE\_C\_ACTIVITY |  | 11 | -0.51 | -1.24 | 0.208 | 0.502 | 1.000 | 3308 | tags=45%, list=28%, signal=63% |
| 2553 | GOBP\_CELL\_KILLING |  | 61 | -0.36 | -1.24 | 0.147 | 0.502 | 1.000 | 2547 | tags=31%, list=21%, signal=39% |
| 2554 | GOBP\_PROLINE\_TRANSPORT |  | 6 | -0.61 | -1.24 | 0.194 | 0.501 | 1.000 | 2825 | tags=67%, list=24%, signal=87% |
| 2555 | GOMF\_ENDOPEPTIDASE\_ACTIVITY |  | 234 | -0.32 | -1.24 | 0.056 | 0.501 | 1.000 | 3036 | tags=29%, list=25%, signal=38% |
| 2556 | GOBP\_MOLTING\_CYCLE |  | 65 | -0.36 | -1.24 | 0.144 | 0.502 | 1.000 | 2212 | tags=23%, list=18%, signal=28% |
| 2557 | GOBP\_REGULATION\_OF\_SYSTEM\_PROCESS |  | 345 | -0.32 | -1.24 | 0.030 | 0.502 | 1.000 | 3934 | tags=36%, list=33%, signal=51% |
| 2558 | GOBP\_RESPONSE\_TO\_FORSKOLIN |  | 8 | -0.56 | -1.24 | 0.199 | 0.502 | 1.000 | 2038 | tags=38%, list=17%, signal=45% |
| 2559 | GOMF\_SYMPORTER\_ACTIVITY |  | 90 | -0.35 | -1.23 | 0.124 | 0.503 | 1.000 | 3006 | tags=38%, list=25%, signal=50% |
| 2560 | GOBP\_MESENDODERM\_DEVELOPMENT |  | 4 | -0.69 | -1.23 | 0.178 | 0.503 | 1.000 | 2886 | tags=50%, list=24%, signal=66% |
| 2561 | GOBP\_REGULATION\_OF\_CATION\_CHANNEL\_ACTIVITY |  | 90 | -0.35 | -1.23 | 0.120 | 0.503 | 1.000 | 2908 | tags=29%, list=24%, signal=38% |
| 2562 | GOBP\_REGULATION\_OF\_CELL\_CYCLE\_PHASE\_TRANSITION |  | 350 | -0.32 | -1.23 | 0.029 | 0.504 | 1.000 | 5379 | tags=50%, list=45%, signal=87% |
| 2563 | GOBP\_SIGNAL\_TRANSDUCTION\_IN\_RESPONSE\_TO\_DNA\_DAMAGE |  | 104 | -0.34 | -1.23 | 0.111 | 0.504 | 1.000 | 2695 | tags=29%, list=22%, signal=37% |
| 2564 | GOBP\_HEAD\_DEVELOPMENT |  | 502 | -0.31 | -1.23 | 0.014 | 0.504 | 1.000 | 4152 | tags=41%, list=35%, signal=60% |
| 2565 | GOMF\_RNA\_POLYMERASE\_III\_GENERAL\_TRANSCRIPTION\_INITIATION\_FACTOR\_ACTIVITY |  | 6 | -0.60 | -1.23 | 0.208 | 0.504 | 1.000 | 1248 | tags=33%, list=10%, signal=37% |
| 2566 | GOBP\_BRANCHING\_INVOLVED\_IN\_SALIVARY\_GLAND\_MORPHOGENESIS |  | 18 | -0.46 | -1.23 | 0.201 | 0.504 | 1.000 | 1816 | tags=28%, list=15%, signal=33% |
| 2567 | GOBP\_RAP\_PROTEIN\_SIGNAL\_TRANSDUCTION |  | 13 | -0.49 | -1.23 | 0.201 | 0.504 | 1.000 | 4848 | tags=62%, list=40%, signal=103% |
| 2568 | GOBP\_MIDGUT\_DEVELOPMENT |  | 10 | -0.52 | -1.23 | 0.204 | 0.504 | 1.000 | 2356 | tags=30%, list=20%, signal=37% |
| 2569 | GOBP\_POSITIVE\_REGULATION\_OF\_INFLAMMATORY\_RESPONSE |  | 75 | -0.35 | -1.23 | 0.132 | 0.504 | 1.000 | 2009 | tags=27%, list=17%, signal=32% |
| 2570 | GOCC\_LAMELLIPODIUM\_MEMBRANE |  | 20 | -0.44 | -1.23 | 0.189 | 0.504 | 1.000 | 2757 | tags=35%, list=23%, signal=45% |
| 2571 | GOBP\_CHROMOSOME\_MOVEMENT\_TOWARDS\_SPINDLE\_POLE |  | 6 | -0.61 | -1.23 | 0.196 | 0.504 | 1.000 | 2909 | tags=33%, list=24%, signal=44% |
| 2572 | GOBP\_MODULATION\_BY\_HOST\_OF\_VIRAL\_GENOME\_REPLICATION |  | 14 | -0.48 | -1.23 | 0.201 | 0.504 | 1.000 | 2916 | tags=43%, list=24%, signal=57% |
| 2573 | GOBP\_CHROMATIN\_ASSEMBLY\_OR\_DISASSEMBLY |  | 108 | -0.34 | -1.23 | 0.116 | 0.504 | 1.000 | 3624 | tags=37%, list=30%, signal=53% |
| 2574 | GOBP\_CORTICOSTEROID\_RECEPTOR\_SIGNALING\_PATHWAY |  | 12 | -0.50 | -1.23 | 0.198 | 0.504 | 1.000 | 2367 | tags=50%, list=20%, signal=62% |
| 2575 | GOCC\_REGION\_OF\_CYTOSOL |  | 19 | -0.45 | -1.23 | 0.187 | 0.504 | 1.000 | 3483 | tags=47%, list=29%, signal=67% |
| 2576 | GOBP\_CHROMATIN\_REMODELING\_AT\_CENTROMERE |  | 31 | -0.41 | -1.23 | 0.179 | 0.504 | 1.000 | 5899 | tags=74%, list=49%, signal=145% |
| 2577 | GOBP\_BRAIN\_DERIVED\_NEUROTROPHIC\_FACTOR\_RECEPTOR\_SIGNALING\_PATHWAY |  | 3 | -0.74 | -1.23 | 0.172 | 0.504 | 1.000 | 23 | tags=33%, list=0%, signal=33% |
| 2578 | GOBP\_POSITIVE\_REGULATION\_OF\_TUMOR\_NECROSIS\_FACTOR\_MEDIATED\_SIGNALING\_PATHWAY |  | 6 | -0.60 | -1.23 | 0.201 | 0.504 | 1.000 | 2261 | tags=33%, list=19%, signal=41% |
| 2579 | GOCC\_AGGRESOME |  | 24 | -0.43 | -1.23 | 0.185 | 0.504 | 1.000 | 3766 | tags=54%, list=31%, signal=79% |
| 2580 | GOBP\_POSITIVE\_REGULATION\_OF\_T\_CELL\_MIGRATION |  | 21 | -0.44 | -1.23 | 0.200 | 0.504 | 1.000 | 3344 | tags=52%, list=28%, signal=72% |
| 2581 | GOBP\_GRANULOCYTE\_MACROPHAGE\_COLONY\_STIMULATING\_FACTOR\_PRODUCTION |  | 7 | -0.58 | -1.23 | 0.203 | 0.504 | 1.000 | 2547 | tags=57%, list=21%, signal=72% |
| 2582 | GOBP\_PSEUDOPODIUM\_ORGANIZATION |  | 11 | -0.51 | -1.23 | 0.205 | 0.505 | 1.000 | 2480 | tags=36%, list=21%, signal=46% |
| 2583 | GOBP\_HISTONE\_H4\_K5\_ACETYLATION |  | 13 | -0.49 | -1.23 | 0.197 | 0.506 | 1.000 | 2798 | tags=38%, list=23%, signal=50% |
| 2584 | GOBP\_CARDIAC\_MUSCLE\_MYOBLAST\_PROLIFERATION |  | 3 | -0.74 | -1.23 | 0.177 | 0.506 | 1.000 | 1838 | tags=67%, list=15%, signal=79% |
| 2585 | GOCC\_TRANSFERASE\_COMPLEX\_TRANSFERRING\_PHOSPHORUS\_CONTAINING\_GROUPS |  | 195 | -0.33 | -1.23 | 0.070 | 0.505 | 1.000 | 4386 | tags=43%, list=37%, signal=66% |
| 2586 | GOBP\_RESPONSE\_TO\_MOLECULE\_OF\_FUNGAL\_ORIGIN |  | 3 | -0.73 | -1.23 | 0.183 | 0.505 | 1.000 | 2357 | tags=67%, list=20%, signal=83% |
| 2587 | GOBP\_SPERM\_CHROMATIN\_CONDENSATION |  | 5 | -0.64 | -1.23 | 0.186 | 0.505 | 1.000 | 4298 | tags=80%, list=36%, signal=125% |
| 2588 | GOBP\_MICROVILLUS\_ORGANIZATION |  | 18 | -0.45 | -1.23 | 0.194 | 0.505 | 1.000 | 5082 | tags=72%, list=42%, signal=125% |
| 2589 | GOMF\_CARBOXYPEPTIDASE\_ACTIVITY |  | 25 | -0.42 | -1.23 | 0.186 | 0.505 | 1.000 | 5105 | tags=68%, list=43%, signal=118% |
| 2590 | GOBP\_T\_CELL\_CYTOKINE\_PRODUCTION |  | 21 | -0.44 | -1.23 | 0.191 | 0.506 | 1.000 | 1781 | tags=38%, list=15%, signal=45% |
| 2591 | GOBP\_PROTEIN\_LOCALIZATION\_TO\_PLASMA\_MEMBRANE |  | 208 | -0.32 | -1.23 | 0.063 | 0.506 | 1.000 | 4398 | tags=46%, list=37%, signal=71% |
| 2592 | GOBP\_CORPUS\_CALLOSUM\_DEVELOPMENT |  | 12 | -0.50 | -1.23 | 0.205 | 0.506 | 1.000 | 1309 | tags=25%, list=11%, signal=28% |
| 2593 | GOBP\_NEGATIVE\_REGULATION\_OF\_CYTOKINE\_PRODUCTION |  | 164 | -0.33 | -1.23 | 0.079 | 0.506 | 1.000 | 3226 | tags=37%, list=27%, signal=49% |
| 2594 | GOBP\_REGULATION\_OF\_VITAMIN\_D\_BIOSYNTHETIC\_PROCESS |  | 4 | -0.68 | -1.23 | 0.182 | 0.506 | 1.000 | 2666 | tags=75%, list=22%, signal=96% |
| 2595 | GOBP\_VERY\_LONG\_CHAIN\_FATTY\_ACID\_CATABOLIC\_PROCESS |  | 5 | -0.64 | -1.23 | 0.185 | 0.506 | 1.000 | 4053 | tags=80%, list=34%, signal=121% |
| 2596 | GOCC\_MLL1\_2\_COMPLEX |  | 18 | -0.45 | -1.23 | 0.209 | 0.506 | 1.000 | 2810 | tags=33%, list=23%, signal=43% |
| 2597 | GOBP\_MUSCLE\_TISSUE\_DEVELOPMENT |  | 236 | -0.32 | -1.23 | 0.058 | 0.506 | 1.000 | 3615 | tags=33%, list=30%, signal=47% |
| 2598 | GOBP\_PROTEIN\_KINASE\_A\_SIGNALING |  | 22 | -0.44 | -1.23 | 0.197 | 0.507 | 1.000 | 3609 | tags=59%, list=30%, signal=84% |
| 2599 | GOCC\_SWI\_SNF\_COMPLEX |  | 15 | -0.47 | -1.23 | 0.200 | 0.506 | 1.000 | 3836 | tags=47%, list=32%, signal=68% |
| 2600 | GOBP\_CRANIAL\_SKELETAL\_SYSTEM\_DEVELOPMENT |  | 34 | -0.40 | -1.23 | 0.179 | 0.506 | 1.000 | 4868 | tags=62%, list=41%, signal=104% |
| 2601 | GOBP\_NEGATIVE\_REGULATION\_OF\_VITAMIN\_METABOLIC\_PROCESS |  | 4 | -0.68 | -1.23 | 0.190 | 0.507 | 1.000 | 2666 | tags=75%, list=22%, signal=96% |
| 2602 | GOBP\_APOPTOTIC\_DNA\_FRAGMENTATION |  | 16 | -0.46 | -1.23 | 0.205 | 0.507 | 1.000 | 5564 | tags=75%, list=46%, signal=140% |
| 2603 | GOBP\_REGULATION\_OF\_PRESYNAPSE\_ORGANIZATION |  | 16 | -0.47 | -1.23 | 0.205 | 0.508 | 1.000 | 5068 | tags=56%, list=42%, signal=97% |
| 2604 | GOBP\_TISSUE\_MIGRATION |  | 239 | -0.32 | -1.23 | 0.057 | 0.508 | 1.000 | 3196 | tags=33%, list=27%, signal=45% |
| 2605 | GOMF\_NARROW\_PORE\_CHANNEL\_ACTIVITY |  | 11 | -0.51 | -1.23 | 0.209 | 0.508 | 1.000 | 1183 | tags=27%, list=10%, signal=30% |
| 2606 | GOBP\_MONONUCLEAR\_CELL\_MIGRATION |  | 112 | -0.34 | -1.23 | 0.116 | 0.508 | 1.000 | 2955 | tags=35%, list=25%, signal=46% |
| 2607 | GOBP\_POSITIVE\_REGULATION\_OF\_GRANULOCYTE\_MACROPHAGE\_COLONY\_STIMULATING\_FACTOR\_PRODUCTION |  | 7 | -0.58 | -1.23 | 0.203 | 0.509 | 1.000 | 2547 | tags=57%, list=21%, signal=72% |
| 2608 | GOBP\_VITAMIN\_BIOSYNTHETIC\_PROCESS |  | 16 | -0.47 | -1.23 | 0.199 | 0.508 | 1.000 | 2666 | tags=44%, list=22%, signal=56% |
| 2609 | GOBP\_FOCAL\_ADHESION\_ASSEMBLY |  | 72 | -0.35 | -1.23 | 0.141 | 0.508 | 1.000 | 3575 | tags=42%, list=30%, signal=59% |
| 2610 | GOBP\_PEPTIDYL\_TYROSINE\_DEPHOSPHORYLATION\_INVOLVED\_IN\_INACTIVATION\_OF\_PROTEIN\_KINASE\_ACTIVITY |  | 6 | -0.60 | -1.23 | 0.211 | 0.509 | 1.000 | 547 | tags=33%, list=5%, signal=35% |
| 2611 | GOBP\_MOTOR\_NEURON\_MIGRATION |  | 3 | -0.73 | -1.23 | 0.188 | 0.509 | 1.000 | 3196 | tags=100%, list=27%, signal=136% |
| 2612 | GOBP\_NEGATIVE\_REGULATION\_OF\_TUBULIN\_DEACETYLATION |  | 4 | -0.68 | -1.23 | 0.196 | 0.510 | 1.000 | 685 | tags=50%, list=6%, signal=53% |
| 2613 | GOMF\_CARGO\_RECEPTOR\_ACTIVITY |  | 42 | -0.38 | -1.23 | 0.166 | 0.510 | 1.000 | 3007 | tags=36%, list=25%, signal=47% |
| 2614 | GOBP\_DNA\_DAMAGE\_INDUCED\_PROTEIN\_PHOSPHORYLATION |  | 8 | -0.55 | -1.23 | 0.211 | 0.509 | 1.000 | 4400 | tags=75%, list=37%, signal=118% |
| 2615 | GOBP\_COLUMNAR\_CUBOIDAL\_EPITHELIAL\_CELL\_MATURATION |  | 6 | -0.60 | -1.23 | 0.201 | 0.509 | 1.000 | 1347 | tags=33%, list=11%, signal=38% |
| 2616 | GOMF\_AP\_2\_ADAPTOR\_COMPLEX\_BINDING |  | 5 | -0.64 | -1.23 | 0.196 | 0.510 | 1.000 | 4050 | tags=80%, list=34%, signal=121% |
| 2617 | GOBP\_GLOMERULUS\_DEVELOPMENT |  | 49 | -0.37 | -1.23 | 0.173 | 0.510 | 1.000 | 4102 | tags=39%, list=34%, signal=59% |
| 2618 | GOMF\_CHEMOKINE\_BINDING |  | 18 | -0.45 | -1.23 | 0.207 | 0.511 | 1.000 | 2871 | tags=50%, list=24%, signal=66% |
| 2619 | GOBP\_REGULATION\_OF\_ACTIN\_FILAMENT\_LENGTH |  | 130 | -0.33 | -1.23 | 0.101 | 0.511 | 1.000 | 3032 | tags=30%, list=25%, signal=40% |
| 2620 | GOBP\_CRANIAL\_NERVE\_DEVELOPMENT |  | 24 | -0.42 | -1.23 | 0.198 | 0.511 | 1.000 | 1660 | tags=25%, list=14%, signal=29% |
| 2621 | GOBP\_REGULATION\_OF\_BMP\_SIGNALING\_PATHWAY |  | 62 | -0.36 | -1.23 | 0.157 | 0.511 | 1.000 | 3126 | tags=40%, list=26%, signal=54% |
| 2622 | GOBP\_MUSCLE\_CONTRACTION |  | 211 | -0.32 | -1.23 | 0.070 | 0.511 | 1.000 | 1928 | tags=21%, list=16%, signal=25% |
| 2623 | GOCC\_SPINDLE\_MICROTUBULE |  | 52 | -0.37 | -1.23 | 0.170 | 0.511 | 1.000 | 5551 | tags=58%, list=46%, signal=107% |
| 2624 | GOBP\_TROPHECTODERMAL\_CELL\_DIFFERENTIATION |  | 12 | -0.50 | -1.23 | 0.201 | 0.511 | 1.000 | 3195 | tags=50%, list=27%, signal=68% |
| 2625 | GOBP\_CELL\_CYCLE\_G1\_S\_PHASE\_TRANSITION |  | 209 | -0.32 | -1.23 | 0.072 | 0.511 | 1.000 | 4428 | tags=44%, list=37%, signal=69% |
| 2626 | GOBP\_REGULATION\_OF\_NUCLEOBASE\_CONTAINING\_COMPOUND\_TRANSPORT |  | 13 | -0.49 | -1.23 | 0.207 | 0.511 | 1.000 | 4278 | tags=54%, list=36%, signal=84% |
| 2627 | GOBP\_REGULATION\_OF\_CALCIUM\_ION\_TRANSMEMBRANE\_TRANSPORT |  | 95 | -0.34 | -1.23 | 0.122 | 0.511 | 1.000 | 3027 | tags=29%, list=25%, signal=39% |
| 2628 | GOBP\_POSITIVE\_REGULATION\_OF\_MAMMARY\_GLAND\_EPITHELIAL\_CELL\_PROLIFERATION |  | 7 | -0.57 | -1.23 | 0.207 | 0.513 | 1.000 | 2615 | tags=57%, list=22%, signal=73% |
| 2629 | GOBP\_ASTROCYTE\_CELL\_MIGRATION |  | 3 | -0.74 | -1.23 | 0.177 | 0.513 | 1.000 | 3178 | tags=100%, list=26%, signal=136% |
| 2630 | GOBP\_POSITIVE\_REGULATION\_OF\_SPINDLE\_CHECKPOINT |  | 7 | -0.58 | -1.23 | 0.210 | 0.513 | 1.000 | 2733 | tags=43%, list=23%, signal=55% |
| 2631 | GOBP\_IN\_UTERO\_EMBRYONIC\_DEVELOPMENT |  | 248 | -0.32 | -1.23 | 0.056 | 0.513 | 1.000 | 4111 | tags=37%, list=34%, signal=55% |
| 2632 | GOBP\_CHYLOMICRON\_ASSEMBLY |  | 7 | -0.58 | -1.23 | 0.205 | 0.513 | 1.000 | 3217 | tags=57%, list=27%, signal=78% |
| 2633 | GOMF\_PHOSPHATASE\_REGULATOR\_ACTIVITY |  | 66 | -0.36 | -1.23 | 0.146 | 0.513 | 1.000 | 4627 | tags=48%, list=39%, signal=78% |
| 2634 | GOBP\_BONE\_MINERALIZATION |  | 72 | -0.35 | -1.23 | 0.142 | 0.513 | 1.000 | 3488 | tags=40%, list=29%, signal=56% |
| 2635 | GOMF\_POLYPEPTIDE\_N\_ACETYLGALACTOSAMINYLTRANSFERASE\_ACTIVITY |  | 14 | -0.48 | -1.23 | 0.205 | 0.514 | 1.000 | 2617 | tags=43%, list=22%, signal=55% |
| 2636 | GOBP\_NEGATIVE\_REGULATION\_OF\_VITAMIN\_D\_BIOSYNTHETIC\_PROCESS |  | 4 | -0.68 | -1.23 | 0.191 | 0.514 | 1.000 | 2666 | tags=75%, list=22%, signal=96% |
| 2637 | GOBP\_CALCIUM\_ACTIVATED\_PHOSPHOLIPID\_SCRAMBLING |  | 3 | -0.73 | -1.23 | 0.183 | 0.514 | 1.000 | 1195 | tags=67%, list=10%, signal=74% |
| 2638 | GOBP\_INTERLEUKIN\_1\_PRODUCTION |  | 51 | -0.37 | -1.23 | 0.172 | 0.514 | 1.000 | 2480 | tags=33%, list=21%, signal=42% |
| 2639 | GOMF\_CHLORIDE\_TRANSMEMBRANE\_TRANSPORTER\_ACTIVITY |  | 50 | -0.37 | -1.23 | 0.165 | 0.514 | 1.000 | 2597 | tags=40%, list=22%, signal=51% |
| 2640 | GOBP\_POSITIVE\_REGULATION\_OF\_ENDOTHELIAL\_CELL\_CHEMOTAXIS |  | 11 | -0.51 | -1.23 | 0.210 | 0.514 | 1.000 | 3588 | tags=45%, list=30%, signal=65% |
| 2641 | GOBP\_ENDOTHELIAL\_CELL\_CHEMOTAXIS |  | 21 | -0.44 | -1.23 | 0.204 | 0.514 | 1.000 | 3588 | tags=52%, list=30%, signal=75% |
| 2642 | GOBP\_MODULATION\_BY\_HOST\_OF\_VIRAL\_PROCESS |  | 21 | -0.43 | -1.23 | 0.210 | 0.514 | 1.000 | 3093 | tags=38%, list=26%, signal=51% |
| 2643 | GOBP\_POSITIVE\_REGULATION\_OF\_ANTIGEN\_PROCESSING\_AND\_PRESENTATION |  | 6 | -0.60 | -1.23 | 0.202 | 0.514 | 1.000 | 4317 | tags=50%, list=36%, signal=78% |
| 2644 | GOBP\_POSITIVE\_REGULATION\_OF\_EPITHELIAL\_CELL\_MIGRATION |  | 121 | -0.33 | -1.23 | 0.114 | 0.514 | 1.000 | 3968 | tags=40%, list=33%, signal=60% |
| 2645 | GOBP\_GOLGI\_TO\_ENDOSOME\_TRANSPORT |  | 13 | -0.49 | -1.22 | 0.223 | 0.515 | 1.000 | 3769 | tags=54%, list=31%, signal=78% |
| 2646 | GOCC\_PRESYNAPSE |  | 310 | -0.32 | -1.22 | 0.042 | 0.515 | 1.000 | 2876 | tags=29%, list=24%, signal=38% |
| 2647 | GOBP\_NEGATIVE\_REGULATION\_OF\_FATTY\_ACID\_BIOSYNTHETIC\_PROCESS |  | 8 | -0.56 | -1.22 | 0.208 | 0.515 | 1.000 | 2656 | tags=63%, list=22%, signal=80% |
| 2648 | GOBP\_POSITIVE\_REGULATION\_OF\_DNA\_REPAIR |  | 52 | -0.37 | -1.22 | 0.173 | 0.515 | 1.000 | 3452 | tags=38%, list=29%, signal=54% |
| 2649 | GOBP\_MUSCLE\_ORGAN\_DEVELOPMENT |  | 197 | -0.32 | -1.22 | 0.073 | 0.515 | 1.000 | 2974 | tags=29%, list=25%, signal=38% |
| 2650 | GOBP\_BLASTOCYST\_GROWTH |  | 18 | -0.45 | -1.22 | 0.211 | 0.515 | 1.000 | 881 | tags=17%, list=7%, signal=18% |
| 2651 | GOBP\_ANIMAL\_ORGAN\_MORPHOGENESIS |  | 658 | -0.31 | -1.22 | 0.008 | 0.515 | 1.000 | 3565 | tags=33%, list=30%, signal=44% |
| 2652 | GOCC\_SUPRAMOLECULAR\_COMPLEX |  | 817 | -0.31 | -1.22 | 0.004 | 0.515 | 1.000 | 4456 | tags=40%, list=37%, signal=59% |
| 2653 | GOCC\_SEH1\_ASSOCIATED\_COMPLEX |  | 14 | -0.48 | -1.22 | 0.208 | 0.515 | 1.000 | 3512 | tags=36%, list=29%, signal=50% |
| 2654 | GOBP\_PROLINE\_TRANSMEMBRANE\_TRANSPORT |  | 6 | -0.61 | -1.22 | 0.204 | 0.515 | 1.000 | 2825 | tags=67%, list=24%, signal=87% |
| 2655 | GOMF\_1\_PHOSPHATIDYLINOSITOL\_4\_PHOSPHATE\_5\_KINASE\_ACTIVITY |  | 6 | -0.60 | -1.22 | 0.210 | 0.515 | 1.000 | 2289 | tags=33%, list=19%, signal=41% |
| 2656 | GOBP\_POSITIVE\_REGULATION\_OF\_PROTEOLYSIS |  | 248 | -0.32 | -1.22 | 0.062 | 0.516 | 1.000 | 2781 | tags=27%, list=23%, signal=34% |
| 2657 | GOBP\_POSITIVE\_REGULATION\_OF\_NIK\_NF\_KAPPAB\_SIGNALING |  | 49 | -0.37 | -1.22 | 0.169 | 0.515 | 1.000 | 2731 | tags=33%, list=23%, signal=42% |
| 2658 | GOBP\_DEOXYRIBONUCLEOSIDE\_MONOPHOSPHATE\_CATABOLIC\_PROCESS |  | 6 | -0.60 | -1.22 | 0.207 | 0.516 | 1.000 | 1107 | tags=50%, list=9%, signal=55% |
| 2659 | GOBP\_HISTONE\_DEACETYLATION |  | 51 | -0.37 | -1.22 | 0.166 | 0.515 | 1.000 | 4481 | tags=47%, list=37%, signal=75% |
| 2660 | GOMF\_BUBBLE\_DNA\_BINDING |  | 7 | -0.58 | -1.22 | 0.211 | 0.516 | 1.000 | 3214 | tags=71%, list=27%, signal=97% |
| 2661 | GOBP\_ASSEMBLY\_OF\_ACTOMYOSIN\_APPARATUS\_INVOLVED\_IN\_CYTOKINESIS |  | 5 | -0.64 | -1.22 | 0.201 | 0.516 | 1.000 | 2588 | tags=60%, list=22%, signal=76% |
| 2662 | GOBP\_RENAL\_FILTRATION |  | 20 | -0.44 | -1.22 | 0.209 | 0.516 | 1.000 | 2480 | tags=25%, list=21%, signal=31% |
| 2663 | GOBP\_REGULATION\_OF\_CILIUM\_ASSEMBLY |  | 40 | -0.39 | -1.22 | 0.176 | 0.516 | 1.000 | 5206 | tags=55%, list=43%, signal=97% |
| 2664 | GOMF\_POLYOL\_TRANSMEMBRANE\_TRANSPORTER\_ACTIVITY |  | 7 | -0.57 | -1.22 | 0.217 | 0.516 | 1.000 | 2215 | tags=43%, list=18%, signal=53% |
| 2665 | GOBP\_REGULATION\_OF\_SYNAPTIC\_VESICLE\_ENDOCYTOSIS |  | 11 | -0.51 | -1.22 | 0.216 | 0.516 | 1.000 | 4673 | tags=55%, list=39%, signal=89% |
| 2666 | GOBP\_TELOMERE\_MAINTENANCE\_IN\_RESPONSE\_TO\_DNA\_DAMAGE |  | 9 | -0.53 | -1.22 | 0.214 | 0.516 | 1.000 | 4024 | tags=67%, list=34%, signal=100% |
| 2667 | GOBP\_RETINAL\_ROD\_CELL\_DEVELOPMENT |  | 5 | -0.63 | -1.22 | 0.200 | 0.516 | 1.000 | 1520 | tags=40%, list=13%, signal=46% |
| 2668 | GOBP\_REGULATION\_OF\_PROTEIN\_LOCALIZATION\_TO\_CELL\_CORTEX |  | 8 | -0.55 | -1.22 | 0.223 | 0.516 | 1.000 | 1814 | tags=25%, list=15%, signal=29% |
| 2669 | GOBP\_NK\_T\_CELL\_ACTIVATION |  | 4 | -0.67 | -1.22 | 0.200 | 0.516 | 1.000 | 1566 | tags=50%, list=13%, signal=57% |
| 2670 | GOBP\_POSITIVE\_REGULATION\_OF\_INTERLEUKIN\_1\_PRODUCTION |  | 25 | -0.42 | -1.22 | 0.195 | 0.516 | 1.000 | 2374 | tags=36%, list=20%, signal=45% |
| 2671 | GOBP\_DIGESTION |  | 68 | -0.36 | -1.22 | 0.150 | 0.517 | 1.000 | 1939 | tags=28%, list=16%, signal=33% |
| 2672 | GOBP\_NEGATIVE\_REGULATION\_OF\_MYELOID\_LEUKOCYTE\_DIFFERENTIATION |  | 29 | -0.41 | -1.22 | 0.193 | 0.517 | 1.000 | 1579 | tags=28%, list=13%, signal=32% |
| 2673 | GOBP\_TORC2\_SIGNALING |  | 8 | -0.55 | -1.22 | 0.213 | 0.517 | 1.000 | 2850 | tags=63%, list=24%, signal=82% |
| 2674 | GOBP\_DEVELOPMENTAL\_MATURATION |  | 175 | -0.32 | -1.22 | 0.092 | 0.518 | 1.000 | 3483 | tags=37%, list=29%, signal=51% |
| 2675 | GOMF\_CATALYTIC\_ACTIVITY\_ACTING\_ON\_A\_GLYCOPROTEIN |  | 16 | -0.46 | -1.22 | 0.218 | 0.518 | 1.000 | 3539 | tags=44%, list=29%, signal=62% |
| 2676 | GOBP\_POSITIVE\_REGULATION\_OF\_RAC\_PROTEIN\_SIGNAL\_TRANSDUCTION |  | 6 | -0.60 | -1.22 | 0.214 | 0.518 | 1.000 | 1804 | tags=50%, list=15%, signal=59% |
| 2677 | GOBP\_REGULATION\_OF\_CIRCADIAN\_SLEEP\_WAKE\_CYCLE |  | 9 | -0.54 | -1.22 | 0.214 | 0.518 | 1.000 | 786 | tags=33%, list=7%, signal=36% |
| 2678 | GOBP\_STRESS\_FIBER\_ASSEMBLY |  | 81 | -0.35 | -1.22 | 0.144 | 0.518 | 1.000 | 2560 | tags=31%, list=21%, signal=39% |
| 2679 | GOBP\_LENS\_MORPHOGENESIS\_IN\_CAMERA\_TYPE\_EYE |  | 14 | -0.48 | -1.22 | 0.220 | 0.517 | 1.000 | 3126 | tags=57%, list=26%, signal=77% |
| 2680 | GOCC\_CLATHRIN\_COAT\_OF\_COATED\_PIT |  | 12 | -0.50 | -1.22 | 0.215 | 0.518 | 1.000 | 4775 | tags=75%, list=40%, signal=124% |
| 2681 | GOMF\_M7G\_5\_PPPN\_DIPHOSPHATASE\_ACTIVITY |  | 4 | -0.68 | -1.22 | 0.200 | 0.518 | 1.000 | 2574 | tags=50%, list=21%, signal=64% |
| 2682 | GOBP\_REGULATION\_OF\_POLYSACCHARIDE\_BIOSYNTHETIC\_PROCESS |  | 24 | -0.42 | -1.22 | 0.205 | 0.518 | 1.000 | 3934 | tags=50%, list=33%, signal=74% |
| 2683 | GOBP\_G\_PROTEIN\_COUPLED\_PURINERGIC\_RECEPTOR\_SIGNALING\_PATHWAY |  | 7 | -0.57 | -1.22 | 0.218 | 0.518 | 1.000 | 1796 | tags=43%, list=15%, signal=50% |
| 2684 | GOBP\_REGULATION\_OF\_RESPONSE\_TO\_DNA\_DAMAGE\_STIMULUS |  | 160 | -0.33 | -1.22 | 0.093 | 0.518 | 1.000 | 4125 | tags=42%, list=34%, signal=63% |
| 2685 | GOBP\_PEPTIDYL\_TYROSINE\_DEPHOSPHORYLATION |  | 81 | -0.35 | -1.22 | 0.146 | 0.517 | 1.000 | 3973 | tags=43%, list=33%, signal=64% |
| 2686 | GOBP\_REGULATION\_OF\_PROTEIN\_GLYCOSYLATION |  | 13 | -0.49 | -1.22 | 0.218 | 0.518 | 1.000 | 1173 | tags=23%, list=10%, signal=26% |
| 2687 | GOBP\_POSITIVE\_REGULATION\_OF\_NEUROTRANSMITTER\_SECRETION |  | 12 | -0.50 | -1.22 | 0.215 | 0.518 | 1.000 | 1699 | tags=33%, list=14%, signal=39% |
| 2688 | GOBP\_NEGATIVE\_REGULATION\_OF\_AUTOPHAGY\_OF\_MITOCHONDRION |  | 5 | -0.64 | -1.22 | 0.204 | 0.518 | 1.000 | 2263 | tags=40%, list=19%, signal=49% |
| 2689 | GOBP\_REGULATION\_OF\_TUBULIN\_DEACETYLATION |  | 7 | -0.57 | -1.22 | 0.218 | 0.518 | 1.000 | 4528 | tags=86%, list=38%, signal=138% |
| 2690 | GOBP\_FOLLICLE\_STIMULATING\_HORMONE\_SECRETION |  | 3 | -0.73 | -1.22 | 0.187 | 0.518 | 1.000 | 226 | tags=33%, list=2%, signal=34% |
| 2691 | GOBP\_POSITIVE\_REGULATION\_OF\_GLIAL\_CELL\_MIGRATION |  | 9 | -0.53 | -1.22 | 0.220 | 0.518 | 1.000 | 4868 | tags=67%, list=41%, signal=112% |
| 2692 | GOMF\_GLUCOSE\_SODIUM\_SYMPORTER\_ACTIVITY |  | 4 | -0.67 | -1.22 | 0.203 | 0.518 | 1.000 | 620 | tags=50%, list=5%, signal=53% |
| 2693 | GOBP\_VITAMIN\_D\_BIOSYNTHETIC\_PROCESS |  | 7 | -0.58 | -1.22 | 0.209 | 0.518 | 1.000 | 2666 | tags=57%, list=22%, signal=73% |
| 2694 | GOBP\_RESPONSE\_TO\_CISPLATIN |  | 6 | -0.60 | -1.22 | 0.206 | 0.519 | 1.000 | 4465 | tags=83%, list=37%, signal=133% |
| 2695 | GOCC\_SUPRAMOLECULAR\_POLYMER |  | 581 | -0.31 | -1.22 | 0.012 | 0.519 | 1.000 | 3559 | tags=32%, list=30%, signal=44% |
| 2696 | GOBP\_ENTRY\_INTO\_HOST |  | 92 | -0.34 | -1.22 | 0.144 | 0.519 | 1.000 | 2894 | tags=34%, list=24%, signal=44% |
| 2697 | GOBP\_HISTONE\_H4\_DEACETYLATION |  | 10 | -0.52 | -1.22 | 0.209 | 0.519 | 1.000 | 2717 | tags=40%, list=23%, signal=52% |
| 2698 | GOBP\_NEGATIVE\_REGULATION\_OF\_INTERLEUKIN\_4\_PRODUCTION |  | 5 | -0.63 | -1.22 | 0.204 | 0.519 | 1.000 | 2308 | tags=60%, list=19%, signal=74% |
| 2699 | GOBP\_HEPATOCYTE\_DIFFERENTIATION |  | 14 | -0.48 | -1.22 | 0.216 | 0.519 | 1.000 | 2187 | tags=36%, list=18%, signal=44% |
| 2700 | GOCC\_JUXTAPARANODE\_REGION\_OF\_AXON |  | 6 | -0.60 | -1.22 | 0.213 | 0.518 | 1.000 | 1125 | tags=33%, list=9%, signal=37% |
| 2701 | GOMF\_G\_QUADRUPLEX\_DNA\_BINDING |  | 10 | -0.52 | -1.22 | 0.216 | 0.518 | 1.000 | 4569 | tags=70%, list=38%, signal=113% |
| 2702 | GOBP\_VESICLE\_LOCALIZATION |  | 177 | -0.32 | -1.22 | 0.088 | 0.519 | 1.000 | 4021 | tags=39%, list=33%, signal=58% |
| 2703 | GOBP\_MITOTIC\_DNA\_REPLICATION\_CHECKPOINT |  | 9 | -0.53 | -1.22 | 0.228 | 0.519 | 1.000 | 4764 | tags=67%, list=40%, signal=110% |
| 2704 | GOBP\_POSITIVE\_REGULATION\_OF\_AXON\_EXTENSION\_INVOLVED\_IN\_AXON\_GUIDANCE |  | 5 | -0.63 | -1.22 | 0.214 | 0.519 | 1.000 | 3196 | tags=80%, list=27%, signal=109% |
| 2705 | GOBP\_IMMUNOGLOBULIN\_PRODUCTION |  | 62 | -0.36 | -1.22 | 0.165 | 0.519 | 1.000 | 3205 | tags=35%, list=27%, signal=48% |
| 2706 | GOCC\_COPII\_VESICLE\_COAT |  | 15 | -0.47 | -1.22 | 0.210 | 0.519 | 1.000 | 4239 | tags=60%, list=35%, signal=93% |
| 2707 | GOCC\_PERINUCLEAR\_REGION\_OF\_CYTOPLASM |  | 522 | -0.31 | -1.22 | 0.019 | 0.519 | 1.000 | 3062 | tags=30%, list=25%, signal=38% |
| 2708 | GOBP\_RIBONUCLEOPROTEIN\_COMPLEX\_DISASSEMBLY |  | 7 | -0.58 | -1.22 | 0.216 | 0.519 | 1.000 | 4104 | tags=71%, list=34%, signal=108% |
| 2709 | GOBP\_ASYMMETRIC\_CELL\_DIVISION |  | 12 | -0.49 | -1.22 | 0.223 | 0.519 | 1.000 | 1803 | tags=25%, list=15%, signal=29% |
| 2710 | GOBP\_NEGATIVE\_REGULATION\_OF\_CD4\_POSITIVE\_ALPHA\_BETA\_T\_CELL\_PROLIFERATION |  | 4 | -0.67 | -1.22 | 0.200 | 0.519 | 1.000 | 3163 | tags=75%, list=26%, signal=102% |
| 2711 | GOBP\_SKIN\_EPIDERMIS\_DEVELOPMENT |  | 59 | -0.36 | -1.22 | 0.170 | 0.519 | 1.000 | 2212 | tags=24%, list=18%, signal=29% |
| 2712 | GOBP\_SUBPALLIUM\_DEVELOPMENT |  | 15 | -0.47 | -1.22 | 0.212 | 0.519 | 1.000 | 2466 | tags=40%, list=21%, signal=50% |
| 2713 | GOBP\_CELLULAR\_EXTRAVASATION |  | 46 | -0.37 | -1.22 | 0.175 | 0.519 | 1.000 | 3538 | tags=43%, list=29%, signal=61% |
| 2714 | GOBP\_NEGATIVE\_REGULATION\_OF\_ORGANELLE\_ORGANIZATION |  | 247 | -0.32 | -1.22 | 0.069 | 0.519 | 1.000 | 3812 | tags=36%, list=32%, signal=52% |
| 2715 | GOBP\_ATTACHMENT\_OF\_SPINDLE\_MICROTUBULES\_TO\_KINETOCHORE |  | 29 | -0.40 | -1.22 | 0.203 | 0.519 | 1.000 | 5627 | tags=69%, list=47%, signal=129% |
| 2716 | GOMF\_ALCOHOL\_BINDING |  | 61 | -0.36 | -1.22 | 0.172 | 0.519 | 1.000 | 3316 | tags=38%, list=28%, signal=52% |
| 2717 | GOBP\_NUCLEUS\_ORGANIZATION |  | 93 | -0.34 | -1.22 | 0.141 | 0.519 | 1.000 | 4378 | tags=42%, list=36%, signal=65% |
| 2718 | GOBP\_CELLULAR\_CARBOHYDRATE\_METABOLIC\_PROCESS |  | 208 | -0.32 | -1.22 | 0.079 | 0.520 | 1.000 | 4280 | tags=43%, list=36%, signal=66% |
| 2719 | GOCC\_OMEGASOME |  | 4 | -0.67 | -1.22 | 0.202 | 0.519 | 1.000 | 895 | tags=50%, list=7%, signal=54% |
| 2720 | GOBP\_REGULATION\_OF\_T\_HELPER\_CELL\_DIFFERENTIATION |  | 24 | -0.42 | -1.22 | 0.207 | 0.520 | 1.000 | 2345 | tags=38%, list=20%, signal=47% |
| 2721 | GOBP\_ANOIKIS |  | 24 | -0.42 | -1.22 | 0.210 | 0.520 | 1.000 | 4822 | tags=50%, list=40%, signal=83% |
| 2722 | GOMF\_COLLAGEN\_BINDING |  | 54 | -0.37 | -1.22 | 0.172 | 0.521 | 1.000 | 3186 | tags=33%, list=27%, signal=45% |
| 2723 | GOBP\_POSITIVE\_REGULATION\_OF\_AMYLOID\_PRECURSOR\_PROTEIN\_CATABOLIC\_PROCESS |  | 16 | -0.46 | -1.22 | 0.215 | 0.521 | 1.000 | 2416 | tags=31%, list=20%, signal=39% |
| 2724 | GOBP\_POSITIVE\_REGULATION\_OF\_PROTEIN\_GLYCOSYLATION |  | 8 | -0.55 | -1.22 | 0.216 | 0.521 | 1.000 | 909 | tags=25%, list=8%, signal=27% |
| 2725 | GOBP\_REGULATION\_OF\_COAGULATION |  | 46 | -0.37 | -1.22 | 0.184 | 0.522 | 1.000 | 1344 | tags=22%, list=11%, signal=24% |
| 2726 | GOBP\_SEMAPHORIN\_PLEXIN\_SIGNALING\_PATHWAY\_INVOLVED\_IN\_NEURON\_PROJECTION\_GUIDANCE |  | 10 | -0.52 | -1.22 | 0.215 | 0.522 | 1.000 | 3895 | tags=60%, list=32%, signal=89% |
| 2727 | GOMF\_INTERLEUKIN\_1\_RECEPTOR\_ACTIVITY |  | 7 | -0.57 | -1.22 | 0.214 | 0.521 | 1.000 | 5068 | tags=86%, list=42%, signal=148% |
| 2728 | GOBP\_POSITIVE\_REGULATION\_OF\_T\_HELPER\_2\_CELL\_DIFFERENTIATION |  | 6 | -0.60 | -1.22 | 0.208 | 0.521 | 1.000 | 1566 | tags=50%, list=13%, signal=57% |
| 2729 | GOBP\_NEGATIVE\_REGULATION\_OF\_EPITHELIAL\_CELL\_PROLIFERATION |  | 90 | -0.34 | -1.22 | 0.150 | 0.522 | 1.000 | 2852 | tags=32%, list=24%, signal=42% |
| 2730 | GOBP\_TRNA\_TRANSCRIPTION |  | 7 | -0.57 | -1.22 | 0.208 | 0.522 | 1.000 | 1760 | tags=43%, list=15%, signal=50% |
| 2731 | GOMF\_CALCIUM\_POTASSIUM\_SODIUM\_ANTIPORTER\_ACTIVITY |  | 3 | -0.73 | -1.22 | 0.191 | 0.522 | 1.000 | 933 | tags=33%, list=8%, signal=36% |
| 2732 | GOBP\_CIRCADIAN\_SLEEP\_WAKE\_CYCLE |  | 12 | -0.50 | -1.22 | 0.221 | 0.522 | 1.000 | 2351 | tags=42%, list=20%, signal=52% |
| 2733 | GOBP\_PEPTIDYL\_ARGININE\_METHYLATION |  | 8 | -0.55 | -1.22 | 0.223 | 0.522 | 1.000 | 3647 | tags=63%, list=30%, signal=90% |
| 2734 | GOMF\_GROWTH\_FACTOR\_BINDING |  | 106 | -0.34 | -1.22 | 0.137 | 0.522 | 1.000 | 3295 | tags=33%, list=27%, signal=45% |
| 2735 | GOBP\_CELL\_FATE\_SPECIFICATION |  | 47 | -0.37 | -1.22 | 0.182 | 0.522 | 1.000 | 2212 | tags=26%, list=18%, signal=31% |
| 2736 | GOBP\_TRANS\_SYNAPTIC\_SIGNALING\_MODULATING\_SYNAPTIC\_TRANSMISSION |  | 5 | -0.63 | -1.22 | 0.202 | 0.522 | 1.000 | 1309 | tags=40%, list=11%, signal=45% |
| 2737 | GOBP\_PURINE\_CONTAINING\_COMPOUND\_CATABOLIC\_PROCESS |  | 37 | -0.39 | -1.22 | 0.189 | 0.521 | 1.000 | 2574 | tags=35%, list=21%, signal=45% |
| 2738 | GOBP\_CARBOHYDRATE\_PHOSPHORYLATION |  | 21 | -0.44 | -1.22 | 0.212 | 0.521 | 1.000 | 3409 | tags=52%, list=28%, signal=73% |
| 2739 | GOBP\_EPOXYGENASE\_P450\_PATHWAY |  | 3 | -0.73 | -1.22 | 0.190 | 0.522 | 1.000 | 25 | tags=33%, list=0%, signal=33% |
| 2740 | GOMF\_MITOGEN\_ACTIVATED\_PROTEIN\_KINASE\_KINASE\_BINDING |  | 8 | -0.55 | -1.22 | 0.218 | 0.522 | 1.000 | 3715 | tags=63%, list=31%, signal=90% |
| 2741 | GOBP\_RESPONSE\_TO\_INTERLEUKIN\_2 |  | 11 | -0.50 | -1.22 | 0.224 | 0.522 | 1.000 | 3692 | tags=55%, list=31%, signal=79% |
| 2742 | GOBP\_TRANSFORMING\_GROWTH\_FACTOR\_BETA\_RECEPTOR\_SIGNALING\_PATHWAY |  | 141 | -0.33 | -1.22 | 0.114 | 0.523 | 1.000 | 3570 | tags=38%, list=30%, signal=54% |
| 2743 | GOBP\_POSITIVE\_REGULATION\_OF\_CYTOKINESIS |  | 26 | -0.42 | -1.22 | 0.207 | 0.522 | 1.000 | 3330 | tags=38%, list=28%, signal=53% |
| 2744 | GOBP\_NEGATIVE\_REGULATION\_OF\_BIOSYNTHETIC\_PROCESS |  | 980 | -0.30 | -1.22 | 0.003 | 0.523 | 1.000 | 4362 | tags=42%, list=36%, signal=60% |
| 2745 | GOMF\_MHC\_CLASS\_II\_PROTEIN\_COMPLEX\_BINDING |  | 9 | -0.53 | -1.22 | 0.223 | 0.522 | 1.000 | 2010 | tags=33%, list=17%, signal=40% |
| 2746 | GOBP\_POSITIVE\_REGULATION\_OF\_CIRCADIAN\_RHYTHM |  | 10 | -0.52 | -1.22 | 0.230 | 0.523 | 1.000 | 5034 | tags=80%, list=42%, signal=138% |
| 2747 | GOMF\_INTERLEUKIN\_17\_RECEPTOR\_ACTIVITY |  | 4 | -0.66 | -1.22 | 0.207 | 0.523 | 1.000 | 824 | tags=50%, list=7%, signal=54% |
| 2748 | GOBP\_REGULATION\_OF\_NK\_T\_CELL\_ACTIVATION |  | 3 | -0.72 | -1.21 | 0.200 | 0.525 | 1.000 | 1566 | tags=67%, list=13%, signal=77% |
| 2749 | GOBP\_TERPENOID\_BIOSYNTHETIC\_PROCESS |  | 11 | -0.51 | -1.21 | 0.228 | 0.525 | 1.000 | 51 | tags=18%, list=0%, signal=18% |
| 2750 | GOMF\_LIGASE\_ACTIVITY\_FORMING\_CARBON\_SULFUR\_BONDS |  | 28 | -0.41 | -1.21 | 0.198 | 0.525 | 1.000 | 4053 | tags=43%, list=34%, signal=65% |
| 2751 | GOBP\_REGULATION\_OF\_REACTIVE\_OXYGEN\_SPECIES\_METABOLIC\_PROCESS |  | 129 | -0.33 | -1.21 | 0.128 | 0.525 | 1.000 | 2871 | tags=29%, list=24%, signal=37% |
| 2752 | GOBP\_ACTIN\_FILAMENT\_BASED\_MOVEMENT |  | 94 | -0.34 | -1.21 | 0.142 | 0.525 | 1.000 | 3336 | tags=32%, list=28%, signal=44% |
| 2753 | GOCC\_ENDODEOXYRIBONUCLEASE\_COMPLEX |  | 4 | -0.67 | -1.21 | 0.204 | 0.525 | 1.000 | 1830 | tags=50%, list=15%, signal=59% |
| 2754 | GOMF\_L\_PROLINE\_TRANSMEMBRANE\_TRANSPORTER\_ACTIVITY |  | 5 | -0.63 | -1.21 | 0.216 | 0.525 | 1.000 | 473 | tags=40%, list=4%, signal=42% |
| 2755 | GOBP\_ESTABLISHMENT\_OF\_PROTEIN\_LOCALIZATION\_TO\_TELOMERE |  | 16 | -0.46 | -1.21 | 0.220 | 0.525 | 1.000 | 1667 | tags=19%, list=14%, signal=22% |
| 2756 | GOBP\_OTOLITH\_DEVELOPMENT |  | 4 | -0.67 | -1.21 | 0.210 | 0.525 | 1.000 | 377 | tags=25%, list=3%, signal=26% |
| 2757 | GOBP\_STRIATED\_MUSCLE\_CELL\_PROLIFERATION |  | 43 | -0.38 | -1.21 | 0.183 | 0.525 | 1.000 | 3565 | tags=44%, list=30%, signal=63% |
| 2758 | GOBP\_REGULATION\_OF\_PROTEIN\_KINASE\_A\_SIGNALING |  | 15 | -0.47 | -1.21 | 0.223 | 0.525 | 1.000 | 4008 | tags=60%, list=33%, signal=90% |
| 2759 | GOBP\_ORGANOPHOSPHATE\_ESTER\_TRANSPORT |  | 87 | -0.34 | -1.21 | 0.156 | 0.525 | 1.000 | 3366 | tags=37%, list=28%, signal=51% |
| 2760 | GOBP\_NEGATIVE\_REGULATION\_OF\_ENDOPLASMIC\_RETICULUM\_UNFOLDED\_PROTEIN\_RESPONSE |  | 11 | -0.50 | -1.21 | 0.225 | 0.525 | 1.000 | 5279 | tags=73%, list=44%, signal=130% |
| 2761 | GOMF\_TUMOR\_NECROSIS\_FACTOR\_RECEPTOR\_BINDING |  | 21 | -0.43 | -1.21 | 0.218 | 0.525 | 1.000 | 1886 | tags=38%, list=16%, signal=45% |
| 2762 | GOBP\_NEGATIVE\_REGULATION\_OF\_HIGH\_VOLTAGE\_GATED\_CALCIUM\_CHANNEL\_ACTIVITY |  | 4 | -0.67 | -1.21 | 0.201 | 0.525 | 1.000 | 276 | tags=25%, list=2%, signal=26% |
| 2763 | GOBP\_CELL\_GROWTH |  | 320 | -0.31 | -1.21 | 0.047 | 0.525 | 1.000 | 3238 | tags=31%, list=27%, signal=41% |
| 2764 | GOBP\_ANIMAL\_ORGAN\_MATURATION |  | 17 | -0.45 | -1.21 | 0.214 | 0.526 | 1.000 | 2933 | tags=53%, list=24%, signal=70% |
| 2765 | GOBP\_REGULATION\_OF\_CARBOHYDRATE\_BIOSYNTHETIC\_PROCESS |  | 64 | -0.36 | -1.21 | 0.170 | 0.527 | 1.000 | 4276 | tags=47%, list=36%, signal=72% |
| 2766 | GOMF\_OXYGEN\_BINDING |  | 15 | -0.47 | -1.21 | 0.230 | 0.526 | 1.000 | 36 | tags=13%, list=0%, signal=13% |
| 2767 | GOBP\_REGULATION\_OF\_DNA\_DIRECTED\_DNA\_POLYMERASE\_ACTIVITY |  | 9 | -0.53 | -1.21 | 0.223 | 0.526 | 1.000 | 1030 | tags=22%, list=9%, signal=24% |
| 2768 | GOBP\_NEGATIVE\_REGULATION\_OF\_RESPONSE\_TO\_EXTERNAL\_STIMULUS |  | 210 | -0.32 | -1.21 | 0.088 | 0.527 | 1.000 | 2920 | tags=31%, list=24%, signal=40% |
| 2769 | GOCC\_CELL\_DIVISION\_SITE |  | 52 | -0.37 | -1.21 | 0.178 | 0.527 | 1.000 | 2995 | tags=31%, list=25%, signal=41% |
| 2770 | GOBP\_NEGATIVE\_REGULATION\_OF\_CD4\_POSITIVE\_ALPHA\_BETA\_T\_CELL\_ACTIVATION |  | 20 | -0.44 | -1.21 | 0.220 | 0.527 | 1.000 | 1951 | tags=35%, list=16%, signal=42% |
| 2771 | GOMF\_G\_PROTEIN\_COUPLED\_CHEMOATTRACTANT\_RECEPTOR\_ACTIVITY |  | 14 | -0.48 | -1.21 | 0.221 | 0.527 | 1.000 | 2871 | tags=57%, list=24%, signal=75% |
| 2772 | GOBP\_REGULATION\_OF\_HEPATOCYTE\_PROLIFERATION |  | 11 | -0.50 | -1.21 | 0.228 | 0.528 | 1.000 | 3937 | tags=64%, list=33%, signal=95% |
| 2773 | GOMF\_INHIBIN\_BINDING |  | 4 | -0.67 | -1.21 | 0.213 | 0.528 | 1.000 | 3657 | tags=75%, list=30%, signal=108% |
| 2774 | GOBP\_REGULATION\_OF\_CELL\_CYCLE\_G2\_M\_PHASE\_TRANSITION |  | 171 | -0.32 | -1.21 | 0.103 | 0.528 | 1.000 | 5426 | tags=49%, list=45%, signal=87% |
| 2775 | GOBP\_B\_CELL\_ACTIVATION\_INVOLVED\_IN\_IMMUNE\_RESPONSE |  | 57 | -0.36 | -1.21 | 0.172 | 0.528 | 1.000 | 3243 | tags=39%, list=27%, signal=53% |
| 2776 | GOBP\_MESENCHYME\_DEVELOPMENT |  | 202 | -0.32 | -1.21 | 0.090 | 0.528 | 1.000 | 3565 | tags=34%, list=30%, signal=48% |
| 2777 | GOBP\_REGULATION\_OF\_CARDIAC\_EPITHELIAL\_TO\_MESENCHYMAL\_TRANSITION |  | 8 | -0.55 | -1.21 | 0.222 | 0.529 | 1.000 | 112 | tags=13%, list=1%, signal=13% |
| 2778 | GOBP\_SKELETAL\_MUSCLE\_TISSUE\_GROWTH |  | 6 | -0.59 | -1.21 | 0.226 | 0.529 | 1.000 | 2503 | tags=33%, list=21%, signal=42% |
| 2779 | GOBP\_SIGNAL\_TRANSDUCTION\_INVOLVED\_IN\_CELL\_CYCLE\_CHECKPOINT |  | 57 | -0.36 | -1.21 | 0.174 | 0.529 | 1.000 | 3888 | tags=37%, list=32%, signal=54% |
| 2780 | GOCC\_HOLLIDAY\_JUNCTION\_RESOLVASE\_COMPLEX |  | 4 | -0.67 | -1.21 | 0.216 | 0.529 | 1.000 | 1830 | tags=50%, list=15%, signal=59% |
| 2781 | GOBP\_PHOSPHATE\_ION\_TRANSPORT |  | 19 | -0.44 | -1.21 | 0.219 | 0.529 | 1.000 | 2395 | tags=32%, list=20%, signal=39% |
| 2782 | GOBP\_NERVOUS\_SYSTEM\_PROCESS |  | 479 | -0.31 | -1.21 | 0.026 | 0.529 | 1.000 | 4188 | tags=41%, list=35%, signal=60% |
| 2783 | GOBP\_PHOSPHATIDYLETHANOLAMINE\_BIOSYNTHETIC\_PROCESS |  | 12 | -0.49 | -1.21 | 0.224 | 0.530 | 1.000 | 4166 | tags=67%, list=35%, signal=102% |
| 2784 | GOBP\_GLUTAMATE\_RECEPTOR\_SIGNALING\_PATHWAY |  | 32 | -0.40 | -1.21 | 0.204 | 0.530 | 1.000 | 4846 | tags=53%, list=40%, signal=89% |
| 2785 | GOBP\_REGULATION\_OF\_SENSORY\_PERCEPTION |  | 16 | -0.46 | -1.21 | 0.218 | 0.530 | 1.000 | 1771 | tags=38%, list=15%, signal=44% |
| 2786 | GOBP\_URINARY\_BLADDER\_SMOOTH\_MUSCLE\_CONTRACTION |  | 4 | -0.67 | -1.21 | 0.203 | 0.530 | 1.000 | 374 | tags=25%, list=3%, signal=26% |
| 2787 | GOBP\_MORPHOGENESIS\_OF\_AN\_EPITHELIUM |  | 366 | -0.31 | -1.21 | 0.043 | 0.530 | 1.000 | 3619 | tags=33%, list=30%, signal=45% |
| 2788 | GOBP\_REGULATION\_OF\_GLUCOSE\_IMPORT |  | 37 | -0.39 | -1.21 | 0.196 | 0.530 | 1.000 | 2590 | tags=32%, list=22%, signal=41% |
| 2789 | GOBP\_NEGATIVE\_REGULATION\_OF\_INTRACELLULAR\_STEROID\_HORMONE\_RECEPTOR\_SIGNALING\_PATHWAY |  | 28 | -0.41 | -1.21 | 0.211 | 0.530 | 1.000 | 2675 | tags=36%, list=22%, signal=46% |
| 2790 | GOBP\_POSITIVE\_REGULATION\_OF\_ARP2\_3\_COMPLEX\_MEDIATED\_ACTIN\_NUCLEATION |  | 8 | -0.55 | -1.21 | 0.222 | 0.530 | 1.000 | 3797 | tags=63%, list=32%, signal=91% |
| 2791 | GOCC\_DENDRITE\_TERMINUS |  | 11 | -0.50 | -1.21 | 0.230 | 0.530 | 1.000 | 2010 | tags=36%, list=17%, signal=44% |
| 2792 | GOCC\_INTRINSIC\_COMPONENT\_OF\_POSTSYNAPTIC\_DENSITY\_MEMBRANE |  | 21 | -0.43 | -1.21 | 0.218 | 0.530 | 1.000 | 1346 | tags=29%, list=11%, signal=32% |
| 2793 | GOBP\_GLYCOSPHINGOLIPID\_METABOLIC\_PROCESS |  | 53 | -0.36 | -1.21 | 0.181 | 0.530 | 1.000 | 3855 | tags=45%, list=32%, signal=66% |
| 2794 | GOBP\_LIPID\_STORAGE |  | 50 | -0.37 | -1.21 | 0.188 | 0.530 | 1.000 | 4103 | tags=52%, list=34%, signal=79% |
| 2795 | GOBP\_SENSORY\_PERCEPTION\_OF\_LIGHT\_STIMULUS |  | 85 | -0.34 | -1.21 | 0.151 | 0.530 | 1.000 | 3525 | tags=41%, list=29%, signal=58% |
| 2796 | GOBP\_POSITIVE\_REGULATION\_OF\_DENDRITE\_MORPHOGENESIS |  | 27 | -0.41 | -1.21 | 0.216 | 0.530 | 1.000 | 5358 | tags=56%, list=45%, signal=100% |
| 2797 | GOBP\_NEGATIVE\_REGULATION\_OF\_CYTOKINESIS |  | 6 | -0.59 | -1.21 | 0.222 | 0.531 | 1.000 | 1818 | tags=33%, list=15%, signal=39% |
| 2798 | GOBP\_PROTEIN\_METHYLATION |  | 120 | -0.33 | -1.21 | 0.134 | 0.531 | 1.000 | 3647 | tags=40%, list=30%, signal=57% |
| 2799 | GOBP\_POSITIVE\_REGULATION\_OF\_VASCULAR\_ASSOCIATED\_SMOOTH\_MUSCLE\_CELL\_DIFFERENTIATION |  | 5 | -0.63 | -1.21 | 0.207 | 0.530 | 1.000 | 199 | tags=20%, list=2%, signal=20% |
| 2800 | GOBP\_INNER\_EAR\_MORPHOGENESIS |  | 46 | -0.37 | -1.21 | 0.189 | 0.530 | 1.000 | 1660 | tags=24%, list=14%, signal=28% |
| 2801 | GOBP\_REGULATION\_OF\_SYNAPSE\_STRUCTURAL\_PLASTICITY |  | 6 | -0.60 | -1.21 | 0.226 | 0.530 | 1.000 | 2466 | tags=50%, list=21%, signal=63% |
| 2802 | GOBP\_MYELOID\_DENDRITIC\_CELL\_DIFFERENTIATION |  | 13 | -0.49 | -1.21 | 0.231 | 0.530 | 1.000 | 6186 | tags=100%, list=52%, signal=206% |
| 2803 | GOBP\_MYELOID\_LEUKOCYTE\_DIFFERENTIATION |  | 132 | -0.33 | -1.21 | 0.122 | 0.530 | 1.000 | 3692 | tags=42%, list=31%, signal=59% |
| 2804 | GOBP\_POSITIVE\_REGULATION\_OF\_NK\_T\_CELL\_ACTIVATION |  | 3 | -0.72 | -1.21 | 0.198 | 0.530 | 1.000 | 1566 | tags=67%, list=13%, signal=77% |
| 2805 | GOMF\_CO\_SMAD\_BINDING |  | 5 | -0.63 | -1.21 | 0.224 | 0.531 | 1.000 | 3306 | tags=60%, list=28%, signal=83% |
| 2806 | GOBP\_NEGATIVE\_REGULATION\_OF\_TOR\_SIGNALING |  | 33 | -0.39 | -1.21 | 0.200 | 0.531 | 1.000 | 3425 | tags=39%, list=29%, signal=55% |
| 2807 | GOBP\_POSITIVE\_REGULATION\_OF\_PROTEIN\_DEUBIQUITINATION |  | 6 | -0.59 | -1.21 | 0.223 | 0.532 | 1.000 | 4909 | tags=100%, list=41%, signal=169% |
| 2808 | GOBP\_HISTAMINE\_PRODUCTION\_INVOLVED\_IN\_INFLAMMATORY\_RESPONSE |  | 5 | -0.63 | -1.21 | 0.210 | 0.532 | 1.000 | 2943 | tags=40%, list=25%, signal=53% |
| 2809 | GOBP\_VESICLE\_MEDIATED\_TRANSPORT\_TO\_THE\_PLASMA\_MEMBRANE |  | 72 | -0.35 | -1.21 | 0.165 | 0.532 | 1.000 | 4110 | tags=43%, list=34%, signal=65% |
| 2810 | GOCC\_NMDA\_SELECTIVE\_GLUTAMATE\_RECEPTOR\_COMPLEX |  | 5 | -0.63 | -1.21 | 0.219 | 0.532 | 1.000 | 2005 | tags=60%, list=17%, signal=72% |
| 2811 | GOBP\_POST\_EMBRYONIC\_DEVELOPMENT |  | 50 | -0.37 | -1.21 | 0.181 | 0.532 | 1.000 | 4506 | tags=56%, list=38%, signal=89% |
| 2812 | GOBP\_REGULATION\_OF\_T\_CIRCLE\_FORMATION |  | 4 | -0.67 | -1.21 | 0.199 | 0.532 | 1.000 | 3927 | tags=100%, list=33%, signal=149% |
| 2813 | GOCC\_CALCITONIN\_FAMILY\_RECEPTOR\_COMPLEX |  | 4 | -0.67 | -1.21 | 0.207 | 0.533 | 1.000 | 3134 | tags=50%, list=26%, signal=68% |
| 2814 | GOMF\_PHOSPHATIDYLINOSITOL\_BINDING |  | 187 | -0.32 | -1.21 | 0.098 | 0.533 | 1.000 | 4097 | tags=39%, list=34%, signal=58% |
| 2815 | GOBP\_MACROPHAGE\_ACTIVATION |  | 60 | -0.36 | -1.21 | 0.171 | 0.533 | 1.000 | 4469 | tags=55%, list=37%, signal=87% |
| 2816 | GOBP\_SIGNAL\_TRANSDUCTION\_IN\_ABSENCE\_OF\_LIGAND |  | 44 | -0.37 | -1.21 | 0.191 | 0.533 | 1.000 | 1146 | tags=20%, list=10%, signal=23% |
| 2817 | GOBP\_POSITIVE\_REGULATION\_OF\_LYMPHOCYTE\_MIGRATION |  | 26 | -0.41 | -1.21 | 0.212 | 0.533 | 1.000 | 2011 | tags=35%, list=17%, signal=41% |
| 2818 | GOBP\_REGULATION\_OF\_T\_HELPER\_17\_TYPE\_IMMUNE\_RESPONSE |  | 8 | -0.55 | -1.21 | 0.234 | 0.533 | 1.000 | 1951 | tags=50%, list=16%, signal=60% |
| 2819 | GOBP\_CONVERGENT\_EXTENSION\_INVOLVED\_IN\_GASTRULATION |  | 6 | -0.59 | -1.21 | 0.220 | 0.533 | 1.000 | 1579 | tags=33%, list=13%, signal=38% |
| 2820 | GOBP\_SENSORY\_PERCEPTION |  | 234 | -0.32 | -1.21 | 0.079 | 0.533 | 1.000 | 1771 | tags=24%, list=15%, signal=28% |
| 2821 | GOBP\_POSITIVE\_REGULATION\_OF\_NITRIC\_OXIDE\_MEDIATED\_SIGNAL\_TRANSDUCTION |  | 3 | -0.72 | -1.21 | 0.201 | 0.533 | 1.000 | 1687 | tags=67%, list=14%, signal=78% |
| 2822 | GOBP\_LIPOXIN\_METABOLIC\_PROCESS |  | 4 | -0.67 | -1.21 | 0.207 | 0.533 | 1.000 | 263 | tags=50%, list=2%, signal=51% |
| 2823 | GOBP\_NEUROMUSCULAR\_JUNCTION\_DEVELOPMENT |  | 38 | -0.39 | -1.21 | 0.195 | 0.533 | 1.000 | 2466 | tags=32%, list=21%, signal=40% |
| 2824 | GOBP\_EMBRYONIC\_PLACENTA\_DEVELOPMENT |  | 55 | -0.36 | -1.21 | 0.184 | 0.533 | 1.000 | 4111 | tags=44%, list=34%, signal=66% |
| 2825 | GOBP\_GENE\_SILENCING |  | 150 | -0.32 | -1.21 | 0.115 | 0.533 | 1.000 | 4666 | tags=46%, list=39%, signal=74% |
| 2826 | GOBP\_PEPTIDYL\_L\_CYSTEINE\_S\_PALMITOYLATION |  | 15 | -0.46 | -1.21 | 0.235 | 0.535 | 1.000 | 2801 | tags=40%, list=23%, signal=52% |
| 2827 | GOBP\_REGULATION\_OF\_LYMPHOCYTE\_MEDIATED\_IMMUNITY |  | 74 | -0.35 | -1.21 | 0.170 | 0.535 | 1.000 | 3285 | tags=39%, list=27%, signal=54% |
| 2828 | GOBP\_LUNG\_MORPHOGENESIS |  | 35 | -0.39 | -1.21 | 0.203 | 0.534 | 1.000 | 3565 | tags=40%, list=30%, signal=57% |
| 2829 | GOMF\_CALCIUM\_DEPENDENT\_PROTEIN\_SERINE\_THREONINE\_PHOSPHATASE\_ACTIVITY |  | 6 | -0.60 | -1.21 | 0.225 | 0.535 | 1.000 | 4282 | tags=67%, list=36%, signal=104% |
| 2830 | GOBP\_REGULATION\_OF\_ACROSOME\_REACTION |  | 3 | -0.72 | -1.21 | 0.208 | 0.535 | 1.000 | 287 | tags=33%, list=2%, signal=34% |
| 2831 | GOBP\_REGULATION\_OF\_ACTIN\_FILAMENT\_ORGANIZATION |  | 198 | -0.32 | -1.21 | 0.097 | 0.535 | 1.000 | 3069 | tags=31%, list=26%, signal=41% |
| 2832 | GOBP\_NEGATIVE\_REGULATION\_OF\_LIPID\_TRANSPORT |  | 19 | -0.44 | -1.21 | 0.232 | 0.535 | 1.000 | 3613 | tags=58%, list=30%, signal=83% |
| 2833 | GOBP\_REGULATION\_OF\_TRAIL\_ACTIVATED\_APOPTOTIC\_SIGNALING\_PATHWAY |  | 4 | -0.66 | -1.21 | 0.221 | 0.535 | 1.000 | 3841 | tags=75%, list=32%, signal=110% |
| 2834 | GOBP\_INTERLEUKIN\_4\_PRODUCTION |  | 19 | -0.44 | -1.21 | 0.225 | 0.536 | 1.000 | 3155 | tags=47%, list=26%, signal=64% |
| 2835 | GOBP\_DIAPHRAGM\_DEVELOPMENT |  | 6 | -0.59 | -1.21 | 0.225 | 0.536 | 1.000 | 1125 | tags=33%, list=9%, signal=37% |
| 2836 | GOBP\_MALE\_MATING\_BEHAVIOR |  | 6 | -0.60 | -1.21 | 0.230 | 0.536 | 1.000 | 4521 | tags=83%, list=38%, signal=134% |
| 2837 | GOBP\_POSITIVE\_REGULATION\_OF\_MYELOID\_CELL\_APOPTOTIC\_PROCESS |  | 7 | -0.57 | -1.21 | 0.229 | 0.536 | 1.000 | 2656 | tags=71%, list=22%, signal=92% |
| 2838 | GOBP\_ASTRAL\_MICROTUBULE\_ORGANIZATION |  | 10 | -0.51 | -1.21 | 0.235 | 0.536 | 1.000 | 2604 | tags=40%, list=22%, signal=51% |
| 2839 | GOBP\_POSITIVE\_REGULATION\_OF\_PEPTIDE\_HORMONE\_SECRETION |  | 59 | -0.36 | -1.20 | 0.183 | 0.536 | 1.000 | 3147 | tags=39%, list=26%, signal=53% |
| 2840 | GOBP\_REGULATION\_OF\_PROTEIN\_KINASE\_C\_SIGNALING |  | 8 | -0.54 | -1.20 | 0.241 | 0.536 | 1.000 | 3609 | tags=50%, list=30%, signal=71% |
| 2841 | GOBP\_MIDBRAIN\_DOPAMINERGIC\_NEURON\_DIFFERENTIATION |  | 12 | -0.49 | -1.20 | 0.229 | 0.536 | 1.000 | 2811 | tags=33%, list=23%, signal=43% |
| 2842 | GOBP\_NITRIC\_OXIDE\_SYNTHASE\_BIOSYNTHETIC\_PROCESS |  | 11 | -0.50 | -1.20 | 0.242 | 0.536 | 1.000 | 3609 | tags=55%, list=30%, signal=78% |
| 2843 | GOBP\_MITOTIC\_G2\_M\_TRANSITION\_CHECKPOINT |  | 32 | -0.39 | -1.20 | 0.208 | 0.536 | 1.000 | 4076 | tags=44%, list=34%, signal=66% |
| 2844 | GOMF\_MICROTUBULE\_PLUS\_END\_BINDING |  | 18 | -0.44 | -1.20 | 0.222 | 0.536 | 1.000 | 5586 | tags=67%, list=47%, signal=124% |
| 2845 | GOBP\_CELLULAR\_CARBOHYDRATE\_BIOSYNTHETIC\_PROCESS |  | 59 | -0.36 | -1.20 | 0.182 | 0.536 | 1.000 | 4276 | tags=51%, list=36%, signal=79% |
| 2846 | GOBP\_PROTEIN\_LOCALIZATION\_TO\_POSTSYNAPSE |  | 26 | -0.41 | -1.20 | 0.221 | 0.536 | 1.000 | 4655 | tags=62%, list=39%, signal=100% |
| 2847 | GOBP\_POSITIVE\_REGULATION\_OF\_SECONDARY\_METABOLITE\_BIOSYNTHETIC\_PROCESS |  | 4 | -0.67 | -1.20 | 0.214 | 0.536 | 1.000 | 1898 | tags=50%, list=16%, signal=59% |
| 2848 | GOMF\_ARACHIDONATE\_COA\_LIGASE\_ACTIVITY |  | 10 | -0.51 | -1.20 | 0.229 | 0.536 | 1.000 | 4053 | tags=60%, list=34%, signal=90% |
| 2849 | GOBP\_NEGATIVE\_REGULATION\_OF\_TELOMERE\_MAINTENANCE\_VIA\_TELOMERASE |  | 15 | -0.47 | -1.20 | 0.232 | 0.537 | 1.000 | 4219 | tags=53%, list=35%, signal=82% |
| 2850 | GOBP\_REGULATION\_OF\_SKELETAL\_MUSCLE\_TISSUE\_GROWTH |  | 6 | -0.59 | -1.20 | 0.230 | 0.537 | 1.000 | 2503 | tags=33%, list=21%, signal=42% |
| 2851 | GOBP\_NEGATIVE\_REGULATION\_OF\_LOCOMOTION |  | 221 | -0.31 | -1.20 | 0.090 | 0.537 | 1.000 | 3728 | tags=37%, list=31%, signal=52% |
| 2852 | GOCC\_MITOTIC\_SPINDLE\_POLE |  | 28 | -0.40 | -1.20 | 0.213 | 0.537 | 1.000 | 1865 | tags=29%, list=16%, signal=34% |
| 2853 | GOBP\_GOLGI\_LOCALIZATION |  | 13 | -0.48 | -1.20 | 0.235 | 0.537 | 1.000 | 5151 | tags=62%, list=43%, signal=108% |
| 2854 | GOBP\_ASTROCYTE\_DEVELOPMENT |  | 29 | -0.40 | -1.20 | 0.213 | 0.537 | 1.000 | 1230 | tags=21%, list=10%, signal=23% |
| 2855 | GOBP\_NEGATIVE\_REGULATION\_OF\_TRANSCRIPTION\_BY\_COMPETITIVE\_PROMOTER\_BINDING |  | 8 | -0.54 | -1.20 | 0.236 | 0.537 | 1.000 | 2290 | tags=50%, list=19%, signal=62% |
| 2856 | GOCC\_ENDORIBONUCLEASE\_COMPLEX |  | 18 | -0.44 | -1.20 | 0.230 | 0.537 | 1.000 | 2127 | tags=33%, list=18%, signal=40% |
| 2857 | GOBP\_REGULATION\_OF\_MUSCLE\_ADAPTATION |  | 57 | -0.36 | -1.20 | 0.178 | 0.537 | 1.000 | 3444 | tags=40%, list=29%, signal=56% |
| 2858 | GOCC\_CD95\_DEATH\_INDUCING\_SIGNALING\_COMPLEX |  | 5 | -0.63 | -1.20 | 0.218 | 0.538 | 1.000 | 2494 | tags=80%, list=21%, signal=101% |
| 2859 | GOBP\_NEGATIVE\_REGULATION\_OF\_CYTOKINE\_PRODUCTION\_INVOLVED\_IN\_INFLAMMATORY\_RESPONSE |  | 13 | -0.48 | -1.20 | 0.240 | 0.538 | 1.000 | 3968 | tags=54%, list=33%, signal=80% |
| 2860 | GOBP\_GLUTATHIONE\_CATABOLIC\_PROCESS |  | 6 | -0.59 | -1.20 | 0.226 | 0.538 | 1.000 | 1878 | tags=50%, list=16%, signal=59% |
| 2861 | GOBP\_TRANS\_SYNAPTIC\_SIGNALING\_BY\_LIPID |  | 6 | -0.59 | -1.20 | 0.233 | 0.539 | 1.000 | 4907 | tags=100%, list=41%, signal=169% |
| 2862 | GOBP\_NEGATIVE\_REGULATION\_OF\_MEMBRANE\_PERMEABILITY |  | 5 | -0.62 | -1.20 | 0.224 | 0.539 | 1.000 | 3843 | tags=80%, list=32%, signal=118% |
| 2863 | GOCC\_MCM\_COMPLEX |  | 9 | -0.53 | -1.20 | 0.238 | 0.539 | 1.000 | 5632 | tags=100%, list=47%, signal=188% |
| 2864 | GOBP\_POSITIVE\_REGULATION\_OF\_PROTEIN\_DEACETYLATION |  | 12 | -0.49 | -1.20 | 0.241 | 0.539 | 1.000 | 3640 | tags=58%, list=30%, signal=84% |
| 2865 | GOCC\_NODE\_OF\_RANVIER |  | 10 | -0.51 | -1.20 | 0.236 | 0.539 | 1.000 | 5745 | tags=80%, list=48%, signal=153% |
| 2866 | GOBP\_COMPLEMENT\_ACTIVATION |  | 36 | -0.39 | -1.20 | 0.204 | 0.539 | 1.000 | 2600 | tags=33%, list=22%, signal=42% |
| 2867 | GOBP\_REGULATION\_OF\_VITAMIN\_D\_RECEPTOR\_SIGNALING\_PATHWAY |  | 5 | -0.62 | -1.20 | 0.223 | 0.539 | 1.000 | 4358 | tags=80%, list=36%, signal=126% |
| 2868 | GOBP\_REGULATION\_OF\_CELL\_AGING |  | 40 | -0.38 | -1.20 | 0.201 | 0.539 | 1.000 | 3668 | tags=40%, list=31%, signal=57% |
| 2869 | GOBP\_ADAPTIVE\_IMMUNE\_RESPONSE\_BASED\_ON\_SOMATIC\_RECOMBINATION\_OF\_IMMUNE\_RECEPTORS\_BUILT\_FROM\_IMMUNOGLOBULIN\_SUPERFAMILY\_DOMAINS |  | 148 | -0.32 | -1.20 | 0.128 | 0.539 | 1.000 | 3240 | tags=36%, list=27%, signal=49% |
| 2870 | GOBP\_REGULATION\_OF\_ION\_TRANSPORT |  | 832 | -0.30 | -1.20 | 0.009 | 0.540 | 1.000 | 3072 | tags=29%, list=26%, signal=36% |
| 2871 | GOBP\_CELL\_CELL\_JUNCTION\_ASSEMBLY |  | 97 | -0.33 | -1.20 | 0.156 | 0.540 | 1.000 | 3552 | tags=39%, list=30%, signal=55% |
| 2872 | GOBP\_CARDIAC\_VASCULAR\_SMOOTH\_MUSCLE\_CELL\_DIFFERENTIATION |  | 6 | -0.60 | -1.20 | 0.225 | 0.540 | 1.000 | 3314 | tags=50%, list=28%, signal=69% |
| 2873 | GOBP\_MONOCYTE\_CHEMOTAXIS |  | 34 | -0.39 | -1.20 | 0.211 | 0.541 | 1.000 | 2769 | tags=38%, list=23%, signal=50% |
| 2874 | GOCC\_NUCLEAR\_BODY |  | 585 | -0.30 | -1.20 | 0.018 | 0.540 | 1.000 | 4704 | tags=44%, list=39%, signal=68% |
| 2875 | GOBP\_NEGATIVE\_REGULATION\_OF\_FIBROBLAST\_APOPTOTIC\_PROCESS |  | 6 | -0.59 | -1.20 | 0.235 | 0.540 | 1.000 | 3534 | tags=50%, list=29%, signal=71% |
| 2876 | GOMF\_NEUROTROPHIN\_BINDING |  | 8 | -0.54 | -1.20 | 0.239 | 0.540 | 1.000 | 23 | tags=13%, list=0%, signal=13% |
| 2877 | GOBP\_UV\_DAMAGE\_EXCISION\_REPAIR |  | 9 | -0.53 | -1.20 | 0.251 | 0.541 | 1.000 | 3199 | tags=56%, list=27%, signal=76% |
| 2878 | GOBP\_CELLULAR\_GLUCAN\_METABOLIC\_PROCESS |  | 52 | -0.36 | -1.20 | 0.190 | 0.541 | 1.000 | 4931 | tags=56%, list=41%, signal=94% |
| 2879 | GOBP\_LIPOPROTEIN\_LOCALIZATION |  | 12 | -0.48 | -1.20 | 0.239 | 0.541 | 1.000 | 3217 | tags=67%, list=27%, signal=91% |
| 2880 | GOBP\_REGULATION\_OF\_APOPTOTIC\_SIGNALING\_PATHWAY |  | 234 | -0.31 | -1.20 | 0.082 | 0.541 | 1.000 | 3483 | tags=36%, list=29%, signal=50% |
| 2881 | GOBP\_RESPONSE\_TO\_MACROPHAGE\_COLONY\_STIMULATING\_FACTOR |  | 9 | -0.52 | -1.20 | 0.236 | 0.542 | 1.000 | 3695 | tags=56%, list=31%, signal=80% |
| 2882 | GOBP\_STRIATED\_MUSCLE\_CONTRACTION |  | 95 | -0.33 | -1.20 | 0.164 | 0.542 | 1.000 | 1890 | tags=22%, list=16%, signal=26% |
| 2883 | GOBP\_POSITIVE\_REGULATION\_OF\_TORC1\_SIGNALING |  | 12 | -0.49 | -1.20 | 0.241 | 0.542 | 1.000 | 5457 | tags=58%, list=45%, signal=107% |
| 2884 | GOBP\_SIGNAL\_TRANSDUCTION\_INVOLVED\_IN\_G2\_DNA\_DAMAGE\_CHECKPOINT |  | 11 | -0.50 | -1.20 | 0.231 | 0.542 | 1.000 | 1964 | tags=27%, list=16%, signal=33% |
| 2885 | GOCC\_DNA\_POLYMERASE\_COMPLEX |  | 17 | -0.45 | -1.20 | 0.238 | 0.542 | 1.000 | 3402 | tags=47%, list=28%, signal=66% |
| 2886 | GOBP\_LEARNED\_VOCALIZATION\_BEHAVIOR\_OR\_VOCAL\_LEARNING |  | 3 | -0.71 | -1.20 | 0.206 | 0.542 | 1.000 | 3431 | tags=100%, list=29%, signal=140% |
| 2887 | GOBP\_PROTEIN\_O\_LINKED\_GLYCOSYLATION\_VIA\_SERINE |  | 6 | -0.59 | -1.20 | 0.238 | 0.542 | 1.000 | 4702 | tags=83%, list=39%, signal=137% |
| 2888 | GOCC\_CYTOPLASMIC\_SIDE\_OF\_MEMBRANE |  | 143 | -0.32 | -1.20 | 0.128 | 0.542 | 1.000 | 2604 | tags=31%, list=22%, signal=40% |
| 2889 | GOBP\_REGULATION\_OF\_VESICLE\_FUSION |  | 15 | -0.46 | -1.20 | 0.239 | 0.542 | 1.000 | 207 | tags=20%, list=2%, signal=20% |
| 2890 | GOBP\_VASCULAR\_ASSOCIATED\_SMOOTH\_MUSCLE\_CONTRACTION |  | 23 | -0.42 | -1.20 | 0.223 | 0.542 | 1.000 | 925 | tags=22%, list=8%, signal=24% |
| 2891 | GOBP\_CALCIUM\_ION\_TRANSPORT |  | 247 | -0.31 | -1.20 | 0.083 | 0.542 | 1.000 | 4173 | tags=38%, list=35%, signal=57% |
| 2892 | GOBP\_OSSIFICATION |  | 261 | -0.31 | -1.20 | 0.078 | 0.542 | 1.000 | 3389 | tags=33%, list=28%, signal=45% |
| 2893 | GOBP\_KERATAN\_SULFATE\_CATABOLIC\_PROCESS |  | 8 | -0.54 | -1.20 | 0.247 | 0.542 | 1.000 | 3178 | tags=38%, list=26%, signal=51% |
| 2894 | GOMF\_CELL\_ADHESIVE\_PROTEIN\_BINDING\_INVOLVED\_IN\_BUNDLE\_OF\_HIS\_CELL\_PURKINJE\_MYOCYTE\_COMMUNICATION |  | 4 | -0.66 | -1.20 | 0.224 | 0.542 | 1.000 | 3918 | tags=75%, list=33%, signal=111% |
| 2895 | GOMF\_NMDA\_GLUTAMATE\_RECEPTOR\_ACTIVITY |  | 5 | -0.63 | -1.20 | 0.225 | 0.542 | 1.000 | 2005 | tags=60%, list=17%, signal=72% |
| 2896 | GOBP\_CELL\_FATE\_DETERMINATION |  | 23 | -0.42 | -1.20 | 0.226 | 0.542 | 1.000 | 2474 | tags=35%, list=21%, signal=44% |
| 2897 | GOBP\_RHOMBOMERE\_DEVELOPMENT |  | 4 | -0.66 | -1.20 | 0.220 | 0.542 | 1.000 | 733 | tags=50%, list=6%, signal=53% |
| 2898 | GOBP\_NEGATIVE\_REGULATION\_OF\_VESICLE\_FUSION |  | 4 | -0.67 | -1.20 | 0.231 | 0.542 | 1.000 | 1537 | tags=50%, list=13%, signal=57% |
| 2899 | GOBP\_POSITIVE\_REGULATION\_OF\_MYOTUBE\_DIFFERENTIATION |  | 5 | -0.62 | -1.20 | 0.224 | 0.542 | 1.000 | 1743 | tags=60%, list=15%, signal=70% |
| 2900 | GOBP\_REGULATION\_OF\_GLUCOCORTICOID\_METABOLIC\_PROCESS |  | 9 | -0.53 | -1.20 | 0.242 | 0.542 | 1.000 | 1899 | tags=44%, list=16%, signal=53% |
| 2901 | GOBP\_CALCIUM\_ION\_REGULATED\_EXOCYTOSIS |  | 44 | -0.37 | -1.20 | 0.207 | 0.542 | 1.000 | 2055 | tags=27%, list=17%, signal=33% |
| 2902 | GOCC\_NUCLEAR\_SPECK |  | 305 | -0.31 | -1.20 | 0.067 | 0.542 | 1.000 | 4799 | tags=44%, list=40%, signal=71% |
| 2903 | GOBP\_PULMONARY\_VALVE\_MORPHOGENESIS |  | 14 | -0.47 | -1.20 | 0.230 | 0.542 | 1.000 | 3561 | tags=43%, list=30%, signal=61% |
| 2904 | GOBP\_EMBRYONIC\_DIGESTIVE\_TRACT\_DEVELOPMENT |  | 21 | -0.43 | -1.20 | 0.232 | 0.542 | 1.000 | 1939 | tags=24%, list=16%, signal=28% |
| 2905 | GOCC\_HETEROCHROMATIN |  | 51 | -0.36 | -1.20 | 0.193 | 0.542 | 1.000 | 3744 | tags=45%, list=31%, signal=65% |
| 2906 | GOMF\_AMYLOID\_BETA\_BINDING |  | 56 | -0.36 | -1.20 | 0.184 | 0.542 | 1.000 | 2218 | tags=30%, list=18%, signal=37% |
| 2907 | GOBP\_CELLULAR\_RESPONSE\_TO\_EXTRACELLULAR\_STIMULUS |  | 178 | -0.32 | -1.20 | 0.114 | 0.542 | 1.000 | 4561 | tags=44%, list=38%, signal=70% |
| 2908 | GOMF\_G\_PROTEIN\_ALPHA\_SUBUNIT\_BINDING |  | 18 | -0.44 | -1.20 | 0.233 | 0.542 | 1.000 | 1544 | tags=28%, list=13%, signal=32% |
| 2909 | GOBP\_REGULATION\_OF\_ANION\_TRANSPORT |  | 577 | -0.30 | -1.20 | 0.025 | 0.542 | 1.000 | 2784 | tags=28%, list=23%, signal=34% |
| 2910 | GOBP\_MACROPHAGE\_MIGRATION |  | 35 | -0.38 | -1.20 | 0.211 | 0.542 | 1.000 | 3921 | tags=49%, list=33%, signal=72% |
| 2911 | GOMF\_DEOXYRIBONUCLEASE\_ACTIVITY |  | 35 | -0.39 | -1.20 | 0.211 | 0.542 | 1.000 | 3298 | tags=40%, list=27%, signal=55% |
| 2912 | GOBP\_ACTIVATION\_OF\_STORE\_OPERATED\_CALCIUM\_CHANNEL\_ACTIVITY |  | 3 | -0.72 | -1.20 | 0.221 | 0.542 | 1.000 | 1315 | tags=33%, list=11%, signal=37% |
| 2913 | GOBP\_CHROMATIN\_REMODELING |  | 139 | -0.32 | -1.20 | 0.131 | 0.543 | 1.000 | 3900 | tags=42%, list=32%, signal=62% |
| 2914 | GOMF\_RNA\_POLYMERASE\_BINDING |  | 50 | -0.36 | -1.20 | 0.195 | 0.543 | 1.000 | 5518 | tags=62%, list=46%, signal=114% |
| 2915 | GOBP\_GOLGI\_TO\_PLASMA\_MEMBRANE\_PROTEIN\_TRANSPORT |  | 33 | -0.39 | -1.20 | 0.216 | 0.543 | 1.000 | 4275 | tags=48%, list=36%, signal=75% |
| 2916 | GOBP\_NEGATIVE\_REGULATION\_OF\_LIPOPROTEIN\_LIPASE\_ACTIVITY |  | 3 | -0.72 | -1.20 | 0.219 | 0.543 | 1.000 | 2747 | tags=67%, list=23%, signal=86% |
| 2917 | GOBP\_GERMINAL\_CENTER\_FORMATION |  | 11 | -0.50 | -1.20 | 0.245 | 0.543 | 1.000 | 2474 | tags=45%, list=21%, signal=57% |
| 2918 | GOMF\_CYSTEINE\_TYPE\_ENDOPEPTIDASE\_REGULATOR\_ACTIVITY\_INVOLVED\_IN\_APOPTOTIC\_PROCESS |  | 22 | -0.42 | -1.20 | 0.224 | 0.543 | 1.000 | 2126 | tags=18%, list=18%, signal=22% |
| 2919 | GOBP\_BUNDLE\_OF\_HIS\_CELL\_TO\_PURKINJE\_MYOCYTE\_SIGNALING |  | 3 | -0.72 | -1.20 | 0.218 | 0.543 | 1.000 | 3295 | tags=67%, list=27%, signal=92% |
| 2920 | GOBP\_SYNAPTIC\_VESICLE\_EXOCYTOSIS |  | 73 | -0.34 | -1.20 | 0.181 | 0.543 | 1.000 | 3946 | tags=47%, list=33%, signal=69% |
| 2921 | GOBP\_BRANCHING\_MORPHOGENESIS\_OF\_AN\_EPITHELIAL\_TUBE |  | 101 | -0.33 | -1.20 | 0.152 | 0.543 | 1.000 | 4341 | tags=44%, list=36%, signal=68% |
| 2922 | GOBP\_REGULATION\_OF\_SYNAPTIC\_VESICLE\_EXOCYTOSIS |  | 42 | -0.37 | -1.20 | 0.215 | 0.543 | 1.000 | 5004 | tags=60%, list=42%, signal=102% |
| 2923 | GOBP\_CELLULAR\_RESPONSE\_TO\_EXTERNAL\_STIMULUS |  | 230 | -0.31 | -1.20 | 0.093 | 0.543 | 1.000 | 3701 | tags=36%, list=31%, signal=51% |
| 2924 | GOCC\_SPERM\_HEAD |  | 5 | -0.62 | -1.20 | 0.227 | 0.543 | 1.000 | 4402 | tags=80%, list=37%, signal=126% |
| 2925 | GOBP\_ACTIN\_FILAMENT\_DEPOLYMERIZATION |  | 45 | -0.37 | -1.20 | 0.200 | 0.542 | 1.000 | 2869 | tags=29%, list=24%, signal=38% |
| 2926 | GOMF\_HISTONE\_DEMETHYLASE\_ACTIVITY\_H3\_K4\_SPECIFIC |  | 4 | -0.66 | -1.20 | 0.220 | 0.542 | 1.000 | 1725 | tags=50%, list=14%, signal=58% |
| 2927 | GOBP\_CELLULAR\_WATER\_HOMEOSTASIS |  | 5 | -0.62 | -1.20 | 0.237 | 0.543 | 1.000 | 1939 | tags=60%, list=16%, signal=72% |
| 2928 | GOBP\_REGULATION\_OF\_TOLL\_LIKE\_RECEPTOR\_SIGNALING\_PATHWAY |  | 45 | -0.37 | -1.20 | 0.210 | 0.543 | 1.000 | 2731 | tags=38%, list=23%, signal=49% |
| 2929 | GOBP\_POSITIVE\_REGULATION\_OF\_DEFENSE\_RESPONSE |  | 201 | -0.31 | -1.20 | 0.110 | 0.543 | 1.000 | 3285 | tags=33%, list=27%, signal=45% |
| 2930 | GOCC\_TRANSPORT\_VESICLE\_MEMBRANE |  | 126 | -0.33 | -1.20 | 0.142 | 0.543 | 1.000 | 4372 | tags=44%, list=36%, signal=68% |
| 2931 | GOCC\_CILIARY\_BASE |  | 20 | -0.43 | -1.20 | 0.229 | 0.543 | 1.000 | 556 | tags=15%, list=5%, signal=16% |
| 2932 | GOBP\_PRODUCTION\_OF\_MOLECULAR\_MEDIATOR\_INVOLVED\_IN\_INFLAMMATORY\_RESPONSE |  | 42 | -0.37 | -1.20 | 0.206 | 0.543 | 1.000 | 1236 | tags=24%, list=10%, signal=26% |
| 2933 | GOMF\_FRIZZLED\_BINDING |  | 20 | -0.43 | -1.20 | 0.238 | 0.543 | 1.000 | 1579 | tags=25%, list=13%, signal=29% |
| 2934 | GOCC\_COLLAGEN\_CONTAINING\_EXTRACELLULAR\_MATRIX |  | 253 | -0.31 | -1.20 | 0.090 | 0.544 | 1.000 | 3186 | tags=30%, list=27%, signal=40% |
| 2935 | GOBP\_NEGATIVE\_REGULATION\_OF\_IMMUNE\_EFFECTOR\_PROCESS |  | 68 | -0.35 | -1.20 | 0.180 | 0.544 | 1.000 | 3031 | tags=37%, list=25%, signal=49% |
| 2936 | GOBP\_POSITIVE\_REGULATION\_OF\_NLRP3\_INFLAMMASOME\_COMPLEX\_ASSEMBLY |  | 4 | -0.66 | -1.20 | 0.225 | 0.544 | 1.000 | 4060 | tags=100%, list=34%, signal=151% |
| 2937 | GOBP\_MUSCLE\_SYSTEM\_PROCESS |  | 276 | -0.31 | -1.20 | 0.079 | 0.544 | 1.000 | 2494 | tags=24%, list=21%, signal=30% |
| 2938 | GOBP\_G\_QUADRUPLEX\_DNA\_UNWINDING |  | 6 | -0.59 | -1.20 | 0.241 | 0.544 | 1.000 | 4922 | tags=100%, list=41%, signal=169% |
| 2939 | GOCC\_TERTIARY\_GRANULE\_MEMBRANE |  | 41 | -0.37 | -1.20 | 0.208 | 0.544 | 1.000 | 1083 | tags=22%, list=9%, signal=24% |
| 2940 | GOBP\_NEURAL\_TUBE\_DEVELOPMENT |  | 106 | -0.33 | -1.20 | 0.145 | 0.544 | 1.000 | 4242 | tags=41%, list=35%, signal=62% |
| 2941 | GOMF\_TRANSMEMBRANE\_RECEPTOR\_PROTEIN\_PHOSPHATASE\_ACTIVITY |  | 16 | -0.45 | -1.20 | 0.250 | 0.544 | 1.000 | 2249 | tags=38%, list=19%, signal=46% |
| 2942 | GOBP\_VASCULAR\_TRANSPORT |  | 63 | -0.35 | -1.20 | 0.190 | 0.544 | 1.000 | 2557 | tags=35%, list=21%, signal=44% |
| 2943 | GOCC\_PRESYNAPTIC\_ACTIVE\_ZONE\_MEMBRANE |  | 18 | -0.44 | -1.20 | 0.250 | 0.544 | 1.000 | 4548 | tags=50%, list=38%, signal=80% |
| 2944 | GOBP\_MUSCLE\_ADAPTATION |  | 71 | -0.35 | -1.20 | 0.189 | 0.544 | 1.000 | 3444 | tags=39%, list=29%, signal=55% |
| 2945 | GOBP\_POSITIVE\_REGULATION\_OF\_ACTIN\_FILAMENT\_BUNDLE\_ASSEMBLY |  | 52 | -0.36 | -1.20 | 0.196 | 0.544 | 1.000 | 2560 | tags=29%, list=21%, signal=37% |
| 2946 | GOBP\_POSITIVE\_REGULATION\_OF\_HISTONE\_H3\_K9\_METHYLATION |  | 5 | -0.62 | -1.20 | 0.234 | 0.544 | 1.000 | 2656 | tags=60%, list=22%, signal=77% |
| 2947 | GOBP\_POSITIVE\_REGULATION\_OF\_TRANSLATION\_IN\_RESPONSE\_TO\_ENDOPLASMIC\_RETICULUM\_STRESS |  | 4 | -0.66 | -1.20 | 0.234 | 0.544 | 1.000 | 3691 | tags=75%, list=31%, signal=108% |
| 2948 | GOBP\_GLOMERULUS\_MORPHOGENESIS |  | 8 | -0.54 | -1.20 | 0.245 | 0.544 | 1.000 | 3286 | tags=50%, list=27%, signal=69% |
| 2949 | GOCC\_P\_BODY |  | 68 | -0.35 | -1.20 | 0.192 | 0.544 | 1.000 | 3063 | tags=32%, list=26%, signal=43% |
| 2950 | GOBP\_POSITIVE\_REGULATION\_OF\_CATECHOLAMINE\_SECRETION |  | 9 | -0.52 | -1.20 | 0.249 | 0.544 | 1.000 | 1699 | tags=33%, list=14%, signal=39% |
| 2951 | GOBP\_NEUTRAL\_LIPID\_BIOSYNTHETIC\_PROCESS |  | 34 | -0.39 | -1.19 | 0.223 | 0.544 | 1.000 | 2667 | tags=32%, list=22%, signal=41% |
| 2952 | GOCC\_LIPID\_DROPLET |  | 66 | -0.35 | -1.19 | 0.191 | 0.544 | 1.000 | 3454 | tags=33%, list=29%, signal=47% |
| 2953 | GOMF\_GLYCOLIPID\_BINDING |  | 17 | -0.45 | -1.19 | 0.229 | 0.544 | 1.000 | 2201 | tags=35%, list=18%, signal=43% |
| 2954 | GOMF\_PROTEIN\_SERINE\_THREONINE\_KINASE\_ACTIVATOR\_ACTIVITY |  | 30 | -0.40 | -1.19 | 0.219 | 0.544 | 1.000 | 3408 | tags=37%, list=28%, signal=51% |
| 2955 | GOBP\_ERYTHROCYTE\_MATURATION |  | 9 | -0.52 | -1.19 | 0.240 | 0.544 | 1.000 | 804 | tags=22%, list=7%, signal=24% |
| 2956 | GOBP\_NEURAL\_CREST\_CELL\_MIGRATION |  | 42 | -0.37 | -1.19 | 0.208 | 0.544 | 1.000 | 1013 | tags=17%, list=8%, signal=18% |
| 2957 | GOBP\_NEGATIVE\_REGULATION\_OF\_NEUROTRANSMITTER\_SECRETION |  | 8 | -0.54 | -1.19 | 0.241 | 0.544 | 1.000 | 2087 | tags=25%, list=17%, signal=30% |
| 2958 | GOBP\_MITOTIC\_G1\_S\_TRANSITION\_CHECKPOINT |  | 48 | -0.36 | -1.19 | 0.207 | 0.544 | 1.000 | 2982 | tags=33%, list=25%, signal=44% |
| 2959 | GOBP\_MIDDLE\_EAR\_MORPHOGENESIS |  | 9 | -0.53 | -1.19 | 0.252 | 0.544 | 1.000 | 1393 | tags=33%, list=12%, signal=38% |
| 2960 | GOBP\_HISTONE\_H3\_K4\_DEMETHYLATION |  | 5 | -0.62 | -1.19 | 0.239 | 0.545 | 1.000 | 1725 | tags=40%, list=14%, signal=47% |
| 2961 | GOBP\_PROTEIN\_LOCALIZATION\_TO\_MICROTUBULE |  | 13 | -0.47 | -1.19 | 0.250 | 0.545 | 1.000 | 1337 | tags=23%, list=11%, signal=26% |
| 2962 | GOBP\_MYD88\_DEPENDENT\_TOLL\_LIKE\_RECEPTOR\_SIGNALING\_PATHWAY |  | 23 | -0.42 | -1.19 | 0.244 | 0.546 | 1.000 | 2322 | tags=35%, list=19%, signal=43% |
| 2963 | GOBP\_EPITHELIAL\_CELL\_PROLIFERATION |  | 274 | -0.31 | -1.19 | 0.083 | 0.546 | 1.000 | 2886 | tags=30%, list=24%, signal=39% |
| 2964 | GOBP\_EPITHELIAL\_CELL\_CELL\_ADHESION |  | 14 | -0.46 | -1.19 | 0.249 | 0.546 | 1.000 | 3158 | tags=50%, list=26%, signal=68% |
| 2965 | GOMF\_SMAD\_BINDING |  | 60 | -0.35 | -1.19 | 0.193 | 0.546 | 1.000 | 3657 | tags=43%, list=30%, signal=62% |
| 2966 | GOBP\_FORELIMB\_MORPHOGENESIS |  | 18 | -0.44 | -1.19 | 0.241 | 0.546 | 1.000 | 852 | tags=22%, list=7%, signal=24% |
| 2967 | GOBP\_REGULATION\_OF\_CENTROMERE\_COMPLEX\_ASSEMBLY |  | 4 | -0.66 | -1.19 | 0.226 | 0.546 | 1.000 | 4126 | tags=100%, list=34%, signal=152% |
| 2968 | GOBP\_POSITIVE\_REGULATION\_OF\_ACTIN\_FILAMENT\_POLYMERIZATION |  | 64 | -0.35 | -1.19 | 0.193 | 0.546 | 1.000 | 3797 | tags=38%, list=32%, signal=55% |
| 2969 | GOBP\_REGULATION\_OF\_CELLULAR\_RESPONSE\_TO\_OSMOTIC\_STRESS |  | 5 | -0.62 | -1.19 | 0.235 | 0.547 | 1.000 | 397 | tags=20%, list=3%, signal=21% |
| 2970 | GOBP\_POSITIVE\_REGULATION\_OF\_CELL\_CELL\_ADHESION\_MEDIATED\_BY\_CADHERIN |  | 10 | -0.51 | -1.19 | 0.257 | 0.547 | 1.000 | 2205 | tags=50%, list=18%, signal=61% |
| 2971 | GOCC\_HISTONE\_DEACETYLASE\_COMPLEX |  | 56 | -0.36 | -1.19 | 0.195 | 0.548 | 1.000 | 4349 | tags=45%, list=36%, signal=70% |
| 2972 | GOBP\_ESTROGEN\_METABOLIC\_PROCESS |  | 17 | -0.45 | -1.19 | 0.240 | 0.548 | 1.000 | 2625 | tags=35%, list=22%, signal=45% |
| 2973 | GOBP\_NEGATIVE\_REGULATION\_OF\_LEUKOCYTE\_MEDIATED\_CYTOTOXICITY |  | 5 | -0.62 | -1.19 | 0.232 | 0.547 | 1.000 | 3031 | tags=60%, list=25%, signal=80% |
| 2974 | GOBP\_RNA\_DEPENDENT\_DNA\_BIOSYNTHETIC\_PROCESS |  | 58 | -0.35 | -1.19 | 0.197 | 0.547 | 1.000 | 4935 | tags=50%, list=41%, signal=84% |
| 2975 | GOBP\_STRIATED\_MUSCLE\_ADAPTATION |  | 28 | -0.40 | -1.19 | 0.228 | 0.547 | 1.000 | 2494 | tags=36%, list=21%, signal=45% |
| 2976 | GOBP\_GLYCEROL\_3\_PHOSPHATE\_METABOLIC\_PROCESS |  | 7 | -0.56 | -1.19 | 0.249 | 0.548 | 1.000 | 2362 | tags=29%, list=20%, signal=36% |
| 2977 | GOCC\_CONDENSED\_NUCLEAR\_CHROMOSOME |  | 54 | -0.36 | -1.19 | 0.196 | 0.547 | 1.000 | 3969 | tags=48%, list=33%, signal=72% |
| 2978 | GOBP\_PURINE\_DEOXYRIBONUCLEOSIDE\_TRIPHOSPHATE\_CATABOLIC\_PROCESS |  | 4 | -0.66 | -1.19 | 0.227 | 0.547 | 1.000 | 3094 | tags=75%, list=26%, signal=101% |
| 2979 | GOBP\_GOLGI\_TO\_PLASMA\_MEMBRANE\_TRANSPORT |  | 49 | -0.36 | -1.19 | 0.208 | 0.547 | 1.000 | 4275 | tags=49%, list=36%, signal=76% |
| 2980 | GOBP\_NEGATIVE\_REGULATION\_OF\_DEVELOPMENTAL\_PROCESS |  | 542 | -0.30 | -1.19 | 0.028 | 0.547 | 1.000 | 3724 | tags=35%, list=31%, signal=48% |
| 2981 | GOBP\_LYMPHOCYTE\_MEDIATED\_IMMUNITY |  | 125 | -0.32 | -1.19 | 0.150 | 0.547 | 1.000 | 2639 | tags=31%, list=22%, signal=40% |
| 2982 | GOBP\_PRE\_B\_CELL\_DIFFERENTIATION |  | 4 | -0.66 | -1.19 | 0.238 | 0.548 | 1.000 | 1667 | tags=50%, list=14%, signal=58% |
| 2983 | GOBP\_POSITIVE\_REGULATION\_OF\_TOLERANCE\_INDUCTION |  | 4 | -0.66 | -1.19 | 0.230 | 0.548 | 1.000 | 3163 | tags=75%, list=26%, signal=102% |
| 2984 | GOBP\_GONADOTROPIN\_SECRETION |  | 7 | -0.56 | -1.19 | 0.239 | 0.548 | 1.000 | 3544 | tags=57%, list=30%, signal=81% |
| 2985 | GOMF\_NFAT\_PROTEIN\_BINDING |  | 3 | -0.71 | -1.19 | 0.218 | 0.548 | 1.000 | 3510 | tags=100%, list=29%, signal=141% |
| 2986 | GOBP\_REGULATION\_OF\_EPITHELIAL\_CELL\_MIGRATION |  | 186 | -0.31 | -1.19 | 0.116 | 0.549 | 1.000 | 3613 | tags=36%, list=30%, signal=51% |
| 2987 | GOBP\_REGULATION\_OF\_LIPID\_STORAGE |  | 35 | -0.38 | -1.19 | 0.218 | 0.549 | 1.000 | 3968 | tags=54%, list=33%, signal=81% |
| 2988 | GOBP\_POSITIVE\_REGULATION\_OF\_NEURON\_MATURATION |  | 5 | -0.62 | -1.19 | 0.244 | 0.549 | 1.000 | 3433 | tags=80%, list=29%, signal=112% |
| 2989 | GOBP\_REGULATION\_OF\_ENDOCRINE\_PROCESS |  | 19 | -0.43 | -1.19 | 0.240 | 0.549 | 1.000 | 4164 | tags=58%, list=35%, signal=88% |
| 2990 | GOBP\_POSITIVE\_REGULATION\_OF\_TRANSCRIPTION\_INITIATION\_FROM\_RNA\_POLYMERASE\_II\_PROMOTER |  | 23 | -0.41 | -1.19 | 0.232 | 0.549 | 1.000 | 4896 | tags=57%, list=41%, signal=95% |
| 2991 | GOBP\_POSITIVE\_REGULATION\_OF\_PEPTIDYL\_LYSINE\_ACETYLATION |  | 23 | -0.42 | -1.19 | 0.235 | 0.549 | 1.000 | 3885 | tags=52%, list=32%, signal=77% |
| 2992 | GOBP\_CIRCADIAN\_SLEEP\_WAKE\_CYCLE\_NON\_REM\_SLEEP |  | 3 | -0.71 | -1.19 | 0.223 | 0.550 | 1.000 | 2052 | tags=67%, list=17%, signal=80% |
| 2993 | GOBP\_POSITIVE\_REGULATION\_OF\_RESPONSE\_TO\_DNA\_DAMAGE\_STIMULUS |  | 75 | -0.34 | -1.19 | 0.188 | 0.550 | 1.000 | 3655 | tags=39%, list=30%, signal=55% |
| 2994 | GOBP\_LYMPHOID\_PROGENITOR\_CELL\_DIFFERENTIATION |  | 19 | -0.44 | -1.19 | 0.233 | 0.550 | 1.000 | 650 | tags=21%, list=5%, signal=22% |
| 2995 | GOBP\_BIOLOGICAL\_PROCESS\_INVOLVED\_IN\_INTRASPECIES\_INTERACTION\_BETWEEN\_ORGANISMS |  | 27 | -0.40 | -1.19 | 0.231 | 0.550 | 1.000 | 3699 | tags=52%, list=31%, signal=75% |
| 2996 | GOMF\_DEAMINASE\_ACTIVITY |  | 18 | -0.44 | -1.19 | 0.239 | 0.550 | 1.000 | 4389 | tags=56%, list=37%, signal=87% |
| 2997 | GOBP\_LUNG\_EPITHELIUM\_DEVELOPMENT |  | 25 | -0.41 | -1.19 | 0.233 | 0.550 | 1.000 | 2267 | tags=36%, list=19%, signal=44% |
| 2998 | GOBP\_POSITIVE\_REGULATION\_OF\_RESPONSE\_TO\_EXTERNAL\_STIMULUS |  | 300 | -0.31 | -1.19 | 0.079 | 0.550 | 1.000 | 3308 | tags=33%, list=28%, signal=44% |
| 2999 | GOBP\_REGULATION\_OF\_SKELETAL\_MUSCLE\_FIBER\_DEVELOPMENT |  | 4 | -0.66 | -1.19 | 0.230 | 0.550 | 1.000 | 2717 | tags=50%, list=23%, signal=65% |
| 3000 | GOMF\_NAD\_P\_H\_OXIDASE\_H2O2\_FORMING\_ACTIVITY |  | 4 | -0.66 | -1.19 | 0.238 | 0.550 | 1.000 | 36 | tags=25%, list=0%, signal=25% |
| 3001 | GOBP\_DEOXYRIBOSE\_PHOSPHATE\_CATABOLIC\_PROCESS |  | 19 | -0.43 | -1.19 | 0.242 | 0.550 | 1.000 | 3094 | tags=42%, list=26%, signal=57% |
| 3002 | GOBP\_BONE\_TRABECULA\_MORPHOGENESIS |  | 8 | -0.54 | -1.19 | 0.242 | 0.550 | 1.000 | 1579 | tags=38%, list=13%, signal=43% |
| 3003 | GOBP\_PROTEIN\_LOCALIZATION\_TO\_NUCLEUS |  | 211 | -0.31 | -1.19 | 0.102 | 0.550 | 1.000 | 4270 | tags=38%, list=36%, signal=59% |
| 3004 | GOBP\_RESPONSE\_TO\_L\_ASCORBIC\_ACID |  | 4 | -0.65 | -1.19 | 0.236 | 0.550 | 1.000 | 1535 | tags=50%, list=13%, signal=57% |
| 3005 | GOBP\_BONE\_CELL\_DEVELOPMENT |  | 21 | -0.42 | -1.19 | 0.241 | 0.550 | 1.000 | 2769 | tags=48%, list=23%, signal=62% |
| 3006 | GOBP\_REGULATION\_OF\_PEPTIDYL\_SERINE\_PHOSPHORYLATION |  | 89 | -0.33 | -1.19 | 0.179 | 0.550 | 1.000 | 4027 | tags=38%, list=34%, signal=57% |
| 3007 | GOBP\_DOUBLE\_STRAND\_BREAK\_REPAIR\_VIA\_BREAK\_INDUCED\_REPLICATION |  | 9 | -0.52 | -1.19 | 0.251 | 0.551 | 1.000 | 5159 | tags=89%, list=43%, signal=156% |
| 3008 | GOBP\_VIRAL\_RNA\_GENOME\_REPLICATION |  | 20 | -0.43 | -1.19 | 0.233 | 0.551 | 1.000 | 5781 | tags=75%, list=48%, signal=144% |
| 3009 | GOBP\_CELLULAR\_BIOGENIC\_AMINE\_METABOLIC\_PROCESS |  | 62 | -0.35 | -1.19 | 0.199 | 0.552 | 1.000 | 2768 | tags=34%, list=23%, signal=44% |
| 3010 | GOCC\_MICROTUBULE\_BUNDLE |  | 4 | -0.65 | -1.19 | 0.234 | 0.552 | 1.000 | 478 | tags=25%, list=4%, signal=26% |
| 3011 | GOBP\_CEREBELLAR\_CORTEX\_DEVELOPMENT |  | 29 | -0.40 | -1.19 | 0.227 | 0.552 | 1.000 | 1452 | tags=21%, list=12%, signal=23% |
| 3012 | GOCC\_AP\_3\_ADAPTOR\_COMPLEX |  | 9 | -0.53 | -1.19 | 0.249 | 0.552 | 1.000 | 3127 | tags=44%, list=26%, signal=60% |
| 3013 | GOBP\_SEMAPHORIN\_PLEXIN\_SIGNALING\_PATHWAY\_INVOLVED\_IN\_AXON\_GUIDANCE |  | 8 | -0.54 | -1.19 | 0.251 | 0.552 | 1.000 | 4739 | tags=75%, list=39%, signal=124% |
| 3014 | GOBP\_GENETIC\_IMPRINTING |  | 14 | -0.46 | -1.19 | 0.246 | 0.552 | 1.000 | 2877 | tags=43%, list=24%, signal=56% |
| 3015 | GOBP\_NEGATIVE\_REGULATION\_OF\_REPRODUCTIVE\_PROCESS |  | 30 | -0.39 | -1.19 | 0.224 | 0.552 | 1.000 | 3026 | tags=30%, list=25%, signal=40% |
| 3016 | GOBP\_REGULATION\_OF\_SODIUM\_ION\_TRANSPORT |  | 56 | -0.35 | -1.19 | 0.198 | 0.552 | 1.000 | 4286 | tags=48%, list=36%, signal=75% |
| 3017 | GOBP\_PEPTIDYL\_LYSINE\_METHYLATION |  | 86 | -0.34 | -1.19 | 0.177 | 0.552 | 1.000 | 3644 | tags=42%, list=30%, signal=60% |
| 3018 | GOBP\_RETROGRADE\_NEURONAL\_DENSE\_CORE\_VESICLE\_TRANSPORT |  | 3 | -0.71 | -1.19 | 0.222 | 0.553 | 1.000 | 1047 | tags=67%, list=9%, signal=73% |
| 3019 | GOCC\_PRC1\_COMPLEX |  | 12 | -0.48 | -1.19 | 0.262 | 0.553 | 1.000 | 4500 | tags=67%, list=37%, signal=107% |
| 3020 | GOBP\_REGULATION\_OF\_NON\_MEMBRANE\_SPANNING\_PROTEIN\_TYROSINE\_KINASE\_ACTIVITY |  | 5 | -0.62 | -1.19 | 0.243 | 0.553 | 1.000 | 871 | tags=40%, list=7%, signal=43% |
| 3021 | GOBP\_SOMATIC\_DIVERSIFICATION\_OF\_IMMUNOGLOBULINS |  | 46 | -0.37 | -1.19 | 0.212 | 0.553 | 1.000 | 3632 | tags=41%, list=30%, signal=59% |
| 3022 | GOBP\_NEURON\_CELL\_CELL\_ADHESION |  | 10 | -0.51 | -1.19 | 0.249 | 0.554 | 1.000 | 3431 | tags=50%, list=29%, signal=70% |
| 3023 | GOBP\_REGULATION\_OF\_CELLULAR\_RESPONSE\_TO\_STRESS |  | 492 | -0.30 | -1.19 | 0.043 | 0.554 | 1.000 | 4125 | tags=39%, list=34%, signal=58% |
| 3024 | GOBP\_REGULATION\_OF\_SKELETAL\_MUSCLE\_ADAPTATION |  | 7 | -0.56 | -1.19 | 0.255 | 0.554 | 1.000 | 1743 | tags=29%, list=15%, signal=33% |
| 3025 | GOBP\_REGULATION\_OF\_SYNAPTIC\_TRANSMISSION\_CHOLINERGIC |  | 5 | -0.62 | -1.19 | 0.246 | 0.553 | 1.000 | 322 | tags=40%, list=3%, signal=41% |
| 3026 | GOBP\_GLUCAN\_BIOSYNTHETIC\_PROCESS |  | 30 | -0.39 | -1.19 | 0.234 | 0.553 | 1.000 | 4203 | tags=50%, list=35%, signal=77% |
| 3027 | GOBP\_B\_CELL\_LINEAGE\_COMMITMENT |  | 5 | -0.62 | -1.19 | 0.236 | 0.554 | 1.000 | 1215 | tags=40%, list=10%, signal=44% |
| 3028 | GOCC\_INTRINSIC\_COMPONENT\_OF\_SYNAPTIC\_MEMBRANE |  | 73 | -0.34 | -1.19 | 0.187 | 0.554 | 1.000 | 3125 | tags=32%, list=26%, signal=42% |
| 3029 | GOBP\_REGULATION\_OF\_GROWTH |  | 424 | -0.30 | -1.19 | 0.052 | 0.554 | 1.000 | 3341 | tags=31%, list=28%, signal=42% |
| 3030 | GOMF\_LYS63\_SPECIFIC\_DEUBIQUITINASE\_ACTIVITY |  | 9 | -0.52 | -1.19 | 0.250 | 0.554 | 1.000 | 1457 | tags=22%, list=12%, signal=25% |
| 3031 | GOBP\_EMBRYONIC\_APPENDAGE\_MORPHOGENESIS |  | 70 | -0.34 | -1.19 | 0.191 | 0.554 | 1.000 | 4097 | tags=41%, list=34%, signal=63% |
| 3032 | GOBP\_FOREBRAIN\_NEURON\_DEVELOPMENT |  | 17 | -0.44 | -1.19 | 0.249 | 0.553 | 1.000 | 2369 | tags=29%, list=20%, signal=37% |
| 3033 | GOBP\_DORSAL\_VENTRAL\_AXIS\_SPECIFICATION |  | 4 | -0.65 | -1.19 | 0.242 | 0.553 | 1.000 | 2886 | tags=50%, list=24%, signal=66% |
| 3034 | GOBP\_CORTICAL\_CYTOSKELETON\_ORGANIZATION |  | 49 | -0.36 | -1.19 | 0.211 | 0.554 | 1.000 | 3678 | tags=39%, list=31%, signal=56% |
| 3035 | GOBP\_INNER\_CELL\_MASS\_CELL\_PROLIFERATION |  | 13 | -0.47 | -1.19 | 0.248 | 0.554 | 1.000 | 2182 | tags=23%, list=18%, signal=28% |
| 3036 | GOCC\_NUCLEAR\_PORE\_NUCLEAR\_BASKET |  | 9 | -0.52 | -1.19 | 0.255 | 0.554 | 1.000 | 3317 | tags=44%, list=28%, signal=61% |
| 3037 | GOBP\_NEGATIVE\_REGULATION\_OF\_INTRACELLULAR\_ESTROGEN\_RECEPTOR\_SIGNALING\_PATHWAY |  | 10 | -0.51 | -1.19 | 0.250 | 0.555 | 1.000 | 3761 | tags=50%, list=31%, signal=73% |
| 3038 | GOBP\_POSITIVE\_REGULATION\_OF\_CELL\_MIGRATION\_BY\_VASCULAR\_ENDOTHELIAL\_GROWTH\_FACTOR\_SIGNALING\_PATHWAY |  | 7 | -0.56 | -1.19 | 0.256 | 0.555 | 1.000 | 3588 | tags=71%, list=30%, signal=102% |
| 3039 | GOBP\_SOMITE\_SPECIFICATION |  | 3 | -0.71 | -1.19 | 0.235 | 0.555 | 1.000 | 1402 | tags=33%, list=12%, signal=38% |
| 3040 | GOBP\_NEGATIVE\_REGULATION\_OF\_INTERLEUKIN\_17\_PRODUCTION |  | 6 | -0.58 | -1.19 | 0.253 | 0.555 | 1.000 | 1788 | tags=50%, list=15%, signal=59% |
| 3041 | GOBP\_REGULATION\_OF\_GROWTH\_RATE |  | 4 | -0.66 | -1.19 | 0.231 | 0.555 | 1.000 | 3821 | tags=75%, list=32%, signal=110% |
| 3042 | GOBP\_RECEPTOR\_MEDIATED\_ENDOCYTOSIS |  | 180 | -0.31 | -1.19 | 0.128 | 0.555 | 1.000 | 3321 | tags=32%, list=28%, signal=43% |
| 3043 | GOBP\_POSITIVE\_REGULATION\_OF\_EPIDERMAL\_CELL\_DIFFERENTIATION |  | 12 | -0.48 | -1.19 | 0.253 | 0.555 | 1.000 | 1031 | tags=25%, list=9%, signal=27% |
| 3044 | GOBP\_ENDOCHONDRAL\_BONE\_MORPHOGENESIS |  | 36 | -0.38 | -1.19 | 0.230 | 0.554 | 1.000 | 4102 | tags=42%, list=34%, signal=63% |
| 3045 | GOCC\_PHOTORECEPTOR\_OUTER\_SEGMENT |  | 37 | -0.38 | -1.19 | 0.225 | 0.555 | 1.000 | 4590 | tags=51%, list=38%, signal=83% |
| 3046 | GOBP\_LEUKOCYTE\_CHEMOTAXIS\_INVOLVED\_IN\_INFLAMMATORY\_RESPONSE |  | 5 | -0.61 | -1.19 | 0.242 | 0.555 | 1.000 | 1734 | tags=60%, list=14%, signal=70% |
| 3047 | GOBP\_REGULATION\_OF\_NITRIC\_OXIDE\_MEDIATED\_SIGNAL\_TRANSDUCTION |  | 6 | -0.59 | -1.19 | 0.245 | 0.555 | 1.000 | 3196 | tags=67%, list=27%, signal=91% |
| 3048 | GOMF\_UBIQUITIN\_LIKE\_PROTEIN\_BINDING |  | 80 | -0.34 | -1.19 | 0.192 | 0.555 | 1.000 | 4298 | tags=44%, list=36%, signal=68% |
| 3049 | GOBP\_OTIC\_VESICLE\_FORMATION |  | 5 | -0.61 | -1.19 | 0.241 | 0.554 | 1.000 | 1146 | tags=40%, list=10%, signal=44% |
| 3050 | GOBP\_REGULATION\_OF\_BLOOD\_CIRCULATION |  | 171 | -0.31 | -1.19 | 0.129 | 0.555 | 1.000 | 1919 | tags=20%, list=16%, signal=23% |
| 3051 | GOBP\_VOCAL\_LEARNING |  | 3 | -0.71 | -1.19 | 0.234 | 0.555 | 1.000 | 3431 | tags=100%, list=29%, signal=140% |
| 3052 | GOBP\_ACTOMYOSIN\_CONTRACTILE\_RING\_ORGANIZATION |  | 6 | -0.59 | -1.19 | 0.251 | 0.555 | 1.000 | 2588 | tags=50%, list=22%, signal=64% |
| 3053 | GOBP\_VENTRICULAR\_CARDIAC\_MUSCLE\_CELL\_DEVELOPMENT |  | 6 | -0.59 | -1.18 | 0.251 | 0.555 | 1.000 | 3331 | tags=67%, list=28%, signal=92% |
| 3054 | GOBP\_BASEMENT\_MEMBRANE\_ORGANIZATION |  | 26 | -0.40 | -1.18 | 0.236 | 0.555 | 1.000 | 4102 | tags=46%, list=34%, signal=70% |
| 3055 | GOBP\_REGULATION\_OF\_SYNAPTIC\_VESICLE\_TRANSPORT |  | 3 | -0.71 | -1.18 | 0.233 | 0.555 | 1.000 | 3483 | tags=100%, list=29%, signal=141% |
| 3056 | GOMF\_PROTEIN\_ARGININE\_N\_METHYLTRANSFERASE\_ACTIVITY |  | 6 | -0.58 | -1.18 | 0.249 | 0.555 | 1.000 | 3647 | tags=67%, list=30%, signal=96% |
| 3057 | GOBP\_MODULATION\_BY\_SYMBIONT\_OF\_HOST\_AUTOPHAGY |  | 6 | -0.58 | -1.18 | 0.247 | 0.556 | 1.000 | 4561 | tags=83%, list=38%, signal=134% |
| 3058 | GOMF\_THROMBIN\_ACTIVATED\_RECEPTOR\_ACTIVITY |  | 4 | -0.65 | -1.18 | 0.242 | 0.556 | 1.000 | 2652 | tags=75%, list=22%, signal=96% |
| 3059 | GOBP\_EPITHELIAL\_TUBE\_BRANCHING\_INVOLVED\_IN\_LUNG\_MORPHOGENESIS |  | 23 | -0.42 | -1.18 | 0.250 | 0.556 | 1.000 | 1603 | tags=26%, list=13%, signal=30% |
| 3060 | GOBP\_MITOTIC\_G2\_DNA\_DAMAGE\_CHECKPOINT |  | 20 | -0.43 | -1.18 | 0.249 | 0.556 | 1.000 | 3852 | tags=50%, list=32%, signal=73% |
| 3061 | GOBP\_REGULATION\_OF\_CELL\_CYCLE\_PROCESS |  | 574 | -0.30 | -1.18 | 0.034 | 0.557 | 1.000 | 4529 | tags=40%, list=38%, signal=62% |
| 3062 | GOBP\_PLATELET\_DEGRANULATION |  | 87 | -0.33 | -1.18 | 0.183 | 0.557 | 1.000 | 4268 | tags=39%, list=36%, signal=60% |
| 3063 | GOCC\_PHAGOCYTIC\_CUP |  | 19 | -0.43 | -1.18 | 0.249 | 0.556 | 1.000 | 3052 | tags=42%, list=25%, signal=56% |
| 3064 | GOBP\_MITOTIC\_SPINDLE\_ORGANIZATION |  | 99 | -0.33 | -1.18 | 0.177 | 0.557 | 1.000 | 5928 | tags=61%, list=49%, signal=119% |
| 3065 | GOBP\_AUTOPHAGY\_OF\_PEROXISOME |  | 5 | -0.61 | -1.18 | 0.242 | 0.557 | 1.000 | 3954 | tags=80%, list=33%, signal=119% |
| 3066 | GOMF\_CALCIUM\_RELEASE\_CHANNEL\_ACTIVITY |  | 14 | -0.46 | -1.18 | 0.249 | 0.557 | 1.000 | 3313 | tags=43%, list=28%, signal=59% |
| 3067 | GOBP\_VOLUNTARY\_MUSCULOSKELETAL\_MOVEMENT |  | 5 | -0.62 | -1.18 | 0.254 | 0.557 | 1.000 | 4084 | tags=80%, list=34%, signal=121% |
| 3068 | GOBP\_NEGATIVE\_REGULATION\_OF\_TRANSLATIONAL\_ELONGATION |  | 4 | -0.66 | -1.18 | 0.241 | 0.557 | 1.000 | 3933 | tags=50%, list=33%, signal=74% |
| 3069 | GOBP\_NEGATIVE\_REGULATION\_OF\_GRANULOCYTE\_DIFFERENTIATION |  | 7 | -0.56 | -1.18 | 0.262 | 0.557 | 1.000 | 774 | tags=29%, list=6%, signal=31% |
| 3070 | GOBP\_CELL\_AGGREGATION |  | 9 | -0.52 | -1.18 | 0.264 | 0.557 | 1.000 | 4111 | tags=56%, list=34%, signal=84% |
| 3071 | GOBP\_NEGATIVE\_REGULATION\_OF\_ENDOTHELIAL\_CELL\_PROLIFERATION |  | 29 | -0.40 | -1.18 | 0.242 | 0.557 | 1.000 | 1239 | tags=28%, list=10%, signal=31% |
| 3072 | GOBP\_POSITIVE\_REGULATION\_OF\_CD4\_POSITIVE\_ALPHA\_BETA\_T\_CELL\_ACTIVATION |  | 23 | -0.41 | -1.18 | 0.240 | 0.557 | 1.000 | 2345 | tags=35%, list=20%, signal=43% |
| 3073 | GOBP\_RNA\_DECAPPING |  | 14 | -0.46 | -1.18 | 0.253 | 0.557 | 1.000 | 4666 | tags=50%, list=39%, signal=82% |
| 3074 | GOBP\_POSITIVE\_REGULATION\_OF\_HETEROTYPIC\_CELL\_CELL\_ADHESION |  | 9 | -0.52 | -1.18 | 0.257 | 0.558 | 1.000 | 2047 | tags=44%, list=17%, signal=54% |
| 3075 | GOBP\_VERY\_LONG\_CHAIN\_FATTY\_ACID\_METABOLIC\_PROCESS |  | 24 | -0.41 | -1.18 | 0.238 | 0.558 | 1.000 | 5007 | tags=67%, list=42%, signal=114% |
| 3076 | GOMF\_SULFURIC\_ESTER\_HYDROLASE\_ACTIVITY |  | 14 | -0.46 | -1.18 | 0.260 | 0.558 | 1.000 | 3456 | tags=50%, list=29%, signal=70% |
| 3077 | GOBP\_GLIAL\_CELL\_DIFFERENTIATION |  | 143 | -0.32 | -1.18 | 0.151 | 0.558 | 1.000 | 1968 | tags=20%, list=16%, signal=24% |
| 3078 | GOBP\_ACIDIC\_AMINO\_ACID\_TRANSPORT |  | 46 | -0.36 | -1.18 | 0.216 | 0.558 | 1.000 | 2313 | tags=33%, list=19%, signal=40% |
| 3079 | GOBP\_VASCULAR\_ENDOTHELIAL\_GROWTH\_FACTOR\_RECEPTOR\_2\_SIGNALING\_PATHWAY |  | 4 | -0.65 | -1.18 | 0.234 | 0.558 | 1.000 | 3196 | tags=75%, list=27%, signal=102% |
| 3080 | GOBP\_RESPONSE\_TO\_HISTAMINE |  | 4 | -0.66 | -1.18 | 0.242 | 0.558 | 1.000 | 1782 | tags=50%, list=15%, signal=59% |
| 3081 | GOMF\_C3HC4\_TYPE\_RING\_FINGER\_DOMAIN\_BINDING |  | 3 | -0.71 | -1.18 | 0.233 | 0.559 | 1.000 | 1793 | tags=67%, list=15%, signal=78% |
| 3082 | GOBP\_AUDITORY\_RECEPTOR\_CELL\_FATE\_COMMITMENT |  | 4 | -0.65 | -1.18 | 0.242 | 0.559 | 1.000 | 2212 | tags=50%, list=18%, signal=61% |
| 3083 | GOBP\_REGULATION\_OF\_CELL\_CYCLE\_G1\_S\_PHASE\_TRANSITION |  | 124 | -0.32 | -1.18 | 0.162 | 0.559 | 1.000 | 4184 | tags=40%, list=35%, signal=61% |
| 3084 | GOCC\_EXTRINSIC\_COMPONENT\_OF\_CYTOPLASMIC\_SIDE\_OF\_PLASMA\_MEMBRANE |  | 69 | -0.34 | -1.18 | 0.201 | 0.559 | 1.000 | 2599 | tags=32%, list=22%, signal=40% |
| 3085 | GOBP\_NEGATIVE\_REGULATION\_OF\_B\_CELL\_DIFFERENTIATION |  | 4 | -0.65 | -1.18 | 0.246 | 0.559 | 1.000 | 2771 | tags=50%, list=23%, signal=65% |
| 3086 | GOBP\_CELL\_CELL\_ADHESION\_MEDIATED\_BY\_CADHERIN |  | 24 | -0.41 | -1.18 | 0.244 | 0.559 | 1.000 | 3269 | tags=46%, list=27%, signal=63% |
| 3087 | GOBP\_URETERIC\_BUD\_ELONGATION |  | 6 | -0.58 | -1.18 | 0.254 | 0.560 | 1.000 | 419 | tags=17%, list=3%, signal=17% |
| 3088 | GOBP\_POSITIVE\_REGULATION\_OF\_T\_CELL\_MEDIATED\_CYTOTOXICITY |  | 8 | -0.53 | -1.18 | 0.256 | 0.560 | 1.000 | 1519 | tags=38%, list=13%, signal=43% |
| 3089 | GOMF\_SODIUM\_PHOSPHATE\_SYMPORTER\_ACTIVITY |  | 4 | -0.65 | -1.18 | 0.236 | 0.560 | 1.000 | 2395 | tags=50%, list=20%, signal=62% |
| 3090 | GOMF\_ORGANIC\_ACID\_TRANSMEMBRANE\_TRANSPORTER\_ACTIVITY |  | 100 | -0.33 | -1.18 | 0.175 | 0.559 | 1.000 | 1410 | tags=23%, list=12%, signal=26% |
| 3091 | GOBP\_REGULATION\_OF\_RESPONSE\_TO\_BIOTIC\_STIMULUS |  | 237 | -0.31 | -1.18 | 0.112 | 0.559 | 1.000 | 3447 | tags=35%, list=29%, signal=48% |
| 3092 | GOBP\_NEGATIVE\_REGULATION\_OF\_CELL\_CYCLE |  | 446 | -0.30 | -1.18 | 0.049 | 0.559 | 1.000 | 4778 | tags=43%, list=40%, signal=68% |
| 3093 | GOBP\_REGULATION\_OF\_GTP\_BINDING |  | 11 | -0.49 | -1.18 | 0.265 | 0.559 | 1.000 | 2311 | tags=27%, list=19%, signal=34% |
| 3094 | GOBP\_MIDBRAIN\_DEVELOPMENT |  | 54 | -0.35 | -1.18 | 0.210 | 0.559 | 1.000 | 3909 | tags=43%, list=33%, signal=63% |
| 3095 | GOBP\_SMAD\_PROTEIN\_SIGNAL\_TRANSDUCTION |  | 52 | -0.36 | -1.18 | 0.221 | 0.560 | 1.000 | 3377 | tags=42%, list=28%, signal=59% |
| 3096 | GOBP\_NEGATIVE\_REGULATION\_OF\_CELL\_DIFFERENTIATION |  | 399 | -0.30 | -1.18 | 0.063 | 0.560 | 1.000 | 3724 | tags=34%, list=31%, signal=48% |
| 3097 | GOMF\_AMINOPEPTIDASE\_ACTIVITY |  | 31 | -0.39 | -1.18 | 0.234 | 0.561 | 1.000 | 2881 | tags=29%, list=24%, signal=38% |
| 3098 | GOBP\_ORGANIC\_HYDROXY\_COMPOUND\_METABOLIC\_PROCESS |  | 368 | -0.30 | -1.18 | 0.071 | 0.560 | 1.000 | 2786 | tags=27%, list=23%, signal=34% |
| 3099 | GOCC\_NEUROFILAMENT |  | 5 | -0.61 | -1.18 | 0.258 | 0.561 | 1.000 | 3765 | tags=80%, list=31%, signal=116% |
| 3100 | GOBP\_T\_CELL\_LINEAGE\_COMMITMENT |  | 15 | -0.45 | -1.18 | 0.257 | 0.560 | 1.000 | 3565 | tags=47%, list=30%, signal=66% |
| 3101 | GOBP\_CEREBRAL\_CORTEX\_RADIALLY\_ORIENTED\_CELL\_MIGRATION |  | 20 | -0.43 | -1.18 | 0.254 | 0.560 | 1.000 | 4003 | tags=45%, list=33%, signal=67% |
| 3102 | GOBP\_NEGATIVE\_REGULATION\_OF\_RELAXATION\_OF\_MUSCLE |  | 3 | -0.71 | -1.18 | 0.237 | 0.560 | 1.000 | 893 | tags=33%, list=7%, signal=36% |
| 3103 | GOBP\_REGULATION\_OF\_HISTONE\_H3\_K9\_METHYLATION |  | 13 | -0.47 | -1.18 | 0.253 | 0.560 | 1.000 | 3644 | tags=62%, list=30%, signal=88% |
| 3104 | GOBP\_AMYLOID\_PRECURSOR\_PROTEIN\_METABOLIC\_PROCESS |  | 57 | -0.35 | -1.18 | 0.213 | 0.560 | 1.000 | 2604 | tags=26%, list=22%, signal=33% |
| 3105 | GOBP\_RESPONSE\_TO\_HYDROXYUREA |  | 9 | -0.52 | -1.18 | 0.257 | 0.561 | 1.000 | 3214 | tags=56%, list=27%, signal=76% |
| 3106 | GOBP\_RHYTHMIC\_PROCESS |  | 207 | -0.31 | -1.18 | 0.127 | 0.561 | 1.000 | 4432 | tags=45%, list=37%, signal=70% |
| 3107 | GOMF\_NUCLEOSOMAL\_HISTONE\_BINDING |  | 4 | -0.65 | -1.18 | 0.241 | 0.561 | 1.000 | 1643 | tags=50%, list=14%, signal=58% |
| 3108 | GOBP\_POSITIVE\_REGULATION\_OF\_CELL\_CYCLE\_ARREST |  | 59 | -0.35 | -1.18 | 0.213 | 0.561 | 1.000 | 2677 | tags=31%, list=22%, signal=39% |
| 3109 | GOBP\_RESPONSE\_TO\_MONOSACCHARIDE |  | 130 | -0.32 | -1.18 | 0.164 | 0.561 | 1.000 | 3978 | tags=41%, list=33%, signal=60% |
| 3110 | GOBP\_ACTIVATION\_OF\_JNKK\_ACTIVITY |  | 6 | -0.58 | -1.18 | 0.254 | 0.561 | 1.000 | 2964 | tags=50%, list=25%, signal=66% |
| 3111 | GOBP\_PYRIMIDINE\_CONTAINING\_COMPOUND\_SALVAGE |  | 10 | -0.50 | -1.18 | 0.257 | 0.561 | 1.000 | 5029 | tags=60%, list=42%, signal=103% |
| 3112 | GOBP\_ORGANIC\_ACID\_TRANSPORT |  | 206 | -0.31 | -1.18 | 0.129 | 0.561 | 1.000 | 2469 | tags=30%, list=21%, signal=37% |
| 3113 | GOBP\_ATF6\_MEDIATED\_UNFOLDED\_PROTEIN\_RESPONSE |  | 9 | -0.52 | -1.18 | 0.255 | 0.561 | 1.000 | 4736 | tags=78%, list=39%, signal=128% |
| 3114 | GOBP\_METANEPHRIC\_PART\_OF\_URETERIC\_BUD\_DEVELOPMENT |  | 3 | -0.71 | -1.18 | 0.244 | 0.561 | 1.000 | 2150 | tags=67%, list=18%, signal=81% |
| 3115 | GOBP\_POSITIVE\_REGULATION\_OF\_STRESS\_ACTIVATED\_PROTEIN\_KINASE\_SIGNALING\_CASCADE |  | 80 | -0.33 | -1.18 | 0.190 | 0.561 | 1.000 | 2769 | tags=35%, list=23%, signal=45% |
| 3116 | GOBP\_GLUCOSE\_IMPORT |  | 46 | -0.36 | -1.18 | 0.222 | 0.562 | 1.000 | 2747 | tags=35%, list=23%, signal=45% |
| 3117 | GOMF\_METALLODIPEPTIDASE\_ACTIVITY |  | 5 | -0.61 | -1.18 | 0.244 | 0.562 | 1.000 | 3400 | tags=80%, list=28%, signal=112% |
| 3118 | GOBP\_DETECTION\_OF\_CHEMICAL\_STIMULUS |  | 25 | -0.41 | -1.18 | 0.243 | 0.562 | 1.000 | 1269 | tags=24%, list=11%, signal=27% |
| 3119 | GOBP\_POSITIVE\_REGULATION\_OF\_TOLL\_LIKE\_RECEPTOR\_4\_SIGNALING\_PATHWAY |  | 8 | -0.54 | -1.18 | 0.261 | 0.562 | 1.000 | 250 | tags=25%, list=2%, signal=26% |
| 3120 | GOMF\_ALPHA\_N\_ACETYLNEURAMINATE\_ALPHA\_2\_8\_SIALYLTRANSFERASE\_ACTIVITY |  | 4 | -0.65 | -1.18 | 0.253 | 0.562 | 1.000 | 3855 | tags=75%, list=32%, signal=110% |
| 3121 | GOBP\_MYELOID\_LEUKOCYTE\_ACTIVATION |  | 427 | -0.30 | -1.18 | 0.060 | 0.562 | 1.000 | 4398 | tags=42%, list=37%, signal=64% |
| 3122 | GOMF\_GLYCERALDEHYDE\_3\_PHOSPHATE\_DEHYDROGENASE\_NADPLUS\_NON\_PHOSPHORYLATING\_ACTIVITY |  | 4 | -0.65 | -1.18 | 0.248 | 0.563 | 1.000 | 2534 | tags=50%, list=21%, signal=63% |
| 3123 | GOBP\_SYNAPTIC\_GROWTH\_AT\_NEUROMUSCULAR\_JUNCTION |  | 6 | -0.58 | -1.18 | 0.261 | 0.563 | 1.000 | 3699 | tags=50%, list=31%, signal=72% |
| 3124 | GOBP\_POSITIVE\_REGULATION\_OF\_MICROTUBULE\_POLYMERIZATION\_OR\_DEPOLYMERIZATION |  | 23 | -0.41 | -1.18 | 0.256 | 0.563 | 1.000 | 3069 | tags=35%, list=26%, signal=47% |
| 3125 | GOBP\_PEPTIDYL\_ARGININE\_N\_METHYLATION |  | 6 | -0.58 | -1.18 | 0.260 | 0.563 | 1.000 | 3647 | tags=67%, list=30%, signal=96% |
| 3126 | GOMF\_G\_PROTEIN\_COUPLED\_SEROTONIN\_RECEPTOR\_BINDING |  | 6 | -0.58 | -1.18 | 0.253 | 0.564 | 1.000 | 4450 | tags=67%, list=37%, signal=106% |
| 3127 | GOBP\_CALCIUM\_ION\_TRANSMEMBRANE\_TRANSPORT |  | 194 | -0.31 | -1.18 | 0.137 | 0.563 | 1.000 | 2938 | tags=27%, list=24%, signal=36% |
| 3128 | GOMF\_RECEPTOR\_REGULATOR\_ACTIVITY |  | 206 | -0.31 | -1.18 | 0.131 | 0.563 | 1.000 | 1944 | tags=24%, list=16%, signal=28% |
| 3129 | GOBP\_PRIMITIVE\_HEMOPOIESIS |  | 7 | -0.56 | -1.18 | 0.264 | 0.563 | 1.000 | 3196 | tags=57%, list=27%, signal=78% |
| 3130 | GOCC\_MIDBODY |  | 145 | -0.32 | -1.18 | 0.158 | 0.563 | 1.000 | 5290 | tags=51%, list=44%, signal=90% |
| 3131 | GOBP\_ACTIVATION\_OF\_PROTEIN\_KINASE\_C\_ACTIVITY |  | 4 | -0.65 | -1.18 | 0.249 | 0.563 | 1.000 | 484 | tags=25%, list=4%, signal=26% |
| 3132 | GOBP\_CYTOKINE\_MEDIATED\_SIGNALING\_PATHWAY |  | 469 | -0.30 | -1.18 | 0.053 | 0.563 | 1.000 | 3196 | tags=32%, list=27%, signal=42% |
| 3133 | GOMF\_PHOSPHOLIPID\_BINDING |  | 327 | -0.30 | -1.18 | 0.081 | 0.563 | 1.000 | 3769 | tags=35%, list=31%, signal=50% |
| 3134 | GOBP\_NEGATIVE\_REGULATION\_OF\_MAPK\_CASCADE |  | 117 | -0.32 | -1.18 | 0.169 | 0.564 | 1.000 | 4530 | tags=45%, list=38%, signal=72% |
| 3135 | GOMF\_UBIQUITIN\_BINDING |  | 63 | -0.35 | -1.18 | 0.210 | 0.564 | 1.000 | 4454 | tags=48%, list=37%, signal=75% |
| 3136 | GOBP\_EPIDERMAL\_GROWTH\_FACTOR\_RECEPTOR\_SIGNALING\_PATHWAY |  | 98 | -0.33 | -1.18 | 0.184 | 0.564 | 1.000 | 1637 | tags=22%, list=14%, signal=26% |
| 3137 | GOBP\_VASCULAR\_PROCESS\_IN\_CIRCULATORY\_SYSTEM |  | 178 | -0.31 | -1.18 | 0.135 | 0.565 | 1.000 | 2563 | tags=30%, list=21%, signal=37% |
| 3138 | GOMF\_NUCLEAR\_LOCALIZATION\_SEQUENCE\_BINDING |  | 19 | -0.43 | -1.18 | 0.262 | 0.565 | 1.000 | 5571 | tags=68%, list=46%, signal=127% |
| 3139 | GOBP\_REGULATION\_OF\_TYPE\_B\_PANCREATIC\_CELL\_DEVELOPMENT |  | 5 | -0.61 | -1.18 | 0.264 | 0.565 | 1.000 | 1347 | tags=40%, list=11%, signal=45% |
| 3140 | GOBP\_POSITIVE\_REGULATION\_OF\_PEPTIDASE\_ACTIVITY |  | 129 | -0.32 | -1.18 | 0.170 | 0.566 | 1.000 | 2982 | tags=30%, list=25%, signal=40% |
| 3141 | GOBP\_ADAPTIVE\_THERMOGENESIS |  | 108 | -0.32 | -1.18 | 0.176 | 0.566 | 1.000 | 2489 | tags=29%, list=21%, signal=36% |
| 3142 | GOBP\_ASTROCYTE\_DIFFERENTIATION |  | 51 | -0.36 | -1.18 | 0.222 | 0.566 | 1.000 | 1881 | tags=24%, list=16%, signal=28% |
| 3143 | GOBP\_RETINAL\_GANGLION\_CELL\_AXON\_GUIDANCE |  | 15 | -0.45 | -1.18 | 0.261 | 0.566 | 1.000 | 3196 | tags=33%, list=27%, signal=45% |
| 3144 | GOBP\_FATTY\_ACID\_TRANSPORT |  | 99 | -0.33 | -1.18 | 0.186 | 0.566 | 1.000 | 2913 | tags=36%, list=24%, signal=48% |
| 3145 | GOBP\_REGULATION\_OF\_REMOVAL\_OF\_SUPEROXIDE\_RADICALS |  | 6 | -0.58 | -1.18 | 0.267 | 0.566 | 1.000 | 3650 | tags=67%, list=30%, signal=96% |
| 3146 | GOBP\_POSITIVE\_REGULATION\_OF\_LEUKOCYTE\_ADHESION\_TO\_VASCULAR\_ENDOTHELIAL\_CELL |  | 13 | -0.47 | -1.18 | 0.267 | 0.566 | 1.000 | 1734 | tags=38%, list=14%, signal=45% |
| 3147 | GOBP\_POSITIVE\_REGULATION\_OF\_VASCULATURE\_DEVELOPMENT |  | 109 | -0.32 | -1.18 | 0.180 | 0.566 | 1.000 | 3308 | tags=35%, list=28%, signal=48% |
| 3148 | GOBP\_POLYSACCHARIDE\_DIGESTION |  | 4 | -0.65 | -1.18 | 0.248 | 0.566 | 1.000 | 290 | tags=50%, list=2%, signal=51% |
| 3149 | GOBP\_AMINO\_ACID\_SALVAGE |  | 6 | -0.58 | -1.18 | 0.265 | 0.566 | 1.000 | 1018 | tags=33%, list=8%, signal=36% |
| 3150 | GOBP\_POSITIVE\_REGULATION\_OF\_B\_CELL\_ACTIVATION |  | 52 | -0.35 | -1.18 | 0.219 | 0.566 | 1.000 | 3326 | tags=40%, list=28%, signal=56% |
| 3151 | GOMF\_GLUTAMATE\_RECEPTOR\_ACTIVITY |  | 9 | -0.52 | -1.18 | 0.262 | 0.566 | 1.000 | 4846 | tags=78%, list=40%, signal=130% |
| 3152 | GOBP\_NEGATIVE\_REGULATION\_OF\_NEURON\_PROJECTION\_REGENERATION |  | 11 | -0.49 | -1.18 | 0.268 | 0.566 | 1.000 | 3392 | tags=45%, list=28%, signal=63% |
| 3153 | GOBP\_ORGANELLE\_INHERITANCE |  | 13 | -0.47 | -1.17 | 0.269 | 0.566 | 1.000 | 3212 | tags=31%, list=27%, signal=42% |
| 3154 | GOBP\_ANTEROGRADE\_DENDRITIC\_TRANSPORT |  | 3 | -0.70 | -1.17 | 0.248 | 0.566 | 1.000 | 3555 | tags=100%, list=30%, signal=142% |
| 3155 | GOBP\_CELLULAR\_RESPONSE\_TO\_GLUCOSE\_STARVATION |  | 42 | -0.37 | -1.17 | 0.239 | 0.566 | 1.000 | 3993 | tags=43%, list=33%, signal=64% |
| 3156 | GOMF\_NITRIC\_OXIDE\_SYNTHASE\_REGULATOR\_ACTIVITY |  | 7 | -0.55 | -1.17 | 0.261 | 0.566 | 1.000 | 3909 | tags=57%, list=33%, signal=85% |
| 3157 | GOBP\_PEPTIDE\_CATABOLIC\_PROCESS |  | 22 | -0.41 | -1.17 | 0.254 | 0.566 | 1.000 | 3400 | tags=45%, list=28%, signal=63% |
| 3158 | GOBP\_PIGMENTATION |  | 66 | -0.34 | -1.17 | 0.219 | 0.566 | 1.000 | 3569 | tags=39%, list=30%, signal=56% |
| 3159 | GOBP\_REGULATION\_OF\_RESPONSE\_TO\_STRESS |  | 890 | -0.29 | -1.17 | 0.013 | 0.566 | 1.000 | 3695 | tags=35%, list=31%, signal=47% |
| 3160 | GOBP\_REGULATION\_OF\_HETEROTYPIC\_CELL\_CELL\_ADHESION |  | 15 | -0.45 | -1.17 | 0.265 | 0.566 | 1.000 | 2955 | tags=40%, list=25%, signal=53% |
| 3161 | GOBP\_NEURONAL\_STEM\_CELL\_POPULATION\_MAINTENANCE |  | 14 | -0.46 | -1.17 | 0.264 | 0.566 | 1.000 | 3864 | tags=43%, list=32%, signal=63% |
| 3162 | GOBP\_POSITIVE\_REGULATION\_OF\_MONOCYTE\_CHEMOTAXIS |  | 9 | -0.51 | -1.17 | 0.266 | 0.567 | 1.000 | 2679 | tags=56%, list=22%, signal=71% |
| 3163 | GOBP\_ZINC\_ION\_IMPORT\_ACROSS\_PLASMA\_MEMBRANE |  | 4 | -0.65 | -1.17 | 0.254 | 0.566 | 1.000 | 2258 | tags=50%, list=19%, signal=62% |
| 3164 | GOMF\_FOLIC\_ACID\_BINDING |  | 6 | -0.58 | -1.17 | 0.253 | 0.567 | 1.000 | 4059 | tags=67%, list=34%, signal=101% |
| 3165 | GOMF\_OXIDOREDUCTASE\_ACTIVITY\_ACTING\_ON\_PAIRED\_DONORS\_WITH\_INCORPORATION\_OR\_REDUCTION\_OF\_MOLECULAR\_OXYGEN\_REDUCED\_FLAVIN\_OR\_FLAVOPROTEIN\_AS\_ONE\_DONOR\_AND\_INCORPORATION\_OF\_ONE\_ATOM\_OF\_OXYGEN |  | 10 | -0.50 | -1.17 | 0.267 | 0.568 | 1.000 | 2705 | tags=50%, list=23%, signal=64% |
| 3166 | GOBP\_PRODUCTION\_OF\_SIRNA\_INVOLVED\_IN\_RNA\_INTERFERENCE |  | 6 | -0.58 | -1.17 | 0.272 | 0.568 | 1.000 | 2086 | tags=33%, list=17%, signal=40% |
| 3167 | GOBP\_DNA\_METABOLIC\_PROCESS |  | 667 | -0.30 | -1.17 | 0.028 | 0.567 | 1.000 | 4799 | tags=45%, list=40%, signal=70% |
| 3168 | GOBP\_REGULATION\_OF\_GENE\_EXPRESSION\_EPIGENETIC |  | 108 | -0.32 | -1.17 | 0.188 | 0.568 | 1.000 | 4649 | tags=46%, list=39%, signal=75% |
| 3169 | GOBP\_PROTEIN\_K63\_LINKED\_UBIQUITINATION |  | 41 | -0.37 | -1.17 | 0.231 | 0.568 | 1.000 | 4645 | tags=51%, list=39%, signal=83% |
| 3170 | GOMF\_RNA\_POLYMERASE\_II\_SPECIFIC\_DNA\_BINDING\_TRANSCRIPTION\_FACTOR\_BINDING |  | 214 | -0.31 | -1.17 | 0.126 | 0.568 | 1.000 | 4455 | tags=42%, list=37%, signal=65% |
| 3171 | GOBP\_DICHOTOMOUS\_SUBDIVISION\_OF\_AN\_EPITHELIAL\_TERMINAL\_UNIT |  | 7 | -0.55 | -1.17 | 0.263 | 0.567 | 1.000 | 3895 | tags=43%, list=32%, signal=63% |
| 3172 | GOMF\_POLY\_A\_SPECIFIC\_RIBONUCLEASE\_ACTIVITY |  | 12 | -0.48 | -1.17 | 0.269 | 0.567 | 1.000 | 2441 | tags=33%, list=20%, signal=42% |
| 3173 | GOMF\_GLUTAMATE\_BINDING |  | 3 | -0.70 | -1.17 | 0.236 | 0.568 | 1.000 | 2746 | tags=67%, list=23%, signal=86% |
| 3174 | GOBP\_LIPOSACCHARIDE\_METABOLIC\_PROCESS |  | 84 | -0.33 | -1.17 | 0.207 | 0.568 | 1.000 | 4067 | tags=42%, list=34%, signal=63% |
| 3175 | GOCC\_PLATELET\_ALPHA\_GRANULE |  | 61 | -0.35 | -1.17 | 0.221 | 0.567 | 1.000 | 3488 | tags=31%, list=29%, signal=44% |
| 3176 | GOBP\_L\_METHIONINE\_SALVAGE\_FROM\_METHYLTHIOADENOSINE |  | 5 | -0.61 | -1.17 | 0.265 | 0.567 | 1.000 | 1018 | tags=40%, list=8%, signal=44% |
| 3177 | GOBP\_BEHAVIORAL\_RESPONSE\_TO\_NICOTINE |  | 4 | -0.65 | -1.17 | 0.250 | 0.567 | 1.000 | 3603 | tags=75%, list=30%, signal=107% |
| 3178 | GOBP\_REGULATION\_OF\_INTRACELLULAR\_TRANSPORT |  | 259 | -0.31 | -1.17 | 0.112 | 0.567 | 1.000 | 3789 | tags=32%, list=32%, signal=46% |
| 3179 | GOBP\_STEROID\_HORMONE\_MEDIATED\_SIGNALING\_PATHWAY |  | 95 | -0.33 | -1.17 | 0.188 | 0.568 | 1.000 | 4512 | tags=51%, list=38%, signal=80% |
| 3180 | GOBP\_METAL\_ION\_TRANSPORT |  | 294 | -0.30 | -1.17 | 0.098 | 0.568 | 1.000 | 4173 | tags=38%, list=35%, signal=56% |
| 3181 | GOMF\_SINGLE\_STRANDED\_TELOMERIC\_DNA\_BINDING |  | 12 | -0.47 | -1.17 | 0.268 | 0.569 | 1.000 | 5648 | tags=83%, list=47%, signal=157% |
| 3182 | GOBP\_NEGATIVE\_REGULATION\_OF\_TRANSCRIPTION\_BY\_RNA\_POLYMERASE\_II |  | 528 | -0.30 | -1.17 | 0.043 | 0.569 | 1.000 | 4362 | tags=43%, list=36%, signal=64% |
| 3183 | GOCC\_VESICLE\_COAT |  | 43 | -0.37 | -1.17 | 0.236 | 0.569 | 1.000 | 5492 | tags=67%, list=46%, signal=124% |
| 3184 | GOBP\_NEGATIVE\_REGULATION\_OF\_CGMP\_MEDIATED\_SIGNALING |  | 3 | -0.70 | -1.17 | 0.248 | 0.569 | 1.000 | 2820 | tags=67%, list=23%, signal=87% |
| 3185 | GOBP\_NEGATIVE\_REGULATION\_OF\_DNA\_DEPENDENT\_DNA\_REPLICATION |  | 14 | -0.46 | -1.17 | 0.261 | 0.569 | 1.000 | 5255 | tags=71%, list=44%, signal=127% |
| 3186 | GOBP\_REGULATION\_OF\_BRANCHING\_INVOLVED\_IN\_SALIVARY\_GLAND\_MORPHOGENESIS |  | 6 | -0.57 | -1.17 | 0.275 | 0.570 | 1.000 | 1146 | tags=33%, list=10%, signal=37% |
| 3187 | GOBP\_POSITIVE\_REGULATION\_OF\_TRANSFORMING\_GROWTH\_FACTOR\_BETA\_PRODUCTION |  | 15 | -0.45 | -1.17 | 0.267 | 0.570 | 1.000 | 2820 | tags=47%, list=23%, signal=61% |
| 3188 | GOMF\_DNA\_SECONDARY\_STRUCTURE\_BINDING |  | 32 | -0.38 | -1.17 | 0.244 | 0.570 | 1.000 | 2771 | tags=38%, list=23%, signal=49% |
| 3189 | GOBP\_POSITIVE\_REGULATION\_OF\_EPITHELIAL\_CELL\_PROLIFERATION |  | 132 | -0.32 | -1.17 | 0.176 | 0.570 | 1.000 | 3615 | tags=37%, list=30%, signal=53% |
| 3190 | GOBP\_REGULATION\_OF\_PROTEIN\_SUMOYLATION |  | 20 | -0.42 | -1.17 | 0.264 | 0.570 | 1.000 | 5772 | tags=65%, list=48%, signal=125% |
| 3191 | GOCC\_SMAD\_PROTEIN\_COMPLEX |  | 7 | -0.55 | -1.17 | 0.273 | 0.570 | 1.000 | 3306 | tags=57%, list=28%, signal=79% |
| 3192 | GOBP\_NEGATIVE\_REGULATION\_OF\_CELL\_MATURATION |  | 3 | -0.70 | -1.17 | 0.253 | 0.570 | 1.000 | 625 | tags=33%, list=5%, signal=35% |
| 3193 | GOBP\_PROTEIN\_MODIFICATION\_BY\_SMALL\_PROTEIN\_CONJUGATION |  | 675 | -0.30 | -1.17 | 0.030 | 0.570 | 1.000 | 4645 | tags=42%, list=39%, signal=65% |
| 3194 | GOCC\_NSL\_COMPLEX |  | 7 | -0.55 | -1.17 | 0.264 | 0.570 | 1.000 | 3599 | tags=43%, list=30%, signal=61% |
| 3195 | GOBP\_AUTOPHAGIC\_CELL\_DEATH |  | 10 | -0.50 | -1.17 | 0.262 | 0.570 | 1.000 | 4317 | tags=50%, list=36%, signal=78% |
| 3196 | GOBP\_RESPONSE\_TO\_UV |  | 104 | -0.32 | -1.17 | 0.190 | 0.570 | 1.000 | 3799 | tags=38%, list=32%, signal=54% |
| 3197 | GOBP\_REGULATION\_OF\_NK\_T\_CELL\_DIFFERENTIATION |  | 4 | -0.64 | -1.17 | 0.254 | 0.571 | 1.000 | 3112 | tags=75%, list=26%, signal=101% |
| 3198 | GOBP\_ORGANIC\_ACID\_TRANSMEMBRANE\_TRANSPORT |  | 104 | -0.32 | -1.17 | 0.193 | 0.571 | 1.000 | 3859 | tags=41%, list=32%, signal=60% |
| 3199 | GOCC\_EXTERNAL\_ENCAPSULATING\_STRUCTURE |  | 329 | -0.30 | -1.17 | 0.087 | 0.571 | 1.000 | 3196 | tags=29%, list=27%, signal=39% |
| 3200 | GOBP\_RETINA\_MORPHOGENESIS\_IN\_CAMERA\_TYPE\_EYE |  | 37 | -0.37 | -1.17 | 0.245 | 0.571 | 1.000 | 1520 | tags=24%, list=13%, signal=28% |
| 3201 | GOBP\_SKELETAL\_MUSCLE\_ORGAN\_DEVELOPMENT |  | 101 | -0.32 | -1.17 | 0.188 | 0.571 | 1.000 | 2503 | tags=25%, list=21%, signal=31% |
| 3202 | GOBP\_POSITIVE\_REGULATION\_OF\_EPITHELIAL\_CELL\_PROLIFERATION\_INVOLVED\_IN\_WOUND\_HEALING |  | 5 | -0.61 | -1.17 | 0.263 | 0.571 | 1.000 | 1146 | tags=40%, list=10%, signal=44% |
| 3203 | GOBP\_NEGATIVE\_REGULATION\_OF\_TYROSINE\_PHOSPHORYLATION\_OF\_STAT\_PROTEIN |  | 11 | -0.49 | -1.17 | 0.281 | 0.571 | 1.000 | 1541 | tags=36%, list=13%, signal=42% |
| 3204 | GOBP\_ANATOMICAL\_STRUCTURE\_HOMEOSTASIS |  | 316 | -0.30 | -1.17 | 0.096 | 0.571 | 1.000 | 3927 | tags=37%, list=33%, signal=54% |
| 3205 | GOBP\_REGULATION\_OF\_NEUROTRANSMITTER\_RECEPTOR\_ACTIVITY |  | 31 | -0.38 | -1.17 | 0.252 | 0.571 | 1.000 | 3699 | tags=45%, list=31%, signal=65% |
| 3206 | GOBP\_INTRACELLULAR\_RECEPTOR\_SIGNALING\_PATHWAY |  | 187 | -0.31 | -1.17 | 0.141 | 0.571 | 1.000 | 4512 | tags=48%, list=38%, signal=76% |
| 3207 | GOBP\_HISTIDINE\_CATABOLIC\_PROCESS |  | 6 | -0.57 | -1.17 | 0.268 | 0.571 | 1.000 | 4371 | tags=83%, list=36%, signal=131% |
| 3208 | GOBP\_SPECIFICATION\_OF\_ANIMAL\_ORGAN\_IDENTITY |  | 22 | -0.41 | -1.17 | 0.273 | 0.571 | 1.000 | 2474 | tags=36%, list=21%, signal=46% |
| 3209 | GOMF\_CELL\_ADHESION\_MOLECULE\_BINDING |  | 398 | -0.30 | -1.17 | 0.074 | 0.571 | 1.000 | 2933 | tags=25%, list=24%, signal=32% |
| 3210 | GOBP\_KETONE\_CATABOLIC\_PROCESS |  | 8 | -0.53 | -1.17 | 0.272 | 0.571 | 1.000 | 2220 | tags=25%, list=18%, signal=31% |
| 3211 | GOBP\_MACROPHAGE\_DIFFERENTIATION |  | 35 | -0.38 | -1.17 | 0.249 | 0.571 | 1.000 | 3836 | tags=43%, list=32%, signal=63% |
| 3212 | GOBP\_AMELOGENESIS |  | 13 | -0.47 | -1.17 | 0.265 | 0.571 | 1.000 | 2968 | tags=46%, list=25%, signal=61% |
| 3213 | GOMF\_JUN\_KINASE\_BINDING |  | 8 | -0.53 | -1.17 | 0.262 | 0.572 | 1.000 | 5545 | tags=88%, list=46%, signal=162% |
| 3214 | GOBP\_HUMORAL\_IMMUNE\_RESPONSE |  | 92 | -0.33 | -1.17 | 0.202 | 0.571 | 1.000 | 3077 | tags=34%, list=26%, signal=45% |
| 3215 | GOBP\_VITAMIN\_D\_METABOLIC\_PROCESS |  | 15 | -0.45 | -1.17 | 0.279 | 0.571 | 1.000 | 3114 | tags=53%, list=26%, signal=72% |
| 3216 | GOMF\_RETINOIC\_ACID\_RESPONSIVE\_ELEMENT\_BINDING |  | 5 | -0.61 | -1.17 | 0.265 | 0.571 | 1.000 | 9 | tags=20%, list=0%, signal=20% |
| 3217 | GOMF\_SERINE\_HYDROLASE\_ACTIVITY |  | 79 | -0.33 | -1.17 | 0.219 | 0.572 | 1.000 | 3030 | tags=32%, list=25%, signal=42% |
| 3218 | GOBP\_REGULATION\_OF\_TUBE\_SIZE |  | 91 | -0.33 | -1.17 | 0.203 | 0.572 | 1.000 | 2563 | tags=30%, list=21%, signal=37% |
| 3219 | GOBP\_REGULATION\_OF\_RNA\_POLYMERASE\_II\_TRANSCRIPTION\_PREINITIATION\_COMPLEX\_ASSEMBLY |  | 10 | -0.50 | -1.17 | 0.273 | 0.571 | 1.000 | 875 | tags=20%, list=7%, signal=22% |
| 3220 | GOBP\_MUSCLE\_CELL\_PROLIFERATION |  | 136 | -0.32 | -1.17 | 0.173 | 0.571 | 1.000 | 3613 | tags=40%, list=30%, signal=56% |
| 3221 | GOBP\_MAST\_CELL\_MIGRATION |  | 11 | -0.49 | -1.17 | 0.282 | 0.572 | 1.000 | 1305 | tags=36%, list=11%, signal=41% |
| 3222 | GOBP\_HIGH\_DENSITY\_LIPOPROTEIN\_PARTICLE\_ASSEMBLY |  | 6 | -0.58 | -1.17 | 0.270 | 0.572 | 1.000 | 2997 | tags=50%, list=25%, signal=67% |
| 3223 | GOBP\_POSITIVE\_REGULATION\_OF\_ORGANELLE\_ORGANIZATION |  | 425 | -0.30 | -1.17 | 0.073 | 0.572 | 1.000 | 3695 | tags=34%, list=31%, signal=48% |
| 3224 | GOBP\_DENDRITIC\_CELL\_MIGRATION |  | 22 | -0.42 | -1.17 | 0.263 | 0.572 | 1.000 | 4173 | tags=55%, list=35%, signal=83% |
| 3225 | GOMF\_EXOPEPTIDASE\_ACTIVITY |  | 65 | -0.34 | -1.17 | 0.225 | 0.572 | 1.000 | 5105 | tags=49%, list=43%, signal=85% |
| 3226 | GOCC\_ORIGIN\_RECOGNITION\_COMPLEX |  | 8 | -0.53 | -1.17 | 0.273 | 0.572 | 1.000 | 4446 | tags=75%, list=37%, signal=119% |
| 3227 | GOBP\_CELL\_RECOGNITION |  | 86 | -0.33 | -1.17 | 0.212 | 0.572 | 1.000 | 2638 | tags=24%, list=22%, signal=31% |
| 3228 | GOCC\_COMMITMENT\_COMPLEX |  | 5 | -0.61 | -1.17 | 0.265 | 0.572 | 1.000 | 1894 | tags=40%, list=16%, signal=47% |
| 3229 | GOBP\_REGULATION\_OF\_CIRCADIAN\_RHYTHM |  | 82 | -0.33 | -1.17 | 0.203 | 0.572 | 1.000 | 4571 | tags=48%, list=38%, signal=76% |
| 3230 | GOBP\_POSITIVE\_REGULATION\_OF\_CIRCADIAN\_SLEEP\_WAKE\_CYCLE\_SLEEP |  | 3 | -0.70 | -1.17 | 0.246 | 0.572 | 1.000 | 2351 | tags=67%, list=20%, signal=83% |
| 3231 | GOBP\_REGULATION\_OF\_CARDIAC\_CONDUCTION |  | 28 | -0.39 | -1.17 | 0.262 | 0.572 | 1.000 | 5191 | tags=46%, list=43%, signal=82% |
| 3232 | GOBP\_REGULATION\_OF\_FIBROBLAST\_MIGRATION |  | 28 | -0.39 | -1.17 | 0.257 | 0.572 | 1.000 | 3613 | tags=39%, list=30%, signal=56% |
| 3233 | GOBP\_NEGATIVE\_REGULATION\_OF\_CELL\_CYCLE\_PROCESS |  | 262 | -0.30 | -1.17 | 0.120 | 0.572 | 1.000 | 4778 | tags=42%, list=40%, signal=68% |
| 3234 | GOBP\_REGULATION\_OF\_ADAPTIVE\_IMMUNE\_RESPONSE |  | 95 | -0.33 | -1.17 | 0.197 | 0.572 | 1.000 | 3344 | tags=40%, list=28%, signal=55% |
| 3235 | GOBP\_POSITIVE\_REGULATION\_OF\_HIPPO\_SIGNALING |  | 3 | -0.70 | -1.17 | 0.259 | 0.572 | 1.000 | 1603 | tags=67%, list=13%, signal=77% |
| 3236 | GOBP\_CENTRAL\_NERVOUS\_SYSTEM\_DEVELOPMENT |  | 644 | -0.29 | -1.17 | 0.044 | 0.573 | 1.000 | 3738 | tags=34%, list=31%, signal=47% |
| 3237 | GOCC\_PERIKARYON |  | 88 | -0.33 | -1.17 | 0.206 | 0.573 | 1.000 | 2373 | tags=27%, list=20%, signal=34% |
| 3238 | GOBP\_REGULATION\_OF\_HUMORAL\_IMMUNE\_RESPONSE |  | 38 | -0.37 | -1.17 | 0.240 | 0.573 | 1.000 | 2600 | tags=34%, list=22%, signal=44% |
| 3239 | GOBP\_POSITIVE\_REGULATION\_OF\_FOCAL\_ADHESION\_ASSEMBLY |  | 22 | -0.41 | -1.17 | 0.266 | 0.573 | 1.000 | 3196 | tags=41%, list=27%, signal=56% |
| 3240 | GOCC\_CELL\_CORTEX |  | 219 | -0.31 | -1.17 | 0.132 | 0.573 | 1.000 | 4122 | tags=41%, list=34%, signal=61% |
| 3241 | GOBP\_ENDOTHELIAL\_CELL\_MIGRATION |  | 167 | -0.31 | -1.17 | 0.153 | 0.573 | 1.000 | 3392 | tags=34%, list=28%, signal=46% |
| 3242 | GOBP\_NEGATIVE\_REGULATION\_OF\_PROTEIN\_DEPHOSPHORYLATION |  | 39 | -0.37 | -1.17 | 0.248 | 0.573 | 1.000 | 3547 | tags=36%, list=30%, signal=51% |
| 3243 | GOBP\_POSITIVE\_REGULATION\_OF\_LIPID\_LOCALIZATION |  | 72 | -0.34 | -1.17 | 0.224 | 0.573 | 1.000 | 4164 | tags=43%, list=35%, signal=66% |
| 3244 | GOMF\_OLIGOSACCHARIDE\_BINDING |  | 4 | -0.65 | -1.17 | 0.262 | 0.573 | 1.000 | 3549 | tags=75%, list=30%, signal=106% |
| 3245 | GOBP\_NEGATIVE\_REGULATION\_OF\_ALPHA\_BETA\_T\_CELL\_ACTIVATION |  | 26 | -0.40 | -1.17 | 0.261 | 0.573 | 1.000 | 1951 | tags=35%, list=16%, signal=41% |
| 3246 | GOBP\_CARDIAC\_MUSCLE\_CELL\_MYOBLAST\_DIFFERENTIATION |  | 10 | -0.50 | -1.17 | 0.279 | 0.574 | 1.000 | 2212 | tags=20%, list=18%, signal=24% |
| 3247 | GOMF\_CHEMOKINE\_RECEPTOR\_BINDING |  | 19 | -0.42 | -1.17 | 0.269 | 0.573 | 1.000 | 2894 | tags=53%, list=24%, signal=69% |
| 3248 | GOMF\_LIGAND\_GATED\_CATION\_CHANNEL\_ACTIVITY |  | 56 | -0.35 | -1.17 | 0.231 | 0.573 | 1.000 | 2005 | tags=27%, list=17%, signal=32% |
| 3249 | GOBP\_SMOOTHENED\_SIGNALING\_PATHWAY\_INVOLVED\_IN\_DORSAL\_VENTRAL\_NEURAL\_TUBE\_PATTERNING |  | 9 | -0.52 | -1.17 | 0.272 | 0.573 | 1.000 | 4097 | tags=67%, list=34%, signal=101% |
| 3250 | GOBP\_NEGATIVE\_REGULATION\_OF\_HYDROLASE\_ACTIVITY |  | 249 | -0.30 | -1.17 | 0.118 | 0.573 | 1.000 | 3547 | tags=33%, list=30%, signal=46% |
| 3251 | GOBP\_UBIQUITIN\_DEPENDENT\_SMAD\_PROTEIN\_CATABOLIC\_PROCESS |  | 6 | -0.58 | -1.17 | 0.268 | 0.574 | 1.000 | 882 | tags=33%, list=7%, signal=36% |
| 3252 | GOBP\_TRANSLATIONAL\_READTHROUGH |  | 8 | -0.53 | -1.17 | 0.276 | 0.574 | 1.000 | 3054 | tags=38%, list=25%, signal=50% |
| 3253 | GOBP\_RESPONSE\_TO\_SORBITOL |  | 5 | -0.61 | -1.17 | 0.265 | 0.574 | 1.000 | 2798 | tags=40%, list=23%, signal=52% |
| 3254 | GOBP\_NEGATIVE\_REGULATION\_OF\_MITOTIC\_CELL\_CYCLE |  | 232 | -0.30 | -1.17 | 0.134 | 0.574 | 1.000 | 4076 | tags=35%, list=34%, signal=52% |
| 3255 | GOBP\_REGULATION\_OF\_DNA\_DAMAGE\_RESPONSE\_SIGNAL\_TRANSDUCTION\_BY\_P53\_CLASS\_MEDIATOR |  | 25 | -0.40 | -1.17 | 0.260 | 0.574 | 1.000 | 2695 | tags=40%, list=22%, signal=51% |
| 3256 | GOBP\_LOOP\_OF\_HENLE\_DEVELOPMENT |  | 4 | -0.65 | -1.17 | 0.254 | 0.574 | 1.000 | 112 | tags=25%, list=1%, signal=25% |
| 3257 | GOBP\_PROTEIN\_LOCALIZATION\_TO\_CYTOSKELETON |  | 47 | -0.35 | -1.17 | 0.240 | 0.574 | 1.000 | 2479 | tags=28%, list=21%, signal=35% |
| 3258 | GOBP\_NEGATIVE\_REGULATION\_OF\_NEURON\_DIFFERENTIATION |  | 41 | -0.37 | -1.17 | 0.244 | 0.574 | 1.000 | 2340 | tags=27%, list=19%, signal=33% |
| 3259 | GOBP\_ENTERIC\_NERVOUS\_SYSTEM\_DEVELOPMENT |  | 7 | -0.55 | -1.17 | 0.275 | 0.575 | 1.000 | 28 | tags=14%, list=0%, signal=14% |
| 3260 | GOBP\_POSITIVE\_REGULATION\_OF\_RECEPTOR\_CLUSTERING |  | 5 | -0.60 | -1.17 | 0.274 | 0.575 | 1.000 | 90 | tags=20%, list=1%, signal=20% |
| 3261 | GOBP\_NEGATIVE\_REGULATION\_OF\_CELL\_CYCLE\_PHASE\_TRANSITION |  | 194 | -0.31 | -1.17 | 0.148 | 0.575 | 1.000 | 4393 | tags=37%, list=37%, signal=58% |
| 3262 | GOBP\_REGULATION\_OF\_OSTEOCLAST\_DIFFERENTIATION |  | 32 | -0.38 | -1.17 | 0.247 | 0.575 | 1.000 | 1999 | tags=38%, list=17%, signal=45% |
| 3263 | GOBP\_RESPONSE\_TO\_HEPARIN |  | 6 | -0.57 | -1.17 | 0.277 | 0.575 | 1.000 | 2374 | tags=50%, list=20%, signal=62% |
| 3264 | GOBP\_LONG\_CHAIN\_FATTY\_ACID\_BIOSYNTHETIC\_PROCESS |  | 16 | -0.44 | -1.17 | 0.263 | 0.575 | 1.000 | 2701 | tags=44%, list=22%, signal=56% |
| 3265 | GOBP\_CERAMIDE\_CATABOLIC\_PROCESS |  | 13 | -0.47 | -1.16 | 0.272 | 0.575 | 1.000 | 4283 | tags=62%, list=36%, signal=96% |
| 3266 | GOBP\_NUCLEAR\_PORE\_ORGANIZATION |  | 13 | -0.47 | -1.16 | 0.282 | 0.575 | 1.000 | 4770 | tags=69%, list=40%, signal=115% |
| 3267 | GOBP\_COHESIN\_LOADING |  | 4 | -0.65 | -1.16 | 0.266 | 0.575 | 1.000 | 3836 | tags=75%, list=32%, signal=110% |
| 3268 | GOBP\_RETROGRADE\_TRANS\_SYNAPTIC\_SIGNALING |  | 7 | -0.55 | -1.16 | 0.276 | 0.576 | 1.000 | 5432 | tags=100%, list=45%, signal=182% |
| 3269 | GOBP\_NEGATIVE\_REGULATION\_OF\_SISTER\_CHROMATID\_COHESION |  | 4 | -0.64 | -1.16 | 0.261 | 0.576 | 1.000 | 3241 | tags=75%, list=27%, signal=103% |
| 3270 | GOMF\_SINGLE\_STRANDED\_DNA\_BINDING |  | 90 | -0.33 | -1.16 | 0.209 | 0.576 | 1.000 | 5700 | tags=67%, list=47%, signal=126% |
| 3271 | GOMF\_EXONUCLEASE\_ACTIVITY |  | 65 | -0.34 | -1.16 | 0.224 | 0.576 | 1.000 | 5337 | tags=51%, list=44%, signal=91% |
| 3272 | GOBP\_REGULATION\_OF\_WNT\_SIGNALING\_PATHWAY |  | 262 | -0.30 | -1.16 | 0.124 | 0.576 | 1.000 | 3565 | tags=33%, list=30%, signal=46% |
| 3273 | GOBP\_FAT\_SOLUBLE\_VITAMIN\_BIOSYNTHETIC\_PROCESS |  | 10 | -0.50 | -1.16 | 0.281 | 0.576 | 1.000 | 5375 | tags=80%, list=45%, signal=145% |
| 3274 | GOMF\_INSULIN\_LIKE\_GROWTH\_FACTOR\_RECEPTOR\_BINDING |  | 12 | -0.48 | -1.16 | 0.275 | 0.577 | 1.000 | 747 | tags=25%, list=6%, signal=27% |
| 3275 | GOBP\_N\_ACYLPHOSPHATIDYLETHANOLAMINE\_METABOLIC\_PROCESS |  | 3 | -0.69 | -1.16 | 0.257 | 0.577 | 1.000 | 3218 | tags=67%, list=27%, signal=91% |
| 3276 | GOBP\_BETA\_CATENIN\_DESTRUCTION\_COMPLEX\_DISASSEMBLY |  | 13 | -0.46 | -1.16 | 0.280 | 0.577 | 1.000 | 3459 | tags=38%, list=29%, signal=54% |
| 3277 | GOBP\_DEVELOPMENTAL\_PROCESS\_INVOLVED\_IN\_REPRODUCTION |  | 526 | -0.30 | -1.16 | 0.056 | 0.577 | 1.000 | 4521 | tags=45%, list=38%, signal=69% |
| 3278 | GOBP\_PLACENTA\_BLOOD\_VESSEL\_DEVELOPMENT |  | 22 | -0.41 | -1.16 | 0.269 | 0.577 | 1.000 | 3613 | tags=41%, list=30%, signal=58% |
| 3279 | GOCC\_ESC\_E\_Z\_COMPLEX |  | 12 | -0.47 | -1.16 | 0.283 | 0.577 | 1.000 | 5049 | tags=67%, list=42%, signal=115% |
| 3280 | GOBP\_NEGATIVE\_REGULATION\_OF\_WOUND\_HEALING |  | 43 | -0.36 | -1.16 | 0.243 | 0.577 | 1.000 | 3551 | tags=40%, list=30%, signal=56% |
| 3281 | GOMF\_MAP\_KINASE\_ACTIVITY |  | 11 | -0.48 | -1.16 | 0.272 | 0.577 | 1.000 | 441 | tags=18%, list=4%, signal=19% |
| 3282 | GOMF\_HYDROLASE\_ACTIVITY\_ACTING\_ON\_ESTER\_BONDS |  | 519 | -0.30 | -1.16 | 0.054 | 0.577 | 1.000 | 3741 | tags=35%, list=31%, signal=49% |
| 3283 | GOMF\_AMINO\_ACID\_SODIUM\_SYMPORTER\_ACTIVITY |  | 10 | -0.50 | -1.16 | 0.277 | 0.577 | 1.000 | 1222 | tags=30%, list=10%, signal=33% |
| 3284 | GOMF\_TRANSFERASE\_ACTIVITY\_TRANSFERRING\_GLYCOSYL\_GROUPS |  | 185 | -0.31 | -1.16 | 0.146 | 0.577 | 1.000 | 3562 | tags=34%, list=30%, signal=47% |
| 3285 | GOBP\_MUSCLE\_HYPERTROPHY\_IN\_RESPONSE\_TO\_STRESS |  | 15 | -0.44 | -1.16 | 0.277 | 0.577 | 1.000 | 2267 | tags=40%, list=19%, signal=49% |
| 3286 | GOBP\_POSITIVE\_REGULATION\_OF\_T\_CELL\_DIFFERENTIATION\_IN\_THYMUS |  | 9 | -0.51 | -1.16 | 0.288 | 0.577 | 1.000 | 1079 | tags=44%, list=9%, signal=49% |
| 3287 | GOMF\_RNA\_7\_METHYLGUANOSINE\_CAP\_BINDING |  | 10 | -0.49 | -1.16 | 0.279 | 0.577 | 1.000 | 3797 | tags=40%, list=32%, signal=58% |
| 3288 | GOBP\_REGULATION\_OF\_HISTONE\_H4\_K16\_ACETYLATION |  | 5 | -0.60 | -1.16 | 0.267 | 0.577 | 1.000 | 2656 | tags=60%, list=22%, signal=77% |
| 3289 | GOBP\_REGULATION\_OF\_RESPONSE\_TO\_CYTOKINE\_STIMULUS |  | 113 | -0.32 | -1.16 | 0.193 | 0.577 | 1.000 | 2606 | tags=29%, list=22%, signal=37% |
| 3290 | GOCC\_RUFFLE |  | 135 | -0.31 | -1.16 | 0.180 | 0.577 | 1.000 | 4540 | tags=48%, list=38%, signal=77% |
| 3291 | GOBP\_HISTONE\_H4\_R3\_METHYLATION |  | 3 | -0.70 | -1.16 | 0.257 | 0.578 | 1.000 | 3647 | tags=100%, list=30%, signal=144% |
| 3292 | GOBP\_DNA\_DEMETHYLATION |  | 14 | -0.46 | -1.16 | 0.277 | 0.578 | 1.000 | 2526 | tags=36%, list=21%, signal=45% |
| 3293 | GOBP\_REGULATION\_OF\_SODIUM\_ION\_TRANSMEMBRANE\_TRANSPORT |  | 41 | -0.37 | -1.16 | 0.246 | 0.578 | 1.000 | 4286 | tags=54%, list=36%, signal=83% |
| 3294 | GOBP\_REGULATION\_OF\_CHEMOKINE\_MEDIATED\_SIGNALING\_PATHWAY |  | 8 | -0.53 | -1.16 | 0.285 | 0.578 | 1.000 | 4696 | tags=63%, list=39%, signal=103% |
| 3295 | GOBP\_SYNAPTIC\_VESICLE\_RECYCLING\_VIA\_ENDOSOME |  | 7 | -0.55 | -1.16 | 0.281 | 0.578 | 1.000 | 1478 | tags=43%, list=12%, signal=49% |
| 3296 | GOBP\_CELLULAR\_RESPONSE\_TO\_PROSTAGLANDIN\_STIMULUS |  | 16 | -0.44 | -1.16 | 0.274 | 0.578 | 1.000 | 3613 | tags=56%, list=30%, signal=80% |
| 3297 | GOBP\_NEGATIVE\_REGULATION\_OF\_TRANSMEMBRANE\_RECEPTOR\_PROTEIN\_SERINE\_THREONINE\_KINASE\_SIGNALING\_PATHWAY |  | 87 | -0.33 | -1.16 | 0.221 | 0.578 | 1.000 | 3530 | tags=38%, list=29%, signal=53% |
| 3298 | GOBP\_MUSCLE\_STRUCTURE\_DEVELOPMENT |  | 386 | -0.30 | -1.16 | 0.093 | 0.578 | 1.000 | 3205 | tags=30%, list=27%, signal=39% |
| 3299 | GOBP\_PROTEIN\_MODIFICATION\_BY\_SMALL\_PROTEIN\_CONJUGATION\_OR\_REMOVAL |  | 814 | -0.29 | -1.16 | 0.027 | 0.578 | 1.000 | 4645 | tags=42%, list=39%, signal=64% |
| 3300 | GOBP\_POSITIVE\_REGULATION\_OF\_STEROID\_HORMONE\_SECRETION |  | 4 | -0.64 | -1.16 | 0.264 | 0.578 | 1.000 | 4164 | tags=75%, list=35%, signal=115% |
| 3301 | GOMF\_DNA\_INSERTION\_OR\_DELETION\_BINDING |  | 6 | -0.57 | -1.16 | 0.280 | 0.578 | 1.000 | 2443 | tags=50%, list=20%, signal=63% |
| 3302 | GOBP\_MYOTUBE\_DIFFERENTIATION\_INVOLVED\_IN\_SKELETAL\_MUSCLE\_REGENERATION |  | 5 | -0.61 | -1.16 | 0.280 | 0.578 | 1.000 | 280 | tags=20%, list=2%, signal=20% |
| 3303 | GOBP\_DICARBOXYLIC\_ACID\_TRANSPORT |  | 62 | -0.34 | -1.16 | 0.230 | 0.578 | 1.000 | 2313 | tags=31%, list=19%, signal=38% |
| 3304 | GOBP\_POSTSYNAPTIC\_ACTIN\_CYTOSKELETON\_ORGANIZATION |  | 6 | -0.57 | -1.16 | 0.278 | 0.578 | 1.000 | 2546 | tags=33%, list=21%, signal=42% |
| 3305 | GOBP\_RIBONUCLEOTIDE\_CATABOLIC\_PROCESS |  | 29 | -0.38 | -1.16 | 0.259 | 0.578 | 1.000 | 2574 | tags=34%, list=21%, signal=44% |
| 3306 | GOCC\_CELL\_LEADING\_EDGE |  | 308 | -0.30 | -1.16 | 0.111 | 0.578 | 1.000 | 2995 | tags=31%, list=25%, signal=40% |
| 3307 | GOBP\_POSITIVE\_REGULATION\_OF\_3\_UTR\_MEDIATED\_MRNA\_STABILIZATION |  | 3 | -0.70 | -1.16 | 0.261 | 0.578 | 1.000 | 75 | tags=33%, list=1%, signal=34% |
| 3308 | GOBP\_DNA\_REPLICATION\_SYNTHESIS\_OF\_RNA\_PRIMER |  | 5 | -0.60 | -1.16 | 0.267 | 0.578 | 1.000 | 2853 | tags=60%, list=24%, signal=79% |
| 3309 | GOMF\_CUPROUS\_ION\_BINDING |  | 6 | -0.57 | -1.16 | 0.278 | 0.578 | 1.000 | 756 | tags=17%, list=6%, signal=18% |
| 3310 | GOBP\_CARBOHYDRATE\_CATABOLIC\_PROCESS |  | 148 | -0.31 | -1.16 | 0.178 | 0.579 | 1.000 | 4291 | tags=47%, list=36%, signal=72% |
| 3311 | GOCC\_ACTOMYOSIN |  | 57 | -0.34 | -1.16 | 0.233 | 0.579 | 1.000 | 3086 | tags=25%, list=26%, signal=33% |
| 3312 | GOBP\_PIRNA\_METABOLIC\_PROCESS |  | 7 | -0.55 | -1.16 | 0.283 | 0.579 | 1.000 | 529 | tags=29%, list=4%, signal=30% |
| 3313 | GOBP\_VENTRICULAR\_TRABECULA\_MYOCARDIUM\_MORPHOGENESIS |  | 12 | -0.48 | -1.16 | 0.284 | 0.579 | 1.000 | 3615 | tags=50%, list=30%, signal=71% |
| 3314 | GOBP\_SULFUR\_AMINO\_ACID\_TRANSPORT |  | 6 | -0.57 | -1.16 | 0.266 | 0.579 | 1.000 | 1222 | tags=50%, list=10%, signal=56% |
| 3315 | GOBP\_NEGATIVE\_REGULATION\_OF\_HEMOPOIESIS |  | 64 | -0.34 | -1.16 | 0.232 | 0.579 | 1.000 | 1951 | tags=25%, list=16%, signal=30% |
| 3316 | GOBP\_RESPONSE\_TO\_CARBOHYDRATE |  | 150 | -0.31 | -1.16 | 0.184 | 0.579 | 1.000 | 3433 | tags=37%, list=29%, signal=51% |
| 3317 | GOBP\_POSITIVE\_REGULATION\_OF\_CALCIUM\_ION\_IMPORT |  | 9 | -0.51 | -1.16 | 0.277 | 0.579 | 1.000 | 3286 | tags=44%, list=27%, signal=61% |
| 3318 | GOMF\_INOSITOL\_TRISPHOSPHATE\_KINASE\_ACTIVITY |  | 4 | -0.65 | -1.16 | 0.273 | 0.580 | 1.000 | 3760 | tags=75%, list=31%, signal=109% |
| 3319 | GOMF\_ANDROGEN\_RECEPTOR\_BINDING |  | 22 | -0.41 | -1.16 | 0.273 | 0.580 | 1.000 | 4358 | tags=45%, list=36%, signal=71% |
| 3320 | GOBP\_INOSITOL\_TRISPHOSPHATE\_METABOLIC\_PROCESS |  | 8 | -0.53 | -1.16 | 0.287 | 0.580 | 1.000 | 1869 | tags=38%, list=16%, signal=44% |
| 3321 | GOCC\_ZYMOGEN\_GRANULE |  | 7 | -0.54 | -1.16 | 0.282 | 0.580 | 1.000 | 3273 | tags=43%, list=27%, signal=59% |
| 3322 | GOBP\_SELECTIVE\_AUTOPHAGY |  | 51 | -0.35 | -1.16 | 0.240 | 0.580 | 1.000 | 3566 | tags=37%, list=30%, signal=53% |
| 3323 | GOMF\_N6\_METHYLADENOSINE\_CONTAINING\_RNA\_BINDING |  | 6 | -0.57 | -1.16 | 0.291 | 0.580 | 1.000 | 4922 | tags=83%, list=41%, signal=141% |
| 3324 | GOBP\_AXIS\_ELONGATION\_INVOLVED\_IN\_SOMITOGENESIS |  | 4 | -0.64 | -1.16 | 0.275 | 0.580 | 1.000 | 3560 | tags=75%, list=30%, signal=107% |
| 3325 | GOBP\_POSTSYNAPSE\_ASSEMBLY |  | 18 | -0.43 | -1.16 | 0.274 | 0.580 | 1.000 | 3699 | tags=50%, list=31%, signal=72% |
| 3326 | GOBP\_CELLULAR\_RESPONSE\_TO\_LOW\_DENSITY\_LIPOPROTEIN\_PARTICLE\_STIMULUS |  | 17 | -0.43 | -1.16 | 0.282 | 0.581 | 1.000 | 3403 | tags=53%, list=28%, signal=74% |
| 3327 | GOBP\_REGULATION\_OF\_DNA\_DEPENDENT\_DNA\_REPLICATION |  | 43 | -0.36 | -1.16 | 0.254 | 0.581 | 1.000 | 5430 | tags=56%, list=45%, signal=102% |
| 3328 | GOBP\_REGULATION\_OF\_HUMORAL\_IMMUNE\_RESPONSE\_MEDIATED\_BY\_CIRCULATING\_IMMUNOGLOBULIN |  | 6 | -0.57 | -1.16 | 0.279 | 0.581 | 1.000 | 3944 | tags=67%, list=33%, signal=99% |
| 3329 | GOCC\_BLOC\_COMPLEX |  | 18 | -0.43 | -1.16 | 0.280 | 0.580 | 1.000 | 3569 | tags=50%, list=30%, signal=71% |
| 3330 | GOBP\_SYNAPTIC\_VESICLE\_ENDOSOMAL\_PROCESSING |  | 9 | -0.52 | -1.16 | 0.279 | 0.580 | 1.000 | 4869 | tags=78%, list=41%, signal=131% |
| 3331 | GOBP\_MELANOCYTE\_DIFFERENTIATION |  | 18 | -0.43 | -1.16 | 0.278 | 0.580 | 1.000 | 2650 | tags=39%, list=22%, signal=50% |
| 3332 | GOMF\_HYDROLASE\_ACTIVITY\_ACTING\_ON\_ACID\_ANHYDRIDES |  | 636 | -0.29 | -1.16 | 0.046 | 0.581 | 1.000 | 4673 | tags=42%, list=39%, signal=66% |
| 3333 | GOBP\_DNA\_TOPOLOGICAL\_CHANGE |  | 9 | -0.51 | -1.16 | 0.286 | 0.581 | 1.000 | 4649 | tags=67%, list=39%, signal=109% |
| 3334 | GOBP\_REGULATION\_OF\_SMOOTH\_MUSCLE\_CELL\_CHEMOTAXIS |  | 4 | -0.64 | -1.16 | 0.282 | 0.581 | 1.000 | 1734 | tags=25%, list=14%, signal=29% |
| 3335 | GOBP\_SYNAPTIC\_VESICLE\_LOCALIZATION |  | 38 | -0.36 | -1.16 | 0.257 | 0.581 | 1.000 | 4369 | tags=55%, list=36%, signal=87% |
| 3336 | GOBP\_POSITIVE\_REGULATION\_OF\_CYCLASE\_ACTIVITY |  | 13 | -0.46 | -1.16 | 0.289 | 0.581 | 1.000 | 1692 | tags=31%, list=14%, signal=36% |
| 3337 | GOBP\_CARBOXYLIC\_ACID\_TRANSPORT |  | 174 | -0.31 | -1.16 | 0.168 | 0.582 | 1.000 | 3022 | tags=35%, list=25%, signal=46% |
| 3338 | GOBP\_REGULATION\_OF\_CELL\_MIGRATION\_INVOLVED\_IN\_SPROUTING\_ANGIOGENESIS |  | 30 | -0.38 | -1.16 | 0.260 | 0.582 | 1.000 | 3196 | tags=37%, list=27%, signal=50% |
| 3339 | GOBP\_MUSCLE\_CELL\_APOPTOTIC\_PROCESS |  | 41 | -0.36 | -1.16 | 0.251 | 0.582 | 1.000 | 3392 | tags=41%, list=28%, signal=58% |
| 3340 | GOBP\_COLLAGEN\_FIBRIL\_ORGANIZATION |  | 40 | -0.36 | -1.16 | 0.257 | 0.582 | 1.000 | 5324 | tags=58%, list=44%, signal=103% |
| 3341 | GOBP\_REGULATION\_OF\_SMOOTHENED\_SIGNALING\_PATHWAY |  | 59 | -0.34 | -1.16 | 0.243 | 0.583 | 1.000 | 3836 | tags=39%, list=32%, signal=57% |
| 3342 | GOBP\_TRIGLYCERIDE\_BIOSYNTHETIC\_PROCESS |  | 30 | -0.38 | -1.16 | 0.264 | 0.583 | 1.000 | 2667 | tags=33%, list=22%, signal=43% |
| 3343 | GOBP\_THYMUS\_DEVELOPMENT |  | 33 | -0.38 | -1.16 | 0.265 | 0.583 | 1.000 | 3966 | tags=45%, list=33%, signal=68% |
| 3344 | GOCC\_SWI\_SNF\_SUPERFAMILY\_TYPE\_COMPLEX |  | 55 | -0.35 | -1.16 | 0.247 | 0.583 | 1.000 | 5049 | tags=51%, list=42%, signal=87% |
| 3345 | GOMF\_SEQUENCE\_SPECIFIC\_SINGLE\_STRANDED\_DNA\_BINDING |  | 12 | -0.47 | -1.16 | 0.283 | 0.583 | 1.000 | 5648 | tags=83%, list=47%, signal=157% |
| 3346 | GOBP\_ENDOTHELIAL\_CELL\_PROLIFERATION |  | 109 | -0.32 | -1.16 | 0.201 | 0.583 | 1.000 | 3473 | tags=36%, list=29%, signal=50% |
| 3347 | GOCC\_NEUROFIBRILLARY\_TANGLE |  | 4 | -0.64 | -1.16 | 0.271 | 0.583 | 1.000 | 4369 | tags=100%, list=36%, signal=157% |
| 3348 | GOBP\_TUBULIN\_DEACETYLATION |  | 9 | -0.51 | -1.16 | 0.291 | 0.583 | 1.000 | 4528 | tags=67%, list=38%, signal=107% |
| 3349 | GOBP\_TRABECULA\_MORPHOGENESIS |  | 33 | -0.38 | -1.16 | 0.261 | 0.584 | 1.000 | 3615 | tags=36%, list=30%, signal=52% |
| 3350 | GOBP\_CATECHOL\_CONTAINING\_COMPOUND\_METABOLIC\_PROCESS |  | 29 | -0.38 | -1.16 | 0.270 | 0.584 | 1.000 | 2569 | tags=38%, list=21%, signal=48% |
| 3351 | GOBP\_BRAINSTEM\_DEVELOPMENT |  | 4 | -0.64 | -1.16 | 0.279 | 0.584 | 1.000 | 3733 | tags=75%, list=31%, signal=109% |
| 3352 | GOBP\_SPERMATOGENESIS\_EXCHANGE\_OF\_CHROMOSOMAL\_PROTEINS |  | 4 | -0.64 | -1.16 | 0.268 | 0.584 | 1.000 | 4298 | tags=75%, list=36%, signal=117% |
| 3353 | GOBP\_REACTIVE\_NITROGEN\_SPECIES\_METABOLIC\_PROCESS |  | 51 | -0.35 | -1.16 | 0.246 | 0.584 | 1.000 | 2518 | tags=31%, list=21%, signal=40% |
| 3354 | GOMF\_MDM2\_MDM4\_FAMILY\_PROTEIN\_BINDING |  | 5 | -0.60 | -1.16 | 0.287 | 0.584 | 1.000 | 2819 | tags=40%, list=23%, signal=52% |
| 3355 | GOBP\_REGULATION\_OF\_TYPE\_2\_IMMUNE\_RESPONSE |  | 19 | -0.42 | -1.16 | 0.280 | 0.584 | 1.000 | 1788 | tags=42%, list=15%, signal=49% |
| 3356 | GOBP\_SEMI\_LUNAR\_VALVE\_DEVELOPMENT |  | 33 | -0.38 | -1.16 | 0.265 | 0.584 | 1.000 | 2416 | tags=30%, list=20%, signal=38% |
| 3357 | GOBP\_POSITIVE\_REGULATION\_OF\_CORTICOSTEROID\_HORMONE\_SECRETION |  | 4 | -0.64 | -1.16 | 0.271 | 0.585 | 1.000 | 4164 | tags=75%, list=35%, signal=115% |
| 3358 | GOCC\_RECYCLING\_ENDOSOME\_MEMBRANE |  | 63 | -0.34 | -1.16 | 0.235 | 0.585 | 1.000 | 3728 | tags=37%, list=31%, signal=53% |
| 3359 | GOBP\_LIPID\_BIOSYNTHETIC\_PROCESS |  | 523 | -0.29 | -1.16 | 0.062 | 0.585 | 1.000 | 4540 | tags=44%, list=38%, signal=67% |
| 3360 | GOMF\_AP\_1\_ADAPTOR\_COMPLEX\_BINDING |  | 3 | -0.69 | -1.16 | 0.267 | 0.585 | 1.000 | 3716 | tags=100%, list=31%, signal=145% |
| 3361 | GOBP\_POSITIVE\_REGULATION\_OF\_DEPHOSPHORYLATION |  | 44 | -0.36 | -1.16 | 0.259 | 0.585 | 1.000 | 4627 | tags=50%, list=39%, signal=81% |
| 3362 | GOMF\_C\_C\_CHEMOKINE\_BINDING |  | 12 | -0.47 | -1.16 | 0.292 | 0.585 | 1.000 | 2871 | tags=58%, list=24%, signal=77% |
| 3363 | GOBP\_REGULATION\_OF\_MONOCYTE\_CHEMOTAXIS |  | 16 | -0.44 | -1.16 | 0.278 | 0.585 | 1.000 | 2679 | tags=44%, list=22%, signal=56% |
| 3364 | GOBP\_SHORT\_TERM\_MEMORY |  | 5 | -0.60 | -1.16 | 0.282 | 0.585 | 1.000 | 3092 | tags=60%, list=26%, signal=81% |
| 3365 | GOBP\_REGULATION\_OF\_PEPTIDYL\_LYSINE\_ACETYLATION |  | 49 | -0.35 | -1.16 | 0.249 | 0.585 | 1.000 | 3885 | tags=39%, list=32%, signal=57% |
| 3366 | GOBP\_POSITIVE\_REGULATION\_OF\_RECEPTOR\_BINDING |  | 5 | -0.60 | -1.16 | 0.283 | 0.585 | 1.000 | 2560 | tags=60%, list=21%, signal=76% |
| 3367 | GOCC\_NONHOMOLOGOUS\_END\_JOINING\_COMPLEX |  | 8 | -0.52 | -1.16 | 0.290 | 0.585 | 1.000 | 4024 | tags=75%, list=34%, signal=113% |
| 3368 | GOBP\_POSITIVE\_REGULATION\_OF\_CHROMATIN\_ASSEMBLY\_OR\_DISASSEMBLY |  | 8 | -0.53 | -1.16 | 0.293 | 0.585 | 1.000 | 1039 | tags=25%, list=9%, signal=27% |
| 3369 | GOBP\_POSITIVE\_REGULATION\_OF\_CALCIUM\_MEDIATED\_SIGNALING |  | 21 | -0.41 | -1.16 | 0.279 | 0.585 | 1.000 | 2938 | tags=48%, list=24%, signal=63% |
| 3370 | GOBP\_PIGMENT\_GRANULE\_ORGANIZATION |  | 20 | -0.42 | -1.16 | 0.285 | 0.585 | 1.000 | 3569 | tags=50%, list=30%, signal=71% |
| 3371 | GOBP\_REGULATION\_OF\_STRIATED\_MUSCLE\_CONTRACTION |  | 50 | -0.35 | -1.16 | 0.253 | 0.585 | 1.000 | 3968 | tags=42%, list=33%, signal=62% |
| 3372 | GOBP\_POSITIVE\_REGULATION\_OF\_VASCULAR\_ENDOTHELIAL\_GROWTH\_FACTOR\_PRODUCTION |  | 21 | -0.41 | -1.15 | 0.276 | 0.585 | 1.000 | 2600 | tags=33%, list=22%, signal=42% |
| 3373 | GOMF\_RETINOL\_BINDING |  | 9 | -0.51 | -1.15 | 0.282 | 0.585 | 1.000 | 3316 | tags=56%, list=28%, signal=77% |
| 3374 | GOCC\_CALCIUM\_AND\_CALMODULIN\_DEPENDENT\_PROTEIN\_KINASE\_COMPLEX |  | 4 | -0.64 | -1.15 | 0.279 | 0.585 | 1.000 | 4282 | tags=75%, list=36%, signal=117% |
| 3375 | GOBP\_LEUKOCYTE\_ADHESION\_TO\_VASCULAR\_ENDOTHELIAL\_CELL |  | 30 | -0.38 | -1.15 | 0.271 | 0.585 | 1.000 | 4171 | tags=53%, list=35%, signal=82% |
| 3376 | GOBP\_REGULATION\_OF\_STEM\_CELL\_PROLIFERATION |  | 28 | -0.39 | -1.15 | 0.271 | 0.585 | 1.000 | 3400 | tags=36%, list=28%, signal=50% |
| 3377 | GOCC\_REPLICATION\_FORK |  | 53 | -0.35 | -1.15 | 0.248 | 0.586 | 1.000 | 3744 | tags=38%, list=31%, signal=55% |
| 3378 | GOCC\_CLATHRIN\_COAT\_OF\_ENDOCYTIC\_VESICLE |  | 7 | -0.54 | -1.15 | 0.289 | 0.586 | 1.000 | 4775 | tags=86%, list=40%, signal=142% |
| 3379 | GOBP\_REGULATION\_OF\_ENDOPLASMIC\_RETICULUM\_STRESS\_INDUCED\_INTRINSIC\_APOPTOTIC\_SIGNALING\_PATHWAY |  | 21 | -0.41 | -1.15 | 0.270 | 0.586 | 1.000 | 3966 | tags=57%, list=33%, signal=85% |
| 3380 | GOBP\_POSITIVE\_REGULATION\_OF\_LYASE\_ACTIVITY |  | 13 | -0.46 | -1.15 | 0.288 | 0.586 | 1.000 | 1292 | tags=23%, list=11%, signal=26% |
| 3381 | GOBP\_MAINTENANCE\_OF\_PROTEIN\_LOCALIZATION\_IN\_ENDOPLASMIC\_RETICULUM |  | 8 | -0.53 | -1.15 | 0.288 | 0.586 | 1.000 | 2313 | tags=38%, list=19%, signal=46% |
| 3382 | GOBP\_REGULATION\_OF\_CARBOHYDRATE\_METABOLIC\_PROCESS |  | 148 | -0.31 | -1.15 | 0.194 | 0.586 | 1.000 | 4649 | tags=45%, list=39%, signal=73% |
| 3383 | GOBP\_DEFENSE\_RESPONSE |  | 843 | -0.29 | -1.15 | 0.033 | 0.586 | 1.000 | 3166 | tags=32%, list=26%, signal=40% |
| 3384 | GOBP\_POSITIVE\_REGULATION\_OF\_EPITHELIAL\_TO\_MESENCHYMAL\_TRANSITION |  | 42 | -0.36 | -1.15 | 0.259 | 0.586 | 1.000 | 3005 | tags=33%, list=25%, signal=44% |
| 3385 | GOMF\_L\_LEUCINE\_TRANSMEMBRANE\_TRANSPORTER\_ACTIVITY |  | 3 | -0.69 | -1.15 | 0.273 | 0.586 | 1.000 | 3155 | tags=67%, list=26%, signal=90% |
| 3386 | GOBP\_SIGNALING\_RECEPTOR\_LIGAND\_PRECURSOR\_PROCESSING |  | 19 | -0.42 | -1.15 | 0.278 | 0.587 | 1.000 | 2664 | tags=42%, list=22%, signal=54% |
| 3387 | GOMF\_INORGANIC\_PHOSPHATE\_TRANSMEMBRANE\_TRANSPORTER\_ACTIVITY |  | 4 | -0.64 | -1.15 | 0.276 | 0.586 | 1.000 | 2395 | tags=50%, list=20%, signal=62% |
| 3388 | GOBP\_NEGATIVE\_REGULATION\_OF\_GLIAL\_CELL\_APOPTOTIC\_PROCESS |  | 7 | -0.54 | -1.15 | 0.296 | 0.586 | 1.000 | 3308 | tags=43%, list=28%, signal=59% |
| 3389 | GOBP\_THYROID\_HORMONE\_GENERATION |  | 9 | -0.51 | -1.15 | 0.289 | 0.587 | 1.000 | 1253 | tags=33%, list=10%, signal=37% |
| 3390 | GOMF\_LOW\_DENSITY\_LIPOPROTEIN\_PARTICLE\_RECEPTOR\_ACTIVITY |  | 11 | -0.48 | -1.15 | 0.287 | 0.587 | 1.000 | 3794 | tags=55%, list=32%, signal=80% |
| 3391 | GOBP\_MESENCHYMAL\_CELL\_DIFFERENTIATION |  | 170 | -0.31 | -1.15 | 0.187 | 0.587 | 1.000 | 3565 | tags=34%, list=30%, signal=47% |
| 3392 | GOBP\_EPITHELIUM\_DEVELOPMENT |  | 740 | -0.29 | -1.15 | 0.038 | 0.587 | 1.000 | 3565 | tags=32%, list=30%, signal=43% |
| 3393 | GOBP\_CALCIUM\_ION\_EXPORT\_ACROSS\_PLASMA\_MEMBRANE |  | 4 | -0.64 | -1.15 | 0.268 | 0.587 | 1.000 | 3881 | tags=75%, list=32%, signal=111% |
| 3394 | GOBP\_POSITIVE\_REGULATION\_OF\_SMOOTH\_MUSCLE\_CELL\_DIFFERENTIATION |  | 10 | -0.49 | -1.15 | 0.289 | 0.588 | 1.000 | 435 | tags=20%, list=4%, signal=21% |
| 3395 | GOBP\_REGULATION\_OF\_HYALURONAN\_BIOSYNTHETIC\_PROCESS |  | 6 | -0.57 | -1.15 | 0.293 | 0.587 | 1.000 | 4433 | tags=67%, list=37%, signal=106% |
| 3396 | GOBP\_REGULATION\_OF\_ACTIN\_NUCLEATION |  | 21 | -0.41 | -1.15 | 0.281 | 0.588 | 1.000 | 3797 | tags=48%, list=32%, signal=70% |
| 3397 | GOBP\_POSITIVE\_REGULATION\_OF\_PROTEIN\_DEPHOSPHORYLATION |  | 35 | -0.37 | -1.15 | 0.270 | 0.588 | 1.000 | 4627 | tags=49%, list=39%, signal=79% |
| 3398 | GOBP\_MULTICELLULAR\_ORGANISM\_REPRODUCTION |  | 421 | -0.29 | -1.15 | 0.097 | 0.588 | 1.000 | 4662 | tags=46%, list=39%, signal=73% |
| 3399 | GOBP\_POSITIVE\_REGULATION\_OF\_NEURON\_DIFFERENTIATION |  | 55 | -0.34 | -1.15 | 0.253 | 0.588 | 1.000 | 3607 | tags=40%, list=30%, signal=57% |
| 3400 | GOBP\_FATTY\_ACID\_DERIVATIVE\_BIOSYNTHETIC\_PROCESS |  | 42 | -0.36 | -1.15 | 0.259 | 0.588 | 1.000 | 4689 | tags=55%, list=39%, signal=90% |
| 3401 | GOMF\_AMINO\_ACID\_TRANSMEMBRANE\_TRANSPORTER\_ACTIVITY |  | 50 | -0.35 | -1.15 | 0.258 | 0.589 | 1.000 | 1246 | tags=22%, list=10%, signal=24% |
| 3402 | GOBP\_NUCLEOSIDE\_MONOPHOSPHATE\_CATABOLIC\_PROCESS |  | 10 | -0.49 | -1.15 | 0.298 | 0.589 | 1.000 | 1107 | tags=40%, list=9%, signal=44% |
| 3403 | GOBP\_REGULATION\_OF\_EOSINOPHIL\_MIGRATION |  | 3 | -0.69 | -1.15 | 0.273 | 0.589 | 1.000 | 2912 | tags=67%, list=24%, signal=88% |
| 3404 | GOBP\_POSITIVE\_REGULATION\_OF\_CHOLESTEROL\_STORAGE |  | 5 | -0.60 | -1.15 | 0.281 | 0.589 | 1.000 | 3403 | tags=80%, list=28%, signal=112% |
| 3405 | GOBP\_REGULATION\_OF\_LONG\_TERM\_SYNAPTIC\_DEPRESSION |  | 7 | -0.54 | -1.15 | 0.296 | 0.589 | 1.000 | 4052 | tags=57%, list=34%, signal=86% |
| 3406 | GOBP\_POLYPHOSPHATE\_METABOLIC\_PROCESS |  | 5 | -0.59 | -1.15 | 0.288 | 0.589 | 1.000 | 490 | tags=40%, list=4%, signal=42% |
| 3407 | GOBP\_POSITIVE\_REGULATION\_OF\_TRANSPORT |  | 608 | -0.29 | -1.15 | 0.061 | 0.589 | 1.000 | 3072 | tags=29%, list=26%, signal=37% |
| 3408 | GOBP\_NEGATIVE\_REGULATION\_OF\_MRNA\_METABOLIC\_PROCESS |  | 57 | -0.34 | -1.15 | 0.250 | 0.589 | 1.000 | 5048 | tags=53%, list=42%, signal=90% |
| 3409 | GOBP\_STRIATED\_MUSCLE\_CELL\_APOPTOTIC\_PROCESS |  | 21 | -0.41 | -1.15 | 0.282 | 0.589 | 1.000 | 5693 | tags=67%, list=47%, signal=127% |
| 3410 | GOBP\_REGULATION\_OF\_CENTRIOLE\_REPLICATION |  | 18 | -0.42 | -1.15 | 0.281 | 0.589 | 1.000 | 5075 | tags=61%, list=42%, signal=106% |
| 3411 | GOBP\_NEGATIVE\_REGULATION\_OF\_CELLULAR\_COMPONENT\_ORGANIZATION |  | 505 | -0.29 | -1.15 | 0.076 | 0.589 | 1.000 | 3724 | tags=34%, list=31%, signal=47% |
| 3412 | GOBP\_REGULATION\_OF\_SPINDLE\_CHECKPOINT |  | 14 | -0.45 | -1.15 | 0.289 | 0.589 | 1.000 | 3331 | tags=36%, list=28%, signal=49% |
| 3413 | GOBP\_LEUKOTRIENE\_D4\_METABOLIC\_PROCESS |  | 3 | -0.69 | -1.15 | 0.284 | 0.589 | 1.000 | 1878 | tags=67%, list=16%, signal=79% |
| 3414 | GOMF\_HISTONE\_THREONINE\_KINASE\_ACTIVITY |  | 5 | -0.60 | -1.15 | 0.290 | 0.590 | 1.000 | 4378 | tags=80%, list=36%, signal=126% |
| 3415 | GOBP\_REGULATION\_OF\_CELLULAR\_EXTRAVASATION |  | 23 | -0.40 | -1.15 | 0.277 | 0.590 | 1.000 | 2011 | tags=35%, list=17%, signal=42% |
| 3416 | GOBP\_RESPONSE\_TO\_PURINE\_CONTAINING\_COMPOUND |  | 94 | -0.32 | -1.15 | 0.227 | 0.590 | 1.000 | 3723 | tags=36%, list=31%, signal=52% |
| 3417 | GOBP\_ENERGY\_RESERVE\_METABOLIC\_PROCESS |  | 59 | -0.34 | -1.15 | 0.251 | 0.591 | 1.000 | 5145 | tags=58%, list=43%, signal=100% |
| 3418 | GOBP\_NUCLEAR\_MIGRATION |  | 18 | -0.42 | -1.15 | 0.284 | 0.591 | 1.000 | 2299 | tags=39%, list=19%, signal=48% |
| 3419 | GOBP\_HEPATOCYTE\_GROWTH\_FACTOR\_RECEPTOR\_SIGNALING\_PATHWAY |  | 14 | -0.45 | -1.15 | 0.294 | 0.591 | 1.000 | 4598 | tags=64%, list=38%, signal=104% |
| 3420 | GOBP\_SPINDLE\_ASSEMBLY |  | 87 | -0.33 | -1.15 | 0.229 | 0.591 | 1.000 | 4589 | tags=44%, list=38%, signal=70% |
| 3421 | GOMF\_GALACTOSIDASE\_ACTIVITY |  | 6 | -0.57 | -1.15 | 0.295 | 0.591 | 1.000 | 3768 | tags=67%, list=31%, signal=97% |
| 3422 | GOBP\_REGULATION\_OF\_POTASSIUM\_ION\_IMPORT |  | 5 | -0.60 | -1.15 | 0.290 | 0.592 | 1.000 | 2955 | tags=60%, list=25%, signal=80% |
| 3423 | GOBP\_KERATINOCYTE\_PROLIFERATION |  | 31 | -0.38 | -1.15 | 0.270 | 0.591 | 1.000 | 1777 | tags=32%, list=15%, signal=38% |
| 3424 | GOMF\_G\_PROTEIN\_COUPLED\_RECEPTOR\_KINASE\_ACTIVITY |  | 5 | -0.60 | -1.15 | 0.286 | 0.592 | 1.000 | 4094 | tags=80%, list=34%, signal=121% |
| 3425 | GOBP\_CARDIOBLAST\_DIFFERENTIATION |  | 13 | -0.46 | -1.15 | 0.297 | 0.591 | 1.000 | 2212 | tags=23%, list=18%, signal=28% |
| 3426 | GOBP\_GLAND\_MORPHOGENESIS |  | 86 | -0.32 | -1.15 | 0.238 | 0.592 | 1.000 | 1816 | tags=22%, list=15%, signal=26% |
| 3427 | GOBP\_TELENCEPHALON\_GLIAL\_CELL\_MIGRATION |  | 15 | -0.44 | -1.15 | 0.303 | 0.592 | 1.000 | 3004 | tags=40%, list=25%, signal=53% |
| 3428 | GOBP\_POSITIVE\_REGULATION\_OF\_AMYLOID\_BETA\_FORMATION |  | 14 | -0.45 | -1.15 | 0.295 | 0.592 | 1.000 | 2416 | tags=29%, list=20%, signal=36% |
| 3429 | GOBP\_NEGATIVE\_REGULATION\_OF\_CELL\_DEATH |  | 638 | -0.29 | -1.15 | 0.057 | 0.592 | 1.000 | 3408 | tags=32%, list=28%, signal=42% |
| 3430 | GOBP\_ENTRY\_OF\_BACTERIUM\_INTO\_HOST\_CELL |  | 13 | -0.46 | -1.15 | 0.296 | 0.592 | 1.000 | 2811 | tags=38%, list=23%, signal=50% |
| 3431 | GOCC\_CYTOPLASMIC\_STRESS\_GRANULE |  | 56 | -0.34 | -1.15 | 0.251 | 0.592 | 1.000 | 5307 | tags=57%, list=44%, signal=102% |
| 3432 | GOBP\_PEPTIDYL\_LYSINE\_DEACETYLATION |  | 6 | -0.57 | -1.15 | 0.295 | 0.592 | 1.000 | 2717 | tags=50%, list=23%, signal=65% |
| 3433 | GOBP\_RESPONSE\_TO\_X\_RAY |  | 24 | -0.39 | -1.15 | 0.280 | 0.592 | 1.000 | 3927 | tags=58%, list=33%, signal=86% |
| 3434 | GOBP\_DETOXIFICATION\_OF\_INORGANIC\_COMPOUND |  | 4 | -0.63 | -1.15 | 0.276 | 0.592 | 1.000 | 3930 | tags=50%, list=33%, signal=74% |
| 3435 | GOBP\_REGULATION\_OF\_FAT\_CELL\_DIFFERENTIATION |  | 89 | -0.32 | -1.15 | 0.231 | 0.592 | 1.000 | 3968 | tags=42%, list=33%, signal=62% |
| 3436 | GOMF\_COLLAGEN\_RECEPTOR\_ACTIVITY |  | 5 | -0.60 | -1.15 | 0.288 | 0.592 | 1.000 | 687 | tags=20%, list=6%, signal=21% |
| 3437 | GOBP\_REGULATION\_OF\_ISOMERASE\_ACTIVITY |  | 6 | -0.56 | -1.15 | 0.286 | 0.592 | 1.000 | 4309 | tags=67%, list=36%, signal=104% |
| 3438 | GOBP\_HISTONE\_THREONINE\_PHOSPHORYLATION |  | 5 | -0.60 | -1.15 | 0.288 | 0.592 | 1.000 | 4378 | tags=80%, list=36%, signal=126% |
| 3439 | GOMF\_SULFUR\_AMINO\_ACID\_TRANSMEMBRANE\_TRANSPORTER\_ACTIVITY |  | 6 | -0.57 | -1.15 | 0.294 | 0.592 | 1.000 | 1222 | tags=50%, list=10%, signal=56% |
| 3440 | GOBP\_NEGATIVE\_REGULATION\_OF\_PROTEIN\_ACETYLATION |  | 17 | -0.43 | -1.15 | 0.287 | 0.593 | 1.000 | 3395 | tags=41%, list=28%, signal=57% |
| 3441 | GOCC\_FHF\_COMPLEX |  | 4 | -0.63 | -1.15 | 0.285 | 0.593 | 1.000 | 1644 | tags=50%, list=14%, signal=58% |
| 3442 | GOBP\_POSITIVE\_REGULATION\_OF\_INTRACELLULAR\_TRANSPORT |  | 159 | -0.31 | -1.15 | 0.184 | 0.592 | 1.000 | 3860 | tags=33%, list=32%, signal=48% |
| 3443 | GOBP\_CELL\_JUNCTION\_DISASSEMBLY |  | 18 | -0.42 | -1.15 | 0.284 | 0.592 | 1.000 | 5246 | tags=67%, list=44%, signal=118% |
| 3444 | GOMF\_CHOLESTEROL\_BINDING |  | 38 | -0.37 | -1.15 | 0.262 | 0.593 | 1.000 | 4280 | tags=45%, list=36%, signal=69% |
| 3445 | GOBP\_POSITIVE\_REGULATION\_OF\_T\_HELPER\_1\_TYPE\_IMMUNE\_RESPONSE |  | 10 | -0.49 | -1.15 | 0.291 | 0.592 | 1.000 | 3487 | tags=50%, list=29%, signal=70% |
| 3446 | GOBP\_POSITIVE\_REGULATION\_OF\_CELL\_SIZE |  | 8 | -0.52 | -1.15 | 0.293 | 0.593 | 1.000 | 2356 | tags=38%, list=20%, signal=47% |
| 3447 | GOBP\_CELLULAR\_RESPONSE\_TO\_CISPLATIN |  | 5 | -0.59 | -1.15 | 0.293 | 0.593 | 1.000 | 4465 | tags=80%, list=37%, signal=127% |
| 3448 | GOMF\_PEPTIDYLTRANSFERASE\_ACTIVITY |  | 3 | -0.69 | -1.15 | 0.276 | 0.593 | 1.000 | 1878 | tags=67%, list=16%, signal=79% |
| 3449 | GOBP\_REGULATION\_OF\_TRANSMEMBRANE\_TRANSPORT |  | 330 | -0.30 | -1.15 | 0.121 | 0.593 | 1.000 | 3934 | tags=36%, list=33%, signal=52% |
| 3450 | GOBP\_COMMON\_MYELOID\_PROGENITOR\_CELL\_PROLIFERATION |  | 3 | -0.68 | -1.15 | 0.290 | 0.593 | 1.000 | 1935 | tags=67%, list=16%, signal=79% |
| 3451 | GOBP\_REGULATION\_OF\_RRNA\_PROCESSING |  | 10 | -0.49 | -1.15 | 0.293 | 0.593 | 1.000 | 4278 | tags=50%, list=36%, signal=78% |
| 3452 | GOBP\_DETECTION\_OF\_MOLECULE\_OF\_BACTERIAL\_ORIGIN |  | 6 | -0.56 | -1.15 | 0.293 | 0.594 | 1.000 | 1230 | tags=33%, list=10%, signal=37% |
| 3453 | GOMF\_PROFILIN\_BINDING |  | 8 | -0.52 | -1.15 | 0.307 | 0.594 | 1.000 | 3946 | tags=50%, list=33%, signal=74% |
| 3454 | GOBP\_POSITIVE\_REGULATION\_OF\_TRANSCRIPTION\_OF\_NOTCH\_RECEPTOR\_TARGET |  | 15 | -0.44 | -1.15 | 0.283 | 0.594 | 1.000 | 4358 | tags=73%, list=36%, signal=115% |
| 3455 | GOBP\_REGULATION\_OF\_MESODERMAL\_CELL\_DIFFERENTIATION |  | 3 | -0.69 | -1.15 | 0.285 | 0.594 | 1.000 | 2886 | tags=67%, list=24%, signal=88% |
| 3456 | GOBP\_NEGATIVE\_REGULATION\_OF\_MUSCLE\_ADAPTATION |  | 9 | -0.50 | -1.15 | 0.302 | 0.594 | 1.000 | 2267 | tags=44%, list=19%, signal=55% |
| 3457 | GOBP\_LUNG\_VASCULATURE\_DEVELOPMENT |  | 3 | -0.69 | -1.15 | 0.284 | 0.594 | 1.000 | 2267 | tags=33%, list=19%, signal=41% |
| 3458 | GOBP\_ER\_ASSOCIATED\_MISFOLDED\_PROTEIN\_CATABOLIC\_PROCESS |  | 9 | -0.50 | -1.15 | 0.294 | 0.595 | 1.000 | 5182 | tags=78%, list=43%, signal=137% |
| 3459 | GOBP\_T\_HELPER\_1\_TYPE\_IMMUNE\_RESPONSE |  | 25 | -0.40 | -1.15 | 0.282 | 0.595 | 1.000 | 2847 | tags=40%, list=24%, signal=52% |
| 3460 | GOMF\_TRANSFERASE\_ACTIVITY\_TRANSFERRING\_ACYL\_GROUPS |  | 166 | -0.30 | -1.15 | 0.194 | 0.595 | 1.000 | 3477 | tags=31%, list=29%, signal=43% |
| 3461 | GOBP\_T\_CELL\_MEDIATED\_CYTOTOXICITY |  | 17 | -0.43 | -1.15 | 0.291 | 0.595 | 1.000 | 1519 | tags=29%, list=13%, signal=34% |
| 3462 | GOBP\_REGULATION\_OF\_GRANULOCYTE\_DIFFERENTIATION |  | 12 | -0.47 | -1.15 | 0.302 | 0.595 | 1.000 | 1605 | tags=33%, list=13%, signal=38% |
| 3463 | GOBP\_POSITIVE\_REGULATION\_OF\_SKELETAL\_MUSCLE\_TISSUE\_DEVELOPMENT |  | 10 | -0.49 | -1.15 | 0.298 | 0.595 | 1.000 | 3565 | tags=50%, list=30%, signal=71% |
| 3464 | GOBP\_DECIDUALIZATION |  | 11 | -0.47 | -1.15 | 0.302 | 0.595 | 1.000 | 2732 | tags=55%, list=23%, signal=71% |
| 3465 | GOBP\_CALCINEURIN\_MEDIATED\_SIGNALING |  | 27 | -0.39 | -1.15 | 0.291 | 0.595 | 1.000 | 3641 | tags=52%, list=30%, signal=74% |
| 3466 | GOBP\_NECROTIC\_CELL\_DEATH |  | 38 | -0.36 | -1.15 | 0.271 | 0.596 | 1.000 | 1869 | tags=24%, list=16%, signal=28% |
| 3467 | GOBP\_GLUCOSE\_IMPORT\_IN\_RESPONSE\_TO\_INSULIN\_STIMULUS |  | 3 | -0.68 | -1.15 | 0.273 | 0.596 | 1.000 | 2162 | tags=67%, list=18%, signal=81% |
| 3468 | GOBP\_B\_CELL\_APOPTOTIC\_PROCESS |  | 20 | -0.41 | -1.15 | 0.298 | 0.596 | 1.000 | 1560 | tags=30%, list=13%, signal=34% |
| 3469 | GOBP\_XENOBIOTIC\_TRANSPORT\_ACROSS\_BLOOD\_BRAIN\_BARRIER |  | 3 | -0.69 | -1.15 | 0.278 | 0.596 | 1.000 | 1410 | tags=67%, list=12%, signal=76% |
| 3470 | GOMF\_LIPOPROTEIN\_LIPASE\_ACTIVITY |  | 3 | -0.69 | -1.15 | 0.283 | 0.596 | 1.000 | 2651 | tags=67%, list=22%, signal=86% |
| 3471 | GOCC\_HOPS\_COMPLEX |  | 11 | -0.48 | -1.15 | 0.297 | 0.596 | 1.000 | 2675 | tags=27%, list=22%, signal=35% |
| 3472 | GOBP\_HISTIDINE\_METABOLIC\_PROCESS |  | 8 | -0.51 | -1.15 | 0.295 | 0.596 | 1.000 | 4371 | tags=75%, list=36%, signal=118% |
| 3473 | GOBP\_RENAL\_SYSTEM\_PROCESS |  | 78 | -0.33 | -1.15 | 0.244 | 0.596 | 1.000 | 2480 | tags=27%, list=21%, signal=34% |
| 3474 | GOBP\_POSITIVE\_REGULATION\_OF\_ANION\_TRANSPORT |  | 335 | -0.29 | -1.15 | 0.127 | 0.596 | 1.000 | 3187 | tags=30%, list=27%, signal=39% |
| 3475 | GOBP\_NEGATIVE\_REGULATION\_OF\_MAST\_CELL\_ACTIVATION\_INVOLVED\_IN\_IMMUNE\_RESPONSE |  | 5 | -0.59 | -1.14 | 0.291 | 0.597 | 1.000 | 726 | tags=40%, list=6%, signal=43% |
| 3476 | GOBP\_CELLULAR\_MODIFIED\_AMINO\_ACID\_BIOSYNTHETIC\_PROCESS |  | 35 | -0.37 | -1.14 | 0.285 | 0.597 | 1.000 | 3650 | tags=43%, list=30%, signal=61% |
| 3477 | GOBP\_REGULATION\_OF\_CARBOHYDRATE\_CATABOLIC\_PROCESS |  | 68 | -0.33 | -1.14 | 0.252 | 0.597 | 1.000 | 4770 | tags=53%, list=40%, signal=87% |
| 3478 | GOBP\_XYLULOSE\_5\_PHOSPHATE\_METABOLIC\_PROCESS |  | 5 | -0.59 | -1.14 | 0.285 | 0.597 | 1.000 | 2332 | tags=60%, list=19%, signal=74% |
| 3479 | GOMF\_SERINE\_TYPE\_EXOPEPTIDASE\_ACTIVITY |  | 7 | -0.54 | -1.14 | 0.295 | 0.597 | 1.000 | 4009 | tags=71%, list=33%, signal=107% |
| 3480 | GOMF\_TRANSFORMING\_GROWTH\_FACTOR\_BETA\_BINDING |  | 18 | -0.42 | -1.14 | 0.296 | 0.598 | 1.000 | 3064 | tags=33%, list=26%, signal=45% |
| 3481 | GOCC\_INTEGRAL\_COMPONENT\_OF\_SYNAPTIC\_VESICLE\_MEMBRANE |  | 18 | -0.42 | -1.14 | 0.288 | 0.598 | 1.000 | 3878 | tags=50%, list=32%, signal=74% |
| 3482 | GOCC\_ALPHA\_DNA\_POLYMERASE\_PRIMASE\_COMPLEX |  | 4 | -0.63 | -1.14 | 0.286 | 0.598 | 1.000 | 3402 | tags=75%, list=28%, signal=105% |
| 3483 | GOBP\_COMPLEMENT\_ACTIVATION\_ALTERNATIVE\_PATHWAY |  | 8 | -0.52 | -1.14 | 0.303 | 0.598 | 1.000 | 4135 | tags=50%, list=34%, signal=76% |
| 3484 | GOMF\_AROMATASE\_ACTIVITY |  | 5 | -0.59 | -1.14 | 0.294 | 0.598 | 1.000 | 2278 | tags=60%, list=19%, signal=74% |
| 3485 | GOBP\_RESPONSE\_TO\_RETINOIC\_ACID |  | 65 | -0.33 | -1.14 | 0.258 | 0.599 | 1.000 | 4846 | tags=54%, list=40%, signal=90% |
| 3486 | GOMF\_LYSOPHOSPHATIDIC\_ACID\_BINDING |  | 8 | -0.52 | -1.14 | 0.306 | 0.598 | 1.000 | 29 | tags=13%, list=0%, signal=13% |
| 3487 | GOBP\_REGULATION\_OF\_FOREBRAIN\_NEURON\_DIFFERENTIATION |  | 4 | -0.63 | -1.14 | 0.301 | 0.599 | 1.000 | 149 | tags=25%, list=1%, signal=25% |
| 3488 | GOBP\_NEGATIVE\_REGULATION\_OF\_AMYLOID\_BETA\_CLEARANCE |  | 6 | -0.56 | -1.14 | 0.296 | 0.599 | 1.000 | 95 | tags=17%, list=1%, signal=17% |
| 3489 | GOBP\_MRNA\_5\_SPLICE\_SITE\_RECOGNITION |  | 5 | -0.60 | -1.14 | 0.284 | 0.599 | 1.000 | 3485 | tags=60%, list=29%, signal=84% |
| 3490 | GOBP\_DORSAL\_AORTA\_MORPHOGENESIS |  | 8 | -0.52 | -1.14 | 0.300 | 0.599 | 1.000 | 3314 | tags=50%, list=28%, signal=69% |
| 3491 | GOBP\_POSITIVE\_REGULATION\_OF\_TYPE\_2\_IMMUNE\_RESPONSE |  | 11 | -0.47 | -1.14 | 0.305 | 0.599 | 1.000 | 1566 | tags=45%, list=13%, signal=52% |
| 3492 | GOBP\_CELL\_ACTIVATION\_INVOLVED\_IN\_IMMUNE\_RESPONSE |  | 460 | -0.29 | -1.14 | 0.098 | 0.599 | 1.000 | 3474 | tags=33%, list=29%, signal=44% |
| 3493 | GOBP\_INNER\_EAR\_RECEPTOR\_CELL\_DEVELOPMENT |  | 25 | -0.39 | -1.14 | 0.284 | 0.598 | 1.000 | 3868 | tags=44%, list=32%, signal=65% |
| 3494 | GOBP\_PEPTIDYL\_ARGININE\_MODIFICATION |  | 12 | -0.47 | -1.14 | 0.304 | 0.598 | 1.000 | 3647 | tags=50%, list=30%, signal=72% |
| 3495 | GOBP\_RESPONSE\_TO\_DEXAMETHASONE |  | 30 | -0.38 | -1.14 | 0.293 | 0.598 | 1.000 | 2803 | tags=30%, list=23%, signal=39% |
| 3496 | GOMF\_TRNA\_GUANINE\_METHYLTRANSFERASE\_ACTIVITY |  | 10 | -0.49 | -1.14 | 0.302 | 0.598 | 1.000 | 3232 | tags=40%, list=27%, signal=55% |
| 3497 | GOMF\_HEME\_TRANSMEMBRANE\_TRANSPORTER\_ACTIVITY |  | 7 | -0.54 | -1.14 | 0.294 | 0.598 | 1.000 | 3318 | tags=43%, list=28%, signal=59% |
| 3498 | GOBP\_NEGATIVE\_REGULATION\_OF\_GROWTH |  | 163 | -0.30 | -1.14 | 0.205 | 0.598 | 1.000 | 2731 | tags=27%, list=23%, signal=34% |
| 3499 | GOCC\_COHESIN\_COMPLEX |  | 8 | -0.52 | -1.14 | 0.306 | 0.598 | 1.000 | 4589 | tags=75%, list=38%, signal=121% |
| 3500 | GOCC\_EXTRACELLULAR\_MEMBRANE\_BOUNDED\_ORGANELLE |  | 3 | -0.68 | -1.14 | 0.286 | 0.598 | 1.000 | 1591 | tags=67%, list=13%, signal=77% |
| 3501 | GOBP\_SMOOTH\_MUSCLE\_CELL\_APOPTOTIC\_PROCESS |  | 17 | -0.43 | -1.14 | 0.298 | 0.598 | 1.000 | 1981 | tags=41%, list=16%, signal=49% |
| 3502 | GOBP\_NEGATIVE\_REGULATION\_OF\_TELOMERE\_CAPPING |  | 6 | -0.56 | -1.14 | 0.304 | 0.598 | 1.000 | 1851 | tags=50%, list=15%, signal=59% |
| 3503 | GOBP\_NEGATIVE\_REGULATION\_OF\_PROTEIN\_MATURATION |  | 17 | -0.43 | -1.14 | 0.302 | 0.598 | 1.000 | 3483 | tags=47%, list=29%, signal=66% |
| 3504 | GOMF\_STEROID\_BINDING |  | 68 | -0.33 | -1.14 | 0.251 | 0.598 | 1.000 | 3157 | tags=34%, list=26%, signal=46% |
| 3505 | GOMF\_PEPTIDASE\_ACTIVITY |  | 355 | -0.29 | -1.14 | 0.122 | 0.598 | 1.000 | 3799 | tags=32%, list=32%, signal=46% |
| 3506 | GOBP\_CELLULAR\_RESPONSE\_TO\_HISTAMINE |  | 3 | -0.68 | -1.14 | 0.287 | 0.598 | 1.000 | 1782 | tags=67%, list=15%, signal=78% |
| 3507 | GOBP\_PROTEIN\_LOCALIZATION\_TO\_NUCLEAR\_ENVELOPE |  | 7 | -0.54 | -1.14 | 0.299 | 0.598 | 1.000 | 3373 | tags=57%, list=28%, signal=79% |
| 3508 | GOBP\_LEUKOTRIENE\_BIOSYNTHETIC\_PROCESS |  | 11 | -0.47 | -1.14 | 0.297 | 0.598 | 1.000 | 2357 | tags=55%, list=20%, signal=68% |
| 3509 | GOBP\_NEGATIVE\_REGULATION\_OF\_PEPTIDYL\_LYSINE\_ACETYLATION |  | 16 | -0.43 | -1.14 | 0.304 | 0.598 | 1.000 | 3395 | tags=44%, list=28%, signal=61% |
| 3510 | GOBP\_POSITIVE\_REGULATION\_OF\_CD4\_POSITIVE\_ALPHA\_BETA\_T\_CELL\_DIFFERENTIATION |  | 19 | -0.41 | -1.14 | 0.292 | 0.598 | 1.000 | 2345 | tags=37%, list=20%, signal=46% |
| 3511 | GOCC\_MRNA\_EDITING\_COMPLEX |  | 7 | -0.54 | -1.14 | 0.306 | 0.598 | 1.000 | 4252 | tags=71%, list=35%, signal=111% |
| 3512 | GOBP\_POSITIVE\_REGULATION\_OF\_CELL\_MATURATION |  | 6 | -0.56 | -1.14 | 0.300 | 0.598 | 1.000 | 3433 | tags=67%, list=29%, signal=93% |
| 3513 | GOCC\_NUCLEAR\_ENVELOPE |  | 345 | -0.29 | -1.14 | 0.128 | 0.598 | 1.000 | 4120 | tags=41%, list=34%, signal=60% |
| 3514 | GOBP\_PYRIDINE\_CONTAINING\_COMPOUND\_BIOSYNTHETIC\_PROCESS |  | 23 | -0.40 | -1.14 | 0.292 | 0.598 | 1.000 | 2865 | tags=30%, list=24%, signal=40% |
| 3515 | GOBP\_PURINE\_NUCLEOSIDE\_MONOPHOSPHATE\_CATABOLIC\_PROCESS |  | 6 | -0.56 | -1.14 | 0.293 | 0.598 | 1.000 | 1107 | tags=50%, list=9%, signal=55% |
| 3516 | GOMF\_PHOSPHATIDYLINOSITOL\_PHOSPHATE\_BINDING |  | 119 | -0.31 | -1.14 | 0.225 | 0.597 | 1.000 | 3861 | tags=36%, list=32%, signal=53% |
| 3517 | GOBP\_REPRODUCTION |  | 794 | -0.29 | -1.14 | 0.046 | 0.597 | 1.000 | 4532 | tags=43%, list=38%, signal=65% |
| 3518 | GOBP\_BASE\_EXCISION\_REPAIR |  | 30 | -0.38 | -1.14 | 0.287 | 0.598 | 1.000 | 3412 | tags=33%, list=28%, signal=46% |
| 3519 | GOBP\_CANONICAL\_WNT\_SIGNALING\_PATHWAY |  | 237 | -0.30 | -1.14 | 0.160 | 0.597 | 1.000 | 3565 | tags=32%, list=30%, signal=45% |
| 3520 | GOBP\_PTERIDINE\_CONTAINING\_COMPOUND\_BIOSYNTHETIC\_PROCESS |  | 13 | -0.45 | -1.14 | 0.309 | 0.597 | 1.000 | 4242 | tags=54%, list=35%, signal=83% |
| 3521 | GOBP\_REGULATION\_OF\_TRANSLATIONAL\_INITIATION |  | 63 | -0.34 | -1.14 | 0.261 | 0.597 | 1.000 | 4629 | tags=41%, list=39%, signal=67% |
| 3522 | GOBP\_POSITIVE\_REGULATION\_OF\_VASCULAR\_ASSOCIATED\_SMOOTH\_MUSCLE\_CELL\_PROLIFERATION |  | 27 | -0.39 | -1.14 | 0.289 | 0.597 | 1.000 | 4069 | tags=44%, list=34%, signal=67% |
| 3523 | GOMF\_INOSITOL\_PHOSPHATE\_PHOSPHATASE\_ACTIVITY |  | 13 | -0.46 | -1.14 | 0.305 | 0.597 | 1.000 | 4540 | tags=62%, list=38%, signal=99% |
| 3524 | GOBP\_VASCULAR\_ENDOTHELIAL\_GROWTH\_FACTOR\_PRODUCTION |  | 26 | -0.39 | -1.14 | 0.286 | 0.597 | 1.000 | 3004 | tags=35%, list=25%, signal=46% |
| 3525 | GOBP\_RETROGRADE\_TRANS\_SYNAPTIC\_SIGNALING\_BY\_LIPID |  | 5 | -0.59 | -1.14 | 0.292 | 0.597 | 1.000 | 4907 | tags=100%, list=41%, signal=169% |
| 3526 | GOBP\_GANGLION\_DEVELOPMENT |  | 12 | -0.46 | -1.14 | 0.303 | 0.597 | 1.000 | 4739 | tags=75%, list=39%, signal=124% |
| 3527 | GOBP\_PRE\_MIRNA\_PROCESSING |  | 8 | -0.52 | -1.14 | 0.311 | 0.598 | 1.000 | 2086 | tags=50%, list=17%, signal=60% |
| 3528 | GOBP\_POSITIVE\_REGULATION\_OF\_TOR\_SIGNALING |  | 33 | -0.37 | -1.14 | 0.290 | 0.598 | 1.000 | 3512 | tags=36%, list=29%, signal=51% |
| 3529 | GOBP\_POSITIVE\_REGULATION\_OF\_CANONICAL\_WNT\_SIGNALING\_PATHWAY |  | 109 | -0.31 | -1.14 | 0.231 | 0.598 | 1.000 | 3483 | tags=32%, list=29%, signal=45% |
| 3530 | GOBP\_GLYCOSYLATION |  | 184 | -0.30 | -1.14 | 0.193 | 0.598 | 1.000 | 2617 | tags=27%, list=22%, signal=34% |
| 3531 | GOBP\_NUCLEOBASE\_CATABOLIC\_PROCESS |  | 5 | -0.60 | -1.14 | 0.306 | 0.598 | 1.000 | 1107 | tags=60%, list=9%, signal=66% |
| 3532 | GOBP\_REGULATION\_OF\_MORPHOGENESIS\_OF\_A\_BRANCHING\_STRUCTURE |  | 32 | -0.37 | -1.14 | 0.290 | 0.598 | 1.000 | 3565 | tags=34%, list=30%, signal=49% |
| 3533 | GOBP\_STRIATED\_MUSCLE\_CELL\_DIFFERENTIATION |  | 154 | -0.30 | -1.14 | 0.207 | 0.598 | 1.000 | 3331 | tags=31%, list=28%, signal=42% |
| 3534 | GOBP\_REGULATION\_OF\_T\_CELL\_MEDIATED\_IMMUNITY |  | 33 | -0.37 | -1.14 | 0.282 | 0.598 | 1.000 | 1781 | tags=33%, list=15%, signal=39% |
| 3535 | GOBP\_CELL\_COMMUNICATION\_BY\_ELECTRICAL\_COUPLING\_INVOLVED\_IN\_CARDIAC\_CONDUCTION |  | 15 | -0.44 | -1.14 | 0.304 | 0.598 | 1.000 | 2313 | tags=27%, list=19%, signal=33% |
| 3536 | GOBP\_ESTABLISHMENT\_OF\_SISTER\_CHROMATID\_COHESION |  | 6 | -0.57 | -1.14 | 0.296 | 0.599 | 1.000 | 4465 | tags=67%, list=37%, signal=106% |
| 3537 | GOBP\_POSITIVE\_REGULATION\_OF\_CHROMOSOME\_SEPARATION |  | 16 | -0.43 | -1.14 | 0.304 | 0.599 | 1.000 | 6486 | tags=69%, list=54%, signal=149% |
| 3538 | GOBP\_POSITIVE\_REGULATION\_OF\_HEART\_RATE |  | 18 | -0.42 | -1.14 | 0.301 | 0.598 | 1.000 | 1222 | tags=28%, list=10%, signal=31% |
| 3539 | GOBP\_NECROPTOTIC\_SIGNALING\_PATHWAY |  | 7 | -0.54 | -1.14 | 0.301 | 0.598 | 1.000 | 1084 | tags=43%, list=9%, signal=47% |
| 3540 | GOCC\_PRESYNAPTIC\_CYTOSOL |  | 10 | -0.49 | -1.14 | 0.306 | 0.598 | 1.000 | 3483 | tags=60%, list=29%, signal=84% |
| 3541 | GOBP\_G\_PROTEIN\_COUPLED\_RECEPTOR\_SIGNALING\_PATHWAY\_COUPLED\_TO\_CYCLIC\_NUCLEOTIDE\_SECOND\_MESSENGER |  | 26 | -0.39 | -1.14 | 0.296 | 0.598 | 1.000 | 3135 | tags=42%, list=26%, signal=57% |
| 3542 | GOBP\_REGULATION\_OF\_CARDIAC\_MUSCLE\_CONTRACTION |  | 47 | -0.35 | -1.14 | 0.269 | 0.598 | 1.000 | 3968 | tags=43%, list=33%, signal=63% |
| 3543 | GOMF\_TRANSCRIPTION\_FACTOR\_BINDING |  | 477 | -0.29 | -1.14 | 0.095 | 0.599 | 1.000 | 2732 | tags=25%, list=23%, signal=31% |
| 3544 | GOMF\_MYOSIN\_HEAVY\_CHAIN\_BINDING |  | 5 | -0.59 | -1.14 | 0.298 | 0.599 | 1.000 | 4047 | tags=60%, list=34%, signal=90% |
| 3545 | GOMF\_ACETYLGALACTOSAMINYLTRANSFERASE\_ACTIVITY |  | 32 | -0.37 | -1.14 | 0.292 | 0.599 | 1.000 | 2617 | tags=31%, list=22%, signal=40% |
| 3546 | GOBP\_MITOCHONDRION\_LOCALIZATION |  | 40 | -0.36 | -1.14 | 0.282 | 0.599 | 1.000 | 5658 | tags=63%, list=47%, signal=118% |
| 3547 | GOBP\_TRANSFORMING\_GROWTH\_FACTOR\_BETA\_PRODUCTION |  | 31 | -0.38 | -1.14 | 0.295 | 0.599 | 1.000 | 3186 | tags=42%, list=27%, signal=57% |
| 3548 | GOBP\_TRYPTOPHAN\_METABOLIC\_PROCESS |  | 6 | -0.56 | -1.14 | 0.304 | 0.599 | 1.000 | 2220 | tags=33%, list=18%, signal=41% |
| 3549 | GOBP\_MRNA\_SPLICE\_SITE\_SELECTION |  | 24 | -0.39 | -1.14 | 0.291 | 0.599 | 1.000 | 5146 | tags=50%, list=43%, signal=87% |
| 3550 | GOMF\_ESTROGEN\_RECEPTOR\_BINDING |  | 32 | -0.37 | -1.14 | 0.290 | 0.599 | 1.000 | 2811 | tags=34%, list=23%, signal=45% |
| 3551 | GOBP\_ENDOTHELIAL\_CELL\_ACTIVATION |  | 7 | -0.54 | -1.14 | 0.308 | 0.599 | 1.000 | 4506 | tags=71%, list=38%, signal=114% |
| 3552 | GOMF\_G\_PROTEIN\_BETA\_SUBUNIT\_BINDING |  | 13 | -0.46 | -1.14 | 0.304 | 0.599 | 1.000 | 934 | tags=23%, list=8%, signal=25% |
| 3553 | GOBP\_CELLULAR\_PROCESS\_INVOLVED\_IN\_REPRODUCTION\_IN\_MULTICELLULAR\_ORGANISM |  | 174 | -0.30 | -1.14 | 0.200 | 0.599 | 1.000 | 4519 | tags=45%, list=38%, signal=72% |
| 3554 | GOBP\_POSITIVE\_REGULATION\_OF\_SYNAPTIC\_VESICLE\_RECYCLING |  | 5 | -0.59 | -1.14 | 0.298 | 0.599 | 1.000 | 4673 | tags=60%, list=39%, signal=98% |
| 3555 | GOBP\_ENDOTHELIAL\_CELL\_MATRIX\_ADHESION |  | 3 | -0.68 | -1.14 | 0.285 | 0.599 | 1.000 | 2257 | tags=67%, list=19%, signal=82% |
| 3556 | GOBP\_PHAGOCYTOSIS\_RECOGNITION |  | 17 | -0.43 | -1.14 | 0.301 | 0.600 | 1.000 | 1613 | tags=29%, list=13%, signal=34% |
| 3557 | GOBP\_BONE\_MARROW\_DEVELOPMENT |  | 7 | -0.54 | -1.14 | 0.317 | 0.600 | 1.000 | 5576 | tags=100%, list=46%, signal=187% |
| 3558 | GOBP\_HEME\_TRANSPORT |  | 7 | -0.54 | -1.14 | 0.300 | 0.600 | 1.000 | 3318 | tags=43%, list=28%, signal=59% |
| 3559 | GOBP\_REGULATION\_OF\_NEURON\_MIGRATION |  | 24 | -0.40 | -1.14 | 0.297 | 0.600 | 1.000 | 4003 | tags=50%, list=33%, signal=75% |
| 3560 | GOCC\_POSTSYNAPTIC\_CYTOSOL |  | 14 | -0.45 | -1.14 | 0.303 | 0.600 | 1.000 | 3392 | tags=43%, list=28%, signal=60% |
| 3561 | GOBP\_PROTEIN\_TARGETING\_TO\_LYSOSOME |  | 20 | -0.41 | -1.14 | 0.297 | 0.599 | 1.000 | 4280 | tags=55%, list=36%, signal=85% |
| 3562 | GOBP\_REGULATION\_OF\_ORGANELLE\_ORGANIZATION |  | 850 | -0.29 | -1.14 | 0.045 | 0.599 | 1.000 | 4716 | tags=42%, list=39%, signal=65% |
| 3563 | GOCC\_BOX\_C\_D\_RNP\_COMPLEX |  | 4 | -0.63 | -1.14 | 0.294 | 0.600 | 1.000 | 1122 | tags=25%, list=9%, signal=28% |
| 3564 | GOBP\_ACTIVATION\_OF\_INNATE\_IMMUNE\_RESPONSE |  | 86 | -0.32 | -1.14 | 0.244 | 0.600 | 1.000 | 4854 | tags=45%, list=40%, signal=76% |
| 3565 | GOCC\_INTERCELLULAR\_BRIDGE |  | 54 | -0.34 | -1.14 | 0.279 | 0.600 | 1.000 | 3241 | tags=35%, list=27%, signal=48% |
| 3566 | GOBP\_ACTIVATION\_OF\_PHOSPHOLIPASE\_D\_ACTIVITY |  | 5 | -0.59 | -1.14 | 0.304 | 0.600 | 1.000 | 1678 | tags=40%, list=14%, signal=46% |
| 3567 | GOMF\_SODIUM\_CHANNEL\_REGULATOR\_ACTIVITY |  | 21 | -0.40 | -1.14 | 0.290 | 0.600 | 1.000 | 2337 | tags=33%, list=19%, signal=41% |
| 3568 | GOCC\_LYSOSOMAL\_LUMEN |  | 73 | -0.33 | -1.14 | 0.260 | 0.600 | 1.000 | 5322 | tags=55%, list=44%, signal=98% |
| 3569 | GOCC\_GOLGI\_ASSOCIATED\_VESICLE |  | 66 | -0.33 | -1.14 | 0.263 | 0.601 | 1.000 | 5265 | tags=58%, list=44%, signal=102% |
| 3570 | GOBP\_NEGATIVE\_REGULATION\_OF\_SIGNALING |  | 934 | -0.29 | -1.14 | 0.043 | 0.601 | 1.000 | 3459 | tags=32%, list=29%, signal=41% |
| 3571 | GOBP\_REGULATION\_OF\_LIPID\_LOCALIZATION |  | 111 | -0.31 | -1.14 | 0.238 | 0.602 | 1.000 | 4164 | tags=43%, list=35%, signal=66% |
| 3572 | GOBP\_NEGATIVE\_REGULATION\_OF\_PROTEIN\_LOCALIZATION\_TO\_MICROTUBULE |  | 4 | -0.63 | -1.14 | 0.292 | 0.601 | 1.000 | 1337 | tags=50%, list=11%, signal=56% |
| 3573 | GOBP\_REGULATION\_OF\_DNA\_REPLICATION |  | 88 | -0.32 | -1.14 | 0.251 | 0.601 | 1.000 | 5292 | tags=56%, list=44%, signal=99% |
| 3574 | GOBP\_BRUSH\_BORDER\_ASSEMBLY |  | 3 | -0.68 | -1.14 | 0.304 | 0.602 | 1.000 | 3868 | tags=100%, list=32%, signal=147% |
| 3575 | GOBP\_CELLULAR\_RESPONSE\_TO\_NUTRIENT |  | 31 | -0.38 | -1.14 | 0.290 | 0.602 | 1.000 | 4358 | tags=52%, list=36%, signal=81% |
| 3576 | GOBP\_PROTEIN\_K11\_LINKED\_DEUBIQUITINATION |  | 9 | -0.50 | -1.14 | 0.308 | 0.602 | 1.000 | 2263 | tags=44%, list=19%, signal=55% |
| 3577 | GOBP\_FAT\_CELL\_DIFFERENTIATION |  | 153 | -0.30 | -1.14 | 0.215 | 0.602 | 1.000 | 3968 | tags=40%, list=33%, signal=59% |
| 3578 | GOBP\_REGULATION\_OF\_PANCREATIC\_JUICE\_SECRETION |  | 3 | -0.68 | -1.14 | 0.292 | 0.602 | 1.000 | 2895 | tags=67%, list=24%, signal=88% |
| 3579 | GOBP\_CELLULAR\_RESPONSE\_TO\_ABIOTIC\_STIMULUS |  | 223 | -0.30 | -1.14 | 0.181 | 0.602 | 1.000 | 3927 | tags=39%, list=33%, signal=56% |
| 3580 | GOBP\_NEGATIVE\_REGULATION\_OF\_PRODUCTION\_OF\_MOLECULAR\_MEDIATOR\_OF\_IMMUNE\_RESPONSE |  | 20 | -0.41 | -1.14 | 0.307 | 0.602 | 1.000 | 2889 | tags=35%, list=24%, signal=46% |
| 3581 | GOBP\_IRON\_COORDINATION\_ENTITY\_TRANSPORT |  | 7 | -0.54 | -1.14 | 0.306 | 0.602 | 1.000 | 3318 | tags=43%, list=28%, signal=59% |
| 3582 | GOBP\_REGULATION\_OF\_CELLULAR\_LOCALIZATION |  | 581 | -0.29 | -1.14 | 0.078 | 0.602 | 1.000 | 3909 | tags=33%, list=33%, signal=46% |
| 3583 | GOMF\_BRANCHED\_CHAIN\_AMINO\_ACID\_TRANSMEMBRANE\_TRANSPORTER\_ACTIVITY |  | 5 | -0.59 | -1.14 | 0.296 | 0.602 | 1.000 | 3155 | tags=60%, list=26%, signal=81% |
| 3584 | GOBP\_HISTONE\_H2A\_UBIQUITINATION |  | 23 | -0.40 | -1.14 | 0.299 | 0.603 | 1.000 | 4500 | tags=61%, list=37%, signal=97% |
| 3585 | GOBP\_MUCOSA\_ASSOCIATED\_LYMPHOID\_TISSUE\_DEVELOPMENT |  | 4 | -0.63 | -1.14 | 0.300 | 0.603 | 1.000 | 2356 | tags=75%, list=20%, signal=93% |
| 3586 | GOBP\_ESTROUS\_CYCLE |  | 11 | -0.47 | -1.14 | 0.309 | 0.604 | 1.000 | 2374 | tags=45%, list=20%, signal=57% |
| 3587 | GOBP\_RESPONSE\_TO\_ORGANOPHOSPHORUS |  | 82 | -0.32 | -1.14 | 0.251 | 0.604 | 1.000 | 4848 | tags=46%, list=40%, signal=77% |
| 3588 | GOMF\_CARBOHYDRATE\_KINASE\_ACTIVITY |  | 18 | -0.42 | -1.14 | 0.298 | 0.604 | 1.000 | 3908 | tags=61%, list=33%, signal=90% |
| 3589 | GOBP\_BODY\_FLUID\_SECRETION |  | 49 | -0.34 | -1.14 | 0.271 | 0.604 | 1.000 | 4561 | tags=49%, list=38%, signal=79% |
| 3590 | GOBP\_TERPENOID\_METABOLIC\_PROCESS |  | 69 | -0.33 | -1.14 | 0.265 | 0.605 | 1.000 | 3525 | tags=33%, list=29%, signal=47% |
| 3591 | GOBP\_REGULATION\_OF\_RELEASE\_OF\_SEQUESTERED\_CALCIUM\_ION\_INTO\_CYTOSOL |  | 49 | -0.35 | -1.14 | 0.276 | 0.605 | 1.000 | 4846 | tags=49%, list=40%, signal=82% |
| 3592 | GOBP\_LUNG\_ASSOCIATED\_MESENCHYME\_DEVELOPMENT |  | 4 | -0.63 | -1.14 | 0.291 | 0.605 | 1.000 | 106 | tags=25%, list=1%, signal=25% |
| 3593 | GOBP\_PROTEIN\_IMPORT |  | 145 | -0.31 | -1.14 | 0.214 | 0.605 | 1.000 | 4321 | tags=39%, list=36%, signal=60% |
| 3594 | GOCC\_POSTSYNAPTIC\_SPECIALIZATION\_INTRACELLULAR\_COMPONENT |  | 11 | -0.47 | -1.14 | 0.317 | 0.605 | 1.000 | 4033 | tags=45%, list=34%, signal=68% |
| 3595 | GOBP\_SIGNAL\_PEPTIDE\_PROCESSING |  | 10 | -0.48 | -1.14 | 0.302 | 0.605 | 1.000 | 111 | tags=10%, list=1%, signal=10% |
| 3596 | GOBP\_NEGATIVE\_REGULATION\_OF\_BONE\_RESORPTION |  | 10 | -0.48 | -1.14 | 0.316 | 0.605 | 1.000 | 1457 | tags=30%, list=12%, signal=34% |
| 3597 | GOCC\_PLATELET\_DENSE\_TUBULAR\_NETWORK\_MEMBRANE |  | 6 | -0.56 | -1.13 | 0.322 | 0.606 | 1.000 | 245 | tags=17%, list=2%, signal=17% |
| 3598 | GOBP\_REGULATION\_OF\_CELL\_CYCLE |  | 867 | -0.29 | -1.13 | 0.052 | 0.606 | 1.000 | 4529 | tags=40%, list=38%, signal=59% |
| 3599 | GOBP\_REGULATION\_OF\_GENE\_SILENCING |  | 82 | -0.32 | -1.13 | 0.251 | 0.606 | 1.000 | 4080 | tags=41%, list=34%, signal=62% |
| 3600 | GOBP\_POSITIVE\_REGULATION\_OF\_UBIQUITIN\_DEPENDENT\_PROTEIN\_CATABOLIC\_PROCESS |  | 75 | -0.33 | -1.13 | 0.267 | 0.606 | 1.000 | 1587 | tags=19%, list=13%, signal=21% |
| 3601 | GOBP\_NEGATIVE\_REGULATION\_OF\_ORGAN\_GROWTH |  | 23 | -0.40 | -1.13 | 0.300 | 0.607 | 1.000 | 4180 | tags=61%, list=35%, signal=93% |
| 3602 | GOBP\_MUSCLE\_CELL\_DIFFERENTIATION |  | 217 | -0.30 | -1.13 | 0.189 | 0.607 | 1.000 | 3658 | tags=34%, list=30%, signal=47% |
| 3603 | GOBP\_SPONTANEOUS\_SYNAPTIC\_TRANSMISSION |  | 7 | -0.54 | -1.13 | 0.308 | 0.608 | 1.000 | 2087 | tags=43%, list=17%, signal=52% |
| 3604 | GOBP\_POSITIVE\_REGULATION\_OF\_ERAD\_PATHWAY |  | 9 | -0.50 | -1.13 | 0.303 | 0.608 | 1.000 | 1587 | tags=33%, list=13%, signal=38% |
| 3605 | GOCC\_MANCHETTE |  | 5 | -0.59 | -1.13 | 0.305 | 0.608 | 1.000 | 1242 | tags=20%, list=10%, signal=22% |
| 3606 | GOBP\_INTESTINAL\_ABSORPTION |  | 27 | -0.38 | -1.13 | 0.298 | 0.608 | 1.000 | 1802 | tags=26%, list=15%, signal=30% |
| 3607 | GOCC\_VARICOSITY |  | 5 | -0.58 | -1.13 | 0.310 | 0.608 | 1.000 | 2543 | tags=40%, list=21%, signal=51% |
| 3608 | GOBP\_POSITIVE\_REGULATION\_OF\_INTERLEUKIN\_1\_ALPHA\_PRODUCTION |  | 4 | -0.63 | -1.13 | 0.311 | 0.609 | 1.000 | 755 | tags=25%, list=6%, signal=27% |
| 3609 | GOCC\_INTEGRAL\_COMPONENT\_OF\_PRESYNAPTIC\_ACTIVE\_ZONE\_MEMBRANE |  | 10 | -0.48 | -1.13 | 0.320 | 0.609 | 1.000 | 4148 | tags=50%, list=35%, signal=76% |
| 3610 | GOBP\_REPRODUCTIVE\_SYSTEM\_DEVELOPMENT |  | 274 | -0.29 | -1.13 | 0.176 | 0.609 | 1.000 | 4521 | tags=46%, list=38%, signal=71% |
| 3611 | GOMF\_TRANSCRIPTION\_COREGULATOR\_ACTIVITY |  | 349 | -0.29 | -1.13 | 0.147 | 0.609 | 1.000 | 4455 | tags=41%, list=37%, signal=64% |
| 3612 | GOCC\_TELOMERE\_CAP\_COMPLEX |  | 11 | -0.47 | -1.13 | 0.316 | 0.610 | 1.000 | 4932 | tags=73%, list=41%, signal=123% |
| 3613 | GOBP\_CILIARY\_BASAL\_BODY\_PLASMA\_MEMBRANE\_DOCKING |  | 77 | -0.32 | -1.13 | 0.266 | 0.609 | 1.000 | 3448 | tags=32%, list=29%, signal=45% |
| 3614 | GOBP\_OVULATION\_CYCLE |  | 44 | -0.35 | -1.13 | 0.292 | 0.610 | 1.000 | 2656 | tags=34%, list=22%, signal=44% |
| 3615 | GOBP\_ERBB\_SIGNALING\_PATHWAY |  | 114 | -0.31 | -1.13 | 0.237 | 0.610 | 1.000 | 2354 | tags=25%, list=20%, signal=31% |
| 3616 | GOBP\_REGULATION\_OF\_CELLULAR\_RESPONSE\_TO\_MACROPHAGE\_COLONY\_STIMULATING\_FACTOR\_STIMULUS |  | 4 | -0.62 | -1.13 | 0.307 | 0.609 | 1.000 | 3695 | tags=75%, list=31%, signal=108% |
| 3617 | GOCC\_INTRINSIC\_COMPONENT\_OF\_PRESYNAPTIC\_ACTIVE\_ZONE\_MEMBRANE |  | 12 | -0.46 | -1.13 | 0.316 | 0.609 | 1.000 | 4148 | tags=50%, list=35%, signal=76% |
| 3618 | GOBP\_POSITIVE\_REGULATION\_OF\_PROTEASOMAL\_UBIQUITIN\_DEPENDENT\_PROTEIN\_CATABOLIC\_PROCESS |  | 65 | -0.33 | -1.13 | 0.276 | 0.609 | 1.000 | 1587 | tags=18%, list=13%, signal=21% |
| 3619 | GOMF\_DEATH\_RECEPTOR\_ACTIVITY |  | 11 | -0.47 | -1.13 | 0.312 | 0.610 | 1.000 | 4300 | tags=64%, list=36%, signal=99% |
| 3620 | GOBP\_CONNECTIVE\_TISSUE\_REPLACEMENT |  | 7 | -0.53 | -1.13 | 0.321 | 0.610 | 1.000 | 2416 | tags=29%, list=20%, signal=36% |
| 3621 | GOBP\_GLYCOLIPID\_BIOSYNTHETIC\_PROCESS |  | 49 | -0.34 | -1.13 | 0.282 | 0.610 | 1.000 | 4067 | tags=41%, list=34%, signal=61% |
| 3622 | GOBP\_EQUILIBRIOCEPTION |  | 3 | -0.68 | -1.13 | 0.294 | 0.611 | 1.000 | 3868 | tags=100%, list=32%, signal=147% |
| 3623 | GOBP\_TROPHOBLAST\_GIANT\_CELL\_DIFFERENTIATION |  | 8 | -0.51 | -1.13 | 0.325 | 0.611 | 1.000 | 2874 | tags=50%, list=24%, signal=66% |
| 3624 | GOMF\_PROTEASE\_BINDING |  | 81 | -0.32 | -1.13 | 0.260 | 0.611 | 1.000 | 3487 | tags=35%, list=29%, signal=48% |
| 3625 | GOBP\_REGULATION\_OF\_TRANSPORTER\_ACTIVITY |  | 162 | -0.30 | -1.13 | 0.216 | 0.612 | 1.000 | 3089 | tags=30%, list=26%, signal=40% |
| 3626 | GOBP\_REGULATION\_OF\_AUTOPHAGIC\_CELL\_DEATH |  | 5 | -0.59 | -1.13 | 0.324 | 0.612 | 1.000 | 4317 | tags=80%, list=36%, signal=125% |
| 3627 | GOBP\_POSITIVE\_REGULATION\_OF\_CYTOKINE\_PRODUCTION\_INVOLVED\_IN\_IMMUNE\_RESPONSE |  | 33 | -0.37 | -1.13 | 0.294 | 0.611 | 1.000 | 4686 | tags=64%, list=39%, signal=104% |
| 3628 | GOMF\_NEUROPEPTIDE\_BINDING |  | 8 | -0.52 | -1.13 | 0.314 | 0.611 | 1.000 | 2741 | tags=50%, list=23%, signal=65% |
| 3629 | GOBP\_NEGATIVE\_REGULATION\_OF\_DENDRITE\_DEVELOPMENT |  | 3 | -0.68 | -1.13 | 0.306 | 0.611 | 1.000 | 2995 | tags=67%, list=25%, signal=89% |
| 3630 | GOBP\_ATRIOVENTRICULAR\_VALVE\_DEVELOPMENT |  | 19 | -0.41 | -1.13 | 0.304 | 0.612 | 1.000 | 3314 | tags=42%, list=28%, signal=58% |
| 3631 | GOMF\_CALCIUM\_ION\_TRANSMEMBRANE\_TRANSPORTER\_ACTIVITY |  | 78 | -0.32 | -1.13 | 0.266 | 0.611 | 1.000 | 2216 | tags=24%, list=18%, signal=30% |
| 3632 | GOBP\_REGULATION\_OF\_STEROID\_METABOLIC\_PROCESS |  | 86 | -0.32 | -1.13 | 0.260 | 0.612 | 1.000 | 3269 | tags=35%, list=27%, signal=48% |
| 3633 | GOBP\_LIPID\_METABOLIC\_PROCESS |  | 968 | -0.28 | -1.13 | 0.047 | 0.613 | 1.000 | 4106 | tags=38%, list=34%, signal=53% |
| 3634 | GOBP\_INTEGRIN\_MEDIATED\_SIGNALING\_PATHWAY |  | 80 | -0.32 | -1.13 | 0.262 | 0.612 | 1.000 | 3977 | tags=39%, list=33%, signal=58% |
| 3635 | GOBP\_CELL\_CYCLE\_ARREST |  | 176 | -0.30 | -1.13 | 0.212 | 0.613 | 1.000 | 4822 | tags=44%, list=40%, signal=72% |
| 3636 | GOBP\_CARDIAC\_MUSCLE\_CELL\_ACTION\_POTENTIAL |  | 50 | -0.34 | -1.13 | 0.280 | 0.612 | 1.000 | 4453 | tags=40%, list=37%, signal=63% |
| 3637 | GOBP\_BONE\_GROWTH |  | 17 | -0.42 | -1.13 | 0.316 | 0.612 | 1.000 | 6131 | tags=71%, list=51%, signal=144% |
| 3638 | GOMF\_PHOSPHODIESTERASE\_I\_ACTIVITY |  | 3 | -0.68 | -1.13 | 0.310 | 0.612 | 1.000 | 2920 | tags=67%, list=24%, signal=88% |
| 3639 | GOBP\_MEMBRANE\_PROTEIN\_ECTODOMAIN\_PROTEOLYSIS |  | 31 | -0.37 | -1.13 | 0.303 | 0.612 | 1.000 | 1913 | tags=23%, list=16%, signal=27% |
| 3640 | GOMF\_TRANSCRIPTION\_COREPRESSOR\_ACTIVITY |  | 131 | -0.31 | -1.13 | 0.238 | 0.613 | 1.000 | 4666 | tags=45%, list=39%, signal=73% |
| 3641 | GOBP\_CELLULAR\_RESPONSE\_TO\_DEXAMETHASONE\_STIMULUS |  | 24 | -0.39 | -1.13 | 0.298 | 0.613 | 1.000 | 2803 | tags=33%, list=23%, signal=43% |
| 3642 | GOBP\_RNA\_DESTABILIZATION |  | 31 | -0.37 | -1.13 | 0.297 | 0.613 | 1.000 | 4925 | tags=61%, list=41%, signal=104% |
| 3643 | GOBP\_NEGATIVE\_REGULATION\_OF\_NUCLEAR\_DIVISION |  | 45 | -0.35 | -1.13 | 0.289 | 0.613 | 1.000 | 6284 | tags=62%, list=52%, signal=130% |
| 3644 | GOBP\_REGULATION\_OF\_STEROID\_BIOSYNTHETIC\_PROCESS |  | 67 | -0.33 | -1.13 | 0.277 | 0.613 | 1.000 | 3403 | tags=40%, list=28%, signal=56% |
| 3645 | GOBP\_NEGATIVE\_REGULATION\_OF\_RESPONSE\_TO\_CYTOKINE\_STIMULUS |  | 38 | -0.36 | -1.13 | 0.283 | 0.613 | 1.000 | 4833 | tags=50%, list=40%, signal=83% |
| 3646 | GOBP\_CYTIDINE\_TO\_URIDINE\_EDITING |  | 3 | -0.67 | -1.13 | 0.305 | 0.613 | 1.000 | 492 | tags=33%, list=4%, signal=35% |
| 3647 | GOBP\_POSITIVE\_REGULATION\_OF\_LIPOPOLYSACCHARIDE\_MEDIATED\_SIGNALING\_PATHWAY |  | 7 | -0.53 | -1.13 | 0.319 | 0.614 | 1.000 | 3308 | tags=43%, list=28%, signal=59% |
| 3648 | GOBP\_POSITIVE\_REGULATION\_OF\_ION\_TRANSPORT |  | 449 | -0.29 | -1.13 | 0.127 | 0.614 | 1.000 | 3072 | tags=29%, list=26%, signal=37% |
| 3649 | GOBP\_CYTOPLASMIC\_PATTERN\_RECOGNITION\_RECEPTOR\_SIGNALING\_PATHWAY |  | 48 | -0.34 | -1.13 | 0.282 | 0.614 | 1.000 | 2256 | tags=31%, list=19%, signal=38% |
| 3650 | GOCC\_CELL\_POLE |  | 5 | -0.58 | -1.13 | 0.311 | 0.615 | 1.000 | 3222 | tags=60%, list=27%, signal=82% |
| 3651 | GOBP\_REGULATION\_OF\_AMYLOID\_PRECURSOR\_PROTEIN\_CATABOLIC\_PROCESS |  | 32 | -0.37 | -1.13 | 0.312 | 0.615 | 1.000 | 2460 | tags=28%, list=20%, signal=35% |
| 3652 | GOBP\_PYRIMIDINE\_NUCLEOSIDE\_BIOSYNTHETIC\_PROCESS |  | 14 | -0.44 | -1.13 | 0.319 | 0.615 | 1.000 | 5240 | tags=50%, list=44%, signal=89% |
| 3653 | GOBP\_NEGATIVE\_REGULATION\_OF\_MYELOID\_LEUKOCYTE\_MEDIATED\_IMMUNITY |  | 4 | -0.62 | -1.13 | 0.306 | 0.615 | 1.000 | 1608 | tags=50%, list=13%, signal=58% |
| 3654 | GOBP\_VACUOLE\_ORGANIZATION |  | 136 | -0.30 | -1.13 | 0.241 | 0.615 | 1.000 | 3366 | tags=32%, list=28%, signal=43% |
| 3655 | GOBP\_EYE\_PHOTORECEPTOR\_CELL\_DEVELOPMENT |  | 13 | -0.45 | -1.13 | 0.326 | 0.615 | 1.000 | 3868 | tags=54%, list=32%, signal=79% |
| 3656 | GOMF\_COLLAGEN\_BINDING\_INVOLVED\_IN\_CELL\_MATRIX\_ADHESION |  | 4 | -0.63 | -1.13 | 0.294 | 0.616 | 1.000 | 2181 | tags=50%, list=18%, signal=61% |
| 3657 | GOBP\_REGULATION\_OF\_CELL\_CYCLE\_ARREST |  | 76 | -0.32 | -1.13 | 0.272 | 0.616 | 1.000 | 2677 | tags=29%, list=22%, signal=37% |
| 3658 | GOMF\_TRANSMEMBRANE\_RECEPTOR\_PROTEIN\_SERINE\_THREONINE\_KINASE\_ACTIVITY |  | 13 | -0.45 | -1.13 | 0.323 | 0.616 | 1.000 | 4624 | tags=54%, list=38%, signal=87% |
| 3659 | GOBP\_UV\_PROTECTION |  | 7 | -0.53 | -1.13 | 0.326 | 0.616 | 1.000 | 2282 | tags=57%, list=19%, signal=71% |
| 3660 | GOMF\_LAMININ\_BINDING |  | 20 | -0.41 | -1.13 | 0.320 | 0.616 | 1.000 | 3565 | tags=40%, list=30%, signal=57% |
| 3661 | GOBP\_RESPONSE\_TO\_STEROL\_DEPLETION |  | 14 | -0.44 | -1.13 | 0.324 | 0.616 | 1.000 | 1393 | tags=29%, list=12%, signal=32% |
| 3662 | GOMF\_PHOSPHOLIPASE\_BINDING |  | 11 | -0.47 | -1.13 | 0.318 | 0.616 | 1.000 | 3905 | tags=55%, list=33%, signal=81% |
| 3663 | GOBP\_AMINO\_ACID\_TRANSMEMBRANE\_TRANSPORT |  | 59 | -0.33 | -1.13 | 0.287 | 0.616 | 1.000 | 3220 | tags=36%, list=27%, signal=48% |
| 3664 | GOBP\_CELLULAR\_TRANSITION\_METAL\_ION\_HOMEOSTASIS |  | 72 | -0.32 | -1.13 | 0.274 | 0.616 | 1.000 | 3318 | tags=38%, list=28%, signal=52% |
| 3665 | GOBP\_REGULATION\_OF\_MAST\_CELL\_CHEMOTAXIS |  | 7 | -0.53 | -1.13 | 0.320 | 0.616 | 1.000 | 4108 | tags=71%, list=34%, signal=108% |
| 3666 | GOBP\_PHENOL\_CONTAINING\_COMPOUND\_METABOLIC\_PROCESS |  | 56 | -0.34 | -1.13 | 0.282 | 0.617 | 1.000 | 2569 | tags=30%, list=21%, signal=38% |
| 3667 | GOCC\_FILAMENTOUS\_ACTIN |  | 25 | -0.39 | -1.13 | 0.315 | 0.618 | 1.000 | 3251 | tags=28%, list=27%, signal=38% |
| 3668 | GOBP\_POSITIVE\_REGULATION\_OF\_CHOLESTEROL\_METABOLIC\_PROCESS |  | 6 | -0.56 | -1.13 | 0.325 | 0.618 | 1.000 | 3716 | tags=67%, list=31%, signal=96% |
| 3669 | GOBP\_REGULATION\_OF\_REPRODUCTIVE\_PROCESS |  | 84 | -0.32 | -1.13 | 0.278 | 0.618 | 1.000 | 3785 | tags=32%, list=32%, signal=47% |
| 3670 | GOBP\_APOPTOTIC\_CELL\_CLEARANCE |  | 36 | -0.36 | -1.13 | 0.300 | 0.618 | 1.000 | 3007 | tags=33%, list=25%, signal=44% |
| 3671 | GOBP\_GRANULOCYTE\_DIFFERENTIATION |  | 23 | -0.39 | -1.13 | 0.311 | 0.618 | 1.000 | 3395 | tags=39%, list=28%, signal=54% |
| 3672 | GOBP\_POSITIVE\_REGULATION\_OF\_MICROGLIAL\_CELL\_ACTIVATION |  | 5 | -0.58 | -1.13 | 0.308 | 0.618 | 1.000 | 4995 | tags=100%, list=42%, signal=171% |
| 3673 | GOBP\_GASTRULATION\_WITH\_MOUTH\_FORMING\_SECOND |  | 17 | -0.42 | -1.13 | 0.313 | 0.618 | 1.000 | 405 | tags=18%, list=3%, signal=18% |
| 3674 | GOMF\_METAL\_ION\_PROTON\_ANTIPORTER\_ACTIVITY |  | 11 | -0.47 | -1.13 | 0.321 | 0.618 | 1.000 | 654 | tags=36%, list=5%, signal=38% |
| 3675 | GOBP\_NEGATIVE\_REGULATION\_OF\_MOLECULAR\_FUNCTION |  | 699 | -0.28 | -1.13 | 0.087 | 0.618 | 1.000 | 4177 | tags=37%, list=35%, signal=54% |
| 3676 | GOBP\_REGULATION\_OF\_SYNAPTIC\_VESICLE\_RECYCLING |  | 16 | -0.43 | -1.13 | 0.322 | 0.618 | 1.000 | 3909 | tags=44%, list=33%, signal=65% |
| 3677 | GOBP\_I\_KAPPAB\_KINASE\_NF\_KAPPAB\_SIGNALING |  | 196 | -0.30 | -1.13 | 0.217 | 0.618 | 1.000 | 3645 | tags=35%, list=30%, signal=50% |
| 3678 | GOMF\_MODIFIED\_AMINO\_ACID\_TRANSMEMBRANE\_TRANSPORTER\_ACTIVITY |  | 16 | -0.43 | -1.13 | 0.321 | 0.618 | 1.000 | 2557 | tags=50%, list=21%, signal=63% |
| 3679 | GOBP\_REGULATION\_OF\_MONOOXYGENASE\_ACTIVITY |  | 39 | -0.35 | -1.13 | 0.297 | 0.619 | 1.000 | 3909 | tags=38%, list=33%, signal=57% |
| 3680 | GOMF\_SECONDARY\_ACTIVE\_TRANSMEMBRANE\_TRANSPORTER\_ACTIVITY |  | 149 | -0.30 | -1.13 | 0.240 | 0.619 | 1.000 | 2395 | tags=29%, list=20%, signal=36% |
| 3681 | GOBP\_PROTON\_TRANSPORTING\_V\_TYPE\_ATPASE\_COMPLEX\_ASSEMBLY |  | 5 | -0.58 | -1.13 | 0.316 | 0.619 | 1.000 | 2924 | tags=60%, list=24%, signal=79% |
| 3682 | GOBP\_POSTSYNAPTIC\_SPECIALIZATION\_ORGANIZATION |  | 20 | -0.40 | -1.12 | 0.316 | 0.620 | 1.000 | 3699 | tags=50%, list=31%, signal=72% |
| 3683 | GOCC\_RIBONUCLEOPROTEIN\_GRANULE |  | 172 | -0.30 | -1.12 | 0.231 | 0.620 | 1.000 | 4666 | tags=41%, list=39%, signal=66% |
| 3684 | GOBP\_PEPTIDE\_SECRETION |  | 262 | -0.29 | -1.12 | 0.188 | 0.620 | 1.000 | 1579 | tags=19%, list=13%, signal=22% |
| 3685 | GOMF\_ADENOSINE\_DEAMINASE\_ACTIVITY |  | 7 | -0.53 | -1.12 | 0.330 | 0.620 | 1.000 | 2903 | tags=57%, list=24%, signal=75% |
| 3686 | GOMF\_ATP\_DEPENDENT\_PEPTIDASE\_ACTIVITY |  | 7 | -0.53 | -1.12 | 0.322 | 0.620 | 1.000 | 2475 | tags=29%, list=21%, signal=36% |
| 3687 | GOMF\_NUCLEOTIDE\_RECEPTOR\_ACTIVITY |  | 16 | -0.43 | -1.12 | 0.318 | 0.620 | 1.000 | 4484 | tags=50%, list=37%, signal=80% |
| 3688 | GOBP\_CELLULAR\_RESPONSE\_TO\_CARBOHYDRATE\_STIMULUS |  | 89 | -0.32 | -1.12 | 0.266 | 0.620 | 1.000 | 3239 | tags=33%, list=27%, signal=44% |
| 3689 | GOBP\_PALLIUM\_DEVELOPMENT |  | 105 | -0.31 | -1.12 | 0.260 | 0.620 | 1.000 | 4903 | tags=46%, list=41%, signal=77% |
| 3690 | GOBP\_MALE\_GAMETE\_GENERATION |  | 263 | -0.29 | -1.12 | 0.201 | 0.620 | 1.000 | 4454 | tags=43%, list=37%, signal=66% |
| 3691 | GOMF\_PHOSPHATIDYLINOSITOL\_4\_5\_BISPHOSPHATE\_5\_PHOSPHATASE\_ACTIVITY |  | 9 | -0.49 | -1.12 | 0.318 | 0.620 | 1.000 | 4540 | tags=67%, list=38%, signal=107% |
| 3692 | GOBP\_AGING |  | 209 | -0.29 | -1.12 | 0.215 | 0.620 | 1.000 | 3675 | tags=34%, list=31%, signal=48% |
| 3693 | GOBP\_NEUROINFLAMMATORY\_RESPONSE |  | 26 | -0.38 | -1.12 | 0.310 | 0.620 | 1.000 | 1667 | tags=31%, list=14%, signal=36% |
| 3694 | GOBP\_REGULATION\_OF\_ASPARTIC\_TYPE\_ENDOPEPTIDASE\_ACTIVITY\_INVOLVED\_IN\_AMYLOID\_PRECURSOR\_PROTEIN\_CATABOLIC\_PROCESS |  | 7 | -0.53 | -1.12 | 0.335 | 0.620 | 1.000 | 2416 | tags=43%, list=20%, signal=54% |
| 3695 | GOBP\_NEGATIVE\_REGULATION\_OF\_CYSTEINE\_TYPE\_ENDOPEPTIDASE\_ACTIVITY |  | 53 | -0.34 | -1.12 | 0.285 | 0.620 | 1.000 | 3345 | tags=34%, list=28%, signal=47% |
| 3696 | GOBP\_REGULATION\_OF\_PRODUCTION\_OF\_MOLECULAR\_MEDIATOR\_OF\_IMMUNE\_RESPONSE |  | 82 | -0.32 | -1.12 | 0.278 | 0.620 | 1.000 | 3344 | tags=39%, list=28%, signal=54% |
| 3697 | GOMF\_MRNA\_BINDING |  | 200 | -0.30 | -1.12 | 0.212 | 0.620 | 1.000 | 5560 | tags=47%, list=46%, signal=85% |
| 3698 | GOBP\_KIDNEY\_EPITHELIUM\_DEVELOPMENT |  | 93 | -0.31 | -1.12 | 0.278 | 0.621 | 1.000 | 4111 | tags=41%, list=34%, signal=62% |
| 3699 | GOMF\_L\_AMINO\_ACID\_TRANSMEMBRANE\_TRANSPORTER\_ACTIVITY |  | 33 | -0.36 | -1.12 | 0.301 | 0.621 | 1.000 | 1222 | tags=21%, list=10%, signal=24% |
| 3700 | GOBP\_MOVEMENT\_IN\_HOST\_ENVIRONMENT |  | 108 | -0.31 | -1.12 | 0.259 | 0.621 | 1.000 | 2894 | tags=31%, list=24%, signal=40% |
| 3701 | GOBP\_BASEMENT\_MEMBRANE\_ASSEMBLY |  | 14 | -0.44 | -1.12 | 0.316 | 0.621 | 1.000 | 3690 | tags=43%, list=31%, signal=62% |
| 3702 | GOBP\_ACTIN\_CYTOSKELETON\_REORGANIZATION |  | 84 | -0.32 | -1.12 | 0.277 | 0.621 | 1.000 | 3728 | tags=37%, list=31%, signal=53% |
| 3703 | GOBP\_INTERMEDIATE\_FILAMENT\_BUNDLE\_ASSEMBLY |  | 3 | -0.67 | -1.12 | 0.314 | 0.621 | 1.000 | 3765 | tags=67%, list=31%, signal=97% |
| 3704 | GOBP\_ANATOMICAL\_STRUCTURE\_REGRESSION |  | 10 | -0.48 | -1.12 | 0.334 | 0.621 | 1.000 | 5085 | tags=70%, list=42%, signal=121% |
| 3705 | GOBP\_REGULATION\_OF\_ATPASE\_COUPLED\_CALCIUM\_TRANSMEMBRANE\_TRANSPORTER\_ACTIVITY |  | 5 | -0.58 | -1.12 | 0.315 | 0.621 | 1.000 | 2518 | tags=60%, list=21%, signal=76% |
| 3706 | GOBP\_NEGATIVE\_REGULATION\_OF\_BLOOD\_CIRCULATION |  | 13 | -0.45 | -1.12 | 0.332 | 0.621 | 1.000 | 4164 | tags=46%, list=35%, signal=71% |
| 3707 | GOMF\_AMINO\_ACID\_CATION\_SYMPORTER\_ACTIVITY |  | 11 | -0.46 | -1.12 | 0.324 | 0.621 | 1.000 | 1222 | tags=27%, list=10%, signal=30% |
| 3708 | GOBP\_POLARIZED\_EPITHELIAL\_CELL\_DIFFERENTIATION |  | 16 | -0.42 | -1.12 | 0.324 | 0.621 | 1.000 | 4949 | tags=50%, list=41%, signal=85% |
| 3709 | GOBP\_REGULATION\_OF\_INNATE\_IMMUNE\_RESPONSE |  | 177 | -0.30 | -1.12 | 0.229 | 0.622 | 1.000 | 3370 | tags=33%, list=28%, signal=46% |
| 3710 | GOBP\_NEGATIVE\_REGULATION\_OF\_VOLTAGE\_GATED\_CALCIUM\_CHANNEL\_ACTIVITY |  | 9 | -0.49 | -1.12 | 0.329 | 0.622 | 1.000 | 276 | tags=11%, list=2%, signal=11% |
| 3711 | GOBP\_NEGATIVE\_REGULATION\_OF\_TISSUE\_REMODELING |  | 13 | -0.45 | -1.12 | 0.327 | 0.623 | 1.000 | 1579 | tags=31%, list=13%, signal=35% |
| 3712 | GOCC\_TRANSPORT\_VESICLE |  | 267 | -0.29 | -1.12 | 0.195 | 0.622 | 1.000 | 3973 | tags=35%, list=33%, signal=51% |
| 3713 | GOBP\_T\_CELL\_MEDIATED\_IMMUNITY |  | 45 | -0.35 | -1.12 | 0.299 | 0.623 | 1.000 | 1781 | tags=29%, list=15%, signal=34% |
| 3714 | GOBP\_LEPTIN\_MEDIATED\_SIGNALING\_PATHWAY |  | 9 | -0.49 | -1.12 | 0.329 | 0.623 | 1.000 | 2915 | tags=33%, list=24%, signal=44% |
| 3715 | GOBP\_FOREBRAIN\_MORPHOGENESIS |  | 6 | -0.56 | -1.12 | 0.324 | 0.623 | 1.000 | 3392 | tags=50%, list=28%, signal=70% |
| 3716 | GOMF\_ANTIPORTER\_ACTIVITY |  | 58 | -0.33 | -1.12 | 0.290 | 0.623 | 1.000 | 1802 | tags=28%, list=15%, signal=32% |
| 3717 | GOMF\_ADP\_BINDING |  | 28 | -0.38 | -1.12 | 0.313 | 0.624 | 1.000 | 4186 | tags=54%, list=35%, signal=82% |
| 3718 | GOBP\_NEGATIVE\_REGULATION\_OF\_SUPRAMOLECULAR\_FIBER\_ORGANIZATION |  | 104 | -0.31 | -1.12 | 0.258 | 0.624 | 1.000 | 2634 | tags=29%, list=22%, signal=37% |
| 3719 | GOBP\_REGULATION\_OF\_MESENCHYMAL\_STEM\_CELL\_DIFFERENTIATION |  | 4 | -0.63 | -1.12 | 0.322 | 0.624 | 1.000 | 1881 | tags=50%, list=16%, signal=59% |
| 3720 | GOBP\_G\_PROTEIN\_COUPLED\_PURINERGIC\_NUCLEOTIDE\_RECEPTOR\_SIGNALING\_PATHWAY |  | 10 | -0.48 | -1.12 | 0.326 | 0.624 | 1.000 | 4148 | tags=50%, list=35%, signal=76% |
| 3721 | GOBP\_IMMATURE\_B\_CELL\_DIFFERENTIATION |  | 8 | -0.51 | -1.12 | 0.333 | 0.624 | 1.000 | 1667 | tags=38%, list=14%, signal=44% |
| 3722 | GOBP\_ANTIGEN\_PROCESSING\_AND\_PRESENTATION |  | 152 | -0.30 | -1.12 | 0.241 | 0.624 | 1.000 | 4343 | tags=39%, list=36%, signal=60% |
| 3723 | GOBP\_NEGATIVE\_REGULATION\_OF\_INTRACELLULAR\_SIGNAL\_TRANSDUCTION |  | 357 | -0.29 | -1.12 | 0.164 | 0.625 | 1.000 | 3530 | tags=34%, list=29%, signal=47% |
| 3724 | GOCC\_MICROTUBULE\_PLUS\_END |  | 18 | -0.41 | -1.12 | 0.321 | 0.625 | 1.000 | 3509 | tags=39%, list=29%, signal=55% |
| 3725 | GOMF\_GLUCOSYLTRANSFERASE\_ACTIVITY |  | 12 | -0.45 | -1.12 | 0.329 | 0.624 | 1.000 | 3305 | tags=42%, list=28%, signal=57% |
| 3726 | GOBP\_TISSUE\_REGENERATION |  | 45 | -0.34 | -1.12 | 0.297 | 0.625 | 1.000 | 2494 | tags=29%, list=21%, signal=36% |
| 3727 | GOBP\_ADHESION\_OF\_SYMBIONT\_TO\_HOST\_CELL |  | 6 | -0.55 | -1.12 | 0.332 | 0.625 | 1.000 | 2399 | tags=33%, list=20%, signal=42% |
| 3728 | GOBP\_REGULATION\_OF\_MAMMARY\_GLAND\_EPITHELIAL\_CELL\_PROLIFERATION |  | 15 | -0.43 | -1.12 | 0.328 | 0.625 | 1.000 | 1580 | tags=33%, list=13%, signal=38% |
| 3729 | GOCC\_INTRINSIC\_COMPONENT\_OF\_SYNAPTIC\_VESICLE\_MEMBRANE |  | 28 | -0.37 | -1.12 | 0.311 | 0.625 | 1.000 | 207 | tags=11%, list=2%, signal=11% |
| 3730 | GOCC\_NEURON\_PROJECTION\_CYTOPLASM |  | 65 | -0.33 | -1.12 | 0.293 | 0.625 | 1.000 | 3834 | tags=42%, list=32%, signal=61% |
| 3731 | GOBP\_RNA\_INTERFERENCE |  | 11 | -0.46 | -1.12 | 0.326 | 0.625 | 1.000 | 2714 | tags=36%, list=23%, signal=47% |
| 3732 | GOBP\_NEURON\_DEATH\_IN\_RESPONSE\_TO\_HYDROGEN\_PEROXIDE |  | 3 | -0.66 | -1.12 | 0.315 | 0.625 | 1.000 | 1935 | tags=33%, list=16%, signal=40% |
| 3733 | GOBP\_NEGATIVE\_REGULATION\_OF\_PROTEIN\_METABOLIC\_PROCESS |  | 690 | -0.28 | -1.12 | 0.092 | 0.625 | 1.000 | 4008 | tags=36%, list=33%, signal=51% |
| 3734 | GOBP\_NEGATIVE\_REGULATION\_OF\_VASCULAR\_ASSOCIATED\_SMOOTH\_MUSCLE\_CELL\_DIFFERENTIATION |  | 8 | -0.51 | -1.12 | 0.333 | 0.625 | 1.000 | 481 | tags=25%, list=4%, signal=26% |
| 3735 | GOBP\_REGULATION\_OF\_CYTOPLASMIC\_TRANSLATIONAL\_INITIATION |  | 4 | -0.62 | -1.12 | 0.317 | 0.625 | 1.000 | 3691 | tags=50%, list=31%, signal=72% |
| 3736 | GOBP\_NEGATIVE\_REGULATION\_OF\_LEUKOCYTE\_DEGRANULATION |  | 4 | -0.62 | -1.12 | 0.324 | 0.625 | 1.000 | 1608 | tags=50%, list=13%, signal=58% |
| 3737 | GOBP\_TELENCEPHALON\_DEVELOPMENT |  | 154 | -0.30 | -1.12 | 0.247 | 0.625 | 1.000 | 4696 | tags=45%, list=39%, signal=73% |
| 3738 | GOBP\_BIOMINERALIZATION |  | 98 | -0.31 | -1.12 | 0.267 | 0.625 | 1.000 | 3488 | tags=37%, list=29%, signal=51% |
| 3739 | GOMF\_BMP\_RECEPTOR\_ACTIVITY |  | 5 | -0.58 | -1.12 | 0.328 | 0.625 | 1.000 | 2886 | tags=60%, list=24%, signal=79% |
| 3740 | GOMF\_IONOTROPIC\_GLUTAMATE\_RECEPTOR\_ACTIVITY |  | 8 | -0.51 | -1.12 | 0.328 | 0.625 | 1.000 | 4846 | tags=75%, list=40%, signal=126% |
| 3741 | GOBP\_HAIR\_FOLLICLE\_MATURATION |  | 10 | -0.48 | -1.12 | 0.327 | 0.625 | 1.000 | 2212 | tags=20%, list=18%, signal=24% |
| 3742 | GOMF\_WIDE\_PORE\_CHANNEL\_ACTIVITY |  | 15 | -0.43 | -1.12 | 0.325 | 0.625 | 1.000 | 2313 | tags=47%, list=19%, signal=58% |
| 3743 | GOBP\_KYNURENINE\_METABOLIC\_PROCESS |  | 7 | -0.52 | -1.12 | 0.326 | 0.625 | 1.000 | 2851 | tags=57%, list=24%, signal=75% |
| 3744 | GOBP\_NEGATIVE\_REGULATION\_OF\_ENDOTHELIAL\_CELL\_APOPTOTIC\_PROCESS |  | 21 | -0.40 | -1.12 | 0.320 | 0.625 | 1.000 | 360 | tags=14%, list=3%, signal=15% |
| 3745 | GOBP\_NEGATIVE\_REGULATION\_OF\_GLUCOCORTICOID\_RECEPTOR\_SIGNALING\_PATHWAY |  | 5 | -0.58 | -1.12 | 0.327 | 0.625 | 1.000 | 4432 | tags=80%, list=37%, signal=127% |
| 3746 | GOCC\_ENDOPLASMIC\_RETICULUM\_PLASMA\_MEMBRANE\_CONTACT\_SITE |  | 8 | -0.51 | -1.12 | 0.337 | 0.625 | 1.000 | 3600 | tags=75%, list=30%, signal=107% |
| 3747 | GOBP\_PROLACTIN\_SECRETION |  | 6 | -0.55 | -1.12 | 0.330 | 0.625 | 1.000 | 1539 | tags=50%, list=13%, signal=57% |
| 3748 | GOCC\_PARASPECKLES |  | 5 | -0.58 | -1.12 | 0.323 | 0.626 | 1.000 | 4571 | tags=80%, list=38%, signal=129% |
| 3749 | GOCC\_INO80\_TYPE\_COMPLEX |  | 19 | -0.41 | -1.12 | 0.322 | 0.625 | 1.000 | 3857 | tags=53%, list=32%, signal=77% |
| 3750 | GOMF\_ACETYLTRANSFERASE\_ACTIVITY |  | 69 | -0.32 | -1.12 | 0.287 | 0.626 | 1.000 | 4219 | tags=41%, list=35%, signal=62% |
| 3751 | GOBP\_RRNA\_TRANSCRIPTION |  | 22 | -0.40 | -1.12 | 0.322 | 0.626 | 1.000 | 5712 | tags=55%, list=48%, signal=104% |
| 3752 | GOBP\_POSITIVE\_REGULATION\_OF\_MYELINATION |  | 13 | -0.45 | -1.12 | 0.340 | 0.626 | 1.000 | 5432 | tags=62%, list=45%, signal=112% |
| 3753 | GOBP\_SYNAPTIC\_TRANSMISSION\_GABAERGIC |  | 21 | -0.40 | -1.12 | 0.321 | 0.626 | 1.000 | 4907 | tags=62%, list=41%, signal=104% |
| 3754 | GOBP\_NEGATIVE\_REGULATION\_OF\_TOLL\_LIKE\_RECEPTOR\_4\_SIGNALING\_PATHWAY |  | 7 | -0.53 | -1.12 | 0.329 | 0.626 | 1.000 | 2420 | tags=57%, list=20%, signal=72% |
| 3755 | GOBP\_RESPONSE\_TO\_TUMOR\_NECROSIS\_FACTOR |  | 201 | -0.29 | -1.12 | 0.223 | 0.626 | 1.000 | 2820 | tags=27%, list=23%, signal=35% |
| 3756 | GOBP\_CORTICOSTEROID\_HORMONE\_SECRETION |  | 10 | -0.48 | -1.12 | 0.332 | 0.626 | 1.000 | 4836 | tags=60%, list=40%, signal=100% |
| 3757 | GOMF\_TRANSFERRIN\_RECEPTOR\_BINDING |  | 8 | -0.50 | -1.12 | 0.337 | 0.626 | 1.000 | 3547 | tags=38%, list=30%, signal=53% |
| 3758 | GOBP\_FOREBRAIN\_NEURON\_FATE\_COMMITMENT |  | 4 | -0.61 | -1.12 | 0.320 | 0.626 | 1.000 | 1871 | tags=50%, list=16%, signal=59% |
| 3759 | GOBP\_REGULATION\_OF\_SMAD\_PROTEIN\_COMPLEX\_ASSEMBLY |  | 5 | -0.58 | -1.12 | 0.335 | 0.626 | 1.000 | 3376 | tags=60%, list=28%, signal=83% |
| 3760 | GOMF\_G\_PROTEIN\_COUPLED\_PURINERGIC\_NUCLEOTIDE\_RECEPTOR\_ACTIVITY |  | 10 | -0.48 | -1.12 | 0.330 | 0.626 | 1.000 | 4148 | tags=50%, list=35%, signal=76% |
| 3761 | GOBP\_NUCLEAR\_TRANSPORT |  | 264 | -0.29 | -1.12 | 0.214 | 0.627 | 1.000 | 4292 | tags=36%, list=36%, signal=54% |
| 3762 | GOBP\_POSITIVE\_REGULATION\_OF\_ESTABLISHMENT\_OF\_PROTEIN\_LOCALIZATION |  | 233 | -0.29 | -1.12 | 0.209 | 0.627 | 1.000 | 3565 | tags=31%, list=30%, signal=44% |
| 3763 | GOBP\_CELLULAR\_RESPONSE\_TO\_MECHANICAL\_STIMULUS |  | 58 | -0.33 | -1.12 | 0.298 | 0.627 | 1.000 | 3269 | tags=36%, list=27%, signal=50% |
| 3764 | GOMF\_KINESIN\_BINDING |  | 30 | -0.37 | -1.12 | 0.310 | 0.627 | 1.000 | 1825 | tags=27%, list=15%, signal=31% |
| 3765 | GOBP\_SALIVARY\_GLAND\_DEVELOPMENT |  | 28 | -0.38 | -1.12 | 0.312 | 0.627 | 1.000 | 2457 | tags=29%, list=20%, signal=36% |
| 3766 | GOBP\_PURINERGIC\_NUCLEOTIDE\_RECEPTOR\_SIGNALING\_PATHWAY |  | 23 | -0.39 | -1.12 | 0.322 | 0.627 | 1.000 | 4484 | tags=48%, list=37%, signal=76% |
| 3767 | GOBP\_PROGRAMMED\_NECROTIC\_CELL\_DEATH |  | 32 | -0.36 | -1.12 | 0.313 | 0.627 | 1.000 | 1869 | tags=25%, list=16%, signal=30% |
| 3768 | GOCC\_EXTRINSIC\_COMPONENT\_OF\_POSTSYNAPTIC\_MEMBRANE |  | 5 | -0.58 | -1.12 | 0.330 | 0.627 | 1.000 | 145 | tags=20%, list=1%, signal=20% |
| 3769 | GOBP\_REGULATION\_OF\_SYSTEMIC\_ARTERIAL\_BLOOD\_PRESSURE\_BY\_CIRCULATORY\_RENIN\_ANGIOTENSIN |  | 13 | -0.44 | -1.12 | 0.332 | 0.627 | 1.000 | 2480 | tags=46%, list=21%, signal=58% |
| 3770 | GOMF\_BASIC\_AMINO\_ACID\_TRANSMEMBRANE\_TRANSPORTER\_ACTIVITY |  | 11 | -0.46 | -1.12 | 0.335 | 0.627 | 1.000 | 2026 | tags=27%, list=17%, signal=33% |
| 3771 | GOCC\_PHOSPHORYLASE\_KINASE\_COMPLEX |  | 4 | -0.62 | -1.12 | 0.326 | 0.627 | 1.000 | 2410 | tags=50%, list=20%, signal=63% |
| 3772 | GOBP\_HORMONE\_MEDIATED\_SIGNALING\_PATHWAY |  | 127 | -0.31 | -1.12 | 0.264 | 0.627 | 1.000 | 4512 | tags=48%, list=38%, signal=76% |
| 3773 | GOBP\_HISTONE\_H2A\_PHOSPHORYLATION |  | 5 | -0.58 | -1.12 | 0.323 | 0.627 | 1.000 | 3136 | tags=60%, list=26%, signal=81% |
| 3774 | GOMF\_EFFLUX\_TRANSMEMBRANE\_TRANSPORTER\_ACTIVITY |  | 12 | -0.46 | -1.12 | 0.328 | 0.627 | 1.000 | 2313 | tags=42%, list=19%, signal=52% |
| 3775 | GOBP\_NEGATIVE\_REGULATION\_OF\_TOLL\_LIKE\_RECEPTOR\_2\_SIGNALING\_PATHWAY |  | 4 | -0.62 | -1.12 | 0.323 | 0.628 | 1.000 | 2420 | tags=75%, list=20%, signal=94% |
| 3776 | GOBP\_REGULATION\_OF\_FOCAL\_ADHESION\_DISASSEMBLY |  | 6 | -0.55 | -1.12 | 0.330 | 0.628 | 1.000 | 2995 | tags=67%, list=25%, signal=89% |
| 3777 | GOBP\_REGULATION\_OF\_CELLULAR\_PROTEIN\_LOCALIZATION |  | 406 | -0.28 | -1.12 | 0.170 | 0.628 | 1.000 | 4373 | tags=37%, list=36%, signal=57% |
| 3778 | GOBP\_REGULATION\_OF\_PATTERN\_RECOGNITION\_RECEPTOR\_SIGNALING\_PATHWAY |  | 62 | -0.33 | -1.12 | 0.299 | 0.628 | 1.000 | 2480 | tags=34%, list=21%, signal=42% |
| 3779 | GOMF\_CATION\_CHANNEL\_ACTIVITY |  | 168 | -0.30 | -1.12 | 0.244 | 0.629 | 1.000 | 2216 | tags=23%, list=18%, signal=28% |
| 3780 | GOBP\_POSITIVE\_REGULATION\_OF\_PROTEIN\_LOCALIZATION\_TO\_NUCLEUS |  | 75 | -0.32 | -1.12 | 0.286 | 0.629 | 1.000 | 3968 | tags=32%, list=33%, signal=47% |
| 3781 | GOBP\_REGULATION\_OF\_PROTEIN\_KINASE\_C\_ACTIVITY |  | 4 | -0.62 | -1.12 | 0.329 | 0.629 | 1.000 | 3860 | tags=75%, list=32%, signal=110% |
| 3782 | GOCC\_PLATELET\_ALPHA\_GRANULE\_LUMEN |  | 41 | -0.35 | -1.12 | 0.306 | 0.629 | 1.000 | 3488 | tags=34%, list=29%, signal=48% |
| 3783 | GOBP\_SEMINIFEROUS\_TUBULE\_DEVELOPMENT |  | 9 | -0.49 | -1.12 | 0.336 | 0.631 | 1.000 | 4454 | tags=67%, list=37%, signal=106% |
| 3784 | GOBP\_MEIOTIC\_MISMATCH\_REPAIR |  | 3 | -0.67 | -1.12 | 0.318 | 0.631 | 1.000 | 3969 | tags=100%, list=33%, signal=149% |
| 3785 | GOBP\_CELL\_PROJECTION\_ASSEMBLY |  | 409 | -0.29 | -1.12 | 0.161 | 0.631 | 1.000 | 4018 | tags=35%, list=33%, signal=51% |
| 3786 | GOBP\_CEREBELLAR\_PURKINJE\_CELL\_LAYER\_DEVELOPMENT |  | 13 | -0.45 | -1.12 | 0.340 | 0.631 | 1.000 | 1351 | tags=31%, list=11%, signal=35% |
| 3787 | GOBP\_DNA\_REPLICATION\_INITIATION |  | 35 | -0.36 | -1.11 | 0.307 | 0.631 | 1.000 | 4446 | tags=54%, list=37%, signal=86% |
| 3788 | GOBP\_NEGATIVE\_REGULATION\_OF\_LYMPHOCYTE\_MIGRATION |  | 7 | -0.52 | -1.11 | 0.338 | 0.631 | 1.000 | 3613 | tags=43%, list=30%, signal=61% |
| 3789 | GOBP\_AMYLOID\_BETA\_METABOLIC\_PROCESS |  | 47 | -0.34 | -1.11 | 0.305 | 0.631 | 1.000 | 2460 | tags=28%, list=20%, signal=35% |
| 3790 | GOMF\_LIPID\_BINDING |  | 510 | -0.28 | -1.11 | 0.144 | 0.631 | 1.000 | 3052 | tags=29%, list=25%, signal=37% |
| 3791 | GOMF\_ANION\_TRANSMEMBRANE\_TRANSPORTER\_ACTIVITY |  | 276 | -0.29 | -1.11 | 0.206 | 0.631 | 1.000 | 3022 | tags=32%, list=25%, signal=42% |
| 3792 | GOBP\_POSITIVE\_REGULATION\_OF\_DNA\_METABOLIC\_PROCESS |  | 142 | -0.30 | -1.11 | 0.261 | 0.631 | 1.000 | 3452 | tags=32%, list=29%, signal=44% |
| 3793 | GOBP\_NEGATIVE\_REGULATION\_OF\_LIPID\_LOCALIZATION |  | 31 | -0.37 | -1.11 | 0.315 | 0.631 | 1.000 | 3962 | tags=58%, list=33%, signal=86% |
| 3794 | GOBP\_REGULATION\_OF\_SUPEROXIDE\_ANION\_GENERATION |  | 13 | -0.44 | -1.11 | 0.338 | 0.631 | 1.000 | 4249 | tags=54%, list=35%, signal=83% |
| 3795 | GOCC\_GOLGI\_APPARATUS\_SUBCOMPARTMENT |  | 630 | -0.28 | -1.11 | 0.116 | 0.631 | 1.000 | 3582 | tags=31%, list=30%, signal=42% |
| 3796 | GOBP\_IMMUNE\_EFFECTOR\_PROCESS |  | 721 | -0.28 | -1.11 | 0.102 | 0.631 | 1.000 | 3549 | tags=35%, list=30%, signal=46% |
| 3797 | GOBP\_PROTEIN\_HOMOOLIGOMERIZATION |  | 108 | -0.31 | -1.11 | 0.279 | 0.631 | 1.000 | 1392 | tags=17%, list=12%, signal=19% |
| 3798 | GOCC\_CLATHRIN\_COMPLEX |  | 7 | -0.53 | -1.11 | 0.336 | 0.631 | 1.000 | 4718 | tags=71%, list=39%, signal=118% |
| 3799 | GOBP\_EPITHELIAL\_CELL\_APOPTOTIC\_PROCESS |  | 73 | -0.32 | -1.11 | 0.290 | 0.631 | 1.000 | 1748 | tags=22%, list=15%, signal=25% |
| 3800 | GOCC\_CELL\_SUBSTRATE\_JUNCTION |  | 345 | -0.29 | -1.11 | 0.184 | 0.632 | 1.000 | 3609 | tags=29%, list=30%, signal=40% |
| 3801 | GOBP\_GLOMERULAR\_EPITHELIAL\_CELL\_DEVELOPMENT |  | 9 | -0.49 | -1.11 | 0.339 | 0.632 | 1.000 | 3962 | tags=44%, list=33%, signal=66% |
| 3802 | GOBP\_RNA\_SECONDARY\_STRUCTURE\_UNWINDING |  | 5 | -0.57 | -1.11 | 0.333 | 0.632 | 1.000 | 2731 | tags=60%, list=23%, signal=78% |
| 3803 | GOBP\_DOUBLE\_STRAND\_BREAK\_REPAIR\_INVOLVED\_IN\_MEIOTIC\_RECOMBINATION |  | 3 | -0.67 | -1.11 | 0.330 | 0.632 | 1.000 | 3701 | tags=67%, list=31%, signal=96% |
| 3804 | GOBP\_REGULATION\_OF\_PROTEIN\_LOCALIZATION |  | 618 | -0.28 | -1.11 | 0.123 | 0.632 | 1.000 | 4373 | tags=38%, list=36%, signal=57% |
| 3805 | GOBP\_REGULATION\_OF\_GLUCOSE\_TRANSMEMBRANE\_TRANSPORT |  | 50 | -0.34 | -1.11 | 0.316 | 0.632 | 1.000 | 2590 | tags=30%, list=22%, signal=38% |
| 3806 | GOBP\_ACTIVATION\_OF\_CYSTEINE\_TYPE\_ENDOPEPTIDASE\_ACTIVITY |  | 9 | -0.49 | -1.11 | 0.335 | 0.632 | 1.000 | 2151 | tags=44%, list=18%, signal=54% |
| 3807 | GOBP\_ESTABLISHMENT\_OF\_PROTEIN\_LOCALIZATION\_TO\_PLASMA\_MEMBRANE |  | 49 | -0.34 | -1.11 | 0.310 | 0.631 | 1.000 | 4314 | tags=45%, list=36%, signal=70% |
| 3808 | GOMF\_SUMO\_LIGASE\_ACTIVITY |  | 7 | -0.53 | -1.11 | 0.349 | 0.631 | 1.000 | 932 | tags=29%, list=8%, signal=31% |
| 3809 | GOBP\_NEGATIVE\_REGULATION\_OF\_ENDOPLASMIC\_RETICULUM\_STRESS\_INDUCED\_EIF2\_ALPHA\_PHOSPHORYLATION |  | 4 | -0.62 | -1.11 | 0.338 | 0.631 | 1.000 | 3691 | tags=50%, list=31%, signal=72% |
| 3810 | GOBP\_REGULATION\_OF\_PEPTIDE\_SECRETION |  | 197 | -0.29 | -1.11 | 0.238 | 0.631 | 1.000 | 2038 | tags=23%, list=17%, signal=28% |
| 3811 | GOBP\_MAMMARY\_GLAND\_DEVELOPMENT |  | 95 | -0.31 | -1.11 | 0.279 | 0.632 | 1.000 | 3205 | tags=29%, list=27%, signal=40% |
| 3812 | GOBP\_MALE\_SEX\_DIFFERENTIATION |  | 102 | -0.31 | -1.11 | 0.272 | 0.632 | 1.000 | 4868 | tags=53%, list=41%, signal=88% |
| 3813 | GOBP\_GLIOGENESIS |  | 193 | -0.29 | -1.11 | 0.237 | 0.632 | 1.000 | 1968 | tags=20%, list=16%, signal=23% |
| 3814 | GOBP\_POSITIVE\_REGULATION\_OF\_PROTEIN\_CATABOLIC\_PROCESS |  | 173 | -0.29 | -1.11 | 0.255 | 0.632 | 1.000 | 3988 | tags=35%, list=33%, signal=51% |
| 3815 | GOBP\_REGULATION\_OF\_CHONDROCYTE\_DEVELOPMENT |  | 4 | -0.62 | -1.11 | 0.325 | 0.632 | 1.000 | 1441 | tags=50%, list=12%, signal=57% |
| 3816 | GOMF\_NADPH\_BINDING |  | 14 | -0.43 | -1.11 | 0.331 | 0.632 | 1.000 | 1223 | tags=29%, list=10%, signal=32% |
| 3817 | GOBP\_NEGATIVE\_REGULATION\_OF\_SCHWANN\_CELL\_PROLIFERATION |  | 5 | -0.58 | -1.11 | 0.333 | 0.632 | 1.000 | 1297 | tags=40%, list=11%, signal=45% |
| 3818 | GOBP\_REGULATION\_OF\_DNA\_CATABOLIC\_PROCESS |  | 7 | -0.52 | -1.11 | 0.343 | 0.632 | 1.000 | 3325 | tags=57%, list=28%, signal=79% |
| 3819 | GOBP\_EXCITATORY\_CHEMICAL\_SYNAPTIC\_TRANSMISSION |  | 5 | -0.57 | -1.11 | 0.335 | 0.632 | 1.000 | 4950 | tags=80%, list=41%, signal=136% |
| 3820 | GOBP\_POSITIVE\_REGULATION\_OF\_DNA\_BIOSYNTHETIC\_PROCESS |  | 55 | -0.33 | -1.11 | 0.296 | 0.632 | 1.000 | 2010 | tags=22%, list=17%, signal=26% |
| 3821 | GOBP\_MONOCARBOXYLIC\_ACID\_TRANSPORT |  | 110 | -0.31 | -1.11 | 0.282 | 0.632 | 1.000 | 2392 | tags=31%, list=20%, signal=38% |
| 3822 | GOBP\_NEGATIVE\_REGULATION\_OF\_HUMORAL\_IMMUNE\_RESPONSE\_MEDIATED\_BY\_CIRCULATING\_IMMUNOGLOBULIN |  | 4 | -0.62 | -1.11 | 0.340 | 0.632 | 1.000 | 3944 | tags=75%, list=33%, signal=112% |
| 3823 | GOBP\_MODIFIED\_AMINO\_ACID\_TRANSPORT |  | 20 | -0.40 | -1.11 | 0.334 | 0.632 | 1.000 | 2557 | tags=45%, list=21%, signal=57% |
| 3824 | GOBP\_RETROGRADE\_TRANSPORT\_ENDOSOME\_TO\_GOLGI |  | 67 | -0.33 | -1.11 | 0.295 | 0.632 | 1.000 | 4260 | tags=40%, list=35%, signal=62% |
| 3825 | GOBP\_REGULATION\_OF\_SYSTEMIC\_ARTERIAL\_BLOOD\_PRESSURE\_BY\_RENIN\_ANGIOTENSIN |  | 16 | -0.42 | -1.11 | 0.337 | 0.632 | 1.000 | 2480 | tags=44%, list=21%, signal=55% |
| 3826 | GOBP\_RETINAL\_CONE\_CELL\_DIFFERENTIATION |  | 5 | -0.58 | -1.11 | 0.336 | 0.632 | 1.000 | 3868 | tags=80%, list=32%, signal=118% |
| 3827 | GOBP\_REGULATION\_OF\_ANIMAL\_ORGAN\_FORMATION |  | 17 | -0.41 | -1.11 | 0.335 | 0.632 | 1.000 | 1459 | tags=29%, list=12%, signal=33% |
| 3828 | GOBP\_CELL\_FATE\_COMMITMENT\_INVOLVED\_IN\_PATTERN\_SPECIFICATION |  | 6 | -0.55 | -1.11 | 0.343 | 0.632 | 1.000 | 1871 | tags=50%, list=16%, signal=59% |
| 3829 | GOBP\_BEHAVIORAL\_RESPONSE\_TO\_COCAINE |  | 9 | -0.49 | -1.11 | 0.343 | 0.633 | 1.000 | 1737 | tags=33%, list=14%, signal=39% |
| 3830 | GOBP\_MICROGLIAL\_CELL\_ACTIVATION |  | 23 | -0.39 | -1.11 | 0.329 | 0.633 | 1.000 | 1667 | tags=30%, list=14%, signal=35% |
| 3831 | GOBP\_POSITIVE\_REGULATION\_OF\_OXIDATIVE\_STRESS\_INDUCED\_NEURON\_DEATH |  | 3 | -0.66 | -1.11 | 0.329 | 0.633 | 1.000 | 1230 | tags=33%, list=10%, signal=37% |
| 3832 | GOBP\_POSITIVE\_REGULATION\_OF\_PEPTIDE\_SECRETION |  | 95 | -0.31 | -1.11 | 0.279 | 0.633 | 1.000 | 3269 | tags=36%, list=27%, signal=49% |
| 3833 | GOBP\_SOMATIC\_RECOMBINATION\_OF\_IMMUNOGLOBULIN\_GENE\_SEGMENTS |  | 40 | -0.35 | -1.11 | 0.326 | 0.633 | 1.000 | 3632 | tags=43%, list=30%, signal=61% |
| 3834 | GOBP\_CELLULAR\_RESPONSE\_TO\_LIPID |  | 349 | -0.28 | -1.11 | 0.190 | 0.633 | 1.000 | 3709 | tags=36%, list=31%, signal=50% |
| 3835 | GOBP\_EXTRAEMBRYONIC\_MEMBRANE\_DEVELOPMENT |  | 6 | -0.54 | -1.11 | 0.335 | 0.633 | 1.000 | 2639 | tags=50%, list=22%, signal=64% |
| 3836 | GOBP\_REGULATION\_OF\_ARP2\_3\_COMPLEX\_MEDIATED\_ACTIN\_NUCLEATION |  | 16 | -0.42 | -1.11 | 0.338 | 0.634 | 1.000 | 3032 | tags=44%, list=25%, signal=58% |
| 3837 | GOBP\_REGULATION\_OF\_PROTEOLYSIS |  | 448 | -0.28 | -1.11 | 0.158 | 0.634 | 1.000 | 3345 | tags=29%, list=28%, signal=38% |
| 3838 | GOBP\_REGULATION\_OF\_GLUCAN\_BIOSYNTHETIC\_PROCESS |  | 18 | -0.41 | -1.11 | 0.325 | 0.634 | 1.000 | 3934 | tags=50%, list=33%, signal=74% |
| 3839 | GOBP\_MAMMARY\_GLAND\_EPITHELIUM\_DEVELOPMENT |  | 47 | -0.34 | -1.11 | 0.311 | 0.634 | 1.000 | 2769 | tags=30%, list=23%, signal=39% |
| 3840 | GOBP\_CELLULAR\_RESPONSE\_TO\_TOPOLOGICALLY\_INCORRECT\_PROTEIN |  | 129 | -0.30 | -1.11 | 0.277 | 0.634 | 1.000 | 4948 | tags=47%, list=41%, signal=80% |
| 3841 | GOBP\_CALCIUM\_MEDIATED\_SIGNALING\_USING\_INTRACELLULAR\_CALCIUM\_SOURCE |  | 13 | -0.44 | -1.11 | 0.342 | 0.634 | 1.000 | 3313 | tags=46%, list=28%, signal=64% |
| 3842 | GOBP\_NEGATIVE\_REGULATION\_OF\_GTPASE\_ACTIVITY |  | 31 | -0.37 | -1.11 | 0.328 | 0.635 | 1.000 | 4904 | tags=55%, list=41%, signal=92% |
| 3843 | GOBP\_CELLULAR\_RESPONSE\_TO\_STARVATION |  | 124 | -0.30 | -1.11 | 0.269 | 0.635 | 1.000 | 3993 | tags=38%, list=33%, signal=56% |
| 3844 | GOBP\_POSITIVE\_REGULATION\_OF\_LYMPHOCYTE\_APOPTOTIC\_PROCESS |  | 8 | -0.50 | -1.11 | 0.341 | 0.635 | 1.000 | 3219 | tags=50%, list=27%, signal=68% |
| 3845 | GOBP\_MODIFICATION\_DEPENDENT\_MACROMOLECULE\_CATABOLIC\_PROCESS |  | 492 | -0.28 | -1.11 | 0.158 | 0.635 | 1.000 | 4036 | tags=34%, list=34%, signal=49% |
| 3846 | GOBP\_INTERLEUKIN\_23\_MEDIATED\_SIGNALING\_PATHWAY |  | 6 | -0.55 | -1.11 | 0.340 | 0.635 | 1.000 | 960 | tags=33%, list=8%, signal=36% |
| 3847 | GOBP\_NUCLEAR\_BODY\_ORGANIZATION |  | 10 | -0.47 | -1.11 | 0.348 | 0.635 | 1.000 | 4104 | tags=50%, list=34%, signal=76% |
| 3848 | GOBP\_POSITIVE\_REGULATION\_OF\_CARDIAC\_MUSCLE\_CELL\_DIFFERENTIATION |  | 7 | -0.52 | -1.11 | 0.340 | 0.635 | 1.000 | 2474 | tags=29%, list=21%, signal=36% |
| 3849 | GOCC\_RNA\_POLYMERASE\_II\_TRANSCRIPTION\_REGULATOR\_COMPLEX |  | 132 | -0.30 | -1.11 | 0.275 | 0.636 | 1.000 | 4007 | tags=36%, list=33%, signal=53% |
| 3850 | GOBP\_REGULATION\_OF\_PEPTIDASE\_ACTIVITY |  | 251 | -0.29 | -1.11 | 0.226 | 0.636 | 1.000 | 3345 | tags=30%, list=28%, signal=41% |
| 3851 | GOBP\_ALCOHOL\_METABOLIC\_PROCESS |  | 258 | -0.29 | -1.11 | 0.219 | 0.636 | 1.000 | 2786 | tags=26%, list=23%, signal=33% |
| 3852 | GOBP\_REGULATION\_OF\_NON\_MOTILE\_CILIUM\_ASSEMBLY |  | 6 | -0.55 | -1.11 | 0.334 | 0.636 | 1.000 | 2688 | tags=33%, list=22%, signal=43% |
| 3853 | GOBP\_POSITIVE\_REGULATION\_OF\_MEIOTIC\_CELL\_CYCLE\_PHASE\_TRANSITION |  | 3 | -0.66 | -1.11 | 0.327 | 0.636 | 1.000 | 441 | tags=33%, list=4%, signal=35% |
| 3854 | GOCC\_POTASSIUM\_CHANNEL\_COMPLEX |  | 50 | -0.34 | -1.11 | 0.309 | 0.636 | 1.000 | 2192 | tags=24%, list=18%, signal=29% |
| 3855 | GOBP\_REGULATION\_OF\_ENDOTHELIAL\_CELL\_CHEMOTAXIS |  | 15 | -0.43 | -1.11 | 0.334 | 0.636 | 1.000 | 3588 | tags=47%, list=30%, signal=66% |
| 3856 | GOBP\_REGULATION\_OF\_UBIQUITIN\_DEPENDENT\_PROTEIN\_CATABOLIC\_PROCESS |  | 120 | -0.30 | -1.11 | 0.280 | 0.636 | 1.000 | 3715 | tags=29%, list=31%, signal=42% |
| 3857 | GOBP\_REGULATION\_OF\_CYCLASE\_ACTIVITY |  | 24 | -0.38 | -1.11 | 0.330 | 0.636 | 1.000 | 3553 | tags=38%, list=30%, signal=53% |
| 3858 | GOBP\_CELL\_DIFFERENTIATION\_IN\_SPINAL\_CORD |  | 27 | -0.38 | -1.11 | 0.341 | 0.637 | 1.000 | 1881 | tags=26%, list=16%, signal=31% |
| 3859 | GOMF\_NON\_MEMBRANE\_SPANNING\_PROTEIN\_TYROSINE\_PHOSPHATASE\_ACTIVITY |  | 7 | -0.53 | -1.11 | 0.339 | 0.637 | 1.000 | 3692 | tags=71%, list=31%, signal=103% |
| 3860 | GOBP\_POSITIVE\_REGULATION\_OF\_TROPHOBLAST\_CELL\_MIGRATION |  | 6 | -0.55 | -1.11 | 0.348 | 0.637 | 1.000 | 3657 | tags=67%, list=30%, signal=96% |
| 3861 | GOBP\_PHASIC\_SMOOTH\_MUSCLE\_CONTRACTION |  | 14 | -0.43 | -1.11 | 0.346 | 0.637 | 1.000 | 374 | tags=21%, list=3%, signal=22% |
| 3862 | GOBP\_REGULATION\_OF\_SPINDLE\_ORGANIZATION |  | 32 | -0.36 | -1.11 | 0.325 | 0.637 | 1.000 | 4111 | tags=44%, list=34%, signal=66% |
| 3863 | GOBP\_NEGATIVE\_REGULATION\_OF\_MUSCLE\_TISSUE\_DEVELOPMENT |  | 8 | -0.50 | -1.11 | 0.343 | 0.637 | 1.000 | 624 | tags=13%, list=5%, signal=13% |
| 3864 | GOBP\_POSITIVE\_REGULATION\_OF\_METAPHASE\_ANAPHASE\_TRANSITION\_OF\_CELL\_CYCLE |  | 14 | -0.43 | -1.11 | 0.346 | 0.637 | 1.000 | 6486 | tags=71%, list=54%, signal=155% |
| 3865 | GOBP\_MITOCHONDRIAL\_PROTEIN\_CATABOLIC\_PROCESS |  | 4 | -0.61 | -1.11 | 0.339 | 0.637 | 1.000 | 4664 | tags=100%, list=39%, signal=163% |
| 3866 | GOBP\_NEGATIVE\_REGULATION\_OF\_CATALYTIC\_ACTIVITY |  | 491 | -0.28 | -1.11 | 0.151 | 0.637 | 1.000 | 4027 | tags=36%, list=34%, signal=52% |
| 3867 | GOMF\_ARP2\_3\_COMPLEX\_BINDING |  | 9 | -0.48 | -1.11 | 0.350 | 0.637 | 1.000 | 3559 | tags=56%, list=30%, signal=79% |
| 3868 | GOBP\_APOLIPOPROTEIN\_A\_I\_MEDIATED\_SIGNALING\_PATHWAY |  | 4 | -0.61 | -1.11 | 0.330 | 0.637 | 1.000 | 2752 | tags=50%, list=23%, signal=65% |
| 3869 | GOBP\_NEGATIVE\_REGULATION\_OF\_CELL\_MIGRATION\_INVOLVED\_IN\_SPROUTING\_ANGIOGENESIS |  | 14 | -0.43 | -1.11 | 0.335 | 0.637 | 1.000 | 2820 | tags=29%, list=23%, signal=37% |
| 3870 | GOBP\_POSITIVE\_REGULATION\_OF\_GRANULOCYTE\_DIFFERENTIATION |  | 6 | -0.54 | -1.11 | 0.342 | 0.637 | 1.000 | 1605 | tags=50%, list=13%, signal=58% |
| 3871 | GOBP\_POSITIVE\_REGULATION\_OF\_PROTEOLYSIS\_INVOLVED\_IN\_CELLULAR\_PROTEIN\_CATABOLIC\_PROCESS |  | 91 | -0.31 | -1.11 | 0.290 | 0.638 | 1.000 | 1637 | tags=18%, list=14%, signal=20% |
| 3872 | GOMF\_CELL\_MATRIX\_ADHESION\_MEDIATOR\_ACTIVITY |  | 5 | -0.57 | -1.11 | 0.352 | 0.638 | 1.000 | 2181 | tags=40%, list=18%, signal=49% |
| 3873 | GOBP\_MESODERMAL\_CELL\_FATE\_COMMITMENT |  | 7 | -0.52 | -1.11 | 0.342 | 0.639 | 1.000 | 14 | tags=14%, list=0%, signal=14% |
| 3874 | GOBP\_POSTSYNAPTIC\_MODULATION\_OF\_CHEMICAL\_SYNAPTIC\_TRANSMISSION |  | 8 | -0.50 | -1.11 | 0.351 | 0.639 | 1.000 | 5340 | tags=63%, list=44%, signal=112% |
| 3875 | GOBP\_REGULATION\_OF\_MRNA\_3\_END\_PROCESSING |  | 23 | -0.38 | -1.11 | 0.339 | 0.639 | 1.000 | 5165 | tags=57%, list=43%, signal=99% |
| 3876 | GOMF\_CARD\_DOMAIN\_BINDING |  | 10 | -0.47 | -1.11 | 0.347 | 0.639 | 1.000 | 5798 | tags=70%, list=48%, signal=135% |
| 3877 | GOBP\_NEGATIVE\_REGULATION\_OF\_CHROMATIN\_ORGANIZATION |  | 38 | -0.35 | -1.11 | 0.328 | 0.639 | 1.000 | 4561 | tags=55%, list=38%, signal=89% |
| 3878 | GOBP\_PHOSPHATE\_ION\_TRANSMEMBRANE\_TRANSPORT |  | 13 | -0.44 | -1.11 | 0.349 | 0.639 | 1.000 | 2395 | tags=31%, list=20%, signal=38% |
| 3879 | GOBP\_REGULATION\_OF\_LIPID\_TRANSPORT |  | 92 | -0.31 | -1.11 | 0.300 | 0.639 | 1.000 | 4338 | tags=45%, list=36%, signal=69% |
| 3880 | GOBP\_AXO\_DENDRITIC\_PROTEIN\_TRANSPORT |  | 9 | -0.49 | -1.11 | 0.349 | 0.639 | 1.000 | 3668 | tags=78%, list=31%, signal=112% |
| 3881 | GOBP\_RESPONSE\_TO\_INORGANIC\_SUBSTANCE |  | 358 | -0.28 | -1.11 | 0.190 | 0.639 | 1.000 | 3405 | tags=32%, list=28%, signal=43% |
| 3882 | GOCC\_CATION\_CHANNEL\_COMPLEX |  | 110 | -0.30 | -1.11 | 0.287 | 0.639 | 1.000 | 2201 | tags=23%, list=18%, signal=28% |
| 3883 | GOBP\_BLOOD\_VESSEL\_ENDOTHELIAL\_CELL\_PROLIFERATION\_INVOLVED\_IN\_SPROUTING\_ANGIOGENESIS |  | 17 | -0.41 | -1.11 | 0.347 | 0.639 | 1.000 | 3473 | tags=47%, list=29%, signal=66% |
| 3884 | GOMF\_ACTIVE\_TRANSMEMBRANE\_TRANSPORTER\_ACTIVITY |  | 192 | -0.29 | -1.11 | 0.258 | 0.639 | 1.000 | 3046 | tags=34%, list=25%, signal=45% |
| 3885 | GOBP\_CELL\_MATURATION |  | 99 | -0.31 | -1.11 | 0.288 | 0.639 | 1.000 | 2411 | tags=28%, list=20%, signal=35% |
| 3886 | GOBP\_REGULATION\_OF\_PEPTIDYL\_SERINE\_DEPHOSPHORYLATION |  | 4 | -0.61 | -1.11 | 0.337 | 0.639 | 1.000 | 3473 | tags=50%, list=29%, signal=70% |
| 3887 | GOBP\_NEGATIVE\_REGULATION\_OF\_LIPID\_KINASE\_ACTIVITY |  | 6 | -0.54 | -1.11 | 0.351 | 0.639 | 1.000 | 2289 | tags=33%, list=19%, signal=41% |
| 3888 | GOBP\_POSITIVE\_REGULATION\_OF\_SMOOTH\_MUSCLE\_CELL\_PROLIFERATION |  | 67 | -0.32 | -1.11 | 0.302 | 0.639 | 1.000 | 3286 | tags=39%, list=27%, signal=53% |
| 3889 | GOBP\_REGULATION\_OF\_ENDOCYTIC\_RECYCLING |  | 6 | -0.54 | -1.11 | 0.347 | 0.640 | 1.000 | 1202 | tags=33%, list=10%, signal=37% |
| 3890 | GOBP\_REGULATION\_OF\_CELL\_CELL\_ADHESION\_MEDIATED\_BY\_CADHERIN |  | 17 | -0.41 | -1.11 | 0.343 | 0.640 | 1.000 | 3269 | tags=47%, list=27%, signal=65% |
| 3891 | GOMF\_CONNEXIN\_BINDING |  | 4 | -0.61 | -1.11 | 0.338 | 0.640 | 1.000 | 3426 | tags=75%, list=29%, signal=105% |
| 3892 | GOBP\_REGULATION\_OF\_RESPONSE\_TO\_INTERFERON\_GAMMA |  | 18 | -0.41 | -1.11 | 0.343 | 0.640 | 1.000 | 1763 | tags=39%, list=15%, signal=46% |
| 3893 | GOBP\_RECEPTOR\_CATABOLIC\_PROCESS |  | 30 | -0.37 | -1.11 | 0.331 | 0.640 | 1.000 | 4775 | tags=53%, list=40%, signal=88% |
| 3894 | GOBP\_POSITIVE\_REGULATION\_OF\_RRNA\_PROCESSING |  | 7 | -0.52 | -1.11 | 0.349 | 0.640 | 1.000 | 4278 | tags=57%, list=36%, signal=89% |
| 3895 | GOMF\_CORTICOTROPIN\_RELEASING\_HORMONE\_RECEPTOR\_BINDING |  | 3 | -0.66 | -1.11 | 0.333 | 0.640 | 1.000 | 3890 | tags=67%, list=32%, signal=99% |
| 3896 | GOBP\_RESPONSE\_TO\_INTERLEUKIN\_1 |  | 136 | -0.30 | -1.11 | 0.274 | 0.641 | 1.000 | 3609 | tags=33%, list=30%, signal=47% |
| 3897 | GOMF\_STRUCTURAL\_MOLECULE\_ACTIVITY\_CONFERRING\_ELASTICITY |  | 7 | -0.52 | -1.11 | 0.350 | 0.641 | 1.000 | 3156 | tags=43%, list=26%, signal=58% |
| 3898 | GOBP\_HEMATOPOIETIC\_STEM\_CELL\_HOMEOSTASIS |  | 10 | -0.47 | -1.11 | 0.346 | 0.641 | 1.000 | 3061 | tags=40%, list=25%, signal=54% |
| 3899 | GOBP\_RELEASE\_OF\_SEQUESTERED\_CALCIUM\_ION\_INTO\_CYTOSOL\_BY\_ENDOPLASMIC\_RETICULUM |  | 19 | -0.40 | -1.11 | 0.354 | 0.641 | 1.000 | 5062 | tags=47%, list=42%, signal=82% |
| 3900 | GOBP\_CELLULAR\_RESPONSE\_TO\_RADIATION |  | 125 | -0.30 | -1.10 | 0.296 | 0.641 | 1.000 | 3927 | tags=39%, list=33%, signal=58% |
| 3901 | GOBP\_N\_ACETYLGLUCOSAMINE\_METABOLIC\_PROCESS |  | 14 | -0.43 | -1.10 | 0.352 | 0.641 | 1.000 | 4780 | tags=57%, list=40%, signal=95% |
| 3902 | GOBP\_REGULATION\_OF\_CALCIUM\_ION\_TRANSPORT\_INTO\_CYTOSOL |  | 62 | -0.33 | -1.10 | 0.306 | 0.641 | 1.000 | 3027 | tags=29%, list=25%, signal=39% |
| 3903 | GOBP\_DRUG\_CATABOLIC\_PROCESS |  | 9 | -0.49 | -1.10 | 0.354 | 0.641 | 1.000 | 3094 | tags=67%, list=26%, signal=90% |
| 3904 | GOBP\_NEGATIVE\_REGULATION\_OF\_PHOSPHATIDYLINOSITOL\_3\_KINASE\_SIGNALING |  | 11 | -0.46 | -1.10 | 0.347 | 0.641 | 1.000 | 6210 | tags=82%, list=52%, signal=169% |
| 3905 | GOBP\_BONE\_RESORPTION |  | 45 | -0.34 | -1.10 | 0.328 | 0.641 | 1.000 | 1457 | tags=24%, list=12%, signal=28% |
| 3906 | GOBP\_VASCULAR\_ASSOCIATED\_SMOOTH\_MUSCLE\_CELL\_DIFFERENTIATION |  | 23 | -0.39 | -1.10 | 0.349 | 0.641 | 1.000 | 3561 | tags=35%, list=30%, signal=49% |
| 3907 | GOMF\_POLYSACCHARIDE\_BINDING |  | 11 | -0.46 | -1.10 | 0.340 | 0.641 | 1.000 | 3566 | tags=45%, list=30%, signal=65% |
| 3908 | GOBP\_SKELETAL\_MUSCLE\_CELL\_PROLIFERATION |  | 11 | -0.46 | -1.10 | 0.348 | 0.641 | 1.000 | 3565 | tags=55%, list=30%, signal=77% |
| 3909 | GOBP\_PROTEIN\_MONOUBIQUITINATION |  | 56 | -0.33 | -1.10 | 0.317 | 0.641 | 1.000 | 4687 | tags=45%, list=39%, signal=73% |
| 3910 | GOBP\_ZINC\_ION\_TRANSPORT |  | 16 | -0.41 | -1.10 | 0.345 | 0.641 | 1.000 | 5393 | tags=69%, list=45%, signal=125% |
| 3911 | GOBP\_OXIDATIVE\_DNA\_DEMETHYLATION |  | 6 | -0.54 | -1.10 | 0.348 | 0.642 | 1.000 | 4508 | tags=67%, list=38%, signal=107% |
| 3912 | GOCC\_MEMBRANE\_COAT |  | 75 | -0.32 | -1.10 | 0.303 | 0.642 | 1.000 | 4424 | tags=49%, list=37%, signal=78% |
| 3913 | GOBP\_ADHERENS\_JUNCTION\_MAINTENANCE |  | 3 | -0.66 | -1.10 | 0.345 | 0.642 | 1.000 | 2813 | tags=67%, list=23%, signal=87% |
| 3914 | GOBP\_EXOCRINE\_SYSTEM\_DEVELOPMENT |  | 34 | -0.36 | -1.10 | 0.339 | 0.642 | 1.000 | 2790 | tags=29%, list=23%, signal=38% |
| 3915 | GOMF\_PHOSPHATIDATE\_PHOSPHATASE\_ACTIVITY |  | 9 | -0.48 | -1.10 | 0.352 | 0.642 | 1.000 | 4467 | tags=56%, list=37%, signal=88% |
| 3916 | GOCC\_AUTOPHAGOSOME |  | 78 | -0.31 | -1.10 | 0.305 | 0.642 | 1.000 | 3967 | tags=37%, list=33%, signal=55% |
| 3917 | GOBP\_REGULATION\_OF\_GLUCOSE\_METABOLIC\_PROCESS |  | 80 | -0.31 | -1.10 | 0.310 | 0.642 | 1.000 | 4276 | tags=40%, list=36%, signal=62% |
| 3918 | GOBP\_POSITIVE\_REGULATION\_OF\_INTERLEUKIN\_6\_PRODUCTION |  | 47 | -0.34 | -1.10 | 0.319 | 0.642 | 1.000 | 2781 | tags=32%, list=23%, signal=41% |
| 3919 | GOBP\_NOTCH\_SIGNALING\_PATHWAY |  | 132 | -0.30 | -1.10 | 0.287 | 0.642 | 1.000 | 4415 | tags=42%, list=37%, signal=66% |
| 3920 | GOBP\_REGULATION\_OF\_ENDODEOXYRIBONUCLEASE\_ACTIVITY |  | 8 | -0.50 | -1.10 | 0.350 | 0.642 | 1.000 | 4649 | tags=63%, list=39%, signal=102% |
| 3921 | GOBP\_SODIUM\_ION\_IMPORT\_ACROSS\_PLASMA\_MEMBRANE |  | 12 | -0.45 | -1.10 | 0.341 | 0.642 | 1.000 | 748 | tags=33%, list=6%, signal=36% |
| 3922 | GOBP\_REGULATION\_OF\_HAIR\_CYCLE |  | 16 | -0.41 | -1.10 | 0.348 | 0.642 | 1.000 | 980 | tags=19%, list=8%, signal=20% |
| 3923 | GOBP\_LONG\_CHAIN\_FATTY\_ACID\_METABOLIC\_PROCESS |  | 66 | -0.32 | -1.10 | 0.315 | 0.642 | 1.000 | 4536 | tags=52%, list=38%, signal=82% |
| 3924 | GOBP\_TELOMERE\_MAINTENANCE\_VIA\_SEMI\_CONSERVATIVE\_REPLICATION |  | 24 | -0.38 | -1.10 | 0.345 | 0.642 | 1.000 | 3722 | tags=42%, list=31%, signal=60% |
| 3925 | GOBP\_POSITIVE\_REGULATION\_OF\_LIPID\_TRANSPORT |  | 58 | -0.33 | -1.10 | 0.318 | 0.642 | 1.000 | 4164 | tags=41%, list=35%, signal=63% |
| 3926 | GOBP\_SPLICEOSOMAL\_TRI\_SNRNP\_COMPLEX\_ASSEMBLY |  | 9 | -0.49 | -1.10 | 0.349 | 0.642 | 1.000 | 3996 | tags=44%, list=33%, signal=67% |
| 3927 | GOBP\_REGULATION\_OF\_INTRACELLULAR\_PROTEIN\_TRANSPORT |  | 187 | -0.29 | -1.10 | 0.261 | 0.642 | 1.000 | 4110 | tags=35%, list=34%, signal=52% |
| 3928 | GOBP\_EPIDERMIS\_DEVELOPMENT |  | 183 | -0.29 | -1.10 | 0.262 | 0.642 | 1.000 | 3657 | tags=36%, list=30%, signal=50% |
| 3929 | GOMF\_TRANSCRIPTION\_COACTIVATOR\_ACTIVITY |  | 193 | -0.29 | -1.10 | 0.252 | 0.642 | 1.000 | 4649 | tags=43%, list=39%, signal=69% |
| 3930 | GOBP\_REGULATORY\_T\_CELL\_DIFFERENTIATION |  | 20 | -0.40 | -1.10 | 0.339 | 0.642 | 1.000 | 2086 | tags=30%, list=17%, signal=36% |
| 3931 | GOBP\_NEGATIVE\_REGULATION\_OF\_RESPONSE\_TO\_ENDOPLASMIC\_RETICULUM\_STRESS |  | 31 | -0.36 | -1.10 | 0.342 | 0.642 | 1.000 | 5036 | tags=55%, list=42%, signal=94% |
| 3932 | GOBP\_MICROVILLUS\_ASSEMBLY |  | 12 | -0.45 | -1.10 | 0.356 | 0.642 | 1.000 | 5082 | tags=75%, list=42%, signal=130% |
| 3933 | GOBP\_REGULATION\_OF\_3\_UTR\_MEDIATED\_MRNA\_STABILIZATION |  | 4 | -0.61 | -1.10 | 0.343 | 0.642 | 1.000 | 75 | tags=25%, list=1%, signal=25% |
| 3934 | GOBP\_GAMETE\_GENERATION |  | 340 | -0.28 | -1.10 | 0.220 | 0.642 | 1.000 | 4907 | tags=49%, list=41%, signal=80% |
| 3935 | GOBP\_REGULATION\_OF\_FIBROBLAST\_GROWTH\_FACTOR\_RECEPTOR\_SIGNALING\_PATHWAY |  | 18 | -0.41 | -1.10 | 0.342 | 0.642 | 1.000 | 3530 | tags=50%, list=29%, signal=71% |
| 3936 | GOBP\_POSITIVE\_REGULATION\_OF\_PROTEIN\_EXIT\_FROM\_ENDOPLASMIC\_RETICULUM |  | 12 | -0.45 | -1.10 | 0.348 | 0.642 | 1.000 | 2924 | tags=42%, list=24%, signal=55% |
| 3937 | GOBP\_REGULATION\_OF\_MITOTIC\_CELL\_CYCLE |  | 444 | -0.28 | -1.10 | 0.181 | 0.643 | 1.000 | 4443 | tags=39%, list=37%, signal=60% |
| 3938 | GOBP\_MULTICELLULAR\_ORGANISM\_AGING |  | 19 | -0.40 | -1.10 | 0.343 | 0.643 | 1.000 | 3483 | tags=47%, list=29%, signal=67% |
| 3939 | GOMF\_EXTRACELLULAR\_MATRIX\_CONSTITUENT\_CONFERRING\_ELASTICITY |  | 7 | -0.52 | -1.10 | 0.355 | 0.643 | 1.000 | 3156 | tags=43%, list=26%, signal=58% |
| 3940 | GOBP\_NEGATIVE\_REGULATION\_OF\_MULTI\_ORGANISM\_PROCESS |  | 8 | -0.50 | -1.10 | 0.352 | 0.643 | 1.000 | 1457 | tags=38%, list=12%, signal=43% |
| 3941 | GOBP\_SA\_NODE\_CELL\_TO\_ATRIAL\_CARDIAC\_MUSCLE\_CELL\_COMMUNICATION |  | 7 | -0.52 | -1.10 | 0.354 | 0.643 | 1.000 | 3295 | tags=57%, list=27%, signal=79% |
| 3942 | GOBP\_PROTEIN\_HOMOTETRAMERIZATION |  | 38 | -0.35 | -1.10 | 0.329 | 0.643 | 1.000 | 1384 | tags=18%, list=12%, signal=21% |
| 3943 | GOBP\_CELLULAR\_RESPONSE\_TO\_ORGANIC\_CYCLIC\_COMPOUND |  | 365 | -0.28 | -1.10 | 0.204 | 0.643 | 1.000 | 3979 | tags=39%, list=33%, signal=56% |
| 3944 | GOCC\_ENDOPLASMIC\_RETICULUM\_GOLGI\_INTERMEDIATE\_COMPARTMENT |  | 101 | -0.31 | -1.10 | 0.300 | 0.643 | 1.000 | 5276 | tags=53%, list=44%, signal=95% |
| 3945 | GOBP\_CRANIAL\_NERVE\_STRUCTURAL\_ORGANIZATION |  | 4 | -0.61 | -1.10 | 0.351 | 0.643 | 1.000 | 642 | tags=25%, list=5%, signal=26% |
| 3946 | GOCC\_CELL\_TRAILING\_EDGE |  | 8 | -0.50 | -1.10 | 0.353 | 0.643 | 1.000 | 1843 | tags=38%, list=15%, signal=44% |
| 3947 | GOBP\_INTESTINAL\_EPITHELIAL\_CELL\_DIFFERENTIATION |  | 18 | -0.41 | -1.10 | 0.346 | 0.643 | 1.000 | 4205 | tags=50%, list=35%, signal=77% |
| 3948 | GOBP\_PROTEIN\_LOCALIZATION\_TO\_EARLY\_ENDOSOME |  | 7 | -0.52 | -1.10 | 0.358 | 0.643 | 1.000 | 2560 | tags=57%, list=21%, signal=73% |
| 3949 | GOCC\_NUCLEAR\_PORE\_OUTER\_RING |  | 9 | -0.48 | -1.10 | 0.351 | 0.643 | 1.000 | 5623 | tags=78%, list=47%, signal=146% |
| 3950 | GOCC\_MITOTIC\_SPINDLE\_MICROTUBULE |  | 8 | -0.50 | -1.10 | 0.350 | 0.643 | 1.000 | 5518 | tags=75%, list=46%, signal=139% |
| 3951 | GOBP\_REGULATION\_OF\_MEMBRANE\_PROTEIN\_ECTODOMAIN\_PROTEOLYSIS |  | 17 | -0.41 | -1.10 | 0.339 | 0.643 | 1.000 | 1913 | tags=24%, list=16%, signal=28% |
| 3952 | GOBP\_STRIATED\_MUSCLE\_CELL\_DEVELOPMENT |  | 57 | -0.33 | -1.10 | 0.315 | 0.643 | 1.000 | 3286 | tags=30%, list=27%, signal=41% |
| 3953 | GOBP\_ACYLGLYCEROL\_ACYL\_CHAIN\_REMODELING |  | 4 | -0.61 | -1.10 | 0.346 | 0.643 | 1.000 | 261 | tags=25%, list=2%, signal=26% |
| 3954 | GOBP\_DETECTION\_OF\_OTHER\_ORGANISM |  | 4 | -0.61 | -1.10 | 0.347 | 0.643 | 1.000 | 3009 | tags=50%, list=25%, signal=67% |
| 3955 | GOBP\_REGULATION\_OF\_PROTEIN\_TYROSINE\_KINASE\_ACTIVITY |  | 65 | -0.32 | -1.10 | 0.314 | 0.643 | 1.000 | 1519 | tags=18%, list=13%, signal=21% |
| 3956 | GOBP\_CAVEOLIN\_MEDIATED\_ENDOCYTOSIS |  | 7 | -0.52 | -1.10 | 0.354 | 0.643 | 1.000 | 3814 | tags=57%, list=32%, signal=84% |
| 3957 | GOMF\_INOSITOL\_1\_3\_4\_5\_TETRAKISPHOSPHATE\_BINDING |  | 3 | -0.66 | -1.10 | 0.343 | 0.644 | 1.000 | 3222 | tags=67%, list=27%, signal=91% |
| 3958 | GOMF\_POTASSIUM\_CHANNEL\_ACTIVITY |  | 59 | -0.32 | -1.10 | 0.318 | 0.644 | 1.000 | 1632 | tags=20%, list=14%, signal=23% |
| 3959 | GOBP\_METAL\_ION\_HOMEOSTASIS |  | 392 | -0.28 | -1.10 | 0.193 | 0.644 | 1.000 | 2938 | tags=29%, list=24%, signal=37% |
| 3960 | GOBP\_LIPID\_TRANSPORT\_ACROSS\_BLOOD\_BRAIN\_BARRIER |  | 5 | -0.57 | -1.10 | 0.359 | 0.644 | 1.000 | 3169 | tags=60%, list=26%, signal=81% |
| 3961 | GOBP\_REGULATION\_OF\_LIPOPROTEIN\_LIPASE\_ACTIVITY |  | 12 | -0.45 | -1.10 | 0.353 | 0.644 | 1.000 | 2747 | tags=33%, list=23%, signal=43% |
| 3962 | GOBP\_CARBOHYDRATE\_HOMEOSTASIS |  | 149 | -0.30 | -1.10 | 0.284 | 0.644 | 1.000 | 4035 | tags=41%, list=34%, signal=61% |
| 3963 | GOCC\_CELL\_BODY\_FIBER |  | 5 | -0.57 | -1.10 | 0.350 | 0.644 | 1.000 | 1420 | tags=40%, list=12%, signal=45% |
| 3964 | GOBP\_ANIMAL\_ORGAN\_FORMATION |  | 41 | -0.35 | -1.10 | 0.336 | 0.644 | 1.000 | 2474 | tags=29%, list=21%, signal=37% |
| 3965 | GOBP\_MOTOR\_BEHAVIOR |  | 8 | -0.50 | -1.10 | 0.348 | 0.645 | 1.000 | 2903 | tags=50%, list=24%, signal=66% |
| 3966 | GOMF\_DYNACTIN\_BINDING |  | 9 | -0.48 | -1.10 | 0.351 | 0.645 | 1.000 | 5153 | tags=78%, list=43%, signal=136% |
| 3967 | GOMF\_HISTONE\_LYSINE\_N\_METHYLTRANSFERASE\_ACTIVITY |  | 31 | -0.36 | -1.10 | 0.338 | 0.645 | 1.000 | 3200 | tags=39%, list=27%, signal=53% |
| 3968 | GOBP\_DOPAMINERGIC\_NEURON\_DIFFERENTIATION |  | 20 | -0.40 | -1.10 | 0.344 | 0.645 | 1.000 | 3565 | tags=40%, list=30%, signal=57% |
| 3969 | GOBP\_POSITIVE\_REGULATION\_OF\_DOPAMINE\_SECRETION |  | 4 | -0.61 | -1.10 | 0.355 | 0.645 | 1.000 | 207 | tags=25%, list=2%, signal=25% |
| 3970 | GOBP\_CELLULAR\_RESPONSE\_TO\_HEAT |  | 91 | -0.31 | -1.10 | 0.314 | 0.646 | 1.000 | 4349 | tags=43%, list=36%, signal=67% |
| 3971 | GOBP\_POLYOL\_METABOLIC\_PROCESS |  | 99 | -0.30 | -1.10 | 0.303 | 0.646 | 1.000 | 4283 | tags=43%, list=36%, signal=67% |
| 3972 | GOBP\_ACID\_SECRETION |  | 22 | -0.39 | -1.10 | 0.347 | 0.646 | 1.000 | 1939 | tags=36%, list=16%, signal=43% |
| 3973 | GOBP\_REGULATION\_OF\_PEPTIDE\_TRANSPORT |  | 403 | -0.28 | -1.10 | 0.199 | 0.646 | 1.000 | 3565 | tags=32%, list=30%, signal=44% |
| 3974 | GOMF\_5\_DEOXYRIBOSE\_5\_PHOSPHATE\_LYASE\_ACTIVITY |  | 5 | -0.58 | -1.10 | 0.357 | 0.646 | 1.000 | 39 | tags=20%, list=0%, signal=20% |
| 3975 | GOBP\_REGULATION\_OF\_SKELETAL\_MUSCLE\_TISSUE\_DEVELOPMENT |  | 15 | -0.43 | -1.10 | 0.357 | 0.645 | 1.000 | 3565 | tags=33%, list=30%, signal=47% |
| 3976 | GOBP\_REGULATION\_OF\_TRANSMEMBRANE\_RECEPTOR\_PROTEIN\_SERINE\_THREONINE\_KINASE\_SIGNALING\_PATHWAY |  | 163 | -0.29 | -1.10 | 0.281 | 0.645 | 1.000 | 3570 | tags=37%, list=30%, signal=52% |
| 3977 | GOBP\_OUTFLOW\_TRACT\_SEPTUM\_MORPHOGENESIS |  | 20 | -0.40 | -1.10 | 0.345 | 0.645 | 1.000 | 2886 | tags=30%, list=24%, signal=39% |
| 3978 | GOBP\_REGULATION\_OF\_IRON\_ION\_TRANSPORT |  | 4 | -0.61 | -1.10 | 0.352 | 0.645 | 1.000 | 989 | tags=25%, list=8%, signal=27% |
| 3979 | GOBP\_REGULATION\_OF\_TOLL\_LIKE\_RECEPTOR\_2\_SIGNALING\_PATHWAY |  | 9 | -0.48 | -1.10 | 0.353 | 0.645 | 1.000 | 2480 | tags=56%, list=21%, signal=70% |
| 3980 | GOBP\_SKELETAL\_MUSCLE\_CELL\_DIFFERENTIATION |  | 39 | -0.35 | -1.10 | 0.337 | 0.645 | 1.000 | 2474 | tags=26%, list=21%, signal=32% |
| 3981 | GOBP\_CELL\_PROLIFERATION\_INVOLVED\_IN\_HEART\_MORPHOGENESIS |  | 11 | -0.45 | -1.10 | 0.358 | 0.646 | 1.000 | 2811 | tags=36%, list=23%, signal=47% |
| 3982 | GOBP\_APOPTOTIC\_SIGNALING\_PATHWAY |  | 400 | -0.28 | -1.10 | 0.201 | 0.646 | 1.000 | 3692 | tags=34%, list=31%, signal=47% |
| 3983 | GOCC\_VESICLE\_LUMEN |  | 214 | -0.29 | -1.10 | 0.263 | 0.646 | 1.000 | 4098 | tags=36%, list=34%, signal=54% |
| 3984 | GOBP\_LUNG\_GROWTH |  | 5 | -0.57 | -1.10 | 0.358 | 0.646 | 1.000 | 3408 | tags=80%, list=28%, signal=112% |
| 3985 | GOCC\_TRANS\_GOLGI\_NETWORK\_TRANSPORT\_VESICLE\_MEMBRANE |  | 9 | -0.49 | -1.10 | 0.359 | 0.646 | 1.000 | 4718 | tags=56%, list=39%, signal=91% |
| 3986 | GOBP\_TRANSITION\_METAL\_ION\_HOMEOSTASIS |  | 88 | -0.31 | -1.10 | 0.317 | 0.646 | 1.000 | 3318 | tags=36%, list=28%, signal=50% |
| 3987 | GOBP\_TRANSPORT\_ALONG\_MICROTUBULE |  | 126 | -0.30 | -1.10 | 0.296 | 0.647 | 1.000 | 3668 | tags=33%, list=31%, signal=46% |
| 3988 | GOMF\_ACTIVE\_ION\_TRANSMEMBRANE\_TRANSPORTER\_ACTIVITY |  | 127 | -0.30 | -1.10 | 0.303 | 0.647 | 1.000 | 3046 | tags=36%, list=25%, signal=48% |
| 3989 | GOBP\_RESPONSE\_TO\_LIGHT\_INTENSITY |  | 3 | -0.66 | -1.10 | 0.343 | 0.647 | 1.000 | 4102 | tags=100%, list=34%, signal=152% |
| 3990 | GOBP\_POSITIVE\_REGULATION\_OF\_MRNA\_3\_END\_PROCESSING |  | 10 | -0.47 | -1.10 | 0.364 | 0.647 | 1.000 | 4569 | tags=50%, list=38%, signal=81% |
| 3991 | GOBP\_REGULATION\_OF\_GLUTAMATE\_RECEPTOR\_SIGNALING\_PATHWAY |  | 6 | -0.54 | -1.10 | 0.365 | 0.647 | 1.000 | 2882 | tags=50%, list=24%, signal=66% |
| 3992 | GOBP\_REGULATION\_OF\_PROTEASOMAL\_UBIQUITIN\_DEPENDENT\_PROTEIN\_CATABOLIC\_PROCESS |  | 99 | -0.30 | -1.10 | 0.313 | 0.647 | 1.000 | 3988 | tags=30%, list=33%, signal=45% |
| 3993 | GOBP\_NEGATIVE\_REGULATION\_OF\_ADENYLATE\_CYCLASE\_ACTIVITY |  | 8 | -0.50 | -1.10 | 0.362 | 0.647 | 1.000 | 4984 | tags=63%, list=41%, signal=107% |
| 3994 | GOBP\_CILIARY\_NEUROTROPHIC\_FACTOR\_MEDIATED\_SIGNALING\_PATHWAY |  | 5 | -0.57 | -1.10 | 0.356 | 0.647 | 1.000 | 4205 | tags=60%, list=35%, signal=92% |
| 3995 | GOCC\_SMOOTH\_ENDOPLASMIC\_RETICULUM\_MEMBRANE |  | 5 | -0.57 | -1.10 | 0.356 | 0.647 | 1.000 | 3141 | tags=40%, list=26%, signal=54% |
| 3996 | GOMF\_SIGNAL\_SEQUENCE\_BINDING |  | 37 | -0.35 | -1.10 | 0.337 | 0.647 | 1.000 | 4866 | tags=51%, list=41%, signal=86% |
| 3997 | GOMF\_HISTONE\_SERINE\_KINASE\_ACTIVITY |  | 4 | -0.61 | -1.10 | 0.356 | 0.647 | 1.000 | 4528 | tags=75%, list=38%, signal=120% |
| 3998 | GOBP\_TRANSCRIPTION\_BY\_RNA\_POLYMERASE\_III |  | 35 | -0.35 | -1.10 | 0.332 | 0.648 | 1.000 | 4704 | tags=46%, list=39%, signal=75% |
| 3999 | GOBP\_RESPONSE\_TO\_HEPATOCYTE\_GROWTH\_FACTOR |  | 16 | -0.41 | -1.10 | 0.354 | 0.647 | 1.000 | 3615 | tags=50%, list=30%, signal=71% |
| 4000 | GOBP\_POSITIVE\_REGULATION\_OF\_PROTEIN\_TYROSINE\_KINASE\_ACTIVITY |  | 42 | -0.34 | -1.10 | 0.341 | 0.649 | 1.000 | 1038 | tags=19%, list=9%, signal=21% |
| 4001 | GOBP\_ENDOPLASMIC\_RETICULUM\_MANNOSE\_TRIMMING |  | 12 | -0.45 | -1.10 | 0.359 | 0.649 | 1.000 | 5615 | tags=75%, list=47%, signal=141% |
| 4002 | GOBP\_REGULATION\_OF\_RESPIRATORY\_BURST |  | 12 | -0.45 | -1.10 | 0.365 | 0.649 | 1.000 | 675 | tags=25%, list=6%, signal=26% |
| 4003 | GOBP\_VACUOLAR\_TRANSPORT |  | 125 | -0.30 | -1.10 | 0.300 | 0.649 | 1.000 | 3227 | tags=30%, list=27%, signal=41% |
| 4004 | GOBP\_NUCLEOSOME\_POSITIONING |  | 5 | -0.57 | -1.10 | 0.365 | 0.649 | 1.000 | 2281 | tags=40%, list=19%, signal=49% |
| 4005 | GOBP\_REGULATION\_OF\_T\_HELPER\_2\_CELL\_DIFFERENTIATION |  | 10 | -0.47 | -1.10 | 0.365 | 0.649 | 1.000 | 1566 | tags=40%, list=13%, signal=46% |
| 4006 | GOBP\_CELLULAR\_COMPONENT\_DISASSEMBLY\_INVOLVED\_IN\_EXECUTION\_PHASE\_OF\_APOPTOSIS |  | 22 | -0.39 | -1.10 | 0.358 | 0.649 | 1.000 | 5684 | tags=68%, list=47%, signal=129% |
| 4007 | GOBP\_STRIATUM\_DEVELOPMENT |  | 13 | -0.43 | -1.10 | 0.356 | 0.650 | 1.000 | 922 | tags=23%, list=8%, signal=25% |
| 4008 | GOCC\_EXTRINSIC\_COMPONENT\_OF\_PLASMA\_MEMBRANE |  | 120 | -0.30 | -1.10 | 0.303 | 0.650 | 1.000 | 2933 | tags=32%, list=24%, signal=41% |
| 4009 | GOBP\_POSITIVE\_REGULATION\_OF\_DIGESTIVE\_SYSTEM\_PROCESS |  | 6 | -0.54 | -1.10 | 0.360 | 0.650 | 1.000 | 2557 | tags=50%, list=21%, signal=63% |
| 4010 | GOMF\_INOSITOL\_TETRAKISPHOSPHATE\_PHOSPHATASE\_ACTIVITY |  | 7 | -0.51 | -1.10 | 0.355 | 0.650 | 1.000 | 567 | tags=29%, list=5%, signal=30% |
| 4011 | GOBP\_ACTION\_POTENTIAL |  | 80 | -0.31 | -1.10 | 0.317 | 0.650 | 1.000 | 4548 | tags=40%, list=38%, signal=64% |
| 4012 | GOBP\_PROTEIN\_DNA\_COVALENT\_CROSS\_LINKING\_REPAIR |  | 5 | -0.57 | -1.10 | 0.351 | 0.650 | 1.000 | 2774 | tags=40%, list=23%, signal=52% |
| 4013 | GOBP\_POSITIVE\_REGULATION\_OF\_ENDOTHELIAL\_CELL\_PROLIFERATION |  | 70 | -0.32 | -1.10 | 0.325 | 0.650 | 1.000 | 3905 | tags=37%, list=33%, signal=55% |
| 4014 | GOBP\_REGULATION\_OF\_GLIAL\_CELL\_APOPTOTIC\_PROCESS |  | 9 | -0.48 | -1.10 | 0.357 | 0.650 | 1.000 | 3609 | tags=44%, list=30%, signal=63% |
| 4015 | GOCC\_GABA\_ERGIC\_SYNAPSE |  | 29 | -0.37 | -1.10 | 0.342 | 0.651 | 1.000 | 5432 | tags=62%, list=45%, signal=113% |
| 4016 | GOMF\_ANNEALING\_HELICASE\_ACTIVITY |  | 5 | -0.57 | -1.09 | 0.353 | 0.651 | 1.000 | 3214 | tags=60%, list=27%, signal=82% |
| 4017 | GOBP\_ENDOPLASMIC\_RETICULUM\_UNFOLDED\_PROTEIN\_RESPONSE |  | 103 | -0.30 | -1.09 | 0.308 | 0.651 | 1.000 | 4946 | tags=48%, list=41%, signal=80% |
| 4018 | GOBP\_GLUCOCORTICOID\_METABOLIC\_PROCESS |  | 12 | -0.45 | -1.09 | 0.357 | 0.651 | 1.000 | 648 | tags=25%, list=5%, signal=26% |
| 4019 | GOMF\_STEROID\_DEHYDROGENASE\_ACTIVITY |  | 16 | -0.41 | -1.09 | 0.360 | 0.652 | 1.000 | 2625 | tags=31%, list=22%, signal=40% |
| 4020 | GOBP\_NEGATIVE\_REGULATION\_OF\_CATECHOLAMINE\_SECRETION |  | 9 | -0.48 | -1.09 | 0.368 | 0.652 | 1.000 | 12 | tags=11%, list=0%, signal=11% |
| 4021 | GOMF\_3\_BETA\_HYDROXY\_DELTA5\_STEROID\_DEHYDROGENASE\_ACTIVITY |  | 3 | -0.66 | -1.09 | 0.356 | 0.651 | 1.000 | 2273 | tags=67%, list=19%, signal=82% |
| 4022 | GOBP\_NEUTROPHIL\_MEDIATED\_CYTOTOXICITY |  | 5 | -0.57 | -1.09 | 0.357 | 0.651 | 1.000 | 5067 | tags=80%, list=42%, signal=138% |
| 4023 | GOMF\_SIALIC\_ACID\_BINDING |  | 5 | -0.57 | -1.09 | 0.355 | 0.652 | 1.000 | 3855 | tags=60%, list=32%, signal=88% |
| 4024 | GOCC\_SECRETORY\_VESICLE |  | 609 | -0.28 | -1.09 | 0.169 | 0.653 | 1.000 | 3510 | tags=31%, list=29%, signal=41% |
| 4025 | GOBP\_REGULATION\_OF\_CATION\_TRANSMEMBRANE\_TRANSPORT |  | 199 | -0.29 | -1.09 | 0.277 | 0.653 | 1.000 | 3909 | tags=35%, list=33%, signal=51% |
| 4026 | GOBP\_COPII\_COATED\_VESICLE\_BUDDING |  | 60 | -0.32 | -1.09 | 0.333 | 0.653 | 1.000 | 2992 | tags=28%, list=25%, signal=38% |
| 4027 | GOBP\_TACHYKININ\_RECEPTOR\_SIGNALING\_PATHWAY |  | 5 | -0.57 | -1.09 | 0.362 | 0.653 | 1.000 | 4402 | tags=80%, list=37%, signal=126% |
| 4028 | GOBP\_POSITIVE\_REGULATION\_OF\_CELL\_PROLIFERATION\_IN\_BONE\_MARROW |  | 4 | -0.60 | -1.09 | 0.367 | 0.654 | 1.000 | 2168 | tags=50%, list=18%, signal=61% |
| 4029 | GOBP\_LIPID\_HOMEOSTASIS |  | 92 | -0.30 | -1.09 | 0.317 | 0.654 | 1.000 | 3526 | tags=37%, list=29%, signal=52% |
| 4030 | GOBP\_INNER\_EAR\_RECEPTOR\_CELL\_STEREOCILIUM\_ORGANIZATION |  | 17 | -0.41 | -1.09 | 0.344 | 0.654 | 1.000 | 5366 | tags=59%, list=45%, signal=106% |
| 4031 | GOBP\_POSITIVE\_REGULATION\_OF\_EXOCYTOSIS |  | 61 | -0.32 | -1.09 | 0.333 | 0.654 | 1.000 | 2599 | tags=28%, list=22%, signal=35% |
| 4032 | GOMF\_NUCLEAR\_HORMONE\_RECEPTOR\_BINDING |  | 113 | -0.30 | -1.09 | 0.317 | 0.654 | 1.000 | 4455 | tags=45%, list=37%, signal=71% |
| 4033 | GOBP\_EMBRYONIC\_PLACENTA\_MORPHOGENESIS |  | 14 | -0.43 | -1.09 | 0.357 | 0.654 | 1.000 | 5693 | tags=71%, list=47%, signal=136% |
| 4034 | GOBP\_CHEMOKINE\_C\_C\_MOTIF\_LIGAND\_5\_PRODUCTION |  | 6 | -0.54 | -1.09 | 0.367 | 0.654 | 1.000 | 2731 | tags=50%, list=23%, signal=65% |
| 4035 | GOBP\_OSTEOCLAST\_FUSION |  | 5 | -0.57 | -1.09 | 0.359 | 0.654 | 1.000 | 2423 | tags=40%, list=20%, signal=50% |
| 4036 | GOCC\_MALE\_PRONUCLEUS |  | 4 | -0.61 | -1.09 | 0.366 | 0.654 | 1.000 | 4361 | tags=75%, list=36%, signal=118% |
| 4037 | GOBP\_PROTEASOMAL\_PROTEIN\_CATABOLIC\_PROCESS |  | 373 | -0.28 | -1.09 | 0.221 | 0.655 | 1.000 | 4036 | tags=33%, list=34%, signal=48% |
| 4038 | GOBP\_PURINE\_NUCLEOSIDE\_TRIPHOSPHATE\_CATABOLIC\_PROCESS |  | 5 | -0.57 | -1.09 | 0.365 | 0.655 | 1.000 | 3094 | tags=60%, list=26%, signal=81% |
| 4039 | GOBP\_REGULATION\_OF\_HISTONE\_H2B\_UBIQUITINATION |  | 4 | -0.60 | -1.09 | 0.349 | 0.655 | 1.000 | 2118 | tags=50%, list=18%, signal=61% |
| 4040 | GOBP\_PROTEIN\_POLY\_ADP\_RIBOSYLATION |  | 5 | -0.57 | -1.09 | 0.367 | 0.655 | 1.000 | 3376 | tags=80%, list=28%, signal=111% |
| 4041 | GOBP\_ENDOCARDIUM\_MORPHOGENESIS |  | 5 | -0.57 | -1.09 | 0.363 | 0.655 | 1.000 | 2212 | tags=40%, list=18%, signal=49% |
| 4042 | GOBP\_MACROMOLECULE\_DEACYLATION |  | 75 | -0.31 | -1.09 | 0.324 | 0.655 | 1.000 | 4609 | tags=48%, list=38%, signal=77% |
| 4043 | GOBP\_RESPONSE\_TO\_PROSTAGLANDIN |  | 22 | -0.38 | -1.09 | 0.358 | 0.655 | 1.000 | 4692 | tags=55%, list=39%, signal=89% |
| 4044 | GOBP\_ACTIN\_POLYMERIZATION\_DEPENDENT\_CELL\_MOTILITY |  | 7 | -0.51 | -1.09 | 0.362 | 0.655 | 1.000 | 2936 | tags=43%, list=24%, signal=57% |
| 4045 | GOBP\_STEROID\_BIOSYNTHETIC\_PROCESS |  | 132 | -0.30 | -1.09 | 0.307 | 0.655 | 1.000 | 4272 | tags=41%, list=36%, signal=63% |
| 4046 | GOBP\_TETRAHYDROBIOPTERIN\_METABOLIC\_PROCESS |  | 7 | -0.51 | -1.09 | 0.368 | 0.655 | 1.000 | 1930 | tags=43%, list=16%, signal=51% |
| 4047 | GOBP\_POSITIVE\_REGULATION\_OF\_INTERLEUKIN\_10\_PRODUCTION |  | 18 | -0.40 | -1.09 | 0.356 | 0.655 | 1.000 | 4649 | tags=56%, list=39%, signal=91% |
| 4048 | GOBP\_DISACCHARIDE\_METABOLIC\_PROCESS |  | 8 | -0.49 | -1.09 | 0.375 | 0.655 | 1.000 | 288 | tags=25%, list=2%, signal=26% |
| 4049 | GOBP\_WATER\_SOLUBLE\_VITAMIN\_BIOSYNTHETIC\_PROCESS |  | 6 | -0.54 | -1.09 | 0.363 | 0.655 | 1.000 | 2518 | tags=50%, list=21%, signal=63% |
| 4050 | GOBP\_RESPONSE\_TO\_BACTERIUM |  | 313 | -0.28 | -1.09 | 0.245 | 0.655 | 1.000 | 3613 | tags=35%, list=30%, signal=49% |
| 4051 | GOMF\_LACTATE\_TRANSMEMBRANE\_TRANSPORTER\_ACTIVITY |  | 5 | -0.57 | -1.09 | 0.363 | 0.655 | 1.000 | 595 | tags=20%, list=5%, signal=21% |
| 4052 | GOBP\_NEGATIVE\_REGULATION\_OF\_PLATELET\_DERIVED\_GROWTH\_FACTOR\_RECEPTOR\_SIGNALING\_PATHWAY |  | 11 | -0.46 | -1.09 | 0.367 | 0.655 | 1.000 | 5429 | tags=55%, list=45%, signal=99% |
| 4053 | GOMF\_SODIUM\_INDEPENDENT\_ORGANIC\_ANION\_TRANSMEMBRANE\_TRANSPORTER\_ACTIVITY |  | 5 | -0.57 | -1.09 | 0.365 | 0.656 | 1.000 | 2185 | tags=40%, list=18%, signal=49% |
| 4054 | GOMF\_BILE\_ACID\_TRANSMEMBRANE\_TRANSPORTER\_ACTIVITY |  | 7 | -0.51 | -1.09 | 0.367 | 0.656 | 1.000 | 924 | tags=29%, list=8%, signal=31% |
| 4055 | GOBP\_ANATOMICAL\_STRUCTURE\_MATURATION |  | 137 | -0.29 | -1.09 | 0.308 | 0.656 | 1.000 | 2411 | tags=26%, list=20%, signal=33% |
| 4056 | GOBP\_MATERNAL\_PLACENTA\_DEVELOPMENT |  | 18 | -0.40 | -1.09 | 0.360 | 0.656 | 1.000 | 2732 | tags=39%, list=23%, signal=50% |
| 4057 | GOBP\_CHAPERONE\_MEDIATED\_AUTOPHAGY |  | 13 | -0.44 | -1.09 | 0.361 | 0.656 | 1.000 | 5757 | tags=62%, list=48%, signal=118% |
| 4058 | GOBP\_DNA\_STRAND\_RENATURATION |  | 5 | -0.57 | -1.09 | 0.363 | 0.656 | 1.000 | 3214 | tags=60%, list=27%, signal=82% |
| 4059 | GOBP\_REGULATION\_OF\_CD8\_POSITIVE\_ALPHA\_BETA\_T\_CELL\_DIFFERENTIATION |  | 5 | -0.57 | -1.09 | 0.368 | 0.656 | 1.000 | 1601 | tags=40%, list=13%, signal=46% |
| 4060 | GOMF\_METAL\_ION\_TRANSMEMBRANE\_TRANSPORTER\_ACTIVITY |  | 244 | -0.28 | -1.09 | 0.265 | 0.656 | 1.000 | 1668 | tags=20%, list=14%, signal=23% |
| 4061 | GOBP\_SMOOTH\_MUSCLE\_CELL\_PROLIFERATION |  | 102 | -0.30 | -1.09 | 0.320 | 0.656 | 1.000 | 3716 | tags=40%, list=31%, signal=58% |
| 4062 | GOBP\_NEGATIVE\_REGULATION\_OF\_TROPHOBLAST\_CELL\_MIGRATION |  | 3 | -0.65 | -1.09 | 0.354 | 0.657 | 1.000 | 2313 | tags=33%, list=19%, signal=41% |
| 4063 | GOBP\_HIPPOCAMPUS\_DEVELOPMENT |  | 46 | -0.34 | -1.09 | 0.345 | 0.657 | 1.000 | 624 | tags=13%, list=5%, signal=14% |
| 4064 | GOBP\_AUDITORY\_RECEPTOR\_CELL\_DEVELOPMENT |  | 11 | -0.45 | -1.09 | 0.370 | 0.657 | 1.000 | 4949 | tags=73%, list=41%, signal=124% |
| 4065 | GOBP\_NEGATIVE\_REGULATION\_OF\_CENTRIOLE\_REPLICATION |  | 6 | -0.54 | -1.09 | 0.367 | 0.657 | 1.000 | 3718 | tags=67%, list=31%, signal=97% |
| 4066 | GOBP\_POSITIVE\_REGULATION\_OF\_CARDIOCYTE\_DIFFERENTIATION |  | 10 | -0.46 | -1.09 | 0.374 | 0.658 | 1.000 | 2474 | tags=30%, list=21%, signal=38% |
| 4067 | GOBP\_ENDOTHELIAL\_CELL\_FATE\_COMMITMENT |  | 8 | -0.50 | -1.09 | 0.376 | 0.658 | 1.000 | 2560 | tags=50%, list=21%, signal=64% |
| 4068 | GOCC\_NUCLEAR\_PROTEIN\_CONTAINING\_COMPLEX |  | 902 | -0.27 | -1.09 | 0.139 | 0.658 | 1.000 | 5069 | tags=44%, list=42%, signal=70% |
| 4069 | GOMF\_RNA\_POLYMERASE\_III\_TRANSCRIPTION\_REGULATORY\_REGION\_SEQUENCE\_SPECIFIC\_DNA\_BINDING |  | 6 | -0.53 | -1.09 | 0.364 | 0.658 | 1.000 | 1743 | tags=33%, list=15%, signal=39% |
| 4070 | GOBP\_REGULATION\_OF\_CILIUM\_BEAT\_FREQUENCY\_INVOLVED\_IN\_CILIARY\_MOTILITY |  | 5 | -0.57 | -1.09 | 0.364 | 0.658 | 1.000 | 1069 | tags=20%, list=9%, signal=22% |
| 4071 | GOBP\_ENGULFMENT\_OF\_APOPTOTIC\_CELL |  | 13 | -0.43 | -1.09 | 0.362 | 0.658 | 1.000 | 1116 | tags=23%, list=9%, signal=25% |
| 4072 | GOBP\_NEGATIVE\_REGULATION\_OF\_STRESS\_ACTIVATED\_PROTEIN\_KINASE\_SIGNALING\_CASCADE |  | 32 | -0.36 | -1.09 | 0.344 | 0.658 | 1.000 | 2894 | tags=31%, list=24%, signal=41% |
| 4073 | GOBP\_NEGATIVE\_REGULATION\_OF\_NEUROINFLAMMATORY\_RESPONSE |  | 11 | -0.45 | -1.09 | 0.366 | 0.658 | 1.000 | 1519 | tags=27%, list=13%, signal=31% |
| 4074 | GOBP\_NEGATIVE\_REGULATION\_OF\_CHROMATIN\_SILENCING |  | 4 | -0.61 | -1.09 | 0.366 | 0.658 | 1.000 | 1487 | tags=50%, list=12%, signal=57% |
| 4075 | GOBP\_NEGATIVE\_REGULATION\_OF\_MONOCYTE\_DIFFERENTIATION |  | 5 | -0.57 | -1.09 | 0.362 | 0.658 | 1.000 | 5061 | tags=80%, list=42%, signal=138% |
| 4076 | GOBP\_REGULATION\_OF\_RESPONSE\_TO\_REACTIVE\_OXYGEN\_SPECIES |  | 24 | -0.38 | -1.09 | 0.361 | 0.659 | 1.000 | 3650 | tags=33%, list=30%, signal=48% |
| 4077 | GOCC\_GTPASE\_COMPLEX |  | 20 | -0.39 | -1.09 | 0.354 | 0.659 | 1.000 | 4700 | tags=55%, list=39%, signal=90% |
| 4078 | GOCC\_HISTONE\_PRE\_MRNA\_3\_END\_PROCESSING\_COMPLEX |  | 5 | -0.56 | -1.09 | 0.375 | 0.659 | 1.000 | 427 | tags=20%, list=4%, signal=21% |
| 4079 | GOBP\_BLASTOCYST\_DEVELOPMENT |  | 67 | -0.32 | -1.09 | 0.334 | 0.660 | 1.000 | 4506 | tags=37%, list=38%, signal=59% |
| 4080 | GOBP\_CARDIAC\_MUSCLE\_CONTRACTION |  | 80 | -0.31 | -1.09 | 0.332 | 0.660 | 1.000 | 1890 | tags=20%, list=16%, signal=24% |
| 4081 | GOCC\_TETRASPANIN\_ENRICHED\_MICRODOMAIN |  | 8 | -0.49 | -1.09 | 0.366 | 0.660 | 1.000 | 4655 | tags=63%, list=39%, signal=102% |
| 4082 | GOMF\_ION\_CHANNEL\_BINDING |  | 93 | -0.31 | -1.09 | 0.327 | 0.660 | 1.000 | 4663 | tags=44%, list=39%, signal=72% |
| 4083 | GOBP\_POSITIVE\_REGULATION\_OF\_KERATINOCYTE\_DIFFERENTIATION |  | 8 | -0.49 | -1.09 | 0.369 | 0.660 | 1.000 | 634 | tags=25%, list=5%, signal=26% |
| 4084 | GOBP\_REGULATION\_OF\_NERVOUS\_SYSTEM\_PROCESS |  | 73 | -0.31 | -1.09 | 0.336 | 0.660 | 1.000 | 3483 | tags=34%, list=29%, signal=48% |
| 4085 | GOBP\_REGULATION\_OF\_CD40\_SIGNALING\_PATHWAY |  | 4 | -0.60 | -1.09 | 0.371 | 0.660 | 1.000 | 4576 | tags=75%, list=38%, signal=121% |
| 4086 | GOBP\_REGULATION\_OF\_MITOTIC\_CYTOKINESIS |  | 3 | -0.65 | -1.09 | 0.372 | 0.660 | 1.000 | 2962 | tags=67%, list=25%, signal=88% |
| 4087 | GOBP\_CATION\_TRANSPORT |  | 675 | -0.27 | -1.09 | 0.176 | 0.660 | 1.000 | 3046 | tags=29%, list=25%, signal=37% |
| 4088 | GOMF\_PHOSPHATIDYLINOSITOL\_3\_4\_BISPHOSPHATE\_BINDING |  | 22 | -0.38 | -1.09 | 0.364 | 0.660 | 1.000 | 494 | tags=14%, list=4%, signal=14% |
| 4089 | GOBP\_PLANAR\_CELL\_POLARITY\_PATHWAY\_INVOLVED\_IN\_NEURAL\_TUBE\_CLOSURE |  | 8 | -0.49 | -1.09 | 0.371 | 0.660 | 1.000 | 1579 | tags=25%, list=13%, signal=29% |
| 4090 | GOCC\_FILOPODIUM\_TIP |  | 11 | -0.45 | -1.09 | 0.383 | 0.660 | 1.000 | 3797 | tags=55%, list=32%, signal=80% |
| 4091 | GOBP\_ELASTIC\_FIBER\_ASSEMBLY |  | 4 | -0.60 | -1.09 | 0.370 | 0.660 | 1.000 | 2706 | tags=50%, list=23%, signal=65% |
| 4092 | GOBP\_CELL\_DIFFERENTIATION\_INVOLVED\_IN\_KIDNEY\_DEVELOPMENT |  | 43 | -0.34 | -1.09 | 0.352 | 0.660 | 1.000 | 4271 | tags=47%, list=36%, signal=72% |
| 4093 | GOBP\_HEPATOCYTE\_APOPTOTIC\_PROCESS |  | 12 | -0.44 | -1.09 | 0.368 | 0.660 | 1.000 | 3836 | tags=58%, list=32%, signal=86% |
| 4094 | GOBP\_NEGATIVE\_REGULATION\_OF\_PERK\_MEDIATED\_UNFOLDED\_PROTEIN\_RESPONSE |  | 3 | -0.65 | -1.09 | 0.362 | 0.660 | 1.000 | 3691 | tags=67%, list=31%, signal=96% |
| 4095 | GOBP\_REGULATION\_OF\_CARDIOCYTE\_DIFFERENTIATION |  | 19 | -0.40 | -1.09 | 0.363 | 0.660 | 1.000 | 2474 | tags=32%, list=21%, signal=40% |
| 4096 | GOBP\_STEROID\_HORMONE\_SECRETION |  | 13 | -0.44 | -1.09 | 0.369 | 0.661 | 1.000 | 5208 | tags=54%, list=43%, signal=95% |
| 4097 | GOBP\_HOMOLOGOUS\_CHROMOSOME\_SEGREGATION |  | 29 | -0.36 | -1.09 | 0.352 | 0.661 | 1.000 | 5866 | tags=62%, list=49%, signal=121% |
| 4098 | GOBP\_REGULATION\_OF\_MIRNA\_METABOLIC\_PROCESS |  | 8 | -0.49 | -1.09 | 0.375 | 0.661 | 1.000 | 2666 | tags=50%, list=22%, signal=64% |
| 4099 | GOBP\_REGULATION\_OF\_HEART\_RATE |  | 60 | -0.32 | -1.09 | 0.340 | 0.661 | 1.000 | 4164 | tags=37%, list=35%, signal=56% |
| 4100 | GOBP\_METANEPHRIC\_GLOMERULAR\_MESANGIUM\_DEVELOPMENT |  | 4 | -0.61 | -1.09 | 0.369 | 0.661 | 1.000 | 3286 | tags=50%, list=27%, signal=69% |
| 4101 | GOBP\_NEGATIVE\_REGULATION\_OF\_T\_HELPER\_17\_TYPE\_IMMUNE\_RESPONSE |  | 6 | -0.53 | -1.09 | 0.359 | 0.661 | 1.000 | 1951 | tags=50%, list=16%, signal=60% |
| 4102 | GOBP\_ORGANIC\_HYDROXY\_COMPOUND\_BIOSYNTHETIC\_PROCESS |  | 174 | -0.29 | -1.09 | 0.303 | 0.661 | 1.000 | 4283 | tags=41%, list=36%, signal=63% |
| 4103 | GOBP\_CARBOHYDRATE\_METABOLIC\_PROCESS |  | 440 | -0.28 | -1.09 | 0.227 | 0.661 | 1.000 | 4302 | tags=43%, list=36%, signal=64% |
| 4104 | GOBP\_CELLULAR\_RESPONSE\_TO\_VITAMIN |  | 21 | -0.39 | -1.09 | 0.366 | 0.661 | 1.000 | 1579 | tags=29%, list=13%, signal=33% |
| 4105 | GOBP\_MRNA\_CLEAVAGE\_INVOLVED\_IN\_MRNA\_PROCESSING |  | 10 | -0.47 | -1.09 | 0.376 | 0.661 | 1.000 | 4261 | tags=40%, list=35%, signal=62% |
| 4106 | GOBP\_PROTEIN\_LOCALIZATION\_TO\_AXON |  | 5 | -0.56 | -1.09 | 0.374 | 0.662 | 1.000 | 5232 | tags=100%, list=44%, signal=177% |
| 4107 | GOBP\_POSITIVE\_REGULATION\_BY\_SYMBIONT\_OF\_ENTRY\_INTO\_HOST |  | 4 | -0.60 | -1.09 | 0.369 | 0.662 | 1.000 | 1779 | tags=50%, list=15%, signal=59% |
| 4108 | GOBP\_REGULATION\_OF\_VASCULATURE\_DEVELOPMENT |  | 184 | -0.29 | -1.09 | 0.301 | 0.663 | 1.000 | 3905 | tags=39%, list=33%, signal=56% |
| 4109 | GOBP\_DISTAL\_TUBULE\_DEVELOPMENT |  | 6 | -0.53 | -1.09 | 0.369 | 0.663 | 1.000 | 2150 | tags=33%, list=18%, signal=41% |
| 4110 | GOBP\_SPLICEOSOMAL\_COMPLEX\_ASSEMBLY |  | 41 | -0.34 | -1.09 | 0.350 | 0.664 | 1.000 | 5146 | tags=46%, list=43%, signal=81% |
| 4111 | GOBP\_MEIOTIC\_SPINDLE\_ORGANIZATION |  | 6 | -0.53 | -1.08 | 0.381 | 0.664 | 1.000 | 1843 | tags=33%, list=15%, signal=39% |
| 4112 | GOBP\_NEGATIVE\_REGULATION\_OF\_INTERLEUKIN\_1\_PRODUCTION |  | 17 | -0.40 | -1.08 | 0.357 | 0.664 | 1.000 | 2871 | tags=35%, list=24%, signal=46% |
| 4113 | GOBP\_POSITIVE\_REGULATION\_OF\_ENDOTHELIAL\_CELL\_MIGRATION |  | 83 | -0.31 | -1.08 | 0.336 | 0.664 | 1.000 | 3968 | tags=35%, list=33%, signal=52% |
| 4114 | GOBP\_PANCREATIC\_JUICE\_SECRETION |  | 7 | -0.51 | -1.08 | 0.375 | 0.665 | 1.000 | 3553 | tags=57%, list=30%, signal=81% |
| 4115 | GOBP\_ESTABLISHMENT\_OF\_TISSUE\_POLARITY |  | 89 | -0.31 | -1.08 | 0.332 | 0.665 | 1.000 | 2174 | tags=17%, list=18%, signal=20% |
| 4116 | GOBP\_INTRACELLULAR\_ESTROGEN\_RECEPTOR\_SIGNALING\_PATHWAY |  | 41 | -0.34 | -1.08 | 0.359 | 0.665 | 1.000 | 3782 | tags=41%, list=31%, signal=60% |
| 4117 | GOBP\_POSITIVE\_REGULATION\_OF\_CELL\_GROWTH |  | 108 | -0.30 | -1.08 | 0.329 | 0.665 | 1.000 | 4180 | tags=39%, list=35%, signal=59% |
| 4118 | GOBP\_POSITIVE\_REGULATION\_OF\_HISTONE\_UBIQUITINATION |  | 4 | -0.60 | -1.08 | 0.372 | 0.665 | 1.000 | 2118 | tags=50%, list=18%, signal=61% |
| 4119 | GOBP\_BODY\_MORPHOGENESIS |  | 34 | -0.35 | -1.08 | 0.361 | 0.665 | 1.000 | 5089 | tags=62%, list=42%, signal=107% |
| 4120 | GOBP\_AORTA\_DEVELOPMENT |  | 42 | -0.34 | -1.08 | 0.357 | 0.665 | 1.000 | 5111 | tags=48%, list=43%, signal=83% |
| 4121 | GOBP\_REGULATION\_OF\_MEMBRANE\_POTENTIAL |  | 231 | -0.28 | -1.08 | 0.286 | 0.665 | 1.000 | 3918 | tags=33%, list=33%, signal=48% |
| 4122 | GOCC\_CYTOSKELETON\_OF\_PRESYNAPTIC\_ACTIVE\_ZONE |  | 5 | -0.56 | -1.08 | 0.379 | 0.665 | 1.000 | 1360 | tags=40%, list=11%, signal=45% |
| 4123 | GOMF\_NUCLEOTIDE\_DIPHOSPHATASE\_ACTIVITY |  | 12 | -0.44 | -1.08 | 0.373 | 0.665 | 1.000 | 3094 | tags=42%, list=26%, signal=56% |
| 4124 | GOBP\_NON\_RECOMBINATIONAL\_REPAIR |  | 70 | -0.31 | -1.08 | 0.344 | 0.666 | 1.000 | 4773 | tags=49%, list=40%, signal=80% |
| 4125 | GOBP\_REGULATION\_OF\_XENOPHAGY |  | 6 | -0.53 | -1.08 | 0.379 | 0.666 | 1.000 | 5272 | tags=67%, list=44%, signal=119% |
| 4126 | GOBP\_POSITIVE\_REGULATION\_OF\_DNA\_TEMPLATED\_TRANSCRIPTION\_ELONGATION |  | 21 | -0.39 | -1.08 | 0.374 | 0.666 | 1.000 | 3084 | tags=33%, list=26%, signal=45% |
| 4127 | GOMF\_EXTRACELLULAR\_MATRIX\_PROTEIN\_BINDING |  | 3 | -0.65 | -1.08 | 0.370 | 0.666 | 1.000 | 2448 | tags=67%, list=20%, signal=84% |
| 4128 | GOBP\_PROTECTION\_FROM\_NON\_HOMOLOGOUS\_END\_JOINING\_AT\_TELOMERE |  | 7 | -0.51 | -1.08 | 0.377 | 0.666 | 1.000 | 5558 | tags=86%, list=46%, signal=159% |
| 4129 | GOBP\_POSITIVE\_REGULATION\_OF\_PRODUCTION\_OF\_MOLECULAR\_MEDIATOR\_OF\_IMMUNE\_RESPONSE |  | 56 | -0.32 | -1.08 | 0.351 | 0.666 | 1.000 | 3487 | tags=43%, list=29%, signal=60% |
| 4130 | GOMF\_POLY\_A\_BINDING |  | 18 | -0.40 | -1.08 | 0.376 | 0.667 | 1.000 | 6318 | tags=72%, list=53%, signal=152% |
| 4131 | GOBP\_NEGATIVE\_REGULATION\_OF\_T\_CELL\_CYTOKINE\_PRODUCTION |  | 3 | -0.65 | -1.08 | 0.372 | 0.666 | 1.000 | 1441 | tags=67%, list=12%, signal=76% |
| 4132 | GOBP\_BONE\_REMODELING |  | 65 | -0.32 | -1.08 | 0.349 | 0.666 | 1.000 | 2457 | tags=31%, list=20%, signal=38% |
| 4133 | GOBP\_NECROPTOTIC\_PROCESS |  | 28 | -0.36 | -1.08 | 0.366 | 0.667 | 1.000 | 1230 | tags=21%, list=10%, signal=24% |
| 4134 | GOBP\_OSTEOCLAST\_PROLIFERATION |  | 6 | -0.53 | -1.08 | 0.385 | 0.667 | 1.000 | 3553 | tags=50%, list=30%, signal=71% |
| 4135 | GOBP\_CELLULAR\_COMPONENT\_ASSEMBLY\_INVOLVED\_IN\_MORPHOGENESIS |  | 61 | -0.32 | -1.08 | 0.343 | 0.667 | 1.000 | 2548 | tags=25%, list=21%, signal=31% |
| 4136 | GOMF\_PRE\_MIRNA\_BINDING |  | 4 | -0.60 | -1.08 | 0.372 | 0.668 | 1.000 | 4653 | tags=75%, list=39%, signal=122% |
| 4137 | GOBP\_POSITIVE\_REGULATION\_OF\_NUCLEAR\_CELL\_CYCLE\_DNA\_REPLICATION |  | 5 | -0.56 | -1.08 | 0.373 | 0.668 | 1.000 | 5292 | tags=100%, list=44%, signal=179% |
| 4138 | GOBP\_SPINAL\_CORD\_DEVELOPMENT |  | 59 | -0.32 | -1.08 | 0.351 | 0.668 | 1.000 | 1881 | tags=20%, list=16%, signal=24% |
| 4139 | GOBP\_NEGATIVE\_REGULATION\_OF\_MITOCHONDRIAL\_MEMBRANE\_PERMEABILITY |  | 4 | -0.60 | -1.08 | 0.379 | 0.669 | 1.000 | 3843 | tags=75%, list=32%, signal=110% |
| 4140 | GOBP\_RESPONSE\_TO\_ABIOTIC\_STIMULUS |  | 820 | -0.27 | -1.08 | 0.172 | 0.669 | 1.000 | 3930 | tags=35%, list=33%, signal=49% |
| 4141 | GOBP\_POSITIVE\_REGULATION\_OF\_FAT\_CELL\_DIFFERENTIATION |  | 46 | -0.33 | -1.08 | 0.355 | 0.669 | 1.000 | 2009 | tags=26%, list=17%, signal=31% |
| 4142 | GOBP\_SYNCYTIUM\_FORMATION |  | 29 | -0.36 | -1.08 | 0.365 | 0.669 | 1.000 | 2494 | tags=31%, list=21%, signal=39% |
| 4143 | GOCC\_PRESYNAPTIC\_CYTOSKELETON |  | 5 | -0.56 | -1.08 | 0.378 | 0.669 | 1.000 | 1360 | tags=40%, list=11%, signal=45% |
| 4144 | GOBP\_RESPONSE\_TO\_METAL\_ION |  | 227 | -0.28 | -1.08 | 0.293 | 0.669 | 1.000 | 3405 | tags=32%, list=28%, signal=43% |
| 4145 | GOBP\_NEGATIVE\_REGULATION\_OF\_MYOSIN\_LIGHT\_CHAIN\_PHOSPHATASE\_ACTIVITY |  | 4 | -0.60 | -1.08 | 0.376 | 0.669 | 1.000 | 3547 | tags=75%, list=30%, signal=106% |
| 4146 | GOBP\_NEPHRON\_DEVELOPMENT |  | 99 | -0.30 | -1.08 | 0.339 | 0.670 | 1.000 | 4111 | tags=38%, list=34%, signal=58% |
| 4147 | GOBP\_REGULATION\_OF\_DNA\_BIOSYNTHETIC\_PROCESS |  | 85 | -0.30 | -1.08 | 0.345 | 0.669 | 1.000 | 2313 | tags=24%, list=19%, signal=29% |
| 4148 | GOMF\_PEPTIDYL\_PROLINE\_DIOXYGENASE\_ACTIVITY |  | 11 | -0.45 | -1.08 | 0.380 | 0.670 | 1.000 | 3164 | tags=36%, list=26%, signal=49% |
| 4149 | GOBP\_REGULATION\_OF\_EXTRINSIC\_APOPTOTIC\_SIGNALING\_PATHWAY\_IN\_ABSENCE\_OF\_LIGAND |  | 27 | -0.37 | -1.08 | 0.373 | 0.670 | 1.000 | 1146 | tags=19%, list=10%, signal=20% |
| 4150 | GOBP\_DNA\_STRAND\_ELONGATION |  | 24 | -0.38 | -1.08 | 0.370 | 0.670 | 1.000 | 4935 | tags=50%, list=41%, signal=85% |
| 4151 | GOBP\_CIRCULATORY\_SYSTEM\_PROCESS |  | 386 | -0.28 | -1.08 | 0.249 | 0.670 | 1.000 | 2633 | tags=25%, list=22%, signal=31% |
| 4152 | GOBP\_SMOOTHENED\_SIGNALING\_PATHWAY |  | 100 | -0.30 | -1.08 | 0.342 | 0.670 | 1.000 | 4102 | tags=37%, list=34%, signal=56% |
| 4153 | GOBP\_NEGATIVE\_REGULATION\_OF\_CELLULAR\_AMIDE\_METABOLIC\_PROCESS |  | 127 | -0.29 | -1.08 | 0.322 | 0.670 | 1.000 | 2731 | tags=28%, list=23%, signal=36% |
| 4154 | GOBP\_COPI\_COATED\_VESICLE\_BUDDING |  | 5 | -0.56 | -1.08 | 0.384 | 0.670 | 1.000 | 4381 | tags=40%, list=36%, signal=63% |
| 4155 | GOCC\_CYCLIN\_CDK\_POSITIVE\_TRANSCRIPTION\_ELONGATION\_FACTOR\_COMPLEX |  | 8 | -0.49 | -1.08 | 0.381 | 0.670 | 1.000 | 4358 | tags=50%, list=36%, signal=78% |
| 4156 | GOBP\_REGULATION\_OF\_CARDIAC\_MUSCLE\_TISSUE\_DEVELOPMENT |  | 3 | -0.65 | -1.08 | 0.383 | 0.670 | 1.000 | 711 | tags=33%, list=6%, signal=35% |
| 4157 | GOBP\_REGULATION\_OF\_MACROPHAGE\_ACTIVATION |  | 38 | -0.34 | -1.08 | 0.367 | 0.671 | 1.000 | 4469 | tags=55%, list=37%, signal=88% |
| 4158 | GOBP\_SKIN\_MORPHOGENESIS |  | 8 | -0.49 | -1.08 | 0.383 | 0.671 | 1.000 | 6186 | tags=100%, list=52%, signal=206% |
| 4159 | GOBP\_SECRETORY\_GRANULE\_ORGANIZATION |  | 20 | -0.39 | -1.08 | 0.377 | 0.671 | 1.000 | 711 | tags=15%, list=6%, signal=16% |
| 4160 | GOBP\_CHROMATIN\_SILENCING\_AT\_TELOMERE |  | 9 | -0.47 | -1.08 | 0.377 | 0.672 | 1.000 | 4362 | tags=56%, list=36%, signal=87% |
| 4161 | GOBP\_EPITHELIAL\_TO\_MESENCHYMAL\_TRANSITION |  | 111 | -0.30 | -1.08 | 0.341 | 0.672 | 1.000 | 3551 | tags=33%, list=30%, signal=47% |
| 4162 | GOBP\_NEGATIVE\_REGULATION\_OF\_CARTILAGE\_DEVELOPMENT |  | 16 | -0.41 | -1.08 | 0.387 | 0.672 | 1.000 | 1452 | tags=25%, list=12%, signal=28% |
| 4163 | GOBP\_LUTEOLYSIS |  | 6 | -0.53 | -1.08 | 0.380 | 0.673 | 1.000 | 4380 | tags=67%, list=36%, signal=105% |
| 4164 | GOBP\_HEPARAN\_SULFATE\_PROTEOGLYCAN\_BIOSYNTHETIC\_PROCESS |  | 20 | -0.39 | -1.08 | 0.371 | 0.673 | 1.000 | 5140 | tags=75%, list=43%, signal=131% |
| 4165 | GOMF\_LYSINE\_N\_ACETYLTRANSFERASE\_ACTIVITY\_ACTING\_ON\_ACETYL\_PHOSPHATE\_AS\_DONOR |  | 5 | -0.56 | -1.08 | 0.383 | 0.673 | 1.000 | 4184 | tags=80%, list=35%, signal=123% |
| 4166 | GOBP\_VASCULOGENESIS |  | 59 | -0.32 | -1.08 | 0.358 | 0.673 | 1.000 | 3723 | tags=37%, list=31%, signal=54% |
| 4167 | GOBP\_BASIC\_AMINO\_ACID\_TRANSPORT |  | 11 | -0.45 | -1.08 | 0.380 | 0.673 | 1.000 | 2847 | tags=36%, list=24%, signal=48% |
| 4168 | GOMF\_HISTONE\_METHYLTRANSFERASE\_ACTIVITY |  | 34 | -0.35 | -1.08 | 0.370 | 0.673 | 1.000 | 4608 | tags=53%, list=38%, signal=86% |
| 4169 | GOBP\_KERATAN\_SULFATE\_METABOLIC\_PROCESS |  | 24 | -0.37 | -1.08 | 0.375 | 0.673 | 1.000 | 1968 | tags=21%, list=16%, signal=25% |
| 4170 | GOBP\_SMOOTH\_MUSCLE\_CELL\_DIFFERENTIATION |  | 46 | -0.33 | -1.08 | 0.356 | 0.674 | 1.000 | 3565 | tags=35%, list=30%, signal=49% |
| 4171 | GOMF\_MRNA\_3\_UTR\_BINDING |  | 60 | -0.32 | -1.08 | 0.356 | 0.674 | 1.000 | 5550 | tags=52%, list=46%, signal=96% |
| 4172 | GOBP\_DETECTION\_OF\_LIGHT\_STIMULUS |  | 30 | -0.36 | -1.08 | 0.363 | 0.674 | 1.000 | 3909 | tags=43%, list=33%, signal=64% |
| 4173 | GOBP\_CHROMATIN\_ORGANIZATION\_INVOLVED\_IN\_REGULATION\_OF\_TRANSCRIPTION |  | 65 | -0.31 | -1.08 | 0.359 | 0.675 | 1.000 | 3624 | tags=35%, list=30%, signal=50% |
| 4174 | GOBP\_BILE\_ACID\_AND\_BILE\_SALT\_TRANSPORT |  | 16 | -0.41 | -1.08 | 0.379 | 0.675 | 1.000 | 924 | tags=25%, list=8%, signal=27% |
| 4175 | GOMF\_TRANSFERASE\_ACTIVITY\_TRANSFERRING\_AMINO\_ACYL\_GROUPS |  | 9 | -0.47 | -1.08 | 0.384 | 0.675 | 1.000 | 1878 | tags=33%, list=16%, signal=39% |
| 4176 | GOBP\_LUTEINIZATION |  | 7 | -0.51 | -1.08 | 0.387 | 0.675 | 1.000 | 5208 | tags=71%, list=43%, signal=126% |
| 4177 | GOBP\_PRIMARY\_AMINO\_COMPOUND\_BIOSYNTHETIC\_PROCESS |  | 4 | -0.59 | -1.08 | 0.382 | 0.675 | 1.000 | 3653 | tags=50%, list=30%, signal=72% |
| 4178 | GOBP\_NUCLEOLAR\_LARGE\_RRNA\_TRANSCRIPTION\_BY\_RNA\_POLYMERASE\_I |  | 12 | -0.44 | -1.08 | 0.379 | 0.675 | 1.000 | 5712 | tags=58%, list=48%, signal=111% |
| 4179 | GOBP\_RESPONSE\_TO\_LEUCINE |  | 11 | -0.45 | -1.08 | 0.391 | 0.675 | 1.000 | 3375 | tags=45%, list=28%, signal=63% |
| 4180 | GOBP\_OPTIC\_NERVE\_DEVELOPMENT |  | 9 | -0.47 | -1.08 | 0.381 | 0.675 | 1.000 | 1309 | tags=22%, list=11%, signal=25% |
| 4181 | GOBP\_CELLULAR\_RESPONSE\_TO\_X\_RAY |  | 9 | -0.47 | -1.08 | 0.376 | 0.675 | 1.000 | 3927 | tags=67%, list=33%, signal=99% |
| 4182 | GOBP\_CELLULAR\_PROTEIN\_CONTAINING\_COMPLEX\_LOCALIZATION |  | 17 | -0.40 | -1.08 | 0.376 | 0.675 | 1.000 | 5688 | tags=59%, list=47%, signal=112% |
| 4183 | GOCC\_PROTEIN\_DNA\_COMPLEX |  | 89 | -0.30 | -1.08 | 0.346 | 0.675 | 1.000 | 5648 | tags=57%, list=47%, signal=107% |
| 4184 | GOBP\_EXCRETION |  | 35 | -0.35 | -1.08 | 0.372 | 0.675 | 1.000 | 1183 | tags=29%, list=10%, signal=32% |
| 4185 | GOBP\_ORGANIC\_HYDROXY\_COMPOUND\_CATABOLIC\_PROCESS |  | 49 | -0.33 | -1.08 | 0.359 | 0.675 | 1.000 | 4011 | tags=35%, list=33%, signal=52% |
| 4186 | GOBP\_LABYRINTHINE\_LAYER\_DEVELOPMENT |  | 31 | -0.35 | -1.08 | 0.366 | 0.675 | 1.000 | 4107 | tags=42%, list=34%, signal=64% |
| 4187 | GOBP\_REGULATION\_OF\_HORMONE\_BIOSYNTHETIC\_PROCESS |  | 17 | -0.40 | -1.08 | 0.379 | 0.675 | 1.000 | 2666 | tags=47%, list=22%, signal=60% |
| 4188 | GOCC\_MITOTIC\_SPINDLE\_MIDZONE |  | 12 | -0.44 | -1.08 | 0.377 | 0.675 | 1.000 | 1540 | tags=25%, list=13%, signal=29% |
| 4189 | GOMF\_PLATELET\_DERIVED\_GROWTH\_FACTOR\_BINDING |  | 11 | -0.45 | -1.08 | 0.396 | 0.675 | 1.000 | 5324 | tags=64%, list=44%, signal=114% |
| 4190 | GOBP\_FEMALE\_GENITALIA\_DEVELOPMENT |  | 10 | -0.46 | -1.08 | 0.385 | 0.675 | 1.000 | 1903 | tags=30%, list=16%, signal=36% |
| 4191 | GOBP\_LEUKOCYTE\_AGGREGATION |  | 10 | -0.46 | -1.08 | 0.383 | 0.676 | 1.000 | 2408 | tags=40%, list=20%, signal=50% |
| 4192 | GOBP\_NEGATIVE\_REGULATION\_OF\_CALCIUM\_ION\_TRANSPORT |  | 30 | -0.36 | -1.08 | 0.372 | 0.675 | 1.000 | 2291 | tags=23%, list=19%, signal=29% |
| 4193 | GOBP\_APOPTOTIC\_PROCESS\_IN\_BONE\_MARROW\_CELL |  | 3 | -0.65 | -1.08 | 0.381 | 0.675 | 1.000 | 624 | tags=33%, list=5%, signal=35% |
| 4194 | GOBP\_TRANSCRIPTION\_INITIATION\_FROM\_RNA\_POLYMERASE\_III\_PROMOTER |  | 7 | -0.51 | -1.08 | 0.380 | 0.675 | 1.000 | 767 | tags=29%, list=6%, signal=31% |
| 4195 | GOBP\_VESICLE\_TARGETING |  | 74 | -0.31 | -1.08 | 0.352 | 0.676 | 1.000 | 2992 | tags=30%, list=25%, signal=39% |
| 4196 | GOBP\_PHOSPHATIDYLETHANOLAMINE\_ACYL\_CHAIN\_REMODELING |  | 12 | -0.44 | -1.08 | 0.386 | 0.676 | 1.000 | 957 | tags=25%, list=8%, signal=27% |
| 4197 | GOBP\_SELF\_PROTEOLYSIS |  | 5 | -0.56 | -1.08 | 0.379 | 0.676 | 1.000 | 2151 | tags=60%, list=18%, signal=73% |
| 4198 | GOBP\_GLYCOPROTEIN\_BIOSYNTHETIC\_PROCESS |  | 238 | -0.28 | -1.08 | 0.304 | 0.676 | 1.000 | 2811 | tags=26%, list=23%, signal=34% |
| 4199 | GOBP\_OLIGOSACCHARIDE\_METABOLIC\_PROCESS |  | 36 | -0.34 | -1.08 | 0.374 | 0.676 | 1.000 | 4171 | tags=50%, list=35%, signal=76% |
| 4200 | GOBP\_DENDRITE\_ARBORIZATION |  | 5 | -0.56 | -1.08 | 0.384 | 0.676 | 1.000 | 3040 | tags=60%, list=25%, signal=80% |
| 4201 | GOCC\_ANCHORED\_COMPONENT\_OF\_MEMBRANE |  | 78 | -0.31 | -1.08 | 0.353 | 0.676 | 1.000 | 2173 | tags=23%, list=18%, signal=28% |
| 4202 | GOBP\_CELL\_SURFACE\_RECEPTOR\_SIGNALING\_PATHWAY\_INVOLVED\_IN\_CELL\_CELL\_SIGNALING |  | 421 | -0.27 | -1.08 | 0.252 | 0.676 | 1.000 | 3613 | tags=31%, list=30%, signal=43% |
| 4203 | GOBP\_PROTEIN\_PROCESSING |  | 137 | -0.29 | -1.08 | 0.337 | 0.676 | 1.000 | 3565 | tags=34%, list=30%, signal=47% |
| 4204 | GOCC\_ATPASE\_DEPENDENT\_TRANSMEMBRANE\_TRANSPORT\_COMPLEX |  | 10 | -0.46 | -1.08 | 0.389 | 0.676 | 1.000 | 3318 | tags=50%, list=28%, signal=69% |
| 4205 | GOBP\_NEGATIVE\_REGULATION\_OF\_APOPTOTIC\_SIGNALING\_PATHWAY |  | 155 | -0.29 | -1.08 | 0.341 | 0.676 | 1.000 | 3613 | tags=36%, list=30%, signal=51% |
| 4206 | GOBP\_POSITIVE\_REGULATION\_OF\_SECRETION |  | 194 | -0.28 | -1.08 | 0.319 | 0.676 | 1.000 | 3031 | tags=30%, list=25%, signal=39% |
| 4207 | GOBP\_RESPONSE\_TO\_STIMULUS\_INVOLVED\_IN\_REGULATION\_OF\_MUSCLE\_ADAPTATION |  | 8 | -0.49 | -1.08 | 0.393 | 0.676 | 1.000 | 3295 | tags=63%, list=27%, signal=86% |
| 4208 | GOBP\_PEPTIDYL\_LYSINE\_TRIMETHYLATION |  | 31 | -0.35 | -1.08 | 0.369 | 0.676 | 1.000 | 4677 | tags=55%, list=39%, signal=90% |
| 4209 | GOBP\_DETECTION\_OF\_MECHANICAL\_STIMULUS\_INVOLVED\_IN\_SENSORY\_PERCEPTION\_OF\_PAIN |  | 8 | -0.49 | -1.08 | 0.386 | 0.676 | 1.000 | 687 | tags=25%, list=6%, signal=26% |
| 4210 | GOBP\_POSITIVE\_REGULATION\_OF\_STEM\_CELL\_DIFFERENTIATION |  | 11 | -0.45 | -1.07 | 0.391 | 0.676 | 1.000 | 1881 | tags=36%, list=16%, signal=43% |
| 4211 | GOBP\_POSITIVE\_REGULATION\_OF\_B\_CELL\_RECEPTOR\_SIGNALING\_PATHWAY |  | 7 | -0.51 | -1.07 | 0.389 | 0.677 | 1.000 | 4337 | tags=57%, list=36%, signal=89% |
| 4212 | GOMF\_LIPOPOLYSACCHARIDE\_BINDING |  | 18 | -0.40 | -1.07 | 0.375 | 0.677 | 1.000 | 2470 | tags=28%, list=21%, signal=35% |
| 4213 | GOBP\_POSITIVE\_REGULATION\_OF\_T\_CELL\_APOPTOTIC\_PROCESS |  | 7 | -0.51 | -1.07 | 0.382 | 0.677 | 1.000 | 1038 | tags=29%, list=9%, signal=31% |
| 4214 | GOBP\_POSITIVE\_REGULATION\_OF\_TRANSCRIPTION\_OF\_NUCLEOLAR\_LARGE\_RRNA\_BY\_RNA\_POLYMERASE\_I |  | 8 | -0.49 | -1.07 | 0.386 | 0.677 | 1.000 | 4465 | tags=50%, list=37%, signal=80% |
| 4215 | GOBP\_DOPAMINE\_CATABOLIC\_PROCESS |  | 3 | -0.64 | -1.07 | 0.380 | 0.677 | 1.000 | 897 | tags=33%, list=7%, signal=36% |
| 4216 | GOBP\_REGULATION\_OF\_NECROTIC\_CELL\_DEATH |  | 20 | -0.39 | -1.07 | 0.381 | 0.676 | 1.000 | 1084 | tags=20%, list=9%, signal=22% |
| 4217 | GOBP\_CELLULAR\_PROTEIN\_CATABOLIC\_PROCESS |  | 601 | -0.27 | -1.07 | 0.222 | 0.676 | 1.000 | 4036 | tags=34%, list=34%, signal=48% |
| 4218 | GOBP\_POSITIVE\_REGULATION\_OF\_PROTEIN\_ACETYLATION |  | 32 | -0.35 | -1.07 | 0.371 | 0.676 | 1.000 | 3136 | tags=38%, list=26%, signal=51% |
| 4219 | GOBP\_HISTONE\_ARGININE\_METHYLATION |  | 4 | -0.60 | -1.07 | 0.391 | 0.676 | 1.000 | 3647 | tags=75%, list=30%, signal=108% |
| 4220 | GOBP\_POLYOL\_CATABOLIC\_PROCESS |  | 24 | -0.37 | -1.07 | 0.379 | 0.676 | 1.000 | 3934 | tags=42%, list=33%, signal=62% |
| 4221 | GOBP\_POSITIVE\_REGULATION\_OF\_DOUBLE\_STRAND\_BREAK\_REPAIR\_VIA\_HOMOLOGOUS\_RECOMBINATION |  | 11 | -0.45 | -1.07 | 0.386 | 0.677 | 1.000 | 3434 | tags=45%, list=29%, signal=64% |
| 4222 | GOBP\_STEM\_CELL\_DIFFERENTIATION |  | 181 | -0.28 | -1.07 | 0.318 | 0.676 | 1.000 | 2646 | tags=22%, list=22%, signal=27% |
| 4223 | GOBP\_POSITIVE\_REGULATION\_OF\_CELL\_CYCLE\_PROCESS |  | 216 | -0.28 | -1.07 | 0.313 | 0.677 | 1.000 | 4201 | tags=37%, list=35%, signal=56% |
| 4224 | GOMF\_ADENYLATE\_CYCLASE\_BINDING |  | 9 | -0.47 | -1.07 | 0.399 | 0.677 | 1.000 | 4627 | tags=56%, list=39%, signal=90% |
| 4225 | GOBP\_REGULATION\_OF\_RECEPTOR\_MEDIATED\_ENDOCYTOSIS |  | 70 | -0.31 | -1.07 | 0.365 | 0.677 | 1.000 | 2629 | tags=26%, list=22%, signal=33% |
| 4226 | GOBP\_ANTIGEN\_PROCESSING\_AND\_PRESENTATION\_OF\_PEPTIDE\_ANTIGEN |  | 126 | -0.29 | -1.07 | 0.344 | 0.677 | 1.000 | 4343 | tags=39%, list=36%, signal=60% |
| 4227 | GOBP\_CELLULAR\_RESPONSE\_TO\_NITROGEN\_COMPOUND |  | 469 | -0.27 | -1.07 | 0.255 | 0.677 | 1.000 | 3797 | tags=37%, list=32%, signal=52% |
| 4228 | GOBP\_REGULATION\_OF\_HORMONE\_METABOLIC\_PROCESS |  | 24 | -0.37 | -1.07 | 0.378 | 0.678 | 1.000 | 2666 | tags=38%, list=22%, signal=48% |
| 4229 | GOBP\_HORMONE\_CATABOLIC\_PROCESS |  | 8 | -0.49 | -1.07 | 0.384 | 0.678 | 1.000 | 3400 | tags=38%, list=28%, signal=52% |
| 4230 | GOBP\_RESPONSE\_TO\_CYTOKINE |  | 735 | -0.27 | -1.07 | 0.205 | 0.678 | 1.000 | 3217 | tags=30%, list=27%, signal=38% |
| 4231 | GOMF\_MITOGEN\_ACTIVATED\_PROTEIN\_KINASE\_P38\_BINDING |  | 5 | -0.56 | -1.07 | 0.395 | 0.678 | 1.000 | 5111 | tags=80%, list=43%, signal=139% |
| 4232 | GOMF\_RIBOSOMAL\_LARGE\_SUBUNIT\_BINDING |  | 11 | -0.44 | -1.07 | 0.386 | 0.678 | 1.000 | 4060 | tags=45%, list=34%, signal=69% |
| 4233 | GOBP\_REGULATION\_OF\_GENE\_SILENCING\_BY\_RNA |  | 73 | -0.31 | -1.07 | 0.356 | 0.678 | 1.000 | 4080 | tags=41%, list=34%, signal=62% |
| 4234 | GOBP\_SOMATIC\_DIVERSIFICATION\_OF\_IMMUNE\_RECEPTORS\_VIA\_SOMATIC\_MUTATION |  | 12 | -0.44 | -1.07 | 0.398 | 0.678 | 1.000 | 2483 | tags=33%, list=21%, signal=42% |
| 4235 | GOBP\_MEMBRANE\_LIPID\_CATABOLIC\_PROCESS |  | 26 | -0.37 | -1.07 | 0.384 | 0.678 | 1.000 | 5114 | tags=65%, list=43%, signal=114% |
| 4236 | GOBP\_NEGATIVE\_REGULATION\_OF\_CHONDROCYTE\_DIFFERENTIATION |  | 15 | -0.41 | -1.07 | 0.378 | 0.678 | 1.000 | 1452 | tags=27%, list=12%, signal=30% |
| 4237 | GOBP\_SCHWANN\_CELL\_MIGRATION |  | 3 | -0.65 | -1.07 | 0.381 | 0.678 | 1.000 | 3007 | tags=67%, list=25%, signal=89% |
| 4238 | GOBP\_REGULATION\_OF\_B\_CELL\_RECEPTOR\_SIGNALING\_PATHWAY |  | 17 | -0.40 | -1.07 | 0.380 | 0.678 | 1.000 | 4526 | tags=59%, list=38%, signal=94% |
| 4239 | GOBP\_NEGATIVE\_REGULATION\_OF\_T\_CELL\_RECEPTOR\_SIGNALING\_PATHWAY |  | 18 | -0.40 | -1.07 | 0.377 | 0.678 | 1.000 | 2857 | tags=39%, list=24%, signal=51% |
| 4240 | GOBP\_RESPONSE\_TO\_TOPOLOGICALLY\_INCORRECT\_PROTEIN |  | 157 | -0.29 | -1.07 | 0.339 | 0.679 | 1.000 | 4948 | tags=46%, list=41%, signal=77% |
| 4241 | GOBP\_L\_ARGININE\_IMPORT\_ACROSS\_PLASMA\_MEMBRANE |  | 4 | -0.60 | -1.07 | 0.384 | 0.679 | 1.000 | 1025 | tags=25%, list=9%, signal=27% |
| 4242 | GOBP\_PML\_BODY\_ORGANIZATION |  | 4 | -0.59 | -1.07 | 0.395 | 0.679 | 1.000 | 3126 | tags=50%, list=26%, signal=68% |
| 4243 | GOBP\_TEMPERATURE\_HOMEOSTASIS |  | 122 | -0.29 | -1.07 | 0.341 | 0.679 | 1.000 | 2489 | tags=27%, list=21%, signal=34% |
| 4244 | GOCC\_CALCIUM\_CHANNEL\_COMPLEX |  | 36 | -0.34 | -1.07 | 0.369 | 0.679 | 1.000 | 2917 | tags=25%, list=24%, signal=33% |
| 4245 | GOMF\_RNA\_DEPENDENT\_ATPASE\_ACTIVITY |  | 4 | -0.59 | -1.07 | 0.389 | 0.679 | 1.000 | 4465 | tags=75%, list=37%, signal=119% |
| 4246 | GOMF\_PHOSPHATIDYLINOSITOL\_TRISPHOSPHATE\_PHOSPHATASE\_ACTIVITY |  | 6 | -0.53 | -1.07 | 0.390 | 0.679 | 1.000 | 4540 | tags=67%, list=38%, signal=107% |
| 4247 | GOMF\_GROWTH\_FACTOR\_RECEPTOR\_BINDING |  | 86 | -0.30 | -1.07 | 0.358 | 0.680 | 1.000 | 1146 | tags=16%, list=10%, signal=18% |
| 4248 | GOBP\_ANIMAL\_ORGAN\_REGENERATION |  | 55 | -0.32 | -1.07 | 0.366 | 0.680 | 1.000 | 2046 | tags=24%, list=17%, signal=28% |
| 4249 | GOBP\_REGULATION\_OF\_GLIAL\_CELL\_PROLIFERATION |  | 22 | -0.38 | -1.07 | 0.381 | 0.680 | 1.000 | 1743 | tags=27%, list=15%, signal=32% |
| 4250 | GOBP\_AORTIC\_VALVE\_MORPHOGENESIS |  | 24 | -0.37 | -1.07 | 0.372 | 0.680 | 1.000 | 5375 | tags=58%, list=45%, signal=105% |
| 4251 | GOCC\_MRE11\_COMPLEX |  | 6 | -0.52 | -1.07 | 0.387 | 0.680 | 1.000 | 4935 | tags=83%, list=41%, signal=141% |
| 4252 | GOBP\_CELL\_DEATH\_IN\_RESPONSE\_TO\_OXIDATIVE\_STRESS |  | 61 | -0.32 | -1.07 | 0.367 | 0.680 | 1.000 | 3675 | tags=36%, list=31%, signal=52% |
| 4253 | GOBP\_NEGATIVE\_REGULATION\_OF\_EPIDERMAL\_GROWTH\_FACTOR\_ACTIVATED\_RECEPTOR\_ACTIVITY |  | 10 | -0.46 | -1.07 | 0.387 | 0.680 | 1.000 | 2267 | tags=30%, list=19%, signal=37% |
| 4254 | GOBP\_HISTONE\_H3\_K4\_DIMETHYLATION |  | 3 | -0.64 | -1.07 | 0.378 | 0.681 | 1.000 | 2041 | tags=67%, list=17%, signal=80% |
| 4255 | GOMF\_PRIMARY\_MIRNA\_BINDING |  | 7 | -0.50 | -1.07 | 0.393 | 0.681 | 1.000 | 5989 | tags=100%, list=50%, signal=199% |
| 4256 | GOBP\_FLUID\_TRANSPORT |  | 22 | -0.38 | -1.07 | 0.389 | 0.681 | 1.000 | 2421 | tags=36%, list=20%, signal=45% |
| 4257 | GOBP\_PROTEIN\_PALMITOYLATION |  | 22 | -0.38 | -1.07 | 0.381 | 0.681 | 1.000 | 2801 | tags=27%, list=23%, signal=36% |
| 4258 | GOMF\_PEPTIDE\_BINDING |  | 190 | -0.28 | -1.07 | 0.324 | 0.681 | 1.000 | 3716 | tags=33%, list=31%, signal=47% |
| 4259 | GOBP\_DIGESTIVE\_SYSTEM\_PROCESS |  | 54 | -0.32 | -1.07 | 0.370 | 0.681 | 1.000 | 2915 | tags=33%, list=24%, signal=44% |
| 4260 | GOMF\_GLYCOGEN\_BINDING |  | 4 | -0.59 | -1.07 | 0.392 | 0.681 | 1.000 | 3566 | tags=75%, list=30%, signal=107% |
| 4261 | GOMF\_NEUROPEPTIDE\_RECEPTOR\_ACTIVITY |  | 13 | -0.43 | -1.07 | 0.387 | 0.681 | 1.000 | 3544 | tags=54%, list=30%, signal=76% |
| 4262 | GOCC\_GERM\_CELL\_NUCLEUS |  | 10 | -0.46 | -1.07 | 0.392 | 0.681 | 1.000 | 3972 | tags=50%, list=33%, signal=75% |
| 4263 | GOBP\_NEGATIVE\_REGULATION\_OF\_PATHWAY\_RESTRICTED\_SMAD\_PROTEIN\_PHOSPHORYLATION |  | 8 | -0.49 | -1.07 | 0.397 | 0.682 | 1.000 | 5720 | tags=75%, list=48%, signal=143% |
| 4264 | GOCC\_MEMBRANE\_ATTACK\_COMPLEX |  | 3 | -0.64 | -1.07 | 0.389 | 0.682 | 1.000 | 4135 | tags=67%, list=34%, signal=102% |
| 4265 | GOBP\_POSITIVE\_REGULATION\_OF\_TRANSMEMBRANE\_TRANSPORT |  | 135 | -0.29 | -1.07 | 0.341 | 0.682 | 1.000 | 2955 | tags=29%, list=25%, signal=38% |
| 4266 | GOBP\_EPOXIDE\_METABOLIC\_PROCESS |  | 4 | -0.59 | -1.07 | 0.387 | 0.682 | 1.000 | 231 | tags=25%, list=2%, signal=25% |
| 4267 | GOBP\_CARDIAC\_CELL\_FATE\_COMMITMENT |  | 6 | -0.53 | -1.07 | 0.395 | 0.682 | 1.000 | 2212 | tags=33%, list=18%, signal=41% |
| 4268 | GOMF\_PROTEIN\_KINASE\_A\_REGULATORY\_SUBUNIT\_BINDING |  | 19 | -0.39 | -1.07 | 0.382 | 0.682 | 1.000 | 5219 | tags=68%, list=43%, signal=121% |
| 4269 | GOBP\_NEGATIVE\_REGULATION\_OF\_PROTEIN\_KINASE\_B\_SIGNALING |  | 34 | -0.35 | -1.07 | 0.381 | 0.682 | 1.000 | 5063 | tags=59%, list=42%, signal=101% |
| 4270 | GOBP\_TRANSFORMING\_GROWTH\_FACTOR\_BETA3\_PRODUCTION |  | 4 | -0.59 | -1.07 | 0.398 | 0.682 | 1.000 | 2229 | tags=75%, list=19%, signal=92% |
| 4271 | GOBP\_NEGATIVE\_REGULATION\_OF\_INFLAMMATORY\_RESPONSE |  | 77 | -0.30 | -1.07 | 0.364 | 0.682 | 1.000 | 3846 | tags=40%, list=32%, signal=59% |
| 4272 | GOBP\_RETICULOPHAGY |  | 8 | -0.48 | -1.07 | 0.394 | 0.682 | 1.000 | 3132 | tags=50%, list=26%, signal=68% |
| 4273 | GOMF\_PHOSPHOLIPASE\_C\_ACTIVITY |  | 22 | -0.38 | -1.07 | 0.390 | 0.682 | 1.000 | 1982 | tags=32%, list=17%, signal=38% |
| 4274 | GOBP\_NEGATIVE\_REGULATION\_OF\_MUSCLE\_CELL\_APOPTOTIC\_PROCESS |  | 21 | -0.38 | -1.07 | 0.386 | 0.682 | 1.000 | 3968 | tags=43%, list=33%, signal=64% |
| 4275 | GOBP\_STEROID\_METABOLIC\_PROCESS |  | 209 | -0.28 | -1.07 | 0.324 | 0.682 | 1.000 | 4106 | tags=37%, list=34%, signal=55% |
| 4276 | GOBP\_THYMOCYTE\_APOPTOTIC\_PROCESS |  | 12 | -0.43 | -1.07 | 0.398 | 0.682 | 1.000 | 786 | tags=25%, list=7%, signal=27% |
| 4277 | GOBP\_POSITIVE\_REGULATION\_OF\_PROTEIN\_MODIFICATION\_BY\_SMALL\_PROTEIN\_CONJUGATION\_OR\_REMOVAL |  | 107 | -0.29 | -1.07 | 0.357 | 0.683 | 1.000 | 5239 | tags=47%, list=44%, signal=82% |
| 4278 | GOMF\_SYNDECAN\_BINDING |  | 5 | -0.55 | -1.07 | 0.394 | 0.683 | 1.000 | 350 | tags=40%, list=3%, signal=41% |
| 4279 | GOBP\_POSITIVE\_REGULATION\_OF\_SYNAPTIC\_VESICLE\_EXOCYTOSIS |  | 6 | -0.53 | -1.07 | 0.381 | 0.683 | 1.000 | 1668 | tags=33%, list=14%, signal=39% |
| 4280 | GOBP\_GLYCINE\_BIOSYNTHETIC\_PROCESS |  | 3 | -0.64 | -1.07 | 0.387 | 0.683 | 1.000 | 3650 | tags=33%, list=30%, signal=48% |
| 4281 | GOBP\_SERTOLI\_CELL\_DIFFERENTIATION |  | 10 | -0.46 | -1.07 | 0.392 | 0.683 | 1.000 | 3709 | tags=50%, list=31%, signal=72% |
| 4282 | GOBP\_PEPTIDE\_MODIFICATION |  | 3 | -0.64 | -1.07 | 0.395 | 0.683 | 1.000 | 4342 | tags=100%, list=36%, signal=157% |
| 4283 | GOBP\_POSITIVE\_REGULATION\_OF\_INTRACELLULAR\_PROTEIN\_TRANSPORT |  | 134 | -0.29 | -1.07 | 0.346 | 0.683 | 1.000 | 3654 | tags=31%, list=30%, signal=43% |
| 4284 | GOBP\_REGULATION\_OF\_POLYSACCHARIDE\_METABOLIC\_PROCESS |  | 29 | -0.35 | -1.07 | 0.374 | 0.683 | 1.000 | 4649 | tags=55%, list=39%, signal=90% |
| 4285 | GOBP\_CARDIAC\_NEURAL\_CREST\_CELL\_MIGRATION\_INVOLVED\_IN\_OUTFLOW\_TRACT\_MORPHOGENESIS |  | 6 | -0.53 | -1.07 | 0.397 | 0.683 | 1.000 | 480 | tags=17%, list=4%, signal=17% |
| 4286 | GOBP\_REGULATION\_OF\_CARDIAC\_MUSCLE\_CELL\_DIFFERENTIATION |  | 12 | -0.44 | -1.07 | 0.401 | 0.683 | 1.000 | 2474 | tags=33%, list=21%, signal=42% |
| 4287 | GOBP\_REGULATION\_OF\_COMPLEMENT\_ACTIVATION |  | 28 | -0.36 | -1.07 | 0.380 | 0.683 | 1.000 | 4135 | tags=46%, list=34%, signal=71% |
| 4288 | GOBP\_NUCLEOSIDE\_TRIPHOSPHATE\_CATABOLIC\_PROCESS |  | 10 | -0.46 | -1.07 | 0.393 | 0.683 | 1.000 | 3601 | tags=50%, list=30%, signal=71% |
| 4289 | GOCC\_AXOLEMMA |  | 8 | -0.48 | -1.07 | 0.403 | 0.683 | 1.000 | 4122 | tags=63%, list=34%, signal=95% |
| 4290 | GOBP\_REGULATION\_OF\_ENDOTHELIAL\_CELL\_MIGRATION |  | 132 | -0.29 | -1.07 | 0.355 | 0.683 | 1.000 | 3613 | tags=33%, list=30%, signal=47% |
| 4291 | GOBP\_POSITIVE\_REGULATION\_OF\_SEQUESTERING\_OF\_TRIGLYCERIDE |  | 5 | -0.56 | -1.07 | 0.396 | 0.683 | 1.000 | 5281 | tags=80%, list=44%, signal=143% |
| 4292 | GOMF\_EPHRIN\_RECEPTOR\_ACTIVITY |  | 10 | -0.45 | -1.07 | 0.401 | 0.684 | 1.000 | 1309 | tags=20%, list=11%, signal=22% |
| 4293 | GOBP\_EMBRYONIC\_BODY\_MORPHOGENESIS |  | 9 | -0.47 | -1.07 | 0.408 | 0.684 | 1.000 | 5432 | tags=67%, list=45%, signal=122% |
| 4294 | GOBP\_NEGATIVE\_REGULATION\_OF\_CELL\_JUNCTION\_ASSEMBLY |  | 26 | -0.36 | -1.07 | 0.389 | 0.684 | 1.000 | 3575 | tags=42%, list=30%, signal=60% |
| 4295 | GOBP\_SPHINGOMYELIN\_BIOSYNTHETIC\_PROCESS |  | 7 | -0.50 | -1.07 | 0.400 | 0.684 | 1.000 | 3931 | tags=57%, list=33%, signal=85% |
| 4296 | GOMF\_EXTRACELLULAR\_MATRIX\_BINDING |  | 42 | -0.33 | -1.07 | 0.380 | 0.685 | 1.000 | 3196 | tags=31%, list=27%, signal=42% |
| 4297 | GOBP\_NEGATIVE\_REGULATION\_OF\_SMALL\_MOLECULE\_METABOLIC\_PROCESS |  | 62 | -0.31 | -1.07 | 0.368 | 0.685 | 1.000 | 2667 | tags=32%, list=22%, signal=41% |
| 4298 | GOBP\_NEGATIVE\_REGULATION\_OF\_MUSCLE\_CELL\_DIFFERENTIATION |  | 38 | -0.34 | -1.07 | 0.370 | 0.684 | 1.000 | 4139 | tags=50%, list=34%, signal=76% |
| 4299 | GOBP\_REGULATION\_OF\_ENDOTHELIAL\_TUBE\_MORPHOGENESIS |  | 3 | -0.64 | -1.07 | 0.394 | 0.684 | 1.000 | 4337 | tags=100%, list=36%, signal=156% |
| 4300 | GOBP\_POSITIVE\_REGULATION\_OF\_MRNA\_CATABOLIC\_PROCESS |  | 44 | -0.33 | -1.07 | 0.380 | 0.684 | 1.000 | 4667 | tags=57%, list=39%, signal=93% |
| 4301 | GOBP\_REGULATION\_OF\_MUSCLE\_HYPERTROPHY |  | 44 | -0.33 | -1.07 | 0.375 | 0.685 | 1.000 | 3376 | tags=36%, list=28%, signal=50% |
| 4302 | GOBP\_HYPOTONIC\_RESPONSE |  | 8 | -0.49 | -1.07 | 0.407 | 0.685 | 1.000 | 687 | tags=25%, list=6%, signal=26% |
| 4303 | GOBP\_REGULATION\_OF\_ADENYLATE\_CYCLASE\_ACTIVITY |  | 17 | -0.40 | -1.07 | 0.392 | 0.685 | 1.000 | 4984 | tags=47%, list=41%, signal=80% |
| 4304 | GOMF\_OXIDIZED\_DNA\_BINDING |  | 8 | -0.49 | -1.07 | 0.404 | 0.685 | 1.000 | 3214 | tags=50%, list=27%, signal=68% |
| 4305 | GOBP\_NEUTROPHIL\_EXTRAVASATION |  | 10 | -0.46 | -1.07 | 0.401 | 0.685 | 1.000 | 3534 | tags=70%, list=29%, signal=99% |
| 4306 | GOBP\_NEGATIVE\_REGULATION\_OF\_HISTONE\_H3\_K4\_METHYLATION |  | 3 | -0.64 | -1.07 | 0.385 | 0.685 | 1.000 | 1964 | tags=67%, list=16%, signal=80% |
| 4307 | GOBP\_CHOLESTEROL\_STORAGE |  | 14 | -0.42 | -1.07 | 0.391 | 0.685 | 1.000 | 3403 | tags=64%, list=28%, signal=90% |
| 4308 | GOBP\_ESTABLISHMENT\_OF\_PROTEIN\_LOCALIZATION\_TO\_CHROMOSOME |  | 18 | -0.39 | -1.07 | 0.402 | 0.685 | 1.000 | 1667 | tags=17%, list=14%, signal=19% |
| 4309 | GOBP\_SEX\_DIFFERENTIATION |  | 169 | -0.28 | -1.07 | 0.343 | 0.685 | 1.000 | 4692 | tags=49%, list=39%, signal=79% |
| 4310 | GOBP\_ESTABLISHMENT\_OR\_MAINTENANCE\_OF\_CYTOSKELETON\_POLARITY |  | 7 | -0.50 | -1.07 | 0.407 | 0.685 | 1.000 | 985 | tags=29%, list=8%, signal=31% |
| 4311 | GOBP\_PROTEIN\_CATABOLIC\_PROCESS |  | 718 | -0.27 | -1.07 | 0.234 | 0.685 | 1.000 | 4036 | tags=34%, list=34%, signal=48% |
| 4312 | GOCC\_MYOSIN\_II\_COMPLEX |  | 4 | -0.59 | -1.07 | 0.410 | 0.686 | 1.000 | 3948 | tags=50%, list=33%, signal=74% |
| 4313 | GOBP\_NEGATIVE\_REGULATION\_OF\_STRIATED\_MUSCLE\_CELL\_APOPTOTIC\_PROCESS |  | 13 | -0.43 | -1.07 | 0.404 | 0.686 | 1.000 | 5693 | tags=69%, list=47%, signal=131% |
| 4314 | GOMF\_OXIDOREDUCTASE\_ACTIVITY\_ACTING\_ON\_THE\_ALDEHYDE\_OR\_OXO\_GROUP\_OF\_DONORS |  | 31 | -0.35 | -1.07 | 0.384 | 0.686 | 1.000 | 2534 | tags=26%, list=21%, signal=33% |
| 4315 | GOMF\_SUPEROXIDE\_GENERATING\_NAD\_P\_H\_OXIDASE\_ACTIVITY |  | 6 | -0.52 | -1.07 | 0.396 | 0.686 | 1.000 | 36 | tags=17%, list=0%, signal=17% |
| 4316 | GOBP\_METANEPHROS\_DEVELOPMENT |  | 55 | -0.32 | -1.07 | 0.370 | 0.686 | 1.000 | 2374 | tags=27%, list=20%, signal=34% |
| 4317 | GOBP\_VENTRAL\_SPINAL\_CORD\_INTERNEURON\_DIFFERENTIATION |  | 8 | -0.48 | -1.07 | 0.396 | 0.686 | 1.000 | 1871 | tags=38%, list=16%, signal=44% |
| 4318 | GOBP\_PROTEIN\_LIPID\_COMPLEX\_SUBUNIT\_ORGANIZATION |  | 29 | -0.36 | -1.07 | 0.380 | 0.686 | 1.000 | 3217 | tags=38%, list=27%, signal=52% |
| 4319 | GOMF\_14\_3\_3\_PROTEIN\_BINDING |  | 23 | -0.37 | -1.07 | 0.391 | 0.687 | 1.000 | 6383 | tags=74%, list=53%, signal=157% |
| 4320 | GOBP\_POSITIVE\_REGULATION\_OF\_CELLULAR\_RESPONSE\_TO\_INSULIN\_STIMULUS |  | 18 | -0.39 | -1.07 | 0.401 | 0.687 | 1.000 | 3433 | tags=39%, list=29%, signal=54% |
| 4321 | GOBP\_POSITIVE\_REGULATION\_OF\_CELL\_PROLIFERATION\_INVOLVED\_IN\_KIDNEY\_DEVELOPMENT |  | 6 | -0.52 | -1.06 | 0.394 | 0.687 | 1.000 | 2494 | tags=33%, list=21%, signal=42% |
| 4322 | GOBP\_REGULATION\_OF\_GOLGI\_TO\_PLASMA\_MEMBRANE\_PROTEIN\_TRANSPORT |  | 6 | -0.53 | -1.06 | 0.406 | 0.687 | 1.000 | 3426 | tags=50%, list=29%, signal=70% |
| 4323 | GOBP\_HOMOLOGOUS\_CHROMOSOME\_PAIRING\_AT\_MEIOSIS |  | 21 | -0.38 | -1.06 | 0.395 | 0.687 | 1.000 | 5365 | tags=57%, list=45%, signal=103% |
| 4324 | GOBP\_SIGNAL\_TRANSDUCTION\_BY\_P53\_CLASS\_MEDIATOR |  | 206 | -0.28 | -1.06 | 0.340 | 0.687 | 1.000 | 3701 | tags=31%, list=31%, signal=43% |
| 4325 | GOBP\_CELLULAR\_RESPONSE\_TO\_NITROSATIVE\_STRESS |  | 5 | -0.56 | -1.06 | 0.399 | 0.687 | 1.000 | 1667 | tags=40%, list=14%, signal=46% |
| 4326 | GOBP\_TRANSCRIPTION\_PREINITIATION\_COMPLEX\_ASSEMBLY |  | 25 | -0.37 | -1.06 | 0.394 | 0.688 | 1.000 | 1505 | tags=16%, list=13%, signal=18% |
| 4327 | GOBP\_FEMALE\_SEX\_DIFFERENTIATION |  | 72 | -0.31 | -1.06 | 0.369 | 0.688 | 1.000 | 2300 | tags=25%, list=19%, signal=31% |
| 4328 | GOBP\_RESPIRATORY\_GASEOUS\_EXCHANGE\_BY\_RESPIRATORY\_SYSTEM |  | 38 | -0.34 | -1.06 | 0.382 | 0.688 | 1.000 | 3112 | tags=39%, list=26%, signal=53% |
| 4329 | GOMF\_GLUTAMATE\_RECEPTOR\_BINDING |  | 24 | -0.37 | -1.06 | 0.387 | 0.688 | 1.000 | 3909 | tags=46%, list=33%, signal=68% |
| 4330 | GOBP\_SYNAPTONEMAL\_COMPLEX\_ORGANIZATION |  | 11 | -0.44 | -1.06 | 0.398 | 0.688 | 1.000 | 2935 | tags=36%, list=24%, signal=48% |
| 4331 | GOCC\_EGG\_COAT |  | 3 | -0.64 | -1.06 | 0.400 | 0.688 | 1.000 | 287 | tags=33%, list=2%, signal=34% |
| 4332 | GOMF\_LIGASE\_ACTIVITY\_FORMING\_PHOSPHORIC\_ESTER\_BONDS |  | 5 | -0.55 | -1.06 | 0.394 | 0.688 | 1.000 | 4773 | tags=80%, list=40%, signal=133% |
| 4333 | GOBP\_GOLGI\_VESICLE\_TRANSPORT |  | 299 | -0.27 | -1.06 | 0.321 | 0.689 | 1.000 | 4381 | tags=39%, list=36%, signal=60% |
| 4334 | GOBP\_SECRETION |  | 948 | -0.27 | -1.06 | 0.213 | 0.689 | 1.000 | 3571 | tags=32%, list=30%, signal=42% |
| 4335 | GOBP\_INTERLEUKIN\_10\_PRODUCTION |  | 25 | -0.37 | -1.06 | 0.394 | 0.688 | 1.000 | 4649 | tags=52%, list=39%, signal=85% |
| 4336 | GOBP\_CARBOHYDRATE\_TRANSPORT |  | 100 | -0.30 | -1.06 | 0.371 | 0.689 | 1.000 | 2162 | tags=24%, list=18%, signal=29% |
| 4337 | GOBP\_POSITIVE\_REGULATION\_OF\_NUCLEOBASE\_CONTAINING\_COMPOUND\_TRANSPORT |  | 5 | -0.55 | -1.06 | 0.403 | 0.688 | 1.000 | 4278 | tags=60%, list=36%, signal=93% |
| 4338 | GOMF\_HSP70\_PROTEIN\_BINDING |  | 27 | -0.36 | -1.06 | 0.402 | 0.689 | 1.000 | 2127 | tags=22%, list=18%, signal=27% |
| 4339 | GOBP\_LENS\_FIBER\_CELL\_MORPHOGENESIS |  | 4 | -0.59 | -1.06 | 0.411 | 0.689 | 1.000 | 1066 | tags=50%, list=9%, signal=55% |
| 4340 | GOMF\_ASPARTIC\_TYPE\_PEPTIDASE\_ACTIVITY |  | 13 | -0.43 | -1.06 | 0.404 | 0.689 | 1.000 | 1618 | tags=31%, list=13%, signal=36% |
| 4341 | GOBP\_MONOCYTE\_EXTRAVASATION |  | 7 | -0.50 | -1.06 | 0.402 | 0.690 | 1.000 | 1608 | tags=29%, list=13%, signal=33% |
| 4342 | GOBP\_NITRIC\_OXIDE\_MEDIATED\_SIGNAL\_TRANSDUCTION |  | 15 | -0.41 | -1.06 | 0.407 | 0.691 | 1.000 | 1687 | tags=33%, list=14%, signal=39% |
| 4343 | GOBP\_RESPONSE\_TO\_ACTIVITY |  | 48 | -0.32 | -1.06 | 0.381 | 0.690 | 1.000 | 3685 | tags=44%, list=31%, signal=63% |
| 4344 | GOBP\_MITOCHONDRIAL\_CALCIUM\_ION\_TRANSMEMBRANE\_TRANSPORT |  | 21 | -0.38 | -1.06 | 0.394 | 0.690 | 1.000 | 4044 | tags=43%, list=34%, signal=64% |
| 4345 | GOMF\_CCR5\_CHEMOKINE\_RECEPTOR\_BINDING |  | 4 | -0.59 | -1.06 | 0.401 | 0.690 | 1.000 | 1038 | tags=50%, list=9%, signal=55% |
| 4346 | GOBP\_NEGATIVE\_REGULATION\_OF\_INTERFERON\_GAMMA\_PRODUCTION |  | 19 | -0.39 | -1.06 | 0.393 | 0.690 | 1.000 | 3163 | tags=42%, list=26%, signal=57% |
| 4347 | GOBP\_NEGATIVE\_REGULATION\_OF\_EPITHELIAL\_CELL\_DIFFERENTIATION |  | 27 | -0.36 | -1.06 | 0.395 | 0.690 | 1.000 | 1580 | tags=26%, list=13%, signal=30% |
| 4348 | GOMF\_PROTEIN\_MEMBRANE\_ADAPTOR\_ACTIVITY |  | 17 | -0.40 | -1.06 | 0.403 | 0.690 | 1.000 | 3158 | tags=59%, list=26%, signal=80% |
| 4349 | GOBP\_TOLL\_LIKE\_RECEPTOR\_2\_SIGNALING\_PATHWAY |  | 12 | -0.43 | -1.06 | 0.400 | 0.690 | 1.000 | 2480 | tags=42%, list=21%, signal=52% |
| 4350 | GOBP\_REGULATION\_OF\_PROTEIN\_MODIFICATION\_BY\_SMALL\_PROTEIN\_CONJUGATION\_OR\_REMOVAL |  | 185 | -0.28 | -1.06 | 0.347 | 0.691 | 1.000 | 4618 | tags=39%, list=38%, signal=63% |
| 4351 | GOBP\_REGULATION\_OF\_HELICASE\_ACTIVITY |  | 10 | -0.45 | -1.06 | 0.407 | 0.691 | 1.000 | 2756 | tags=50%, list=23%, signal=65% |
| 4352 | GOCC\_NUCLEAR\_CYCLIN\_DEPENDENT\_PROTEIN\_KINASE\_HOLOENZYME\_COMPLEX |  | 12 | -0.43 | -1.06 | 0.412 | 0.691 | 1.000 | 4358 | tags=50%, list=36%, signal=78% |
| 4353 | GOBP\_REGULATION\_OF\_BONE\_DEVELOPMENT |  | 5 | -0.55 | -1.06 | 0.406 | 0.691 | 1.000 | 4963 | tags=80%, list=41%, signal=136% |
| 4354 | GOBP\_POSITIVE\_REGULATION\_OF\_RESPONSE\_TO\_ENDOPLASMIC\_RETICULUM\_STRESS |  | 25 | -0.36 | -1.06 | 0.400 | 0.691 | 1.000 | 3966 | tags=48%, list=33%, signal=72% |
| 4355 | GOBP\_DORSAL\_ROOT\_GANGLION\_DEVELOPMENT |  | 4 | -0.58 | -1.06 | 0.402 | 0.691 | 1.000 | 4139 | tags=75%, list=34%, signal=114% |
| 4356 | GOBP\_EARLY\_ENDOSOME\_TO\_GOLGI\_TRANSPORT |  | 8 | -0.48 | -1.06 | 0.406 | 0.691 | 1.000 | 3227 | tags=38%, list=27%, signal=51% |
| 4357 | GOBP\_POSITIVE\_REGULATION\_OF\_GLUCOSE\_METABOLIC\_PROCESS |  | 26 | -0.36 | -1.06 | 0.393 | 0.692 | 1.000 | 3692 | tags=42%, list=31%, signal=61% |
| 4358 | GOBP\_CELL\_COMMUNICATION\_INVOLVED\_IN\_CARDIAC\_CONDUCTION |  | 33 | -0.34 | -1.06 | 0.390 | 0.692 | 1.000 | 3918 | tags=39%, list=33%, signal=58% |
| 4359 | GOBP\_T\_HELPER\_1\_CELL\_CYTOKINE\_PRODUCTION |  | 5 | -0.55 | -1.06 | 0.412 | 0.692 | 1.000 | 3487 | tags=60%, list=29%, signal=85% |
| 4360 | GOBP\_ACTIVATION\_OF\_CYSTEINE\_TYPE\_ENDOPEPTIDASE\_ACTIVITY\_INVOLVED\_IN\_APOPTOTIC\_PROCESS |  | 64 | -0.31 | -1.06 | 0.382 | 0.692 | 1.000 | 2982 | tags=31%, list=25%, signal=41% |
| 4361 | GOBP\_NEGATIVE\_REGULATION\_OF\_PHOSPHATIDYLINOSITOL\_3\_KINASE\_ACTIVITY |  | 3 | -0.64 | -1.06 | 0.401 | 0.692 | 1.000 | 1637 | tags=33%, list=14%, signal=39% |
| 4362 | GOBP\_REGULATION\_OF\_CELL\_GROWTH\_INVOLVED\_IN\_CARDIAC\_MUSCLE\_CELL\_DEVELOPMENT |  | 12 | -0.43 | -1.06 | 0.405 | 0.692 | 1.000 | 3177 | tags=50%, list=26%, signal=68% |
| 4363 | GOBP\_REGULATION\_OF\_EPITHELIAL\_CELL\_DIFFERENTIATION |  | 86 | -0.30 | -1.06 | 0.374 | 0.692 | 1.000 | 3615 | tags=36%, list=30%, signal=51% |
| 4364 | GOMF\_HEPARAN\_SULFATE\_PROTEOGLYCAN\_BINDING |  | 12 | -0.44 | -1.06 | 0.405 | 0.692 | 1.000 | 3007 | tags=50%, list=25%, signal=67% |
| 4365 | GOBP\_ALCOHOL\_CATABOLIC\_PROCESS |  | 38 | -0.34 | -1.06 | 0.395 | 0.692 | 1.000 | 4011 | tags=34%, list=33%, signal=51% |
| 4366 | GOBP\_LEUKOCYTE\_MEDIATED\_IMMUNITY |  | 462 | -0.27 | -1.06 | 0.295 | 0.692 | 1.000 | 4398 | tags=41%, list=37%, signal=62% |
| 4367 | GOBP\_CHROMATIN\_MEDIATED\_MAINTENANCE\_OF\_TRANSCRIPTION |  | 9 | -0.47 | -1.06 | 0.416 | 0.693 | 1.000 | 3521 | tags=44%, list=29%, signal=63% |
| 4368 | GOMF\_MISMATCH\_REPAIR\_COMPLEX\_BINDING |  | 10 | -0.45 | -1.06 | 0.397 | 0.693 | 1.000 | 5678 | tags=80%, list=47%, signal=152% |
| 4369 | GOBP\_POSITIVE\_REGULATION\_OF\_PROTEIN\_LOCALIZATION\_TO\_CELL\_PERIPHERY |  | 46 | -0.32 | -1.06 | 0.388 | 0.693 | 1.000 | 4803 | tags=48%, list=40%, signal=79% |
| 4370 | GOCC\_PRESYNAPTIC\_ACTIVE\_ZONE |  | 48 | -0.32 | -1.06 | 0.388 | 0.693 | 1.000 | 3125 | tags=35%, list=26%, signal=48% |
| 4371 | GOBP\_PHOTOPERIODISM |  | 20 | -0.38 | -1.06 | 0.406 | 0.694 | 1.000 | 2667 | tags=35%, list=22%, signal=45% |
| 4372 | GOBP\_HISTONE\_H2B\_UBIQUITINATION |  | 11 | -0.44 | -1.06 | 0.397 | 0.694 | 1.000 | 4687 | tags=55%, list=39%, signal=89% |
| 4373 | GOBP\_NEURON\_DEATH\_IN\_RESPONSE\_TO\_OXIDATIVE\_STRESS |  | 18 | -0.39 | -1.06 | 0.405 | 0.694 | 1.000 | 3675 | tags=39%, list=31%, signal=56% |
| 4374 | GOBP\_PRIMARY\_MIRNA\_PROCESSING |  | 8 | -0.48 | -1.06 | 0.409 | 0.694 | 1.000 | 3306 | tags=50%, list=28%, signal=69% |
| 4375 | GOBP\_CARDIAC\_MUSCLE\_CELL\_PROLIFERATION |  | 32 | -0.35 | -1.06 | 0.391 | 0.694 | 1.000 | 3510 | tags=41%, list=29%, signal=57% |
| 4376 | GOBP\_CHORIO\_ALLANTOIC\_FUSION |  | 4 | -0.59 | -1.06 | 0.411 | 0.694 | 1.000 | 624 | tags=25%, list=5%, signal=26% |
| 4377 | GOBP\_POSITIVE\_REGULATION\_OF\_CALCIUM\_ION\_TRANSPORT |  | 74 | -0.30 | -1.06 | 0.384 | 0.694 | 1.000 | 2908 | tags=27%, list=24%, signal=35% |
| 4378 | GOBP\_GOLGI\_ORGANIZATION |  | 104 | -0.29 | -1.06 | 0.374 | 0.694 | 1.000 | 4397 | tags=40%, list=37%, signal=63% |
| 4379 | GOBP\_UBIQUITIN\_DEPENDENT\_ENDOCYTOSIS |  | 4 | -0.59 | -1.06 | 0.410 | 0.694 | 1.000 | 950 | tags=25%, list=8%, signal=27% |
| 4380 | GOBP\_REGULATION\_OF\_SKELETAL\_MUSCLE\_CELL\_DIFFERENTIATION |  | 12 | -0.43 | -1.06 | 0.402 | 0.694 | 1.000 | 4432 | tags=42%, list=37%, signal=66% |
| 4381 | GOBP\_CELLULAR\_METABOLIC\_COMPOUND\_SALVAGE |  | 28 | -0.36 | -1.06 | 0.391 | 0.694 | 1.000 | 1018 | tags=18%, list=8%, signal=19% |
| 4382 | GOBP\_REGULATION\_OF\_MYOBLAST\_PROLIFERATION |  | 9 | -0.46 | -1.06 | 0.404 | 0.694 | 1.000 | 2905 | tags=44%, list=24%, signal=59% |
| 4383 | GOBP\_MAST\_CELL\_DIFFERENTIATION |  | 5 | -0.55 | -1.06 | 0.412 | 0.694 | 1.000 | 2308 | tags=60%, list=19%, signal=74% |
| 4384 | GOBP\_REGULATION\_OF\_TRANSCRIPTION\_INVOLVED\_IN\_G1\_S\_TRANSITION\_OF\_MITOTIC\_CELL\_CYCLE |  | 28 | -0.36 | -1.06 | 0.388 | 0.694 | 1.000 | 5323 | tags=54%, list=44%, signal=96% |
| 4385 | GOMF\_CHEMOKINE\_ACTIVITY |  | 15 | -0.41 | -1.06 | 0.414 | 0.694 | 1.000 | 1845 | tags=40%, list=15%, signal=47% |
| 4386 | GOMF\_BETA\_2\_ADRENERGIC\_RECEPTOR\_BINDING |  | 4 | -0.59 | -1.06 | 0.412 | 0.694 | 1.000 | 4949 | tags=100%, list=41%, signal=170% |
| 4387 | GOBP\_LYSOSOMAL\_PROTEIN\_CATABOLIC\_PROCESS |  | 11 | -0.44 | -1.06 | 0.411 | 0.694 | 1.000 | 3007 | tags=45%, list=25%, signal=61% |
| 4388 | GOBP\_ACTIN\_MEDIATED\_CELL\_CONTRACTION |  | 68 | -0.31 | -1.06 | 0.385 | 0.694 | 1.000 | 1890 | tags=19%, list=16%, signal=23% |
| 4389 | GOBP\_REGULATION\_OF\_CELLULAR\_RESPONSE\_TO\_VASCULAR\_ENDOTHELIAL\_GROWTH\_FACTOR\_STIMULUS |  | 17 | -0.39 | -1.06 | 0.400 | 0.694 | 1.000 | 3724 | tags=41%, list=31%, signal=60% |
| 4390 | GOBP\_APICAL\_JUNCTION\_ASSEMBLY |  | 56 | -0.32 | -1.06 | 0.397 | 0.694 | 1.000 | 2702 | tags=32%, list=22%, signal=41% |
| 4391 | GOBP\_CDP\_DIACYLGLYCEROL\_BIOSYNTHETIC\_PROCESS |  | 12 | -0.43 | -1.06 | 0.402 | 0.694 | 1.000 | 47 | tags=8%, list=0%, signal=8% |
| 4392 | GOBP\_BIOLOGICAL\_PROCESS\_INVOLVED\_IN\_INTERACTION\_WITH\_HOST |  | 141 | -0.29 | -1.06 | 0.383 | 0.694 | 1.000 | 3126 | tags=30%, list=26%, signal=41% |
| 4393 | GOBP\_CARDIAC\_VENTRICLE\_MORPHOGENESIS |  | 43 | -0.33 | -1.06 | 0.386 | 0.694 | 1.000 | 3615 | tags=28%, list=30%, signal=40% |
| 4394 | GOBP\_ANION\_TRANSMEMBRANE\_TRANSPORT |  | 355 | -0.27 | -1.06 | 0.320 | 0.694 | 1.000 | 3181 | tags=31%, list=26%, signal=41% |
| 4395 | GOBP\_REGULATION\_OF\_GLYCOPROTEIN\_METABOLIC\_PROCESS |  | 39 | -0.34 | -1.06 | 0.390 | 0.694 | 1.000 | 1173 | tags=15%, list=10%, signal=17% |
| 4396 | GOBP\_POSITIVE\_REGULATION\_OF\_DNA\_REPLICATION |  | 30 | -0.35 | -1.06 | 0.399 | 0.694 | 1.000 | 2888 | tags=33%, list=24%, signal=44% |
| 4397 | GOBP\_MACROMOLECULE\_METHYLATION |  | 210 | -0.28 | -1.06 | 0.357 | 0.695 | 1.000 | 3647 | tags=34%, list=30%, signal=48% |
| 4398 | GOBP\_RESPONSE\_TO\_ESTRADIOL |  | 88 | -0.30 | -1.06 | 0.386 | 0.695 | 1.000 | 2829 | tags=32%, list=24%, signal=41% |
| 4399 | GOMF\_CYCLOHYDROLASE\_ACTIVITY |  | 5 | -0.55 | -1.06 | 0.409 | 0.695 | 1.000 | 210 | tags=20%, list=2%, signal=20% |
| 4400 | GOBP\_NEGATIVE\_REGULATION\_OF\_TUMOR\_NECROSIS\_FACTOR\_MEDIATED\_SIGNALING\_PATHWAY |  | 11 | -0.44 | -1.06 | 0.417 | 0.695 | 1.000 | 5278 | tags=55%, list=44%, signal=97% |
| 4401 | GOBP\_RRNA\_5\_END\_PROCESSING |  | 3 | -0.63 | -1.06 | 0.403 | 0.695 | 1.000 | 1700 | tags=33%, list=14%, signal=39% |
| 4402 | GOBP\_REGULATION\_OF\_MYELOID\_LEUKOCYTE\_DIFFERENTIATION |  | 72 | -0.30 | -1.06 | 0.388 | 0.696 | 1.000 | 1743 | tags=25%, list=15%, signal=29% |
| 4403 | GOMF\_N\_ACETYLGLUCOSAMINE\_6\_O\_SULFOTRANSFERASE\_ACTIVITY |  | 5 | -0.55 | -1.06 | 0.410 | 0.696 | 1.000 | 696 | tags=20%, list=6%, signal=21% |
| 4404 | GOCC\_ZYMOGEN\_GRANULE\_MEMBRANE |  | 6 | -0.52 | -1.06 | 0.408 | 0.696 | 1.000 | 2878 | tags=33%, list=24%, signal=44% |
| 4405 | GOBP\_MUSCLE\_FIBER\_DEVELOPMENT |  | 27 | -0.36 | -1.06 | 0.398 | 0.697 | 1.000 | 3196 | tags=30%, list=27%, signal=40% |
| 4406 | GOBP\_REGULATION\_OF\_TRANSCRIPTION\_ELONGATION\_FROM\_RNA\_POLYMERASE\_II\_PROMOTER |  | 26 | -0.36 | -1.06 | 0.397 | 0.697 | 1.000 | 4336 | tags=50%, list=36%, signal=78% |
| 4407 | GOMF\_MYOSIN\_V\_BINDING |  | 12 | -0.43 | -1.06 | 0.415 | 0.697 | 1.000 | 2 | tags=8%, list=0%, signal=8% |
| 4408 | GOBP\_POSITIVE\_REGULATION\_OF\_SIGNAL\_TRANSDUCTION\_BY\_P53\_CLASS\_MEDIATOR |  | 15 | -0.40 | -1.06 | 0.411 | 0.697 | 1.000 | 3568 | tags=40%, list=30%, signal=57% |
| 4409 | GOMF\_LIM\_DOMAIN\_BINDING |  | 6 | -0.52 | -1.06 | 0.410 | 0.697 | 1.000 | 5080 | tags=67%, list=42%, signal=115% |
| 4410 | GOCC\_AP\_TYPE\_MEMBRANE\_COAT\_ADAPTOR\_COMPLEX |  | 29 | -0.35 | -1.06 | 0.403 | 0.698 | 1.000 | 4369 | tags=55%, list=36%, signal=87% |
| 4411 | GOBP\_INTERLEUKIN\_17\_PRODUCTION |  | 16 | -0.40 | -1.06 | 0.408 | 0.698 | 1.000 | 2322 | tags=38%, list=19%, signal=46% |
| 4412 | GOBP\_B\_CELL\_MEDIATED\_IMMUNITY |  | 70 | -0.31 | -1.06 | 0.392 | 0.698 | 1.000 | 3200 | tags=34%, list=27%, signal=46% |
| 4413 | GOBP\_REGULATION\_OF\_CAVEOLIN\_MEDIATED\_ENDOCYTOSIS |  | 4 | -0.59 | -1.06 | 0.410 | 0.698 | 1.000 | 198 | tags=25%, list=2%, signal=25% |
| 4414 | GOBP\_POSITIVE\_REGULATION\_OF\_PHAGOCYTOSIS |  | 43 | -0.33 | -1.06 | 0.401 | 0.698 | 1.000 | 3130 | tags=35%, list=26%, signal=47% |
| 4415 | GOBP\_COBALAMIN\_TRANSPORT |  | 6 | -0.52 | -1.06 | 0.415 | 0.699 | 1.000 | 4320 | tags=83%, list=36%, signal=130% |
| 4416 | GOBP\_DOUBLE\_STRAND\_BREAK\_REPAIR\_VIA\_SYNTHESIS\_DEPENDENT\_STRAND\_ANNEALING |  | 6 | -0.52 | -1.06 | 0.421 | 0.699 | 1.000 | 5752 | tags=100%, list=48%, signal=192% |
| 4417 | GOMF\_LIGAND\_ACTIVATED\_TRANSCRIPTION\_FACTOR\_ACTIVITY |  | 39 | -0.33 | -1.06 | 0.402 | 0.699 | 1.000 | 2732 | tags=36%, list=23%, signal=46% |
| 4418 | GOMF\_PEPTIDYL\_PROLINE\_4\_DIOXYGENASE\_ACTIVITY |  | 7 | -0.50 | -1.06 | 0.407 | 0.699 | 1.000 | 3164 | tags=43%, list=26%, signal=58% |
| 4419 | GOBP\_REGULATION\_OF\_MULTI\_ORGANISM\_PROCESS |  | 28 | -0.35 | -1.05 | 0.395 | 0.699 | 1.000 | 1539 | tags=25%, list=13%, signal=29% |
| 4420 | GOBP\_DOPAMINE\_RECEPTOR\_SIGNALING\_PATHWAY |  | 25 | -0.36 | -1.05 | 0.409 | 0.699 | 1.000 | 4277 | tags=44%, list=36%, signal=68% |
| 4421 | GOBP\_REGULATION\_OF\_SNARE\_COMPLEX\_ASSEMBLY |  | 8 | -0.48 | -1.05 | 0.412 | 0.700 | 1.000 | 3049 | tags=38%, list=25%, signal=50% |
| 4422 | GOBP\_PROTEIN\_C\_LINKED\_GLYCOSYLATION |  | 3 | -0.63 | -1.05 | 0.411 | 0.700 | 1.000 | 4461 | tags=100%, list=37%, signal=159% |
| 4423 | GOBP\_REGULATION\_OF\_BROWN\_FAT\_CELL\_DIFFERENTIATION |  | 16 | -0.40 | -1.05 | 0.400 | 0.700 | 1.000 | 3685 | tags=50%, list=31%, signal=72% |
| 4424 | GOBP\_REGULATION\_OF\_MELANOCYTE\_DIFFERENTIATION |  | 5 | -0.55 | -1.05 | 0.421 | 0.700 | 1.000 | 4277 | tags=80%, list=36%, signal=124% |
| 4425 | GOCC\_SPERM\_PLASMA\_MEMBRANE |  | 3 | -0.63 | -1.05 | 0.406 | 0.700 | 1.000 | 4190 | tags=67%, list=35%, signal=102% |
| 4426 | GOBP\_POSITIVE\_REGULATION\_OF\_REPRODUCTIVE\_PROCESS |  | 42 | -0.33 | -1.05 | 0.394 | 0.700 | 1.000 | 3657 | tags=36%, list=30%, signal=51% |
| 4427 | GOBP\_REGULATION\_OF\_COMPLEMENT\_DEPENDENT\_CYTOTOXICITY |  | 4 | -0.59 | -1.05 | 0.422 | 0.701 | 1.000 | 2237 | tags=50%, list=19%, signal=61% |
| 4428 | GOBP\_POSITIVE\_REGULATION\_OF\_RESPONSE\_TO\_BIOTIC\_STIMULUS |  | 140 | -0.28 | -1.05 | 0.381 | 0.701 | 1.000 | 3615 | tags=36%, list=30%, signal=50% |
| 4429 | GOMF\_COA\_TRANSFERASE\_ACTIVITY |  | 4 | -0.58 | -1.05 | 0.411 | 0.701 | 1.000 | 2993 | tags=50%, list=25%, signal=67% |
| 4430 | GOBP\_REGULATION\_OF\_SODIUM\_ION\_TRANSMEMBRANE\_TRANSPORTER\_ACTIVITY |  | 33 | -0.34 | -1.05 | 0.400 | 0.701 | 1.000 | 4286 | tags=55%, list=36%, signal=85% |
| 4431 | GOBP\_REGULATION\_OF\_CALCIDIOL\_1\_MONOOXYGENASE\_ACTIVITY |  | 4 | -0.58 | -1.05 | 0.415 | 0.701 | 1.000 | 2732 | tags=75%, list=23%, signal=97% |
| 4432 | GOBP\_MEMBRANE\_PROTEIN\_INTRACELLULAR\_DOMAIN\_PROTEOLYSIS |  | 18 | -0.39 | -1.05 | 0.412 | 0.701 | 1.000 | 2760 | tags=33%, list=23%, signal=43% |
| 4433 | GOBP\_POSITIVE\_REGULATION\_OF\_LIPID\_STORAGE |  | 14 | -0.41 | -1.05 | 0.413 | 0.701 | 1.000 | 5281 | tags=71%, list=44%, signal=127% |
| 4434 | GOBP\_CHEMICAL\_HOMEOSTASIS |  | 743 | -0.27 | -1.05 | 0.278 | 0.701 | 1.000 | 3318 | tags=33%, list=28%, signal=42% |
| 4435 | GOMF\_AXON\_GUIDANCE\_RECEPTOR\_ACTIVITY |  | 6 | -0.52 | -1.05 | 0.415 | 0.702 | 1.000 | 2300 | tags=33%, list=19%, signal=41% |
| 4436 | GOBP\_MODULATION\_BY\_HOST\_OF\_SYMBIONT\_PROCESS |  | 45 | -0.32 | -1.05 | 0.398 | 0.702 | 1.000 | 4743 | tags=44%, list=39%, signal=73% |
| 4437 | GOBP\_SYNAPTIC\_VESICLE\_TRANSPORT |  | 32 | -0.34 | -1.05 | 0.407 | 0.702 | 1.000 | 3569 | tags=44%, list=30%, signal=62% |
| 4438 | GOBP\_CARDIAC\_MYOFIBRIL\_ASSEMBLY |  | 10 | -0.45 | -1.05 | 0.415 | 0.702 | 1.000 | 3286 | tags=50%, list=27%, signal=69% |
| 4439 | GOMF\_STEROID\_HYDROXYLASE\_ACTIVITY |  | 14 | -0.41 | -1.05 | 0.413 | 0.702 | 1.000 | 2705 | tags=36%, list=23%, signal=46% |
| 4440 | GOBP\_ARTERIAL\_ENDOTHELIAL\_CELL\_DIFFERENTIATION |  | 6 | -0.52 | -1.05 | 0.423 | 0.702 | 1.000 | 3314 | tags=33%, list=28%, signal=46% |
| 4441 | GOBP\_NEGATIVE\_REGULATION\_OF\_BMP\_SIGNALING\_PATHWAY |  | 36 | -0.34 | -1.05 | 0.400 | 0.702 | 1.000 | 3126 | tags=39%, list=26%, signal=52% |
| 4442 | GOMF\_UBIQUITIN\_LIGASE\_SUBSTRATE\_ADAPTOR\_ACTIVITY |  | 12 | -0.43 | -1.05 | 0.407 | 0.702 | 1.000 | 2809 | tags=33%, list=23%, signal=43% |
| 4443 | GOBP\_NEGATIVE\_REGULATION\_OF\_RETINOIC\_ACID\_RECEPTOR\_SIGNALING\_PATHWAY |  | 5 | -0.55 | -1.05 | 0.423 | 0.702 | 1.000 | 5000 | tags=80%, list=42%, signal=137% |
| 4444 | GOBP\_MICROTUBULE\_ORGANIZING\_CENTER\_LOCALIZATION |  | 27 | -0.35 | -1.05 | 0.410 | 0.702 | 1.000 | 5219 | tags=56%, list=43%, signal=98% |
| 4445 | GOBP\_MULTI\_ORGANISM\_LOCALIZATION |  | 53 | -0.32 | -1.05 | 0.400 | 0.702 | 1.000 | 5843 | tags=62%, list=49%, signal=121% |
| 4446 | GOMF\_JUN\_KINASE\_KINASE\_KINASE\_ACTIVITY |  | 5 | -0.54 | -1.05 | 0.410 | 0.702 | 1.000 | 142 | tags=20%, list=1%, signal=20% |
| 4447 | GOBP\_ALCOHOL\_BIOSYNTHETIC\_PROCESS |  | 113 | -0.29 | -1.05 | 0.389 | 0.702 | 1.000 | 4283 | tags=44%, list=36%, signal=68% |
| 4448 | GOBP\_REGULATION\_OF\_SPINDLE\_ASSEMBLY |  | 21 | -0.38 | -1.05 | 0.416 | 0.702 | 1.000 | 3970 | tags=43%, list=33%, signal=64% |
| 4449 | GOBP\_RESPONSE\_TO\_STARVATION |  | 152 | -0.28 | -1.05 | 0.379 | 0.702 | 1.000 | 3993 | tags=37%, list=33%, signal=54% |
| 4450 | GOBP\_ORGAN\_INDUCTION |  | 13 | -0.42 | -1.05 | 0.416 | 0.703 | 1.000 | 2300 | tags=38%, list=19%, signal=48% |
| 4451 | GOCC\_MITOCHONDRIA\_ASSOCIATED\_ENDOPLASMIC\_RETICULUM\_MEMBRANE |  | 16 | -0.40 | -1.05 | 0.414 | 0.703 | 1.000 | 3410 | tags=50%, list=28%, signal=70% |
| 4452 | GOBP\_NEGATIVE\_REGULATION\_OF\_JNK\_CASCADE |  | 23 | -0.37 | -1.05 | 0.409 | 0.703 | 1.000 | 2894 | tags=35%, list=24%, signal=46% |
| 4453 | GOBP\_POSITIVE\_REGULATION\_OF\_CELL\_CYCLE\_PHASE\_TRANSITION |  | 75 | -0.30 | -1.05 | 0.392 | 0.702 | 1.000 | 5629 | tags=57%, list=47%, signal=107% |
| 4454 | GOBP\_NEGATIVE\_REGULATION\_OF\_CELL\_GROWTH |  | 128 | -0.29 | -1.05 | 0.387 | 0.702 | 1.000 | 2731 | tags=25%, list=23%, signal=32% |
| 4455 | GOBP\_CELLULAR\_RESPONSE\_TO\_OXYGEN\_GLUCOSE\_DEPRIVATION |  | 4 | -0.58 | -1.05 | 0.410 | 0.702 | 1.000 | 139 | tags=25%, list=1%, signal=25% |
| 4456 | GOBP\_REGULATION\_OF\_CARDIAC\_MUSCLE\_CELL\_PROLIFERATION |  | 26 | -0.36 | -1.05 | 0.410 | 0.702 | 1.000 | 3510 | tags=42%, list=29%, signal=60% |
| 4457 | GOBP\_REGULATION\_OF\_PROTEIN\_ACETYLATION |  | 58 | -0.31 | -1.05 | 0.393 | 0.703 | 1.000 | 3626 | tags=34%, list=30%, signal=49% |
| 4458 | GOBP\_DEVELOPMENT\_OF\_PRIMARY\_FEMALE\_SEXUAL\_CHARACTERISTICS |  | 63 | -0.31 | -1.05 | 0.396 | 0.703 | 1.000 | 4692 | tags=48%, list=39%, signal=78% |
| 4459 | GOBP\_NEGATIVE\_REGULATION\_OF\_MAST\_CELL\_ACTIVATION |  | 7 | -0.50 | -1.05 | 0.425 | 0.703 | 1.000 | 726 | tags=29%, list=6%, signal=30% |
| 4460 | GOBP\_NLS\_BEARING\_PROTEIN\_IMPORT\_INTO\_NUCLEUS |  | 9 | -0.46 | -1.05 | 0.423 | 0.703 | 1.000 | 6064 | tags=89%, list=50%, signal=179% |
| 4461 | GOBP\_POSITIVE\_REGULATION\_OF\_HEXOKINASE\_ACTIVITY |  | 3 | -0.63 | -1.05 | 0.411 | 0.703 | 1.000 | 320 | tags=33%, list=3%, signal=34% |
| 4462 | GOBP\_CELLULAR\_RESPONSE\_TO\_INTERFERON\_ALPHA |  | 6 | -0.52 | -1.05 | 0.412 | 0.703 | 1.000 | 2482 | tags=50%, list=21%, signal=63% |
| 4463 | GOBP\_PEPTIDYL\_ARGININE\_OMEGA\_N\_METHYLATION |  | 4 | -0.58 | -1.05 | 0.429 | 0.703 | 1.000 | 3647 | tags=75%, list=30%, signal=108% |
| 4464 | GOBP\_SENSORY\_PERCEPTION\_OF\_MECHANICAL\_STIMULUS |  | 85 | -0.30 | -1.05 | 0.398 | 0.703 | 1.000 | 1741 | tags=21%, list=14%, signal=25% |
| 4465 | GOBP\_BASE\_EXCISION\_REPAIR\_GAP\_FILLING |  | 5 | -0.54 | -1.05 | 0.416 | 0.704 | 1.000 | 1545 | tags=20%, list=13%, signal=23% |
| 4466 | GOBP\_POSITIVE\_REGULATION\_OF\_FC\_GAMMA\_RECEPTOR\_SIGNALING\_PATHWAY\_INVOLVED\_IN\_PHAGOCYTOSIS |  | 5 | -0.55 | -1.05 | 0.426 | 0.704 | 1.000 | 1519 | tags=40%, list=13%, signal=46% |
| 4467 | GOBP\_POSITIVE\_REGULATION\_OF\_PLASMINOGEN\_ACTIVATION |  | 3 | -0.63 | -1.05 | 0.425 | 0.704 | 1.000 | 1454 | tags=67%, list=12%, signal=76% |
| 4468 | GOBP\_REGULATION\_OF\_NITRIC\_OXIDE\_METABOLIC\_PROCESS |  | 39 | -0.33 | -1.05 | 0.401 | 0.704 | 1.000 | 2871 | tags=28%, list=24%, signal=37% |
| 4469 | GOBP\_NEGATIVE\_REGULATION\_OF\_CATABOLIC\_PROCESS |  | 223 | -0.27 | -1.05 | 0.370 | 0.704 | 1.000 | 2518 | tags=23%, list=21%, signal=29% |
| 4470 | GOBP\_REGULATION\_OF\_LIPOPOLYSACCHARIDE\_MEDIATED\_SIGNALING\_PATHWAY |  | 16 | -0.40 | -1.05 | 0.415 | 0.704 | 1.000 | 1457 | tags=25%, list=12%, signal=28% |
| 4471 | GOBP\_NEGATIVE\_REGULATION\_OF\_INTERLEUKIN\_8\_PRODUCTION |  | 8 | -0.47 | -1.05 | 0.407 | 0.704 | 1.000 | 1072 | tags=25%, list=9%, signal=27% |
| 4472 | GOBP\_POSITIVE\_REGULATION\_OF\_PEPTIDYL\_SERINE\_PHOSPHORYLATION |  | 63 | -0.31 | -1.05 | 0.398 | 0.705 | 1.000 | 3613 | tags=32%, list=30%, signal=45% |
| 4473 | GOBP\_MITOPHAGY |  | 19 | -0.38 | -1.05 | 0.414 | 0.704 | 1.000 | 2263 | tags=32%, list=19%, signal=39% |
| 4474 | GOBP\_REGULATION\_OF\_ERBB\_SIGNALING\_PATHWAY |  | 73 | -0.30 | -1.05 | 0.403 | 0.705 | 1.000 | 2354 | tags=26%, list=20%, signal=32% |
| 4475 | GOBP\_POSITIVE\_REGULATION\_OF\_WNT\_SIGNALING\_PATHWAY |  | 133 | -0.28 | -1.05 | 0.385 | 0.705 | 1.000 | 3565 | tags=33%, list=30%, signal=47% |
| 4476 | GOMF\_OXIDOREDUCTASE\_ACTIVITY\_ACTING\_ON\_CH\_OR\_CH2\_GROUPS |  | 6 | -0.52 | -1.05 | 0.418 | 0.705 | 1.000 | 5284 | tags=83%, list=44%, signal=149% |
| 4477 | GOBP\_LONG\_TERM\_MEMORY |  | 21 | -0.37 | -1.05 | 0.418 | 0.704 | 1.000 | 2456 | tags=38%, list=20%, signal=48% |
| 4478 | GOMF\_PHOSPHATIDYLINOSITOL\_3\_4\_5\_TRISPHOSPHATE\_3\_PHOSPHATASE\_ACTIVITY |  | 4 | -0.58 | -1.05 | 0.421 | 0.705 | 1.000 | 4540 | tags=75%, list=38%, signal=121% |
| 4479 | GOMF\_STRUCTURAL\_CONSTITUENT\_OF\_PRESYNAPTIC\_ACTIVE\_ZONE |  | 3 | -0.63 | -1.05 | 0.418 | 0.705 | 1.000 | 4455 | tags=100%, list=37%, signal=159% |
| 4480 | GOMF\_TOXIN\_TRANSMEMBRANE\_TRANSPORTER\_ACTIVITY |  | 4 | -0.58 | -1.05 | 0.424 | 0.705 | 1.000 | 2392 | tags=50%, list=20%, signal=62% |
| 4481 | GOBP\_BROWN\_FAT\_CELL\_DIFFERENTIATION |  | 33 | -0.34 | -1.05 | 0.410 | 0.705 | 1.000 | 3784 | tags=45%, list=32%, signal=66% |
| 4482 | GOCC\_EXTRINSIC\_COMPONENT\_OF\_ORGANELLE\_MEMBRANE |  | 38 | -0.33 | -1.05 | 0.409 | 0.705 | 1.000 | 1565 | tags=21%, list=13%, signal=24% |
| 4483 | GOBP\_REGULATION\_OF\_CAMP\_DEPENDENT\_PROTEIN\_KINASE\_ACTIVITY |  | 17 | -0.39 | -1.05 | 0.414 | 0.707 | 1.000 | 4008 | tags=41%, list=33%, signal=62% |
| 4484 | GOBP\_REGULATION\_OF\_APOPTOTIC\_CELL\_CLEARANCE |  | 4 | -0.58 | -1.05 | 0.429 | 0.707 | 1.000 | 4649 | tags=75%, list=39%, signal=122% |
| 4485 | GOCC\_TRANS\_GOLGI\_NETWORK |  | 180 | -0.28 | -1.05 | 0.387 | 0.707 | 1.000 | 4444 | tags=39%, list=37%, signal=62% |
| 4486 | GOBP\_N\_TERMINAL\_PROTEIN\_AMINO\_ACID\_ACETYLATION |  | 12 | -0.43 | -1.05 | 0.421 | 0.707 | 1.000 | 4976 | tags=50%, list=41%, signal=85% |
| 4487 | GOBP\_CELLULAR\_LIPID\_METABOLIC\_PROCESS |  | 730 | -0.26 | -1.05 | 0.294 | 0.707 | 1.000 | 4194 | tags=39%, list=35%, signal=56% |
| 4488 | GOBP\_COMPLEMENT\_DEPENDENT\_CYTOTOXICITY |  | 6 | -0.51 | -1.05 | 0.424 | 0.707 | 1.000 | 2237 | tags=33%, list=19%, signal=41% |
| 4489 | GOMF\_G\_RICH\_STRAND\_TELOMERIC\_DNA\_BINDING |  | 10 | -0.45 | -1.05 | 0.422 | 0.707 | 1.000 | 5648 | tags=80%, list=47%, signal=151% |
| 4490 | GOBP\_REGULATION\_OF\_COHESIN\_LOADING |  | 3 | -0.63 | -1.05 | 0.425 | 0.708 | 1.000 | 3836 | tags=67%, list=32%, signal=98% |
| 4491 | GOCC\_SAGA\_TYPE\_COMPLEX |  | 23 | -0.37 | -1.05 | 0.420 | 0.708 | 1.000 | 3626 | tags=35%, list=30%, signal=50% |
| 4492 | GOBP\_POSITIVE\_REGULATION\_OF\_DOUBLE\_STRAND\_BREAK\_REPAIR |  | 29 | -0.35 | -1.05 | 0.412 | 0.708 | 1.000 | 3434 | tags=38%, list=29%, signal=53% |
| 4493 | GOBP\_TRANSMISSION\_OF\_NERVE\_IMPULSE |  | 29 | -0.35 | -1.05 | 0.414 | 0.708 | 1.000 | 4110 | tags=38%, list=34%, signal=58% |
| 4494 | GOBP\_POSITIVE\_REGULATION\_OF\_EXTRACELLULAR\_MATRIX\_ORGANIZATION |  | 18 | -0.39 | -1.05 | 0.421 | 0.708 | 1.000 | 3836 | tags=56%, list=32%, signal=82% |
| 4495 | GOBP\_PROTEIN\_POLYUBIQUITINATION |  | 245 | -0.27 | -1.05 | 0.373 | 0.708 | 1.000 | 4419 | tags=37%, list=37%, signal=57% |
| 4496 | GOBP\_CHROMOSOME\_ATTACHMENT\_TO\_THE\_NUCLEAR\_ENVELOPE |  | 3 | -0.63 | -1.05 | 0.423 | 0.708 | 1.000 | 357 | tags=33%, list=3%, signal=34% |
| 4497 | GOBP\_CELL\_PROLIFERATION\_IN\_BONE\_MARROW |  | 5 | -0.54 | -1.05 | 0.428 | 0.708 | 1.000 | 3400 | tags=60%, list=28%, signal=84% |
| 4498 | GOBP\_ESTABLISHMENT\_OF\_PROTEIN\_LOCALIZATION\_TO\_VACUOLE |  | 41 | -0.33 | -1.05 | 0.403 | 0.708 | 1.000 | 3112 | tags=34%, list=26%, signal=46% |
| 4499 | GOBP\_RESPONSE\_TO\_TEMPERATURE\_STIMULUS |  | 164 | -0.28 | -1.05 | 0.388 | 0.708 | 1.000 | 4193 | tags=40%, list=35%, signal=60% |
| 4500 | GOBP\_POSITIVE\_REGULATION\_OF\_LIPASE\_ACTIVITY |  | 49 | -0.32 | -1.05 | 0.409 | 0.709 | 1.000 | 1678 | tags=20%, list=14%, signal=24% |
| 4501 | GOBP\_SPLICEOSOMAL\_CONFORMATIONAL\_CHANGES\_TO\_GENERATE\_CATALYTIC\_CONFORMATION |  | 4 | -0.58 | -1.05 | 0.426 | 0.709 | 1.000 | 794 | tags=25%, list=7%, signal=27% |
| 4502 | GOBP\_ORGANIC\_ANION\_TRANSPORT |  | 235 | -0.27 | -1.05 | 0.376 | 0.709 | 1.000 | 3022 | tags=32%, list=25%, signal=42% |
| 4503 | GOBP\_POSITIVE\_REGULATION\_OF\_ENDOTHELIAL\_CELL\_CHEMOTAXIS\_BY\_VEGF\_ACTIVATED\_VASCULAR\_ENDOTHELIAL\_GROWTH\_FACTOR\_RECEPTOR\_SIGNALING\_PATHWAY |  | 4 | -0.58 | -1.05 | 0.426 | 0.709 | 1.000 | 3588 | tags=75%, list=30%, signal=107% |
| 4504 | GOBP\_POSITIVE\_REGULATION\_OF\_URINE\_VOLUME |  | 9 | -0.46 | -1.05 | 0.429 | 0.710 | 1.000 | 5384 | tags=67%, list=45%, signal=121% |
| 4505 | GOBP\_NEGATIVE\_REGULATION\_OF\_PEPTIDYL\_SERINE\_PHOSPHORYLATION |  | 23 | -0.36 | -1.05 | 0.413 | 0.710 | 1.000 | 3978 | tags=43%, list=33%, signal=65% |
| 4506 | GOCC\_9PLUS0\_NON\_MOTILE\_CILIUM |  | 59 | -0.31 | -1.05 | 0.414 | 0.710 | 1.000 | 4590 | tags=42%, list=38%, signal=68% |
| 4507 | GOBP\_REGULATION\_OF\_NUCLEOTIDE\_METABOLIC\_PROCESS |  | 84 | -0.30 | -1.05 | 0.405 | 0.711 | 1.000 | 4317 | tags=45%, list=36%, signal=70% |
| 4508 | GOBP\_DEFENSE\_RESPONSE\_TO\_OTHER\_ORGANISM |  | 534 | -0.27 | -1.05 | 0.331 | 0.712 | 1.000 | 3166 | tags=30%, list=26%, signal=39% |
| 4509 | GOBP\_POSITIVE\_REGULATION\_OF\_PHOSPHOLIPID\_TRANSLOCATION |  | 4 | -0.58 | -1.05 | 0.432 | 0.712 | 1.000 | 5062 | tags=100%, list=42%, signal=173% |
| 4510 | GOCC\_G\_PROTEIN\_COUPLED\_RECEPTOR\_DIMERIC\_COMPLEX |  | 4 | -0.58 | -1.05 | 0.426 | 0.712 | 1.000 | 5053 | tags=75%, list=42%, signal=129% |
| 4511 | GOBP\_REGULATION\_OF\_MUCUS\_SECRETION |  | 3 | -0.62 | -1.05 | 0.422 | 0.712 | 1.000 | 4561 | tags=100%, list=38%, signal=161% |
| 4512 | GOBP\_POSITIVE\_REGULATION\_OF\_SYNAPTIC\_VESICLE\_ENDOCYTOSIS |  | 4 | -0.58 | -1.04 | 0.435 | 0.713 | 1.000 | 4673 | tags=50%, list=39%, signal=82% |
| 4513 | GOBP\_ORGANELLE\_TRANSPORT\_ALONG\_MICROTUBULE |  | 69 | -0.30 | -1.04 | 0.409 | 0.713 | 1.000 | 4052 | tags=38%, list=34%, signal=57% |
| 4514 | GOMF\_NADH\_PYROPHOSPHATASE\_ACTIVITY |  | 6 | -0.51 | -1.04 | 0.429 | 0.713 | 1.000 | 3094 | tags=50%, list=26%, signal=67% |
| 4515 | GOMF\_S\_ACYLTRANSFERASE\_ACTIVITY |  | 24 | -0.36 | -1.04 | 0.416 | 0.714 | 1.000 | 2801 | tags=25%, list=23%, signal=33% |
| 4516 | GOBP\_ECTODERM\_DEVELOPMENT |  | 11 | -0.43 | -1.04 | 0.430 | 0.714 | 1.000 | 3565 | tags=55%, list=30%, signal=77% |
| 4517 | GOBP\_RESPONSE\_TO\_ACETYLCHOLINE |  | 20 | -0.38 | -1.04 | 0.421 | 0.714 | 1.000 | 929 | tags=15%, list=8%, signal=16% |
| 4518 | GOBP\_TRIGLYCERIDE\_CATABOLIC\_PROCESS |  | 25 | -0.36 | -1.04 | 0.415 | 0.714 | 1.000 | 3936 | tags=44%, list=33%, signal=65% |
| 4519 | GOBP\_CORONARY\_VASCULATURE\_MORPHOGENESIS |  | 13 | -0.42 | -1.04 | 0.423 | 0.714 | 1.000 | 3348 | tags=54%, list=28%, signal=75% |
| 4520 | GOBP\_CARBOHYDRATE\_TRANSMEMBRANE\_TRANSPORT |  | 76 | -0.30 | -1.04 | 0.411 | 0.714 | 1.000 | 3997 | tags=42%, list=33%, signal=63% |
| 4521 | GOBP\_ROUNDABOUT\_SIGNALING\_PATHWAY |  | 5 | -0.54 | -1.04 | 0.430 | 0.714 | 1.000 | 4696 | tags=60%, list=39%, signal=98% |
| 4522 | GOBP\_VENTRAL\_SPINAL\_CORD\_DEVELOPMENT |  | 27 | -0.35 | -1.04 | 0.418 | 0.714 | 1.000 | 2342 | tags=26%, list=19%, signal=32% |
| 4523 | GOBP\_CORONARY\_VASCULATURE\_DEVELOPMENT |  | 35 | -0.34 | -1.04 | 0.411 | 0.714 | 1.000 | 4775 | tags=51%, list=40%, signal=85% |
| 4524 | GOBP\_AMINOGLYCAN\_BIOSYNTHETIC\_PROCESS |  | 83 | -0.30 | -1.04 | 0.411 | 0.715 | 1.000 | 4102 | tags=37%, list=34%, signal=56% |
| 4525 | GOBP\_CEREBRAL\_CORTEX\_GABAERGIC\_INTERNEURON\_DIFFERENTIATION |  | 4 | -0.58 | -1.04 | 0.434 | 0.715 | 1.000 | 2563 | tags=50%, list=21%, signal=64% |
| 4526 | GOBP\_REGULATION\_OF\_BONE\_REMODELING |  | 38 | -0.33 | -1.04 | 0.408 | 0.715 | 1.000 | 1999 | tags=29%, list=17%, signal=35% |
| 4527 | GOBP\_POSITIVE\_REGULATION\_OF\_HISTONE\_PHOSPHORYLATION |  | 7 | -0.49 | -1.04 | 0.436 | 0.715 | 1.000 | 3331 | tags=57%, list=28%, signal=79% |
| 4528 | GOBP\_PHOSPHATIDYLCHOLINE\_METABOLIC\_PROCESS |  | 53 | -0.31 | -1.04 | 0.418 | 0.715 | 1.000 | 1864 | tags=21%, list=16%, signal=24% |
| 4529 | GOBP\_RESPONSE\_TO\_COPPER\_ION |  | 18 | -0.39 | -1.04 | 0.433 | 0.715 | 1.000 | 1782 | tags=22%, list=15%, signal=26% |
| 4530 | GOBP\_UROGENITAL\_SYSTEM\_DEVELOPMENT |  | 243 | -0.27 | -1.04 | 0.388 | 0.715 | 1.000 | 4111 | tags=37%, list=34%, signal=56% |
| 4531 | GOBP\_XENOPHAGY |  | 9 | -0.46 | -1.04 | 0.438 | 0.714 | 1.000 | 5272 | tags=56%, list=44%, signal=99% |
| 4532 | GOBP\_NEGATIVE\_REGULATION\_OF\_HUMORAL\_IMMUNE\_RESPONSE |  | 7 | -0.49 | -1.04 | 0.425 | 0.716 | 1.000 | 2249 | tags=43%, list=19%, signal=53% |
| 4533 | GOBP\_CELL\_CYCLE\_DNA\_REPLICATION\_INITIATION |  | 5 | -0.54 | -1.04 | 0.433 | 0.716 | 1.000 | 4233 | tags=80%, list=35%, signal=123% |
| 4534 | GOCC\_ANCHORED\_COMPONENT\_OF\_SYNAPTIC\_VESICLE\_MEMBRANE |  | 10 | -0.45 | -1.04 | 0.435 | 0.716 | 1.000 | 2 | tags=10%, list=0%, signal=10% |
| 4535 | GOBP\_PORE\_COMPLEX\_ASSEMBLY |  | 18 | -0.39 | -1.04 | 0.431 | 0.716 | 1.000 | 4770 | tags=61%, list=40%, signal=101% |
| 4536 | GOBP\_ENDOCRINE\_SYSTEM\_DEVELOPMENT |  | 76 | -0.30 | -1.04 | 0.415 | 0.717 | 1.000 | 2351 | tags=26%, list=20%, signal=33% |
| 4537 | GOBP\_LYSOSOMAL\_MEMBRANE\_ORGANIZATION |  | 5 | -0.54 | -1.04 | 0.433 | 0.716 | 1.000 | 5224 | tags=60%, list=43%, signal=106% |
| 4538 | GOBP\_PURKINJE\_MYOCYTE\_TO\_VENTRICULAR\_CARDIAC\_MUSCLE\_CELL\_SIGNALING |  | 3 | -0.63 | -1.04 | 0.435 | 0.717 | 1.000 | 3295 | tags=67%, list=27%, signal=92% |
| 4539 | GOBP\_NEGATIVE\_REGULATION\_OF\_RECEPTOR\_SIGNALING\_PATHWAY\_VIA\_JAK\_STAT |  | 20 | -0.37 | -1.04 | 0.427 | 0.717 | 1.000 | 1541 | tags=25%, list=13%, signal=29% |
| 4540 | GOCC\_VACUOLAR\_LUMEN |  | 117 | -0.28 | -1.04 | 0.406 | 0.717 | 1.000 | 5390 | tags=50%, list=45%, signal=91% |
| 4541 | GOMF\_U3\_SNORNA\_BINDING |  | 7 | -0.49 | -1.04 | 0.431 | 0.717 | 1.000 | 315 | tags=14%, list=3%, signal=15% |
| 4542 | GOBP\_REGULATION\_OF\_RESPONSE\_TO\_ENDOPLASMIC\_RETICULUM\_STRESS |  | 59 | -0.31 | -1.04 | 0.411 | 0.717 | 1.000 | 4797 | tags=49%, list=40%, signal=81% |
| 4543 | GOBP\_REGULATION\_OF\_EXTRACELLULAR\_MATRIX\_ORGANIZATION |  | 37 | -0.33 | -1.04 | 0.417 | 0.717 | 1.000 | 3836 | tags=43%, list=32%, signal=63% |
| 4544 | GOBP\_CELLULAR\_RESPONSE\_TO\_PEPTIDE\_HORMONE\_STIMULUS |  | 236 | -0.27 | -1.04 | 0.384 | 0.717 | 1.000 | 3797 | tags=43%, list=32%, signal=61% |
| 4545 | GOMF\_POLY\_PURINE\_TRACT\_BINDING |  | 24 | -0.36 | -1.04 | 0.420 | 0.717 | 1.000 | 5700 | tags=54%, list=47%, signal=103% |
| 4546 | GOBP\_PARANODAL\_JUNCTION\_ASSEMBLY |  | 5 | -0.54 | -1.04 | 0.437 | 0.717 | 1.000 | 5264 | tags=80%, list=44%, signal=142% |
| 4547 | GOBP\_DIGESTIVE\_SYSTEM\_DEVELOPMENT |  | 91 | -0.29 | -1.04 | 0.419 | 0.717 | 1.000 | 2008 | tags=21%, list=17%, signal=25% |
| 4548 | GOBP\_REGULATION\_OF\_SYNAPTIC\_VESICLE\_PRIMING |  | 7 | -0.49 | -1.04 | 0.421 | 0.717 | 1.000 | 2312 | tags=57%, list=19%, signal=71% |
| 4549 | GOCC\_SEX\_CHROMOSOME |  | 18 | -0.39 | -1.04 | 0.430 | 0.717 | 1.000 | 3137 | tags=33%, list=26%, signal=45% |
| 4550 | GOMF\_EXORIBONUCLEASE\_ACTIVITY |  | 35 | -0.34 | -1.04 | 0.423 | 0.718 | 1.000 | 5755 | tags=54%, list=48%, signal=104% |
| 4551 | GOBP\_ENDOTHELIAL\_CELL\_APOPTOTIC\_PROCESS |  | 37 | -0.33 | -1.04 | 0.419 | 0.718 | 1.000 | 1637 | tags=19%, list=14%, signal=22% |
| 4552 | GOBP\_POSITIVE\_REGULATION\_OF\_HUMORAL\_IMMUNE\_RESPONSE |  | 9 | -0.46 | -1.04 | 0.420 | 0.718 | 1.000 | 1519 | tags=22%, list=13%, signal=25% |
| 4553 | GOBP\_GLUTAMATE\_HOMEOSTASIS |  | 3 | -0.62 | -1.04 | 0.434 | 0.718 | 1.000 | 1222 | tags=33%, list=10%, signal=37% |
| 4554 | GOBP\_RESPONSE\_TO\_GROWTH\_HORMONE |  | 25 | -0.36 | -1.04 | 0.421 | 0.718 | 1.000 | 960 | tags=20%, list=8%, signal=22% |
| 4555 | GOBP\_NEGATIVE\_REGULATION\_OF\_TRANSLATIONAL\_INITIATION |  | 13 | -0.42 | -1.04 | 0.427 | 0.718 | 1.000 | 1039 | tags=23%, list=9%, signal=25% |
| 4556 | GOBP\_ARGININE\_TRANSPORT |  | 7 | -0.49 | -1.04 | 0.435 | 0.718 | 1.000 | 2847 | tags=43%, list=24%, signal=56% |
| 4557 | GOMF\_ENDONUCLEASE\_ACTIVITY\_ACTIVE\_WITH\_EITHER\_RIBO\_OR\_DEOXYRIBONUCLEIC\_ACIDS\_AND\_PRODUCING\_5\_PHOSPHOMONOESTERS |  | 23 | -0.36 | -1.04 | 0.420 | 0.718 | 1.000 | 2733 | tags=35%, list=23%, signal=45% |
| 4558 | GOBP\_ORGANOPHOSPHATE\_METABOLIC\_PROCESS |  | 732 | -0.26 | -1.04 | 0.328 | 0.719 | 1.000 | 4255 | tags=38%, list=35%, signal=56% |
| 4559 | GOMF\_TESTOSTERONE\_DEHYDROGENASE\_NAD\_P\_ACTIVITY |  | 3 | -0.63 | -1.04 | 0.443 | 0.719 | 1.000 | 101 | tags=33%, list=1%, signal=34% |
| 4560 | GOBP\_POSITIVE\_REGULATION\_OF\_DNA\_DAMAGE\_RESPONSE\_SIGNAL\_TRANSDUCTION\_BY\_P53\_CLASS\_MEDIATOR |  | 9 | -0.46 | -1.04 | 0.443 | 0.719 | 1.000 | 3568 | tags=56%, list=30%, signal=79% |
| 4561 | GOMF\_METALLOCARBOXYPEPTIDASE\_ACTIVITY |  | 16 | -0.40 | -1.04 | 0.431 | 0.719 | 1.000 | 4305 | tags=50%, list=36%, signal=78% |
| 4562 | GOCC\_MYOSIN\_COMPLEX |  | 33 | -0.34 | -1.04 | 0.417 | 0.719 | 1.000 | 4122 | tags=45%, list=34%, signal=69% |
| 4563 | GOMF\_INTERFERON\_RECEPTOR\_ACTIVITY |  | 4 | -0.57 | -1.04 | 0.436 | 0.719 | 1.000 | 2482 | tags=50%, list=21%, signal=63% |
| 4564 | GOBP\_NEGATIVE\_REGULATION\_OF\_RIBOSOME\_BIOGENESIS |  | 4 | -0.57 | -1.04 | 0.439 | 0.719 | 1.000 | 3392 | tags=50%, list=28%, signal=70% |
| 4565 | GOMF\_TRANSFERASE\_ACTIVITY\_TRANSFERRING\_SULFUR\_CONTAINING\_GROUPS |  | 45 | -0.32 | -1.04 | 0.426 | 0.720 | 1.000 | 3745 | tags=40%, list=31%, signal=58% |
| 4566 | GOBP\_BRONCHUS\_DEVELOPMENT |  | 7 | -0.49 | -1.04 | 0.429 | 0.720 | 1.000 | 106 | tags=14%, list=1%, signal=14% |
| 4567 | GOCC\_BASAL\_CORTEX |  | 5 | -0.54 | -1.04 | 0.436 | 0.720 | 1.000 | 3551 | tags=60%, list=30%, signal=85% |
| 4568 | GOBP\_REGULATION\_OF\_ASTROCYTE\_ACTIVATION |  | 3 | -0.62 | -1.04 | 0.445 | 0.720 | 1.000 | 219 | tags=33%, list=2%, signal=34% |
| 4569 | GOBP\_CELL\_CELL\_SIGNALING\_BY\_WNT |  | 372 | -0.27 | -1.04 | 0.382 | 0.721 | 1.000 | 3565 | tags=30%, list=30%, signal=42% |
| 4570 | GOMF\_ENZYME\_INHIBITOR\_ACTIVITY |  | 204 | -0.27 | -1.04 | 0.403 | 0.720 | 1.000 | 4027 | tags=31%, list=34%, signal=46% |
| 4571 | GOBP\_NEGATIVE\_REGULATION\_OF\_DEFENSE\_RESPONSE\_TO\_BACTERIUM |  | 3 | -0.62 | -1.04 | 0.436 | 0.720 | 1.000 | 1788 | tags=33%, list=15%, signal=39% |
| 4572 | GOCC\_SYNAPTIC\_CLEFT |  | 8 | -0.47 | -1.04 | 0.438 | 0.721 | 1.000 | 1222 | tags=25%, list=10%, signal=28% |
| 4573 | GOBP\_CELLULAR\_RESPONSE\_TO\_HORMONE\_STIMULUS |  | 434 | -0.27 | -1.04 | 0.371 | 0.722 | 1.000 | 4008 | tags=41%, list=33%, signal=60% |
| 4574 | GOCC\_SYNAPTIC\_VESICLE\_MEMBRANE |  | 62 | -0.31 | -1.04 | 0.431 | 0.722 | 1.000 | 3909 | tags=40%, list=33%, signal=59% |
| 4575 | GOBP\_TAURINE\_TRANSPORT |  | 5 | -0.54 | -1.04 | 0.444 | 0.722 | 1.000 | 4189 | tags=80%, list=35%, signal=123% |
| 4576 | GOMF\_ENDONUCLEASE\_ACTIVITY\_ACTIVE\_WITH\_EITHER\_RIBO\_OR\_DEOXYRIBONUCLEIC\_ACIDS\_AND\_PRODUCING\_3\_PHOSPHOMONOESTERS |  | 8 | -0.47 | -1.04 | 0.444 | 0.723 | 1.000 | 5752 | tags=75%, list=48%, signal=144% |
| 4577 | GOMF\_QUATERNARY\_AMMONIUM\_GROUP\_BINDING |  | 20 | -0.37 | -1.04 | 0.436 | 0.723 | 1.000 | 4280 | tags=50%, list=36%, signal=78% |
| 4578 | GOMF\_ANGIOTENSIN\_RECEPTOR\_BINDING |  | 4 | -0.58 | -1.04 | 0.442 | 0.723 | 1.000 | 889 | tags=25%, list=7%, signal=27% |
| 4579 | GOBP\_REGULATION\_OF\_PROTEIN\_LOCALIZATION\_TO\_NUCLEUS |  | 112 | -0.28 | -1.04 | 0.418 | 0.723 | 1.000 | 4032 | tags=32%, list=34%, signal=48% |
| 4580 | GOBP\_REGULATION\_OF\_EMBRYONIC\_DEVELOPMENT |  | 43 | -0.32 | -1.04 | 0.429 | 0.724 | 1.000 | 3551 | tags=33%, list=30%, signal=46% |
| 4581 | GOBP\_MULTICELLULAR\_ORGANISMAL\_HOMEOSTASIS |  | 334 | -0.27 | -1.04 | 0.389 | 0.724 | 1.000 | 4112 | tags=39%, list=34%, signal=58% |
| 4582 | GOBP\_RESPONSE\_TO\_CAMP |  | 56 | -0.31 | -1.04 | 0.425 | 0.724 | 1.000 | 3973 | tags=36%, list=33%, signal=53% |
| 4583 | GOBP\_ENDOTHELIUM\_DEVELOPMENT |  | 99 | -0.29 | -1.04 | 0.416 | 0.723 | 1.000 | 3547 | tags=35%, list=30%, signal=50% |
| 4584 | GOBP\_REGULATION\_OF\_RETINOIC\_ACID\_RECEPTOR\_SIGNALING\_PATHWAY |  | 10 | -0.44 | -1.04 | 0.427 | 0.723 | 1.000 | 5000 | tags=80%, list=42%, signal=137% |
| 4585 | GOBP\_REGULATION\_OF\_TRANSCRIPTION\_OF\_NUCLEOLAR\_LARGE\_RRNA\_BY\_RNA\_POLYMERASE\_I |  | 9 | -0.46 | -1.04 | 0.443 | 0.724 | 1.000 | 4465 | tags=44%, list=37%, signal=71% |
| 4586 | GOBP\_PROTEIN\_LOCALIZATION\_TO\_CILIARY\_MEMBRANE |  | 9 | -0.46 | -1.04 | 0.441 | 0.724 | 1.000 | 3889 | tags=44%, list=32%, signal=66% |
| 4587 | GOCC\_HOST\_CELLULAR\_COMPONENT |  | 49 | -0.31 | -1.04 | 0.426 | 0.724 | 1.000 | 5623 | tags=61%, list=47%, signal=115% |
| 4588 | GOBP\_POSITIVE\_REGULATION\_OF\_ADAPTIVE\_IMMUNE\_RESPONSE |  | 57 | -0.31 | -1.04 | 0.423 | 0.725 | 1.000 | 3240 | tags=37%, list=27%, signal=50% |
| 4589 | GOBP\_REGULATION\_OF\_DNA\_TOPOISOMERASE\_ATP\_HYDROLYZING\_ACTIVITY |  | 5 | -0.54 | -1.04 | 0.445 | 0.725 | 1.000 | 5518 | tags=80%, list=46%, signal=148% |
| 4590 | GOBP\_HISTONE\_DEUBIQUITINATION |  | 20 | -0.37 | -1.04 | 0.436 | 0.725 | 1.000 | 2719 | tags=25%, list=23%, signal=32% |
| 4591 | GOBP\_L\_ARGININE\_TRANSPORT |  | 7 | -0.49 | -1.04 | 0.449 | 0.725 | 1.000 | 2847 | tags=43%, list=24%, signal=56% |
| 4592 | GOMF\_ORGANIC\_ANION\_TRANSMEMBRANE\_TRANSPORTER\_ACTIVITY |  | 117 | -0.28 | -1.04 | 0.427 | 0.727 | 1.000 | 2392 | tags=27%, list=20%, signal=34% |
| 4593 | GOBP\_NEGATIVE\_REGULATION\_OF\_PEPTIDASE\_ACTIVITY |  | 122 | -0.28 | -1.04 | 0.419 | 0.727 | 1.000 | 3345 | tags=29%, list=28%, signal=39% |
| 4594 | GOBP\_CELLULAR\_GLUCOSE\_HOMEOSTASIS |  | 95 | -0.29 | -1.03 | 0.425 | 0.727 | 1.000 | 3978 | tags=40%, list=33%, signal=59% |
| 4595 | GOBP\_RIBONUCLEOSIDE\_MONOPHOSPHATE\_CATABOLIC\_PROCESS |  | 8 | -0.47 | -1.03 | 0.442 | 0.727 | 1.000 | 1107 | tags=38%, list=9%, signal=41% |
| 4596 | GOBP\_REGULATION\_OF\_KERATINOCYTE\_MIGRATION |  | 13 | -0.41 | -1.03 | 0.444 | 0.727 | 1.000 | 3392 | tags=69%, list=28%, signal=96% |
| 4597 | GOBP\_SPINDLE\_ELONGATION |  | 7 | -0.49 | -1.03 | 0.444 | 0.727 | 1.000 | 4165 | tags=57%, list=35%, signal=87% |
| 4598 | GOBP\_NEGATIVE\_REGULATION\_OF\_ERK1\_AND\_ERK2\_CASCADE |  | 50 | -0.31 | -1.03 | 0.425 | 0.728 | 1.000 | 5082 | tags=52%, list=42%, signal=90% |
| 4599 | GOBP\_PROTEIN\_QUALITY\_CONTROL\_FOR\_MISFOLDED\_OR\_INCOMPLETELY\_SYNTHESIZED\_PROTEINS |  | 22 | -0.37 | -1.03 | 0.432 | 0.728 | 1.000 | 2774 | tags=32%, list=23%, signal=41% |
| 4600 | GOBP\_NEGATIVE\_REGULATION\_OF\_CELL\_AGING |  | 21 | -0.37 | -1.03 | 0.441 | 0.728 | 1.000 | 3668 | tags=43%, list=31%, signal=62% |
| 4601 | GOMF\_LIPASE\_BINDING |  | 4 | -0.57 | -1.03 | 0.441 | 0.728 | 1.000 | 4853 | tags=75%, list=40%, signal=126% |
| 4602 | GOMF\_PROTEIN\_SERINE\_THREONINE\_KINASE\_INHIBITOR\_ACTIVITY |  | 22 | -0.37 | -1.03 | 0.436 | 0.728 | 1.000 | 784 | tags=18%, list=7%, signal=19% |
| 4603 | GOBP\_CARDIAC\_MUSCLE\_CELL\_CONTRACTION |  | 47 | -0.32 | -1.03 | 0.431 | 0.728 | 1.000 | 3918 | tags=36%, list=33%, signal=53% |
| 4604 | GOBP\_REGULATION\_OF\_CALCIUM\_MEDIATED\_SIGNALING |  | 45 | -0.32 | -1.03 | 0.433 | 0.728 | 1.000 | 4597 | tags=56%, list=38%, signal=90% |
| 4605 | GOBP\_NEGATIVE\_REGULATION\_OF\_CIRCADIAN\_RHYTHM |  | 10 | -0.44 | -1.03 | 0.448 | 0.729 | 1.000 | 3235 | tags=60%, list=27%, signal=82% |
| 4606 | GOBP\_POSITIVE\_REGULATION\_OF\_MACROPHAGE\_DERIVED\_FOAM\_CELL\_DIFFERENTIATION |  | 10 | -0.45 | -1.03 | 0.439 | 0.729 | 1.000 | 3217 | tags=60%, list=27%, signal=82% |
| 4607 | GOBP\_ACTIVIN\_RECEPTOR\_SIGNALING\_PATHWAY |  | 29 | -0.35 | -1.03 | 0.435 | 0.729 | 1.000 | 2578 | tags=31%, list=21%, signal=39% |
| 4608 | GOBP\_SYNAPTIC\_VESICLE\_DOCKING |  | 8 | -0.46 | -1.03 | 0.442 | 0.730 | 1.000 | 2024 | tags=63%, list=17%, signal=75% |
| 4609 | GOBP\_MUSCLE\_ORGAN\_MORPHOGENESIS |  | 39 | -0.32 | -1.03 | 0.434 | 0.730 | 1.000 | 3615 | tags=33%, list=30%, signal=48% |
| 4610 | GOBP\_NEGATIVE\_REGULATION\_OF\_SIGNAL\_TRANSDUCTION\_IN\_ABSENCE\_OF\_LIGAND |  | 19 | -0.38 | -1.03 | 0.434 | 0.730 | 1.000 | 1146 | tags=21%, list=10%, signal=23% |
| 4611 | GOBP\_POSITIVE\_REGULATION\_OF\_LEUKOCYTE\_MEDIATED\_IMMUNITY |  | 62 | -0.30 | -1.03 | 0.429 | 0.730 | 1.000 | 2639 | tags=29%, list=22%, signal=37% |
| 4612 | GOBP\_NEUROTRANSMITTER\_CATABOLIC\_PROCESS |  | 8 | -0.46 | -1.03 | 0.442 | 0.730 | 1.000 | 3792 | tags=63%, list=32%, signal=91% |
| 4613 | GOBP\_CELLULAR\_RESPONSE\_TO\_ZINC\_ION |  | 10 | -0.44 | -1.03 | 0.440 | 0.730 | 1.000 | 1583 | tags=30%, list=13%, signal=35% |
| 4614 | GOBP\_ORGANOPHOSPHATE\_BIOSYNTHETIC\_PROCESS |  | 421 | -0.26 | -1.03 | 0.393 | 0.730 | 1.000 | 4460 | tags=40%, list=37%, signal=61% |
| 4615 | GOBP\_NERVOUS\_SYSTEM\_PROCESS\_INVOLVED\_IN\_REGULATION\_OF\_SYSTEMIC\_ARTERIAL\_BLOOD\_PRESSURE |  | 5 | -0.53 | -1.03 | 0.444 | 0.730 | 1.000 | 1799 | tags=40%, list=15%, signal=47% |
| 4616 | GOBP\_MONOCARBOXYLIC\_ACID\_BIOSYNTHETIC\_PROCESS |  | 145 | -0.28 | -1.03 | 0.437 | 0.729 | 1.000 | 4190 | tags=41%, list=35%, signal=62% |
| 4617 | GOBP\_POSITIVE\_REGULATION\_OF\_BASEMENT\_MEMBRANE\_ASSEMBLY\_INVOLVED\_IN\_EMBRYONIC\_BODY\_MORPHOGENESIS |  | 5 | -0.54 | -1.03 | 0.446 | 0.730 | 1.000 | 3551 | tags=60%, list=30%, signal=85% |
| 4618 | GOBP\_AMINO\_ACID\_HOMEOSTASIS |  | 3 | -0.62 | -1.03 | 0.440 | 0.730 | 1.000 | 1222 | tags=33%, list=10%, signal=37% |
| 4619 | GOBP\_NEGATIVE\_REGULATION\_OF\_CD4\_POSITIVE\_ALPHA\_BETA\_T\_CELL\_DIFFERENTIATION |  | 16 | -0.39 | -1.03 | 0.436 | 0.730 | 1.000 | 1951 | tags=31%, list=16%, signal=37% |
| 4620 | GOBP\_SMAD\_PROTEIN\_COMPLEX\_ASSEMBLY |  | 9 | -0.45 | -1.03 | 0.436 | 0.730 | 1.000 | 3376 | tags=56%, list=28%, signal=77% |
| 4621 | GOCC\_CHROMOCENTER |  | 10 | -0.44 | -1.03 | 0.443 | 0.731 | 1.000 | 5805 | tags=70%, list=48%, signal=135% |
| 4622 | GOBP\_CELLULAR\_RESPONSE\_TO\_KETONE |  | 71 | -0.30 | -1.03 | 0.428 | 0.731 | 1.000 | 3147 | tags=35%, list=26%, signal=47% |
| 4623 | GOBP\_ESTABLISHMENT\_OF\_CENTROSOME\_LOCALIZATION |  | 8 | -0.47 | -1.03 | 0.455 | 0.731 | 1.000 | 5082 | tags=63%, list=42%, signal=108% |
| 4624 | GOBP\_INTRA\_S\_DNA\_DAMAGE\_CHECKPOINT |  | 12 | -0.42 | -1.03 | 0.435 | 0.731 | 1.000 | 4935 | tags=58%, list=41%, signal=99% |
| 4625 | GOBP\_VITAMIN\_TRANSPORT |  | 28 | -0.35 | -1.03 | 0.443 | 0.731 | 1.000 | 4059 | tags=57%, list=34%, signal=86% |
| 4626 | GOBP\_NEGATIVE\_REGULATION\_OF\_DNA\_BIOSYNTHETIC\_PROCESS |  | 28 | -0.35 | -1.03 | 0.435 | 0.731 | 1.000 | 2888 | tags=36%, list=24%, signal=47% |
| 4627 | GOBP\_CATECHOL\_CONTAINING\_COMPOUND\_CATABOLIC\_PROCESS |  | 5 | -0.54 | -1.03 | 0.452 | 0.732 | 1.000 | 5138 | tags=80%, list=43%, signal=140% |
| 4628 | GOCC\_HFE\_TRANSFERRIN\_RECEPTOR\_COMPLEX |  | 4 | -0.57 | -1.03 | 0.446 | 0.732 | 1.000 | 2886 | tags=50%, list=24%, signal=66% |
| 4629 | GOBP\_MEMBRANE\_DOCKING |  | 147 | -0.28 | -1.03 | 0.427 | 0.731 | 1.000 | 3476 | tags=31%, list=29%, signal=43% |
| 4630 | GOBP\_POSITIVE\_REGULATION\_OF\_ACTIVATED\_T\_CELL\_PROLIFERATION |  | 13 | -0.41 | -1.03 | 0.445 | 0.731 | 1.000 | 3066 | tags=46%, list=26%, signal=62% |
| 4631 | GOBP\_HEXOSE\_PHOSPHATE\_TRANSPORT |  | 4 | -0.57 | -1.03 | 0.447 | 0.732 | 1.000 | 1679 | tags=50%, list=14%, signal=58% |
| 4632 | GOBP\_RESPONSE\_TO\_OSMOTIC\_STRESS |  | 53 | -0.31 | -1.03 | 0.431 | 0.731 | 1.000 | 4353 | tags=47%, list=36%, signal=74% |
| 4633 | GOMF\_CENTROMERIC\_DNA\_BINDING |  | 5 | -0.54 | -1.03 | 0.448 | 0.731 | 1.000 | 4660 | tags=80%, list=39%, signal=131% |
| 4634 | GOBP\_POSITIVE\_REGULATION\_OF\_MUSCLE\_HYPERTROPHY |  | 21 | -0.37 | -1.03 | 0.437 | 0.731 | 1.000 | 3376 | tags=43%, list=28%, signal=60% |
| 4635 | GOMF\_RETINOID\_X\_RECEPTOR\_BINDING |  | 14 | -0.40 | -1.03 | 0.445 | 0.731 | 1.000 | 2732 | tags=43%, list=23%, signal=55% |
| 4636 | GOBP\_METANEPHRIC\_GLOMERULUS\_DEVELOPMENT |  | 11 | -0.43 | -1.03 | 0.442 | 0.731 | 1.000 | 3286 | tags=36%, list=27%, signal=50% |
| 4637 | GOBP\_INOSITOL\_PHOSPHATE\_MEDIATED\_SIGNALING |  | 37 | -0.33 | -1.03 | 0.440 | 0.731 | 1.000 | 4084 | tags=46%, list=34%, signal=69% |
| 4638 | GOBP\_REGULATION\_OF\_HIGH\_VOLTAGE\_GATED\_CALCIUM\_CHANNEL\_ACTIVITY |  | 10 | -0.44 | -1.03 | 0.451 | 0.731 | 1.000 | 276 | tags=10%, list=2%, signal=10% |
| 4639 | GOBP\_HISTONE\_H3\_K14\_ACETYLATION |  | 12 | -0.42 | -1.03 | 0.438 | 0.731 | 1.000 | 3411 | tags=50%, list=28%, signal=70% |
| 4640 | GOBP\_RESPONSE\_TO\_MOLECULE\_OF\_BACTERIAL\_ORIGIN |  | 201 | -0.27 | -1.03 | 0.421 | 0.731 | 1.000 | 3613 | tags=34%, list=30%, signal=48% |
| 4641 | GOBP\_PRESYNAPTIC\_MEMBRANE\_ORGANIZATION |  | 6 | -0.51 | -1.03 | 0.446 | 0.731 | 1.000 | 5232 | tags=83%, list=44%, signal=148% |
| 4642 | GOBP\_MYELOID\_CELL\_DEVELOPMENT |  | 46 | -0.32 | -1.03 | 0.435 | 0.731 | 1.000 | 2769 | tags=35%, list=23%, signal=45% |
| 4643 | GOBP\_NEGATIVE\_REGULATION\_OF\_LEUKOCYTE\_MIGRATION |  | 27 | -0.35 | -1.03 | 0.432 | 0.731 | 1.000 | 3695 | tags=41%, list=31%, signal=59% |
| 4644 | GOCC\_MLL3\_4\_COMPLEX |  | 7 | -0.49 | -1.03 | 0.446 | 0.731 | 1.000 | 2639 | tags=43%, list=22%, signal=55% |
| 4645 | GOBP\_HORMONE\_BIOSYNTHETIC\_PROCESS |  | 40 | -0.32 | -1.03 | 0.440 | 0.731 | 1.000 | 2666 | tags=33%, list=22%, signal=42% |
| 4646 | GOBP\_RESPONSE\_TO\_ISOQUINOLINE\_ALKALOID |  | 19 | -0.38 | -1.03 | 0.441 | 0.731 | 1.000 | 4521 | tags=63%, list=38%, signal=101% |
| 4647 | GOBP\_REGULATION\_OF\_AMPA\_RECEPTOR\_ACTIVITY |  | 6 | -0.50 | -1.03 | 0.434 | 0.731 | 1.000 | 6011 | tags=100%, list=50%, signal=200% |
| 4648 | GOMF\_PROTEIN\_KINASE\_C\_BINDING |  | 38 | -0.33 | -1.03 | 0.437 | 0.731 | 1.000 | 3613 | tags=34%, list=30%, signal=49% |
| 4649 | GOBP\_RETINA\_LAYER\_FORMATION |  | 15 | -0.40 | -1.03 | 0.439 | 0.732 | 1.000 | 3589 | tags=47%, list=30%, signal=66% |
| 4650 | GOMF\_OXIDOREDUCTASE\_ACTIVITY\_ACTING\_ON\_NAD\_P\_H\_OXYGEN\_AS\_ACCEPTOR |  | 9 | -0.45 | -1.03 | 0.450 | 0.732 | 1.000 | 5225 | tags=67%, list=44%, signal=118% |
| 4651 | GOBP\_POSITIVE\_REGULATION\_OF\_PROTEIN\_LOCALIZATION\_TO\_SYNAPSE |  | 7 | -0.49 | -1.03 | 0.446 | 0.732 | 1.000 | 1346 | tags=29%, list=11%, signal=32% |
| 4652 | GOBP\_POSITIVE\_REGULATION\_OF\_OXIDOREDUCTASE\_ACTIVITY |  | 40 | -0.32 | -1.03 | 0.437 | 0.732 | 1.000 | 4180 | tags=40%, list=35%, signal=61% |
| 4653 | GOBP\_PEROXISOMAL\_MEMBRANE\_TRANSPORT |  | 17 | -0.38 | -1.03 | 0.445 | 0.732 | 1.000 | 4321 | tags=35%, list=36%, signal=55% |
| 4654 | GOMF\_SUMO\_SPECIFIC\_PROTEASE\_ACTIVITY |  | 7 | -0.48 | -1.03 | 0.453 | 0.732 | 1.000 | 3762 | tags=57%, list=31%, signal=83% |
| 4655 | GOBP\_LEUKOTRIENE\_SIGNALING\_PATHWAY |  | 3 | -0.62 | -1.03 | 0.456 | 0.732 | 1.000 | 4593 | tags=100%, list=38%, signal=162% |
| 4656 | GOBP\_PROTEOGLYCAN\_BIOSYNTHETIC\_PROCESS |  | 46 | -0.32 | -1.03 | 0.441 | 0.732 | 1.000 | 4102 | tags=46%, list=34%, signal=69% |
| 4657 | GOBP\_ENDOPLASMIC\_RETICULUM\_TO\_CYTOSOL\_TRANSPORT |  | 24 | -0.36 | -1.03 | 0.437 | 0.732 | 1.000 | 2774 | tags=29%, list=23%, signal=38% |
| 4658 | GOBP\_NEGATIVE\_REGULATION\_OF\_BINDING |  | 100 | -0.29 | -1.03 | 0.437 | 0.732 | 1.000 | 2308 | tags=21%, list=19%, signal=26% |
| 4659 | GOBP\_REGULATION\_OF\_SEQUESTERING\_OF\_TRIGLYCERIDE |  | 9 | -0.45 | -1.03 | 0.444 | 0.732 | 1.000 | 2009 | tags=44%, list=17%, signal=53% |
| 4660 | GOBP\_REGULATION\_OF\_SYNAPTIC\_TRANSMISSION\_GABAERGIC |  | 17 | -0.38 | -1.03 | 0.453 | 0.732 | 1.000 | 4907 | tags=65%, list=41%, signal=109% |
| 4661 | GOBP\_PROTEIN\_LOCALIZATION\_TO\_CILIARY\_TRANSITION\_ZONE |  | 6 | -0.51 | -1.03 | 0.446 | 0.732 | 1.000 | 5444 | tags=83%, list=45%, signal=152% |
| 4662 | GOBP\_INNATE\_IMMUNE\_RESPONSE |  | 435 | -0.26 | -1.03 | 0.410 | 0.733 | 1.000 | 3166 | tags=30%, list=26%, signal=40% |
| 4663 | GOBP\_NEGATIVE\_REGULATION\_OF\_ERBB\_SIGNALING\_PATHWAY |  | 42 | -0.32 | -1.03 | 0.442 | 0.733 | 1.000 | 2354 | tags=31%, list=20%, signal=38% |
| 4664 | GOBP\_BRANCHING\_MORPHOGENESIS\_OF\_A\_NERVE |  | 7 | -0.49 | -1.03 | 0.442 | 0.733 | 1.000 | 3483 | tags=57%, list=29%, signal=80% |
| 4665 | GOCC\_CILIUM |  | 346 | -0.26 | -1.03 | 0.421 | 0.733 | 1.000 | 4190 | tags=38%, list=35%, signal=56% |
| 4666 | GOMF\_G\_PROTEIN\_COUPLED\_GLUTAMATE\_RECEPTOR\_BINDING |  | 10 | -0.44 | -1.03 | 0.445 | 0.733 | 1.000 | 1495 | tags=30%, list=12%, signal=34% |
| 4667 | GOBP\_RESPONSE\_TO\_AMINO\_ACID\_STARVATION |  | 43 | -0.32 | -1.03 | 0.435 | 0.733 | 1.000 | 3872 | tags=35%, list=32%, signal=51% |
| 4668 | GOBP\_MEMBRANE\_DEPOLARIZATION\_DURING\_CARDIAC\_MUSCLE\_CELL\_ACTION\_POTENTIAL |  | 13 | -0.41 | -1.03 | 0.449 | 0.733 | 1.000 | 748 | tags=15%, list=6%, signal=16% |
| 4669 | GOBP\_ACYL\_CARNITINE\_TRANSPORT |  | 3 | -0.61 | -1.03 | 0.459 | 0.733 | 1.000 | 2469 | tags=67%, list=21%, signal=84% |
| 4670 | GOBP\_NEUTROPHIL\_MEDIATED\_KILLING\_OF\_GRAM\_NEGATIVE\_BACTERIUM |  | 3 | -0.62 | -1.03 | 0.444 | 0.733 | 1.000 | 4228 | tags=67%, list=35%, signal=103% |
| 4671 | GOMF\_1\_PHOSPHATIDYLINOSITOL\_BINDING |  | 14 | -0.40 | -1.03 | 0.436 | 0.734 | 1.000 | 3552 | tags=57%, list=30%, signal=81% |
| 4672 | GOMF\_APOLIPOPROTEIN\_BINDING |  | 14 | -0.40 | -1.03 | 0.446 | 0.734 | 1.000 | 3007 | tags=43%, list=25%, signal=57% |
| 4673 | GOBP\_RESPONSE\_TO\_INDOLE\_3\_METHANOL |  | 5 | -0.53 | -1.03 | 0.450 | 0.734 | 1.000 | 5645 | tags=100%, list=47%, signal=189% |
| 4674 | GOMF\_PROTEIN\_HETERODIMERIZATION\_ACTIVITY |  | 180 | -0.27 | -1.03 | 0.434 | 0.735 | 1.000 | 3673 | tags=29%, list=31%, signal=41% |
| 4675 | GOMF\_HISTONE\_DEACETYLASE\_ACTIVITY\_H3\_K14\_SPECIFIC |  | 8 | -0.46 | -1.03 | 0.449 | 0.735 | 1.000 | 88 | tags=13%, list=1%, signal=13% |
| 4676 | GOBP\_REGULATION\_OF\_UBIQUITIN\_SPECIFIC\_PROTEASE\_ACTIVITY |  | 4 | -0.57 | -1.03 | 0.457 | 0.735 | 1.000 | 4909 | tags=75%, list=41%, signal=127% |
| 4677 | GOBP\_CELL\_PROLIFERATION\_IN\_HINDBRAIN |  | 10 | -0.44 | -1.03 | 0.453 | 0.735 | 1.000 | 3565 | tags=40%, list=30%, signal=57% |
| 4678 | GOBP\_BERGMANN\_GLIAL\_CELL\_DIFFERENTIATION |  | 6 | -0.50 | -1.03 | 0.447 | 0.734 | 1.000 | 3565 | tags=50%, list=30%, signal=71% |
| 4679 | GOBP\_RESPONSE\_TO\_MANGANESE\_ION |  | 14 | -0.41 | -1.03 | 0.445 | 0.735 | 1.000 | 4814 | tags=57%, list=40%, signal=95% |
| 4680 | GOCC\_HEMIDESMOSOME |  | 4 | -0.57 | -1.03 | 0.452 | 0.736 | 1.000 | 3856 | tags=75%, list=32%, signal=110% |
| 4681 | GOMF\_GTPASE\_ACTIVATING\_PROTEIN\_BINDING |  | 12 | -0.41 | -1.03 | 0.453 | 0.736 | 1.000 | 4292 | tags=50%, list=36%, signal=78% |
| 4682 | GOBP\_POSITIVE\_REGULATION\_OF\_CD4\_POSITIVE\_ALPHA\_BETA\_T\_CELL\_PROLIFERATION |  | 4 | -0.57 | -1.03 | 0.455 | 0.736 | 1.000 | 429 | tags=25%, list=4%, signal=26% |
| 4683 | GOBP\_POSITIVE\_REGULATION\_OF\_CELL\_AGING |  | 10 | -0.44 | -1.03 | 0.448 | 0.736 | 1.000 | 3568 | tags=50%, list=30%, signal=71% |
| 4684 | GOMF\_PYRIMIDINE\_NUCLEOTIDE\_BINDING |  | 4 | -0.57 | -1.03 | 0.457 | 0.736 | 1.000 | 4973 | tags=75%, list=41%, signal=128% |
| 4685 | GOBP\_CELLULAR\_RESPONSE\_TO\_MAGNESIUM\_ION |  | 4 | -0.57 | -1.03 | 0.452 | 0.736 | 1.000 | 5191 | tags=100%, list=43%, signal=176% |
| 4686 | GOBP\_NEGATIVE\_REGULATION\_OF\_CARDIAC\_MUSCLE\_CONTRACTION |  | 4 | -0.57 | -1.03 | 0.455 | 0.736 | 1.000 | 3968 | tags=75%, list=33%, signal=112% |
| 4687 | GOBP\_NON\_CANONICAL\_WNT\_SIGNALING\_PATHWAY |  | 115 | -0.28 | -1.03 | 0.433 | 0.736 | 1.000 | 2174 | tags=18%, list=18%, signal=22% |
| 4688 | GOBP\_CYTOKINESIS |  | 125 | -0.28 | -1.03 | 0.447 | 0.736 | 1.000 | 4165 | tags=36%, list=35%, signal=55% |
| 4689 | GOBP\_DORSAL\_VENTRAL\_PATTERN\_FORMATION |  | 43 | -0.32 | -1.03 | 0.443 | 0.736 | 1.000 | 1871 | tags=21%, list=16%, signal=25% |
| 4690 | GOBP\_MEIOTIC\_CELL\_CYCLE\_PHASE\_TRANSITION |  | 5 | -0.53 | -1.03 | 0.461 | 0.736 | 1.000 | 5604 | tags=100%, list=47%, signal=187% |
| 4691 | GOBP\_RESPONSE\_TO\_ENDOPLASMIC\_RETICULUM\_STRESS |  | 224 | -0.27 | -1.03 | 0.433 | 0.737 | 1.000 | 5958 | tags=54%, list=50%, signal=105% |
| 4692 | GOBP\_POSITIVE\_REGULATION\_OF\_T\_CELL\_MEDIATED\_IMMUNITY |  | 24 | -0.35 | -1.03 | 0.443 | 0.736 | 1.000 | 1781 | tags=29%, list=15%, signal=34% |
| 4693 | GOCC\_HOST\_CELL\_PART |  | 3 | -0.61 | -1.03 | 0.457 | 0.738 | 1.000 | 2227 | tags=67%, list=19%, signal=82% |
| 4694 | GOBP\_REGULATION\_OF\_AUTOPHAGOSOME\_ASSEMBLY |  | 31 | -0.34 | -1.03 | 0.446 | 0.738 | 1.000 | 3703 | tags=29%, list=31%, signal=42% |
| 4695 | GOMF\_TUMOR\_NECROSIS\_FACTOR\_BINDING |  | 3 | -0.61 | -1.03 | 0.456 | 0.738 | 1.000 | 1781 | tags=33%, list=15%, signal=39% |
| 4696 | GOBP\_MYELOID\_DENDRITIC\_CELL\_CYTOKINE\_PRODUCTION |  | 4 | -0.57 | -1.03 | 0.460 | 0.738 | 1.000 | 4686 | tags=75%, list=39%, signal=123% |
| 4697 | GOBP\_NEGATIVE\_REGULATION\_OF\_STRIATED\_MUSCLE\_CELL\_DIFFERENTIATION |  | 21 | -0.37 | -1.02 | 0.440 | 0.738 | 1.000 | 4139 | tags=52%, list=34%, signal=80% |
| 4698 | GOBP\_RESPONSE\_TO\_CELL\_CYCLE\_CHECKPOINT\_SIGNALING |  | 6 | -0.50 | -1.02 | 0.460 | 0.738 | 1.000 | 3325 | tags=50%, list=28%, signal=69% |
| 4699 | GOCC\_SECRETORY\_GRANULE |  | 512 | -0.26 | -1.02 | 0.413 | 0.738 | 1.000 | 3510 | tags=29%, list=29%, signal=40% |
| 4700 | GOBP\_CHROMOSOME\_LOCALIZATION |  | 64 | -0.30 | -1.02 | 0.444 | 0.738 | 1.000 | 4126 | tags=36%, list=34%, signal=54% |
| 4701 | GOCC\_CIS\_GOLGI\_NETWORK |  | 36 | -0.33 | -1.02 | 0.450 | 0.739 | 1.000 | 5705 | tags=61%, list=47%, signal=116% |
| 4702 | GOBP\_DNA\_LIGATION |  | 14 | -0.40 | -1.02 | 0.453 | 0.739 | 1.000 | 4773 | tags=64%, list=40%, signal=107% |
| 4703 | GOBP\_CILIUM\_OR\_FLAGELLUM\_DEPENDENT\_CELL\_MOTILITY |  | 53 | -0.31 | -1.02 | 0.458 | 0.739 | 1.000 | 3612 | tags=38%, list=30%, signal=54% |
| 4704 | GOBP\_REGULATION\_OF\_CELL\_COMMUNICATION\_BY\_ELECTRICAL\_COUPLING\_INVOLVED\_IN\_CARDIAC\_CONDUCTION |  | 6 | -0.51 | -1.02 | 0.460 | 0.739 | 1.000 | 1304 | tags=33%, list=11%, signal=37% |
| 4705 | GOBP\_RENAL\_TUBULE\_DEVELOPMENT |  | 61 | -0.30 | -1.02 | 0.444 | 0.740 | 1.000 | 3196 | tags=34%, list=27%, signal=47% |
| 4706 | GOBP\_ADENYLATE\_CYCLASE\_INHIBITING\_G\_PROTEIN\_COUPLED\_RECEPTOR\_SIGNALING\_PATHWAY |  | 42 | -0.32 | -1.02 | 0.448 | 0.740 | 1.000 | 1392 | tags=19%, list=12%, signal=21% |
| 4707 | GOMF\_ASPARTIC\_ENDOPEPTIDASE\_ACTIVITY\_INTRAMEMBRANE\_CLEAVING |  | 6 | -0.50 | -1.02 | 0.456 | 0.740 | 1.000 | 1618 | tags=33%, list=13%, signal=39% |
| 4708 | GOBP\_POSITIVE\_REGULATION\_OF\_OSSIFICATION |  | 31 | -0.34 | -1.02 | 0.446 | 0.740 | 1.000 | 3269 | tags=42%, list=27%, signal=57% |
| 4709 | GOBP\_DETECTION\_OF\_EXTERNAL\_BIOTIC\_STIMULUS |  | 8 | -0.47 | -1.02 | 0.448 | 0.740 | 1.000 | 1230 | tags=25%, list=10%, signal=28% |
| 4710 | GOBP\_POSITIVE\_REGULATION\_OF\_BLOOD\_VESSEL\_ENDOTHELIAL\_CELL\_PROLIFERATION\_INVOLVED\_IN\_SPROUTING\_ANGIOGENESIS |  | 7 | -0.48 | -1.02 | 0.457 | 0.740 | 1.000 | 4490 | tags=71%, list=37%, signal=114% |
| 4711 | GOBP\_NEGATIVE\_REGULATION\_OF\_NLRP3\_INFLAMMASOME\_COMPLEX\_ASSEMBLY |  | 3 | -0.61 | -1.02 | 0.452 | 0.740 | 1.000 | 673 | tags=33%, list=6%, signal=35% |
| 4712 | GOBP\_MULTI\_ORGANISM\_PROCESS |  | 578 | -0.26 | -1.02 | 0.409 | 0.740 | 1.000 | 4882 | tags=45%, list=41%, signal=72% |
| 4713 | GOBP\_POST\_TRANSLATIONAL\_PROTEIN\_MODIFICATION |  | 269 | -0.27 | -1.02 | 0.434 | 0.740 | 1.000 | 3958 | tags=32%, list=33%, signal=47% |
| 4714 | GOBP\_UDP\_GLUCOSE\_METABOLIC\_PROCESS |  | 5 | -0.53 | -1.02 | 0.461 | 0.740 | 1.000 | 4825 | tags=80%, list=40%, signal=134% |
| 4715 | GOBP\_REGULATION\_OF\_PHOSPHOLIPID\_TRANSLOCATION |  | 5 | -0.53 | -1.02 | 0.458 | 0.740 | 1.000 | 5062 | tags=80%, list=42%, signal=138% |
| 4716 | GOBP\_GLOBAL\_GENOME\_NUCLEOTIDE\_EXCISION\_REPAIR |  | 22 | -0.36 | -1.02 | 0.456 | 0.740 | 1.000 | 5370 | tags=64%, list=45%, signal=115% |
| 4717 | GOBP\_RESPONSE\_TO\_LAMINAR\_FLUID\_SHEAR\_STRESS |  | 11 | -0.43 | -1.02 | 0.445 | 0.740 | 1.000 | 3403 | tags=45%, list=28%, signal=63% |
| 4718 | GOBP\_REGULATION\_OF\_PROGRAMMED\_NECROTIC\_CELL\_DEATH |  | 14 | -0.40 | -1.02 | 0.442 | 0.740 | 1.000 | 1084 | tags=21%, list=9%, signal=24% |
| 4719 | GOBP\_NEGATIVE\_REGULATION\_OF\_WNT\_SIGNALING\_PATHWAY |  | 151 | -0.27 | -1.02 | 0.448 | 0.740 | 1.000 | 3565 | tags=30%, list=30%, signal=42% |
| 4720 | GOBP\_POSITIVE\_REGULATION\_OF\_CELLULAR\_PROTEIN\_CATABOLIC\_PROCESS |  | 110 | -0.28 | -1.02 | 0.453 | 0.740 | 1.000 | 3036 | tags=25%, list=25%, signal=34% |
| 4721 | GOMF\_COBALAMIN\_BINDING |  | 6 | -0.50 | -1.02 | 0.464 | 0.740 | 1.000 | 5955 | tags=100%, list=50%, signal=198% |
| 4722 | GOBP\_SIGNAL\_COMPLEX\_ASSEMBLY |  | 5 | -0.53 | -1.02 | 0.462 | 0.740 | 1.000 | 5607 | tags=100%, list=47%, signal=187% |
| 4723 | GOBP\_POSITIVE\_REGULATION\_OF\_DENDRITIC\_CELL\_CYTOKINE\_PRODUCTION |  | 4 | -0.57 | -1.02 | 0.471 | 0.740 | 1.000 | 4686 | tags=75%, list=39%, signal=123% |
| 4724 | GOMF\_RETINOIC\_ACID\_RECEPTOR\_BINDING |  | 21 | -0.37 | -1.02 | 0.461 | 0.740 | 1.000 | 4455 | tags=57%, list=37%, signal=91% |
| 4725 | GOBP\_COCHLEA\_DEVELOPMENT |  | 28 | -0.34 | -1.02 | 0.455 | 0.741 | 1.000 | 3433 | tags=39%, list=29%, signal=55% |
| 4726 | GOCC\_SHELTERIN\_COMPLEX |  | 5 | -0.53 | -1.02 | 0.460 | 0.740 | 1.000 | 5648 | tags=100%, list=47%, signal=189% |
| 4727 | GOBP\_POSITIVE\_REGULATION\_OF\_CELL\_MIGRATION\_INVOLVED\_IN\_SPROUTING\_ANGIOGENESIS |  | 15 | -0.39 | -1.02 | 0.453 | 0.741 | 1.000 | 3196 | tags=47%, list=27%, signal=64% |
| 4728 | GOBP\_RESPONSE\_TO\_LIPID |  | 545 | -0.26 | -1.02 | 0.418 | 0.741 | 1.000 | 2871 | tags=27%, list=24%, signal=34% |
| 4729 | GOBP\_DEVELOPMENTAL\_PIGMENTATION |  | 30 | -0.34 | -1.02 | 0.454 | 0.741 | 1.000 | 2911 | tags=40%, list=24%, signal=53% |
| 4730 | GOBP\_CELLULAR\_RESPONSE\_TO\_OXYGEN\_CONTAINING\_COMPOUND |  | 779 | -0.26 | -1.02 | 0.403 | 0.741 | 1.000 | 3978 | tags=36%, list=33%, signal=50% |
| 4731 | GOBP\_REGULATION\_OF\_PROTEIN\_CATABOLIC\_PROCESS |  | 295 | -0.26 | -1.02 | 0.442 | 0.741 | 1.000 | 3988 | tags=33%, list=33%, signal=48% |
| 4732 | GOBP\_NEGATIVE\_REGULATION\_OF\_CHROMATIN\_ASSEMBLY\_OR\_DISASSEMBLY |  | 3 | -0.61 | -1.02 | 0.470 | 0.741 | 1.000 | 268 | tags=33%, list=2%, signal=34% |
| 4733 | GOBP\_RESPONSE\_TO\_ALUMINUM\_ION |  | 5 | -0.53 | -1.02 | 0.452 | 0.741 | 1.000 | 897 | tags=40%, list=7%, signal=43% |
| 4734 | GOBP\_PROTEIN\_LOCALIZATION\_TO\_ENDOPLASMIC\_RETICULUM\_EXIT\_SITE |  | 7 | -0.48 | -1.02 | 0.462 | 0.741 | 1.000 | 3483 | tags=57%, list=29%, signal=80% |
| 4735 | GOBP\_EXOCYTOSIS |  | 601 | -0.26 | -1.02 | 0.413 | 0.741 | 1.000 | 3571 | tags=30%, list=30%, signal=41% |
| 4736 | GOBP\_NEGATIVE\_REGULATION\_OF\_T\_HELPER\_1\_TYPE\_IMMUNE\_RESPONSE |  | 4 | -0.56 | -1.02 | 0.457 | 0.742 | 1.000 | 2538 | tags=50%, list=21%, signal=63% |
| 4737 | GOBP\_NEGATIVE\_REGULATION\_OF\_DNA\_BINDING |  | 36 | -0.33 | -1.02 | 0.458 | 0.742 | 1.000 | 2281 | tags=25%, list=19%, signal=31% |
| 4738 | GOCC\_CYTOPLASMIC\_REGION |  | 158 | -0.27 | -1.02 | 0.448 | 0.742 | 1.000 | 4097 | tags=40%, list=34%, signal=60% |
| 4739 | GOBP\_HEART\_PROCESS |  | 169 | -0.27 | -1.02 | 0.454 | 0.742 | 1.000 | 4164 | tags=36%, list=35%, signal=54% |
| 4740 | GOBP\_SYNAPTIC\_VESICLE\_CYTOSKELETAL\_TRANSPORT |  | 16 | -0.39 | -1.02 | 0.455 | 0.742 | 1.000 | 3569 | tags=44%, list=30%, signal=62% |
| 4741 | GOBP\_POSITIVE\_REGULATION\_OF\_WNT\_SIGNALING\_PATHWAY\_PLANAR\_CELL\_POLARITY\_PATHWAY |  | 7 | -0.48 | -1.02 | 0.459 | 0.742 | 1.000 | 3941 | tags=57%, list=33%, signal=85% |
| 4742 | GOBP\_METHYLATION |  | 245 | -0.27 | -1.02 | 0.441 | 0.742 | 1.000 | 4420 | tags=40%, list=37%, signal=61% |
| 4743 | GOBP\_CELLULAR\_RESPONSE\_TO\_INSULIN\_LIKE\_GROWTH\_FACTOR\_STIMULUS |  | 7 | -0.49 | -1.02 | 0.468 | 0.743 | 1.000 | 3392 | tags=43%, list=28%, signal=60% |
| 4744 | GOMF\_IRON\_ION\_TRANSMEMBRANE\_TRANSPORTER\_ACTIVITY |  | 10 | -0.44 | -1.02 | 0.457 | 0.743 | 1.000 | 3141 | tags=40%, list=26%, signal=54% |
| 4745 | GOBP\_NEGATIVE\_REGULATION\_OF\_HISTONE\_H4\_ACETYLATION |  | 5 | -0.53 | -1.02 | 0.461 | 0.743 | 1.000 | 4561 | tags=80%, list=38%, signal=129% |
| 4746 | GOBP\_NATURAL\_KILLER\_CELL\_DEGRANULATION |  | 5 | -0.53 | -1.02 | 0.456 | 0.743 | 1.000 | 1825 | tags=20%, list=15%, signal=24% |
| 4747 | GOBP\_LEUKOTRIENE\_METABOLIC\_PROCESS |  | 17 | -0.38 | -1.02 | 0.457 | 0.743 | 1.000 | 2842 | tags=47%, list=24%, signal=62% |
| 4748 | GOBP\_MYELOID\_LEUKOCYTE\_MEDIATED\_IMMUNITY |  | 357 | -0.26 | -1.02 | 0.439 | 0.743 | 1.000 | 4424 | tags=39%, list=37%, signal=60% |
| 4749 | GOBP\_EGG\_ACTIVATION |  | 3 | -0.61 | -1.02 | 0.468 | 0.743 | 1.000 | 4695 | tags=100%, list=39%, signal=164% |
| 4750 | GOBP\_TUBULIN\_COMPLEX\_ASSEMBLY |  | 7 | -0.48 | -1.02 | 0.461 | 0.743 | 1.000 | 1915 | tags=14%, list=16%, signal=17% |
| 4751 | GOMF\_PHOSPHATIDYL\_PHOSPHOLIPASE\_B\_ACTIVITY |  | 8 | -0.46 | -1.02 | 0.460 | 0.743 | 1.000 | 4905 | tags=63%, list=41%, signal=106% |
| 4752 | GOBP\_NEGATIVE\_REGULATION\_OF\_HISTONE\_MODIFICATION |  | 31 | -0.33 | -1.02 | 0.451 | 0.743 | 1.000 | 4561 | tags=55%, list=38%, signal=88% |
| 4753 | GOBP\_CELLULAR\_ALDEHYDE\_METABOLIC\_PROCESS |  | 47 | -0.31 | -1.02 | 0.454 | 0.743 | 1.000 | 3644 | tags=30%, list=30%, signal=43% |
| 4754 | GOBP\_NEGATIVE\_REGULATION\_OF\_CELL\_SUBSTRATE\_JUNCTION\_ORGANIZATION |  | 16 | -0.39 | -1.02 | 0.455 | 0.743 | 1.000 | 6049 | tags=75%, list=50%, signal=151% |
| 4755 | GOBP\_ALDITOL\_PHOSPHATE\_METABOLIC\_PROCESS |  | 9 | -0.45 | -1.02 | 0.468 | 0.743 | 1.000 | 2362 | tags=22%, list=20%, signal=28% |
| 4756 | GOMF\_DNA\_TOPOISOMERASE\_BINDING |  | 5 | -0.53 | -1.02 | 0.461 | 0.743 | 1.000 | 4309 | tags=40%, list=36%, signal=62% |
| 4757 | GOBP\_GERM\_CELL\_DEVELOPMENT |  | 129 | -0.28 | -1.02 | 0.450 | 0.744 | 1.000 | 4519 | tags=44%, list=38%, signal=70% |
| 4758 | GOBP\_MITOTIC\_SPINDLE\_ASSEMBLY |  | 54 | -0.30 | -1.02 | 0.445 | 0.745 | 1.000 | 4716 | tags=43%, list=39%, signal=70% |
| 4759 | GOBP\_REGULATION\_OF\_LONG\_CHAIN\_FATTY\_ACID\_IMPORT\_ACROSS\_PLASMA\_MEMBRANE |  | 5 | -0.53 | -1.02 | 0.465 | 0.745 | 1.000 | 5145 | tags=80%, list=43%, signal=140% |
| 4760 | GOBP\_RESPONSE\_TO\_NITROGEN\_COMPOUND |  | 741 | -0.26 | -1.02 | 0.421 | 0.745 | 1.000 | 2879 | tags=27%, list=24%, signal=34% |
| 4761 | GOBP\_REGULATION\_OF\_LIPASE\_ACTIVITY |  | 65 | -0.30 | -1.02 | 0.458 | 0.745 | 1.000 | 2747 | tags=28%, list=23%, signal=36% |
| 4762 | GOBP\_NEGATIVE\_REGULATION\_OF\_IRE1\_MEDIATED\_UNFOLDED\_PROTEIN\_RESPONSE |  | 5 | -0.53 | -1.02 | 0.466 | 0.745 | 1.000 | 5279 | tags=80%, list=44%, signal=143% |
| 4763 | GOBP\_LABYRINTHINE\_LAYER\_MORPHOGENESIS |  | 12 | -0.41 | -1.02 | 0.465 | 0.745 | 1.000 | 5693 | tags=75%, list=47%, signal=142% |
| 4764 | GOBP\_NEGATIVE\_REGULATION\_OF\_CELL\_CYCLE\_ARREST |  | 16 | -0.39 | -1.02 | 0.461 | 0.745 | 1.000 | 5158 | tags=63%, list=43%, signal=109% |
| 4765 | GOBP\_DENTINOGENESIS |  | 3 | -0.61 | -1.02 | 0.485 | 0.745 | 1.000 | 4433 | tags=67%, list=37%, signal=106% |
| 4766 | GOCC\_GOLGI\_CIS\_CISTERNA |  | 8 | -0.46 | -1.02 | 0.458 | 0.745 | 1.000 | 2113 | tags=25%, list=18%, signal=30% |
| 4767 | GOBP\_RHYTHMIC\_BEHAVIOR |  | 26 | -0.35 | -1.02 | 0.457 | 0.745 | 1.000 | 3526 | tags=42%, list=29%, signal=60% |
| 4768 | GOBP\_REGULATION\_OF\_SYSTEMIC\_ARTERIAL\_BLOOD\_PRESSURE |  | 55 | -0.30 | -1.02 | 0.452 | 0.746 | 1.000 | 2480 | tags=25%, list=21%, signal=32% |
| 4769 | GOBP\_NEGATIVE\_REGULATION\_OF\_CELLULAR\_CATABOLIC\_PROCESS |  | 182 | -0.27 | -1.02 | 0.464 | 0.746 | 1.000 | 2518 | tags=22%, list=21%, signal=27% |
| 4770 | GOBP\_AXON\_MIDLINE\_CHOICE\_POINT\_RECOGNITION |  | 3 | -0.61 | -1.02 | 0.465 | 0.746 | 1.000 | 4696 | tags=67%, list=39%, signal=109% |
| 4771 | GOBP\_INSULIN\_SECRETION\_INVOLVED\_IN\_CELLULAR\_RESPONSE\_TO\_GLUCOSE\_STIMULUS |  | 42 | -0.32 | -1.02 | 0.449 | 0.746 | 1.000 | 1466 | tags=21%, list=12%, signal=24% |
| 4772 | GOBP\_GABAERGIC\_NEURON\_DIFFERENTIATION |  | 6 | -0.50 | -1.02 | 0.458 | 0.746 | 1.000 | 2563 | tags=33%, list=21%, signal=42% |
| 4773 | GOBP\_RETROGRADE\_AXONAL\_TRANSPORT |  | 16 | -0.39 | -1.02 | 0.458 | 0.747 | 1.000 | 1047 | tags=19%, list=9%, signal=21% |
| 4774 | GOBP\_POSITIVE\_REGULATION\_OF\_NEUROINFLAMMATORY\_RESPONSE |  | 7 | -0.48 | -1.02 | 0.456 | 0.748 | 1.000 | 4995 | tags=86%, list=42%, signal=147% |
| 4775 | GOBP\_REGULATION\_OF\_DEOXYRIBONUCLEASE\_ACTIVITY |  | 10 | -0.43 | -1.02 | 0.466 | 0.748 | 1.000 | 4649 | tags=50%, list=39%, signal=82% |
| 4776 | GOBP\_FAT\_SOLUBLE\_VITAMIN\_METABOLIC\_PROCESS |  | 29 | -0.34 | -1.02 | 0.457 | 0.748 | 1.000 | 3525 | tags=45%, list=29%, signal=63% |
| 4777 | GOCC\_BASEMENT\_MEMBRANE\_COLLAGEN\_TRIMER |  | 5 | -0.53 | -1.02 | 0.462 | 0.748 | 1.000 | 2333 | tags=60%, list=19%, signal=74% |
| 4778 | GOBP\_CELL\_FATE\_SPECIFICATION\_INVOLVED\_IN\_PATTERN\_SPECIFICATION |  | 3 | -0.61 | -1.02 | 0.471 | 0.748 | 1.000 | 556 | tags=33%, list=5%, signal=35% |
| 4779 | GOBP\_SECRETION\_BY\_TISSUE |  | 16 | -0.39 | -1.02 | 0.466 | 0.748 | 1.000 | 3553 | tags=50%, list=30%, signal=71% |
| 4780 | GOBP\_POSITIVE\_REGULATION\_OF\_MEMBRANE\_PROTEIN\_ECTODOMAIN\_PROTEOLYSIS |  | 11 | -0.42 | -1.02 | 0.466 | 0.748 | 1.000 | 1913 | tags=18%, list=16%, signal=22% |
| 4781 | GOBP\_SNARE\_COMPLEX\_ASSEMBLY |  | 14 | -0.40 | -1.02 | 0.458 | 0.749 | 1.000 | 3815 | tags=50%, list=32%, signal=73% |
| 4782 | GOBP\_SEXUAL\_REPRODUCTION |  | 419 | -0.26 | -1.02 | 0.446 | 0.749 | 1.000 | 4662 | tags=43%, list=39%, signal=69% |
| 4783 | GOMF\_CADMIUM\_ION\_BINDING |  | 3 | -0.61 | -1.02 | 0.473 | 0.749 | 1.000 | 1292 | tags=33%, list=11%, signal=37% |
| 4784 | GOMF\_GLUCURONOSYL\_N\_ACETYLGALACTOSAMINYL\_PROTEOGLYCAN\_4\_BETA\_N\_ACETYLGALACTOSAMINYLTRANSFERASE\_ACTIVITY |  | 6 | -0.50 | -1.02 | 0.467 | 0.750 | 1.000 | 2028 | tags=33%, list=17%, signal=40% |
| 4785 | GOBP\_LACTATE\_METABOLIC\_PROCESS |  | 11 | -0.42 | -1.02 | 0.467 | 0.749 | 1.000 | 320 | tags=9%, list=3%, signal=9% |
| 4786 | GOBP\_POSITIVE\_REGULATION\_OF\_SMAD\_PROTEIN\_SIGNAL\_TRANSDUCTION |  | 11 | -0.42 | -1.02 | 0.455 | 0.749 | 1.000 | 4770 | tags=64%, list=40%, signal=105% |
| 4787 | GOBP\_RESPONSE\_TO\_ORGANIC\_CYCLIC\_COMPOUND |  | 588 | -0.26 | -1.02 | 0.441 | 0.750 | 1.000 | 4217 | tags=39%, list=35%, signal=57% |
| 4788 | GOMF\_TRANSPORTER\_ACTIVITY |  | 669 | -0.26 | -1.02 | 0.442 | 0.750 | 1.000 | 3318 | tags=31%, list=28%, signal=41% |
| 4789 | GOBP\_INTRACELLULAR\_DISTRIBUTION\_OF\_MITOCHONDRIA |  | 8 | -0.46 | -1.02 | 0.474 | 0.750 | 1.000 | 5560 | tags=75%, list=46%, signal=140% |
| 4790 | GOBP\_POSITIVE\_REGULATION\_OF\_CELLULAR\_AMIDE\_METABOLIC\_PROCESS |  | 112 | -0.28 | -1.02 | 0.465 | 0.750 | 1.000 | 4629 | tags=37%, list=39%, signal=59% |
| 4791 | GOBP\_CELL\_MOTILITY\_INVOLVED\_IN\_CEREBRAL\_CORTEX\_RADIAL\_GLIA\_GUIDED\_MIGRATION |  | 4 | -0.56 | -1.02 | 0.469 | 0.750 | 1.000 | 4456 | tags=75%, list=37%, signal=119% |
| 4792 | GOMF\_STEROL\_TRANSFER\_ACTIVITY |  | 17 | -0.38 | -1.01 | 0.460 | 0.750 | 1.000 | 3600 | tags=47%, list=30%, signal=67% |
| 4793 | GOBP\_PROTEIN\_DESUMOYLATION |  | 7 | -0.48 | -1.01 | 0.461 | 0.750 | 1.000 | 3762 | tags=57%, list=31%, signal=83% |
| 4794 | GOBP\_LINOLEIC\_ACID\_METABOLIC\_PROCESS |  | 9 | -0.44 | -1.01 | 0.468 | 0.751 | 1.000 | 1860 | tags=33%, list=15%, signal=39% |
| 4795 | GOBP\_GLYCOSYLCERAMIDE\_METABOLIC\_PROCESS |  | 13 | -0.40 | -1.01 | 0.464 | 0.751 | 1.000 | 2915 | tags=46%, list=24%, signal=61% |
| 4796 | GOBP\_DETECTION\_OF\_MUSCLE\_STRETCH |  | 3 | -0.60 | -1.01 | 0.472 | 0.751 | 1.000 | 2879 | tags=67%, list=24%, signal=88% |
| 4797 | GOBP\_AXONAL\_FASCICULATION |  | 14 | -0.39 | -1.01 | 0.468 | 0.751 | 1.000 | 1638 | tags=21%, list=14%, signal=25% |
| 4798 | GOBP\_REGULATION\_OF\_EXIT\_FROM\_MITOSIS |  | 10 | -0.43 | -1.01 | 0.474 | 0.752 | 1.000 | 4521 | tags=60%, list=38%, signal=96% |
| 4799 | GOBP\_GLYCOPROTEIN\_METABOLIC\_PROCESS |  | 284 | -0.26 | -1.01 | 0.463 | 0.752 | 1.000 | 2811 | tags=25%, list=23%, signal=32% |
| 4800 | GOBP\_REGULATION\_OF\_RETINAL\_GANGLION\_CELL\_AXON\_GUIDANCE |  | 4 | -0.56 | -1.01 | 0.478 | 0.752 | 1.000 | 3196 | tags=50%, list=27%, signal=68% |
| 4801 | GOBP\_CILIUM\_MOVEMENT |  | 64 | -0.30 | -1.01 | 0.468 | 0.753 | 1.000 | 2518 | tags=28%, list=21%, signal=35% |
| 4802 | GOBP\_DNA\_REPLICATION\_CHECKPOINT |  | 15 | -0.39 | -1.01 | 0.468 | 0.752 | 1.000 | 4764 | tags=53%, list=40%, signal=88% |
| 4803 | GOBP\_REGULATION\_OF\_EPITHELIAL\_TO\_MESENCHYMAL\_TRANSITION |  | 74 | -0.29 | -1.01 | 0.457 | 0.753 | 1.000 | 3551 | tags=35%, list=30%, signal=50% |
| 4804 | GOBP\_POSITIVE\_REGULATION\_OF\_CHEMOKINE\_C\_C\_MOTIF\_LIGAND\_5\_PRODUCTION |  | 3 | -0.61 | -1.01 | 0.471 | 0.753 | 1.000 | 4686 | tags=100%, list=39%, signal=164% |
| 4805 | GOBP\_REGULATION\_OF\_SKELETAL\_MUSCLE\_ACETYLCHOLINE\_GATED\_CHANNEL\_CLUSTERING |  | 4 | -0.56 | -1.01 | 0.466 | 0.753 | 1.000 | 90 | tags=25%, list=1%, signal=25% |
| 4806 | GOBP\_POSITIVE\_REGULATION\_OF\_MUSCLE\_TISSUE\_DEVELOPMENT |  | 13 | -0.40 | -1.01 | 0.467 | 0.753 | 1.000 | 2503 | tags=38%, list=21%, signal=49% |
| 4807 | GOCC\_MALE\_GERM\_CELL\_NUCLEUS |  | 7 | -0.48 | -1.01 | 0.470 | 0.753 | 1.000 | 3972 | tags=57%, list=33%, signal=85% |
| 4808 | GOBP\_REGULATION\_OF\_G\_PROTEIN\_COUPLED\_RECEPTOR\_SIGNALING\_PATHWAY |  | 89 | -0.28 | -1.01 | 0.467 | 0.754 | 1.000 | 4787 | tags=45%, list=40%, signal=74% |
| 4809 | GOBP\_DIOL\_BIOSYNTHETIC\_PROCESS |  | 16 | -0.38 | -1.01 | 0.469 | 0.754 | 1.000 | 1930 | tags=31%, list=16%, signal=37% |
| 4810 | GOBP\_REGULATION\_OF\_CAMP\_MEDIATED\_SIGNALING |  | 23 | -0.35 | -1.01 | 0.467 | 0.754 | 1.000 | 1939 | tags=26%, list=16%, signal=31% |
| 4811 | GOBP\_INTERLEUKIN\_4\_MEDIATED\_SIGNALING\_PATHWAY |  | 6 | -0.50 | -1.01 | 0.469 | 0.754 | 1.000 | 6053 | tags=100%, list=50%, signal=201% |
| 4812 | GOMF\_LIPASE\_INHIBITOR\_ACTIVITY |  | 8 | -0.46 | -1.01 | 0.469 | 0.754 | 1.000 | 1072 | tags=25%, list=9%, signal=27% |
| 4813 | GOBP\_MONOUBIQUITINATED\_PROTEIN\_DEUBIQUITINATION |  | 8 | -0.46 | -1.01 | 0.468 | 0.754 | 1.000 | 5867 | tags=75%, list=49%, signal=147% |
| 4814 | GOBP\_LIPOXYGENASE\_PATHWAY |  | 7 | -0.48 | -1.01 | 0.478 | 0.754 | 1.000 | 1421 | tags=43%, list=12%, signal=49% |
| 4815 | GOBP\_REGULATION\_OF\_PROTEIN\_K63\_LINKED\_UBIQUITINATION |  | 7 | -0.48 | -1.01 | 0.471 | 0.754 | 1.000 | 2731 | tags=57%, list=23%, signal=74% |
| 4816 | GOBP\_CARBOHYDRATE\_DERIVATIVE\_CATABOLIC\_PROCESS |  | 130 | -0.27 | -1.01 | 0.467 | 0.754 | 1.000 | 2802 | tags=28%, list=23%, signal=36% |
| 4817 | GOBP\_POSITIVE\_REGULATION\_OF\_MAST\_CELL\_CHEMOTAXIS |  | 5 | -0.52 | -1.01 | 0.470 | 0.754 | 1.000 | 3196 | tags=60%, list=27%, signal=82% |
| 4818 | GOMF\_DIPEPTIDASE\_ACTIVITY |  | 12 | -0.41 | -1.01 | 0.475 | 0.754 | 1.000 | 3630 | tags=50%, list=30%, signal=72% |
| 4819 | GOBP\_CELL\_SURFACE\_RECEPTOR\_SIGNALING\_PATHWAY\_INVOLVED\_IN\_HEART\_DEVELOPMENT |  | 20 | -0.37 | -1.01 | 0.455 | 0.754 | 1.000 | 3314 | tags=40%, list=28%, signal=55% |
| 4820 | GOMF\_K63\_LINKED\_POLYUBIQUITIN\_MODIFICATION\_DEPENDENT\_PROTEIN\_BINDING |  | 16 | -0.39 | -1.01 | 0.470 | 0.754 | 1.000 | 5717 | tags=81%, list=48%, signal=155% |
| 4821 | GOBP\_PROTEIN\_HETEROTETRAMERIZATION |  | 10 | -0.43 | -1.01 | 0.475 | 0.755 | 1.000 | 5939 | tags=80%, list=49%, signal=158% |
| 4822 | GOBP\_TOLL\_LIKE\_RECEPTOR\_9\_SIGNALING\_PATHWAY |  | 19 | -0.37 | -1.01 | 0.463 | 0.755 | 1.000 | 4649 | tags=58%, list=39%, signal=94% |
| 4823 | GOBP\_REGULATION\_OF\_PHAGOCYTOSIS |  | 62 | -0.30 | -1.01 | 0.465 | 0.755 | 1.000 | 3130 | tags=32%, list=26%, signal=43% |
| 4824 | GOBP\_BLOOD\_VESSEL\_ENDOTHELIAL\_CELL\_MIGRATION |  | 91 | -0.28 | -1.01 | 0.473 | 0.755 | 1.000 | 3308 | tags=31%, list=28%, signal=42% |
| 4825 | GOBP\_REGULATION\_OF\_PRODUCTION\_OF\_SMALL\_RNA\_INVOLVED\_IN\_GENE\_SILENCING\_BY\_RNA |  | 17 | -0.38 | -1.01 | 0.461 | 0.755 | 1.000 | 2384 | tags=24%, list=20%, signal=29% |
| 4826 | GOBP\_ATTACHMENT\_OF\_MITOTIC\_SPINDLE\_MICROTUBULES\_TO\_KINETOCHORE |  | 13 | -0.40 | -1.01 | 0.468 | 0.755 | 1.000 | 3509 | tags=38%, list=29%, signal=54% |
| 4827 | GOBP\_REGULATION\_OF\_HISTONE\_UBIQUITINATION |  | 10 | -0.43 | -1.01 | 0.472 | 0.755 | 1.000 | 2222 | tags=40%, list=18%, signal=49% |
| 4828 | GOBP\_ADRENAL\_GLAND\_DEVELOPMENT |  | 16 | -0.38 | -1.01 | 0.471 | 0.755 | 1.000 | 2002 | tags=31%, list=17%, signal=37% |
| 4829 | GOBP\_MAMMARY\_GLAND\_EPITHELIAL\_CELL\_PROLIFERATION |  | 23 | -0.35 | -1.01 | 0.462 | 0.755 | 1.000 | 2769 | tags=35%, list=23%, signal=45% |
| 4830 | GOBP\_DNA\_MODIFICATION |  | 73 | -0.29 | -1.01 | 0.467 | 0.755 | 1.000 | 4130 | tags=38%, list=34%, signal=58% |
| 4831 | GOBP\_POSITIVE\_REGULATION\_OF\_CELL\_DIVISION |  | 58 | -0.30 | -1.01 | 0.464 | 0.755 | 1.000 | 3330 | tags=31%, list=28%, signal=43% |
| 4832 | GOBP\_EMBRYONIC\_FORELIMB\_MORPHOGENESIS |  | 15 | -0.39 | -1.01 | 0.466 | 0.755 | 1.000 | 852 | tags=20%, list=7%, signal=22% |
| 4833 | GOBP\_REGULATION\_OF\_LIPOPHAGY |  | 5 | -0.53 | -1.01 | 0.470 | 0.755 | 1.000 | 3931 | tags=60%, list=33%, signal=89% |
| 4834 | GOBP\_REGULATION\_OF\_RELAXATION\_OF\_CARDIAC\_MUSCLE |  | 4 | -0.56 | -1.01 | 0.476 | 0.755 | 1.000 | 1304 | tags=50%, list=11%, signal=56% |
| 4835 | GOMF\_SUPERCOILED\_DNA\_BINDING |  | 5 | -0.53 | -1.01 | 0.479 | 0.755 | 1.000 | 4649 | tags=60%, list=39%, signal=98% |
| 4836 | GOCC\_POSTSYNAPTIC\_GOLGI\_APPARATUS |  | 6 | -0.50 | -1.01 | 0.488 | 0.755 | 1.000 | 3007 | tags=50%, list=25%, signal=67% |
| 4837 | GOBP\_MONOCYTE\_ACTIVATION |  | 6 | -0.50 | -1.01 | 0.476 | 0.755 | 1.000 | 4831 | tags=67%, list=40%, signal=111% |
| 4838 | GOBP\_THYROID\_HORMONE\_METABOLIC\_PROCESS |  | 13 | -0.40 | -1.01 | 0.471 | 0.755 | 1.000 | 1253 | tags=23%, list=10%, signal=26% |
| 4839 | GOBP\_DEVELOPMENT\_OF\_PRIMARY\_SEXUAL\_CHARACTERISTICS |  | 143 | -0.27 | -1.01 | 0.476 | 0.755 | 1.000 | 4868 | tags=48%, list=41%, signal=80% |
| 4840 | GOBP\_NEGATIVE\_REGULATION\_OF\_NON\_CANONICAL\_WNT\_SIGNALING\_PATHWAY |  | 5 | -0.52 | -1.01 | 0.476 | 0.756 | 1.000 | 1579 | tags=40%, list=13%, signal=46% |
| 4841 | GOBP\_REGULATION\_OF\_CENTROSOME\_CYCLE |  | 39 | -0.32 | -1.01 | 0.466 | 0.757 | 1.000 | 5835 | tags=64%, list=49%, signal=124% |
| 4842 | GOBP\_POSITIVE\_REGULATION\_OF\_SYNAPTIC\_TRANSMISSION\_GABAERGIC |  | 8 | -0.46 | -1.01 | 0.480 | 0.757 | 1.000 | 4164 | tags=50%, list=35%, signal=76% |
| 4843 | GOBP\_DETERMINATION\_OF\_LEFT\_RIGHT\_ASYMMETRY\_IN\_LATERAL\_MESODERM |  | 3 | -0.60 | -1.01 | 0.479 | 0.757 | 1.000 | 3565 | tags=33%, list=30%, signal=47% |
| 4844 | GOBP\_REGULATION\_OF\_POSTSYNAPTIC\_CYTOSOLIC\_CALCIUM\_ION\_CONCENTRATION |  | 6 | -0.50 | -1.01 | 0.483 | 0.757 | 1.000 | 5158 | tags=67%, list=43%, signal=117% |
| 4845 | GOBP\_VITAMIN\_D\_RECEPTOR\_SIGNALING\_PATHWAY |  | 8 | -0.46 | -1.01 | 0.482 | 0.757 | 1.000 | 4358 | tags=63%, list=36%, signal=98% |
| 4846 | GOBP\_REGULATION\_OF\_ENDOPLASMIC\_RETICULUM\_UNFOLDED\_PROTEIN\_RESPONSE |  | 24 | -0.35 | -1.01 | 0.471 | 0.757 | 1.000 | 4797 | tags=63%, list=40%, signal=104% |
| 4847 | GOBP\_POSITIVE\_REGULATION\_OF\_MUSCLE\_CONTRACTION |  | 24 | -0.35 | -1.01 | 0.467 | 0.757 | 1.000 | 786 | tags=21%, list=7%, signal=22% |
| 4848 | GOMF\_ENDODEOXYRIBONUCLEASE\_ACTIVITY\_PRODUCING\_3\_PHOSPHOMONOESTERS |  | 5 | -0.52 | -1.01 | 0.473 | 0.757 | 1.000 | 5752 | tags=100%, list=48%, signal=192% |
| 4849 | GOBP\_C\_TERMINAL\_PROTEIN\_AMINO\_ACID\_MODIFICATION |  | 10 | -0.43 | -1.01 | 0.475 | 0.757 | 1.000 | 6631 | tags=90%, list=55%, signal=201% |
| 4850 | GOMF\_RNA\_POLYMERASE\_CORE\_ENZYME\_BINDING |  | 35 | -0.32 | -1.01 | 0.474 | 0.757 | 1.000 | 6008 | tags=69%, list=50%, signal=137% |
| 4851 | GOBP\_PROTEIN\_HETEROOLIGOMERIZATION |  | 15 | -0.39 | -1.01 | 0.462 | 0.757 | 1.000 | 5939 | tags=67%, list=49%, signal=132% |
| 4852 | GOBP\_CELLULAR\_ION\_HOMEOSTASIS |  | 420 | -0.26 | -1.01 | 0.475 | 0.757 | 1.000 | 3046 | tags=30%, list=25%, signal=39% |
| 4853 | GOBP\_EMBRYONIC\_VISCEROCRANIUM\_MORPHOGENESIS |  | 5 | -0.52 | -1.01 | 0.476 | 0.758 | 1.000 | 4827 | tags=80%, list=40%, signal=134% |
| 4854 | GOMF\_LYSOPHOSPHATIDIC\_ACID\_ACYLTRANSFERASE\_ACTIVITY |  | 14 | -0.40 | -1.01 | 0.471 | 0.758 | 1.000 | 47 | tags=7%, list=0%, signal=7% |
| 4855 | GOCC\_MPP7\_DLG1\_LIN7\_COMPLEX |  | 4 | -0.56 | -1.01 | 0.484 | 0.758 | 1.000 | 3825 | tags=50%, list=32%, signal=73% |
| 4856 | GOBP\_CELLULAR\_HORMONE\_METABOLIC\_PROCESS |  | 76 | -0.29 | -1.01 | 0.480 | 0.758 | 1.000 | 2374 | tags=22%, list=20%, signal=28% |
| 4857 | GOBP\_MEMBRANE\_RAFT\_LOCALIZATION |  | 6 | -0.50 | -1.01 | 0.478 | 0.758 | 1.000 | 1519 | tags=50%, list=13%, signal=57% |
| 4858 | GOBP\_POSITIVE\_REGULATION\_OF\_GENE\_EXPRESSION\_EPIGENETIC |  | 39 | -0.32 | -1.01 | 0.468 | 0.758 | 1.000 | 4185 | tags=41%, list=35%, signal=63% |
| 4859 | GOBP\_RESPONSE\_TO\_MECHANICAL\_STIMULUS |  | 132 | -0.27 | -1.01 | 0.473 | 0.758 | 1.000 | 3331 | tags=34%, list=28%, signal=47% |
| 4860 | GOBP\_NEGATIVE\_REGULATION\_OF\_ENDOPLASMIC\_RETICULUM\_STRESS\_INDUCED\_INTRINSIC\_APOPTOTIC\_SIGNALING\_PATHWAY |  | 11 | -0.42 | -1.01 | 0.480 | 0.758 | 1.000 | 3483 | tags=55%, list=29%, signal=77% |
| 4861 | GOMF\_ESTRADIOL\_17\_BETA\_DEHYDROGENASE\_ACTIVITY |  | 7 | -0.48 | -1.01 | 0.475 | 0.758 | 1.000 | 101 | tags=14%, list=1%, signal=14% |
| 4862 | GOCC\_ANCHORED\_COMPONENT\_OF\_PLASMA\_MEMBRANE |  | 29 | -0.34 | -1.01 | 0.470 | 0.758 | 1.000 | 2066 | tags=24%, list=17%, signal=29% |
| 4863 | GOMF\_DOPAMINE\_RECEPTOR\_BINDING |  | 10 | -0.43 | -1.01 | 0.474 | 0.759 | 1.000 | 4986 | tags=60%, list=42%, signal=102% |
| 4864 | GOBP\_METAPHASE\_PLATE\_CONGRESSION |  | 53 | -0.30 | -1.01 | 0.474 | 0.759 | 1.000 | 4126 | tags=38%, list=34%, signal=57% |
| 4865 | GOBP\_CELLULAR\_RESPONSE\_TO\_BIOTIC\_STIMULUS |  | 130 | -0.27 | -1.01 | 0.482 | 0.759 | 1.000 | 4035 | tags=38%, list=34%, signal=57% |
| 4866 | GOBP\_POSITIVE\_REGULATION\_OF\_CELLULAR\_PROTEIN\_LOCALIZATION |  | 219 | -0.26 | -1.01 | 0.488 | 0.759 | 1.000 | 4182 | tags=32%, list=35%, signal=49% |
| 4867 | GOMF\_AMIDE\_BINDING |  | 245 | -0.26 | -1.01 | 0.481 | 0.759 | 1.000 | 3789 | tags=32%, list=32%, signal=46% |
| 4868 | GOBP\_NEGATIVE\_REGULATION\_OF\_PROTEIN\_LOCALIZATION\_TO\_CELL\_SURFACE |  | 11 | -0.42 | -1.01 | 0.475 | 0.759 | 1.000 | 5929 | tags=82%, list=49%, signal=161% |
| 4869 | GOBP\_OPIOID\_RECEPTOR\_SIGNALING\_PATHWAY |  | 7 | -0.47 | -1.01 | 0.476 | 0.759 | 1.000 | 5271 | tags=71%, list=44%, signal=127% |
| 4870 | GOBP\_LYTIC\_VACUOLE\_ORGANIZATION |  | 55 | -0.30 | -1.01 | 0.473 | 0.759 | 1.000 | 3483 | tags=38%, list=29%, signal=54% |
| 4871 | GOBP\_DNA\_STRAND\_ELONGATION\_INVOLVED\_IN\_DNA\_REPLICATION |  | 17 | -0.37 | -1.01 | 0.463 | 0.759 | 1.000 | 4773 | tags=47%, list=40%, signal=78% |
| 4872 | GOBP\_NEGATIVE\_REGULATION\_OF\_DENDRITIC\_SPINE\_DEVELOPMENT |  | 8 | -0.46 | -1.01 | 0.484 | 0.759 | 1.000 | 23 | tags=13%, list=0%, signal=13% |
| 4873 | GOBP\_ARGININE\_TRANSMEMBRANE\_TRANSPORT |  | 6 | -0.50 | -1.01 | 0.477 | 0.759 | 1.000 | 2026 | tags=33%, list=17%, signal=40% |
| 4874 | GOMF\_POTASSIUM\_CHANNEL\_INHIBITOR\_ACTIVITY |  | 6 | -0.50 | -1.01 | 0.486 | 0.759 | 1.000 | 3814 | tags=50%, list=32%, signal=73% |
| 4875 | GOBP\_CARBOHYDRATE\_MEDIATED\_SIGNALING |  | 5 | -0.52 | -1.01 | 0.486 | 0.759 | 1.000 | 4091 | tags=60%, list=34%, signal=91% |
| 4876 | GOBP\_VESICLE\_TARGETING\_TO\_FROM\_OR\_WITHIN\_GOLGI |  | 60 | -0.30 | -1.01 | 0.478 | 0.760 | 1.000 | 5983 | tags=62%, list=50%, signal=122% |
| 4877 | GOBP\_REGULATION\_OF\_GLUCONEOGENESIS |  | 33 | -0.33 | -1.01 | 0.474 | 0.760 | 1.000 | 2667 | tags=33%, list=22%, signal=43% |
| 4878 | GOBP\_P38MAPK\_CASCADE |  | 33 | -0.33 | -1.01 | 0.473 | 0.760 | 1.000 | 3647 | tags=45%, list=30%, signal=65% |
| 4879 | GOMF\_SULFOTRANSFERASE\_ACTIVITY |  | 29 | -0.33 | -1.01 | 0.470 | 0.760 | 1.000 | 3719 | tags=45%, list=31%, signal=65% |
| 4880 | GOBP\_NEGATIVE\_REGULATION\_OF\_ANTIGEN\_RECEPTOR\_MEDIATED\_SIGNALING\_PATHWAY |  | 20 | -0.36 | -1.01 | 0.474 | 0.760 | 1.000 | 2857 | tags=35%, list=24%, signal=46% |
| 4881 | GOBP\_CD40\_SIGNALING\_PATHWAY |  | 11 | -0.42 | -1.01 | 0.474 | 0.760 | 1.000 | 4803 | tags=55%, list=40%, signal=91% |
| 4882 | GOMF\_PROTEIN\_DIMERIZATION\_ACTIVITY |  | 650 | -0.25 | -1.01 | 0.475 | 0.760 | 1.000 | 3914 | tags=32%, list=33%, signal=46% |
| 4883 | GOBP\_AMPA\_GLUTAMATE\_RECEPTOR\_CLUSTERING |  | 3 | -0.60 | -1.01 | 0.480 | 0.760 | 1.000 | 4715 | tags=67%, list=39%, signal=110% |
| 4884 | GOCC\_EXOCYST |  | 15 | -0.39 | -1.01 | 0.476 | 0.760 | 1.000 | 4055 | tags=47%, list=34%, signal=70% |
| 4885 | GOBP\_VITAMIN\_D3\_METABOLIC\_PROCESS |  | 3 | -0.60 | -1.01 | 0.492 | 0.760 | 1.000 | 4011 | tags=67%, list=33%, signal=100% |
| 4886 | GOBP\_NEGATIVE\_REGULATION\_OF\_SMAD\_PROTEIN\_COMPLEX\_ASSEMBLY |  | 4 | -0.56 | -1.01 | 0.491 | 0.760 | 1.000 | 3072 | tags=50%, list=26%, signal=67% |
| 4887 | GOCC\_G\_PROTEIN\_COUPLED\_RECEPTOR\_COMPLEX |  | 5 | -0.52 | -1.01 | 0.488 | 0.761 | 1.000 | 5053 | tags=60%, list=42%, signal=104% |
| 4888 | GOBP\_EPIDERMAL\_CELL\_DIFFERENTIATION |  | 115 | -0.28 | -1.01 | 0.478 | 0.761 | 1.000 | 4265 | tags=40%, list=36%, signal=61% |
| 4889 | GOBP\_MACROPHAGE\_INFLAMMATORY\_PROTEIN\_1\_ALPHA\_PRODUCTION |  | 4 | -0.55 | -1.01 | 0.497 | 0.761 | 1.000 | 1788 | tags=50%, list=15%, signal=59% |
| 4890 | GOBP\_POSITIVE\_REGULATION\_OF\_TRANSCRIPTION\_ELONGATION\_FROM\_RNA\_POLYMERASE\_II\_PROMOTER |  | 9 | -0.45 | -1.01 | 0.490 | 0.761 | 1.000 | 2692 | tags=33%, list=22%, signal=43% |
| 4891 | GOCC\_STEREOCILIA\_COUPLING\_LINK |  | 3 | -0.60 | -1.01 | 0.492 | 0.761 | 1.000 | 4813 | tags=100%, list=40%, signal=167% |
| 4892 | GOBP\_FATTY\_ACID\_DERIVATIVE\_METABOLIC\_PROCESS |  | 50 | -0.30 | -1.00 | 0.480 | 0.761 | 1.000 | 4801 | tags=50%, list=40%, signal=83% |
| 4893 | GOBP\_ACTIVATION\_OF\_NF\_KAPPAB\_INDUCING\_KINASE\_ACTIVITY |  | 11 | -0.42 | -1.00 | 0.478 | 0.761 | 1.000 | 1781 | tags=36%, list=15%, signal=43% |
| 4894 | GOBP\_REGULATION\_OF\_INTRINSIC\_APOPTOTIC\_SIGNALING\_PATHWAY\_IN\_RESPONSE\_TO\_DNA\_DAMAGE\_BY\_P53\_CLASS\_MEDIATOR |  | 10 | -0.43 | -1.00 | 0.475 | 0.761 | 1.000 | 2656 | tags=40%, list=22%, signal=51% |
| 4895 | GOCC\_EARLY\_ENDOSOME |  | 272 | -0.26 | -1.00 | 0.483 | 0.761 | 1.000 | 3401 | tags=31%, list=28%, signal=42% |
| 4896 | GOBP\_NEGATIVE\_REGULATION\_OF\_MHC\_CLASS\_II\_BIOSYNTHETIC\_PROCESS |  | 4 | -0.56 | -1.00 | 0.491 | 0.761 | 1.000 | 3395 | tags=50%, list=28%, signal=70% |
| 4897 | GOBP\_SUBSTANTIA\_NIGRA\_DEVELOPMENT |  | 29 | -0.33 | -1.00 | 0.474 | 0.761 | 1.000 | 1753 | tags=24%, list=15%, signal=28% |
| 4898 | GOMF\_RETINAL\_BINDING |  | 8 | -0.45 | -1.00 | 0.484 | 0.761 | 1.000 | 3316 | tags=50%, list=28%, signal=69% |
| 4899 | GOBP\_REGULATION\_OF\_MITOTIC\_RECOMBINATION |  | 4 | -0.55 | -1.00 | 0.486 | 0.761 | 1.000 | 5365 | tags=100%, list=45%, signal=181% |
| 4900 | GOBP\_REGULATION\_OF\_CYCLIN\_DEPENDENT\_PROTEIN\_KINASE\_ACTIVITY |  | 73 | -0.29 | -1.00 | 0.487 | 0.761 | 1.000 | 3687 | tags=33%, list=31%, signal=47% |
| 4901 | GOBP\_CORPUS\_CALLOSUM\_MORPHOGENESIS |  | 4 | -0.56 | -1.00 | 0.476 | 0.761 | 1.000 | 5153 | tags=75%, list=43%, signal=131% |
| 4902 | GOBP\_GLUCOSE\_METABOLIC\_PROCESS |  | 148 | -0.27 | -1.00 | 0.485 | 0.761 | 1.000 | 4302 | tags=41%, list=36%, signal=63% |
| 4903 | GOBP\_NUCLEAR\_TRANSCRIBED\_MRNA\_CATABOLIC\_PROCESS\_DEADENYLATION\_INDEPENDENT\_DECAY |  | 6 | -0.49 | -1.00 | 0.484 | 0.761 | 1.000 | 4331 | tags=50%, list=36%, signal=78% |
| 4904 | GOBP\_NUCLEAR\_TRANSCRIBED\_MRNA\_CATABOLIC\_PROCESS\_DEADENYLATION\_DEPENDENT\_DECAY |  | 62 | -0.30 | -1.00 | 0.483 | 0.761 | 1.000 | 2080 | tags=23%, list=17%, signal=27% |
| 4905 | GOMF\_PHOSPHATIDYLINOSITOL\_4\_PHOSPHATE\_BINDING |  | 22 | -0.35 | -1.00 | 0.479 | 0.761 | 1.000 | 4986 | tags=50%, list=42%, signal=85% |
| 4906 | GOBP\_NEGATIVE\_REGULATION\_OF\_INCLUSION\_BODY\_ASSEMBLY |  | 6 | -0.49 | -1.00 | 0.479 | 0.761 | 1.000 | 3345 | tags=67%, list=28%, signal=92% |
| 4907 | GOBP\_GLOMERULAR\_EPITHELIAL\_CELL\_DIFFERENTIATION |  | 15 | -0.39 | -1.00 | 0.481 | 0.762 | 1.000 | 1432 | tags=20%, list=12%, signal=23% |
| 4908 | GOBP\_REGULATION\_OF\_BONE\_MINERALIZATION |  | 46 | -0.31 | -1.00 | 0.474 | 0.762 | 1.000 | 3488 | tags=37%, list=29%, signal=52% |
| 4909 | GOBP\_L\_ARGININE\_TRANSMEMBRANE\_TRANSPORT |  | 6 | -0.50 | -1.00 | 0.482 | 0.761 | 1.000 | 2026 | tags=33%, list=17%, signal=40% |
| 4910 | GOBP\_THYROID\_HORMONE\_MEDIATED\_SIGNALING\_PATHWAY |  | 4 | -0.56 | -1.00 | 0.496 | 0.761 | 1.000 | 4001 | tags=75%, list=33%, signal=112% |
| 4911 | GOCC\_POSTSYNAPTIC\_DENSITY\_INTRACELLULAR\_COMPONENT |  | 8 | -0.46 | -1.00 | 0.487 | 0.761 | 1.000 | 5152 | tags=50%, list=43%, signal=87% |
| 4912 | GOMF\_DNA\_TOPOISOMERASE\_ACTIVITY |  | 6 | -0.49 | -1.00 | 0.477 | 0.761 | 1.000 | 4534 | tags=67%, list=38%, signal=107% |
| 4913 | GOBP\_NUCLEAR\_EXPORT |  | 159 | -0.27 | -1.00 | 0.487 | 0.761 | 1.000 | 5194 | tags=42%, list=43%, signal=73% |
| 4914 | GOMF\_ARGININE\_TRANSMEMBRANE\_TRANSPORTER\_ACTIVITY |  | 6 | -0.50 | -1.00 | 0.482 | 0.761 | 1.000 | 2026 | tags=33%, list=17%, signal=40% |
| 4915 | GOBP\_ESTABLISHMENT\_OF\_PLANAR\_POLARITY\_INVOLVED\_IN\_NEURAL\_TUBE\_CLOSURE |  | 9 | -0.44 | -1.00 | 0.481 | 0.761 | 1.000 | 2992 | tags=33%, list=25%, signal=44% |
| 4916 | GOMF\_PROTEIN\_SERINE\_THREONINE\_PHOSPHATASE\_ACTIVITY |  | 73 | -0.29 | -1.00 | 0.485 | 0.761 | 1.000 | 3483 | tags=34%, list=29%, signal=48% |
| 4917 | GOBP\_VIRAL\_LIFE\_CYCLE |  | 231 | -0.26 | -1.00 | 0.491 | 0.761 | 1.000 | 4112 | tags=39%, list=34%, signal=58% |
| 4918 | GOBP\_NAIL\_DEVELOPMENT |  | 4 | -0.55 | -1.00 | 0.501 | 0.761 | 1.000 | 3856 | tags=50%, list=32%, signal=74% |
| 4919 | GOMF\_RNA\_POLYMERASE\_II\_TRANSCRIPTION\_COREPRESSOR\_BINDING |  | 11 | -0.42 | -1.00 | 0.487 | 0.761 | 1.000 | 6440 | tags=82%, list=54%, signal=176% |
| 4920 | GOMF\_CARBONATE\_DEHYDRATASE\_ACTIVITY |  | 9 | -0.44 | -1.00 | 0.482 | 0.761 | 1.000 | 809 | tags=33%, list=7%, signal=36% |
| 4921 | GOCC\_CMG\_COMPLEX |  | 9 | -0.44 | -1.00 | 0.469 | 0.761 | 1.000 | 5533 | tags=78%, list=46%, signal=144% |
| 4922 | GOMF\_PHOSPHATIDIC\_ACID\_BINDING |  | 17 | -0.37 | -1.00 | 0.470 | 0.762 | 1.000 | 4585 | tags=53%, list=38%, signal=86% |
| 4923 | GOBP\_MULTICELLULAR\_ORGANISMAL\_IRON\_ION\_HOMEOSTASIS |  | 6 | -0.49 | -1.00 | 0.491 | 0.762 | 1.000 | 3269 | tags=50%, list=27%, signal=69% |
| 4924 | GOBP\_POSITIVE\_REGULATION\_OF\_BLOOD\_VESSEL\_ENDOTHELIAL\_CELL\_MIGRATION |  | 41 | -0.31 | -1.00 | 0.477 | 0.762 | 1.000 | 3905 | tags=37%, list=33%, signal=54% |
| 4925 | GOBP\_PURINE\_NUCLEOTIDE\_SALVAGE |  | 6 | -0.49 | -1.00 | 0.493 | 0.762 | 1.000 | 786 | tags=33%, list=7%, signal=36% |
| 4926 | GOMF\_RNA\_POLYMERASE\_I\_TRANSCRIPTION\_REGULATORY\_REGION\_SEQUENCE\_SPECIFIC\_DNA\_BINDING |  | 5 | -0.52 | -1.00 | 0.485 | 0.762 | 1.000 | 1505 | tags=20%, list=13%, signal=23% |
| 4927 | GOBP\_NEUROTRANSMITTER\_RECEPTOR\_TRANSPORT\_TO\_PLASMA\_MEMBRANE |  | 12 | -0.41 | -1.00 | 0.478 | 0.762 | 1.000 | 1222 | tags=25%, list=10%, signal=28% |
| 4928 | GOBP\_CELLULAR\_RESPONSE\_TO\_PEPTIDE |  | 280 | -0.26 | -1.00 | 0.499 | 0.762 | 1.000 | 3797 | tags=40%, list=32%, signal=57% |
| 4929 | GOMF\_HISTONE\_ACETYLTRANSFERASE\_ACTIVITY\_H3\_K23\_SPECIFIC |  | 5 | -0.52 | -1.00 | 0.488 | 0.762 | 1.000 | 5045 | tags=60%, list=42%, signal=103% |
| 4930 | GOBP\_REGULATION\_OF\_MACROPHAGE\_DIFFERENTIATION |  | 17 | -0.37 | -1.00 | 0.477 | 0.761 | 1.000 | 3836 | tags=41%, list=32%, signal=60% |
| 4931 | GOBP\_DEFINITIVE\_ERYTHROCYTE\_DIFFERENTIATION |  | 4 | -0.56 | -1.00 | 0.491 | 0.761 | 1.000 | 2308 | tags=25%, list=19%, signal=31% |
| 4932 | GOBP\_TRANSMEMBRANE\_TRANSPORT |  | 959 | -0.25 | -1.00 | 0.487 | 0.762 | 1.000 | 2955 | tags=27%, list=25%, signal=32% |
| 4933 | GOMF\_UBIQUITIN\_SPECIFIC\_PROTEASE\_BINDING |  | 17 | -0.38 | -1.00 | 0.477 | 0.762 | 1.000 | 3392 | tags=41%, list=28%, signal=57% |
| 4934 | GOCC\_LEADING\_EDGE\_MEMBRANE |  | 110 | -0.28 | -1.00 | 0.491 | 0.762 | 1.000 | 2599 | tags=25%, list=22%, signal=32% |
| 4935 | GOBP\_OOCYTE\_DIFFERENTIATION |  | 24 | -0.35 | -1.00 | 0.477 | 0.762 | 1.000 | 2740 | tags=33%, list=23%, signal=43% |
| 4936 | GOBP\_POSITIVE\_REGULATION\_OF\_NEUROTRANSMITTER\_TRANSPORT |  | 15 | -0.39 | -1.00 | 0.480 | 0.763 | 1.000 | 1699 | tags=27%, list=14%, signal=31% |
| 4937 | GOBP\_CELLULAR\_RESPONSE\_TO\_MANGANESE\_ION |  | 9 | -0.44 | -1.00 | 0.490 | 0.763 | 1.000 | 4814 | tags=67%, list=40%, signal=111% |
| 4938 | GOBP\_OXYGEN\_METABOLIC\_PROCESS |  | 5 | -0.52 | -1.00 | 0.481 | 0.763 | 1.000 | 1033 | tags=20%, list=9%, signal=22% |
| 4939 | GOBP\_REGULATION\_OF\_CGMP\_MEDIATED\_SIGNALING |  | 5 | -0.52 | -1.00 | 0.489 | 0.763 | 1.000 | 4846 | tags=60%, list=40%, signal=101% |
| 4940 | GOBP\_NEGATIVE\_REGULATION\_OF\_CHOLESTEROL\_METABOLIC\_PROCESS |  | 4 | -0.56 | -1.00 | 0.500 | 0.763 | 1.000 | 746 | tags=25%, list=6%, signal=27% |
| 4941 | GOMF\_ARMADILLO\_REPEAT\_DOMAIN\_BINDING |  | 7 | -0.47 | -1.00 | 0.486 | 0.763 | 1.000 | 3761 | tags=57%, list=31%, signal=83% |
| 4942 | GOBP\_REGULATION\_OF\_CHROMATIN\_SILENCING |  | 8 | -0.45 | -1.00 | 0.485 | 0.763 | 1.000 | 3610 | tags=50%, list=30%, signal=71% |
| 4943 | GOBP\_REGULATION\_OF\_HAIR\_FOLLICLE\_DEVELOPMENT |  | 11 | -0.42 | -1.00 | 0.486 | 0.763 | 1.000 | 658 | tags=18%, list=5%, signal=19% |
| 4944 | GOMF\_CADHERIN\_BINDING |  | 258 | -0.26 | -1.00 | 0.500 | 0.763 | 1.000 | 2811 | tags=22%, list=23%, signal=28% |
| 4945 | GOBP\_REGULATION\_OF\_ENTRY\_OF\_BACTERIUM\_INTO\_HOST\_CELL |  | 5 | -0.52 | -1.00 | 0.497 | 0.763 | 1.000 | 2610 | tags=40%, list=22%, signal=51% |
| 4946 | GOCC\_CENTRIOLAR\_SATELLITE |  | 83 | -0.28 | -1.00 | 0.491 | 0.763 | 1.000 | 3727 | tags=33%, list=31%, signal=47% |
| 4947 | GOMF\_DELAYED\_RECTIFIER\_POTASSIUM\_CHANNEL\_ACTIVITY |  | 14 | -0.39 | -1.00 | 0.475 | 0.763 | 1.000 | 400 | tags=14%, list=3%, signal=15% |
| 4948 | GOBP\_GENITALIA\_MORPHOGENESIS |  | 5 | -0.52 | -1.00 | 0.494 | 0.763 | 1.000 | 1146 | tags=40%, list=10%, signal=44% |
| 4949 | GOBP\_PROTEIN\_TETRAMERIZATION |  | 52 | -0.30 | -1.00 | 0.483 | 0.763 | 1.000 | 1635 | tags=17%, list=14%, signal=20% |
| 4950 | GOBP\_BUD\_ELONGATION\_INVOLVED\_IN\_LUNG\_BRANCHING |  | 7 | -0.48 | -1.00 | 0.494 | 0.762 | 1.000 | 3530 | tags=57%, list=29%, signal=81% |
| 4951 | GOBP\_PEPTIDYL\_PROLINE\_HYDROXYLATION |  | 14 | -0.39 | -1.00 | 0.490 | 0.763 | 1.000 | 3164 | tags=29%, list=26%, signal=39% |
| 4952 | GOBP\_PRE\_REPLICATIVE\_COMPLEX\_ASSEMBLY\_INVOLVED\_IN\_CELL\_CYCLE\_DNA\_REPLICATION |  | 6 | -0.49 | -1.00 | 0.493 | 0.763 | 1.000 | 5159 | tags=83%, list=43%, signal=146% |
| 4953 | GOBP\_CHROMATIN\_MAINTENANCE |  | 6 | -0.49 | -1.00 | 0.485 | 0.763 | 1.000 | 2656 | tags=50%, list=22%, signal=64% |
| 4954 | GOBP\_POSITIVE\_REGULATION\_OF\_ALCOHOL\_BIOSYNTHETIC\_PROCESS |  | 14 | -0.39 | -1.00 | 0.481 | 0.763 | 1.000 | 788 | tags=21%, list=7%, signal=23% |
| 4955 | GOBP\_SCF\_DEPENDENT\_PROTEASOMAL\_UBIQUITIN\_DEPENDENT\_PROTEIN\_CATABOLIC\_PROCESS |  | 71 | -0.29 | -1.00 | 0.491 | 0.763 | 1.000 | 3958 | tags=30%, list=33%, signal=44% |
| 4956 | GOBP\_REGULATION\_OF\_INTRINSIC\_APOPTOTIC\_SIGNALING\_PATHWAY\_BY\_P53\_CLASS\_MEDIATOR |  | 17 | -0.37 | -1.00 | 0.477 | 0.763 | 1.000 | 2656 | tags=29%, list=22%, signal=38% |
| 4957 | GOBP\_NEGATIVE\_REGULATION\_OF\_FATTY\_ACID\_TRANSPORT |  | 7 | -0.47 | -1.00 | 0.488 | 0.763 | 1.000 | 5710 | tags=86%, list=48%, signal=163% |
| 4958 | GOBP\_RESPONSE\_TO\_BIOTIC\_STIMULUS |  | 778 | -0.25 | -1.00 | 0.504 | 0.763 | 1.000 | 3385 | tags=31%, list=28%, signal=40% |
| 4959 | GOMF\_CORECEPTOR\_ACTIVITY\_INVOLVED\_IN\_WNT\_SIGNALING\_PATHWAY |  | 8 | -0.45 | -1.00 | 0.494 | 0.763 | 1.000 | 5850 | tags=75%, list=49%, signal=146% |
| 4960 | GOBP\_NEGATIVE\_REGULATION\_OF\_FIBROBLAST\_PROLIFERATION |  | 18 | -0.37 | -1.00 | 0.486 | 0.763 | 1.000 | 2007 | tags=33%, list=17%, signal=40% |
| 4961 | GOCC\_MYELIN\_SHEATH |  | 30 | -0.33 | -1.00 | 0.475 | 0.763 | 1.000 | 4312 | tags=43%, list=36%, signal=67% |
| 4962 | GOBP\_REGULATION\_OF\_LIPID\_METABOLIC\_PROCESS |  | 288 | -0.26 | -1.00 | 0.504 | 0.765 | 1.000 | 4001 | tags=36%, list=33%, signal=53% |
| 4963 | GOCC\_INFLAMMASOME\_COMPLEX |  | 5 | -0.51 | -1.00 | 0.490 | 0.765 | 1.000 | 5278 | tags=80%, list=44%, signal=143% |
| 4964 | GOBP\_FOLATE\_IMPORT\_ACROSS\_PLASMA\_MEMBRANE |  | 3 | -0.60 | -1.00 | 0.491 | 0.765 | 1.000 | 240 | tags=33%, list=2%, signal=34% |
| 4965 | GOBP\_LACTATE\_TRANSMEMBRANE\_TRANSPORT |  | 6 | -0.49 | -1.00 | 0.495 | 0.765 | 1.000 | 2638 | tags=33%, list=22%, signal=43% |
| 4966 | GOBP\_SPECIFICATION\_OF\_SYMMETRY |  | 80 | -0.29 | -1.00 | 0.481 | 0.765 | 1.000 | 2203 | tags=20%, list=18%, signal=24% |
| 4967 | GOBP\_REGULATION\_OF\_ATTACHMENT\_OF\_SPINDLE\_MICROTUBULES\_TO\_KINETOCHORE |  | 13 | -0.40 | -1.00 | 0.490 | 0.765 | 1.000 | 6680 | tags=85%, list=56%, signal=190% |
| 4968 | GOBP\_POSITIVE\_REGULATION\_OF\_MYELOID\_LEUKOCYTE\_DIFFERENTIATION |  | 35 | -0.32 | -1.00 | 0.483 | 0.765 | 1.000 | 1999 | tags=31%, list=17%, signal=38% |
| 4969 | GOBP\_CELLULAR\_RESPONSE\_TO\_CHEMICAL\_STRESS |  | 253 | -0.26 | -1.00 | 0.506 | 0.765 | 1.000 | 3675 | tags=34%, list=31%, signal=48% |
| 4970 | GOBP\_ADULT\_BEHAVIOR |  | 65 | -0.29 | -1.00 | 0.493 | 0.766 | 1.000 | 3699 | tags=35%, list=31%, signal=51% |
| 4971 | GOCC\_ER\_UBIQUITIN\_LIGASE\_COMPLEX |  | 6 | -0.49 | -1.00 | 0.496 | 0.766 | 1.000 | 2242 | tags=33%, list=19%, signal=41% |
| 4972 | GOBP\_REGULATION\_OF\_NITRIC\_OXIDE\_SYNTHASE\_ACTIVITY |  | 32 | -0.33 | -1.00 | 0.484 | 0.766 | 1.000 | 3909 | tags=38%, list=33%, signal=55% |
| 4973 | GOBP\_IMMUNOGLOBULIN\_PRODUCTION\_INVOLVED\_IN\_IMMUNOGLOBULIN\_MEDIATED\_IMMUNE\_RESPONSE |  | 37 | -0.32 | -1.00 | 0.482 | 0.766 | 1.000 | 3452 | tags=38%, list=29%, signal=53% |
| 4974 | GOCC\_CLATHRIN\_SCULPTED\_GAMMA\_AMINOBUTYRIC\_ACID\_TRANSPORT\_VESICLE |  | 5 | -0.52 | -1.00 | 0.492 | 0.767 | 1.000 | 3390 | tags=80%, list=28%, signal=111% |
| 4975 | GOBP\_REGULATION\_OF\_INFLAMMATORY\_RESPONSE\_TO\_WOUNDING |  | 4 | -0.55 | -1.00 | 0.490 | 0.767 | 1.000 | 1734 | tags=50%, list=14%, signal=58% |
| 4976 | GOBP\_DNA\_DAMAGE\_RESPONSE\_SIGNAL\_TRANSDUCTION\_RESULTING\_IN\_TRANSCRIPTION |  | 12 | -0.41 | -1.00 | 0.490 | 0.767 | 1.000 | 4751 | tags=67%, list=40%, signal=110% |
| 4977 | GOBP\_POSITIVE\_REGULATION\_OF\_GLUCONEOGENESIS |  | 11 | -0.42 | -1.00 | 0.487 | 0.767 | 1.000 | 2656 | tags=45%, list=22%, signal=58% |
| 4978 | GOBP\_POSITIVE\_REGULATION\_OF\_ENDOPLASMIC\_RETICULUM\_UNFOLDED\_PROTEIN\_RESPONSE |  | 11 | -0.41 | -1.00 | 0.484 | 0.767 | 1.000 | 3966 | tags=64%, list=33%, signal=95% |
| 4979 | GOMF\_G\_QUADRUPLEX\_RNA\_BINDING |  | 5 | -0.52 | -1.00 | 0.506 | 0.767 | 1.000 | 2025 | tags=40%, list=17%, signal=48% |
| 4980 | GOCC\_BLOC\_1\_COMPLEX |  | 13 | -0.40 | -1.00 | 0.491 | 0.767 | 1.000 | 3569 | tags=46%, list=30%, signal=66% |
| 4981 | GOBP\_NEGATIVE\_REGULATION\_OF\_PROTEASOMAL\_UBIQUITIN\_DEPENDENT\_PROTEIN\_CATABOLIC\_PROCESS |  | 27 | -0.34 | -1.00 | 0.485 | 0.767 | 1.000 | 3565 | tags=26%, list=30%, signal=37% |
| 4982 | GOBP\_NEGATIVE\_REGULATION\_OF\_DEFENSE\_RESPONSE |  | 114 | -0.27 | -1.00 | 0.501 | 0.767 | 1.000 | 3846 | tags=37%, list=32%, signal=54% |
| 4983 | GOCC\_HRD1P\_UBIQUITIN\_LIGASE\_ERAD\_L\_COMPLEX |  | 6 | -0.49 | -1.00 | 0.498 | 0.767 | 1.000 | 2242 | tags=33%, list=19%, signal=41% |
| 4984 | GOBP\_REGULATION\_OF\_KERATINOCYTE\_DIFFERENTIATION |  | 21 | -0.35 | -1.00 | 0.486 | 0.768 | 1.000 | 2732 | tags=24%, list=23%, signal=31% |
| 4985 | GOBP\_SCHWANN\_CELL\_PROLIFERATION |  | 6 | -0.49 | -1.00 | 0.485 | 0.768 | 1.000 | 1297 | tags=33%, list=11%, signal=37% |
| 4986 | GOBP\_POSITIVE\_REGULATION\_OF\_PHOSPHOLIPASE\_A2\_ACTIVITY |  | 3 | -0.59 | -1.00 | 0.501 | 0.768 | 1.000 | 2620 | tags=67%, list=22%, signal=85% |
| 4987 | GOBP\_DNA\_TEMPLATED\_TRANSCRIPTION\_INITIATION |  | 175 | -0.26 | -1.00 | 0.506 | 0.768 | 1.000 | 4243 | tags=36%, list=35%, signal=55% |
| 4988 | GOBP\_SUPEROXIDE\_ANION\_GENERATION |  | 20 | -0.36 | -1.00 | 0.479 | 0.768 | 1.000 | 4564 | tags=50%, list=38%, signal=81% |
| 4989 | GOBP\_POSITIVE\_REGULATION\_OF\_TRANSPORTER\_ACTIVITY |  | 72 | -0.29 | -1.00 | 0.498 | 0.768 | 1.000 | 2955 | tags=29%, list=25%, signal=38% |
| 4990 | GOBP\_HISTONE\_H4\_K20\_METHYLATION |  | 8 | -0.45 | -1.00 | 0.490 | 0.768 | 1.000 | 3587 | tags=63%, list=30%, signal=89% |
| 4991 | GOBP\_NEUTROPHIL\_MEDIATED\_KILLING\_OF\_SYMBIONT\_CELL |  | 4 | -0.56 | -1.00 | 0.510 | 0.768 | 1.000 | 5067 | tags=75%, list=42%, signal=130% |
| 4992 | GOBP\_POSITIVE\_REGULATION\_OF\_HAIR\_FOLLICLE\_DEVELOPMENT |  | 7 | -0.47 | -1.00 | 0.495 | 0.768 | 1.000 | 658 | tags=29%, list=5%, signal=30% |
| 4993 | GOBP\_COBALAMIN\_METABOLIC\_PROCESS |  | 13 | -0.40 | -1.00 | 0.487 | 0.768 | 1.000 | 2950 | tags=38%, list=25%, signal=51% |
| 4994 | GOBP\_MULTICELLULAR\_ORGANISMAL\_SIGNALING |  | 103 | -0.28 | -1.00 | 0.501 | 0.768 | 1.000 | 4110 | tags=33%, list=34%, signal=50% |
| 4995 | GOCC\_INTERCELLULAR\_CANALICULUS |  | 4 | -0.55 | -1.00 | 0.497 | 0.767 | 1.000 | 3930 | tags=75%, list=33%, signal=111% |
| 4996 | GOBP\_TRANSEPITHELIAL\_TRANSPORT |  | 23 | -0.35 | -1.00 | 0.482 | 0.767 | 1.000 | 2038 | tags=35%, list=17%, signal=42% |
| 4997 | GOBP\_CHEMOKINE\_C\_X\_C\_MOTIF\_LIGAND\_1\_PRODUCTION |  | 3 | -0.59 | -1.00 | 0.499 | 0.767 | 1.000 | 2322 | tags=67%, list=19%, signal=83% |
| 4998 | GOBP\_POSITIVE\_REGULATION\_OF\_T\_HELPER\_CELL\_DIFFERENTIATION |  | 15 | -0.38 | -1.00 | 0.487 | 0.768 | 1.000 | 2345 | tags=33%, list=20%, signal=41% |
| 4999 | GOCC\_CLATHRIN\_COATED\_ENDOCYTIC\_VESICLE |  | 27 | -0.34 | -1.00 | 0.481 | 0.768 | 1.000 | 4775 | tags=56%, list=40%, signal=92% |
| 5000 | GOBP\_POSITIVE\_REGULATION\_OF\_STRIATED\_MUSCLE\_CELL\_APOPTOTIC\_PROCESS |  | 6 | -0.49 | -1.00 | 0.494 | 0.768 | 1.000 | 3392 | tags=50%, list=28%, signal=70% |
| 5001 | GOCC\_TRANS\_GOLGI\_NETWORK\_TRANSPORT\_VESICLE |  | 20 | -0.36 | -1.00 | 0.480 | 0.768 | 1.000 | 4891 | tags=55%, list=41%, signal=93% |
| 5002 | GOBP\_NEGATIVE\_REGULATION\_OF\_SEQUESTERING\_OF\_TRIGLYCERIDE |  | 4 | -0.55 | -1.00 | 0.499 | 0.768 | 1.000 | 1748 | tags=75%, list=15%, signal=88% |
| 5003 | GOBP\_VASODILATION |  | 25 | -0.34 | -1.00 | 0.487 | 0.768 | 1.000 | 2563 | tags=32%, list=21%, signal=41% |
| 5004 | GOBP\_POSITIVE\_REGULATION\_OF\_NON\_CANONICAL\_WNT\_SIGNALING\_PATHWAY |  | 12 | -0.41 | -1.00 | 0.488 | 0.768 | 1.000 | 3941 | tags=42%, list=33%, signal=62% |
| 5005 | GOBP\_POSITIVE\_REGULATION\_OF\_BLOOD\_PRESSURE |  | 15 | -0.38 | -1.00 | 0.481 | 0.768 | 1.000 | 893 | tags=20%, list=7%, signal=22% |
| 5006 | GOBP\_REGULATION\_OF\_DEVELOPMENTAL\_PIGMENTATION |  | 11 | -0.41 | -1.00 | 0.489 | 0.768 | 1.000 | 4116 | tags=64%, list=34%, signal=97% |
| 5007 | GOBP\_REGULATION\_OF\_ICOSANOID\_SECRETION |  | 12 | -0.41 | -1.00 | 0.486 | 0.768 | 1.000 | 2769 | tags=42%, list=23%, signal=54% |
| 5008 | GOBP\_RESPONSE\_TO\_PEPTIDE |  | 366 | -0.26 | -1.00 | 0.520 | 0.769 | 1.000 | 3527 | tags=36%, list=29%, signal=49% |
| 5009 | GOBP\_PHAGOSOME\_LYSOSOME\_FUSION |  | 8 | -0.45 | -1.00 | 0.489 | 0.769 | 1.000 | 1954 | tags=38%, list=16%, signal=45% |
| 5010 | GOMF\_CATION\_TRANSMEMBRANE\_TRANSPORTER\_ACTIVITY |  | 356 | -0.26 | -1.00 | 0.521 | 0.769 | 1.000 | 1302 | tags=17%, list=11%, signal=19% |
| 5011 | GOMF\_DYNEIN\_HEAVY\_CHAIN\_BINDING |  | 6 | -0.49 | -1.00 | 0.496 | 0.769 | 1.000 | 4518 | tags=50%, list=38%, signal=80% |
| 5012 | GOBP\_INORGANIC\_ION\_TRANSMEMBRANE\_TRANSPORT |  | 495 | -0.25 | -0.99 | 0.528 | 0.769 | 1.000 | 3046 | tags=28%, list=25%, signal=37% |
| 5013 | GOMF\_LIPOPROTEIN\_PARTICLE\_RECEPTOR\_ACTIVITY |  | 13 | -0.40 | -0.99 | 0.488 | 0.769 | 1.000 | 4408 | tags=54%, list=37%, signal=85% |
| 5014 | GOMF\_RETROMER\_COMPLEX\_BINDING |  | 5 | -0.51 | -0.99 | 0.500 | 0.769 | 1.000 | 5536 | tags=80%, list=46%, signal=148% |
| 5015 | GOBP\_CHONDROCYTE\_DEVELOPMENT\_INVOLVED\_IN\_ENDOCHONDRAL\_BONE\_MORPHOGENESIS |  | 6 | -0.49 | -0.99 | 0.500 | 0.769 | 1.000 | 5967 | tags=83%, list=50%, signal=166% |
| 5016 | GOMF\_CYCLIN\_DEPENDENT\_PROTEIN\_KINASE\_ACTIVITY |  | 20 | -0.36 | -0.99 | 0.485 | 0.769 | 1.000 | 5323 | tags=60%, list=44%, signal=108% |
| 5017 | GOMF\_TFIID\_CLASS\_TRANSCRIPTION\_FACTOR\_COMPLEX\_BINDING |  | 6 | -0.49 | -0.99 | 0.495 | 0.769 | 1.000 | 1114 | tags=17%, list=9%, signal=18% |
| 5018 | GOBP\_POSITIVE\_REGULATION\_OF\_PROTEIN\_LOCALIZATION\_TO\_MEMBRANE |  | 87 | -0.28 | -0.99 | 0.504 | 0.769 | 1.000 | 4945 | tags=43%, list=41%, signal=72% |
| 5019 | GOBP\_RESPONSE\_TO\_NITRIC\_OXIDE |  | 15 | -0.38 | -0.99 | 0.499 | 0.769 | 1.000 | 2829 | tags=27%, list=24%, signal=35% |
| 5020 | GOCC\_ZONULA\_ADHERENS |  | 5 | -0.51 | -0.99 | 0.493 | 0.769 | 1.000 | 4033 | tags=60%, list=34%, signal=90% |
| 5021 | GOBP\_NEGATIVE\_REGULATION\_OF\_LYMPHOCYTE\_DIFFERENTIATION |  | 36 | -0.32 | -0.99 | 0.497 | 0.770 | 1.000 | 1951 | tags=25%, list=16%, signal=30% |
| 5022 | GOMF\_DYSTROGLYCAN\_BINDING |  | 9 | -0.43 | -0.99 | 0.494 | 0.771 | 1.000 | 3551 | tags=44%, list=30%, signal=63% |
| 5023 | GOBP\_POSITIVE\_REGULATION\_OF\_AUTOPHAGOSOME\_ASSEMBLY |  | 9 | -0.44 | -0.99 | 0.493 | 0.771 | 1.000 | 6736 | tags=89%, list=56%, signal=202% |
| 5024 | GOMF\_CALMODULIN\_DEPENDENT\_PROTEIN\_KINASE\_ACTIVITY |  | 22 | -0.35 | -0.99 | 0.493 | 0.772 | 1.000 | 4864 | tags=59%, list=40%, signal=99% |
| 5025 | GOMF\_BICARBONATE\_TRANSMEMBRANE\_TRANSPORTER\_ACTIVITY |  | 17 | -0.37 | -0.99 | 0.493 | 0.772 | 1.000 | 2644 | tags=53%, list=22%, signal=68% |
| 5026 | GOBP\_PYRIMIDINE\_NUCLEOSIDE\_TRANSPORT |  | 3 | -0.59 | -0.99 | 0.503 | 0.772 | 1.000 | 1024 | tags=33%, list=9%, signal=36% |
| 5027 | GOBP\_REGULATION\_OF\_SYNAPTIC\_VESICLE\_CLUSTERING |  | 3 | -0.59 | -0.99 | 0.505 | 0.772 | 1.000 | 3392 | tags=67%, list=28%, signal=93% |
| 5028 | GOMF\_PYRIMIDINE\_NUCLEOSIDE\_TRANSMEMBRANE\_TRANSPORTER\_ACTIVITY |  | 3 | -0.59 | -0.99 | 0.506 | 0.772 | 1.000 | 1024 | tags=33%, list=9%, signal=36% |
| 5029 | GOCC\_CALCINEURIN\_COMPLEX |  | 4 | -0.55 | -0.99 | 0.514 | 0.772 | 1.000 | 4084 | tags=50%, list=34%, signal=76% |
| 5030 | GOBP\_PITUITARY\_GLAND\_DEVELOPMENT |  | 24 | -0.35 | -0.99 | 0.490 | 0.772 | 1.000 | 3374 | tags=38%, list=28%, signal=52% |
| 5031 | GOBP\_SPERMATID\_NUCLEUS\_DIFFERENTIATION |  | 11 | -0.41 | -0.99 | 0.493 | 0.772 | 1.000 | 4298 | tags=45%, list=36%, signal=71% |
| 5032 | GOBP\_MAMMARY\_GLAND\_MORPHOGENESIS |  | 30 | -0.33 | -0.99 | 0.498 | 0.772 | 1.000 | 1146 | tags=17%, list=10%, signal=18% |
| 5033 | GOMF\_SIGNAL\_RECOGNITION\_PARTICLE\_BINDING |  | 7 | -0.47 | -0.99 | 0.500 | 0.772 | 1.000 | 4890 | tags=43%, list=41%, signal=72% |
| 5034 | GOMF\_VOLTAGE\_GATED\_POTASSIUM\_CHANNEL\_ACTIVITY |  | 39 | -0.31 | -0.99 | 0.493 | 0.773 | 1.000 | 1627 | tags=21%, list=14%, signal=24% |
| 5035 | GOMF\_ERBB\_3\_CLASS\_RECEPTOR\_BINDING |  | 5 | -0.51 | -0.99 | 0.495 | 0.773 | 1.000 | 747 | tags=20%, list=6%, signal=21% |
| 5036 | GOBP\_HISTONE\_H3\_K23\_ACETYLATION |  | 5 | -0.52 | -0.99 | 0.503 | 0.773 | 1.000 | 5045 | tags=60%, list=42%, signal=103% |
| 5037 | GOBP\_REGULATION\_OF\_DEFENSE\_RESPONSE\_TO\_VIRUS |  | 38 | -0.31 | -0.99 | 0.483 | 0.773 | 1.000 | 3447 | tags=42%, list=29%, signal=59% |
| 5038 | GOBP\_POSITIVE\_REGULATION\_OF\_I\_KAPPAB\_PHOSPHORYLATION |  | 5 | -0.52 | -0.99 | 0.505 | 0.773 | 1.000 | 3613 | tags=60%, list=30%, signal=86% |
| 5039 | GOBP\_CELLULAR\_RESPONSE\_TO\_PROSTAGLANDIN\_E\_STIMULUS |  | 13 | -0.39 | -0.99 | 0.485 | 0.773 | 1.000 | 3613 | tags=54%, list=30%, signal=77% |
| 5040 | GOBP\_DIENCEPHALON\_DEVELOPMENT |  | 42 | -0.31 | -0.99 | 0.494 | 0.773 | 1.000 | 3565 | tags=33%, list=30%, signal=47% |
| 5041 | GOBP\_REGULATION\_OF\_RNA\_BINDING |  | 9 | -0.44 | -0.99 | 0.502 | 0.773 | 1.000 | 5307 | tags=44%, list=44%, signal=80% |
| 5042 | GOBP\_HEPARIN\_METABOLIC\_PROCESS |  | 11 | -0.41 | -0.99 | 0.487 | 0.773 | 1.000 | 5140 | tags=55%, list=43%, signal=95% |
| 5043 | GOBP\_HETEROCHROMATIN\_ORGANIZATION |  | 40 | -0.31 | -0.99 | 0.492 | 0.774 | 1.000 | 2656 | tags=30%, list=22%, signal=38% |
| 5044 | GOCC\_NLRP3\_INFLAMMASOME\_COMPLEX |  | 5 | -0.51 | -0.99 | 0.504 | 0.774 | 1.000 | 5278 | tags=80%, list=44%, signal=143% |
| 5045 | GOCC\_PLATELET\_DENSE\_GRANULE |  | 11 | -0.41 | -0.99 | 0.494 | 0.774 | 1.000 | 4268 | tags=73%, list=36%, signal=113% |
| 5046 | GOBP\_RESPONSE\_TO\_ETHANOL |  | 80 | -0.28 | -0.99 | 0.506 | 0.774 | 1.000 | 2456 | tags=26%, list=20%, signal=33% |
| 5047 | GOBP\_PODOSOME\_ASSEMBLY |  | 11 | -0.41 | -0.99 | 0.493 | 0.774 | 1.000 | 4958 | tags=64%, list=41%, signal=108% |
| 5048 | GOBP\_MITOTIC\_CYTOKINESIS |  | 59 | -0.29 | -0.99 | 0.498 | 0.775 | 1.000 | 4165 | tags=39%, list=35%, signal=59% |
| 5049 | GOBP\_NEGATIVE\_REGULATION\_OF\_INTERLEUKIN\_2\_PRODUCTION |  | 19 | -0.36 | -0.99 | 0.489 | 0.776 | 1.000 | 2261 | tags=32%, list=19%, signal=39% |
| 5050 | GOCC\_CYTOPLASMIC\_SIDE\_OF\_LYSOSOMAL\_MEMBRANE |  | 5 | -0.51 | -0.99 | 0.510 | 0.776 | 1.000 | 1620 | tags=40%, list=13%, signal=46% |
| 5051 | GOBP\_POSITIVE\_REGULATION\_OF\_AUTOPHAGOSOME\_MATURATION |  | 5 | -0.51 | -0.99 | 0.503 | 0.776 | 1.000 | 3967 | tags=60%, list=33%, signal=90% |
| 5052 | GOCC\_CYCLIN\_DEPENDENT\_PROTEIN\_KINASE\_HOLOENZYME\_COMPLEX |  | 35 | -0.32 | -0.99 | 0.499 | 0.776 | 1.000 | 4358 | tags=43%, list=36%, signal=67% |
| 5053 | GOMF\_PROTEIN\_PHOSPHATASE\_ACTIVATOR\_ACTIVITY |  | 8 | -0.45 | -0.99 | 0.488 | 0.776 | 1.000 | 5501 | tags=63%, list=46%, signal=115% |
| 5054 | GOBP\_SKIN\_DEVELOPMENT |  | 159 | -0.26 | -0.99 | 0.515 | 0.776 | 1.000 | 3318 | tags=29%, list=28%, signal=39% |
| 5055 | GOBP\_NUCLEOSIDE\_PHOSPHATE\_CATABOLIC\_PROCESS |  | 62 | -0.29 | -0.99 | 0.502 | 0.776 | 1.000 | 3094 | tags=31%, list=26%, signal=41% |
| 5056 | GOMF\_SEROTONIN\_RECEPTOR\_ACTIVITY |  | 13 | -0.39 | -0.99 | 0.497 | 0.776 | 1.000 | 929 | tags=23%, list=8%, signal=25% |
| 5057 | GOBP\_MYOBLAST\_PROLIFERATION |  | 13 | -0.39 | -0.99 | 0.501 | 0.776 | 1.000 | 1838 | tags=31%, list=15%, signal=36% |
| 5058 | GOBP\_REGULATION\_OF\_MORPHOGENESIS\_OF\_AN\_EPITHELIUM |  | 38 | -0.31 | -0.99 | 0.501 | 0.776 | 1.000 | 2313 | tags=24%, list=19%, signal=29% |
| 5059 | GOMF\_HISTONE\_METHYLTRANSFERASE\_ACTIVITY\_H3\_K36\_SPECIFIC |  | 7 | -0.47 | -0.99 | 0.504 | 0.776 | 1.000 | 2630 | tags=43%, list=22%, signal=55% |
| 5060 | GOBP\_REGULATION\_OF\_MYELINATION |  | 31 | -0.33 | -0.99 | 0.498 | 0.777 | 1.000 | 5437 | tags=58%, list=45%, signal=106% |
| 5061 | GOBP\_REGULATION\_OF\_CARDIAC\_MUSCLE\_CONTRACTION\_BY\_REGULATION\_OF\_THE\_RELEASE\_OF\_SEQUESTERED\_CALCIUM\_ION |  | 13 | -0.39 | -0.99 | 0.497 | 0.777 | 1.000 | 4627 | tags=38%, list=39%, signal=62% |
| 5062 | GOMF\_ALDO\_KETO\_REDUCTASE\_NADP\_ACTIVITY |  | 16 | -0.38 | -0.99 | 0.498 | 0.777 | 1.000 | 2163 | tags=31%, list=18%, signal=38% |
| 5063 | GOBP\_EPITHELIAL\_CELL\_DIFFERENTIATION |  | 393 | -0.25 | -0.99 | 0.543 | 0.778 | 1.000 | 4205 | tags=38%, list=35%, signal=57% |
| 5064 | GOBP\_MICROGLIAL\_CELL\_MIGRATION |  | 6 | -0.49 | -0.99 | 0.502 | 0.778 | 1.000 | 4317 | tags=50%, list=36%, signal=78% |
| 5065 | GOBP\_POSITIVE\_REGULATION\_OF\_CELL\_CYCLE |  | 275 | -0.26 | -0.99 | 0.530 | 0.778 | 1.000 | 4201 | tags=35%, list=35%, signal=53% |
| 5066 | GOBP\_NUCLEOTIDE\_EXCISION\_REPAIR\_PREINCISION\_COMPLEX\_STABILIZATION |  | 18 | -0.36 | -0.99 | 0.496 | 0.778 | 1.000 | 5351 | tags=72%, list=45%, signal=130% |
| 5067 | GOBP\_REGULATION\_OF\_CATECHOLAMINE\_METABOLIC\_PROCESS |  | 8 | -0.45 | -0.99 | 0.510 | 0.779 | 1.000 | 2405 | tags=38%, list=20%, signal=47% |
| 5068 | GOBP\_PHENOL\_CONTAINING\_COMPOUND\_CATABOLIC\_PROCESS |  | 7 | -0.47 | -0.99 | 0.506 | 0.779 | 1.000 | 1924 | tags=43%, list=16%, signal=51% |
| 5069 | GOBP\_SALIVA\_SECRETION |  | 5 | -0.51 | -0.99 | 0.509 | 0.779 | 1.000 | 1146 | tags=40%, list=10%, signal=44% |
| 5070 | GOBP\_CELL\_FATE\_COMMITMENT\_INVOLVED\_IN\_FORMATION\_OF\_PRIMARY\_GERM\_LAYER |  | 16 | -0.38 | -0.99 | 0.498 | 0.779 | 1.000 | 4677 | tags=50%, list=39%, signal=82% |
| 5071 | GOBP\_NEGATIVE\_REGULATION\_OF\_CARDIAC\_MUSCLE\_ADAPTATION |  | 6 | -0.49 | -0.99 | 0.508 | 0.779 | 1.000 | 2267 | tags=50%, list=19%, signal=62% |
| 5072 | GOMF\_NITRIC\_OXIDE\_SYNTHASE\_BINDING |  | 10 | -0.42 | -0.99 | 0.504 | 0.779 | 1.000 | 3909 | tags=40%, list=33%, signal=59% |
| 5073 | GOMF\_PHOSPHATIDYLCHOLINE\_FLOPPASE\_ACTIVITY |  | 3 | -0.59 | -0.99 | 0.517 | 0.779 | 1.000 | 2752 | tags=67%, list=23%, signal=86% |
| 5074 | GOBP\_REGULATION\_OF\_RESPONSE\_TO\_WOUNDING |  | 104 | -0.27 | -0.99 | 0.521 | 0.780 | 1.000 | 3027 | tags=29%, list=25%, signal=38% |
| 5075 | GOBP\_REGULATION\_OF\_SKELETAL\_MUSCLE\_CELL\_PROLIFERATION |  | 9 | -0.43 | -0.99 | 0.500 | 0.780 | 1.000 | 3565 | tags=56%, list=30%, signal=79% |
| 5076 | GOBP\_REGULATION\_OF\_SREBP\_SIGNALING\_PATHWAY |  | 5 | -0.51 | -0.99 | 0.517 | 0.780 | 1.000 | 5628 | tags=80%, list=47%, signal=150% |
| 5077 | GOMF\_TRANSCRIPTION\_COACTIVATOR\_BINDING |  | 21 | -0.35 | -0.99 | 0.507 | 0.781 | 1.000 | 2708 | tags=33%, list=23%, signal=43% |
| 5078 | GOBP\_CATION\_TRANSMEMBRANE\_TRANSPORT |  | 516 | -0.25 | -0.99 | 0.559 | 0.781 | 1.000 | 3046 | tags=28%, list=25%, signal=36% |
| 5079 | GOBP\_NEURON\_NEURON\_SYNAPTIC\_TRANSMISSION |  | 3 | -0.59 | -0.99 | 0.514 | 0.781 | 1.000 | 3392 | tags=67%, list=28%, signal=93% |
| 5080 | GOBP\_DIAPEDESIS |  | 6 | -0.48 | -0.99 | 0.513 | 0.782 | 1.000 | 2011 | tags=33%, list=17%, signal=40% |
| 5081 | GOMF\_TRANSITION\_METAL\_ION\_TRANSMEMBRANE\_TRANSPORTER\_ACTIVITY |  | 25 | -0.34 | -0.99 | 0.503 | 0.782 | 1.000 | 5420 | tags=56%, list=45%, signal=102% |
| 5082 | GOBP\_GLOMERULAR\_EPITHELIUM\_DEVELOPMENT |  | 17 | -0.37 | -0.99 | 0.498 | 0.782 | 1.000 | 1432 | tags=18%, list=12%, signal=20% |
| 5083 | GOBP\_RESPONSE\_TO\_INTERFERON\_GAMMA |  | 101 | -0.27 | -0.99 | 0.508 | 0.781 | 1.000 | 1845 | tags=22%, list=15%, signal=26% |
| 5084 | GOBP\_REGULATION\_OF\_DIGESTIVE\_SYSTEM\_PROCESS |  | 21 | -0.35 | -0.99 | 0.503 | 0.782 | 1.000 | 2895 | tags=38%, list=24%, signal=50% |
| 5085 | GOBP\_REGULATION\_OF\_TISSUE\_REMODELING |  | 51 | -0.30 | -0.99 | 0.516 | 0.782 | 1.000 | 2416 | tags=27%, list=20%, signal=34% |
| 5086 | GOMF\_URIDINE\_TRANSMEMBRANE\_TRANSPORTER\_ACTIVITY |  | 3 | -0.59 | -0.99 | 0.516 | 0.782 | 1.000 | 1024 | tags=33%, list=9%, signal=36% |
| 5087 | GOBP\_POSTSYNAPTIC\_SPECIALIZATION\_ASSEMBLY |  | 12 | -0.40 | -0.99 | 0.493 | 0.782 | 1.000 | 5068 | tags=75%, list=42%, signal=130% |
| 5088 | GOMF\_CALCIUM\_DEPENDENT\_CYSTEINE\_TYPE\_ENDOPEPTIDASE\_ACTIVITY |  | 11 | -0.41 | -0.99 | 0.513 | 0.782 | 1.000 | 2070 | tags=27%, list=17%, signal=33% |
| 5089 | GOBP\_REGULATION\_OF\_SMAD\_PROTEIN\_SIGNAL\_TRANSDUCTION |  | 16 | -0.37 | -0.99 | 0.497 | 0.782 | 1.000 | 4770 | tags=63%, list=40%, signal=104% |
| 5090 | GOCC\_VESICLE\_MEMBRANE |  | 524 | -0.25 | -0.99 | 0.559 | 0.782 | 1.000 | 4401 | tags=40%, list=37%, signal=61% |
| 5091 | GOBP\_SEROTONIN\_RECEPTOR\_SIGNALING\_PATHWAY |  | 13 | -0.39 | -0.99 | 0.501 | 0.782 | 1.000 | 929 | tags=23%, list=8%, signal=25% |
| 5092 | GOBP\_NEGATIVE\_REGULATION\_OF\_VASCULATURE\_DEVELOPMENT |  | 63 | -0.29 | -0.98 | 0.513 | 0.782 | 1.000 | 3806 | tags=41%, list=32%, signal=60% |
| 5093 | GOBP\_REGULATION\_OF\_INTRACELLULAR\_STEROID\_HORMONE\_RECEPTOR\_SIGNALING\_PATHWAY |  | 56 | -0.29 | -0.98 | 0.516 | 0.782 | 1.000 | 4512 | tags=46%, list=38%, signal=74% |
| 5094 | GOMF\_PHOSPHOLIPASE\_INHIBITOR\_ACTIVITY |  | 6 | -0.49 | -0.98 | 0.518 | 0.782 | 1.000 | 1072 | tags=33%, list=9%, signal=37% |
| 5095 | GOBP\_REGULATION\_OF\_SMOOTHENED\_SIGNALING\_PATHWAY\_INVOLVED\_IN\_DORSAL\_VENTRAL\_NEURAL\_TUBE\_PATTERNING |  | 7 | -0.47 | -0.98 | 0.516 | 0.781 | 1.000 | 5085 | tags=71%, list=42%, signal=124% |
| 5096 | GOMF\_PROMOTER\_SPECIFIC\_CHROMATIN\_BINDING |  | 36 | -0.31 | -0.98 | 0.506 | 0.781 | 1.000 | 3615 | tags=39%, list=30%, signal=55% |
| 5097 | GOBP\_ARP2\_3\_COMPLEX\_MEDIATED\_ACTIN\_NUCLEATION |  | 28 | -0.33 | -0.98 | 0.508 | 0.782 | 1.000 | 3797 | tags=39%, list=32%, signal=57% |
| 5098 | GOMF\_GALACTOSYLTRANSFERASE\_ACTIVITY |  | 15 | -0.38 | -0.98 | 0.508 | 0.782 | 1.000 | 402 | tags=20%, list=3%, signal=21% |
| 5099 | GOBP\_LAYER\_FORMATION\_IN\_CEREBRAL\_CORTEX |  | 7 | -0.47 | -0.98 | 0.507 | 0.782 | 1.000 | 3004 | tags=43%, list=25%, signal=57% |
| 5100 | GOCC\_INTEGRAL\_COMPONENT\_OF\_CYTOPLASMIC\_SIDE\_OF\_ENDOPLASMIC\_RETICULUM\_MEMBRANE |  | 5 | -0.51 | -0.98 | 0.514 | 0.782 | 1.000 | 5330 | tags=80%, list=44%, signal=144% |
| 5101 | GOBP\_ORGANOPHOSPHATE\_CATABOLIC\_PROCESS |  | 117 | -0.27 | -0.98 | 0.529 | 0.782 | 1.000 | 2930 | tags=28%, list=24%, signal=37% |
| 5102 | GOBP\_ION\_HOMEOSTASIS |  | 493 | -0.25 | -0.98 | 0.554 | 0.782 | 1.000 | 3046 | tags=30%, list=25%, signal=38% |
| 5103 | GOBP\_CD4\_POSITIVE\_ALPHA\_BETA\_T\_CELL\_PROLIFERATION |  | 7 | -0.46 | -0.98 | 0.510 | 0.782 | 1.000 | 3163 | tags=43%, list=26%, signal=58% |
| 5104 | GOMF\_MISMATCHED\_DNA\_BINDING |  | 13 | -0.39 | -0.98 | 0.494 | 0.783 | 1.000 | 6485 | tags=85%, list=54%, signal=184% |
| 5105 | GOBP\_MAINTENANCE\_OF\_ORGANELLE\_LOCATION |  | 7 | -0.46 | -0.98 | 0.499 | 0.783 | 1.000 | 5219 | tags=71%, list=43%, signal=126% |
| 5106 | GOBP\_POSITIVE\_REGULATION\_OF\_INTERLEUKIN\_4\_PRODUCTION |  | 14 | -0.39 | -0.98 | 0.505 | 0.783 | 1.000 | 3155 | tags=43%, list=26%, signal=58% |
| 5107 | GOBP\_TETRAHYDROFOLATE\_INTERCONVERSION |  | 9 | -0.43 | -0.98 | 0.508 | 0.783 | 1.000 | 2580 | tags=33%, list=21%, signal=42% |
| 5108 | GOBP\_NEGATIVE\_REGULATION\_OF\_INTERLEUKIN\_13\_PRODUCTION |  | 3 | -0.59 | -0.98 | 0.515 | 0.783 | 1.000 | 1788 | tags=67%, list=15%, signal=78% |
| 5109 | GOBP\_CD4\_POSITIVE\_OR\_CD8\_POSITIVE\_ALPHA\_BETA\_T\_CELL\_LINEAGE\_COMMITMENT |  | 12 | -0.40 | -0.98 | 0.506 | 0.782 | 1.000 | 2308 | tags=33%, list=19%, signal=41% |
| 5110 | GOBP\_RESPONSE\_TO\_INSULIN |  | 203 | -0.26 | -0.98 | 0.541 | 0.782 | 1.000 | 3692 | tags=42%, list=31%, signal=60% |
| 5111 | GOBP\_LACRIMAL\_GLAND\_DEVELOPMENT |  | 5 | -0.51 | -0.98 | 0.516 | 0.782 | 1.000 | 1989 | tags=40%, list=17%, signal=48% |
| 5112 | GOBP\_NEUROMUSCULAR\_PROCESS\_CONTROLLING\_BALANCE |  | 23 | -0.34 | -0.98 | 0.504 | 0.782 | 1.000 | 5463 | tags=57%, list=45%, signal=103% |
| 5113 | GOMF\_HISTAMINE\_RECEPTOR\_ACTIVITY |  | 3 | -0.59 | -0.98 | 0.517 | 0.782 | 1.000 | 517 | tags=33%, list=4%, signal=35% |
| 5114 | GOBP\_HINDGUT\_DEVELOPMENT |  | 6 | -0.48 | -0.98 | 0.509 | 0.783 | 1.000 | 556 | tags=17%, list=5%, signal=17% |
| 5115 | GOMF\_SPHINGOSINE\_N\_ACYLTRANSFERASE\_ACTIVITY |  | 6 | -0.48 | -0.98 | 0.513 | 0.783 | 1.000 | 335 | tags=33%, list=3%, signal=34% |
| 5116 | GOBP\_DEOXYRIBONUCLEOSIDE\_TRIPHOSPHATE\_CATABOLIC\_PROCESS |  | 6 | -0.48 | -0.98 | 0.511 | 0.783 | 1.000 | 3094 | tags=50%, list=26%, signal=67% |
| 5117 | GOBP\_POLYOL\_BIOSYNTHETIC\_PROCESS |  | 46 | -0.30 | -0.98 | 0.509 | 0.782 | 1.000 | 4283 | tags=46%, list=36%, signal=71% |
| 5118 | GOBP\_FACIAL\_NERVE\_MORPHOGENESIS |  | 5 | -0.51 | -0.98 | 0.521 | 0.782 | 1.000 | 4739 | tags=60%, list=39%, signal=99% |
| 5119 | GOBP\_NEGATIVE\_REGULATION\_OF\_TRANSPORTER\_ACTIVITY |  | 52 | -0.30 | -0.98 | 0.516 | 0.783 | 1.000 | 4177 | tags=38%, list=35%, signal=59% |
| 5120 | GOMF\_PROTEIN\_ADP\_RIBOSYLASE\_ACTIVITY |  | 12 | -0.40 | -0.98 | 0.502 | 0.783 | 1.000 | 1704 | tags=33%, list=14%, signal=39% |
| 5121 | GOBP\_HISTAMINE\_METABOLIC\_PROCESS |  | 5 | -0.51 | -0.98 | 0.512 | 0.784 | 1.000 | 5948 | tags=100%, list=50%, signal=198% |
| 5122 | GOBP\_PROTEIN\_LOCALIZATION\_TO\_PHAGOPHORE\_ASSEMBLY\_SITE |  | 9 | -0.43 | -0.98 | 0.505 | 0.784 | 1.000 | 2361 | tags=33%, list=20%, signal=41% |
| 5123 | GOMF\_RNA\_POLYMERASE\_II\_C\_TERMINAL\_DOMAIN\_PHOSPHOSERINE\_BINDING |  | 5 | -0.51 | -0.98 | 0.523 | 0.783 | 1.000 | 5010 | tags=80%, list=42%, signal=137% |
| 5124 | GOBP\_REGULATION\_OF\_SMOOTH\_MUSCLE\_CELL\_DIFFERENTIATION |  | 26 | -0.34 | -0.98 | 0.509 | 0.783 | 1.000 | 481 | tags=15%, list=4%, signal=16% |
| 5125 | GOBP\_NEGATIVE\_REGULATION\_OF\_TYPE\_I\_INTERFERON\_MEDIATED\_SIGNALING\_PATHWAY |  | 11 | -0.41 | -0.98 | 0.498 | 0.784 | 1.000 | 4833 | tags=55%, list=40%, signal=91% |
| 5126 | GOMF\_METHYL\_CPG\_BINDING |  | 15 | -0.38 | -0.98 | 0.507 | 0.784 | 1.000 | 2828 | tags=47%, list=24%, signal=61% |
| 5127 | GOBP\_MYOFIBRIL\_ASSEMBLY |  | 33 | -0.32 | -0.98 | 0.512 | 0.784 | 1.000 | 3286 | tags=30%, list=27%, signal=42% |
| 5128 | GOBP\_POSITIVE\_REGULATION\_OF\_HISTONE\_H3\_K27\_METHYLATION |  | 3 | -0.59 | -0.98 | 0.522 | 0.784 | 1.000 | 3492 | tags=67%, list=29%, signal=94% |
| 5129 | GOBP\_I\_KAPPAB\_PHOSPHORYLATION |  | 16 | -0.37 | -0.98 | 0.512 | 0.784 | 1.000 | 3613 | tags=56%, list=30%, signal=80% |
| 5130 | GOBP\_POSITIVE\_REGULATION\_OF\_T\_CELL\_CYTOKINE\_PRODUCTION |  | 15 | -0.38 | -0.98 | 0.498 | 0.784 | 1.000 | 1781 | tags=33%, list=15%, signal=39% |
| 5131 | GOCC\_INTERSTITIAL\_MATRIX |  | 6 | -0.49 | -0.98 | 0.517 | 0.784 | 1.000 | 350 | tags=17%, list=3%, signal=17% |
| 5132 | GOBP\_PURINE\_RIBONUCLEOSIDE\_MONOPHOSPHATE\_CATABOLIC\_PROCESS |  | 4 | -0.54 | -0.98 | 0.514 | 0.784 | 1.000 | 1107 | tags=50%, list=9%, signal=55% |
| 5133 | GOBP\_NEGATIVE\_REGULATION\_OF\_IMMUNE\_RESPONSE |  | 77 | -0.28 | -0.98 | 0.524 | 0.784 | 1.000 | 1951 | tags=26%, list=16%, signal=31% |
| 5134 | GOBP\_HISTONE\_H3\_K9\_TRIMETHYLATION |  | 7 | -0.46 | -0.98 | 0.517 | 0.784 | 1.000 | 3106 | tags=57%, list=26%, signal=77% |
| 5135 | GOMF\_PHOSPHATIDYLINOSITOL\_3\_PHOSPHATE\_BINDING |  | 33 | -0.32 | -0.98 | 0.512 | 0.784 | 1.000 | 5260 | tags=45%, list=44%, signal=81% |
| 5136 | GOBP\_REGULATION\_OF\_B\_CELL\_MEDIATED\_IMMUNITY |  | 32 | -0.32 | -0.98 | 0.501 | 0.784 | 1.000 | 2639 | tags=31%, list=22%, signal=40% |
| 5137 | GOBP\_POSITIVE\_REGULATION\_OF\_INSULIN\_SECRETION |  | 44 | -0.31 | -0.98 | 0.514 | 0.785 | 1.000 | 3147 | tags=34%, list=26%, signal=46% |
| 5138 | GOBP\_POSITIVE\_REGULATION\_OF\_DEACETYLASE\_ACTIVITY |  | 5 | -0.51 | -0.98 | 0.523 | 0.785 | 1.000 | 5904 | tags=100%, list=49%, signal=197% |
| 5139 | GOBP\_DEVELOPMENTAL\_INDUCTION |  | 20 | -0.35 | -0.98 | 0.510 | 0.785 | 1.000 | 1459 | tags=25%, list=12%, signal=28% |
| 5140 | GOBP\_RESPONSE\_TO\_PROSTAGLANDIN\_E |  | 18 | -0.36 | -0.98 | 0.503 | 0.785 | 1.000 | 4692 | tags=56%, list=39%, signal=91% |
| 5141 | GOMF\_LYS48\_SPECIFIC\_DEUBIQUITINASE\_ACTIVITY |  | 7 | -0.46 | -0.98 | 0.522 | 0.786 | 1.000 | 731 | tags=14%, list=6%, signal=15% |
| 5142 | GOBP\_TUMOR\_NECROSIS\_FACTOR\_MEDIATED\_SIGNALING\_PATHWAY |  | 114 | -0.27 | -0.98 | 0.536 | 0.786 | 1.000 | 2606 | tags=22%, list=22%, signal=28% |
| 5143 | GOBP\_REGULATION\_OF\_INTRACELLULAR\_ESTROGEN\_RECEPTOR\_SIGNALING\_PATHWAY |  | 31 | -0.32 | -0.98 | 0.503 | 0.786 | 1.000 | 3761 | tags=42%, list=31%, signal=61% |
| 5144 | GOBP\_FAT\_CELL\_PROLIFERATION |  | 6 | -0.48 | -0.98 | 0.524 | 0.786 | 1.000 | 1981 | tags=33%, list=16%, signal=40% |
| 5145 | GOMF\_PHOSPHOSERINE\_RESIDUE\_BINDING |  | 9 | -0.43 | -0.98 | 0.506 | 0.786 | 1.000 | 5010 | tags=56%, list=42%, signal=95% |
| 5146 | GOBP\_CELLULAR\_RESPONSE\_TO\_RETINOIC\_ACID |  | 37 | -0.31 | -0.98 | 0.507 | 0.786 | 1.000 | 3195 | tags=32%, list=27%, signal=44% |
| 5147 | GOCC\_ER\_TO\_GOLGI\_TRANSPORT\_VESICLE\_MEMBRANE |  | 37 | -0.31 | -0.98 | 0.515 | 0.786 | 1.000 | 4239 | tags=38%, list=35%, signal=58% |
| 5148 | GOBP\_POSITIVE\_REGULATION\_OF\_BONE\_MINERALIZATION |  | 26 | -0.33 | -0.98 | 0.513 | 0.787 | 1.000 | 3269 | tags=42%, list=27%, signal=58% |
| 5149 | GOBP\_NEGATIVE\_REGULATION\_OF\_ANOIKIS |  | 13 | -0.39 | -0.98 | 0.507 | 0.788 | 1.000 | 1092 | tags=15%, list=9%, signal=17% |
| 5150 | GOBP\_NODAL\_SIGNALING\_PATHWAY |  | 7 | -0.46 | -0.98 | 0.511 | 0.788 | 1.000 | 3657 | tags=43%, list=30%, signal=62% |
| 5151 | GOBP\_PULMONARY\_ARTERY\_MORPHOGENESIS |  | 5 | -0.51 | -0.98 | 0.518 | 0.788 | 1.000 | 3314 | tags=60%, list=28%, signal=83% |
| 5152 | GOBP\_ORGANONITROGEN\_COMPOUND\_CATABOLIC\_PROCESS |  | 980 | -0.25 | -0.98 | 0.610 | 0.788 | 1.000 | 4037 | tags=33%, list=34%, signal=46% |
| 5153 | GOBP\_PROSTATE\_GLAND\_GROWTH |  | 9 | -0.43 | -0.98 | 0.509 | 0.788 | 1.000 | 3565 | tags=44%, list=30%, signal=63% |
| 5154 | GOBP\_CARDIAC\_LEFT\_VENTRICLE\_MORPHOGENESIS |  | 11 | -0.40 | -0.98 | 0.510 | 0.788 | 1.000 | 3314 | tags=27%, list=28%, signal=38% |
| 5155 | GOBP\_MITOCHONDRIAL\_DNA\_REPAIR |  | 5 | -0.51 | -0.98 | 0.523 | 0.789 | 1.000 | 4773 | tags=80%, list=40%, signal=133% |
| 5156 | GOMF\_DEAD\_H\_BOX\_RNA\_HELICASE\_BINDING |  | 7 | -0.46 | -0.98 | 0.518 | 0.789 | 1.000 | 6101 | tags=86%, list=51%, signal=174% |
| 5157 | GOBP\_PROXIMAL\_TUBULE\_DEVELOPMENT |  | 5 | -0.51 | -0.98 | 0.526 | 0.789 | 1.000 | 5289 | tags=80%, list=44%, signal=143% |
| 5158 | GOBP\_AUTOPHAGY\_OF\_NUCLEUS |  | 11 | -0.40 | -0.98 | 0.512 | 0.789 | 1.000 | 3675 | tags=45%, list=31%, signal=65% |
| 5159 | GOBP\_EMBRYONIC\_HEART\_TUBE\_DEVELOPMENT |  | 49 | -0.30 | -0.98 | 0.520 | 0.789 | 1.000 | 2629 | tags=22%, list=22%, signal=29% |
| 5160 | GOBP\_PRIMARY\_ALCOHOL\_BIOSYNTHETIC\_PROCESS |  | 9 | -0.43 | -0.98 | 0.518 | 0.789 | 1.000 | 2059 | tags=44%, list=17%, signal=54% |
| 5161 | GOMF\_SODIUM\_PROTON\_ANTIPORTER\_ACTIVITY |  | 9 | -0.43 | -0.98 | 0.516 | 0.789 | 1.000 | 654 | tags=33%, list=5%, signal=35% |
| 5162 | GOBP\_REGULATION\_OF\_ATRIAL\_CARDIAC\_MUSCLE\_CELL\_MEMBRANE\_REPOLARIZATION |  | 4 | -0.54 | -0.98 | 0.528 | 0.789 | 1.000 | 69 | tags=25%, list=1%, signal=25% |
| 5163 | GOBP\_IRE1\_MEDIATED\_UNFOLDED\_PROTEIN\_RESPONSE |  | 54 | -0.29 | -0.98 | 0.525 | 0.789 | 1.000 | 5279 | tags=52%, list=44%, signal=92% |
| 5164 | GOCC\_MISMATCH\_REPAIR\_COMPLEX |  | 7 | -0.46 | -0.98 | 0.515 | 0.789 | 1.000 | 6485 | tags=100%, list=54%, signal=217% |
| 5165 | GOBP\_URONIC\_ACID\_METABOLIC\_PROCESS |  | 9 | -0.43 | -0.98 | 0.514 | 0.789 | 1.000 | 3027 | tags=56%, list=25%, signal=74% |
| 5166 | GOBP\_RESPONSE\_TO\_OXYGEN\_LEVELS |  | 279 | -0.25 | -0.98 | 0.569 | 0.789 | 1.000 | 3331 | tags=28%, list=28%, signal=38% |
| 5167 | GOBP\_POSITIVE\_REGULATION\_OF\_TRANSCRIPTION\_FROM\_RNA\_POLYMERASE\_II\_PROMOTER\_IN\_RESPONSE\_TO\_HYPOXIA |  | 5 | -0.51 | -0.98 | 0.524 | 0.789 | 1.000 | 3196 | tags=40%, list=27%, signal=54% |
| 5168 | GOBP\_REGULATION\_OF\_POTASSIUM\_ION\_TRANSMEMBRANE\_TRANSPORTER\_ACTIVITY |  | 34 | -0.32 | -0.98 | 0.516 | 0.789 | 1.000 | 4347 | tags=50%, list=36%, signal=78% |
| 5169 | GOBP\_ORGANELLE\_ASSEMBLY |  | 611 | -0.25 | -0.98 | 0.600 | 0.789 | 1.000 | 3718 | tags=30%, list=31%, signal=41% |
| 5170 | GOBP\_FOREGUT\_MORPHOGENESIS |  | 6 | -0.48 | -0.98 | 0.518 | 0.789 | 1.000 | 3565 | tags=50%, list=30%, signal=71% |
| 5171 | GOCC\_SECRETORY\_GRANULE\_MEMBRANE |  | 197 | -0.26 | -0.98 | 0.560 | 0.789 | 1.000 | 4458 | tags=39%, list=37%, signal=61% |
| 5172 | GOBP\_REGULATION\_OF\_ANIMAL\_ORGAN\_MORPHOGENESIS |  | 121 | -0.27 | -0.98 | 0.546 | 0.789 | 1.000 | 2300 | tags=17%, list=19%, signal=21% |
| 5173 | GOBP\_NEGATIVE\_REGULATION\_OF\_CELLULAR\_PROTEIN\_LOCALIZATION |  | 88 | -0.28 | -0.98 | 0.532 | 0.789 | 1.000 | 4369 | tags=38%, list=36%, signal=59% |
| 5174 | GOBP\_G1\_TO\_G0\_TRANSITION |  | 5 | -0.50 | -0.98 | 0.523 | 0.789 | 1.000 | 5011 | tags=80%, list=42%, signal=137% |
| 5175 | GOMF\_ATP\_GATED\_ION\_CHANNEL\_ACTIVITY |  | 8 | -0.45 | -0.98 | 0.518 | 0.789 | 1.000 | 4484 | tags=50%, list=37%, signal=80% |
| 5176 | GOBP\_REGULATION\_OF\_SECONDARY\_METABOLIC\_PROCESS |  | 8 | -0.44 | -0.98 | 0.521 | 0.789 | 1.000 | 1898 | tags=25%, list=16%, signal=30% |
| 5177 | GOBP\_RESPONSE\_TO\_INTERLEUKIN\_7 |  | 22 | -0.34 | -0.98 | 0.516 | 0.789 | 1.000 | 960 | tags=18%, list=8%, signal=20% |
| 5178 | GOBP\_NEGATIVE\_REGULATION\_OF\_CALCIUM\_ION\_TRANSMEMBRANE\_TRANSPORT |  | 24 | -0.34 | -0.98 | 0.518 | 0.789 | 1.000 | 2291 | tags=21%, list=19%, signal=26% |
| 5179 | GOMF\_UBIQUITINATION\_LIKE\_MODIFICATION\_DEPENDENT\_PROTEIN\_BINDING |  | 10 | -0.42 | -0.98 | 0.514 | 0.789 | 1.000 | 2261 | tags=40%, list=19%, signal=49% |
| 5180 | GOMF\_UDP\_GLUCOSYLTRANSFERASE\_ACTIVITY |  | 8 | -0.44 | -0.98 | 0.518 | 0.789 | 1.000 | 6504 | tags=88%, list=54%, signal=191% |
| 5181 | GOBP\_NEGATIVE\_REGULATION\_OF\_CARDIOCYTE\_DIFFERENTIATION |  | 8 | -0.44 | -0.98 | 0.524 | 0.789 | 1.000 | 1881 | tags=38%, list=16%, signal=44% |
| 5182 | GOMF\_PROTEIN\_N\_TERMINUS\_BINDING |  | 82 | -0.28 | -0.98 | 0.537 | 0.789 | 1.000 | 3465 | tags=34%, list=29%, signal=48% |
| 5183 | GOBP\_POSITIVE\_REGULATION\_OF\_MEMBRANE\_TUBULATION |  | 4 | -0.54 | -0.98 | 0.523 | 0.790 | 1.000 | 1201 | tags=25%, list=10%, signal=28% |
| 5184 | GOBP\_REGULATION\_OF\_MITOTIC\_NUCLEAR\_DIVISION |  | 88 | -0.27 | -0.98 | 0.528 | 0.790 | 1.000 | 4529 | tags=38%, list=38%, signal=60% |
| 5185 | GOBP\_MESENCHYMAL\_STEM\_CELL\_DIFFERENTIATION |  | 6 | -0.48 | -0.98 | 0.522 | 0.790 | 1.000 | 1881 | tags=33%, list=16%, signal=40% |
| 5186 | GOBP\_REGULATION\_OF\_THYMOCYTE\_APOPTOTIC\_PROCESS |  | 8 | -0.44 | -0.98 | 0.524 | 0.791 | 1.000 | 786 | tags=25%, list=7%, signal=27% |
| 5187 | GOBP\_REGULATION\_OF\_PHOSPHOLIPASE\_ACTIVITY |  | 50 | -0.29 | -0.98 | 0.526 | 0.791 | 1.000 | 2695 | tags=28%, list=22%, signal=36% |
| 5188 | GOBP\_REGULATION\_OF\_TOLL\_LIKE\_RECEPTOR\_3\_SIGNALING\_PATHWAY |  | 7 | -0.46 | -0.98 | 0.527 | 0.791 | 1.000 | 3343 | tags=57%, list=28%, signal=79% |
| 5189 | GOCC\_INTRINSIC\_COMPONENT\_OF\_PRESYNAPTIC\_MEMBRANE |  | 39 | -0.31 | -0.98 | 0.516 | 0.791 | 1.000 | 4631 | tags=44%, list=39%, signal=71% |
| 5190 | GOMF\_SPECTRIN\_BINDING |  | 20 | -0.35 | -0.98 | 0.509 | 0.791 | 1.000 | 4110 | tags=50%, list=34%, signal=76% |
| 5191 | GOBP\_CHYLOMICRON\_REMODELING |  | 4 | -0.54 | -0.98 | 0.538 | 0.791 | 1.000 | 3217 | tags=50%, list=27%, signal=68% |
| 5192 | GOCC\_ACTIN\_FILAMENT\_BUNDLE |  | 56 | -0.29 | -0.97 | 0.524 | 0.792 | 1.000 | 3086 | tags=20%, list=26%, signal=26% |
| 5193 | GOBP\_RESPONSE\_TO\_LITHIUM\_ION |  | 15 | -0.38 | -0.97 | 0.516 | 0.792 | 1.000 | 3632 | tags=47%, list=30%, signal=67% |
| 5194 | GOBP\_NEGATIVE\_REGULATION\_OF\_ANDROGEN\_RECEPTOR\_SIGNALING\_PATHWAY |  | 12 | -0.40 | -0.97 | 0.526 | 0.792 | 1.000 | 2656 | tags=33%, list=22%, signal=43% |
| 5195 | GOBP\_FATTY\_ACID\_HOMEOSTASIS |  | 9 | -0.43 | -0.97 | 0.522 | 0.792 | 1.000 | 4801 | tags=78%, list=40%, signal=129% |
| 5196 | GOBP\_LONG\_CHAIN\_FATTY\_ACID\_TRANSPORT |  | 46 | -0.30 | -0.97 | 0.526 | 0.792 | 1.000 | 3844 | tags=48%, list=32%, signal=70% |
| 5197 | GOBP\_MODULATION\_OF\_PROCESS\_OF\_OTHER\_ORGANISM\_INVOLVED\_IN\_SYMBIOTIC\_INTERACTION |  | 72 | -0.28 | -0.97 | 0.537 | 0.792 | 1.000 | 4866 | tags=46%, list=41%, signal=77% |
| 5198 | GOMF\_AMP\_ACTIVATED\_PROTEIN\_KINASE\_ACTIVITY |  | 4 | -0.54 | -0.97 | 0.526 | 0.793 | 1.000 | 4528 | tags=75%, list=38%, signal=120% |
| 5199 | GOMF\_OXYGEN\_CARRIER\_ACTIVITY |  | 4 | -0.54 | -0.97 | 0.526 | 0.792 | 1.000 | 662 | tags=25%, list=6%, signal=26% |
| 5200 | GOBP\_NEGATIVE\_REGULATION\_OF\_RECEPTOR\_SIGNALING\_PATHWAY\_VIA\_STAT |  | 21 | -0.35 | -0.97 | 0.511 | 0.793 | 1.000 | 1541 | tags=24%, list=13%, signal=27% |
| 5201 | GOBP\_NEGATIVE\_REGULATION\_OF\_CENTROSOME\_DUPLICATION |  | 10 | -0.41 | -0.97 | 0.517 | 0.793 | 1.000 | 3718 | tags=50%, list=31%, signal=72% |
| 5202 | GOBP\_CONVERGENT\_EXTENSION\_INVOLVED\_IN\_AXIS\_ELONGATION |  | 4 | -0.54 | -0.97 | 0.531 | 0.794 | 1.000 | 1579 | tags=25%, list=13%, signal=29% |
| 5203 | GOBP\_SMOOTH\_MUSCLE\_TISSUE\_DEVELOPMENT |  | 16 | -0.37 | -0.97 | 0.522 | 0.794 | 1.000 | 2604 | tags=31%, list=22%, signal=40% |
| 5204 | GOBP\_NEGATIVE\_REGULATION\_OF\_AUTOPHAGOSOME\_MATURATION |  | 5 | -0.50 | -0.97 | 0.528 | 0.794 | 1.000 | 1111 | tags=20%, list=9%, signal=22% |
| 5205 | GOMF\_INTERLEUKIN\_1\_RECEPTOR\_BINDING |  | 7 | -0.46 | -0.97 | 0.523 | 0.794 | 1.000 | 4022 | tags=71%, list=33%, signal=107% |
| 5206 | GOBP\_REGULATION\_OF\_TAU\_PROTEIN\_KINASE\_ACTIVITY |  | 7 | -0.46 | -0.97 | 0.532 | 0.794 | 1.000 | 4598 | tags=71%, list=38%, signal=116% |
| 5207 | GOBP\_RESPONSE\_TO\_NERVE\_GROWTH\_FACTOR |  | 39 | -0.31 | -0.97 | 0.524 | 0.793 | 1.000 | 4892 | tags=54%, list=41%, signal=91% |
| 5208 | GOBP\_REGULATION\_OF\_ADHERENS\_JUNCTION\_ORGANIZATION |  | 5 | -0.50 | -0.97 | 0.527 | 0.794 | 1.000 | 3269 | tags=60%, list=27%, signal=82% |
| 5209 | GOBP\_REGULATION\_OF\_PROSTAGLANDIN\_SECRETION |  | 8 | -0.44 | -0.97 | 0.520 | 0.794 | 1.000 | 2769 | tags=38%, list=23%, signal=49% |
| 5210 | GOBP\_POSITIVE\_REGULATION\_OF\_CENTRIOLE\_REPLICATION |  | 6 | -0.48 | -0.97 | 0.522 | 0.794 | 1.000 | 6285 | tags=100%, list=52%, signal=210% |
| 5211 | GOBP\_ER\_NUCLEUS\_SIGNALING\_PATHWAY |  | 43 | -0.30 | -0.97 | 0.523 | 0.794 | 1.000 | 4035 | tags=44%, list=34%, signal=66% |
| 5212 | GOBP\_POSITIVE\_REGULATION\_OF\_PRODUCTION\_OF\_MIRNAS\_INVOLVED\_IN\_GENE\_SILENCING\_BY\_MIRNA |  | 9 | -0.43 | -0.97 | 0.524 | 0.794 | 1.000 | 2384 | tags=22%, list=20%, signal=28% |
| 5213 | GOMF\_UBIQUITIN\_DEPENDENT\_PROTEIN\_BINDING |  | 10 | -0.42 | -0.97 | 0.529 | 0.794 | 1.000 | 2261 | tags=40%, list=19%, signal=49% |
| 5214 | GOMF\_ATP\_ACTIVATED\_INWARD\_RECTIFIER\_POTASSIUM\_CHANNEL\_ACTIVITY |  | 3 | -0.58 | -0.97 | 0.534 | 0.794 | 1.000 | 561 | tags=33%, list=5%, signal=35% |
| 5215 | GOMF\_RIBONUCLEOPROTEIN\_COMPLEX\_BINDING |  | 104 | -0.27 | -0.97 | 0.552 | 0.794 | 1.000 | 5576 | tags=47%, list=46%, signal=87% |
| 5216 | GOBP\_HINDBRAIN\_DEVELOPMENT |  | 92 | -0.27 | -0.97 | 0.542 | 0.794 | 1.000 | 4064 | tags=35%, list=34%, signal=52% |
| 5217 | GOBP\_POSITIVE\_REGULATION\_OF\_DENDRITE\_DEVELOPMENT |  | 14 | -0.38 | -0.97 | 0.517 | 0.794 | 1.000 | 4064 | tags=57%, list=34%, signal=86% |
| 5218 | GOMF\_DTTP\_DIPHOSPHATASE\_ACTIVITY |  | 4 | -0.54 | -0.97 | 0.528 | 0.794 | 1.000 | 3094 | tags=50%, list=26%, signal=67% |
| 5219 | GOBP\_SYMPATHETIC\_GANGLION\_DEVELOPMENT |  | 8 | -0.44 | -0.97 | 0.528 | 0.794 | 1.000 | 2811 | tags=50%, list=23%, signal=65% |
| 5220 | GOBP\_POSITIVE\_REGULATION\_OF\_HISTONE\_DEACETYLATION |  | 10 | -0.42 | -0.97 | 0.523 | 0.794 | 1.000 | 3640 | tags=50%, list=30%, signal=72% |
| 5221 | GOBP\_POSITIVE\_REGULATION\_OF\_PROTEIN\_LOCALIZATION\_TO\_EARLY\_ENDOSOME |  | 6 | -0.48 | -0.97 | 0.520 | 0.794 | 1.000 | 2416 | tags=50%, list=20%, signal=63% |
| 5222 | GOBP\_PEPTIDYL\_PROLINE\_HYDROXYLATION\_TO\_4\_HYDROXY\_L\_PROLINE |  | 9 | -0.43 | -0.97 | 0.530 | 0.794 | 1.000 | 3164 | tags=33%, list=26%, signal=45% |
| 5223 | GOCC\_CLEAVAGE\_FURROW |  | 41 | -0.30 | -0.97 | 0.531 | 0.794 | 1.000 | 2995 | tags=27%, list=25%, signal=36% |
| 5224 | GOBP\_T\_HELPER\_2\_CELL\_CYTOKINE\_PRODUCTION |  | 8 | -0.44 | -0.97 | 0.523 | 0.794 | 1.000 | 1267 | tags=38%, list=11%, signal=42% |
| 5225 | GOBP\_MYOBLAST\_FUSION |  | 20 | -0.35 | -0.97 | 0.522 | 0.794 | 1.000 | 2494 | tags=30%, list=21%, signal=38% |
| 5226 | GOCC\_CARBOXY\_TERMINAL\_DOMAIN\_PROTEIN\_KINASE\_COMPLEX |  | 17 | -0.36 | -0.97 | 0.522 | 0.794 | 1.000 | 5069 | tags=53%, list=42%, signal=91% |
| 5227 | GOBP\_REGULATION\_OF\_ACTIN\_CYTOSKELETON\_REORGANIZATION |  | 32 | -0.32 | -0.97 | 0.534 | 0.794 | 1.000 | 3695 | tags=38%, list=31%, signal=54% |
| 5228 | GOBP\_MEMBRANE\_ORGANIZATION |  | 626 | -0.25 | -0.97 | 0.621 | 0.794 | 1.000 | 4485 | tags=38%, list=37%, signal=57% |
| 5229 | GOBP\_NEURON\_PROJECTION\_ARBORIZATION |  | 21 | -0.34 | -0.97 | 0.520 | 0.795 | 1.000 | 3483 | tags=43%, list=29%, signal=60% |
| 5230 | GOBP\_DNA\_BIOSYNTHETIC\_PROCESS |  | 145 | -0.26 | -0.97 | 0.568 | 0.795 | 1.000 | 5061 | tags=47%, list=42%, signal=80% |
| 5231 | GOMF\_LAMIN\_BINDING |  | 11 | -0.41 | -0.97 | 0.527 | 0.795 | 1.000 | 3352 | tags=45%, list=28%, signal=63% |
| 5232 | GOMF\_ACTIVIN\_ACTIVATED\_RECEPTOR\_ACTIVITY |  | 8 | -0.44 | -0.97 | 0.525 | 0.795 | 1.000 | 4506 | tags=50%, list=38%, signal=80% |
| 5233 | GOMF\_LIGASE\_ACTIVITY\_FORMING\_CARBON\_CARBON\_BONDS |  | 6 | -0.48 | -0.97 | 0.525 | 0.795 | 1.000 | 1822 | tags=33%, list=15%, signal=39% |
| 5234 | GOBP\_REGULATION\_OF\_PROTEIN\_EXPORT\_FROM\_NUCLEUS |  | 25 | -0.33 | -0.97 | 0.530 | 0.795 | 1.000 | 5157 | tags=52%, list=43%, signal=91% |
| 5235 | GOBP\_PROTEIN\_LOCALIZATION\_TO\_CELL\_CORTEX |  | 10 | -0.41 | -0.97 | 0.524 | 0.795 | 1.000 | 1814 | tags=20%, list=15%, signal=24% |
| 5236 | GOMF\_L\_ORNITHINE\_TRANSMEMBRANE\_TRANSPORTER\_ACTIVITY |  | 4 | -0.54 | -0.97 | 0.533 | 0.795 | 1.000 | 1025 | tags=25%, list=9%, signal=27% |
| 5237 | GOBP\_SEROTONIN\_UPTAKE |  | 9 | -0.43 | -0.97 | 0.533 | 0.795 | 1.000 | 2392 | tags=33%, list=20%, signal=42% |
| 5238 | GOBP\_MORPHOGENESIS\_OF\_AN\_ENDOTHELIUM |  | 14 | -0.38 | -0.97 | 0.516 | 0.796 | 1.000 | 4337 | tags=57%, list=36%, signal=89% |
| 5239 | GOCC\_CORVET\_COMPLEX |  | 5 | -0.51 | -0.97 | 0.532 | 0.795 | 1.000 | 2675 | tags=40%, list=22%, signal=51% |
| 5240 | GOCC\_CELL\_CORTEX\_REGION |  | 33 | -0.32 | -0.97 | 0.525 | 0.796 | 1.000 | 4047 | tags=45%, list=34%, signal=68% |
| 5241 | GOBP\_LONG\_CHAIN\_FATTY\_ACYL\_COA\_BIOSYNTHETIC\_PROCESS |  | 16 | -0.37 | -0.97 | 0.528 | 0.796 | 1.000 | 4131 | tags=50%, list=34%, signal=76% |
| 5242 | GOBP\_GERMINAL\_CENTER\_B\_CELL\_DIFFERENTIATION |  | 4 | -0.53 | -0.97 | 0.536 | 0.796 | 1.000 | 3977 | tags=75%, list=33%, signal=112% |
| 5243 | GOBP\_REGULATION\_OF\_HISTONE\_H3\_K36\_METHYLATION |  | 5 | -0.50 | -0.97 | 0.537 | 0.796 | 1.000 | 6008 | tags=100%, list=50%, signal=200% |
| 5244 | GOCC\_INTRINSIC\_COMPONENT\_OF\_VACUOLAR\_MEMBRANE |  | 8 | -0.44 | -0.97 | 0.533 | 0.796 | 1.000 | 6144 | tags=75%, list=51%, signal=153% |
| 5245 | GOCC\_DNA\_REPLICATION\_PREINITIATION\_COMPLEX |  | 11 | -0.41 | -0.97 | 0.537 | 0.796 | 1.000 | 5533 | tags=73%, list=46%, signal=135% |
| 5246 | GOBP\_POSITIVE\_REGULATION\_OF\_ATPASE\_ACTIVITY |  | 45 | -0.30 | -0.97 | 0.525 | 0.797 | 1.000 | 3590 | tags=38%, list=30%, signal=54% |
| 5247 | GOBP\_NEGATIVE\_REGULATION\_OF\_AMYLOID\_PRECURSOR\_PROTEIN\_CATABOLIC\_PROCESS |  | 18 | -0.36 | -0.97 | 0.522 | 0.797 | 1.000 | 1597 | tags=22%, list=13%, signal=26% |
| 5248 | GOCC\_MICROVILLUS |  | 63 | -0.29 | -0.97 | 0.544 | 0.797 | 1.000 | 3619 | tags=41%, list=30%, signal=59% |
| 5249 | GOCC\_PHOTORECEPTOR\_INNER\_SEGMENT |  | 22 | -0.34 | -0.97 | 0.519 | 0.797 | 1.000 | 2209 | tags=36%, list=18%, signal=44% |
| 5250 | GOBP\_ESTABLISHMENT\_OF\_PLANAR\_POLARITY\_OF\_EMBRYONIC\_EPITHELIUM |  | 11 | -0.40 | -0.97 | 0.520 | 0.797 | 1.000 | 2992 | tags=27%, list=25%, signal=36% |
| 5251 | GOBP\_T\_CELL\_MEDIATED\_IMMUNE\_RESPONSE\_TO\_TUMOR\_CELL |  | 3 | -0.58 | -0.97 | 0.543 | 0.797 | 1.000 | 4649 | tags=67%, list=39%, signal=109% |
| 5252 | GOBP\_NEUROTRANSMITTER\_GATED\_ION\_CHANNEL\_CLUSTERING |  | 5 | -0.50 | -0.97 | 0.525 | 0.797 | 1.000 | 4715 | tags=60%, list=39%, signal=99% |
| 5253 | GOBP\_POSITIVE\_REGULATION\_OF\_TISSUE\_REMODELING |  | 6 | -0.48 | -0.97 | 0.540 | 0.797 | 1.000 | 2416 | tags=33%, list=20%, signal=42% |
| 5254 | GOMF\_MHC\_CLASS\_II\_PROTEIN\_BINDING |  | 5 | -0.50 | -0.97 | 0.529 | 0.797 | 1.000 | 1138 | tags=20%, list=9%, signal=22% |
| 5255 | GOCC\_GOLGI\_LUMEN |  | 49 | -0.30 | -0.97 | 0.534 | 0.797 | 1.000 | 2019 | tags=16%, list=17%, signal=20% |
| 5256 | GOBP\_STEROL\_METABOLIC\_PROCESS |  | 112 | -0.27 | -0.97 | 0.562 | 0.797 | 1.000 | 3247 | tags=28%, list=27%, signal=38% |
| 5257 | GOBP\_AMINE\_METABOLIC\_PROCESS |  | 104 | -0.27 | -0.97 | 0.551 | 0.797 | 1.000 | 2569 | tags=23%, list=21%, signal=29% |
| 5258 | GOBP\_NEGATIVE\_REGULATION\_OF\_ALPHA\_BETA\_T\_CELL\_DIFFERENTIATION |  | 20 | -0.35 | -0.97 | 0.529 | 0.797 | 1.000 | 1951 | tags=30%, list=16%, signal=36% |
| 5259 | GOBP\_RESPONSE\_TO\_COLD |  | 29 | -0.32 | -0.97 | 0.528 | 0.797 | 1.000 | 2340 | tags=34%, list=19%, signal=43% |
| 5260 | GOBP\_POSITIVE\_REGULATION\_OF\_FATTY\_ACID\_BETA\_OXIDATION |  | 7 | -0.46 | -0.97 | 0.531 | 0.797 | 1.000 | 5145 | tags=71%, list=43%, signal=125% |
| 5261 | GOBP\_REGULATION\_OF\_CATABOLIC\_PROCESS |  | 764 | -0.24 | -0.97 | 0.642 | 0.797 | 1.000 | 4324 | tags=36%, list=36%, signal=53% |
| 5262 | GOBP\_PROTEIN\_AUTO\_ADP\_RIBOSYLATION |  | 9 | -0.43 | -0.97 | 0.529 | 0.797 | 1.000 | 1704 | tags=33%, list=14%, signal=39% |
| 5263 | GOBP\_REGULATION\_OF\_T\_CELL\_MEDIATED\_IMMUNE\_RESPONSE\_TO\_TUMOR\_CELL |  | 3 | -0.58 | -0.97 | 0.542 | 0.797 | 1.000 | 4649 | tags=67%, list=39%, signal=109% |
| 5264 | GOBP\_SINGLE\_STRANDED\_VIRAL\_RNA\_REPLICATION\_VIA\_DOUBLE\_STRANDED\_DNA\_INTERMEDIATE |  | 8 | -0.44 | -0.97 | 0.527 | 0.797 | 1.000 | 3934 | tags=50%, list=33%, signal=74% |
| 5265 | GOBP\_PERIPHERAL\_NERVOUS\_SYSTEM\_MYELIN\_MAINTENANCE |  | 5 | -0.50 | -0.97 | 0.533 | 0.797 | 1.000 | 4312 | tags=60%, list=36%, signal=94% |
| 5266 | GOBP\_REGULATION\_OF\_ATPASE\_ACTIVITY |  | 55 | -0.29 | -0.97 | 0.538 | 0.797 | 1.000 | 3590 | tags=36%, list=30%, signal=52% |
| 5267 | GOBP\_AXON\_ENSHEATHMENT\_IN\_CENTRAL\_NERVOUS\_SYSTEM |  | 11 | -0.40 | -0.97 | 0.532 | 0.797 | 1.000 | 6560 | tags=91%, list=55%, signal=200% |
| 5268 | GOBP\_ODONTOGENESIS |  | 83 | -0.27 | -0.97 | 0.551 | 0.797 | 1.000 | 2515 | tags=29%, list=21%, signal=36% |
| 5269 | GOMF\_MICROTUBULE\_SEVERING\_ATPASE\_ACTIVITY |  | 6 | -0.48 | -0.97 | 0.534 | 0.797 | 1.000 | 5455 | tags=83%, list=45%, signal=153% |
| 5270 | GOMF\_SERINE\_TYPE\_CARBOXYPEPTIDASE\_ACTIVITY |  | 4 | -0.53 | -0.97 | 0.545 | 0.797 | 1.000 | 4009 | tags=75%, list=33%, signal=113% |
| 5271 | GOBP\_FOLATE\_TRANSMEMBRANE\_TRANSPORT |  | 4 | -0.53 | -0.97 | 0.542 | 0.797 | 1.000 | 2179 | tags=50%, list=18%, signal=61% |
| 5272 | GOMF\_OLIGOSACCHARYL\_TRANSFERASE\_ACTIVITY |  | 6 | -0.48 | -0.97 | 0.529 | 0.798 | 1.000 | 6295 | tags=100%, list=52%, signal=210% |
| 5273 | GOBP\_CELLULAR\_RESPONSE\_TO\_STEROID\_HORMONE\_STIMULUS |  | 143 | -0.26 | -0.97 | 0.572 | 0.798 | 1.000 | 4512 | tags=45%, list=38%, signal=71% |
| 5274 | GOBP\_IRON\_ION\_HOMEOSTASIS |  | 63 | -0.28 | -0.97 | 0.550 | 0.798 | 1.000 | 2847 | tags=32%, list=24%, signal=41% |
| 5275 | GOBP\_POSITIVE\_REGULATION\_OF\_TRANSLATION |  | 90 | -0.27 | -0.97 | 0.557 | 0.798 | 1.000 | 4629 | tags=38%, list=39%, signal=61% |
| 5276 | GOBP\_WATER\_HOMEOSTASIS |  | 45 | -0.30 | -0.97 | 0.533 | 0.798 | 1.000 | 4102 | tags=51%, list=34%, signal=77% |
| 5277 | GOBP\_NUCLEOBASE\_METABOLIC\_PROCESS |  | 26 | -0.33 | -0.97 | 0.534 | 0.798 | 1.000 | 2929 | tags=31%, list=24%, signal=41% |
| 5278 | GOCC\_SNARE\_COMPLEX |  | 39 | -0.31 | -0.97 | 0.532 | 0.798 | 1.000 | 3698 | tags=36%, list=31%, signal=52% |
| 5279 | GOBP\_RETINOIC\_ACID\_METABOLIC\_PROCESS |  | 15 | -0.37 | -0.97 | 0.533 | 0.797 | 1.000 | 51 | tags=13%, list=0%, signal=13% |
| 5280 | GOBP\_POSITIVE\_REGULATION\_OF\_EPIDERMIS\_DEVELOPMENT |  | 15 | -0.37 | -0.97 | 0.534 | 0.797 | 1.000 | 1635 | tags=27%, list=14%, signal=31% |
| 5281 | GOBP\_REACTIVE\_OXYGEN\_SPECIES\_METABOLIC\_PROCESS |  | 178 | -0.26 | -0.97 | 0.583 | 0.797 | 1.000 | 3864 | tags=34%, list=32%, signal=50% |
| 5282 | GOBP\_SEROTONIN\_TRANSPORT |  | 14 | -0.38 | -0.97 | 0.533 | 0.797 | 1.000 | 2392 | tags=36%, list=20%, signal=45% |
| 5283 | GOBP\_PROSTAGLANDIN\_TRANSPORT |  | 13 | -0.39 | -0.97 | 0.528 | 0.798 | 1.000 | 2769 | tags=38%, list=23%, signal=50% |
| 5284 | GOBP\_NUCLEOTIDE\_EXCISION\_REPAIR\_DNA\_INCISION |  | 32 | -0.32 | -0.97 | 0.529 | 0.798 | 1.000 | 5351 | tags=53%, list=45%, signal=96% |
| 5285 | GOCC\_INTERMEDIATE\_FILAMENT\_CYTOSKELETON |  | 81 | -0.27 | -0.97 | 0.547 | 0.798 | 1.000 | 3765 | tags=33%, list=31%, signal=48% |
| 5286 | GOBP\_POSITIVE\_REGULATION\_OF\_CHROMATIN\_BINDING |  | 10 | -0.41 | -0.97 | 0.532 | 0.797 | 1.000 | 1725 | tags=30%, list=14%, signal=35% |
| 5287 | GOBP\_NEGATIVE\_REGULATION\_OF\_GASTRIC\_ACID\_SECRETION |  | 4 | -0.54 | -0.97 | 0.546 | 0.798 | 1.000 | 1939 | tags=50%, list=16%, signal=60% |
| 5288 | GOBP\_MODULATION\_OF\_PROCESS\_OF\_OTHER\_ORGANISM |  | 77 | -0.28 | -0.97 | 0.550 | 0.798 | 1.000 | 4866 | tags=47%, list=41%, signal=78% |
| 5289 | GOBP\_NOTCH\_RECEPTOR\_PROCESSING |  | 9 | -0.42 | -0.97 | 0.525 | 0.798 | 1.000 | 6677 | tags=89%, list=56%, signal=200% |
| 5290 | GOBP\_POSITIVE\_REGULATION\_OF\_DNA\_METHYLATION\_DEPENDENT\_HETEROCHROMATIN\_ASSEMBLY |  | 5 | -0.50 | -0.97 | 0.540 | 0.798 | 1.000 | 3624 | tags=40%, list=30%, signal=57% |
| 5291 | GOBP\_REGULATION\_OF\_PROTEIN\_LOCALIZATION\_TO\_NUCLEOLUS |  | 8 | -0.44 | -0.97 | 0.524 | 0.798 | 1.000 | 586 | tags=13%, list=5%, signal=13% |
| 5292 | GOBP\_REGULATION\_OF\_POSTSYNAPTIC\_SPECIALIZATION\_ASSEMBLY |  | 6 | -0.48 | -0.97 | 0.540 | 0.798 | 1.000 | 29 | tags=17%, list=0%, signal=17% |
| 5293 | GOBP\_NEGATIVE\_REGULATION\_OF\_MEMBRANE\_PROTEIN\_ECTODOMAIN\_PROTEOLYSIS |  | 6 | -0.48 | -0.97 | 0.535 | 0.799 | 1.000 | 3168 | tags=50%, list=26%, signal=68% |
| 5294 | GOBP\_POSITIVE\_REGULATION\_OF\_PROTEIN\_LOCALIZATION\_TO\_CILIUM |  | 3 | -0.58 | -0.97 | 0.553 | 0.799 | 1.000 | 880 | tags=33%, list=7%, signal=36% |
| 5295 | GOBP\_ALPHA\_LINOLENIC\_ACID\_METABOLIC\_PROCESS |  | 10 | -0.41 | -0.97 | 0.532 | 0.799 | 1.000 | 4385 | tags=60%, list=37%, signal=94% |
| 5296 | GOBP\_LIPID\_HYDROXYLATION |  | 3 | -0.58 | -0.97 | 0.545 | 0.799 | 1.000 | 2278 | tags=67%, list=19%, signal=82% |
| 5297 | GOBP\_DUCTUS\_ARTERIOSUS\_CLOSURE |  | 3 | -0.58 | -0.97 | 0.546 | 0.800 | 1.000 | 4069 | tags=33%, list=34%, signal=50% |
| 5298 | GOBP\_SKELETAL\_MUSCLE\_ACETYLCHOLINE\_GATED\_CHANNEL\_CLUSTERING |  | 9 | -0.43 | -0.96 | 0.527 | 0.800 | 1.000 | 1318 | tags=22%, list=11%, signal=25% |
| 5299 | GOBP\_PLATELET\_DERIVED\_GROWTH\_FACTOR\_RECEPTOR\_BETA\_SIGNALING\_PATHWAY |  | 14 | -0.37 | -0.96 | 0.528 | 0.800 | 1.000 | 3692 | tags=43%, list=31%, signal=62% |
| 5300 | GOBP\_LIPID\_DROPLET\_ORGANIZATION |  | 17 | -0.36 | -0.96 | 0.532 | 0.800 | 1.000 | 280 | tags=12%, list=2%, signal=12% |
| 5301 | GOBP\_POSITIVE\_REGULATION\_OF\_CELLULAR\_CARBOHYDRATE\_METABOLIC\_PROCESS |  | 34 | -0.31 | -0.96 | 0.536 | 0.800 | 1.000 | 3692 | tags=38%, list=31%, signal=55% |
| 5302 | GOBP\_POSITIVE\_REGULATION\_OF\_SYNAPSE\_MATURATION |  | 7 | -0.46 | -0.96 | 0.538 | 0.800 | 1.000 | 3431 | tags=29%, list=29%, signal=40% |
| 5303 | GOBP\_NEGATIVE\_REGULATION\_OF\_MAST\_CELL\_DEGRANULATION |  | 3 | -0.58 | -0.96 | 0.554 | 0.800 | 1.000 | 726 | tags=33%, list=6%, signal=35% |
| 5304 | GOMF\_VINCULIN\_BINDING |  | 9 | -0.42 | -0.96 | 0.531 | 0.801 | 1.000 | 6315 | tags=78%, list=53%, signal=164% |
| 5305 | GOMF\_MISFOLDED\_PROTEIN\_BINDING |  | 19 | -0.35 | -0.96 | 0.533 | 0.801 | 1.000 | 5182 | tags=58%, list=43%, signal=102% |
| 5306 | GOMF\_EPIDERMAL\_GROWTH\_FACTOR\_RECEPTOR\_BINDING |  | 26 | -0.33 | -0.96 | 0.530 | 0.801 | 1.000 | 998 | tags=19%, list=8%, signal=21% |
| 5307 | GOBP\_REGULATION\_OF\_MACROPHAGE\_PROLIFERATION |  | 4 | -0.53 | -0.96 | 0.551 | 0.801 | 1.000 | 3093 | tags=75%, list=26%, signal=101% |
| 5308 | GOBP\_INNER\_CELL\_MASS\_CELL\_DIFFERENTIATION |  | 5 | -0.50 | -0.96 | 0.540 | 0.801 | 1.000 | 3061 | tags=60%, list=25%, signal=80% |
| 5309 | GOBP\_PROSTAGLANDIN\_SECRETION |  | 10 | -0.41 | -0.96 | 0.532 | 0.801 | 1.000 | 1408 | tags=30%, list=12%, signal=34% |
| 5310 | GOBP\_POSITIVE\_REGULATION\_OF\_MACROPHAGE\_DIFFERENTIATION |  | 11 | -0.40 | -0.96 | 0.538 | 0.801 | 1.000 | 3836 | tags=55%, list=32%, signal=80% |
| 5311 | GOMF\_RDNA\_BINDING |  | 5 | -0.50 | -0.96 | 0.537 | 0.801 | 1.000 | 5346 | tags=80%, list=45%, signal=144% |
| 5312 | GOBP\_REGULATION\_OF\_HEART\_RATE\_BY\_CARDIAC\_CONDUCTION |  | 27 | -0.33 | -0.96 | 0.531 | 0.801 | 1.000 | 3918 | tags=37%, list=33%, signal=55% |
| 5313 | GOBP\_RESPONSE\_TO\_CORTICOTROPIN\_RELEASING\_HORMONE |  | 4 | -0.53 | -0.96 | 0.544 | 0.801 | 1.000 | 2405 | tags=75%, list=20%, signal=94% |
| 5314 | GOCC\_ACTIVIN\_RECEPTOR\_COMPLEX |  | 5 | -0.50 | -0.96 | 0.549 | 0.802 | 1.000 | 4506 | tags=60%, list=38%, signal=96% |
| 5315 | GOMF\_FMN\_BINDING |  | 12 | -0.39 | -0.96 | 0.533 | 0.802 | 1.000 | 1292 | tags=17%, list=11%, signal=19% |
| 5316 | GOBP\_C\_TERMINAL\_PROTEIN\_LIPIDATION |  | 4 | -0.53 | -0.96 | 0.558 | 0.802 | 1.000 | 4561 | tags=75%, list=38%, signal=121% |
| 5317 | GOBP\_EMBRYONIC\_CRANIAL\_SKELETON\_MORPHOGENESIS |  | 22 | -0.34 | -0.96 | 0.532 | 0.802 | 1.000 | 5077 | tags=59%, list=42%, signal=102% |
| 5318 | GOMF\_RNA\_POLYMERASE\_II\_CTD\_HEPTAPEPTIDE\_REPEAT\_KINASE\_ACTIVITY |  | 10 | -0.41 | -0.96 | 0.538 | 0.803 | 1.000 | 4906 | tags=50%, list=41%, signal=84% |
| 5319 | GOBP\_CHONDROCYTE\_PROLIFERATION |  | 13 | -0.39 | -0.96 | 0.530 | 0.803 | 1.000 | 2555 | tags=31%, list=21%, signal=39% |
| 5320 | GOBP\_NEGATIVE\_REGULATION\_OF\_STRIATED\_MUSCLE\_CONTRACTION |  | 5 | -0.50 | -0.96 | 0.541 | 0.803 | 1.000 | 3968 | tags=60%, list=33%, signal=90% |
| 5321 | GOBP\_CARDIAC\_VENTRICLE\_FORMATION |  | 6 | -0.48 | -0.96 | 0.535 | 0.803 | 1.000 | 2474 | tags=33%, list=21%, signal=42% |
| 5322 | GOBP\_CELLULAR\_HOMEOSTASIS |  | 606 | -0.24 | -0.96 | 0.657 | 0.803 | 1.000 | 3269 | tags=30%, list=27%, signal=39% |
| 5323 | GOBP\_WNT\_SIGNALING\_PATHWAY\_INVOLVED\_IN\_MIDBRAIN\_DOPAMINERGIC\_NEURON\_DIFFERENTIATION |  | 8 | -0.44 | -0.96 | 0.536 | 0.803 | 1.000 | 2811 | tags=25%, list=23%, signal=33% |
| 5324 | GOBP\_REGULATION\_OF\_BINDING |  | 247 | -0.25 | -0.96 | 0.605 | 0.803 | 1.000 | 2560 | tags=21%, list=21%, signal=26% |
| 5325 | GOBP\_REGULATION\_OF\_DNA\_TEMPLATED\_TRANSCRIPTION\_ELONGATION |  | 42 | -0.30 | -0.96 | 0.542 | 0.803 | 1.000 | 4336 | tags=43%, list=36%, signal=67% |
| 5326 | GOBP\_BARBED\_END\_ACTIN\_FILAMENT\_CAPPING |  | 19 | -0.35 | -0.96 | 0.546 | 0.803 | 1.000 | 2598 | tags=26%, list=22%, signal=34% |
| 5327 | GOCC\_FIBRILLAR\_CENTER |  | 106 | -0.27 | -0.96 | 0.567 | 0.803 | 1.000 | 3722 | tags=34%, list=31%, signal=49% |
| 5328 | GOBP\_POSITIVE\_REGULATION\_OF\_CYSTEINE\_TYPE\_ENDOPEPTIDASE\_ACTIVITY\_INVOLVED\_IN\_APOPTOTIC\_SIGNALING\_PATHWAY |  | 9 | -0.43 | -0.96 | 0.539 | 0.802 | 1.000 | 2960 | tags=44%, list=25%, signal=59% |
| 5329 | GOBP\_SEQUESTERING\_OF\_TRIGLYCERIDE |  | 10 | -0.41 | -0.96 | 0.535 | 0.802 | 1.000 | 2009 | tags=40%, list=17%, signal=48% |
| 5330 | GOMF\_TELOMERASE\_RNA\_BINDING |  | 16 | -0.37 | -0.96 | 0.532 | 0.802 | 1.000 | 5754 | tags=56%, list=48%, signal=108% |
| 5331 | GOBP\_MORPHOGENESIS\_OF\_AN\_EPITHELIAL\_FOLD |  | 16 | -0.37 | -0.96 | 0.534 | 0.802 | 1.000 | 5693 | tags=63%, list=47%, signal=119% |
| 5332 | GOBP\_INNATE\_IMMUNE\_RESPONSE\_ACTIVATING\_SIGNAL\_TRANSDUCTION |  | 71 | -0.28 | -0.96 | 0.562 | 0.802 | 1.000 | 3136 | tags=25%, list=26%, signal=34% |
| 5333 | GOBP\_POSITIVE\_REGULATION\_OF\_RNA\_POLYMERASE\_II\_TRANSCRIPTION\_PREINITIATION\_COMPLEX\_ASSEMBLY |  | 7 | -0.45 | -0.96 | 0.545 | 0.802 | 1.000 | 711 | tags=14%, list=6%, signal=15% |
| 5334 | GOBP\_MACROPHAGE\_CHEMOTAXIS |  | 25 | -0.33 | -0.96 | 0.532 | 0.802 | 1.000 | 3921 | tags=48%, list=33%, signal=71% |
| 5335 | GOBP\_CELLULAR\_MODIFIED\_AMINO\_ACID\_CATABOLIC\_PROCESS |  | 19 | -0.35 | -0.96 | 0.545 | 0.802 | 1.000 | 2391 | tags=37%, list=20%, signal=46% |
| 5336 | GOBP\_DTMP\_METABOLIC\_PROCESS |  | 3 | -0.58 | -0.96 | 0.563 | 0.803 | 1.000 | 1728 | tags=33%, list=14%, signal=39% |
| 5337 | GOBP\_POSITIVE\_REGULATION\_OF\_GLYCOGEN\_METABOLIC\_PROCESS |  | 10 | -0.41 | -0.96 | 0.532 | 0.803 | 1.000 | 551 | tags=20%, list=5%, signal=21% |
| 5338 | GOBP\_REGULATION\_OF\_VASCULAR\_WOUND\_HEALING |  | 6 | -0.47 | -0.96 | 0.547 | 0.803 | 1.000 | 4112 | tags=67%, list=34%, signal=101% |
| 5339 | GOMF\_ARYL\_HYDROCARBON\_RECEPTOR\_BINDING |  | 9 | -0.42 | -0.96 | 0.536 | 0.804 | 1.000 | 4432 | tags=44%, list=37%, signal=70% |
| 5340 | GOBP\_RIBONUCLEOSIDE\_MONOPHOSPHATE\_BIOSYNTHETIC\_PROCESS |  | 28 | -0.32 | -0.96 | 0.540 | 0.804 | 1.000 | 4296 | tags=36%, list=36%, signal=55% |
| 5341 | GOBP\_POSITIVE\_REGULATION\_OF\_TRANSMEMBRANE\_RECEPTOR\_PROTEIN\_SERINE\_THREONINE\_KINASE\_SIGNALING\_PATHWAY |  | 73 | -0.28 | -0.96 | 0.561 | 0.804 | 1.000 | 3756 | tags=36%, list=31%, signal=52% |
| 5342 | GOBP\_MACROPHAGE\_COLONY\_STIMULATING\_FACTOR\_SIGNALING\_PATHWAY |  | 6 | -0.47 | -0.96 | 0.548 | 0.804 | 1.000 | 3695 | tags=50%, list=31%, signal=72% |
| 5343 | GOCC\_NPBAF\_COMPLEX |  | 12 | -0.39 | -0.96 | 0.533 | 0.803 | 1.000 | 3279 | tags=33%, list=27%, signal=46% |
| 5344 | GOBP\_CELLULAR\_COMPONENT\_DISASSEMBLY |  | 422 | -0.25 | -0.96 | 0.641 | 0.804 | 1.000 | 3348 | tags=27%, list=28%, signal=36% |
| 5345 | GOBP\_SODIUM\_INDEPENDENT\_ORGANIC\_ANION\_TRANSPORT |  | 7 | -0.45 | -0.96 | 0.548 | 0.804 | 1.000 | 2185 | tags=29%, list=18%, signal=35% |
| 5346 | GOMF\_ADENYLATE\_CYCLASE\_ACTIVATOR\_ACTIVITY |  | 4 | -0.53 | -0.96 | 0.558 | 0.805 | 1.000 | 4627 | tags=75%, list=39%, signal=122% |
| 5347 | GOMF\_DNA\_TRANSLOCASE\_ACTIVITY |  | 4 | -0.53 | -0.96 | 0.560 | 0.805 | 1.000 | 2596 | tags=50%, list=22%, signal=64% |
| 5348 | GOMF\_CARBOHYDRATE\_TRANSMEMBRANE\_TRANSPORTER\_ACTIVITY |  | 24 | -0.33 | -0.96 | 0.534 | 0.805 | 1.000 | 894 | tags=21%, list=7%, signal=22% |
| 5349 | GOBP\_RESPONSE\_TO\_DRUG |  | 250 | -0.25 | -0.96 | 0.616 | 0.805 | 1.000 | 2941 | tags=28%, list=24%, signal=37% |
| 5350 | GOBP\_EXECUTION\_PHASE\_OF\_APOPTOSIS |  | 56 | -0.29 | -0.96 | 0.553 | 0.805 | 1.000 | 3613 | tags=36%, list=30%, signal=51% |
| 5351 | GOBP\_POSITIVE\_REGULATION\_OF\_INTRINSIC\_APOPTOTIC\_SIGNALING\_PATHWAY\_IN\_RESPONSE\_TO\_DNA\_DAMAGE |  | 4 | -0.53 | -0.96 | 0.551 | 0.805 | 1.000 | 4618 | tags=75%, list=38%, signal=122% |
| 5352 | GOBP\_NEGATIVE\_REGULATION\_OF\_CREB\_TRANSCRIPTION\_FACTOR\_ACTIVITY |  | 3 | -0.57 | -0.96 | 0.553 | 0.805 | 1.000 | 2667 | tags=67%, list=22%, signal=86% |
| 5353 | GOBP\_MATING |  | 25 | -0.33 | -0.96 | 0.544 | 0.805 | 1.000 | 4984 | tags=60%, list=41%, signal=102% |
| 5354 | GOBP\_PYRIDINE\_CONTAINING\_COMPOUND\_METABOLIC\_PROCESS |  | 26 | -0.33 | -0.96 | 0.542 | 0.805 | 1.000 | 2865 | tags=27%, list=24%, signal=35% |
| 5355 | GOMF\_NUCLEASE\_ACTIVITY |  | 135 | -0.26 | -0.96 | 0.586 | 0.806 | 1.000 | 5337 | tags=47%, list=44%, signal=83% |
| 5356 | GOBP\_NEGATIVE\_REGULATION\_OF\_REACTIVE\_OXYGEN\_SPECIES\_BIOSYNTHETIC\_PROCESS |  | 19 | -0.35 | -0.96 | 0.536 | 0.806 | 1.000 | 3968 | tags=47%, list=33%, signal=71% |
| 5357 | GOBP\_GLOMERULAR\_MESANGIAL\_CELL\_PROLIFERATION |  | 8 | -0.44 | -0.96 | 0.554 | 0.806 | 1.000 | 3286 | tags=38%, list=27%, signal=52% |
| 5358 | GOBP\_REGULATION\_OF\_MACROPHAGE\_FUSION |  | 3 | -0.57 | -0.96 | 0.556 | 0.806 | 1.000 | 5154 | tags=100%, list=43%, signal=175% |
| 5359 | GOBP\_HISTONE\_H3\_K4\_DEMETHYLATION\_TRIMETHYL\_H3\_K4\_SPECIFIC |  | 3 | -0.58 | -0.96 | 0.559 | 0.806 | 1.000 | 977 | tags=33%, list=8%, signal=36% |
| 5360 | GOBP\_CELLULAR\_PIGMENTATION |  | 41 | -0.30 | -0.96 | 0.550 | 0.806 | 1.000 | 3569 | tags=34%, list=30%, signal=48% |
| 5361 | GOBP\_ECTODERMAL\_CELL\_DIFFERENTIATION |  | 3 | -0.57 | -0.96 | 0.569 | 0.806 | 1.000 | 3278 | tags=67%, list=27%, signal=92% |
| 5362 | GOBP\_EXTRACELLULAR\_MATRIX\_CELL\_SIGNALING |  | 5 | -0.50 | -0.96 | 0.541 | 0.806 | 1.000 | 575 | tags=20%, list=5%, signal=21% |
| 5363 | GOCC\_ANCHORED\_COMPONENT\_OF\_PRESYNAPTIC\_MEMBRANE |  | 5 | -0.50 | -0.96 | 0.542 | 0.806 | 1.000 | 6046 | tags=100%, list=50%, signal=201% |
| 5364 | GOBP\_BRANCHIOMOTOR\_NEURON\_AXON\_GUIDANCE |  | 3 | -0.57 | -0.96 | 0.549 | 0.806 | 1.000 | 1401 | tags=33%, list=12%, signal=38% |
| 5365 | GOCC\_GRANULAR\_COMPONENT |  | 5 | -0.50 | -0.96 | 0.555 | 0.805 | 1.000 | 303 | tags=20%, list=3%, signal=21% |
| 5366 | GOBP\_REGULATION\_OF\_BLOOD\_PRESSURE |  | 108 | -0.26 | -0.96 | 0.573 | 0.805 | 1.000 | 2895 | tags=26%, list=24%, signal=34% |
| 5367 | GOMF\_DYNEIN\_LIGHT\_INTERMEDIATE\_CHAIN\_BINDING |  | 14 | -0.37 | -0.96 | 0.548 | 0.806 | 1.000 | 1644 | tags=29%, list=14%, signal=33% |
| 5368 | GOBP\_URATE\_METABOLIC\_PROCESS |  | 4 | -0.53 | -0.96 | 0.548 | 0.806 | 1.000 | 1061 | tags=50%, list=9%, signal=55% |
| 5369 | GOBP\_POSITIVE\_REGULATION\_OF\_GLIAL\_CELL\_PROLIFERATION |  | 12 | -0.39 | -0.96 | 0.546 | 0.807 | 1.000 | 3132 | tags=42%, list=26%, signal=56% |
| 5370 | GOMF\_HYDROLASE\_ACTIVITY\_ACTING\_ON\_CARBON\_NITROGEN\_BUT\_NOT\_PEPTIDE\_BONDS\_IN\_LINEAR\_AMIDINES |  | 4 | -0.53 | -0.96 | 0.561 | 0.807 | 1.000 | 3653 | tags=75%, list=30%, signal=108% |
| 5371 | GOBP\_PERIPHERAL\_NERVOUS\_SYSTEM\_NEURON\_DIFFERENTIATION |  | 10 | -0.41 | -0.96 | 0.532 | 0.807 | 1.000 | 1749 | tags=20%, list=15%, signal=23% |
| 5372 | GOMF\_THIAMINE\_PYROPHOSPHATE\_BINDING |  | 6 | -0.47 | -0.96 | 0.551 | 0.807 | 1.000 | 1442 | tags=33%, list=12%, signal=38% |
| 5373 | GOBP\_NEGATIVE\_REGULATION\_OF\_CYCLIN\_DEPENDENT\_PROTEIN\_KINASE\_ACTIVITY |  | 20 | -0.34 | -0.96 | 0.539 | 0.807 | 1.000 | 3687 | tags=45%, list=31%, signal=65% |
| 5374 | GOBP\_DORSAL\_AORTA\_DEVELOPMENT |  | 9 | -0.42 | -0.96 | 0.547 | 0.807 | 1.000 | 3314 | tags=44%, list=28%, signal=61% |
| 5375 | GOBP\_NUCLEOBASE\_TRANSPORT |  | 6 | -0.47 | -0.96 | 0.548 | 0.807 | 1.000 | 1024 | tags=33%, list=9%, signal=36% |
| 5376 | GOMF\_PHOSPHOLIPID\_TRANSFER\_ACTIVITY |  | 17 | -0.36 | -0.96 | 0.534 | 0.807 | 1.000 | 2926 | tags=29%, list=24%, signal=39% |
| 5377 | GOBP\_NEGATIVE\_REGULATION\_OF\_PHAGOCYTOSIS |  | 14 | -0.38 | -0.96 | 0.538 | 0.808 | 1.000 | 4831 | tags=43%, list=40%, signal=72% |
| 5378 | GOMF\_SUMO\_POLYMER\_BINDING |  | 5 | -0.50 | -0.96 | 0.557 | 0.807 | 1.000 | 108 | tags=20%, list=1%, signal=20% |
| 5379 | GOBP\_INTRINSIC\_APOPTOTIC\_SIGNALING\_PATHWAY |  | 204 | -0.25 | -0.96 | 0.614 | 0.807 | 1.000 | 3692 | tags=33%, list=31%, signal=47% |
| 5380 | GOBP\_REGULATION\_OF\_MYOSIN\_LIGHT\_CHAIN\_PHOSPHATASE\_ACTIVITY |  | 6 | -0.47 | -0.96 | 0.546 | 0.807 | 1.000 | 6383 | tags=100%, list=53%, signal=213% |
| 5381 | GOBP\_VITAMIN\_TRANSMEMBRANE\_TRANSPORT |  | 12 | -0.39 | -0.96 | 0.541 | 0.808 | 1.000 | 3277 | tags=50%, list=27%, signal=69% |
| 5382 | GOBP\_NEGATIVE\_REGULATION\_OF\_LYASE\_ACTIVITY |  | 11 | -0.40 | -0.96 | 0.540 | 0.808 | 1.000 | 4984 | tags=55%, list=41%, signal=93% |
| 5383 | GOBP\_CELLULAR\_MACROMOLECULE\_CATABOLIC\_PROCESS |  | 903 | -0.24 | -0.96 | 0.705 | 0.807 | 1.000 | 4690 | tags=36%, list=39%, signal=55% |
| 5384 | GOBP\_ESTABLISHMENT\_OF\_MITOCHONDRION\_LOCALIZATION |  | 22 | -0.34 | -0.96 | 0.550 | 0.808 | 1.000 | 2050 | tags=23%, list=17%, signal=27% |
| 5385 | GOBP\_CENTROMERIC\_SISTER\_CHROMATID\_COHESION |  | 10 | -0.41 | -0.96 | 0.549 | 0.808 | 1.000 | 5866 | tags=70%, list=49%, signal=137% |
| 5386 | GOBP\_RESPONSE\_TO\_PEPTIDE\_HORMONE |  | 307 | -0.25 | -0.96 | 0.635 | 0.808 | 1.000 | 3701 | tags=39%, list=31%, signal=55% |
| 5387 | GOBP\_AUDITORY\_RECEPTOR\_CELL\_MORPHOGENESIS |  | 9 | -0.42 | -0.96 | 0.544 | 0.808 | 1.000 | 4949 | tags=67%, list=41%, signal=113% |
| 5388 | GOMF\_PROTEIN\_SELF\_ASSOCIATION |  | 39 | -0.30 | -0.96 | 0.548 | 0.808 | 1.000 | 2466 | tags=31%, list=21%, signal=39% |
| 5389 | GOBP\_POSITIVE\_REGULATION\_OF\_MRNA\_METABOLIC\_PROCESS |  | 66 | -0.28 | -0.96 | 0.569 | 0.808 | 1.000 | 4667 | tags=48%, list=39%, signal=79% |
| 5390 | GOBP\_NEGATIVE\_REGULATION\_OF\_HELICASE\_ACTIVITY |  | 3 | -0.58 | -0.96 | 0.564 | 0.809 | 1.000 | 2756 | tags=67%, list=23%, signal=86% |
| 5391 | GOCC\_UNCONVENTIONAL\_MYOSIN\_COMPLEX |  | 8 | -0.43 | -0.96 | 0.547 | 0.809 | 1.000 | 4025 | tags=50%, list=34%, signal=75% |
| 5392 | GOBP\_NEGATIVE\_REGULATION\_OF\_CANONICAL\_WNT\_SIGNALING\_PATHWAY |  | 129 | -0.26 | -0.96 | 0.587 | 0.809 | 1.000 | 2442 | tags=21%, list=20%, signal=26% |
| 5393 | GOCC\_OXOGLUTARATE\_DEHYDROGENASE\_COMPLEX |  | 6 | -0.47 | -0.96 | 0.552 | 0.809 | 1.000 | 931 | tags=17%, list=8%, signal=18% |
| 5394 | GOMF\_CALCIUM\_TRANSMEMBRANE\_TRANSPORTER\_ACTIVITY\_PHOSPHORYLATIVE\_MECHANISM |  | 7 | -0.45 | -0.96 | 0.544 | 0.809 | 1.000 | 5903 | tags=71%, list=49%, signal=140% |
| 5395 | GOBP\_REGULATION\_OF\_RECEPTOR\_CLUSTERING |  | 8 | -0.43 | -0.95 | 0.548 | 0.809 | 1.000 | 90 | tags=13%, list=1%, signal=13% |
| 5396 | GOBP\_REGULATION\_OF\_CELLULAR\_CARBOHYDRATE\_METABOLIC\_PROCESS |  | 96 | -0.27 | -0.95 | 0.579 | 0.809 | 1.000 | 4280 | tags=39%, list=36%, signal=59% |
| 5397 | GOMF\_NUCLEOBASE\_TRANSMEMBRANE\_TRANSPORTER\_ACTIVITY |  | 5 | -0.50 | -0.95 | 0.570 | 0.809 | 1.000 | 1024 | tags=40%, list=9%, signal=44% |
| 5398 | GOMF\_CHONDROITIN\_SULFOTRANSFERASE\_ACTIVITY |  | 4 | -0.52 | -0.95 | 0.555 | 0.809 | 1.000 | 270 | tags=25%, list=2%, signal=26% |
| 5399 | GOBP\_NOREPINEPHRINE\_TRANSPORT |  | 15 | -0.37 | -0.95 | 0.543 | 0.809 | 1.000 | 2633 | tags=27%, list=22%, signal=34% |
| 5400 | GOMF\_TRANSFORMING\_GROWTH\_FACTOR\_BETA\_ACTIVATED\_RECEPTOR\_ACTIVITY |  | 10 | -0.41 | -0.95 | 0.549 | 0.810 | 1.000 | 3064 | tags=30%, list=26%, signal=40% |
| 5401 | GOBP\_NEGATIVE\_REGULATION\_OF\_PROTEIN\_BINDING |  | 58 | -0.28 | -0.95 | 0.569 | 0.810 | 1.000 | 2308 | tags=21%, list=19%, signal=25% |
| 5402 | GOBP\_LEFT\_RIGHT\_PATTERN\_FORMATION |  | 13 | -0.38 | -0.95 | 0.543 | 0.810 | 1.000 | 1764 | tags=23%, list=15%, signal=27% |
| 5403 | GOBP\_DNA\_DEALKYLATION |  | 20 | -0.34 | -0.95 | 0.546 | 0.811 | 1.000 | 3598 | tags=35%, list=30%, signal=50% |
| 5404 | GOBP\_RESPONSE\_TO\_STEROL |  | 23 | -0.33 | -0.95 | 0.546 | 0.811 | 1.000 | 4506 | tags=43%, list=38%, signal=69% |
| 5405 | GOBP\_SA\_NODE\_CELL\_TO\_ATRIAL\_CARDIAC\_MUSCLE\_CELL\_SIGNALING |  | 6 | -0.47 | -0.95 | 0.553 | 0.811 | 1.000 | 3295 | tags=50%, list=27%, signal=69% |
| 5406 | GOCC\_COSTAMERE |  | 14 | -0.37 | -0.95 | 0.547 | 0.811 | 1.000 | 4110 | tags=36%, list=34%, signal=54% |
| 5407 | GOBP\_RESPONSE\_TO\_CORTISOL |  | 5 | -0.49 | -0.95 | 0.559 | 0.811 | 1.000 | 4431 | tags=60%, list=37%, signal=95% |
| 5408 | GOBP\_MACROPHAGE\_FUSION |  | 4 | -0.52 | -0.95 | 0.560 | 0.811 | 1.000 | 5154 | tags=75%, list=43%, signal=131% |
| 5409 | GOBP\_REGULATION\_OF\_PLATELET\_DERIVED\_GROWTH\_FACTOR\_RECEPTOR\_SIGNALING\_PATHWAY |  | 21 | -0.34 | -0.95 | 0.556 | 0.811 | 1.000 | 3007 | tags=29%, list=25%, signal=38% |
| 5410 | GOBP\_RESPONSE\_TO\_PROTOZOAN |  | 9 | -0.42 | -0.95 | 0.547 | 0.811 | 1.000 | 5403 | tags=78%, list=45%, signal=141% |
| 5411 | GOBP\_PROSTATE\_GLAND\_DEVELOPMENT |  | 33 | -0.31 | -0.95 | 0.558 | 0.811 | 1.000 | 1579 | tags=24%, list=13%, signal=28% |
| 5412 | GOBP\_MONOSACCHARIDE\_METABOLIC\_PROCESS |  | 195 | -0.25 | -0.95 | 0.618 | 0.812 | 1.000 | 4302 | tags=41%, list=36%, signal=62% |
| 5413 | GOBP\_REGULATION\_OF\_RETROGRADE\_PROTEIN\_TRANSPORT\_ER\_TO\_CYTOSOL |  | 11 | -0.40 | -0.95 | 0.550 | 0.812 | 1.000 | 2715 | tags=27%, list=23%, signal=35% |
| 5414 | GOCC\_U5\_SNRNP |  | 14 | -0.37 | -0.95 | 0.540 | 0.812 | 1.000 | 3604 | tags=29%, list=30%, signal=41% |
| 5415 | GOBP\_MICROTUBULE\_BASED\_TRANSPORT |  | 143 | -0.26 | -0.95 | 0.604 | 0.814 | 1.000 | 3668 | tags=31%, list=31%, signal=45% |
| 5416 | GOBP\_EPITHELIAL\_CELL\_DIFFERENTIATION\_INVOLVED\_IN\_KIDNEY\_DEVELOPMENT |  | 34 | -0.31 | -0.95 | 0.557 | 0.814 | 1.000 | 4102 | tags=41%, list=34%, signal=62% |
| 5417 | GOBP\_POSITIVE\_REGULATION\_OF\_CYTOPLASMIC\_TRANSLATION |  | 8 | -0.43 | -0.95 | 0.547 | 0.814 | 1.000 | 4569 | tags=38%, list=38%, signal=60% |
| 5418 | GOCC\_LUMENAL\_SIDE\_OF\_ENDOPLASMIC\_RETICULUM\_MEMBRANE |  | 10 | -0.41 | -0.95 | 0.546 | 0.814 | 1.000 | 1618 | tags=30%, list=13%, signal=35% |
| 5419 | GOBP\_NEGATIVE\_REGULATION\_OF\_INTERLEUKIN\_1\_BETA\_PRODUCTION |  | 14 | -0.37 | -0.95 | 0.545 | 0.814 | 1.000 | 2871 | tags=36%, list=24%, signal=47% |
| 5420 | GOBP\_POST\_CHAPERONIN\_TUBULIN\_FOLDING\_PATHWAY |  | 5 | -0.50 | -0.95 | 0.560 | 0.814 | 1.000 | 1915 | tags=20%, list=16%, signal=24% |
| 5421 | GOCC\_CONNEXIN\_COMPLEX |  | 8 | -0.43 | -0.95 | 0.545 | 0.814 | 1.000 | 2313 | tags=50%, list=19%, signal=62% |
| 5422 | GOCC\_CUL4A\_RING\_E3\_UBIQUITIN\_LIGASE\_COMPLEX |  | 11 | -0.40 | -0.95 | 0.552 | 0.814 | 1.000 | 3958 | tags=55%, list=33%, signal=81% |
| 5423 | GOBP\_REGULATION\_OF\_ANDROGEN\_RECEPTOR\_SIGNALING\_PATHWAY |  | 18 | -0.35 | -0.95 | 0.543 | 0.814 | 1.000 | 4337 | tags=44%, list=36%, signal=69% |
| 5424 | GOBP\_NEGATIVE\_REGULATION\_OF\_GENE\_EXPRESSION |  | 776 | -0.24 | -0.95 | 0.718 | 0.814 | 1.000 | 3730 | tags=29%, list=31%, signal=40% |
| 5425 | GOCC\_PHOSPHATASE\_COMPLEX |  | 38 | -0.30 | -0.95 | 0.559 | 0.814 | 1.000 | 4120 | tags=39%, list=34%, signal=60% |
| 5426 | GOMF\_PYRIMIDINE\_NUCLEOTIDE\_SUGAR\_TRANSMEMBRANE\_TRANSPORTER\_ACTIVITY |  | 6 | -0.47 | -0.95 | 0.556 | 0.814 | 1.000 | 2868 | tags=33%, list=24%, signal=44% |
| 5427 | GOCC\_CONDENSED\_CHROMOSOME\_INNER\_KINETOCHORE |  | 5 | -0.49 | -0.95 | 0.564 | 0.815 | 1.000 | 3577 | tags=60%, list=30%, signal=85% |
| 5428 | GOMF\_NEUREGULIN\_BINDING |  | 5 | -0.50 | -0.95 | 0.570 | 0.815 | 1.000 | 3856 | tags=60%, list=32%, signal=88% |
| 5429 | GOBP\_EPITHELIAL\_CELL\_DEVELOPMENT |  | 152 | -0.25 | -0.95 | 0.614 | 0.815 | 1.000 | 4447 | tags=42%, list=37%, signal=66% |
| 5430 | GOBP\_INDUCTION\_OF\_POSITIVE\_CHEMOTAXIS |  | 9 | -0.42 | -0.95 | 0.556 | 0.816 | 1.000 | 3196 | tags=44%, list=27%, signal=61% |
| 5431 | GOBP\_NEURONAL\_ION\_CHANNEL\_CLUSTERING |  | 8 | -0.43 | -0.95 | 0.564 | 0.816 | 1.000 | 5462 | tags=63%, list=45%, signal=115% |
| 5432 | GOBP\_MESODERMAL\_CELL\_DIFFERENTIATION |  | 19 | -0.35 | -0.95 | 0.549 | 0.816 | 1.000 | 3856 | tags=32%, list=32%, signal=46% |
| 5433 | GOBP\_NLRP3\_INFLAMMASOME\_COMPLEX\_ASSEMBLY |  | 8 | -0.43 | -0.95 | 0.557 | 0.817 | 1.000 | 1236 | tags=38%, list=10%, signal=42% |
| 5434 | GOMF\_DOUBLE\_STRANDED\_TELOMERIC\_DNA\_BINDING |  | 8 | -0.43 | -0.95 | 0.559 | 0.817 | 1.000 | 3927 | tags=75%, list=33%, signal=111% |
| 5435 | GOBP\_ESTABLISHMENT\_OF\_EPITHELIAL\_CELL\_APICAL\_BASAL\_POLARITY |  | 12 | -0.39 | -0.95 | 0.548 | 0.817 | 1.000 | 4949 | tags=42%, list=41%, signal=71% |
| 5436 | GOBP\_REGULATION\_OF\_AUTOPHAGY |  | 257 | -0.25 | -0.95 | 0.640 | 0.817 | 1.000 | 4324 | tags=38%, list=36%, signal=58% |
| 5437 | GOBP\_POSITIVE\_REGULATION\_OF\_ACTIVIN\_RECEPTOR\_SIGNALING\_PATHWAY |  | 4 | -0.53 | -0.95 | 0.567 | 0.818 | 1.000 | 3657 | tags=75%, list=30%, signal=108% |
| 5438 | GOBP\_PROTEIN\_AUTOUBIQUITINATION |  | 51 | -0.29 | -0.95 | 0.569 | 0.818 | 1.000 | 6115 | tags=61%, list=51%, signal=123% |
| 5439 | GOBP\_CGMP\_MEDIATED\_SIGNALING |  | 13 | -0.38 | -0.95 | 0.551 | 0.818 | 1.000 | 2820 | tags=31%, list=23%, signal=40% |
| 5440 | GOBP\_PHOSPHATIDYLCHOLINE\_ACYL\_CHAIN\_REMODELING |  | 14 | -0.37 | -0.95 | 0.552 | 0.818 | 1.000 | 957 | tags=21%, list=8%, signal=23% |
| 5441 | GOBP\_REGULATION\_OF\_STEROID\_HORMONE\_BIOSYNTHETIC\_PROCESS |  | 9 | -0.42 | -0.95 | 0.561 | 0.818 | 1.000 | 2059 | tags=44%, list=17%, signal=54% |
| 5442 | GOMF\_POTASSIUM\_ION\_TRANSMEMBRANE\_TRANSPORTER\_ACTIVITY |  | 78 | -0.27 | -0.95 | 0.586 | 0.818 | 1.000 | 1632 | tags=19%, list=14%, signal=22% |
| 5443 | GOBP\_DNA\_LIGATION\_INVOLVED\_IN\_DNA\_REPAIR |  | 4 | -0.52 | -0.95 | 0.575 | 0.818 | 1.000 | 4773 | tags=75%, list=40%, signal=124% |
| 5444 | GOBP\_ISOPRENOID\_METABOLIC\_PROCESS |  | 84 | -0.27 | -0.95 | 0.593 | 0.818 | 1.000 | 2278 | tags=20%, list=19%, signal=25% |
| 5445 | GOBP\_TELOMERASE\_RNA\_LOCALIZATION |  | 16 | -0.36 | -0.95 | 0.559 | 0.818 | 1.000 | 292 | tags=6%, list=2%, signal=6% |
| 5446 | GOBP\_NEGATIVE\_REGULATION\_OF\_PROTEOLYSIS |  | 181 | -0.25 | -0.95 | 0.628 | 0.818 | 1.000 | 3345 | tags=27%, list=28%, signal=37% |
| 5447 | GOBP\_PEPTIDYL\_LYSINE\_DIMETHYLATION |  | 14 | -0.37 | -0.95 | 0.559 | 0.818 | 1.000 | 4608 | tags=64%, list=38%, signal=104% |
| 5448 | GOBP\_3\_UTR\_MEDIATED\_MRNA\_STABILIZATION |  | 13 | -0.38 | -0.95 | 0.548 | 0.819 | 1.000 | 5048 | tags=62%, list=42%, signal=106% |
| 5449 | GOBP\_NUCLEOTIDE\_EXCISION\_REPAIR\_PREINCISION\_COMPLEX\_ASSEMBLY |  | 26 | -0.32 | -0.95 | 0.552 | 0.819 | 1.000 | 5370 | tags=65%, list=45%, signal=118% |
| 5450 | GOBP\_REGULATION\_OF\_DNA\_BINDING |  | 79 | -0.27 | -0.95 | 0.588 | 0.818 | 1.000 | 3376 | tags=29%, list=28%, signal=40% |
| 5451 | GOBP\_OVULATION\_CYCLE\_PROCESS |  | 29 | -0.31 | -0.95 | 0.564 | 0.819 | 1.000 | 4380 | tags=48%, list=36%, signal=76% |
| 5452 | GOBP\_HISTONE\_H3\_K36\_METHYLATION |  | 11 | -0.40 | -0.95 | 0.548 | 0.819 | 1.000 | 2639 | tags=36%, list=22%, signal=47% |
| 5453 | GOBP\_RESPONSE\_TO\_LEUKEMIA\_INHIBITORY\_FACTOR |  | 68 | -0.28 | -0.95 | 0.583 | 0.819 | 1.000 | 4455 | tags=41%, list=37%, signal=65% |
| 5454 | GOBP\_CELL\_MIGRATION\_INVOLVED\_IN\_GASTRULATION |  | 9 | -0.42 | -0.95 | 0.555 | 0.819 | 1.000 | 5235 | tags=78%, list=44%, signal=138% |
| 5455 | GOBP\_SKELETAL\_MUSCLE\_THIN\_FILAMENT\_ASSEMBLY |  | 3 | -0.56 | -0.95 | 0.577 | 0.819 | 1.000 | 149 | tags=33%, list=1%, signal=34% |
| 5456 | GOBP\_LATE\_ENDOSOME\_TO\_VACUOLE\_TRANSPORT\_VIA\_MULTIVESICULAR\_BODY\_SORTING\_PATHWAY |  | 14 | -0.37 | -0.95 | 0.559 | 0.819 | 1.000 | 1824 | tags=21%, list=15%, signal=25% |
| 5457 | GOCC\_PI\_BODY |  | 3 | -0.57 | -0.95 | 0.572 | 0.819 | 1.000 | 189 | tags=33%, list=2%, signal=34% |
| 5458 | GOBP\_REGULATION\_OF\_CDC42\_PROTEIN\_SIGNAL\_TRANSDUCTION |  | 4 | -0.52 | -0.95 | 0.574 | 0.819 | 1.000 | 2752 | tags=50%, list=23%, signal=65% |
| 5459 | GOMF\_GAP\_JUNCTION\_CHANNEL\_ACTIVITY |  | 8 | -0.43 | -0.95 | 0.552 | 0.819 | 1.000 | 2313 | tags=50%, list=19%, signal=62% |
| 5460 | GOBP\_CELL\_JUNCTION\_MAINTENANCE |  | 19 | -0.35 | -0.95 | 0.562 | 0.820 | 1.000 | 3093 | tags=47%, list=26%, signal=64% |
| 5461 | GOBP\_MAINTENANCE\_OF\_DNA\_METHYLATION |  | 6 | -0.47 | -0.95 | 0.567 | 0.820 | 1.000 | 5867 | tags=83%, list=49%, signal=163% |
| 5462 | GOBP\_CARDIAC\_MUSCLE\_CELL\_ACTION\_POTENTIAL\_INVOLVED\_IN\_CONTRACTION |  | 37 | -0.30 | -0.95 | 0.555 | 0.820 | 1.000 | 3918 | tags=35%, list=33%, signal=52% |
| 5463 | GOBP\_IMMUNOLOGICAL\_MEMORY\_FORMATION\_PROCESS |  | 5 | -0.49 | -0.95 | 0.580 | 0.820 | 1.000 | 1709 | tags=40%, list=14%, signal=47% |
| 5464 | GOBP\_POSITIVE\_REGULATION\_OF\_HEMATOPOIETIC\_PROGENITOR\_CELL\_DIFFERENTIATION |  | 4 | -0.53 | -0.95 | 0.571 | 0.820 | 1.000 | 5139 | tags=75%, list=43%, signal=131% |
| 5465 | GOMF\_CROSSOVER\_JUNCTION\_ENDODEOXYRIBONUCLEASE\_ACTIVITY |  | 4 | -0.52 | -0.95 | 0.578 | 0.820 | 1.000 | 5752 | tags=100%, list=48%, signal=192% |
| 5466 | GOBP\_POSITIVE\_REGULATION\_OF\_AMYLOID\_BETA\_CLEARANCE |  | 4 | -0.52 | -0.95 | 0.577 | 0.820 | 1.000 | 5744 | tags=100%, list=48%, signal=192% |
| 5467 | GOBP\_INFLAMMATORY\_CELL\_APOPTOTIC\_PROCESS |  | 12 | -0.39 | -0.95 | 0.553 | 0.820 | 1.000 | 2656 | tags=50%, list=22%, signal=64% |
| 5468 | GOBP\_LOCALIZATION\_WITHIN\_MEMBRANE |  | 49 | -0.29 | -0.95 | 0.567 | 0.820 | 1.000 | 3914 | tags=33%, list=33%, signal=48% |
| 5469 | GOBP\_LUNG\_LOBE\_DEVELOPMENT |  | 5 | -0.49 | -0.95 | 0.561 | 0.820 | 1.000 | 5879 | tags=80%, list=49%, signal=157% |
| 5470 | GOBP\_OLIGODENDROCYTE\_PROGENITOR\_PROLIFERATION |  | 6 | -0.47 | -0.95 | 0.570 | 0.820 | 1.000 | 1847 | tags=33%, list=15%, signal=39% |
| 5471 | GOBP\_CARDIAC\_MUSCLE\_TISSUE\_MORPHOGENESIS |  | 33 | -0.31 | -0.95 | 0.565 | 0.820 | 1.000 | 3615 | tags=33%, list=30%, signal=48% |
| 5472 | GOBP\_REGULATION\_OF\_EPITHELIAL\_CELL\_APOPTOTIC\_PROCESS |  | 55 | -0.28 | -0.95 | 0.581 | 0.819 | 1.000 | 2820 | tags=27%, list=23%, signal=35% |
| 5473 | GOBP\_REGULATION\_OF\_TRANSPOSITION |  | 7 | -0.44 | -0.95 | 0.558 | 0.820 | 1.000 | 529 | tags=29%, list=4%, signal=30% |
| 5474 | GOBP\_CHAPERONE\_COFACTOR\_DEPENDENT\_PROTEIN\_REFOLDING |  | 23 | -0.33 | -0.95 | 0.566 | 0.820 | 1.000 | 2394 | tags=26%, list=20%, signal=33% |
| 5475 | GOMF\_EXONUCLEASE\_ACTIVITY\_ACTIVE\_WITH\_EITHER\_RIBO\_OR\_DEOXYRIBONUCLEIC\_ACIDS\_AND\_PRODUCING\_5\_PHOSPHOMONOESTERS |  | 45 | -0.29 | -0.95 | 0.579 | 0.820 | 1.000 | 5337 | tags=47%, list=44%, signal=84% |
| 5476 | GOBP\_POSITIVE\_REGULATION\_OF\_CELL\_CELL\_ADHESION\_MEDIATED\_BY\_INTEGRIN |  | 6 | -0.46 | -0.94 | 0.561 | 0.820 | 1.000 | 1774 | tags=50%, list=15%, signal=59% |
| 5477 | GOBP\_CELLULAR\_RESPONSE\_TO\_TESTOSTERONE\_STIMULUS |  | 7 | -0.45 | -0.94 | 0.569 | 0.820 | 1.000 | 3782 | tags=57%, list=31%, signal=83% |
| 5478 | GOBP\_MONOACYLGLYCEROL\_BIOSYNTHETIC\_PROCESS |  | 3 | -0.56 | -0.94 | 0.582 | 0.820 | 1.000 | 5263 | tags=100%, list=44%, signal=178% |
| 5479 | GOMF\_PROCOLLAGEN\_PROLINE\_DIOXYGENASE\_ACTIVITY |  | 8 | -0.43 | -0.94 | 0.557 | 0.820 | 1.000 | 3164 | tags=38%, list=26%, signal=51% |
| 5480 | GOBP\_SEQUESTERING\_OF\_METAL\_ION |  | 6 | -0.46 | -0.94 | 0.566 | 0.820 | 1.000 | 2899 | tags=67%, list=24%, signal=88% |
| 5481 | GOBP\_REGULATION\_OF\_BRANCHING\_MORPHOGENESIS\_OF\_A\_NERVE |  | 4 | -0.52 | -0.94 | 0.575 | 0.820 | 1.000 | 3483 | tags=75%, list=29%, signal=106% |
| 5482 | GOBP\_N\_TERMINAL\_PEPTIDYL\_METHIONINE\_ACETYLATION |  | 6 | -0.46 | -0.94 | 0.566 | 0.821 | 1.000 | 4976 | tags=67%, list=41%, signal=114% |
| 5483 | GOMF\_POTASSIUM\_ION\_LEAK\_CHANNEL\_ACTIVITY |  | 8 | -0.43 | -0.94 | 0.561 | 0.821 | 1.000 | 1183 | tags=25%, list=10%, signal=28% |
| 5484 | GOBP\_POSITIVE\_REGULATION\_OF\_INTERLEUKIN\_17\_PRODUCTION |  | 7 | -0.44 | -0.94 | 0.568 | 0.821 | 1.000 | 3155 | tags=57%, list=26%, signal=77% |
| 5485 | GOBP\_NEGATIVE\_REGULATION\_OF\_OSTEOBLAST\_DIFFERENTIATION |  | 33 | -0.31 | -0.94 | 0.567 | 0.821 | 1.000 | 2002 | tags=21%, list=17%, signal=25% |
| 5486 | GOBP\_REGULATION\_OF\_BIOMINERALIZATION |  | 56 | -0.28 | -0.94 | 0.582 | 0.821 | 1.000 | 3488 | tags=34%, list=29%, signal=48% |
| 5487 | GOBP\_POSITIVE\_REGULATION\_OF\_LIPID\_CATABOLIC\_PROCESS |  | 16 | -0.36 | -0.94 | 0.557 | 0.821 | 1.000 | 3919 | tags=44%, list=33%, signal=65% |
| 5488 | GOBP\_TRANSPOSITION |  | 7 | -0.44 | -0.94 | 0.564 | 0.821 | 1.000 | 529 | tags=29%, list=4%, signal=30% |
| 5489 | GOBP\_CERAMIDE\_TRANSPORT |  | 9 | -0.41 | -0.94 | 0.556 | 0.821 | 1.000 | 2926 | tags=44%, list=24%, signal=59% |
| 5490 | GOBP\_HEPARAN\_SULFATE\_PROTEOGLYCAN\_METABOLIC\_PROCESS |  | 25 | -0.32 | -0.94 | 0.566 | 0.821 | 1.000 | 5140 | tags=64%, list=43%, signal=112% |
| 5491 | GOBP\_PHOSPHORYLATION\_OF\_RNA\_POLYMERASE\_II\_C\_TERMINAL\_DOMAIN |  | 11 | -0.39 | -0.94 | 0.557 | 0.821 | 1.000 | 7304 | tags=100%, list=61%, signal=255% |
| 5492 | GOBP\_REGULATION\_OF\_SIGNAL\_TRANSDUCTION\_BY\_P53\_CLASS\_MEDIATOR |  | 142 | -0.25 | -0.94 | 0.613 | 0.821 | 1.000 | 3888 | tags=30%, list=32%, signal=43% |
| 5493 | GOBP\_RESPONSE\_TO\_REACTIVE\_OXYGEN\_SPECIES |  | 158 | -0.25 | -0.94 | 0.625 | 0.821 | 1.000 | 3650 | tags=30%, list=30%, signal=42% |
| 5494 | GOBP\_POSITIVE\_REGULATION\_OF\_PODOSOME\_ASSEMBLY |  | 5 | -0.49 | -0.94 | 0.573 | 0.821 | 1.000 | 6150 | tags=100%, list=51%, signal=205% |
| 5495 | GOBP\_INTESTINAL\_HEXOSE\_ABSORPTION |  | 5 | -0.49 | -0.94 | 0.573 | 0.821 | 1.000 | 5427 | tags=80%, list=45%, signal=146% |
| 5496 | GOBP\_POSITIVE\_REGULATION\_OF\_VASCULAR\_ENDOTHELIAL\_GROWTH\_FACTOR\_SIGNALING\_PATHWAY |  | 5 | -0.49 | -0.94 | 0.580 | 0.821 | 1.000 | 257 | tags=20%, list=2%, signal=20% |
| 5497 | GOBP\_NUCLEOTIDE\_SALVAGE |  | 12 | -0.38 | -0.94 | 0.557 | 0.820 | 1.000 | 786 | tags=17%, list=7%, signal=18% |
| 5498 | GOBP\_ADULT\_LOCOMOTORY\_BEHAVIOR |  | 37 | -0.30 | -0.94 | 0.565 | 0.821 | 1.000 | 2563 | tags=27%, list=21%, signal=34% |
| 5499 | GOBP\_MONOCYTE\_AGGREGATION |  | 5 | -0.49 | -0.94 | 0.572 | 0.821 | 1.000 | 1935 | tags=40%, list=16%, signal=48% |
| 5500 | GOCC\_EXCITATORY\_SYNAPSE |  | 20 | -0.34 | -0.94 | 0.558 | 0.821 | 1.000 | 3797 | tags=45%, list=32%, signal=66% |
| 5501 | GOMF\_PROTEIN\_HOMODIMERIZATION\_ACTIVITY |  | 461 | -0.24 | -0.94 | 0.700 | 0.821 | 1.000 | 3768 | tags=30%, list=31%, signal=43% |
| 5502 | GOBP\_SPINDLE\_MIDZONE\_ASSEMBLY |  | 9 | -0.42 | -0.94 | 0.558 | 0.821 | 1.000 | 4165 | tags=56%, list=35%, signal=85% |
| 5503 | GOMF\_NADPLUS\_BINDING |  | 12 | -0.39 | -0.94 | 0.562 | 0.821 | 1.000 | 2698 | tags=33%, list=22%, signal=43% |
| 5504 | GOBP\_MACROPHAGE\_APOPTOTIC\_PROCESS |  | 7 | -0.44 | -0.94 | 0.568 | 0.821 | 1.000 | 2656 | tags=57%, list=22%, signal=73% |
| 5505 | GOMF\_RNA\_POLYMERASE\_II\_C\_TERMINAL\_DOMAIN\_BINDING |  | 7 | -0.44 | -0.94 | 0.571 | 0.821 | 1.000 | 6008 | tags=86%, list=50%, signal=171% |
| 5506 | GOBP\_ERYTHROCYTE\_DEVELOPMENT |  | 25 | -0.32 | -0.94 | 0.566 | 0.821 | 1.000 | 1872 | tags=28%, list=16%, signal=33% |
| 5507 | GOCC\_RUFFLE\_MEMBRANE |  | 68 | -0.27 | -0.94 | 0.583 | 0.822 | 1.000 | 3575 | tags=34%, list=30%, signal=48% |
| 5508 | GOBP\_ZYMOGEN\_INHIBITION |  | 3 | -0.57 | -0.94 | 0.586 | 0.821 | 1.000 | 3289 | tags=67%, list=27%, signal=92% |
| 5509 | GOMF\_N\_ACYLSPHINGOSINE\_AMIDOHYDROLASE\_ACTIVITY |  | 5 | -0.49 | -0.94 | 0.578 | 0.822 | 1.000 | 5114 | tags=80%, list=43%, signal=139% |
| 5510 | GOMF\_U1\_SNRNA\_BINDING |  | 4 | -0.52 | -0.94 | 0.583 | 0.822 | 1.000 | 3604 | tags=50%, list=30%, signal=71% |
| 5511 | GOCC\_NEUROTRANSMITTER\_RECEPTOR\_COMPLEX |  | 16 | -0.36 | -0.94 | 0.558 | 0.822 | 1.000 | 3699 | tags=44%, list=31%, signal=63% |
| 5512 | GOBP\_CIRCADIAN\_REGULATION\_OF\_GENE\_EXPRESSION |  | 52 | -0.28 | -0.94 | 0.583 | 0.823 | 1.000 | 2907 | tags=33%, list=24%, signal=43% |
| 5513 | GOBP\_SPINAL\_CORD\_OLIGODENDROCYTE\_CELL\_DIFFERENTIATION |  | 4 | -0.52 | -0.94 | 0.578 | 0.823 | 1.000 | 1881 | tags=50%, list=16%, signal=59% |
| 5514 | GOMF\_CHEMOATTRACTANT\_ACTIVITY |  | 20 | -0.34 | -0.94 | 0.566 | 0.823 | 1.000 | 1181 | tags=20%, list=10%, signal=22% |
| 5515 | GOMF\_ZINC\_ION\_TRANSMEMBRANE\_TRANSPORTER\_ACTIVITY |  | 11 | -0.39 | -0.94 | 0.562 | 0.823 | 1.000 | 2899 | tags=36%, list=24%, signal=48% |
| 5516 | GOBP\_OLEFINIC\_COMPOUND\_METABOLIC\_PROCESS |  | 65 | -0.27 | -0.94 | 0.591 | 0.823 | 1.000 | 2705 | tags=28%, list=23%, signal=36% |
| 5517 | GOBP\_GLOMERULAR\_VISCERAL\_EPITHELIAL\_CELL\_MIGRATION |  | 5 | -0.49 | -0.94 | 0.579 | 0.823 | 1.000 | 308 | tags=20%, list=3%, signal=21% |
| 5518 | GOBP\_NEGATIVE\_REGULATION\_BY\_HOST\_OF\_VIRAL\_PROCESS |  | 9 | -0.41 | -0.94 | 0.567 | 0.823 | 1.000 | 3968 | tags=33%, list=33%, signal=50% |
| 5519 | GOBP\_RAB\_PROTEIN\_SIGNAL\_TRANSDUCTION |  | 17 | -0.35 | -0.94 | 0.561 | 0.823 | 1.000 | 5570 | tags=65%, list=46%, signal=120% |
| 5520 | GOBP\_IRON\_ION\_TRANSMEMBRANE\_TRANSPORT |  | 14 | -0.37 | -0.94 | 0.563 | 0.823 | 1.000 | 3318 | tags=36%, list=28%, signal=49% |
| 5521 | GOBP\_CELLULAR\_RESPONSE\_TO\_MOLECULE\_OF\_BACTERIAL\_ORIGIN |  | 113 | -0.26 | -0.94 | 0.618 | 0.823 | 1.000 | 3695 | tags=35%, list=31%, signal=49% |
| 5522 | GOBP\_REGULATION\_OF\_ACTIVIN\_RECEPTOR\_SIGNALING\_PATHWAY |  | 16 | -0.35 | -0.94 | 0.565 | 0.823 | 1.000 | 4105 | tags=56%, list=34%, signal=85% |
| 5523 | GOBP\_REGULATION\_OF\_CELLULAR\_AMIDE\_METABOLIC\_PROCESS |  | 319 | -0.24 | -0.94 | 0.679 | 0.823 | 1.000 | 3968 | tags=30%, list=33%, signal=44% |
| 5524 | GOCC\_DYNEIN\_COMPLEX |  | 26 | -0.32 | -0.94 | 0.564 | 0.823 | 1.000 | 3505 | tags=35%, list=29%, signal=49% |
| 5525 | GOBP\_REGULATION\_OF\_VASCULOGENESIS |  | 14 | -0.37 | -0.94 | 0.554 | 0.823 | 1.000 | 4355 | tags=43%, list=36%, signal=67% |
| 5526 | GOBP\_REGULATION\_OF\_CELLULAR\_PROTEIN\_CATABOLIC\_PROCESS |  | 186 | -0.25 | -0.94 | 0.640 | 0.823 | 1.000 | 3715 | tags=29%, list=31%, signal=41% |
| 5527 | GOBP\_REGULATION\_OF\_ALCOHOL\_BIOSYNTHETIC\_PROCESS |  | 56 | -0.28 | -0.94 | 0.593 | 0.825 | 1.000 | 4272 | tags=46%, list=36%, signal=72% |
| 5528 | GOBP\_THYROID\_HORMONE\_TRANSPORT |  | 6 | -0.46 | -0.94 | 0.565 | 0.825 | 1.000 | 3930 | tags=33%, list=33%, signal=50% |
| 5529 | GOBP\_T\_HELPER\_CELL\_LINEAGE\_COMMITMENT |  | 9 | -0.41 | -0.94 | 0.566 | 0.825 | 1.000 | 2308 | tags=33%, list=19%, signal=41% |
| 5530 | GOBP\_NEGATIVE\_REGULATION\_OF\_FIBROBLAST\_GROWTH\_FACTOR\_RECEPTOR\_SIGNALING\_PATHWAY |  | 14 | -0.37 | -0.94 | 0.561 | 0.826 | 1.000 | 3530 | tags=50%, list=29%, signal=71% |
| 5531 | GOBP\_POSITIVE\_REGULATION\_OF\_PROTEIN\_NEDDYLATION |  | 5 | -0.48 | -0.94 | 0.581 | 0.826 | 1.000 | 916 | tags=20%, list=8%, signal=22% |
| 5532 | GOBP\_CELLULAR\_RESPONSE\_TO\_STEROL\_DEPLETION |  | 13 | -0.37 | -0.94 | 0.569 | 0.826 | 1.000 | 1393 | tags=23%, list=12%, signal=26% |
| 5533 | GOBP\_TRANSMEMBRANE\_RECEPTOR\_PROTEIN\_TYROSINE\_PHOSPHATASE\_SIGNALING\_PATHWAY |  | 6 | -0.46 | -0.94 | 0.569 | 0.826 | 1.000 | 2846 | tags=50%, list=24%, signal=65% |
| 5534 | GOBP\_REGULATION\_OF\_NUCLEAR\_DIVISION |  | 99 | -0.26 | -0.94 | 0.603 | 0.826 | 1.000 | 4529 | tags=35%, list=38%, signal=56% |
| 5535 | GOBP\_ADIPOSE\_TISSUE\_DEVELOPMENT |  | 28 | -0.31 | -0.94 | 0.569 | 0.826 | 1.000 | 3459 | tags=36%, list=29%, signal=50% |
| 5536 | GOBP\_NEPHRON\_EPITHELIUM\_DEVELOPMENT |  | 71 | -0.27 | -0.94 | 0.600 | 0.827 | 1.000 | 4341 | tags=41%, list=36%, signal=64% |
| 5537 | GOBP\_OTIC\_VESICLE\_DEVELOPMENT |  | 8 | -0.43 | -0.94 | 0.576 | 0.826 | 1.000 | 1146 | tags=25%, list=10%, signal=28% |
| 5538 | GOBP\_PROTEIN\_LOCALIZATION\_TO\_MEMBRANE |  | 477 | -0.24 | -0.94 | 0.722 | 0.826 | 1.000 | 4398 | tags=33%, list=37%, signal=51% |
| 5539 | GOBP\_ACYLGLYCEROL\_TRANSPORT |  | 5 | -0.49 | -0.94 | 0.578 | 0.826 | 1.000 | 2684 | tags=60%, list=22%, signal=77% |
| 5540 | GOMF\_ACYLGLYCEROL\_O\_ACYLTRANSFERASE\_ACTIVITY |  | 18 | -0.35 | -0.94 | 0.560 | 0.826 | 1.000 | 639 | tags=11%, list=5%, signal=12% |
| 5541 | GOBP\_REGULATION\_OF\_PEPTIDYL\_THREONINE\_PHOSPHORYLATION |  | 32 | -0.31 | -0.94 | 0.582 | 0.826 | 1.000 | 3934 | tags=34%, list=33%, signal=51% |
| 5542 | GOBP\_NEGATIVE\_REGULATION\_OF\_LIPOPOLYSACCHARIDE\_MEDIATED\_SIGNALING\_PATHWAY |  | 6 | -0.46 | -0.94 | 0.577 | 0.826 | 1.000 | 1457 | tags=33%, list=12%, signal=38% |
| 5543 | GOBP\_NEGATIVE\_REGULATION\_OF\_ESTABLISHMENT\_OF\_PROTEIN\_LOCALIZATION\_TO\_MITOCHONDRION |  | 5 | -0.49 | -0.94 | 0.581 | 0.826 | 1.000 | 4052 | tags=60%, list=34%, signal=91% |
| 5544 | GOBP\_CYTOSKELETON\_DEPENDENT\_CYTOKINESIS |  | 72 | -0.27 | -0.94 | 0.606 | 0.826 | 1.000 | 4165 | tags=35%, list=35%, signal=53% |
| 5545 | GOBP\_MITOCHONDRIAL\_GENOME\_MAINTENANCE |  | 18 | -0.34 | -0.94 | 0.564 | 0.826 | 1.000 | 3433 | tags=39%, list=29%, signal=54% |
| 5546 | GOBP\_PROTEIN\_TRANSPORT\_ALONG\_MICROTUBULE |  | 52 | -0.28 | -0.94 | 0.586 | 0.826 | 1.000 | 3668 | tags=33%, list=31%, signal=47% |
| 5547 | GOBP\_NEGATIVE\_REGULATION\_OF\_MITOCHONDRIAL\_FUSION |  | 7 | -0.44 | -0.94 | 0.577 | 0.826 | 1.000 | 4673 | tags=57%, list=39%, signal=93% |
| 5548 | GOCC\_AUTOPHAGOSOME\_MEMBRANE |  | 31 | -0.31 | -0.94 | 0.570 | 0.826 | 1.000 | 3401 | tags=32%, list=28%, signal=45% |
| 5549 | GOBP\_POSTSYNAPTIC\_DENSITY\_PROTEIN\_95\_CLUSTERING |  | 4 | -0.52 | -0.94 | 0.596 | 0.826 | 1.000 | 3431 | tags=50%, list=29%, signal=70% |
| 5550 | GOBP\_VASCULAR\_ASSOCIATED\_SMOOTH\_MUSCLE\_CELL\_APOPTOTIC\_PROCESS |  | 9 | -0.41 | -0.94 | 0.575 | 0.826 | 1.000 | 1981 | tags=44%, list=16%, signal=53% |
| 5551 | GOBP\_HEAD\_MORPHOGENESIS |  | 23 | -0.33 | -0.94 | 0.566 | 0.826 | 1.000 | 4319 | tags=52%, list=36%, signal=81% |
| 5552 | GOBP\_LABYRINTHINE\_LAYER\_BLOOD\_VESSEL\_DEVELOPMENT |  | 12 | -0.38 | -0.94 | 0.565 | 0.826 | 1.000 | 13 | tags=8%, list=0%, signal=8% |
| 5553 | GOCC\_NUCLEAR\_LAMINA |  | 8 | -0.43 | -0.94 | 0.560 | 0.827 | 1.000 | 2808 | tags=50%, list=23%, signal=65% |
| 5554 | GOBP\_REGULATION\_OF\_RESPONSE\_TO\_OXIDATIVE\_STRESS |  | 60 | -0.28 | -0.94 | 0.593 | 0.827 | 1.000 | 3675 | tags=35%, list=31%, signal=50% |
| 5555 | GOBP\_EPITHELIAL\_CELL\_FATE\_COMMITMENT |  | 13 | -0.37 | -0.94 | 0.571 | 0.827 | 1.000 | 2560 | tags=38%, list=21%, signal=49% |
| 5556 | GOBP\_NEGATIVE\_REGULATION\_OF\_CYCLIC\_NUCLEOTIDE\_PHOSPHODIESTERASE\_ACTIVITY |  | 4 | -0.52 | -0.94 | 0.589 | 0.826 | 1.000 | 2518 | tags=25%, list=21%, signal=32% |
| 5557 | GOBP\_ODONTOGENESIS\_OF\_DENTIN\_CONTAINING\_TOOTH |  | 56 | -0.28 | -0.94 | 0.592 | 0.827 | 1.000 | 2515 | tags=32%, list=21%, signal=40% |
| 5558 | GOBP\_RUFFLE\_ORGANIZATION |  | 38 | -0.30 | -0.94 | 0.577 | 0.827 | 1.000 | 4619 | tags=45%, list=38%, signal=72% |
| 5559 | GOBP\_CARDIAC\_RIGHT\_VENTRICLE\_MORPHOGENESIS |  | 14 | -0.37 | -0.94 | 0.574 | 0.827 | 1.000 | 3314 | tags=29%, list=28%, signal=39% |
| 5560 | GOBP\_LATE\_NUCLEOPHAGY |  | 3 | -0.56 | -0.94 | 0.592 | 0.827 | 1.000 | 3675 | tags=67%, list=31%, signal=96% |
| 5561 | GOBP\_INCLUSION\_BODY\_ASSEMBLY |  | 14 | -0.37 | -0.94 | 0.565 | 0.828 | 1.000 | 4052 | tags=57%, list=34%, signal=86% |
| 5562 | GOBP\_L\_KYNURENINE\_METABOLIC\_PROCESS |  | 5 | -0.48 | -0.94 | 0.586 | 0.828 | 1.000 | 2851 | tags=60%, list=24%, signal=79% |
| 5563 | GOBP\_PROTEIN\_PHOSPHOPANTETHEINYLATION |  | 231 | -0.25 | -0.94 | 0.666 | 0.828 | 1.000 | 4909 | tags=43%, list=41%, signal=71% |
| 5564 | GOBP\_PROTEIN\_MODIFICATION\_BY\_SMALL\_PROTEIN\_REMOVAL |  | 215 | -0.24 | -0.94 | 0.655 | 0.828 | 1.000 | 4266 | tags=34%, list=36%, signal=52% |
| 5565 | GOMF\_CLATHRIN\_HEAVY\_CHAIN\_BINDING |  | 8 | -0.42 | -0.94 | 0.577 | 0.828 | 1.000 | 4718 | tags=63%, list=39%, signal=103% |
| 5566 | GOBP\_PRI\_MIRNA\_TRANSCRIPTION\_BY\_RNA\_POLYMERASE\_II |  | 40 | -0.29 | -0.94 | 0.576 | 0.828 | 1.000 | 3540 | tags=38%, list=29%, signal=53% |
| 5567 | GOBP\_CELLULAR\_IRON\_ION\_HOMEOSTASIS |  | 49 | -0.29 | -0.94 | 0.589 | 0.828 | 1.000 | 3318 | tags=35%, list=28%, signal=48% |
| 5568 | GOBP\_LYMPHOCYTE\_AGGREGATION |  | 4 | -0.52 | -0.94 | 0.586 | 0.828 | 1.000 | 4606 | tags=75%, list=38%, signal=122% |
| 5569 | GOBP\_PHOSPHORYLATED\_CARBOHYDRATE\_DEPHOSPHORYLATION |  | 15 | -0.36 | -0.94 | 0.560 | 0.829 | 1.000 | 5437 | tags=67%, list=45%, signal=122% |
| 5570 | GOBP\_PROSTATIC\_BUD\_FORMATION |  | 8 | -0.43 | -0.93 | 0.582 | 0.828 | 1.000 | 1146 | tags=25%, list=10%, signal=28% |
| 5571 | GOBP\_MRNA\_EXPORT\_FROM\_NUCLEUS |  | 91 | -0.26 | -0.93 | 0.613 | 0.829 | 1.000 | 4175 | tags=32%, list=35%, signal=48% |
| 5572 | GOBP\_VITAMIN\_METABOLIC\_PROCESS |  | 87 | -0.26 | -0.93 | 0.617 | 0.829 | 1.000 | 3316 | tags=34%, list=28%, signal=47% |
| 5573 | GOMF\_DEOXYRIBONUCLEOTIDE\_BINDING |  | 3 | -0.56 | -0.93 | 0.598 | 0.829 | 1.000 | 4973 | tags=67%, list=41%, signal=114% |
| 5574 | GOBP\_ADIPONECTIN\_ACTIVATED\_SIGNALING\_PATHWAY |  | 7 | -0.44 | -0.93 | 0.574 | 0.829 | 1.000 | 337 | tags=14%, list=3%, signal=15% |
| 5575 | GOBP\_PROTEIN\_IMPORT\_INTO\_PEROXISOME\_MEMBRANE |  | 5 | -0.48 | -0.93 | 0.590 | 0.829 | 1.000 | 5816 | tags=80%, list=48%, signal=155% |
| 5576 | GOBP\_VESICLE\_ORGANIZATION |  | 263 | -0.24 | -0.93 | 0.684 | 0.830 | 1.000 | 2992 | tags=26%, list=25%, signal=34% |
| 5577 | GOCC\_PLASMA\_MEMBRANE\_BOUNDED\_CELL\_PROJECTION\_CYTOPLASM |  | 124 | -0.25 | -0.93 | 0.632 | 0.830 | 1.000 | 3834 | tags=35%, list=32%, signal=52% |
| 5578 | GOBP\_GLIAL\_CELL\_PROLIFERATION |  | 33 | -0.30 | -0.93 | 0.590 | 0.830 | 1.000 | 1743 | tags=21%, list=15%, signal=25% |
| 5579 | GOBP\_SKELETAL\_MUSCLE\_ADAPTATION |  | 14 | -0.37 | -0.93 | 0.563 | 0.830 | 1.000 | 2494 | tags=29%, list=21%, signal=36% |
| 5580 | GOBP\_REGULATION\_OF\_HISTONE\_PHOSPHORYLATION |  | 10 | -0.40 | -0.93 | 0.580 | 0.830 | 1.000 | 3331 | tags=40%, list=28%, signal=55% |
| 5581 | GOBP\_NEURAL\_CREST\_CELL\_MIGRATION\_INVOLVED\_IN\_AUTONOMIC\_NERVOUS\_SYSTEM\_DEVELOPMENT |  | 5 | -0.48 | -0.93 | 0.589 | 0.830 | 1.000 | 4739 | tags=60%, list=39%, signal=99% |
| 5582 | GOBP\_PLANAR\_CELL\_POLARITY\_PATHWAY\_INVOLVED\_IN\_AXIS\_ELONGATION |  | 4 | -0.51 | -0.93 | 0.594 | 0.830 | 1.000 | 1579 | tags=25%, list=13%, signal=29% |
| 5583 | GOBP\_EXPORT\_ACROSS\_PLASMA\_MEMBRANE |  | 35 | -0.30 | -0.93 | 0.585 | 0.830 | 1.000 | 2754 | tags=37%, list=23%, signal=48% |
| 5584 | GOMF\_GLYCINE\_BINDING |  | 5 | -0.48 | -0.93 | 0.579 | 0.830 | 1.000 | 2005 | tags=40%, list=17%, signal=48% |
| 5585 | GOMF\_CYSTEINE\_TYPE\_ENDOPEPTIDASE\_INHIBITOR\_ACTIVITY |  | 21 | -0.33 | -0.93 | 0.580 | 0.830 | 1.000 | 1559 | tags=19%, list=13%, signal=22% |
| 5586 | GOBP\_REGULATION\_OF\_CORE\_PROMOTER\_BINDING |  | 5 | -0.49 | -0.93 | 0.592 | 0.830 | 1.000 | 6186 | tags=100%, list=52%, signal=206% |
| 5587 | GOMF\_OMEGA\_PEPTIDASE\_ACTIVITY |  | 73 | -0.27 | -0.93 | 0.605 | 0.830 | 1.000 | 4909 | tags=47%, list=41%, signal=78% |
| 5588 | GOBP\_REGULATION\_OF\_CYTOPLASMIC\_TRANSLATION |  | 15 | -0.36 | -0.93 | 0.568 | 0.830 | 1.000 | 5307 | tags=47%, list=44%, signal=84% |
| 5589 | GOBP\_AMINE\_TRANSPORT |  | 59 | -0.28 | -0.93 | 0.608 | 0.830 | 1.000 | 1799 | tags=19%, list=15%, signal=22% |
| 5590 | GOMF\_PHOSPHOLIPASE\_ACTIVITY |  | 68 | -0.27 | -0.93 | 0.607 | 0.830 | 1.000 | 3033 | tags=34%, list=25%, signal=45% |
| 5591 | GOBP\_REGULATION\_OF\_SHORT\_TERM\_NEURONAL\_SYNAPTIC\_PLASTICITY |  | 5 | -0.48 | -0.93 | 0.582 | 0.830 | 1.000 | 3349 | tags=80%, list=28%, signal=111% |
| 5592 | GOBP\_HISTONE\_H3\_K9\_ACETYLATION |  | 8 | -0.42 | -0.93 | 0.581 | 0.830 | 1.000 | 3378 | tags=50%, list=28%, signal=70% |
| 5593 | GOBP\_TOLL\_LIKE\_RECEPTOR\_7\_SIGNALING\_PATHWAY |  | 7 | -0.44 | -0.93 | 0.583 | 0.831 | 1.000 | 856 | tags=29%, list=7%, signal=31% |
| 5594 | GOBP\_REGULATION\_OF\_L\_GLUTAMATE\_IMPORT\_ACROSS\_PLASMA\_MEMBRANE |  | 5 | -0.49 | -0.93 | 0.585 | 0.831 | 1.000 | 6186 | tags=100%, list=52%, signal=206% |
| 5595 | GOBP\_BIOLOGICAL\_PROCESS\_INVOLVED\_IN\_INTERACTION\_WITH\_SYMBIONT |  | 55 | -0.28 | -0.93 | 0.601 | 0.831 | 1.000 | 5067 | tags=45%, list=42%, signal=78% |
| 5596 | GOBP\_REGULATION\_OF\_DEVELOPMENT\_HETEROCHRONIC |  | 4 | -0.52 | -0.93 | 0.592 | 0.831 | 1.000 | 2212 | tags=50%, list=18%, signal=61% |
| 5597 | GOBP\_VIRAL\_TRANSLATION |  | 14 | -0.36 | -0.93 | 0.569 | 0.831 | 1.000 | 2893 | tags=29%, list=24%, signal=38% |
| 5598 | GOBP\_NERVE\_GROWTH\_FACTOR\_SIGNALING\_PATHWAY |  | 7 | -0.43 | -0.93 | 0.579 | 0.831 | 1.000 | 4848 | tags=71%, list=40%, signal=120% |
| 5599 | GOBP\_REGULATION\_OF\_METAPHASE\_PLATE\_CONGRESSION |  | 8 | -0.42 | -0.93 | 0.580 | 0.831 | 1.000 | 6680 | tags=75%, list=56%, signal=169% |
| 5600 | GOMF\_DOLICHYL\_DIPHOSPHOOLIGOSACCHARIDE\_PROTEIN\_GLYCOTRANSFERASE\_ACTIVITY |  | 5 | -0.48 | -0.93 | 0.588 | 0.831 | 1.000 | 6274 | tags=100%, list=52%, signal=209% |
| 5601 | GOBP\_REGULATION\_OF\_INTERLEUKIN\_6\_MEDIATED\_SIGNALING\_PATHWAY |  | 4 | -0.52 | -0.93 | 0.588 | 0.832 | 1.000 | 3692 | tags=50%, list=31%, signal=72% |
| 5602 | GOBP\_NEGATIVE\_REGULATION\_OF\_INTRINSIC\_APOPTOTIC\_SIGNALING\_PATHWAY\_BY\_P53\_CLASS\_MEDIATOR |  | 13 | -0.37 | -0.93 | 0.575 | 0.832 | 1.000 | 2656 | tags=31%, list=22%, signal=39% |
| 5603 | GOMF\_U4\_SNRNA\_BINDING |  | 3 | -0.56 | -0.93 | 0.597 | 0.832 | 1.000 | 3088 | tags=67%, list=26%, signal=90% |
| 5604 | GOBP\_SUBSTRATE\_INDEPENDENT\_TELENCEPHALIC\_TANGENTIAL\_MIGRATION |  | 3 | -0.56 | -0.93 | 0.601 | 0.832 | 1.000 | 3889 | tags=67%, list=32%, signal=99% |
| 5605 | GOBP\_RECEPTOR\_LOCALIZATION\_TO\_SYNAPSE |  | 30 | -0.31 | -0.93 | 0.584 | 0.833 | 1.000 | 4655 | tags=47%, list=39%, signal=76% |
| 5606 | GOBP\_ENDOCRINE\_PROCESS |  | 50 | -0.28 | -0.93 | 0.592 | 0.833 | 1.000 | 2480 | tags=28%, list=21%, signal=35% |
| 5607 | GOBP\_METHIONINE\_BIOSYNTHETIC\_PROCESS |  | 11 | -0.39 | -0.93 | 0.575 | 0.833 | 1.000 | 4242 | tags=45%, list=35%, signal=70% |
| 5608 | GOCC\_ENDOPLASMIC\_RETICULUM\_QUALITY\_CONTROL\_COMPARTMENT |  | 20 | -0.33 | -0.93 | 0.580 | 0.833 | 1.000 | 4890 | tags=55%, list=41%, signal=93% |
| 5609 | GOBP\_HEART\_TRABECULA\_MORPHOGENESIS |  | 24 | -0.32 | -0.93 | 0.583 | 0.833 | 1.000 | 3615 | tags=33%, list=30%, signal=48% |
| 5610 | GOBP\_SPHINGOMYELIN\_METABOLIC\_PROCESS |  | 14 | -0.37 | -0.93 | 0.579 | 0.833 | 1.000 | 4433 | tags=50%, list=37%, signal=79% |
| 5611 | GOCC\_SPERM\_MIDPIECE |  | 14 | -0.36 | -0.93 | 0.576 | 0.833 | 1.000 | 1802 | tags=36%, list=15%, signal=42% |
| 5612 | GOBP\_TRANSCRIPTION\_ELONGATION\_FROM\_RNA\_POLYMERASE\_II\_PROMOTER |  | 72 | -0.27 | -0.93 | 0.605 | 0.833 | 1.000 | 5332 | tags=46%, list=44%, signal=82% |
| 5613 | GOBP\_REGULATION\_OF\_HEART\_CONTRACTION |  | 138 | -0.25 | -0.93 | 0.640 | 0.833 | 1.000 | 1919 | tags=17%, list=16%, signal=20% |
| 5614 | GOBP\_FRUCTOSE\_CATABOLIC\_PROCESS\_TO\_HYDROXYACETONE\_PHOSPHATE\_AND\_GLYCERALDEHYDE\_3\_PHOSPHATE |  | 5 | -0.48 | -0.93 | 0.594 | 0.833 | 1.000 | 3191 | tags=40%, list=27%, signal=54% |
| 5615 | GOCC\_PWP2P\_CONTAINING\_SUBCOMPLEX\_OF\_90S\_PRERIBOSOME |  | 5 | -0.48 | -0.93 | 0.596 | 0.833 | 1.000 | 4061 | tags=60%, list=34%, signal=91% |
| 5616 | GOBP\_RESPONSE\_TO\_FUNGICIDE |  | 4 | -0.51 | -0.93 | 0.600 | 0.833 | 1.000 | 5881 | tags=100%, list=49%, signal=196% |
| 5617 | GOBP\_FATTY\_ACYL\_COA\_BIOSYNTHETIC\_PROCESS |  | 26 | -0.32 | -0.93 | 0.595 | 0.833 | 1.000 | 4689 | tags=50%, list=39%, signal=82% |
| 5618 | GOMF\_PRIMARY\_ACTIVE\_TRANSMEMBRANE\_TRANSPORTER\_ACTIVITY |  | 42 | -0.29 | -0.93 | 0.593 | 0.834 | 1.000 | 3046 | tags=38%, list=25%, signal=51% |
| 5619 | GOBP\_T\_HELPER\_2\_CELL\_DIFFERENTIATION |  | 12 | -0.38 | -0.93 | 0.575 | 0.834 | 1.000 | 1566 | tags=33%, list=13%, signal=38% |
| 5620 | GOBP\_ERAD\_PATHWAY |  | 79 | -0.27 | -0.93 | 0.620 | 0.834 | 1.000 | 2774 | tags=22%, list=23%, signal=28% |
| 5621 | GOBP\_REGULATION\_OF\_CYSTEINE\_TYPE\_ENDOPEPTIDASE\_ACTIVITY\_INVOLVED\_IN\_EXECUTION\_PHASE\_OF\_APOPTOSIS |  | 4 | -0.52 | -0.93 | 0.598 | 0.834 | 1.000 | 1070 | tags=25%, list=9%, signal=27% |
| 5622 | GOBP\_ACTIN\_NUCLEATION |  | 36 | -0.30 | -0.93 | 0.596 | 0.834 | 1.000 | 3797 | tags=33%, list=32%, signal=49% |
| 5623 | GOBP\_INTRINSIC\_APOPTOTIC\_SIGNALING\_PATHWAY\_IN\_RESPONSE\_TO\_OXIDATIVE\_STRESS |  | 38 | -0.29 | -0.93 | 0.592 | 0.834 | 1.000 | 3613 | tags=34%, list=30%, signal=49% |
| 5624 | GOMF\_ARRESTIN\_FAMILY\_PROTEIN\_BINDING |  | 5 | -0.48 | -0.93 | 0.590 | 0.834 | 1.000 | 2894 | tags=40%, list=24%, signal=53% |
| 5625 | GOBP\_POSITIVE\_REGULATION\_OF\_LIPID\_METABOLIC\_PROCESS |  | 104 | -0.26 | -0.93 | 0.628 | 0.835 | 1.000 | 3978 | tags=34%, list=33%, signal=50% |
| 5626 | GOBP\_CANNABINOID\_SIGNALING\_PATHWAY |  | 6 | -0.46 | -0.93 | 0.593 | 0.835 | 1.000 | 4907 | tags=67%, list=41%, signal=113% |
| 5627 | GOBP\_EXTRACELLULAR\_EXOSOME\_ASSEMBLY |  | 7 | -0.44 | -0.93 | 0.584 | 0.835 | 1.000 | 2019 | tags=29%, list=17%, signal=34% |
| 5628 | GOCC\_MYELIN\_SHEATH\_ADAXONAL\_REGION |  | 3 | -0.56 | -0.93 | 0.597 | 0.835 | 1.000 | 4312 | tags=67%, list=36%, signal=104% |
| 5629 | GOBP\_REGULATION\_OF\_CARDIAC\_MUSCLE\_ADAPTATION |  | 8 | -0.42 | -0.93 | 0.591 | 0.836 | 1.000 | 2267 | tags=38%, list=19%, signal=46% |
| 5630 | GOMF\_ALPHA\_CATENIN\_BINDING |  | 7 | -0.44 | -0.93 | 0.588 | 0.836 | 1.000 | 3158 | tags=43%, list=26%, signal=58% |
| 5631 | GOBP\_POSITIVE\_REGULATION\_OF\_PROTEIN\_EXPORT\_FROM\_NUCLEUS |  | 15 | -0.36 | -0.93 | 0.571 | 0.836 | 1.000 | 3072 | tags=33%, list=26%, signal=45% |
| 5632 | GOBP\_REGULATION\_OF\_ANION\_TRANSMEMBRANE\_TRANSPORT |  | 75 | -0.27 | -0.93 | 0.618 | 0.836 | 1.000 | 2145 | tags=24%, list=18%, signal=29% |
| 5633 | GOBP\_PHOTORECEPTOR\_CELL\_DEVELOPMENT |  | 20 | -0.33 | -0.93 | 0.576 | 0.836 | 1.000 | 6012 | tags=65%, list=50%, signal=130% |
| 5634 | GOBP\_NEGATIVE\_REGULATION\_OF\_B\_CELL\_MEDIATED\_IMMUNITY |  | 8 | -0.42 | -0.93 | 0.576 | 0.836 | 1.000 | 2249 | tags=38%, list=19%, signal=46% |
| 5635 | GOBP\_CELLULAR\_RESPONSE\_TO\_NITROGEN\_STARVATION |  | 9 | -0.41 | -0.93 | 0.580 | 0.837 | 1.000 | 6407 | tags=78%, list=53%, signal=167% |
| 5636 | GOBP\_REGULATION\_OF\_CELLULAR\_CATABOLIC\_PROCESS |  | 640 | -0.23 | -0.93 | 0.777 | 0.837 | 1.000 | 4324 | tags=35%, list=36%, signal=51% |
| 5637 | GOCC\_TRANSCRIPTION\_FACTOR\_TFTC\_COMPLEX |  | 13 | -0.37 | -0.93 | 0.581 | 0.837 | 1.000 | 1880 | tags=23%, list=16%, signal=27% |
| 5638 | GOBP\_DERMATAN\_SULFATE\_PROTEOGLYCAN\_METABOLIC\_PROCESS |  | 11 | -0.39 | -0.93 | 0.577 | 0.837 | 1.000 | 3406 | tags=36%, list=28%, signal=51% |
| 5639 | GOBP\_FOLIC\_ACID\_TRANSPORT |  | 5 | -0.48 | -0.93 | 0.587 | 0.837 | 1.000 | 2179 | tags=40%, list=18%, signal=49% |
| 5640 | GOMF\_LONG\_CHAIN\_FATTY\_ACYL\_COA\_BINDING |  | 6 | -0.46 | -0.93 | 0.606 | 0.837 | 1.000 | 3992 | tags=67%, list=33%, signal=100% |
| 5641 | GOBP\_HISTONE\_H3\_K27\_TRIMETHYLATION |  | 3 | -0.56 | -0.93 | 0.612 | 0.837 | 1.000 | 5346 | tags=100%, list=45%, signal=180% |
| 5642 | GOBP\_SYMPATHETIC\_NERVOUS\_SYSTEM\_DEVELOPMENT |  | 13 | -0.37 | -0.93 | 0.578 | 0.837 | 1.000 | 2811 | tags=38%, list=23%, signal=50% |
| 5643 | GOBP\_METENCEPHALON\_DEVELOPMENT |  | 69 | -0.27 | -0.93 | 0.623 | 0.837 | 1.000 | 3295 | tags=29%, list=27%, signal=40% |
| 5644 | GOBP\_HUMORAL\_IMMUNE\_RESPONSE\_MEDIATED\_BY\_CIRCULATING\_IMMUNOGLOBULIN |  | 23 | -0.32 | -0.93 | 0.583 | 0.837 | 1.000 | 4135 | tags=39%, list=34%, signal=60% |
| 5645 | GOBP\_POSITIVE\_REGULATION\_OF\_PEROXISOME\_PROLIFERATOR\_ACTIVATED\_RECEPTOR\_SIGNALING\_PATHWAY |  | 4 | -0.51 | -0.93 | 0.592 | 0.837 | 1.000 | 124 | tags=25%, list=1%, signal=25% |
| 5646 | GOBP\_LUNG\_SACCULE\_DEVELOPMENT |  | 5 | -0.48 | -0.93 | 0.601 | 0.837 | 1.000 | 1838 | tags=60%, list=15%, signal=71% |
| 5647 | GOBP\_EPITHELIAL\_CELL\_PROLIFERATION\_INVOLVED\_IN\_SALIVARY\_GLAND\_MORPHOGENESIS |  | 3 | -0.55 | -0.93 | 0.606 | 0.837 | 1.000 | 3565 | tags=67%, list=30%, signal=95% |
| 5648 | GOBP\_REGULATION\_OF\_MEIOTIC\_CELL\_CYCLE |  | 24 | -0.32 | -0.93 | 0.583 | 0.837 | 1.000 | 6075 | tags=54%, list=51%, signal=109% |
| 5649 | GOBP\_CARBOHYDRATE\_BIOSYNTHETIC\_PROCESS |  | 145 | -0.25 | -0.93 | 0.660 | 0.838 | 1.000 | 4203 | tags=41%, list=35%, signal=63% |
| 5650 | GOBP\_ACTIVATION\_OF\_JANUS\_KINASE\_ACTIVITY |  | 8 | -0.42 | -0.93 | 0.590 | 0.838 | 1.000 | 1038 | tags=25%, list=9%, signal=27% |
| 5651 | GOCC\_A\_BAND |  | 25 | -0.32 | -0.93 | 0.591 | 0.838 | 1.000 | 2292 | tags=24%, list=19%, signal=30% |
| 5652 | GOBP\_FLAVIN\_CONTAINING\_COMPOUND\_METABOLIC\_PROCESS |  | 5 | -0.48 | -0.93 | 0.595 | 0.838 | 1.000 | 2774 | tags=60%, list=23%, signal=78% |
| 5653 | GOBP\_METANEPHRIC\_DISTAL\_TUBULE\_DEVELOPMENT |  | 3 | -0.56 | -0.93 | 0.616 | 0.837 | 1.000 | 5330 | tags=100%, list=44%, signal=180% |
| 5654 | GOMF\_EXTRACELLULAR\_MATRIX\_STRUCTURAL\_CONSTITUENT\_CONFERRING\_COMPRESSION\_RESISTANCE |  | 12 | -0.38 | -0.93 | 0.584 | 0.838 | 1.000 | 2434 | tags=25%, list=20%, signal=31% |
| 5655 | GOBP\_LONG\_TERM\_SYNAPTIC\_DEPRESSION |  | 19 | -0.34 | -0.93 | 0.588 | 0.838 | 1.000 | 6005 | tags=68%, list=50%, signal=137% |
| 5656 | GOBP\_B\_CELL\_PROLIFERATION\_INVOLVED\_IN\_IMMUNE\_RESPONSE |  | 4 | -0.51 | -0.92 | 0.604 | 0.838 | 1.000 | 5883 | tags=100%, list=49%, signal=196% |
| 5657 | GOCC\_3M\_COMPLEX |  | 3 | -0.55 | -0.92 | 0.606 | 0.838 | 1.000 | 1623 | tags=33%, list=14%, signal=39% |
| 5658 | GOBP\_VIRAL\_GENOME\_REPLICATION |  | 82 | -0.26 | -0.92 | 0.628 | 0.837 | 1.000 | 3142 | tags=34%, list=26%, signal=46% |
| 5659 | GOBP\_INTRACELLULAR\_LIPID\_TRANSPORT |  | 33 | -0.30 | -0.92 | 0.599 | 0.838 | 1.000 | 3789 | tags=39%, list=32%, signal=57% |
| 5660 | GOMF\_L\_GLUTAMINE\_TRANSMEMBRANE\_TRANSPORTER\_ACTIVITY |  | 6 | -0.45 | -0.92 | 0.596 | 0.838 | 1.000 | 311 | tags=17%, list=3%, signal=17% |
| 5661 | GOMF\_POTASSIUM\_ION\_ANTIPORTER\_ACTIVITY |  | 11 | -0.39 | -0.92 | 0.588 | 0.838 | 1.000 | 933 | tags=27%, list=8%, signal=30% |
| 5662 | GOMF\_CYTOSKELETON\_NUCLEAR\_MEMBRANE\_ANCHOR\_ACTIVITY |  | 5 | -0.48 | -0.92 | 0.601 | 0.838 | 1.000 | 5638 | tags=80%, list=47%, signal=151% |
| 5663 | GOBP\_FIBRINOLYSIS |  | 12 | -0.38 | -0.92 | 0.586 | 0.838 | 1.000 | 2820 | tags=42%, list=23%, signal=54% |
| 5664 | GOCC\_PICLN\_SM\_PROTEIN\_COMPLEX |  | 6 | -0.46 | -0.92 | 0.594 | 0.838 | 1.000 | 1068 | tags=17%, list=9%, signal=18% |
| 5665 | GOMF\_CYSTEINE\_TYPE\_ENDOPEPTIDASE\_ACTIVITY |  | 72 | -0.27 | -0.92 | 0.630 | 0.838 | 1.000 | 3666 | tags=35%, list=31%, signal=50% |
| 5666 | GOBP\_REGULATION\_OF\_ANOIKIS |  | 16 | -0.35 | -0.92 | 0.581 | 0.838 | 1.000 | 2879 | tags=25%, list=24%, signal=33% |
| 5667 | GOBP\_REGULATION\_OF\_GLUCOCORTICOID\_BIOSYNTHETIC\_PROCESS |  | 7 | -0.43 | -0.92 | 0.586 | 0.838 | 1.000 | 1899 | tags=43%, list=16%, signal=51% |
| 5668 | GOBP\_DNA\_TEMPLATED\_TRANSCRIPTION\_ELONGATION |  | 93 | -0.26 | -0.92 | 0.636 | 0.838 | 1.000 | 5549 | tags=47%, list=46%, signal=87% |
| 5669 | GOMF\_TOLL\_LIKE\_RECEPTOR\_BINDING |  | 7 | -0.44 | -0.92 | 0.593 | 0.838 | 1.000 | 2357 | tags=43%, list=20%, signal=53% |
| 5670 | GOBP\_REGULATION\_OF\_GENERATION\_OF\_PRECURSOR\_METABOLITES\_AND\_ENERGY |  | 109 | -0.25 | -0.92 | 0.644 | 0.838 | 1.000 | 4229 | tags=39%, list=35%, signal=60% |
| 5671 | GOBP\_PURINE\_CONTAINING\_COMPOUND\_SALVAGE |  | 12 | -0.38 | -0.92 | 0.577 | 0.838 | 1.000 | 1018 | tags=25%, list=8%, signal=27% |
| 5672 | GOMF\_N\_METHYLTRANSFERASE\_ACTIVITY |  | 63 | -0.27 | -0.92 | 0.624 | 0.838 | 1.000 | 3200 | tags=33%, list=27%, signal=45% |
| 5673 | GOBP\_POSITIVE\_REGULATION\_OF\_INTRINSIC\_APOPTOTIC\_SIGNALING\_PATHWAY |  | 38 | -0.29 | -0.92 | 0.606 | 0.838 | 1.000 | 4673 | tags=42%, list=39%, signal=69% |
| 5674 | GOBP\_ENDOCARDIAL\_CUSHION\_DEVELOPMENT |  | 34 | -0.30 | -0.92 | 0.605 | 0.839 | 1.000 | 3314 | tags=29%, list=28%, signal=41% |
| 5675 | GOMF\_HYDROLASE\_ACTIVITY\_ACTING\_ON\_CARBON\_NITROGEN\_BUT\_NOT\_PEPTIDE\_BONDS\_IN\_CYCLIC\_AMIDES |  | 4 | -0.51 | -0.92 | 0.610 | 0.839 | 1.000 | 1559 | tags=50%, list=13%, signal=57% |
| 5676 | GOBP\_KILLING\_OF\_CELLS\_IN\_OTHER\_ORGANISM\_INVOLVED\_IN\_SYMBIOTIC\_INTERACTION |  | 7 | -0.43 | -0.92 | 0.593 | 0.839 | 1.000 | 5067 | tags=57%, list=42%, signal=99% |
| 5677 | GOCC\_LATERAL\_PLASMA\_MEMBRANE |  | 48 | -0.28 | -0.92 | 0.609 | 0.839 | 1.000 | 4111 | tags=40%, list=34%, signal=60% |
| 5678 | GOBP\_MRNA\_TRANSPORT |  | 114 | -0.25 | -0.92 | 0.644 | 0.840 | 1.000 | 6160 | tags=54%, list=51%, signal=109% |
| 5679 | GOMF\_GLIAL\_CELL\_DERIVED\_NEUROTROPHIC\_FACTOR\_RECEPTOR\_ACTIVITY |  | 4 | -0.51 | -0.92 | 0.615 | 0.840 | 1.000 | 4267 | tags=50%, list=36%, signal=78% |
| 5680 | GOCC\_MICROFIBRIL |  | 7 | -0.44 | -0.92 | 0.593 | 0.840 | 1.000 | 3090 | tags=43%, list=26%, signal=58% |
| 5681 | GOMF\_MONOCARBOXYLIC\_ACID\_TRANSMEMBRANE\_TRANSPORTER\_ACTIVITY |  | 39 | -0.29 | -0.92 | 0.609 | 0.840 | 1.000 | 1324 | tags=21%, list=11%, signal=23% |
| 5682 | GOMF\_PROTEOGLYCAN\_BINDING |  | 27 | -0.31 | -0.92 | 0.590 | 0.841 | 1.000 | 3186 | tags=37%, list=27%, signal=50% |
| 5683 | GOBP\_REGULATION\_OF\_MRNA\_PROCESSING |  | 101 | -0.26 | -0.92 | 0.638 | 0.841 | 1.000 | 6168 | tags=59%, list=51%, signal=121% |
| 5684 | GOBP\_DENTATE\_GYRUS\_DEVELOPMENT |  | 9 | -0.40 | -0.92 | 0.598 | 0.842 | 1.000 | 2021 | tags=33%, list=17%, signal=40% |
| 5685 | GOBP\_GROWTH\_PLATE\_CARTILAGE\_CHONDROCYTE\_DIFFERENTIATION |  | 6 | -0.45 | -0.92 | 0.604 | 0.842 | 1.000 | 6131 | tags=67%, list=51%, signal=136% |
| 5686 | GOMF\_D\_LOOP\_DNA\_BINDING |  | 4 | -0.51 | -0.92 | 0.606 | 0.842 | 1.000 | 5884 | tags=100%, list=49%, signal=196% |
| 5687 | GOBP\_GALACTOSE\_CATABOLIC\_PROCESS |  | 8 | -0.42 | -0.92 | 0.597 | 0.842 | 1.000 | 1234 | tags=25%, list=10%, signal=28% |
| 5688 | GOBP\_FORMATION\_OF\_TRANSLATION\_PREINITIATION\_COMPLEX |  | 9 | -0.40 | -0.92 | 0.594 | 0.842 | 1.000 | 690 | tags=11%, list=6%, signal=12% |
| 5689 | GOBP\_REGULATION\_OF\_STEM\_CELL\_POPULATION\_MAINTENANCE |  | 20 | -0.33 | -0.92 | 0.591 | 0.842 | 1.000 | 5693 | tags=65%, list=47%, signal=123% |
| 5690 | GOBP\_CELLULAR\_RESPONSE\_TO\_BACTERIAL\_LIPOPROTEIN |  | 3 | -0.55 | -0.92 | 0.613 | 0.842 | 1.000 | 1236 | tags=33%, list=10%, signal=37% |
| 5691 | GOMF\_OPIOID\_RECEPTOR\_BINDING |  | 5 | -0.48 | -0.92 | 0.604 | 0.842 | 1.000 | 3890 | tags=60%, list=32%, signal=89% |
| 5692 | GOBP\_NEURAL\_FOLD\_FORMATION |  | 5 | -0.48 | -0.92 | 0.603 | 0.842 | 1.000 | 5693 | tags=80%, list=47%, signal=152% |
| 5693 | GOCC\_INTEGRAL\_COMPONENT\_OF\_LYSOSOMAL\_MEMBRANE |  | 6 | -0.45 | -0.92 | 0.600 | 0.842 | 1.000 | 4585 | tags=50%, list=38%, signal=81% |
| 5694 | GOBP\_OXIDATIVE\_RNA\_DEMETHYLATION |  | 5 | -0.47 | -0.92 | 0.606 | 0.842 | 1.000 | 4508 | tags=80%, list=38%, signal=128% |
| 5695 | GOBP\_ORGANELLE\_MEMBRANE\_FUSION |  | 83 | -0.26 | -0.92 | 0.642 | 0.842 | 1.000 | 3917 | tags=35%, list=33%, signal=51% |
| 5696 | GOMF\_XENOBIOTIC\_TRANSMEMBRANE\_TRANSPORTER\_ACTIVITY |  | 22 | -0.32 | -0.92 | 0.590 | 0.842 | 1.000 | 2842 | tags=36%, list=24%, signal=48% |
| 5697 | GOBP\_REGULATION\_OF\_MRNA\_EXPORT\_FROM\_NUCLEUS |  | 5 | -0.47 | -0.92 | 0.606 | 0.843 | 1.000 | 2630 | tags=40%, list=22%, signal=51% |
| 5698 | GOBP\_ADENYLATE\_CYCLASE\_ACTIVATING\_ADRENERGIC\_RECEPTOR\_SIGNALING\_PATHWAY\_INVOLVED\_IN\_HEART\_PROCESS |  | 6 | -0.45 | -0.92 | 0.601 | 0.842 | 1.000 | 1522 | tags=33%, list=13%, signal=38% |
| 5699 | GOMF\_NF\_KAPPAB\_INDUCING\_KINASE\_ACTIVITY |  | 4 | -0.51 | -0.92 | 0.608 | 0.842 | 1.000 | 1431 | tags=50%, list=12%, signal=57% |
| 5700 | GOBP\_OLIGODENDROCYTE\_DEVELOPMENT |  | 27 | -0.31 | -0.92 | 0.592 | 0.842 | 1.000 | 4865 | tags=44%, list=41%, signal=75% |
| 5701 | GOBP\_GLUTAMINE\_TRANSPORT |  | 6 | -0.45 | -0.92 | 0.599 | 0.843 | 1.000 | 311 | tags=17%, list=3%, signal=17% |
| 5702 | GOBP\_POSITIVE\_REGULATION\_OF\_ER\_ASSOCIATED\_UBIQUITIN\_DEPENDENT\_PROTEIN\_CATABOLIC\_PROCESS |  | 6 | -0.45 | -0.92 | 0.601 | 0.844 | 1.000 | 1587 | tags=33%, list=13%, signal=38% |
| 5703 | GOBP\_POSITIVE\_REGULATION\_OF\_PROTEIN\_AUTOPHOSPHORYLATION |  | 20 | -0.33 | -0.92 | 0.597 | 0.844 | 1.000 | 5607 | tags=55%, list=47%, signal=103% |
| 5704 | GOBP\_PROCESS\_UTILIZING\_AUTOPHAGIC\_MECHANISM |  | 420 | -0.23 | -0.92 | 0.761 | 0.844 | 1.000 | 4324 | tags=35%, list=36%, signal=53% |
| 5705 | GOBP\_RETINAL\_ROD\_CELL\_DIFFERENTIATION |  | 8 | -0.42 | -0.92 | 0.600 | 0.844 | 1.000 | 1520 | tags=25%, list=13%, signal=29% |
| 5706 | GOMF\_BETA\_1\_ADRENERGIC\_RECEPTOR\_BINDING |  | 4 | -0.50 | -0.92 | 0.618 | 0.844 | 1.000 | 5950 | tags=75%, list=50%, signal=149% |
| 5707 | GOBP\_ADHESION\_OF\_SYMBIONT\_TO\_HOST |  | 7 | -0.43 | -0.92 | 0.594 | 0.844 | 1.000 | 2399 | tags=29%, list=20%, signal=36% |
| 5708 | GOBP\_POSITIVE\_REGULATION\_OF\_OXIDATIVE\_STRESS\_INDUCED\_INTRINSIC\_APOPTOTIC\_SIGNALING\_PATHWAY |  | 6 | -0.45 | -0.92 | 0.595 | 0.844 | 1.000 | 5544 | tags=50%, list=46%, signal=93% |
| 5709 | GOCC\_MICROBODY\_MEMBRANE |  | 53 | -0.28 | -0.92 | 0.613 | 0.844 | 1.000 | 4383 | tags=40%, list=36%, signal=62% |
| 5710 | GOBP\_REGULATION\_OF\_TRANSLATION\_AT\_SYNAPSE\_MODULATING\_SYNAPTIC\_TRANSMISSION |  | 5 | -0.47 | -0.92 | 0.612 | 0.844 | 1.000 | 3797 | tags=40%, list=32%, signal=58% |
| 5711 | GOBP\_CLATHRIN\_COAT\_ASSEMBLY |  | 12 | -0.37 | -0.92 | 0.585 | 0.844 | 1.000 | 4775 | tags=67%, list=40%, signal=111% |
| 5712 | GOBP\_NEGATIVE\_REGULATION\_OF\_ORGANELLE\_ASSEMBLY |  | 29 | -0.31 | -0.92 | 0.602 | 0.844 | 1.000 | 3718 | tags=34%, list=31%, signal=50% |
| 5713 | GOBP\_B\_CELL\_CHEMOTAXIS |  | 6 | -0.45 | -0.92 | 0.604 | 0.844 | 1.000 | 1305 | tags=33%, list=11%, signal=37% |
| 5714 | GOBP\_REGULATION\_OF\_BILE\_ACID\_SECRETION |  | 3 | -0.55 | -0.92 | 0.617 | 0.844 | 1.000 | 685 | tags=33%, list=6%, signal=35% |
| 5715 | GOBP\_TRANSCRIPTION\_INITIATION\_FROM\_RNA\_POLYMERASE\_II\_PROMOTER |  | 140 | -0.25 | -0.92 | 0.676 | 0.845 | 1.000 | 3999 | tags=34%, list=33%, signal=50% |
| 5716 | GOBP\_REGULATION\_OF\_NEUTROPHIL\_ACTIVATION |  | 5 | -0.48 | -0.92 | 0.610 | 0.845 | 1.000 | 3050 | tags=60%, list=25%, signal=80% |
| 5717 | GOBP\_NEGATIVE\_REGULATION\_OF\_ENDOTHELIAL\_CELL\_DIFFERENTIATION |  | 6 | -0.45 | -0.92 | 0.608 | 0.845 | 1.000 | 1107 | tags=33%, list=9%, signal=37% |
| 5718 | GOBP\_REGULATION\_OF\_ATP\_METABOLIC\_PROCESS |  | 80 | -0.26 | -0.92 | 0.642 | 0.845 | 1.000 | 4770 | tags=46%, list=40%, signal=76% |
| 5719 | GOBP\_REGULATION\_OF\_TRANSLATION\_IN\_RESPONSE\_TO\_STRESS |  | 18 | -0.34 | -0.92 | 0.601 | 0.845 | 1.000 | 3872 | tags=39%, list=32%, signal=57% |
| 5720 | GOCC\_CRD\_MEDIATED\_MRNA\_STABILITY\_COMPLEX |  | 5 | -0.48 | -0.92 | 0.609 | 0.845 | 1.000 | 6262 | tags=100%, list=52%, signal=209% |
| 5721 | GOBP\_SMOOTHENED\_SIGNALING\_PATHWAY\_INVOLVED\_IN\_VENTRAL\_SPINAL\_CORD\_PATTERNING |  | 5 | -0.48 | -0.92 | 0.613 | 0.845 | 1.000 | 556 | tags=20%, list=5%, signal=21% |
| 5722 | GOBP\_REGULATION\_OF\_MACROPHAGE\_DERIVED\_FOAM\_CELL\_DIFFERENTIATION |  | 21 | -0.32 | -0.92 | 0.591 | 0.845 | 1.000 | 3217 | tags=43%, list=27%, signal=58% |
| 5723 | GOBP\_EMBRYONIC\_HEART\_TUBE\_MORPHOGENESIS |  | 44 | -0.28 | -0.92 | 0.617 | 0.845 | 1.000 | 2474 | tags=20%, list=21%, signal=26% |
| 5724 | GOBP\_STRESS\_INDUCED\_PREMATURE\_SENESCENCE |  | 6 | -0.45 | -0.92 | 0.599 | 0.845 | 1.000 | 4432 | tags=83%, list=37%, signal=132% |
| 5725 | GOMF\_FRUCTOSE\_BINDING |  | 3 | -0.55 | -0.92 | 0.617 | 0.846 | 1.000 | 5427 | tags=100%, list=45%, signal=182% |
| 5726 | GOBP\_PROTEIN\_MATURATION |  | 186 | -0.24 | -0.92 | 0.690 | 0.846 | 1.000 | 4305 | tags=35%, list=36%, signal=54% |
| 5727 | GOBP\_NAD\_BIOSYNTHETIC\_PROCESS |  | 17 | -0.34 | -0.92 | 0.603 | 0.846 | 1.000 | 2865 | tags=29%, list=24%, signal=39% |
| 5728 | GOBP\_CARBOHYDRATE\_DERIVATIVE\_TRANSPORT |  | 56 | -0.27 | -0.92 | 0.623 | 0.846 | 1.000 | 3427 | tags=30%, list=29%, signal=42% |
| 5729 | GOMF\_MYOSIN\_BINDING |  | 46 | -0.28 | -0.92 | 0.628 | 0.846 | 1.000 | 2115 | tags=20%, list=18%, signal=24% |
| 5730 | GOBP\_REGULATION\_OF\_VASCULAR\_ASSOCIATED\_SMOOTH\_MUSCLE\_CELL\_PROLIFERATION |  | 42 | -0.28 | -0.92 | 0.615 | 0.846 | 1.000 | 3392 | tags=33%, list=28%, signal=46% |
| 5731 | GOBP\_POSITIVE\_REGULATION\_OF\_EXTRINSIC\_APOPTOTIC\_SIGNALING\_PATHWAY\_IN\_ABSENCE\_OF\_LIGAND |  | 8 | -0.41 | -0.92 | 0.598 | 0.846 | 1.000 | 2356 | tags=25%, list=20%, signal=31% |
| 5732 | GOBP\_DETERMINATION\_OF\_ADULT\_LIFESPAN |  | 11 | -0.38 | -0.92 | 0.597 | 0.846 | 1.000 | 4660 | tags=73%, list=39%, signal=119% |
| 5733 | GOBP\_PYRUVATE\_METABOLIC\_PROCESS |  | 114 | -0.25 | -0.92 | 0.663 | 0.846 | 1.000 | 4255 | tags=42%, list=35%, signal=65% |
| 5734 | GOCC\_MEIOTIC\_COHESIN\_COMPLEX |  | 4 | -0.51 | -0.92 | 0.620 | 0.846 | 1.000 | 5914 | tags=100%, list=49%, signal=197% |
| 5735 | GOMF\_PEPTIDASE\_REGULATOR\_ACTIVITY |  | 104 | -0.25 | -0.92 | 0.656 | 0.846 | 1.000 | 3325 | tags=24%, list=28%, signal=33% |
| 5736 | GOBP\_ER\_OVERLOAD\_RESPONSE |  | 10 | -0.39 | -0.92 | 0.602 | 0.846 | 1.000 | 4035 | tags=50%, list=34%, signal=75% |
| 5737 | GOBP\_NEGATIVE\_REGULATION\_OF\_G\_PROTEIN\_COUPLED\_RECEPTOR\_SIGNALING\_PATHWAY |  | 31 | -0.30 | -0.92 | 0.614 | 0.847 | 1.000 | 2629 | tags=29%, list=22%, signal=37% |
| 5738 | GOBP\_ENDOCARDIAL\_CUSHION\_MORPHOGENESIS |  | 27 | -0.31 | -0.92 | 0.605 | 0.847 | 1.000 | 6049 | tags=67%, list=50%, signal=134% |
| 5739 | GOBP\_IRON\_ION\_IMPORT\_ACROSS\_PLASMA\_MEMBRANE |  | 3 | -0.55 | -0.92 | 0.629 | 0.847 | 1.000 | 2258 | tags=33%, list=19%, signal=41% |
| 5740 | GOBP\_CATECHOLAMINE\_UPTAKE |  | 11 | -0.38 | -0.92 | 0.605 | 0.846 | 1.000 | 2563 | tags=27%, list=21%, signal=35% |
| 5741 | GOBP\_CD4\_POSITIVE\_ALPHA\_BETA\_T\_CELL\_CYTOKINE\_PRODUCTION |  | 13 | -0.36 | -0.91 | 0.598 | 0.846 | 1.000 | 1566 | tags=31%, list=13%, signal=35% |
| 5742 | GOBP\_METAL\_ION\_EXPORT |  | 25 | -0.31 | -0.91 | 0.605 | 0.846 | 1.000 | 3046 | tags=40%, list=25%, signal=53% |
| 5743 | GOMF\_G\_PROTEIN\_COUPLED\_AMINE\_RECEPTOR\_ACTIVITY |  | 18 | -0.34 | -0.91 | 0.604 | 0.846 | 1.000 | 929 | tags=17%, list=8%, signal=18% |
| 5744 | GOBP\_NEGATIVE\_REGULATION\_OF\_LIPID\_METABOLIC\_PROCESS |  | 62 | -0.27 | -0.91 | 0.634 | 0.846 | 1.000 | 2667 | tags=29%, list=22%, signal=37% |
| 5745 | GOCC\_CENTRAL\_ELEMENT |  | 3 | -0.55 | -0.91 | 0.621 | 0.846 | 1.000 | 5052 | tags=67%, list=42%, signal=115% |
| 5746 | GOBP\_PROTEIN\_REFOLDING |  | 16 | -0.35 | -0.91 | 0.593 | 0.847 | 1.000 | 3242 | tags=38%, list=27%, signal=51% |
| 5747 | GOBP\_NEGATIVE\_REGULATION\_OF\_MACROPHAGE\_CYTOKINE\_PRODUCTION |  | 4 | -0.51 | -0.91 | 0.627 | 0.847 | 1.000 | 2889 | tags=50%, list=24%, signal=66% |
| 5748 | GOBP\_LIPOPHAGY |  | 7 | -0.43 | -0.91 | 0.612 | 0.847 | 1.000 | 3931 | tags=43%, list=33%, signal=64% |
| 5749 | GOBP\_REGULATION\_OF\_GOLGI\_ORGANIZATION |  | 13 | -0.36 | -0.91 | 0.593 | 0.847 | 1.000 | 5328 | tags=54%, list=44%, signal=97% |
| 5750 | GOBP\_CELLULAR\_RESPONSE\_TO\_DRUG |  | 49 | -0.28 | -0.91 | 0.626 | 0.847 | 1.000 | 4868 | tags=49%, list=41%, signal=82% |
| 5751 | GOBP\_AGGREPHAGY |  | 9 | -0.40 | -0.91 | 0.599 | 0.847 | 1.000 | 3401 | tags=56%, list=28%, signal=77% |
| 5752 | GOBP\_RESPONSE\_TO\_HORMONE |  | 604 | -0.23 | -0.91 | 0.813 | 0.847 | 1.000 | 4008 | tags=38%, list=33%, signal=54% |
| 5753 | GOMF\_TAT\_PROTEIN\_BINDING |  | 9 | -0.40 | -0.91 | 0.600 | 0.847 | 1.000 | 3177 | tags=22%, list=26%, signal=30% |
| 5754 | GOBP\_SUGAR\_MEDIATED\_SIGNALING\_PATHWAY |  | 4 | -0.50 | -0.91 | 0.616 | 0.847 | 1.000 | 5712 | tags=75%, list=48%, signal=143% |
| 5755 | GOBP\_REGULATION\_OF\_PROTEIN\_AUTOPHOSPHORYLATION |  | 36 | -0.29 | -0.91 | 0.617 | 0.847 | 1.000 | 5607 | tags=50%, list=47%, signal=93% |
| 5756 | GOBP\_NON\_MOTILE\_CILIUM\_ASSEMBLY |  | 38 | -0.29 | -0.91 | 0.615 | 0.847 | 1.000 | 3100 | tags=24%, list=26%, signal=32% |
| 5757 | GOBP\_INTERLEUKIN\_21\_MEDIATED\_SIGNALING\_PATHWAY |  | 7 | -0.43 | -0.91 | 0.600 | 0.847 | 1.000 | 3436 | tags=71%, list=29%, signal=100% |
| 5758 | GOBP\_NEGATIVE\_REGULATION\_OF\_PROTEIN\_MODIFICATION\_BY\_SMALL\_PROTEIN\_CONJUGATION\_OR\_REMOVAL |  | 65 | -0.27 | -0.91 | 0.643 | 0.847 | 1.000 | 3132 | tags=28%, list=26%, signal=37% |
| 5759 | GOBP\_REGULATION\_OF\_IMMUNOGLOBULIN\_PRODUCTION |  | 36 | -0.29 | -0.91 | 0.625 | 0.847 | 1.000 | 3205 | tags=33%, list=27%, signal=45% |
| 5760 | GOBP\_LIPOPROTEIN\_BIOSYNTHETIC\_PROCESS |  | 68 | -0.27 | -0.91 | 0.639 | 0.847 | 1.000 | 4392 | tags=37%, list=37%, signal=58% |
| 5761 | GOCC\_CILIARY\_MEMBRANE |  | 42 | -0.28 | -0.91 | 0.621 | 0.847 | 1.000 | 4351 | tags=40%, list=36%, signal=63% |
| 5762 | GOMF\_AMINO\_ACID\_BINDING |  | 28 | -0.31 | -0.91 | 0.609 | 0.847 | 1.000 | 2746 | tags=36%, list=23%, signal=46% |
| 5763 | GOBP\_TRANS\_SYNAPTIC\_SIGNALING\_BY\_TRANS\_SYNAPTIC\_COMPLEX |  | 5 | -0.47 | -0.91 | 0.614 | 0.847 | 1.000 | 6320 | tags=100%, list=53%, signal=211% |
| 5764 | GOBP\_CELLULAR\_RESPONSE\_TO\_INSULIN\_STIMULUS |  | 172 | -0.24 | -0.91 | 0.692 | 0.847 | 1.000 | 3934 | tags=44%, list=33%, signal=65% |
| 5765 | GOMF\_CALCIUM\_DEPENDENT\_PROTEIN\_KINASE\_ACTIVITY |  | 17 | -0.34 | -0.91 | 0.599 | 0.847 | 1.000 | 3308 | tags=35%, list=28%, signal=49% |
| 5766 | GOBP\_NUCLEOTIDE\_EXCISION\_REPAIR\_DNA\_DUPLEX\_UNWINDING |  | 19 | -0.33 | -0.91 | 0.601 | 0.848 | 1.000 | 5370 | tags=63%, list=45%, signal=114% |
| 5767 | GOBP\_GLYCOSYLCERAMIDE\_CATABOLIC\_PROCESS |  | 5 | -0.47 | -0.91 | 0.618 | 0.848 | 1.000 | 3768 | tags=60%, list=31%, signal=87% |
| 5768 | GOBP\_POSITIVE\_REGULATION\_OF\_HELICASE\_ACTIVITY |  | 7 | -0.43 | -0.91 | 0.601 | 0.848 | 1.000 | 2432 | tags=43%, list=20%, signal=54% |
| 5769 | GOBP\_REGULATION\_OF\_PLASMA\_LIPOPROTEIN\_PARTICLE\_LEVELS |  | 52 | -0.27 | -0.91 | 0.626 | 0.848 | 1.000 | 5079 | tags=54%, list=42%, signal=93% |
| 5770 | GOBP\_REGULATION\_OF\_T\_HELPER\_1\_TYPE\_IMMUNE\_RESPONSE |  | 18 | -0.34 | -0.91 | 0.600 | 0.848 | 1.000 | 3487 | tags=39%, list=29%, signal=55% |
| 5771 | GOBP\_AXONOGENESIS\_INVOLVED\_IN\_INNERVATION |  | 3 | -0.55 | -0.91 | 0.621 | 0.848 | 1.000 | 2560 | tags=33%, list=21%, signal=42% |
| 5772 | GOMF\_LOW\_DENSITY\_LIPOPROTEIN\_PARTICLE\_RECEPTOR\_BINDING |  | 16 | -0.35 | -0.91 | 0.605 | 0.848 | 1.000 | 4758 | tags=69%, list=40%, signal=114% |
| 5773 | GOBP\_REGULATION\_OF\_PROTEIN\_LOCALIZATION\_TO\_MEMBRANE |  | 134 | -0.25 | -0.91 | 0.673 | 0.848 | 1.000 | 4945 | tags=41%, list=41%, signal=69% |
| 5774 | GOBP\_REGULATION\_OF\_PROTEIN\_LOCALIZATION\_TO\_ENDOSOME |  | 10 | -0.39 | -0.91 | 0.592 | 0.848 | 1.000 | 2416 | tags=40%, list=20%, signal=50% |
| 5775 | GOBP\_BIOLOGICAL\_PROCESS\_INVOLVED\_IN\_SYMBIOTIC\_INTERACTION |  | 713 | -0.23 | -0.91 | 0.836 | 0.848 | 1.000 | 3212 | tags=27%, list=27%, signal=34% |
| 5776 | GOCC\_MICROBODY |  | 109 | -0.25 | -0.91 | 0.666 | 0.848 | 1.000 | 4383 | tags=38%, list=36%, signal=59% |
| 5777 | GOMF\_CYCLIN\_DEPENDENT\_PROTEIN\_SERINE\_THREONINE\_KINASE\_REGULATOR\_ACTIVITY |  | 34 | -0.29 | -0.91 | 0.613 | 0.848 | 1.000 | 3687 | tags=35%, list=31%, signal=51% |
| 5778 | GOMF\_OXIDATIVE\_RNA\_DEMETHYLASE\_ACTIVITY |  | 5 | -0.47 | -0.91 | 0.620 | 0.848 | 1.000 | 4508 | tags=80%, list=38%, signal=128% |
| 5779 | GOMF\_LIPOPROTEIN\_PARTICLE\_RECEPTOR\_BINDING |  | 19 | -0.33 | -0.91 | 0.604 | 0.848 | 1.000 | 4758 | tags=63%, list=40%, signal=104% |
| 5780 | GOBP\_10\_FORMYLTETRAHYDROFOLATE\_METABOLIC\_PROCESS |  | 4 | -0.51 | -0.91 | 0.620 | 0.848 | 1.000 | 4242 | tags=75%, list=35%, signal=116% |
| 5781 | GOBP\_REGULATION\_OF\_ACTION\_POTENTIAL |  | 36 | -0.29 | -0.91 | 0.619 | 0.848 | 1.000 | 4164 | tags=39%, list=35%, signal=59% |
| 5782 | GOBP\_ATRIAL\_CARDIAC\_MUSCLE\_CELL\_MEMBRANE\_REPOLARIZATION |  | 6 | -0.45 | -0.91 | 0.609 | 0.849 | 1.000 | 69 | tags=17%, list=1%, signal=17% |
| 5783 | GOBP\_NEGATIVE\_REGULATION\_OF\_MUSCLE\_HYPERTROPHY |  | 22 | -0.32 | -0.91 | 0.608 | 0.849 | 1.000 | 3177 | tags=32%, list=26%, signal=43% |
| 5784 | GOMF\_ACETYLCHOLINE\_RECEPTOR\_BINDING |  | 5 | -0.47 | -0.91 | 0.623 | 0.849 | 1.000 | 4691 | tags=60%, list=39%, signal=98% |
| 5785 | GOBP\_SARCOPLASMIC\_RETICULUM\_CALCIUM\_ION\_TRANSPORT |  | 22 | -0.32 | -0.91 | 0.606 | 0.849 | 1.000 | 4627 | tags=36%, list=39%, signal=59% |
| 5786 | GOCC\_NADPH\_OXIDASE\_COMPLEX |  | 8 | -0.41 | -0.91 | 0.608 | 0.849 | 1.000 | 36 | tags=13%, list=0%, signal=13% |
| 5787 | GOBP\_LIMB\_BUD\_FORMATION |  | 8 | -0.41 | -0.91 | 0.600 | 0.849 | 1.000 | 5967 | tags=63%, list=50%, signal=124% |
| 5788 | GOBP\_REGULATION\_OF\_EPIDERMIS\_DEVELOPMENT |  | 41 | -0.28 | -0.91 | 0.615 | 0.849 | 1.000 | 3615 | tags=32%, list=30%, signal=45% |
| 5789 | GOBP\_RNA\_EXPORT\_FROM\_NUCLEUS |  | 112 | -0.25 | -0.91 | 0.672 | 0.850 | 1.000 | 4278 | tags=32%, list=36%, signal=49% |
| 5790 | GOBP\_CELLULAR\_RESPONSE\_TO\_OSMOTIC\_STRESS |  | 28 | -0.31 | -0.91 | 0.619 | 0.850 | 1.000 | 4353 | tags=46%, list=36%, signal=73% |
| 5791 | GOBP\_FATTY\_ACYL\_COA\_METABOLIC\_PROCESS |  | 32 | -0.30 | -0.91 | 0.613 | 0.850 | 1.000 | 4801 | tags=50%, list=40%, signal=83% |
| 5792 | GOBP\_REGULATION\_OF\_LIPID\_BIOSYNTHETIC\_PROCESS |  | 144 | -0.24 | -0.91 | 0.689 | 0.850 | 1.000 | 2760 | tags=25%, list=23%, signal=32% |
| 5793 | GOBP\_DEOXYRIBONUCLEOSIDE\_METABOLIC\_PROCESS |  | 6 | -0.45 | -0.91 | 0.611 | 0.850 | 1.000 | 2053 | tags=50%, list=17%, signal=60% |
| 5794 | GOBP\_NEGATIVE\_REGULATION\_OF\_CHEMOTAXIS |  | 40 | -0.28 | -0.91 | 0.621 | 0.850 | 1.000 | 3724 | tags=38%, list=31%, signal=54% |
| 5795 | GOBP\_NEGATIVE\_REGULATION\_OF\_AMYLOID\_PRECURSOR\_PROTEIN\_BIOSYNTHETIC\_PROCESS |  | 5 | -0.47 | -0.91 | 0.632 | 0.850 | 1.000 | 190 | tags=20%, list=2%, signal=20% |
| 5796 | GOBP\_LEUCINE\_METABOLIC\_PROCESS |  | 7 | -0.43 | -0.91 | 0.616 | 0.850 | 1.000 | 5543 | tags=71%, list=46%, signal=133% |
| 5797 | GOBP\_REGULATION\_OF\_ER\_TO\_GOLGI\_VESICLE\_MEDIATED\_TRANSPORT |  | 15 | -0.35 | -0.91 | 0.607 | 0.850 | 1.000 | 1372 | tags=20%, list=11%, signal=23% |
| 5798 | GOBP\_ENDOMEMBRANE\_SYSTEM\_ORGANIZATION |  | 344 | -0.23 | -0.91 | 0.770 | 0.850 | 1.000 | 4564 | tags=38%, list=38%, signal=60% |
| 5799 | GOBP\_MAMMARY\_GLAND\_DUCT\_MORPHOGENESIS |  | 22 | -0.32 | -0.91 | 0.608 | 0.850 | 1.000 | 1146 | tags=18%, list=10%, signal=20% |
| 5800 | GOBP\_NEGATIVE\_REGULATION\_OF\_PROTEIN\_LOCALIZATION\_TO\_CELL\_PERIPHERY |  | 19 | -0.33 | -0.91 | 0.609 | 0.851 | 1.000 | 2891 | tags=32%, list=24%, signal=42% |
| 5801 | GOMF\_STEROID\_HORMONE\_RECEPTOR\_ACTIVITY |  | 17 | -0.34 | -0.91 | 0.611 | 0.851 | 1.000 | 1935 | tags=29%, list=16%, signal=35% |
| 5802 | GOBP\_GLYCOSIDE\_CATABOLIC\_PROCESS |  | 7 | -0.42 | -0.91 | 0.603 | 0.850 | 1.000 | 4037 | tags=57%, list=34%, signal=86% |
| 5803 | GOBP\_ICOSANOID\_SECRETION |  | 24 | -0.31 | -0.91 | 0.607 | 0.850 | 1.000 | 2913 | tags=42%, list=24%, signal=55% |
| 5804 | GOBP\_LATERAL\_SPROUTING\_FROM\_AN\_EPITHELIUM |  | 10 | -0.39 | -0.91 | 0.605 | 0.851 | 1.000 | 1146 | tags=20%, list=10%, signal=22% |
| 5805 | GOMF\_PHOSPHATIDYLINOSITOL\_5\_PHOSPHATE\_BINDING |  | 12 | -0.37 | -0.91 | 0.603 | 0.851 | 1.000 | 4986 | tags=50%, list=42%, signal=85% |
| 5806 | GOMF\_HEPARIN\_BINDING |  | 93 | -0.25 | -0.91 | 0.670 | 0.851 | 1.000 | 3217 | tags=27%, list=27%, signal=36% |
| 5807 | GOBP\_RESPONSE\_TO\_TOXIC\_SUBSTANCE |  | 140 | -0.24 | -0.91 | 0.689 | 0.852 | 1.000 | 2803 | tags=28%, list=23%, signal=36% |
| 5808 | GOBP\_NUCLEOTIDE\_EXCISION\_REPAIR |  | 90 | -0.26 | -0.91 | 0.671 | 0.852 | 1.000 | 5370 | tags=48%, list=45%, signal=86% |
| 5809 | GOBP\_ARACHIDONIC\_ACID\_METABOLIC\_PROCESS |  | 29 | -0.30 | -0.91 | 0.615 | 0.852 | 1.000 | 1692 | tags=24%, list=14%, signal=28% |
| 5810 | GOBP\_REGULATION\_OF\_STORE\_OPERATED\_CALCIUM\_ENTRY |  | 5 | -0.47 | -0.91 | 0.628 | 0.852 | 1.000 | 3226 | tags=40%, list=27%, signal=55% |
| 5811 | GOBP\_DNA\_METHYLATION\_DEPENDENT\_HETEROCHROMATIN\_ASSEMBLY |  | 12 | -0.37 | -0.91 | 0.599 | 0.852 | 1.000 | 875 | tags=17%, list=7%, signal=18% |
| 5812 | GOBP\_RESPONSE\_TO\_DISACCHARIDE |  | 4 | -0.50 | -0.91 | 0.633 | 0.852 | 1.000 | 3191 | tags=50%, list=27%, signal=68% |
| 5813 | GOBP\_PROTEIN\_REPAIR |  | 6 | -0.44 | -0.91 | 0.619 | 0.852 | 1.000 | 5184 | tags=50%, list=43%, signal=88% |
| 5814 | GOBP\_RESPONSE\_TO\_ALCOHOL |  | 153 | -0.24 | -0.91 | 0.694 | 0.852 | 1.000 | 3395 | tags=31%, list=28%, signal=43% |
| 5815 | GOBP\_RESPONSE\_TO\_GONADOTROPIN |  | 14 | -0.36 | -0.91 | 0.606 | 0.852 | 1.000 | 3833 | tags=43%, list=32%, signal=63% |
| 5816 | GOBP\_CERAMIDE\_METABOLIC\_PROCESS |  | 74 | -0.26 | -0.91 | 0.661 | 0.852 | 1.000 | 4467 | tags=47%, list=37%, signal=75% |
| 5817 | GOMF\_PYRUVATE\_DEHYDROGENASE\_ACETYL\_TRANSFERRING\_KINASE\_ACTIVITY |  | 3 | -0.54 | -0.91 | 0.633 | 0.852 | 1.000 | 1956 | tags=33%, list=16%, signal=40% |
| 5818 | GOBP\_POSITIVE\_REGULATION\_OF\_TRIGLYCERIDE\_CATABOLIC\_PROCESS |  | 5 | -0.47 | -0.91 | 0.627 | 0.852 | 1.000 | 261 | tags=20%, list=2%, signal=20% |
| 5819 | GOBP\_IRES\_DEPENDENT\_VIRAL\_TRANSLATIONAL\_INITIATION |  | 11 | -0.38 | -0.91 | 0.604 | 0.852 | 1.000 | 4127 | tags=36%, list=34%, signal=55% |
| 5820 | GOBP\_PEPTIDYL\_THREONINE\_DEPHOSPHORYLATION |  | 18 | -0.34 | -0.91 | 0.606 | 0.853 | 1.000 | 3447 | tags=33%, list=29%, signal=47% |
| 5821 | GOCC\_PLATELET\_ALPHA\_GRANULE\_MEMBRANE |  | 15 | -0.35 | -0.91 | 0.612 | 0.853 | 1.000 | 1236 | tags=13%, list=10%, signal=15% |
| 5822 | GOBP\_REGULATION\_OF\_RESTING\_MEMBRANE\_POTENTIAL |  | 5 | -0.47 | -0.91 | 0.620 | 0.853 | 1.000 | 1183 | tags=20%, list=10%, signal=22% |
| 5823 | GOBP\_REGULATION\_OF\_MICROVILLUS\_ORGANIZATION |  | 11 | -0.38 | -0.91 | 0.610 | 0.853 | 1.000 | 5082 | tags=64%, list=42%, signal=110% |
| 5824 | GOBP\_REGULATION\_OF\_MATRIX\_METALLOPEPTIDASE\_SECRETION |  | 4 | -0.50 | -0.91 | 0.625 | 0.853 | 1.000 | 1230 | tags=25%, list=10%, signal=28% |
| 5825 | GOCC\_BLOOD\_MICROPARTICLE |  | 54 | -0.27 | -0.91 | 0.643 | 0.853 | 1.000 | 2320 | tags=26%, list=19%, signal=32% |
| 5826 | GOBP\_PROTEIN\_LOCALIZATION\_TO\_ENDOSOME |  | 19 | -0.33 | -0.91 | 0.610 | 0.853 | 1.000 | 4338 | tags=53%, list=36%, signal=82% |
| 5827 | GOBP\_REGULATION\_OF\_CALCIUM\_ION\_TRANSMEMBRANE\_TRANSPORTER\_ACTIVITY |  | 53 | -0.27 | -0.91 | 0.642 | 0.853 | 1.000 | 2908 | tags=23%, list=24%, signal=30% |
| 5828 | GOMF\_CAMP\_DEPENDENT\_PROTEIN\_KINASE\_REGULATOR\_ACTIVITY |  | 9 | -0.40 | -0.91 | 0.601 | 0.853 | 1.000 | 56 | tags=11%, list=0%, signal=11% |
| 5829 | GOBP\_NEGATIVE\_REGULATION\_OF\_SMOOTHENED\_SIGNALING\_PATHWAY\_INVOLVED\_IN\_DORSAL\_VENTRAL\_NEURAL\_TUBE\_PATTERNING |  | 4 | -0.50 | -0.91 | 0.623 | 0.853 | 1.000 | 4097 | tags=75%, list=34%, signal=114% |
| 5830 | GOBP\_PLASMA\_LIPOPROTEIN\_PARTICLE\_CLEARANCE |  | 31 | -0.30 | -0.91 | 0.619 | 0.853 | 1.000 | 4801 | tags=52%, list=40%, signal=86% |
| 5831 | GOCC\_CYTOPLASMIC\_DYNEIN\_COMPLEX |  | 16 | -0.34 | -0.91 | 0.609 | 0.853 | 1.000 | 4760 | tags=50%, list=40%, signal=83% |
| 5832 | GOMF\_ORGANOPHOSPHATE\_ESTER\_TRANSMEMBRANE\_TRANSPORTER\_ACTIVITY |  | 22 | -0.32 | -0.90 | 0.610 | 0.854 | 1.000 | 2605 | tags=27%, list=22%, signal=35% |
| 5833 | GOMF\_PROTEIN\_CARBOXYL\_O\_METHYLTRANSFERASE\_ACTIVITY |  | 6 | -0.45 | -0.90 | 0.623 | 0.854 | 1.000 | 6544 | tags=83%, list=54%, signal=183% |
| 5834 | GOMF\_INSULIN\_LIKE\_GROWTH\_FACTOR\_BINDING |  | 20 | -0.33 | -0.90 | 0.620 | 0.854 | 1.000 | 4424 | tags=55%, list=37%, signal=87% |
| 5835 | GOBP\_NUCLEAR\_PORE\_COMPLEX\_ASSEMBLY |  | 9 | -0.40 | -0.90 | 0.609 | 0.855 | 1.000 | 4770 | tags=67%, list=40%, signal=111% |
| 5836 | GOBP\_POSITIVE\_REGULATION\_OF\_RELEASE\_OF\_CYTOCHROME\_C\_FROM\_MITOCHONDRIA |  | 16 | -0.34 | -0.90 | 0.616 | 0.855 | 1.000 | 1886 | tags=25%, list=16%, signal=30% |
| 5837 | GOBP\_REGULATION\_OF\_LYASE\_ACTIVITY |  | 26 | -0.31 | -0.90 | 0.624 | 0.855 | 1.000 | 5262 | tags=46%, list=44%, signal=82% |
| 5838 | GOMF\_UBIQUITIN\_LIKE\_PROTEIN\_LIGASE\_BINDING |  | 237 | -0.24 | -0.90 | 0.746 | 0.854 | 1.000 | 2010 | tags=19%, list=17%, signal=22% |
| 5839 | GOBP\_NEUROTRANSMITTER\_RECEPTOR\_TRANSPORT |  | 26 | -0.31 | -0.90 | 0.623 | 0.854 | 1.000 | 3651 | tags=38%, list=30%, signal=55% |
| 5840 | GOBP\_REGULATION\_OF\_MEMBRANE\_TUBULATION |  | 5 | -0.47 | -0.90 | 0.616 | 0.855 | 1.000 | 1201 | tags=20%, list=10%, signal=22% |
| 5841 | GOBP\_TRIVALENT\_INORGANIC\_ANION\_HOMEOSTASIS |  | 4 | -0.50 | -0.90 | 0.635 | 0.855 | 1.000 | 1302 | tags=25%, list=11%, signal=28% |
| 5842 | GOMF\_MRNA\_3\_UTR\_AU\_RICH\_REGION\_BINDING |  | 18 | -0.33 | -0.90 | 0.607 | 0.854 | 1.000 | 4867 | tags=44%, list=41%, signal=75% |
| 5843 | GOCC\_INTERMEDIATE\_FILAMENT |  | 49 | -0.28 | -0.90 | 0.641 | 0.854 | 1.000 | 3765 | tags=33%, list=31%, signal=47% |
| 5844 | GOBP\_BONE\_TRABECULA\_FORMATION |  | 6 | -0.45 | -0.90 | 0.624 | 0.855 | 1.000 | 6622 | tags=100%, list=55%, signal=223% |
| 5845 | GOCC\_CLATHRIN\_COATED\_VESICLE |  | 126 | -0.25 | -0.90 | 0.688 | 0.855 | 1.000 | 3476 | tags=29%, list=29%, signal=41% |
| 5846 | GOMF\_FIBROBLAST\_GROWTH\_FACTOR\_BINDING |  | 18 | -0.33 | -0.90 | 0.615 | 0.855 | 1.000 | 3295 | tags=33%, list=27%, signal=46% |
| 5847 | GOBP\_SKELETAL\_MUSCLE\_TISSUE\_REGENERATION |  | 21 | -0.32 | -0.90 | 0.623 | 0.855 | 1.000 | 280 | tags=14%, list=2%, signal=15% |
| 5848 | GOBP\_NEGATIVE\_REGULATION\_OF\_GLUCOSE\_TRANSMEMBRANE\_TRANSPORT |  | 10 | -0.39 | -0.90 | 0.618 | 0.855 | 1.000 | 3934 | tags=50%, list=33%, signal=74% |
| 5849 | GOBP\_CHONDROCYTE\_HYPERTROPHY |  | 3 | -0.54 | -0.90 | 0.641 | 0.855 | 1.000 | 1441 | tags=33%, list=12%, signal=38% |
| 5850 | GOBP\_NEURAL\_RETINA\_DEVELOPMENT |  | 41 | -0.28 | -0.90 | 0.630 | 0.855 | 1.000 | 3589 | tags=37%, list=30%, signal=52% |
| 5851 | GOBP\_POSITIVE\_REGULATION\_OF\_AMPA\_RECEPTOR\_ACTIVITY |  | 4 | -0.50 | -0.90 | 0.629 | 0.855 | 1.000 | 6011 | tags=100%, list=50%, signal=200% |
| 5852 | GOBP\_DIET\_INDUCED\_THERMOGENESIS |  | 6 | -0.44 | -0.90 | 0.617 | 0.855 | 1.000 | 1939 | tags=33%, list=16%, signal=40% |
| 5853 | GOBP\_REGULATION\_OF\_POTASSIUM\_ION\_TRANSPORT |  | 60 | -0.27 | -0.90 | 0.645 | 0.855 | 1.000 | 4453 | tags=45%, list=37%, signal=71% |
| 5854 | GOCC\_ISWI\_TYPE\_COMPLEX |  | 7 | -0.43 | -0.90 | 0.624 | 0.855 | 1.000 | 6451 | tags=86%, list=54%, signal=185% |
| 5855 | GOBP\_POSITIVE\_REGULATION\_OF\_BLOOD\_PRESSURE\_BY\_EPINEPHRINE\_NOREPINEPHRINE |  | 4 | -0.50 | -0.90 | 0.623 | 0.855 | 1.000 | 893 | tags=25%, list=7%, signal=27% |
| 5856 | GOCC\_MEMBRANE\_PROTEIN\_COMPLEX |  | 762 | -0.23 | -0.90 | 0.870 | 0.855 | 1.000 | 3179 | tags=28%, list=26%, signal=35% |
| 5857 | GOBP\_NEURON\_RECOGNITION |  | 33 | -0.29 | -0.90 | 0.632 | 0.855 | 1.000 | 5564 | tags=55%, list=46%, signal=101% |
| 5858 | GOBP\_PROTEIN\_COMPLEX\_OLIGOMERIZATION |  | 144 | -0.24 | -0.90 | 0.696 | 0.856 | 1.000 | 1635 | tags=15%, list=14%, signal=17% |
| 5859 | GOBP\_NEUROTRANSMITTER\_RECEPTOR\_LOCALIZATION\_TO\_POSTSYNAPTIC\_SPECIALIZATION\_MEMBRANE |  | 10 | -0.39 | -0.90 | 0.611 | 0.856 | 1.000 | 4655 | tags=60%, list=39%, signal=98% |
| 5860 | GOBP\_NEGATIVE\_REGULATION\_OF\_STEROL\_TRANSPORT |  | 7 | -0.43 | -0.90 | 0.627 | 0.856 | 1.000 | 3565 | tags=71%, list=30%, signal=102% |
| 5861 | GOMF\_PHOSPHATASE\_INHIBITOR\_ACTIVITY |  | 24 | -0.31 | -0.90 | 0.619 | 0.856 | 1.000 | 4521 | tags=42%, list=38%, signal=67% |
| 5862 | GOBP\_NEGATIVE\_REGULATION\_OF\_GLUCOSE\_IMPORT |  | 6 | -0.45 | -0.90 | 0.634 | 0.856 | 1.000 | 289 | tags=17%, list=2%, signal=17% |
| 5863 | GOBP\_DETECTION\_OF\_STIMULUS |  | 139 | -0.24 | -0.90 | 0.699 | 0.857 | 1.000 | 4475 | tags=39%, list=37%, signal=61% |
| 5864 | GOBP\_ESTABLISHMENT\_OF\_PROTEIN\_LOCALIZATION\_TO\_POSTSYNAPTIC\_MEMBRANE |  | 10 | -0.38 | -0.90 | 0.617 | 0.857 | 1.000 | 3651 | tags=50%, list=30%, signal=72% |
| 5865 | GOMF\_STRUCTURAL\_CONSTITUENT\_OF\_CYTOSKELETON |  | 60 | -0.27 | -0.90 | 0.649 | 0.857 | 1.000 | 2721 | tags=20%, list=23%, signal=26% |
| 5866 | GOCC\_ADA2\_GCN5\_ADA3\_TRANSCRIPTION\_ACTIVATOR\_COMPLEX |  | 13 | -0.36 | -0.90 | 0.616 | 0.857 | 1.000 | 4145 | tags=38%, list=35%, signal=59% |
| 5867 | GOMF\_O\_METHYLTRANSFERASE\_ACTIVITY |  | 18 | -0.33 | -0.90 | 0.615 | 0.857 | 1.000 | 4082 | tags=39%, list=34%, signal=59% |
| 5868 | GOBP\_MEIOSIS\_II\_CELL\_CYCLE\_PROCESS |  | 6 | -0.45 | -0.90 | 0.621 | 0.857 | 1.000 | 5866 | tags=83%, list=49%, signal=163% |
| 5869 | GOBP\_RESPONSE\_TO\_CORTICOSTERONE |  | 9 | -0.39 | -0.90 | 0.621 | 0.857 | 1.000 | 1580 | tags=33%, list=13%, signal=38% |
| 5870 | GOBP\_ORGAN\_OR\_TISSUE\_SPECIFIC\_IMMUNE\_RESPONSE |  | 7 | -0.42 | -0.90 | 0.629 | 0.857 | 1.000 | 429 | tags=14%, list=4%, signal=15% |
| 5871 | GOBP\_RIBONUCLEOSIDE\_MONOPHOSPHATE\_METABOLIC\_PROCESS |  | 46 | -0.28 | -0.90 | 0.647 | 0.857 | 1.000 | 3428 | tags=30%, list=29%, signal=42% |
| 5872 | GOCC\_NUCLEAR\_ENVELOPE\_LUMEN |  | 8 | -0.41 | -0.90 | 0.615 | 0.857 | 1.000 | 231 | tags=13%, list=2%, signal=13% |
| 5873 | GOMF\_LONG\_CHAIN\_FATTY\_ACID\_TRANSPORTER\_ACTIVITY |  | 14 | -0.35 | -0.90 | 0.611 | 0.857 | 1.000 | 1534 | tags=29%, list=13%, signal=33% |
| 5874 | GOBP\_FAT\_PAD\_DEVELOPMENT |  | 5 | -0.47 | -0.90 | 0.637 | 0.856 | 1.000 | 1269 | tags=20%, list=11%, signal=22% |
| 5875 | GOBP\_ENDOSOME\_ORGANIZATION |  | 69 | -0.26 | -0.90 | 0.663 | 0.856 | 1.000 | 1824 | tags=19%, list=15%, signal=22% |
| 5876 | GOBP\_AMYLOID\_FIBRIL\_FORMATION |  | 18 | -0.33 | -0.90 | 0.616 | 0.856 | 1.000 | 1236 | tags=22%, list=10%, signal=25% |
| 5877 | GOCC\_CATALYTIC\_STEP\_2\_SPLICEOSOME |  | 70 | -0.26 | -0.90 | 0.662 | 0.856 | 1.000 | 6242 | tags=54%, list=52%, signal=112% |
| 5878 | GOBP\_REGULATION\_OF\_IRE1\_MEDIATED\_UNFOLDED\_PROTEIN\_RESPONSE |  | 13 | -0.36 | -0.90 | 0.618 | 0.856 | 1.000 | 5279 | tags=69%, list=44%, signal=123% |
| 5879 | GOMF\_NUCLEAR\_IMPORT\_SIGNAL\_RECEPTOR\_ACTIVITY |  | 17 | -0.34 | -0.90 | 0.619 | 0.856 | 1.000 | 5571 | tags=53%, list=46%, signal=99% |
| 5880 | GOMF\_ALPHA\_ACTININ\_BINDING |  | 18 | -0.33 | -0.90 | 0.622 | 0.857 | 1.000 | 2171 | tags=22%, list=18%, signal=27% |
| 5881 | GOBP\_NOTCH\_RECEPTOR\_PROCESSING\_LIGAND\_DEPENDENT |  | 6 | -0.44 | -0.90 | 0.627 | 0.857 | 1.000 | 6677 | tags=100%, list=56%, signal=225% |
| 5882 | GOCC\_MRNA\_CAP\_BINDING\_COMPLEX |  | 8 | -0.41 | -0.90 | 0.622 | 0.857 | 1.000 | 3797 | tags=38%, list=32%, signal=55% |
| 5883 | GOBP\_POTASSIUM\_ION\_TRANSPORT |  | 127 | -0.24 | -0.90 | 0.707 | 0.857 | 1.000 | 3854 | tags=34%, list=32%, signal=49% |
| 5884 | GOBP\_RESPONSE\_TO\_GRAVITY |  | 5 | -0.46 | -0.90 | 0.633 | 0.857 | 1.000 | 1699 | tags=20%, list=14%, signal=23% |
| 5885 | GOBP\_REGULATION\_OF\_ERAD\_PATHWAY |  | 14 | -0.35 | -0.90 | 0.617 | 0.857 | 1.000 | 1587 | tags=21%, list=13%, signal=25% |
| 5886 | GOCC\_COPII\_COATED\_ER\_TO\_GOLGI\_TRANSPORT\_VESICLE |  | 58 | -0.27 | -0.90 | 0.652 | 0.856 | 1.000 | 4021 | tags=36%, list=33%, signal=54% |
| 5887 | GOBP\_RESPONSE\_TO\_FLUID\_SHEAR\_STRESS |  | 26 | -0.31 | -0.90 | 0.633 | 0.856 | 1.000 | 3613 | tags=42%, list=30%, signal=60% |
| 5888 | GOBP\_NEGATIVE\_REGULATION\_OF\_GLYCOGEN\_METABOLIC\_PROCESS |  | 4 | -0.50 | -0.90 | 0.638 | 0.857 | 1.000 | 498 | tags=25%, list=4%, signal=26% |
| 5889 | GOMF\_LIPOPOLYSACCHARIDE\_IMMUNE\_RECEPTOR\_ACTIVITY |  | 4 | -0.50 | -0.90 | 0.638 | 0.857 | 1.000 | 1230 | tags=25%, list=10%, signal=28% |
| 5890 | GOBP\_REGULATION\_OF\_MRNA\_POLYADENYLATION |  | 13 | -0.36 | -0.90 | 0.609 | 0.857 | 1.000 | 4968 | tags=54%, list=41%, signal=92% |
| 5891 | GOMF\_CHANNEL\_REGULATOR\_ACTIVITY |  | 87 | -0.25 | -0.90 | 0.684 | 0.857 | 1.000 | 2604 | tags=22%, list=22%, signal=28% |
| 5892 | GOMF\_GLYCOSAMINOGLYCAN\_BINDING |  | 131 | -0.24 | -0.90 | 0.703 | 0.857 | 1.000 | 3266 | tags=26%, list=27%, signal=35% |
| 5893 | GOBP\_NEGATIVE\_REGULATION\_OF\_NUCLEOTIDE\_BIOSYNTHETIC\_PROCESS |  | 4 | -0.50 | -0.90 | 0.644 | 0.857 | 1.000 | 6028 | tags=100%, list=50%, signal=201% |
| 5894 | GOBP\_MICROTUBULE\_SEVERING |  | 9 | -0.39 | -0.90 | 0.622 | 0.857 | 1.000 | 5455 | tags=56%, list=45%, signal=102% |
| 5895 | GOBP\_POSITIVE\_REGULATION\_OF\_BROWN\_FAT\_CELL\_DIFFERENTIATION |  | 11 | -0.38 | -0.90 | 0.623 | 0.857 | 1.000 | 1477 | tags=27%, list=12%, signal=31% |
| 5896 | GOBP\_REGULATION\_OF\_CHROMOSOME\_CONDENSATION |  | 3 | -0.54 | -0.90 | 0.644 | 0.857 | 1.000 | 5579 | tags=100%, list=46%, signal=187% |
| 5897 | GOBP\_KIDNEY\_MORPHOGENESIS |  | 59 | -0.27 | -0.90 | 0.661 | 0.857 | 1.000 | 4341 | tags=44%, list=36%, signal=69% |
| 5898 | GOBP\_REGULATION\_OF\_ARTERY\_MORPHOGENESIS |  | 3 | -0.54 | -0.90 | 0.641 | 0.857 | 1.000 | 4979 | tags=67%, list=41%, signal=114% |
| 5899 | GOBP\_POSITIVE\_REGULATION\_OF\_CYCLIN\_DEPENDENT\_PROTEIN\_KINASE\_ACTIVITY |  | 28 | -0.30 | -0.90 | 0.633 | 0.857 | 1.000 | 597 | tags=11%, list=5%, signal=11% |
| 5900 | GOMF\_ATP\_DEPENDENT\_PROTEIN\_BINDING |  | 5 | -0.47 | -0.90 | 0.627 | 0.857 | 1.000 | 3390 | tags=40%, list=28%, signal=56% |
| 5901 | GOBP\_EXIT\_FROM\_MITOSIS |  | 22 | -0.32 | -0.90 | 0.622 | 0.857 | 1.000 | 4521 | tags=50%, list=38%, signal=80% |
| 5902 | GOBP\_ISOPRENOID\_BIOSYNTHETIC\_PROCESS |  | 21 | -0.32 | -0.90 | 0.635 | 0.857 | 1.000 | 51 | tags=10%, list=0%, signal=10% |
| 5903 | GOBP\_RESPONSE\_TO\_STAUROSPORINE |  | 3 | -0.54 | -0.90 | 0.650 | 0.857 | 1.000 | 5564 | tags=100%, list=46%, signal=186% |
| 5904 | GOBP\_POSITIVE\_REGULATION\_OF\_EPITHELIAL\_CELL\_DIFFERENTIATION |  | 23 | -0.31 | -0.90 | 0.622 | 0.858 | 1.000 | 3615 | tags=35%, list=30%, signal=50% |
| 5905 | GOBP\_REGULATION\_OF\_ASTROCYTE\_DIFFERENTIATION |  | 14 | -0.35 | -0.90 | 0.624 | 0.858 | 1.000 | 1597 | tags=21%, list=13%, signal=25% |
| 5906 | GOMF\_INTERLEUKIN\_6\_RECEPTOR\_BINDING |  | 5 | -0.47 | -0.90 | 0.633 | 0.858 | 1.000 | 4205 | tags=40%, list=35%, signal=62% |
| 5907 | GOBP\_STEM\_CELL\_DIVISION |  | 18 | -0.33 | -0.90 | 0.628 | 0.858 | 1.000 | 1187 | tags=17%, list=10%, signal=18% |
| 5908 | GOMF\_GROWTH\_FACTOR\_ACTIVITY |  | 72 | -0.26 | -0.90 | 0.665 | 0.858 | 1.000 | 1917 | tags=19%, list=16%, signal=23% |
| 5909 | GOBP\_INTERLEUKIN\_12\_PRODUCTION |  | 33 | -0.29 | -0.90 | 0.635 | 0.858 | 1.000 | 3510 | tags=36%, list=29%, signal=51% |
| 5910 | GOBP\_CYTOPLASM\_ORGANIZATION |  | 7 | -0.42 | -0.90 | 0.621 | 0.858 | 1.000 | 3862 | tags=71%, list=32%, signal=105% |
| 5911 | GOBP\_REGULATION\_OF\_WOUND\_HEALING |  | 81 | -0.25 | -0.90 | 0.673 | 0.858 | 1.000 | 3551 | tags=33%, list=30%, signal=47% |
| 5912 | GOMF\_INOSITOL\_HEXAKISPHOSPHATE\_5\_KINASE\_ACTIVITY |  | 4 | -0.50 | -0.90 | 0.652 | 0.858 | 1.000 | 1500 | tags=25%, list=12%, signal=29% |
| 5913 | GOBP\_VESICLE\_MEDIATED\_TRANSPORT\_BETWEEN\_ENDOSOMAL\_COMPARTMENTS |  | 36 | -0.29 | -0.90 | 0.633 | 0.858 | 1.000 | 2303 | tags=19%, list=19%, signal=24% |
| 5914 | GOBP\_REGULATION\_OF\_PROTEIN\_BINDING |  | 139 | -0.24 | -0.90 | 0.719 | 0.859 | 1.000 | 2308 | tags=18%, list=19%, signal=22% |
| 5915 | GOMF\_HYDROLASE\_ACTIVITY\_ACTING\_ON\_ETHER\_BONDS |  | 8 | -0.40 | -0.90 | 0.613 | 0.859 | 1.000 | 1918 | tags=25%, list=16%, signal=30% |
| 5916 | GOBP\_CELLULAR\_TRIGLYCERIDE\_HOMEOSTASIS |  | 4 | -0.49 | -0.90 | 0.628 | 0.859 | 1.000 | 6091 | tags=100%, list=51%, signal=203% |
| 5917 | GOBP\_FIBROBLAST\_APOPTOTIC\_PROCESS |  | 17 | -0.33 | -0.90 | 0.620 | 0.859 | 1.000 | 1579 | tags=24%, list=13%, signal=27% |
| 5918 | GOBP\_POSITIVE\_REGULATION\_OF\_HISTONE\_DEACETYLASE\_ACTIVITY |  | 3 | -0.54 | -0.90 | 0.652 | 0.860 | 1.000 | 3483 | tags=67%, list=29%, signal=94% |
| 5919 | GOBP\_PROTEIN\_LOCALIZATION\_TO\_CYTOPLASMIC\_STRESS\_GRANULE |  | 6 | -0.44 | -0.90 | 0.631 | 0.860 | 1.000 | 2731 | tags=33%, list=23%, signal=43% |
| 5920 | GOCC\_GAMMA\_SECRETASE\_COMPLEX |  | 6 | -0.44 | -0.90 | 0.631 | 0.860 | 1.000 | 6677 | tags=83%, list=56%, signal=188% |
| 5921 | GOBP\_NOREPINEPHRINE\_SECRETION |  | 10 | -0.38 | -0.90 | 0.630 | 0.859 | 1.000 | 2633 | tags=30%, list=22%, signal=38% |
| 5922 | GOMF\_ION\_TRANSMEMBRANE\_TRANSPORTER\_ACTIVITY |  | 535 | -0.23 | -0.90 | 0.841 | 0.859 | 1.000 | 3022 | tags=27%, list=25%, signal=35% |
| 5923 | GOMF\_PROTEIN\_ARGININE\_OMEGA\_N\_MONOMETHYLTRANSFERASE\_ACTIVITY |  | 3 | -0.54 | -0.90 | 0.652 | 0.859 | 1.000 | 3647 | tags=67%, list=30%, signal=96% |
| 5924 | GOCC\_TRAPP\_COMPLEX |  | 12 | -0.36 | -0.90 | 0.623 | 0.860 | 1.000 | 4301 | tags=50%, list=36%, signal=78% |
| 5925 | GOBP\_POSITIVE\_REGULATION\_OF\_VASCULAR\_PERMEABILITY |  | 10 | -0.38 | -0.90 | 0.623 | 0.860 | 1.000 | 3824 | tags=60%, list=32%, signal=88% |
| 5926 | GOBP\_G\_PROTEIN\_COUPLED\_GLUTAMATE\_RECEPTOR\_SIGNALING\_PATHWAY |  | 7 | -0.42 | -0.90 | 0.627 | 0.860 | 1.000 | 4819 | tags=71%, list=40%, signal=119% |
| 5927 | GOBP\_MYELOID\_CELL\_APOPTOTIC\_PROCESS |  | 19 | -0.32 | -0.89 | 0.631 | 0.860 | 1.000 | 3392 | tags=47%, list=28%, signal=66% |
| 5928 | GOBP\_REGULATION\_OF\_STRESS\_GRANULE\_ASSEMBLY |  | 4 | -0.49 | -0.89 | 0.634 | 0.860 | 1.000 | 685 | tags=25%, list=6%, signal=27% |
| 5929 | GOBP\_ICOSANOID\_TRANSPORT |  | 31 | -0.29 | -0.89 | 0.635 | 0.860 | 1.000 | 2913 | tags=42%, list=24%, signal=55% |
| 5930 | GOBP\_NEGATIVE\_REGULATION\_OF\_HISTONE\_H3\_K9\_METHYLATION |  | 8 | -0.41 | -0.89 | 0.635 | 0.860 | 1.000 | 3644 | tags=63%, list=30%, signal=90% |
| 5931 | GOBP\_RESPONSE\_TO\_GLYCOSIDE |  | 3 | -0.54 | -0.89 | 0.662 | 0.860 | 1.000 | 3888 | tags=67%, list=32%, signal=99% |
| 5932 | GOBP\_REGULATION\_OF\_CHOLESTEROL\_METABOLIC\_PROCESS |  | 45 | -0.28 | -0.89 | 0.646 | 0.860 | 1.000 | 4272 | tags=42%, list=36%, signal=65% |
| 5933 | GOMF\_MINOR\_GROOVE\_OF\_ADENINE\_THYMINE\_RICH\_DNA\_BINDING |  | 8 | -0.41 | -0.89 | 0.628 | 0.860 | 1.000 | 2474 | tags=38%, list=21%, signal=47% |
| 5934 | GOBP\_SURFACTANT\_HOMEOSTASIS |  | 7 | -0.42 | -0.89 | 0.629 | 0.860 | 1.000 | 4329 | tags=57%, list=36%, signal=89% |
| 5935 | GOMF\_C\_X3\_C\_CHEMOKINE\_BINDING |  | 5 | -0.47 | -0.89 | 0.641 | 0.860 | 1.000 | 6421 | tags=100%, list=53%, signal=215% |
| 5936 | GOBP\_BRANCHED\_CHAIN\_AMINO\_ACID\_TRANSPORT |  | 6 | -0.44 | -0.89 | 0.643 | 0.860 | 1.000 | 3155 | tags=50%, list=26%, signal=68% |
| 5937 | GOBP\_REGULATION\_OF\_AUTOPHAGOSOME\_MATURATION |  | 11 | -0.37 | -0.89 | 0.624 | 0.860 | 1.000 | 3967 | tags=36%, list=33%, signal=54% |
| 5938 | GOBP\_SOMATOSTATIN\_RECEPTOR\_SIGNALING\_PATHWAY |  | 3 | -0.53 | -0.89 | 0.652 | 0.860 | 1.000 | 2741 | tags=67%, list=23%, signal=86% |
| 5939 | GOBP\_REGULATION\_OF\_GLYCOGEN\_METABOLIC\_PROCESS |  | 23 | -0.31 | -0.89 | 0.628 | 0.860 | 1.000 | 3934 | tags=43%, list=33%, signal=65% |
| 5940 | GOCC\_CILIARY\_BASAL\_BODY |  | 107 | -0.25 | -0.89 | 0.696 | 0.860 | 1.000 | 2299 | tags=19%, list=19%, signal=23% |
| 5941 | GOBP\_PEPTIDE\_CROSS\_LINKING |  | 17 | -0.33 | -0.89 | 0.634 | 0.860 | 1.000 | 3266 | tags=41%, list=27%, signal=56% |
| 5942 | GOBP\_NEGATIVE\_REGULATION\_OF\_NEUROBLAST\_PROLIFERATION |  | 3 | -0.54 | -0.89 | 0.654 | 0.860 | 1.000 | 501 | tags=33%, list=4%, signal=35% |
| 5943 | GOMF\_PROTEIN\_FOLDING\_CHAPERONE |  | 27 | -0.30 | -0.89 | 0.636 | 0.861 | 1.000 | 2201 | tags=22%, list=18%, signal=27% |
| 5944 | GOBP\_CERAMIDE\_BIOSYNTHETIC\_PROCESS |  | 50 | -0.27 | -0.89 | 0.655 | 0.860 | 1.000 | 2621 | tags=28%, list=22%, signal=36% |
| 5945 | GOBP\_REGULATION\_OF\_RENAL\_SYSTEM\_PROCESS |  | 20 | -0.32 | -0.89 | 0.629 | 0.861 | 1.000 | 2480 | tags=25%, list=21%, signal=31% |
| 5946 | GOBP\_REGULATION\_OF\_RESPIRATORY\_SYSTEM\_PROCESS |  | 4 | -0.49 | -0.89 | 0.649 | 0.861 | 1.000 | 1149 | tags=25%, list=10%, signal=28% |
| 5947 | GOBP\_ENDOCRINE\_HORMONE\_SECRETION |  | 27 | -0.30 | -0.89 | 0.635 | 0.860 | 1.000 | 2367 | tags=26%, list=20%, signal=32% |
| 5948 | GOBP\_REGULATION\_OF\_EPITHELIAL\_CELL\_PROLIFERATION\_INVOLVED\_IN\_LUNG\_MORPHOGENESIS |  | 6 | -0.44 | -0.89 | 0.630 | 0.860 | 1.000 | 408 | tags=17%, list=3%, signal=17% |
| 5949 | GOBP\_RESPONSE\_TO\_HYDROSTATIC\_PRESSURE |  | 5 | -0.46 | -0.89 | 0.649 | 0.861 | 1.000 | 5903 | tags=80%, list=49%, signal=157% |
| 5950 | GOBP\_NEGATIVE\_REGULATION\_OF\_BICELLULAR\_TIGHT\_JUNCTION\_ASSEMBLY |  | 3 | -0.53 | -0.89 | 0.654 | 0.861 | 1.000 | 3547 | tags=67%, list=30%, signal=95% |
| 5951 | GOMF\_PEROXISOME\_TARGETING\_SEQUENCE\_BINDING |  | 3 | -0.53 | -0.89 | 0.658 | 0.861 | 1.000 | 5600 | tags=100%, list=47%, signal=187% |
| 5952 | GOBP\_LONG\_CHAIN\_FATTY\_ACYL\_COA\_METABOLIC\_PROCESS |  | 20 | -0.32 | -0.89 | 0.627 | 0.861 | 1.000 | 4801 | tags=55%, list=40%, signal=91% |
| 5953 | GOMF\_CAMP\_DEPENDENT\_PROTEIN\_KINASE\_INHIBITOR\_ACTIVITY |  | 8 | -0.41 | -0.89 | 0.628 | 0.861 | 1.000 | 56 | tags=13%, list=0%, signal=13% |
| 5954 | GOMF\_P53\_BINDING |  | 47 | -0.27 | -0.89 | 0.655 | 0.861 | 1.000 | 3214 | tags=26%, list=27%, signal=35% |
| 5955 | GOBP\_MONOCARBOXYLIC\_ACID\_METABOLIC\_PROCESS |  | 435 | -0.23 | -0.89 | 0.829 | 0.861 | 1.000 | 4302 | tags=39%, list=36%, signal=58% |
| 5956 | GOBP\_VESICLE\_BUDDING\_FROM\_MEMBRANE |  | 87 | -0.25 | -0.89 | 0.691 | 0.861 | 1.000 | 3212 | tags=28%, list=27%, signal=37% |
| 5957 | GOCC\_COMPLEX\_OF\_COLLAGEN\_TRIMERS |  | 13 | -0.36 | -0.89 | 0.626 | 0.861 | 1.000 | 5349 | tags=69%, list=45%, signal=125% |
| 5958 | GOCC\_NUCLEAR\_UBIQUITIN\_LIGASE\_COMPLEX |  | 35 | -0.29 | -0.89 | 0.640 | 0.861 | 1.000 | 4767 | tags=46%, list=40%, signal=76% |
| 5959 | GOBP\_MEMBRANE\_LIPID\_METABOLIC\_PROCESS |  | 157 | -0.24 | -0.89 | 0.732 | 0.861 | 1.000 | 4467 | tags=40%, list=37%, signal=63% |
| 5960 | GOMF\_LAMININ\_1\_BINDING |  | 5 | -0.46 | -0.89 | 0.640 | 0.861 | 1.000 | 3565 | tags=40%, list=30%, signal=57% |
| 5961 | GOBP\_TISSUE\_HOMEOSTASIS |  | 164 | -0.24 | -0.89 | 0.728 | 0.862 | 1.000 | 3899 | tags=35%, list=32%, signal=51% |
| 5962 | GOBP\_REGULATION\_OF\_RELAXATION\_OF\_MUSCLE |  | 7 | -0.42 | -0.89 | 0.634 | 0.862 | 1.000 | 1304 | tags=29%, list=11%, signal=32% |
| 5963 | GOBP\_MODULATION\_BY\_HOST\_OF\_SYMBIONT\_MOLECULAR\_FUNCTION |  | 4 | -0.49 | -0.89 | 0.652 | 0.862 | 1.000 | 4657 | tags=50%, list=39%, signal=82% |
| 5964 | GOMF\_FLOPPASE\_ACTIVITY |  | 4 | -0.49 | -0.89 | 0.653 | 0.862 | 1.000 | 2752 | tags=50%, list=23%, signal=65% |
| 5965 | GOBP\_REGULATION\_OF\_CELL\_PROJECTION\_SIZE |  | 6 | -0.44 | -0.89 | 0.638 | 0.862 | 1.000 | 5082 | tags=67%, list=42%, signal=116% |
| 5966 | GOBP\_REGULATION\_OF\_PENILE\_ERECTION |  | 6 | -0.44 | -0.89 | 0.634 | 0.862 | 1.000 | 4984 | tags=83%, list=41%, signal=142% |
| 5967 | GOCC\_FLOTILLIN\_COMPLEX |  | 6 | -0.44 | -0.89 | 0.639 | 0.862 | 1.000 | 139 | tags=17%, list=1%, signal=17% |
| 5968 | GOMF\_SOMATOSTATIN\_RECEPTOR\_ACTIVITY |  | 3 | -0.53 | -0.89 | 0.661 | 0.862 | 1.000 | 2741 | tags=67%, list=23%, signal=86% |
| 5969 | GOBP\_KERATINOCYTE\_MIGRATION |  | 15 | -0.34 | -0.89 | 0.626 | 0.862 | 1.000 | 3392 | tags=60%, list=28%, signal=84% |
| 5970 | GOBP\_NEGATIVE\_REGULATION\_OF\_NUCLEASE\_ACTIVITY |  | 7 | -0.42 | -0.89 | 0.638 | 0.863 | 1.000 | 3722 | tags=43%, list=31%, signal=62% |
| 5971 | GOCC\_HIPPOCAMPAL\_MOSSY\_FIBER |  | 4 | -0.50 | -0.89 | 0.654 | 0.863 | 1.000 | 2869 | tags=75%, list=24%, signal=99% |
| 5972 | GOBP\_LONG\_CHAIN\_FATTY\_ACID\_IMPORT\_INTO\_CELL |  | 13 | -0.36 | -0.89 | 0.630 | 0.863 | 1.000 | 4053 | tags=62%, list=34%, signal=93% |
| 5973 | GOBP\_POSITIVE\_REGULATION\_OF\_FIBROBLAST\_APOPTOTIC\_PROCESS |  | 7 | -0.42 | -0.89 | 0.632 | 0.863 | 1.000 | 1579 | tags=43%, list=13%, signal=49% |
| 5974 | GOMF\_NADPLUS\_ADP\_RIBOSYLTRANSFERASE\_ACTIVITY |  | 16 | -0.34 | -0.89 | 0.634 | 0.863 | 1.000 | 1704 | tags=25%, list=14%, signal=29% |
| 5975 | GOBP\_CELL\_CELL\_SIGNALING\_INVOLVED\_IN\_CARDIAC\_CONDUCTION |  | 19 | -0.32 | -0.89 | 0.633 | 0.863 | 1.000 | 3295 | tags=32%, list=27%, signal=43% |
| 5976 | GOMF\_WNT\_ACTIVATED\_RECEPTOR\_ACTIVITY |  | 13 | -0.36 | -0.89 | 0.629 | 0.863 | 1.000 | 5283 | tags=46%, list=44%, signal=82% |
| 5977 | GOMF\_RIBOSE\_PHOSPHATE\_DIPHOSPHOKINASE\_ACTIVITY |  | 4 | -0.49 | -0.89 | 0.643 | 0.862 | 1.000 | 4231 | tags=75%, list=35%, signal=116% |
| 5978 | GOBP\_CELLULAR\_RESPONSE\_TO\_REACTIVE\_OXYGEN\_SPECIES |  | 112 | -0.24 | -0.89 | 0.704 | 0.862 | 1.000 | 4495 | tags=38%, list=37%, signal=61% |
| 5979 | GOCC\_TRANSCRIPTION\_EXPORT\_COMPLEX\_2 |  | 4 | -0.49 | -0.89 | 0.654 | 0.862 | 1.000 | 5370 | tags=50%, list=45%, signal=90% |
| 5980 | GOBP\_COLLAGEN\_CATABOLIC\_PROCESS |  | 27 | -0.30 | -0.89 | 0.640 | 0.863 | 1.000 | 5043 | tags=44%, list=42%, signal=76% |
| 5981 | GOBP\_RNA\_POLYMERASE\_II\_PREINITIATION\_COMPLEX\_ASSEMBLY |  | 17 | -0.33 | -0.89 | 0.623 | 0.863 | 1.000 | 875 | tags=12%, list=7%, signal=13% |
| 5982 | GOBP\_CARDIAC\_CONDUCTION |  | 78 | -0.25 | -0.89 | 0.688 | 0.863 | 1.000 | 5259 | tags=44%, list=44%, signal=77% |
| 5983 | GOBP\_REGULATION\_OF\_PROTEIN\_LOCALIZATION\_TO\_CELL\_PERIPHERY |  | 86 | -0.25 | -0.89 | 0.694 | 0.863 | 1.000 | 4803 | tags=42%, list=40%, signal=69% |
| 5984 | GOBP\_POSTTRANSCRIPTIONAL\_REGULATION\_OF\_GENE\_EXPRESSION |  | 450 | -0.23 | -0.89 | 0.845 | 0.863 | 1.000 | 4316 | tags=32%, list=36%, signal=49% |
| 5985 | GOCC\_EXTRINSIC\_COMPONENT\_OF\_MITOCHONDRIAL\_INNER\_MEMBRANE |  | 11 | -0.37 | -0.89 | 0.640 | 0.863 | 1.000 | 341 | tags=9%, list=3%, signal=9% |
| 5986 | GOMF\_3\_HYDROXYACYL\_COA\_DEHYDROGENASE\_ACTIVITY |  | 6 | -0.44 | -0.89 | 0.640 | 0.863 | 1.000 | 2698 | tags=50%, list=22%, signal=64% |
| 5987 | GOBP\_POSITIVE\_REGULATION\_OF\_LYMPHOCYTE\_CHEMOTAXIS |  | 13 | -0.35 | -0.89 | 0.623 | 0.863 | 1.000 | 2955 | tags=46%, list=25%, signal=61% |
| 5988 | GOBP\_ESTABLISHMENT\_OF\_ENDOTHELIAL\_INTESTINAL\_BARRIER |  | 10 | -0.38 | -0.89 | 0.628 | 0.864 | 1.000 | 4549 | tags=60%, list=38%, signal=96% |
| 5989 | GOBP\_SPERM\_MOTILITY |  | 43 | -0.28 | -0.89 | 0.653 | 0.863 | 1.000 | 3612 | tags=37%, list=30%, signal=53% |
| 5990 | GOBP\_REGULATION\_OF\_TRIGLYCERIDE\_METABOLIC\_PROCESS |  | 23 | -0.31 | -0.89 | 0.631 | 0.863 | 1.000 | 4853 | tags=52%, list=40%, signal=87% |
| 5991 | GOBP\_REGULATION\_OF\_HEXOKINASE\_ACTIVITY |  | 6 | -0.43 | -0.89 | 0.639 | 0.864 | 1.000 | 320 | tags=17%, list=3%, signal=17% |
| 5992 | GOBP\_HISTONE\_H4\_K20\_TRIMETHYLATION |  | 5 | -0.46 | -0.89 | 0.650 | 0.864 | 1.000 | 3587 | tags=60%, list=30%, signal=86% |
| 5993 | GOBP\_NEGATIVE\_REGULATION\_OF\_REGULATORY\_T\_CELL\_DIFFERENTIATION |  | 4 | -0.49 | -0.89 | 0.662 | 0.864 | 1.000 | 1734 | tags=25%, list=14%, signal=29% |
| 5994 | GOBP\_REGULATION\_OF\_PERK\_MEDIATED\_UNFOLDED\_PROTEIN\_RESPONSE |  | 8 | -0.41 | -0.89 | 0.641 | 0.864 | 1.000 | 4646 | tags=63%, list=39%, signal=102% |
| 5995 | GOMF\_EPOXIDE\_HYDROLASE\_ACTIVITY |  | 6 | -0.43 | -0.89 | 0.653 | 0.864 | 1.000 | 645 | tags=17%, list=5%, signal=18% |
| 5996 | GOCC\_ANCHORED\_COMPONENT\_OF\_SYNAPTIC\_MEMBRANE |  | 9 | -0.39 | -0.89 | 0.629 | 0.863 | 1.000 | 6046 | tags=89%, list=50%, signal=179% |
| 5997 | GOBP\_TRAIL\_ACTIVATED\_APOPTOTIC\_SIGNALING\_PATHWAY |  | 8 | -0.40 | -0.89 | 0.631 | 0.864 | 1.000 | 3841 | tags=50%, list=32%, signal=73% |
| 5998 | GOBP\_REGULATION\_OF\_SEROTONIN\_SECRETION |  | 4 | -0.49 | -0.89 | 0.659 | 0.864 | 1.000 | 1650 | tags=50%, list=14%, signal=58% |
| 5999 | GOBP\_ENDODERMAL\_CELL\_FATE\_COMMITMENT |  | 8 | -0.40 | -0.89 | 0.636 | 0.864 | 1.000 | 4677 | tags=63%, list=39%, signal=102% |
| 6000 | GOBP\_NEGATIVE\_REGULATION\_OF\_ION\_TRANSPORT |  | 192 | -0.23 | -0.89 | 0.754 | 0.865 | 1.000 | 4148 | tags=35%, list=35%, signal=52% |
| 6001 | GOBP\_REGULATION\_OF\_HEMATOPOIETIC\_STEM\_CELL\_PROLIFERATION |  | 7 | -0.42 | -0.89 | 0.641 | 0.865 | 1.000 | 3400 | tags=57%, list=28%, signal=80% |
| 6002 | GOBP\_PURINE\_DEOXYRIBONUCLEOSIDE\_MONOPHOSPHATE\_METABOLIC\_PROCESS |  | 4 | -0.49 | -0.89 | 0.660 | 0.865 | 1.000 | 1107 | tags=50%, list=9%, signal=55% |
| 6003 | GOCC\_BETA\_CATENIN\_TCF\_COMPLEX |  | 12 | -0.36 | -0.89 | 0.631 | 0.865 | 1.000 | 5425 | tags=58%, list=45%, signal=106% |
| 6004 | GOMF\_HSP90\_PROTEIN\_BINDING |  | 30 | -0.29 | -0.89 | 0.649 | 0.865 | 1.000 | 2901 | tags=27%, list=24%, signal=35% |
| 6005 | GOBP\_NEUTRAL\_LIPID\_CATABOLIC\_PROCESS |  | 32 | -0.29 | -0.89 | 0.657 | 0.865 | 1.000 | 4190 | tags=44%, list=35%, signal=67% |
| 6006 | GOBP\_POSITIVE\_REGULATION\_OF\_CAMP\_DEPENDENT\_PROTEIN\_KINASE\_ACTIVITY |  | 5 | -0.46 | -0.89 | 0.654 | 0.865 | 1.000 | 5903 | tags=80%, list=49%, signal=157% |
| 6007 | GOCC\_NASCENT\_POLYPEPTIDE\_ASSOCIATED\_COMPLEX |  | 3 | -0.53 | -0.89 | 0.654 | 0.865 | 1.000 | 2736 | tags=33%, list=23%, signal=43% |
| 6008 | GOBP\_T\_CELL\_CHEMOTAXIS |  | 15 | -0.34 | -0.89 | 0.639 | 0.865 | 1.000 | 3534 | tags=53%, list=29%, signal=75% |
| 6009 | GOMF\_BIOACTIVE\_LIPID\_RECEPTOR\_ACTIVITY |  | 11 | -0.37 | -0.89 | 0.641 | 0.865 | 1.000 | 1199 | tags=18%, list=10%, signal=20% |
| 6010 | GOBP\_RECEPTOR\_CLUSTERING |  | 35 | -0.29 | -0.89 | 0.650 | 0.865 | 1.000 | 3460 | tags=26%, list=29%, signal=36% |
| 6011 | GOBP\_POSITIVE\_REGULATION\_OF\_NEURON\_DEATH |  | 60 | -0.26 | -0.89 | 0.676 | 0.865 | 1.000 | 2374 | tags=22%, list=20%, signal=27% |
| 6012 | GOBP\_REGULATION\_OF\_MICROVILLUS\_ASSEMBLY |  | 5 | -0.46 | -0.89 | 0.640 | 0.865 | 1.000 | 3619 | tags=60%, list=30%, signal=86% |
| 6013 | GOCC\_VACUOLE |  | 570 | -0.22 | -0.89 | 0.867 | 0.865 | 1.000 | 3512 | tags=30%, list=29%, signal=41% |
| 6014 | GOBP\_RESPONSE\_TO\_IRON\_ION |  | 17 | -0.33 | -0.89 | 0.638 | 0.865 | 1.000 | 1580 | tags=24%, list=13%, signal=27% |
| 6015 | GOMF\_ENDOPEPTIDASE\_REGULATOR\_ACTIVITY |  | 79 | -0.25 | -0.89 | 0.693 | 0.865 | 1.000 | 3289 | tags=23%, list=27%, signal=31% |
| 6016 | GOBP\_PYRIMIDINE\_CONTAINING\_COMPOUND\_CATABOLIC\_PROCESS |  | 25 | -0.30 | -0.89 | 0.643 | 0.865 | 1.000 | 874 | tags=12%, list=7%, signal=13% |
| 6017 | GOBP\_REGULATION\_OF\_PRESYNAPTIC\_CYTOSOLIC\_CALCIUM\_ION\_CONCENTRATION |  | 13 | -0.35 | -0.89 | 0.625 | 0.865 | 1.000 | 4148 | tags=54%, list=35%, signal=82% |
| 6018 | GOBP\_RESPONSE\_TO\_DIETARY\_EXCESS |  | 11 | -0.37 | -0.89 | 0.630 | 0.865 | 1.000 | 1939 | tags=36%, list=16%, signal=43% |
| 6019 | GOBP\_BETA\_CATENIN\_DESTRUCTION\_COMPLEX\_ASSEMBLY |  | 4 | -0.49 | -0.89 | 0.665 | 0.865 | 1.000 | 6135 | tags=100%, list=51%, signal=204% |
| 6020 | GOBP\_NEGATIVE\_REGULATION\_OF\_T\_HELPER\_CELL\_DIFFERENTIATION |  | 12 | -0.36 | -0.89 | 0.633 | 0.865 | 1.000 | 1951 | tags=33%, list=16%, signal=40% |
| 6021 | GOBP\_AMINO\_ACID\_NEUROTRANSMITTER\_REUPTAKE |  | 3 | -0.52 | -0.88 | 0.665 | 0.866 | 1.000 | 5711 | tags=100%, list=48%, signal=191% |
| 6022 | GOBP\_REGULATION\_OF\_MICROTUBULE\_MOTOR\_ACTIVITY |  | 8 | -0.40 | -0.88 | 0.648 | 0.866 | 1.000 | 5153 | tags=63%, list=43%, signal=109% |
| 6023 | GOBP\_HYDROGEN\_PEROXIDE\_BIOSYNTHETIC\_PROCESS |  | 8 | -0.40 | -0.88 | 0.635 | 0.866 | 1.000 | 897 | tags=25%, list=7%, signal=27% |
| 6024 | GOBP\_HETEROCHROMATIN\_ORGANIZATION\_INVOLVED\_IN\_CHROMATIN\_SILENCING |  | 5 | -0.46 | -0.88 | 0.655 | 0.866 | 1.000 | 2656 | tags=40%, list=22%, signal=51% |
| 6025 | GOMF\_RECEPTOR\_ANTAGONIST\_ACTIVITY |  | 8 | -0.40 | -0.88 | 0.633 | 0.866 | 1.000 | 1899 | tags=38%, list=16%, signal=45% |
| 6026 | GOBP\_MITOTIC\_NUCLEAR\_MEMBRANE\_ORGANIZATION |  | 8 | -0.40 | -0.88 | 0.637 | 0.866 | 1.000 | 1683 | tags=25%, list=14%, signal=29% |
| 6027 | GOMF\_LYSINE\_N\_METHYLTRANSFERASE\_ACTIVITY |  | 42 | -0.28 | -0.88 | 0.666 | 0.866 | 1.000 | 3200 | tags=31%, list=27%, signal=42% |
| 6028 | GOBP\_PLASMA\_MEMBRANE\_TO\_ENDOSOME\_TRANSPORT |  | 8 | -0.40 | -0.88 | 0.645 | 0.866 | 1.000 | 3506 | tags=38%, list=29%, signal=53% |
| 6029 | GOMF\_INOSITOL\_HEXAKISPHOSPHATE\_KINASE\_ACTIVITY |  | 6 | -0.44 | -0.88 | 0.654 | 0.866 | 1.000 | 4916 | tags=50%, list=41%, signal=85% |
| 6030 | GOBP\_REGULATION\_OF\_TRANSCRIPTION\_FROM\_RNA\_POLYMERASE\_II\_PROMOTER\_IN\_RESPONSE\_TO\_OXIDATIVE\_STRESS |  | 9 | -0.38 | -0.88 | 0.639 | 0.866 | 1.000 | 3235 | tags=33%, list=27%, signal=46% |
| 6031 | GOBP\_REGULATION\_OF\_MRNA\_METABOLIC\_PROCESS |  | 247 | -0.23 | -0.88 | 0.785 | 0.866 | 1.000 | 4925 | tags=40%, list=41%, signal=66% |
| 6032 | GOBP\_INTERLEUKIN\_1\_MEDIATED\_SIGNALING\_PATHWAY |  | 77 | -0.25 | -0.88 | 0.698 | 0.866 | 1.000 | 4211 | tags=32%, list=35%, signal=50% |
| 6033 | GOMF\_URIDYLYLTRANSFERASE\_ACTIVITY |  | 4 | -0.49 | -0.88 | 0.662 | 0.866 | 1.000 | 79 | tags=25%, list=1%, signal=25% |
| 6034 | GOBP\_TRANSCRIPTION\_DEPENDENT\_TETHERING\_OF\_RNA\_POLYMERASE\_II\_GENE\_DNA\_AT\_NUCLEAR\_PERIPHERY |  | 6 | -0.43 | -0.88 | 0.658 | 0.866 | 1.000 | 3822 | tags=33%, list=32%, signal=49% |
| 6035 | GOMF\_DIACYLGLYCEROL\_BINDING |  | 9 | -0.39 | -0.88 | 0.630 | 0.866 | 1.000 | 3723 | tags=67%, list=31%, signal=97% |
| 6036 | GOBP\_REGULATION\_OF\_EXTRACELLULAR\_MATRIX\_ASSEMBLY |  | 13 | -0.35 | -0.88 | 0.647 | 0.866 | 1.000 | 5967 | tags=69%, list=50%, signal=137% |
| 6037 | GOMF\_UDP\_GALACTOSE\_BETA\_N\_ACETYLGLUCOSAMINE\_BETA\_1\_3\_GALACTOSYLTRANSFERASE\_ACTIVITY |  | 3 | -0.53 | -0.88 | 0.670 | 0.866 | 1.000 | 339 | tags=33%, list=3%, signal=34% |
| 6038 | GOCC\_TUBULAR\_ENDOSOME |  | 4 | -0.49 | -0.88 | 0.663 | 0.866 | 1.000 | 4986 | tags=75%, list=42%, signal=128% |
| 6039 | GOBP\_RNA\_POLYADENYLATION |  | 34 | -0.29 | -0.88 | 0.651 | 0.866 | 1.000 | 5165 | tags=44%, list=43%, signal=77% |
| 6040 | GOBP\_REGULATION\_OF\_RNA\_SPLICING |  | 107 | -0.24 | -0.88 | 0.722 | 0.867 | 1.000 | 4441 | tags=34%, list=37%, signal=53% |
| 6041 | GOBP\_PROTEIN\_LOCALIZATION\_TO\_LYSOSOME |  | 34 | -0.29 | -0.88 | 0.658 | 0.867 | 1.000 | 4585 | tags=50%, list=38%, signal=81% |
| 6042 | GOBP\_AUTOCRINE\_SIGNALING |  | 3 | -0.53 | -0.88 | 0.665 | 0.867 | 1.000 | 2871 | tags=33%, list=24%, signal=44% |
| 6043 | GOCC\_FIBRILLAR\_COLLAGEN\_TRIMER |  | 9 | -0.39 | -0.88 | 0.646 | 0.867 | 1.000 | 6081 | tags=78%, list=51%, signal=157% |
| 6044 | GOBP\_CELLULAR\_HYPOTONIC\_RESPONSE |  | 6 | -0.43 | -0.88 | 0.646 | 0.867 | 1.000 | 148 | tags=17%, list=1%, signal=17% |
| 6045 | GOMF\_SULFURTRANSFERASE\_ACTIVITY |  | 10 | -0.38 | -0.88 | 0.648 | 0.868 | 1.000 | 40 | tags=10%, list=0%, signal=10% |
| 6046 | GOBP\_RELAXATION\_OF\_CARDIAC\_MUSCLE |  | 14 | -0.35 | -0.88 | 0.643 | 0.868 | 1.000 | 1304 | tags=21%, list=11%, signal=24% |
| 6047 | GOBP\_NEGATIVE\_REGULATION\_OF\_PROTEIN\_SUMOYLATION |  | 6 | -0.43 | -0.88 | 0.649 | 0.868 | 1.000 | 4256 | tags=50%, list=35%, signal=77% |
| 6048 | GOMF\_D1\_DOPAMINE\_RECEPTOR\_BINDING |  | 5 | -0.46 | -0.88 | 0.659 | 0.868 | 1.000 | 6533 | tags=100%, list=54%, signal=219% |
| 6049 | GOBP\_ANTIMICROBIAL\_HUMORAL\_IMMUNE\_RESPONSE\_MEDIATED\_BY\_ANTIMICROBIAL\_PEPTIDE |  | 11 | -0.37 | -0.88 | 0.639 | 0.868 | 1.000 | 1011 | tags=18%, list=8%, signal=20% |
| 6050 | GOBP\_REGULATION\_OF\_MICROVILLUS\_LENGTH |  | 6 | -0.44 | -0.88 | 0.654 | 0.868 | 1.000 | 5082 | tags=67%, list=42%, signal=116% |
| 6051 | GOBP\_REGULATION\_OF\_LYMPHOCYTE\_CHEMOTAXIS |  | 13 | -0.35 | -0.88 | 0.640 | 0.869 | 1.000 | 2955 | tags=46%, list=25%, signal=61% |
| 6052 | GOCC\_ENDOSOME |  | 694 | -0.22 | -0.88 | 0.897 | 0.869 | 1.000 | 3401 | tags=28%, list=28%, signal=37% |
| 6053 | GOBP\_REGULATION\_OF\_VESICLE\_SIZE |  | 10 | -0.37 | -0.88 | 0.651 | 0.869 | 1.000 | 1804 | tags=30%, list=15%, signal=35% |
| 6054 | GOBP\_REGULATION\_OF\_MRNA\_CATABOLIC\_PROCESS |  | 159 | -0.24 | -0.88 | 0.754 | 0.870 | 1.000 | 5048 | tags=42%, list=42%, signal=72% |
| 6055 | GOMF\_3\_5\_EXONUCLEASE\_ACTIVITY |  | 42 | -0.27 | -0.88 | 0.666 | 0.870 | 1.000 | 5337 | tags=48%, list=44%, signal=85% |
| 6056 | GOCC\_RIBOSE\_PHOSPHATE\_DIPHOSPHOKINASE\_COMPLEX |  | 4 | -0.49 | -0.88 | 0.667 | 0.871 | 1.000 | 4231 | tags=75%, list=35%, signal=116% |
| 6057 | GOBP\_NEGATIVE\_REGULATION\_OF\_IMMUNOGLOBULIN\_PRODUCTION |  | 8 | -0.40 | -0.88 | 0.643 | 0.871 | 1.000 | 1560 | tags=25%, list=13%, signal=29% |
| 6058 | GOBP\_OVARIAN\_FOLLICLE\_DEVELOPMENT |  | 29 | -0.29 | -0.88 | 0.660 | 0.871 | 1.000 | 2188 | tags=24%, list=18%, signal=29% |
| 6059 | GOBP\_REGULATION\_OF\_GLIOGENESIS |  | 61 | -0.26 | -0.88 | 0.694 | 0.871 | 1.000 | 1847 | tags=20%, list=15%, signal=23% |
| 6060 | GOCC\_CHROMOSOME\_PASSENGER\_COMPLEX |  | 3 | -0.53 | -0.88 | 0.677 | 0.871 | 1.000 | 2962 | tags=33%, list=25%, signal=44% |
| 6061 | GOBP\_AMYLOID\_BETA\_CLEARANCE\_BY\_TRANSCYTOSIS |  | 7 | -0.42 | -0.88 | 0.652 | 0.872 | 1.000 | 5695 | tags=71%, list=47%, signal=136% |
| 6062 | GOBP\_TOOTH\_MINERALIZATION |  | 12 | -0.36 | -0.88 | 0.646 | 0.871 | 1.000 | 2515 | tags=33%, list=21%, signal=42% |
| 6063 | GOBP\_DENDRITIC\_CELL\_CHEMOTAXIS |  | 17 | -0.33 | -0.88 | 0.645 | 0.872 | 1.000 | 4649 | tags=53%, list=39%, signal=86% |
| 6064 | GOBP\_ESTABLISHMENT\_OF\_GOLGI\_LOCALIZATION |  | 7 | -0.41 | -0.88 | 0.653 | 0.872 | 1.000 | 3115 | tags=29%, list=26%, signal=39% |
| 6065 | GOBP\_AGGRESOME\_ASSEMBLY |  | 3 | -0.53 | -0.88 | 0.679 | 0.871 | 1.000 | 5680 | tags=100%, list=47%, signal=190% |
| 6066 | GOBP\_REGULATION\_OF\_ER\_ASSOCIATED\_UBIQUITIN\_DEPENDENT\_PROTEIN\_CATABOLIC\_PROCESS |  | 8 | -0.40 | -0.88 | 0.650 | 0.872 | 1.000 | 1587 | tags=25%, list=13%, signal=29% |
| 6067 | GOBP\_CELL\_CELL\_RECOGNITION |  | 30 | -0.29 | -0.88 | 0.658 | 0.872 | 1.000 | 2604 | tags=20%, list=22%, signal=25% |
| 6068 | GOBP\_RECEPTOR\_METABOLIC\_PROCESS |  | 118 | -0.24 | -0.88 | 0.732 | 0.872 | 1.000 | 4803 | tags=44%, list=40%, signal=73% |
| 6069 | GOBP\_RESPONSE\_TO\_COCAINE |  | 28 | -0.29 | -0.88 | 0.661 | 0.872 | 1.000 | 2829 | tags=29%, list=24%, signal=37% |
| 6070 | GOBP\_METANEPHRIC\_COLLECTING\_DUCT\_DEVELOPMENT |  | 7 | -0.42 | -0.88 | 0.651 | 0.872 | 1.000 | 4111 | tags=71%, list=34%, signal=109% |
| 6071 | GOBP\_KERATAN\_SULFATE\_BIOSYNTHETIC\_PROCESS |  | 20 | -0.31 | -0.88 | 0.650 | 0.872 | 1.000 | 1968 | tags=15%, list=16%, signal=18% |
| 6072 | GOBP\_OXYGEN\_TRANSPORT |  | 5 | -0.45 | -0.88 | 0.659 | 0.873 | 1.000 | 662 | tags=20%, list=6%, signal=21% |
| 6073 | GOBP\_SOMATIC\_STEM\_CELL\_POPULATION\_MAINTENANCE |  | 46 | -0.27 | -0.88 | 0.676 | 0.873 | 1.000 | 5080 | tags=46%, list=42%, signal=79% |
| 6074 | GOBP\_PROTEIN\_LOCALIZATION\_TO\_GOLGI\_APPARATUS |  | 23 | -0.31 | -0.88 | 0.656 | 0.873 | 1.000 | 674 | tags=13%, list=6%, signal=14% |
| 6075 | GOBP\_NEGATIVE\_REGULATION\_OF\_SODIUM\_ION\_TRANSPORT |  | 14 | -0.34 | -0.88 | 0.648 | 0.873 | 1.000 | 4140 | tags=57%, list=34%, signal=87% |
| 6076 | GOBP\_REGULATION\_OF\_LENS\_FIBER\_CELL\_DIFFERENTIATION |  | 4 | -0.48 | -0.88 | 0.672 | 0.873 | 1.000 | 3530 | tags=75%, list=29%, signal=106% |
| 6077 | GOBP\_RESPONSE\_TO\_ISOLATION\_STRESS |  | 4 | -0.48 | -0.88 | 0.678 | 0.873 | 1.000 | 448 | tags=25%, list=4%, signal=26% |
| 6078 | GOBP\_ORGANELLE\_FUSION |  | 109 | -0.24 | -0.88 | 0.731 | 0.873 | 1.000 | 3698 | tags=32%, list=31%, signal=46% |
| 6079 | GOBP\_PROTEIN\_CONTAINING\_COMPLEX\_DISASSEMBLY |  | 254 | -0.23 | -0.88 | 0.799 | 0.873 | 1.000 | 3148 | tags=24%, list=26%, signal=32% |
| 6080 | GOCC\_CLATHRIN\_SCULPTED\_VESICLE |  | 7 | -0.41 | -0.88 | 0.659 | 0.874 | 1.000 | 2055 | tags=43%, list=17%, signal=52% |
| 6081 | GOBP\_PROTEIN\_TARGETING\_TO\_VACUOLE |  | 36 | -0.28 | -0.88 | 0.672 | 0.874 | 1.000 | 4280 | tags=42%, list=36%, signal=65% |
| 6082 | GOMF\_BIOTIN\_BINDING |  | 4 | -0.48 | -0.88 | 0.668 | 0.874 | 1.000 | 2808 | tags=50%, list=23%, signal=65% |
| 6083 | GOBP\_REGULATION\_OF\_NUCLEAR\_CELL\_CYCLE\_DNA\_REPLICATION |  | 13 | -0.35 | -0.88 | 0.641 | 0.874 | 1.000 | 5292 | tags=62%, list=44%, signal=110% |
| 6084 | GOBP\_MITOCHONDRIAL\_DNA\_REPLICATION |  | 11 | -0.37 | -0.88 | 0.642 | 0.874 | 1.000 | 4773 | tags=55%, list=40%, signal=90% |
| 6085 | GOBP\_CELLULAR\_RESPONSE\_TO\_ALKALOID |  | 19 | -0.32 | -0.88 | 0.661 | 0.874 | 1.000 | 3778 | tags=47%, list=31%, signal=69% |
| 6086 | GOBP\_CILIUM\_ORGANIZATION |  | 261 | -0.23 | -0.88 | 0.811 | 0.874 | 1.000 | 3612 | tags=28%, list=30%, signal=40% |
| 6087 | GOBP\_ADENOSINE\_METABOLIC\_PROCESS |  | 6 | -0.43 | -0.88 | 0.652 | 0.874 | 1.000 | 6860 | tags=100%, list=57%, signal=233% |
| 6088 | GOBP\_REGULATION\_OF\_CYCLIC\_NUCLEOTIDE\_PHOSPHODIESTERASE\_ACTIVITY |  | 7 | -0.41 | -0.88 | 0.662 | 0.874 | 1.000 | 4627 | tags=43%, list=39%, signal=70% |
| 6089 | GOBP\_EYE\_PHOTORECEPTOR\_CELL\_DIFFERENTIATION |  | 22 | -0.31 | -0.87 | 0.653 | 0.875 | 1.000 | 3868 | tags=41%, list=32%, signal=60% |
| 6090 | GOMF\_MUTSALPHA\_COMPLEX\_BINDING |  | 6 | -0.43 | -0.87 | 0.660 | 0.875 | 1.000 | 5678 | tags=83%, list=47%, signal=158% |
| 6091 | GOBP\_PHAGOLYSOSOME\_ASSEMBLY |  | 11 | -0.36 | -0.87 | 0.641 | 0.875 | 1.000 | 1954 | tags=27%, list=16%, signal=33% |
| 6092 | GOBP\_MEMBRANE\_TO\_MEMBRANE\_DOCKING |  | 3 | -0.52 | -0.87 | 0.681 | 0.875 | 1.000 | 5744 | tags=100%, list=48%, signal=192% |
| 6093 | GOBP\_RESPONSE\_TO\_MISFOLDED\_PROTEIN |  | 17 | -0.33 | -0.87 | 0.648 | 0.875 | 1.000 | 5182 | tags=53%, list=43%, signal=93% |
| 6094 | GOBP\_INOSITOL\_PHOSPHATE\_BIOSYNTHETIC\_PROCESS |  | 18 | -0.32 | -0.87 | 0.647 | 0.875 | 1.000 | 4916 | tags=50%, list=41%, signal=85% |
| 6095 | GOBP\_CELLULAR\_RESPONSE\_TO\_OXYGEN\_LEVELS |  | 170 | -0.23 | -0.87 | 0.765 | 0.875 | 1.000 | 3701 | tags=29%, list=31%, signal=42% |
| 6096 | GOMF\_NF\_KAPPAB\_BINDING |  | 24 | -0.30 | -0.87 | 0.663 | 0.875 | 1.000 | 2702 | tags=25%, list=22%, signal=32% |
| 6097 | GOCC\_I\_BAND |  | 89 | -0.25 | -0.87 | 0.716 | 0.875 | 1.000 | 3345 | tags=26%, list=28%, signal=36% |
| 6098 | GOBP\_CELLULAR\_RESPONSE\_TO\_COPPER\_ION |  | 11 | -0.37 | -0.87 | 0.657 | 0.876 | 1.000 | 1782 | tags=18%, list=15%, signal=21% |
| 6099 | GOMF\_TRANSCRIPTION\_COREGULATOR\_BINDING |  | 45 | -0.27 | -0.87 | 0.679 | 0.876 | 1.000 | 2708 | tags=22%, list=23%, signal=29% |
| 6100 | GOBP\_NEGATIVE\_REGULATION\_OF\_SMOOTH\_MUSCLE\_CELL\_MIGRATION |  | 16 | -0.33 | -0.87 | 0.652 | 0.876 | 1.000 | 3007 | tags=31%, list=25%, signal=42% |
| 6101 | GOCC\_POLAR\_MICROTUBULE |  | 5 | -0.45 | -0.87 | 0.667 | 0.876 | 1.000 | 2265 | tags=20%, list=19%, signal=25% |
| 6102 | GOMF\_THYROID\_HORMONE\_TRANSMEMBRANE\_TRANSPORTER\_ACTIVITY |  | 4 | -0.49 | -0.87 | 0.687 | 0.876 | 1.000 | 3155 | tags=25%, list=26%, signal=34% |
| 6103 | GOCC\_NEURON\_PROJECTION\_MEMBRANE |  | 26 | -0.30 | -0.87 | 0.656 | 0.876 | 1.000 | 790 | tags=15%, list=7%, signal=16% |
| 6104 | GOBP\_CALCIUM\_ION\_REGULATED\_EXOCYTOSIS\_OF\_NEUROTRANSMITTER |  | 12 | -0.35 | -0.87 | 0.657 | 0.876 | 1.000 | 2024 | tags=33%, list=17%, signal=40% |
| 6105 | GOBP\_ICOSANOID\_METABOLIC\_PROCESS |  | 62 | -0.26 | -0.87 | 0.706 | 0.875 | 1.000 | 3299 | tags=39%, list=27%, signal=53% |
| 6106 | GOMF\_MONOOXYGENASE\_ACTIVITY |  | 44 | -0.27 | -0.87 | 0.679 | 0.876 | 1.000 | 3078 | tags=30%, list=26%, signal=40% |
| 6107 | GOCC\_NUCLEAR\_OUTER\_MEMBRANE\_ENDOPLASMIC\_RETICULUM\_MEMBRANE\_NETWORK |  | 782 | -0.22 | -0.87 | 0.930 | 0.876 | 1.000 | 4239 | tags=34%, list=35%, signal=49% |
| 6108 | GOBP\_CELLULAR\_RESPONSE\_TO\_UV |  | 66 | -0.25 | -0.87 | 0.704 | 0.876 | 1.000 | 2656 | tags=23%, list=22%, signal=29% |
| 6109 | GOBP\_DE\_NOVO\_PYRIMIDINE\_NUCLEOBASE\_BIOSYNTHETIC\_PROCESS |  | 6 | -0.43 | -0.87 | 0.666 | 0.876 | 1.000 | 1743 | tags=17%, list=15%, signal=19% |
| 6110 | GOMF\_COMPLEMENT\_COMPONENT\_C1Q\_COMPLEX\_BINDING |  | 6 | -0.43 | -0.87 | 0.668 | 0.876 | 1.000 | 1126 | tags=17%, list=9%, signal=18% |
| 6111 | GOCC\_GOLGI\_MEDIAL\_CISTERNA |  | 8 | -0.40 | -0.87 | 0.652 | 0.876 | 1.000 | 147 | tags=13%, list=1%, signal=13% |
| 6112 | GOBP\_REGULATION\_OF\_HISTONE\_H3\_K9\_ACETYLATION |  | 6 | -0.43 | -0.87 | 0.670 | 0.876 | 1.000 | 3378 | tags=50%, list=28%, signal=70% |
| 6113 | GOBP\_PREASSEMBLY\_OF\_GPI\_ANCHOR\_IN\_ER\_MEMBRANE |  | 14 | -0.34 | -0.87 | 0.653 | 0.876 | 1.000 | 5828 | tags=57%, list=49%, signal=111% |
| 6114 | GOBP\_NEGATIVE\_REGULATION\_OF\_BLOOD\_VESSEL\_ENDOTHELIAL\_CELL\_PROLIFERATION\_INVOLVED\_IN\_SPROUTING\_ANGIOGENESIS |  | 4 | -0.48 | -0.87 | 0.683 | 0.876 | 1.000 | 2820 | tags=50%, list=23%, signal=65% |
| 6115 | GOMF\_GENERAL\_TRANSCRIPTION\_INITIATION\_FACTOR\_ACTIVITY |  | 33 | -0.28 | -0.87 | 0.674 | 0.876 | 1.000 | 3287 | tags=24%, list=27%, signal=33% |
| 6116 | GOCC\_COATED\_VESICLE |  | 200 | -0.23 | -0.87 | 0.792 | 0.876 | 1.000 | 4295 | tags=35%, list=36%, signal=53% |
| 6117 | GOBP\_FATTY\_ACID\_OMEGA\_OXIDATION |  | 3 | -0.52 | -0.87 | 0.683 | 0.876 | 1.000 | 3078 | tags=67%, list=26%, signal=90% |
| 6118 | GOBP\_RESPONSE\_TO\_INTERLEUKIN\_4 |  | 23 | -0.31 | -0.87 | 0.665 | 0.877 | 1.000 | 3692 | tags=39%, list=31%, signal=56% |
| 6119 | GOBP\_NEGATIVE\_REGULATION\_OF\_EXCITATORY\_POSTSYNAPTIC\_POTENTIAL |  | 7 | -0.41 | -0.87 | 0.661 | 0.877 | 1.000 | 5829 | tags=57%, list=49%, signal=111% |
| 6120 | GOCC\_TERTIARY\_GRANULE |  | 98 | -0.24 | -0.87 | 0.735 | 0.877 | 1.000 | 4398 | tags=40%, list=37%, signal=62% |
| 6121 | GOBP\_RESPONSE\_TO\_OXYGEN\_GLUCOSE\_DEPRIVATION |  | 6 | -0.43 | -0.87 | 0.663 | 0.877 | 1.000 | 139 | tags=17%, list=1%, signal=17% |
| 6122 | GOBP\_NCRNA\_TRANSCRIPTION |  | 89 | -0.25 | -0.87 | 0.734 | 0.878 | 1.000 | 5934 | tags=49%, list=49%, signal=97% |
| 6123 | GOCC\_APICAL\_CORTEX |  | 6 | -0.43 | -0.87 | 0.668 | 0.878 | 1.000 | 159 | tags=17%, list=1%, signal=17% |
| 6124 | GOBP\_SYNAPTIC\_VESICLE\_CLUSTERING |  | 7 | -0.41 | -0.87 | 0.664 | 0.878 | 1.000 | 3946 | tags=71%, list=33%, signal=106% |
| 6125 | GOBP\_NEGATIVE\_REGULATION\_OF\_EXOCYTOSIS |  | 20 | -0.31 | -0.87 | 0.665 | 0.878 | 1.000 | 1608 | tags=15%, list=13%, signal=17% |
| 6126 | GOBP\_CHEMICAL\_HOMEOSTASIS\_WITHIN\_A\_TISSUE |  | 9 | -0.38 | -0.87 | 0.654 | 0.878 | 1.000 | 3196 | tags=44%, list=27%, signal=61% |
| 6127 | GOMF\_PEPTIDE\_ANTIGEN\_BINDING |  | 6 | -0.43 | -0.87 | 0.674 | 0.878 | 1.000 | 848 | tags=33%, list=7%, signal=36% |
| 6128 | GOCC\_LUMENAL\_SIDE\_OF\_MEMBRANE |  | 13 | -0.34 | -0.87 | 0.660 | 0.878 | 1.000 | 1793 | tags=31%, list=15%, signal=36% |
| 6129 | GOMF\_GLYCOLIPID\_MANNOSYLTRANSFERASE\_ACTIVITY |  | 5 | -0.45 | -0.87 | 0.666 | 0.878 | 1.000 | 888 | tags=20%, list=7%, signal=22% |
| 6130 | GOMF\_ROUNDABOUT\_BINDING |  | 5 | -0.45 | -0.87 | 0.678 | 0.878 | 1.000 | 5496 | tags=60%, list=46%, signal=111% |
| 6131 | GOBP\_MULTI\_ORGANISM\_CELLULAR\_PROCESS |  | 13 | -0.35 | -0.87 | 0.652 | 0.878 | 1.000 | 2227 | tags=23%, list=19%, signal=28% |
| 6132 | GOCC\_COATED\_VESICLE\_MEMBRANE |  | 122 | -0.24 | -0.87 | 0.754 | 0.878 | 1.000 | 4424 | tags=39%, list=37%, signal=60% |
| 6133 | GOBP\_CARBOHYDRATE\_DERIVATIVE\_BIOSYNTHETIC\_PROCESS |  | 468 | -0.22 | -0.87 | 0.878 | 0.878 | 1.000 | 4433 | tags=37%, list=37%, signal=56% |
| 6134 | GOBP\_POSITIVE\_REGULATION\_OF\_RESPONSE\_TO\_CYTOKINE\_STIMULUS |  | 36 | -0.28 | -0.87 | 0.685 | 0.878 | 1.000 | 3615 | tags=36%, list=30%, signal=52% |
| 6135 | GOCC\_COP9\_SIGNALOSOME |  | 26 | -0.30 | -0.87 | 0.669 | 0.879 | 1.000 | 2608 | tags=23%, list=22%, signal=29% |
| 6136 | GOBP\_SPINAL\_CORD\_PATTERNING |  | 12 | -0.35 | -0.87 | 0.657 | 0.879 | 1.000 | 1871 | tags=25%, list=16%, signal=30% |
| 6137 | GOBP\_POSITIVE\_REGULATION\_OF\_DENDRITIC\_CELL\_CHEMOTAXIS |  | 5 | -0.45 | -0.87 | 0.671 | 0.879 | 1.000 | 1715 | tags=20%, list=14%, signal=23% |
| 6138 | GOMF\_HYDROLASE\_ACTIVITY\_ACTING\_ON\_CARBON\_NITROGEN\_BUT\_NOT\_PEPTIDE\_BONDS |  | 78 | -0.25 | -0.87 | 0.727 | 0.879 | 1.000 | 3710 | tags=31%, list=31%, signal=44% |
| 6139 | GOBP\_CELLULAR\_TRIVALENT\_INORGANIC\_ANION\_HOMEOSTASIS |  | 3 | -0.52 | -0.87 | 0.686 | 0.879 | 1.000 | 1302 | tags=33%, list=11%, signal=37% |
| 6140 | GOBP\_NEGATIVE\_REGULATION\_OF\_COMPLEMENT\_ACTIVATION |  | 5 | -0.45 | -0.87 | 0.683 | 0.879 | 1.000 | 1808 | tags=40%, list=15%, signal=47% |
| 6141 | GOBP\_REGULATION\_OF\_SMALL\_MOLECULE\_METABOLIC\_PROCESS |  | 309 | -0.22 | -0.87 | 0.838 | 0.879 | 1.000 | 3403 | tags=28%, list=28%, signal=37% |
| 6142 | GOBP\_NEGATIVE\_REGULATION\_OF\_CAMP\_MEDIATED\_SIGNALING |  | 11 | -0.36 | -0.87 | 0.663 | 0.879 | 1.000 | 1914 | tags=27%, list=16%, signal=32% |
| 6143 | GOBP\_PUTRESCINE\_METABOLIC\_PROCESS |  | 7 | -0.41 | -0.87 | 0.660 | 0.879 | 1.000 | 5093 | tags=57%, list=42%, signal=99% |
| 6144 | GOBP\_POSITIVE\_REGULATION\_OF\_NITRIC\_OXIDE\_SYNTHASE\_ACTIVITY |  | 18 | -0.32 | -0.87 | 0.672 | 0.880 | 1.000 | 4846 | tags=50%, list=40%, signal=84% |
| 6145 | GOMF\_UREA\_TRANSMEMBRANE\_TRANSPORTER\_ACTIVITY |  | 3 | -0.52 | -0.87 | 0.691 | 0.879 | 1.000 | 2778 | tags=33%, list=23%, signal=43% |
| 6146 | GOBP\_CELLULAR\_RESPONSE\_TO\_LEPTIN\_STIMULUS |  | 15 | -0.33 | -0.87 | 0.664 | 0.879 | 1.000 | 3392 | tags=40%, list=28%, signal=56% |
| 6147 | GOCC\_BETA\_CATENIN\_DESTRUCTION\_COMPLEX |  | 11 | -0.36 | -0.87 | 0.648 | 0.879 | 1.000 | 2811 | tags=27%, list=23%, signal=36% |
| 6148 | GOBP\_CHEMOREPULSION\_OF\_AXON |  | 4 | -0.48 | -0.87 | 0.685 | 0.879 | 1.000 | 223 | tags=25%, list=2%, signal=25% |
| 6149 | GOBP\_SPHINGOLIPID\_METABOLIC\_PROCESS |  | 123 | -0.24 | -0.87 | 0.751 | 0.879 | 1.000 | 4711 | tags=46%, list=39%, signal=74% |
| 6150 | GOCC\_N\_TERMINAL\_PROTEIN\_ACETYLTRANSFERASE\_COMPLEX |  | 8 | -0.39 | -0.87 | 0.675 | 0.880 | 1.000 | 4976 | tags=50%, list=41%, signal=85% |
| 6151 | GOBP\_NEGATIVE\_REGULATION\_OF\_PROTEIN\_CATABOLIC\_PROCESS |  | 91 | -0.24 | -0.87 | 0.735 | 0.880 | 1.000 | 1724 | tags=16%, list=14%, signal=19% |
| 6152 | GOBP\_PURINE\_CONTAINING\_COMPOUND\_METABOLIC\_PROCESS |  | 305 | -0.22 | -0.87 | 0.844 | 0.880 | 1.000 | 4317 | tags=39%, list=36%, signal=59% |
| 6153 | GOBP\_NEGATIVE\_REGULATION\_OF\_EXECUTION\_PHASE\_OF\_APOPTOSIS |  | 8 | -0.39 | -0.87 | 0.654 | 0.880 | 1.000 | 1070 | tags=25%, list=9%, signal=27% |
| 6154 | GOBP\_REGULATION\_OF\_INCLUSION\_BODY\_ASSEMBLY |  | 10 | -0.37 | -0.87 | 0.663 | 0.880 | 1.000 | 3345 | tags=50%, list=28%, signal=69% |
| 6155 | GOMF\_SULFUR\_COMPOUND\_TRANSMEMBRANE\_TRANSPORTER\_ACTIVITY |  | 36 | -0.28 | -0.87 | 0.696 | 0.880 | 1.000 | 2884 | tags=36%, list=24%, signal=47% |
| 6156 | GOBP\_RESPONSE\_TO\_ALKALOID |  | 67 | -0.25 | -0.87 | 0.716 | 0.880 | 1.000 | 4217 | tags=42%, list=35%, signal=64% |
| 6157 | GOBP\_CORTISOL\_METABOLIC\_PROCESS |  | 7 | -0.41 | -0.87 | 0.658 | 0.880 | 1.000 | 1899 | tags=43%, list=16%, signal=51% |
| 6158 | GOBP\_SEROTONIN\_SECRETION |  | 6 | -0.43 | -0.87 | 0.676 | 0.880 | 1.000 | 3708 | tags=67%, list=31%, signal=96% |
| 6159 | GOBP\_DEFENSE\_RESPONSE\_TO\_GRAM\_POSITIVE\_BACTERIUM |  | 29 | -0.29 | -0.87 | 0.677 | 0.880 | 1.000 | 3344 | tags=31%, list=28%, signal=43% |
| 6160 | GOBP\_NUCLEAR\_MEMBRANE\_ORGANIZATION |  | 13 | -0.34 | -0.87 | 0.664 | 0.880 | 1.000 | 2032 | tags=23%, list=17%, signal=28% |
| 6161 | GOBP\_CRD\_MEDIATED\_MRNA\_STABILIZATION |  | 4 | -0.48 | -0.87 | 0.697 | 0.880 | 1.000 | 6262 | tags=100%, list=52%, signal=209% |
| 6162 | GOBP\_TOXIN\_METABOLIC\_PROCESS |  | 8 | -0.39 | -0.87 | 0.658 | 0.881 | 1.000 | 25 | tags=13%, list=0%, signal=13% |
| 6163 | GOMF\_VITAMIN\_D\_RECEPTOR\_BINDING |  | 15 | -0.33 | -0.87 | 0.660 | 0.881 | 1.000 | 5972 | tags=73%, list=50%, signal=146% |
| 6164 | GOMF\_PROTEIN\_SERINE\_PHOSPHATASE\_ACTIVITY |  | 56 | -0.26 | -0.87 | 0.707 | 0.881 | 1.000 | 3447 | tags=30%, list=29%, signal=42% |
| 6165 | GOBP\_POSITIVE\_REGULATION\_OF\_DOUBLE\_STRAND\_BREAK\_REPAIR\_VIA\_NONHOMOLOGOUS\_END\_JOINING |  | 11 | -0.36 | -0.87 | 0.661 | 0.881 | 1.000 | 702 | tags=18%, list=6%, signal=19% |
| 6166 | GOBP\_WATER\_SOLUBLE\_VITAMIN\_METABOLIC\_PROCESS |  | 58 | -0.26 | -0.87 | 0.711 | 0.881 | 1.000 | 2950 | tags=31%, list=25%, signal=41% |
| 6167 | GOBP\_SPINAL\_CORD\_MOTOR\_NEURON\_CELL\_FATE\_SPECIFICATION |  | 6 | -0.43 | -0.87 | 0.675 | 0.881 | 1.000 | 556 | tags=17%, list=5%, signal=17% |
| 6168 | GOBP\_NEGATIVE\_REGULATION\_OF\_TRANSPORT |  | 267 | -0.22 | -0.87 | 0.831 | 0.881 | 1.000 | 4177 | tags=33%, list=35%, signal=50% |
| 6169 | GOBP\_FIBROBLAST\_PROLIFERATION |  | 58 | -0.26 | -0.86 | 0.713 | 0.882 | 1.000 | 3331 | tags=36%, list=28%, signal=50% |
| 6170 | GOBP\_UBIQUITIN\_DEPENDENT\_ERAD\_PATHWAY |  | 62 | -0.26 | -0.86 | 0.710 | 0.882 | 1.000 | 5704 | tags=47%, list=47%, signal=89% |
| 6171 | GOBP\_ALDEHYDE\_BIOSYNTHETIC\_PROCESS |  | 10 | -0.37 | -0.86 | 0.665 | 0.882 | 1.000 | 4894 | tags=70%, list=41%, signal=118% |
| 6172 | GOBP\_REGULATION\_OF\_PEROXISOME\_PROLIFERATOR\_ACTIVATED\_RECEPTOR\_SIGNALING\_PATHWAY |  | 8 | -0.39 | -0.86 | 0.674 | 0.882 | 1.000 | 4853 | tags=63%, list=40%, signal=105% |
| 6173 | GOBP\_POSITIVE\_REGULATION\_OF\_NUCLEASE\_ACTIVITY |  | 7 | -0.41 | -0.86 | 0.671 | 0.882 | 1.000 | 6931 | tags=71%, list=58%, signal=169% |
| 6174 | GOBP\_GROWTH\_PLATE\_CARTILAGE\_DEVELOPMENT |  | 9 | -0.38 | -0.86 | 0.663 | 0.882 | 1.000 | 6131 | tags=67%, list=51%, signal=136% |
| 6175 | GOMF\_MRF\_BINDING |  | 5 | -0.45 | -0.86 | 0.680 | 0.882 | 1.000 | 4170 | tags=40%, list=35%, signal=61% |
| 6176 | GOBP\_REGULATION\_OF\_NEURON\_PROJECTION\_REGENERATION |  | 24 | -0.30 | -0.86 | 0.670 | 0.883 | 1.000 | 3392 | tags=29%, list=28%, signal=41% |
| 6177 | GOBP\_INTRINSIC\_APOPTOTIC\_SIGNALING\_PATHWAY\_BY\_P53\_CLASS\_MEDIATOR |  | 48 | -0.26 | -0.86 | 0.707 | 0.882 | 1.000 | 3278 | tags=29%, list=27%, signal=40% |
| 6178 | GOMF\_HISTONE\_METHYLTRANSFERASE\_ACTIVITY\_H3\_K9\_SPECIFIC |  | 8 | -0.39 | -0.86 | 0.672 | 0.882 | 1.000 | 1243 | tags=25%, list=10%, signal=28% |
| 6179 | GOBP\_STEROID\_ESTERIFICATION |  | 10 | -0.37 | -0.86 | 0.668 | 0.882 | 1.000 | 68 | tags=10%, list=1%, signal=10% |
| 6180 | GOBP\_TRANSCRIPTION\_FACTOR\_CATABOLIC\_PROCESS |  | 4 | -0.48 | -0.86 | 0.696 | 0.882 | 1.000 | 1174 | tags=25%, list=10%, signal=28% |
| 6181 | GOBP\_PROTEIN\_STABILIZATION |  | 136 | -0.23 | -0.86 | 0.768 | 0.883 | 1.000 | 4527 | tags=33%, list=38%, signal=53% |
| 6182 | GOBP\_REGULATION\_OF\_THYROID\_HORMONE\_MEDIATED\_SIGNALING\_PATHWAY |  | 3 | -0.51 | -0.86 | 0.698 | 0.883 | 1.000 | 4001 | tags=67%, list=33%, signal=100% |
| 6183 | GOBP\_CHONDROITIN\_SULFATE\_BIOSYNTHETIC\_PROCESS |  | 20 | -0.31 | -0.86 | 0.668 | 0.883 | 1.000 | 1256 | tags=20%, list=10%, signal=22% |
| 6184 | GOBP\_NK\_T\_CELL\_PROLIFERATION |  | 3 | -0.52 | -0.86 | 0.696 | 0.883 | 1.000 | 1566 | tags=33%, list=13%, signal=38% |
| 6185 | GOBP\_NEGATIVE\_REGULATION\_OF\_STEM\_CELL\_POPULATION\_MAINTENANCE |  | 3 | -0.52 | -0.86 | 0.684 | 0.883 | 1.000 | 1723 | tags=33%, list=14%, signal=39% |
| 6186 | GOBP\_POSITIVE\_REGULATION\_OF\_PROTEIN\_BINDING |  | 66 | -0.25 | -0.86 | 0.719 | 0.883 | 1.000 | 4993 | tags=41%, list=42%, signal=70% |
| 6187 | GOBP\_NUCLEIC\_ACID\_PHOSPHODIESTER\_BOND\_HYDROLYSIS |  | 205 | -0.23 | -0.86 | 0.812 | 0.883 | 1.000 | 5351 | tags=45%, list=45%, signal=80% |
| 6188 | GOCC\_BASAL\_PART\_OF\_CELL |  | 164 | -0.23 | -0.86 | 0.790 | 0.883 | 1.000 | 4188 | tags=38%, list=35%, signal=57% |
| 6189 | GOMF\_INTRAMOLECULAR\_TRANSFERASE\_ACTIVITY\_PHOSPHOTRANSFERASES |  | 8 | -0.39 | -0.86 | 0.668 | 0.883 | 1.000 | 4255 | tags=63%, list=35%, signal=97% |
| 6190 | GOBP\_POSITIVE\_REGULATION\_OF\_AUTOPHAGY\_OF\_MITOCHONDRION |  | 8 | -0.39 | -0.86 | 0.667 | 0.883 | 1.000 | 3080 | tags=38%, list=26%, signal=50% |
| 6191 | GOBP\_MITOCHONDRIAL\_CALCIUM\_ION\_HOMEOSTASIS |  | 17 | -0.32 | -0.86 | 0.669 | 0.883 | 1.000 | 397 | tags=12%, list=3%, signal=12% |
| 6192 | GOCC\_DESMOSOME |  | 15 | -0.33 | -0.86 | 0.663 | 0.883 | 1.000 | 4880 | tags=47%, list=41%, signal=79% |
| 6193 | GOBP\_RESPONSE\_TO\_MUSCLE\_ACTIVITY |  | 18 | -0.32 | -0.86 | 0.668 | 0.883 | 1.000 | 2070 | tags=28%, list=17%, signal=34% |
| 6194 | GOBP\_AMP\_BIOSYNTHETIC\_PROCESS |  | 5 | -0.45 | -0.86 | 0.681 | 0.883 | 1.000 | 6275 | tags=80%, list=52%, signal=167% |
| 6195 | GOMF\_EXTRACELLULARLY\_ATP\_GATED\_CATION\_CHANNEL\_ACTIVITY |  | 6 | -0.43 | -0.86 | 0.677 | 0.883 | 1.000 | 374 | tags=17%, list=3%, signal=17% |
| 6196 | GOBP\_POSITIVE\_REGULATION\_OF\_TELOMERASE\_ACTIVITY |  | 27 | -0.29 | -0.86 | 0.683 | 0.883 | 1.000 | 2010 | tags=22%, list=17%, signal=27% |
| 6197 | GOBP\_POSITIVE\_REGULATION\_OF\_VIRAL\_PROCESS |  | 72 | -0.25 | -0.86 | 0.735 | 0.884 | 1.000 | 3520 | tags=29%, list=29%, signal=41% |
| 6198 | GOBP\_L\_LYSINE\_TRANSMEMBRANE\_TRANSPORT |  | 4 | -0.48 | -0.86 | 0.691 | 0.884 | 1.000 | 1025 | tags=25%, list=9%, signal=27% |
| 6199 | GOBP\_EPINEPHRINE\_TRANSPORT |  | 6 | -0.42 | -0.86 | 0.684 | 0.884 | 1.000 | 2392 | tags=33%, list=20%, signal=42% |
| 6200 | GOMF\_L\_LYSINE\_TRANSMEMBRANE\_TRANSPORTER\_ACTIVITY |  | 4 | -0.48 | -0.86 | 0.692 | 0.884 | 1.000 | 1025 | tags=25%, list=9%, signal=27% |
| 6201 | GOMF\_PROTEIN\_BINDING\_INVOLVED\_IN\_HETEROTYPIC\_CELL\_CELL\_ADHESION |  | 7 | -0.40 | -0.86 | 0.671 | 0.884 | 1.000 | 3918 | tags=43%, list=33%, signal=64% |
| 6202 | GOBP\_NEGATIVE\_REGULATION\_OF\_ACTIVIN\_RECEPTOR\_SIGNALING\_PATHWAY |  | 8 | -0.39 | -0.86 | 0.672 | 0.884 | 1.000 | 1297 | tags=25%, list=11%, signal=28% |
| 6203 | GOBP\_ERBB2\_SIGNALING\_PATHWAY |  | 26 | -0.29 | -0.86 | 0.690 | 0.884 | 1.000 | 3308 | tags=38%, list=28%, signal=53% |
| 6204 | GOBP\_UDP\_N\_ACETYLGLUCOSAMINE\_TRANSMEMBRANE\_TRANSPORT |  | 3 | -0.52 | -0.86 | 0.698 | 0.884 | 1.000 | 1556 | tags=33%, list=13%, signal=38% |
| 6205 | GOBP\_DE\_NOVO\_PROTEIN\_FOLDING |  | 32 | -0.28 | -0.86 | 0.689 | 0.884 | 1.000 | 2394 | tags=22%, list=20%, signal=27% |
| 6206 | GOBP\_NEGATIVE\_REGULATION\_OF\_ASTROCYTE\_DIFFERENTIATION |  | 5 | -0.45 | -0.86 | 0.680 | 0.884 | 1.000 | 507 | tags=20%, list=4%, signal=21% |
| 6207 | GOBP\_GROWTH\_PLATE\_CARTILAGE\_CHONDROCYTE\_DEVELOPMENT |  | 4 | -0.48 | -0.86 | 0.701 | 0.884 | 1.000 | 5967 | tags=75%, list=50%, signal=149% |
| 6208 | GOMF\_ISOPEPTIDASE\_ACTIVITY |  | 15 | -0.33 | -0.86 | 0.675 | 0.884 | 1.000 | 3762 | tags=33%, list=31%, signal=48% |
| 6209 | GOBP\_POSITIVE\_REGULATION\_OF\_TRANSCRIPTION\_FROM\_RNA\_POLYMERASE\_II\_PROMOTER\_IN\_RESPONSE\_TO\_STRESS |  | 18 | -0.31 | -0.86 | 0.677 | 0.885 | 1.000 | 3841 | tags=39%, list=32%, signal=57% |
| 6210 | GOCC\_DNA\_RECOMBINASE\_MEDIATOR\_COMPLEX |  | 6 | -0.42 | -0.86 | 0.680 | 0.884 | 1.000 | 5752 | tags=67%, list=48%, signal=128% |
| 6211 | GOBP\_REGULATION\_OF\_GASTRULATION |  | 13 | -0.34 | -0.86 | 0.667 | 0.885 | 1.000 | 3551 | tags=31%, list=30%, signal=44% |
| 6212 | GOBP\_ENDOPLASMIC\_RETICULUM\_TO\_GOLGI\_VESICLE\_MEDIATED\_TRANSPORT |  | 163 | -0.23 | -0.86 | 0.792 | 0.885 | 1.000 | 5609 | tags=49%, list=47%, signal=91% |
| 6213 | GOBP\_GLUCOCORTICOID\_BIOSYNTHETIC\_PROCESS |  | 8 | -0.39 | -0.86 | 0.668 | 0.885 | 1.000 | 1899 | tags=38%, list=16%, signal=45% |
| 6214 | GOBP\_FEMALE\_MEIOSIS\_CHROMOSOME\_SEGREGATION |  | 5 | -0.44 | -0.86 | 0.690 | 0.885 | 1.000 | 6682 | tags=100%, list=56%, signal=225% |
| 6215 | GOBP\_RESPONSE\_TO\_HYPEROXIA |  | 16 | -0.33 | -0.86 | 0.675 | 0.886 | 1.000 | 3675 | tags=44%, list=31%, signal=63% |
| 6216 | GOMF\_UDP\_N\_ACETYLGLUCOSAMINE\_TRANSMEMBRANE\_TRANSPORTER\_ACTIVITY |  | 3 | -0.52 | -0.86 | 0.704 | 0.886 | 1.000 | 1556 | tags=33%, list=13%, signal=38% |
| 6217 | GOBP\_COLLECTING\_DUCT\_DEVELOPMENT |  | 9 | -0.38 | -0.86 | 0.674 | 0.886 | 1.000 | 4111 | tags=56%, list=34%, signal=84% |
| 6218 | GOBP\_LEUCINE\_CATABOLIC\_PROCESS |  | 6 | -0.42 | -0.86 | 0.681 | 0.886 | 1.000 | 6936 | tags=100%, list=58%, signal=237% |
| 6219 | GOBP\_EPITHELIAL\_TO\_MESENCHYMAL\_TRANSITION\_INVOLVED\_IN\_ENDOCARDIAL\_CUSHION\_FORMATION |  | 12 | -0.35 | -0.86 | 0.675 | 0.886 | 1.000 | 5879 | tags=58%, list=49%, signal=114% |
| 6220 | GOBP\_ENDOSOMAL\_TRANSPORT |  | 186 | -0.23 | -0.86 | 0.804 | 0.886 | 1.000 | 3483 | tags=27%, list=29%, signal=38% |
| 6221 | GOBP\_RESPONSE\_TO\_MAGNESIUM\_ION |  | 11 | -0.36 | -0.86 | 0.674 | 0.886 | 1.000 | 5191 | tags=64%, list=43%, signal=112% |
| 6222 | GOBP\_NUCLEOTIDE\_PHOSPHORYLATION |  | 98 | -0.24 | -0.86 | 0.754 | 0.886 | 1.000 | 4255 | tags=41%, list=35%, signal=63% |
| 6223 | GOBP\_POSITIVE\_REGULATION\_OF\_TOLL\_LIKE\_RECEPTOR\_2\_SIGNALING\_PATHWAY |  | 6 | -0.42 | -0.86 | 0.677 | 0.886 | 1.000 | 2480 | tags=50%, list=21%, signal=63% |
| 6224 | GOBP\_POSITIVE\_REGULATION\_OF\_RNA\_SPLICING |  | 26 | -0.29 | -0.86 | 0.690 | 0.886 | 1.000 | 3335 | tags=31%, list=28%, signal=43% |
| 6225 | GOBP\_REGULATION\_OF\_ACTIVATION\_OF\_JANUS\_KINASE\_ACTIVITY |  | 3 | -0.51 | -0.86 | 0.706 | 0.886 | 1.000 | 1038 | tags=33%, list=9%, signal=36% |
| 6226 | GOMF\_CYSTEINE\_TYPE\_PEPTIDASE\_ACTIVITY |  | 110 | -0.24 | -0.86 | 0.763 | 0.886 | 1.000 | 3876 | tags=32%, list=32%, signal=47% |
| 6227 | GOBP\_PEPTIDYL\_CYSTEINE\_MODIFICATION |  | 32 | -0.28 | -0.86 | 0.695 | 0.886 | 1.000 | 2162 | tags=19%, list=18%, signal=23% |
| 6228 | GOBP\_IMMUNOLOGICAL\_MEMORY\_PROCESS |  | 7 | -0.40 | -0.86 | 0.677 | 0.886 | 1.000 | 3344 | tags=43%, list=28%, signal=59% |
| 6229 | GOBP\_REGULATION\_OF\_POSTSYNAPTIC\_NEUROTRANSMITTER\_RECEPTOR\_ACTIVITY |  | 6 | -0.42 | -0.86 | 0.688 | 0.887 | 1.000 | 5607 | tags=83%, list=47%, signal=156% |
| 6230 | GOBP\_MIDBRAIN\_HINDBRAIN\_BOUNDARY\_DEVELOPMENT |  | 3 | -0.52 | -0.86 | 0.716 | 0.887 | 1.000 | 405 | tags=33%, list=3%, signal=34% |
| 6231 | GOBP\_NEGATIVE\_REGULATION\_OF\_TRANSMEMBRANE\_TRANSPORT |  | 76 | -0.25 | -0.86 | 0.741 | 0.888 | 1.000 | 4177 | tags=38%, list=35%, signal=58% |
| 6232 | GOBP\_TYPE\_I\_INTERFERON\_PRODUCTION |  | 88 | -0.24 | -0.86 | 0.746 | 0.888 | 1.000 | 3504 | tags=36%, list=29%, signal=51% |
| 6233 | GOBP\_SKELETAL\_MYOFIBRIL\_ASSEMBLY |  | 5 | -0.44 | -0.86 | 0.690 | 0.887 | 1.000 | 2494 | tags=40%, list=21%, signal=50% |
| 6234 | GOBP\_POSITIVE\_REGULATION\_BY\_HOST\_OF\_VIRAL\_TRANSCRIPTION |  | 14 | -0.34 | -0.86 | 0.676 | 0.888 | 1.000 | 5712 | tags=57%, list=48%, signal=109% |
| 6235 | GOCC\_SARCOPLASM |  | 53 | -0.26 | -0.86 | 0.718 | 0.888 | 1.000 | 4456 | tags=42%, list=37%, signal=66% |
| 6236 | GOBP\_GAS\_TRANSPORT |  | 9 | -0.38 | -0.86 | 0.671 | 0.888 | 1.000 | 662 | tags=22%, list=6%, signal=24% |
| 6237 | GOBP\_NEGATIVE\_REGULATION\_OF\_PROTEIN\_TYROSINE\_KINASE\_ACTIVITY |  | 21 | -0.31 | -0.86 | 0.692 | 0.888 | 1.000 | 2267 | tags=19%, list=19%, signal=23% |
| 6238 | GOBP\_CADMIUM\_ION\_TRANSMEMBRANE\_TRANSPORT |  | 4 | -0.47 | -0.86 | 0.702 | 0.888 | 1.000 | 2847 | tags=50%, list=24%, signal=66% |
| 6239 | GOBP\_INTRINSIC\_APOPTOTIC\_SIGNALING\_PATHWAY\_IN\_RESPONSE\_TO\_ENDOPLASMIC\_RETICULUM\_STRESS |  | 41 | -0.27 | -0.86 | 0.705 | 0.887 | 1.000 | 4084 | tags=41%, list=34%, signal=63% |
| 6240 | GOBP\_POSITIVE\_REGULATION\_OF\_CATION\_TRANSMEMBRANE\_TRANSPORT |  | 87 | -0.24 | -0.86 | 0.748 | 0.887 | 1.000 | 2955 | tags=26%, list=25%, signal=35% |
| 6241 | GOBP\_POSITIVE\_REGULATION\_OF\_MITOCHONDRIAL\_FISSION |  | 13 | -0.34 | -0.86 | 0.678 | 0.887 | 1.000 | 4934 | tags=46%, list=41%, signal=78% |
| 6242 | GOBP\_REGULATION\_OF\_FATTY\_ACID\_TRANSPORT |  | 30 | -0.28 | -0.86 | 0.698 | 0.887 | 1.000 | 5711 | tags=67%, list=48%, signal=127% |
| 6243 | GOCC\_CHROMAFFIN\_GRANULE\_MEMBRANE |  | 6 | -0.42 | -0.86 | 0.691 | 0.888 | 1.000 | 207 | tags=17%, list=2%, signal=17% |
| 6244 | GOBP\_MISMATCH\_REPAIR |  | 25 | -0.30 | -0.86 | 0.689 | 0.888 | 1.000 | 6931 | tags=84%, list=58%, signal=198% |
| 6245 | GOMF\_PROCOLLAGEN\_PROLINE\_4\_DIOXYGENASE\_ACTIVITY |  | 5 | -0.44 | -0.86 | 0.694 | 0.888 | 1.000 | 5079 | tags=60%, list=42%, signal=104% |
| 6246 | GOBP\_REGULATION\_OF\_LYMPHOID\_PROGENITOR\_CELL\_DIFFERENTIATION |  | 8 | -0.39 | -0.85 | 0.675 | 0.889 | 1.000 | 5139 | tags=63%, list=43%, signal=109% |
| 6247 | GOBP\_PYRIMIDINE\_NUCLEOTIDE\_SALVAGE |  | 5 | -0.45 | -0.85 | 0.701 | 0.890 | 1.000 | 4722 | tags=40%, list=39%, signal=66% |
| 6248 | GOBP\_CARBOHYDRATE\_DERIVATIVE\_METABOLIC\_PROCESS |  | 778 | -0.21 | -0.85 | 0.953 | 0.890 | 1.000 | 4317 | tags=36%, list=36%, signal=52% |
| 6249 | GOBP\_NEGATIVE\_REGULATION\_OF\_MONOOXYGENASE\_ACTIVITY |  | 7 | -0.40 | -0.85 | 0.675 | 0.890 | 1.000 | 5903 | tags=71%, list=49%, signal=140% |
| 6250 | GOBP\_REGULATION\_OF\_MRNA\_SPLICING\_VIA\_SPLICEOSOME |  | 72 | -0.25 | -0.85 | 0.737 | 0.890 | 1.000 | 6168 | tags=60%, list=51%, signal=122% |
| 6251 | GOBP\_NEGATIVE\_REGULATION\_OF\_NEURAL\_PRECURSOR\_CELL\_PROLIFERATION |  | 13 | -0.34 | -0.85 | 0.674 | 0.890 | 1.000 | 2021 | tags=23%, list=17%, signal=28% |
| 6252 | GOBP\_LACTATION |  | 30 | -0.28 | -0.85 | 0.691 | 0.890 | 1.000 | 4532 | tags=40%, list=38%, signal=64% |
| 6253 | GOBP\_REGULATION\_OF\_ASPARTIC\_TYPE\_PEPTIDASE\_ACTIVITY |  | 8 | -0.39 | -0.85 | 0.676 | 0.890 | 1.000 | 2416 | tags=38%, list=20%, signal=47% |
| 6254 | GOBP\_NEGATIVE\_REGULATION\_OF\_DNA\_TEMPLATED\_TRANSCRIPTION\_ELONGATION |  | 15 | -0.33 | -0.85 | 0.681 | 0.890 | 1.000 | 4298 | tags=53%, list=36%, signal=83% |
| 6255 | GOBP\_REGULATION\_OF\_T\_CELL\_CHEMOTAXIS |  | 10 | -0.36 | -0.85 | 0.666 | 0.891 | 1.000 | 2955 | tags=50%, list=25%, signal=66% |
| 6256 | GOMF\_INOSITOL\_TRISPHOSPHATE\_PHOSPHATASE\_ACTIVITY |  | 7 | -0.40 | -0.85 | 0.680 | 0.891 | 1.000 | 5298 | tags=71%, list=44%, signal=128% |
| 6257 | GOCC\_SARCOPLASMIC\_RETICULUM\_MEMBRANE |  | 27 | -0.29 | -0.85 | 0.689 | 0.891 | 1.000 | 1632 | tags=19%, list=14%, signal=21% |
| 6258 | GOBP\_VESICLE\_DOCKING\_INVOLVED\_IN\_EXOCYTOSIS |  | 38 | -0.27 | -0.85 | 0.711 | 0.891 | 1.000 | 3476 | tags=37%, list=29%, signal=52% |
| 6259 | GOBP\_MRNA\_TRANSCRIPTION\_BY\_RNA\_POLYMERASE\_II |  | 8 | -0.39 | -0.85 | 0.683 | 0.891 | 1.000 | 5139 | tags=75%, list=43%, signal=131% |
| 6260 | GOMF\_OXIDOREDUCTASE\_ACTIVITY\_ACTING\_ON\_PAIRED\_DONORS\_WITH\_INCORPORATION\_OR\_REDUCTION\_OF\_MOLECULAR\_OXYGEN\_NAD\_P\_H\_AS\_ONE\_DONOR\_AND\_INCORPORATION\_OF\_ONE\_ATOM\_OF\_OXYGEN |  | 23 | -0.30 | -0.85 | 0.686 | 0.892 | 1.000 | 2869 | tags=30%, list=24%, signal=40% |
| 6261 | GOBP\_ADRENERGIC\_RECEPTOR\_SIGNALING\_PATHWAY |  | 20 | -0.31 | -0.85 | 0.683 | 0.892 | 1.000 | 1522 | tags=20%, list=13%, signal=23% |
| 6262 | GOCC\_INSULIN\_RESPONSIVE\_COMPARTMENT |  | 7 | -0.40 | -0.85 | 0.683 | 0.892 | 1.000 | 3007 | tags=43%, list=25%, signal=57% |
| 6263 | GOBP\_RESPONSE\_TO\_METHOTREXATE |  | 3 | -0.51 | -0.85 | 0.715 | 0.892 | 1.000 | 3930 | tags=67%, list=33%, signal=99% |
| 6264 | GOBP\_PROTEIN\_RETENTION\_IN\_GOLGI\_APPARATUS |  | 5 | -0.44 | -0.85 | 0.690 | 0.892 | 1.000 | 2784 | tags=40%, list=23%, signal=52% |
| 6265 | GOMF\_DNA\_CLAMP\_LOADER\_ACTIVITY |  | 9 | -0.37 | -0.85 | 0.681 | 0.892 | 1.000 | 4607 | tags=44%, list=38%, signal=72% |
| 6266 | GOBP\_INTRINSIC\_APOPTOTIC\_SIGNALING\_PATHWAY\_IN\_RESPONSE\_TO\_DNA\_DAMAGE |  | 68 | -0.25 | -0.85 | 0.742 | 0.893 | 1.000 | 3381 | tags=32%, list=28%, signal=45% |
| 6267 | GOBP\_ACUTE\_PHASE\_RESPONSE |  | 21 | -0.30 | -0.85 | 0.691 | 0.893 | 1.000 | 3186 | tags=33%, list=27%, signal=45% |
| 6268 | GOBP\_ISOTYPE\_SWITCHING\_TO\_IGA\_ISOTYPES |  | 5 | -0.44 | -0.85 | 0.702 | 0.893 | 1.000 | 6715 | tags=100%, list=56%, signal=227% |
| 6269 | GOBP\_MEMBRANE\_LIPID\_BIOSYNTHETIC\_PROCESS |  | 109 | -0.23 | -0.85 | 0.767 | 0.894 | 1.000 | 4467 | tags=39%, list=37%, signal=62% |
| 6270 | GOBP\_NEGATIVE\_REGULATION\_OF\_CHROMATIN\_BINDING |  | 5 | -0.44 | -0.85 | 0.694 | 0.894 | 1.000 | 3241 | tags=40%, list=27%, signal=55% |
| 6271 | GOBP\_NEURAL\_PLATE\_PATTERN\_SPECIFICATION |  | 3 | -0.51 | -0.85 | 0.730 | 0.894 | 1.000 | 2886 | tags=67%, list=24%, signal=88% |
| 6272 | GOBP\_POSITIVE\_REGULATION\_OF\_ENDOPLASMIC\_RETICULUM\_STRESS\_INDUCED\_INTRINSIC\_APOPTOTIC\_SIGNALING\_PATHWAY |  | 8 | -0.38 | -0.85 | 0.691 | 0.894 | 1.000 | 5893 | tags=75%, list=49%, signal=147% |
| 6273 | GOBP\_CELL\_CELL\_ADHESION\_INVOLVED\_IN\_GASTRULATION |  | 7 | -0.40 | -0.85 | 0.684 | 0.894 | 1.000 | 6600 | tags=71%, list=55%, signal=158% |
| 6274 | GOBP\_DEFENSE\_RESPONSE\_TO\_BACTERIUM |  | 82 | -0.24 | -0.85 | 0.752 | 0.894 | 1.000 | 3446 | tags=30%, list=29%, signal=42% |
| 6275 | GOMF\_POLYNUCLEOTIDE\_ADENYLYLTRANSFERASE\_ACTIVITY |  | 3 | -0.51 | -0.85 | 0.718 | 0.894 | 1.000 | 4765 | tags=67%, list=40%, signal=110% |
| 6276 | GOBP\_REGULATION\_OF\_ENDOTHELIAL\_CELL\_DIFFERENTIATION |  | 28 | -0.28 | -0.85 | 0.712 | 0.895 | 1.000 | 3547 | tags=36%, list=30%, signal=51% |
| 6277 | GOBP\_MESENCHYME\_MORPHOGENESIS |  | 38 | -0.27 | -0.85 | 0.713 | 0.895 | 1.000 | 3314 | tags=29%, list=28%, signal=40% |
| 6278 | GOMF\_VASCULAR\_ENDOTHELIAL\_GROWTH\_FACTOR\_RECEPTOR\_2\_BINDING |  | 7 | -0.40 | -0.85 | 0.698 | 0.896 | 1.000 | 3196 | tags=43%, list=27%, signal=58% |
| 6279 | GOBP\_ENDOPLASMIC\_RETICULUM\_ORGANIZATION |  | 60 | -0.25 | -0.85 | 0.740 | 0.896 | 1.000 | 4391 | tags=37%, list=37%, signal=58% |
| 6280 | GOCC\_CAJAL\_BODY |  | 42 | -0.26 | -0.85 | 0.713 | 0.896 | 1.000 | 3300 | tags=26%, list=27%, signal=36% |
| 6281 | GOMF\_TRANSFORMING\_GROWTH\_FACTOR\_BETA\_RECEPTOR\_BINDING |  | 18 | -0.31 | -0.85 | 0.700 | 0.896 | 1.000 | 2457 | tags=28%, list=20%, signal=35% |
| 6282 | GOBP\_OOGENESIS |  | 42 | -0.26 | -0.85 | 0.720 | 0.896 | 1.000 | 3331 | tags=36%, list=28%, signal=49% |
| 6283 | GOBP\_NEURON\_INTRINSIC\_APOPTOTIC\_SIGNALING\_PATHWAY\_IN\_RESPONSE\_TO\_OXIDATIVE\_STRESS |  | 6 | -0.41 | -0.85 | 0.693 | 0.896 | 1.000 | 5544 | tags=50%, list=46%, signal=93% |
| 6284 | GOBP\_PRESYNAPTIC\_ACTIVE\_ZONE\_ORGANIZATION |  | 5 | -0.44 | -0.85 | 0.704 | 0.896 | 1.000 | 1131 | tags=40%, list=9%, signal=44% |
| 6285 | GOBP\_POSITIVE\_REGULATION\_OF\_RELEASE\_OF\_SEQUESTERED\_CALCIUM\_ION\_INTO\_CYTOSOL |  | 27 | -0.29 | -0.85 | 0.706 | 0.896 | 1.000 | 4738 | tags=48%, list=39%, signal=79% |
| 6286 | GOBP\_EMBRYONIC\_DIGESTIVE\_TRACT\_MORPHOGENESIS |  | 11 | -0.35 | -0.85 | 0.676 | 0.896 | 1.000 | 1146 | tags=18%, list=10%, signal=20% |
| 6287 | GOBP\_NEGATIVE\_REGULATION\_OF\_STEM\_CELL\_DIFFERENTIATION |  | 13 | -0.34 | -0.85 | 0.687 | 0.896 | 1.000 | 3195 | tags=23%, list=27%, signal=31% |
| 6288 | GOBP\_HEMATOPOIETIC\_PROGENITOR\_CELL\_DIFFERENTIATION |  | 125 | -0.23 | -0.85 | 0.791 | 0.896 | 1.000 | 3400 | tags=25%, list=28%, signal=34% |
| 6289 | GOBP\_RESPONSE\_TO\_ISCHEMIA |  | 39 | -0.27 | -0.85 | 0.718 | 0.896 | 1.000 | 3521 | tags=38%, list=29%, signal=54% |
| 6290 | GOCC\_CLATHRIN\_COATED\_VESICLE\_MEMBRANE |  | 76 | -0.24 | -0.85 | 0.749 | 0.897 | 1.000 | 4424 | tags=42%, list=37%, signal=66% |
| 6291 | GOCC\_GOLGI\_STACK |  | 102 | -0.23 | -0.85 | 0.776 | 0.897 | 1.000 | 4554 | tags=38%, list=38%, signal=61% |
| 6292 | GOBP\_ENUCLEATE\_ERYTHROCYTE\_DIFFERENTIATION |  | 4 | -0.47 | -0.85 | 0.700 | 0.897 | 1.000 | 3836 | tags=75%, list=32%, signal=110% |
| 6293 | GOBP\_REGULATION\_OF\_SIGNALING\_RECEPTOR\_ACTIVITY |  | 86 | -0.24 | -0.85 | 0.758 | 0.897 | 1.000 | 2604 | tags=27%, list=22%, signal=34% |
| 6294 | GOBP\_NADPH\_REGENERATION |  | 14 | -0.33 | -0.85 | 0.692 | 0.897 | 1.000 | 2485 | tags=36%, list=21%, signal=45% |
| 6295 | GOBP\_RETROGRADE\_VESICLE\_MEDIATED\_TRANSPORT\_GOLGI\_TO\_ENDOPLASMIC\_RETICULUM |  | 75 | -0.24 | -0.85 | 0.750 | 0.897 | 1.000 | 4381 | tags=37%, list=36%, signal=58% |
| 6296 | GOBP\_SPHINGOLIPID\_BIOSYNTHETIC\_PROCESS |  | 81 | -0.24 | -0.85 | 0.758 | 0.897 | 1.000 | 4467 | tags=42%, list=37%, signal=66% |
| 6297 | GOBP\_POSITIVE\_REGULATION\_OF\_EXTRACELLULAR\_MATRIX\_ASSEMBLY |  | 9 | -0.37 | -0.85 | 0.689 | 0.897 | 1.000 | 3551 | tags=44%, list=30%, signal=63% |
| 6298 | GOBP\_NOTOCHORD\_DEVELOPMENT |  | 12 | -0.34 | -0.85 | 0.692 | 0.897 | 1.000 | 3195 | tags=42%, list=27%, signal=57% |
| 6299 | GOBP\_NEGATIVE\_REGULATION\_OF\_LIPOPROTEIN\_METABOLIC\_PROCESS |  | 5 | -0.44 | -0.85 | 0.706 | 0.897 | 1.000 | 3992 | tags=40%, list=33%, signal=60% |
| 6300 | GOBP\_SEGMENT\_SPECIFICATION |  | 10 | -0.36 | -0.85 | 0.690 | 0.897 | 1.000 | 4827 | tags=50%, list=40%, signal=84% |
| 6301 | GOBP\_NEGATIVE\_REGULATION\_OF\_POTASSIUM\_ION\_TRANSPORT |  | 20 | -0.30 | -0.85 | 0.683 | 0.897 | 1.000 | 4139 | tags=45%, list=34%, signal=69% |
| 6302 | GOMF\_DELTA\_CATENIN\_BINDING |  | 5 | -0.44 | -0.84 | 0.706 | 0.897 | 1.000 | 2813 | tags=40%, list=23%, signal=52% |
| 6303 | GOBP\_POSITIVE\_REGULATION\_OF\_SMOOTH\_MUSCLE\_CELL\_APOPTOTIC\_PROCESS |  | 10 | -0.36 | -0.84 | 0.698 | 0.898 | 1.000 | 1981 | tags=40%, list=16%, signal=48% |
| 6304 | GOBP\_POSITIVE\_REGULATION\_OF\_SMALL\_MOLECULE\_METABOLIC\_PROCESS |  | 92 | -0.24 | -0.84 | 0.758 | 0.898 | 1.000 | 5175 | tags=49%, list=43%, signal=85% |
| 6305 | GOBP\_RESPONSE\_TO\_AMMONIUM\_ION |  | 3 | -0.51 | -0.84 | 0.729 | 0.898 | 1.000 | 1222 | tags=33%, list=10%, signal=37% |
| 6306 | GOBP\_NEGATIVE\_REGULATION\_OF\_HISTONE\_METHYLATION |  | 14 | -0.33 | -0.84 | 0.690 | 0.898 | 1.000 | 3644 | tags=50%, list=30%, signal=72% |
| 6307 | GOBP\_REGULATION\_OF\_LYSOSOMAL\_PROTEIN\_CATABOLIC\_PROCESS |  | 7 | -0.40 | -0.84 | 0.697 | 0.898 | 1.000 | 5224 | tags=71%, list=43%, signal=126% |
| 6308 | GOMF\_3\_HYDROXYACYL\_COA\_DEHYDRATASE\_ACTIVITY |  | 5 | -0.43 | -0.84 | 0.710 | 0.898 | 1.000 | 4656 | tags=80%, list=39%, signal=131% |
| 6309 | GOBP\_POSITIVE\_REGULATION\_OF\_NEURON\_PROJECTION\_ARBORIZATION |  | 6 | -0.41 | -0.84 | 0.695 | 0.898 | 1.000 | 2964 | tags=33%, list=25%, signal=44% |
| 6310 | GOBP\_NEGATIVE\_REGULATION\_OF\_SMOOTH\_MUSCLE\_CELL\_DIFFERENTIATION |  | 14 | -0.33 | -0.84 | 0.697 | 0.898 | 1.000 | 3565 | tags=50%, list=30%, signal=71% |
| 6311 | GOCC\_RNA\_CAP\_BINDING\_COMPLEX |  | 9 | -0.37 | -0.84 | 0.686 | 0.898 | 1.000 | 5847 | tags=56%, list=49%, signal=108% |
| 6312 | GOBP\_ESTABLISHMENT\_OF\_RNA\_LOCALIZATION |  | 152 | -0.23 | -0.84 | 0.811 | 0.899 | 1.000 | 4315 | tags=34%, list=36%, signal=52% |
| 6313 | GOMF\_THYROID\_HORMONE\_RECEPTOR\_BINDING |  | 25 | -0.29 | -0.84 | 0.713 | 0.899 | 1.000 | 5972 | tags=68%, list=50%, signal=135% |
| 6314 | GOBP\_POSITIVE\_REGULATION\_OF\_BIOMINERALIZATION |  | 30 | -0.28 | -0.84 | 0.714 | 0.899 | 1.000 | 3269 | tags=37%, list=27%, signal=50% |
| 6315 | GOBP\_RESPONSE\_TO\_INTERLEUKIN\_18 |  | 9 | -0.37 | -0.84 | 0.695 | 0.899 | 1.000 | 3613 | tags=44%, list=30%, signal=64% |
| 6316 | GOBP\_MONOCYTE\_CHEMOTACTIC\_PROTEIN\_1\_PRODUCTION |  | 11 | -0.35 | -0.84 | 0.694 | 0.899 | 1.000 | 2357 | tags=27%, list=20%, signal=34% |
| 6317 | GOMF\_ER\_RETENTION\_SEQUENCE\_BINDING |  | 4 | -0.47 | -0.84 | 0.718 | 0.900 | 1.000 | 4347 | tags=50%, list=36%, signal=78% |
| 6318 | GOBP\_RESPONSE\_TO\_OXIDATIVE\_STRESS |  | 312 | -0.22 | -0.84 | 0.876 | 0.899 | 1.000 | 3690 | tags=29%, list=31%, signal=41% |
| 6319 | GOMF\_PROTEIN\_SERINE\_THREONINE\_PHOSPHATASE\_INHIBITOR\_ACTIVITY |  | 6 | -0.42 | -0.84 | 0.699 | 0.899 | 1.000 | 348 | tags=17%, list=3%, signal=17% |
| 6320 | GOBP\_REGULATION\_OF\_CARDIAC\_MUSCLE\_CONTRACTION\_BY\_CALCIUM\_ION\_SIGNALING |  | 17 | -0.31 | -0.84 | 0.695 | 0.900 | 1.000 | 1632 | tags=18%, list=14%, signal=20% |
| 6321 | GOMF\_CARBOXYLIC\_ACID\_BINDING |  | 101 | -0.23 | -0.84 | 0.785 | 0.900 | 1.000 | 3858 | tags=39%, list=32%, signal=56% |
| 6322 | GOBP\_CEREBRAL\_CORTEX\_NEURON\_DIFFERENTIATION |  | 11 | -0.35 | -0.84 | 0.694 | 0.900 | 1.000 | 3235 | tags=45%, list=27%, signal=62% |
| 6323 | GOBP\_SYNAPTIC\_VESICLE\_BUDDING |  | 7 | -0.40 | -0.84 | 0.697 | 0.900 | 1.000 | 1478 | tags=43%, list=12%, signal=49% |
| 6324 | GOBP\_INTRACELLULAR\_PROTEIN\_TRANSPORT |  | 892 | -0.21 | -0.84 | 0.971 | 0.899 | 1.000 | 4383 | tags=32%, list=36%, signal=47% |
| 6325 | GOCC\_DENTATE\_GYRUS\_MOSSY\_FIBER |  | 5 | -0.44 | -0.84 | 0.714 | 0.900 | 1.000 | 2869 | tags=60%, list=24%, signal=79% |
| 6326 | GOBP\_NEGATIVE\_REGULATION\_OF\_GLIAL\_CELL\_PROLIFERATION |  | 10 | -0.36 | -0.84 | 0.689 | 0.900 | 1.000 | 1297 | tags=20%, list=11%, signal=22% |
| 6327 | GOCC\_CHROMATOID\_BODY |  | 9 | -0.37 | -0.84 | 0.688 | 0.900 | 1.000 | 2591 | tags=33%, list=22%, signal=42% |
| 6328 | GOCC\_KICSTOR\_COMPLEX |  | 4 | -0.46 | -0.84 | 0.717 | 0.900 | 1.000 | 3425 | tags=50%, list=29%, signal=70% |
| 6329 | GOBP\_PARKIN\_MEDIATED\_STIMULATION\_OF\_MITOPHAGY\_IN\_RESPONSE\_TO\_MITOCHONDRIAL\_DEPOLARIZATION |  | 3 | -0.50 | -0.84 | 0.723 | 0.900 | 1.000 | 1362 | tags=33%, list=11%, signal=38% |
| 6330 | GOBP\_ESTABLISHMENT\_OF\_ENDOTHELIAL\_BARRIER |  | 40 | -0.27 | -0.84 | 0.739 | 0.900 | 1.000 | 5178 | tags=52%, list=43%, signal=92% |
| 6331 | GOBP\_REGULATION\_OF\_DNA\_DEMETHYLATION |  | 4 | -0.46 | -0.84 | 0.712 | 0.901 | 1.000 | 727 | tags=25%, list=6%, signal=27% |
| 6332 | GOBP\_BRANCHING\_INVOLVED\_IN\_MAMMARY\_GLAND\_DUCT\_MORPHOGENESIS |  | 16 | -0.32 | -0.84 | 0.693 | 0.901 | 1.000 | 405 | tags=13%, list=3%, signal=13% |
| 6333 | GOBP\_CATECHOL\_CONTAINING\_COMPOUND\_BIOSYNTHETIC\_PROCESS |  | 13 | -0.33 | -0.84 | 0.700 | 0.902 | 1.000 | 2516 | tags=31%, list=21%, signal=39% |
| 6334 | GOBP\_SMALL\_MOLECULE\_BIOSYNTHETIC\_PROCESS |  | 482 | -0.21 | -0.84 | 0.924 | 0.902 | 1.000 | 4283 | tags=36%, list=36%, signal=54% |
| 6335 | GOBP\_RIBOSOME\_DISASSEMBLY |  | 7 | -0.39 | -0.84 | 0.701 | 0.902 | 1.000 | 2182 | tags=29%, list=18%, signal=35% |
| 6336 | GOCC\_U4\_SNRNP |  | 7 | -0.40 | -0.84 | 0.699 | 0.902 | 1.000 | 1068 | tags=14%, list=9%, signal=16% |
| 6337 | GOBP\_FEMALE\_GAMETE\_GENERATION |  | 73 | -0.24 | -0.84 | 0.766 | 0.902 | 1.000 | 3331 | tags=34%, list=28%, signal=47% |
| 6338 | GOBP\_REGULATION\_OF\_CHROMATIN\_BINDING |  | 15 | -0.32 | -0.84 | 0.704 | 0.902 | 1.000 | 4465 | tags=47%, list=37%, signal=74% |
| 6339 | GOBP\_NEGATIVE\_REGULATION\_OF\_CHOLESTEROL\_EFFLUX |  | 3 | -0.50 | -0.84 | 0.733 | 0.902 | 1.000 | 3565 | tags=67%, list=30%, signal=95% |
| 6340 | GOMF\_IMPORTIN\_ALPHA\_FAMILY\_PROTEIN\_BINDING |  | 6 | -0.41 | -0.84 | 0.707 | 0.902 | 1.000 | 7096 | tags=100%, list=59%, signal=244% |
| 6341 | GOBP\_PROTEIN\_LOCALIZATION\_TO\_ORGANELLE |  | 763 | -0.21 | -0.84 | 0.967 | 0.901 | 1.000 | 4395 | tags=33%, list=37%, signal=48% |
| 6342 | GOBP\_TRIGLYCERIDE\_RICH\_LIPOPROTEIN\_PARTICLE\_REMODELING |  | 6 | -0.41 | -0.84 | 0.708 | 0.902 | 1.000 | 3217 | tags=33%, list=27%, signal=46% |
| 6343 | GOBP\_BETA\_CATENIN\_TCF\_COMPLEX\_ASSEMBLY |  | 29 | -0.28 | -0.84 | 0.715 | 0.902 | 1.000 | 5967 | tags=59%, list=50%, signal=116% |
| 6344 | GOBP\_DEOXYRIBONUCLEOSIDE\_MONOPHOSPHATE\_METABOLIC\_PROCESS |  | 13 | -0.34 | -0.84 | 0.695 | 0.902 | 1.000 | 1728 | tags=31%, list=14%, signal=36% |
| 6345 | GOBP\_CELLULAR\_RESPONSE\_TO\_AMMONIUM\_ION |  | 3 | -0.51 | -0.84 | 0.734 | 0.902 | 1.000 | 1222 | tags=33%, list=10%, signal=37% |
| 6346 | GOMF\_MANGANESE\_ION\_TRANSMEMBRANE\_TRANSPORTER\_ACTIVITY |  | 9 | -0.36 | -0.84 | 0.696 | 0.902 | 1.000 | 2847 | tags=33%, list=24%, signal=44% |
| 6347 | GOBP\_NUCLEOSIDE\_MONOPHOSPHATE\_BIOSYNTHETIC\_PROCESS |  | 36 | -0.27 | -0.84 | 0.736 | 0.902 | 1.000 | 4363 | tags=39%, list=36%, signal=61% |
| 6348 | GOBP\_REGULATION\_OF\_METALLOENDOPEPTIDASE\_ACTIVITY |  | 6 | -0.41 | -0.84 | 0.718 | 0.903 | 1.000 | 4369 | tags=67%, list=36%, signal=105% |
| 6349 | GOBP\_PROTEIN\_CONTAINING\_COMPLEX\_LOCALIZATION |  | 213 | -0.22 | -0.84 | 0.849 | 0.903 | 1.000 | 4373 | tags=32%, list=36%, signal=49% |
| 6350 | GOBP\_NEGATIVE\_REGULATION\_BY\_HOST\_OF\_VIRAL\_GENOME\_REPLICATION |  | 5 | -0.43 | -0.84 | 0.720 | 0.903 | 1.000 | 3968 | tags=40%, list=33%, signal=60% |
| 6351 | GOBP\_POSITIVE\_REGULATION\_OF\_VESICLE\_FUSION |  | 6 | -0.41 | -0.84 | 0.702 | 0.903 | 1.000 | 207 | tags=17%, list=2%, signal=17% |
| 6352 | GOBP\_LIPOPROTEIN\_METABOLIC\_PROCESS |  | 92 | -0.23 | -0.84 | 0.782 | 0.903 | 1.000 | 4609 | tags=39%, list=38%, signal=63% |
| 6353 | GOBP\_POSITIVE\_REGULATION\_OF\_PATTERN\_RECOGNITION\_RECEPTOR\_SIGNALING\_PATHWAY |  | 28 | -0.28 | -0.84 | 0.715 | 0.903 | 1.000 | 2731 | tags=39%, list=23%, signal=51% |
| 6354 | GOBP\_FOAM\_CELL\_DIFFERENTIATION |  | 26 | -0.28 | -0.84 | 0.704 | 0.903 | 1.000 | 2752 | tags=35%, list=23%, signal=45% |
| 6355 | GOMF\_VITAMIN\_TRANSMEMBRANE\_TRANSPORTER\_ACTIVITY |  | 20 | -0.30 | -0.84 | 0.715 | 0.903 | 1.000 | 4320 | tags=60%, list=36%, signal=94% |
| 6356 | GOCC\_U7\_SNRNP |  | 5 | -0.43 | -0.84 | 0.715 | 0.903 | 1.000 | 427 | tags=20%, list=4%, signal=21% |
| 6357 | GOBP\_ANTIGEN\_PROCESSING\_AND\_PRESENTATION\_OF\_LIPID\_ANTIGEN\_VIA\_MHC\_CLASS\_IB |  | 4 | -0.46 | -0.84 | 0.723 | 0.903 | 1.000 | 3112 | tags=50%, list=26%, signal=67% |
| 6358 | GOMF\_AMINOACYLASE\_ACTIVITY |  | 4 | -0.46 | -0.84 | 0.729 | 0.903 | 1.000 | 3710 | tags=50%, list=31%, signal=72% |
| 6359 | GOBP\_REGULATION\_OF\_DNA\_DEPENDENT\_DNA\_REPLICATION\_INITIATION |  | 6 | -0.41 | -0.84 | 0.707 | 0.904 | 1.000 | 2823 | tags=33%, list=24%, signal=44% |
| 6360 | GOBP\_POSITIVE\_REGULATION\_OF\_AMINE\_TRANSPORT |  | 25 | -0.29 | -0.84 | 0.719 | 0.904 | 1.000 | 1799 | tags=24%, list=15%, signal=28% |
| 6361 | GOBP\_MRNA\_METHYLATION |  | 15 | -0.32 | -0.84 | 0.703 | 0.904 | 1.000 | 4925 | tags=47%, list=41%, signal=79% |
| 6362 | GOBP\_MAINTENANCE\_OF\_PROTEIN\_LOCATION\_IN\_CELL |  | 46 | -0.26 | -0.84 | 0.740 | 0.904 | 1.000 | 4240 | tags=37%, list=35%, signal=57% |
| 6363 | GOCC\_PLATELET\_DENSE\_GRANULE\_MEMBRANE |  | 4 | -0.46 | -0.84 | 0.729 | 0.904 | 1.000 | 4268 | tags=75%, list=36%, signal=116% |
| 6364 | GOBP\_ACTIN\_FILAMENT\_SEVERING |  | 13 | -0.33 | -0.84 | 0.696 | 0.904 | 1.000 | 4003 | tags=38%, list=33%, signal=58% |
| 6365 | GOBP\_NEGATIVE\_REGULATION\_OF\_CELL\_CELL\_ADHESION\_MEDIATED\_BY\_CADHERIN |  | 7 | -0.39 | -0.84 | 0.706 | 0.904 | 1.000 | 3269 | tags=43%, list=27%, signal=59% |
| 6366 | GOMF\_DIPEPTIDYL\_PEPTIDASE\_ACTIVITY |  | 8 | -0.38 | -0.84 | 0.692 | 0.904 | 1.000 | 6237 | tags=75%, list=52%, signal=156% |
| 6367 | GOBP\_NEGATIVE\_REGULATION\_OF\_ATP\_BIOSYNTHETIC\_PROCESS |  | 3 | -0.50 | -0.84 | 0.736 | 0.904 | 1.000 | 6028 | tags=100%, list=50%, signal=201% |
| 6368 | GOBP\_CORONARY\_ARTERY\_MORPHOGENESIS |  | 7 | -0.39 | -0.84 | 0.704 | 0.904 | 1.000 | 5316 | tags=71%, list=44%, signal=128% |
| 6369 | GOMF\_ACYL\_COA\_OXIDASE\_ACTIVITY |  | 6 | -0.41 | -0.83 | 0.712 | 0.905 | 1.000 | 5371 | tags=50%, list=45%, signal=90% |
| 6370 | GOBP\_POSITIVE\_REGULATION\_OF\_MUSCLE\_ADAPTATION |  | 4 | -0.46 | -0.83 | 0.729 | 0.905 | 1.000 | 3444 | tags=50%, list=29%, signal=70% |
| 6371 | GOBP\_PROTEIN\_SULFATION |  | 5 | -0.43 | -0.83 | 0.714 | 0.905 | 1.000 | 5731 | tags=60%, list=48%, signal=115% |
| 6372 | GOBP\_NEGATIVE\_REGULATION\_OF\_INTERLEUKIN\_12\_PRODUCTION |  | 7 | -0.39 | -0.83 | 0.710 | 0.905 | 1.000 | 2820 | tags=43%, list=23%, signal=56% |
| 6373 | GOBP\_REGULATION\_OF\_RYANODINE\_SENSITIVE\_CALCIUM\_RELEASE\_CHANNEL\_ACTIVITY |  | 16 | -0.32 | -0.83 | 0.714 | 0.905 | 1.000 | 1304 | tags=19%, list=11%, signal=21% |
| 6374 | GOBP\_REGULATION\_OF\_OXIDOREDUCTASE\_ACTIVITY |  | 66 | -0.24 | -0.83 | 0.764 | 0.905 | 1.000 | 3921 | tags=36%, list=33%, signal=54% |
| 6375 | GOMF\_NADP\_BINDING |  | 38 | -0.26 | -0.83 | 0.730 | 0.905 | 1.000 | 1535 | tags=21%, list=13%, signal=24% |
| 6376 | GOCC\_NUCLEOLUS |  | 684 | -0.21 | -0.83 | 0.961 | 0.905 | 1.000 | 3635 | tags=27%, list=30%, signal=37% |
| 6377 | GOBP\_CALCIUM\_IMPORT\_INTO\_THE\_MITOCHONDRION |  | 11 | -0.35 | -0.83 | 0.697 | 0.906 | 1.000 | 3682 | tags=45%, list=31%, signal=65% |
| 6378 | GOBP\_POLYPHOSPHATE\_CATABOLIC\_PROCESS |  | 4 | -0.46 | -0.83 | 0.729 | 0.906 | 1.000 | 273 | tags=25%, list=2%, signal=26% |
| 6379 | GOBP\_ORNITHINE\_METABOLIC\_PROCESS |  | 6 | -0.41 | -0.83 | 0.719 | 0.906 | 1.000 | 1788 | tags=17%, list=15%, signal=20% |
| 6380 | GOBP\_REGULATION\_OF\_SUPEROXIDE\_DISMUTASE\_ACTIVITY |  | 4 | -0.46 | -0.83 | 0.732 | 0.906 | 1.000 | 3425 | tags=50%, list=29%, signal=70% |
| 6381 | GOBP\_EMBRYONIC\_DIGIT\_MORPHOGENESIS |  | 37 | -0.26 | -0.83 | 0.734 | 0.906 | 1.000 | 4097 | tags=35%, list=34%, signal=53% |
| 6382 | GOMF\_CHROMATIN\_INSULATOR\_SEQUENCE\_BINDING |  | 3 | -0.50 | -0.83 | 0.745 | 0.906 | 1.000 | 5641 | tags=67%, list=47%, signal=126% |
| 6383 | GOBP\_BENZENE\_CONTAINING\_COMPOUND\_METABOLIC\_PROCESS |  | 11 | -0.34 | -0.83 | 0.701 | 0.906 | 1.000 | 2851 | tags=36%, list=24%, signal=48% |
| 6384 | GOBP\_IMPORT\_INTO\_CELL |  | 135 | -0.22 | -0.83 | 0.822 | 0.906 | 1.000 | 3011 | tags=27%, list=25%, signal=35% |
| 6385 | GOMF\_CHLORIDE\_CHANNEL\_REGULATOR\_ACTIVITY |  | 12 | -0.34 | -0.83 | 0.702 | 0.906 | 1.000 | 2955 | tags=42%, list=25%, signal=55% |
| 6386 | GOBP\_ALTERNATIVE\_MRNA\_SPLICING\_VIA\_SPLICEOSOME |  | 50 | -0.25 | -0.83 | 0.746 | 0.906 | 1.000 | 6125 | tags=56%, list=51%, signal=114% |
| 6387 | GOBP\_CELLULAR\_RESPONSE\_TO\_HYDROPEROXIDE |  | 7 | -0.39 | -0.83 | 0.708 | 0.907 | 1.000 | 1236 | tags=29%, list=10%, signal=32% |
| 6388 | GOMF\_DEATH\_DOMAIN\_BINDING |  | 8 | -0.38 | -0.83 | 0.705 | 0.907 | 1.000 | 658 | tags=13%, list=5%, signal=13% |
| 6389 | GOBP\_PLUS\_END\_DIRECTED\_ORGANELLE\_TRANSPORT\_ALONG\_MICROTUBULE |  | 7 | -0.39 | -0.83 | 0.717 | 0.907 | 1.000 | 4052 | tags=71%, list=34%, signal=108% |
| 6390 | GOCC\_AZUROPHIL\_GRANULE\_LUMEN |  | 55 | -0.25 | -0.83 | 0.760 | 0.907 | 1.000 | 5390 | tags=47%, list=45%, signal=85% |
| 6391 | GOBP\_REGULATION\_OF\_PROTEIN\_LOCALIZATION\_TO\_PLASMA\_MEMBRANE |  | 70 | -0.24 | -0.83 | 0.777 | 0.907 | 1.000 | 4369 | tags=39%, list=36%, signal=60% |
| 6392 | GOMF\_ALDEHYDE\_LYASE\_ACTIVITY |  | 6 | -0.41 | -0.83 | 0.725 | 0.908 | 1.000 | 4966 | tags=67%, list=41%, signal=114% |
| 6393 | GOBP\_PURINE\_NUCLEOSIDE\_MONOPHOSPHATE\_METABOLIC\_PROCESS |  | 34 | -0.27 | -0.83 | 0.737 | 0.908 | 1.000 | 4363 | tags=44%, list=36%, signal=69% |
| 6394 | GOBP\_GLIAL\_CELL\_APOPTOTIC\_PROCESS |  | 12 | -0.34 | -0.83 | 0.713 | 0.908 | 1.000 | 3836 | tags=42%, list=32%, signal=61% |
| 6395 | GOMF\_POTASSIUM\_CHANNEL\_REGULATOR\_ACTIVITY |  | 35 | -0.27 | -0.83 | 0.741 | 0.908 | 1.000 | 1125 | tags=14%, list=9%, signal=16% |
| 6396 | GOBP\_MULTI\_CILIATED\_EPITHELIAL\_CELL\_DIFFERENTIATION |  | 3 | -0.50 | -0.83 | 0.751 | 0.908 | 1.000 | 6052 | tags=100%, list=50%, signal=202% |
| 6397 | GOBP\_RESPONSE\_TO\_BACTERIAL\_LIPOPROTEIN |  | 4 | -0.46 | -0.83 | 0.743 | 0.908 | 1.000 | 1236 | tags=25%, list=10%, signal=28% |
| 6398 | GOBP\_REGULATION\_OF\_I\_KAPPAB\_PHOSPHORYLATION |  | 6 | -0.41 | -0.83 | 0.718 | 0.908 | 1.000 | 3613 | tags=50%, list=30%, signal=71% |
| 6399 | GOCC\_MULTIVESICULAR\_BODY |  | 39 | -0.26 | -0.83 | 0.742 | 0.908 | 1.000 | 4845 | tags=46%, list=40%, signal=77% |
| 6400 | GOBP\_REGULATION\_OF\_INTEGRIN\_ACTIVATION |  | 14 | -0.32 | -0.83 | 0.705 | 0.908 | 1.000 | 1774 | tags=21%, list=15%, signal=25% |
| 6401 | GOCC\_TRANSCRIPTION\_FACTOR\_TFIIH\_HOLO\_COMPLEX |  | 9 | -0.36 | -0.83 | 0.700 | 0.908 | 1.000 | 6024 | tags=78%, list=50%, signal=156% |
| 6402 | GOMF\_PHOSPHATIDYLCHOLINE\_TRANSFER\_ACTIVITY |  | 5 | -0.43 | -0.83 | 0.733 | 0.908 | 1.000 | 2919 | tags=40%, list=24%, signal=53% |
| 6403 | GOBP\_MANGANESE\_ION\_TRANSMEMBRANE\_TRANSPORT |  | 9 | -0.36 | -0.83 | 0.708 | 0.908 | 1.000 | 2847 | tags=33%, list=24%, signal=44% |
| 6404 | GOBP\_TANGENTIAL\_MIGRATION\_FROM\_THE\_SUBVENTRICULAR\_ZONE\_TO\_THE\_OLFACTORY\_BULB |  | 4 | -0.46 | -0.83 | 0.730 | 0.908 | 1.000 | 5989 | tags=75%, list=50%, signal=150% |
| 6405 | GOBP\_RESPONSE\_TO\_STEROID\_HORMONE |  | 224 | -0.22 | -0.83 | 0.870 | 0.908 | 1.000 | 4521 | tags=41%, list=38%, signal=64% |
| 6406 | GOBP\_NUCLEOLUS\_ORGANIZATION |  | 5 | -0.43 | -0.83 | 0.727 | 0.908 | 1.000 | 1122 | tags=20%, list=9%, signal=22% |
| 6407 | GOBP\_RNA\_LOCALIZATION |  | 178 | -0.22 | -0.83 | 0.852 | 0.908 | 1.000 | 4799 | tags=35%, list=40%, signal=58% |
| 6408 | GOBP\_NEGATIVE\_REGULATION\_OF\_LIPID\_STORAGE |  | 15 | -0.32 | -0.83 | 0.714 | 0.908 | 1.000 | 2752 | tags=40%, list=23%, signal=52% |
| 6409 | GOCC\_MESSENGER\_RIBONUCLEOPROTEIN\_COMPLEX |  | 8 | -0.38 | -0.83 | 0.713 | 0.908 | 1.000 | 6745 | tags=75%, list=56%, signal=171% |
| 6410 | GOBP\_HOMOCYSTEINE\_METABOLIC\_PROCESS |  | 10 | -0.35 | -0.83 | 0.703 | 0.908 | 1.000 | 2580 | tags=30%, list=21%, signal=38% |
| 6411 | GOMF\_ASPARTIC\_TYPE\_ENDOPEPTIDASE\_INHIBITOR\_ACTIVITY |  | 5 | -0.43 | -0.83 | 0.722 | 0.908 | 1.000 | 3005 | tags=40%, list=25%, signal=53% |
| 6412 | GOBP\_ENDOPLASMIC\_RETICULUM\_CALCIUM\_ION\_HOMEOSTASIS |  | 20 | -0.30 | -0.83 | 0.713 | 0.908 | 1.000 | 2291 | tags=20%, list=19%, signal=25% |
| 6413 | GOMF\_HYALURONIC\_ACID\_BINDING |  | 13 | -0.33 | -0.83 | 0.715 | 0.908 | 1.000 | 3860 | tags=38%, list=32%, signal=57% |
| 6414 | GOBP\_PEPTIDYL\_SERINE\_DEPHOSPHORYLATION |  | 17 | -0.31 | -0.83 | 0.707 | 0.908 | 1.000 | 3691 | tags=29%, list=31%, signal=42% |
| 6415 | GOMF\_ACYL\_COA\_BINDING |  | 20 | -0.30 | -0.83 | 0.728 | 0.909 | 1.000 | 104 | tags=10%, list=1%, signal=10% |
| 6416 | GOMF\_1\_PHOSPHATIDYLINOSITOL\_4\_KINASE\_ACTIVITY |  | 4 | -0.46 | -0.83 | 0.738 | 0.909 | 1.000 | 4600 | tags=50%, list=38%, signal=81% |
| 6417 | GOBP\_REGULATION\_OF\_POSTSYNAPTIC\_MEMBRANE\_NEUROTRANSMITTER\_RECEPTOR\_LEVELS |  | 35 | -0.27 | -0.83 | 0.738 | 0.908 | 1.000 | 4775 | tags=49%, list=40%, signal=80% |
| 6418 | GOBP\_NEGATIVE\_REGULATION\_OF\_DENDRITIC\_CELL\_APOPTOTIC\_PROCESS |  | 5 | -0.43 | -0.83 | 0.720 | 0.909 | 1.000 | 1903 | tags=20%, list=16%, signal=24% |
| 6419 | GOBP\_VITAMIN\_A\_METABOLIC\_PROCESS |  | 7 | -0.39 | -0.83 | 0.717 | 0.909 | 1.000 | 3525 | tags=57%, list=29%, signal=81% |
| 6420 | GOBP\_NEGATIVE\_REGULATION\_OF\_ANION\_TRANSPORT |  | 130 | -0.22 | -0.83 | 0.816 | 0.909 | 1.000 | 3934 | tags=34%, list=33%, signal=50% |
| 6421 | GOBP\_POSITIVE\_REGULATION\_OF\_RUFFLE\_ASSEMBLY |  | 8 | -0.37 | -0.83 | 0.721 | 0.909 | 1.000 | 4018 | tags=50%, list=33%, signal=75% |
| 6422 | GOBP\_CELLULAR\_RESPONSE\_TO\_SALT\_STRESS |  | 9 | -0.36 | -0.83 | 0.707 | 0.909 | 1.000 | 4353 | tags=56%, list=36%, signal=87% |
| 6423 | GOMF\_7SK\_SNRNA\_BINDING |  | 6 | -0.41 | -0.83 | 0.727 | 0.909 | 1.000 | 4182 | tags=50%, list=35%, signal=77% |
| 6424 | GOBP\_REGULATION\_OF\_GLIAL\_CELL\_DIFFERENTIATION |  | 44 | -0.26 | -0.83 | 0.753 | 0.909 | 1.000 | 1743 | tags=18%, list=15%, signal=21% |
| 6425 | GOBP\_REGULATION\_OF\_SYSTEMIC\_ARTERIAL\_BLOOD\_PRESSURE\_BY\_NOREPINEPHRINE\_EPINEPHRINE |  | 5 | -0.43 | -0.83 | 0.732 | 0.909 | 1.000 | 893 | tags=20%, list=7%, signal=22% |
| 6426 | GOCC\_DERLIN\_1\_RETROTRANSLOCATION\_COMPLEX |  | 7 | -0.39 | -0.83 | 0.711 | 0.910 | 1.000 | 265 | tags=14%, list=2%, signal=15% |
| 6427 | GOMF\_LIPASE\_ACTIVITY |  | 80 | -0.23 | -0.83 | 0.787 | 0.910 | 1.000 | 4727 | tags=49%, list=39%, signal=80% |
| 6428 | GOBP\_GLYCEROLIPID\_CATABOLIC\_PROCESS |  | 52 | -0.25 | -0.83 | 0.762 | 0.910 | 1.000 | 4905 | tags=50%, list=41%, signal=84% |
| 6429 | GOBP\_NEGATIVE\_REGULATION\_OF\_MESENCHYMAL\_CELL\_PROLIFERATION |  | 5 | -0.43 | -0.83 | 0.731 | 0.910 | 1.000 | 408 | tags=20%, list=3%, signal=21% |
| 6430 | GOBP\_OLFACTORY\_BULB\_INTERNEURON\_DEVELOPMENT |  | 4 | -0.46 | -0.83 | 0.734 | 0.910 | 1.000 | 4696 | tags=50%, list=39%, signal=82% |
| 6431 | GOCC\_CYTOPLASMIC\_SIDE\_OF\_ENDOPLASMIC\_RETICULUM\_MEMBRANE |  | 13 | -0.33 | -0.83 | 0.710 | 0.910 | 1.000 | 2291 | tags=31%, list=19%, signal=38% |
| 6432 | GOBP\_MRNA\_MODIFICATION |  | 23 | -0.29 | -0.83 | 0.723 | 0.911 | 1.000 | 492 | tags=9%, list=4%, signal=9% |
| 6433 | GOMF\_SUMO\_BINDING |  | 14 | -0.33 | -0.83 | 0.721 | 0.911 | 1.000 | 4022 | tags=43%, list=33%, signal=64% |
| 6434 | GOMF\_RIBOSOMAL\_SMALL\_SUBUNIT\_BINDING |  | 16 | -0.31 | -0.83 | 0.718 | 0.911 | 1.000 | 3527 | tags=31%, list=29%, signal=44% |
| 6435 | GOBP\_REGULATION\_OF\_PHOSPHATIDYLINOSITOL\_DEPHOSPHORYLATION |  | 5 | -0.43 | -0.83 | 0.733 | 0.911 | 1.000 | 3244 | tags=40%, list=27%, signal=55% |
| 6436 | GOBP\_REGULATION\_OF\_DNA\_DUPLEX\_UNWINDING |  | 6 | -0.40 | -0.82 | 0.722 | 0.911 | 1.000 | 2756 | tags=50%, list=23%, signal=65% |
| 6437 | GOCC\_CALYX\_OF\_HELD |  | 11 | -0.34 | -0.82 | 0.709 | 0.911 | 1.000 | 2150 | tags=36%, list=18%, signal=44% |
| 6438 | GOBP\_NEGATIVE\_REGULATION\_OF\_PRODUCTION\_OF\_MIRNAS\_INVOLVED\_IN\_GENE\_SILENCING\_BY\_MIRNA |  | 9 | -0.36 | -0.82 | 0.713 | 0.911 | 1.000 | 1806 | tags=22%, list=15%, signal=26% |
| 6439 | GOBP\_REGULATION\_OF\_ALTERNATIVE\_MRNA\_SPLICING\_VIA\_SPLICEOSOME |  | 39 | -0.26 | -0.82 | 0.753 | 0.911 | 1.000 | 6059 | tags=54%, list=50%, signal=108% |
| 6440 | GOBP\_CELLULAR\_PROTEIN\_COMPLEX\_DISASSEMBLY |  | 173 | -0.22 | -0.82 | 0.853 | 0.911 | 1.000 | 2634 | tags=21%, list=22%, signal=27% |
| 6441 | GOBP\_POSITIVE\_REGULATION\_OF\_RNA\_BINDING |  | 7 | -0.39 | -0.82 | 0.720 | 0.912 | 1.000 | 5307 | tags=43%, list=44%, signal=77% |
| 6442 | GOCC\_RIPOPTOSOME |  | 5 | -0.43 | -0.82 | 0.737 | 0.912 | 1.000 | 2494 | tags=40%, list=21%, signal=50% |
| 6443 | GOMF\_EXOPOLYPHOSPHATASE\_ACTIVITY |  | 4 | -0.46 | -0.82 | 0.750 | 0.913 | 1.000 | 273 | tags=25%, list=2%, signal=26% |
| 6444 | GOBP\_PROTEIN\_CATABOLIC\_PROCESS\_IN\_THE\_VACUOLE |  | 14 | -0.32 | -0.82 | 0.713 | 0.913 | 1.000 | 3007 | tags=36%, list=25%, signal=48% |
| 6445 | GOBP\_NEGATIVE\_REGULATION\_OF\_SYNAPTIC\_TRANSMISSION |  | 41 | -0.26 | -0.82 | 0.751 | 0.913 | 1.000 | 6005 | tags=54%, list=50%, signal=107% |
| 6446 | GOCC\_VACUOLAR\_MEMBRANE |  | 322 | -0.21 | -0.82 | 0.915 | 0.913 | 1.000 | 3427 | tags=30%, list=29%, signal=41% |
| 6447 | GOBP\_ORGANIC\_ACID\_BIOSYNTHETIC\_PROCESS |  | 217 | -0.22 | -0.82 | 0.875 | 0.914 | 1.000 | 4272 | tags=36%, list=36%, signal=55% |
| 6448 | GOBP\_NEGATIVE\_REGULATION\_OF\_ALCOHOL\_BIOSYNTHETIC\_PROCESS |  | 10 | -0.35 | -0.82 | 0.716 | 0.914 | 1.000 | 2666 | tags=40%, list=22%, signal=51% |
| 6449 | GOBP\_GOLGI\_DISASSEMBLY |  | 6 | -0.40 | -0.82 | 0.725 | 0.914 | 1.000 | 5705 | tags=50%, list=47%, signal=95% |
| 6450 | GOCC\_MICROVILLUS\_MEMBRANE |  | 15 | -0.31 | -0.82 | 0.720 | 0.914 | 1.000 | 3619 | tags=53%, list=30%, signal=76% |
| 6451 | GOBP\_NEGATIVE\_REGULATION\_OF\_CAMP\_DEPENDENT\_PROTEIN\_KINASE\_ACTIVITY |  | 9 | -0.36 | -0.82 | 0.716 | 0.914 | 1.000 | 4008 | tags=44%, list=33%, signal=67% |
| 6452 | GOMF\_CHROMATIN\_DNA\_BINDING |  | 56 | -0.24 | -0.82 | 0.772 | 0.914 | 1.000 | 4184 | tags=38%, list=35%, signal=57% |
| 6453 | GOMF\_PRE\_MRNA\_INTRONIC\_BINDING |  | 10 | -0.35 | -0.82 | 0.725 | 0.914 | 1.000 | 75 | tags=10%, list=1%, signal=10% |
| 6454 | GOBP\_FATTY\_ACID\_BIOSYNTHETIC\_PROCESS |  | 105 | -0.23 | -0.82 | 0.807 | 0.914 | 1.000 | 4689 | tags=47%, list=39%, signal=76% |
| 6455 | GOBP\_REGULATION\_OF\_PROTEIN\_TARGETING |  | 57 | -0.24 | -0.82 | 0.772 | 0.914 | 1.000 | 4110 | tags=32%, list=34%, signal=48% |
| 6456 | GOMF\_7S\_RNA\_BINDING |  | 7 | -0.39 | -0.82 | 0.719 | 0.914 | 1.000 | 4839 | tags=43%, list=40%, signal=72% |
| 6457 | GOCC\_DENDRITE\_CYTOPLASM |  | 22 | -0.29 | -0.82 | 0.721 | 0.914 | 1.000 | 3732 | tags=45%, list=31%, signal=66% |
| 6458 | GOBP\_POSITIVE\_REGULATION\_OF\_MUSCLE\_CELL\_APOPTOTIC\_PROCESS |  | 16 | -0.31 | -0.82 | 0.722 | 0.914 | 1.000 | 1981 | tags=31%, list=16%, signal=37% |
| 6459 | GOBP\_MAINTENANCE\_OF\_PROTEIN\_LOCATION |  | 68 | -0.24 | -0.82 | 0.791 | 0.914 | 1.000 | 4240 | tags=35%, list=35%, signal=54% |
| 6460 | GOBP\_CHONDROITIN\_SULFATE\_PROTEOGLYCAN\_BIOSYNTHETIC\_PROCESS |  | 22 | -0.29 | -0.82 | 0.729 | 0.914 | 1.000 | 1256 | tags=18%, list=10%, signal=20% |
| 6461 | GOBP\_POSITIVE\_REGULATION\_OF\_DEOXYRIBONUCLEASE\_ACTIVITY |  | 5 | -0.42 | -0.82 | 0.752 | 0.915 | 1.000 | 6931 | tags=100%, list=58%, signal=236% |
| 6462 | GOBP\_MACROPHAGE\_PROLIFERATION |  | 7 | -0.39 | -0.82 | 0.725 | 0.915 | 1.000 | 4317 | tags=57%, list=36%, signal=89% |
| 6463 | GOBP\_NEGATIVE\_REGULATION\_OF\_REACTIVE\_OXYGEN\_SPECIES\_METABOLIC\_PROCESS |  | 35 | -0.26 | -0.82 | 0.743 | 0.914 | 1.000 | 4249 | tags=40%, list=35%, signal=62% |
| 6464 | GOBP\_NUCLEOSIDE\_DIPHOSPHATE\_METABOLIC\_PROCESS |  | 116 | -0.22 | -0.82 | 0.830 | 0.915 | 1.000 | 4255 | tags=40%, list=35%, signal=61% |
| 6465 | GOBP\_POSITIVE\_REGULATION\_OF\_GLUTAMATE\_SECRETION |  | 6 | -0.40 | -0.82 | 0.734 | 0.915 | 1.000 | 801 | tags=33%, list=7%, signal=36% |
| 6466 | GOCC\_PRONUCLEUS |  | 7 | -0.39 | -0.82 | 0.729 | 0.917 | 1.000 | 4361 | tags=71%, list=36%, signal=112% |
| 6467 | GOBP\_PURINE\_NUCLEOSIDE\_CATABOLIC\_PROCESS |  | 7 | -0.38 | -0.82 | 0.728 | 0.918 | 1.000 | 1107 | tags=29%, list=9%, signal=31% |
| 6468 | GOBP\_SMOOTH\_MUSCLE\_ADAPTATION |  | 3 | -0.49 | -0.82 | 0.764 | 0.918 | 1.000 | 726 | tags=33%, list=6%, signal=35% |
| 6469 | GOBP\_NEGATIVE\_REGULATION\_OF\_MUSCLE\_CONTRACTION |  | 16 | -0.31 | -0.82 | 0.728 | 0.917 | 1.000 | 3968 | tags=44%, list=33%, signal=65% |
| 6470 | GOBP\_VESICLE\_DOCKING |  | 54 | -0.25 | -0.82 | 0.782 | 0.918 | 1.000 | 3476 | tags=31%, list=29%, signal=44% |
| 6471 | GOBP\_INTESTINE\_SMOOTH\_MUSCLE\_CONTRACTION |  | 5 | -0.42 | -0.82 | 0.735 | 0.918 | 1.000 | 199 | tags=20%, list=2%, signal=20% |
| 6472 | GOBP\_NEGATIVE\_REGULATION\_OF\_CYTOSOLIC\_CALCIUM\_ION\_CONCENTRATION |  | 6 | -0.40 | -0.82 | 0.736 | 0.918 | 1.000 | 3007 | tags=50%, list=25%, signal=67% |
| 6473 | GOBP\_ATRIOVENTRICULAR\_CANAL\_DEVELOPMENT |  | 7 | -0.38 | -0.82 | 0.730 | 0.918 | 1.000 | 2212 | tags=43%, list=18%, signal=53% |
| 6474 | GOBP\_REGULATION\_OF\_MICROTUBULE\_BASED\_MOVEMENT |  | 23 | -0.28 | -0.82 | 0.730 | 0.918 | 1.000 | 3544 | tags=35%, list=30%, signal=49% |
| 6475 | GOCC\_PROTEIN\_PHOSPHATASE\_TYPE\_1\_COMPLEX |  | 10 | -0.35 | -0.82 | 0.724 | 0.918 | 1.000 | 4521 | tags=40%, list=38%, signal=64% |
| 6476 | GOMF\_FIBROBLAST\_GROWTH\_FACTOR\_ACTIVATED\_RECEPTOR\_ACTIVITY |  | 5 | -0.42 | -0.82 | 0.740 | 0.918 | 1.000 | 2555 | tags=20%, list=21%, signal=25% |
| 6477 | GOCC\_POST\_MRNA\_RELEASE\_SPLICEOSOMAL\_COMPLEX |  | 10 | -0.35 | -0.82 | 0.727 | 0.919 | 1.000 | 5920 | tags=80%, list=49%, signal=158% |
| 6478 | GOBP\_REGULATION\_OF\_CHOLESTEROL\_BIOSYNTHETIC\_PROCESS |  | 37 | -0.26 | -0.82 | 0.759 | 0.919 | 1.000 | 4272 | tags=43%, list=36%, signal=67% |
| 6479 | GOBP\_PHENOL\_CONTAINING\_COMPOUND\_BIOSYNTHETIC\_PROCESS |  | 24 | -0.28 | -0.82 | 0.747 | 0.919 | 1.000 | 2516 | tags=25%, list=21%, signal=32% |
| 6480 | GOBP\_RESPONSE\_TO\_CAFFEINE |  | 14 | -0.32 | -0.82 | 0.723 | 0.919 | 1.000 | 1632 | tags=21%, list=14%, signal=25% |
| 6481 | GOBP\_INORGANIC\_ION\_IMPORT\_ACROSS\_PLASMA\_MEMBRANE |  | 49 | -0.25 | -0.82 | 0.770 | 0.919 | 1.000 | 3660 | tags=35%, list=30%, signal=50% |
| 6482 | GOBP\_VERY\_LOW\_DENSITY\_LIPOPROTEIN\_PARTICLE\_CLEARANCE |  | 3 | -0.49 | -0.82 | 0.766 | 0.919 | 1.000 | 4758 | tags=67%, list=40%, signal=110% |
| 6483 | GOMF\_L\_ARGININE\_TRANSMEMBRANE\_TRANSPORTER\_ACTIVITY |  | 4 | -0.45 | -0.82 | 0.750 | 0.919 | 1.000 | 2026 | tags=25%, list=17%, signal=30% |
| 6484 | GOBP\_3\_UTR\_MEDIATED\_MRNA\_DESTABILIZATION |  | 14 | -0.32 | -0.82 | 0.729 | 0.919 | 1.000 | 4569 | tags=57%, list=38%, signal=92% |
| 6485 | GOBP\_REGULATION\_OF\_PROTEIN\_STABILITY |  | 218 | -0.21 | -0.82 | 0.884 | 0.919 | 1.000 | 4395 | tags=31%, list=37%, signal=48% |
| 6486 | GOBP\_OSTEOBLAST\_DIFFERENTIATION |  | 149 | -0.22 | -0.82 | 0.851 | 0.919 | 1.000 | 3389 | tags=26%, list=28%, signal=36% |
| 6487 | GOBP\_EPITHELIAL\_CELL\_PROLIFERATION\_INVOLVED\_IN\_LUNG\_MORPHOGENESIS |  | 8 | -0.37 | -0.81 | 0.727 | 0.919 | 1.000 | 408 | tags=13%, list=3%, signal=13% |
| 6488 | GOMF\_MRNA\_METHYLTRANSFERASE\_ACTIVITY |  | 12 | -0.33 | -0.81 | 0.724 | 0.919 | 1.000 | 133 | tags=8%, list=1%, signal=8% |
| 6489 | GOBP\_AMP\_METABOLIC\_PROCESS |  | 12 | -0.33 | -0.81 | 0.719 | 0.920 | 1.000 | 4296 | tags=42%, list=36%, signal=65% |
| 6490 | GOBP\_POSITIVE\_REGULATION\_OF\_BONE\_RESORPTION |  | 15 | -0.31 | -0.81 | 0.735 | 0.919 | 1.000 | 3308 | tags=53%, list=28%, signal=74% |
| 6491 | GOMF\_HEAT\_SHOCK\_PROTEIN\_BINDING |  | 91 | -0.23 | -0.81 | 0.813 | 0.920 | 1.000 | 3440 | tags=25%, list=29%, signal=35% |
| 6492 | GOBP\_NEGATIVE\_REGULATION\_OF\_SODIUM\_ION\_TRANSMEMBRANE\_TRANSPORT |  | 11 | -0.34 | -0.81 | 0.737 | 0.920 | 1.000 | 4140 | tags=64%, list=34%, signal=97% |
| 6493 | GOCC\_CATALYTIC\_STEP\_1\_SPLICEOSOME |  | 10 | -0.35 | -0.81 | 0.733 | 0.920 | 1.000 | 6476 | tags=60%, list=54%, signal=130% |
| 6494 | GOBP\_MUCOPOLYSACCHARIDE\_METABOLIC\_PROCESS |  | 87 | -0.23 | -0.81 | 0.810 | 0.920 | 1.000 | 2028 | tags=17%, list=17%, signal=21% |
| 6495 | GOBP\_PHOSPHATIDYLINOSITOL\_ACYL\_CHAIN\_REMODELING |  | 7 | -0.38 | -0.81 | 0.731 | 0.920 | 1.000 | 639 | tags=29%, list=5%, signal=30% |
| 6496 | GOCC\_TRANSCRIPTION\_PREINITIATION\_COMPLEX |  | 10 | -0.35 | -0.81 | 0.731 | 0.921 | 1.000 | 721 | tags=10%, list=6%, signal=11% |
| 6497 | GOMF\_BMP\_BINDING |  | 7 | -0.38 | -0.81 | 0.732 | 0.921 | 1.000 | 314 | tags=14%, list=3%, signal=15% |
| 6498 | GOBP\_AV\_NODE\_CELL\_TO\_BUNDLE\_OF\_HIS\_CELL\_COMMUNICATION |  | 7 | -0.38 | -0.81 | 0.741 | 0.921 | 1.000 | 3295 | tags=29%, list=27%, signal=39% |
| 6499 | GOBP\_NEGATIVE\_REGULATION\_OF\_EPITHELIAL\_CELL\_MIGRATION |  | 58 | -0.24 | -0.81 | 0.780 | 0.921 | 1.000 | 3392 | tags=29%, list=28%, signal=41% |
| 6500 | GOBP\_POSITIVE\_REGULATION\_OF\_MYOBLAST\_PROLIFERATION |  | 6 | -0.40 | -0.81 | 0.745 | 0.921 | 1.000 | 2905 | tags=50%, list=24%, signal=66% |
| 6501 | GOBP\_NEUTROPHIL\_APOPTOTIC\_PROCESS |  | 3 | -0.49 | -0.81 | 0.769 | 0.921 | 1.000 | 1677 | tags=67%, list=14%, signal=77% |
| 6502 | GOCC\_BOX\_H\_ACA\_RNP\_COMPLEX |  | 4 | -0.45 | -0.81 | 0.756 | 0.922 | 1.000 | 1122 | tags=25%, list=9%, signal=28% |
| 6503 | GOBP\_NUCLEAR\_ENVELOPE\_ORGANIZATION |  | 41 | -0.25 | -0.81 | 0.762 | 0.922 | 1.000 | 4770 | tags=37%, list=40%, signal=60% |
| 6504 | GOMF\_ORGANIC\_ACID\_BINDING |  | 62 | -0.24 | -0.81 | 0.782 | 0.922 | 1.000 | 3789 | tags=39%, list=32%, signal=56% |
| 6505 | GOBP\_MORPHOGENESIS\_OF\_AN\_EPITHELIAL\_BUD |  | 12 | -0.33 | -0.81 | 0.735 | 0.922 | 1.000 | 1146 | tags=17%, list=10%, signal=18% |
| 6506 | GOMF\_SINGLE\_STRANDED\_RNA\_BINDING |  | 66 | -0.24 | -0.81 | 0.793 | 0.922 | 1.000 | 1820 | tags=17%, list=15%, signal=20% |
| 6507 | GOBP\_PYRIDINE\_CONTAINING\_COMPOUND\_CATABOLIC\_PROCESS |  | 3 | -0.49 | -0.81 | 0.767 | 0.922 | 1.000 | 2865 | tags=33%, list=24%, signal=44% |
| 6508 | GOBP\_VASCULAR\_WOUND\_HEALING |  | 14 | -0.32 | -0.81 | 0.744 | 0.922 | 1.000 | 3205 | tags=36%, list=27%, signal=49% |
| 6509 | GOMF\_INOSITOL\_1\_3\_4\_5\_TETRAKISPHOSPHATE\_5\_PHOSPHATASE\_ACTIVITY |  | 5 | -0.42 | -0.81 | 0.748 | 0.922 | 1.000 | 567 | tags=20%, list=5%, signal=21% |
| 6510 | GOMF\_BOX\_H\_ACA\_SNORNA\_BINDING |  | 4 | -0.45 | -0.81 | 0.750 | 0.922 | 1.000 | 1122 | tags=25%, list=9%, signal=28% |
| 6511 | GOBP\_SECRETION\_OF\_LYSOSOMAL\_ENZYMES |  | 4 | -0.45 | -0.81 | 0.748 | 0.922 | 1.000 | 5536 | tags=75%, list=46%, signal=139% |
| 6512 | GOCC\_CLATHRIN\_COATED\_ENDOCYTIC\_VESICLE\_MEMBRANE |  | 17 | -0.30 | -0.81 | 0.745 | 0.922 | 1.000 | 4775 | tags=59%, list=40%, signal=98% |
| 6513 | GOBP\_TRANSCRIPTION\_BY\_RNA\_POLYMERASE\_I |  | 48 | -0.25 | -0.81 | 0.779 | 0.922 | 1.000 | 4647 | tags=42%, list=39%, signal=68% |
| 6514 | GOBP\_CELLULAR\_HYPEROSMOTIC\_SALINITY\_RESPONSE |  | 6 | -0.39 | -0.81 | 0.744 | 0.922 | 1.000 | 4353 | tags=67%, list=36%, signal=105% |
| 6515 | GOCC\_GATOR1\_COMPLEX |  | 6 | -0.40 | -0.81 | 0.742 | 0.922 | 1.000 | 3425 | tags=33%, list=29%, signal=47% |
| 6516 | GOBP\_HISTONE\_H3\_K27\_METHYLATION |  | 11 | -0.34 | -0.81 | 0.730 | 0.922 | 1.000 | 4362 | tags=55%, list=36%, signal=86% |
| 6517 | GOMF\_GTPASE\_INHIBITOR\_ACTIVITY |  | 9 | -0.36 | -0.81 | 0.744 | 0.922 | 1.000 | 3527 | tags=33%, list=29%, signal=47% |
| 6518 | GOCC\_NON\_MOTILE\_CILIUM |  | 77 | -0.23 | -0.81 | 0.807 | 0.923 | 1.000 | 4590 | tags=38%, list=38%, signal=61% |
| 6519 | GOMF\_INTERMEDIATE\_FILAMENT\_BINDING |  | 8 | -0.37 | -0.81 | 0.746 | 0.923 | 1.000 | 2656 | tags=25%, list=22%, signal=32% |
| 6520 | GOCC\_BOX\_H\_ACA\_SNORNP\_COMPLEX |  | 4 | -0.45 | -0.81 | 0.768 | 0.924 | 1.000 | 1122 | tags=25%, list=9%, signal=28% |
| 6521 | GOBP\_PROTEIN\_LOCALIZATION\_TO\_BICELLULAR\_TIGHT\_JUNCTION |  | 3 | -0.48 | -0.81 | 0.763 | 0.924 | 1.000 | 1996 | tags=33%, list=17%, signal=40% |
| 6522 | GOMF\_CERAMIDE\_BINDING |  | 12 | -0.33 | -0.81 | 0.727 | 0.924 | 1.000 | 5276 | tags=67%, list=44%, signal=119% |
| 6523 | GOBP\_NEGATIVE\_REGULATION\_OF\_CALCIUM\_ION\_TRANSMEMBRANE\_TRANSPORTER\_ACTIVITY |  | 20 | -0.29 | -0.81 | 0.743 | 0.924 | 1.000 | 2291 | tags=15%, list=19%, signal=19% |
| 6524 | GOMF\_SINGLE\_STRANDED\_DNA\_ENDODEOXYRIBONUCLEASE\_ACTIVITY |  | 8 | -0.37 | -0.81 | 0.732 | 0.924 | 1.000 | 5296 | tags=63%, list=44%, signal=112% |
| 6525 | GOBP\_STEROL\_BIOSYNTHETIC\_PROCESS |  | 58 | -0.24 | -0.81 | 0.791 | 0.924 | 1.000 | 4665 | tags=41%, list=39%, signal=67% |
| 6526 | GOMF\_5\_3\_EXODEOXYRIBONUCLEASE\_ACTIVITY |  | 6 | -0.40 | -0.81 | 0.741 | 0.924 | 1.000 | 4024 | tags=50%, list=34%, signal=75% |
| 6527 | GOBP\_LIPID\_CATABOLIC\_PROCESS |  | 229 | -0.21 | -0.81 | 0.892 | 0.924 | 1.000 | 4103 | tags=35%, list=34%, signal=52% |
| 6528 | GOBP\_RNA\_POLYMERASE\_I\_PREINITIATION\_COMPLEX\_ASSEMBLY |  | 6 | -0.40 | -0.81 | 0.738 | 0.924 | 1.000 | 5712 | tags=50%, list=48%, signal=95% |
| 6529 | GOBP\_REGULATION\_OF\_MITOTIC\_SPINDLE\_ASSEMBLY |  | 14 | -0.32 | -0.81 | 0.734 | 0.924 | 1.000 | 1818 | tags=21%, list=15%, signal=25% |
| 6530 | GOBP\_CELLULAR\_PROTEIN\_CONTAINING\_COMPLEX\_ASSEMBLY |  | 711 | -0.20 | -0.81 | 0.987 | 0.924 | 1.000 | 3088 | tags=23%, list=26%, signal=30% |
| 6531 | GOCC\_STAGA\_COMPLEX |  | 12 | -0.33 | -0.81 | 0.731 | 0.924 | 1.000 | 3626 | tags=33%, list=30%, signal=48% |
| 6532 | GOBP\_OLIGODENDROCYTE\_DIFFERENTIATION |  | 65 | -0.24 | -0.81 | 0.803 | 0.924 | 1.000 | 1968 | tags=17%, list=16%, signal=20% |
| 6533 | GOMF\_CHLORIDE\_ION\_BINDING |  | 6 | -0.40 | -0.81 | 0.757 | 0.924 | 1.000 | 4995 | tags=67%, list=42%, signal=114% |
| 6534 | GOMF\_DICARBOXYLIC\_ACID\_TRANSMEMBRANE\_TRANSPORTER\_ACTIVITY |  | 22 | -0.28 | -0.81 | 0.750 | 0.924 | 1.000 | 1802 | tags=23%, list=15%, signal=27% |
| 6535 | GOBP\_NEGATIVE\_REGULATION\_OF\_NATURAL\_KILLER\_CELL\_ACTIVATION |  | 4 | -0.44 | -0.81 | 0.761 | 0.924 | 1.000 | 3009 | tags=50%, list=25%, signal=67% |
| 6536 | GOMF\_ENDONUCLEASE\_ACTIVITY |  | 73 | -0.23 | -0.81 | 0.810 | 0.924 | 1.000 | 5296 | tags=48%, list=44%, signal=85% |
| 6537 | GOBP\_POSITIVE\_REGULATION\_OF\_CYCLIC\_NUCLEOTIDE\_PHOSPHODIESTERASE\_ACTIVITY |  | 3 | -0.48 | -0.81 | 0.765 | 0.924 | 1.000 | 4627 | tags=67%, list=39%, signal=108% |
| 6538 | GOMF\_N\_ACETYLLACTOSAMINIDE\_BETA\_1\_3\_N\_ACETYLGLUCOSAMINYLTRANSFERASE\_ACTIVITY |  | 6 | -0.39 | -0.81 | 0.745 | 0.924 | 1.000 | 339 | tags=17%, list=3%, signal=17% |
| 6539 | GOBP\_KERATINOCYTE\_DIFFERENTIATION |  | 75 | -0.23 | -0.81 | 0.810 | 0.925 | 1.000 | 3195 | tags=27%, list=27%, signal=36% |
| 6540 | GOBP\_REGULATION\_OF\_PROTEIN\_LIPIDATION |  | 6 | -0.40 | -0.81 | 0.735 | 0.925 | 1.000 | 5672 | tags=67%, list=47%, signal=126% |
| 6541 | GOBP\_RECOGNITION\_OF\_APOPTOTIC\_CELL |  | 5 | -0.42 | -0.81 | 0.753 | 0.926 | 1.000 | 1158 | tags=20%, list=10%, signal=22% |
| 6542 | GOBP\_MODULATION\_BY\_SYMBIONT\_OF\_HOST\_CELLULAR\_PROCESS |  | 19 | -0.29 | -0.81 | 0.745 | 0.926 | 1.000 | 4866 | tags=58%, list=41%, signal=97% |
| 6543 | GOBP\_POSITIVE\_REGULATION\_OF\_MONOOXYGENASE\_ACTIVITY |  | 23 | -0.28 | -0.81 | 0.756 | 0.926 | 1.000 | 4846 | tags=43%, list=40%, signal=73% |
| 6544 | GOCC\_PRESYNAPTIC\_ACTIVE\_ZONE\_CYTOPLASMIC\_COMPONENT |  | 10 | -0.35 | -0.81 | 0.748 | 0.926 | 1.000 | 2024 | tags=40%, list=17%, signal=48% |
| 6545 | GOBP\_POSITIVE\_REGULATION\_OF\_ANIMAL\_ORGAN\_MORPHOGENESIS |  | 21 | -0.29 | -0.81 | 0.754 | 0.925 | 1.000 | 1459 | tags=19%, list=12%, signal=22% |
| 6546 | GOMF\_GPI\_ANCHOR\_BINDING |  | 5 | -0.42 | -0.81 | 0.763 | 0.926 | 1.000 | 4057 | tags=40%, list=34%, signal=60% |
| 6547 | GOCC\_GOLGI\_CISTERNA |  | 76 | -0.23 | -0.80 | 0.821 | 0.926 | 1.000 | 4554 | tags=41%, list=38%, signal=65% |
| 6548 | GOBP\_REGULATION\_OF\_INTRINSIC\_APOPTOTIC\_SIGNALING\_PATHWAY |  | 108 | -0.22 | -0.80 | 0.847 | 0.926 | 1.000 | 3692 | tags=32%, list=31%, signal=46% |
| 6549 | GOMF\_RNA\_HELICASE\_ACTIVITY |  | 54 | -0.24 | -0.80 | 0.791 | 0.926 | 1.000 | 4717 | tags=44%, list=39%, signal=73% |
| 6550 | GOBP\_REGULATION\_OF\_RIBONUCLEOPROTEIN\_COMPLEX\_LOCALIZATION |  | 6 | -0.39 | -0.80 | 0.754 | 0.926 | 1.000 | 4278 | tags=50%, list=36%, signal=78% |
| 6551 | GOBP\_PROTEOGLYCAN\_METABOLIC\_PROCESS |  | 66 | -0.24 | -0.80 | 0.802 | 0.926 | 1.000 | 3719 | tags=36%, list=31%, signal=52% |
| 6552 | GOMF\_RNA\_2\_O\_METHYLTRANSFERASE\_ACTIVITY |  | 3 | -0.48 | -0.80 | 0.781 | 0.926 | 1.000 | 3494 | tags=67%, list=29%, signal=94% |
| 6553 | GOCC\_VESICLE\_TETHERING\_COMPLEX |  | 60 | -0.24 | -0.80 | 0.802 | 0.926 | 1.000 | 4346 | tags=35%, list=36%, signal=55% |
| 6554 | GOBP\_SUPEROXIDE\_METABOLIC\_PROCESS |  | 44 | -0.25 | -0.80 | 0.781 | 0.926 | 1.000 | 2706 | tags=25%, list=23%, signal=32% |
| 6555 | GOMF\_COPPER\_ION\_BINDING |  | 40 | -0.25 | -0.80 | 0.780 | 0.926 | 1.000 | 4975 | tags=38%, list=41%, signal=64% |
| 6556 | GOBP\_HISTONE\_H3\_K36\_TRIMETHYLATION |  | 4 | -0.45 | -0.80 | 0.762 | 0.926 | 1.000 | 6656 | tags=100%, list=55%, signal=224% |
| 6557 | GOMF\_PROTEIN\_ANTIGEN\_BINDING |  | 3 | -0.48 | -0.80 | 0.785 | 0.927 | 1.000 | 1646 | tags=33%, list=14%, signal=39% |
| 6558 | GOBP\_NEGATIVE\_REGULATION\_OF\_ANTIGEN\_PROCESSING\_AND\_PRESENTATION |  | 3 | -0.48 | -0.80 | 0.786 | 0.927 | 1.000 | 4776 | tags=67%, list=40%, signal=111% |
| 6559 | GOBP\_NEGATIVE\_REGULATION\_OF\_BLOOD\_VESSEL\_ENDOTHELIAL\_CELL\_MIGRATION |  | 29 | -0.27 | -0.80 | 0.769 | 0.927 | 1.000 | 3158 | tags=28%, list=26%, signal=37% |
| 6560 | GOBP\_LIPID\_GLYCOSYLATION |  | 4 | -0.44 | -0.80 | 0.773 | 0.927 | 1.000 | 1051 | tags=25%, list=9%, signal=27% |
| 6561 | GOMF\_ATP\_DEPENDENT\_MICROTUBULE\_MOTOR\_ACTIVITY\_MINUS\_END\_DIRECTED |  | 7 | -0.38 | -0.80 | 0.736 | 0.927 | 1.000 | 5065 | tags=71%, list=42%, signal=123% |
| 6562 | GOBP\_RESPONSE\_TO\_LEPTIN |  | 19 | -0.29 | -0.80 | 0.750 | 0.926 | 1.000 | 3392 | tags=37%, list=28%, signal=51% |
| 6563 | GOBP\_POSITIVE\_REGULATION\_OF\_MEMBRANE\_POTENTIAL |  | 10 | -0.35 | -0.80 | 0.744 | 0.926 | 1.000 | 4110 | tags=60%, list=34%, signal=91% |
| 6564 | GOCC\_MITOTIC\_COHESIN\_COMPLEX |  | 3 | -0.48 | -0.80 | 0.781 | 0.926 | 1.000 | 4465 | tags=67%, list=37%, signal=106% |
| 6565 | GOCC\_REPLISOME |  | 19 | -0.29 | -0.80 | 0.738 | 0.926 | 1.000 | 3402 | tags=32%, list=28%, signal=44% |
| 6566 | GOMF\_NUCLEOTIDYLTRANSFERASE\_ACTIVITY |  | 85 | -0.22 | -0.80 | 0.822 | 0.926 | 1.000 | 1929 | tags=16%, list=16%, signal=19% |
| 6567 | GOBP\_NUCLEOTIDE\_TRANSPORT |  | 22 | -0.28 | -0.80 | 0.753 | 0.926 | 1.000 | 3277 | tags=32%, list=27%, signal=44% |
| 6568 | GOMF\_VIRION\_BINDING |  | 6 | -0.39 | -0.80 | 0.751 | 0.926 | 1.000 | 3126 | tags=33%, list=26%, signal=45% |
| 6569 | GOBP\_RENAL\_VESICLE\_FORMATION |  | 7 | -0.38 | -0.80 | 0.746 | 0.927 | 1.000 | 6550 | tags=57%, list=55%, signal=126% |
| 6570 | GOMF\_CORECEPTOR\_ACTIVITY\_INVOLVED\_IN\_WNT\_SIGNALING\_PATHWAY\_PLANAR\_CELL\_POLARITY\_PATHWAY |  | 5 | -0.41 | -0.80 | 0.757 | 0.927 | 1.000 | 5283 | tags=60%, list=44%, signal=107% |
| 6571 | GOMF\_CALCITONIN\_FAMILY\_RECEPTOR\_ACTIVITY |  | 5 | -0.42 | -0.80 | 0.770 | 0.927 | 1.000 | 3134 | tags=40%, list=26%, signal=54% |
| 6572 | GOBP\_CALMODULIN\_DEPENDENT\_KINASE\_SIGNALING\_PATHWAY |  | 6 | -0.39 | -0.80 | 0.755 | 0.927 | 1.000 | 4317 | tags=67%, list=36%, signal=104% |
| 6573 | GOMF\_IMMUNOGLOBULIN\_RECEPTOR\_BINDING |  | 4 | -0.44 | -0.80 | 0.772 | 0.927 | 1.000 | 5687 | tags=75%, list=47%, signal=142% |
| 6574 | GOBP\_IODIDE\_TRANSPORT |  | 3 | -0.48 | -0.80 | 0.789 | 0.927 | 1.000 | 1743 | tags=33%, list=15%, signal=39% |
| 6575 | GOBP\_PROTEIN\_K63\_LINKED\_DEUBIQUITINATION |  | 23 | -0.28 | -0.80 | 0.765 | 0.928 | 1.000 | 5867 | tags=52%, list=49%, signal=102% |
| 6576 | GOBP\_REGULATION\_OF\_AMYLOID\_BETA\_CLEARANCE |  | 10 | -0.34 | -0.80 | 0.743 | 0.928 | 1.000 | 95 | tags=10%, list=1%, signal=10% |
| 6577 | GOBP\_POSITIVE\_REGULATION\_OF\_RECEPTOR\_INTERNALIZATION |  | 19 | -0.29 | -0.80 | 0.750 | 0.928 | 1.000 | 3538 | tags=42%, list=29%, signal=60% |
| 6578 | GOMF\_PROTEIN\_METHYLTRANSFERASE\_ACTIVITY |  | 58 | -0.24 | -0.80 | 0.803 | 0.928 | 1.000 | 4608 | tags=41%, list=38%, signal=67% |
| 6579 | GOMF\_TRANSFERASE\_ACTIVITY\_TRANSFERRING\_ONE\_CARBON\_GROUPS |  | 158 | -0.21 | -0.80 | 0.880 | 0.928 | 1.000 | 4371 | tags=34%, list=36%, signal=53% |
| 6580 | GOBP\_POSITIVE\_REGULATION\_OF\_PROTEIN\_LOCALIZATION\_TO\_CELL\_CORTEX |  | 5 | -0.41 | -0.80 | 0.766 | 0.928 | 1.000 | 6044 | tags=60%, list=50%, signal=121% |
| 6581 | GOBP\_REGULATION\_OF\_MEMBRANE\_INVAGINATION |  | 13 | -0.32 | -0.80 | 0.745 | 0.928 | 1.000 | 4317 | tags=54%, list=36%, signal=84% |
| 6582 | GOBP\_INTRINSIC\_APOPTOTIC\_SIGNALING\_PATHWAY\_IN\_RESPONSE\_TO\_DNA\_DAMAGE\_BY\_P53\_CLASS\_MEDIATOR |  | 26 | -0.27 | -0.80 | 0.761 | 0.928 | 1.000 | 4660 | tags=50%, list=39%, signal=82% |
| 6583 | GOCC\_CILIARY\_PLASM |  | 58 | -0.24 | -0.80 | 0.801 | 0.929 | 1.000 | 2186 | tags=21%, list=18%, signal=25% |
| 6584 | GOBP\_REDUCTION\_OF\_FOOD\_INTAKE\_IN\_RESPONSE\_TO\_DIETARY\_EXCESS |  | 3 | -0.48 | -0.80 | 0.785 | 0.928 | 1.000 | 1719 | tags=67%, list=14%, signal=78% |
| 6585 | GOMF\_CHLORIDE\_CHANNEL\_INHIBITOR\_ACTIVITY |  | 7 | -0.37 | -0.80 | 0.748 | 0.928 | 1.000 | 2955 | tags=43%, list=25%, signal=57% |
| 6586 | GOBP\_POSITIVE\_REGULATION\_OF\_DNA\_BINDING |  | 35 | -0.26 | -0.80 | 0.788 | 0.928 | 1.000 | 1455 | tags=17%, list=12%, signal=19% |
| 6587 | GOBP\_NEGATIVE\_REGULATION\_OF\_MONOCYTE\_CHEMOTACTIC\_PROTEIN\_1\_PRODUCTION |  | 4 | -0.44 | -0.80 | 0.784 | 0.929 | 1.000 | 6091 | tags=75%, list=51%, signal=152% |
| 6588 | GOBP\_NEGATIVE\_REGULATION\_OF\_INTRACELLULAR\_TRANSPORT |  | 43 | -0.25 | -0.80 | 0.788 | 0.929 | 1.000 | 2763 | tags=21%, list=23%, signal=27% |
| 6589 | GOBP\_ENDOCYTIC\_RECYCLING |  | 35 | -0.26 | -0.80 | 0.784 | 0.929 | 1.000 | 1202 | tags=11%, list=10%, signal=13% |
| 6590 | GOBP\_NEGATIVE\_REGULATION\_OF\_TYPE\_2\_IMMUNE\_RESPONSE |  | 8 | -0.36 | -0.80 | 0.746 | 0.929 | 1.000 | 1788 | tags=38%, list=15%, signal=44% |
| 6591 | GOBP\_RESPONSE\_TO\_NITROSATIVE\_STRESS |  | 7 | -0.38 | -0.80 | 0.760 | 0.928 | 1.000 | 1667 | tags=29%, list=14%, signal=33% |
| 6592 | GOBP\_REGULATION\_OF\_TRANSLATIONAL\_TERMINATION |  | 7 | -0.38 | -0.80 | 0.750 | 0.928 | 1.000 | 4437 | tags=43%, list=37%, signal=68% |
| 6593 | GOBP\_IMP\_METABOLIC\_PROCESS |  | 9 | -0.35 | -0.80 | 0.748 | 0.928 | 1.000 | 129 | tags=11%, list=1%, signal=11% |
| 6594 | GOBP\_POSITIVE\_REGULATION\_OF\_CALCIUM\_ION\_TRANSPORT\_INTO\_CYTOSOL |  | 36 | -0.26 | -0.80 | 0.781 | 0.930 | 1.000 | 4738 | tags=44%, list=39%, signal=73% |
| 6595 | GOBP\_REGULATION\_OF\_OSTEOBLAST\_DIFFERENTIATION |  | 82 | -0.23 | -0.80 | 0.827 | 0.930 | 1.000 | 3389 | tags=29%, list=28%, signal=40% |
| 6596 | GOBP\_PHOSPHATIDYLSERINE\_EXPOSURE\_ON\_APOPTOTIC\_CELL\_SURFACE |  | 5 | -0.41 | -0.80 | 0.765 | 0.930 | 1.000 | 135 | tags=20%, list=1%, signal=20% |
| 6597 | GOBP\_MESONEPHRIC\_TUBULE\_MORPHOGENESIS |  | 40 | -0.25 | -0.80 | 0.785 | 0.930 | 1.000 | 2008 | tags=20%, list=17%, signal=24% |
| 6598 | GOBP\_RESPONSE\_TO\_INTERLEUKIN\_9 |  | 7 | -0.37 | -0.80 | 0.757 | 0.930 | 1.000 | 960 | tags=29%, list=8%, signal=31% |
| 6599 | GOBP\_NEGATIVE\_REGULATION\_OF\_DNA\_TEMPLATED\_TRANSCRIPTION\_INITIATION |  | 6 | -0.39 | -0.80 | 0.765 | 0.930 | 1.000 | 4649 | tags=67%, list=39%, signal=109% |
| 6600 | GOBP\_RUFFLE\_ASSEMBLY |  | 28 | -0.27 | -0.80 | 0.778 | 0.930 | 1.000 | 4018 | tags=39%, list=33%, signal=59% |
| 6601 | GOBP\_VENTRICULAR\_COMPACT\_MYOCARDIUM\_MORPHOGENESIS |  | 6 | -0.39 | -0.80 | 0.769 | 0.930 | 1.000 | 4506 | tags=50%, list=38%, signal=80% |
| 6602 | GOBP\_XENOBIOTIC\_TRANSPORT |  | 30 | -0.26 | -0.80 | 0.774 | 0.930 | 1.000 | 3220 | tags=40%, list=27%, signal=55% |
| 6603 | GOBP\_REGULATION\_OF\_GLUTAMATE\_SECRETION |  | 7 | -0.38 | -0.80 | 0.757 | 0.930 | 1.000 | 801 | tags=29%, list=7%, signal=31% |
| 6604 | GOBP\_POSITIVE\_REGULATION\_OF\_VIRAL\_LIFE\_CYCLE |  | 21 | -0.28 | -0.80 | 0.760 | 0.930 | 1.000 | 1903 | tags=24%, list=16%, signal=28% |
| 6605 | GOBP\_REGULATION\_OF\_TRANSCRIPTION\_REGULATORY\_REGION\_DNA\_BINDING |  | 35 | -0.25 | -0.80 | 0.789 | 0.930 | 1.000 | 6186 | tags=66%, list=52%, signal=135% |
| 6606 | GOMF\_ENDORIBONUCLEASE\_ACTIVITY\_PRODUCING\_3\_PHOSPHOMONOESTERS |  | 3 | -0.48 | -0.80 | 0.794 | 0.930 | 1.000 | 3634 | tags=33%, list=30%, signal=48% |
| 6607 | GOBP\_POSITIVE\_REGULATION\_OF\_MEIOTIC\_CELL\_CYCLE |  | 10 | -0.34 | -0.80 | 0.753 | 0.930 | 1.000 | 5884 | tags=70%, list=49%, signal=137% |
| 6608 | GOBP\_COBALT\_ION\_TRANSPORT |  | 5 | -0.41 | -0.80 | 0.774 | 0.930 | 1.000 | 5420 | tags=80%, list=45%, signal=146% |
| 6609 | GOMF\_OXIDOREDUCTASE\_ACTIVITY\_ACTING\_ON\_A\_SULFUR\_GROUP\_OF\_DONORS\_DISULFIDE\_AS\_ACCEPTOR |  | 8 | -0.36 | -0.80 | 0.750 | 0.930 | 1.000 | 2523 | tags=25%, list=21%, signal=32% |
| 6610 | GOBP\_CELLULAR\_RESPONSE\_TO\_LIGHT\_STIMULUS |  | 82 | -0.23 | -0.80 | 0.838 | 0.930 | 1.000 | 3574 | tags=28%, list=30%, signal=40% |
| 6611 | GOBP\_ADULT\_HEART\_DEVELOPMENT |  | 7 | -0.37 | -0.80 | 0.751 | 0.930 | 1.000 | 5875 | tags=71%, list=49%, signal=140% |
| 6612 | GOBP\_AMINOGLYCAN\_METABOLIC\_PROCESS |  | 121 | -0.22 | -0.80 | 0.859 | 0.930 | 1.000 | 3613 | tags=30%, list=30%, signal=42% |
| 6613 | GOBP\_NUCLEOBASE\_CONTAINING\_COMPOUND\_TRANSPORT |  | 186 | -0.21 | -0.80 | 0.892 | 0.930 | 1.000 | 4080 | tags=31%, list=34%, signal=46% |
| 6614 | GOBP\_REGULATION\_OF\_STEM\_CELL\_DIVISION |  | 6 | -0.39 | -0.79 | 0.757 | 0.931 | 1.000 | 2184 | tags=33%, list=18%, signal=41% |
| 6615 | GOMF\_SOLUTE\_PROTON\_SYMPORTER\_ACTIVITY |  | 13 | -0.32 | -0.79 | 0.746 | 0.931 | 1.000 | 2258 | tags=31%, list=19%, signal=38% |
| 6616 | GOBP\_REGULATION\_OF\_TRIGLYCERIDE\_BIOSYNTHETIC\_PROCESS |  | 14 | -0.31 | -0.79 | 0.761 | 0.932 | 1.000 | 4853 | tags=50%, list=40%, signal=84% |
| 6617 | GOMF\_HMG\_BOX\_DOMAIN\_BINDING |  | 5 | -0.41 | -0.79 | 0.767 | 0.932 | 1.000 | 2646 | tags=40%, list=22%, signal=51% |
| 6618 | GOMF\_WD40\_REPEAT\_DOMAIN\_BINDING |  | 4 | -0.44 | -0.79 | 0.777 | 0.932 | 1.000 | 5922 | tags=75%, list=49%, signal=148% |
| 6619 | GOBP\_MODULATION\_BY\_SYMBIONT\_OF\_ENTRY\_INTO\_HOST |  | 19 | -0.29 | -0.79 | 0.759 | 0.932 | 1.000 | 2610 | tags=32%, list=22%, signal=40% |
| 6620 | GOBP\_POSITIVE\_REGULATION\_OF\_TRANSCRIPTION\_REGULATORY\_REGION\_DNA\_BINDING |  | 17 | -0.30 | -0.79 | 0.769 | 0.932 | 1.000 | 4184 | tags=41%, list=35%, signal=63% |
| 6621 | GOMF\_SCAVENGER\_RECEPTOR\_ACTIVITY |  | 26 | -0.27 | -0.79 | 0.768 | 0.932 | 1.000 | 4280 | tags=38%, list=36%, signal=60% |
| 6622 | GOBP\_REGULATION\_OF\_NEURON\_PROJECTION\_ARBORIZATION |  | 13 | -0.32 | -0.79 | 0.757 | 0.932 | 1.000 | 3125 | tags=38%, list=26%, signal=52% |
| 6623 | GOBP\_AROMATIC\_AMINO\_ACID\_TRANSPORT |  | 9 | -0.35 | -0.79 | 0.751 | 0.933 | 1.000 | 3155 | tags=33%, list=26%, signal=45% |
| 6624 | GOBP\_PHOSPHOLIPID\_CATABOLIC\_PROCESS |  | 35 | -0.25 | -0.79 | 0.782 | 0.933 | 1.000 | 1315 | tags=17%, list=11%, signal=19% |
| 6625 | GOBP\_POLY\_N\_ACETYLLACTOSAMINE\_METABOLIC\_PROCESS |  | 7 | -0.37 | -0.79 | 0.761 | 0.933 | 1.000 | 1968 | tags=29%, list=16%, signal=34% |
| 6626 | GOMF\_UBIQUITIN\_LIKE\_MODIFIER\_ACTIVATING\_ENZYME\_ACTIVITY |  | 8 | -0.36 | -0.79 | 0.745 | 0.933 | 1.000 | 6245 | tags=75%, list=52%, signal=156% |
| 6627 | GOBP\_IMP\_BIOSYNTHETIC\_PROCESS |  | 8 | -0.36 | -0.79 | 0.752 | 0.933 | 1.000 | 129 | tags=13%, list=1%, signal=13% |
| 6628 | GOBP\_PATTERN\_SPECIFICATION\_INVOLVED\_IN\_KIDNEY\_DEVELOPMENT |  | 3 | -0.47 | -0.79 | 0.789 | 0.933 | 1.000 | 1100 | tags=33%, list=9%, signal=37% |
| 6629 | GOBP\_GMP\_METABOLIC\_PROCESS |  | 12 | -0.32 | -0.79 | 0.755 | 0.933 | 1.000 | 2604 | tags=42%, list=22%, signal=53% |
| 6630 | GOBP\_NEGATIVE\_REGULATION\_OF\_FAT\_CELL\_PROLIFERATION |  | 4 | -0.44 | -0.79 | 0.790 | 0.934 | 1.000 | 5711 | tags=75%, list=48%, signal=143% |
| 6631 | GOMF\_N\_ACETYLGALACTOSAMINE\_4\_O\_SULFOTRANSFERASE\_ACTIVITY |  | 3 | -0.47 | -0.79 | 0.783 | 0.933 | 1.000 | 2404 | tags=67%, list=20%, signal=83% |
| 6632 | GOCC\_ACROSOMAL\_VESICLE |  | 49 | -0.24 | -0.79 | 0.810 | 0.934 | 1.000 | 3189 | tags=29%, list=27%, signal=39% |
| 6633 | GOBP\_NEGATIVE\_REGULATION\_OF\_POTASSIUM\_ION\_TRANSMEMBRANE\_TRANSPORT |  | 15 | -0.31 | -0.79 | 0.755 | 0.934 | 1.000 | 4139 | tags=47%, list=34%, signal=71% |
| 6634 | GOBP\_RENAL\_SYSTEM\_VASCULATURE\_MORPHOGENESIS |  | 6 | -0.39 | -0.79 | 0.765 | 0.934 | 1.000 | 5330 | tags=67%, list=44%, signal=120% |
| 6635 | GOMF\_OXIDOREDUCTASE\_ACTIVITY\_ACTING\_ON\_THE\_CH\_NH\_GROUP\_OF\_DONORS\_NAD\_OR\_NADP\_AS\_ACCEPTOR |  | 12 | -0.32 | -0.79 | 0.757 | 0.934 | 1.000 | 4242 | tags=50%, list=35%, signal=77% |
| 6636 | GOBP\_NEGATIVE\_REGULATION\_OF\_CARDIAC\_MUSCLE\_CELL\_DIFFERENTIATION |  | 5 | -0.41 | -0.79 | 0.778 | 0.934 | 1.000 | 1881 | tags=40%, list=16%, signal=47% |
| 6637 | GOMF\_PROTEIN\_DEACETYLASE\_ACTIVITY |  | 19 | -0.28 | -0.79 | 0.760 | 0.935 | 1.000 | 2717 | tags=21%, list=23%, signal=27% |
| 6638 | GOMF\_3\_5\_EXODEOXYRIBONUCLEASE\_ACTIVITY |  | 3 | -0.48 | -0.79 | 0.796 | 0.935 | 1.000 | 1545 | tags=33%, list=13%, signal=38% |
| 6639 | GOBP\_POSITIVE\_REGULATION\_OF\_CALCIUM\_ION\_TRANSMEMBRANE\_TRANSPORT |  | 47 | -0.24 | -0.79 | 0.803 | 0.935 | 1.000 | 4738 | tags=43%, list=39%, signal=70% |
| 6640 | GOBP\_PROGRAMMED\_CELL\_DEATH\_IN\_RESPONSE\_TO\_REACTIVE\_OXYGEN\_SPECIES |  | 8 | -0.36 | -0.79 | 0.760 | 0.935 | 1.000 | 1151 | tags=13%, list=10%, signal=14% |
| 6641 | GOBP\_MITOTIC\_METAPHASE\_PLATE\_CONGRESSION |  | 41 | -0.25 | -0.79 | 0.787 | 0.935 | 1.000 | 3970 | tags=32%, list=33%, signal=47% |
| 6642 | GOBP\_COPULATION |  | 13 | -0.31 | -0.79 | 0.760 | 0.935 | 1.000 | 4984 | tags=69%, list=41%, signal=118% |
| 6643 | GOMF\_REPRESSING\_TRANSCRIPTION\_FACTOR\_BINDING |  | 57 | -0.23 | -0.79 | 0.817 | 0.935 | 1.000 | 3235 | tags=26%, list=27%, signal=36% |
| 6644 | GOBP\_POSITIVE\_REGULATION\_OF\_VASCULOGENESIS |  | 10 | -0.33 | -0.79 | 0.761 | 0.935 | 1.000 | 5825 | tags=60%, list=48%, signal=116% |
| 6645 | GOBP\_REGULATION\_OF\_TELOMERASE\_ACTIVITY |  | 39 | -0.25 | -0.79 | 0.798 | 0.935 | 1.000 | 2101 | tags=21%, list=17%, signal=25% |
| 6646 | GOMF\_LIGASE\_ACTIVITY |  | 104 | -0.22 | -0.79 | 0.863 | 0.936 | 1.000 | 4245 | tags=32%, list=35%, signal=49% |
| 6647 | GOBP\_ORGANELLE\_DISASSEMBLY |  | 86 | -0.22 | -0.79 | 0.848 | 0.936 | 1.000 | 5874 | tags=53%, list=49%, signal=104% |
| 6648 | GOBP\_REGULATION\_OF\_SYSTEMIC\_ARTERIAL\_BLOOD\_PRESSURE\_MEDIATED\_BY\_A\_CHEMICAL\_SIGNAL |  | 31 | -0.26 | -0.79 | 0.793 | 0.936 | 1.000 | 2480 | tags=26%, list=21%, signal=32% |
| 6649 | GOBP\_GLYCOSYL\_COMPOUND\_CATABOLIC\_PROCESS |  | 28 | -0.26 | -0.79 | 0.797 | 0.936 | 1.000 | 2073 | tags=21%, list=17%, signal=26% |
| 6650 | GOBP\_PURINE\_NUCLEOTIDE\_TRANSPORT |  | 18 | -0.29 | -0.79 | 0.775 | 0.936 | 1.000 | 2605 | tags=28%, list=22%, signal=35% |
| 6651 | GOBP\_POSITIVE\_REGULATION\_OF\_PHAGOCYTOSIS\_ENGULFMENT |  | 12 | -0.32 | -0.79 | 0.768 | 0.936 | 1.000 | 4317 | tags=58%, list=36%, signal=91% |
| 6652 | GOBP\_DENDRITIC\_CELL\_CYTOKINE\_PRODUCTION |  | 8 | -0.36 | -0.79 | 0.757 | 0.936 | 1.000 | 1230 | tags=25%, list=10%, signal=28% |
| 6653 | GOMF\_TRANSCRIPTION\_COREPRESSOR\_BINDING |  | 20 | -0.28 | -0.79 | 0.777 | 0.936 | 1.000 | 6456 | tags=70%, list=54%, signal=151% |
| 6654 | GOCC\_PHAGOLYSOSOME |  | 5 | -0.41 | -0.79 | 0.785 | 0.936 | 1.000 | 323 | tags=20%, list=3%, signal=21% |
| 6655 | GOBP\_ENAMEL\_MINERALIZATION |  | 9 | -0.35 | -0.79 | 0.761 | 0.936 | 1.000 | 2515 | tags=33%, list=21%, signal=42% |
| 6656 | GOBP\_REGULATION\_OF\_OXIDATIVE\_STRESS\_INDUCED\_NEURON\_DEATH |  | 14 | -0.31 | -0.79 | 0.773 | 0.936 | 1.000 | 3675 | tags=36%, list=31%, signal=51% |
| 6657 | GOBP\_NUCLEOBASE\_CONTAINING\_SMALL\_MOLECULE\_BIOSYNTHETIC\_PROCESS |  | 87 | -0.22 | -0.79 | 0.856 | 0.936 | 1.000 | 3094 | tags=24%, list=26%, signal=32% |
| 6658 | GOBP\_FIBROBLAST\_GROWTH\_FACTOR\_PRODUCTION |  | 4 | -0.44 | -0.79 | 0.780 | 0.936 | 1.000 | 2416 | tags=50%, list=20%, signal=63% |
| 6659 | GOBP\_POSITIVE\_REGULATION\_OF\_ER\_TO\_GOLGI\_VESICLE\_MEDIATED\_TRANSPORT |  | 5 | -0.41 | -0.79 | 0.787 | 0.936 | 1.000 | 2916 | tags=40%, list=24%, signal=53% |
| 6660 | GOMF\_GDP\_DISSOCIATION\_INHIBITOR\_ACTIVITY |  | 14 | -0.31 | -0.79 | 0.755 | 0.936 | 1.000 | 5890 | tags=50%, list=49%, signal=98% |
| 6661 | GOBP\_POSITIVE\_REGULATION\_OF\_TOLL\_LIKE\_RECEPTOR\_SIGNALING\_PATHWAY |  | 19 | -0.29 | -0.79 | 0.777 | 0.936 | 1.000 | 2731 | tags=37%, list=23%, signal=48% |
| 6662 | GOBP\_POSITIVE\_REGULATION\_OF\_UBIQUITIN\_PROTEIN\_TRANSFERASE\_ACTIVITY |  | 26 | -0.27 | -0.79 | 0.786 | 0.936 | 1.000 | 5705 | tags=46%, list=47%, signal=88% |
| 6663 | GOBP\_NEGATIVE\_REGULATION\_OF\_RNA\_SPLICING |  | 20 | -0.28 | -0.79 | 0.780 | 0.936 | 1.000 | 4223 | tags=35%, list=35%, signal=54% |
| 6664 | GOBP\_SULFUR\_COMPOUND\_TRANSPORT |  | 38 | -0.25 | -0.79 | 0.796 | 0.936 | 1.000 | 3705 | tags=39%, list=31%, signal=57% |
| 6665 | GOBP\_NEGATIVE\_REGULATION\_OF\_PROTEIN\_KINASE\_ACTIVITY\_BY\_REGULATION\_OF\_PROTEIN\_PHOSPHORYLATION |  | 8 | -0.36 | -0.79 | 0.768 | 0.936 | 1.000 | 4425 | tags=38%, list=37%, signal=59% |
| 6666 | GOBP\_POSITIVE\_REGULATION\_OF\_SIGNALING\_RECEPTOR\_ACTIVITY |  | 29 | -0.26 | -0.79 | 0.786 | 0.936 | 1.000 | 2560 | tags=28%, list=21%, signal=35% |
| 6667 | GOCC\_SCAR\_COMPLEX |  | 9 | -0.34 | -0.79 | 0.757 | 0.936 | 1.000 | 3797 | tags=56%, list=32%, signal=81% |
| 6668 | GOBP\_NEGATIVE\_REGULATION\_OF\_PROTEIN\_LOCALIZATION\_TO\_NUCLEUS |  | 23 | -0.27 | -0.79 | 0.779 | 0.936 | 1.000 | 3235 | tags=30%, list=27%, signal=42% |
| 6669 | GOBP\_RESPONSE\_TO\_ETHER |  | 7 | -0.37 | -0.79 | 0.769 | 0.936 | 1.000 | 3968 | tags=57%, list=33%, signal=85% |
| 6670 | GOBP\_NUCLEOSIDE\_MONOPHOSPHATE\_METABOLIC\_PROCESS |  | 59 | -0.23 | -0.79 | 0.825 | 0.936 | 1.000 | 4363 | tags=41%, list=36%, signal=64% |
| 6671 | GOBP\_DOUBLE\_STRAND\_BREAK\_REPAIR\_VIA\_SINGLE\_STRAND\_ANNEALING |  | 4 | -0.43 | -0.78 | 0.787 | 0.937 | 1.000 | 4387 | tags=75%, list=37%, signal=118% |
| 6672 | GOBP\_MACROPHAGE\_ACTIVATION\_INVOLVED\_IN\_IMMUNE\_RESPONSE |  | 12 | -0.32 | -0.78 | 0.760 | 0.937 | 1.000 | 4831 | tags=58%, list=40%, signal=97% |
| 6673 | GOBP\_ORNITHINE\_TRANSPORT |  | 5 | -0.41 | -0.78 | 0.778 | 0.937 | 1.000 | 1025 | tags=20%, list=9%, signal=22% |
| 6674 | GOBP\_COLUMNAR\_CUBOIDAL\_EPITHELIAL\_CELL\_DEVELOPMENT |  | 34 | -0.25 | -0.78 | 0.795 | 0.936 | 1.000 | 4432 | tags=35%, list=37%, signal=56% |
| 6675 | GOCC\_CHROMAFFIN\_GRANULE |  | 8 | -0.36 | -0.78 | 0.763 | 0.936 | 1.000 | 1279 | tags=25%, list=11%, signal=28% |
| 6676 | GOCC\_FICOLIN\_1\_RICH\_GRANULE\_MEMBRANE |  | 36 | -0.25 | -0.78 | 0.795 | 0.936 | 1.000 | 4268 | tags=42%, list=36%, signal=64% |
| 6677 | GOMF\_DNA\_APURINIC\_OR\_APYRIMIDINIC\_SITE\_ENDONUCLEASE\_ACTIVITY |  | 8 | -0.36 | -0.78 | 0.766 | 0.936 | 1.000 | 6904 | tags=75%, list=57%, signal=176% |
| 6678 | GOBP\_REGULATION\_OF\_NEUTROPHIL\_EXTRAVASATION |  | 6 | -0.39 | -0.78 | 0.774 | 0.936 | 1.000 | 2011 | tags=50%, list=17%, signal=60% |
| 6679 | GOBP\_INTESTINAL\_EPITHELIAL\_CELL\_DEVELOPMENT |  | 12 | -0.32 | -0.78 | 0.757 | 0.936 | 1.000 | 106 | tags=8%, list=1%, signal=8% |
| 6680 | GOCC\_PERICHROMATIN\_FIBRILS |  | 4 | -0.43 | -0.78 | 0.789 | 0.936 | 1.000 | 6831 | tags=100%, list=57%, signal=232% |
| 6681 | GOMF\_GAMMA\_AMINOBUTYRIC\_ACID\_TRANSMEMBRANE\_TRANSPORTER\_ACTIVITY |  | 4 | -0.43 | -0.78 | 0.783 | 0.937 | 1.000 | 421 | tags=25%, list=4%, signal=26% |
| 6682 | GOBP\_O\_GLYCAN\_PROCESSING |  | 34 | -0.25 | -0.78 | 0.797 | 0.936 | 1.000 | 2617 | tags=24%, list=22%, signal=30% |
| 6683 | GOBP\_NEGATIVE\_REGULATION\_OF\_CELL\_SIZE |  | 6 | -0.39 | -0.78 | 0.778 | 0.936 | 1.000 | 3613 | tags=67%, list=30%, signal=95% |
| 6684 | GOCC\_ENDOSOME\_MEMBRANE |  | 376 | -0.20 | -0.78 | 0.960 | 0.936 | 1.000 | 3353 | tags=26%, list=28%, signal=35% |
| 6685 | GOBP\_GLYCOSYLCERAMIDE\_BIOSYNTHETIC\_PROCESS |  | 6 | -0.39 | -0.78 | 0.774 | 0.936 | 1.000 | 2915 | tags=50%, list=24%, signal=66% |
| 6686 | GOBP\_NEGATIVE\_REGULATION\_OF\_LAMELLIPODIUM\_ORGANIZATION |  | 3 | -0.47 | -0.78 | 0.810 | 0.936 | 1.000 | 5674 | tags=67%, list=47%, signal=126% |
| 6687 | GOBP\_NEGATIVE\_REGULATION\_OF\_CILIUM\_ASSEMBLY |  | 11 | -0.32 | -0.78 | 0.763 | 0.936 | 1.000 | 5206 | tags=55%, list=43%, signal=96% |
| 6688 | GOBP\_POSITIVE\_REGULATION\_OF\_BINDING |  | 124 | -0.21 | -0.78 | 0.880 | 0.937 | 1.000 | 1764 | tags=15%, list=15%, signal=17% |
| 6689 | GOCC\_EARLY\_PHAGOSOME |  | 11 | -0.33 | -0.78 | 0.765 | 0.937 | 1.000 | 856 | tags=18%, list=7%, signal=20% |
| 6690 | GOBP\_MACROAUTOPHAGY |  | 256 | -0.20 | -0.78 | 0.934 | 0.937 | 1.000 | 4075 | tags=32%, list=34%, signal=47% |
| 6691 | GOCC\_CLATHRIN\_SCULPTED\_MONOAMINE\_TRANSPORT\_VESICLE |  | 5 | -0.41 | -0.78 | 0.774 | 0.937 | 1.000 | 2055 | tags=40%, list=17%, signal=48% |
| 6692 | GOBP\_NEGATIVE\_REGULATION\_OF\_GENE\_SILENCING |  | 16 | -0.29 | -0.78 | 0.773 | 0.937 | 1.000 | 1806 | tags=25%, list=15%, signal=29% |
| 6693 | GOMF\_ACETYL\_COA\_HYDROLASE\_ACTIVITY |  | 5 | -0.41 | -0.78 | 0.779 | 0.937 | 1.000 | 3207 | tags=40%, list=27%, signal=55% |
| 6694 | GOBP\_POSITIVE\_REGULATION\_OF\_NAD\_P\_H\_OXIDASE\_ACTIVITY |  | 5 | -0.41 | -0.78 | 0.788 | 0.937 | 1.000 | 3273 | tags=40%, list=27%, signal=55% |
| 6695 | GOBP\_POSITIVE\_REGULATION\_OF\_INHIBITORY\_POSTSYNAPTIC\_POTENTIAL |  | 4 | -0.44 | -0.78 | 0.792 | 0.937 | 1.000 | 2024 | tags=50%, list=17%, signal=60% |
| 6696 | GOBP\_TYPE\_B\_PANCREATIC\_CELL\_PROLIFERATION |  | 14 | -0.31 | -0.78 | 0.768 | 0.937 | 1.000 | 2267 | tags=36%, list=19%, signal=44% |
| 6697 | GOBP\_AUTOPHAGOSOME\_LYSOSOME\_FUSION |  | 4 | -0.43 | -0.78 | 0.793 | 0.937 | 1.000 | 6812 | tags=100%, list=57%, signal=231% |
| 6698 | GOCC\_FICOLIN\_1\_RICH\_GRANULE |  | 135 | -0.21 | -0.78 | 0.892 | 0.937 | 1.000 | 4343 | tags=34%, list=36%, signal=53% |
| 6699 | GOCC\_ENZYME\_ACTIVATOR\_COMPLEX |  | 5 | -0.40 | -0.78 | 0.790 | 0.937 | 1.000 | 3635 | tags=40%, list=30%, signal=57% |
| 6700 | GOBP\_GAP\_JUNCTION\_ASSEMBLY |  | 10 | -0.33 | -0.78 | 0.773 | 0.937 | 1.000 | 2313 | tags=40%, list=19%, signal=49% |
| 6701 | GOBP\_CELL\_VOLUME\_HOMEOSTASIS |  | 21 | -0.28 | -0.78 | 0.787 | 0.937 | 1.000 | 4895 | tags=52%, list=41%, signal=88% |
| 6702 | GOBP\_OVULATION\_FROM\_OVARIAN\_FOLLICLE |  | 4 | -0.43 | -0.78 | 0.796 | 0.937 | 1.000 | 2656 | tags=50%, list=22%, signal=64% |
| 6703 | GOBP\_FATTY\_ACID\_METABOLIC\_PROCESS |  | 258 | -0.20 | -0.78 | 0.936 | 0.937 | 1.000 | 4190 | tags=37%, list=35%, signal=56% |
| 6704 | GOBP\_REGULATION\_OF\_MEIOTIC\_NUCLEAR\_DIVISION |  | 14 | -0.31 | -0.78 | 0.764 | 0.937 | 1.000 | 306 | tags=7%, list=3%, signal=7% |
| 6705 | GOBP\_REGULATION\_OF\_PEPTIDYL\_TYROSINE\_AUTOPHOSPHORYLATION |  | 4 | -0.43 | -0.78 | 0.799 | 0.937 | 1.000 | 3400 | tags=50%, list=28%, signal=70% |
| 6706 | GOMF\_CLASS\_I\_DNA\_APURINIC\_OR\_APYRIMIDINIC\_SITE\_ENDONUCLEASE\_ACTIVITY |  | 5 | -0.40 | -0.78 | 0.786 | 0.938 | 1.000 | 4508 | tags=40%, list=38%, signal=64% |
| 6707 | GOMF\_UDP\_GALACTOSYLTRANSFERASE\_ACTIVITY |  | 13 | -0.31 | -0.78 | 0.765 | 0.938 | 1.000 | 2309 | tags=31%, list=19%, signal=38% |
| 6708 | GOBP\_LOW\_DENSITY\_LIPOPROTEIN\_PARTICLE\_CLEARANCE |  | 24 | -0.27 | -0.78 | 0.789 | 0.938 | 1.000 | 4801 | tags=50%, list=40%, signal=83% |
| 6709 | GOBP\_CHRONIC\_INFLAMMATORY\_RESPONSE |  | 10 | -0.34 | -0.78 | 0.762 | 0.938 | 1.000 | 2820 | tags=50%, list=23%, signal=65% |
| 6710 | GOBP\_MIRNA\_CATABOLIC\_PROCESS |  | 4 | -0.43 | -0.78 | 0.787 | 0.938 | 1.000 | 4799 | tags=50%, list=40%, signal=83% |
| 6711 | GOBP\_NEGATIVE\_REGULATION\_OF\_ACTIN\_FILAMENT\_DEPOLYMERIZATION |  | 33 | -0.25 | -0.78 | 0.799 | 0.938 | 1.000 | 2598 | tags=21%, list=22%, signal=27% |
| 6712 | GOBP\_CARDIAC\_EPITHELIAL\_TO\_MESENCHYMAL\_TRANSITION |  | 22 | -0.28 | -0.78 | 0.789 | 0.938 | 1.000 | 3530 | tags=32%, list=29%, signal=45% |
| 6713 | GOBP\_PROTEIN\_TRIMERIZATION |  | 12 | -0.32 | -0.78 | 0.773 | 0.938 | 1.000 | 1138 | tags=17%, list=9%, signal=18% |
| 6714 | GOCC\_XY\_BODY |  | 9 | -0.34 | -0.78 | 0.771 | 0.939 | 1.000 | 3137 | tags=33%, list=26%, signal=45% |
| 6715 | GOBP\_ERROR\_PRONE\_TRANSLESION\_SYNTHESIS |  | 19 | -0.28 | -0.78 | 0.781 | 0.939 | 1.000 | 5968 | tags=58%, list=50%, signal=115% |
| 6716 | GOBP\_STEROID\_CATABOLIC\_PROCESS |  | 16 | -0.30 | -0.78 | 0.777 | 0.939 | 1.000 | 4011 | tags=31%, list=33%, signal=47% |
| 6717 | GOBP\_SUCCINATE\_TRANSMEMBRANE\_TRANSPORT |  | 5 | -0.41 | -0.78 | 0.786 | 0.939 | 1.000 | 872 | tags=20%, list=7%, signal=22% |
| 6718 | GOBP\_RIBONUCLEOPROTEIN\_COMPLEX\_SUBUNIT\_ORGANIZATION |  | 150 | -0.21 | -0.78 | 0.899 | 0.939 | 1.000 | 3611 | tags=23%, list=30%, signal=32% |
| 6719 | GOBP\_ENDOTHELIAL\_CELL\_DEVELOPMENT |  | 52 | -0.23 | -0.78 | 0.828 | 0.939 | 1.000 | 4848 | tags=48%, list=40%, signal=80% |
| 6720 | GOBP\_REGULATION\_OF\_RESPIRATORY\_GASEOUS\_EXCHANGE |  | 8 | -0.36 | -0.78 | 0.776 | 0.939 | 1.000 | 2405 | tags=38%, list=20%, signal=47% |
| 6721 | GOBP\_INTERLEUKIN\_13\_PRODUCTION |  | 9 | -0.34 | -0.78 | 0.774 | 0.940 | 1.000 | 1788 | tags=33%, list=15%, signal=39% |
| 6722 | GOMF\_C2H2\_ZINC\_FINGER\_DOMAIN\_BINDING |  | 8 | -0.35 | -0.78 | 0.776 | 0.940 | 1.000 | 624 | tags=13%, list=5%, signal=13% |
| 6723 | GOMF\_SUCCINATE\_TRANSMEMBRANE\_TRANSPORTER\_ACTIVITY |  | 5 | -0.41 | -0.78 | 0.796 | 0.940 | 1.000 | 872 | tags=20%, list=7%, signal=22% |
| 6724 | GOBP\_GALACTOSE\_METABOLIC\_PROCESS |  | 12 | -0.32 | -0.78 | 0.768 | 0.940 | 1.000 | 1234 | tags=17%, list=10%, signal=19% |
| 6725 | GOBP\_REGULATION\_OF\_UBIQUITIN\_PROTEIN\_TRANSFERASE\_ACTIVITY |  | 45 | -0.24 | -0.78 | 0.822 | 0.940 | 1.000 | 5705 | tags=44%, list=47%, signal=84% |
| 6726 | GOBP\_RESPONSE\_TO\_MITOCHONDRIAL\_DEPOLARISATION |  | 13 | -0.31 | -0.78 | 0.774 | 0.940 | 1.000 | 1362 | tags=23%, list=11%, signal=26% |
| 6727 | GOBP\_POSITIVE\_REGULATION\_OF\_INTERLEUKIN\_12\_PRODUCTION |  | 24 | -0.27 | -0.78 | 0.789 | 0.940 | 1.000 | 2357 | tags=25%, list=20%, signal=31% |
| 6728 | GOCC\_ENDOCYTIC\_VESICLE |  | 210 | -0.20 | -0.78 | 0.921 | 0.940 | 1.000 | 4369 | tags=39%, list=36%, signal=60% |
| 6729 | GOBP\_POSITIVE\_REGULATION\_OF\_PROTEIN\_POLYUBIQUITINATION |  | 5 | -0.40 | -0.78 | 0.796 | 0.940 | 1.000 | 3901 | tags=60%, list=32%, signal=89% |
| 6730 | GOBP\_PURINE\_NUCLEOSIDE\_METABOLIC\_PROCESS |  | 47 | -0.24 | -0.78 | 0.822 | 0.940 | 1.000 | 3917 | tags=36%, list=33%, signal=53% |
| 6731 | GOBP\_NUCLEOSIDE\_TRANSPORT |  | 5 | -0.40 | -0.78 | 0.785 | 0.941 | 1.000 | 3427 | tags=40%, list=29%, signal=56% |
| 6732 | GOMF\_CALCIUM\_DEPENDENT\_PROTEIN\_SERINE\_THREONINE\_PHOSPHATASE\_REGULATOR\_ACTIVITY |  | 4 | -0.43 | -0.78 | 0.804 | 0.941 | 1.000 | 6143 | tags=75%, list=51%, signal=153% |
| 6733 | GOBP\_NOREPINEPHRINE\_UPTAKE |  | 5 | -0.40 | -0.78 | 0.791 | 0.941 | 1.000 | 6895 | tags=80%, list=57%, signal=188% |
| 6734 | GOBP\_PROTEIN\_LOCALIZATION\_TO\_VACUOLE |  | 56 | -0.23 | -0.78 | 0.833 | 0.941 | 1.000 | 4690 | tags=43%, list=39%, signal=70% |
| 6735 | GOBP\_ALKANESULFONATE\_METABOLIC\_PROCESS |  | 5 | -0.40 | -0.77 | 0.791 | 0.941 | 1.000 | 6720 | tags=80%, list=56%, signal=182% |
| 6736 | GOBP\_NEGATIVE\_REGULATION\_OF\_LONG\_TERM\_SYNAPTIC\_POTENTIATION |  | 7 | -0.37 | -0.77 | 0.775 | 0.942 | 1.000 | 3575 | tags=43%, list=30%, signal=61% |
| 6737 | GOMF\_MONOSACCHARIDE\_BINDING |  | 47 | -0.24 | -0.77 | 0.822 | 0.942 | 1.000 | 4424 | tags=45%, list=37%, signal=70% |
| 6738 | GOMF\_PEPTIDE\_ALPHA\_N\_ACETYLTRANSFERASE\_ACTIVITY |  | 7 | -0.36 | -0.77 | 0.784 | 0.942 | 1.000 | 4976 | tags=43%, list=41%, signal=73% |
| 6739 | GOBP\_POSITIVE\_REGULATION\_OF\_B\_CELL\_MEDIATED\_IMMUNITY |  | 22 | -0.27 | -0.77 | 0.790 | 0.942 | 1.000 | 3200 | tags=32%, list=27%, signal=43% |
| 6740 | GOBP\_POSITIVE\_REGULATION\_OF\_P38MAPK\_CASCADE |  | 20 | -0.28 | -0.77 | 0.790 | 0.942 | 1.000 | 3968 | tags=55%, list=33%, signal=82% |
| 6741 | GOBP\_NEGATIVE\_REGULATION\_OF\_MORPHOGENESIS\_OF\_AN\_EPITHELIUM |  | 6 | -0.38 | -0.77 | 0.782 | 0.942 | 1.000 | 866 | tags=17%, list=7%, signal=18% |
| 6742 | GOBP\_NEGATIVE\_REGULATION\_OF\_GLUCONEOGENESIS |  | 12 | -0.31 | -0.77 | 0.772 | 0.943 | 1.000 | 4276 | tags=50%, list=36%, signal=78% |
| 6743 | GOBP\_OOCYTE\_MATURATION |  | 12 | -0.32 | -0.77 | 0.776 | 0.943 | 1.000 | 1820 | tags=25%, list=15%, signal=29% |
| 6744 | GOBP\_POSITIVE\_REGULATION\_OF\_ASTROCYTE\_DIFFERENTIATION |  | 8 | -0.35 | -0.77 | 0.785 | 0.943 | 1.000 | 1597 | tags=25%, list=13%, signal=29% |
| 6745 | GOBP\_ORGANIC\_CYCLIC\_COMPOUND\_CATABOLIC\_PROCESS |  | 461 | -0.20 | -0.77 | 0.979 | 0.943 | 1.000 | 3654 | tags=23%, list=30%, signal=32% |
| 6746 | GOBP\_NUCLEOSIDE\_TRANSMEMBRANE\_TRANSPORT |  | 5 | -0.40 | -0.77 | 0.790 | 0.944 | 1.000 | 3427 | tags=40%, list=29%, signal=56% |
| 6747 | GOBP\_REGULATION\_OF\_CONNECTIVE\_TISSUE\_REPLACEMENT |  | 4 | -0.43 | -0.77 | 0.806 | 0.944 | 1.000 | 6453 | tags=75%, list=54%, signal=162% |
| 6748 | GOBP\_CHONDROITIN\_SULFATE\_PROTEOGLYCAN\_METABOLIC\_PROCESS |  | 33 | -0.25 | -0.77 | 0.816 | 0.944 | 1.000 | 3406 | tags=33%, list=28%, signal=46% |
| 6749 | GOCC\_DNA\_REPLICATION\_FACTOR\_A\_COMPLEX |  | 11 | -0.32 | -0.77 | 0.775 | 0.944 | 1.000 | 2282 | tags=27%, list=19%, signal=34% |
| 6750 | GOCC\_BASOLATERAL\_PLASMA\_MEMBRANE |  | 133 | -0.21 | -0.77 | 0.899 | 0.944 | 1.000 | 3046 | tags=27%, list=25%, signal=36% |
| 6751 | GOBP\_NEGATIVE\_REGULATION\_OF\_GONADOTROPIN\_SECRETION |  | 4 | -0.43 | -0.77 | 0.807 | 0.944 | 1.000 | 3544 | tags=50%, list=30%, signal=71% |
| 6752 | GOBP\_POSITIVE\_REGULATION\_OF\_SODIUM\_ION\_TRANSPORT |  | 26 | -0.26 | -0.77 | 0.800 | 0.945 | 1.000 | 4286 | tags=42%, list=36%, signal=66% |
| 6753 | GOMF\_DEACETYLASE\_ACTIVITY |  | 29 | -0.26 | -0.77 | 0.812 | 0.944 | 1.000 | 2717 | tags=21%, list=23%, signal=27% |
| 6754 | GOBP\_DEFENSE\_RESPONSE\_TO\_GRAM\_NEGATIVE\_BACTERIUM |  | 25 | -0.27 | -0.77 | 0.804 | 0.945 | 1.000 | 3344 | tags=28%, list=28%, signal=39% |
| 6755 | GOBP\_DIOL\_METABOLIC\_PROCESS |  | 22 | -0.27 | -0.77 | 0.795 | 0.945 | 1.000 | 4467 | tags=50%, list=37%, signal=79% |
| 6756 | GOMF\_PATCHED\_BINDING |  | 7 | -0.36 | -0.77 | 0.781 | 0.945 | 1.000 | 3565 | tags=43%, list=30%, signal=61% |
| 6757 | GOBP\_REGULATION\_OF\_GONADOTROPIN\_SECRETION |  | 4 | -0.43 | -0.77 | 0.810 | 0.945 | 1.000 | 3544 | tags=50%, list=30%, signal=71% |
| 6758 | GOMF\_UBIQUITIN\_UBIQUITIN\_LIGASE\_ACTIVITY |  | 11 | -0.32 | -0.77 | 0.781 | 0.946 | 1.000 | 3771 | tags=36%, list=31%, signal=53% |
| 6759 | GOBP\_UBIQUITIN\_RECYCLING |  | 5 | -0.40 | -0.77 | 0.803 | 0.946 | 1.000 | 2620 | tags=40%, list=22%, signal=51% |
| 6760 | GOBP\_POSITIVE\_REGULATION\_OF\_RYANODINE\_SENSITIVE\_CALCIUM\_RELEASE\_CHANNEL\_ACTIVITY |  | 5 | -0.40 | -0.77 | 0.803 | 0.946 | 1.000 | 6004 | tags=80%, list=50%, signal=160% |
| 6761 | GOBP\_REGULATION\_OF\_GONAD\_DEVELOPMENT |  | 7 | -0.36 | -0.77 | 0.786 | 0.946 | 1.000 | 7679 | tags=100%, list=64%, signal=277% |
| 6762 | GOMF\_NUCLEOSIDE\_TRANSMEMBRANE\_TRANSPORTER\_ACTIVITY |  | 5 | -0.40 | -0.77 | 0.802 | 0.946 | 1.000 | 3427 | tags=40%, list=29%, signal=56% |
| 6763 | GOMF\_LEUKOTRIENE\_C4\_SYNTHASE\_ACTIVITY |  | 3 | -0.46 | -0.77 | 0.822 | 0.946 | 1.000 | 263 | tags=33%, list=2%, signal=34% |
| 6764 | GOBP\_ENDOCARDIAL\_CUSHION\_FORMATION |  | 19 | -0.28 | -0.77 | 0.802 | 0.946 | 1.000 | 5879 | tags=68%, list=49%, signal=134% |
| 6765 | GOBP\_LYMPHOCYTE\_MIGRATION\_INTO\_LYMPHOID\_ORGANS |  | 6 | -0.38 | -0.77 | 0.795 | 0.946 | 1.000 | 2955 | tags=50%, list=25%, signal=66% |
| 6766 | GOBP\_RESPONSE\_TO\_HYDROGEN\_PEROXIDE |  | 102 | -0.21 | -0.77 | 0.878 | 0.946 | 1.000 | 3574 | tags=27%, list=30%, signal=39% |
| 6767 | GOBP\_NEGATIVE\_REGULATION\_OF\_LEUKOCYTE\_CHEMOTAXIS |  | 12 | -0.31 | -0.77 | 0.789 | 0.946 | 1.000 | 2600 | tags=33%, list=22%, signal=42% |
| 6768 | GOBP\_REGULATION\_OF\_HEART\_RATE\_BY\_CHEMICAL\_SIGNAL |  | 6 | -0.38 | -0.77 | 0.786 | 0.947 | 1.000 | 893 | tags=17%, list=7%, signal=18% |
| 6769 | GOBP\_MALE\_GENITALIA\_DEVELOPMENT |  | 13 | -0.31 | -0.77 | 0.783 | 0.947 | 1.000 | 3565 | tags=46%, list=30%, signal=66% |
| 6770 | GOBP\_DNA\_METHYLATION\_INVOLVED\_IN\_GAMETE\_GENERATION |  | 9 | -0.34 | -0.77 | 0.787 | 0.947 | 1.000 | 3972 | tags=44%, list=33%, signal=66% |
| 6771 | GOMF\_RIBOSOME\_BINDING |  | 44 | -0.24 | -0.77 | 0.838 | 0.947 | 1.000 | 4525 | tags=34%, list=38%, signal=54% |
| 6772 | GOBP\_NEGATIVE\_REGULATION\_OF\_MESENCHYMAL\_CELL\_APOPTOTIC\_PROCESS |  | 4 | -0.42 | -0.77 | 0.810 | 0.947 | 1.000 | 6948 | tags=100%, list=58%, signal=237% |
| 6773 | GOBP\_GLUTAMATE\_METABOLIC\_PROCESS |  | 22 | -0.27 | -0.77 | 0.800 | 0.947 | 1.000 | 1171 | tags=14%, list=10%, signal=15% |
| 6774 | GOBP\_POSITIVE\_REGULATION\_OF\_DNA\_RECOMBINATION |  | 29 | -0.25 | -0.77 | 0.808 | 0.947 | 1.000 | 3434 | tags=34%, list=29%, signal=48% |
| 6775 | GOBP\_REGULATION\_OF\_STEM\_CELL\_DIFFERENTIATION |  | 81 | -0.22 | -0.77 | 0.865 | 0.947 | 1.000 | 2174 | tags=15%, list=18%, signal=18% |
| 6776 | GOCC\_SMALL\_SUBUNIT\_PROCESSOME |  | 30 | -0.25 | -0.77 | 0.818 | 0.948 | 1.000 | 4061 | tags=30%, list=34%, signal=45% |
| 6777 | GOBP\_DNA\_CATABOLIC\_PROCESS\_EXONUCLEOLYTIC |  | 5 | -0.40 | -0.77 | 0.802 | 0.948 | 1.000 | 4635 | tags=60%, list=39%, signal=98% |
| 6778 | GOBP\_NEURON\_FATE\_COMMITMENT |  | 28 | -0.26 | -0.77 | 0.810 | 0.948 | 1.000 | 2342 | tags=25%, list=19%, signal=31% |
| 6779 | GOBP\_RESPIRATORY\_BURST\_INVOLVED\_IN\_DEFENSE\_RESPONSE |  | 9 | -0.34 | -0.77 | 0.793 | 0.948 | 1.000 | 1305 | tags=22%, list=11%, signal=25% |
| 6780 | GOCC\_ANCHORED\_COMPONENT\_OF\_POSTSYNAPTIC\_MEMBRANE |  | 5 | -0.40 | -0.76 | 0.799 | 0.948 | 1.000 | 2623 | tags=40%, list=22%, signal=51% |
| 6781 | GOMF\_O\_ACETYLTRANSFERASE\_ACTIVITY |  | 6 | -0.38 | -0.76 | 0.803 | 0.948 | 1.000 | 1085 | tags=17%, list=9%, signal=18% |
| 6782 | GOBP\_REGULATION\_OF\_PODOSOME\_ASSEMBLY |  | 8 | -0.35 | -0.76 | 0.784 | 0.948 | 1.000 | 6150 | tags=75%, list=51%, signal=154% |
| 6783 | GOBP\_NEGATIVE\_REGULATION\_OF\_SIGNALING\_RECEPTOR\_ACTIVITY |  | 27 | -0.26 | -0.76 | 0.820 | 0.948 | 1.000 | 2267 | tags=30%, list=19%, signal=36% |
| 6784 | GOBP\_NEGATIVE\_REGULATION\_OF\_EPIDERMIS\_DEVELOPMENT |  | 7 | -0.36 | -0.76 | 0.795 | 0.949 | 1.000 | 552 | tags=14%, list=5%, signal=15% |
| 6785 | GOBP\_POSITIVE\_REGULATION\_OF\_MULTI\_ORGANISM\_PROCESS |  | 14 | -0.30 | -0.76 | 0.794 | 0.949 | 1.000 | 3308 | tags=36%, list=28%, signal=49% |
| 6786 | GOBP\_DUMP\_METABOLIC\_PROCESS |  | 5 | -0.39 | -0.76 | 0.800 | 0.949 | 1.000 | 1728 | tags=40%, list=14%, signal=47% |
| 6787 | GOBP\_CENTRIOLE\_ELONGATION |  | 6 | -0.37 | -0.76 | 0.798 | 0.949 | 1.000 | 2108 | tags=33%, list=18%, signal=40% |
| 6788 | GOBP\_NEGATIVE\_REGULATION\_OF\_BLOOD\_PRESSURE |  | 24 | -0.26 | -0.76 | 0.805 | 0.949 | 1.000 | 1748 | tags=21%, list=15%, signal=24% |
| 6789 | GOBP\_MUSCLE\_ATROPHY |  | 8 | -0.34 | -0.76 | 0.787 | 0.949 | 1.000 | 3444 | tags=50%, list=29%, signal=70% |
| 6790 | GOBP\_REGULATION\_OF\_INTERLEUKIN\_1\_MEDIATED\_SIGNALING\_PATHWAY |  | 4 | -0.42 | -0.76 | 0.814 | 0.949 | 1.000 | 3487 | tags=50%, list=29%, signal=70% |
| 6791 | GOBP\_REGULATION\_OF\_PROTEASOMAL\_PROTEIN\_CATABOLIC\_PROCESS |  | 133 | -0.21 | -0.76 | 0.902 | 0.949 | 1.000 | 1724 | tags=14%, list=14%, signal=16% |
| 6792 | GOBP\_ENDOSOMAL\_VESICLE\_FUSION |  | 8 | -0.35 | -0.76 | 0.797 | 0.949 | 1.000 | 1642 | tags=25%, list=14%, signal=29% |
| 6793 | GOBP\_CELLULAR\_RESPONSE\_TO\_CORTICOSTEROID\_STIMULUS |  | 43 | -0.24 | -0.76 | 0.831 | 0.949 | 1.000 | 2803 | tags=28%, list=23%, signal=36% |
| 6794 | GOCC\_MRNA\_CLEAVAGE\_FACTOR\_COMPLEX |  | 17 | -0.29 | -0.76 | 0.797 | 0.949 | 1.000 | 4889 | tags=41%, list=41%, signal=69% |
| 6795 | GOBP\_NUCLEOBASE\_BIOSYNTHETIC\_PROCESS |  | 16 | -0.29 | -0.76 | 0.794 | 0.949 | 1.000 | 2929 | tags=25%, list=24%, signal=33% |
| 6796 | GOBP\_CIRCADIAN\_SLEEP\_WAKE\_CYCLE\_REM\_SLEEP |  | 3 | -0.45 | -0.76 | 0.828 | 0.949 | 1.000 | 6581 | tags=100%, list=55%, signal=221% |
| 6797 | GOBP\_NEGATIVE\_REGULATION\_OF\_CELL\_CYCLE\_CHECKPOINT |  | 5 | -0.39 | -0.76 | 0.801 | 0.949 | 1.000 | 7312 | tags=100%, list=61%, signal=256% |
| 6798 | GOBP\_DIADENOSINE\_POLYPHOSPHATE\_METABOLIC\_PROCESS |  | 3 | -0.45 | -0.76 | 0.833 | 0.949 | 1.000 | 6549 | tags=100%, list=55%, signal=220% |
| 6799 | GOBP\_NONRIBOSOMAL\_PEPTIDE\_BIOSYNTHETIC\_PROCESS |  | 13 | -0.30 | -0.76 | 0.787 | 0.949 | 1.000 | 3800 | tags=38%, list=32%, signal=56% |
| 6800 | GOBP\_BRANCHING\_INVOLVED\_IN\_LABYRINTHINE\_LAYER\_MORPHOGENESIS |  | 7 | -0.36 | -0.76 | 0.798 | 0.950 | 1.000 | 5622 | tags=71%, list=47%, signal=134% |
| 6801 | GOMF\_DIPHOSPHOTRANSFERASE\_ACTIVITY |  | 5 | -0.39 | -0.76 | 0.808 | 0.950 | 1.000 | 4231 | tags=60%, list=35%, signal=93% |
| 6802 | GOCC\_SPERM\_PRINCIPAL\_PIECE |  | 9 | -0.33 | -0.76 | 0.792 | 0.950 | 1.000 | 2959 | tags=44%, list=25%, signal=59% |
| 6803 | GOBP\_NEGATIVE\_REGULATION\_OF\_DNA\_DAMAGE\_CHECKPOINT |  | 3 | -0.46 | -0.76 | 0.832 | 0.950 | 1.000 | 6543 | tags=100%, list=54%, signal=220% |
| 6804 | GOMF\_CARBOHYDRATE\_DERIVATIVE\_TRANSMEMBRANE\_TRANSPORTER\_ACTIVITY |  | 33 | -0.25 | -0.76 | 0.825 | 0.950 | 1.000 | 2868 | tags=24%, list=24%, signal=32% |
| 6805 | GOBP\_METANEPHRIC\_TUBULE\_FORMATION |  | 3 | -0.46 | -0.76 | 0.831 | 0.950 | 1.000 | 6545 | tags=100%, list=54%, signal=220% |
| 6806 | GOMF\_GUANYLATE\_KINASE\_ACTIVITY |  | 7 | -0.36 | -0.76 | 0.798 | 0.950 | 1.000 | 3824 | tags=57%, list=32%, signal=84% |
| 6807 | GOBP\_ANTIGEN\_PROCESSING\_AND\_PRESENTATION\_VIA\_MHC\_CLASS\_IB |  | 5 | -0.40 | -0.76 | 0.815 | 0.950 | 1.000 | 3112 | tags=40%, list=26%, signal=54% |
| 6808 | GOMF\_TETRAPYRROLE\_BINDING |  | 72 | -0.22 | -0.76 | 0.868 | 0.950 | 1.000 | 2278 | tags=19%, list=19%, signal=24% |
| 6809 | GOBP\_PROTEIN\_LOCALIZATION\_TO\_SITE\_OF\_DOUBLE\_STRAND\_BREAK |  | 5 | -0.39 | -0.76 | 0.811 | 0.950 | 1.000 | 3632 | tags=60%, list=30%, signal=86% |
| 6810 | GOMF\_ALANINE\_TRANSMEMBRANE\_TRANSPORTER\_ACTIVITY |  | 7 | -0.36 | -0.76 | 0.794 | 0.950 | 1.000 | 3006 | tags=43%, list=25%, signal=57% |
| 6811 | GOMF\_MOLECULAR\_CARRIER\_ACTIVITY |  | 48 | -0.23 | -0.76 | 0.846 | 0.950 | 1.000 | 4866 | tags=40%, list=41%, signal=66% |
| 6812 | GOBP\_RESPONSE\_TO\_FRUCTOSE |  | 8 | -0.34 | -0.76 | 0.796 | 0.950 | 1.000 | 3205 | tags=50%, list=27%, signal=68% |
| 6813 | GOBP\_REGULATION\_OF\_INTRINSIC\_APOPTOTIC\_SIGNALING\_PATHWAY\_IN\_RESPONSE\_TO\_DNA\_DAMAGE |  | 24 | -0.26 | -0.76 | 0.817 | 0.951 | 1.000 | 3381 | tags=33%, list=28%, signal=46% |
| 6814 | GOCC\_ENDOPLASMIC\_RETICULUM\_TUBULAR\_NETWORK |  | 20 | -0.27 | -0.76 | 0.807 | 0.951 | 1.000 | 846 | tags=10%, list=7%, signal=11% |
| 6815 | GOBP\_RESPONSE\_TO\_CORTICOSTEROID |  | 97 | -0.21 | -0.76 | 0.895 | 0.951 | 1.000 | 2814 | tags=25%, list=23%, signal=32% |
| 6816 | GOBP\_COPPER\_ION\_TRANSMEMBRANE\_TRANSPORT |  | 3 | -0.45 | -0.76 | 0.839 | 0.951 | 1.000 | 3660 | tags=67%, list=30%, signal=96% |
| 6817 | GOBP\_NEGATIVE\_REGULATION\_OF\_CELL\_MORPHOGENESIS\_INVOLVED\_IN\_DIFFERENTIATION |  | 12 | -0.31 | -0.76 | 0.785 | 0.951 | 1.000 | 2687 | tags=25%, list=22%, signal=32% |
| 6818 | GOBP\_DIADENOSINE\_POLYPHOSPHATE\_CATABOLIC\_PROCESS |  | 3 | -0.45 | -0.76 | 0.844 | 0.951 | 1.000 | 6549 | tags=100%, list=55%, signal=220% |
| 6819 | GOBP\_REGULATION\_OF\_DEFENSE\_RESPONSE\_TO\_VIRUS\_BY\_HOST |  | 18 | -0.28 | -0.76 | 0.812 | 0.951 | 1.000 | 3235 | tags=39%, list=27%, signal=53% |
| 6820 | GOBP\_GENITALIA\_DEVELOPMENT |  | 25 | -0.26 | -0.76 | 0.816 | 0.951 | 1.000 | 2008 | tags=24%, list=17%, signal=29% |
| 6821 | GOMF\_PROTEIN\_KINASE\_A\_CATALYTIC\_SUBUNIT\_BINDING |  | 12 | -0.31 | -0.76 | 0.796 | 0.951 | 1.000 | 56 | tags=8%, list=0%, signal=8% |
| 6822 | GOBP\_INHIBITORY\_POSTSYNAPTIC\_POTENTIAL |  | 6 | -0.37 | -0.76 | 0.799 | 0.951 | 1.000 | 2024 | tags=33%, list=17%, signal=40% |
| 6823 | GOBP\_NEUROTROPHIN\_TRK\_RECEPTOR\_SIGNALING\_PATHWAY |  | 23 | -0.26 | -0.76 | 0.813 | 0.951 | 1.000 | 3797 | tags=35%, list=32%, signal=51% |
| 6824 | GOBP\_NEGATIVE\_REGULATION\_OF\_INTERLEUKIN\_10\_PRODUCTION |  | 6 | -0.37 | -0.76 | 0.800 | 0.951 | 1.000 | 3715 | tags=50%, list=31%, signal=72% |
| 6825 | GOMF\_COPPER\_ION\_TRANSMEMBRANE\_TRANSPORTER\_ACTIVITY |  | 3 | -0.45 | -0.76 | 0.838 | 0.951 | 1.000 | 3660 | tags=67%, list=30%, signal=96% |
| 6826 | GOBP\_RESPONSE\_TO\_ZINC\_ION |  | 26 | -0.26 | -0.76 | 0.821 | 0.952 | 1.000 | 3392 | tags=38%, list=28%, signal=53% |
| 6827 | GOBP\_REGULATION\_OF\_NUCLEAR\_TRANSCRIBED\_MRNA\_CATABOLIC\_PROCESS\_DEADENYLATION\_DEPENDENT\_DECAY |  | 10 | -0.32 | -0.76 | 0.799 | 0.952 | 1.000 | 4666 | tags=60%, list=39%, signal=98% |
| 6828 | GOBP\_INTERFERON\_BETA\_PRODUCTION |  | 36 | -0.24 | -0.76 | 0.830 | 0.952 | 1.000 | 3447 | tags=39%, list=29%, signal=54% |
| 6829 | GOCC\_AZUROPHIL\_GRANULE |  | 103 | -0.21 | -0.76 | 0.895 | 0.952 | 1.000 | 4379 | tags=36%, list=36%, signal=56% |
| 6830 | GOBP\_NEGATIVE\_REGULATION\_OF\_SMOOTH\_MUSCLE\_CONTRACTION |  | 11 | -0.31 | -0.76 | 0.800 | 0.953 | 1.000 | 925 | tags=18%, list=8%, signal=20% |
| 6831 | GOMF\_BASAL\_TRANSCRIPTION\_MACHINERY\_BINDING |  | 42 | -0.23 | -0.76 | 0.843 | 0.953 | 1.000 | 6008 | tags=60%, list=50%, signal=119% |
| 6832 | GOBP\_POSITIVE\_REGULATION\_OF\_UBIQUITIN\_PROTEIN\_LIGASE\_ACTIVITY |  | 10 | -0.32 | -0.76 | 0.809 | 0.953 | 1.000 | 4314 | tags=40%, list=36%, signal=62% |
| 6833 | GOBP\_REGULATION\_OF\_EPINEPHRINE\_SECRETION |  | 4 | -0.42 | -0.75 | 0.826 | 0.953 | 1.000 | 1539 | tags=25%, list=13%, signal=29% |
| 6834 | GOBP\_REGULATION\_OF\_DOPAMINE\_RECEPTOR\_SIGNALING\_PATHWAY |  | 7 | -0.36 | -0.75 | 0.805 | 0.953 | 1.000 | 4139 | tags=43%, list=34%, signal=65% |
| 6835 | GOCC\_LATE\_ENDOSOME |  | 202 | -0.20 | -0.75 | 0.945 | 0.954 | 1.000 | 4845 | tags=41%, list=40%, signal=67% |
| 6836 | GOBP\_DERMATAN\_SULFATE\_METABOLIC\_PROCESS |  | 9 | -0.33 | -0.75 | 0.798 | 0.954 | 1.000 | 3406 | tags=33%, list=28%, signal=46% |
| 6837 | GOBP\_FATTY\_ACID\_ALPHA\_OXIDATION |  | 8 | -0.34 | -0.75 | 0.802 | 0.954 | 1.000 | 2170 | tags=25%, list=18%, signal=30% |
| 6838 | GOBP\_RNA\_SPLICING\_VIA\_ENDONUCLEOLYTIC\_CLEAVAGE\_AND\_LIGATION |  | 11 | -0.31 | -0.75 | 0.799 | 0.954 | 1.000 | 2127 | tags=18%, list=18%, signal=22% |
| 6839 | GOBP\_REGULATION\_OF\_CELLULAR\_RESPIRATION |  | 14 | -0.29 | -0.75 | 0.801 | 0.954 | 1.000 | 3080 | tags=36%, list=26%, signal=48% |
| 6840 | GOBP\_MANNOSE\_METABOLIC\_PROCESS |  | 8 | -0.34 | -0.75 | 0.805 | 0.954 | 1.000 | 147 | tags=13%, list=1%, signal=13% |
| 6841 | GOBP\_POSITIVE\_REGULATION\_OF\_PROTEIN\_MONOUBIQUITINATION |  | 4 | -0.41 | -0.75 | 0.828 | 0.954 | 1.000 | 1706 | tags=25%, list=14%, signal=29% |
| 6842 | GOBP\_WOUND\_HEALING\_SPREADING\_OF\_EPIDERMAL\_CELLS |  | 13 | -0.30 | -0.75 | 0.797 | 0.954 | 1.000 | 3551 | tags=46%, list=30%, signal=65% |
| 6843 | GOBP\_TERMINATION\_OF\_RNA\_POLYMERASE\_II\_TRANSCRIPTION |  | 27 | -0.26 | -0.75 | 0.833 | 0.954 | 1.000 | 3084 | tags=26%, list=26%, signal=35% |
| 6844 | GOMF\_TRIGLYCERIDE\_LIPASE\_ACTIVITY |  | 13 | -0.30 | -0.75 | 0.803 | 0.954 | 1.000 | 2651 | tags=23%, list=22%, signal=30% |
| 6845 | GOBP\_GLOMERULUS\_VASCULATURE\_MORPHOGENESIS |  | 5 | -0.39 | -0.75 | 0.820 | 0.954 | 1.000 | 3286 | tags=40%, list=27%, signal=55% |
| 6846 | GOBP\_PARASYMPATHETIC\_NERVOUS\_SYSTEM\_DEVELOPMENT |  | 6 | -0.37 | -0.75 | 0.821 | 0.954 | 1.000 | 2903 | tags=33%, list=24%, signal=44% |
| 6847 | GOBP\_CEREBRAL\_CORTEX\_GABAERGIC\_INTERNEURON\_DEVELOPMENT |  | 3 | -0.45 | -0.75 | 0.842 | 0.954 | 1.000 | 6588 | tags=100%, list=55%, signal=221% |
| 6848 | GOBP\_REPLICATION\_FORK\_PROTECTION |  | 7 | -0.35 | -0.75 | 0.804 | 0.954 | 1.000 | 3214 | tags=29%, list=27%, signal=39% |
| 6849 | GOBP\_NEGATIVE\_REGULATION\_OF\_PROTEIN\_TARGETING\_TO\_MITOCHONDRION |  | 3 | -0.45 | -0.75 | 0.838 | 0.954 | 1.000 | 6650 | tags=100%, list=55%, signal=224% |
| 6850 | GOBP\_RESPONSE\_TO\_SALT |  | 7 | -0.35 | -0.75 | 0.804 | 0.955 | 1.000 | 1143 | tags=14%, list=10%, signal=16% |
| 6851 | GOBP\_NEGATIVE\_REGULATION\_OF\_NITRIC\_OXIDE\_METABOLIC\_PROCESS |  | 10 | -0.32 | -0.75 | 0.809 | 0.955 | 1.000 | 3968 | tags=40%, list=33%, signal=60% |
| 6852 | GOBP\_REGULATION\_OF\_LIPOPROTEIN\_METABOLIC\_PROCESS |  | 12 | -0.31 | -0.75 | 0.805 | 0.955 | 1.000 | 5672 | tags=50%, list=47%, signal=95% |
| 6853 | GOCC\_FEMALE\_GERM\_CELL\_NUCLEUS |  | 3 | -0.45 | -0.75 | 0.838 | 0.955 | 1.000 | 3195 | tags=33%, list=27%, signal=45% |
| 6854 | GOBP\_POSITIVE\_REGULATION\_OF\_MITOTIC\_CELL\_CYCLE |  | 84 | -0.21 | -0.75 | 0.883 | 0.956 | 1.000 | 5463 | tags=52%, list=45%, signal=95% |
| 6855 | GOMF\_VASCULAR\_ENDOTHELIAL\_GROWTH\_FACTOR\_BINDING |  | 5 | -0.39 | -0.75 | 0.828 | 0.956 | 1.000 | 3286 | tags=60%, list=27%, signal=83% |
| 6856 | GOMF\_INWARD\_RECTIFIER\_POTASSIUM\_CHANNEL\_ACTIVITY |  | 11 | -0.31 | -0.75 | 0.807 | 0.956 | 1.000 | 1627 | tags=18%, list=14%, signal=21% |
| 6857 | GOBP\_NEGATIVE\_REGULATION\_OF\_SYNAPSE\_ASSEMBLY |  | 5 | -0.39 | -0.75 | 0.822 | 0.956 | 1.000 | 2300 | tags=20%, list=19%, signal=25% |
| 6858 | GOBP\_ATTACHMENT\_OF\_GPI\_ANCHOR\_TO\_PROTEIN |  | 5 | -0.39 | -0.75 | 0.824 | 0.956 | 1.000 | 1101 | tags=20%, list=9%, signal=22% |
| 6859 | GOBP\_POSITIVE\_REGULATION\_OF\_MITOCHONDRIAL\_CALCIUM\_ION\_CONCENTRATION |  | 8 | -0.34 | -0.75 | 0.814 | 0.956 | 1.000 | 3682 | tags=38%, list=31%, signal=54% |
| 6860 | GOBP\_COLLAGEN\_METABOLIC\_PROCESS |  | 67 | -0.22 | -0.75 | 0.885 | 0.956 | 1.000 | 4844 | tags=42%, list=40%, signal=70% |
| 6861 | GOMF\_CAMP\_RESPONSE\_ELEMENT\_BINDING\_PROTEIN\_BINDING |  | 8 | -0.34 | -0.75 | 0.811 | 0.956 | 1.000 | 2667 | tags=38%, list=22%, signal=48% |
| 6862 | GOBP\_NUCLEOBASE\_CONTAINING\_SMALL\_MOLECULE\_METABOLIC\_PROCESS |  | 424 | -0.19 | -0.75 | 0.980 | 0.956 | 1.000 | 4255 | tags=34%, list=35%, signal=51% |
| 6863 | GOMF\_OXIDOREDUCTASE\_ACTIVITY\_ACTING\_ON\_A\_SULFUR\_GROUP\_OF\_DONORS\_OXYGEN\_AS\_ACCEPTOR |  | 10 | -0.32 | -0.75 | 0.814 | 0.956 | 1.000 | 2363 | tags=30%, list=20%, signal=37% |
| 6864 | GOBP\_POSITIVE\_REGULATION\_OF\_MONOCYTE\_CHEMOTACTIC\_PROTEIN\_1\_PRODUCTION |  | 7 | -0.35 | -0.75 | 0.804 | 0.956 | 1.000 | 2357 | tags=29%, list=20%, signal=36% |
| 6865 | GOBP\_INTEGRATED\_STRESS\_RESPONSE\_SIGNALING |  | 22 | -0.26 | -0.75 | 0.820 | 0.956 | 1.000 | 3872 | tags=41%, list=32%, signal=60% |
| 6866 | GOBP\_POSITIVE\_REGULATION\_OF\_LIPOPROTEIN\_METABOLIC\_PROCESS |  | 4 | -0.41 | -0.75 | 0.831 | 0.956 | 1.000 | 4324 | tags=50%, list=36%, signal=78% |
| 6867 | GOBP\_BASE\_CONVERSION\_OR\_SUBSTITUTION\_EDITING |  | 7 | -0.35 | -0.75 | 0.811 | 0.956 | 1.000 | 492 | tags=14%, list=4%, signal=15% |
| 6868 | GOBP\_ANGIOGENESIS\_INVOLVED\_IN\_WOUND\_HEALING |  | 22 | -0.26 | -0.75 | 0.823 | 0.956 | 1.000 | 3205 | tags=32%, list=27%, signal=43% |
| 6869 | GOBP\_FRUCTOSE\_1\_6\_BISPHOSPHATE\_METABOLIC\_PROCESS |  | 6 | -0.37 | -0.75 | 0.818 | 0.956 | 1.000 | 3908 | tags=50%, list=33%, signal=74% |
| 6870 | GOBP\_CELLULAR\_RESPONSE\_TO\_REACTIVE\_NITROGEN\_SPECIES |  | 13 | -0.30 | -0.75 | 0.811 | 0.956 | 1.000 | 2829 | tags=31%, list=24%, signal=40% |
| 6871 | GOBP\_ANTIGEN\_PROCESSING\_AND\_PRESENTATION\_OF\_PEPTIDE\_ANTIGEN\_VIA\_MHC\_CLASS\_I |  | 69 | -0.22 | -0.75 | 0.883 | 0.956 | 1.000 | 3801 | tags=29%, list=32%, signal=42% |
| 6872 | GOMF\_KERATIN\_FILAMENT\_BINDING |  | 3 | -0.45 | -0.75 | 0.849 | 0.956 | 1.000 | 6595 | tags=100%, list=55%, signal=222% |
| 6873 | GOBP\_CELLULAR\_RESPONSE\_TO\_PEPTIDOGLYCAN |  | 3 | -0.45 | -0.75 | 0.843 | 0.956 | 1.000 | 5729 | tags=67%, list=48%, signal=127% |
| 6874 | GOBP\_REPRODUCTIVE\_BEHAVIOR |  | 17 | -0.28 | -0.75 | 0.816 | 0.956 | 1.000 | 4532 | tags=53%, list=38%, signal=85% |
| 6875 | GOBP\_ESTABLISHMENT\_OF\_MEIOTIC\_SPINDLE\_LOCALIZATION |  | 3 | -0.45 | -0.75 | 0.844 | 0.956 | 1.000 | 1843 | tags=33%, list=15%, signal=39% |
| 6876 | GOMF\_MONOAMINE\_TRANSMEMBRANE\_TRANSPORTER\_ACTIVITY |  | 6 | -0.37 | -0.75 | 0.822 | 0.956 | 1.000 | 2392 | tags=33%, list=20%, signal=42% |
| 6877 | GOBP\_REGULATION\_OF\_BICELLULAR\_TIGHT\_JUNCTION\_ASSEMBLY |  | 18 | -0.27 | -0.75 | 0.812 | 0.956 | 1.000 | 2416 | tags=28%, list=20%, signal=35% |
| 6878 | GOBP\_PYRIMIDINE\_DEOXYRIBONUCLEOSIDE\_MONOPHOSPHATE\_METABOLIC\_PROCESS |  | 7 | -0.35 | -0.75 | 0.805 | 0.956 | 1.000 | 1728 | tags=29%, list=14%, signal=33% |
| 6879 | GOMF\_INSULIN\_LIKE\_GROWTH\_FACTOR\_I\_BINDING |  | 12 | -0.30 | -0.75 | 0.807 | 0.956 | 1.000 | 2448 | tags=33%, list=20%, signal=42% |
| 6880 | GOBP\_REGULATION\_OF\_HISTONE\_H3\_K27\_METHYLATION |  | 5 | -0.39 | -0.75 | 0.814 | 0.956 | 1.000 | 3492 | tags=60%, list=29%, signal=85% |
| 6881 | GOBP\_RESPIRATORY\_SYSTEM\_PROCESS |  | 18 | -0.27 | -0.75 | 0.807 | 0.956 | 1.000 | 3112 | tags=33%, list=26%, signal=45% |
| 6882 | GOBP\_MRNA\_PROCESSING |  | 382 | -0.19 | -0.75 | 0.979 | 0.956 | 1.000 | 5215 | tags=37%, list=43%, signal=63% |
| 6883 | GOBP\_UNSATURATED\_FATTY\_ACID\_METABOLIC\_PROCESS |  | 64 | -0.22 | -0.75 | 0.872 | 0.956 | 1.000 | 4536 | tags=48%, list=38%, signal=77% |
| 6884 | GOBP\_OPSONIZATION |  | 8 | -0.34 | -0.75 | 0.824 | 0.957 | 1.000 | 1613 | tags=25%, list=13%, signal=29% |
| 6885 | GOCC\_OUTER\_MEMBRANE |  | 159 | -0.20 | -0.75 | 0.934 | 0.957 | 1.000 | 3966 | tags=33%, list=33%, signal=49% |
| 6886 | GOBP\_POSITIVE\_REGULATION\_OF\_TRANSCRIPTION\_BY\_RNA\_POLYMERASE\_I |  | 18 | -0.27 | -0.75 | 0.818 | 0.957 | 1.000 | 4587 | tags=50%, list=38%, signal=81% |
| 6887 | GOMF\_POLY\_G\_BINDING |  | 8 | -0.34 | -0.75 | 0.796 | 0.957 | 1.000 | 938 | tags=13%, list=8%, signal=14% |
| 6888 | GOBP\_NEGATIVE\_REGULATION\_OF\_CYTOPLASMIC\_TRANSLATION |  | 7 | -0.35 | -0.75 | 0.809 | 0.957 | 1.000 | 5307 | tags=57%, list=44%, signal=102% |
| 6889 | GOBP\_SNORNA\_LOCALIZATION |  | 3 | -0.45 | -0.74 | 0.845 | 0.957 | 1.000 | 4403 | tags=33%, list=37%, signal=53% |
| 6890 | GOMF\_AMIDINE\_LYASE\_ACTIVITY |  | 6 | -0.37 | -0.74 | 0.822 | 0.957 | 1.000 | 4342 | tags=50%, list=36%, signal=78% |
| 6891 | GOBP\_RESPONSE\_TO\_KETONE |  | 135 | -0.20 | -0.74 | 0.922 | 0.957 | 1.000 | 4215 | tags=36%, list=35%, signal=55% |
| 6892 | GOBP\_POSITIVE\_REGULATION\_OF\_TYPE\_I\_INTERFERON\_PRODUCTION |  | 57 | -0.22 | -0.74 | 0.869 | 0.957 | 1.000 | 3504 | tags=35%, list=29%, signal=49% |
| 6893 | GOMF\_Y\_FORM\_DNA\_BINDING |  | 4 | -0.41 | -0.74 | 0.836 | 0.957 | 1.000 | 3214 | tags=50%, list=27%, signal=68% |
| 6894 | GOBP\_SULFUR\_AMINO\_ACID\_BIOSYNTHETIC\_PROCESS |  | 14 | -0.29 | -0.74 | 0.817 | 0.957 | 1.000 | 1018 | tags=14%, list=8%, signal=16% |
| 6895 | GOMF\_G\_PROTEIN\_COUPLED\_NEUROTRANSMITTER\_RECEPTOR\_ACTIVITY |  | 3 | -0.44 | -0.74 | 0.849 | 0.958 | 1.000 | 929 | tags=33%, list=8%, signal=36% |
| 6896 | GOBP\_DNA\_TEMPLATED\_TRANSCRIPTION\_TERMINATION |  | 53 | -0.22 | -0.74 | 0.873 | 0.958 | 1.000 | 5069 | tags=42%, list=42%, signal=72% |
| 6897 | GOBP\_APOPTOTIC\_PROCESS\_INVOLVED\_IN\_DEVELOPMENT |  | 21 | -0.26 | -0.74 | 0.817 | 0.958 | 1.000 | 624 | tags=10%, list=5%, signal=10% |
| 6898 | GOCC\_M\_BAND |  | 18 | -0.27 | -0.74 | 0.820 | 0.958 | 1.000 | 3818 | tags=39%, list=32%, signal=57% |
| 6899 | GOMF\_FLAVIN\_ADENINE\_DINUCLEOTIDE\_BINDING |  | 65 | -0.22 | -0.74 | 0.881 | 0.958 | 1.000 | 2580 | tags=18%, list=21%, signal=23% |
| 6900 | GOBP\_CELLULAR\_LIPID\_CATABOLIC\_PROCESS |  | 173 | -0.20 | -0.74 | 0.941 | 0.958 | 1.000 | 4068 | tags=34%, list=34%, signal=50% |
| 6901 | GOMF\_CALCIUM\_ACTIVATED\_POTASSIUM\_CHANNEL\_ACTIVITY |  | 6 | -0.37 | -0.74 | 0.828 | 0.958 | 1.000 | 1042 | tags=17%, list=9%, signal=18% |
| 6902 | GOBP\_MODULATION\_BY\_SYMBIONT\_OF\_HOST\_PROCESS |  | 28 | -0.25 | -0.74 | 0.829 | 0.958 | 1.000 | 4866 | tags=50%, list=41%, signal=84% |
| 6903 | GOBP\_REGULATION\_OF\_DEFENSE\_RESPONSE\_TO\_VIRUS\_BY\_VIRUS |  | 23 | -0.26 | -0.74 | 0.828 | 0.958 | 1.000 | 1825 | tags=22%, list=15%, signal=26% |
| 6904 | GOMF\_CASPASE\_BINDING |  | 5 | -0.39 | -0.74 | 0.826 | 0.958 | 1.000 | 1084 | tags=20%, list=9%, signal=22% |
| 6905 | GOMF\_HISTONE\_METHYLTRANSFERASE\_ACTIVITY\_H3\_K27\_SPECIFIC |  | 3 | -0.45 | -0.74 | 0.846 | 0.958 | 1.000 | 5831 | tags=67%, list=49%, signal=130% |
| 6906 | GOBP\_REGULATION\_OF\_MACROPHAGE\_APOPTOTIC\_PROCESS |  | 6 | -0.36 | -0.74 | 0.823 | 0.958 | 1.000 | 2656 | tags=50%, list=22%, signal=64% |
| 6907 | GOBP\_IMPORT\_ACROSS\_PLASMA\_MEMBRANE |  | 93 | -0.21 | -0.74 | 0.901 | 0.958 | 1.000 | 3011 | tags=27%, list=25%, signal=36% |
| 6908 | GOBP\_DE\_NOVO\_IMP\_BIOSYNTHETIC\_PROCESS |  | 5 | -0.38 | -0.74 | 0.838 | 0.959 | 1.000 | 2929 | tags=20%, list=24%, signal=26% |
| 6909 | GOCC\_EPSILON\_DNA\_POLYMERASE\_COMPLEX |  | 5 | -0.38 | -0.74 | 0.835 | 0.959 | 1.000 | 4779 | tags=60%, list=40%, signal=100% |
| 6910 | GOBP\_REGULATION\_OF\_FIBRINOLYSIS |  | 7 | -0.35 | -0.74 | 0.818 | 0.959 | 1.000 | 2820 | tags=43%, list=23%, signal=56% |
| 6911 | GOBP\_REGULATION\_OF\_SALIVA\_SECRETION |  | 3 | -0.44 | -0.74 | 0.859 | 0.959 | 1.000 | 1146 | tags=33%, list=10%, signal=37% |
| 6912 | GOMF\_VASCULAR\_ENDOTHELIAL\_GROWTH\_FACTOR\_RECEPTOR\_BINDING |  | 11 | -0.31 | -0.74 | 0.813 | 0.960 | 1.000 | 3196 | tags=36%, list=27%, signal=50% |
| 6913 | GOBP\_POSITIVE\_REGULATION\_OF\_ISOTYPE\_SWITCHING\_TO\_IGA\_ISOTYPES |  | 3 | -0.44 | -0.74 | 0.853 | 0.960 | 1.000 | 6715 | tags=100%, list=56%, signal=227% |
| 6914 | GOBP\_REGULATION\_OF\_FATTY\_ACID\_BIOSYNTHETIC\_PROCESS |  | 33 | -0.24 | -0.74 | 0.854 | 0.960 | 1.000 | 2656 | tags=24%, list=22%, signal=31% |
| 6915 | GOBP\_EPIBOLY |  | 24 | -0.25 | -0.74 | 0.829 | 0.960 | 1.000 | 3551 | tags=33%, list=30%, signal=47% |
| 6916 | GOMF\_G\_PROTEIN\_COUPLED\_ACETYLCHOLINE\_RECEPTOR\_ACTIVITY |  | 3 | -0.44 | -0.74 | 0.852 | 0.960 | 1.000 | 929 | tags=33%, list=8%, signal=36% |
| 6917 | GOBP\_DNA\_UNWINDING\_INVOLVED\_IN\_DNA\_REPLICATION |  | 12 | -0.30 | -0.74 | 0.826 | 0.960 | 1.000 | 4233 | tags=67%, list=35%, signal=103% |
| 6918 | GOBP\_OLEFINIC\_COMPOUND\_BIOSYNTHETIC\_PROCESS |  | 12 | -0.30 | -0.74 | 0.816 | 0.960 | 1.000 | 2374 | tags=42%, list=20%, signal=52% |
| 6919 | GOBP\_DELAMINATION |  | 3 | -0.44 | -0.74 | 0.852 | 0.960 | 1.000 | 4274 | tags=33%, list=36%, signal=52% |
| 6920 | GOBP\_RECEPTOR\_INTERNALIZATION |  | 74 | -0.21 | -0.74 | 0.890 | 0.960 | 1.000 | 5462 | tags=54%, list=45%, signal=99% |
| 6921 | GOBP\_REGULATION\_OF\_PHOSPHATE\_TRANSPORT |  | 4 | -0.41 | -0.74 | 0.840 | 0.960 | 1.000 | 972 | tags=25%, list=8%, signal=27% |
| 6922 | GOMF\_INOSITOL\_POLYPHOSPHATE\_5\_PHOSPHATASE\_ACTIVITY |  | 6 | -0.36 | -0.74 | 0.829 | 0.960 | 1.000 | 5298 | tags=67%, list=44%, signal=119% |
| 6923 | GOBP\_NEGATIVE\_REGULATION\_OF\_GUANYL\_NUCLEOTIDE\_EXCHANGE\_FACTOR\_ACTIVITY |  | 4 | -0.41 | -0.74 | 0.839 | 0.960 | 1.000 | 4824 | tags=50%, list=40%, signal=84% |
| 6924 | GOBP\_REGULATION\_OF\_PHOSPHOLIPASE\_A2\_ACTIVITY |  | 7 | -0.35 | -0.74 | 0.824 | 0.960 | 1.000 | 3007 | tags=57%, list=25%, signal=76% |
| 6925 | GOBP\_NEUROTROPHIN\_SIGNALING\_PATHWAY |  | 28 | -0.25 | -0.74 | 0.855 | 0.960 | 1.000 | 5950 | tags=61%, list=50%, signal=120% |
| 6926 | GOBP\_POSITIVE\_REGULATION\_OF\_KIDNEY\_DEVELOPMENT |  | 7 | -0.35 | -0.74 | 0.828 | 0.960 | 1.000 | 5061 | tags=57%, list=42%, signal=99% |
| 6927 | GOBP\_NEGATIVE\_REGULATION\_OF\_GLIAL\_CELL\_MIGRATION |  | 3 | -0.44 | -0.74 | 0.862 | 0.960 | 1.000 | 5741 | tags=67%, list=48%, signal=128% |
| 6928 | GOBP\_RIBONUCLEOSIDE\_CATABOLIC\_PROCESS |  | 13 | -0.29 | -0.74 | 0.821 | 0.960 | 1.000 | 1107 | tags=15%, list=9%, signal=17% |
| 6929 | GOMF\_SULFUR\_COMPOUND\_BINDING |  | 162 | -0.20 | -0.74 | 0.942 | 0.961 | 1.000 | 3217 | tags=25%, list=27%, signal=33% |
| 6930 | GOBP\_ADENYLATE\_CYCLASE\_INHIBITING\_G\_PROTEIN\_COUPLED\_ACETYLCHOLINE\_RECEPTOR\_SIGNALING\_PATHWAY |  | 3 | -0.44 | -0.74 | 0.854 | 0.962 | 1.000 | 929 | tags=33%, list=8%, signal=36% |
| 6931 | GOCC\_STEREOCILIUM\_BUNDLE |  | 31 | -0.24 | -0.74 | 0.854 | 0.962 | 1.000 | 4949 | tags=45%, list=41%, signal=77% |
| 6932 | GOMF\_NAD\_DEPENDENT\_PROTEIN\_DEACETYLASE\_ACTIVITY |  | 14 | -0.29 | -0.74 | 0.831 | 0.962 | 1.000 | 2717 | tags=21%, list=23%, signal=28% |
| 6933 | GOBP\_ATRIAL\_CARDIAC\_MUSCLE\_CELL\_TO\_AV\_NODE\_CELL\_SIGNALING |  | 13 | -0.29 | -0.74 | 0.818 | 0.962 | 1.000 | 3295 | tags=38%, list=27%, signal=53% |
| 6934 | GOBP\_REGULATION\_OF\_MYOBLAST\_DIFFERENTIATION |  | 37 | -0.23 | -0.74 | 0.853 | 0.962 | 1.000 | 2474 | tags=22%, list=21%, signal=27% |
| 6935 | GOBP\_NEGATIVE\_REGULATION\_OF\_UBIQUITIN\_DEPENDENT\_PROTEIN\_CATABOLIC\_PROCESS |  | 37 | -0.23 | -0.74 | 0.861 | 0.962 | 1.000 | 3565 | tags=22%, list=30%, signal=31% |
| 6936 | GOBP\_REGULATION\_OF\_INNER\_EAR\_AUDITORY\_RECEPTOR\_CELL\_DIFFERENTIATION |  | 6 | -0.36 | -0.73 | 0.835 | 0.962 | 1.000 | 4204 | tags=50%, list=35%, signal=77% |
| 6937 | GOBP\_CALCITONIN\_FAMILY\_RECEPTOR\_SIGNALING\_PATHWAY |  | 6 | -0.36 | -0.73 | 0.833 | 0.962 | 1.000 | 3134 | tags=33%, list=26%, signal=45% |
| 6938 | GOBP\_ALANINE\_TRANSPORT |  | 8 | -0.33 | -0.73 | 0.820 | 0.962 | 1.000 | 3006 | tags=38%, list=25%, signal=50% |
| 6939 | GOBP\_INTERLEUKIN\_5\_PRODUCTION |  | 9 | -0.32 | -0.73 | 0.819 | 0.962 | 1.000 | 893 | tags=22%, list=7%, signal=24% |
| 6940 | GOMF\_GUANYL\_NUCLEOTIDE\_BINDING |  | 271 | -0.19 | -0.73 | 0.974 | 0.962 | 1.000 | 4277 | tags=29%, list=36%, signal=44% |
| 6941 | GOBP\_PURINE\_NUCLEOBASE\_BIOSYNTHETIC\_PROCESS |  | 8 | -0.33 | -0.73 | 0.828 | 0.962 | 1.000 | 2929 | tags=38%, list=24%, signal=50% |
| 6942 | GOBP\_MITOCHONDRIAL\_FISSION |  | 31 | -0.24 | -0.73 | 0.855 | 0.962 | 1.000 | 4128 | tags=42%, list=34%, signal=64% |
| 6943 | GOBP\_DETECTION\_OF\_STIMULUS\_INVOLVED\_IN\_SENSORY\_PERCEPTION\_OF\_PAIN |  | 15 | -0.28 | -0.73 | 0.822 | 0.962 | 1.000 | 687 | tags=13%, list=6%, signal=14% |
| 6944 | GOBP\_NEGATIVE\_REGULATION\_OF\_INTERFERON\_ALPHA\_PRODUCTION |  | 3 | -0.44 | -0.73 | 0.855 | 0.962 | 1.000 | 673 | tags=33%, list=6%, signal=35% |
| 6945 | GOCC\_NUCLEAR\_OUTER\_MEMBRANE |  | 17 | -0.27 | -0.73 | 0.838 | 0.962 | 1.000 | 1096 | tags=18%, list=9%, signal=19% |
| 6946 | GOBP\_PROTEIN\_ACTIVATION\_CASCADE |  | 17 | -0.27 | -0.73 | 0.821 | 0.962 | 1.000 | 3394 | tags=35%, list=28%, signal=49% |
| 6947 | GOBP\_REGULATION\_OF\_SYSTEMIC\_ARTERIAL\_BLOOD\_PRESSURE\_BY\_HORMONE |  | 25 | -0.25 | -0.73 | 0.848 | 0.962 | 1.000 | 2480 | tags=28%, list=21%, signal=35% |
| 6948 | GOBP\_POSITIVE\_REGULATION\_OF\_ANOIKIS |  | 4 | -0.41 | -0.73 | 0.844 | 0.962 | 1.000 | 3888 | tags=50%, list=32%, signal=74% |
| 6949 | GOCC\_ANNULATE\_LAMELLAE |  | 4 | -0.40 | -0.73 | 0.846 | 0.962 | 1.000 | 7172 | tags=100%, list=60%, signal=248% |
| 6950 | GOBP\_RESPONSE\_TO\_FUNGUS |  | 18 | -0.27 | -0.73 | 0.837 | 0.962 | 1.000 | 2357 | tags=28%, list=20%, signal=35% |
| 6951 | GOBP\_NEGATIVE\_REGULATION\_OF\_BIOMINERALIZATION |  | 14 | -0.29 | -0.73 | 0.821 | 0.963 | 1.000 | 6948 | tags=79%, list=58%, signal=186% |
| 6952 | GOBP\_REGULATION\_OF\_GUANYLATE\_CYCLASE\_ACTIVITY |  | 4 | -0.41 | -0.73 | 0.849 | 0.963 | 1.000 | 2421 | tags=50%, list=20%, signal=63% |
| 6953 | GOCC\_PLATELET\_DENSE\_GRANULE\_LUMEN |  | 6 | -0.36 | -0.73 | 0.836 | 0.963 | 1.000 | 4098 | tags=67%, list=34%, signal=101% |
| 6954 | GOBP\_NEGATIVE\_REGULATION\_OF\_MRNA\_PROCESSING |  | 23 | -0.25 | -0.73 | 0.848 | 0.963 | 1.000 | 4968 | tags=48%, list=41%, signal=81% |
| 6955 | GOBP\_REGULATION\_OF\_MALE\_GONAD\_DEVELOPMENT |  | 6 | -0.36 | -0.73 | 0.838 | 0.964 | 1.000 | 7679 | tags=100%, list=64%, signal=277% |
| 6956 | GOBP\_CELLULAR\_RESPONSE\_TO\_ACID\_CHEMICAL |  | 51 | -0.22 | -0.73 | 0.877 | 0.964 | 1.000 | 5778 | tags=51%, list=48%, signal=98% |
| 6957 | GOMF\_PROTEASOME\_BINDING |  | 13 | -0.29 | -0.73 | 0.822 | 0.964 | 1.000 | 2174 | tags=23%, list=18%, signal=28% |
| 6958 | GOMF\_RIBONUCLEASE\_ACTIVITY |  | 76 | -0.21 | -0.73 | 0.900 | 0.964 | 1.000 | 5337 | tags=43%, list=44%, signal=78% |
| 6959 | GOBP\_CELL\_ADHESION\_INVOLVED\_IN\_HEART\_MORPHOGENESIS |  | 5 | -0.38 | -0.73 | 0.834 | 0.964 | 1.000 | 1459 | tags=20%, list=12%, signal=23% |
| 6960 | GOBP\_REGULATION\_OF\_CARDIOBLAST\_DIFFERENTIATION |  | 4 | -0.40 | -0.73 | 0.848 | 0.964 | 1.000 | 4569 | tags=50%, list=38%, signal=81% |
| 6961 | GOBP\_RETINOIC\_ACID\_RECEPTOR\_SIGNALING\_PATHWAY |  | 20 | -0.26 | -0.73 | 0.836 | 0.964 | 1.000 | 5000 | tags=60%, list=42%, signal=103% |
| 6962 | GOBP\_POSITIVE\_REGULATION\_OF\_IRE1\_MEDIATED\_UNFOLDED\_PROTEIN\_RESPONSE |  | 6 | -0.36 | -0.73 | 0.830 | 0.964 | 1.000 | 1637 | tags=33%, list=14%, signal=39% |
| 6963 | GOBP\_NEGATIVE\_REGULATION\_OF\_I\_KAPPAB\_KINASE\_NF\_KAPPAB\_SIGNALING |  | 34 | -0.24 | -0.73 | 0.851 | 0.964 | 1.000 | 5463 | tags=62%, list=45%, signal=113% |
| 6964 | GOBP\_POSITIVE\_REGULATION\_OF\_REGULATORY\_T\_CELL\_DIFFERENTIATION |  | 4 | -0.40 | -0.73 | 0.841 | 0.965 | 1.000 | 1560 | tags=25%, list=13%, signal=29% |
| 6965 | GOMF\_RNA\_POLYMERASE\_II\_TRANSCRIPTION\_COREGULATOR\_BINDING |  | 16 | -0.28 | -0.73 | 0.836 | 0.965 | 1.000 | 6440 | tags=75%, list=54%, signal=161% |
| 6966 | GOCC\_ELASTIC\_FIBER |  | 3 | -0.44 | -0.73 | 0.872 | 0.965 | 1.000 | 2706 | tags=33%, list=23%, signal=43% |
| 6967 | GOBP\_ABSCISSION |  | 5 | -0.38 | -0.73 | 0.839 | 0.965 | 1.000 | 1818 | tags=20%, list=15%, signal=24% |
| 6968 | GOBP\_NEGATIVE\_REGULATION\_OF\_CATION\_TRANSMEMBRANE\_TRANSPORT |  | 56 | -0.22 | -0.73 | 0.886 | 0.965 | 1.000 | 4140 | tags=34%, list=34%, signal=52% |
| 6969 | GOBP\_POSITIVE\_REGULATION\_OF\_OXIDATIVE\_STRESS\_INDUCED\_CELL\_DEATH |  | 10 | -0.31 | -0.73 | 0.833 | 0.965 | 1.000 | 5544 | tags=40%, list=46%, signal=74% |
| 6970 | GOMF\_MHC\_PROTEIN\_BINDING |  | 15 | -0.28 | -0.73 | 0.835 | 0.965 | 1.000 | 2774 | tags=27%, list=23%, signal=35% |
| 6971 | GOBP\_REGULATION\_OF\_BRANCHING\_INVOLVED\_IN\_PROSTATE\_GLAND\_MORPHOGENESIS |  | 4 | -0.40 | -0.73 | 0.848 | 0.966 | 1.000 | 1579 | tags=25%, list=13%, signal=29% |
| 6972 | GOBP\_MUCUS\_SECRETION |  | 4 | -0.40 | -0.73 | 0.853 | 0.966 | 1.000 | 4561 | tags=75%, list=38%, signal=121% |
| 6973 | GOBP\_POSITIVE\_REGULATION\_OF\_MYOBLAST\_DIFFERENTIATION |  | 12 | -0.29 | -0.73 | 0.838 | 0.966 | 1.000 | 5825 | tags=67%, list=48%, signal=129% |
| 6974 | GOBP\_PEPTIDYL\_ASPARAGINE\_MODIFICATION |  | 25 | -0.25 | -0.73 | 0.851 | 0.966 | 1.000 | 6295 | tags=72%, list=52%, signal=151% |
| 6975 | GOBP\_MAINTENANCE\_OF\_PROTEIN\_LOCALIZATION\_IN\_ORGANELLE |  | 29 | -0.24 | -0.73 | 0.861 | 0.966 | 1.000 | 4240 | tags=38%, list=35%, signal=58% |
| 6976 | GOBP\_INSULIN\_LIKE\_GROWTH\_FACTOR\_RECEPTOR\_SIGNALING\_PATHWAY |  | 24 | -0.25 | -0.72 | 0.849 | 0.967 | 1.000 | 2351 | tags=25%, list=20%, signal=31% |
| 6977 | GOBP\_REGULATION\_OF\_RETROGRADE\_TRANSPORT\_ENDOSOME\_TO\_GOLGI |  | 3 | -0.43 | -0.72 | 0.868 | 0.968 | 1.000 | 5867 | tags=67%, list=49%, signal=130% |
| 6978 | GOBP\_GAMMA\_AMINOBUTYRIC\_ACID\_SIGNALING\_PATHWAY |  | 6 | -0.36 | -0.72 | 0.840 | 0.968 | 1.000 | 462 | tags=17%, list=4%, signal=17% |
| 6979 | GOBP\_PURINE\_DEOXYRIBONUCLEOSIDE\_TRIPHOSPHATE\_METABOLIC\_PROCESS |  | 6 | -0.36 | -0.72 | 0.846 | 0.968 | 1.000 | 3094 | tags=50%, list=26%, signal=67% |
| 6980 | GOBP\_AXONAL\_TRANSPORT\_OF\_MITOCHONDRION |  | 12 | -0.29 | -0.72 | 0.834 | 0.968 | 1.000 | 5320 | tags=50%, list=44%, signal=90% |
| 6981 | GOCC\_POSTSYNAPTIC\_ENDOSOME |  | 13 | -0.29 | -0.72 | 0.836 | 0.968 | 1.000 | 3651 | tags=31%, list=30%, signal=44% |
| 6982 | GOBP\_POSITIVE\_REGULATION\_OF\_COLLAGEN\_METABOLIC\_PROCESS |  | 20 | -0.26 | -0.72 | 0.848 | 0.968 | 1.000 | 3286 | tags=30%, list=27%, signal=41% |
| 6983 | GOBP\_POSITIVE\_REGULATION\_OF\_CELL\_CYCLE\_G1\_S\_PHASE\_TRANSITION |  | 36 | -0.23 | -0.72 | 0.866 | 0.968 | 1.000 | 5279 | tags=50%, list=44%, signal=89% |
| 6984 | GOMF\_CORE\_PROMOTER\_SEQUENCE\_SPECIFIC\_DNA\_BINDING |  | 26 | -0.25 | -0.72 | 0.863 | 0.968 | 1.000 | 1849 | tags=15%, list=15%, signal=18% |
| 6985 | GOBP\_GDP\_METABOLIC\_PROCESS |  | 8 | -0.32 | -0.72 | 0.829 | 0.968 | 1.000 | 3824 | tags=50%, list=32%, signal=73% |
| 6986 | GOBP\_REELIN\_MEDIATED\_SIGNALING\_PATHWAY |  | 8 | -0.33 | -0.72 | 0.830 | 0.968 | 1.000 | 6221 | tags=75%, list=52%, signal=155% |
| 6987 | GOBP\_PURINE\_NUCLEOSIDE\_MONOPHOSPHATE\_BIOSYNTHETIC\_PROCESS |  | 18 | -0.27 | -0.72 | 0.838 | 0.968 | 1.000 | 4363 | tags=44%, list=36%, signal=70% |
| 6988 | GOBP\_POSITIVE\_REGULATION\_OF\_ASPARTIC\_TYPE\_PEPTIDASE\_ACTIVITY |  | 5 | -0.38 | -0.72 | 0.850 | 0.969 | 1.000 | 2416 | tags=40%, list=20%, signal=50% |
| 6989 | GOBP\_DIACYLGLYCEROL\_BIOSYNTHETIC\_PROCESS |  | 7 | -0.34 | -0.72 | 0.841 | 0.969 | 1.000 | 5263 | tags=71%, list=44%, signal=127% |
| 6990 | GOMF\_2\_ACYLGLYCEROL\_O\_ACYLTRANSFERASE\_ACTIVITY |  | 4 | -0.39 | -0.72 | 0.860 | 0.969 | 1.000 | 639 | tags=25%, list=5%, signal=26% |
| 6991 | GOBP\_POSITIVE\_REGULATION\_OF\_TRANSCRIPTION\_FROM\_RNA\_POLYMERASE\_II\_PROMOTER\_IN\_RESPONSE\_TO\_ENDOPLASMIC\_RETICULUM\_STRESS |  | 9 | -0.32 | -0.72 | 0.823 | 0.969 | 1.000 | 4736 | tags=56%, list=39%, signal=92% |
| 6992 | GOBP\_SPINAL\_CORD\_MOTOR\_NEURON\_DIFFERENTIATION |  | 18 | -0.27 | -0.72 | 0.844 | 0.969 | 1.000 | 1633 | tags=17%, list=14%, signal=19% |
| 6993 | GOBP\_NEGATIVE\_REGULATION\_OF\_ACUTE\_INFLAMMATORY\_RESPONSE |  | 4 | -0.40 | -0.72 | 0.857 | 0.969 | 1.000 | 1267 | tags=50%, list=11%, signal=56% |
| 6994 | GOMF\_S\_ADENOSYLMETHIONINE\_DEPENDENT\_METHYLTRANSFERASE\_ACTIVITY |  | 114 | -0.20 | -0.72 | 0.935 | 0.969 | 1.000 | 4612 | tags=36%, list=38%, signal=58% |
| 6995 | GOBP\_COLUMNAR\_CUBOIDAL\_EPITHELIAL\_CELL\_DIFFERENTIATION |  | 59 | -0.21 | -0.72 | 0.900 | 0.969 | 1.000 | 4432 | tags=39%, list=37%, signal=61% |
| 6996 | GOBP\_GLUCOSAMINE\_CONTAINING\_COMPOUND\_METABOLIC\_PROCESS |  | 17 | -0.27 | -0.72 | 0.843 | 0.969 | 1.000 | 4780 | tags=53%, list=40%, signal=88% |
| 6997 | GOCC\_MITOCHONDRIAL\_PYRUVATE\_DEHYDROGENASE\_COMPLEX |  | 4 | -0.40 | -0.72 | 0.864 | 0.969 | 1.000 | 3922 | tags=50%, list=33%, signal=74% |
| 6998 | GOBP\_POSITIVE\_REGULATION\_OF\_RECEPTOR\_CATABOLIC\_PROCESS |  | 6 | -0.36 | -0.72 | 0.845 | 0.969 | 1.000 | 2894 | tags=50%, list=24%, signal=66% |
| 6999 | GOBP\_MEMBRANE\_BIOGENESIS |  | 28 | -0.24 | -0.72 | 0.863 | 0.970 | 1.000 | 4631 | tags=46%, list=39%, signal=75% |
| 7000 | GOBP\_POSITIVE\_REGULATION\_OF\_MYOBLAST\_FUSION |  | 7 | -0.34 | -0.72 | 0.839 | 0.970 | 1.000 | 1116 | tags=29%, list=9%, signal=31% |
| 7001 | GOBP\_METANEPHROS\_MORPHOGENESIS |  | 19 | -0.26 | -0.72 | 0.850 | 0.970 | 1.000 | 2150 | tags=21%, list=18%, signal=26% |
| 7002 | GOBP\_REGULATION\_OF\_MEMBRANE\_DEPOLARIZATION |  | 30 | -0.24 | -0.72 | 0.868 | 0.970 | 1.000 | 4110 | tags=40%, list=34%, signal=61% |
| 7003 | GOBP\_SULFUR\_AMINO\_ACID\_METABOLIC\_PROCESS |  | 28 | -0.24 | -0.72 | 0.861 | 0.970 | 1.000 | 2746 | tags=25%, list=23%, signal=32% |
| 7004 | GOBP\_REGULATION\_OF\_ESTABLISHMENT\_OR\_MAINTENANCE\_OF\_CELL\_POLARITY |  | 22 | -0.25 | -0.72 | 0.858 | 0.970 | 1.000 | 5833 | tags=59%, list=49%, signal=115% |
| 7005 | GOBP\_POSITIVE\_REGULATION\_OF\_OSTEOBLAST\_DIFFERENTIATION |  | 41 | -0.23 | -0.72 | 0.885 | 0.970 | 1.000 | 3306 | tags=34%, list=28%, signal=47% |
| 7006 | GOMF\_DYNEIN\_INTERMEDIATE\_CHAIN\_BINDING |  | 13 | -0.28 | -0.72 | 0.834 | 0.970 | 1.000 | 5065 | tags=62%, list=42%, signal=106% |
| 7007 | GOBP\_NEGATIVE\_REGULATION\_OF\_DEFENSE\_RESPONSE\_TO\_VIRUS |  | 18 | -0.26 | -0.72 | 0.852 | 0.970 | 1.000 | 3447 | tags=39%, list=29%, signal=54% |
| 7008 | GOBP\_MICROTUBULE\_BUNDLE\_FORMATION |  | 52 | -0.22 | -0.72 | 0.896 | 0.970 | 1.000 | 1971 | tags=17%, list=16%, signal=21% |
| 7009 | GOBP\_CELLULAR\_RESPONSE\_TO\_ARSENIC\_CONTAINING\_SUBSTANCE |  | 15 | -0.27 | -0.72 | 0.836 | 0.970 | 1.000 | 4569 | tags=47%, list=38%, signal=75% |
| 7010 | GOBP\_MATERNAL\_PROCESS\_INVOLVED\_IN\_FEMALE\_PREGNANCY |  | 38 | -0.23 | -0.72 | 0.878 | 0.970 | 1.000 | 1743 | tags=18%, list=15%, signal=21% |
| 7011 | GOBP\_NEGATIVE\_REGULATION\_OF\_CALCIUM\_MEDIATED\_SIGNALING |  | 12 | -0.29 | -0.72 | 0.848 | 0.971 | 1.000 | 6341 | tags=83%, list=53%, signal=176% |
| 7012 | GOBP\_MEMBRANE\_DEPOLARIZATION\_DURING\_AV\_NODE\_CELL\_ACTION\_POTENTIAL |  | 4 | -0.40 | -0.72 | 0.872 | 0.971 | 1.000 | 6880 | tags=75%, list=57%, signal=176% |
| 7013 | GOMF\_TRANSFORMING\_GROWTH\_FACTOR\_BETA\_RECEPTOR\_ACTIVITY\_TYPE\_I |  | 4 | -0.40 | -0.72 | 0.861 | 0.971 | 1.000 | 6049 | tags=75%, list=50%, signal=151% |
| 7014 | GOBP\_NEGATIVE\_REGULATION\_OF\_CELL\_CYCLE\_G2\_M\_PHASE\_TRANSITION |  | 87 | -0.20 | -0.72 | 0.924 | 0.972 | 1.000 | 4076 | tags=25%, list=34%, signal=38% |
| 7015 | GOBP\_REGULATION\_OF\_AUTOPHAGY\_OF\_MITOCHONDRION |  | 26 | -0.24 | -0.72 | 0.871 | 0.972 | 1.000 | 3640 | tags=31%, list=30%, signal=44% |
| 7016 | GOBP\_ENDOCANNABINOID\_SIGNALING\_PATHWAY |  | 4 | -0.39 | -0.72 | 0.864 | 0.972 | 1.000 | 3919 | tags=50%, list=33%, signal=74% |
| 7017 | GOBP\_REGULATION\_OF\_APOPTOTIC\_DNA\_FRAGMENTATION |  | 5 | -0.37 | -0.72 | 0.855 | 0.972 | 1.000 | 3325 | tags=40%, list=28%, signal=55% |
| 7018 | GOBP\_NEURONAL\_ACTION\_POTENTIAL\_PROPAGATION |  | 3 | -0.43 | -0.72 | 0.881 | 0.972 | 1.000 | 6878 | tags=100%, list=57%, signal=234% |
| 7019 | GOBP\_DORSAL\_VENTRAL\_NEURAL\_TUBE\_PATTERNING |  | 16 | -0.27 | -0.72 | 0.849 | 0.972 | 1.000 | 4097 | tags=44%, list=34%, signal=66% |
| 7020 | GOBP\_REGULATION\_OF\_CILIUM\_DEPENDENT\_CELL\_MOTILITY |  | 11 | -0.30 | -0.71 | 0.848 | 0.972 | 1.000 | 4402 | tags=45%, list=37%, signal=72% |
| 7021 | GOBP\_RESPONSE\_TO\_DOPAMINE |  | 44 | -0.22 | -0.71 | 0.893 | 0.972 | 1.000 | 4277 | tags=36%, list=36%, signal=56% |
| 7022 | GOBP\_XENOBIOTIC\_CATABOLIC\_PROCESS |  | 6 | -0.35 | -0.71 | 0.849 | 0.972 | 1.000 | 3930 | tags=50%, list=33%, signal=74% |
| 7023 | GOBP\_POSITIVE\_REGULATION\_OF\_G1\_S\_TRANSITION\_OF\_MITOTIC\_CELL\_CYCLE |  | 28 | -0.24 | -0.71 | 0.871 | 0.972 | 1.000 | 5269 | tags=54%, list=44%, signal=95% |
| 7024 | GOBP\_REGULATION\_OF\_TRANSLATIONAL\_INITIATION\_BY\_EIF2\_ALPHA\_PHOSPHORYLATION |  | 9 | -0.32 | -0.71 | 0.839 | 0.972 | 1.000 | 3872 | tags=44%, list=32%, signal=66% |
| 7025 | GOBP\_NEGATIVE\_REGULATION\_OF\_ATPASE\_ACTIVITY |  | 8 | -0.32 | -0.71 | 0.849 | 0.972 | 1.000 | 2756 | tags=38%, list=23%, signal=49% |
| 7026 | GOBP\_ANTIBACTERIAL\_HUMORAL\_RESPONSE |  | 8 | -0.32 | -0.71 | 0.847 | 0.972 | 1.000 | 989 | tags=13%, list=8%, signal=14% |
| 7027 | GOBP\_REGULATION\_OF\_EXECUTION\_PHASE\_OF\_APOPTOSIS |  | 14 | -0.28 | -0.71 | 0.843 | 0.972 | 1.000 | 1070 | tags=14%, list=9%, signal=16% |
| 7028 | GOCC\_FILTRATION\_DIAPHRAGM |  | 4 | -0.39 | -0.71 | 0.866 | 0.972 | 1.000 | 5950 | tags=50%, list=50%, signal=99% |
| 7029 | GOBP\_PROTEIN\_DESTABILIZATION |  | 36 | -0.23 | -0.71 | 0.886 | 0.972 | 1.000 | 4184 | tags=36%, list=35%, signal=55% |
| 7030 | GOBP\_PROTEIN\_IMPORT\_INTO\_PEROXISOME\_MATRIX |  | 12 | -0.29 | -0.71 | 0.846 | 0.972 | 1.000 | 6662 | tags=58%, list=55%, signal=131% |
| 7031 | GOCC\_PRESPLICEOSOME |  | 13 | -0.29 | -0.71 | 0.842 | 0.972 | 1.000 | 5041 | tags=46%, list=42%, signal=79% |
| 7032 | GOBP\_MITOCHONDRIAL\_FUSION |  | 23 | -0.25 | -0.71 | 0.860 | 0.972 | 1.000 | 4673 | tags=48%, list=39%, signal=78% |
| 7033 | GOBP\_ACTIVATION\_OF\_MAPKKK\_ACTIVITY |  | 9 | -0.31 | -0.71 | 0.843 | 0.972 | 1.000 | 2233 | tags=33%, list=19%, signal=41% |
| 7034 | GOBP\_ENTRAINMENT\_OF\_CIRCADIAN\_CLOCK |  | 22 | -0.25 | -0.71 | 0.858 | 0.972 | 1.000 | 4306 | tags=41%, list=36%, signal=64% |
| 7035 | GOBP\_POSITIVE\_REGULATION\_OF\_SODIUM\_ION\_TRANSMEMBRANE\_TRANSPORT |  | 15 | -0.27 | -0.71 | 0.861 | 0.972 | 1.000 | 4286 | tags=47%, list=36%, signal=72% |
| 7036 | GOBP\_NEGATIVE\_REGULATION\_OF\_RESPONSE\_TO\_OXIDATIVE\_STRESS |  | 16 | -0.27 | -0.71 | 0.845 | 0.972 | 1.000 | 1935 | tags=19%, list=16%, signal=22% |
| 7037 | GOMF\_CYCLIN\_DEPENDENT\_PROTEIN\_SERINE\_THREONINE\_KINASE\_ACTIVATOR\_ACTIVITY |  | 7 | -0.34 | -0.71 | 0.840 | 0.972 | 1.000 | 3331 | tags=29%, list=28%, signal=40% |
| 7038 | GOBP\_CELLULAR\_RESPONSE\_TO\_FRUCTOSE\_STIMULUS |  | 5 | -0.37 | -0.71 | 0.856 | 0.972 | 1.000 | 3205 | tags=60%, list=27%, signal=82% |
| 7039 | GOCC\_DENDRITIC\_SPINE\_NECK |  | 6 | -0.35 | -0.71 | 0.857 | 0.972 | 1.000 | 6533 | tags=83%, list=54%, signal=183% |
| 7040 | GOBP\_REGULATION\_OF\_SYNAPTIC\_VESICLE\_CYCLE |  | 6 | -0.35 | -0.71 | 0.850 | 0.972 | 1.000 | 4008 | tags=67%, list=33%, signal=100% |
| 7041 | GOBP\_CORNEA\_DEVELOPMENT\_IN\_CAMERA\_TYPE\_EYE |  | 3 | -0.43 | -0.71 | 0.889 | 0.972 | 1.000 | 2342 | tags=33%, list=19%, signal=41% |
| 7042 | GOBP\_TRACHEA\_FORMATION |  | 4 | -0.40 | -0.71 | 0.862 | 0.972 | 1.000 | 1692 | tags=25%, list=14%, signal=29% |
| 7043 | GOBP\_CELLULAR\_GLUCURONIDATION |  | 4 | -0.39 | -0.71 | 0.860 | 0.972 | 1.000 | 4100 | tags=75%, list=34%, signal=114% |
| 7044 | GOBP\_POSITIVE\_REGULATION\_OF\_MORPHOGENESIS\_OF\_AN\_EPITHELIUM |  | 19 | -0.26 | -0.71 | 0.857 | 0.972 | 1.000 | 3196 | tags=26%, list=27%, signal=36% |
| 7045 | GOBP\_POSITIVE\_REGULATION\_OF\_CATION\_CHANNEL\_ACTIVITY |  | 41 | -0.22 | -0.71 | 0.886 | 0.972 | 1.000 | 2908 | tags=24%, list=24%, signal=32% |
| 7046 | GOBP\_NEGATIVE\_REGULATION\_OF\_VASCULAR\_ASSOCIATED\_SMOOTH\_MUSCLE\_CELL\_MIGRATION |  | 6 | -0.35 | -0.71 | 0.860 | 0.972 | 1.000 | 2474 | tags=17%, list=21%, signal=21% |
| 7047 | GOBP\_PHARYNGEAL\_ARCH\_ARTERY\_MORPHOGENESIS |  | 6 | -0.35 | -0.71 | 0.858 | 0.972 | 1.000 | 314 | tags=17%, list=3%, signal=17% |
| 7048 | GOCC\_HULC\_COMPLEX |  | 4 | -0.39 | -0.71 | 0.872 | 0.972 | 1.000 | 2118 | tags=25%, list=18%, signal=30% |
| 7049 | GOMF\_MAGNESIUM\_ION\_TRANSMEMBRANE\_TRANSPORTER\_ACTIVITY |  | 12 | -0.29 | -0.71 | 0.849 | 0.972 | 1.000 | 5782 | tags=67%, list=48%, signal=128% |
| 7050 | GOBP\_PARALLEL\_ACTIN\_FILAMENT\_BUNDLE\_ASSEMBLY |  | 4 | -0.39 | -0.71 | 0.869 | 0.972 | 1.000 | 3868 | tags=50%, list=32%, signal=74% |
| 7051 | GOBP\_HISTONE\_H4\_K12\_ACETYLATION |  | 7 | -0.33 | -0.71 | 0.854 | 0.972 | 1.000 | 4393 | tags=43%, list=37%, signal=68% |
| 7052 | GOBP\_POSITIVE\_REGULATION\_OF\_HEAT\_GENERATION |  | 9 | -0.31 | -0.71 | 0.845 | 0.972 | 1.000 | 1408 | tags=22%, list=12%, signal=25% |
| 7053 | GOBP\_POST\_EMBRYONIC\_ANIMAL\_ORGAN\_DEVELOPMENT |  | 10 | -0.30 | -0.71 | 0.847 | 0.972 | 1.000 | 4694 | tags=50%, list=39%, signal=82% |
| 7054 | GOBP\_CHONDROITIN\_SULFATE\_CATABOLIC\_PROCESS |  | 10 | -0.30 | -0.71 | 0.840 | 0.972 | 1.000 | 1517 | tags=20%, list=13%, signal=23% |
| 7055 | GOMF\_SINGLE\_STRANDED\_DNA\_EXODEOXYRIBONUCLEASE\_ACTIVITY |  | 5 | -0.37 | -0.71 | 0.863 | 0.972 | 1.000 | 3298 | tags=40%, list=27%, signal=55% |
| 7056 | GOBP\_FATTY\_ACID\_TRANSMEMBRANE\_TRANSPORT |  | 34 | -0.23 | -0.71 | 0.880 | 0.972 | 1.000 | 1759 | tags=18%, list=15%, signal=21% |
| 7057 | GOMF\_LYASE\_ACTIVITY |  | 139 | -0.19 | -0.71 | 0.953 | 0.972 | 1.000 | 2053 | tags=16%, list=17%, signal=19% |
| 7058 | GOBP\_REGULATION\_OF\_GAP\_JUNCTION\_ASSEMBLY |  | 4 | -0.39 | -0.71 | 0.878 | 0.972 | 1.000 | 3400 | tags=50%, list=28%, signal=70% |
| 7059 | GOBP\_GLOMERULAR\_MESANGIUM\_DEVELOPMENT |  | 12 | -0.29 | -0.71 | 0.852 | 0.972 | 1.000 | 3286 | tags=25%, list=27%, signal=34% |
| 7060 | GOBP\_NUCLEAR\_TRANSCRIBED\_MRNA\_CATABOLIC\_PROCESS |  | 172 | -0.19 | -0.71 | 0.965 | 0.972 | 1.000 | 2182 | tags=13%, list=18%, signal=15% |
| 7061 | GOBP\_HIGH\_DENSITY\_LIPOPROTEIN\_PARTICLE\_CLEARANCE |  | 6 | -0.35 | -0.71 | 0.851 | 0.973 | 1.000 | 4421 | tags=50%, list=37%, signal=79% |
| 7062 | GOBP\_REGULATION\_OF\_HYDROGEN\_PEROXIDE\_BIOSYNTHETIC\_PROCESS |  | 4 | -0.39 | -0.71 | 0.872 | 0.973 | 1.000 | 623 | tags=25%, list=5%, signal=26% |
| 7063 | GOBP\_POSITIVE\_REGULATION\_OF\_TRANSCRIPTION\_FROM\_RNA\_POLYMERASE\_II\_PROMOTER\_INVOLVED\_IN\_CELLULAR\_RESPONSE\_TO\_CHEMICAL\_STIMULUS |  | 17 | -0.26 | -0.71 | 0.859 | 0.973 | 1.000 | 5014 | tags=53%, list=42%, signal=91% |
| 7064 | GOBP\_CELL\_MIGRATION\_INVOLVED\_IN\_KIDNEY\_DEVELOPMENT |  | 5 | -0.37 | -0.71 | 0.861 | 0.973 | 1.000 | 5775 | tags=40%, list=48%, signal=77% |
| 7065 | GOBP\_REGULATION\_OF\_GLUCOCORTICOID\_RECEPTOR\_SIGNALING\_PATHWAY |  | 6 | -0.35 | -0.71 | 0.857 | 0.973 | 1.000 | 4432 | tags=67%, list=37%, signal=106% |
| 7066 | GOBP\_PEPTIDYL\_LYSINE\_HYDROXYLATION |  | 8 | -0.32 | -0.71 | 0.848 | 0.973 | 1.000 | 5624 | tags=50%, list=47%, signal=94% |
| 7067 | GOBP\_GLYCOLIPID\_TRANSPORT |  | 6 | -0.35 | -0.71 | 0.861 | 0.973 | 1.000 | 921 | tags=17%, list=8%, signal=18% |
| 7068 | GOMF\_ADRENERGIC\_RECEPTOR\_BINDING |  | 14 | -0.28 | -0.71 | 0.851 | 0.973 | 1.000 | 3890 | tags=36%, list=32%, signal=53% |
| 7069 | GOMF\_TRNA\_CYTOSINE\_METHYLTRANSFERASE\_ACTIVITY |  | 7 | -0.33 | -0.71 | 0.857 | 0.973 | 1.000 | 2138 | tags=29%, list=18%, signal=35% |
| 7070 | GOBP\_INOSITOL\_PHOSPHATE\_CATABOLIC\_PROCESS |  | 17 | -0.27 | -0.71 | 0.851 | 0.973 | 1.000 | 3934 | tags=41%, list=33%, signal=61% |
| 7071 | GOBP\_RELAXATION\_OF\_MUSCLE |  | 21 | -0.25 | -0.71 | 0.854 | 0.973 | 1.000 | 1304 | tags=14%, list=11%, signal=16% |
| 7072 | GOBP\_REGULATION\_OF\_ACYL\_COA\_BIOSYNTHETIC\_PROCESS |  | 7 | -0.33 | -0.71 | 0.858 | 0.973 | 1.000 | 55 | tags=14%, list=0%, signal=14% |
| 7073 | GOBP\_SPERMIDINE\_METABOLIC\_PROCESS |  | 5 | -0.36 | -0.71 | 0.857 | 0.973 | 1.000 | 4740 | tags=60%, list=39%, signal=99% |
| 7074 | GOBP\_SNRNA\_TRANSCRIPTION |  | 66 | -0.21 | -0.71 | 0.917 | 0.973 | 1.000 | 4962 | tags=36%, list=41%, signal=62% |
| 7075 | GOMF\_ACTININ\_BINDING |  | 25 | -0.24 | -0.71 | 0.875 | 0.973 | 1.000 | 322 | tags=8%, list=3%, signal=8% |
| 7076 | GOBP\_POST\_EMBRYONIC\_CAMERA\_TYPE\_EYE\_DEVELOPMENT |  | 4 | -0.39 | -0.71 | 0.872 | 0.973 | 1.000 | 3196 | tags=50%, list=27%, signal=68% |
| 7077 | GOBP\_REGULATION\_OF\_CARDIAC\_MUSCLE\_CELL\_MEMBRANE\_REPOLARIZATION |  | 16 | -0.27 | -0.71 | 0.859 | 0.973 | 1.000 | 69 | tags=6%, list=1%, signal=6% |
| 7078 | GOBP\_REGULATION\_OF\_EXOSOMAL\_SECRETION |  | 14 | -0.28 | -0.71 | 0.849 | 0.973 | 1.000 | 4716 | tags=43%, list=39%, signal=70% |
| 7079 | GOBP\_SPHINGOSINE\_BIOSYNTHETIC\_PROCESS |  | 9 | -0.31 | -0.71 | 0.843 | 0.973 | 1.000 | 1286 | tags=22%, list=11%, signal=25% |
| 7080 | GOCC\_FICOLIN\_1\_RICH\_GRANULE\_LUMEN |  | 99 | -0.20 | -0.71 | 0.939 | 0.973 | 1.000 | 4212 | tags=30%, list=35%, signal=46% |
| 7081 | GOBP\_OLFACTORY\_BULB\_INTERNEURON\_DIFFERENTIATION |  | 5 | -0.37 | -0.70 | 0.862 | 0.973 | 1.000 | 4696 | tags=40%, list=39%, signal=66% |
| 7082 | GOBP\_REGULATION\_OF\_MITOCHONDRIAL\_FISSION |  | 21 | -0.25 | -0.70 | 0.867 | 0.975 | 1.000 | 4934 | tags=48%, list=41%, signal=81% |
| 7083 | GOBP\_PHOTOTRANSDUCTION\_VISIBLE\_LIGHT |  | 15 | -0.27 | -0.70 | 0.860 | 0.975 | 1.000 | 4787 | tags=47%, list=40%, signal=77% |
| 7084 | GOBP\_REGULATION\_OF\_VACUOLE\_ORGANIZATION |  | 36 | -0.23 | -0.70 | 0.895 | 0.975 | 1.000 | 6443 | tags=58%, list=54%, signal=125% |
| 7085 | GOMF\_NUCLEOSIDE\_TRIPHOSPHATE\_DIPHOSPHATASE\_ACTIVITY |  | 6 | -0.34 | -0.70 | 0.853 | 0.976 | 1.000 | 6065 | tags=67%, list=50%, signal=135% |
| 7086 | GOBP\_MULTI\_MULTICELLULAR\_ORGANISM\_PROCESS |  | 119 | -0.19 | -0.70 | 0.948 | 0.975 | 1.000 | 4342 | tags=35%, list=36%, signal=55% |
| 7087 | GOBP\_MEMBRANE\_FUSION |  | 114 | -0.19 | -0.70 | 0.947 | 0.976 | 1.000 | 3698 | tags=27%, list=31%, signal=39% |
| 7088 | GOBP\_REGULATION\_OF\_ANTIMICROBIAL\_HUMORAL\_RESPONSE |  | 4 | -0.39 | -0.70 | 0.883 | 0.976 | 1.000 | 1838 | tags=25%, list=15%, signal=30% |
| 7089 | GOBP\_PLASMA\_MEMBRANE\_FUSION |  | 3 | -0.42 | -0.70 | 0.890 | 0.976 | 1.000 | 5264 | tags=67%, list=44%, signal=119% |
| 7090 | GOBP\_INTRACILIARY\_RETROGRADE\_TRANSPORT |  | 7 | -0.33 | -0.70 | 0.856 | 0.976 | 1.000 | 4760 | tags=43%, list=40%, signal=71% |
| 7091 | GOCC\_INTEGRATOR\_COMPLEX |  | 14 | -0.28 | -0.70 | 0.860 | 0.976 | 1.000 | 5928 | tags=71%, list=49%, signal=141% |
| 7092 | GOBP\_FEVER\_GENERATION |  | 8 | -0.32 | -0.70 | 0.849 | 0.976 | 1.000 | 5179 | tags=75%, list=43%, signal=132% |
| 7093 | GOBP\_REGULATION\_OF\_SULFUR\_METABOLIC\_PROCESS |  | 9 | -0.31 | -0.70 | 0.862 | 0.976 | 1.000 | 55 | tags=11%, list=0%, signal=11% |
| 7094 | GOBP\_POSITIVE\_REGULATION\_OF\_MESENCHYMAL\_STEM\_CELL\_PROLIFERATION |  | 3 | -0.42 | -0.70 | 0.893 | 0.976 | 1.000 | 7012 | tags=100%, list=58%, signal=240% |
| 7095 | GOMF\_PHOSPHATE\_ION\_BINDING |  | 4 | -0.39 | -0.70 | 0.882 | 0.976 | 1.000 | 1302 | tags=25%, list=11%, signal=28% |
| 7096 | GOCC\_CORTICAL\_ACTIN\_CYTOSKELETON |  | 58 | -0.21 | -0.70 | 0.918 | 0.975 | 1.000 | 2303 | tags=19%, list=19%, signal=23% |
| 7097 | GOMF\_OXIDOREDUCTASE\_ACTIVITY\_ACTING\_ON\_THE\_CH\_CH\_GROUP\_OF\_DONORS\_NAD\_OR\_NADP\_AS\_ACCEPTOR |  | 21 | -0.25 | -0.70 | 0.872 | 0.976 | 1.000 | 1665 | tags=19%, list=14%, signal=22% |
| 7098 | GOMF\_LBD\_DOMAIN\_BINDING |  | 6 | -0.34 | -0.70 | 0.865 | 0.975 | 1.000 | 149 | tags=17%, list=1%, signal=17% |
| 7099 | GOBP\_CELLULAR\_RESPONSE\_TO\_ANTIBIOTIC |  | 10 | -0.30 | -0.70 | 0.853 | 0.976 | 1.000 | 2913 | tags=40%, list=24%, signal=53% |
| 7100 | GOBP\_REGULATION\_OF\_SYNAPTIC\_TRANSMISSION\_DOPAMINERGIC |  | 4 | -0.38 | -0.70 | 0.878 | 0.976 | 1.000 | 1650 | tags=25%, list=14%, signal=29% |
| 7101 | GOBP\_MAINTENANCE\_OF\_APICAL\_BASAL\_CELL\_POLARITY |  | 8 | -0.32 | -0.70 | 0.861 | 0.976 | 1.000 | 4949 | tags=50%, list=41%, signal=85% |
| 7102 | GOBP\_REGULATION\_OF\_PHOSPHOLIPID\_TRANSPORT |  | 10 | -0.30 | -0.70 | 0.864 | 0.976 | 1.000 | 5062 | tags=50%, list=42%, signal=86% |
| 7103 | GOBP\_RNA\_PHOSPHODIESTER\_BOND\_HYDROLYSIS\_EXONUCLEOLYTIC |  | 38 | -0.22 | -0.70 | 0.902 | 0.976 | 1.000 | 5337 | tags=45%, list=44%, signal=80% |
| 7104 | GOBP\_NEGATIVE\_REGULATION\_OF\_MEIOTIC\_CELL\_CYCLE |  | 9 | -0.31 | -0.70 | 0.851 | 0.976 | 1.000 | 6075 | tags=56%, list=51%, signal=112% |
| 7105 | GOMF\_TRNA\_SPECIFIC\_ADENOSINE\_DEAMINASE\_ACTIVITY |  | 4 | -0.39 | -0.70 | 0.883 | 0.975 | 1.000 | 2903 | tags=50%, list=24%, signal=66% |
| 7106 | GOBP\_PROTEIN\_HYDROXYLATION |  | 25 | -0.24 | -0.70 | 0.875 | 0.975 | 1.000 | 6678 | tags=60%, list=56%, signal=135% |
| 7107 | GOBP\_POSITIVE\_REGULATION\_OF\_CARDIOBLAST\_DIFFERENTIATION |  | 3 | -0.42 | -0.70 | 0.901 | 0.975 | 1.000 | 4569 | tags=67%, list=38%, signal=108% |
| 7108 | GOBP\_NEGATIVE\_REGULATION\_OF\_FATTY\_ACID\_METABOLIC\_PROCESS |  | 17 | -0.26 | -0.70 | 0.861 | 0.975 | 1.000 | 6047 | tags=82%, list=50%, signal=166% |
| 7109 | GOMF\_STRUCTURAL\_MOLECULE\_ACTIVITY |  | 423 | -0.18 | -0.70 | 0.995 | 0.975 | 1.000 | 3210 | tags=21%, list=27%, signal=28% |
| 7110 | GOBP\_INTRA\_GOLGI\_VESICLE\_MEDIATED\_TRANSPORT |  | 29 | -0.23 | -0.70 | 0.886 | 0.975 | 1.000 | 6156 | tags=69%, list=51%, signal=141% |
| 7111 | GOBP\_CELL\_PROLIFERATION\_INVOLVED\_IN\_METANEPHROS\_DEVELOPMENT |  | 9 | -0.31 | -0.70 | 0.859 | 0.975 | 1.000 | 3565 | tags=56%, list=30%, signal=79% |
| 7112 | GOBP\_RESPONSE\_TO\_INCREASED\_OXYGEN\_LEVELS |  | 22 | -0.25 | -0.70 | 0.878 | 0.976 | 1.000 | 3675 | tags=41%, list=31%, signal=59% |
| 7113 | GOBP\_NEGATIVE\_REGULATION\_OF\_MACROPHAGE\_ACTIVATION |  | 14 | -0.27 | -0.70 | 0.859 | 0.975 | 1.000 | 4329 | tags=43%, list=36%, signal=67% |
| 7114 | GOBP\_TRANSCRIPTION\_INITIATION\_FROM\_RNA\_POLYMERASE\_I\_PROMOTER |  | 26 | -0.24 | -0.70 | 0.873 | 0.975 | 1.000 | 5069 | tags=42%, list=42%, signal=73% |
| 7115 | GOCC\_GOLGI\_CISTERNA\_MEMBRANE |  | 53 | -0.21 | -0.70 | 0.911 | 0.975 | 1.000 | 4540 | tags=42%, list=38%, signal=66% |
| 7116 | GOMF\_ACTIVIN\_RECEPTOR\_ACTIVITY\_TYPE\_I |  | 4 | -0.39 | -0.70 | 0.880 | 0.975 | 1.000 | 6049 | tags=75%, list=50%, signal=151% |
| 7117 | GOBP\_DNA\_DEALKYLATION\_INVOLVED\_IN\_DNA\_REPAIR |  | 10 | -0.30 | -0.70 | 0.862 | 0.975 | 1.000 | 4508 | tags=50%, list=38%, signal=80% |
| 7118 | GOBP\_PENILE\_ERECTION |  | 8 | -0.32 | -0.70 | 0.861 | 0.975 | 1.000 | 2106 | tags=38%, list=18%, signal=45% |
| 7119 | GOBP\_HEPATIC\_STELLATE\_CELL\_ACTIVATION |  | 5 | -0.36 | -0.70 | 0.886 | 0.975 | 1.000 | 4698 | tags=60%, list=39%, signal=99% |
| 7120 | GOBP\_POSITIVE\_REGULATION\_OF\_MEGAKARYOCYTE\_DIFFERENTIATION |  | 8 | -0.32 | -0.70 | 0.861 | 0.975 | 1.000 | 2287 | tags=25%, list=19%, signal=31% |
| 7121 | GOMF\_DNA\_N\_GLYCOSYLASE\_ACTIVITY |  | 10 | -0.30 | -0.70 | 0.862 | 0.975 | 1.000 | 2346 | tags=20%, list=20%, signal=25% |
| 7122 | GOCC\_DNA\_HELICASE\_COMPLEX |  | 11 | -0.29 | -0.70 | 0.860 | 0.975 | 1.000 | 3697 | tags=45%, list=31%, signal=66% |
| 7123 | GOBP\_RETINA\_HOMEOSTASIS |  | 37 | -0.22 | -0.70 | 0.898 | 0.975 | 1.000 | 3868 | tags=32%, list=32%, signal=48% |
| 7124 | GOMF\_INTERLEUKIN\_1\_BINDING |  | 4 | -0.39 | -0.70 | 0.880 | 0.975 | 1.000 | 3690 | tags=75%, list=31%, signal=108% |
| 7125 | GOBP\_MESENCHYMAL\_STEM\_CELL\_PROLIFERATION |  | 3 | -0.42 | -0.70 | 0.899 | 0.976 | 1.000 | 7012 | tags=100%, list=58%, signal=240% |
| 7126 | GOBP\_POSITIVE\_REGULATION\_OF\_CHROMOSOME\_SEGREGATION |  | 13 | -0.28 | -0.70 | 0.867 | 0.975 | 1.000 | 6407 | tags=54%, list=53%, signal=115% |
| 7127 | GOBP\_DETECTION\_OF\_ABIOTIC\_STIMULUS |  | 65 | -0.20 | -0.70 | 0.926 | 0.975 | 1.000 | 4402 | tags=40%, list=37%, signal=63% |
| 7128 | GOBP\_TERTIARY\_ALCOHOL\_METABOLIC\_PROCESS |  | 12 | -0.28 | -0.70 | 0.862 | 0.976 | 1.000 | 1899 | tags=25%, list=16%, signal=30% |
| 7129 | GOBP\_ENDOTHELIAL\_CELL\_MORPHOGENESIS |  | 8 | -0.32 | -0.70 | 0.866 | 0.976 | 1.000 | 4265 | tags=50%, list=36%, signal=77% |
| 7130 | GOMF\_OXIDOREDUCTASE\_ACTIVITY\_ACTING\_ON\_THE\_CH\_NH\_GROUP\_OF\_DONORS |  | 19 | -0.25 | -0.70 | 0.872 | 0.976 | 1.000 | 4734 | tags=42%, list=39%, signal=69% |
| 7131 | GOMF\_THIOL\_OXIDASE\_ACTIVITY |  | 6 | -0.34 | -0.70 | 0.865 | 0.976 | 1.000 | 2363 | tags=33%, list=20%, signal=41% |
| 7132 | GOBP\_MITOCHONDRIAL\_DNA\_METABOLIC\_PROCESS |  | 11 | -0.29 | -0.69 | 0.869 | 0.976 | 1.000 | 3376 | tags=36%, list=28%, signal=51% |
| 7133 | GOBP\_NEGATIVE\_REGULATION\_OF\_AUTOPHAGOSOME\_ASSEMBLY |  | 11 | -0.29 | -0.69 | 0.864 | 0.976 | 1.000 | 3483 | tags=27%, list=29%, signal=38% |
| 7134 | GOMF\_ADENYLYLTRANSFERASE\_ACTIVITY |  | 14 | -0.27 | -0.69 | 0.873 | 0.976 | 1.000 | 4765 | tags=57%, list=40%, signal=95% |
| 7135 | GOBP\_POTASSIUM\_ION\_EXPORT\_ACROSS\_PLASMA\_MEMBRANE |  | 9 | -0.30 | -0.69 | 0.867 | 0.976 | 1.000 | 2604 | tags=33%, list=22%, signal=43% |
| 7136 | GOBP\_GOLGI\_RIBBON\_FORMATION |  | 10 | -0.29 | -0.69 | 0.865 | 0.976 | 1.000 | 6578 | tags=80%, list=55%, signal=177% |
| 7137 | GOBP\_NUCLEOSIDE\_BISPHOSPHATE\_BIOSYNTHETIC\_PROCESS |  | 56 | -0.21 | -0.69 | 0.914 | 0.977 | 1.000 | 504 | tags=9%, list=4%, signal=9% |
| 7138 | GOBP\_POSITIVE\_REGULATION\_OF\_STEROID\_BIOSYNTHETIC\_PROCESS |  | 11 | -0.29 | -0.69 | 0.866 | 0.977 | 1.000 | 4271 | tags=55%, list=36%, signal=85% |
| 7139 | GOBP\_C21\_STEROID\_HORMONE\_METABOLIC\_PROCESS |  | 22 | -0.24 | -0.69 | 0.885 | 0.977 | 1.000 | 2374 | tags=27%, list=20%, signal=34% |
| 7140 | GOBP\_REGULATION\_OF\_SERINE\_C\_PALMITOYLTRANSFERASE\_ACTIVITY |  | 5 | -0.36 | -0.69 | 0.879 | 0.977 | 1.000 | 1501 | tags=40%, list=12%, signal=46% |
| 7141 | GOBP\_EXCITATORY\_SYNAPSE\_ASSEMBLY |  | 14 | -0.27 | -0.69 | 0.867 | 0.977 | 1.000 | 5415 | tags=64%, list=45%, signal=117% |
| 7142 | GOBP\_RESPONSE\_TO\_PEPTIDOGLYCAN |  | 6 | -0.34 | -0.69 | 0.870 | 0.977 | 1.000 | 5729 | tags=67%, list=48%, signal=127% |
| 7143 | GOCC\_SIN3\_TYPE\_COMPLEX |  | 12 | -0.28 | -0.69 | 0.868 | 0.977 | 1.000 | 6612 | tags=83%, list=55%, signal=185% |
| 7144 | GOBP\_CHROMATIN\_DISASSEMBLY |  | 13 | -0.28 | -0.69 | 0.866 | 0.977 | 1.000 | 3348 | tags=23%, list=28%, signal=32% |
| 7145 | GOMF\_FATTY\_ACID\_OMEGA\_HYDROXYLASE\_ACTIVITY |  | 4 | -0.38 | -0.69 | 0.884 | 0.978 | 1.000 | 3078 | tags=50%, list=26%, signal=67% |
| 7146 | GOBP\_REGULATION\_OF\_RECEPTOR\_BINDING |  | 14 | -0.27 | -0.69 | 0.870 | 0.978 | 1.000 | 1455 | tags=21%, list=12%, signal=24% |
| 7147 | GOBP\_TRANSLOCATION\_OF\_MOLECULES\_INTO\_HOST |  | 7 | -0.33 | -0.69 | 0.873 | 0.978 | 1.000 | 1775 | tags=14%, list=15%, signal=17% |
| 7148 | GOMF\_SPHINGOMYELIN\_PHOSPHODIESTERASE\_ACTIVITY |  | 5 | -0.36 | -0.69 | 0.875 | 0.978 | 1.000 | 4433 | tags=60%, list=37%, signal=95% |
| 7149 | GOBP\_ETHER\_BIOSYNTHETIC\_PROCESS |  | 9 | -0.30 | -0.69 | 0.867 | 0.978 | 1.000 | 231 | tags=11%, list=2%, signal=11% |
| 7150 | GOCC\_METHYLOSOME |  | 9 | -0.30 | -0.69 | 0.876 | 0.978 | 1.000 | 1068 | tags=11%, list=9%, signal=12% |
| 7151 | GOBP\_POST\_EMBRYONIC\_ANIMAL\_MORPHOGENESIS |  | 8 | -0.31 | -0.69 | 0.866 | 0.978 | 1.000 | 4694 | tags=63%, list=39%, signal=103% |
| 7152 | GOBP\_REGULATION\_OF\_SKELETAL\_MUSCLE\_CONTRACTION |  | 4 | -0.38 | -0.69 | 0.886 | 0.978 | 1.000 | 7453 | tags=100%, list=62%, signal=263% |
| 7153 | GOBP\_RESPONSE\_TO\_FOLIC\_ACID |  | 6 | -0.34 | -0.69 | 0.877 | 0.978 | 1.000 | 2580 | tags=33%, list=21%, signal=42% |
| 7154 | GOBP\_NEGATIVE\_REGULATION\_OF\_SEROTONIN\_SECRETION |  | 3 | -0.41 | -0.69 | 0.911 | 0.979 | 1.000 | 897 | tags=33%, list=7%, signal=36% |
| 7155 | GOCC\_TRANSLATION\_PREINITIATION\_COMPLEX |  | 15 | -0.26 | -0.69 | 0.880 | 0.979 | 1.000 | 2370 | tags=13%, list=20%, signal=17% |
| 7156 | GOBP\_CAMERA\_TYPE\_EYE\_PHOTORECEPTOR\_CELL\_DIFFERENTIATION |  | 15 | -0.26 | -0.69 | 0.875 | 0.979 | 1.000 | 3868 | tags=40%, list=32%, signal=59% |
| 7157 | GOBP\_MAGNESIUM\_ION\_HOMEOSTASIS |  | 6 | -0.34 | -0.69 | 0.877 | 0.979 | 1.000 | 597 | tags=17%, list=5%, signal=18% |
| 7158 | GOBP\_EPITHELIAL\_CELL\_MORPHOGENESIS |  | 20 | -0.25 | -0.69 | 0.880 | 0.979 | 1.000 | 4429 | tags=50%, list=37%, signal=79% |
| 7159 | GOMF\_CYCLIN\_DEPENDENT\_PROTEIN\_SERINE\_THREONINE\_KINASE\_INHIBITOR\_ACTIVITY |  | 7 | -0.32 | -0.69 | 0.863 | 0.979 | 1.000 | 5564 | tags=71%, list=46%, signal=133% |
| 7160 | GOMF\_FATTY\_ACID\_TRANSMEMBRANE\_TRANSPORTER\_ACTIVITY |  | 12 | -0.28 | -0.69 | 0.876 | 0.979 | 1.000 | 1236 | tags=17%, list=10%, signal=19% |
| 7161 | GOBP\_REGULATION\_OF\_PROTEIN\_LOCALIZATION\_TO\_SYNAPSE |  | 18 | -0.25 | -0.69 | 0.884 | 0.979 | 1.000 | 4655 | tags=44%, list=39%, signal=72% |
| 7162 | GOBP\_NEGATIVE\_REGULATION\_OF\_CHEMOKINE\_PRODUCTION |  | 12 | -0.28 | -0.69 | 0.878 | 0.979 | 1.000 | 2480 | tags=25%, list=21%, signal=31% |
| 7163 | GOBP\_PLATELET\_DENSE\_GRANULE\_ORGANIZATION |  | 7 | -0.32 | -0.69 | 0.875 | 0.979 | 1.000 | 3112 | tags=43%, list=26%, signal=58% |
| 7164 | GOBP\_THIOESTER\_BIOSYNTHETIC\_PROCESS |  | 44 | -0.21 | -0.69 | 0.919 | 0.979 | 1.000 | 4131 | tags=36%, list=34%, signal=55% |
| 7165 | GOBP\_INTESTINAL\_EPITHELIAL\_STRUCTURE\_MAINTENANCE |  | 5 | -0.36 | -0.69 | 0.891 | 0.979 | 1.000 | 2557 | tags=40%, list=21%, signal=51% |
| 7166 | GOBP\_MAINTENANCE\_OF\_DNA\_REPEAT\_ELEMENTS |  | 6 | -0.34 | -0.69 | 0.880 | 0.979 | 1.000 | 694 | tags=17%, list=6%, signal=18% |
| 7167 | GOBP\_TOLL\_LIKE\_RECEPTOR\_3\_SIGNALING\_PATHWAY |  | 16 | -0.26 | -0.69 | 0.878 | 0.979 | 1.000 | 4211 | tags=50%, list=35%, signal=77% |
| 7168 | GOBP\_APOPTOTIC\_PROCESS\_INVOLVED\_IN\_BLOOD\_VESSEL\_MORPHOGENESIS |  | 4 | -0.38 | -0.69 | 0.884 | 0.979 | 1.000 | 624 | tags=25%, list=5%, signal=26% |
| 7169 | GOMF\_PROTEIN\_PRENYLTRANSFERASE\_ACTIVITY |  | 7 | -0.32 | -0.69 | 0.868 | 0.979 | 1.000 | 330 | tags=14%, list=3%, signal=15% |
| 7170 | GOMF\_TYPE\_I\_TRANSFORMING\_GROWTH\_FACTOR\_BETA\_RECEPTOR\_BINDING |  | 8 | -0.31 | -0.69 | 0.874 | 0.979 | 1.000 | 2457 | tags=25%, list=20%, signal=31% |
| 7171 | GOBP\_TRIGEMINAL\_NERVE\_DEVELOPMENT |  | 3 | -0.41 | -0.69 | 0.909 | 0.979 | 1.000 | 4739 | tags=33%, list=39%, signal=55% |
| 7172 | GOBP\_NEGATIVE\_REGULATION\_OF\_GOLGI\_TO\_PLASMA\_MEMBRANE\_PROTEIN\_TRANSPORT |  | 3 | -0.41 | -0.68 | 0.906 | 0.980 | 1.000 | 2891 | tags=33%, list=24%, signal=44% |
| 7173 | GOBP\_AUTOPHAGY\_OF\_MITOCHONDRION |  | 60 | -0.20 | -0.68 | 0.934 | 0.980 | 1.000 | 3843 | tags=30%, list=32%, signal=44% |
| 7174 | GOBP\_POSITIVE\_REGULATION\_OF\_ORGANIC\_ACID\_TRANSPORT |  | 25 | -0.24 | -0.68 | 0.890 | 0.979 | 1.000 | 2769 | tags=28%, list=23%, signal=36% |
| 7175 | GOMF\_BUTYRATE\_COA\_LIGASE\_ACTIVITY |  | 3 | -0.41 | -0.68 | 0.894 | 0.980 | 1.000 | 3147 | tags=33%, list=26%, signal=45% |
| 7176 | GOCC\_EXTRINSIC\_COMPONENT\_OF\_ENDOPLASMIC\_RETICULUM\_MEMBRANE |  | 5 | -0.35 | -0.68 | 0.888 | 0.979 | 1.000 | 1051 | tags=20%, list=9%, signal=22% |
| 7177 | GOMF\_INOSITOL\_1\_4\_5\_TRISPHOSPHATE\_BINDING |  | 9 | -0.30 | -0.68 | 0.878 | 0.979 | 1.000 | 245 | tags=11%, list=2%, signal=11% |
| 7178 | GOBP\_THALAMUS\_DEVELOPMENT |  | 9 | -0.30 | -0.68 | 0.868 | 0.980 | 1.000 | 405 | tags=11%, list=3%, signal=11% |
| 7179 | GOBP\_PROTEIN\_DEUBIQUITINATION\_INVOLVED\_IN\_UBIQUITIN\_DEPENDENT\_PROTEIN\_CATABOLIC\_PROCESS |  | 4 | -0.38 | -0.68 | 0.894 | 0.980 | 1.000 | 1457 | tags=25%, list=12%, signal=28% |
| 7180 | GOCC\_POSTSYNAPTIC\_RECYCLING\_ENDOSOME |  | 7 | -0.32 | -0.68 | 0.880 | 0.980 | 1.000 | 3323 | tags=29%, list=28%, signal=39% |
| 7181 | GOCC\_CCR4\_NOT\_COMPLEX |  | 13 | -0.27 | -0.68 | 0.876 | 0.979 | 1.000 | 2441 | tags=23%, list=20%, signal=29% |
| 7182 | GOBP\_DNA\_SYNTHESIS\_INVOLVED\_IN\_DNA\_REPAIR |  | 37 | -0.22 | -0.68 | 0.912 | 0.980 | 1.000 | 5351 | tags=51%, list=45%, signal=92% |
| 7183 | GOBP\_FATTY\_ACID\_ELONGATION |  | 9 | -0.30 | -0.68 | 0.873 | 0.980 | 1.000 | 4689 | tags=67%, list=39%, signal=109% |
| 7184 | GOBP\_PYRIMIDINE\_NUCLEOSIDE\_MONOPHOSPHATE\_BIOSYNTHETIC\_PROCESS |  | 13 | -0.27 | -0.68 | 0.872 | 0.980 | 1.000 | 4973 | tags=31%, list=41%, signal=52% |
| 7185 | GOBP\_NEGATIVE\_REGULATION\_OF\_SMAD\_PROTEIN\_SIGNAL\_TRANSDUCTION |  | 4 | -0.38 | -0.68 | 0.894 | 0.980 | 1.000 | 2852 | tags=50%, list=24%, signal=66% |
| 7186 | GOBP\_FOREBRAIN\_REGIONALIZATION |  | 9 | -0.30 | -0.68 | 0.874 | 0.980 | 1.000 | 1101 | tags=22%, list=9%, signal=24% |
| 7187 | GOBP\_L\_AMINO\_ACID\_TRANSPORT |  | 37 | -0.22 | -0.68 | 0.913 | 0.980 | 1.000 | 3155 | tags=27%, list=26%, signal=37% |
| 7188 | GOBP\_POSITIVE\_REGULATION\_OF\_SKELETAL\_MUSCLE\_CELL\_DIFFERENTIATION |  | 4 | -0.38 | -0.68 | 0.894 | 0.980 | 1.000 | 4432 | tags=50%, list=37%, signal=79% |
| 7189 | GOBP\_HEMATOPOIETIC\_STEM\_CELL\_PROLIFERATION |  | 18 | -0.25 | -0.68 | 0.879 | 0.980 | 1.000 | 3862 | tags=33%, list=32%, signal=49% |
| 7190 | GOBP\_PROTEIN\_K48\_LINKED\_UBIQUITINATION |  | 49 | -0.21 | -0.68 | 0.926 | 0.980 | 1.000 | 3837 | tags=29%, list=32%, signal=42% |
| 7191 | GOBP\_POSITIVE\_REGULATION\_OF\_VASCULAR\_WOUND\_HEALING |  | 3 | -0.41 | -0.68 | 0.908 | 0.980 | 1.000 | 4112 | tags=67%, list=34%, signal=101% |
| 7192 | GOBP\_ICOSANOID\_CATABOLIC\_PROCESS |  | 3 | -0.41 | -0.68 | 0.911 | 0.981 | 1.000 | 7078 | tags=100%, list=59%, signal=243% |
| 7193 | GOBP\_REGULATION\_OF\_TYPE\_I\_INTERFERON\_MEDIATED\_SIGNALING\_PATHWAY |  | 27 | -0.23 | -0.68 | 0.901 | 0.980 | 1.000 | 4965 | tags=48%, list=41%, signal=82% |
| 7194 | GOMF\_TRANSLATION\_INITIATION\_FACTOR\_BINDING |  | 24 | -0.24 | -0.68 | 0.899 | 0.980 | 1.000 | 765 | tags=8%, list=6%, signal=9% |
| 7195 | GOBP\_REGULATION\_OF\_PROSTATIC\_BUD\_FORMATION |  | 6 | -0.33 | -0.68 | 0.880 | 0.980 | 1.000 | 866 | tags=17%, list=7%, signal=18% |
| 7196 | GOBP\_PROGESTERONE\_BIOSYNTHETIC\_PROCESS |  | 4 | -0.38 | -0.68 | 0.899 | 0.980 | 1.000 | 2374 | tags=50%, list=20%, signal=62% |
| 7197 | GOBP\_LATE\_ENDOSOME\_TO\_GOLGI\_TRANSPORT |  | 5 | -0.35 | -0.68 | 0.874 | 0.980 | 1.000 | 2296 | tags=20%, list=19%, signal=25% |
| 7198 | GOBP\_MAINTENANCE\_OF\_PROTEIN\_LOCATION\_IN\_EXTRACELLULAR\_REGION |  | 5 | -0.35 | -0.68 | 0.884 | 0.981 | 1.000 | 3090 | tags=40%, list=26%, signal=54% |
| 7199 | GOBP\_REGULATION\_OF\_DOUBLE\_STRAND\_BREAK\_REPAIR\_VIA\_NONHOMOLOGOUS\_END\_JOINING |  | 19 | -0.25 | -0.68 | 0.885 | 0.980 | 1.000 | 1490 | tags=16%, list=12%, signal=18% |
| 7200 | GOBP\_PLASMA\_MEMBRANE\_ORGANIZATION |  | 78 | -0.19 | -0.68 | 0.944 | 0.980 | 1.000 | 4112 | tags=35%, list=34%, signal=52% |
| 7201 | GOBP\_RENAL\_WATER\_TRANSPORT |  | 5 | -0.35 | -0.68 | 0.893 | 0.980 | 1.000 | 3934 | tags=40%, list=33%, signal=59% |
| 7202 | GOBP\_UBIQUITIN\_DEPENDENT\_PROTEIN\_CATABOLIC\_PROCESS\_VIA\_THE\_MULTIVESICULAR\_BODY\_SORTING\_PATHWAY |  | 21 | -0.24 | -0.68 | 0.896 | 0.980 | 1.000 | 2620 | tags=24%, list=22%, signal=30% |
| 7203 | GOCC\_U1\_SNRNP |  | 14 | -0.26 | -0.68 | 0.875 | 0.980 | 1.000 | 5041 | tags=36%, list=42%, signal=61% |
| 7204 | GOMF\_AROMATIC\_AMINO\_ACID\_TRANSMEMBRANE\_TRANSPORTER\_ACTIVITY |  | 7 | -0.32 | -0.68 | 0.878 | 0.980 | 1.000 | 3155 | tags=29%, list=26%, signal=39% |
| 7205 | GOBP\_OSTEOBLAST\_DEVELOPMENT |  | 12 | -0.28 | -0.68 | 0.878 | 0.980 | 1.000 | 2002 | tags=25%, list=17%, signal=30% |
| 7206 | GOBP\_MRNA\_3\_END\_PROCESSING |  | 78 | -0.19 | -0.68 | 0.953 | 0.981 | 1.000 | 5194 | tags=35%, list=43%, signal=61% |
| 7207 | GOBP\_PATHWAY\_RESTRICTED\_SMAD\_PROTEIN\_PHOSPHORYLATION |  | 45 | -0.21 | -0.68 | 0.923 | 0.981 | 1.000 | 3269 | tags=27%, list=27%, signal=37% |
| 7208 | GOCC\_DYSTROGLYCAN\_COMPLEX |  | 5 | -0.35 | -0.68 | 0.889 | 0.981 | 1.000 | 5997 | tags=60%, list=50%, signal=120% |
| 7209 | GOBP\_NUCLEOSIDE\_CATABOLIC\_PROCESS |  | 21 | -0.24 | -0.68 | 0.895 | 0.981 | 1.000 | 2053 | tags=19%, list=17%, signal=23% |
| 7210 | GOCC\_MEDIATOR\_COMPLEX |  | 33 | -0.22 | -0.68 | 0.915 | 0.981 | 1.000 | 5477 | tags=52%, list=46%, signal=94% |
| 7211 | GOBP\_NEGATIVE\_REGULATION\_OF\_MRNA\_SPLICING\_VIA\_SPLICEOSOME |  | 15 | -0.26 | -0.68 | 0.882 | 0.980 | 1.000 | 4223 | tags=40%, list=35%, signal=62% |
| 7212 | GOBP\_SRP\_DEPENDENT\_COTRANSLATIONAL\_PROTEIN\_TARGETING\_TO\_MEMBRANE\_SIGNAL\_SEQUENCE\_RECOGNITION |  | 3 | -0.41 | -0.68 | 0.916 | 0.981 | 1.000 | 6470 | tags=67%, list=54%, signal=144% |
| 7213 | GOMF\_EXODEOXYRIBONUCLEASE\_ACTIVITY |  | 13 | -0.27 | -0.68 | 0.887 | 0.981 | 1.000 | 4024 | tags=38%, list=34%, signal=58% |
| 7214 | GOMF\_SERINE\_TYPE\_ENDOPEPTIDASE\_INHIBITOR\_ACTIVITY |  | 32 | -0.22 | -0.68 | 0.907 | 0.981 | 1.000 | 1010 | tags=9%, list=8%, signal=10% |
| 7215 | GOBP\_REGULATION\_OF\_TRIGLYCERIDE\_CATABOLIC\_PROCESS |  | 8 | -0.31 | -0.68 | 0.881 | 0.981 | 1.000 | 261 | tags=13%, list=2%, signal=13% |
| 7216 | GOCC\_EUKARYOTIC\_TRANSLATION\_INITIATION\_FACTOR\_3\_COMPLEX\_EIF3M |  | 6 | -0.33 | -0.68 | 0.891 | 0.982 | 1.000 | 2370 | tags=17%, list=20%, signal=21% |
| 7217 | GOCC\_THO\_COMPLEX |  | 5 | -0.35 | -0.67 | 0.894 | 0.982 | 1.000 | 1775 | tags=20%, list=15%, signal=23% |
| 7218 | GOBP\_MAGNESIUM\_ION\_TRANSPORT |  | 13 | -0.27 | -0.67 | 0.881 | 0.982 | 1.000 | 5782 | tags=62%, list=48%, signal=119% |
| 7219 | GOBP\_RNA\_SPLICING |  | 334 | -0.17 | -0.67 | 0.996 | 0.982 | 1.000 | 6335 | tags=48%, list=53%, signal=99% |
| 7220 | GOBP\_NEURAL\_TUBE\_PATTERNING |  | 25 | -0.23 | -0.67 | 0.900 | 0.982 | 1.000 | 1031 | tags=16%, list=9%, signal=17% |
| 7221 | GOBP\_REGULATION\_OF\_BLOOD\_VOLUME\_BY\_RENIN\_ANGIOTENSIN |  | 5 | -0.35 | -0.67 | 0.895 | 0.982 | 1.000 | 4836 | tags=60%, list=40%, signal=100% |
| 7222 | GOBP\_SECONDARY\_HEART\_FIELD\_SPECIFICATION |  | 8 | -0.31 | -0.67 | 0.874 | 0.982 | 1.000 | 2474 | tags=25%, list=21%, signal=31% |
| 7223 | GOBP\_C\_TERMINAL\_PROTEIN\_DEGLUTAMYLATION |  | 3 | -0.41 | -0.67 | 0.920 | 0.982 | 1.000 | 6305 | tags=67%, list=52%, signal=140% |
| 7224 | GOBP\_PRENYLATION |  | 8 | -0.31 | -0.67 | 0.886 | 0.981 | 1.000 | 330 | tags=13%, list=3%, signal=13% |
| 7225 | GOBP\_N\_TERMINAL\_PROTEIN\_LIPIDATION |  | 5 | -0.35 | -0.67 | 0.893 | 0.981 | 1.000 | 3447 | tags=40%, list=29%, signal=56% |
| 7226 | GOBP\_INTRACELLULAR\_MRNA\_LOCALIZATION |  | 10 | -0.29 | -0.67 | 0.876 | 0.981 | 1.000 | 5584 | tags=40%, list=46%, signal=75% |
| 7227 | GOMF\_1\_4\_ALPHA\_OLIGOGLUCAN\_PHOSPHORYLASE\_ACTIVITY |  | 4 | -0.37 | -0.67 | 0.891 | 0.982 | 1.000 | 1018 | tags=25%, list=8%, signal=27% |
| 7228 | GOBP\_POSITIVE\_REGULATION\_OF\_TRIGLYCERIDE\_METABOLIC\_PROCESS |  | 15 | -0.26 | -0.67 | 0.880 | 0.982 | 1.000 | 4853 | tags=47%, list=40%, signal=78% |
| 7229 | GOBP\_NEGATIVE\_REGULATION\_OF\_DELAYED\_RECTIFIER\_POTASSIUM\_CHANNEL\_ACTIVITY |  | 6 | -0.33 | -0.67 | 0.883 | 0.982 | 1.000 | 1125 | tags=17%, list=9%, signal=18% |
| 7230 | GOBP\_T\_HELPER\_1\_CELL\_DIFFERENTIATION |  | 13 | -0.27 | -0.67 | 0.881 | 0.982 | 1.000 | 2345 | tags=23%, list=20%, signal=29% |
| 7231 | GOBP\_POSITIVE\_REGULATION\_OF\_GLIOGENESIS |  | 45 | -0.21 | -0.67 | 0.925 | 0.982 | 1.000 | 1847 | tags=18%, list=15%, signal=21% |
| 7232 | GOMF\_PHOSPHATIDYLCHOLINE\_FLIPPASE\_ACTIVITY |  | 5 | -0.35 | -0.67 | 0.891 | 0.982 | 1.000 | 3366 | tags=40%, list=28%, signal=56% |
| 7233 | GOBP\_NEGATIVE\_REGULATION\_OF\_EPITHELIAL\_TO\_MESENCHYMAL\_TRANSITION |  | 25 | -0.23 | -0.67 | 0.904 | 0.981 | 1.000 | 3530 | tags=36%, list=29%, signal=51% |
| 7234 | GOBP\_REGULATION\_OF\_RELEASE\_OF\_CYTOCHROME\_C\_FROM\_MITOCHONDRIA |  | 27 | -0.23 | -0.67 | 0.910 | 0.982 | 1.000 | 4673 | tags=44%, list=39%, signal=73% |
| 7235 | GOBP\_VESTIBULOCOCHLEAR\_NERVE\_DEVELOPMENT |  | 4 | -0.37 | -0.67 | 0.911 | 0.983 | 1.000 | 1660 | tags=25%, list=14%, signal=29% |
| 7236 | GOBP\_REGULATION\_OF\_PHENOTYPIC\_SWITCHING |  | 3 | -0.40 | -0.67 | 0.917 | 0.983 | 1.000 | 7004 | tags=67%, list=58%, signal=160% |
| 7237 | GOBP\_PROTEIN\_DEGLUTAMYLATION |  | 4 | -0.37 | -0.67 | 0.901 | 0.983 | 1.000 | 7536 | tags=100%, list=63%, signal=268% |
| 7238 | GOCC\_PROTEIN\_LIPID\_COMPLEX |  | 18 | -0.25 | -0.67 | 0.891 | 0.983 | 1.000 | 2159 | tags=22%, list=18%, signal=27% |
| 7239 | GOCC\_CYTOPLASMIC\_UBIQUITIN\_LIGASE\_COMPLEX |  | 10 | -0.29 | -0.67 | 0.886 | 0.983 | 1.000 | 4890 | tags=50%, list=41%, signal=84% |
| 7240 | GOMF\_ABC\_TYPE\_XENOBIOTIC\_TRANSPORTER\_ACTIVITY |  | 10 | -0.28 | -0.67 | 0.883 | 0.983 | 1.000 | 2842 | tags=40%, list=24%, signal=52% |
| 7241 | GOBP\_PHARYNGEAL\_SYSTEM\_DEVELOPMENT |  | 14 | -0.26 | -0.67 | 0.889 | 0.983 | 1.000 | 1031 | tags=14%, list=9%, signal=16% |
| 7242 | GOBP\_BEHAVIORAL\_RESPONSE\_TO\_ETHANOL |  | 3 | -0.40 | -0.67 | 0.919 | 0.983 | 1.000 | 7189 | tags=100%, list=60%, signal=249% |
| 7243 | GOBP\_PONS\_DEVELOPMENT |  | 5 | -0.35 | -0.67 | 0.891 | 0.982 | 1.000 | 2992 | tags=40%, list=25%, signal=53% |
| 7244 | GOBP\_CELL\_DEATH\_IN\_RESPONSE\_TO\_HYDROGEN\_PEROXIDE |  | 19 | -0.24 | -0.67 | 0.894 | 0.982 | 1.000 | 1935 | tags=16%, list=16%, signal=19% |
| 7245 | GOBP\_ENDONUCLEOLYTIC\_CLEAVAGE\_IN\_5\_ETS\_OF\_TRICISTRONIC\_RRNA\_TRANSCRIPT\_SSU\_RRNA\_5\_8S\_RRNA\_LSU\_RRNA |  | 5 | -0.35 | -0.67 | 0.892 | 0.982 | 1.000 | 1700 | tags=20%, list=14%, signal=23% |
| 7246 | GOMF\_FAD\_BINDING |  | 28 | -0.22 | -0.67 | 0.911 | 0.982 | 1.000 | 2869 | tags=25%, list=24%, signal=33% |
| 7247 | GOMF\_HISTONE\_METHYLTRANSFERASE\_ACTIVITY\_H4\_K20\_SPECIFIC |  | 4 | -0.37 | -0.67 | 0.905 | 0.982 | 1.000 | 3200 | tags=50%, list=27%, signal=68% |
| 7248 | GOBP\_INTEGRIN\_ACTIVATION |  | 20 | -0.24 | -0.67 | 0.901 | 0.983 | 1.000 | 4172 | tags=35%, list=35%, signal=54% |
| 7249 | GOBP\_PHOTORECEPTOR\_CELL\_DIFFERENTIATION |  | 31 | -0.22 | -0.67 | 0.912 | 0.983 | 1.000 | 6012 | tags=55%, list=50%, signal=110% |
| 7250 | GOBP\_PYRIMIDINE\_CONTAINING\_COMPOUND\_TRANSMEMBRANE\_TRANSPORT |  | 7 | -0.32 | -0.67 | 0.889 | 0.982 | 1.000 | 3277 | tags=29%, list=27%, signal=39% |
| 7251 | GOCC\_TRANS\_GOLGI\_NETWORK\_MEMBRANE |  | 66 | -0.19 | -0.67 | 0.946 | 0.982 | 1.000 | 4718 | tags=38%, list=39%, signal=62% |
| 7252 | GOBP\_NEGATIVE\_REGULATION\_OF\_RESPONSE\_TO\_BIOTIC\_STIMULUS |  | 51 | -0.20 | -0.67 | 0.940 | 0.982 | 1.000 | 2483 | tags=24%, list=21%, signal=30% |
| 7253 | GOBP\_PYROPTOSIS |  | 5 | -0.35 | -0.67 | 0.901 | 0.982 | 1.000 | 679 | tags=20%, list=6%, signal=21% |
| 7254 | GOMF\_AMP\_BINDING |  | 10 | -0.29 | -0.67 | 0.887 | 0.983 | 1.000 | 5050 | tags=50%, list=42%, signal=86% |
| 7255 | GOMF\_CHAPERONE\_BINDING |  | 74 | -0.19 | -0.67 | 0.952 | 0.983 | 1.000 | 3519 | tags=26%, list=29%, signal=36% |
| 7256 | GOBP\_MITOCHONDRION\_MORPHOGENESIS |  | 15 | -0.26 | -0.67 | 0.890 | 0.983 | 1.000 | 5164 | tags=47%, list=43%, signal=82% |
| 7257 | GOBP\_CELLULAR\_RESPONSE\_TO\_ETHER |  | 5 | -0.35 | -0.67 | 0.908 | 0.983 | 1.000 | 3968 | tags=60%, list=33%, signal=90% |
| 7258 | GOBP\_REGULATION\_OF\_EARLY\_ENDOSOME\_TO\_LATE\_ENDOSOME\_TRANSPORT |  | 15 | -0.26 | -0.67 | 0.898 | 0.983 | 1.000 | 6824 | tags=60%, list=57%, signal=139% |
| 7259 | GOBP\_POSITIVE\_REGULATION\_OF\_CYTOPLASMIC\_TRANSPORT |  | 9 | -0.29 | -0.67 | 0.886 | 0.983 | 1.000 | 2303 | tags=22%, list=19%, signal=27% |
| 7260 | GOBP\_NEGATIVE\_REGULATION\_OF\_RUFFLE\_ASSEMBLY |  | 5 | -0.34 | -0.67 | 0.898 | 0.983 | 1.000 | 5674 | tags=40%, list=47%, signal=76% |
| 7261 | GOBP\_EXTRACELLULAR\_MATRIX\_CONSTITUENT\_SECRETION |  | 9 | -0.29 | -0.67 | 0.885 | 0.983 | 1.000 | 1632 | tags=22%, list=14%, signal=26% |
| 7262 | GOBP\_SYNAPSE\_PRUNING |  | 9 | -0.29 | -0.67 | 0.886 | 0.983 | 1.000 | 5123 | tags=44%, list=43%, signal=77% |
| 7263 | GOBP\_POSITIVE\_REGULATION\_OF\_POTASSIUM\_ION\_TRANSPORT |  | 27 | -0.22 | -0.67 | 0.911 | 0.983 | 1.000 | 2955 | tags=30%, list=25%, signal=39% |
| 7264 | GOMF\_SNAP\_RECEPTOR\_ACTIVITY |  | 29 | -0.22 | -0.67 | 0.913 | 0.983 | 1.000 | 3698 | tags=31%, list=31%, signal=45% |
| 7265 | GOMF\_AZOLE\_TRANSMEMBRANE\_TRANSPORTER\_ACTIVITY |  | 7 | -0.31 | -0.67 | 0.891 | 0.983 | 1.000 | 3277 | tags=29%, list=27%, signal=39% |
| 7266 | GOBP\_UBIQUITIN\_INDEPENDENT\_PROTEIN\_CATABOLIC\_PROCESS\_VIA\_THE\_MULTIVESICULAR\_BODY\_SORTING\_PATHWAY |  | 5 | -0.34 | -0.67 | 0.899 | 0.983 | 1.000 | 4716 | tags=60%, list=39%, signal=99% |
| 7267 | GOMF\_PEROXISOME\_PROLIFERATOR\_ACTIVATED\_RECEPTOR\_BINDING |  | 9 | -0.29 | -0.67 | 0.900 | 0.983 | 1.000 | 124 | tags=11%, list=1%, signal=11% |
| 7268 | GOBP\_HYPOXIA\_INDUCIBLE\_FACTOR\_1ALPHA\_SIGNALING\_PATHWAY |  | 5 | -0.34 | -0.67 | 0.903 | 0.983 | 1.000 | 5226 | tags=60%, list=44%, signal=106% |
| 7269 | GOBP\_NEGATIVE\_REGULATION\_OF\_NITRIC\_OXIDE\_SYNTHASE\_ACTIVITY |  | 4 | -0.37 | -0.67 | 0.914 | 0.983 | 1.000 | 5903 | tags=75%, list=49%, signal=147% |
| 7270 | GOBP\_POSITIVE\_REGULATION\_OF\_EMBRYONIC\_DEVELOPMENT |  | 11 | -0.27 | -0.66 | 0.889 | 0.983 | 1.000 | 4904 | tags=45%, list=41%, signal=77% |
| 7271 | GOCC\_MULTIVESICULAR\_BODY\_MEMBRANE |  | 10 | -0.29 | -0.66 | 0.898 | 0.983 | 1.000 | 5224 | tags=60%, list=43%, signal=106% |
| 7272 | GOBP\_BLOOD\_VESSEL\_MATURATION |  | 7 | -0.31 | -0.66 | 0.885 | 0.982 | 1.000 | 6049 | tags=71%, list=50%, signal=144% |
| 7273 | GOBP\_MAINTENANCE\_OF\_SYNAPSE\_STRUCTURE |  | 11 | -0.28 | -0.66 | 0.888 | 0.982 | 1.000 | 4455 | tags=55%, list=37%, signal=87% |
| 7274 | GOBP\_TRABECULA\_FORMATION |  | 15 | -0.26 | -0.66 | 0.909 | 0.982 | 1.000 | 3314 | tags=27%, list=28%, signal=37% |
| 7275 | GOMF\_ANAPHASE\_PROMOTING\_COMPLEX\_BINDING |  | 7 | -0.32 | -0.66 | 0.896 | 0.982 | 1.000 | 3392 | tags=29%, list=28%, signal=40% |
| 7276 | GOBP\_ADENYLATE\_CYCLASE\_ACTIVATING\_DOPAMINE\_RECEPTOR\_SIGNALING\_PATHWAY |  | 7 | -0.31 | -0.66 | 0.896 | 0.982 | 1.000 | 336 | tags=14%, list=3%, signal=15% |
| 7277 | GOBP\_NEGATIVE\_REGULATION\_OF\_NEUTROPHIL\_MIGRATION |  | 3 | -0.40 | -0.66 | 0.930 | 0.982 | 1.000 | 1944 | tags=33%, list=16%, signal=40% |
| 7278 | GOBP\_RESPONSE\_TO\_SALT\_STRESS |  | 12 | -0.27 | -0.66 | 0.890 | 0.982 | 1.000 | 4353 | tags=42%, list=36%, signal=65% |
| 7279 | GOBP\_PROTEIN\_K48\_LINKED\_DEUBIQUITINATION |  | 22 | -0.24 | -0.66 | 0.915 | 0.982 | 1.000 | 5372 | tags=50%, list=45%, signal=90% |
| 7280 | GOBP\_COENZYME\_A\_METABOLIC\_PROCESS |  | 14 | -0.26 | -0.66 | 0.893 | 0.982 | 1.000 | 104 | tags=7%, list=1%, signal=7% |
| 7281 | GOBP\_METHIONINE\_METABOLIC\_PROCESS |  | 14 | -0.26 | -0.66 | 0.889 | 0.982 | 1.000 | 2580 | tags=29%, list=21%, signal=36% |
| 7282 | GOBP\_REGULATION\_OF\_PROTEIN\_POLYUBIQUITINATION |  | 13 | -0.26 | -0.66 | 0.893 | 0.982 | 1.000 | 2731 | tags=31%, list=23%, signal=40% |
| 7283 | GOBP\_GTP\_METABOLIC\_PROCESS |  | 18 | -0.24 | -0.66 | 0.897 | 0.982 | 1.000 | 3917 | tags=39%, list=33%, signal=58% |
| 7284 | GOBP\_POSITIVE\_REGULATION\_OF\_RENAL\_SODIUM\_EXCRETION |  | 8 | -0.30 | -0.66 | 0.890 | 0.982 | 1.000 | 322 | tags=13%, list=3%, signal=13% |
| 7285 | GOMF\_FIBRINOGEN\_BINDING |  | 5 | -0.34 | -0.66 | 0.898 | 0.982 | 1.000 | 6643 | tags=80%, list=55%, signal=179% |
| 7286 | GOBP\_RESPONSE\_TO\_PROGESTERONE |  | 29 | -0.22 | -0.66 | 0.916 | 0.982 | 1.000 | 4215 | tags=38%, list=35%, signal=58% |
| 7287 | GOBP\_CYTOPLASMIC\_TRANSLATIONAL\_INITIATION |  | 27 | -0.22 | -0.66 | 0.915 | 0.982 | 1.000 | 2370 | tags=11%, list=20%, signal=14% |
| 7288 | GOBP\_PEROXISOMAL\_TRANSPORT |  | 63 | -0.19 | -0.66 | 0.951 | 0.982 | 1.000 | 4383 | tags=32%, list=36%, signal=50% |
| 7289 | GOBP\_CELLULAR\_HYPEROSMOTIC\_RESPONSE |  | 11 | -0.28 | -0.66 | 0.894 | 0.982 | 1.000 | 4353 | tags=55%, list=36%, signal=85% |
| 7290 | GOBP\_REGULATION\_OF\_RECEPTOR\_CATABOLIC\_PROCESS |  | 9 | -0.29 | -0.66 | 0.885 | 0.982 | 1.000 | 2894 | tags=33%, list=24%, signal=44% |
| 7291 | GOBP\_REGULATION\_OF\_VOLTAGE\_GATED\_CALCIUM\_CHANNEL\_ACTIVITY |  | 23 | -0.23 | -0.66 | 0.909 | 0.982 | 1.000 | 276 | tags=4%, list=2%, signal=4% |
| 7292 | GOBP\_RESPONSE\_TO\_REDOX\_STATE |  | 12 | -0.27 | -0.66 | 0.896 | 0.982 | 1.000 | 4433 | tags=33%, list=37%, signal=53% |
| 7293 | GOMF\_ABC\_TYPE\_GLUTATHIONE\_S\_CONJUGATE\_TRANSPORTER\_ACTIVITY |  | 5 | -0.34 | -0.66 | 0.897 | 0.982 | 1.000 | 2842 | tags=60%, list=24%, signal=79% |
| 7294 | GOCC\_TRNA\_SPLICING\_LIGASE\_COMPLEX |  | 5 | -0.34 | -0.66 | 0.901 | 0.982 | 1.000 | 4245 | tags=40%, list=35%, signal=62% |
| 7295 | GOMF\_ION\_CHANNEL\_REGULATOR\_ACTIVITY |  | 71 | -0.19 | -0.66 | 0.957 | 0.982 | 1.000 | 2604 | tags=20%, list=22%, signal=25% |
| 7296 | GOCC\_SARCOLEMMA |  | 90 | -0.18 | -0.66 | 0.965 | 0.982 | 1.000 | 3104 | tags=23%, list=26%, signal=31% |
| 7297 | GOCC\_T\_TUBULE |  | 32 | -0.22 | -0.66 | 0.916 | 0.982 | 1.000 | 3566 | tags=31%, list=30%, signal=44% |
| 7298 | GOBP\_REGULATION\_OF\_ENDOTHELIAL\_CELL\_DEVELOPMENT |  | 15 | -0.25 | -0.66 | 0.903 | 0.982 | 1.000 | 3547 | tags=33%, list=30%, signal=47% |
| 7299 | GOBP\_ESTABLISHMENT\_OF\_PROTEIN\_LOCALIZATION\_TO\_ORGANELLE |  | 441 | -0.17 | -0.66 | 0.999 | 0.982 | 1.000 | 4383 | tags=28%, list=36%, signal=42% |
| 7300 | GOBP\_NEGATIVE\_REGULATION\_OF\_TRANSCRIPTION\_INITIATION\_FROM\_RNA\_POLYMERASE\_II\_PROMOTER |  | 5 | -0.34 | -0.66 | 0.897 | 0.982 | 1.000 | 4649 | tags=60%, list=39%, signal=98% |
| 7301 | GOBP\_REGULATION\_OF\_PROTEIN\_NEDDYLATION |  | 10 | -0.28 | -0.66 | 0.896 | 0.982 | 1.000 | 2411 | tags=20%, list=20%, signal=25% |
| 7302 | GOMF\_GTPASE\_ACTIVITY |  | 227 | -0.17 | -0.66 | 0.992 | 0.982 | 1.000 | 4850 | tags=32%, list=40%, signal=53% |
| 7303 | GOBP\_5\_PHOSPHORIBOSE\_1\_DIPHOSPHATE\_METABOLIC\_PROCESS |  | 5 | -0.35 | -0.66 | 0.898 | 0.982 | 1.000 | 4231 | tags=60%, list=35%, signal=93% |
| 7304 | GOBP\_REGULATION\_OF\_RUFFLE\_ASSEMBLY |  | 18 | -0.24 | -0.66 | 0.909 | 0.982 | 1.000 | 4425 | tags=39%, list=37%, signal=61% |
| 7305 | GOBP\_DRUG\_TRANSPORT |  | 10 | -0.28 | -0.66 | 0.900 | 0.983 | 1.000 | 4338 | tags=60%, list=36%, signal=94% |
| 7306 | GOBP\_NEGATIVE\_REGULATION\_OF\_PROTEIN\_AUTOPHOSPHORYLATION |  | 10 | -0.28 | -0.66 | 0.897 | 0.983 | 1.000 | 3574 | tags=30%, list=30%, signal=43% |
| 7307 | GOBP\_SULFATION |  | 12 | -0.27 | -0.66 | 0.900 | 0.983 | 1.000 | 1000 | tags=17%, list=8%, signal=18% |
| 7308 | GOMF\_PRE\_MRNA\_BINDING |  | 27 | -0.22 | -0.66 | 0.925 | 0.983 | 1.000 | 6228 | tags=56%, list=52%, signal=115% |
| 7309 | GOBP\_REGULATION\_OF\_KIDNEY\_DEVELOPMENT |  | 19 | -0.24 | -0.66 | 0.907 | 0.983 | 1.000 | 2356 | tags=21%, list=20%, signal=26% |
| 7310 | GOBP\_FUCOSE\_METABOLIC\_PROCESS |  | 11 | -0.27 | -0.66 | 0.897 | 0.983 | 1.000 | 4291 | tags=64%, list=36%, signal=99% |
| 7311 | GOBP\_PROTEIN\_LOCALIZATION\_TO\_CILIUM |  | 43 | -0.20 | -0.66 | 0.938 | 0.983 | 1.000 | 4760 | tags=37%, list=40%, signal=61% |
| 7312 | GOBP\_NEPHRON\_MORPHOGENESIS |  | 46 | -0.20 | -0.66 | 0.940 | 0.983 | 1.000 | 1100 | tags=13%, list=9%, signal=14% |
| 7313 | GOBP\_DENDRITIC\_TRANSPORT |  | 7 | -0.31 | -0.66 | 0.902 | 0.983 | 1.000 | 3555 | tags=57%, list=30%, signal=81% |
| 7314 | GOBP\_POSITIVE\_REGULATION\_OF\_IMMUNOGLOBULIN\_PRODUCTION |  | 23 | -0.23 | -0.66 | 0.914 | 0.983 | 1.000 | 3205 | tags=30%, list=27%, signal=41% |
| 7315 | GOBP\_NUCLEOSIDE\_METABOLIC\_PROCESS |  | 77 | -0.19 | -0.66 | 0.960 | 0.983 | 1.000 | 4363 | tags=32%, list=36%, signal=51% |
| 7316 | GOBP\_REGULATION\_OF\_ATRIAL\_CARDIAC\_MUSCLE\_CELL\_MEMBRANE\_DEPOLARIZATION |  | 4 | -0.36 | -0.66 | 0.921 | 0.982 | 1.000 | 3295 | tags=50%, list=27%, signal=69% |
| 7317 | GOBP\_REGULATION\_OF\_MYOBLAST\_FUSION |  | 8 | -0.30 | -0.66 | 0.906 | 0.983 | 1.000 | 3510 | tags=50%, list=29%, signal=71% |
| 7318 | GOBP\_POSITIVE\_REGULATION\_OF\_STEROID\_METABOLIC\_PROCESS |  | 15 | -0.25 | -0.66 | 0.903 | 0.983 | 1.000 | 4271 | tags=47%, list=36%, signal=72% |
| 7319 | GOMF\_ENDORIBONUCLEASE\_ACTIVITY\_PRODUCING\_5\_PHOSPHOMONOESTERS |  | 17 | -0.24 | -0.66 | 0.907 | 0.983 | 1.000 | 585 | tags=12%, list=5%, signal=12% |
| 7320 | GOBP\_AMINO\_ACID\_IMPORT |  | 34 | -0.21 | -0.66 | 0.929 | 0.983 | 1.000 | 3155 | tags=29%, list=26%, signal=40% |
| 7321 | GOBP\_TRANSCRIPTION\_COUPLED\_NUCLEOTIDE\_EXCISION\_REPAIR |  | 62 | -0.19 | -0.66 | 0.952 | 0.983 | 1.000 | 6021 | tags=50%, list=50%, signal=100% |
| 7322 | GOBP\_NUCLEOSIDE\_SALVAGE |  | 15 | -0.25 | -0.66 | 0.900 | 0.982 | 1.000 | 5029 | tags=53%, list=42%, signal=92% |
| 7323 | GOBP\_HEART\_FIELD\_SPECIFICATION |  | 13 | -0.26 | -0.66 | 0.898 | 0.982 | 1.000 | 2474 | tags=31%, list=21%, signal=39% |
| 7324 | GOBP\_EPITHELIAL\_CILIUM\_MOVEMENT\_INVOLVED\_IN\_DETERMINATION\_OF\_LEFT\_RIGHT\_ASYMMETRY |  | 4 | -0.36 | -0.66 | 0.916 | 0.982 | 1.000 | 1347 | tags=25%, list=11%, signal=28% |
| 7325 | GOBP\_NEGATIVE\_REGULATION\_OF\_INTERFERON\_BETA\_PRODUCTION |  | 9 | -0.29 | -0.66 | 0.899 | 0.982 | 1.000 | 4549 | tags=44%, list=38%, signal=71% |
| 7326 | GOBP\_REGULATION\_OF\_ANGIOTENSIN\_LEVELS\_IN\_BLOOD |  | 8 | -0.29 | -0.65 | 0.903 | 0.983 | 1.000 | 2133 | tags=38%, list=18%, signal=46% |
| 7327 | GOBP\_MRNA\_METABOLIC\_PROCESS |  | 647 | -0.16 | -0.65 | 1.000 | 0.983 | 1.000 | 4968 | tags=32%, list=41%, signal=52% |
| 7328 | GOBP\_REGULATION\_OF\_UBIQUITIN\_PROTEIN\_LIGASE\_ACTIVITY |  | 20 | -0.23 | -0.65 | 0.913 | 0.984 | 1.000 | 5705 | tags=45%, list=47%, signal=86% |
| 7329 | GOMF\_NUCLEOBASE\_CONTAINING\_COMPOUND\_TRANSMEMBRANE\_TRANSPORTER\_ACTIVITY |  | 32 | -0.21 | -0.65 | 0.926 | 0.984 | 1.000 | 3427 | tags=28%, list=29%, signal=39% |
| 7330 | GOCC\_NATA\_COMPLEX |  | 3 | -0.39 | -0.65 | 0.924 | 0.984 | 1.000 | 7290 | tags=100%, list=61%, signal=254% |
| 7331 | GOCC\_ENDOPLASMIC\_RETICULUM\_CHAPERONE\_COMPLEX |  | 6 | -0.32 | -0.65 | 0.901 | 0.984 | 1.000 | 5182 | tags=67%, list=43%, signal=117% |
| 7332 | GOBP\_HOMEOSTASIS\_OF\_NUMBER\_OF\_CELLS\_WITHIN\_A\_TISSUE |  | 19 | -0.24 | -0.65 | 0.909 | 0.984 | 1.000 | 6023 | tags=58%, list=50%, signal=116% |
| 7333 | GOBP\_MATING\_BEHAVIOR |  | 13 | -0.26 | -0.65 | 0.904 | 0.984 | 1.000 | 4521 | tags=46%, list=38%, signal=74% |
| 7334 | GOBP\_CELLULAR\_RESPONSE\_TO\_GONADOTROPIN\_STIMULUS |  | 8 | -0.30 | -0.65 | 0.899 | 0.984 | 1.000 | 3833 | tags=50%, list=32%, signal=73% |
| 7335 | GOBP\_REGULATION\_OF\_ISOTYPE\_SWITCHING |  | 23 | -0.23 | -0.65 | 0.921 | 0.984 | 1.000 | 3200 | tags=30%, list=27%, signal=41% |
| 7336 | GOBP\_CELLULAR\_RESPONSE\_TO\_HYPEROXIA |  | 7 | -0.31 | -0.65 | 0.900 | 0.984 | 1.000 | 162 | tags=14%, list=1%, signal=14% |
| 7337 | GOBP\_REGULATION\_OF\_SYNCYTIUM\_FORMATION\_BY\_PLASMA\_MEMBRANE\_FUSION |  | 13 | -0.26 | -0.65 | 0.903 | 0.984 | 1.000 | 2494 | tags=31%, list=21%, signal=39% |
| 7338 | GOBP\_NEGATIVE\_REGULATION\_OF\_RECEPTOR\_INTERNALIZATION |  | 9 | -0.29 | -0.65 | 0.905 | 0.984 | 1.000 | 251 | tags=11%, list=2%, signal=11% |
| 7339 | GOBP\_LYSINE\_METABOLIC\_PROCESS |  | 13 | -0.26 | -0.65 | 0.904 | 0.984 | 1.000 | 298 | tags=8%, list=2%, signal=8% |
| 7340 | GOBP\_METANEPHRIC\_TUBULE\_DEVELOPMENT |  | 13 | -0.26 | -0.65 | 0.902 | 0.984 | 1.000 | 4271 | tags=54%, list=36%, signal=83% |
| 7341 | GOBP\_ESTABLISHMENT\_OF\_PROTEIN\_LOCALIZATION\_TO\_MEMBRANE |  | 259 | -0.17 | -0.65 | 0.996 | 0.984 | 1.000 | 1285 | tags=8%, list=11%, signal=9% |
| 7342 | GOBP\_PROTEIN\_TRANSPORT\_WITHIN\_LIPID\_BILAYER |  | 6 | -0.32 | -0.65 | 0.908 | 0.984 | 1.000 | 455 | tags=17%, list=4%, signal=17% |
| 7343 | GOBP\_TRANSCRIPTION\_ELONGATION\_FROM\_RNA\_POLYMERASE\_I\_PROMOTER |  | 23 | -0.23 | -0.65 | 0.927 | 0.984 | 1.000 | 5069 | tags=43%, list=42%, signal=75% |
| 7344 | GOCC\_SET1C\_COMPASS\_COMPLEX |  | 6 | -0.32 | -0.65 | 0.907 | 0.984 | 1.000 | 2041 | tags=17%, list=17%, signal=20% |
| 7345 | GOBP\_POSITIVE\_REGULATION\_OF\_NEURON\_APOPTOTIC\_PROCESS |  | 37 | -0.20 | -0.65 | 0.937 | 0.984 | 1.000 | 2181 | tags=19%, list=18%, signal=23% |
| 7346 | GOBP\_REGULATION\_OF\_MITOCHONDRIAL\_FUSION |  | 11 | -0.27 | -0.65 | 0.904 | 0.984 | 1.000 | 4673 | tags=55%, list=39%, signal=89% |
| 7347 | GOBP\_NEGATIVE\_REGULATION\_OF\_COLD\_INDUCED\_THERMOGENESIS |  | 37 | -0.21 | -0.65 | 0.934 | 0.984 | 1.000 | 4526 | tags=35%, list=38%, signal=56% |
| 7348 | GOBP\_DICARBOXYLIC\_ACID\_METABOLIC\_PROCESS |  | 68 | -0.19 | -0.65 | 0.963 | 0.984 | 1.000 | 3008 | tags=22%, list=25%, signal=29% |
| 7349 | GOBP\_CEREBELLAR\_PURKINJE\_CELL\_GRANULE\_CELL\_PRECURSOR\_CELL\_SIGNALING\_INVOLVED\_IN\_REGULATION\_OF\_GRANULE\_CELL\_PRECURSOR\_CELL\_PROLIFERATION |  | 5 | -0.33 | -0.65 | 0.903 | 0.984 | 1.000 | 5085 | tags=40%, list=42%, signal=69% |
| 7350 | GOMF\_GENERAL\_TRANSCRIPTION\_INITIATION\_FACTOR\_BINDING |  | 33 | -0.21 | -0.65 | 0.937 | 0.984 | 1.000 | 1743 | tags=12%, list=15%, signal=14% |
| 7351 | GOCC\_INTRINSIC\_COMPONENT\_OF\_ENDOPLASMIC\_RETICULUM\_MEMBRANE |  | 110 | -0.18 | -0.65 | 0.977 | 0.984 | 1.000 | 4890 | tags=38%, list=41%, signal=64% |
| 7352 | GOBP\_ANTIMICROBIAL\_HUMORAL\_RESPONSE |  | 28 | -0.22 | -0.65 | 0.924 | 0.984 | 1.000 | 3009 | tags=25%, list=25%, signal=33% |
| 7353 | GOBP\_SMOOTHENED\_SIGNALING\_PATHWAY\_INVOLVED\_IN\_REGULATION\_OF\_CEREBELLAR\_GRANULE\_CELL\_PRECURSOR\_CELL\_PROLIFERATION |  | 5 | -0.33 | -0.65 | 0.914 | 0.984 | 1.000 | 5085 | tags=40%, list=42%, signal=69% |
| 7354 | GOBP\_REGULATION\_OF\_LONG\_CHAIN\_FATTY\_ACID\_IMPORT\_INTO\_CELL |  | 6 | -0.32 | -0.65 | 0.910 | 0.984 | 1.000 | 5145 | tags=67%, list=43%, signal=117% |
| 7355 | GOBP\_VENTRICULAR\_CARDIAC\_MUSCLE\_CELL\_ACTION\_POTENTIAL |  | 24 | -0.22 | -0.65 | 0.923 | 0.984 | 1.000 | 3918 | tags=38%, list=33%, signal=56% |
| 7356 | GOBP\_EXTRACELLULAR\_MATRIX\_ASSEMBLY |  | 34 | -0.21 | -0.65 | 0.933 | 0.984 | 1.000 | 5967 | tags=56%, list=50%, signal=111% |
| 7357 | GOBP\_REGULATION\_OF\_ARACHIDONIC\_ACID\_SECRETION |  | 3 | -0.39 | -0.65 | 0.933 | 0.984 | 1.000 | 2695 | tags=67%, list=22%, signal=86% |
| 7358 | GOBP\_GLUTAMINE\_FAMILY\_AMINO\_ACID\_METABOLIC\_PROCESS |  | 47 | -0.20 | -0.65 | 0.945 | 0.984 | 1.000 | 3653 | tags=28%, list=30%, signal=40% |
| 7359 | GOBP\_NEGATIVE\_REGULATION\_OF\_MULTICELLULAR\_ORGANISM\_GROWTH |  | 7 | -0.30 | -0.65 | 0.916 | 0.984 | 1.000 | 2670 | tags=43%, list=22%, signal=55% |
| 7360 | GOBP\_REGULATION\_OF\_PHOSPHATIDYLCHOLINE\_BIOSYNTHETIC\_PROCESS |  | 4 | -0.36 | -0.65 | 0.927 | 0.984 | 1.000 | 1765 | tags=25%, list=15%, signal=29% |
| 7361 | GOBP\_HYALURONAN\_BIOSYNTHETIC\_PROCESS |  | 10 | -0.28 | -0.65 | 0.904 | 0.984 | 1.000 | 4433 | tags=50%, list=37%, signal=79% |
| 7362 | GOBP\_VIRAL\_LATENCY |  | 10 | -0.28 | -0.65 | 0.912 | 0.984 | 1.000 | 3927 | tags=60%, list=33%, signal=89% |
| 7363 | GOBP\_PYRIMIDINE\_NUCLEOBASE\_BIOSYNTHETIC\_PROCESS |  | 8 | -0.29 | -0.65 | 0.912 | 0.984 | 1.000 | 1743 | tags=13%, list=15%, signal=15% |
| 7364 | GOBP\_NEGATIVE\_REGULATION\_OF\_VOLTAGE\_GATED\_POTASSIUM\_CHANNEL\_ACTIVITY |  | 7 | -0.30 | -0.65 | 0.913 | 0.984 | 1.000 | 1125 | tags=14%, list=9%, signal=16% |
| 7365 | GOCC\_AXONEMAL\_DYNEIN\_COMPLEX |  | 5 | -0.33 | -0.65 | 0.919 | 0.984 | 1.000 | 2990 | tags=40%, list=25%, signal=53% |
| 7366 | GOBP\_NEGATIVE\_REGULATION\_OF\_MACROPHAGE\_MIGRATION |  | 7 | -0.30 | -0.65 | 0.913 | 0.984 | 1.000 | 249 | tags=14%, list=2%, signal=15% |
| 7367 | GOCC\_CTF18\_RFC\_LIKE\_COMPLEX |  | 8 | -0.29 | -0.64 | 0.909 | 0.984 | 1.000 | 1030 | tags=13%, list=9%, signal=14% |
| 7368 | GOBP\_NEGATIVE\_REGULATION\_OF\_SECRETION |  | 92 | -0.18 | -0.64 | 0.974 | 0.984 | 1.000 | 2367 | tags=17%, list=20%, signal=21% |
| 7369 | GOBP\_NUCLEOTIDE\_EXCISION\_REPAIR\_DNA\_GAP\_FILLING |  | 19 | -0.24 | -0.64 | 0.921 | 0.985 | 1.000 | 5351 | tags=42%, list=45%, signal=76% |
| 7370 | GOMF\_PHOSPHATASE\_ACTIVATOR\_ACTIVITY |  | 10 | -0.27 | -0.64 | 0.906 | 0.985 | 1.000 | 4627 | tags=50%, list=39%, signal=81% |
| 7371 | GOMF\_MRNA\_5\_UTR\_BINDING |  | 22 | -0.23 | -0.64 | 0.924 | 0.985 | 1.000 | 5307 | tags=41%, list=44%, signal=73% |
| 7372 | GOBP\_CELLULAR\_RESPONSE\_TO\_HYDROGEN\_PEROXIDE |  | 68 | -0.19 | -0.64 | 0.964 | 0.986 | 1.000 | 4698 | tags=38%, list=39%, signal=62% |
| 7373 | GOBP\_POSITIVE\_REGULATION\_OF\_DNA\_LIGATION |  | 4 | -0.35 | -0.64 | 0.927 | 0.986 | 1.000 | 4649 | tags=75%, list=39%, signal=122% |
| 7374 | GOBP\_PROTEIN\_PROCESSING\_INVOLVED\_IN\_PROTEIN\_TARGETING\_TO\_MITOCHONDRION |  | 7 | -0.30 | -0.64 | 0.909 | 0.985 | 1.000 | 5302 | tags=43%, list=44%, signal=77% |
| 7375 | GOBP\_SECONDARY\_METABOLIC\_PROCESS |  | 28 | -0.22 | -0.64 | 0.934 | 0.986 | 1.000 | 337 | tags=7%, list=3%, signal=7% |
| 7376 | GOBP\_NEGATIVE\_REGULATION\_OF\_NUCLEOCYTOPLASMIC\_TRANSPORT |  | 18 | -0.24 | -0.64 | 0.915 | 0.985 | 1.000 | 2763 | tags=22%, list=23%, signal=29% |
| 7377 | GOBP\_SODIUM\_DEPENDENT\_PHOSPHATE\_TRANSPORT |  | 4 | -0.35 | -0.64 | 0.920 | 0.985 | 1.000 | 1302 | tags=25%, list=11%, signal=28% |
| 7378 | GOBP\_S\_ADENOSYLMETHIONINE\_CYCLE |  | 7 | -0.30 | -0.64 | 0.914 | 0.985 | 1.000 | 5312 | tags=57%, list=44%, signal=102% |
| 7379 | GOBP\_STEM\_CELL\_FATE\_COMMITMENT |  | 4 | -0.35 | -0.64 | 0.925 | 0.985 | 1.000 | 1579 | tags=25%, list=13%, signal=29% |
| 7380 | GOBP\_MELANOSOME\_ASSEMBLY |  | 4 | -0.35 | -0.64 | 0.925 | 0.986 | 1.000 | 2911 | tags=50%, list=24%, signal=66% |
| 7381 | GOBP\_PROTEIN\_LOCALIZATION\_TO\_NUCLEOLUS |  | 13 | -0.25 | -0.64 | 0.912 | 0.986 | 1.000 | 2848 | tags=23%, list=24%, signal=30% |
| 7382 | GOBP\_RNA\_CATABOLIC\_PROCESS |  | 319 | -0.16 | -0.64 | 0.997 | 0.986 | 1.000 | 4822 | tags=29%, list=40%, signal=48% |
| 7383 | GOBP\_SPHINGOMYELIN\_CATABOLIC\_PROCESS |  | 6 | -0.31 | -0.64 | 0.919 | 0.986 | 1.000 | 4433 | tags=50%, list=37%, signal=79% |
| 7384 | GOBP\_SEQUESTERING\_OF\_ACTIN\_MONOMERS |  | 6 | -0.32 | -0.64 | 0.914 | 0.986 | 1.000 | 1159 | tags=17%, list=10%, signal=18% |
| 7385 | GOBP\_REGULATION\_OF\_ADIPOSE\_TISSUE\_DEVELOPMENT |  | 4 | -0.35 | -0.64 | 0.928 | 0.986 | 1.000 | 3239 | tags=50%, list=27%, signal=68% |
| 7386 | GOMF\_POLYUBIQUITIN\_MODIFICATION\_DEPENDENT\_PROTEIN\_BINDING |  | 32 | -0.21 | -0.64 | 0.934 | 0.986 | 1.000 | 5334 | tags=53%, list=44%, signal=95% |
| 7387 | GOBP\_PLASMA\_MEMBRANE\_TUBULATION |  | 15 | -0.24 | -0.64 | 0.922 | 0.986 | 1.000 | 3798 | tags=33%, list=32%, signal=49% |
| 7388 | GOBP\_NEGATIVE\_REGULATION\_OF\_ACTION\_POTENTIAL |  | 5 | -0.33 | -0.64 | 0.925 | 0.986 | 1.000 | 1597 | tags=20%, list=13%, signal=23% |
| 7389 | GOBP\_REGULATION\_OF\_VASCULAR\_PERMEABILITY |  | 30 | -0.21 | -0.64 | 0.941 | 0.986 | 1.000 | 3824 | tags=37%, list=32%, signal=54% |
| 7390 | GOCC\_TELOMERASE\_HOLOENZYME\_COMPLEX |  | 14 | -0.25 | -0.64 | 0.915 | 0.986 | 1.000 | 5754 | tags=50%, list=48%, signal=96% |
| 7391 | GOBP\_REGULATION\_OF\_FERTILIZATION |  | 5 | -0.33 | -0.64 | 0.920 | 0.986 | 1.000 | 440 | tags=20%, list=4%, signal=21% |
| 7392 | GOBP\_NEGATIVE\_REGULATION\_OF\_ANION\_TRANSMEMBRANE\_TRANSPORT |  | 21 | -0.23 | -0.64 | 0.930 | 0.986 | 1.000 | 4139 | tags=48%, list=34%, signal=73% |
| 7393 | GOBP\_REGULATION\_OF\_TRANSLATIONAL\_INITIATION\_IN\_RESPONSE\_TO\_STRESS |  | 13 | -0.25 | -0.64 | 0.916 | 0.986 | 1.000 | 3872 | tags=38%, list=32%, signal=57% |
| 7394 | GOBP\_LATE\_ENDOSOME\_TO\_VACUOLE\_TRANSPORT |  | 21 | -0.22 | -0.64 | 0.926 | 0.987 | 1.000 | 1824 | tags=14%, list=15%, signal=17% |
| 7395 | GOBP\_REGULATION\_OF\_MICROGLIAL\_CELL\_ACTIVATION |  | 14 | -0.25 | -0.64 | 0.919 | 0.987 | 1.000 | 3695 | tags=43%, list=31%, signal=62% |
| 7396 | GOBP\_SPLICEOSOMAL\_SNRNP\_ASSEMBLY |  | 29 | -0.21 | -0.64 | 0.938 | 0.987 | 1.000 | 3996 | tags=24%, list=33%, signal=36% |
| 7397 | GOBP\_PEROXISOME\_ORGANIZATION |  | 71 | -0.18 | -0.64 | 0.965 | 0.987 | 1.000 | 5175 | tags=39%, list=43%, signal=69% |
| 7398 | GOBP\_REGULATION\_OF\_NEUROTROPHIN\_TRK\_RECEPTOR\_SIGNALING\_PATHWAY |  | 10 | -0.27 | -0.63 | 0.914 | 0.987 | 1.000 | 3797 | tags=40%, list=32%, signal=58% |
| 7399 | GOMF\_RNA\_DIRECTED\_DNA\_POLYMERASE\_ACTIVITY |  | 5 | -0.33 | -0.63 | 0.921 | 0.987 | 1.000 | 3722 | tags=40%, list=31%, signal=58% |
| 7400 | GOCC\_CUL2\_RING\_UBIQUITIN\_LIGASE\_COMPLEX |  | 10 | -0.27 | -0.63 | 0.914 | 0.987 | 1.000 | 4582 | tags=50%, list=38%, signal=81% |
| 7401 | GOMF\_PHOSPHATIDYLGLYCEROL\_BINDING |  | 10 | -0.27 | -0.63 | 0.913 | 0.987 | 1.000 | 3525 | tags=40%, list=29%, signal=57% |
| 7402 | GOMF\_MECHANOSENSITIVE\_ION\_CHANNEL\_ACTIVITY |  | 11 | -0.27 | -0.63 | 0.917 | 0.987 | 1.000 | 3160 | tags=36%, list=26%, signal=49% |
| 7403 | GOBP\_POSITIVE\_REGULATION\_OF\_KILLING\_OF\_CELLS\_OF\_OTHER\_ORGANISM |  | 5 | -0.33 | -0.63 | 0.924 | 0.987 | 1.000 | 3966 | tags=60%, list=33%, signal=90% |
| 7404 | GOBP\_MITOCHONDRIAL\_PROTEIN\_PROCESSING |  | 13 | -0.25 | -0.63 | 0.912 | 0.986 | 1.000 | 6502 | tags=54%, list=54%, signal=117% |
| 7405 | GOMF\_ABC\_TYPE\_TRANSPORTER\_ACTIVITY |  | 18 | -0.23 | -0.63 | 0.927 | 0.986 | 1.000 | 3318 | tags=39%, list=28%, signal=54% |
| 7406 | GOBP\_METANEPHRIC\_EPITHELIUM\_DEVELOPMENT |  | 15 | -0.24 | -0.63 | 0.920 | 0.986 | 1.000 | 4271 | tags=47%, list=36%, signal=72% |
| 7407 | GOCC\_GLYCOPROTEIN\_COMPLEX |  | 13 | -0.25 | -0.63 | 0.919 | 0.986 | 1.000 | 3104 | tags=23%, list=26%, signal=31% |
| 7408 | GOBP\_CALCIUM\_DEPENDENT\_CELL\_CELL\_ADHESION\_VIA\_PLASMA\_MEMBRANE\_CELL\_ADHESION\_MOLECULES |  | 15 | -0.24 | -0.63 | 0.918 | 0.986 | 1.000 | 4304 | tags=40%, list=36%, signal=62% |
| 7409 | GOBP\_REGULATION\_OF\_HEAT\_GENERATION |  | 12 | -0.26 | -0.63 | 0.916 | 0.987 | 1.000 | 1408 | tags=17%, list=12%, signal=19% |
| 7410 | GOMF\_U2\_SNRNA\_BINDING |  | 4 | -0.35 | -0.63 | 0.931 | 0.987 | 1.000 | 3604 | tags=50%, list=30%, signal=71% |
| 7411 | GOBP\_REGULATION\_OF\_CALCINEURIN\_MEDIATED\_SIGNALING |  | 19 | -0.23 | -0.63 | 0.934 | 0.988 | 1.000 | 3641 | tags=42%, list=30%, signal=60% |
| 7412 | GOMF\_PHOSPHATIDYLINOSITOL\_BISPHOSPHATE\_BINDING |  | 68 | -0.18 | -0.63 | 0.972 | 0.988 | 1.000 | 5260 | tags=43%, list=44%, signal=75% |
| 7413 | GOBP\_NUCLEAR\_TRANSCRIBED\_MRNA\_CATABOLIC\_PROCESS\_EXONUCLEOLYTIC |  | 32 | -0.21 | -0.63 | 0.943 | 0.988 | 1.000 | 2080 | tags=16%, list=17%, signal=19% |
| 7414 | GOBP\_REGULATION\_OF\_CYTOPLASMIC\_TRANSPORT |  | 20 | -0.23 | -0.63 | 0.930 | 0.988 | 1.000 | 5937 | tags=45%, list=49%, signal=89% |
| 7415 | GOBP\_REGULATION\_OF\_VENTRICULAR\_CARDIAC\_MUSCLE\_CELL\_MEMBRANE\_DEPOLARIZATION |  | 4 | -0.35 | -0.63 | 0.936 | 0.988 | 1.000 | 3295 | tags=50%, list=27%, signal=69% |
| 7416 | GOMF\_VITAMIN\_B6\_BINDING |  | 37 | -0.20 | -0.63 | 0.950 | 0.988 | 1.000 | 2851 | tags=24%, list=24%, signal=32% |
| 7417 | GOMF\_TELOMERASE\_ACTIVITY |  | 5 | -0.33 | -0.63 | 0.923 | 0.988 | 1.000 | 3722 | tags=40%, list=31%, signal=58% |
| 7418 | GOBP\_DNA\_METHYLATION\_ON\_CYTOSINE |  | 3 | -0.38 | -0.63 | 0.944 | 0.988 | 1.000 | 3595 | tags=33%, list=30%, signal=48% |
| 7419 | GOBP\_GLYCOSYL\_COMPOUND\_METABOLIC\_PROCESS |  | 92 | -0.18 | -0.63 | 0.978 | 0.988 | 1.000 | 4363 | tags=32%, list=36%, signal=49% |
| 7420 | GOBP\_T\_HELPER\_17\_CELL\_LINEAGE\_COMMITMENT |  | 7 | -0.30 | -0.63 | 0.923 | 0.988 | 1.000 | 960 | tags=14%, list=8%, signal=16% |
| 7421 | GOBP\_REGULATION\_OF\_BRANCHING\_INVOLVED\_IN\_URETERIC\_BUD\_MORPHOGENESIS |  | 10 | -0.27 | -0.63 | 0.926 | 0.988 | 1.000 | 6702 | tags=60%, list=56%, signal=136% |
| 7422 | GOBP\_RNA\_SPLICING\_VIA\_TRANSESTERIFICATION\_REACTIONS |  | 264 | -0.16 | -0.63 | 0.998 | 0.988 | 1.000 | 6171 | tags=45%, list=51%, signal=91% |
| 7423 | GOBP\_CHROMOSOME\_LOCALIZATION\_TO\_NUCLEAR\_ENVELOPE\_INVOLVED\_IN\_HOMOLOGOUS\_CHROMOSOME\_SEGREGATION |  | 4 | -0.35 | -0.63 | 0.932 | 0.988 | 1.000 | 5365 | tags=75%, list=45%, signal=135% |
| 7424 | GOBP\_PROGESTERONE\_METABOLIC\_PROCESS |  | 6 | -0.31 | -0.63 | 0.920 | 0.988 | 1.000 | 2374 | tags=33%, list=20%, signal=42% |
| 7425 | GOBP\_POSITIVE\_REGULATION\_OF\_ATTACHMENT\_OF\_SPINDLE\_MICROTUBULES\_TO\_KINETOCHORE |  | 6 | -0.31 | -0.63 | 0.923 | 0.989 | 1.000 | 6407 | tags=67%, list=53%, signal=143% |
| 7426 | GOMF\_RNA\_POLYMERASE\_II\_GENERAL\_TRANSCRIPTION\_INITIATION\_FACTOR\_ACTIVITY |  | 29 | -0.21 | -0.63 | 0.946 | 0.989 | 1.000 | 3287 | tags=21%, list=27%, signal=28% |
| 7427 | GOBP\_PEPTIDYL\_METHIONINE\_MODIFICATION |  | 11 | -0.26 | -0.63 | 0.924 | 0.989 | 1.000 | 6773 | tags=82%, list=56%, signal=187% |
| 7428 | GOMF\_TITIN\_BINDING |  | 6 | -0.31 | -0.63 | 0.928 | 0.989 | 1.000 | 4627 | tags=67%, list=39%, signal=108% |
| 7429 | GOBP\_POSITIVE\_REGULATION\_OF\_MITOTIC\_NUCLEAR\_DIVISION |  | 33 | -0.21 | -0.63 | 0.953 | 0.989 | 1.000 | 1856 | tags=15%, list=15%, signal=18% |
| 7430 | GOBP\_REGULATION\_OF\_HEMATOPOIETIC\_PROGENITOR\_CELL\_DIFFERENTIATION |  | 70 | -0.18 | -0.62 | 0.975 | 0.989 | 1.000 | 3219 | tags=19%, list=27%, signal=25% |
| 7431 | GOBP\_POSITIVE\_REGULATION\_OF\_POTASSIUM\_ION\_TRANSMEMBRANE\_TRANSPORTER\_ACTIVITY |  | 12 | -0.25 | -0.62 | 0.930 | 0.989 | 1.000 | 2908 | tags=33%, list=24%, signal=44% |
| 7432 | GOCC\_PIGMENT\_GRANULE |  | 82 | -0.18 | -0.62 | 0.980 | 0.989 | 1.000 | 3390 | tags=24%, list=28%, signal=34% |
| 7433 | GOBP\_DIGESTIVE\_TRACT\_MORPHOGENESIS |  | 31 | -0.21 | -0.62 | 0.948 | 0.988 | 1.000 | 2002 | tags=16%, list=17%, signal=19% |
| 7434 | GOBP\_OUTER\_MITOCHONDRIAL\_MEMBRANE\_ORGANIZATION |  | 4 | -0.34 | -0.62 | 0.939 | 0.988 | 1.000 | 2010 | tags=25%, list=17%, signal=30% |
| 7435 | GOBP\_REGULATION\_OF\_GLYCOGEN\_STARCH\_SYNTHASE\_ACTIVITY |  | 4 | -0.35 | -0.62 | 0.939 | 0.989 | 1.000 | 3934 | tags=25%, list=33%, signal=37% |
| 7436 | GOBP\_REGULATION\_OF\_LIPID\_CATABOLIC\_PROCESS |  | 38 | -0.20 | -0.62 | 0.953 | 0.989 | 1.000 | 1939 | tags=18%, list=16%, signal=22% |
| 7437 | GOBP\_POSITIVE\_REGULATION\_OF\_CAMP\_MEDIATED\_SIGNALING |  | 6 | -0.31 | -0.62 | 0.925 | 0.989 | 1.000 | 1939 | tags=33%, list=16%, signal=40% |
| 7438 | GOBP\_VIRAL\_TRANSLATIONAL\_TERMINATION\_REINITIATION |  | 5 | -0.32 | -0.62 | 0.928 | 0.989 | 1.000 | 2370 | tags=20%, list=20%, signal=25% |
| 7439 | GOBP\_HYPEROSMOTIC\_SALINITY\_RESPONSE |  | 7 | -0.29 | -0.62 | 0.922 | 0.989 | 1.000 | 4353 | tags=57%, list=36%, signal=90% |
| 7440 | GOBP\_POSITIVE\_REGULATION\_OF\_EARLY\_ENDOSOME\_TO\_LATE\_ENDOSOME\_TRANSPORT |  | 7 | -0.29 | -0.62 | 0.927 | 0.989 | 1.000 | 6580 | tags=71%, list=55%, signal=158% |
| 7441 | GOBP\_METANEPHRIC\_GLOMERULUS\_VASCULATURE\_DEVELOPMENT |  | 7 | -0.29 | -0.62 | 0.930 | 0.989 | 1.000 | 3286 | tags=29%, list=27%, signal=39% |
| 7442 | GOBP\_SECONDARY\_METABOLITE\_BIOSYNTHETIC\_PROCESS |  | 11 | -0.26 | -0.62 | 0.922 | 0.989 | 1.000 | 3800 | tags=36%, list=32%, signal=53% |
| 7443 | GOBP\_CYTOKINETIC\_PROCESS |  | 34 | -0.20 | -0.62 | 0.955 | 0.989 | 1.000 | 1875 | tags=15%, list=16%, signal=17% |
| 7444 | GOBP\_TRANSCYTOSIS |  | 17 | -0.23 | -0.62 | 0.936 | 0.989 | 1.000 | 6342 | tags=59%, list=53%, signal=124% |
| 7445 | GOCC\_ESCRT\_COMPLEX |  | 24 | -0.21 | -0.62 | 0.940 | 0.989 | 1.000 | 2006 | tags=17%, list=17%, signal=20% |
| 7446 | GOBP\_CELLULAR\_RESPONSE\_TO\_NICOTINE |  | 3 | -0.37 | -0.62 | 0.945 | 0.989 | 1.000 | 2666 | tags=33%, list=22%, signal=43% |
| 7447 | GOBP\_POSITIVE\_REGULATION\_OF\_PENILE\_ERECTION |  | 4 | -0.34 | -0.62 | 0.945 | 0.990 | 1.000 | 4984 | tags=75%, list=41%, signal=128% |
| 7448 | GOBP\_CHAPERONE\_MEDIATED\_PROTEIN\_FOLDING |  | 46 | -0.19 | -0.62 | 0.966 | 0.990 | 1.000 | 4294 | tags=28%, list=36%, signal=44% |
| 7449 | GOMF\_DNA\_BINDING\_BENDING |  | 12 | -0.25 | -0.62 | 0.930 | 0.990 | 1.000 | 2771 | tags=33%, list=23%, signal=43% |
| 7450 | GOBP\_NEGATIVE\_REGULATION\_OF\_MONONUCLEAR\_CELL\_MIGRATION |  | 15 | -0.24 | -0.62 | 0.932 | 0.990 | 1.000 | 4894 | tags=40%, list=41%, signal=67% |
| 7451 | GOBP\_MESONEPHRIC\_DUCT\_DEVELOPMENT |  | 4 | -0.34 | -0.62 | 0.944 | 0.990 | 1.000 | 5330 | tags=75%, list=44%, signal=135% |
| 7452 | GOBP\_RENAL\_SYSTEM\_VASCULATURE\_DEVELOPMENT |  | 22 | -0.22 | -0.62 | 0.944 | 0.990 | 1.000 | 3286 | tags=23%, list=27%, signal=31% |
| 7453 | GOBP\_MACROPHAGE\_COLONY\_STIMULATING\_FACTOR\_PRODUCTION |  | 3 | -0.37 | -0.62 | 0.948 | 0.990 | 1.000 | 7591 | tags=100%, list=63%, signal=272% |
| 7454 | GOBP\_RIBONUCLEOSIDE\_METABOLIC\_PROCESS |  | 51 | -0.19 | -0.62 | 0.967 | 0.990 | 1.000 | 3917 | tags=31%, list=33%, signal=46% |
| 7455 | GOBP\_REGULATION\_OF\_NUCLEASE\_ACTIVITY |  | 18 | -0.23 | -0.62 | 0.940 | 0.990 | 1.000 | 4649 | tags=39%, list=39%, signal=63% |
| 7456 | GOBP\_POSITIVE\_REGULATION\_OF\_VACUOLE\_ORGANIZATION |  | 10 | -0.26 | -0.62 | 0.931 | 0.990 | 1.000 | 4324 | tags=40%, list=36%, signal=62% |
| 7457 | GOCC\_DNA\_REPLICATION\_FACTOR\_C\_COMPLEX |  | 5 | -0.32 | -0.62 | 0.935 | 0.990 | 1.000 | 5960 | tags=60%, list=50%, signal=119% |
| 7458 | GOBP\_NEGATIVE\_REGULATION\_OF\_CELL\_GROWTH\_INVOLVED\_IN\_CARDIAC\_MUSCLE\_CELL\_DEVELOPMENT |  | 7 | -0.29 | -0.62 | 0.926 | 0.990 | 1.000 | 4139 | tags=57%, list=34%, signal=87% |
| 7459 | GOBP\_INOSITOL\_TRISPHOSPHATE\_BIOSYNTHETIC\_PROCESS |  | 6 | -0.30 | -0.62 | 0.932 | 0.990 | 1.000 | 4846 | tags=50%, list=40%, signal=84% |
| 7460 | GOBP\_POSITIVE\_REGULATION\_OF\_LIPID\_BIOSYNTHETIC\_PROCESS |  | 58 | -0.18 | -0.62 | 0.976 | 0.990 | 1.000 | 5026 | tags=41%, list=42%, signal=71% |
| 7461 | GOMF\_CARBON\_SULFUR\_LYASE\_ACTIVITY |  | 9 | -0.27 | -0.61 | 0.930 | 0.991 | 1.000 | 263 | tags=11%, list=2%, signal=11% |
| 7462 | GOBP\_KIDNEY\_MESENCHYME\_DEVELOPMENT |  | 13 | -0.25 | -0.61 | 0.933 | 0.991 | 1.000 | 3565 | tags=38%, list=30%, signal=55% |
| 7463 | GOBP\_HEAT\_GENERATION |  | 13 | -0.24 | -0.61 | 0.939 | 0.991 | 1.000 | 1408 | tags=15%, list=12%, signal=17% |
| 7464 | GOBP\_POLY\_A\_PLUS\_MRNA\_EXPORT\_FROM\_NUCLEUS |  | 14 | -0.24 | -0.61 | 0.940 | 0.991 | 1.000 | 4770 | tags=43%, list=40%, signal=71% |
| 7465 | GOMF\_ANNEALING\_ACTIVITY |  | 11 | -0.26 | -0.61 | 0.937 | 0.991 | 1.000 | 3214 | tags=36%, list=27%, signal=50% |
| 7466 | GOCC\_SPINDLE\_POLE\_CENTROSOME |  | 11 | -0.26 | -0.61 | 0.930 | 0.991 | 1.000 | 4983 | tags=27%, list=41%, signal=47% |
| 7467 | GOBP\_NEGATIVE\_REGULATION\_OF\_PROTEIN\_TARGETING\_TO\_MEMBRANE |  | 4 | -0.34 | -0.61 | 0.942 | 0.990 | 1.000 | 5890 | tags=75%, list=49%, signal=147% |
| 7468 | GOBP\_PYRIMIDINE\_NUCLEOSIDE\_METABOLIC\_PROCESS |  | 25 | -0.21 | -0.61 | 0.948 | 0.991 | 1.000 | 5240 | tags=40%, list=44%, signal=71% |
| 7469 | GOBP\_PHOTORECEPTOR\_CELL\_OUTER\_SEGMENT\_ORGANIZATION |  | 7 | -0.29 | -0.61 | 0.931 | 0.991 | 1.000 | 6012 | tags=57%, list=50%, signal=114% |
| 7470 | GOBP\_REGULATION\_OF\_SNRNA\_TRANSCRIPTION\_BY\_RNA\_POLYMERASE\_II |  | 4 | -0.34 | -0.61 | 0.949 | 0.991 | 1.000 | 4182 | tags=50%, list=35%, signal=77% |
| 7471 | GOBP\_OXYGEN\_HOMEOSTASIS |  | 5 | -0.32 | -0.61 | 0.934 | 0.992 | 1.000 | 4557 | tags=40%, list=38%, signal=64% |
| 7472 | GOMF\_HIGH\_DENSITY\_LIPOPROTEIN\_PARTICLE\_BINDING |  | 7 | -0.29 | -0.61 | 0.937 | 0.992 | 1.000 | 1236 | tags=14%, list=10%, signal=16% |
| 7473 | GOBP\_REGULATION\_OF\_MEMBRANE\_REPOLARIZATION |  | 21 | -0.22 | -0.61 | 0.939 | 0.992 | 1.000 | 69 | tags=5%, list=1%, signal=5% |
| 7474 | GOMF\_NUCLEAR\_RECEPTOR\_COACTIVATOR\_ACTIVITY |  | 42 | -0.19 | -0.61 | 0.969 | 0.992 | 1.000 | 4583 | tags=38%, list=38%, signal=61% |
| 7475 | GOBP\_MAINTENANCE\_OF\_GASTROINTESTINAL\_EPITHELIUM |  | 10 | -0.26 | -0.61 | 0.934 | 0.992 | 1.000 | 2852 | tags=30%, list=24%, signal=39% |
| 7476 | GOMF\_UBIQUITIN\_PROTEIN\_TRANSFERASE\_REGULATOR\_ACTIVITY |  | 15 | -0.23 | -0.61 | 0.941 | 0.991 | 1.000 | 3715 | tags=27%, list=31%, signal=39% |
| 7477 | GOBP\_CYTOPLASMIC\_SEQUESTERING\_OF\_PROTEIN |  | 19 | -0.22 | -0.61 | 0.943 | 0.991 | 1.000 | 1861 | tags=16%, list=15%, signal=19% |
| 7478 | GOBP\_POSITIVE\_REGULATION\_OF\_ACTIN\_CYTOSKELETON\_REORGANIZATION |  | 17 | -0.23 | -0.61 | 0.935 | 0.991 | 1.000 | 3695 | tags=29%, list=31%, signal=42% |
| 7479 | GOMF\_RNA\_POLYMERASE\_II\_TRANSCRIPTION\_COACTIVATOR\_BINDING |  | 5 | -0.31 | -0.61 | 0.946 | 0.992 | 1.000 | 711 | tags=20%, list=6%, signal=21% |
| 7480 | GOMF\_TRNA\_METHYLTRANSFERASE\_ACTIVITY |  | 27 | -0.20 | -0.61 | 0.951 | 0.992 | 1.000 | 3379 | tags=26%, list=28%, signal=36% |
| 7481 | GOBP\_COCHLEA\_MORPHOGENESIS |  | 9 | -0.27 | -0.61 | 0.937 | 0.992 | 1.000 | 1318 | tags=11%, list=11%, signal=12% |
| 7482 | GOCC\_INTRINSIC\_COMPONENT\_OF\_ORGANELLE\_MEMBRANE |  | 287 | -0.16 | -0.61 | 1.000 | 0.993 | 1.000 | 4801 | tags=36%, list=40%, signal=58% |
| 7483 | GOBP\_POSITIVE\_REGULATION\_OF\_TRIGLYCERIDE\_BIOSYNTHETIC\_PROCESS |  | 10 | -0.26 | -0.61 | 0.937 | 0.992 | 1.000 | 4853 | tags=50%, list=40%, signal=84% |
| 7484 | GOCC\_WASH\_COMPLEX |  | 6 | -0.30 | -0.61 | 0.937 | 0.992 | 1.000 | 6184 | tags=67%, list=51%, signal=137% |
| 7485 | GOBP\_LEUKOTRIENE\_TRANSPORT |  | 6 | -0.30 | -0.61 | 0.935 | 0.992 | 1.000 | 3930 | tags=67%, list=33%, signal=99% |
| 7486 | GOBP\_NEGATIVE\_REGULATION\_OF\_CARDIAC\_MUSCLE\_TISSUE\_GROWTH |  | 16 | -0.23 | -0.60 | 0.947 | 0.992 | 1.000 | 3392 | tags=38%, list=28%, signal=52% |
| 7487 | GOBP\_REGULATION\_OF\_FLAGELLATED\_SPERM\_MOTILITY |  | 6 | -0.30 | -0.60 | 0.938 | 0.992 | 1.000 | 4402 | tags=67%, list=37%, signal=105% |
| 7488 | GOBP\_TRNA\_METHYLATION |  | 31 | -0.20 | -0.60 | 0.958 | 0.992 | 1.000 | 3379 | tags=26%, list=28%, signal=36% |
| 7489 | GOBP\_INTRACELLULAR\_STEROL\_TRANSPORT |  | 19 | -0.22 | -0.60 | 0.953 | 0.992 | 1.000 | 3789 | tags=37%, list=32%, signal=54% |
| 7490 | GOBP\_STORE\_OPERATED\_CALCIUM\_ENTRY |  | 8 | -0.27 | -0.60 | 0.935 | 0.992 | 1.000 | 3226 | tags=25%, list=27%, signal=34% |
| 7491 | GOBP\_POSITIVE\_REGULATION\_OF\_GUANYLATE\_CYCLASE\_ACTIVITY |  | 3 | -0.36 | -0.60 | 0.953 | 0.993 | 1.000 | 1292 | tags=33%, list=11%, signal=37% |
| 7492 | GOBP\_NUCLEOTIDE\_EXCISION\_REPAIR\_DNA\_DAMAGE\_RECOGNITION |  | 21 | -0.21 | -0.60 | 0.951 | 0.993 | 1.000 | 6021 | tags=52%, list=50%, signal=105% |
| 7493 | GOBP\_POSITIVE\_REGULATION\_OF\_HYDROGEN\_PEROXIDE\_METABOLIC\_PROCESS |  | 4 | -0.33 | -0.60 | 0.949 | 0.993 | 1.000 | 2455 | tags=25%, list=20%, signal=31% |
| 7494 | GOMF\_NUCLEOTIDE\_TRANSMEMBRANE\_TRANSPORTER\_ACTIVITY |  | 18 | -0.22 | -0.60 | 0.947 | 0.993 | 1.000 | 3277 | tags=28%, list=27%, signal=38% |
| 7495 | GOCC\_PRESYNAPTIC\_ENDOCYTIC\_ZONE |  | 6 | -0.30 | -0.60 | 0.946 | 0.993 | 1.000 | 4718 | tags=67%, list=39%, signal=110% |
| 7496 | GOBP\_REGULATION\_OF\_DNA\_HELICASE\_ACTIVITY |  | 5 | -0.31 | -0.60 | 0.940 | 0.993 | 1.000 | 2756 | tags=40%, list=23%, signal=52% |
| 7497 | GOBP\_PEPTIDE\_CROSS\_LINKING\_VIA\_CHONDROITIN\_4\_SULFATE\_GLYCOSAMINOGLYCAN |  | 6 | -0.30 | -0.60 | 0.944 | 0.993 | 1.000 | 3266 | tags=33%, list=27%, signal=46% |
| 7498 | GOBP\_CHOLESTEROL\_CATABOLIC\_PROCESS |  | 8 | -0.27 | -0.60 | 0.938 | 0.993 | 1.000 | 4011 | tags=25%, list=33%, signal=38% |
| 7499 | GOBP\_POSITIVE\_REGULATION\_OF\_PROTEIN\_KINASE\_C\_SIGNALING |  | 4 | -0.33 | -0.60 | 0.951 | 0.993 | 1.000 | 6535 | tags=75%, list=54%, signal=164% |
| 7500 | GOMF\_PHOSPHATIDYLINOSITOL\_N\_ACETYLGLUCOSAMINYLTRANSFERASE\_ACTIVITY |  | 5 | -0.31 | -0.60 | 0.944 | 0.993 | 1.000 | 5495 | tags=60%, list=46%, signal=111% |
| 7501 | GOBP\_PROTEIN\_GERANYLGERANYLATION |  | 6 | -0.30 | -0.60 | 0.947 | 0.993 | 1.000 | 6388 | tags=83%, list=53%, signal=178% |
| 7502 | GOBP\_REGULATION\_OF\_RNA\_POLYMERASE\_II\_REGULATORY\_REGION\_SEQUENCE\_SPECIFIC\_DNA\_BINDING |  | 12 | -0.24 | -0.60 | 0.945 | 0.993 | 1.000 | 5826 | tags=67%, list=49%, signal=129% |
| 7503 | GOCC\_PORE\_COMPLEX |  | 17 | -0.22 | -0.60 | 0.947 | 0.993 | 1.000 | 4655 | tags=41%, list=39%, signal=67% |
| 7504 | GOBP\_METANEPHRIC\_MESENCHYME\_DEVELOPMENT |  | 10 | -0.25 | -0.60 | 0.943 | 0.993 | 1.000 | 161 | tags=10%, list=1%, signal=10% |
| 7505 | GOCC\_SIN3\_COMPLEX |  | 10 | -0.26 | -0.60 | 0.944 | 0.993 | 1.000 | 6612 | tags=80%, list=55%, signal=178% |
| 7506 | GOBP\_NEGATIVE\_REGULATION\_OF\_ISOTYPE\_SWITCHING |  | 4 | -0.33 | -0.60 | 0.957 | 0.993 | 1.000 | 1560 | tags=25%, list=13%, signal=29% |
| 7507 | GOBP\_REGULATION\_OF\_POSTSYNAPTIC\_DENSITY\_ORGANIZATION |  | 8 | -0.27 | -0.60 | 0.942 | 0.994 | 1.000 | 506 | tags=13%, list=4%, signal=13% |
| 7508 | GOBP\_RESPONSE\_TO\_MUSCLE\_STRETCH |  | 16 | -0.23 | -0.60 | 0.947 | 0.994 | 1.000 | 3510 | tags=31%, list=29%, signal=44% |
| 7509 | GOCC\_SODIUM\_POTASSIUM\_EXCHANGING\_ATPASE\_COMPLEX |  | 4 | -0.33 | -0.60 | 0.954 | 0.994 | 1.000 | 4566 | tags=50%, list=38%, signal=81% |
| 7510 | GOBP\_PROTEIN\_K6\_LINKED\_UBIQUITINATION |  | 7 | -0.28 | -0.59 | 0.948 | 0.994 | 1.000 | 4767 | tags=71%, list=40%, signal=118% |
| 7511 | GOBP\_REGULATION\_OF\_CEREBELLAR\_GRANULE\_CELL\_PRECURSOR\_PROLIFERATION |  | 7 | -0.28 | -0.59 | 0.945 | 0.994 | 1.000 | 5085 | tags=43%, list=42%, signal=74% |
| 7512 | GOBP\_APOPTOTIC\_PROCESS\_INVOLVED\_IN\_MORPHOGENESIS |  | 13 | -0.24 | -0.59 | 0.951 | 0.994 | 1.000 | 5855 | tags=54%, list=49%, signal=105% |
| 7513 | GOBP\_NEGATIVE\_REGULATION\_OF\_TRIGLYCERIDE\_METABOLIC\_PROCESS |  | 5 | -0.31 | -0.59 | 0.951 | 0.994 | 1.000 | 5544 | tags=80%, list=46%, signal=149% |
| 7514 | GOMF\_APOLIPOPROTEIN\_A\_I\_BINDING |  | 5 | -0.31 | -0.59 | 0.958 | 0.994 | 1.000 | 4317 | tags=40%, list=36%, signal=62% |
| 7515 | GOBP\_REGULATION\_OF\_DNA\_LIGATION |  | 5 | -0.31 | -0.59 | 0.952 | 0.994 | 1.000 | 5558 | tags=80%, list=46%, signal=149% |
| 7516 | GOBP\_NEGATIVE\_REGULATION\_OF\_PROTEIN\_CONTAINING\_COMPLEX\_ASSEMBLY |  | 98 | -0.16 | -0.59 | 0.991 | 0.994 | 1.000 | 3072 | tags=18%, list=26%, signal=24% |
| 7517 | GOBP\_PROTEIN\_POLYGLUTAMYLATION |  | 10 | -0.25 | -0.59 | 0.949 | 0.994 | 1.000 | 5978 | tags=60%, list=50%, signal=119% |
| 7518 | GOBP\_HISTONE\_H2A\_K63\_LINKED\_UBIQUITINATION |  | 5 | -0.31 | -0.59 | 0.950 | 0.994 | 1.000 | 4409 | tags=80%, list=37%, signal=126% |
| 7519 | GOBP\_NEGATIVE\_REGULATION\_OF\_NOTCH\_SIGNALING\_PATHWAY |  | 25 | -0.20 | -0.59 | 0.957 | 0.994 | 1.000 | 4129 | tags=28%, list=34%, signal=43% |
| 7520 | GOMF\_PEPTIDASE\_ACTIVATOR\_ACTIVITY |  | 28 | -0.20 | -0.59 | 0.963 | 0.994 | 1.000 | 3325 | tags=21%, list=28%, signal=30% |
| 7521 | GOCC\_SPLICEOSOMAL\_TRI\_SNRNP\_COMPLEX |  | 26 | -0.20 | -0.59 | 0.963 | 0.994 | 1.000 | 3604 | tags=19%, list=30%, signal=27% |
| 7522 | GOBP\_REGULATION\_OF\_GLYCOGEN\_CATABOLIC\_PROCESS |  | 4 | -0.33 | -0.59 | 0.950 | 0.995 | 1.000 | 4649 | tags=50%, list=39%, signal=82% |
| 7523 | GOBP\_GLANDULAR\_EPITHELIAL\_CELL\_DEVELOPMENT |  | 21 | -0.21 | -0.59 | 0.955 | 0.995 | 1.000 | 4894 | tags=38%, list=41%, signal=64% |
| 7524 | GOBP\_REGULATION\_OF\_GUANYL\_NUCLEOTIDE\_EXCHANGE\_FACTOR\_ACTIVITY |  | 7 | -0.28 | -0.59 | 0.950 | 0.995 | 1.000 | 4824 | tags=29%, list=40%, signal=48% |
| 7525 | GOMF\_IGE\_BINDING |  | 3 | -0.35 | -0.59 | 0.971 | 0.995 | 1.000 | 2836 | tags=33%, list=24%, signal=44% |
| 7526 | GOCC\_UBIQUITIN\_CONJUGATING\_ENZYME\_COMPLEX |  | 8 | -0.27 | -0.59 | 0.949 | 0.995 | 1.000 | 2118 | tags=13%, list=18%, signal=15% |
| 7527 | GOBP\_NEGATIVE\_REGULATION\_OF\_HORMONE\_METABOLIC\_PROCESS |  | 7 | -0.28 | -0.59 | 0.949 | 0.995 | 1.000 | 2666 | tags=43%, list=22%, signal=55% |
| 7528 | GOBP\_NEGATIVE\_REGULATION\_OF\_DOUBLE\_STRAND\_BREAK\_REPAIR\_VIA\_NONHOMOLOGOUS\_END\_JOINING |  | 4 | -0.33 | -0.59 | 0.957 | 0.995 | 1.000 | 1490 | tags=25%, list=12%, signal=29% |
| 7529 | GOMF\_PROTEIN\_GLUTAMIC\_ACID\_LIGASE\_ACTIVITY |  | 7 | -0.28 | -0.59 | 0.955 | 0.995 | 1.000 | 4002 | tags=43%, list=33%, signal=64% |
| 7530 | GOBP\_CARDIAC\_MUSCLE\_CELL\_MEMBRANE\_REPOLARIZATION |  | 24 | -0.20 | -0.59 | 0.957 | 0.994 | 1.000 | 69 | tags=4%, list=1%, signal=4% |
| 7531 | GOBP\_PYRIMIDINE\_NUCLEOBASE\_METABOLIC\_PROCESS |  | 14 | -0.23 | -0.59 | 0.944 | 0.994 | 1.000 | 5029 | tags=43%, list=42%, signal=74% |
| 7532 | GOBP\_REGULATION\_OF\_SPONTANEOUS\_SYNAPTIC\_TRANSMISSION |  | 4 | -0.33 | -0.59 | 0.955 | 0.994 | 1.000 | 4803 | tags=50%, list=40%, signal=83% |
| 7533 | GOBP\_NEGATIVE\_REGULATION\_OF\_NECROTIC\_CELL\_DEATH |  | 10 | -0.25 | -0.59 | 0.947 | 0.994 | 1.000 | 1084 | tags=10%, list=9%, signal=11% |
| 7534 | GOBP\_DEVELOPMENT\_OF\_SECONDARY\_FEMALE\_SEXUAL\_CHARACTERISTICS |  | 3 | -0.35 | -0.59 | 0.963 | 0.995 | 1.000 | 3615 | tags=33%, list=30%, signal=48% |
| 7535 | GOBP\_REGULATION\_OF\_EXCRETION |  | 10 | -0.25 | -0.59 | 0.949 | 0.995 | 1.000 | 322 | tags=10%, list=3%, signal=10% |
| 7536 | GOBP\_METANEPHRIC\_TUBULE\_MORPHOGENESIS |  | 6 | -0.29 | -0.59 | 0.954 | 0.995 | 1.000 | 4271 | tags=50%, list=36%, signal=78% |
| 7537 | GOMF\_CHANNEL\_INHIBITOR\_ACTIVITY |  | 27 | -0.20 | -0.59 | 0.967 | 0.995 | 1.000 | 4084 | tags=37%, list=34%, signal=56% |
| 7538 | GOCC\_U2\_SNRNP |  | 18 | -0.21 | -0.59 | 0.953 | 0.995 | 1.000 | 3507 | tags=22%, list=29%, signal=31% |
| 7539 | GOBP\_EMBRYO\_IMPLANTATION |  | 34 | -0.19 | -0.59 | 0.969 | 0.995 | 1.000 | 710 | tags=9%, list=6%, signal=9% |
| 7540 | GOMF\_PHOSPHATIDYLETHANOLAMINE\_BINDING |  | 9 | -0.26 | -0.58 | 0.947 | 0.995 | 1.000 | 501 | tags=11%, list=4%, signal=12% |
| 7541 | GOMF\_CERAMIDE\_1\_PHOSPHATE\_TRANSFER\_ACTIVITY |  | 5 | -0.30 | -0.58 | 0.955 | 0.995 | 1.000 | 4444 | tags=60%, list=37%, signal=95% |
| 7542 | GOBP\_PROTEIN\_LOCALIZATION\_TO\_MEMBRANE\_RAFT |  | 8 | -0.27 | -0.58 | 0.955 | 0.994 | 1.000 | 455 | tags=13%, list=4%, signal=13% |
| 7543 | GOBP\_POSITIVE\_REGULATION\_OF\_MITOCHONDRIAL\_MEMBRANE\_POTENTIAL |  | 7 | -0.28 | -0.58 | 0.952 | 0.994 | 1.000 | 3917 | tags=57%, list=33%, signal=85% |
| 7544 | GOBP\_PROTEIN\_HEXAMERIZATION |  | 7 | -0.28 | -0.58 | 0.957 | 0.994 | 1.000 | 4189 | tags=57%, list=35%, signal=88% |
| 7545 | GOBP\_CERAMIDE\_1\_PHOSPHATE\_TRANSPORT |  | 5 | -0.30 | -0.58 | 0.957 | 0.994 | 1.000 | 4444 | tags=60%, list=37%, signal=95% |
| 7546 | GOMF\_WNT\_PROTEIN\_BINDING |  | 25 | -0.20 | -0.58 | 0.966 | 0.994 | 1.000 | 1582 | tags=12%, list=13%, signal=14% |
| 7547 | GOCC\_OUTER\_DYNEIN\_ARM |  | 4 | -0.32 | -0.58 | 0.954 | 0.994 | 1.000 | 1569 | tags=25%, list=13%, signal=29% |
| 7548 | GOBP\_MITOCHONDRIAL\_FRAGMENTATION\_INVOLVED\_IN\_APOPTOTIC\_PROCESS |  | 5 | -0.30 | -0.58 | 0.956 | 0.994 | 1.000 | 7028 | tags=80%, list=59%, signal=193% |
| 7549 | GOBP\_PROTEIN\_DNA\_COMPLEX\_DISASSEMBLY |  | 14 | -0.23 | -0.58 | 0.954 | 0.994 | 1.000 | 3348 | tags=21%, list=28%, signal=30% |
| 7550 | GOCC\_PYRUVATE\_DEHYDROGENASE\_COMPLEX |  | 6 | -0.29 | -0.58 | 0.956 | 0.994 | 1.000 | 3922 | tags=33%, list=33%, signal=49% |
| 7551 | GOBP\_POSITIVE\_REGULATION\_OF\_RESPONSE\_TO\_INTERFERON\_GAMMA |  | 3 | -0.35 | -0.58 | 0.966 | 0.994 | 1.000 | 3615 | tags=67%, list=30%, signal=95% |
| 7552 | GOBP\_RESPONSE\_TO\_FATTY\_ACID |  | 40 | -0.18 | -0.58 | 0.974 | 0.994 | 1.000 | 3968 | tags=33%, list=33%, signal=48% |
| 7553 | GOBP\_NEGATIVE\_REGULATION\_OF\_METALLOPEPTIDASE\_ACTIVITY |  | 6 | -0.29 | -0.58 | 0.958 | 0.995 | 1.000 | 4369 | tags=50%, list=36%, signal=79% |
| 7554 | GOMF\_RNA\_POLYMERASE\_II\_REPRESSING\_TRANSCRIPTION\_FACTOR\_BINDING |  | 30 | -0.19 | -0.58 | 0.968 | 0.995 | 1.000 | 2692 | tags=23%, list=22%, signal=30% |
| 7555 | GOBP\_REGULATION\_OF\_DNA\_TEMPLATED\_TRANSCRIPTION\_IN\_RESPONSE\_TO\_STRESS |  | 85 | -0.16 | -0.58 | 0.991 | 0.995 | 1.000 | 4292 | tags=26%, list=36%, signal=40% |
| 7556 | GOBP\_MONOCYTE\_DIFFERENTIATION |  | 26 | -0.20 | -0.58 | 0.967 | 0.995 | 1.000 | 1843 | tags=19%, list=15%, signal=23% |
| 7557 | GOBP\_PYRIMIDINE\_NUCLEOSIDE\_MONOPHOSPHATE\_METABOLIC\_PROCESS |  | 16 | -0.22 | -0.58 | 0.958 | 0.995 | 1.000 | 5118 | tags=38%, list=43%, signal=65% |
| 7558 | GOBP\_MEIOTIC\_CELL\_CYCLE\_CHECKPOINT |  | 6 | -0.28 | -0.58 | 0.955 | 0.995 | 1.000 | 3976 | tags=33%, list=33%, signal=50% |
| 7559 | GOBP\_LONG\_CHAIN\_FATTY\_ACID\_IMPORT\_ACROSS\_PLASMA\_MEMBRANE |  | 8 | -0.26 | -0.58 | 0.953 | 0.995 | 1.000 | 3844 | tags=50%, list=32%, signal=73% |
| 7560 | GOBP\_NEGATIVE\_REGULATION\_OF\_SYSTEMIC\_ARTERIAL\_BLOOD\_PRESSURE |  | 8 | -0.26 | -0.58 | 0.956 | 0.995 | 1.000 | 314 | tags=13%, list=3%, signal=13% |
| 7561 | GOCC\_CILIARY\_TIP |  | 39 | -0.18 | -0.58 | 0.979 | 0.995 | 1.000 | 910 | tags=8%, list=8%, signal=8% |
| 7562 | GOMF\_ACTIVIN\_BINDING |  | 14 | -0.23 | -0.58 | 0.957 | 0.996 | 1.000 | 2106 | tags=21%, list=18%, signal=26% |
| 7563 | GOBP\_GLYCERALDEHYDE\_3\_PHOSPHATE\_METABOLIC\_PROCESS |  | 7 | -0.27 | -0.58 | 0.956 | 0.996 | 1.000 | 3191 | tags=29%, list=27%, signal=39% |
| 7564 | GOCC\_MRNA\_CLEAVAGE\_AND\_POLYADENYLATION\_SPECIFICITY\_FACTOR\_COMPLEX |  | 13 | -0.23 | -0.58 | 0.960 | 0.996 | 1.000 | 4261 | tags=31%, list=35%, signal=48% |
| 7565 | GOBP\_NEGATIVE\_REGULATION\_OF\_PROGRAMMED\_NECROTIC\_CELL\_DEATH |  | 9 | -0.25 | -0.58 | 0.951 | 0.995 | 1.000 | 1084 | tags=11%, list=9%, signal=12% |
| 7566 | GOBP\_REGULATION\_OF\_INOSITOL\_1\_4\_5\_TRISPHOSPHATE\_SENSITIVE\_CALCIUM\_RELEASE\_CHANNEL\_ACTIVITY |  | 4 | -0.32 | -0.58 | 0.965 | 0.995 | 1.000 | 5330 | tags=50%, list=44%, signal=90% |
| 7567 | GOBP\_UTERUS\_DEVELOPMENT |  | 11 | -0.24 | -0.57 | 0.954 | 0.995 | 1.000 | 2921 | tags=27%, list=24%, signal=36% |
| 7568 | GOBP\_PROTEIN\_UFMYLATION |  | 6 | -0.28 | -0.57 | 0.961 | 0.995 | 1.000 | 3132 | tags=33%, list=26%, signal=45% |
| 7569 | GOBP\_RNA\_METHYLATION |  | 62 | -0.17 | -0.57 | 0.990 | 0.995 | 1.000 | 4252 | tags=31%, list=35%, signal=47% |
| 7570 | GOBP\_RESPONSE\_TO\_OLEIC\_ACID |  | 5 | -0.30 | -0.57 | 0.960 | 0.996 | 1.000 | 4801 | tags=60%, list=40%, signal=100% |
| 7571 | GOMF\_PHOSPHATIDYLINOSITOL\_4\_5\_BISPHOSPHATE\_BINDING |  | 51 | -0.17 | -0.57 | 0.986 | 0.996 | 1.000 | 4695 | tags=35%, list=39%, signal=58% |
| 7572 | GOCC\_CORTICAL\_CYTOSKELETON |  | 79 | -0.16 | -0.57 | 0.993 | 0.995 | 1.000 | 2303 | tags=18%, list=19%, signal=22% |
| 7573 | GOMF\_PROSTAGLANDIN\_E\_RECEPTOR\_ACTIVITY |  | 4 | -0.32 | -0.57 | 0.962 | 0.995 | 1.000 | 4069 | tags=50%, list=34%, signal=76% |
| 7574 | GOMF\_PROTEIN\_LYSINE\_6\_OXIDASE\_ACTIVITY |  | 5 | -0.30 | -0.57 | 0.959 | 0.995 | 1.000 | 1723 | tags=20%, list=14%, signal=23% |
| 7575 | GOBP\_TERMINATION\_OF\_RNA\_POLYMERASE\_I\_TRANSCRIPTION |  | 24 | -0.20 | -0.57 | 0.967 | 0.995 | 1.000 | 5069 | tags=42%, list=42%, signal=72% |
| 7576 | GOCC\_EUKARYOTIC\_TRANSLATION\_INITIATION\_FACTOR\_3\_COMPLEX |  | 14 | -0.22 | -0.57 | 0.961 | 0.995 | 1.000 | 2731 | tags=14%, list=23%, signal=18% |
| 7577 | GOBP\_ATP\_TRANSPORT |  | 12 | -0.23 | -0.57 | 0.962 | 0.995 | 1.000 | 2605 | tags=25%, list=22%, signal=32% |
| 7578 | GOBP\_LOCOMOTOR\_RHYTHM |  | 11 | -0.24 | -0.57 | 0.958 | 0.995 | 1.000 | 3392 | tags=36%, list=28%, signal=51% |
| 7579 | GOCC\_CUL4B\_RING\_E3\_UBIQUITIN\_LIGASE\_COMPLEX |  | 5 | -0.30 | -0.57 | 0.960 | 0.995 | 1.000 | 995 | tags=20%, list=8%, signal=22% |
| 7580 | GOCC\_MICROBODY\_LUMEN |  | 44 | -0.18 | -0.57 | 0.981 | 0.995 | 1.000 | 4181 | tags=32%, list=35%, signal=49% |
| 7581 | GOBP\_POSITIVE\_REGULATION\_OF\_FIBROBLAST\_GROWTH\_FACTOR\_RECEPTOR\_SIGNALING\_PATHWAY |  | 3 | -0.34 | -0.57 | 0.974 | 0.995 | 1.000 | 2613 | tags=33%, list=22%, signal=43% |
| 7582 | GOBP\_NEUROTRANSMITTER\_REUPTAKE |  | 20 | -0.20 | -0.57 | 0.965 | 0.995 | 1.000 | 2563 | tags=20%, list=21%, signal=25% |
| 7583 | GOBP\_RIBOSOMAL\_SMALL\_SUBUNIT\_EXPORT\_FROM\_NUCLEUS |  | 7 | -0.27 | -0.57 | 0.958 | 0.995 | 1.000 | 4278 | tags=29%, list=36%, signal=44% |
| 7584 | GOBP\_REGULATION\_OF\_PROTEIN\_MONOUBIQUITINATION |  | 5 | -0.30 | -0.57 | 0.962 | 0.995 | 1.000 | 1706 | tags=20%, list=14%, signal=23% |
| 7585 | GOBP\_RNA\_PROCESSING |  | 712 | -0.14 | -0.57 | 1.000 | 0.995 | 1.000 | 5215 | tags=34%, list=43%, signal=56% |
| 7586 | GOBP\_PEPTIDYL\_LYSINE\_OXIDATION |  | 5 | -0.30 | -0.57 | 0.965 | 0.995 | 1.000 | 1723 | tags=20%, list=14%, signal=23% |
| 7587 | GOBP\_BRANCHED\_CHAIN\_AMINO\_ACID\_BIOSYNTHETIC\_PROCESS |  | 3 | -0.34 | -0.57 | 0.975 | 0.995 | 1.000 | 2726 | tags=33%, list=23%, signal=43% |
| 7588 | GOMF\_PROTEIN\_GERANYLGERANYLTRANSFERASE\_ACTIVITY |  | 5 | -0.29 | -0.57 | 0.963 | 0.995 | 1.000 | 5013 | tags=60%, list=42%, signal=103% |
| 7589 | GOBP\_POSITIVE\_REGULATION\_OF\_METALLOPEPTIDASE\_ACTIVITY |  | 5 | -0.30 | -0.57 | 0.962 | 0.995 | 1.000 | 2781 | tags=40%, list=23%, signal=52% |
| 7590 | GOBP\_NEGATIVE\_REGULATION\_OF\_INTRACELLULAR\_PROTEIN\_TRANSPORT |  | 33 | -0.19 | -0.57 | 0.976 | 0.995 | 1.000 | 3934 | tags=24%, list=33%, signal=36% |
| 7591 | GOBP\_REGULATION\_OF\_MONOCYTE\_DIFFERENTIATION |  | 16 | -0.22 | -0.57 | 0.962 | 0.995 | 1.000 | 1138 | tags=13%, list=9%, signal=14% |
| 7592 | GOCC\_COMPACT\_MYELIN |  | 9 | -0.25 | -0.57 | 0.958 | 0.995 | 1.000 | 5305 | tags=56%, list=44%, signal=99% |
| 7593 | GOMF\_PHOTORECEPTOR\_ACTIVITY |  | 3 | -0.34 | -0.57 | 0.972 | 0.995 | 1.000 | 4306 | tags=67%, list=36%, signal=104% |
| 7594 | GOBP\_PHOTORECEPTOR\_CELL\_MAINTENANCE |  | 22 | -0.20 | -0.57 | 0.972 | 0.995 | 1.000 | 5276 | tags=50%, list=44%, signal=89% |
| 7595 | GOMF\_EUKARYOTIC\_INITIATION\_FACTOR\_4E\_BINDING |  | 7 | -0.26 | -0.57 | 0.962 | 0.995 | 1.000 | 2731 | tags=29%, list=23%, signal=37% |
| 7596 | GOBP\_ENDOSOME\_TRANSPORT\_VIA\_MULTIVESICULAR\_BODY\_SORTING\_PATHWAY |  | 26 | -0.19 | -0.57 | 0.975 | 0.995 | 1.000 | 2774 | tags=19%, list=23%, signal=25% |
| 7597 | GOBP\_POSITIVE\_REGULATION\_OF\_FEVER\_GENERATION |  | 6 | -0.28 | -0.56 | 0.966 | 0.995 | 1.000 | 5026 | tags=67%, list=42%, signal=115% |
| 7598 | GOBP\_REGULATION\_OF\_RECEPTOR\_INTERNALIZATION |  | 36 | -0.18 | -0.56 | 0.979 | 0.995 | 1.000 | 5595 | tags=53%, list=47%, signal=99% |
| 7599 | GOBP\_N\_TERMINAL\_PROTEIN\_AMINO\_ACID\_MODIFICATION |  | 23 | -0.20 | -0.56 | 0.970 | 0.995 | 1.000 | 4976 | tags=39%, list=41%, signal=67% |
| 7600 | GOBP\_PROTEIN\_TARGETING\_TO\_MEMBRANE |  | 152 | -0.15 | -0.56 | 0.999 | 0.995 | 1.000 | 1222 | tags=7%, list=10%, signal=7% |
| 7601 | GOBP\_PROTEIN\_CHROMOPHORE\_LINKAGE |  | 3 | -0.34 | -0.56 | 0.972 | 0.995 | 1.000 | 4306 | tags=67%, list=36%, signal=104% |
| 7602 | GOBP\_POSITIVE\_REGULATION\_OF\_CALCIUM\_ION\_TRANSMEMBRANE\_TRANSPORTER\_ACTIVITY |  | 26 | -0.19 | -0.56 | 0.976 | 0.995 | 1.000 | 2908 | tags=23%, list=24%, signal=30% |
| 7603 | GOBP\_MEMBRANE\_REPOLARIZATION |  | 28 | -0.19 | -0.56 | 0.976 | 0.995 | 1.000 | 5438 | tags=43%, list=45%, signal=78% |
| 7604 | GOBP\_POSITIVE\_REGULATION\_OF\_NUCLEAR\_DIVISION |  | 39 | -0.18 | -0.56 | 0.985 | 0.995 | 1.000 | 576 | tags=8%, list=5%, signal=8% |
| 7605 | GOMF\_MUSCLE\_ALPHA\_ACTININ\_BINDING |  | 9 | -0.25 | -0.56 | 0.968 | 0.995 | 1.000 | 2171 | tags=11%, list=18%, signal=14% |
| 7606 | GOBP\_GLYCINE\_TRANSPORT |  | 6 | -0.28 | -0.56 | 0.967 | 0.995 | 1.000 | 473 | tags=17%, list=4%, signal=17% |
| 7607 | GOBP\_NEGATIVE\_REGULATION\_OF\_ARP2\_3\_COMPLEX\_MEDIATED\_ACTIN\_NUCLEATION |  | 4 | -0.31 | -0.56 | 0.968 | 0.995 | 1.000 | 3032 | tags=50%, list=25%, signal=67% |
| 7608 | GOCC\_SPLICEOSOMAL\_COMPLEX |  | 147 | -0.15 | -0.56 | 0.998 | 0.995 | 1.000 | 6515 | tags=48%, list=54%, signal=104% |
| 7609 | GOBP\_REGULATION\_OF\_DELAYED\_RECTIFIER\_POTASSIUM\_CHANNEL\_ACTIVITY |  | 11 | -0.23 | -0.56 | 0.962 | 0.995 | 1.000 | 2866 | tags=27%, list=24%, signal=36% |
| 7610 | GOBP\_CHONDROCYTE\_DIFFERENTIATION\_INVOLVED\_IN\_ENDOCHONDRAL\_BONE\_MORPHOGENESIS |  | 11 | -0.23 | -0.56 | 0.960 | 0.995 | 1.000 | 6131 | tags=64%, list=51%, signal=130% |
| 7611 | GOMF\_INTRAMOLECULAR\_TRANSFERASE\_ACTIVITY |  | 20 | -0.20 | -0.56 | 0.972 | 0.995 | 1.000 | 1234 | tags=10%, list=10%, signal=11% |
| 7612 | GOBP\_NEGATIVE\_REGULATION\_OF\_ACTIN\_NUCLEATION |  | 4 | -0.31 | -0.56 | 0.970 | 0.995 | 1.000 | 3032 | tags=50%, list=25%, signal=67% |
| 7613 | GOMF\_CARBON\_NITROGEN\_LIGASE\_ACTIVITY\_WITH\_GLUTAMINE\_AS\_AMIDO\_N\_DONOR |  | 9 | -0.24 | -0.56 | 0.966 | 0.996 | 1.000 | 3188 | tags=22%, list=27%, signal=30% |
| 7614 | GOMF\_MANNOSE\_BINDING |  | 10 | -0.24 | -0.56 | 0.967 | 0.996 | 1.000 | 1181 | tags=20%, list=10%, signal=22% |
| 7615 | GOBP\_MAINTENANCE\_OF\_BLOOD\_BRAIN\_BARRIER |  | 25 | -0.19 | -0.56 | 0.975 | 0.996 | 1.000 | 3196 | tags=28%, list=27%, signal=38% |
| 7616 | GOBP\_PROTEIN\_POLYUFMYLATION |  | 5 | -0.29 | -0.56 | 0.970 | 0.996 | 1.000 | 3132 | tags=40%, list=26%, signal=54% |
| 7617 | GOMF\_TRANSFERASE\_ACTIVITY\_TRANSFERRING\_ACYL\_GROUPS\_ACYL\_GROUPS\_CONVERTED\_INTO\_ALKYL\_ON\_TRANSFER |  | 4 | -0.31 | -0.56 | 0.971 | 0.996 | 1.000 | 4095 | tags=50%, list=34%, signal=76% |
| 7618 | GOBP\_NEGATIVE\_REGULATION\_OF\_MRNA\_3\_END\_PROCESSING |  | 8 | -0.25 | -0.55 | 0.964 | 0.996 | 1.000 | 4968 | tags=50%, list=41%, signal=85% |
| 7619 | GOMF\_MYOSIN\_II\_BINDING |  | 8 | -0.25 | -0.55 | 0.971 | 0.996 | 1.000 | 1298 | tags=13%, list=11%, signal=14% |
| 7620 | GOMF\_ALCOHOL\_DEHYDROGENASE\_NAD\_P\_PLUS\_ACTIVITY |  | 5 | -0.29 | -0.55 | 0.971 | 0.996 | 1.000 | 3008 | tags=40%, list=25%, signal=53% |
| 7621 | GOBP\_REGULATION\_OF\_PROTEIN\_CATABOLIC\_PROCESS\_IN\_THE\_VACUOLE |  | 9 | -0.24 | -0.55 | 0.968 | 0.996 | 1.000 | 1456 | tags=22%, list=12%, signal=25% |
| 7622 | GOBP\_CARTILAGE\_DEVELOPMENT\_INVOLVED\_IN\_ENDOCHONDRAL\_BONE\_MORPHOGENESIS |  | 18 | -0.20 | -0.55 | 0.970 | 0.996 | 1.000 | 6131 | tags=61%, list=51%, signal=125% |
| 7623 | GOBP\_NUCLEOTIDE\_SUGAR\_TRANSMEMBRANE\_TRANSPORT |  | 8 | -0.25 | -0.55 | 0.963 | 0.996 | 1.000 | 5044 | tags=50%, list=42%, signal=86% |
| 7624 | GOMF\_OUTWARD\_RECTIFIER\_POTASSIUM\_CHANNEL\_ACTIVITY |  | 7 | -0.26 | -0.55 | 0.965 | 0.996 | 1.000 | 5330 | tags=71%, list=44%, signal=128% |
| 7625 | GOBP\_POSITIVE\_REGULATION\_OF\_RECEPTOR\_RECYCLING |  | 10 | -0.23 | -0.55 | 0.965 | 0.996 | 1.000 | 2144 | tags=20%, list=18%, signal=24% |
| 7626 | GOBP\_WALKING\_BEHAVIOR |  | 14 | -0.21 | -0.55 | 0.967 | 0.996 | 1.000 | 3126 | tags=29%, list=26%, signal=39% |
| 7627 | GOBP\_PROTEIN\_FOLDING |  | 166 | -0.15 | -0.55 | 1.000 | 0.996 | 1.000 | 5097 | tags=35%, list=42%, signal=60% |
| 7628 | GOBP\_HEMOGLOBIN\_METABOLIC\_PROCESS |  | 10 | -0.23 | -0.55 | 0.969 | 0.996 | 1.000 | 7122 | tags=70%, list=59%, signal=172% |
| 7629 | GOCC\_SMALL\_NUCLEAR\_RIBONUCLEOPROTEIN\_COMPLEX |  | 53 | -0.16 | -0.55 | 0.993 | 0.996 | 1.000 | 4182 | tags=25%, list=35%, signal=37% |
| 7630 | GOBP\_MAINTENANCE\_OF\_PRESYNAPTIC\_ACTIVE\_ZONE\_STRUCTURE |  | 4 | -0.30 | -0.55 | 0.975 | 0.996 | 1.000 | 4455 | tags=75%, list=37%, signal=119% |
| 7631 | GOBP\_POSITIVE\_REGULATION\_OF\_ISOTYPE\_SWITCHING\_TO\_IGG\_ISOTYPES |  | 7 | -0.26 | -0.55 | 0.975 | 0.996 | 1.000 | 2639 | tags=29%, list=22%, signal=37% |
| 7632 | GOMF\_CALCIUM\_CHANNEL\_INHIBITOR\_ACTIVITY |  | 8 | -0.25 | -0.55 | 0.970 | 0.996 | 1.000 | 4627 | tags=50%, list=39%, signal=81% |
| 7633 | GOCC\_CILIARY\_TRANSITION\_ZONE |  | 48 | -0.17 | -0.55 | 0.991 | 0.996 | 1.000 | 4047 | tags=31%, list=34%, signal=47% |
| 7634 | GOBP\_RNA\_3\_END\_PROCESSING |  | 110 | -0.15 | -0.55 | 0.997 | 0.996 | 1.000 | 6160 | tags=46%, list=51%, signal=94% |
| 7635 | GOBP\_NEGATIVE\_REGULATION\_OF\_PROTEIN\_POLYMERIZATION |  | 55 | -0.16 | -0.55 | 0.992 | 0.996 | 1.000 | 2598 | tags=16%, list=22%, signal=21% |
| 7636 | GOBP\_PROTEIN\_K27\_LINKED\_UBIQUITINATION |  | 3 | -0.33 | -0.55 | 0.979 | 0.996 | 1.000 | 4645 | tags=67%, list=39%, signal=109% |
| 7637 | GOBP\_RESPONSE\_TO\_LEAD\_ION |  | 16 | -0.21 | -0.55 | 0.971 | 0.996 | 1.000 | 3062 | tags=25%, list=25%, signal=34% |
| 7638 | GOMF\_CATALYTIC\_ACTIVITY\_ACTING\_ON\_RNA |  | 269 | -0.14 | -0.54 | 1.000 | 0.996 | 1.000 | 5527 | tags=39%, list=46%, signal=71% |
| 7639 | GOCC\_INHIBITORY\_SYNAPSE |  | 8 | -0.25 | -0.54 | 0.971 | 0.996 | 1.000 | 2294 | tags=13%, list=19%, signal=15% |
| 7640 | GOCC\_INTRACILIARY\_TRANSPORT\_PARTICLE\_A |  | 5 | -0.28 | -0.54 | 0.973 | 0.996 | 1.000 | 3051 | tags=20%, list=25%, signal=27% |
| 7641 | GOBP\_NEGATIVE\_REGULATION\_OF\_RESPONSE\_TO\_REACTIVE\_OXYGEN\_SPECIES |  | 12 | -0.22 | -0.54 | 0.971 | 0.996 | 1.000 | 1935 | tags=17%, list=16%, signal=20% |
| 7642 | GOBP\_IRON\_IMPORT\_INTO\_CELL |  | 5 | -0.28 | -0.54 | 0.973 | 0.996 | 1.000 | 2258 | tags=20%, list=19%, signal=25% |
| 7643 | GOMF\_CALCIUM\_CHANNEL\_REGULATOR\_ACTIVITY |  | 27 | -0.18 | -0.54 | 0.983 | 0.997 | 1.000 | 4819 | tags=37%, list=40%, signal=62% |
| 7644 | GOBP\_TETRAPYRROLE\_METABOLIC\_PROCESS |  | 43 | -0.17 | -0.54 | 0.992 | 0.997 | 1.000 | 4320 | tags=30%, list=36%, signal=47% |
| 7645 | GOBP\_RENAL\_SODIUM\_EXCRETION |  | 10 | -0.23 | -0.54 | 0.977 | 0.997 | 1.000 | 322 | tags=10%, list=3%, signal=10% |
| 7646 | GOBP\_PHOSPHOLIPID\_HOMEOSTASIS |  | 12 | -0.22 | -0.54 | 0.973 | 0.997 | 1.000 | 2752 | tags=25%, list=23%, signal=32% |
| 7647 | GOBP\_REGULATION\_OF\_PLATELET\_DERIVED\_GROWTH\_FACTOR\_RECEPTOR\_BETA\_SIGNALING\_PATHWAY |  | 8 | -0.25 | -0.54 | 0.975 | 0.997 | 1.000 | 5607 | tags=63%, list=47%, signal=117% |
| 7648 | GOBP\_MULTI\_ORGANISM\_MEMBRANE\_ORGANIZATION |  | 8 | -0.24 | -0.54 | 0.971 | 0.997 | 1.000 | 4173 | tags=38%, list=35%, signal=57% |
| 7649 | GOBP\_NEGATIVE\_REGULATION\_OF\_TRANSLATION\_IN\_RESPONSE\_TO\_STRESS |  | 5 | -0.28 | -0.54 | 0.974 | 0.997 | 1.000 | 3872 | tags=40%, list=32%, signal=59% |
| 7650 | GOBP\_NEGATIVE\_REGULATION\_OF\_CALCINEURIN\_MEDIATED\_SIGNALING |  | 9 | -0.23 | -0.54 | 0.973 | 0.997 | 1.000 | 3230 | tags=33%, list=27%, signal=46% |
| 7651 | GOBP\_RHODOPSIN\_MEDIATED\_SIGNALING\_PATHWAY |  | 12 | -0.22 | -0.54 | 0.975 | 0.997 | 1.000 | 6229 | tags=67%, list=52%, signal=138% |
| 7652 | GOCC\_ENDOSOME\_LUMEN |  | 18 | -0.20 | -0.54 | 0.979 | 0.997 | 1.000 | 4758 | tags=39%, list=40%, signal=64% |
| 7653 | GOBP\_POSITIVE\_REGULATION\_OF\_MRNA\_PROCESSING |  | 24 | -0.19 | -0.54 | 0.978 | 0.997 | 1.000 | 4569 | tags=38%, list=38%, signal=60% |
| 7654 | GOMF\_OXIDOREDUCTASE\_ACTIVITY\_ACTING\_ON\_THE\_ALDEHYDE\_OR\_OXO\_GROUP\_OF\_DONORS\_DISULFIDE\_AS\_ACCEPTOR |  | 7 | -0.25 | -0.54 | 0.977 | 0.997 | 1.000 | 931 | tags=14%, list=8%, signal=15% |
| 7655 | GOBP\_PARATHYROID\_GLAND\_DEVELOPMENT |  | 4 | -0.29 | -0.54 | 0.983 | 0.997 | 1.000 | 1141 | tags=25%, list=9%, signal=28% |
| 7656 | GOBP\_TYPE\_B\_PANCREATIC\_CELL\_DEVELOPMENT |  | 17 | -0.20 | -0.54 | 0.979 | 0.997 | 1.000 | 4894 | tags=41%, list=41%, signal=69% |
| 7657 | GOBP\_NEGATIVE\_REGULATION\_OF\_PLASMA\_MEMBRANE\_BOUNDED\_CELL\_PROJECTION\_ASSEMBLY |  | 21 | -0.19 | -0.54 | 0.976 | 0.997 | 1.000 | 5206 | tags=38%, list=43%, signal=67% |
| 7658 | GOBP\_GOLGI\_VESICLE\_BUDDING |  | 9 | -0.23 | -0.53 | 0.975 | 0.997 | 1.000 | 5305 | tags=44%, list=44%, signal=80% |
| 7659 | GOCC\_ENDOPLASMIC\_RETICULUM\_PROTEIN\_CONTAINING\_COMPLEX |  | 94 | -0.15 | -0.53 | 0.998 | 0.997 | 1.000 | 2242 | tags=15%, list=19%, signal=18% |
| 7660 | GOBP\_NCRNA\_CATABOLIC\_PROCESS |  | 23 | -0.19 | -0.53 | 0.982 | 0.997 | 1.000 | 3968 | tags=35%, list=33%, signal=52% |
| 7661 | GOBP\_ANTIGEN\_PROCESSING\_AND\_PRESENTATION\_OF\_ENDOGENOUS\_ANTIGEN |  | 10 | -0.23 | -0.53 | 0.973 | 0.997 | 1.000 | 2881 | tags=30%, list=24%, signal=39% |
| 7662 | GOBP\_POSITIVE\_REGULATION\_OF\_DEFENSE\_RESPONSE\_TO\_VIRUS\_BY\_HOST |  | 14 | -0.21 | -0.53 | 0.975 | 0.997 | 1.000 | 3235 | tags=36%, list=27%, signal=49% |
| 7663 | GOBP\_NEGATIVE\_REGULATION\_OF\_TYPE\_B\_PANCREATIC\_CELL\_APOPTOTIC\_PROCESS |  | 5 | -0.28 | -0.53 | 0.979 | 0.997 | 1.000 | 959 | tags=20%, list=8%, signal=22% |
| 7664 | GOMF\_CARGO\_ADAPTOR\_ACTIVITY |  | 12 | -0.22 | -0.53 | 0.977 | 0.997 | 1.000 | 6575 | tags=75%, list=55%, signal=166% |
| 7665 | GOMF\_PORIN\_ACTIVITY |  | 4 | -0.29 | -0.53 | 0.983 | 0.997 | 1.000 | 1952 | tags=25%, list=16%, signal=30% |
| 7666 | GOBP\_POSITIVE\_REGULATION\_OF\_MONOCYTE\_DIFFERENTIATION |  | 9 | -0.23 | -0.53 | 0.981 | 0.997 | 1.000 | 1138 | tags=11%, list=9%, signal=12% |
| 7667 | GOBP\_ERROR\_FREE\_TRANSLESION\_SYNTHESIS |  | 19 | -0.19 | -0.53 | 0.981 | 0.997 | 1.000 | 5351 | tags=42%, list=45%, signal=76% |
| 7668 | GOCC\_CYTOSOLIC\_PROTEASOME\_COMPLEX |  | 5 | -0.27 | -0.53 | 0.978 | 0.997 | 1.000 | 2218 | tags=20%, list=18%, signal=25% |
| 7669 | GOBP\_PYRIMIDINE\_CONTAINING\_COMPOUND\_METABOLIC\_PROCESS |  | 59 | -0.16 | -0.53 | 0.994 | 0.997 | 1.000 | 5240 | tags=37%, list=44%, signal=66% |
| 7670 | GOMF\_RNA\_POLYMERASE\_II\_GENERAL\_TRANSCRIPTION\_INITIATION\_FACTOR\_BINDING |  | 12 | -0.21 | -0.53 | 0.976 | 0.997 | 1.000 | 3177 | tags=17%, list=26%, signal=23% |
| 7671 | GOMF\_GLUTATHIONE\_DISULFIDE\_OXIDOREDUCTASE\_ACTIVITY |  | 4 | -0.29 | -0.53 | 0.984 | 0.997 | 1.000 | 2658 | tags=25%, list=22%, signal=32% |
| 7672 | GOBP\_PROTEIN\_K29\_LINKED\_UBIQUITINATION |  | 3 | -0.32 | -0.52 | 0.983 | 0.997 | 1.000 | 4645 | tags=67%, list=39%, signal=109% |
| 7673 | GOBP\_LIPOPROTEIN\_CATABOLIC\_PROCESS |  | 14 | -0.20 | -0.52 | 0.977 | 0.997 | 1.000 | 4609 | tags=50%, list=38%, signal=81% |
| 7674 | GOCC\_PHOTORECEPTOR\_CONNECTING\_CILIUM |  | 23 | -0.18 | -0.52 | 0.983 | 0.997 | 1.000 | 4047 | tags=30%, list=34%, signal=46% |
| 7675 | GOCC\_NURD\_COMPLEX |  | 13 | -0.21 | -0.52 | 0.980 | 0.997 | 1.000 | 6456 | tags=62%, list=54%, signal=133% |
| 7676 | GOBP\_REGULATION\_OF\_LIGASE\_ACTIVITY |  | 9 | -0.23 | -0.52 | 0.975 | 0.997 | 1.000 | 3632 | tags=33%, list=30%, signal=48% |
| 7677 | GOBP\_INTRACILIARY\_TRANSPORT |  | 41 | -0.16 | -0.52 | 0.992 | 0.997 | 1.000 | 3606 | tags=22%, list=30%, signal=31% |
| 7678 | GOBP\_NEGATIVE\_REGULATION\_OF\_PROTEIN\_LOCALIZATION\_TO\_MEMBRANE |  | 23 | -0.18 | -0.52 | 0.988 | 0.997 | 1.000 | 4369 | tags=35%, list=36%, signal=55% |
| 7679 | GOMF\_RNA\_STRAND\_ANNEALING\_ACTIVITY |  | 5 | -0.27 | -0.52 | 0.980 | 0.998 | 1.000 | 5944 | tags=60%, list=49%, signal=119% |
| 7680 | GOMF\_SNRNA\_BINDING |  | 33 | -0.17 | -0.52 | 0.991 | 0.997 | 1.000 | 4182 | tags=27%, list=35%, signal=42% |
| 7681 | GOBP\_REGULATION\_OF\_FATTY\_ACID\_BETA\_OXIDATION |  | 16 | -0.19 | -0.52 | 0.983 | 0.998 | 1.000 | 1748 | tags=19%, list=15%, signal=22% |
| 7682 | GOMF\_GLYCINE\_TRANSMEMBRANE\_TRANSPORTER\_ACTIVITY |  | 3 | -0.31 | -0.52 | 0.988 | 0.998 | 1.000 | 3022 | tags=33%, list=25%, signal=45% |
| 7683 | GOBP\_CELLULAR\_RESPONSE\_TO\_THYROID\_HORMONE\_STIMULUS |  | 12 | -0.21 | -0.51 | 0.984 | 0.998 | 1.000 | 199 | tags=8%, list=2%, signal=8% |
| 7684 | GOCC\_DIHYDROLIPOYL\_DEHYDROGENASE\_COMPLEX |  | 9 | -0.23 | -0.51 | 0.980 | 0.998 | 1.000 | 931 | tags=11%, list=8%, signal=12% |
| 7685 | GOBP\_REGULATION\_OF\_INOSITOL\_PHOSPHATE\_BIOSYNTHETIC\_PROCESS |  | 9 | -0.22 | -0.51 | 0.983 | 0.998 | 1.000 | 788 | tags=11%, list=7%, signal=12% |
| 7686 | GOCC\_90S\_PRERIBOSOME |  | 24 | -0.18 | -0.51 | 0.991 | 0.998 | 1.000 | 3380 | tags=25%, list=28%, signal=35% |
| 7687 | GOMF\_NUCLEOTIDE\_SUGAR\_TRANSMEMBRANE\_TRANSPORTER\_ACTIVITY |  | 8 | -0.23 | -0.51 | 0.985 | 0.999 | 1.000 | 2868 | tags=25%, list=24%, signal=33% |
| 7688 | GOMF\_CARDIOLIPIN\_BINDING |  | 7 | -0.24 | -0.51 | 0.984 | 0.999 | 1.000 | 3525 | tags=43%, list=29%, signal=61% |
| 7689 | GOBP\_POSITIVE\_REGULATION\_OF\_CELL\_PROLIFERATION\_INVOLVED\_IN\_HEART\_MORPHOGENESIS |  | 4 | -0.28 | -0.51 | 0.986 | 0.998 | 1.000 | 5967 | tags=75%, list=50%, signal=149% |
| 7690 | GOBP\_NEGATIVE\_REGULATION\_OF\_UBIQUITIN\_PROTEIN\_TRANSFERASE\_ACTIVITY |  | 16 | -0.19 | -0.51 | 0.984 | 0.999 | 1.000 | 1885 | tags=13%, list=16%, signal=15% |
| 7691 | GOMF\_UNFOLDED\_PROTEIN\_BINDING |  | 83 | -0.14 | -0.51 | 1.000 | 0.999 | 1.000 | 3519 | tags=20%, list=29%, signal=29% |
| 7692 | GOBP\_HYPOTHALAMUS\_DEVELOPMENT |  | 11 | -0.21 | -0.51 | 0.983 | 0.998 | 1.000 | 647 | tags=9%, list=5%, signal=10% |
| 7693 | GOBP\_EPITHELIAL\_STRUCTURE\_MAINTENANCE |  | 13 | -0.20 | -0.51 | 0.990 | 0.998 | 1.000 | 2852 | tags=23%, list=24%, signal=30% |
| 7694 | GOBP\_POSTREPLICATION\_REPAIR |  | 42 | -0.16 | -0.51 | 0.995 | 0.998 | 1.000 | 5351 | tags=43%, list=45%, signal=77% |
| 7695 | GOMF\_CARBON\_NITROGEN\_LYASE\_ACTIVITY |  | 11 | -0.21 | -0.51 | 0.987 | 0.998 | 1.000 | 4859 | tags=45%, list=40%, signal=76% |
| 7696 | GOMF\_PROTEIN\_LIPID\_COMPLEX\_BINDING |  | 20 | -0.18 | -0.51 | 0.990 | 0.998 | 1.000 | 4317 | tags=35%, list=36%, signal=55% |
| 7697 | GOBP\_INACTIVATION\_OF\_MAPK\_ACTIVITY |  | 17 | -0.19 | -0.50 | 0.988 | 0.998 | 1.000 | 3211 | tags=24%, list=27%, signal=32% |
| 7698 | GOBP\_CELLULAR\_DETOXIFICATION\_OF\_ALDEHYDE |  | 6 | -0.25 | -0.50 | 0.982 | 0.998 | 1.000 | 2163 | tags=17%, list=18%, signal=20% |
| 7699 | GOCC\_U2\_TYPE\_CATALYTIC\_STEP\_2\_SPLICEOSOME |  | 25 | -0.17 | -0.50 | 0.992 | 0.998 | 1.000 | 2076 | tags=12%, list=17%, signal=14% |
| 7700 | GOCC\_CCR4\_NOT\_CORE\_COMPLEX |  | 5 | -0.26 | -0.50 | 0.991 | 0.998 | 1.000 | 6440 | tags=80%, list=54%, signal=172% |
| 7701 | GOMF\_PURINE\_NUCLEOTIDE\_TRANSMEMBRANE\_TRANSPORTER\_ACTIVITY |  | 14 | -0.20 | -0.50 | 0.987 | 0.998 | 1.000 | 2605 | tags=21%, list=22%, signal=27% |
| 7702 | GOMF\_SPHINGOLIPID\_TRANSFER\_ACTIVITY |  | 7 | -0.24 | -0.50 | 0.986 | 0.998 | 1.000 | 4444 | tags=43%, list=37%, signal=68% |
| 7703 | GOBP\_ISOTYPE\_SWITCHING\_TO\_IGG\_ISOTYPES |  | 10 | -0.22 | -0.50 | 0.990 | 0.998 | 1.000 | 2639 | tags=20%, list=22%, signal=26% |
| 7704 | GOBP\_HEMATOPOIETIC\_STEM\_CELL\_DIFFERENTIATION |  | 68 | -0.15 | -0.50 | 0.998 | 0.998 | 1.000 | 3400 | tags=19%, list=28%, signal=27% |
| 7705 | GOMF\_CALCIUM\_ACTIVATED\_CATION\_CHANNEL\_ACTIVITY |  | 13 | -0.20 | -0.50 | 0.986 | 0.998 | 1.000 | 3398 | tags=23%, list=28%, signal=32% |
| 7706 | GOBP\_GAS\_HOMEOSTASIS |  | 6 | -0.25 | -0.50 | 0.986 | 0.998 | 1.000 | 4557 | tags=33%, list=38%, signal=54% |
| 7707 | GOBP\_MATURATION\_OF\_5\_8S\_RRNA |  | 30 | -0.17 | -0.50 | 0.991 | 0.998 | 1.000 | 3494 | tags=20%, list=29%, signal=28% |
| 7708 | GOBP\_NEGATIVE\_REGULATION\_OF\_CARDIAC\_MUSCLE\_CELL\_PROLIFERATION |  | 9 | -0.22 | -0.50 | 0.984 | 0.998 | 1.000 | 3392 | tags=33%, list=28%, signal=46% |
| 7709 | GOCC\_CUTICULAR\_PLATE |  | 4 | -0.27 | -0.49 | 0.990 | 0.998 | 1.000 | 4590 | tags=50%, list=38%, signal=81% |
| 7710 | GOMF\_LEUCINE\_ZIPPER\_DOMAIN\_BINDING |  | 7 | -0.23 | -0.49 | 0.986 | 0.998 | 1.000 | 1131 | tags=14%, list=9%, signal=16% |
| 7711 | GOCC\_GLYCOSYLPHOSPHATIDYLINOSITOL\_N\_ACETYLGLUCOSAMINYLTRANSFERASE\_GPI\_GNT\_COMPLEX |  | 6 | -0.24 | -0.49 | 0.986 | 0.998 | 1.000 | 5495 | tags=50%, list=46%, signal=92% |
| 7712 | GOBP\_SPINAL\_CORD\_ASSOCIATION\_NEURON\_DIFFERENTIATION |  | 4 | -0.27 | -0.49 | 0.989 | 0.998 | 1.000 | 1871 | tags=25%, list=16%, signal=30% |
| 7713 | GOCC\_RNA\_POLYMERASE\_COMPLEX |  | 80 | -0.14 | -0.49 | 0.999 | 0.998 | 1.000 | 5069 | tags=35%, list=42%, signal=60% |
| 7714 | GOBP\_ALDEHYDE\_CATABOLIC\_PROCESS |  | 8 | -0.22 | -0.49 | 0.989 | 0.998 | 1.000 | 2163 | tags=13%, list=18%, signal=15% |
| 7715 | GOBP\_MULTIVESICULAR\_BODY\_SORTING\_PATHWAY |  | 30 | -0.16 | -0.49 | 0.993 | 0.998 | 1.000 | 2774 | tags=17%, list=23%, signal=22% |
| 7716 | GOBP\_REGULATION\_OF\_ENDOSOME\_SIZE |  | 5 | -0.25 | -0.49 | 0.990 | 0.998 | 1.000 | 1804 | tags=20%, list=15%, signal=24% |
| 7717 | GOMF\_PROTEIN\_TYROSINE\_THREONINE\_PHOSPHATASE\_ACTIVITY |  | 8 | -0.22 | -0.49 | 0.988 | 0.998 | 1.000 | 4835 | tags=38%, list=40%, signal=63% |
| 7718 | GOMF\_ADENYL\_NUCLEOTIDE\_EXCHANGE\_FACTOR\_ACTIVITY |  | 11 | -0.20 | -0.49 | 0.991 | 0.998 | 1.000 | 838 | tags=9%, list=7%, signal=10% |
| 7719 | GOCC\_TRANSCRIPTION\_FACTOR\_TFIID\_COMPLEX |  | 25 | -0.17 | -0.49 | 0.993 | 0.998 | 1.000 | 6097 | tags=48%, list=51%, signal=97% |
| 7720 | GOBP\_REGULATION\_OF\_CELL\_FATE\_SPECIFICATION |  | 8 | -0.22 | -0.49 | 0.989 | 0.998 | 1.000 | 2886 | tags=25%, list=24%, signal=33% |
| 7721 | GOBP\_UREA\_TRANSPORT |  | 4 | -0.27 | -0.49 | 0.989 | 0.998 | 1.000 | 2778 | tags=25%, list=23%, signal=33% |
| 7722 | GOBP\_PHOTOTRANSDUCTION |  | 22 | -0.17 | -0.48 | 0.991 | 0.998 | 1.000 | 6233 | tags=59%, list=52%, signal=123% |
| 7723 | GOBP\_DEPYRIMIDINATION |  | 6 | -0.24 | -0.48 | 0.989 | 0.998 | 1.000 | 2346 | tags=17%, list=20%, signal=21% |
| 7724 | GOBP\_PRONEPHROS\_DEVELOPMENT |  | 4 | -0.26 | -0.48 | 0.994 | 0.999 | 1.000 | 4761 | tags=50%, list=40%, signal=83% |
| 7725 | GOBP\_POSITIVE\_REGULATION\_OF\_MIRNA\_METABOLIC\_PROCESS |  | 5 | -0.25 | -0.47 | 0.992 | 0.999 | 1.000 | 3968 | tags=40%, list=33%, signal=60% |
| 7726 | GOBP\_RNA\_CAPPING |  | 28 | -0.16 | -0.47 | 0.996 | 0.999 | 1.000 | 3896 | tags=25%, list=32%, signal=37% |
| 7727 | GOBP\_G\_PROTEIN\_COUPLED\_ACETYLCHOLINE\_RECEPTOR\_SIGNALING\_PATHWAY |  | 12 | -0.19 | -0.47 | 0.990 | 0.999 | 1.000 | 929 | tags=8%, list=8%, signal=9% |
| 7728 | GOCC\_ENDOLYSOSOME\_LUMEN |  | 4 | -0.26 | -0.47 | 0.996 | 0.999 | 1.000 | 2151 | tags=25%, list=18%, signal=30% |
| 7729 | GOBP\_ETHANOL\_OXIDATION |  | 5 | -0.24 | -0.47 | 0.993 | 0.999 | 1.000 | 5368 | tags=60%, list=45%, signal=108% |
| 7730 | GOBP\_GPI\_ANCHOR\_METABOLIC\_PROCESS |  | 24 | -0.16 | -0.47 | 0.993 | 0.999 | 1.000 | 5495 | tags=42%, list=46%, signal=77% |
| 7731 | GOBP\_NEGATIVE\_REGULATION\_BY\_HOST\_OF\_VIRAL\_TRANSCRIPTION |  | 11 | -0.20 | -0.47 | 0.992 | 0.999 | 1.000 | 2386 | tags=18%, list=20%, signal=23% |
| 7732 | GOBP\_MATURATION\_OF\_LSU\_RRNA\_FROM\_TRICISTRONIC\_RRNA\_TRANSCRIPT\_SSU\_RRNA\_5\_8S\_RRNA\_LSU\_RRNA |  | 15 | -0.18 | -0.47 | 0.992 | 0.999 | 1.000 | 923 | tags=7%, list=8%, signal=7% |
| 7733 | GOCC\_SM\_LIKE\_PROTEIN\_FAMILY\_COMPLEX |  | 62 | -0.14 | -0.47 | 0.999 | 0.998 | 1.000 | 3611 | tags=19%, list=30%, signal=28% |
| 7734 | GOBP\_EXTRACELLULAR\_VESICLE\_BIOGENESIS |  | 20 | -0.17 | -0.47 | 0.993 | 0.998 | 1.000 | 358 | tags=5%, list=3%, signal=5% |
| 7735 | GOBP\_TYPE\_B\_PANCREATIC\_CELL\_APOPTOTIC\_PROCESS |  | 8 | -0.21 | -0.47 | 0.993 | 0.998 | 1.000 | 959 | tags=13%, list=8%, signal=14% |
| 7736 | GOBP\_PROLINE\_METABOLIC\_PROCESS |  | 6 | -0.23 | -0.47 | 0.993 | 0.998 | 1.000 | 2105 | tags=17%, list=18%, signal=20% |
| 7737 | GOBP\_POSITIVE\_REGULATION\_OF\_CHOLESTEROL\_ESTERIFICATION |  | 4 | -0.26 | -0.47 | 0.996 | 0.998 | 1.000 | 2584 | tags=25%, list=22%, signal=32% |
| 7738 | GOBP\_HEART\_TRABECULA\_FORMATION |  | 9 | -0.21 | -0.46 | 0.994 | 0.998 | 1.000 | 3314 | tags=22%, list=28%, signal=31% |
| 7739 | GOBP\_INTRACILIARY\_TRANSPORT\_INVOLVED\_IN\_CILIUM\_ASSEMBLY |  | 34 | -0.15 | -0.46 | 0.998 | 0.998 | 1.000 | 3051 | tags=18%, list=25%, signal=24% |
| 7740 | GOMF\_SUPEROXIDE\_GENERATING\_NADPH\_OXIDASE\_ACTIVATOR\_ACTIVITY |  | 8 | -0.21 | -0.46 | 0.992 | 0.999 | 1.000 | 5190 | tags=50%, list=43%, signal=88% |
| 7741 | GOBP\_NEGATIVE\_REGULATION\_OF\_UBIQUITIN\_PROTEIN\_LIGASE\_ACTIVITY |  | 9 | -0.20 | -0.45 | 0.997 | 0.999 | 1.000 | 9629 | tags=100%, list=80%, signal=504% |
| 7742 | GOCC\_SPECTRIN |  | 5 | -0.23 | -0.45 | 0.995 | 0.999 | 1.000 | 4047 | tags=40%, list=34%, signal=60% |
| 7743 | GOBP\_SUCKLING\_BEHAVIOR |  | 8 | -0.20 | -0.45 | 0.995 | 0.999 | 1.000 | 3733 | tags=25%, list=31%, signal=36% |
| 7744 | GOBP\_RNA\_MODIFICATION |  | 120 | -0.12 | -0.45 | 1.000 | 0.999 | 1.000 | 5211 | tags=33%, list=43%, signal=57% |
| 7745 | GOBP\_METANEPHRIC\_NEPHRON\_TUBULE\_MORPHOGENESIS |  | 5 | -0.23 | -0.44 | 0.999 | 0.999 | 1.000 | 4111 | tags=40%, list=34%, signal=61% |
| 7746 | GOBP\_PROTEIN\_INSERTION\_INTO\_MITOCHONDRIAL\_MEMBRANE\_INVOLVED\_IN\_APOPTOTIC\_SIGNALING\_PATHWAY |  | 20 | -0.16 | -0.44 | 0.997 | 0.999 | 1.000 | 1710 | tags=10%, list=14%, signal=12% |
| 7747 | GOMF\_CELL\_ADHESION\_MEDIATOR\_ACTIVITY |  | 41 | -0.14 | -0.44 | 1.000 | 0.999 | 1.000 | 3439 | tags=22%, list=29%, signal=31% |
| 7748 | GOBP\_MAINTENANCE\_OF\_PROTEIN\_LOCATION\_IN\_NUCLEUS |  | 17 | -0.16 | -0.44 | 0.996 | 0.999 | 1.000 | 5638 | tags=47%, list=47%, signal=89% |
| 7749 | GOBP\_METANEPHRIC\_NEPHRON\_DEVELOPMENT |  | 25 | -0.15 | -0.43 | 0.998 | 0.999 | 1.000 | 2374 | tags=16%, list=20%, signal=20% |
| 7750 | GOCC\_RNA\_POLYMERASE\_II\_HOLOENZYME |  | 61 | -0.12 | -0.42 | 1.000 | 1.000 | 1.000 | 4852 | tags=30%, list=40%, signal=49% |
| 7751 | GOMF\_HYDROLASE\_ACTIVITY\_ACTING\_ON\_CARBON\_NITROGEN\_BUT\_NOT\_PEPTIDE\_BONDS\_IN\_LINEAR\_AMIDES |  | 49 | -0.13 | -0.42 | 1.000 | 0.999 | 1.000 | 5118 | tags=37%, list=43%, signal=64% |
| 7752 | GOCC\_POLYSOME |  | 57 | -0.12 | -0.41 | 1.000 | 0.999 | 1.000 | 5423 | tags=32%, list=45%, signal=57% |
| 7753 | GOBP\_PROTEIN\_TARGETING |  | 342 | -0.10 | -0.41 | 1.000 | 1.000 | 1.000 | 4383 | tags=24%, list=36%, signal=36% |
| 7754 | GOBP\_MATURATION\_OF\_LSU\_RRNA |  | 27 | -0.14 | -0.40 | 1.000 | 0.999 | 1.000 | 10373 | tags=100%, list=86%, signal=732% |
| 7755 | GOBP\_NEGATIVE\_REGULATION\_OF\_TRANSCRIPTION\_REGULATORY\_REGION\_DNA\_BINDING |  | 13 | -0.16 | -0.40 | 0.999 | 0.999 | 1.000 | 6186 | tags=62%, list=52%, signal=127% |
| 7756 | GOMF\_CHONDROITIN\_SULFATE\_BINDING |  | 6 | -0.20 | -0.40 | 0.999 | 0.999 | 1.000 | 1734 | tags=17%, list=14%, signal=19% |
| 7757 | GOBP\_BRANCHED\_CHAIN\_AMINO\_ACID\_METABOLIC\_PROCESS |  | 20 | -0.14 | -0.39 | 0.999 | 0.999 | 1.000 | 5137 | tags=45%, list=43%, signal=78% |
| 7758 | GOBP\_MANGANESE\_ION\_TRANSPORT |  | 14 | -0.15 | -0.39 | 0.999 | 0.999 | 1.000 | 5103 | tags=43%, list=42%, signal=74% |
| 7759 | GOCC\_U2\_TYPE\_SPLICEOSOMAL\_COMPLEX |  | 75 | -0.11 | -0.38 | 1.000 | 0.999 | 1.000 | 6476 | tags=44%, list=54%, signal=95% |
| 7760 | GOCC\_TRANSCRIPTION\_EXPORT\_COMPLEX |  | 10 | -0.16 | -0.36 | 0.999 | 0.999 | 1.000 | 10126 | tags=100%, list=84%, signal=637% |
| 7761 | GOBP\_TRNA\_MODIFICATION |  | 69 | -0.10 | -0.34 | 1.000 | 0.999 | 1.000 | 5647 | tags=33%, list=47%, signal=63% |
Table: Gene sets enriched in phenotype **0 (3 samples) **[plain text format]****
